# GWAS Meets Microarray: Are the Results of Genome-Wide Association Studies and Gene-Expression Profiling Consistent? Prostate Cancer as an Example

**DOI:** 10.1371/journal.pone.0006511

**Published:** 2009-08-04

**Authors:** Ivan P. Gorlov, Gary E. Gallick, Olga Y. Gorlova, Christopher Amos, Christopher J. Logothetis

**Affiliations:** 1 Department of Genitourinary Medical Oncology, The University of Texas M. D. Anderson Cancer Center, Houston, Texas, United States of America; 2 Department of Epidemiology, The University of Texas M. D. Anderson Cancer Center, Houston, Texas, United States of America; Tel Aviv University, Israel

## Abstract

**Background:**

Genome-wide association studies (GWASs) and global profiling of gene expression (microarrays) are two major technological breakthroughs that allow hypothesis-free identification of candidate genes associated with tumorigenesis. It is not obvious whether there is a consistency between the candidate genes identified by GWAS (GWAS genes) and those identified by profiling gene expression (microarray genes).

**Methodology/Principal Findings:**

We used the Cancer Genetic Markers Susceptibility database to retrieve single nucleotide polymorphisms from candidate genes for prostate cancer. In addition, we conducted a large meta-analysis of gene expression data in normal prostate and prostate tumor tissue. We identified 13,905 genes that were interrogated by both GWASs and microarrays. On the basis of P values from GWASs, we selected 1,649 most significantly associated genes for functional annotation by the Database for Annotation, Visualization and Integrated Discovery. We also conducted functional annotation analysis using same number of the top genes identified in the meta-analysis of the gene expression data. We found that genes involved in cell adhesion were overrepresented among both the GWAS and microarray genes.

**Conclusions/Significance:**

We conclude that the results of these analyses suggest that combining GWAS and microarray data would be a more effective approach than analyzing individual datasets and can help to refine the identification of candidate genes and functions associated with tumor development.

## Introduction

Microarray technology allows simultaneous assessment of the expression of virtually all genes in the genome. This approach has been widely used to identify candidate genes associated with cancer development and progression [1]–[3]. Genome-wide association studies (GWASs) have recently emerged as a powerful tool to identify genetic polymorphisms associated with cancer risk [4], [5]. In a GWAS, hundreds of thousands of single nucleotide polymorphisms (SNPs) are genotyped in a large number of cases and controls. A difference in allelic or genotype frequencies between cases and controls suggests an association between cancer risk and the SNP and a linked gene or regulatory region.

Whether these two approaches produce comparable results has not been examined. Recently Chen et al. [6] identified the genes that tend to be differentially expressed across various experiential conditions and states using gene-expression data from the Gene Expression Omnibus (GEO). They found that differentially expressed genes are more likely to be detected as disease variants in association studies.

In this study, we undertook a more direct approach to link GWAS and microarray data. We performed functional annotations of the top genes identified in prostate cancer GWASs and the same number of the top candidate genes identified in a meta-analysis of the gene-expression data for normal prostate and prostate tumor. The results of our analyses indicate that these two approaches yield similar results at the functional level.

## Materials and Methods

Several prostate cancer GWASs were recently conducted, and a number of candidate genes were identified (Table 1) [7]–[10]. Though only a few SNPs with the genome-wide level of significance, 10^−7^, were identified in these studies, a number of SNPs were significant at the level of individual tests but nonsignificant after correction for multiple testing. Such SNPs likely indicate enrichment with causal SNPs that do not reach the genome-wide significance level because of their small effect size or low allele frequency [11].

**Table 1:** Candidate genes identified in prostate cancer GWAS.

Gene	GeneID	Comparison	PMID	Microarray Ps
*CTBP2*	1488	nonaggressive vs. aggressive prostate cancer	18264096	4.6×10^−6^
*EHBP1*	23301	case control study	18264098	0.63
*SLC22A3*	6581	case control study	18264097	1.1×10^−4^
*LMTK2*	22853	case control study	18264097	0.38
*KLK2*	3817	case control study	18264097	1.4×10^−7^
*KLK3*	354	case control study	18264097	2.1×10^−9^
*NUDT10*	170685	case control study	18264097	5.6×10^−11^
*DAB2IP*	153090	nonaggressive vs. aggressive prostate cancer	18073375	8.0×10^−7^
*HNF1B*	6928	nonaggressive vs. aggressive prostate cancer	18264096	0.31
*HPC1(RNASEL)*	6041	controls vs. sporadic prostate cancer	17876339	1.1×10^−3^
*JAZF1*	221895	nonaggressive vs. aggressive prostate cancer	18264096	1.5×10^−10^
*MSMB*	4477	nonaggressive vs. aggressive prostate cancer	18264096	4.7×10^−4^
*MYC*	4609	controls vs. cases	17401366	2.6×10^−40^

The GWAS data for this analysis were retrieved from the Cancer Genetic Markers Susceptibility (CGEMS) database, http://cgems.cancer.gov/about/executive_summary.asp. We used the Oncomine database http://www.oncomine.org/main/index.jsp to conduct a meta-analysis of the number of studies comparing gene expression in normal prostate tissue with that of localized prostate tumor tissue [12]. The complete list of the studies used in the meta-analysis can be found in the supplementary materials (Table S1). We used an extension of Stouffer's method [13] for the meta-analysis. This approach is based on estimating the standard normal deviation, *Z*, and is similar to the approach recently proposed by Ochsner et al. [14]. The meta-analysis identified a number of genes differentially expressed between normal prostate and prostate tumor.

## Results

As an initial validation of our hypothesis that GWASs and microarrays tend to identify the same genes, we used a meta-analysis of the Oncomine gene-expression data to assess the expression of the GWAS-identified genes (Table 1). We found that all but three (*HNF1B, EHBP1*, and *LMTK2*) of the genes were differentially expressed between the normal and tumorous prostate. Therefore, 10 of 13 (77%) of the GWAS genes were differentially expressed in the transition from normal prostate to prostate cancer that is higher than one can expect to detect among randomly chosen 13 genes −1.1 (χ^2^ = 20.9, df = 1, P<0.0001).

The prostate cancer GWAS data from CGEMS Phase 1A and Phase 1B, were used in the analysis. We limited our analysis to the gene-associated SNPs to make GWAS and microarray results comparable. We followed the CGEMS designation of the gene-associated SNPs. A total 63,831 gene-associated SNPs belonging to 16,550 unique genes were identified. For each gene, a SNP with the smallest P value was used to characterize an association. If a given SNP was associated with multiple genes, all those associations were included in our analysis. Because in many cases aliases rather than official gene names were used in GWAS, we linked various gene identifiers to the official gene names and EntrezIDs using the latest version of the NCBI gene database (accessed January 17, 2009). Overlapping of the unique GWAS and microarray genes demonstrated that 13,905 genes were assessed in both GWAS and microarray analyses. The list of the genes with corresponding GWAS and microarray P values is shown in Table S1.

**Figure 1:**
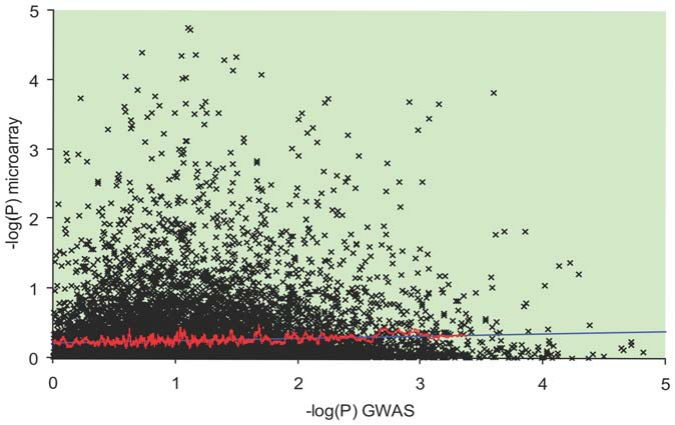
The plot of GWAS and microarray –log(P)s. Black line shows the linear regression curve, red line – moving average computed using a sliding window of 100 points. Spearman's rank-order correlation coefficient: r = 0.043, N = 13905, P = 0.0000001.

To assess whether the GWAS and microarray analyses tend to identify similar sets of genes we assess a correlation between –log(P) values based on GWAS data and –log(P) values based on the analysis of gene expression. We found a small but significant (because of the large sample size) positive correlation between GWAS and microarray –log(P)s (Figure 1).

The Database for Annotation, Visualization and Integrated Discovery (DAVID) [15] was used for the functional annotation of GWAS and microarray genes. We selected genes with GWAS P values ≤0.01. A total of 1,649 genes were identified. We used exactly the same number of the top genes identified in the meta-analysis of the gene-expression data. To control for possible biases in gene selection, we used the list of 13,905 genes as background. Functional annotation charts were used to retrieve an extended annotation coverage that included more than 40 annotation categories [15]. A functional chart for the top GWAS genes can be found in Table S2. Many cell adhesion–related categories are among the top annotation categories. Clustering of the terms of functional annotations summarized all types of the functional description used by DAVID, identifying cell adhesion as the top cluster, followed by plasma membrane and fibronectin.

**Figure 2:**
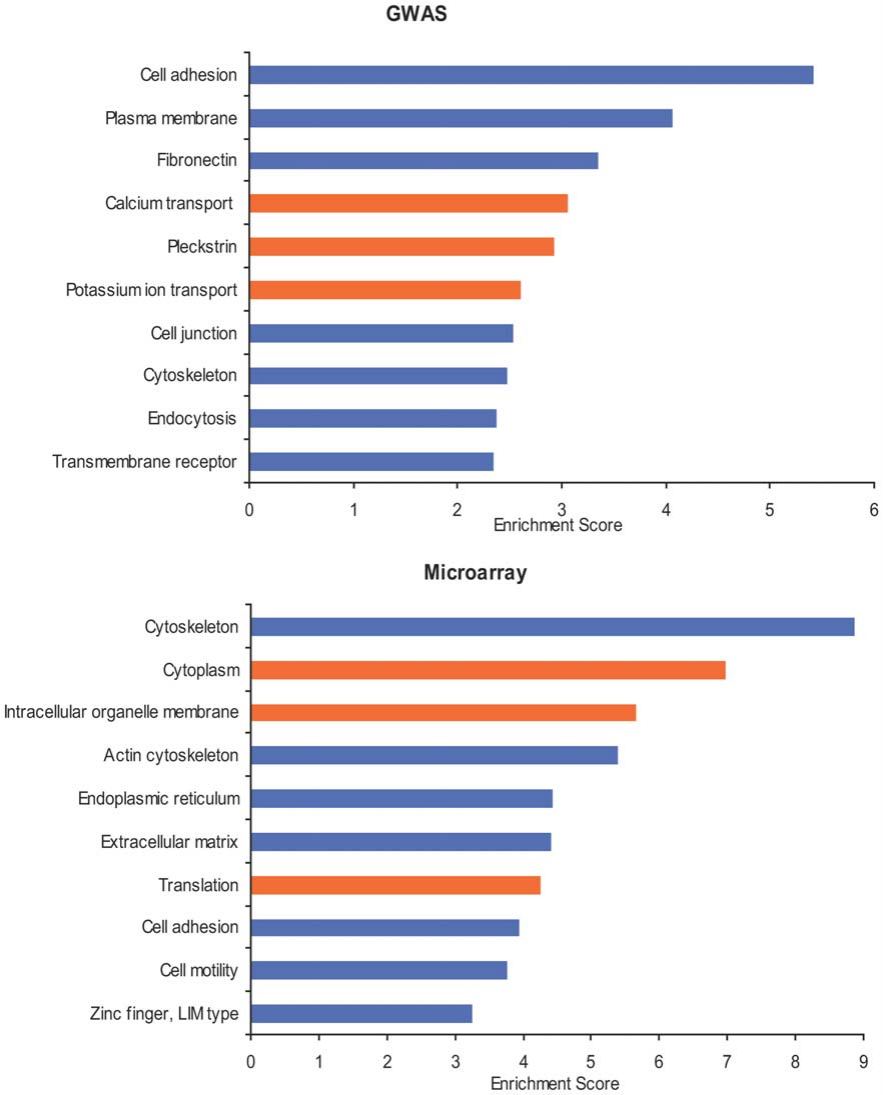
Clustering of the functional annotation terms based on GWAS- (upper panel) and microarray-derived genes (lower panel). Functional clusters related to cell adhesion are shown in blue. Detailed information on the composition of clusters can be found in Table S4.

Functional annotation of the top differentially expressed genes identified cytoskeleton, focal adhesion, extracellular matrix, and cell adhesion as the top annotation terms (Table S3). Clustering of the terms of functional annotation demonstrated cytoskeleton, actin cytoskeleton, extracellular matrix, and cell adhesion among the top identified clusters. Figure 2 shows the results of the clustering of functional terms by DAVID based on the analysis of the top GWAS and differentially expressed genes (see also Table S4). In both lists, most of the top functional clusters derived from GWAS and microarray data are directly or indirectly related to cell adhesion.

We next looked for an overlap between the top 1,649 GWAS and the top 1,649 differentially expressed genes. We identified 248 appearing in both lists genes (see supplementary materials for the list of the genes). This number is higher than would be expected by chance. If we randomly sample 1,649 genes from among the 13,905 genes, the expected number of the genes found in two independent samples would be (1,649/13,905)ˆ2*1,649 = 23.2. The functional annotation of these 248 genes identified cytoskeleton, focal adhesion, and actin binding as top functional categories. Functional clustering of the genes identified cell migration, cell motility, cytoskeleton, and cell adhesion as the top clusters.

## Discussion

GWAS and microarray analyses both allow unbiased identification of candidate genes and pathways associated with cancer development. These two approaches each have advantages and drawbacks. By combining data from multiple expression studies, analyses of gene expressions have the statistical power to detect even small differences in gene expression between normal and tumor tissues. On the other hand, because genes in the human genome are involved in multiple interactions, modulation of the expression of a single gene may cause a “ripple effect” on multiple downstream targets, making it difficult to separate causal and induced changes in gene expression. This is unlikely to be an issue in GWASs. GWASs, however, are often statistically underpowered to detect SNPs with small effect size.

When we compared candidate genes for prostate cancer identified by GWAS with those identified by microarray, we noted a significant positive correlation between the GWAS and microarray –log(P)s. The correlation was small, with the Pearson rank correlation coefficient being only 0.04, but positive correlation between two ranks is expected to be driven by a relatively small number of causal genes. Not all causal genes will be detected by GWAS. Even if the gene is mechanistically linked to prostate tumorigenesis, it can be detected by GWAS only if it carries genetic variants that modulate its function. On the other hand, genes identified by microarray analysis are expected to be a mix of causal genes and the genes that are differentially expressed because of the ripple effect of the causal genes. This suggests that only a fraction of the genes significant in both analyses are causal genes.

We found that the top GWAS and differentially expressed candidates were enriched in cell adhesion genes. If we consider all known cell adhesion genes in the genome, only 74 genes or 10% of them were among the top differentially expressed genes. If the cell adhesion pathway is associated with prostate tumorigenesis, one can expect that other cell adhesion genes—those that did not make it to the top 1,649 genes—also will tend to be significantly positively associated. We found that the average GWAS-derived P value for the cell adhesion genes that failed to reach the top 1649 was lower than the average value for the GWAS genes (t test = 2.9, df = 13,902, P = 0.001). A similar result was obtained for the P values derived from the analysis of the gene expression: the absolute Z score was higher among cell adhesion genes (excluding those among the top 1649 genes) than was the average Z score (t test = 1.81, df = 17811, P = 0.07 on the two-tailed test and P = 0.03 on the one-tailed test). This suggests that cell adhesion function as a whole is associated with prostate tumorigenesis.

Both GWAS and microarray genes form functional clusters related to different aspects of cell adhesion, including cell adhesion itself, cell junction, extracellular matrix glycoproteins, fibronectin, actin cytoskeleton, and cell motility. Several other clusters also show a mechanistic association with cell adhesion. For example, cadherin uptake from the cell surface by endocytosis regulates the level of the free cadherins on the cell surface and therefore cell adhesion [16]. Also, zinc finger proteins with the LIM domain are important for focal adhesion and cell adhesion to fibronectin [17], [18]. The modulation of the cell adhesion function seems not to be limited to any specific adhesion type but includes cadherins, integrins, and selectins as well as adhesion molecules associated with tight junctions.

The results of a number of studies suggested the involvement of the cell adhesion system in prostate cancer development. Cadherins play a role in regulating tumor cell proliferation through cyclins and cyclin-dependent kinases [19]. Integrins are involved in different aspects of prostate tumorigenesis, including cell proliferation, cell motility, and apoptosis [20]–[22]. Modulation of cell adhesion can play an important role in epithelial-to-mesenchymal transition that is believed to be a key step in malignant transformation [23]–[25]. Also the results of a number of studies suggestd an involvement of cell adhesion in angiogenesis [26]–[28].

GWAS-identified genes are considered to be cancer susceptibility genes that are mainly associated with tumor initiation. We believe, however, that genes identified by GWAS are also likely to include genes important for tumor progression. Indeed, the detection of tumor is usually symptomatic: the tumor needs to reach a certain size to be detected. This suggests that genes involved in tumor progression will be among GWAS-detected candidate genes. Therefore, GWAS and gene expression analysis may target essentially the same set of genes, providing the theoretical basis for the joint analysis of GWAS and microarray data.

In summary, our analysis found a considerable overlap between prostate cancer genes identified by GWAS and those identified by global profiling of the gene expression. We identified cell adhesion as a biological function associated with prostate tumorigenesis. The results of this study also suggest that combining GWAS and microarray data might be a more effective approach than using just the analysis of the individual datasets, and can help to refine the identification of candidate genes and/or functions involved in tumor development.

## Supporting Information

Table S1 (0.83 MB PDF)

Table S2 (0.07 MB XLS)

Table S3 (0.33 MB XLS)

Table S4 (0.33 MB XLS)
