# Supplementary material for: GWAS Meets Microarray: Are the Results of Genome-Wide Association Studies and Gene-Expression Profiling Consistent? Prostate Cancer as an Example
Source: PLoS One. 2009 Aug 4;4(8):e6511. doi: 10.1371/journal.pone.0006511 (PMC2714961; doi:10.1371/journal.pone.0006511)
Supplement: Table S1 — (0.83 MB PDF) [file pone.0006511.s001.pdf]

gwas_MA_together

The list of 13,905 genes studied in prostate GWAS and in meta-analysis of the gene expression in the transition from normal prostate to prostate tumor

SNP	Gene_ID	Gene_name	Chr	Position	GWAS_P	GWAS_rank	MA_P	Direction	ABS_Z	MA_rank	-log(P)_GWAS	-log(P)_MA
rs7736101	23194	FBXL7	5	15683653	9.00E-06	1	1.20E-14	Down	7.72	415	5.05	1.39
rs2598272	136332	LRGUK	7	133400080	1.50E-05	2	1.15E-01	Up	1.58	7216	4.82	0.09
rs12421354	54538	ROBO4	11	124270378	1.90E-05	3	3.64E-03	Down	2.91	3878	4.72	0.24
rs1712391	23339	VPS39	15	40272182	1.90E-05	4	1.26E-02	Down	2.49	4664	4.72	0.19
rs923231	7328	UBE2H	7	129085642	2.20E-05	5	6.23E-02	Up	1.86	6285	4.66	0.12
rs4496780	54901	CDKAL1	6	21187627	2.30E-05	6	2.92E-02	Up	2.18	5435	4.64	0.15
rs12219619	387694	SH2D4B	10	82372533	3.20E-05	7	4.90E-01	Down	0.69	10654	4.49	0.03
rs6708456	547	KIF1A	2	241449983	3.90E-05	8	2.31E-01	Down	1.20	8572	4.41	0.06
rs7775507	84456	L3MBTL3	6	130440261	4.10E-05	9	1.50E-05	Up	4.33	2036	4.39	0.48
rs1570555	4008	LMO7	13	75269877	4.20E-05	10	9.53E-01	Up	0.06	13579	4.38	0.00
rs2218724	27303	RBMS3	3	29900522	5.10E-05	11	7.27E-13	Down	7.17	543	4.29	1.21
rs2371438	2066	ERBB4	2	212791037	5.70E-05	12	8.56E-01	Up	0.18	12989	4.24	0.01
rs241704	2272	FHIT	3	60487929	6.00E-05	13	1.59E-14	Up	7.68	425	4.22	1.38
rs2371136	25924	MYRIP	3	40250659	7.40E-05	14	4.25E-14	Up	7.55	449	4.13	1.34
rs11118988	5362	PLXNA2	1	204684478	7.50E-05	15	1.96E-01	Down	1.29	8213	4.12	0.07
rs465543	2015	EMR1	19	6892867	7.60E-05	16	5.02E-01	Up	0.67	10731	4.12	0.03
rs1180937	7268	TTC4	1	54903665	8.20E-05	17	1.64E-02	Down	2.40	4906	4.09	0.18
rs4852261	8291	DYSF	2	71633751	8.30E-05	18	9.26E-01	Down	0.09	13401	4.08	0.00
rs11042008	65975	STK33	11	8582393	8.60E-05	19	2.13E-01	Down	1.25	8387	4.07	0.07
rs13336298	57020	C16orf62	16	19577949	8.90E-05	20	2.91E-03	Down	2.98	3760	4.05	0.25
rs17551174	1993	ELAVL2	9	23807548	8.90E-05	21	7.54E-03	Up	2.67	4305	4.05	0.21
rs4654092	64754	SMYD3	1	242645523	9.00E-05	22	6.53E-05	Up	3.99	2347	4.05	0.42
rs6076157	1473	CST5	20	23810844	9.00E-05	23	3.66E-01	Down	0.90	9723	4.05	0.04
rs4924675	825	CAPN3	15	40468349	9.20E-05	24	2.08E-01	Down	1.26	8328	4.04	0.07
rs11658803	10693	CCT6B	17	30310777	9.30E-05	25	9.03E-02	Up	1.69	6825	4.03	0.10
rs12032117	5142	PDE4B	1	66417992	9.60E-05	26	3.25E-11	Down	6.63	695	4.02	1.05
rs3778607	3662	IRF4	6	348799	9.90E-05	27	7.76E-01	Down	0.28	12535	4.00	0.01
rs10121085	2790	GNG10	9	111466154	1.01E-04	28	6.36E-01	Down	0.47	11679	4.00	0.02
rs803371	55929	DMAP1	1	44335438	1.03E-04	29	9.63E-01	Up	0.05	13635	3.99	0.00
rs3806839	56114	PCDHGA1	5	140701630	1.08E-04	30	2.01E-02	Up	2.32	5083	3.97	0.17
rs3806839	56112	PCDHGA3	5	140701630	1.08E-04	31	5.79E-02	Up	1.90	6186	3.97	0.12
rs2293877	8747	ADAM21	14	70005628	1.17E-04	32	2.27E-01	Up	1.21	8532	3.93	0.06
rs1518805	53353	LRP1B	2	141785676	1.26E-04	33	6.22E-01	Down	0.49	11597	3.90	0.02
rs3802893	1740	DLG2	11	84219051	1.27E-04	34	8.30E-01	Up	0.22	12852	3.90	0.01
rs10912899	9674	KIAA0040	1	171852372	1.31E-04	35	1.50E-04	Down	3.79	2552	3.88	0.38
rs10912899	63923	TNN	1	171852372	1.31E-04	36	1.30E-01	Up	1.51	7446	3.88	0.09
rs3017183	6925	TCF4	18	51319241	1.37E-04	37	1.35E-08	Down	5.68	1080	3.86	0.79
rs2298126	9317	PTER	10	16598991	1.38E-04	38	6.17E-02	Up	1.87	6273	3.86	0.12
rs316365	1992	SERPINB1	6	2775522	1.39E-04	39	4.86E-19	Down	8.92	228	3.86	1.83
rs2077725	4898	NRD1	1	52005591	1.42E-04	40	3.10E-08	Up	5.53	1172	3.85	0.75
rs656068	5797	PTPRM	18	8381829	1.50E-04	41	1.30E-02	Up	2.48	4693	3.82	0.19
rs1452647	8715	NOL4	18	29690523	1.50E-04	42	1.24E-01	Up	1.54	7359	3.82	0.09
rs16841542	84448	ABLIM2	4	8120462	1.53E-04	43	3.17E-01	Down	1.00	9332	3.82	0.05
rs1440606	56884	FSTL5	4	163184382	1.61E-04	44	9.98E-01	Up	0.00	13900	3.79	0.00
rs1344098	10129	FRY	13	31555756	1.71E-04	45	3.20E-02	Down	2.14	5528	3.77	0.15
rs3918278	4318	MMP9	20	44069061	1.71E-04	46	8.83E-01	Down	0.15	13147	3.77	0.01
rs3751832	51741	WWOX	16	77771773	1.72E-04	47	1.19E-02	Down	2.52	4628	3.76	0.19
rs4334675	114884	OSBPL10	3	31908623	1.79E-04	48	2.05E-02	Down	2.32	5095	3.75	0.17
rs8073077	22994	AZI1	17	76809616	1.89E-04	49	1.70E-01	Up	1.37	7912	3.72	0.08
rs8073077	146705	C17orf56	17	76809616	1.89E-04	50	9.98E-01	Down	0.00	13895	3.72	0.00
rs11633732	55466	DNAJA4	15	76330694	1.92E-04	51	1.12E-01	Down	1.59	7180	3.72	0.09
rs11633732	23205	ACSBG1	15	76330694	1.92E-04	52	5.14E-01	Down	0.65	10817	3.72	0.03
rs2179845	10559	SLC35A1	6	88222998	1.94E-04	53	1.17E-01	Down	1.57	7242	3.71	0.09
rs594223	3710	ITPR3	6	33775943	2.05E-04	54	5.69E-19	Up	8.91	230	3.69	1.82
rs594223	84300	C6orf125	6	33775943	2.05E-04	55	1.74E-01	Up	1.36	7953	3.69	0.08
rs4775230	27023	FOXB1	15	58091041	2.16E-04	56	3.30E-02	Down	2.13	5561	3.67	0.15
rs2075110	1956	EGFR	7	54993368	2.18E-04	57	2.95E-03	Down	2.97	3771	3.66	0.25
rs978528	23400	ATP13A2	1	17095104	2.26E-04	58	1.92E-04	Up	3.73	2619	3.65	0.37
rs978528	6390	SDHB	1	17095104	2.26E-04	59	3.10E-02	Up	2.16	5495	3.65	0.15
rs1190976	51466	EVL	14	99621154	2.36E-04	60	6.30E-02	Up	1.86	6297	3.63	0.12
rs11879821	84436	ZNF528	19	57588761	2.37E-04	61	4.34E-02	Up	2.02	5836	3.63	0.14
rs1861427	6546	SLC8A1	2	40299140	2.38E-04	62	2.94E-04	Down	3.62	2766	3.62	0.35
rs10994094	283025	C10orf40	10	61398202	2.39E-04	63	5.53E-02	Down	1.92	6121	3.62	0.13
rs210648	285761	DCBLD1	6	117950765	2.40E-04	64	5.23E-06	Up	4.56	1826	3.62	0.53
rs7456402	221935	SDK1	7	3883101	2.44E-04	65	1.39E-18	Up	8.79	246	3.61	1.79
rs7837328	5462	POU5F1P1	8	128492309	2.44E-04	66	7.95E-01	Up	0.26	12644	3.61	0.01
rs12755775	2153	F5	1	166279375	2.54E-04	67	5.83E-39	Up	12.94	20	3.60	3.82
rs10257807	222894	FERD3L	7	18959705	2.56E-04	68	6.98E-01	Up	0.39	12058	3.59	0.02
rs7617277	3680	ITGA9	3	37619427	2.57E-04	69	6.54E-11	Down	6.53	732	3.59	1.02
rs1350888	1012	CDH13	16	81342444	2.57E-04	70	1.25E-03	Up	3.23	3315	3.59	0.29
rs36258	79603	LASS4	19	8232244	2.58E-04	71	3.80E-05	Up	4.12	2216	3.59	0.44
rs2304150	6261	RYR1	19	43647423	2.64E-04	72	3.96E-01	Up	0.85	9956	3.58	0.04
rs2222723	55607	PPP1R9A	7	94519376	2.66E-04	73	2.44E-12	Up	6.99	597	3.58	1.16
rs7986346	8660	IRS2	13	109251608	2.70E-04	74	1.27E-01	Down	1.52	7411	3.57	0.09
rs7971324	7004	TEAD4	12	2969185	2.85E-04	75	1.04E-01	Down	1.63	7057	3.55	0.10
rs2293505	26053	AUTS2	7	69681438	2.91E-04	76	2.06E-06	Down	4.75	1673	3.54	0.57

rs2244185	7267	TTC3	21	37460989	2.96E-04	77	3.53E-06	Up	4.64	1764	3.53	0.55
rs798612	8131	C16orf35	16	95320	2.98E-04	78	4.03E-02	Up	2.05	5755	3.53	0.14
rs10773647	121256	TMEM132D	12	128404159	3.00E-04	79	4.51E-01	Down	0.75	10351	3.52	0.03
rs2320433	51011	FAHD2A	2	95478885	3.09E-04	80	4.84E-03	Down	2.82	4040	3.51	0.23
rs12564019	26476	OR10J1	1	156222063	3.22E-04	81	8.22E-01	Up	0.23	12800	3.49	0.01
rs2253319	861	RUNX1	21	35109916	3.25E-04	82	3.98E-03	Down	2.88	3931	3.49	0.24
rs3788723	9620	CELSR1	22	45226052	3.25E-04	83	1.51E-01	Down	1.44	7694	3.49	0.08
rs6039512	57144	PAK7	20	9510922	3.32E-04	84	9.81E-01	Up	0.02	13782	3.48	0.00
rs8066534	4642	MYO1D	17	27921448	3.53E-04	85	3.82E-10	Up	6.26	828	3.45	0.94
rs1747019	161176	C14orf49	14	94955650	3.77E-04	86	8.91E-02	Up	1.70	6786	3.42	0.10
rs2062608	79674	VEPH1	3	158488491	3.84E-04	87	1.88E-04	Down	3.73	2605	3.42	0.37
rs13344313	170463	SSBP4	19	18378767	3.93E-04	88	6.90E-05	Down	3.98	2359	3.41	0.42
rs13344313	126364	LRRC25	19	18378767	3.93E-04	89	8.55E-01	Down	0.18	12984	3.41	0.01
rs12252380	5592	PRKG1	10	53510561	3.97E-04	90	2.30E-06	Down	4.73	1689	3.40	0.56
rs2581199	8320	EOMES	3	27731255	3.99E-04	91	2.47E-01	Up	1.16	8730	3.40	0.06
rs7974103	11176	BAZ2A	12	55300111	4.05E-04	92	3.13E-03	Up	2.95	3799	3.39	0.25
rs208354	2768	GNA12	7	2554104	4.05E-04	93	3.07E-01	Down	1.02	9241	3.39	0.05
rs10492827	2903	GRIN2A	16	9776259	4.05E-04	94	9.57E-01	Down	0.05	13601	3.39	0.00
rs2207527	81563	C1orf21	1	181173853	4.06E-04	95	1.33E-02	Down	2.48	4710	3.39	0.19
rs4962416	1488	CTBP2	10	126686862	4.09E-04	96	4.56E-06	Up	4.58	1798	3.39	0.53
rs17121287	178	AGL	1	100014535	4.16E-04	97	1.03E-02	Down	2.56	4536	3.38	0.20
rs6045868	5173	PDYN	20	1915278	4.25E-04	98	2.81E-01	Down	1.08	9037	3.37	0.06
rs803939	23245	ASTN2	9	116533009	4.25E-04	99	3.47E-01	Up	0.94	9568	3.37	0.05
rs803939	22954	TRIM32	9	116533009	4.25E-04	100	6.84E-01	Down	0.41	11971	3.37	0.02
rs17057619	253714	C6orf167	6	97847372	4.25E-04	101	9.33E-01	Down	0.08	13452	3.37	0.00
rs11758699	51390	AIG1	6	143580992	4.28E-04	102	3.58E-07	Up	5.09	1437	3.37	0.64
rs3822795	9037	SEMA5A	5	9491905	4.31E-04	103	1.84E-05	Down	4.28	2076	3.37	0.47
rs1972933	5608	MAP2K6	17	64952406	4.39E-04	104	1.05E-09	Up	6.11	894	3.36	0.90
rs2067060	5104	SERPINA5	14	94128490	4.44E-04	105	4.24E-04	Down	3.52	2901	3.35	0.34
rs6570995	2898	GRIK2	6	102418880	4.52E-04	106	3.30E-01	Up	0.97	9442	3.34	0.05
rs4277638	2720	GLB1	3	33055360	4.59E-04	107	8.41E-06	Up	4.45	1916	3.34	0.51
rs13388421	25780	RASGRP3	2	33598552	4.60E-04	108	2.05E-01	Up	1.27	8303	3.34	0.07
rs1883767	7485	WRB	1	6109755	4.75E-04	109	3.73E-05	Down	4.12	2213	3.32	0.44
rs1883767	84181	CHD6	1	6109755	4.75E-04	110	2.66E-04	Up	3.65	2736	3.32	0.36
rs1883767	26038	CHD5	1	6109755	4.75E-04	111	3.82E-01	Down	0.87	9859	3.32	0.04
rs7386237	9705	ST18	8	53424589	4.90E-04	112	3.27E-01	Down	0.98	9419	3.31	0.05
rs1786045	3910	LAMA4	18	19791960	4.92E-04	113	5.01E-09	Down	5.85	1012	3.31	0.83
rs1786045	3909	LAMA3	18	19791960	4.92E-04	114	1.32E-07	Down	5.28	1322	3.31	0.69
rs17639988	266727	MDGA1	6	37788570	5.04E-04	115	4.95E-02	Down	1.96	5984	3.30	0.13
rs7537301	10451	VAV3	1	107909201	5.13E-04	116	5.44E-09	Down	5.83	1019	3.29	0.83
rs7153837	29091	STXBP6	14	24542222	5.20E-04	117	3.03E-01	Down	1.03	9211	3.28	0.05
rs10906142	57118	CAMK1D	10	12478789	5.22E-04	118	5.30E-01	Up	0.63	10941	3.28	0.03
rs13079094	165829	GPR156	3	121364778	5.23E-04	119	9.88E-01	Up	0.01	13832	3.28	0.00
rs10219	90957	DHX57	2	38995691	5.25E-04	120	9.89E-01	Up	0.01	13838	3.28	0.00
rs7617287	89876	C3orf15	3	120901221	5.36E-04	121	7.72E-02	Up	1.77	6579	3.27	0.11
rs12136553	149111	CNIH3	1	221172379	5.39E-04	122	2.59E-01	Up	1.13	8853	3.27	0.06
rs2824499	54149	C21orf91	21	18101142	5.40E-04	123	8.19E-02	Down	1.74	6666	3.27	0.11
rs4757842	89797	NAV2	11	19727630	5.49E-04	124	3.98E-06	Down	4.61	1782	3.26	0.54
rs9651966	5074	PAWR	12	78531206	5.49E-04	125	4.62E-02	Up	1.99	5898	3.26	0.13
rs11791662	138240	C9orf57	9	71924251	5.51E-04	126	4.31E-01	Down	0.79	10201	3.26	0.04
rs4926293	773	CACNA1A	19	13458003	5.58E-04	127	7.41E-01	Down	0.33	12322	3.25	0.01
rs9355655	56995	TULP4	6	158872218	5.64E-04	128	9.39E-03	Up	2.60	4462	3.25	0.20
rs6832007	54969	C4orf27	4	171032728	5.67E-04	129	3.41E-04	Up	3.58	2825	3.25	0.35
rs9525340	22821	RASA3	13	113817835	5.73E-04	130	5.38E-03	Down	2.78	4111	3.24	0.23
rs883011	114786	XKR4	8	56340679	5.73E-04	131	3.54E-01	Down	0.93	9633	3.24	0.05
rs10495376	56605	ERO1LB	1	232787151	5.75E-04	132	1.05E-01	Up	1.62	7070	3.24	0.10
rs9620752	23760	PITPNB	22	26649420	5.80E-04	133	8.43E-02	Down	1.73	6707	3.24	0.11
rs4924687	255252	LRRC57	15	40647883	5.84E-04	134	1.15E-01	Up	1.58	7213	3.23	0.09
rs9423702	5214	PFKP	10	3172686	5.85E-04	135	7.55E-03	Down	2.67	4307	3.23	0.21
rs9423702	10531	PITRM1	10	3172686	5.85E-04	136	1.11E-01	Up	1.59	7158	3.23	0.10
rs7109994	374378	GALNTL4	11	11273635	5.85E-04	137	1.28E-01	Up	1.52	7421	3.23	0.09
rs10417008	126129	CPT1C	19	54895365	5.85E-04	138	3.08E-01	Down	1.02	9246	3.23	0.05
rs1525608	3485	IGFBP2	2	217319261	5.86E-04	139	2.18E-04	Up	3.70	2661	3.23	0.37
rs465388	3842	TNPO1	5	72171655	5.94E-04	140	2.29E-11	Up	6.69	675	3.23	1.06
rs4257882	404672	GTF2H5	6	158592534	6.10E-04	141	3.28E-04	Up	3.59	2807	3.21	0.35
rs4627599	6869	TACR1	2	75318478	6.18E-04	142	9.77E-01	Up	0.03	13749	3.21	0.00
rs2017246	57158	JPH2	20	42209257	6.21E-04	143	1.59E-04	Down	3.78	2564	3.21	0.38
rs7814569	7163	TPD52	8	81252421	6.23E-04	144	1.36E-12	Up	7.29	513	3.21	1.19
rs897411	10656	KHDRBS3	8	136546450	6.27E-04	145	2.63E-04	Up	3.65	2733	3.20	0.36
rs10491412	3670	ISL1	5	50706878	6.38E-04	146	3.19E-07	Down	5.11	1423	3.20	0.65
rs1557530	4627	MYH9	22	35030122	6.38E-04	147	6.49E-02	Down	1.85	6340	3.20	0.12
rs17036007	50515	CHST11	12	103535048	6.39E-04	148	7.18E-01	Down	0.36	12185	3.19	0.01
rs1494813	25974	MMACHC	1	45626383	6.40E-04	149	7.22E-07	Up	4.96	1525	3.19	0.61
rs1494813	10420	TESK2	1	45626383	6.40E-04	150	2.87E-04	Up	3.63	2762	3.19	0.35
rs17087049	64078	SLC28A3	9	84143926	6.41E-04	151	8.69E-01	Down	0.17	13057	3.19	0.01
rs4557160	6476	SI	3	166230632	6.46E-04	152	1.97E-04	Up	3.72	2624	3.19	0.37
rs4557160	6490	SILV	3	166230632	6.46E-04	153	3.39E-01	Up	0.96	9516	3.19	0.05
rs9327012	57556	SEMA6A	5	115950568	6.48E-04	154	1.99E-02	Up	2.33	5069	3.19	0.17
rs16875084	170690	ADAMTS16	5	5277299	6.48E-04	155	6.19E-01	Up	0.50	11573	3.19	0.02
rs4147359	3559	IL2RA	10	6148445	6.49E-04	156	5.40E-03	Up	2.78	4113	3.19	0.23
rs11980456	1124	CHN2	7	29092489	6.50E-04	157	4.00E-10	Up	6.27	819	3.19	0.94

rs7203029	146279	TEKT5	16	10639820	6.55E-04	158	7.63E-01	Up	0.30	12456	3.18	0.01
rs12371422	10867	TSPAN9	12	3231207	6.60E-04	159	2.55E-01	Up	1.14	8807	3.18	0.06
rs17156416	221895	JAZF1	7	27934492	6.66E-04	160	1.54E-10	Down	6.40	780	3.18	0.98
rs185037	1258	CNGB1	16	56476268	6.69E-04	161	9.78E-01	Down	0.03	13756	3.17	0.00
rs11579488	149041	RC3H1	1	170673274	6.78E-04	162	2.36E-01	Up	1.18	8622	3.17	0.06
rs2043299	115727	RASGRP4	19	43612222	6.82E-04	163	2.33E-01	Down	1.19	8594	3.17	0.06
rs240073	9215	LARGE	22	32411048	6.90E-04	164	1.45E-12	Down	7.08	567	3.16	1.18
rs17025898	7399	USH2A	1	212592883	6.90E-04	165	4.17E-01	Up	0.81	10102	3.16	0.04
rs7159779	6547	SLC8A3	14	69730226	6.93E-04	166	5.27E-01	Up	0.63	10926	3.16	0.03
rs2420201	56603	CYP26B1	2	72268025	7.05E-04	167	1.65E-02	Down	2.40	4914	3.15	0.18
rs2836370	2078	ERG	21	38692234	7.12E-04	168	2.71E-37	Up	12.74	27	3.15	3.66
rs3755259	3241	HPCAL1	2	10516631	7.24E-04	169	2.30E-04	Up	3.68	2680	3.14	0.36
rs12734513	9928	KIF14	1	197258274	7.33E-04	170	4.64E-04	Up	3.50	2931	3.13	0.33
rs16829978	140609	NEK7	1	195017762	7.35E-04	171	6.97E-06	Down	4.49	1873	3.13	0.52
rs16849778	59352	LGR6	1	198938502	7.36E-04	172	9.05E-05	Down	3.91	2426	3.13	0.40
rs17834783	4335	MNT	17	2270267	7.36E-04	173	1.91E-01	Up	1.31	8158	3.13	0.07
rs17834783	79066	METT10D	17	2270267	7.36E-04	174	2.44E-01	Down	1.17	8702	3.13	0.06
rs1741490	6710	SPTB	14	64312821	7.46E-04	175	2.06E-05	Down	4.26	2097	3.13	0.47
rs291336	9759	HDAC4	2	239884293	7.46E-04	176	3.71E-03	Down	2.90	3887	3.13	0.24
rs7610584	5010	CLDN11	3	171635885	7.51E-04	177	4.19E-02	Down	2.03	5796	3.12	0.14
rs2485652	128209	KLF17	1	44264503	7.56E-04	178	9.35E-01	Up	0.08	13460	3.12	0.00
rs12608558	5794	PTPRH	19	60406902	7.71E-04	179	1.71E-02	Down	2.39	4940	3.11	0.18
rs221726	10846	PDE10A	6	165855338	7.73E-04	180	2.91E-01	Up	1.06	9121	3.11	0.05
rs1503839	25890	ABI3BP	3	101988749	7.85E-04	181	5.55E-02	Up	1.92	6126	3.11	0.13
rs1565948	203228	C9orf72	9	27549733	7.86E-04	182	2.40E-02	Up	2.26	5247	3.10	0.16
rs9874534	55799	CACNA2D3	3	54307410	7.87E-04	183	9.01E-01	Up	0.12	13246	3.10	0.00
rs13114243	3815	KIT	4	55455454	7.88E-04	184	7.68E-05	Down	3.95	2380	3.10	0.41
rs4933983	92211	PCDH21	10	85970875	7.93E-04	185	2.90E-01	Down	1.06	9115	3.10	0.05
rs2300613	2740	GLP1R	6	39157183	8.03E-04	186	6.41E-01	Down	0.47	11718	3.10	0.02
rs13134432	57620	STIM2	4	26655065	8.10E-04	187	3.58E-02	Down	2.10	5642	3.09	0.14
rs1558701	5212	VIT	2	36939470	8.12E-04	188	2.63E-04	Down	3.65	2732	3.09	0.36
rs11597689	657	BMPR1A	10	88639156	8.17E-04	189	1.06E-06	Down	4.88	1581	3.09	0.60
rs16990597	997	CDC34	19	489531	8.39E-04	190	4.18E-01	Down	0.81	10108	3.08	0.04
rs16990597	3004	GZMM	19	489531	8.39E-04	191	5.13E-01	Up	0.65	10796	3.08	0.03
rs6000199	80832	APOL4	22	34934432	8.44E-04	192	1.00E-01	Down	1.64	6997	3.07	0.10
rs1106042	9271	PIWIL1	12	129366518	8.45E-04	193	6.38E-02	Up	1.85	6319	3.07	0.12
rs418142	2533	FYB	5	39134560	8.49E-04	194	3.74E-02	Down	2.08	5687	3.07	0.14
rs4568818	6000	RGS7	1	237260878	8.51E-04	195	7.06E-01	Up	0.38	12104	3.07	0.02
rs4274307	10257	ABCC4	13	94478310	8.53E-04	196	3.71E-35	Up	12.38	40	3.07	3.44
rs1679012	25963	TMEM87A	15	40315968	8.55E-04	197	5.23E-10	Up	6.21	860	3.07	0.93
rs10504451	23213	SULF1	8	70626182	8.56E-04	198	8.34E-03	Down	2.64	4370	3.07	0.21
rs5945619	55190	NUDT11		51074708	8.56E-04	199	7.55E-01	Down	0.31	12411	3.07	0.01
rs12501287	6649	SOD3	4	24453251	8.57E-04	200	3.81E-10	Down	6.26	826	3.07	0.94
rs3804993	3708	ITPR1	3	4725674	8.58E-04	201	1.42E-13	Down	7.39	482	3.07	1.28
rs2560718	3295	HSD17B4	5	118914260	8.64E-04	202	9.45E-16	Up	7.90	377	3.06	1.50
rs5764983	23779	ARHGAP8	22	43466761	8.67E-04	203	7.79E-01	Up	0.28	12548	3.06	0.01
rs11113013	55703	POLR3B	12	105386947	8.68E-04	204	4.14E-01	Down	0.82	10086	3.06	0.04
rs38276	1607	DGKB	7	14078240	8.69E-04	205	4.15E-01	Up	0.82	10089	3.06	0.04
rs11111018	79158	GNPTAB	12	100672420	8.74E-04	206	2.35E-03	Down	3.04	3647	3.06	0.26
rs7091141	93550	ANUBL1	10	45468332	8.86E-04	207	1.84E-04	Up	3.74	2598	3.05	0.37
rs611060	1952	CELSR2	1	109494232	8.91E-04	208	2.73E-04	Down	3.64	2745	3.05	0.36
rs611060	6301	SARS	1	109494232	8.91E-04	209	3.01E-04	Down	3.61	2774	3.05	0.35
rs611060	54938	SARS2	1	109494232	8.91E-04	210	6.09E-01	Down	0.51	11492	3.05	0.02
rs6102912	11122	PTPRT	20	40636349	8.93E-04	211	1.98E-09	Up	6.00	937	3.05	0.87
rs7873986	116443	GRIN3A	9	101417860	8.93E-04	212	7.06E-01	Up	0.38	12105	3.05	0.02
rs1004968	121512	FGD4	12	32654741	8.95E-04	213	2.07E-06	Down	4.75	1675	3.05	0.57
rs1921564	133121	ENPP6	4	185453225	8.97E-04	214	3.92E-01	Down	0.86	9926	3.05	0.04
rs7022613	10670	RRAGA	9	19041208	9.08E-04	215	2.11E-07	Down	5.19	1380	3.04	0.67
rs7022613	54801	FAM29A	9	19041208	9.08E-04	216	4.71E-01	Down	0.72	10502	3.04	0.03
rs2107331	7045	TGFBI	5	135405248	9.10E-04	217	5.54E-02	Down	1.92	6125	3.04	0.13
rs10779353	1379	CR1L	1	204225985	9.10E-04	218	5.53E-01	Down	0.59	11097	3.04	0.03
rs13007683	151531	UPP2	2	158721163	9.16E-04	219	6.91E-01	Up	0.40	12016	3.04	0.02
rs9359124	27145	FILIP1	6	76257234	9.18E-04	220	3.99E-04	Down	3.54	2882	3.04	0.34
rs8027126	641	BLM	15	89138090	9.23E-04	221	8.99E-02	Up	1.70	6808	3.03	0.10
rs2014725	11240	PADI2	1	17162559	9.27E-04	222	4.26E-03	Down	2.86	3974	3.03	0.24
rs6945128	9069	CLDN12	7	89667730	9.28E-04	223	7.04E-02	Up	1.81	6438	3.03	0.12
rs13032262	1838	DTNB	2	25557972	9.29E-04	224	5.56E-01	Up	0.59	11122	3.03	0.03
rs4664106	114793	FMNL2	2	153067687	9.32E-04	225	1.66E-02	Up	2.39	4919	3.03	0.18
rs2285550	8450	CUL4B		119477506	9.34E-04	226	9.77E-02	Up	1.66	6957	3.03	0.10
rs4297845	2571	GAD1	2	171545046	9.35E-04	227	8.64E-04	Up	3.33	3162	3.03	0.31
rs10518245	9667	SAFB2	19	5576802	9.37E-04	228	7.32E-02	Up	1.79	6489	3.03	0.11
rs10518245	6294	SAFB	19	5576802	9.37E-04	229	9.10E-01	Down	0.11	13310	3.03	0.00
rs2467307	9135	RABEP1	17	5109042	9.38E-04	230	1.80E-03	Up	3.12	3477	3.03	0.27
rs2356559	5052	PRDX1	1	45650605	9.41E-04	231	4.20E-04	Down	3.53	2898	3.03	0.34
rs6061598	1002	CDH4	20	59537111	9.44E-04	232	1.48E-01	Up	1.45	7648	3.03	0.08
rs1168671	54509	RHOF	12	120692568	9.55E-04	233	5.26E-02	Down	1.94	6050	3.02	0.13
rs1168671	23067	SETD1B	12	120692568	9.55E-04	234	5.44E-01	Down	0.61	11046	3.02	0.03
rs1321329	221264	C6orf199	6	110087489	9.55E-04	235	9.61E-01	Up	0.05	13628	3.02	0.00
rs12515835	23286	WWC1	5	167824916	9.59E-04	236	1.40E-08	Up	5.66	1089	3.02	0.79
rs3826628	81035	COLEC12	18	336821	9.62E-04	237	3.58E-08	Up	5.51	1189	3.02	0.74
rs7902783	57512	GPR158	10	25513753	9.62E-04	238	3.15E-01	Down	1.00	9323	3.02	0.05

rs838989	90874	ZNF697	1	119894948	9.64E-04	239	1.78E-05	Up	4.29	2068	3.02	0.48
rs17024584	4853	NOTCH2	1	120223428	9.65E-04	240	1.91E-04	Up	3.73	2612	3.02	0.37
rs10090395	286	ANK1	8	41786031	9.73E-04	241	1.03E-01	Down	1.63	7051	3.01	0.10
rs4973893	54986	ULK4	3	41811474	9.74E-04	242	1.52E-02	Down	2.43	4821	3.01	0.18
rs4902815	55333	SYNJ2BP	14	69950782	9.76E-04	243	5.75E-26	Up	10.55	97	3.01	2.52
rs1859804	9771	RAPGEF5	7	21942318	9.81E-04	244	3.63E-01	Up	0.91	9700	3.01	0.04
rs3777018	7518	XRCC4	5	82662675	9.85E-04	245	1.12E-01	Up	1.59	7185	3.01	0.09
rs17416794	83479	DDX59	1	197349190	9.87E-04	246	2.57E-02	Up	2.23	5310	3.01	0.16
rs1960385	79625	C4orf31	4	122361857	9.91E-04	247	2.43E-06	Down	4.71	1698	3.00	0.56
rs4723979	79783	C7orf10	7	40641381	9.99E-04	248	4.64E-01	Down	0.73	10454	3.00	0.03
rs216666	1496	CTNNA2	2	80745015	1.01E-03	249	2.33E-01	Up	1.19	8588	3.00	0.06
rs877633	6907	TBL1X		9407521	1.01E-03	250	2.17E-16	Down	8.21	335	3.00	1.57
rs7665597	987	LRBA	4	152293754	1.01E-03	251	4.07E-09	Up	5.88	994	3.00	0.84
rs2642627	366	AQP9	15	56205187	1.02E-03	252	8.27E-03	Down	2.64	4366	2.99	0.21
rs2642627	364	AQP7	15	56205187	1.02E-03	253	1.80E-01	Up	1.34	8028	2.99	0.07
rs1584010	8424	BBOX1	11	27111429	1.03E-03	254	3.28E-01	Up	0.98	9426	2.99	0.05
rs8031050	51285	RASL12	15	63122916	1.04E-03	255	2.30E-03	Down	3.05	3627	2.98	0.26
rs8031050	123263	MTFMT	15	63122916	1.04E-03	256	6.33E-02	Down	1.86	6307	2.98	0.12
rs994823	7291	TWIST1	7	18938504	1.04E-03	257	4.93E-13	Up	7.23	529	2.98	1.23
rs4682542	26137	ZBTB20	3	115729815	1.04E-03	258	3.08E-10	Down	6.29	811	2.98	0.95
rs7633966	4921	DDR2	3	53264567	1.05E-03	259	1.82E-33	Down	12.06	48	2.98	3.27
rs7633966	7086	TKT	3	53264567	1.05E-03	260	2.29E-09	Up	5.98	942	2.98	0.86
rs7329659	10160	FARP1	13	97872564	1.05E-03	261	3.56E-04	Up	3.57	2842	2.98	0.34
rs3134711	131450	CD200R1	6	55211851	1.06E-03	262	5.80E-03	Up	2.76	4159	2.98	0.22
rs10492388	10138	YAF2	12	40894268	1.06E-03	263	9.79E-02	Down	1.65	6962	2.98	0.10
rs3134711	3062	HCRTR2	6	55211851	1.06E-03	264	3.05E-01	Down	1.03	9232	2.98	0.05
rs7630511	86	ACTL6A	3	180755183	1.07E-03	265	3.24E-07	Up	5.11	1426	2.97	0.65
rs800870	7227	TRPS1	8	116555618	1.08E-03	266	5.05E-15	Down	7.83	389	2.96	1.43
rs11991141	157807	RLBP1L1	8	62531379	1.11E-03	267	7.98E-01	Down	0.26	12662	2.96	0.01
rs3850389	54207	KCNK10	14	87857231	1.11E-03	268	2.35E-01	Down	1.19	8615	2.96	0.06
rs2453451	168975	CNBD1	8	87947962	1.11E-03	269	5.40E-01	Up	0.61	11008	2.95	0.03
rs4682284	10225	CD96	3	112732005	1.12E-03	270	6.70E-01	Down	0.43	11887	2.95	0.02
rs12046258	9580	SOX13	1	200770817	1.12E-03	271	4.20E-03	Down	2.86	3965	2.95	0.24
rs13117055	5522	PPP2R2C	4	6605798	1.14E-03	272	8.49E-01	Up	0.19	12947	2.94	0.01
rs4757276	9645	MICAL2	11	12216156	1.14E-03	273	8.32E-11	Up	6.49	749	2.94	1.01
rs2957090	9228	DLGAP2	8	1555327	1.16E-03	274	3.11E-01	Up	1.01	9274	2.94	0.05
rs16977161	6014	RIT2	18	38799120	1.17E-03	275	2.20E-02	Up	2.29	5160	2.93	0.17
rs7855669	7111	TMOD1	9	97390266	1.17E-03	276	3.18E-03	Down	2.95	3812	2.93	0.25
rs1284442	753	C18orf1	18	13585055	1.17E-03	277	6.55E-01	Up	0.45	11795	2.93	0.02
rs10124289	4781	NFIB	9	14282640	1.17E-03	278	1.47E-01	Down	1.45	7645	2.93	0.08
rs10455038	5480	PPIC	5	122395975	1.18E-03	279	1.33E-01	Down	1.50	7482	2.93	0.09
rs13203608	93663	ARHGAP18	6	130035588	1.19E-03	280	3.02E-01	Up	1.03	9206	2.92	0.05
rs3770585	8647	ABCB11	2	169649374	1.20E-03	281	6.89E-01	Down	0.40	12000	2.92	0.02
rs1569942	25943	C20orf194	20	3184015	1.20E-03	282	4.94E-06	Down	4.57	1814	2.92	0.53
rs1569942	83959	SLC4A11	20	3184015	1.20E-03	283	7.23E-01	Down	0.35	12219	2.92	0.01
rs1820253	170692	ADAMTS18	16	75876072	1.20E-03	284	2.15E-01	Down	1.24	8417	2.92	0.07
rs11580456	60676	PAPPA2	1	173178051	1.20E-03	285	9.70E-01	Up	0.04	13702	2.92	0.00
rs7026686	51116	MRPS2	9	135609158	1.20E-03	286	7.38E-10	Up	6.15	876	2.92	0.91
rs7026686	9858	KIAA0649	9	135609158	1.20E-03	287	1.12E-03	Up	3.26	3263	2.92	0.30
rs7026686	138162	C9orf116	9	135609158	1.20E-03	288	1.62E-01	Up	1.40	7838	2.92	0.08
rs2149833	64067	NPAS3	14	33121283	1.20E-03	289	9.42E-01	Down	0.07	13507	2.92	0.00
rs2526105	221981	THSD7A	7	11430451	1.21E-03	290	5.21E-03	Up	2.79	4095	2.92	0.23
rs746677	6623	SNCG	10	88690618	1.21E-03	291	3.61E-05	Down	4.13	2209	2.92	0.44
rs746677	79812	MMRN2	10	88690618	1.21E-03	292	5.93E-01	Down	0.53	11394	2.92	0.02
rs13112390	55247	NEIL3	4	178649984	1.21E-03	293	1.07E-02	Up	2.55	4560	2.92	0.20
rs1451538	9154	SLC28A1	15	83265634	1.22E-03	294	2.55E-01	Down	1.14	8806	2.91	0.06
rs10818700	26740	OR1J2	9	122320180	1.22E-03	295	8.68E-01	Down	0.17	13053	2.91	0.01
rs6478233	5069	PAPPA	9	116214429	1.22E-03	296	1.45E-03	Up	3.18	3362	2.91	0.28
rs7170993	9236	CCPG1	15	53475261	1.22E-03	297	3.06E-01	Up	1.02	9238	2.91	0.05
rs1349344	4212	MEIS2	15	34987349	1.23E-03	298	1.44E-37	Down	12.81	24	2.91	3.68
rs2903582	64409	WBSCR17	7	70494307	1.23E-03	299	9.01E-01	Down	0.12	13250	2.91	0.00
rs4676593	6336	SCN10A	3	38817916	1.24E-03	300	1.01E-01	Down	1.64	7005	2.91	0.10
rs513287	5396	PRRX1	1	167395895	1.25E-03	301	9.13E-06	Down	4.44	1929	2.90	0.50
rs3444	79639	TMEM53	1	44789058	1.25E-03	302	1.18E-02	Down	2.52	4623	2.90	0.19
rs7164787	57538	ALPK3	15	83180500	1.26E-03	303	8.07E-01	Down	0.24	12709	2.90	0.01
rs1558134	1943	EFNA2	19	1259057	1.27E-03	304	2.35E-01	Up	1.19	8616	2.90	0.06
rs2304790	8895	CPNE3	8	87636147	1.28E-03	305	2.03E-04	Up	3.72	2635	2.89	0.37
rs9628	55802	DCP1A	3	53296671	1.28E-03	306	6.21E-01	Down	0.50	11583	2.89	0.02
rs3172604	55330	CNO	4	6836847	1.29E-03	307	1.57E-01	Up	1.41	7775	2.89	0.08
rs2297325	56243	KIAA1217	10	24863082	1.29E-03	308	1.62E-07	Down	5.24	1350	2.89	0.68
rs240734	1303	COL12A1	6	75903756	1.30E-03	309	2.49E-05	Up	4.22	2129	2.89	0.46
rs4075663	84867	PTPN5	11	18749975	1.31E-03	310	1.77E-02	Up	2.37	4970	2.88	0.18
rs16830730	2932	GSK3B	3	121311202	1.32E-03	311	3.98E-10	Up	6.25	837	2.88	0.94
rs16911799	7003	TEAD1	11	12907129	1.33E-03	312	1.15E-05	Down	4.39	1976	2.87	0.49
rs3736360	3339	HSPG2	1	21895426	1.34E-03	313	9.66E-03	Down	2.59	4483	2.87	0.20
rs7945127	54503	ZDHHC13	11	19084800	1.34E-03	314	8.50E-01	Down	0.19	12955	2.87	0.01
rs3803761	201163	FLCN	17	17057137	1.36E-03	315	6.88E-01	Down	0.40	11993	2.87	0.02
rs10898071	27314	RAB30	11	82405539	1.37E-03	316	3.63E-03	Up	2.91	3877	2.86	0.24
rs11880474	147657	ZNF480	19	57498124	1.37E-03	317	6.04E-04	Up	3.43	3029	2.86	0.32
rs2839127	84221	C21orf56	21	46397978	1.38E-03	318	2.88E-01	Up	1.06	9099	2.86	0.05
rs2839127	10841	FTCD	21	46397978	1.38E-03	319	3.14E-01	Up	1.01	9311	2.86	0.05

rs1931745	1793	DOCK1	10	129134971	1.38E-03	320	5.64E-07	Down	5.00	1498	2.86	0.62
rs10090810	64478	CSMD1	8	3353711	1.38E-03	321	9.02E-02	Up	1.69	6823	2.86	0.10
rs11064342	8079	MLF2	12	6718070	1.38E-03	322	3.00E-01	Up	1.04	9190	2.86	0.05
rs11064342	50813	COPS7A	12	6718070	1.38E-03	323	4.33E-01	Down	0.78	10215	2.86	0.04
rs3093040	1435	CSF1	1	110182466	1.39E-03	324	2.05E-01	Down	1.27	8306	2.86	0.07
rs13390393	253782	LASS6	2	169424936	1.39E-03	325	4.17E-16	Up	8.14	344	2.86	1.54
rs17053477	6444	SGCD	5	155724992	1.39E-03	326	3.12E-03	Down	2.96	3797	2.86	0.25
rs7660583	23240	KIAA0922	4	154829533	1.39E-03	327	2.06E-01	Down	1.26	8316	2.86	0.07
rs2991966	10327	AKR1A1	1	45668596	1.40E-03	328	1.76E-10	Up	6.44	763	2.86	0.98
rs11706137	254827	NAALADL2	3	176448133	1.40E-03	329	2.39E-01	Down	1.18	8651	2.86	0.06
rs8021668	4293	MAP3K9	14	70258350	1.43E-03	330	3.50E-01	Up	0.93	9588	2.85	0.05
rs12593461	89978	ATPBD4	15	33613069	1.43E-03	331	1.94E-07	Up	5.21	1369	2.85	0.67
rs10518156	57619	SHROOM3	4	78052283	1.43E-03	332	5.20E-04	Up	3.47	2971	2.85	0.33
rs1328710	22999	RIMS1	6	72659285	1.43E-03	333	4.08E-03	Up	2.87	3943	2.85	0.24
rs11191229	9221	NOLC1	10	103921921	1.43E-03	334	3.87E-01	Down	0.86	9894	2.85	0.04
rs9959147	1828	DSG1	18	27141088	1.43E-03	335	7.19E-01	Down	0.36	12195	2.84	0.01
rs6108263	5332	PLCB4	20	9059633	1.44E-03	336	1.04E-12	Up	7.11	559	2.84	1.20
rs6131519	55968	NSFL1C	20	1376567	1.44E-03	337	8.40E-06	Down	4.45	1914	2.84	0.51
rs10261505	1804	DPP6	7	153935783	1.46E-03	338	2.66E-01	Up	1.11	8917	2.84	0.06
rs16851303	214	ALCAM	3	106760324	1.46E-03	339	1.82E-22	Up	9.73	154	2.84	2.17
rs16933155	83938	C10orf11	10	77708012	1.47E-03	340	8.97E-01	Up	0.13	13227	2.83	0.00
rs718744	160492	IFLTD1	12	25548787	1.48E-03	341	2.93E-01	Up	1.05	9139	2.83	0.05
rs820361	4638	MYLK	3	124881758	1.49E-03	342	5.08E-26	Down	10.55	98	2.83	2.53
rs9633646	783	CACNB2	10	18604928	1.49E-03	343	1.20E-05	Down	4.38	1991	2.83	0.49
rs7213035	22843	PPM1E	17	54212280	1.50E-03	344	2.02E-08	Up	5.61	1121	2.83	0.77
rs12014135	1756	DMD		31671065	1.50E-03	345	2.07E-08	Down	5.61	1129	2.82	0.77
rs9889631	284083	C17orf47	17	53985358	1.53E-03	346	5.70E-01	Up	0.57	11239	2.82	0.02
rs9889631	56155	TEX14	17	53985358	1.53E-03	347	9.79E-01	Down	0.03	13768	2.82	0.00
rs7842128	1808	DPYSL2	8	26574697	1.53E-03	348	4.57E-08	Down	5.47	1214	2.81	0.73
rs1431486	119548	PNLIPRP3	10	118183122	1.54E-03	349	1.10E-01	Up	1.60	7150	2.81	0.10
rs1320042	143425	SYT9	11	7400603	1.54E-03	350	2.43E-02	Down	2.25	5262	2.81	0.16
rs17699658	27086	FOXP1	3	71338999	1.54E-03	351	5.29E-01	Up	0.63	10936	2.81	0.03
rs2169455	23424	TDRD7	9	97241972	1.56E-03	352	8.29E-06	Down	4.46	1910	2.81	0.51
rs10798959	114784	CSMD2	1	33652112	1.57E-03	353	5.03E-02	Up	1.96	6006	2.81	0.13
rs4756226	6506	SLC1A2	11	35361510	1.57E-03	354	1.32E-02	Up	2.48	4705	2.80	0.19
rs7158848	10001	MED6	14	70131709	1.59E-03	355	4.23E-04	Down	3.53	2899	2.80	0.34
rs11245016	118611	C10orf90	10	128202819	1.60E-03	356	8.76E-05	Down	3.92	2418	2.80	0.41
rs11709247	55	ACPP	3	133513756	1.61E-03	357	5.65E-01	Up	0.58	11201	2.79	0.02
rs2119758	5101	PCDH9	13	66331636	1.62E-03	358	5.61E-02	Down	1.91	6140	2.79	0.13
rs734028	57475	PLEKHH1	14	67123555	1.66E-03	359	1.59E-08	Up	5.65	1097	2.78	0.78
rs734028	5283	PIGH	14	67123555	1.66E-03	360	2.20E-03	Up	3.06	3596	2.78	0.27
rs7934514	53942	CNTN5	11	99214368	1.68E-03	361	1.75E-04	Up	3.75	2583	2.77	0.38
rs601902	1718	DHCR24	1	55062044	1.69E-03	362	3.04E-05	Up	4.17	2173	2.77	0.45
rs13183577	134353	LSM11	5	157107238	1.70E-03	363	7.47E-03	Down	2.67	4301	2.77	0.21
rs13183577	54974	THG1L	5	157107238	1.70E-03	364	4.46E-01	Down	0.76	10319	2.77	0.04
rs8033723	5046	PCSK6	15	99863100	1.70E-03	365	2.78E-14	Up	7.61	436	2.77	1.36
rs933843	4026	LPP	3	189723817	1.70E-03	366	1.40E-15	Down	7.99	362	2.77	1.49
rs4386077	56204	KIAA1370	15	50741225	1.70E-03	367	2.02E-05	Up	4.26	2088	2.77	0.47
rs7106026	57689	LRRC4C	11	40177990	1.72E-03	368	9.82E-01	Up	0.02	13793	2.76	0.00
rs10831706	430	ASCL2	11	2262602	1.72E-03	369	7.86E-01	Up	0.27	12585	2.76	0.01
rs9457304	83887	TTLL2	6	167725132	1.73E-03	370	9.18E-02	Up	1.69	6855	2.76	0.10
rs3747502	57653	KIAA1529	9	97156451	1.74E-03	371	2.48E-01	Up	1.15	8753	2.76	0.06
rs3761646	64219	PJA1		68164933	1.74E-03	372	1.63E-08	Down	5.65	1099	2.76	0.78
rs4328074	1380	CR2	1	204035318	1.75E-03	373	4.77E-01	Up	0.71	10555	2.76	0.03
rs12281674	9479	MAPK8IP1	11	45856538	1.76E-03	374	3.72E-02	Up	2.08	5682	2.76	0.14
rs12281674	1408	CRY2	11	45856538	1.76E-03	375	3.38E-01	Down	0.96	9508	2.76	0.05
rs9535486	220107	DLEU7	13	50277766	1.76E-03	376	7.69E-01	Down	0.29	12485	2.75	0.01
rs2166703	26151	NAT9	17	70293407	1.77E-03	377	6.00E-02	Up	1.88	6236	2.75	0.12
rs2166703	54868	TMEM104	17	70293407	1.77E-03	378	8.42E-01	Down	0.20	12915	2.75	0.01
rs574700	2206	MS4A2	11	59612059	1.77E-03	379	7.40E-04	Down	3.37	3100	2.75	0.31
rs574700	931	MS4A1	11	59612059	1.77E-03	380	1.16E-01	Up	1.57	7227	2.75	0.09
rs9916423	5889	RAD51C	17	54128833	1.78E-03	381	2.08E-01	Up	1.26	8335	2.75	0.07
rs4930941	28957	MRPS28	12	27793799	1.80E-03	382	3.65E-06	Up	4.63	1771	2.75	0.54
rs4930941	60488	MRPS35	12	27793799	1.80E-03	383	2.10E-03	Up	3.08	3564	2.75	0.27
rs10305860	1909	EDNRA	4	148763492	1.81E-03	384	1.39E-16	Down	8.27	330	2.74	1.59
rs4941462	55068	ENOX1	13	43040022	1.81E-03	385	8.00E-04	Up	3.35	3139	2.74	0.31
rs3731037	26018	LRIG1	19	53315036	1.83E-03	386	1.23E-11	Up	6.78	642	2.74	1.09
rs3731037	3978	LIG1	19	53315036	1.83E-03	387	9.06E-03	Up	2.61	4439	2.74	0.20
rs3731037	8605	PLA2G4C	19	53315036	1.83E-03	388	9.27E-01	Up	0.09	13411	2.74	0.00
rs11245450	54764	ZRANB1	10	126648065	1.84E-03	389	1.53E-01	Down	1.43	7707	2.74	0.08
rs13241957	9734	HDAC9	7	18581636	1.84E-03	390	8.39E-05	Up	3.93	2408	2.73	0.41
rs12590404	87	ACTN1	14	68401406	1.86E-03	391	9.32E-29	Down	11.13	73	2.73	2.80
rs973837	220108	FAM124A	13	50737211	1.87E-03	392	3.17E-04	Down	3.60	2795	2.73	0.35
rs150385	23327	NEDD4L	18	54063403	1.89E-03	393	1.70E-18	Up	8.77	253	2.72	1.78
rs201395	56899	ANKS1B	12	97738107	1.89E-03	394	8.42E-02	Up	1.73	6703	2.72	0.11
rs1936158	3351	HTR1B	6	78241094	1.90E-03	395	7.20E-01	Up	0.36	12196	2.72	0.01
rs9397722	81706	PPP1R14C	6	150594161	1.90E-03	396	8.21E-01	Up	0.23	12798	2.72	0.01
rs7099903	288	ANK3	10	61451727	1.91E-03	397	1.09E-24	Up	10.24	119	2.72	2.40
rs7894429	282974	STK32C	10	133974861	1.91E-03	398	4.44E-01	Down	0.77	10297	2.72	0.04
rs201124	10659	CUGBP2	10	11093055	1.91E-03	399	1.81E-12	Down	7.05	575	2.72	1.17
rs10950875	8701	DNAH11	7	21608311	1.92E-03	400	2.45E-04	Down	3.67	2702	2.72	0.36

rs865403	57451	ODZ2	5	167291366	1.92E-03	401	2.06E-08	Down	5.61	1128	2.72	0.77
rs900253	114134	SLC2A13	12	38789012	1.93E-03	402	9.58E-02	Up	1.67	6923	2.72	0.10
rs710649	27345	KCNMB4	12	69084610	1.95E-03	403	6.07E-01	Up	0.51	11484	2.71	0.02
rs1048084	84913	ATOH8	2	85913961	1.95E-03	404	2.97E-05	Down	4.18	2169	2.71	0.45
rs2564489	147372	CCBE1	18	55390977	1.96E-03	405	5.66E-01	Down	0.57	11213	2.71	0.02
rs9298497	6917	TCEA1	8	55098835	1.96E-03	406	4.30E-01	Down	0.79	10194	2.71	0.04
rs11626007	114088	TRIM9	14	50625582	1.97E-03	407	8.63E-01	Down	0.17	13024	2.71	0.01
rs12031180	23089	PEG10	1	207041080	1.97E-03	408	3.44E-05	Down	4.14	2196	2.71	0.45
rs12031180	55733	HHAT	1	207041080	1.97E-03	409	6.20E-01	Down	0.50	11581	2.71	0.02
rs1948158	22871	NLGN1	3	175035484	1.97E-03	410	1.08E-01	Down	1.61	7120	2.70	0.10
rs4571590	10345	TRDN	6	123829931	1.99E-03	411	1.62E-02	Up	2.40	4898	2.70	0.18
rs2053872	4659	PPP1R12A	12	78676705	1.99E-03	412	1.00E-19	Down	9.09	208	2.70	1.90
rs5030472	7189	TRAF6	11	36470362	1.99E-03	413	2.66E-02	Down	2.22	5344	2.70	0.16
rs2254715	5253	PHF2	9	93443255	2.01E-03	414	9.99E-02	Down	1.65	6996	2.70	0.10
rs3814772	78998	C8orf51	8	144536428	2.03E-03	415	3.29E-02	Up	2.13	5555	2.69	0.15
rs3814772	114822	RHPN1	8	144536428	2.03E-03	416	1.70E-01	Down	1.37	7921	2.69	0.08
rs3748462	140710	C20orf117	20	34941149	2.04E-03	417	5.47E-02	Up	1.92	6107	2.69	0.13
rs10062990	81792	ADAMTS12	5	33721620	2.04E-03	418	2.26E-02	Down	2.28	5183	2.69	0.16
rs2453666	6674	SPAG1	8	101283009	2.04E-03	419	1.57E-01	Down	1.42	7767	2.69	0.08
rs2173049	6497	SKI	1	2272168	2.04E-03	420	4.35E-01	Up	0.78	10232	2.69	0.04
rs6555491	108	ADCY2	5	7799792	2.05E-03	421	5.07E-09	Down	5.84	1014	2.69	0.83
rs11244795	8038	ADAM12	10	127744276	2.05E-03	422	2.43E-04	Down	3.67	2698	2.69	0.36
rs319405	5205	ATP8B1	18	53526813	2.05E-03	423	4.98E-01	Up	0.68	10699	2.69	0.03
rs12930202	56942	C16orf61	16	79566263	2.06E-03	424	2.75E-01	Up	1.09	8991	2.69	0.06
rs10941251	79925	SPEF2	5	35651758	2.07E-03	425	9.01E-04	Up	3.32	3175	2.68	0.30
rs1050395	490	ATP2B1	12	88531369	2.07E-03	426	7.28E-10	Up	6.15	874	2.68	0.91
rs10952891	2913	GRM3	7	86111902	2.07E-03	427	2.33E-01	Down	1.19	8587	2.68	0.06
rs12950972	114757	CYGB	17	72030895	2.07E-03	428	5.78E-01	Up	0.56	11296	2.68	0.02
rs1835950	5521	PPP2R2B	5	146234024	2.08E-03	429	6.10E-02	Down	1.87	6256	2.68	0.12
rs3072	60526	C2orf43	2	20800034	2.08E-03	430	5.64E-17	Down	8.37	312	2.68	1.62
rs11165115	5708	PSMD2	1	91813056	2.09E-03	431	2.93E-03	Down	2.98	3764	2.68	0.25
rs11211654	7173	TPO	2	1453138	2.10E-03	432	6.41E-01	Up	0.47	11720	2.68	0.02
rs11211654	7066	THPO	2	1453138	2.10E-03	433	6.44E-01	Up	0.46	11743	2.68	0.02
rs3800993	8972	MGAM	7	141254191	2.10E-03	434	5.15E-01	Up	0.65	10821	2.68	0.03
rs10870295	80313	LRRC27	10	134025568	2.11E-03	435	7.61E-02	Down	1.77	6549	2.68	0.11
rs2884800	57537	SORCS2	4	7746477	2.12E-03	436	5.96E-03	Down	2.75	4168	2.67	0.22
rs9533317	94240	EPSTI1	13	42419227	2.12E-03	437	2.46E-03	Down	3.03	3673	2.67	0.26
rs460879	1800	DPEP1	16	88240390	2.13E-03	438	6.91E-02	Down	1.82	6414	2.67	0.12
rs460879	124045	C16orf55	16	88240390	2.13E-03	439	3.45E-01	Up	0.95	9556	2.67	0.05
rs7605321		B3GNT1	2	62343784	2.13E-03	440	7.56E-03	Down	2.67	4313	2.67	0.21
rs17535387	148281	SYT6	1	114425401	2.14E-03	441	5.45E-01	Down	0.60	11051	2.67	0.03
rs12738895	23261	CAMTA1	1	7027479	2.14E-03	442	7.14E-01	Down	0.37	12157	2.67	0.01
rs17116806	1806	DPYD	1	97685273	2.14E-03	443	3.41E-06	Down	4.64	1759	2.67	0.55
rs4095095	2917	GRM7	3	7284658	2.15E-03	444	9.10E-01	Down	0.11	13305	2.67	0.00
rs7138514	6857	SYT1	12	78297026	2.15E-03	445	8.85E-05	Down	3.92	2422	2.67	0.41
rs4777585	4756	NEO1	15	71162900	2.15E-03	446	3.67E-01	Up	0.90	9735	2.67	0.04
rs2237468	2887	GRB10	7	50508571	2.15E-03	447	3.20E-03	Down	2.95	3817	2.67	0.25
rs10508489	8516	ITGA8	10	15702396	2.16E-03	448	4.99E-08	Down	5.45	1222	2.67	0.73
rs2208370	26052	DNM3	1	168686988	2.16E-03	449	4.37E-01	Down	0.78	10246	2.67	0.04
rs11641723	9956	HS3ST2	16	22778813	2.17E-03	450	5.24E-02	Down	1.94	6046	2.66	0.13
rs10952739	26047	CNTNAP2	7	147395782	2.17E-03	451	3.54E-04	Up	3.57	2840	2.66	0.35
rs4113643	3756	KCNH1	1	207398316	2.19E-03	452	1.02E-01	Down	1.64	7033	2.66	0.10
rs7789699	51422	PRKAG2	7	150928784	2.19E-03	453	2.15E-01	Up	1.24	8410	2.66	0.07
rs2839512	53347	UBASH3A	21	42725229	2.21E-03	454	2.81E-01	Down	1.08	9040	2.66	0.06
rs16830318	4249	MGAT5	2	134870605	2.21E-03	455	5.79E-01	Down	0.55	11308	2.66	0.02
rs7020887	51450	PRRX2	9	129496390	2.21E-03	456	3.24E-01	Down	0.99	9399	2.66	0.05
rs7020887	140459	ASB6	9	129496390	2.21E-03	457	6.34E-01	Down	0.48	11665	2.66	0.02
rs966866	64084	CLSTN2	3	141164746	2.22E-03	458	4.28E-01	Down	0.79	10174	2.65	0.04
rs12331393	2121	EVC	4	5877419	2.24E-03	459	4.14E-01	Down	0.82	10080	2.65	0.04
rs3737717	54973	CPSF3L	1	1282007	2.25E-03	460	3.91E-04	Up	3.55	2874	2.65	0.34
rs3737717	126789	PUSL1	1	1282007	2.25E-03	461	9.84E-01	Up	0.02	13810	2.65	0.00
rs7855377	81704	DOCK8	9	339518	2.25E-03	462	2.16E-04	Down	3.70	2654	2.65	0.37
rs2241868	3158	HMGCS2	1	120005211	2.25E-03	463	4.53E-01	Down	0.75	10363	2.65	0.03
rs2241868	26227	PHGDH	1	120005211	2.25E-03	464	5.40E-01	Down	0.61	11013	2.65	0.03
rs2432552	1459	CSNK2A2	16	56791957	2.26E-03	465	4.55E-02	Down	2.00	5884	2.65	0.13
rs9640291	155051	CRYGN	7	150590313	2.26E-03	466	9.39E-01	Up	0.08	13490	2.65	0.00
rs10088006	3037	HAS2	8	122734302	2.26E-03	467	5.67E-01	Up	0.57	11219	2.65	0.02
rs7297961	6337	SCNN1A	12	6324558	2.26E-03	468	2.30E-12	Down	7.01	589	2.65	1.16
rs7297961	7132	TNFRSF1A	12	6324558	2.26E-03	469	1.36E-08	Down	5.68	1082	2.65	0.79
rs10997528	29119	CTNNA3	10	68571228	2.27E-03	470	9.66E-01	Up	0.04	13671	2.64	0.00
rs11623091	2009	EML1	14	99314706	2.28E-03	471	5.16E-01	Up	0.65	10826	2.64	0.03
rs4345583	114788	CSMD3	8	113759975	2.28E-03	472	9.89E-01	Up	0.01	13834	2.64	0.00
rs7521729	25790	CCDC19	1	156657892	2.28E-03	473	5.42E-02	Down	1.92	6095	2.64	0.13
rs3093390	2975	GTF3C1	16	27370949	2.28E-03	474	3.32E-05	Up	4.15	2187	2.64	0.45
rs3093390	50615	IL21R	16	27370949	2.28E-03	475	4.03E-01	Up	0.84	10002	2.64	0.04
rs11130274	1795	DOCK3	3	51138936	2.30E-03	476	3.19E-03	Down	2.95	3815	2.64	0.25
rs17332226	9844	ELMO1	7	36695526	2.31E-03	477	2.25E-03	Down	3.05	3614	2.64	0.26
rs3181121	953	ENTPD1	10	97608570	2.31E-03	478	2.55E-05	Down	4.21	2137	2.64	0.46
rs4266383	23440	OTP	5	76985514	2.32E-03	479	9.38E-02	Down	1.68	6898	2.64	0.10
rs11157874	22795	NID2	14	51611293	2.32E-03	480	1.15E-09	Down	6.09	902	2.63	0.89
rs12237054	19	ABCA1	9	104756495	2.33E-03	481	1.39E-02	Up	2.46	4752	2.63	0.19

rs1409165	5733	PTGER3	1	71076126	2.34E-03	482	3.34E-03	Down	2.93	3834	2.63	0.25
rs2253971	221223	CES7	16	54468305	2.36E-03	483	1.29E-01	Down	1.52	7444	2.63	0.09
rs868824	83943	IMMP2L	7	109985872	2.39E-03	484	9.98E-03	Down	2.58	4509	2.62	0.20
rs4661607	55092	TMEM51	1	15283164	2.40E-03	485	1.25E-08	Down	5.69	1075	2.62	0.79
rs7119498	272	AMPD3	11	10419684	2.41E-03	486	3.82E-03	Up	2.89	3904	2.62	0.24
rs1943132	1630	DCC	18	48720895	2.42E-03	487	1.74E-01	Up	1.36	7949	2.62	0.08
rs3828417	60678	EEFSEC	3	129610952	2.42E-03	488	2.78E-01	Up	1.08	9021	2.62	0.06
rs2301680	799	CALCR	7	92760950	2.42E-03	489	8.63E-02	Up	1.72	6743	2.62	0.11
rs2268878	23428	SLC7A8	14	22689742	2.43E-03	490	7.57E-01	Down	0.31	12419	2.62	0.01
rs11047508	6660	SOX5	12	24616919	2.44E-03	491	7.82E-04	Down	3.36	3127	2.61	0.31
rs10903009	1186	CLCN7	16	1436006	2.44E-03	492	5.51E-03	Up	2.78	4130	2.61	0.23
rs7632977	4197	MDS1	3	170815535	2.44E-03	493	2.01E-01	Down	1.28	8261	2.61	0.07
rs2637978	6457	SH3GL3	15	81997618	2.45E-03	494	6.14E-01	Down	0.50	11534	2.61	0.02
rs2559619	57551	TAOK1	17	24900687	2.46E-03	495	6.45E-01	Up	0.46	11747	2.61	0.02
rs3782077	833	CARS	11	3024263	2.46E-03	496	6.85E-01	Up	0.41	11974	2.61	0.02
rs7191886	7874	USP7	16	8976412	2.47E-03	497	1.04E-01	Up	1.62	7064	2.61	0.10
rs2792818	210	ALAD	9	113238653	2.51E-03	498	1.66E-09	Down	6.03	927	2.60	0.88
rs2765843	10529	NEBL	10	21107689	2.51E-03	499	9.34E-06	Down	4.43	1932	2.60	0.50
rs2792818	257169	C9orf43	9	113238653	2.51E-03	500	4.05E-01	Up	0.83	10019	2.60	0.04
rs4565970	160335	TMTC2	12	81693585	2.51E-03	501	8.60E-08	Up	5.36	1278	2.60	0.71
rs11265165	2205	FCER1A	1	156096819	2.51E-03	502	7.61E-01	Up	0.30	12447	2.60	0.01
rs9343330	4646	MYO6	6	76692463	2.52E-03	503	-1.41E-58	Up	17.76	2	2.60	#NUM!
rs9343330	3617	IMPG1	6	76692463	2.52E-03	504	3.36E-01	Up	0.96	9492	2.60	0.05
rs8075102	55666	NPLOC4	17	77169413	2.52E-03	505	2.11E-01	Down	1.25	8368	2.60	0.07
rs2391671	9586	CREB5	7	28325617	2.53E-03	506	5.50E-01	Up	0.60	11080	2.60	0.03
rs7769144	23595	ORC3L	6	88381989	2.53E-03	507	2.16E-01	Down	1.24	8424	2.60	0.07
rs10842794	3709	ITPR2	12	26847584	2.55E-03	508	2.15E-15	Up	7.95	367	2.59	1.47
rs3744350	57125	PLXDC1	17	34515405	2.55E-03	509	1.13E-02	Up	2.53	4598	2.59	0.19
rs763545	7756	ZNF207	17	27649844	2.55E-03	510	3.46E-06	Up	4.64	1761	2.59	0.55
rs10832908	6289	SAA2	11	18216278	2.56E-03	511	4.62E-01	Up	0.74	10438	2.59	0.03
rs10832908	6291	SAA4	11	18216278	2.56E-03	512	6.13E-01	Down	0.51	11524	2.59	0.02
rs861079	4753	NELL2	2	206414627	2.57E-03	513	1.06E-08	Down	5.72	1063	2.59	0.80
rs861079	8828	NRP2	2	206414627	2.57E-03	514	1.88E-03	Down	3.11	3503	2.59	0.27
rs1390358	81539	SLC38A1	8	18297035	2.58E-03	515	4.65E-12	Down	6.92	622	2.59	1.13
rs1390358	10	NAT2	8	18297035	2.58E-03	516	4.29E-01	Down	0.79	10182	2.59	0.04
rs9327886	83594	NUDT12	5	102931646	2.58E-03	517	3.51E-01	Up	0.93	9594	2.59	0.05
rs7661752	2895	GRID2	4	94445336	2.58E-03	518	2.95E-02	Down	2.18	5448	2.59	0.15
rs10513494	6747	SSR3	3	157773965	2.58E-03	519	8.32E-07	Up	4.93	1552	2.59	0.61
rs3789199	10723	SLC12A7	5	1120535	2.59E-03	520	1.94E-07	Up	5.21	1368	2.59	0.67
rs4625350	7042	TGFB2	1	214931706	2.60E-03	521	1.01E-16	Down	8.30	320	2.58	1.60
rs4984834	64718	UNKL	16	1416466	2.61E-03	522	1.04E-02	Up	2.56	4542	2.58	0.20
rs2835983	3763	KCNJ6	21	38110476	2.61E-03	523	3.89E-01	Up	0.86	9906	2.58	0.04
rs684903	57713	SFMBT2	10	7438806	2.61E-03	524	1.41E-02	Down	2.46	4758	2.58	0.19
rs8102476	10653	SPINT2	19	43427453	2.63E-03	525	1.36E-05	Up	4.35	2020	2.58	0.49
rs8102476	94274	PPP1R14A	19	43427453	2.63E-03	526	1.02E-03	Down	3.29	3227	2.58	0.30
rs4804202	163049	ZNF791	19	12579619	2.64E-03	527	7.36E-02	Down	1.79	6494	2.58	0.11
rs4804202	57474	ZNF490	19	12579619	2.64E-03	528	2.05E-01	Up	1.27	8305	2.58	0.07
rs11990277	640	BLK	8	11409494	2.65E-03	529	1.92E-02	Down	2.34	5032	2.58	0.17
rs17864668	54576	UGT1A8	2	234300422	2.66E-03	530	5.91E-01	Up	0.54	11376	2.58	0.02
rs1424842	9369	NRXN3	14	79157408	2.66E-03	531	1.18E-01	Down	1.56	7268	2.58	0.09
rs1565585	51768	TM7SF3	12	27037709	2.67E-03	532	9.58E-07	Down	4.90	1570	2.57	0.60
rs11673172	9422	ZNF264	19	62430106	2.69E-03	533	4.87E-01	Up	0.69	10632	2.57	0.03
rs11673172	6795	AURKC	19	62430106	2.69E-03	534	7.03E-01	Down	0.38	12091	2.57	0.02
rs7102329	117194	MRGPRX2	11	19036160	2.70E-03	535	6.56E-02	Up	1.84	6353	2.57	0.12
rs6791650	91653	BOC	3	114427665	2.70E-03	536	2.20E-01	Down	1.23	8451	2.57	0.07
rs7411507	22854	NTNG1	1	107578020	2.70E-03	537	5.03E-01	Up	0.67	10737	2.57	0.03
rs3772069	5937	RBMS1	2	160957269	2.71E-03	538	1.57E-19	Down	9.04	215	2.57	1.88
rs686912	79784	MYH14	19	55448112	2.72E-03	539	5.44E-01	Down	0.61	11042	2.57	0.03
rs686912	57644	MYH7B	19	55448112	2.72E-03	540	9.67E-01	Down	0.04	13678	2.57	0.00
rs6133002	5786	PTPRA	20	2939560	2.72E-03	541	3.25E-01	Down	0.98	9405	2.57	0.05
rs2737683	2494	NR5A2	1	196824105	2.75E-03	542	1.28E-02	Up	2.49	4675	2.56	0.19
rs17432497	23301	EHBP1	2	62938988	2.76E-03	543	7.25E-01	Up	0.35	12229	2.56	0.01
rs4794737	124540	MSI2	17	52855906	2.77E-03	544	4.70E-01	Down	0.72	10498	2.56	0.03
rs8080537	54828	BCAS3	17	56806918	2.77E-03	545	3.16E-01	Down	1.00	9330	2.56	0.05
rs447735	8558	CDK10	16	88261850	2.77E-03	546	3.49E-03	Up	2.92	3858	2.56	0.25
rs3802980	10346	TRIM22	11	5662887	2.78E-03	547	2.55E-10	Down	6.32	800	2.56	0.96
rs3802980	85363	TRIM5	11	5662887	2.78E-03	548	5.74E-01	Up	0.56	11271	2.56	0.02
rs16949434	84465	MEGF11	15	64171408	2.79E-03	549	9.38E-01	Down	0.08	13476	2.56	0.00
rs2595199	5581	PRKCE	2	46099111	2.80E-03	550	1.10E-05	Down	4.40	1968	2.55	0.50
rs5955861	30011	SH3KBP1		19663648	2.80E-03	551	9.24E-01	Up	0.10	13394	2.55	0.00
rs10491724	7464	CORO2A	9	98007406	2.81E-03	552	1.06E-05	Up	4.40	1961	2.55	0.50
rs1400745	11177	BAZ1A	14	34425051	2.81E-03	553	3.83E-10	Up	6.25	833	2.55	0.94
rs10916852	57576	KIF17	1	20797999	2.82E-03	554	7.67E-01	Down	0.30	12473	2.55	0.01
rs1042704	122704	MRPL52	14	22382434	2.82E-03	555	6.15E-02	Up	1.87	6266	2.55	0.12
rs1042704	4323	MMP14	14	22382434	2.82E-03	556	1.25E-01	Up	1.53	7377	2.55	0.09
rs9489457	387119	C6orf204	6	119026978	2.83E-03	557	5.60E-02	Down	1.91	6136	2.55	0.13
rs10815092	10171	RCL1	9	4826899	2.83E-03	558	1.35E-14	Up	7.71	416	2.55	1.39
rs7445770	1010	CDH12	5	22509105	2.84E-03	559	3.01E-05	Up	4.17	2171	2.55	0.45
rs4822654	84700	MYO18B	22	24508405	2.84E-03	560	8.17E-02	Up	1.74	6664	2.55	0.11
rs6787312	23132	RAD54L2	3	51625550	2.85E-03	561	1.98E-01	Down	1.29	8242	2.55	0.07
rs2908277	58498	MYL7	7	43956673	2.86E-03	562	9.30E-02	Down	1.68	6886	2.54	0.10

rs2908277	2645	GCK	7	43956673	2.86E-03	563	4.03E-01	Down	0.84	10007	2.54	0.04
rs2908277	5871	MAP4K2	7	43956673	2.86E-03	564	9.84E-01	Up	0.02	13804	2.54	0.00
rs17056364	831	CAST	3	56020188	2.87E-03	565	7.30E-11	Down	6.51	737	2.54	1.01
rs17056364	10849	CD3EAP	3	56020188	2.87E-03	566	3.77E-03	Up	2.90	3898	2.54	0.24
rs5909374	4810	NHS		17330039	2.87E-03	567	3.27E-02	Down	2.14	5550	2.54	0.15
rs17056364	26059	ERC2	3	56020188	2.87E-03	568	4.04E-01	Down	0.83	10016	2.54	0.04
rs717344	9717	SEC14L5	16	4979249	2.89E-03	569	1.90E-04	Up	3.73	2609	2.54	0.37
rs895521	4862	NPAS2	2	101010613	2.89E-03	570	5.73E-01	Down	0.56	11263	2.54	0.02
rs16842755	2047	EPHB1	3	136383081	2.89E-03	571	3.92E-07	Down	5.07	1454	2.54	0.64
rs945032	624	BDKRB2	14	95740414	2.91E-03	572	1.25E-04	Down	3.84	2502	2.54	0.39
rs633473	81544	GDPD5	11	74930524	2.92E-03	573	2.90E-02	Down	2.18	5424	2.54	0.15
rs1533178	79698	ZMAT4	8	40858406	2.93E-03	574	3.31E-01	Up	0.97	9450	2.53	0.05
rs4436141	11178	LZTS1	8	20172358	2.94E-03	575	9.17E-01	Up	0.10	13357	2.53	0.00
rs2977983	10499	NCOA2	8	71233342	2.97E-03	576	2.69E-01	Up	1.10	8947	2.53	0.06
rs11853680	6638	SNRPN	15	22695455	2.99E-03	577	5.97E-01	Down	0.53	11413	2.52	0.02
rs8037864	6263	RYR3	15	31801713	3.02E-03	578	7.36E-01	Up	0.34	12291	2.52	0.01
rs11200147	11101	ATE1	10	123532603	3.02E-03	579	6.77E-02	Down	1.83	6389	2.52	0.12
rs472650	50937	CDON	11	125430655	3.02E-03	580	5.72E-02	Up	1.90	6166	2.52	0.12
rs6988087	8428	STK24	8	99799827	3.03E-03	581	6.29E-14	Down	7.50	457	2.52	1.32
rs6988087	6788	STK3	8	99799827	3.03E-03	582	6.90E-01	Up	0.40	12011	2.52	0.02
rs4900141	79890	RIN3	14	92221973	3.04E-03	583	9.51E-02	Down	1.67	6909	2.52	0.10
rs12820881	51535	PPHLN1	12	41013245	3.04E-03	584	8.44E-03	Up	2.63	4379	2.52	0.21
rs4809745	3745	KCNB1	20	47521278	3.04E-03	585	3.99E-02	Down	2.05	5747	2.52	0.14
rs12820881	85437	ZCRB1	12	41013245	3.04E-03	586	3.09E-01	Down	1.02	9257	2.52	0.05
rs2287348	27248	C2orf30	2	53951464	3.05E-03	587	4.63E-01	Down	0.73	10449	2.52	0.03
rs10200225	55289	ACOXL	2	111494795	3.06E-03	588	6.76E-01	Up	0.42	11926	2.51	0.02
rs6888245	23092	ARHGAP26	5	142519152	3.06E-03	589	4.09E-05	Up	4.10	2233	2.51	0.44
rs4245977	729	C6	5	41231457	3.07E-03	590	3.95E-01	Down	0.85	9942	2.51	0.04
rs4245977	5688	PSMA7	5	41231457	3.07E-03	591	4.35E-01	Up	0.78	10236	2.51	0.04
rs681570	84623	KIRREL3	11	126002739	3.07E-03	592	5.99E-02	Down	1.88	6234	2.51	0.12
rs749915	6133	RPL9	4	39297434	3.09E-03	593	4.19E-01	Up	0.81	10115	2.51	0.04
rs749915	11019	LIAS	4	39297434	3.09E-03	594	9.82E-01	Down	0.02	13788	2.51	0.00
rs1401790	4745	NELL1	11	20694175	3.09E-03	595	3.68E-02	Down	2.09	5674	2.51	0.14
rs6664207	57568	SIPA1L2	1	228882274	3.10E-03	596	2.92E-01	Up	1.05	9126	2.51	0.05
rs4931181	5307	PITX1	12	29440625	3.10E-03	597	8.30E-09	Down	5.76	1041	2.51	0.81
rs4931181	51290	ERGIC2	12	29440625	3.10E-03	598	5.18E-03	Up	2.80	4089	2.51	0.23
rs4931181	1401	CRP	12	29440625	3.10E-03	599	6.81E-01	Down	0.41	11953	2.51	0.02
rs11944152	132864	CPEB2	4	14726995	3.11E-03	600	1.33E-02	Down	2.47	4712	2.51	0.19
rs9283862	64094	SMOC2	6	168840770	3.11E-03	601	1.70E-13	Up	7.37	486	2.51	1.28
rs6739975	57217	TTC7A	2	47153991	3.11E-03	602	2.45E-02	Down	2.25	5269	2.51	0.16
rs3830041	4855	NOTCH4	6	32299317	3.13E-03	603	5.62E-01	Up	0.58	11175	2.50	0.03
rs1127231	9518	GDF15	16	67912464	3.13E-03	604	3.37E-12	Up	6.96	610	2.50	1.15
rs1127231	51388	NIP7	16	67912464	3.13E-03	605	1.45E-03	Up	3.18	3363	2.50	0.28
rs1127231	64146	PDF	16	67912464	3.13E-03	606	1.58E-01	Up	1.41	7787	2.50	0.08
rs1127231	84342	COG8	16	67912464	3.13E-03	607	2.26E-01	Down	1.21	8516	2.50	0.06
rs1127231	27183	VPS4A	16	67912464	3.13E-03	608	6.29E-01	Up	0.48	11642	2.50	0.02
rs241966	84766	EFCAB4B	12	3710226	3.14E-03	609	1.47E-03	Down	3.18	3374	2.50	0.28
rs4288259	11281	POU6F2	7	39120359	3.14E-03	610	9.41E-01	Up	0.07	13502	2.50	0.00
rs12527640	5071	PARK2	6	161929443	3.14E-03	611	3.73E-01	Down	0.89	9786	2.50	0.04
rs528320	221044	C10orf49	10	13326337	3.15E-03	612	7.49E-01	Down	0.32	12367	2.50	0.01
rs12971499	3643	INSR	19	7165282	3.18E-03	613	3.12E-02	Down	2.15	5501	2.50	0.15
rs1234887	64072	CDH23	10	73107026	3.18E-03	614	8.11E-02	Down	1.74	6651	2.50	0.11
rs4395543	10396	ATP8A1	4	42393362	3.19E-03	615	9.13E-30	Up	11.30	68	2.50	2.90
rs948640	5268	SERPINB5	18	59286862	3.20E-03	616	7.13E-08	Down	5.39	1258	2.50	0.71
rs9640758	93986	FOXP2	7	113919766	3.20E-03	617	7.07E-01	Up	0.38	12110	2.49	0.02
rs960965	57528	KCTD16	5	143683870	3.20E-03	618	9.92E-01	Down	0.01	13859	2.49	0.00
rs2771994	158067	C9orf98	9	132754455	3.21E-03	619	7.04E-01	Up	0.38	12094	2.49	0.02
rs10801604	23418	CRB1	1	194074382	3.21E-03	620	9.72E-01	Up	0.04	13710	2.49	0.00
rs4252170	5340	PLG	6	161132817	3.21E-03	621	5.57E-06	Down	4.54	1836	2.49	0.53
rs9474720	90523	C6orf142	6	54029086	3.21E-03	622	6.81E-02	Up	1.82	6398	2.49	0.12
rs1538677	219793	C10orf27	10	72213585	3.23E-03	623	6.22E-02	Up	1.86	6284	2.49	0.12
rs4949653	26289	AK5	1	77534540	3.24E-03	624	4.69E-01	Up	0.72	10491	2.49	0.03
rs2839439	49854	ZNF295	21	42312631	3.24E-03	625	7.31E-02	Down	1.79	6488	2.49	0.11
rs5753669	9814	SFI1	22	30230373	3.25E-03	626	9.75E-01	Down	0.03	13731	2.49	0.00
rs2511989	710	SERPING1	11	57134901	3.25E-03	627	6.36E-06	Down	4.51	1858	2.49	0.52
rs6125084	55959	SULF2	20	45777680	3.25E-03	628	4.29E-01	Down	0.79	10184	2.49	0.04
rs6540512	8444	DYRK3	1	203202132	3.26E-03	629	1.19E-03	Down	3.24	3288	2.49	0.29
rs3817512	10551	AGR2	7	16612340	3.26E-03	630	4.12E-09	Up	5.88	995	2.49	0.84
rs11610353	23012	STK38L	12	27282464	3.28E-03	631	8.10E-10	Down	6.14	879	2.48	0.91
rs1020997	152330	CNTN4	3	2628577	3.28E-03	632	3.30E-01	Down	0.97	9448	2.48	0.05
rs2592960	10369	CACNG2	22	35420601	3.28E-03	633	6.56E-01	Up	0.45	11810	2.48	0.02
rs9976971	3460	IFNGR2	21	33689967	3.28E-03	634	7.51E-11	Up	6.51	739	2.48	1.01
rs7639767	5806	PTX3	3	158618297	3.28E-03	635	1.66E-01	Down	1.39	7874	2.48	0.08
rs17114771	2890	GRIA1	5	152896155	3.28E-03	636	4.43E-01	Down	0.77	10293	2.48	0.04
rs8567	7165	TPD52L2	20	61992759	3.29E-03	637	2.97E-03	Down	2.97	3772	2.48	0.25
rs8567	80331	DNAJC5	20	61992759	3.29E-03	638	9.71E-01	Up	0.04	13707	2.48	0.00
rs6929137	80129	C6orf97	6	152028791	3.29E-03	639	4.74E-01	Up	0.72	10527	2.48	0.03
rs3213287	7515	XRCC1	19	48763248	3.30E-03	640	9.82E-02	Down	1.65	6968	2.48	0.10
rs10949714	5799	PTPRN2	7	157609910	3.31E-03	641	5.72E-17	Up	8.37	313	2.48	1.62
rs796271	1418	CRYGA	2	208846738	3.32E-03	642	8.95E-02	Down	1.70	6795	2.48	0.10
rs7860650	5125	PCSK5	9	75951243	3.32E-03	643	4.45E-01	Down	0.76	10306	2.48	0.04

rs796271	1419	CRYGB	2	208846738	3.32E-03	644	7.51E-01	Down	0.32	12381	2.48	0.01
rs1766923	138639	PTPDC1	9	93882452	3.32E-03	645	7.67E-02	Up	1.77	6568	2.48	0.11
rs253427	6671	SP4	7	21224183	3.32E-03	646	1.76E-01	Up	1.35	7975	2.48	0.08
rs4144761	286183	NKAIN3	8	63508974	3.32E-03	647	5.11E-01	Up	0.66	10786	2.48	0.03
rs1751848	6452	SH3BP2	4	2841681	3.32E-03	648	5.14E-01	Down	0.65	10806	2.48	0.03
rs7595126	90134	KCNH7	2	163118475	3.33E-03	649	8.48E-01	Down	0.19	12945	2.48	0.01
rs6707186	55677	IWS1	2	128019594	3.33E-03	650	1.84E-03	Down	3.11	3489	2.48	0.27
rs4786772	9894	TELO2	16	1472838	3.35E-03	651	8.15E-04	Up	3.35	3142	2.47	0.31
rs11894280	64284	RAB17	2	238291759	3.36E-03	652	3.63E-17	Up	8.42	301	2.47	1.64
rs725779	196513	DCP1B	12	1999243	3.36E-03	653	1.72E-01	Up	1.37	7938	2.47	0.08
rs10845715	2012	EMP1	12	13252583	3.36E-03	654	3.68E-02	Up	2.09	5672	2.47	0.14
rs12014699	9185	REPS2		16773623	3.37E-03	655	3.36E-25	Up	10.39	110	2.47	2.45
rs7576924	114800	CCDC85A	2	56413472	3.38E-03	656	4.27E-03	Down	2.86	3976	2.47	0.24
rs7575508	57520	HECW2	2	196935802	3.40E-03	657	1.32E-01	Up	1.51	7462	2.47	0.09
rs12662355	1769	DNAH8	6	39010448	3.40E-03	658	6.88E-01	Down	0.40	11997	2.47	0.02
rs17061202	26002	MOXD1	6	132725455	3.40E-03	659	9.03E-01	Down	0.12	13264	2.47	0.00
rs235246	55187	VPS13D	1	12250850	3.41E-03	660	2.00E-05	Down	4.26	2087	2.47	0.47
rs12679771	291	SLC25A4	8	140971097	3.41E-03	661	4.53E-10	Down	6.23	846	2.47	0.93
rs12679771	921	CD5	8	140971097	3.41E-03	662	8.88E-02	Up	1.70	6781	2.47	0.11
rs12679771	9173	IL1RL1	8	140971097	3.41E-03	663	1.96E-01	Down	1.29	8216	2.47	0.07
rs13308578	9863	MAGI2	7	78053210	3.41E-03	664	3.18E-12	Down	6.97	609	2.47	1.15
rs13308578	23204	ARL6IP1	7	78053210	3.41E-03	665	7.33E-10	Up	6.15	875	2.47	0.91
rs13308578	153090	DAB2IP	7	78053210	3.41E-03	666	7.96E-07	Down	4.94	1544	2.47	0.61
rs13308578	10015	PDCD6IP	7	78053210	3.41E-03	667	2.58E-01	Down	1.13	8842	2.47	0.06
rs17545038	7253	TSHR	14	80527325	3.41E-03	668	5.19E-01	Down	0.64	10852	2.47	0.03
rs293796	4968	OGG1	3	9784082	3.41E-03	669	9.06E-02	Down	1.69	6839	2.47	0.10
rs293796	8536	CAMK1	3	9784082	3.41E-03	670	4.08E-01	Up	0.83	10037	2.47	0.04
rs16990499	4983	OPHN1		67069088	3.41E-03	671	5.91E-01	Down	0.54	11379	2.47	0.02
rs7623610	4286	MITF	3	70087971	3.42E-03	672	4.63E-03	Down	2.83	4014	2.47	0.23
rs11755449	1915	EEF1A1	6	74283074	3.42E-03	673	1.81E-02	Up	2.36	4989	2.47	0.17
rs826385	7068	THRB	3	24162088	3.42E-03	674	1.41E-09	Up	6.05	918	2.47	0.89
rs884851	3785	KCNQ2	20	61569740	3.42E-03	675	5.58E-01	Up	0.59	11149	2.47	0.03
rs10490775	5793	PTPRG	3	62011764	3.42E-03	676	3.05E-02	Up	2.16	5480	2.47	0.15
rs1361265	3195	TLX1	10	102864658	3.42E-03	677	4.73E-01	Down	0.72	10518	2.47	0.03
rs2189972	27445	PCLO	7	82169314	3.42E-03	678	4.10E-01	Up	0.82	10058	2.47	0.04
rs1264701	11074	TRIM31	6	30174337	3.43E-03	679	2.48E-02	Up	2.24	5279	2.46	0.16
rs4140836	6549	SLC9A2	2	102727029	3.44E-03	680	7.31E-01	Up	0.34	12258	2.46	0.01
rs249869	84516	DCTN5	16	23574058	3.44E-03	681	5.53E-03	Up	2.77	4134	2.46	0.23
rs249869	79728	PALB2	16	23574058	3.44E-03	682	6.69E-03	Up	2.71	4229	2.46	0.22
rs10865143	6432	SFRS7	2	38903607	3.45E-03	683	5.03E-10	Up	6.22	857	2.46	0.93
rs10865143	79833	GEMIN6	2	38903607	3.45E-03	684	6.81E-02	Up	1.82	6396	2.46	0.12
rs341497	81624	DIAPH3	13	59327002	3.46E-03	685	1.90E-02	Up	2.35	5021	2.46	0.17
rs11080335	162394	SLFN5	17	30623671	3.46E-03	686	1.33E-05	Down	4.35	2017	2.46	0.49
rs10137185	2100	ESR2	14	63845529	3.47E-03	687	1.26E-01	Up	1.53	7381	2.46	0.09
rs9953923	115701	ALPK2	18	54311662	3.47E-03	688	4.08E-01	Down	0.83	10036	2.46	0.04
rs16980628	11283	CYP4F8	19	15587345	3.48E-03	689	4.38E-01	Up	0.78	10256	2.46	0.04
rs6414283	55254	TMEM39A	3	120678603	3.51E-03	690	5.60E-08	Up	5.43	1235	2.46	0.73
rs6414283	56983	KTELC1	3	120678603	3.51E-03	691	9.40E-04	Up	3.31	3191	2.46	0.30
rs8038669	6017	RLBP1	15	87568029	3.52E-03	692	5.79E-01	Up	0.55	11305	2.45	0.02
rs7332098	171425	CLYBL	13	99261899	3.53E-03	693	3.67E-01	Up	0.90	9733	2.45	0.04
rs2075377	8496	PPFIBP1	12	27678902	3.53E-03	694	1.00E-03	Down	3.29	3222	2.45	0.30
rs7634185	29970	SCHIP1	3	160667271	3.55E-03	695	7.72E-16	Down	8.06	356	2.45	1.51
rs2271567	29088	MRPL15	8	55211808	3.55E-03	696	3.32E-06	Up	4.65	1757	2.45	0.55
rs6086491	23236	PLCB1	20	8473777	3.56E-03	697	2.93E-03	Down	2.98	3765	2.45	0.25
rs854889	347902	AMIGO2	12	45757304	3.57E-03	698	2.03E-07	Down	5.20	1372	2.45	0.67
rs7037043	6096	RORB	9	74390369	3.57E-03	699	2.10E-01	Up	1.25	8355	2.45	0.07
rs1300836	10004	NAALADL1	11	64588605	3.58E-03	700	2.23E-01	Down	1.22	8484	2.45	0.07
rs1300836	7542	ZFPL1	11	64588605	3.58E-03	701	9.14E-01	Up	0.11	13343	2.45	0.00
rs10852054	11214	AKAP13	15	84068301	3.60E-03	702	1.60E-02	Down	2.41	4885	2.44	0.18
rs7812348	79815	NPAL2	8	99384758	3.60E-03	703	3.91E-04	Down	3.55	2873	2.44	0.34
rs11582097	25909	AHCTF1	1	243361072	3.60E-03	704	2.30E-02	Up	2.27	5200	2.44	0.16
rs10505346	4982	TNFRSF11B	8	120033024	3.61E-03	705	5.17E-01	Down	0.65	10832	2.44	0.03
rs6132261	51126	NAT5	20	19962955	3.61E-03	706	1.12E-05	Up	4.39	1971	2.44	0.50
rs6132261	80218	NAT13	20	19962955	3.61E-03	707	1.29E-04	Up	3.83	2512	2.44	0.39
rs6132261	26074	C20orf26	20	19962955	3.61E-03	708	5.48E-02	Down	1.92	6109	2.44	0.13
rs6132261	51340	CRNKL1	20	19962955	3.61E-03	709	1.91E-01	Up	1.31	8165	2.44	0.07
rs10175486	57539	WDR35	2	20030689	3.63E-03	710	4.45E-02	Up	2.01	5867	2.44	0.14
rs1557203	80307	FER1L4	20	33627877	3.63E-03	711	6.67E-01	Down	0.43	11865	2.44	0.02
rs2332284	151636	DTX3L	3	123766502	3.65E-03	712	1.02E-10	Up	6.46	757	2.44	1.00
rs2332284	83666	PARP9	3	123766502	3.65E-03	713	9.69E-11	Up	6.45	759	2.44	1.00
rs1078305	2934	GSN	9	121130954	3.66E-03	714	1.69E-18	Down	8.78	250	2.44	1.78
rs852787	1600	DAB1	1	58014123	3.66E-03	715	5.21E-03	Down	2.79	4096	2.44	0.23
rs11625706	5427	POLE2	14	49214807	3.68E-03	716	1.53E-03	Up	3.17	3392	2.43	0.28
rs11625706	122773	KLHDC1	14	49214807	3.68E-03	717	2.62E-01	Down	1.12	8875	2.43	0.06
rs737887	113791	PIK3IP1	22	30021169	3.69E-03	718	6.17E-02	Down	1.87	6274	2.43	0.12
rs7957299	160760	PPTC7	12	109465031	3.69E-03	719	3.81E-02	Down	2.07	5707	2.43	0.14
rs2600208	9922	IQSEC1	3	12945494	3.70E-03	720	6.85E-01	Up	0.41	11980	2.43	0.02
rs10095169	7013	TERF1	8	74092275	3.72E-03	721	2.62E-04	Down	3.65	2730	2.43	0.36
rs17683985	23335	WDR7	18	52821051	3.73E-03	722	3.73E-10	Down	6.26	823	2.43	0.94
rs17007017	27309	ZNF330	4	142500334	3.74E-03	723	8.08E-03	Down	2.65	4349	2.43	0.21
rs1322984	1761	DMRT1	9	906220	3.77E-03	724	4.59E-01	Down	0.74	10418	2.42	0.03

rs5758756	94009	SERHL	22	41209495	3.78E-03	725	2.62E-01	Up	1.12	8877	2.42	0.06
rs4347296	5791	PTPRE	10	129648261	3.78E-03	726	3.89E-01	Down	0.86	9909	2.42	0.04
rs7630867	27255	CNTN6	3	1423641	3.78E-03	727	6.03E-01	Down	0.52	11454	2.42	0.02
rs16998892	5126	PCSK2	20	17200930	3.79E-03	728	9.83E-01	Down	0.02	13797	2.42	0.00
rs7183546	2595	GANC	15	40425795	3.79E-03	729	4.56E-01	Down	0.75	10388	2.42	0.03
rs1920230	64770	CCDC14	3	125124563	3.80E-03	730	8.20E-05	Up	3.94	2400	2.42	0.41
rs208739	23328	SASH1	6	148916667	3.82E-03	731	1.14E-07	Up	5.30	1309	2.42	0.69
rs17104710	84871	AGBL4	1	48891518	3.82E-03	732	3.28E-01	Down	0.98	9422	2.42	0.05
rs505802	116085	SLC22A12	11	64113648	3.84E-03	733	8.42E-01	Down	0.20	12910	2.42	0.01
rs2845712	4477	MSMB	11	117279501	3.84E-03	734	4.72E-04	Down	3.50	2942	2.42	0.33
rs2845712	4485	MST1	11	117279501	3.84E-03	735	7.48E-04	Down	3.37	3109	2.42	0.31
rs2845712	89782	LMLN	11	117279501	3.84E-03	736	2.53E-03	Up	3.02	3695	2.42	0.26
rs1472772	132204	SYNPR	3	63554309	3.86E-03	737	6.80E-01	Down	0.41	11948	2.41	0.02
rs3825305	57460	PPM1H	12	61324965	3.87E-03	738	3.50E-10	Up	6.28	817	2.41	0.95
rs12601925	342527	SMTNL2	17	4449944	3.87E-03	739	8.28E-01	Up	0.22	12841	2.41	0.01
rs209729	9923	ZBTB40	1	22580983	3.87E-03	740	3.78E-02	Up	2.08	5702	2.41	0.14
rs1827770	23284	LPHN3	4	61932878	3.88E-03	741	1.46E-03	Down	3.18	3369	2.41	0.28
rs6953356	84626	KRBA1	7	148801894	3.88E-03	742	3.67E-03	Down	2.91	3884	2.41	0.24
rs4270569	166614	DCLK2	4	151353073	3.89E-03	743	2.20E-01	Up	1.23	8461	2.41	0.07
rs12623642	92749	C2orf39	2	26588046	3.89E-03	744	1.51E-01	Down	1.43	7697	2.41	0.08
rs12623642	9381	OTOF	2	26588046	3.89E-03	745	1.69E-01	Down	1.38	7905	2.41	0.08
rs917066	22990	PCNX	14	70466936	3.90E-03	746	3.62E-01	Up	0.91	9690	2.41	0.04
rs17003846	59353	TMEM35		100142011	3.90E-03	747	4.66E-04	Down	3.50	2934	2.41	0.33
rs16875887	57152	SLURP1	5	78108781	3.91E-03	748	8.26E-02	Down	1.74	6677	2.41	0.11
rs16875887	411	ARSB	5	78108781	3.91E-03	749	9.07E-02	Down	1.69	6840	2.41	0.10
rs11068103	84915	C12orf34	12	108649273	3.91E-03	750	5.43E-03	Up	2.78	4118	2.41	0.23
rs12369411	4815	NINJ2	12	577921	3.91E-03	751	1.72E-02	Up	2.38	4950	2.41	0.18
rs2499833	25903	OLFML2B	1	158703322	3.92E-03	752	1.49E-01	Up	1.44	7668	2.41	0.08
rs2946340	31	ACACA	17	32823829	3.92E-03	753	1.48E-26	Up	10.64	91	2.41	2.58
rs9490191	221322	C6orf170	6	121713643	3.92E-03	754	1.23E-03	Up	3.23	3302	2.41	0.29
rs2946340	6871	TADA2L	17	32823829	3.92E-03	755	1.38E-01	Up	1.48	7545	2.41	0.09
rs2946340	284099	C17orf78	17	32823829	3.92E-03	756	5.59E-01	Down	0.58	11156	2.41	0.03
rs3734899	79981	FRMD1	6	168276522	3.93E-03	757	3.17E-01	Down	1.00	9340	2.41	0.05
rs6739399	129684	CNTNAP5	2	125132837	3.93E-03	758	3.12E-01	Up	1.01	9289	2.41	0.05
rs909486	1439	CSF2RB	22	35648488	3.93E-03	759	7.09E-01	Up	0.37	12121	2.41	0.01
rs2268509	131096	KCNH8	12	95102248	3.94E-03	760	1.82E-03	Up	3.12	3483	2.41	0.27
rs2268509	2004	ELK3	12	95102248	3.94E-03	761	9.50E-01	Up	0.06	13549	2.41	0.00
rs16919663	636	BICD1	12	32345199	3.94E-03	762	7.86E-33	Up	11.93	51	2.40	3.21
rs643423	79671	NLRX1	11	118548866	3.96E-03	763	9.01E-02	Down	1.69	6818	2.40	0.10
rs6918742	988	CDC5L	6	44449958	3.96E-03	764	1.59E-02	Up	2.41	4879	2.40	0.18
rs6918742	221409	SPATS1	6	44449958	3.96E-03	765	9.82E-02	Up	1.65	6967	2.40	0.10
rs351982	5725	PTBP1	19	755013	3.96E-03	766	-4.03E-15	Up	7.41	480	2.40	#NUM!
rs13213933	5796	PTPRK	6	128590678	3.96E-03	767	3.89E-01	Down	0.86	9907	2.40	0.04
rs714026	23551	RASD2	22	34251961	3.97E-03	768	2.46E-03	Down	3.03	3675	2.40	0.26
rs7131394	4978	OPCML	11	132127966	3.98E-03	769	2.68E-02	Down	2.21	5352	2.40	0.16
rs17842067	64359	NXN	17	793231	3.99E-03	770	9.54E-02	Down	1.67	6914	2.40	0.10
rs17794518	81832	NETO1	18	68632198	3.99E-03	771	8.19E-01	Up	0.23	12781	2.40	0.01
rs942515	1620	DBC1	9	119204343	4.00E-03	772	2.24E-03	Down	3.06	3611	2.40	0.26
rs942515	57805	KIAA1967	9	119204343	4.00E-03	773	7.36E-01	Up	0.34	12292	2.40	0.01
rs289747	1071	CETP	16	55581439	4.00E-03	774	8.13E-02	Up	1.74	6655	2.40	0.11
rs2194051	285601	GPR150	5	94981358	4.00E-03	775	6.44E-01	Down	0.46	11742	2.40	0.02
rs10796021	10133	OPTN	10	13175052	4.01E-03	776	1.30E-16	Down	8.27	327	2.40	1.59
rs2027765	5031	P2RY6	11	72661678	4.01E-03	777	8.40E-01	Down	0.20	12905	2.40	0.01
rs13094089	64778	FNDC3B	3	173456303	4.01E-03	778	3.78E-03	Up	2.90	3900	2.40	0.24
rs12530902	3751	KCND2	7	119860409	4.01E-03	779	8.90E-01	Down	0.14	13189	2.40	0.01
rs10521107	55275	VPS53	17	457906	4.02E-03	780	1.56E-02	Down	2.42	4850	2.40	0.18
rs763354	79940	C6orf155	6	72172342	4.02E-03	781	4.20E-01	Up	0.81	10119	2.40	0.04
rs3791731	3030	HADHA	2	26325019	4.02E-03	782	4.12E-03	Down	2.87	3952	2.40	0.24
rs2980619	84955	NUDCD1	8	110371223	4.03E-03	783	4.65E-02	Up	1.99	5914	2.40	0.13
rs4382429	94137	RP1L1	8	10510048	4.04E-03	784	7.84E-01	Up	0.27	12571	2.39	0.01
rs10103750	3084	NRG1	8	32716540	4.05E-03	785	3.17E-05	Down	4.16	2181	2.39	0.45
rs10002842	166647	GPR125	4	22090292	4.05E-03	786	6.94E-01	Down	0.39	12026	2.39	0.02
rs16854812	26996	GPR160	3	171257085	4.06E-03	787	4.97E-26	Up	10.55	96	2.39	2.53
rs6752943	238	ALK	2	29333148	4.06E-03	788	4.88E-01	Up	0.69	10638	2.39	0.03
rs7445039	91522	COL23A1	5	177728233	4.06E-03	789	2.87E-02	Down	2.19	5415	2.39	0.15
rs12588574	4053	LTBP2	14	74075062	4.08E-03	790	4.83E-09	Down	5.85	1008	2.39	0.83
rs2004107	4071	TM4SF1	3	150584509	4.08E-03	791	1.85E-01	Up	1.33	8078	2.39	0.07
rs11250458	105	ADARB2	10	1393092	4.08E-03	792	3.64E-01	Up	0.91	9716	2.39	0.04
rs758944	7532	YWHAG	7	75597948	4.09E-03	793	2.22E-07	Up	5.18	1387	2.39	0.67
rs4889173	124359	CDYL2	16	79218899	4.09E-03	794	6.46E-01	Up	0.46	11752	2.39	0.02
rs11246311	79751	SLC25A22	11	773512	4.12E-03	795	9.34E-03	Up	2.60	4459	2.39	0.20
rs11246311	347862	PDDC1	11	773512	4.12E-03	796	2.07E-02	Up	2.31	5102	2.39	0.17
rs11246311	51286	CEND1	11	773512	4.12E-03	797	9.97E-01	Up	0.00	13889	2.39	0.00
rs10780986	5239	PGM5	9	68353301	4.13E-03	798	3.37E-05	Down	4.15	2192	2.38	0.45
rs5754083	254240	BPIL2	22	31156624	4.13E-03	799	7.53E-01	Down	0.31	12398	2.38	0.01
rs3118536	6256	RXRA	9	134534407	4.13E-03	800	1.15E-03	Down	3.25	3275	2.38	0.29
rs11873271	11031	RAB31	18	9726809	4.14E-03	801	1.60E-17	Down	8.52	287	2.38	1.68
rs541245	1500	CTNND1	11	57302524	4.14E-03	802	9.88E-01	Up	0.02	13828	2.38	0.00
rs17116178	3359	HTR3A	11	113332536	4.14E-03	803	9.99E-02	Down	1.65	6994	2.38	0.10
rs7886010	10159	ATP6AP2		40180196	4.15E-03	804	4.77E-02	Down	1.98	5939	2.38	0.13
rs2302107	1352	COX10	17	13913856	4.18E-03	805	4.74E-02	Up	1.98	5932	2.38	0.13

rs3738815	4237	MFAP2	1	17060008	4.20E-03	806	1.85E-01	Down	1.32	8088	2.38	0.07
rs6502997	239	ALOX12	17	6828482	4.20E-03	807	8.39E-01	Up	0.20	12896	2.38	0.01
rs638590	55754	TMEM30A	6	76021696	4.21E-03	808	2.41E-01	Up	1.17	8659	2.38	0.06
rs638590	1347	COX7A2	6	76021696	4.21E-03	809	2.53E-01	Up	1.14	8789	2.38	0.06
rs606590	54941	RNF125	18	27881529	4.22E-03	810	5.36E-06	Up	4.55	1830	2.37	0.53
rs1045763	10273	STUB1	16	664085	4.23E-03	811	1.53E-02	Up	2.43	4824	2.37	0.18
rs1045763	89941	RHOT2	16	664085	4.23E-03	812	1.06E-01	Down	1.62	7095	2.37	0.10
rs1045763	9028	RHBDL1	16	664085	4.23E-03	813	1.21E-01	Up	1.55	7321	2.37	0.09
rs7982132	3839	KPNA3	13	49198503	4.23E-03	814	7.55E-15	Down	7.77	400	2.37	1.41
rs10438701	54549	SDK2	17	68906768	4.24E-03	815	2.34E-01	Up	1.19	8607	2.37	0.06
rs8103597	23094	SIPA1L3	19	43244459	4.24E-03	816	5.16E-06	Up	4.56	1823	2.37	0.53
rs17743079	5500	PPP1CB	2	28913642	4.28E-03	817	5.52E-01	Up	0.59	11094	2.37	0.03
rs11741316	6507	SLC1A3	5	36636567	4.29E-03	818	4.88E-01	Down	0.69	10640	2.37	0.03
rs11653095	3682	ITGAE	17	3635881	4.31E-03	819	2.74E-03	Down	3.00	3738	2.37	0.26
rs16965644	9572	NR1D1	17	35517723	4.31E-03	820	3.34E-03	Down	2.93	3833	2.37	0.25
rs2069600	623	BDKRB1	14	95782782	4.33E-03	821	7.62E-02	Down	1.77	6555	2.36	0.11
rs2616104	219738	C10orf35	10	71053383	4.34E-03	822	2.71E-02	Down	2.21	5365	2.36	0.16
rs13120831	1182	CLCN3	4	170937328	4.34E-03	823	6.74E-05	Up	3.99	2353	2.36	0.42
rs17166188	85477	SCIN	7	12392004	4.35E-03	824	3.03E-04	Up	3.61	2779	2.36	0.35
rs10828695	57584	ARHGAP21	10	25071921	4.35E-03	825	3.06E-01	Up	1.02	9236	2.36	0.05
rs869179	249	ALPL	1	21585435	4.35E-03	826	2.31E-01	Down	1.20	8577	2.36	0.06
rs1570587	79670	ZCCHC6	9	86183076	4.37E-03	827	3.45E-21	Up	9.47	174	2.36	2.05
rs3024390	2162	F13A1	6	6194299	4.40E-03	828	5.69E-02	Down	1.90	6157	2.36	0.12
rs11242001	171019	ADAMTS19	5	128830414	4.40E-03	829	7.40E-02	Up	1.79	6506	2.36	0.11
rs430433	1826	DSCAM	21	40423027	4.41E-03	830	5.12E-01	Down	0.66	10793	2.36	0.03
rs2190453	10083	USH1C	11	17489973	4.41E-03	831	8.08E-01	Down	0.24	12713	2.36	0.01
rs711252	51232	CRIM1	2	36603602	4.41E-03	832	5.61E-07	Down	5.00	1497	2.36	0.63
rs1006938	23521	RPL13A	19	54666198	4.43E-03	833	3.80E-11	Up	6.64	689	2.35	1.04
rs1006938	55011	PIH1D1	19	54666198	4.43E-03	834	6.04E-02	Down	1.88	6244	2.35	0.12
rs1006938	2323	FLT3LG	19	54666198	4.43E-03	835	2.18E-01	Down	1.23	8436	2.35	0.07
rs1006938	126133	ALDH16A1	19	54666198	4.43E-03	836	3.03E-01	Down	1.03	9210	2.35	0.05
rs17505781	7092	TLL1	4	167325109	4.43E-03	837	5.10E-01	Up	0.66	10783	2.35	0.03
rs10516556	10427	SEC24B	4	110752750	4.45E-03	838	7.99E-01	Down	0.25	12669	2.35	0.01
rs12900552	123606	NIPA1	15	20629298	4.46E-03	839	5.86E-01	Up	0.55	11344	2.35	0.02
rs2776961	6541	SLC7A1	13	28999280	4.49E-03	840	2.88E-14	Up	7.59	440	2.35	1.35
rs398734	960	CD44	11	35110454	4.49E-03	841	1.42E-05	Down	4.34	2027	2.35	0.48
rs588677	8641	PCDHGB4	18	57364695	4.49E-03	842	1.89E-01	Down	1.31	8136	2.35	0.07
rs588677	28316	CDH20	18	57364695	4.49E-03	843	3.44E-01	Down	0.95	9553	2.35	0.05
rs1826977	2104	ESRRG	1	213171923	4.50E-03	844	1.15E-03	Up	3.25	3277	2.35	0.29
rs3809325	84650	EBPL	13	49164110	4.51E-03	845	6.42E-01	Down	0.47	11724	2.35	0.02
rs10957821	79776	ZFHX4	8	77900329	4.51E-03	846	2.59E-01	Down	1.13	8848	2.35	0.06
rs11229087	25921	ZDHHC5	11	57182275	4.53E-03	847	4.08E-01	Up	0.83	10039	2.34	0.04
rs11229087	219539	YPEL4	11	57182275	4.53E-03	848	4.11E-01	Down	0.82	10062	2.34	0.04
rs11229087	10978	CLP1	11	57182275	4.53E-03	849	8.00E-01	Down	0.25	12677	2.34	0.01
rs704329	777	CACNA1E	1	178458930	4.54E-03	850	5.41E-06	Down	4.55	1831	2.34	0.53
rs1211975	56912	C11orf60	11	64642100	4.55E-03	851	2.06E-05	Down	4.26	2094	2.34	0.47
rs1211975	7108	TM7SF2	11	64642100	4.55E-03	852	2.97E-03	Up	2.97	3774	2.34	0.25
rs1211975	740	MRPL49	11	64642100	4.55E-03	853	3.47E-02	Down	2.11	5613	2.34	0.15
rs1211975	2197	FAU	11	64642100	4.55E-03	854	7.03E-02	Up	1.81	6433	2.34	0.12
rs1211975	738	C11orf2	11	64642100	4.55E-03	855	8.38E-02	Up	1.73	6697	2.34	0.11
rs1211975	741	ZNHIT2	11	64642100	4.55E-03	856	3.68E-01	Up	0.90	9745	2.34	0.04
rs1211975	84447	SYVN1	11	64642100	4.55E-03	857	7.69E-01	Up	0.29	12483	2.34	0.01
rs6509088	57126	CD177	19	48562727	4.56E-03	858	3.94E-06	Down	4.61	1778	2.34	0.54
rs608604	9328	GTF3C5	9	132932516	4.56E-03	859	2.15E-03	Up	3.07	3578	2.34	0.27
rs11597218	85442	KNDC1	10	134926969	4.56E-03	860	1.43E-01	Down	1.47	7606	2.34	0.08
rs11597218	8433	UTF1	10	134926969	4.56E-03	861	4.35E-01	Down	0.78	10230	2.34	0.04
rs250413	23600	AMACR	5	34018325	4.57E-03	862	-2.12E-62	Up	21.87	1	2.34	#NUM!
rs17158583	10371	SEMA3A	7	83347670	4.57E-03	863	9.44E-03	Down	2.60	4467	2.34	0.20
rs265077	169693	C9orf71	9	68376087	4.59E-03	864	1.35E-01	Up	1.49	7520	2.34	0.09
rs601663	80724	ACAD10	12	110586004	4.59E-03	865	8.48E-06	Up	4.45	1918	2.34	0.51
rs601663	8315	BRAP	12	110586004	4.59E-03	866	1.82E-01	Down	1.33	8049	2.34	0.07
rs1420624	5783	PTPN13	4	88036821	4.59E-03	867	9.00E-08	Up	5.34	1286	2.34	0.70
rs7920896	10579	TACC2	10	123836480	4.61E-03	868	6.30E-02	Down	1.86	6298	2.34	0.12
rs11632524	9824	ARHGAP11A	15	30713368	4.62E-03	869	8.76E-01	Down	0.16	13094	2.34	0.01
rs3751050	57758	SCUBE2	11	9047820	4.62E-03	870	5.01E-04	Down	3.48	2963	2.34	0.33
rs2900467	55359	STYK1	12	10665965	4.63E-03	871	1.07E-01	Up	1.61	7101	2.33	0.10
rs2826655	4685	NCAM2	21	21404118	4.64E-03	872	2.78E-02	Up	2.20	5388	2.33	0.16
rs3826861	80207	OPA3	19	50748460	4.64E-03	873	4.16E-02	Up	2.04	5789	2.33	0.14
rs4684230	80852	GRIP2	3	14542670	4.64E-03	874	4.79E-01	Down	0.71	10562	2.33	0.03
rs461251	79850	FAM57A	17	565912	4.68E-03	875	3.18E-02	Up	2.15	5518	2.33	0.15
rs2306335	23431	AP4E1	15	49063420	4.68E-03	876	5.37E-01	Up	0.62	10987	2.33	0.03
rs9649969	3786	KCNQ3	8	133406845	4.68E-03	877	4.10E-02	Down	2.04	5776	2.33	0.14
rs3797207	2745	GLRX	5	95195665	4.69E-03	878	3.23E-08	Down	5.53	1174	2.33	0.75
rs11625855	64093	SMOC1	14	69474198	4.69E-03	879	4.84E-05	Down	4.06	2274	2.33	0.43
rs675388	3758	KCNJ1	11	128213219	4.69E-03	880	7.11E-01	Up	0.37	12133	2.33	0.01
rs2243639	6441	SFTPD	10	81691702	4.70E-03	881	8.87E-01	Down	0.14	13169	2.33	0.01
rs12537363	28996	HIPK2	7	138757231	4.70E-03	882	8.57E-22	Up	9.59	164	2.33	2.11
rs11730650	53407	STX18	4	4576309	4.71E-03	883	9.91E-01	Down	0.01	13850	2.33	0.00
rs2874670	665	BNIP3L	8	26301575	4.71E-03	884	1.02E-09	Down	6.11	895	2.33	0.90
rs7298766	283358	B4GALNT3	12	531917	4.71E-03	885	7.18E-02	Down	1.80	6463	2.33	0.11
rs3735016	64764	CREB3L2	7	137016710	4.72E-03	886	4.81E-09	Up	5.85	1007	2.33	0.83

rs11665341	9811	KIAA0427	18	44607131	4.72E-03	887	9.93E-02	Down	1.65	6987	2.33	0.10
rs3774533	776	CACNA1D	3	53713360	4.73E-03	888	1.04E-08	Up	5.75	1047	2.33	0.80
rs10934493	51300	C3orf1	3	120728647	4.74E-03	889	4.49E-03	Up	2.84	3992	2.32	0.23
rs10934493	941	CD80	3	120728647	4.74E-03	890	9.12E-01	Up	0.11	13319	2.32	0.00
rs4761611	51134	CCDC41	12	93272226	4.76E-03	891	1.87E-03	Up	3.11	3498	2.32	0.27
rs6854303	8671	SLC4A4	4	72647788	4.76E-03	892	1.13E-11	Up	6.81	638	2.32	1.09
rs12003803	79817	MOBKL2B	9	27339580	4.77E-03	893	9.00E-02	Down	1.70	6813	2.32	0.10
rs2273840	10858	CYP46A1	14	99257897	4.78E-03	894	9.56E-01	Down	0.06	13591	2.32	0.00
rs2669432	1807	DPYS	8	105529206	4.79E-03	895	3.59E-05	Down	4.13	2208	2.32	0.44
rs6529638	10761	PLAC1		133449936	4.80E-03	896	7.24E-01	Down	0.35	12226	2.32	0.01
rs1580820	5130	PCYT1A	3	197454568	4.80E-03	897	2.21E-04	Down	3.69	2665	2.32	0.37
rs2455603	56676	ASCL3	11	8921514	4.81E-03	898	1.17E-01	Down	1.57	7243	2.32	0.09
rs2455603	56673	C11orf16	11	8921514	4.81E-03	899	3.17E-01	Down	1.00	9342	2.32	0.05
rs17628931	51166	AADAT	4	171405537	4.81E-03	900	4.61E-01	Up	0.74	10436	2.32	0.03
rs6536919	60592	SCOC	4	141636084	4.82E-03	901	4.60E-03	Up	2.83	4010	2.32	0.23
rs12354465	10580	SORBS1	10	97323448	4.82E-03	902	2.12E-10	Down	6.35	795	2.32	0.97
rs16891304	6575	SLC20A2	8	42401409	4.82E-03	903	1.23E-10	Down	6.44	764	2.32	0.99
rs9995522	7358	UGDH	4	39325762	4.83E-03	904	6.35E-27	Up	10.76	86	2.32	2.62
rs1995317	23167	EFR3A	8	132967044	4.85E-03	905	2.53E-01	Up	1.14	8794	2.31	0.06
rs11167990	1809	DPYSL3	5	146771949	4.85E-03	906	2.67E-21	Down	9.47	173	2.31	2.06
rs7641462	9076	CLDN1	3	191536478	4.85E-03	907	8.56E-01	Up	0.18	12991	2.31	0.01
rs8102860	55620	STAP2	19	4297348	4.86E-03	908	7.25E-02	Up	1.80	6477	2.31	0.11
rs16904712	23639	LRRC6	8	133694094	4.87E-03	909	9.91E-01	Down	0.01	13848	2.31	0.00
rs11949551	153642	ARSK	5	94963432	4.88E-03	910	3.20E-01	Up	0.99	9359	2.31	0.05
rs2284227	10842	C7orf16	7	31504040	4.88E-03	911	3.41E-01	Up	0.95	9532	2.31	0.05
rs6048226	85364	ZCCHC3	20	233323	4.88E-03	912	5.63E-09	Down	5.83	1022	2.31	0.82
rs6048226	140680	C20orf96	20	233323	4.88E-03	913	1.80E-01	Down	1.34	8027	2.31	0.07
rs3003609	1759	DNM1	9	128064309	4.89E-03	914	6.04E-02	Down	1.88	6243	2.31	0.12
rs3003609	25792	CIZ1	9	128064309	4.89E-03	915	9.07E-01	Up	0.12	13288	2.31	0.00
rs308052	2767	GNA11	19	3055629	4.91E-03	916	4.97E-08	Down	5.45	1221	2.31	0.73
rs7304239	3747	KCNC2	12	73840326	4.93E-03	917	3.70E-01	Down	0.90	9763	2.31	0.04
rs6573560	145376	C14orf50	14	64101287	4.94E-03	918	9.66E-01	Up	0.04	13663	2.31	0.00
rs8192306	649	BMP1	8	22071798	4.94E-03	919	2.82E-03	Down	2.99	3752	2.31	0.26
rs8192306	203190	LGI3	8	22071798	4.94E-03	920	5.78E-02	Down	1.90	6183	2.31	0.12
rs8192306	6440	SFTPC	8	22071798	4.94E-03	921	8.46E-01	Up	0.19	12934	2.31	0.01
rs10007572	55714	ODZ3	4	184066696	4.94E-03	922	7.96E-01	Up	0.26	12645	2.31	0.01
rs2322592	93649	MYOCD	17	12574875	4.95E-03	923	2.31E-03	Down	3.05	3634	2.31	0.26
rs2176135	57282	SLC4A10	2	162626961	4.96E-03	924	5.18E-02	Up	1.94	6035	2.30	0.13
rs2668207	590	BCHE	3	166976426	4.99E-03	925	4.00E-04	Down	3.54	2886	2.30	0.34
rs2306630	8148	TAF15	17	31206454	5.00E-03	926	6.80E-04	Up	3.40	3069	2.30	0.32
rs2306630	256957	C17orf66	17	31206454	5.00E-03	927	3.98E-01	Down	0.85	9970	2.30	0.04
rs4285886	2313	FLI1	11	128138864	5.01E-03	928	1.21E-01	Up	1.55	7307	2.30	0.09
rs4285886	2314	FLII	11	128138864	5.01E-03	929	3.99E-01	Down	0.84	9979	2.30	0.04
rs13068999	25917	THUMPD3	3	9371030	5.01E-03	930	5.36E-01	Down	0.62	10983	2.30	0.03
rs1863086	114805	GALNT13	2	154725833	5.02E-03	931	5.72E-01	Up	0.56	11259	2.30	0.02
rs17775172	284040	CDRT4	17	15298132	5.02E-03	932	6.88E-01	Up	0.40	11992	2.30	0.02
rs9940751	64785	GINS3	16	56993437	5.02E-03	933	2.90E-02	Up	2.18	5427	2.30	0.15
rs13105185	64579	NDST4	4	116389943	5.03E-03	934	6.06E-01	Up	0.52	11472	2.30	0.02
rs9532388	10186	LHFP	13	38991623	5.05E-03	935	3.00E-04	Down	3.62	2773	2.30	0.35
rs112750	25996	REXO2	11	113833495	5.07E-03	936	3.46E-02	Up	2.11	5610	2.29	0.15
rs10490910	374355	C10orf96	10	118066445	5.08E-03	937	7.83E-01	Up	0.28	12559	2.29	0.01
rs4883196	144568	A2ML1	12	8925139	5.08E-03	938	8.74E-02	Up	1.71	6762	2.29	0.11
rs10079031	5924	RASGRF2	5	80521854	5.08E-03	939	1.92E-03	Down	3.10	3512	2.29	0.27
rs549831	83446	CCDC70	13	51336114	5.09E-03	940	1.96E-03	Down	3.10	3520	2.29	0.27
rs11791772	169792	GLIS3	9	3948355	5.10E-03	941	2.07E-09	Down	5.99	939	2.29	0.87
rs7182576	23191	CYFIP1	15	20546036	5.10E-03	942	1.75E-06	Up	4.78	1650	2.29	0.58
rs16972337	57214	KIAA1199	15	78841066	5.10E-03	943	1.98E-01	Down	1.29	8240	2.29	0.07
rs671689	2888	GRB14	2	165177912	5.11E-03	944	9.53E-01	Down	0.06	13578	2.29	0.00
rs11001217	23522	MYST4	10	76380082	5.11E-03	945	5.89E-02	Down	1.89	6216	2.29	0.12
rs6752022	55022	PID1	2	229915463	5.13E-03	946	1.11E-01	Down	1.60	7153	2.29	0.10
rs7037389	3452	IFNA21	9	21160364	5.13E-03	947	4.71E-01	Down	0.72	10504	2.29	0.03
rs1569985	55816	DOK5	20	52690581	5.14E-03	948	6.27E-01	Down	0.49	11629	2.29	0.02
rs9297594	114569	MAL2	8	120287483	5.16E-03	949	6.69E-25	Up	10.30	116	2.29	2.42
rs7728957	11174	ADAMTS6	5	64541733	5.17E-03	950	3.76E-01	Down	0.89	9809	2.29	0.04
rs4492611	1382	CRABP2	1	153476290	5.19E-03	951	2.26E-04	Down	3.69	2671	2.28	0.36
rs4492611	10763	NES	1	153476290	5.19E-03	952	3.69E-01	Down	0.90	9751	2.28	0.04
rs4592619	81614	NIPA2	15	20585244	5.20E-03	953	4.06E-02	Up	2.05	5760	2.28	0.14
rs1532010	114876	OSBPL1A	18	20182039	5.20E-03	954	7.32E-04	Down	3.38	3094	2.28	0.31
rs4263667	202865	C7orf33	7	147745827	5.20E-03	955	2.96E-01	Up	1.05	9162	2.28	0.05
rs2805	79611	ACSS3	12	80152977	5.21E-03	956	5.39E-22	Down	9.64	157	2.28	2.13
rs2805	8499	PPFIA2	12	80152977	5.21E-03	957	5.40E-08	Up	5.44	1229	2.28	0.73
rs10455750	401288	C6orf122	6	170031086	5.21E-03	958	7.78E-02	Up	1.76	6591	2.28	0.11
rs12700120	23288	IQCE	7	2402261	5.24E-03	959	5.01E-04	Up	3.48	2962	2.28	0.33
rs3734444	653	BMP5	6	55847512	5.24E-03	960	1.88E-03	Down	3.11	3501	2.28	0.27
rs803401	57480	PLEKHG1	6	151251744	5.26E-03	961	9.64E-02	Down	1.66	6929	2.28	0.10
rs11079974	56934	CA10	17	47073126	5.26E-03	962	1.51E-01	Down	1.44	7688	2.28	0.08
rs13378730	54937	SOHLH2	13	35682800	5.26E-03	963	8.77E-01	Up	0.16	13098	2.28	0.01
rs4921133	114898	C1QTNF2	5	159719684	5.26E-03	964	7.11E-01	Down	0.37	12137	2.28	0.01
rs9328341	51299	NRN1	6	5972081	5.28E-03	965	6.85E-03	Up	2.70	4246	2.28	0.22
rs10267275	222235	FBXL13	7	102093027	5.29E-03	966	6.12E-01	Down	0.51	11519	2.28	0.02
rs11121449	388591	RNF207	1	6186709	5.29E-03	967	2.25E-06	Down	4.73	1685	2.28	0.56

rs11121449	6146	RPL22	1	6186709	5.29E-03	968	6.90E-03	Up	2.70	4249	2.28	0.22
rs2809823	54112	GPR88	1	100715783	5.29E-03	969	4.65E-01	Down	0.73	10465	2.28	0.03
rs4712827	55003	PAK1IP1	6	10794047	5.30E-03	970	7.36E-14	Down	7.48	461	2.28	1.31
rs4712827	347744	C6orf52	6	10794047	5.30E-03	971	1.28E-03	Down	3.22	3321	2.28	0.29
rs10817577	85301	COL27A1	9	114023371	5.30E-03	972	3.34E-02	Down	2.13	5575	2.28	0.15
rs4410884	8840	WISP1	8	134256900	5.31E-03	973	9.68E-01	Down	0.04	13683	2.28	0.00
rs704145	212	ALAS2		54918304	5.32E-03	974	1.56E-03	Up	3.16	3405	2.27	0.28
rs1923886	3356	HTR2A	13	46321292	5.32E-03	975	9.27E-02	Up	1.68	6878	2.27	0.10
rs704145	27301	APEX2		54918304	5.32E-03	976	2.96E-01	Up	1.04	9163	2.27	0.05
rs704145	5207	PFKFB1		54918304	5.32E-03	977	8.11E-01	Down	0.24	12726	2.27	0.01
rs11174561	283416	C12orf61	12	61294203	5.32E-03	978	9.74E-01	Up	0.03	13722	2.27	0.00
rs4267826	65056	GPBP1	5	56565313	5.33E-03	979	2.17E-01	Up	1.23	8433	2.27	0.07
rs11769723	93664	CADPS2	7	121832614	5.34E-03	980	1.89E-01	Up	1.31	8135	2.27	0.07
rs6740374	10651	MTX2	2	176949731	5.34E-03	981	3.02E-09	Up	5.93	969	2.27	0.85
rs12051877	10458	BAIAP2	17	76632362	5.35E-03	982	3.28E-07	Up	5.11	1429	2.27	0.65
rs6727435	22848	AAK1	2	69775887	5.35E-03	983	3.64E-02	Up	2.09	5663	2.27	0.14
rs179660	1690	COCH	14	30398905	5.35E-03	984	1.16E-05	Down	4.39	1977	2.27	0.49
rs6838613	201780	SLC10A4	4	48317467	5.35E-03	985	2.04E-02	Up	2.32	5090	2.27	0.17
rs6838613	326340	ZAR1	4	48317467	5.35E-03	986	7.18E-01	Up	0.36	12190	2.27	0.01
rs9554080	51082	POLR1D	13	27108301	5.36E-03	987	7.73E-01	Up	0.29	12517	2.27	0.01
rs2455236	10788	IQGAP2	5	75977734	5.37E-03	988	5.61E-16	Up	8.10	351	2.27	1.53
rs5917925	54880	BCOR		39661646	5.39E-03	989	7.68E-07	Up	4.94	1534	2.27	0.61
rs8025124	5923	RASGRF1	15	77091525	5.40E-03	990	1.93E-01	Down	1.30	8178	2.27	0.07
rs9511184	338872	C1QTNF9	13	23778292	5.41E-03	991	4.92E-01	Down	0.69	10665	2.27	0.03
rs9511184	221178	SPATA13	13	23778292	5.41E-03	992	8.78E-01	Down	0.15	13106	2.27	0.01
rs7331661	9882	TBC1D4	13	74936997	5.43E-03	993	4.25E-12	Up	6.93	619	2.27	1.14
rs2313090	7702	ZNF143	11	9459168	5.43E-03	994	1.25E-01	Down	1.54	7367	2.27	0.09
rs8108998	55850	USE1	19	17175874	5.43E-03	995	7.60E-03	Up	2.67	4316	2.26	0.21
rs8108998	4650	MYO9B	19	17175874	5.43E-03	996	6.75E-01	Down	0.42	11916	2.26	0.02
rs9457309	6953	TCP10	6	167738572	5.44E-03	997	9.35E-01	Down	0.08	13464	2.26	0.00
rs12202737	79465	ULBP3	6	150479860	5.44E-03	998	4.30E-02	Down	2.02	5827	2.26	0.14
rs11709661	285282	RABL3	3	121959848	5.44E-03	999	4.87E-03	Down	2.82	4047	2.26	0.23
rs11709661	2960	GTF2E1	3	121959848	5.44E-03	1000	8.94E-01	Down	0.13	13211	2.26	0.00
rs6873550	57507	ZNF608	5	124064454	5.44E-03	1001	6.86E-01	Down	0.40	11983	2.26	0.02
rs1914525	119587	CPXM2	10	125535626	5.45E-03	1002	7.85E-01	Up	0.27	12578	2.26	0.01
rs11648690	1006	CDH8	16	60297418	5.45E-03	1003	5.38E-01	Down	0.62	10999	2.26	0.03
rs730959	51676	ASB2	14	93492099	5.46E-03	1004	1.16E-09	Down	6.09	903	2.26	0.89
rs7654329	1363	CPE	4	166680583	5.46E-03	1005	8.59E-02	Up	1.72	6733	2.26	0.11
rs3124502	5524	PPP2R4	9	128953795	5.47E-03	1006	1.74E-04	Up	3.75	2581	2.26	0.38
rs3124502	1384	CRAT	9	128953795	5.47E-03	1007	4.40E-04	Up	3.51	2912	2.26	0.34
rs2761691	259	AMBP	9	113921296	5.48E-03	1008	6.32E-01	Up	0.48	11657	2.26	0.02
rs1418396	2894	GRID1	10	87690805	5.48E-03	1009	7.15E-02	Down	1.80	6460	2.26	0.11
rs7959876	55885	LMO3	12	16663658	5.49E-03	1010	1.09E-04	Down	3.87	2474	2.26	0.40
rs12806627	24147	FJX1	11	35580059	5.49E-03	1011	7.11E-01	Up	0.37	12138	2.26	0.01
rs3858145	220202	ATOH7	10	69681844	5.49E-03	1012	6.85E-01	Down	0.41	11975	2.26	0.02
rs11174554	23041	MON2	12	61255935	5.50E-03	1013	4.45E-01	Up	0.76	10304	2.26	0.04
rs17539365	57733	GBA3	4	22391074	5.51E-03	1014	2.12E-02	Up	2.30	5131	2.26	0.17
rs12099971	160418	TMTC3	12	87074983	5.52E-03	1015	3.03E-01	Up	1.03	9216	2.26	0.05
rs1068940	283455	KSR2	12	116370480	5.52E-03	1016	2.24E-01	Up	1.22	8494	2.26	0.07
rs1068940	22866	CNKSR2	12	116370480	5.52E-03	1017	2.32E-01	Up	1.20	8584	2.26	0.06
rs13155727	10085	EDIL3	5	83530960	5.52E-03	1018	6.20E-04	Down	3.42	3040	2.26	0.32
rs7612669	285220	EPHA6	3	98262016	5.53E-03	1019	8.80E-01	Up	0.15	13131	2.26	0.01
rs4920309	84966	IGSF21	1	18308898	5.54E-03	1020	1.78E-01	Down	1.35	7994	2.26	0.07
rs1052502	54806	AHI1	6	135648258	5.55E-03	1021	2.24E-01	Down	1.22	8498	2.26	0.06
rs10899433	65987	KCTD14	11	77426876	5.56E-03	1022	3.89E-02	Down	2.07	5725	2.26	0.14
rs10502330	23136	EPB41L3	18	5407253	5.56E-03	1023	9.50E-15	Down	7.75	408	2.25	1.40
rs12548629	79870	BAALC	8	104270577	5.58E-03	1024	6.44E-02	Down	1.85	6330	2.25	0.12
rs410262	2151	F2RL2	5	75962944	5.58E-03	1025	4.44E-11	Up	6.58	708	2.25	1.04
rs1411921	401546	C9orf152	9	110064448	5.59E-03	1026	1.47E-05	Up	4.33	2033	2.25	0.48
rs1063134	3761	KCNJ4	22	37147198	5.59E-03	1027	7.90E-01	Up	0.27	12607	2.25	0.01
rs744459	8991	SELENBP1	1	148154490	5.62E-03	1028	2.61E-05	Down	4.20	2143	2.25	0.46
rs2073917	3683	ITGAL	16	30423993	5.64E-03	1029	6.94E-01	Up	0.39	12027	2.25	0.02
rs228274	5691	PSMB3	17	34162587	5.64E-03	1030	4.86E-06	Up	4.57	1808	2.25	0.53
rs759818	353514	LILRA5	19	59513631	5.64E-03	1031	2.67E-01	Down	1.11	8926	2.25	0.06
rs759818	11026	LILRA3	19	59513631	5.64E-03	1032	4.47E-01	Up	0.76	10323	2.25	0.04
rs4428268	55203	LGI2	4	24704427	5.65E-03	1033	2.88E-02	Down	2.19	5419	2.25	0.15
rs11713248	11073	TOPBP1	3	134874889	5.65E-03	1034	2.79E-02	Up	2.20	5391	2.25	0.16
rs6770339	54763	ROPN1	3	125186729	5.65E-03	1035	8.08E-01	Up	0.24	12711	2.25	0.01
rs11941841	65997	RASL11B	4	53566842	5.66E-03	1036	2.67E-04	Down	3.65	2737	2.25	0.36
rs6590611	6239	RREB1	11	131405767	5.67E-03	1037	6.57E-01	Down	0.44	11816	2.25	0.02
rs7157207	1053	CEBPE	14	22668689	5.68E-03	1038	8.73E-01	Down	0.16	13081	2.25	0.01
rs1147700	11167	FSTL1	3	121606246	5.71E-03	1039	3.72E-01	Down	0.89	9783	2.24	0.04
rs6469877	64798	DEPDC6	8	121005702	5.71E-03	1040	1.55E-01	Down	1.42	7748	2.24	0.08
rs7103676	26541	OR10D1P	11	123516790	5.72E-03	1041	1.89E-01	Up	1.31	8144	2.24	0.07
rs1209621	113130	CDCA5	11	64627355	5.73E-03	1042	1.92E-02	Up	2.34	5036	2.24	0.17
rs17714461	9622	KLK4	19	56116918	5.73E-03	1043	2.79E-04	Up	3.63	2753	2.24	0.36
rs4141404	3985	LIMK2	22	29999739	5.73E-03	1044	4.02E-04	Down	3.54	2887	2.24	0.34
rs1377296	800	CALD1	7	133922872	5.73E-03	1045	4.48E-38	Down	12.90	22	2.24	3.73
rs2296227	23032	USP33	1	77913766	5.76E-03	1046	4.25E-03	Up	2.86	3970	2.24	0.24
rs8023115	8812	CCNK	14	99008093	5.76E-03	1047	4.65E-01	Up	0.73	10461	2.24	0.03
rs2830052	351	APP	21	26387468	5.77E-03	1048	5.97E-01	Down	0.53	11414	2.24	0.02

rs1286762	5915	RARB	3	25573913	5.77E-03	1049	1.53E-09	Down	6.04	923	2.24	0.88
rs1013062	7913	DEK	6	18345640	5.78E-03	1050	2.06E-05	Down	4.26	2095	2.24	0.47
rs10059384	80315	CPEB4	5	173281722	5.78E-03	1051	7.60E-01	Down	0.31	12440	2.24	0.01
rs1539243	9641	IKBKE	1	203036182	5.79E-03	1052	4.59E-01	Down	0.74	10416	2.24	0.03
rs525380	5322	PLA2G5	1	20154680	5.79E-03	1053	5.23E-01	Up	0.64	10883	2.24	0.03
rs12031994	10000	AKT3	1	240243350	5.80E-03	1054	3.02E-01	Down	1.03	9205	2.24	0.05
rs11000204	51008	ASCC1	10	73608572	5.80E-03	1055	6.11E-01	Down	0.51	11514	2.24	0.02
rs5750824	10454	MAP3K7IP1	22	38154623	5.80E-03	1056	1.09E-01	Up	1.60	7137	2.24	0.10
rs12587301	26037	SIPA1L1	14	71156470	5.82E-03	1057	8.80E-02	Down	1.71	6771	2.24	0.11
rs7799	1997	ELF1	13	40431052	5.82E-03	1058	1.92E-06	Down	4.76	1662	2.24	0.57
rs7799	84337	ELOF1	13	40431052	5.82E-03	1059	1.03E-04	Up	3.88	2460	2.24	0.40
rs2076003	4524	MTHFR	1	11818413	5.84E-03	1060	7.19E-01	Down	0.36	12194	2.23	0.01
rs2076003	1185	CLCN6	1	11818413	5.84E-03	1061	9.56E-01	Up	0.06	13592	2.23	0.00
rs1018578	2098	ESD	13	46274029	5.84E-03	1062	1.51E-12	Down	7.07	570	2.23	1.18
rs2899446	79895	ATP8B4	15	48094708	5.85E-03	1063	8.02E-01	Up	0.25	12687	2.23	0.01
rs3784244	91875	TTC5	14	19850207	5.86E-03	1064	1.53E-01	Down	1.43	7714	2.23	0.08
rs3784244	57820	CCNB1IP1	14	19850207	5.86E-03	1065	3.85E-01	Down	0.87	9881	2.23	0.04
rs8069296	51751	HIGD1B	17	40290585	5.86E-03	1066	1.10E-01	Down	1.60	7148	2.23	0.10
rs8069296	9343	EFTUD2	17	40290585	5.86E-03	1067	4.11E-01	Down	0.82	10064	2.23	0.04
rs1630656	8533	COPS3	17	17102612	5.88E-03	1068	5.72E-01	Down	0.56	11261	2.23	0.02
rs4395024	83660	TLN2	15	60929514	5.88E-03	1069	8.58E-03	Up	2.63	4390	2.23	0.21
rs1057617	117177	RAB3IP	12	68500427	5.90E-03	1070	1.10E-14	Up	7.73	411	2.23	1.40
rs381575	4724	NDUFS4	5	52948648	5.90E-03	1071	1.57E-06	Down	4.80	1639	2.23	0.58
rs7069703	119504	C10orf104	10	73640457	5.91E-03	1072	8.45E-11	Down	6.49	750	2.23	1.01
rs601436	57617	VPS18	15	38958424	5.91E-03	1073	4.08E-02	Down	2.05	5767	2.23	0.14
rs601436	171177	RHOV	15	38958424	5.91E-03	1074	9.57E-02	Down	1.67	6918	2.23	0.10
rs859141	4033	LRMP	12	25113291	5.93E-03	1075	1.19E-01	Down	1.56	7274	2.23	0.09
rs17101367	80344	WDR23	14	23657507	5.93E-03	1076	2.52E-04	Up	3.66	2712	2.23	0.36
rs17101367	5720	PSME1	14	23657507	5.93E-03	1077	3.84E-01	Up	0.87	9869	2.23	0.04
rs1149024	4146	MATN1	1	30846166	5.95E-03	1078	4.13E-02	Down	2.04	5781	2.23	0.14
rs17624212	51705	EMCN	4	101707863	5.95E-03	1079	5.10E-03	Down	2.80	4080	2.23	0.23
rs1487745	23362	PSD3	8	18665612	5.95E-03	1080	6.43E-12	Up	6.88	627	2.23	1.12
rs2200457	8001	GLRA3	4	176074006	5.96E-03	1081	3.82E-04	Up	3.55	2864	2.22	0.34
rs10928665	11249	NXPH2	2	139330463	5.96E-03	1082	5.25E-01	Up	0.64	10904	2.22	0.03
rs12930054	4629	MYH11	16	15790710	5.99E-03	1083	2.21E-37	Down	12.78	26	2.22	3.67
rs454376	7596	ZNF45	19	49127007	5.99E-03	1084	9.50E-01	Up	0.06	13552	2.22	0.00
rs10512309	1539	CYLC2	9	102869282	6.00E-03	1085	8.51E-01	Up	0.19	12963	2.22	0.01
rs7226272	10040	TOM1L1	17	50401446	6.00E-03	1086	2.22E-18	Up	8.73	257	2.22	1.77
rs7226272	252983	STXBP4	17	50401446	6.00E-03	1087	1.43E-03	Up	3.19	3358	2.22	0.28
rs7226272	1353	COX11	17	50401446	6.00E-03	1088	2.07E-01	Down	1.26	8323	2.22	0.07
rs456699	3281	HSBP1	16	82391190	6.00E-03	1089	1.84E-07	Up	5.21	1363	2.22	0.67
rs17664384	7410	VAV2	9	133903620	6.01E-03	1090	3.74E-07	Up	5.08	1447	2.22	0.64
rs4901053	51199	NIN	14	50287324	6.01E-03	1091	3.77E-10	Up	6.25	838	2.22	0.94
rs4941077	220441	RNF152	18	57697774	6.02E-03	1092	4.62E-01	Down	0.74	10444	2.22	0.03
rs7870226	80380	PDCD1LG2	9	5520017	6.02E-03	1093	4.09E-01	Up	0.83	10047	2.22	0.04
rs17022799	57458	TMCC3	12	93488192	6.02E-03	1094	3.72E-01	Up	0.89	9784	2.22	0.04
rs4326896	23281	KIAA0774	13	28950150	6.03E-03	1095	8.42E-02	Up	1.73	6704	2.22	0.11
rs660361	4684	NCAM1	11	112659335	6.03E-03	1096	9.32E-15	Down	7.75	406	2.22	1.40
rs17607104	1417	CRYBB3	22	23911216	6.03E-03	1097	2.38E-01	Down	1.18	8643	2.22	0.06
rs1468503	10577	NPC2	14	74031105	6.04E-03	1098	3.31E-02	Up	2.13	5567	2.22	0.15
rs6632882	94056	SYAP1		16524877	6.04E-03	1099	3.72E-02	Up	2.08	5679	2.22	0.14
rs12780135	55680	RUFY2	10	69797702	6.04E-03	1100	4.54E-01	Down	0.75	10371	2.22	0.03
rs208381	8537	BCAS1	20	52044947	6.05E-03	1101	8.94E-01	Down	0.13	13209	2.22	0.00
rs747063	4773	NFATC2	20	49535610	6.05E-03	1102	2.54E-04	Down	3.66	2714	2.22	0.36
rs2382573	5931	RBBP7		16638152	6.07E-03	1103	1.74E-03	Down	3.13	3456	2.22	0.28
rs2306393	56890	MDM1	12	66995028	6.09E-03	1104	7.45E-04	Down	3.37	3104	2.22	0.31
rs7512555	25893	TRIM58	1	244327607	6.09E-03	1105	2.83E-01	Up	1.07	9061	2.22	0.05
rs8030479	123355	LRRC28	15	97712007	6.10E-03	1106	7.18E-03	Up	2.69	4274	2.21	0.21
rs7999702	3621	ING1	13	110180265	6.11E-03	1107	8.51E-04	Up	3.34	3155	2.21	0.31
rs1056607	3930	LBR	1	221918396	6.12E-03	1108	3.44E-01	Up	0.95	9552	2.21	0.05
rs6740224	10254	STAM2	2	152858298	6.13E-03	1109	3.76E-02	Up	2.08	5696	2.21	0.14
rs7693621	54502	RBM47	4	40288842	6.13E-03	1110	4.30E-01	Down	0.79	10190	2.21	0.04
rs12994970	150572	SMYD1	2	88255647	6.14E-03	1111	1.52E-01	Up	1.43	7700	2.21	0.08
rs12994970	2168	FABP1	2	88255647	6.14E-03	1112	4.26E-01	Down	0.80	10162	2.21	0.04
rs3789257	1056	CEL	9	132950160	6.15E-03	1113	3.22E-01	Down	0.99	9372	2.21	0.05
rs3936674	2099	ESR1	6	152259425	6.16E-03	1114	9.37E-04	Up	3.31	3189	2.21	0.30
rs8134520	5211	PFKL	21	44548483	6.16E-03	1115	6.04E-02	Up	1.88	6245	2.21	0.12
rs10472069	115827	RAB3C	5	58041554	6.16E-03	1116	1.12E-01	Down	1.59	7177	2.21	0.10
rs8134520	326	AIRE	21	44548483	6.16E-03	1117	9.44E-01	Down	0.07	13515	2.21	0.00
rs16928536	219699	UNC5B	10	72667088	6.17E-03	1118	4.12E-05	Down	4.10	2235	2.21	0.44
rs2571575	3908	LAMA2	6	129875898	6.18E-03	1119	6.59E-09	Down	5.80	1029	2.21	0.82
rs11108730	121441	NEDD1	12	95798404	6.18E-03	1120	6.66E-01	Up	0.43	11858	2.21	0.02
rs2288058	583	BBS2	16	55106278	6.21E-03	1121	5.11E-02	Down	1.95	6016	2.21	0.13
rs3763913	6764	ST5	11	8907806	6.22E-03	1122	1.57E-27	Down	10.87	82	2.21	2.68
rs3763913	56672	C11orf17	11	8907806	6.22E-03	1123	2.41E-01	Down	1.17	8661	2.21	0.06
rs12463169	79629	OCEL1	19	17182669	6.22E-03	1124	7.86E-03	Up	2.66	4329	2.21	0.21
rs7609252	699	BUB1	2	111135659	6.23E-03	1125	2.62E-05	Up	4.20	2144	2.21	0.46
rs1787861	4161	MC5R	18	13823487	6.23E-03	1126	1.27E-01	Up	1.53	7398	2.21	0.09
rs11218301	6653	SORL1	11	120847297	6.29E-03	1127	6.69E-01	Down	0.43	11877	2.20	0.02
rs11750538	4651	MYO10	5	16822273	6.31E-03	1128	6.70E-10	Up	6.24	843	2.20	0.92
rs12254965	387718	C10orf122	10	127287028	6.32E-03	1129	9.35E-01	Up	0.08	13462	2.20	0.00

rs1871626	22884	WDR37	10	1156400	6.32E-03	1130	1.23E-02	Up	2.50	4649	2.20	0.19
rs11574631	3687	ITGAX	16	31271478	6.32E-03	1131	8.75E-03	Up	2.62	4410	2.20	0.21
rs2001776	6595	SMARCA2	9	2110825	6.34E-03	1132	1.47E-02	Down	2.44	4797	2.20	0.18
rs8042680	9055	PRC1	15	89322341	6.34E-03	1133	1.99E-01	Up	1.28	8249	2.20	0.07
rs7559158	25940	FAM98A	2	33748573	6.34E-03	1134	1.76E-01	Up	1.35	7968	2.20	0.08
rs1795107	66000	TMEM108	3	134588211	6.35E-03	1135	2.14E-01	Down	1.24	8395	2.20	0.07
rs1795107	8419	BFSP2	3	134588211	6.35E-03	1136	3.24E-01	Down	0.99	9391	2.20	0.05
rs10850830	59341	TRPV4	12	108734619	6.35E-03	1137	6.79E-02	Down	1.83	6391	2.20	0.12
rs13296679	8395	PIP5K1B	9	68577542	6.37E-03	1138	6.80E-01	Down	0.41	11946	2.20	0.02
rs7856692	113220	KIF12	9	113946159	6.37E-03	1139	9.58E-02	Up	1.67	6922	2.20	0.10
rs4906205	9895	KIAA0329	14	102002200	6.38E-03	1140	9.38E-06	Down	4.43	1933	2.20	0.50
rs2803572	11155	LDB3	10	88445222	6.38E-03	1141	2.17E-24	Down	10.19	122	2.19	2.37
rs1706902	60561	RINT1	7	104789227	6.40E-03	1142	2.83E-01	Up	1.07	9062	2.19	0.05
rs7000333	54714	CNGB3	8	87647247	6.40E-03	1143	5.22E-01	Down	0.64	10871	2.19	0.03
rs11666431	58492	ZNF77	19	2904087	6.41E-03	1144	7.99E-01	Down	0.25	12668	2.19	0.01
rs3087813	167153	PAPD4	5	79017306	6.45E-03	1145	1.17E-02	Down	2.52	4621	2.19	0.19
rs7127746	57586	SYT13	11	45216520	6.45E-03	1146	8.43E-01	Up	0.20	12920	2.19	0.01
rs8103241	4784	NFIX	19	12983612	6.46E-03	1147	5.00E-01	Up	0.67	10715	2.19	0.03
rs1411780	118932	ANKRD22	10	90568519	6.47E-03	1148	3.51E-01	Up	0.93	9602	2.19	0.05
rs10928399	55777	MBD5	2	149111628	6.48E-03	1149	2.34E-03	Down	3.04	3643	2.19	0.26
rs7590594	151230	KLHL23	2	170397621	6.50E-03	1150	1.94E-03	Up	3.10	3517	2.19	0.27
rs2241763	2863	GPR39	2	133008731	6.50E-03	1151	7.47E-01	Down	0.32	12355	2.19	0.01
rs1171134	167681	PRSS35	6	84261196	6.50E-03	1152	3.81E-01	Up	0.88	9840	2.19	0.04
rs660981	7405	UVRAG	11	75537827	6.50E-03	1153	4.29E-01	Up	0.79	10181	2.19	0.04
rs27061	81037	CLPTM1L	5	1415793	6.50E-03	1154	5.65E-02	Up	1.91	6148	2.19	0.12
rs11667809	57348	TTYH1	19	59605732	6.51E-03	1155	5.81E-01	Up	0.55	11324	2.19	0.02
rs12322866	8076	MFAP5	12	8696354	6.52E-03	1156	2.43E-01	Down	1.17	8683	2.19	0.06
rs17387019	55259	CASC1	12	25155526	6.53E-03	1157	4.06E-01	Up	0.83	10028	2.19	0.04
rs33840	5184	PEPD	19	38702129	6.53E-03	1158	2.97E-01	Down	1.04	9171	2.19	0.05
rs159157	7862	BRPF1	3	9753315	6.56E-03	1159	2.32E-02	Up	2.27	5213	2.18	0.16
rs880774	2257	FGF12	3	193873452	6.56E-03	1160	9.15E-01	Up	0.11	13349	2.18	0.00
rs1466018	7849	PAX8	2	113737078	6.56E-03	1161	6.14E-01	Up	0.50	11536	2.18	0.02
rs17099839	10329	TMEM5	12	62488500	6.56E-03	1162	7.56E-13	Up	7.14	554	2.18	1.21
rs2070484	9940	DLEC1	3	38102205	6.56E-03	1163	2.61E-01	Down	1.12	8873	2.18	0.06
rs12869881	688	KLF5	13	72556255	6.58E-03	1164	4.93E-05	Down	4.06	2280	2.18	0.43
rs12617170	129563	DIS3L2	2	232987270	6.58E-03	1165	6.37E-01	Up	0.47	11682	2.18	0.02
rs356663	2176	FANCC	9	95118218	6.58E-03	1166	5.44E-01	Down	0.61	11044	2.18	0.03
rs6978731	10165	SLC25A13	7	95399575	6.58E-03	1167	2.07E-13	Up	7.34	495	2.18	1.27
rs9478329	23345	SYNE1	6	152799177	6.58E-03	1168	5.74E-04	Down	3.44	3010	2.18	0.32
rs6451185	5618	PRLR	5	35197908	6.60E-03	1169	6.71E-01	Up	0.43	11893	2.18	0.02
rs230661	57558	USP35	11	77562430	6.60E-03	1170	6.64E-01	Down	0.43	11851	2.18	0.02
rs2242160	8622	PDE8B	5	76750887	6.64E-03	1171	1.19E-10	Down	6.44	761	2.18	0.99
rs2569686	3802	KIR2DL1	19	59993623	6.65E-03	1172	5.18E-01	Down	0.65	10846	2.18	0.03
rs1336199	65217	PCDH15	10	56172299	6.65E-03	1173	7.37E-01	Down	0.34	12299	2.18	0.01
rs2569686	3811	KIR3DL1	19	59993623	6.65E-03	1174	8.44E-01	Down	0.20	12926	2.18	0.01
rs13342692	162515	SLC16A11	17	6887011	6.66E-03	1175	4.78E-02	Down	1.98	5945	2.18	0.13
rs12598144	10368	CACNG3	16	24218383	6.67E-03	1176	3.30E-01	Up	0.97	9441	2.18	0.05
rs7618021	1608	DGKG	3	187566313	6.67E-03	1177	7.63E-04	Up	3.37	3117	2.18	0.31
rs757439	7337	UBE3A	15	23123915	6.69E-03	1178	9.03E-01	Down	0.12	13271	2.17	0.00
rs12516233	55819	RNF130	5	179393919	6.69E-03	1179	6.84E-01	Up	0.41	11969	2.17	0.02
rs3753151	6009	RHEB	7	150622633	6.69E-03	1180	1.21E-02	Down	2.51	4644	2.17	0.19
rs11714038	166336	PRICKLE2	3	64109160	6.69E-03	1181	2.07E-06	Down	4.75	1674	2.17	0.57
rs4920323	127707	KLHDC7A	1	18551443	6.70E-03	1182	2.15E-01	Up	1.24	8414	2.17	0.07
rs12356233	56647	BCCIP	10	127524920	6.70E-03	1183	2.55E-04	Up	3.66	2715	2.17	0.36
rs12356233	55760	DHX32	10	127524920	6.70E-03	1184	1.37E-01	Up	1.49	7531	2.17	0.09
rs2051428	130	ADH6	4	100480364	6.70E-03	1185	1.91E-01	Up	1.31	8156	2.17	0.07
rs2116459	5361	PLXNA1	3	128240203	6.70E-03	1186	1.44E-01	Down	1.46	7621	2.17	0.08
rs2074647	9628	RGS6	14	72098931	6.70E-03	1187	2.09E-01	Down	1.26	8342	2.17	0.07
rs9456496	3482	IGF2R	6	160413801	6.71E-03	1188	5.79E-02	Up	1.90	6187	2.17	0.12
rs7216881	247	ALOX15B	17	7895090	6.71E-03	1189	7.38E-01	Up	0.33	12301	2.17	0.01
rs754437	79845	RNF122	8	33541076	6.71E-03	1190	7.96E-02	Down	1.75	6625	2.17	0.11
rs4444235	652	BMP4	14	53480669	6.73E-03	1191	1.09E-01	Down	1.60	7140	2.17	0.10
rs2817168	63976	PRDM16	1	3036847	6.74E-03	1192	4.20E-01	Down	0.81	10118	2.17	0.04
rs6532011	1834	DSPP	4	88867737	6.75E-03	1193	9.07E-01	Down	0.12	13289	2.17	0.00
rs11860295	146212	KCTD19	16	65873735	6.76E-03	1194	9.68E-02	Down	1.66	6940	2.17	0.10
rs11860295	25894	PLEKHG4	16	65873735	6.76E-03	1195	9.13E-01	Up	0.11	13337	2.17	0.00
rs10491310	6879	TAF7	5	140669944	6.76E-03	1196	2.64E-04	Up	3.65	2734	2.17	0.36
rs10491310	83884	SLC25A2	5	140669944	6.76E-03	1197	5.87E-01	Up	0.54	11352	2.17	0.02
rs1407300	7010	TEK	9	27220196	6.76E-03	1198	1.80E-03	Down	3.12	3475	2.17	0.27
rs876380	154197	PNLDC1	6	160209679	6.77E-03	1199	6.46E-01	Up	0.46	11751	2.17	0.02
rs4686692	10644	IGF2BP2	3	186963770	6.78E-03	1200	9.62E-01	Up	0.05	13633	2.17	0.00
rs6029941	25939	SAMHD1	20	34952889	6.78E-03	1201	1.00E-01	Down	1.64	6998	2.17	0.10
rs381872	7638	ZNF221	19	49157996	6.78E-03	1202	1.58E-01	Up	1.41	7786	2.17	0.08
rs1539567	90678	LRSAM1	9	127321720	6.78E-03	1203	1.50E-01	Up	1.44	7682	2.17	0.08
rs10797094	4720	NDUFS2	1	157995745	6.79E-03	1204	1.21E-01	Down	1.55	7311	2.17	0.09
rs10797094	9507	ADAMTS4	1	157995745	6.79E-03	1205	2.31E-01	Up	1.20	8580	2.17	0.06
rs10797094	2207	FCER1G	1	157995745	6.79E-03	1206	5.01E-01	Up	0.67	10727	2.17	0.03
rs10797094	336	APOA2	1	157995745	6.79E-03	1207	5.24E-01	Down	0.64	10900	2.17	0.03
rs10797094	84134	TOMM40L	1	157995745	6.79E-03	1208	6.95E-01	Up	0.39	12038	2.17	0.02
rs10778284	55576	STAB2	12	102632977	6.81E-03	1209	7.83E-01	Up	0.28	12560	2.17	0.01
rs2838760	104	ADARB1	21	45305933	6.82E-03	1210	6.79E-07	Down	4.97	1519	2.17	0.62

rs17005845	1047	CLGN	4	141683343	6.82E-03	1211	5.08E-02	Up	1.95	6011	2.17	0.13
rs10867123	64975	MRPL41	9	137708155	6.83E-03	1212	3.90E-01	Up	0.86	9918	2.17	0.04
rs10888740	5865	RAB3B	1	52112118	6.84E-03	1213	6.80E-08	Up	5.40	1253	2.17	0.72
rs1079067	8292	COLQ	3	15470930	6.85E-03	1214	1.12E-02	Down	2.54	4587	2.16	0.19
rs441810	54097	FAM3B	21	41620777	6.85E-03	1215	1.06E-06	Up	4.88	1584	2.16	0.60
rs754784	54557	SGTB	5	65004108	6.86E-03	1216	9.44E-06	Down	4.43	1934	2.16	0.50
rs1729674	130872	AHSA2	2	61301388	6.86E-03	1217	7.60E-04	Up	3.37	3113	2.16	0.31
rs6959703	3382	ICA1	7	7918880	6.87E-03	1218	1.32E-22	Up	9.78	151	2.16	2.19
rs9573972	1203	CLN5	13	76467673	6.89E-03	1219	5.37E-07	Down	5.01	1489	2.16	0.63
rs1736450	89796	NAV1	1	198348492	6.90E-03	1220	4.97E-03	Down	2.81	4066	2.16	0.23
rs9608854	5008	OSM	22	28977001	6.91E-03	1221	7.03E-01	Up	0.38	12092	2.16	0.02
rs9608854	3976	LIF	22	28977001	6.91E-03	1222	9.28E-01	Down	0.09	13419	2.16	0.00
rs7302925	4285	MIPEP	12	55147725	6.92E-03	1223	2.18E-17	Up	8.47	293	2.16	1.67
rs7302925	8914	TIMELESS	12	55147725	6.92E-03	1224	7.47E-04	Up	3.37	3108	2.16	0.31
rs7302925	4284	MIP	12	55147725	6.92E-03	1225	5.32E-01	Down	0.62	10957	2.16	0.03
rs7302925	27165	GLS2	12	55147725	6.92E-03	1226	6.87E-01	Up	0.40	11987	2.16	0.02
rs4412975	9951	HS3ST4	16	25926323	6.92E-03	1227	5.34E-02	Up	1.93	6074	2.16	0.13
rs10507089	196475	RMST	12	96401844	6.93E-03	1228	6.42E-03	Down	2.73	4203	2.16	0.22
rs1537590	84918	LRP11	6	150274743	6.93E-03	1229	4.26E-04	Up	3.52	2902	2.16	0.34
rs9847681	205428	C3orf58	3	145187214	6.94E-03	1230	1.36E-02	Down	2.47	4733	2.16	0.19
rs6488494	79370	BCL2L14	12	12121219	6.94E-03	1231	6.37E-01	Up	0.47	11681	2.16	0.02
rs535985	7074	TIAM1	21	31697210	6.94E-03	1232	9.96E-01	Up	0.00	13886	2.16	0.00
rs28909	124912	SPACA3	17	28327481	6.95E-03	1233	4.67E-01	Down	0.73	10479	2.16	0.03
rs17464525	10717	AP4B1	1	114155941	6.95E-03	1234	5.29E-01	Up	0.63	10932	2.16	0.03
rs17464525	64858	DCLRE1B	1	114155941	6.95E-03	1235	5.33E-01	Up	0.62	10961	2.16	0.03
rs3769870	22837	COBLL1	2	165389991	6.95E-03	1236	2.72E-10	Up	6.34	797	2.16	0.96
rs9318115	79866	C13orf34	13	72183522	6.96E-03	1237	1.41E-01	Down	1.47	7583	2.16	0.09
rs1268789	80144	FRAS1	4	79637872	6.96E-03	1238	1.17E-07	Down	5.30	1310	2.16	0.69
rs12599264	5336	PLCG2	16	80397623	6.99E-03	1239	1.62E-01	Down	1.40	7835	2.16	0.08
rs4077238	64407	RGS18	1	188852610	6.99E-03	1240	7.48E-01	Up	0.32	12360	2.16	0.01
rs2696216	3092	HIP1	7	74870740	7.00E-03	1241	3.38E-05	Up	4.15	2193	2.16	0.45
rs12531852	8896	BUD31	7	98654629	7.00E-03	1242	1.69E-03	Up	3.14	3444	2.16	0.28
rs12531852	11333	PDAP1	7	98654629	7.00E-03	1243	4.77E-01	Up	0.71	10554	2.16	0.03
rs12531852	26024	PTCD1	7	98654629	7.00E-03	1244	8.85E-01	Down	0.15	13153	2.16	0.01
rs7480781	55327	LIN7C	11	27483050	7.00E-03	1245	5.73E-01	Down	0.56	11266	2.15	0.02
rs7192855	1009	CDH11	16	63615892	7.03E-03	1246	6.94E-03	Up	2.70	4255	2.15	0.22
rs7631671	83939	EIF2A	3	151775469	7.03E-03	1247	4.04E-05	Up	4.11	2231	2.15	0.44
rs7631671	1965	EIF2S1	3	151775469	7.03E-03	1248	8.36E-05	Down	3.93	2405	2.15	0.41
rs981292	1612	DAPK1	9	87386917	7.04E-03	1249	1.09E-31	Up	11.59	57	2.15	3.10
rs6710147	84168	ANTXR1	2	69254621	7.05E-03	1250	3.25E-23	Down	9.92	137	2.15	2.25
rs10899394	1207	CLNS1A	11	77001909	7.05E-03	1251	3.06E-03	Up	2.96	3782	2.15	0.25
rs10899394	282679	AQP11	11	77001909	7.05E-03	1252	4.34E-02	Up	2.02	5838	2.15	0.14
rs3822360	29102	RNASEN	5	31578423	7.08E-03	1253	2.24E-01	Up	1.21	8502	2.15	0.06
rs3822360	55322	C5orf22	5	31578423	7.08E-03	1254	9.72E-01	Down	0.03	13713	2.15	0.00
rs12030578	84196	USP48	1	21793970	7.10E-03	1255	2.24E-03	Up	3.06	3609	2.15	0.26
rs3745705	57664	PLEKHA4	19	54034081	7.10E-03	1256	2.55E-01	Down	1.14	8814	2.15	0.06
rs12270727	159963	SLC5A12	11	26672194	7.11E-03	1257	3.31E-01	Up	0.97	9451	2.15	0.05
rs7988265	7174	TPP2	13	102139165	7.14E-03	1258	9.22E-02	Down	1.68	6867	2.15	0.10
rs2833610	30811	HUNK	21	32307057	7.14E-03	1259	1.68E-01	Down	1.38	7897	2.15	0.08
rs6015264	9217	VAPB	20	56400932	7.16E-03	1260	2.57E-03	Up	3.02	3702	2.15	0.26
rs2034004	950	SCARB2	4	77501861	7.20E-03	1261	6.80E-06	Up	4.50	1869	2.14	0.52
rs1389790	26009	ZZZ3	1	77801420	7.20E-03	1262	2.12E-01	Up	1.25	8377	2.14	0.07
rs12197079	285848	PNPLA1	6	36382131	7.21E-03	1263	7.93E-01	Down	0.26	12630	2.14	0.01
rs922104	23507	LRRC8B	1	89753920	7.23E-03	1264	3.24E-04	Up	3.60	2804	2.14	0.35
rs739834	22895	RPH3A	12	111722051	7.23E-03	1265	2.07E-01	Down	1.26	8321	2.14	0.07
rs12133663	54996	MOSC2	1	217308478	7.23E-03	1266	4.66E-01	Down	0.73	10472	2.14	0.03
rs12192975	1871	E2F3	6	20539720	7.24E-03	1267	2.42E-10	Up	6.34	796	2.14	0.96
rs1807101	6538	SLC6A11	3	10912056	7.26E-03	1268	7.76E-01	Down	0.28	12533	2.14	0.01
rs8041887	9915	ARNT2	15	78539124	7.26E-03	1269	1.86E-01	Up	1.32	8096	2.14	0.07
rs12426891	120892	LRRK2	12	38947548	7.28E-03	1270	1.20E-05	Down	4.38	1992	2.14	0.49
rs1427283	6672	SP100	2	231108296	7.29E-03	1271	1.20E-03	Down	3.24	3296	2.14	0.29
rs4341630	51084	CRYL1	13	20016721	7.30E-03	1272	1.10E-06	Up	4.87	1588	2.14	0.60
rs1465959	78986	DUSP26	8	33582222	7.30E-03	1273	4.41E-01	Down	0.77	10275	2.14	0.04
rs9851068	7324	UBE2E1	3	23814888	7.31E-03	1274	8.64E-01	Up	0.17	13031	2.14	0.01
rs2292780	27161	EIF2C2	8	141630539	7.31E-03	1275	1.41E-04	Up	3.81	2532	2.14	0.39
rs2281479	994	CDC25B	20	3710095	7.32E-03	1276	1.63E-06	Down	4.79	1642	2.14	0.58
rs2281479	1059	CENPB	20	3710095	7.32E-03	1277	6.57E-03	Down	2.72	4216	2.14	0.22
rs2281479	54976	C20orf27	20	3710095	7.32E-03	1278	2.83E-01	Up	1.07	9055	2.14	0.05
rs7280237	10311	DSCR3	21	37578409	7.33E-03	1279	1.87E-01	Down	1.32	8108	2.13	0.07
rs1212146	9379	NRXN2	11	64169453	7.34E-03	1280	2.17E-01	Down	1.23	8434	2.13	0.07
rs11526176	11112	HIBADH	7	27353013	7.34E-03	1281	9.76E-01	Down	0.03	13735	2.13	0.00
rs13404220	23677	SH3BP4	2	235753971	7.34E-03	1282	5.65E-03	Up	2.77	4146	2.13	0.22
rs9474794	27283	TINAG	6	54275213	7.36E-03	1283	6.40E-01	Up	0.47	11711	2.13	0.02
rs10841085	89869	PLCZ1	12	18801189	7.37E-03	1284	1.98E-01	Up	1.29	8239	2.13	0.07
rs10193444	5495	PPM1B	2	44304253	7.38E-03	1285	1.47E-02	Down	2.44	4795	2.13	0.18
rs6059193	51297	PLUNC	20	31299058	7.39E-03	1286	9.57E-02	Down	1.67	6916	2.13	0.10
rs9958503	2235	FECH	18	53362220	7.40E-03	1287	2.06E-03	Up	3.08	3552	2.13	0.27
rs834082	9162	DGKI	7	136673769	7.41E-03	1288	6.05E-01	Down	0.52	11470	2.13	0.02
rs11902010	7260	TSSC1	2	4864700	7.44E-03	1289	1.21E-01	Up	1.55	7306	2.13	0.09
rs1641107	18	ABAT	16	8680078	7.45E-03	1290	6.20E-08	Up	5.42	1242	2.13	0.72
rs12029086	10806	SDCCAG8	1	239890763	7.45E-03	1291	2.66E-01	Down	1.11	8915	2.13	0.06

rs4611808	9730	VPRBP	3	51444147	7.47E-03	1292	1.79E-01	Up	1.34	8012	2.13	0.07
rs10846667	9612	NCOR2	12	123409304	7.47E-03	1293	7.70E-01	Down	0.29	12499	2.13	0.01
rs1223954	64927	TTC23	15	97584594	7.48E-03	1294	3.65E-02	Down	2.09	5664	2.13	0.14
rs11020303	159989	CCDC67	11	92769541	7.49E-03	1295	6.71E-02	Down	1.83	6379	2.13	0.12
rs949664	5801	PTPRR	12	69368222	7.51E-03	1296	1.16E-01	Up	1.57	7230	2.12	0.09
rs6062177	9885	OSBPL2	20	60276328	7.52E-03	1297	1.08E-03	Down	3.27	3251	2.12	0.30
rs12881869	11183	MAP4K5	14	49992999	7.52E-03	1298	4.54E-03	Down	2.84	4001	2.12	0.23
rs5996274	638	BIK	22	41822612	7.52E-03	1299	4.59E-13	Up	7.24	524	2.12	1.23
rs5996274	25809	TTLL1	22	41822612	7.52E-03	1300	3.15E-01	Up	1.01	9316	2.12	0.05
rs7009521	6867	TACC1	8	38820486	7.53E-03	1301	8.43E-34	Down	12.12	45	2.12	3.31
rs1570009	3755	KCNG1	20	49078471	7.53E-03	1302	2.70E-01	Down	1.10	8959	2.12	0.06
rs11682000	94097	SFXN5	2	73078240	7.53E-03	1303	5.90E-01	Down	0.54	11370	2.12	0.02
rs11682000	2016	EMX1	2	73078240	7.53E-03	1304	9.69E-01	Up	0.04	13691	2.12	0.00
rs12751502	832	CAPZB	1	19425838	7.54E-03	1305	5.12E-18	Down	8.65	269	2.12	1.73
rs10152363	54989	ZNF770	15	33048562	7.54E-03	1306	1.05E-01	Up	1.62	7088	2.12	0.10
rs10152363	9716	AQR	15	33048562	7.54E-03	1307	6.35E-01	Down	0.48	11670	2.12	0.02
rs12091371	56776	FMN2	1	236931093	7.54E-03	1308	2.57E-01	Down	1.13	8831	2.12	0.06
rs6784599	8975	USP13	3	180904206	7.55E-03	1309	5.43E-03	Up	2.78	4119	2.12	0.23
rs3755768	4241	MFI2	3	198247134	7.55E-03	1310	1.05E-01	Up	1.62	7076	2.12	0.10
rs3863298	54494	C11orf71	11	113780966	7.56E-03	1311	2.07E-02	Up	2.31	5099	2.12	0.17
rs3863298	10179	RBM7	11	113780966	7.56E-03	1312	1.08E-01	Down	1.61	7124	2.12	0.10
rs2008854	246269	LACE1	6	108719760	7.57E-03	1313	1.40E-01	Up	1.48	7573	2.12	0.09
rs2232176	50487	PLA2G3	22	29860687	7.57E-03	1314	2.51E-01	Down	1.15	8768	2.12	0.06
rs1552074	7873	ARMET	3	51383091	7.57E-03	1315	3.99E-18	Up	8.66	268	2.12	1.74
rs8095338	284252	KCTD1	18	22352034	7.59E-03	1316	1.30E-01	Down	1.51	7453	2.12	0.09
rs2738173	1672	DEFB1	8	6728348	7.59E-03	1317	1.82E-06	Down	4.77	1654	2.12	0.57
rs2296138	84893	FBXO18	10	5993154	7.59E-03	1318	3.06E-02	Down	2.16	5483	2.12	0.15
rs4073505	2220	FCN2	9	134987049	7.59E-03	1319	1.21E-01	Up	1.55	7313	2.12	0.09
rs4251719	79148	MMP28	17	31166475	7.60E-03	1320	2.09E-01	Down	1.26	8345	2.12	0.07
rs8033222	145781	GCOM1	15	55651647	7.62E-03	1321	2.44E-04	Down	3.67	2699	2.12	0.36
rs13256087	51305	KCNK9	8	140719986	7.62E-03	1322	5.47E-01	Down	0.60	11060	2.12	0.03
rs6889761	6793	STK10	5	171446104	7.63E-03	1323	2.91E-02	Down	2.18	5431	2.12	0.15
rs4687569	29890	RBM15B	3	51407935	7.63E-03	1324	1.46E-01	Up	1.45	7639	2.12	0.08
rs10169375	151473	SLC16A14	2	230771498	7.64E-03	1325	5.49E-03	Down	2.78	4126	2.12	0.23
rs6656763	54596	L1TD1	1	62373813	7.67E-03	1326	6.01E-01	Up	0.52	11436	2.12	0.02
rs1713222	338	APOB	2	21182975	7.69E-03	1327	6.34E-01	Up	0.48	11669	2.11	0.02
rs7792024	5137	PDE1C	7	31759963	7.69E-03	1328	6.79E-02	Down	1.83	6392	2.11	0.12
rs10813835	4712	NDUFB6	9	32549114	7.73E-03	1329	1.05E-01	Up	1.62	7072	2.11	0.10
rs10813835	10210	TOPORS	9	32549114	7.73E-03	1330	3.87E-01	Down	0.87	9891	2.11	0.04
rs9651726	220001	VWCE	11	60825934	7.74E-03	1331	4.78E-03	Down	2.82	4030	2.11	0.23
rs9651726	1642	DDB1	11	60825934	7.74E-03	1332	2.65E-01	Down	1.11	8911	2.11	0.06
rs13218313	5570	PKIB	6	123071368	7.75E-03	1333	1.49E-14	Up	7.69	420	2.11	1.38
rs8107173	811	CALR	19	12896638	7.75E-03	1334	1.06E-06	Up	4.88	1582	2.11	0.60
rs1589615	5968	REG1B	2	79283280	7.76E-03	1335	5.73E-01	Up	0.56	11262	2.11	0.02
rs2524366	30817	EMR2	19	14701575	7.76E-03	1336	6.26E-01	Down	0.49	11623	2.11	0.02
rs2524366	84449	ZNF333	19	14701575	7.76E-03	1337	7.28E-01	Down	0.35	12237	2.11	0.01
rs11619599	222484	LNX2	13	27024745	7.77E-03	1338	4.81E-01	Down	0.70	10582	2.11	0.03
rs4255978	84417	C2orf40	2	106136741	7.78E-03	1339	1.33E-07	Down	5.27	1324	2.11	0.69
rs3793071	1297	COL9A1	6	71015067	7.80E-03	1340	9.18E-07	Down	4.91	1566	2.11	0.60
rs17630660	256764	WDR72	15	51597250	7.80E-03	1341	1.46E-01	Up	1.46	7633	2.11	0.08
rs6494120	9245	GCNT3	15	57681909	7.81E-03	1342	1.55E-01	Down	1.42	7746	2.11	0.08
rs11591580	84435	GPR123	10	134761050	7.81E-03	1343	4.36E-01	Down	0.78	10241	2.11	0.04
rs6910129	60468	BACH2	6	90740308	7.83E-03	1344	3.08E-04	Down	3.61	2783	2.11	0.35
rs272508	57489	ODF2L	1	86551406	7.84E-03	1345	2.55E-01	Down	1.14	8804	2.11	0.06
rs12525721	387357	C6orf190	6	128265628	7.84E-03	1346	9.59E-01	Down	0.05	13615	2.11	0.00
rs17122926	9056	SLC7A7	14	22369755	7.85E-03	1347	2.31E-01	Up	1.20	8578	2.11	0.06
rs931838	287	ANK2	4	114331556	7.86E-03	1348	2.86E-03	Down	2.98	3757	2.10	0.25
rs2043172	54437	SEMA5B	3	124145445	7.87E-03	1349	7.97E-01	Down	0.26	12657	2.10	0.01
rs8083841	57045	TWSG1	18	9399461	7.88E-03	1350	3.10E-03	Down	2.96	3793	2.10	0.25
rs11579176	4678	NASP	1	45719654	7.89E-03	1351	8.30E-08	Down	5.36	1276	2.10	0.71
rs6791556	285195	SLC9A9	3	144746881	7.90E-03	1352	9.76E-01	Down	0.03	13740	2.10	0.00
rs687623	161582	DYX1C1	15	53518473	7.90E-03	1353	1.56E-02	Up	2.42	4849	2.10	0.18
rs1743720	9786	KIAA0586	14	57995479	7.90E-03	1354	8.08E-01	Down	0.24	12715	2.10	0.01
rs17569702	9378	NRXN1	2	50971487	7.91E-03	1355	2.17E-03	Down	3.07	3589	2.10	0.27
rs999986	79789	CLMN	14	94873609	7.91E-03	1356	1.49E-01	Down	1.44	7661	2.10	0.08
rs6475441	4300	MLLT3	9	20475603	7.92E-03	1357	4.28E-03	Up	2.86	3978	2.10	0.24
rs12447769	93107	KCNG4	16	82844645	7.92E-03	1358	3.62E-01	Down	0.91	9692	2.10	0.04
rs3846211	51726	DNAJB11	3	187789243	7.93E-03	1359	1.24E-01	Up	1.54	7365	2.10	0.09
rs573264	7704	ZBTB16	11	113597792	7.93E-03	1360	7.55E-02	Up	1.78	6536	2.10	0.11
rs10926008	1131	CHRM3	1	236388164	7.94E-03	1361	8.42E-10	Up	6.13	881	2.10	0.91
rs2580815	4880	NPPC	2	232623200	7.94E-03	1362	1.88E-01	Down	1.32	8118	2.10	0.07
rs11770876	136227	EMID2	7	100635543	7.96E-03	1363	5.42E-01	Down	0.61	11035	2.10	0.03
rs3764421	51379	CRLF3	17	26191779	7.96E-03	1364	5.41E-05	Up	4.04	2306	2.10	0.43
rs3764421	79915	ATAD5	17	26191779	7.96E-03	1365	4.86E-01	Up	0.70	10623	2.10	0.03
rs5951547	158506	ZNF645		22052456	7.96E-03	1366	6.63E-01	Down	0.44	11843	2.10	0.02
rs870549	163702	IL28RA	1	24271302	7.99E-03	1367	4.39E-01	Down	0.77	10265	2.10	0.04
rs11894892	57498	KIDINS220	2	8931618	7.99E-03	1368	5.96E-01	Down	0.53	11410	2.10	0.02
rs11947923	152579	SCFD2	4	53752265	8.00E-03	1369	8.37E-01	Up	0.21	12887	2.10	0.01
rs1865806	5373	PMM2	16	8790153	8.01E-03	1370	2.13E-11	Up	6.73	663	2.10	1.07
rs1865806	25880	TMEM186	16	8790153	8.01E-03	1371	8.01E-02	Up	1.75	6632	2.10	0.11
rs9353525	1268	CNR1	6	88900096	8.02E-03	1372	1.15E-01	Down	1.57	7220	2.10	0.09

rs171801	5144	PDE4D	5	58747585	8.03E-03	1373	6.48E-15	Down	7.79	396	2.10	1.42
rs2278133	4308	TRPM1	15	29140680	8.04E-03	1374	5.35E-02	Down	1.93	6075	2.09	0.13
rs11745636	55521	TRIM36	5	114526046	8.05E-03	1375	7.81E-06	Up	4.47	1896	2.09	0.51
rs12493471	1238	CCBP2	3	45926682	8.05E-03	1376	3.76E-03	Down	2.90	3895	2.09	0.24
rs12493471	79443	FYCO1	3	45926682	8.05E-03	1377	9.78E-02	Down	1.66	6959	2.09	0.10
rs12493471	10803	CCR9	3	45926682	8.05E-03	1378	6.69E-01	Down	0.43	11879	2.09	0.02
rs3131274	6594	SMARCA1		128383877	8.05E-03	1379	2.18E-07	Up	5.18	1384	2.09	0.67
rs3131274	4952	OCRL		128383877	8.05E-03	1380	3.33E-05	Up	4.15	2188	2.09	0.45
rs4770101	26524	LATS2	13	20549036	8.07E-03	1381	5.77E-01	Down	0.56	11289	2.09	0.02
rs4233979	200728	TMEM17	2	62639553	8.07E-03	1382	7.45E-01	Down	0.33	12346	2.09	0.01
rs9600179	11278	KLF12	13	73338001	8.07E-03	1383	4.48E-02	Up	2.01	5870	2.09	0.13
rs5918486	8406	SRPX		37773488	8.08E-03	1384	2.04E-19	Down	9.01	217	2.09	1.87
rs11672416	6633	SNRPD2	19	50898265	8.10E-03	1385	7.07E-15	Up	7.79	397	2.09	1.42
rs11672416	23403	FBXO46	19	50898265	8.10E-03	1386	7.13E-05	Up	3.97	2366	2.09	0.41
rs11672416	54814	QPCTL	19	50898265	8.10E-03	1387	2.14E-01	Down	1.24	8403	2.09	0.07
rs4076588	201266	SLC39A11	17	68198036	8.13E-03	1388	9.29E-01	Down	0.09	13424	2.09	0.00
rs1861589	121551	BTBD11	12	106548685	8.13E-03	1389	2.17E-04	Up	3.70	2655	2.09	0.37
rs12198813	221749	C6orf145	6	3702740	8.13E-03	1390	4.66E-04	Down	3.50	2933	2.09	0.33
rs6062340	6919	TCEA2	20	62153719	8.14E-03	1391	1.09E-02	Down	2.55	4566	2.09	0.20
rs6062340	54345	SOX18	20	62153719	8.14E-03	1392	1.56E-01	Up	1.42	7755	2.09	0.08
rs6076013	7056	THBD	20	22969597	8.14E-03	1393	9.86E-03	Down	2.58	4497	2.09	0.20
rs6076013	6754	SSTR4	20	22969597	8.14E-03	1394	1.80E-01	Up	1.34	8025	2.09	0.07
rs1983590	11141	IL1RAPL1		29159124	8.15E-03	1395	1.42E-01	Up	1.47	7598	2.09	0.08
rs3826777	22809	ATF5	19	55123200	8.18E-03	1396	5.27E-09	Up	5.89	989	2.09	0.83
rs3826777	23636	NUP62	19	55123200	8.18E-03	1397	2.13E-02	Up	2.30	5135	2.09	0.17
rs9866959	2042	EPHA3	3	89462625	8.18E-03	1398	4.78E-01	Down	0.71	10557	2.09	0.03
rs1109374	50808	AK3	1	65345518	8.21E-03	1399	7.91E-01	Up	0.26	12619	2.09	0.01
rs6674176	9048	ARTN	1	44049050	8.22E-03	1400	1.72E-02	Down	2.38	4947	2.09	0.18
rs16898497	114907	FBXO32	8	124599554	8.22E-03	1401	7.26E-13	Down	7.17	542	2.09	1.21
rs11636576	54822	TRPM7	15	48734053	8.23E-03	1402	2.99E-01	Up	1.04	9183	2.08	0.05
rs7946884	80310	PDGFD	11	103544199	8.23E-03	1403	6.52E-09	Down	5.80	1028	2.08	0.82
rs847693	5164	PDK2	17	45539376	8.24E-03	1404	3.23E-05	Down	4.16	2182	2.08	0.45
rs12944679	9256	BZRAP1	17	53727279	8.25E-03	1405	5.73E-02	Down	1.90	6168	2.08	0.12
rs12944679	4353	MPO	17	53727279	8.25E-03	1406	2.48E-01	Up	1.15	8751	2.08	0.06
rs1538844	23081	JMJD2C	9	6953799	8.28E-03	1407	1.91E-02	Up	2.34	5024	2.08	0.17
rs2067043	3767	KCNJ11	11	17381287	8.28E-03	1408	4.60E-03	Up	2.83	4009	2.08	0.23
rs2067043	6833	ABCC8	11	17381287	8.28E-03	1409	6.34E-02	Up	1.86	6308	2.08	0.12
rs4650047	23584	VSIG2	1	70579311	8.28E-03	1410	6.25E-10	Down	6.18	866	2.08	0.92
rs4650047	1491	CTH	1	70579311	8.28E-03	1411	7.43E-02	Up	1.78	6513	2.08	0.11
rs2433	79736	C17orf42	17	26250354	8.30E-03	1412	3.11E-01	Up	1.01	9276	2.08	0.05
rs2242566	8519	IFITM1	11	285876	8.31E-03	1413	2.99E-19	Down	8.97	221	2.08	1.85
rs2242566	10581	IFITM2	11	285876	8.31E-03	1414	1.20E-18	Down	8.81	243	2.08	1.79
rs2242566	80162	ATHL1	11	285876	8.31E-03	1415	1.56E-01	Down	1.42	7750	2.08	0.08
rs9296672	54438	GFOD1	6	13533511	8.31E-03	1416	1.61E-02	Down	2.41	4894	2.08	0.18
rs11765530	54443	ANLN	7	36253288	8.35E-03	1417	1.04E-04	Up	3.88	2464	2.08	0.40
rs10275665	26211	OR2F1	7	143075623	8.35E-03	1418	3.13E-01	Up	1.01	9297	2.08	0.05
rs2839188	8888	MCM3AP	21	46517226	8.36E-03	1419	3.59E-04	Up	3.57	2845	2.08	0.34
rs4706515	56479	KCNQ5	6	73635136	8.37E-03	1420	8.92E-01	Down	0.14	13195	2.08	0.00
rs3824388	6456	SH3GL2	9	17614902	8.38E-03	1421	4.42E-01	Down	0.77	10282	2.08	0.04
rs17323670	4163	MCC	5	112608189	8.39E-03	1422	6.46E-12	Down	6.87	629	2.08	1.12
rs2182909	1378	CR1	1	204041501	8.39E-03	1423	1.57E-01	Down	1.42	7763	2.08	0.08
rs11210273	1907	EDN2	1	41616837	8.39E-03	1424	5.48E-01	Up	0.60	11066	2.08	0.03
rs1045753	80727	TTYH3	7	2425871	8.39E-03	1425	3.57E-10	Up	6.25	839	2.08	0.94
rs17112245	26148	C10orf12	10	98718911	8.40E-03	1426	1.44E-03	Up	3.19	3360	2.08	0.28
rs2847286	5771	PTPN2	18	12807815	8.41E-03	1427	6.62E-04	Up	3.40	3060	2.08	0.32
rs5977184	63035	BCORL1		128825957	8.41E-03	1428	3.62E-02	Down	2.09	5656	2.08	0.14
rs647077	3613	IMPA2	18	11974196	8.42E-03	1429	3.53E-06	Up	4.64	1763	2.07	0.55
rs3745180	55786	ZNF415	19	58311203	8.42E-03	1430	5.46E-02	Down	1.92	6104	2.07	0.13
rs3745180	90338	ZNF160	19	58311203	8.42E-03	1431	2.58E-01	Down	1.13	8847	2.07	0.06
rs2119187	131566	DCBLD2	3	100077189	8.42E-03	1432	6.97E-04	Down	3.39	3074	2.07	0.32
rs3819197	124	ADH1A	4	100557687	8.42E-03	1433	1.85E-01	Down	1.32	8091	2.07	0.07
rs2420543	5585	PKN1	19	14387036	8.43E-03	1434	1.36E-06	Up	4.83	1613	2.07	0.59
rs2420543	10212	DDX39	19	14387036	8.43E-03	1435	1.21E-02	Up	2.51	4640	2.07	0.19
rs2420543	976	CD97	19	14387036	8.43E-03	1436	5.27E-01	Down	0.63	10918	2.07	0.03
rs7791537	50617	ATP6V0A4	7	137924395	8.44E-03	1437	2.01E-02	Down	2.32	5079	2.07	0.17
rs17056770	7223	TRPC4	13	37350095	8.44E-03	1438	5.66E-03	Down	2.77	4147	2.07	0.22
rs27076	140890	SFRS12	5	65485952	8.44E-03	1439	4.60E-01	Up	0.74	10427	2.07	0.03
rs11780669	4481	MSR1	8	16018641	8.45E-03	1440	2.22E-01	Up	1.22	8481	2.07	0.07
rs11647246	79724	ZNF768	16	30435633	8.45E-03	1441	2.93E-01	Down	1.05	9136	2.07	0.05
rs6498011	3566	IL4R	16	27239395	8.45E-03	1442	4.55E-08	Down	5.47	1213	2.07	0.73
rs926198	857	CAV1	7	115761159	8.46E-03	1443	5.48E-33	Down	11.96	49	2.07	3.23
rs4656729	22920	KIFAP3	1	166703150	8.46E-03	1444	7.30E-08	Down	5.38	1262	2.07	0.71
rs2288709	130271	PLEKHH2	2	43915661	8.48E-03	1445	1.25E-04	Down	3.84	2501	2.07	0.39
rs2288709	51626	DYNC2LI1	2	43915661	8.48E-03	1446	2.12E-01	Up	1.25	8385	2.07	0.07
rs9864293	3556	IL1RAP	3	191695408	8.49E-03	1447	3.89E-02	Down	2.07	5726	2.07	0.14
rs6461670	54543	TOMM7	7	22642224	8.49E-03	1448	1.75E-01	Up	1.36	7967	2.07	0.08
rs868907	528	ATP6V1C1	8	104091445	8.51E-03	1449	9.94E-05	Up	3.89	2451	2.07	0.40
rs11823971	5138	PDE2A	11	72066209	8.51E-03	1450	1.43E-01	Down	1.46	7611	2.07	0.08
rs3865451	79934	ADCK4	19	45897379	8.52E-03	1451	1.76E-01	Down	1.35	7976	2.07	0.08
rs3865451	9253	NUMBL	19	45897379	8.52E-03	1452	3.02E-01	Down	1.03	9207	2.07	0.05
rs3865451	80271	ITPKC	19	45897379	8.52E-03	1453	9.49E-01	Up	0.06	13543	2.07	0.00

rs2422975	9770	RASSF2	20	4710869	8.52E-03	1454	7.69E-01	Down	0.29	12487	2.07	0.01
rs7951109	154810	AMOTL1	11	94165985	8.53E-03	1455	1.30E-07	Down	5.28	1320	2.07	0.69
rs1953241	23224	SYNE2	14	63444007	8.53E-03	1456	3.15E-01	Up	1.00	9322	2.07	0.05
rs10757193	3467	IFNW1	9	21147373	8.54E-03	1457	6.84E-01	Down	0.41	11970	2.07	0.02
rs16861	5858	PZP	12	9250511	8.54E-03	1458	9.45E-01	Up	0.07	13526	2.07	0.00
rs2490385	6262	RYR2	1	233609012	8.56E-03	1459	4.40E-01	Up	0.77	10268	2.07	0.04
rs12398674	79135	APOO		23639499	8.57E-03	1460	2.88E-03	Up	2.98	3758	2.07	0.25
rs10263398	6604	SMARCD3	7	150414875	8.58E-03	1461	3.16E-23	Down	9.93	136	2.07	2.25
rs1789888	125	ADH1B	4	100604748	8.58E-03	1462	1.27E-06	Down	4.85	1607	2.07	0.59
rs8073426	8313	AXIN2	17	60993743	8.61E-03	1463	1.24E-01	Down	1.54	7364	2.07	0.09
rs2468221	80184	CEP290	12	87034885	8.61E-03	1464	3.48E-03	Up	2.92	3856	2.06	0.25
rs836860	10954	PDIA5	3	124290685	8.62E-03	1465	1.19E-20	Up	9.31	184	2.06	1.99
rs836860	64714	PDIA2	3	124290685	8.62E-03	1466	8.14E-01	Up	0.24	12748	2.06	0.01
rs11786308	2137	EXTL3	8	28677088	8.62E-03	1467	3.01E-02	Down	2.17	5468	2.06	0.15
rs11786308	55756	INTS9	8	28677088	8.62E-03	1468	7.35E-01	Down	0.34	12284	2.06	0.01
rs12525249	257218	SHPRH	6	146300431	8.63E-03	1469	4.06E-04	Up	3.54	2890	2.06	0.34
rs1148556	10540	DCTN2	12	56203792	8.63E-03	1470	4.45E-02	Down	2.01	5864	2.06	0.14
rs1148556	114785	MBD6	12	56203792	8.63E-03	1471	1.20E-01	Up	1.56	7289	2.06	0.09
rs1148556	1649	DDIT3	12	56203792	8.63E-03	1472	1.25E-01	Up	1.53	7374	2.06	0.09
rs1148556	4141	MARS	12	56203792	8.63E-03	1473	5.54E-01	Up	0.59	11105	2.06	0.03
rs7977818	5318	PKP2	12	32953290	8.63E-03	1474	5.17E-03	Down	2.80	4086	2.06	0.23
rs7107058	56946	C11orf30	11	75845867	8.64E-03	1475	1.68E-01	Up	1.38	7894	2.06	0.08
rs2581651	284273	ZADH2	18	71055030	8.65E-03	1476	5.20E-02	Up	1.94	6038	2.06	0.13
rs4978774	54566	EPB41L4B	9	109004870	8.66E-03	1477	3.82E-10	Up	6.26	829	2.06	0.94
rs4978774	23732	C9orf4	9	109004870	8.66E-03	1478	2.99E-01	Down	1.04	9186	2.06	0.05
rs7096662	54518	APBB1IP	10	26841727	8.67E-03	1479	4.96E-02	Down	1.96	5988	2.06	0.13
rs1923541	8512	MBL1P1	10	81680275	8.67E-03	1480	8.24E-01	Up	0.22	12815	2.06	0.01
rs12304122	23504	RIMBP2	12	129427980	8.68E-03	1481	9.93E-01	Up	0.01	13865	2.06	0.00
rs10744383	92293	TMEM132C	12	127620485	8.71E-03	1482	1.05E-04	Down	3.88	2468	2.06	0.40
rs1414622	266722	HS6ST3	13	96006899	8.72E-03	1483	9.39E-01	Up	0.08	13487	2.06	0.00
rs714827	23186	RCOR1	14	102183305	8.72E-03	1484	5.08E-01	Up	0.66	10769	2.06	0.03
rs1170189	160851	DGKH	13	41574804	8.73E-03	1485	3.54E-04	Down	3.57	2839	2.06	0.35
rs7710366	3781	KCNN2	5	113808086	8.73E-03	1486	2.59E-04	Up	3.65	2719	2.06	0.36
rs1122729	51348	KLRF1	12	9859355	8.74E-03	1487	4.64E-01	Up	0.73	10452	2.06	0.03
rs615568	64949	MRPS26	20	2956775	8.74E-03	1488	2.10E-02	Up	2.31	5116	2.06	0.17
rs615568	2797	GNRH2	20	2956775	8.74E-03	1489	5.12E-01	Down	0.66	10791	2.06	0.03
rs2377024	284217	LAMA1	18	7076310	8.76E-03	1490	1.33E-01	Up	1.50	7480	2.06	0.09
rs10112728	57094	CPA6	8	68736913	8.77E-03	1491	1.49E-03	Down	3.18	3380	2.06	0.28
rs10112728	1548	CYP2A6	8	68736913	8.77E-03	1492	4.75E-01	Down	0.71	10537	2.06	0.03
rs562	10057	ABCC5	3	185120547	8.78E-03	1493	2.92E-02	Up	2.18	5434	2.06	0.15
rs11786992	54845	RBM35A	8	95754323	8.78E-03	1494	3.55E-17	Up	8.43	299	2.06	1.64
rs1527808	139411	PTCHD1		23099721	8.78E-03	1495	1.80E-01	Down	1.34	8031	2.06	0.07
rs7545292	7750	ZMYM2	1	208667453	8.81E-03	1496	2.65E-11	Up	6.67	682	2.06	1.06
rs7545292	51514	DTL	1	208667453	8.81E-03	1497	1.39E-01	Up	1.48	7565	2.06	0.09
rs506597	10248	POP7	7	99958071	8.83E-03	1498	3.46E-06	Up	4.64	1760	2.05	0.55
rs506597	2056	EPO	7	99958071	8.83E-03	1499	1.62E-01	Down	1.40	7836	2.05	0.08
rs506597	7076	TIMP1	7	99958071	8.83E-03	1500	3.41E-01	Down	0.95	9530	2.05	0.05
rs506597	7455	ZAN	7	99958071	8.83E-03	1501	4.86E-01	Down	0.70	10621	2.05	0.03
rs506597	8288	EPX	7	99958071	8.83E-03	1502	6.59E-01	Down	0.44	11821	2.05	0.02
rs4346062	57522	SRGAP1	12	62570550	8.85E-03	1503	1.21E-04	Up	3.84	2494	2.05	0.39
rs2469206	5955	RCN2	15	75034323	8.86E-03	1504	5.40E-08	Up	5.44	1232	2.05	0.73
rs12415087	55130	ARMC4	10	28311289	8.89E-03	1505	3.26E-01	Down	0.98	9410	2.05	0.05
rs8066276	81033	KCNH6	17	58942997	8.90E-03	1506	1.72E-01	Up	1.37	7937	2.05	0.08
rs8066276	1636	ACE	17	58942997	8.90E-03	1507	3.05E-01	Down	1.03	9229	2.05	0.05
rs3017438	54101	RIPK4	21	42073301	8.90E-03	1508	1.04E-01	Down	1.63	7063	2.05	0.10
rs12152912	64839	FBXL17	5	107363023	8.91E-03	1509	1.59E-01	Down	1.41	7794	2.05	0.08
rs605383	23586	DDX58	9	32482903	8.92E-03	1510	1.89E-02	Up	2.35	5019	2.05	0.17
rs12648093	11162	NUDT6	4	124196363	8.94E-03	1511	1.42E-01	Up	1.47	7601	2.05	0.08
rs12648093	166378	SPATA5	4	124196363	8.94E-03	1512	5.75E-01	Up	0.56	11279	2.05	0.02
rs884127	55532	SLC30A10	1	216470545	8.95E-03	1513	7.83E-02	Down	1.76	6602	2.05	0.11
rs4531288	59269	HIVEP3	1	41930170	8.97E-03	1514	8.93E-01	Down	0.14	13198	2.05	0.00
rs2564114	5194	PEX13	2	61142298	8.98E-03	1515	2.34E-02	Up	2.27	5220	2.05	0.16
rs11793993	8777	MPDZ	9	13255893	9.02E-03	1516	1.13E-02	Down	2.53	4599	2.05	0.19
rs11793993	84708	LNX1	9	13255893	9.02E-03	1517	1.12E-01	Up	1.59	7174	2.05	0.10
rs4820972	4733	DRG1	22	30160932	9.02E-03	1518	1.25E-02	Down	2.50	4661	2.04	0.19
rs4820972	10397	NDRG1	22	30160932	9.02E-03	1519	4.14E-02	Up	2.04	5784	2.04	0.14
rs8137776	84271	POLDIP3	22	41309081	9.02E-03	1520	4.78E-02	Up	1.98	5947	2.04	0.13
rs4820972	56478	EIF4ENIF1	22	30160932	9.02E-03	1521	6.60E-01	Down	0.44	11832	2.04	0.02
rs2295348	55317	C20orf29	20	3733034	9.02E-03	1522	2.97E-01	Up	1.04	9175	2.04	0.05
rs3138353	8930	MBD4	3	130635282	9.02E-03	1523	8.25E-01	Up	0.22	12821	2.04	0.01
rs10492251	23479	ISCU	12	107467548	9.02E-03	1524	7.76E-18	Down	8.60	275	2.04	1.71
rs10492251	9733	SART3	12	107467548	9.02E-03	1525	2.36E-02	Up	2.26	5226	2.04	0.16
rs11929107	5357	PLS1	3	143893898	9.03E-03	1526	6.10E-03	Up	2.74	4177	2.04	0.22
rs12981699	10224	ZNF443	19	12391752	9.04E-03	1527	9.65E-09	Up	5.73	1057	2.04	0.80
rs559987	148641	SLC35F3	1	230740855	9.04E-03	1528	2.04E-01	Up	1.27	8292	2.04	0.07
rs7582260	23040	MYT1L	2	2214685	9.05E-03	1529	5.73E-02	Up	1.90	6169	2.04	0.12
rs3814258	8451	CUL4A	13	112963486	9.06E-03	1530	5.41E-01	Down	0.61	11024	2.04	0.03
rs13071640	165721	DNAJB8	3	129665076	9.07E-03	1531	3.99E-01	Down	0.84	9977	2.04	0.04
rs6073285	5933	RBL1	20	35151129	9.09E-03	1532	9.39E-01	Down	0.08	13489	2.04	0.00
rs11538677	26960	NBEA	13	35100269	9.09E-03	1533	3.50E-01	Down	0.94	9583	2.04	0.05
rs9845467	2632	GBE1	3	81697163	9.10E-03	1534	2.03E-01	Down	1.27	8285	2.04	0.07

rs2068381	84251	SGIP1	1	66724209	9.11E-03	1535	9.53E-01	Up	0.06	13575	2.04	0.00
rs205965	222553	SLC35F1	6	118585200	9.12E-03	1536	2.70E-01	Down	1.10	8958	2.04	0.06
rs9297985	90362	FAM110B	8	59215846	9.13E-03	1537	4.67E-02	Up	1.99	5918	2.04	0.13
rs2153208	8407	TAGLN2	1	156710400	9.13E-03	1538	5.31E-07	Down	5.01	1486	2.04	0.63
rs2153208	57549	IGSF9	1	156710400	9.13E-03	1539	6.13E-01	Down	0.51	11529	2.04	0.02
rs3825885	4916	NTRK3	15	86403845	9.13E-03	1540	6.14E-01	Down	0.50	11532	2.04	0.02
rs11174456	9958	USP15	12	61070234	9.14E-03	1541	4.61E-01	Up	0.74	10432	2.04	0.03
rs1426926	23682	RAB38	11	87542789	9.15E-03	1542	1.30E-03	Down	3.22	3324	2.04	0.29
rs3826322	94015	TTYH2	17	69710256	9.15E-03	1543	1.91E-04	Down	3.73	2614	2.04	0.37
rs3826322	6169	RPL38	17	69710256	9.15E-03	1544	1.68E-02	Up	2.39	4924	2.04	0.18
rs1191246	63895	FAM38B	18	10673819	9.16E-03	1545	1.10E-02	Down	2.54	4577	2.04	0.20
rs10244884	358	AQP1	7	30745035	9.18E-03	1546	3.74E-04	Down	3.56	2857	2.04	0.34
rs2277547	11173	ADAMTS7	15	76869486	9.18E-03	1547	5.39E-01	Down	0.61	11006	2.04	0.03
rs13257046	54928	IMPAD1	8	58064942	9.19E-03	1548	3.01E-01	Down	1.04	9195	2.04	0.05
rs730993	55553	SOX6	11	15941840	9.21E-03	1549	7.50E-01	Down	0.32	12378	2.04	0.01
rs26866	57524	CASKIN1	16	2190732	9.22E-03	1550	8.91E-01	Down	0.14	13192	2.04	0.01
rs26866	283870	C16orf79	16	2190732	9.22E-03	1551	9.57E-01	Up	0.05	13594	2.04	0.00
rs10092214	55636	CHD7	8	61889433	9.24E-03	1552	8.21E-02	Down	1.74	6669	2.03	0.11
rs7100441	6001	RGS10	10	121256597	9.24E-03	1553	7.75E-36	Up	12.43	37	2.03	3.51
rs3761392	755	C21orf2	21	44576604	9.26E-03	1554	1.53E-02	Down	2.42	4829	2.03	0.18
rs7896064	55691	FRMD4A	10	13823583	9.26E-03	1555	2.09E-03	Up	3.08	3560	2.03	0.27
rs13248839	59339	PLEKHA2	8	38867769	9.27E-03	1556	1.75E-13	Down	7.37	488	2.03	1.28
rs9285484	4217	MAP3K5	6	137092247	9.28E-03	1557	2.51E-01	Down	1.15	8772	2.03	0.06
rs3765524	51196	PLCE1	10	96048288	9.29E-03	1558	8.69E-02	Down	1.71	6754	2.03	0.11
rs4764409	5288	PIK3C2G	12	18675519	9.29E-03	1559	4.82E-02	Down	1.98	5956	2.03	0.13
rs624561	5936	RBM4	11	66171491	9.30E-03	1560	5.18E-07	Up	5.02	1484	2.03	0.63
rs4862396	836	CASP3	4	185918821	9.31E-03	1561	4.73E-01	Up	0.72	10519	2.03	0.03
rs9611302	23112	TNRC6B	22	38974784	9.32E-03	1562	1.54E-02	Up	2.42	4831	2.03	0.18
rs9903337	125206	SLC5A10	17	18798782	9.32E-03	1563	9.18E-01	Up	0.10	13363	2.03	0.00
rs2875955	9425	CDYL	6	4635759	9.33E-03	1564	2.25E-01	Down	1.21	8511	2.03	0.06
rs1426738	27071	DAPP1	4	101085876	9.36E-03	1565	3.06E-03	Down	2.96	3783	2.03	0.25
rs4820946	91445	RNF185	22	29875714	9.37E-03	1566	3.69E-01	Down	0.90	9755	2.03	0.04
rs10517929	50859	SPOCK3	4	168543600	9.38E-03	1567	1.83E-05	Down	4.28	2074	2.03	0.47
rs2067793	955	ENTPD6	20	25162072	9.38E-03	1568	5.38E-11	Up	6.64	693	2.03	1.03
rs2067793	5834	PYGB	20	25162072	9.38E-03	1569	1.49E-02	Down	2.44	4806	2.03	0.18
rs4853462	4430	MYO1B	2	191949310	9.38E-03	1570	4.17E-09	Up	5.88	996	2.03	0.84
rs1414703	2262	GPC5	13	90947413	9.40E-03	1571	1.89E-01	Up	1.31	8139	2.03	0.07
rs17052610	2554	GABRA1	5	161248390	9.41E-03	1572	3.30E-01	Down	0.97	9443	2.03	0.05
rs923942	2786	GNG4	1	232041070	9.42E-03	1573	7.70E-01	Up	0.29	12493	2.03	0.01
rs625880	79683	ZDHHC14	6	158057321	9.42E-03	1574	7.37E-02	Down	1.79	6498	2.03	0.11
rs3138112	6364	CCL20	2	228492961	9.43E-03	1575	6.43E-01	Up	0.46	11730	2.03	0.02
rs7545388	9910	RABGAP1L	1	170997080	9.44E-03	1576	8.88E-02	Down	1.70	6782	2.03	0.11
rs3732360	8856	NR1I2	3	121019271	9.46E-03	1577	1.42E-01	Up	1.47	7605	2.02	0.08
rs12623237	57405	SPC25	2	169581565	9.46E-03	1578	8.81E-03	Up	2.62	4416	2.02	0.21
rs12623237	57818	G6PC2	2	169581565	9.46E-03	1579	2.43E-01	Down	1.17	8698	2.02	0.06
rs2764448	89884	LHX4	1	176966299	9.47E-03	1580	6.59E-01	Up	0.44	11827	2.02	0.02
rs12401573	9673	SLC25A44	1	152959291	9.47E-03	1581	1.28E-01	Down	1.52	7425	2.02	0.09
rs12401573	64218	SEMA4A	1	152959291	9.47E-03	1582	4.44E-01	Down	0.77	10301	2.02	0.04
rs10071838	92255	LMBRD2	5	36183563	9.48E-03	1583	8.57E-02	Up	1.72	6730	2.02	0.11
rs10071838	6502	SKP2	5	36183563	9.48E-03	1584	7.00E-01	Down	0.39	12069	2.02	0.02
rs1555084	2911	GRM1	6	146662358	9.50E-03	1585	5.47E-01	Up	0.60	11062	2.02	0.03
rs705117	2638	GC	4	72973150	9.51E-03	1586	4.81E-03	Up	2.82	4033	2.02	0.23
rs194522	261729	STEAP2	7	89504628	9.52E-03	1587	1.34E-02	Up	2.47	4716	2.02	0.19
rs4823197	29780	PARVB	22	42881964	9.53E-03	1588	3.22E-04	Up	3.60	2800	2.02	0.35
rs4823197	64098	PARVG	22	42881964	9.53E-03	1589	1.17E-01	Up	1.57	7253	2.02	0.09
rs2256928	84978	FRMD5	15	42262198	9.53E-03	1590	1.04E-01	Down	1.63	7052	2.02	0.10
rs713397	130535	KCTD18	2	201183130	9.53E-03	1591	4.89E-01	Up	0.69	10645	2.02	0.03
rs10858049	163259	DENND2C	1	114908208	9.55E-03	1592	1.80E-02	Down	2.36	4987	2.02	0.17
rs287943	57492	ARID1B	6	157316325	9.56E-03	1593	7.33E-01	Up	0.34	12271	2.02	0.01
rs7994305	1282	COL4A1	13	109666461	9.57E-03	1594	6.42E-05	Down	4.00	2343	2.02	0.42
rs4767364	80018	C12orf30	12	110984168	9.57E-03	1595	1.28E-02	Up	2.49	4672	2.02	0.19
rs3915790	7554	ZNF8	19	63507665	9.57E-03	1596	8.86E-02	Up	1.70	6777	2.02	0.11
rs352492	170961	ANKRD24	19	4130636	9.57E-03	1597	3.38E-01	Up	0.96	9507	2.02	0.05
rs352492	51548	SIRT6	19	4130636	9.57E-03	1598	9.68E-01	Down	0.04	13688	2.02	0.00
rs6512211	23025	UNC13A	19	17643217	9.58E-03	1599	4.69E-02	Down	1.99	5921	2.02	0.13
rs17096088	23002	DAAM1	14	58861960	9.59E-03	1600	1.35E-01	Down	1.49	7514	2.02	0.09
rs1495946	116228	FAM36A	1	241339848	9.60E-03	1601	1.21E-03	Up	3.24	3297	2.02	0.29
rs9935192	84256	FLYWCH1	16	2909857	9.61E-03	1602	5.96E-01	Down	0.53	11408	2.02	0.02
rs2741584	51604	PIGT	20	43477925	9.61E-03	1603	9.79E-01	Down	0.03	13764	2.02	0.00
rs6136684	57419	SLC24A3	20	19263263	9.65E-03	1604	3.83E-04	Down	3.55	2866	2.02	0.34
rs2267549	4704	NDUFA9	12	4641943	9.65E-03	1605	4.23E-02	Up	2.03	5804	2.02	0.14
rs2267549	10566	AKAP3	12	4641943	9.65E-03	1606	6.56E-01	Up	0.45	11807	2.02	0.02
rs16530	6143	RPL19	17	34610560	9.66E-03	1607	6.40E-06	Up	4.51	1860	2.02	0.52
rs16530	342667	STAC2	17	34610560	9.66E-03	1608	4.32E-01	Down	0.79	10210	2.02	0.04
rs16530	782	CACNB1	17	34610560	9.66E-03	1609	4.63E-01	Down	0.73	10447	2.02	0.03
rs1805419	581	BAX	19	54150916	9.67E-03	1610	2.26E-05	Up	4.24	2112	2.01	0.46
rs1805419	2512	FTL	19	54150916	9.67E-03	1611	2.62E-02	Up	2.22	5330	2.01	0.16
rs2726556	9760	TOX	8	59955332	9.70E-03	1612	1.44E-02	Up	2.45	4777	2.01	0.18
rs17566701	27342	RABGEF1	7	65637333	9.71E-03	1613	2.42E-02	Down	2.25	5256	2.01	0.16
rs8116803	7150	TOP1	20	39167195	9.71E-03	1614	1.36E-07	Up	5.27	1329	2.01	0.69
rs17839317	1016	CDH18	5	19778490	9.72E-03	1615	1.43E-01	Down	1.46	7612	2.01	0.08

rs1345451	201134	CCDC46	17	61335698	9.72E-03	1616	2.35E-01	Down	1.19	8617	2.01	0.06
rs1040530	2762	GMDS	6	1703178	9.73E-03	1617	2.80E-07	Up	5.14	1410	2.01	0.66
rs10490182	92737	DNER	2	230137766	9.74E-03	1618	7.08E-02	Down	1.81	6447	2.01	0.12
rs732096	132001	C3orf31	3	11868950	9.75E-03	1619	4.53E-01	Up	0.75	10365	2.01	0.03
rs11189513	27291	C10orf28	10	99959558	9.75E-03	1620	9.33E-01	Up	0.08	13455	2.01	0.00
rs1354106	945	CD33	19	56429803	9.76E-03	1621	1.04E-01	Up	1.63	7062	2.01	0.10
rs12306394	3678	ITGA5	12	53117545	9.77E-03	1622	4.80E-08	Down	5.46	1216	2.01	0.73
rs2899632	3990	LIPC	15	56599161	9.78E-03	1623	2.55E-01	Up	1.14	8803	2.01	0.06
rs6822076	84570	COL25A1	4	110577988	9.83E-03	1624	7.98E-01	Down	0.26	12660	2.01	0.01
rs1005204	284353	NKPD1	19	50342528	9.85E-03	1625	9.86E-01	Up	0.02	13819	2.01	0.00
rs3829020	116447	TOP1MT	8	144480156	9.86E-03	1626	1.66E-01	Up	1.39	7879	2.01	0.08
rs10487472	2918	GRM8	7	126459988	9.86E-03	1627	5.45E-03	Up	2.78	4122	2.01	0.23
rs2675511	185	AGTR1	3	149928316	9.86E-03	1628	3.73E-06	Up	4.63	1773	2.01	0.54
rs9481825	5350	PLN	6	118982785	9.87E-03	1629	2.41E-12	Down	7.01	591	2.01	1.16
rs9897022	23614	PPY2	17	23578902	9.88E-03	1630	2.83E-01	Up	1.07	9064	2.01	0.05
rs2239206	7837	PXDN	12	119138810	9.88E-03	1631	-3.69E-11	Up	6.43	767	2.01	#NUM!
rs2239206	5829	PXN	12	119138810	9.88E-03	1632	1.53E-08	Up	5.64	1108	2.01	0.78
rs9936204	54758	KLHDC4	16	86366198	9.88E-03	1633	7.50E-01	Down	0.32	12375	2.01	0.01
rs9478020	253769	WDR27	6	169666278	9.89E-03	1634	2.04E-01	Down	1.27	8299	2.00	0.07
rs1470461	1767	DNAH5	5	13914175	9.90E-03	1635	8.17E-30	Up	11.32	67	2.00	2.91
rs17236487	286827	TRIM59	3	161654628	9.92E-03	1636	9.55E-04	Up	3.30	3202	2.00	0.30
rs10173426	9577	BRE	2	28221228	9.92E-03	1637	6.31E-02	Up	1.86	6301	2.00	0.12
rs3736495	22801	ITGA11	15	66400544	9.92E-03	1638	4.85E-01	Down	0.70	10614	2.00	0.03
rs3766236	8569	MKNK1	1	46759461	9.93E-03	1639	6.28E-05	Down	4.00	2338	2.00	0.42
rs871748	79754	ASB13	10	5741801	9.94E-03	1640	4.05E-03	Up	2.87	3940	2.00	0.24
rs1512019	8618	CADPS	3	62537831	9.96E-03	1641	1.34E-01	Up	1.50	7495	2.00	0.09
rs697963	84692	CCDC54	3	108561177	9.97E-03	1642	4.85E-01	Down	0.70	10606	2.00	0.03
rs7183000	170691	ADAMTS17	15	98508979	9.97E-03	1643	6.57E-01	Down	0.44	11817	2.00	0.02
rs7635040	11148	HHLA2	3	109582550	9.98E-03	1644	6.40E-01	Up	0.47	11712	2.00	0.02
rs7635040	22989	MYH15	3	109582550	9.98E-03	1645	7.56E-01	Up	0.31	12416	2.00	0.01
rs755730	9953	HS3ST3B1	17	14138176	9.98E-03	1646	1.85E-03	Down	3.11	3492	2.00	0.27
rs16894099	373	TRIM23	5	64945243	9.99E-03	1647	1.00E-04	Down	3.89	2453	2.00	0.40
rs3794716	3607	FOXK2	17	78152456	9.99E-03	1648	4.04E-02	Up	2.05	5757	2.00	0.14
rs7117376	7762	ZNF215	11	6943002	1.00E-02	1649	5.59E-01	Down	0.58	11158	2.00	0.03
rs12913189	28232	SLCO3A1	15	90366611	1.00E-02	1650	2.56E-09	Down	5.96	952	2.00	0.86
rs6976789	9805	SCRN1	7	29736174	1.00E-02	1651	4.50E-35	Down	12.36	41	2.00	3.43
rs7173713	2562	GABRB3	15	24437247	1.00E-02	1652	4.23E-25	Up	10.34	113	2.00	2.44
rs10832312	5140	PDE3B	11	14844406	1.00E-02	1653	3.69E-08	Up	5.50	1194	2.00	0.74
rs10021966	8633	UNC5C	4	96807271	1.00E-02	1654	2.33E-01	Up	1.19	8590	2.00	0.06
rs11080994	84034	EMILIN2	18	2887993	1.00E-02	1655	3.36E-01	Up	0.96	9493	2.00	0.05
rs10428802	167826	OLIG3	6	137875749	1.00E-02	1656	8.34E-01	Down	0.21	12872	2.00	0.01
rs7013140	1666	DECR1	8	91068793	1.01E-02	1657	1.50E-04	Up	3.79	2553	2.00	0.38
rs7013140	55655	NLRP2	8	91068793	1.01E-02	1658	1.34E-01	Up	1.50	7486	2.00	0.09
rs352480	54878	DPP8	15	63587263	1.01E-02	1659	1.77E-01	Down	1.35	7983	2.00	0.08
rs4965133	145946	SPATA8	15	95116029	1.01E-02	1660	8.03E-01	Down	0.25	12692	2.00	0.01
rs3769579	55622	TTC27	2	32887738	1.01E-02	1661	1.95E-01	Up	1.30	8202	2.00	0.07
rs4235151	57205	ATP10D	4	47345089	1.01E-02	1662	2.22E-03	Down	3.06	3603	2.00	0.27
rs2303054	8541	PPFIA3	19	54332106	1.01E-02	1663	7.68E-01	Down	0.29	12478	2.00	0.01
rs4294134	23165	NUP205	7	134750383	1.01E-02	1664	7.40E-03	Up	2.68	4294	1.99	0.21
rs12123388	24	ABCA4	1	94225122	1.01E-02	1665	9.74E-01	Up	0.03	13723	1.99	0.00
rs3745642	8725	C19orf2	19	35106427	1.01E-02	1666	5.98E-06	Up	4.53	1842	1.99	0.52
rs7873936	23413	FREQ	9	130072716	1.02E-02	1667	9.68E-08	Down	5.33	1294	1.99	0.70
rs2618402	6854	SYN2	3	12014799	1.02E-02	1668	4.17E-01	Down	0.81	10105	1.99	0.04
rs6684	29078	C6orf66	6	97445799	1.02E-02	1669	2.41E-01	Up	1.17	8671	1.99	0.06
rs2108639	93589	CACNA2D4	12	1843645	1.02E-02	1670	7.29E-03	Down	2.68	4283	1.99	0.21
rs11602817	4676	NAP1L4	11	2933287	1.02E-02	1671	6.57E-01	Up	0.44	11811	1.99	0.02
rs7400029	348013	FAM70B	13	113611527	1.02E-02	1672	1.97E-01	Down	1.29	8227	1.99	0.07
rs2735839	354	KLK3	19	56056435	1.02E-02	1673	3.14E-09	Up	5.92	977	1.99	0.85
rs2735839	3817	KLK2	19	56056435	1.02E-02	1674	2.70E-07	Up	5.14	1403	1.99	0.66
rs7865565	84628	NTNG2	9	132111068	1.02E-02	1675	5.34E-04	Down	3.46	2981	1.99	0.33
rs2043385	80333	KCNIP4	4	20824383	1.02E-02	1676	2.36E-02	Up	2.26	5222	1.99	0.16
rs1552813	26188	OR1C1	1	244247393	1.02E-02	1677	1.26E-01	Down	1.53	7382	1.99	0.09
rs3755496	54520	CCDC93	2	118423904	1.02E-02	1678	3.48E-01	Down	0.94	9574	1.99	0.05
rs1567778	54847	SIDT1	3	114809359	1.03E-02	1679	1.21E-01	Down	1.55	7319	1.99	0.09
rs2305317	92105	INTS4	11	77349192	1.03E-02	1680	1.46E-01	Down	1.45	7637	1.99	0.08
rs17122349	5018	OXA1L	14	22316671	1.03E-02	1681	1.78E-02	Up	2.37	4977	1.99	0.17
rs847057	64849	SLC13A3	20	44666440	1.03E-02	1682	1.88E-07	Up	5.21	1365	1.99	0.67
rs1777276	8613	PPAP2B	1	56700498	1.03E-02	1683	4.23E-24	Down	10.13	124	1.99	2.34
rs485211	9097	USP14	18	156277	1.03E-02	1684	7.29E-01	Up	0.35	12241	1.99	0.01
rs701755	1356	CP	3	150385412	1.03E-02	1685	4.15E-01	Down	0.82	10088	1.99	0.04
rs550328	84441	MAML2	11	95604702	1.03E-02	1686	8.73E-05	Down	3.92	2417	1.99	0.41
rs9290412	23043	TNIK	3	172591301	1.03E-02	1687	1.50E-01	Down	1.44	7687	1.99	0.08
rs10771892	55196	C12orf35	12	32011514	1.04E-02	1688	1.18E-01	Down	1.56	7263	1.98	0.09
rs2301346	5826	ABCD4	14	73835833	1.04E-02	1689	5.24E-02	Up	1.94	6047	1.98	0.13
rs10166971	7360	UGP2	2	64029595	1.04E-02	1690	8.68E-13	Down	7.15	553	1.98	1.21
rs10166971	51542	VPS54	2	64029595	1.04E-02	1691	2.54E-06	Up	4.70	1703	1.98	0.56
rs4830807	51311	TLR8		12691854	1.04E-02	1692	8.53E-02	Down	1.72	6728	1.98	0.11
rs7204722	81631	MAP1LC3B	16	85987740	1.04E-02	1693	1.99E-13	Down	7.35	492	1.98	1.27
rs7204722	23174	ZCCHC14	16	85987740	1.04E-02	1694	5.19E-04	Down	3.47	2970	1.98	0.33
rs7204722	26271	FBXO5	16	85987740	1.04E-02	1695	3.18E-02	Up	2.15	5520	1.98	0.15
rs7204722	79791	FBXO31	16	85987740	1.04E-02	1696	3.10E-01	Up	1.01	9268	1.98	0.05

rs9332684	6403	SELP	1	166283193	1.04E-02	1697	2.43E-01	Down	1.17	8695	1.98	0.06
rs7155416	2103	ESRRB	14	76021126	1.04E-02	1698	6.46E-01	Down	0.46	11754	1.98	0.02
rs11084211	399669	ZNF321	19	58152673	1.04E-02	1699	6.00E-02	Down	1.88	6235	1.98	0.12
rs2189461	30010	NXPH1	7	8367603	1.05E-02	1700	2.90E-01	Up	1.06	9112	1.98	0.05
rs709767	126820	WDR63	1	85273975	1.05E-02	1701	7.56E-01	Up	0.31	12418	1.98	0.01
rs12974044	6223	RPS19	19	47060469	1.05E-02	1702	4.79E-15	Up	7.83	388	1.98	1.43
rs12974044	973	CD79A	19	47060469	1.05E-02	1703	5.24E-01	Up	0.64	10895	1.98	0.03
rs12974044	9138	ARHGEF1	19	47060469	1.05E-02	1704	5.75E-01	Up	0.56	11278	1.98	0.02
rs10490450	4052	LTBP1	2	33244589	1.05E-02	1705	3.05E-06	Down	4.67	1742	1.98	0.55
rs946236	9223	MAGI1	6	154895996	1.05E-02	1706	5.23E-01	Down	0.64	10875	1.98	0.03
rs2402960	4779	NFE2L1	7	128999725	1.06E-02	1707	4.73E-10	Down	6.23	850	1.98	0.93
rs2402960	4899	NRF1	7	128999725	1.06E-02	1708	5.51E-01	Down	0.60	11087	1.98	0.03
rs7827617	8989	TRPA1	8	73096586	1.06E-02	1709	7.38E-01	Up	0.33	12302	1.98	0.01
rs751503	10486	CAP2	6	17601072	1.06E-02	1710	8.48E-15	Down	7.76	403	1.98	1.41
rs751503	5271	SERPINB8	6	17601072	1.06E-02	1711	8.36E-01	Down	0.21	12884	1.98	0.01
rs12545375	8794	TNFRSF10C	8	23007769	1.06E-02	1712	1.54E-02	Down	2.42	4832	1.98	0.18
rs17148984	53838	C11orf24	11	67784557	1.06E-02	1713	3.88E-01	Up	0.86	9902	1.98	0.04
rs8088001	9984	THOC1	18	277705	1.06E-02	1714	1.40E-01	Up	1.47	7579	1.97	0.09
rs2232041	55791	C1orf103	1	111206236	1.06E-02	1715	1.41E-08	Up	5.64	1110	1.97	0.79
rs2232041	55183	RIF1	1	111206236	1.06E-02	1716	1.01E-01	Up	1.64	7010	1.97	0.10
rs135757	1454	CSNK1E	22	37028153	1.06E-02	1717	1.53E-02	Up	2.43	4823	1.97	0.18
rs6793960	26032	SUSD5	3	33189680	1.07E-02	1718	1.21E-01	Up	1.55	7310	1.97	0.09
rs1027990	4067	LYN	8	56976863	1.07E-02	1719	1.70E-02	Up	2.39	4934	1.97	0.18
rs12596169	23019	CNOT1	16	57172724	1.07E-02	1720	1.75E-01	Up	1.36	7961	1.97	0.08
rs12490645	5580	PRKCD	3	53203959	1.07E-02	1721	9.20E-08	Up	5.34	1288	1.97	0.70
rs2276432	9152	SLC6A5	11	20632829	1.07E-02	1722	1.08E-01	Up	1.61	7117	1.97	0.10
rs1250255	2335	FN1	2	216141993	1.07E-02	1723	3.52E-09	Down	5.91	986	1.97	0.85
rs2197444	143187	VTI1A	10	114413909	1.07E-02	1724	1.58E-01	Down	1.41	7789	1.97	0.08
rs807812	56949	XAB2	19	7587351	1.07E-02	1725	2.99E-02	Up	2.17	5458	1.97	0.15
rs807812	57662	KIAA1543	19	7587351	1.07E-02	1726	4.13E-01	Up	0.82	10076	1.97	0.04
rs1026277	79745	CLIP4	2	29324945	1.07E-02	1727	3.53E-15	Down	7.87	382	1.97	1.45
rs712039	11072	DUSP14	17	32924666	1.08E-02	1728	7.63E-01	Up	0.30	12455	1.97	0.01
rs7398481	5715	PSMD9	12	120811466	1.08E-02	1729	1.53E-01	Up	1.43	7718	1.97	0.08
rs7398481	144406	WDR66	12	120811466	1.08E-02	1730	2.55E-01	Up	1.14	8805	1.97	0.06
rs2820093	27159	CHIA	1	111575348	1.08E-02	1731	9.81E-04	Down	3.30	3215	1.97	0.30
rs6686101	55970	GNG12	1	68016661	1.08E-02	1732	1.26E-06	Down	4.85	1606	1.97	0.59
rs13231277	168507	PKD1L1	7	47613627	1.08E-02	1733	9.58E-01	Up	0.05	13609	1.97	0.00
rs11089573	8224	SYN3	22	31269332	1.08E-02	1734	6.69E-01	Down	0.43	11878	1.97	0.02
rs11071474	663	BNIP2	15	57788352	1.08E-02	1735	1.30E-01	Down	1.51	7448	1.97	0.09
rs2071409	4025	LPO	17	53703229	1.08E-02	1736	7.75E-01	Down	0.29	12529	1.97	0.01
rs1053006	9209	LRRFIP2	3	37199103	1.09E-02	1737	3.90E-05	Down	4.11	2224	1.96	0.44
rs11608732	283431	GAS2L3	12	99496139	1.09E-02	1738	5.46E-02	Up	1.92	6102	1.96	0.13
rs4389435	5333	PLCD1	3	38055105	1.09E-02	1739	3.45E-03	Down	2.92	3847	1.96	0.25
rs3800688	5420	PODXL	7	130649730	1.09E-02	1740	7.85E-02	Up	1.76	6604	1.96	0.11
rs3820548	6513	SLC2A1	1	43067489	1.09E-02	1741	5.23E-01	Down	0.64	10882	1.96	0.03
rs1860068	9955	HS3ST3A1	17	13348823	1.09E-02	1742	1.15E-03	Down	3.25	3276	1.96	0.29
rs13180883	7204	TRIO	5	14550279	1.09E-02	1743	1.53E-06	Up	4.81	1631	1.96	0.58
rs2077513	5161	PDHA2	4	97118072	1.09E-02	1744	1.32E-02	Down	2.48	4706	1.96	0.19
rs221946	79921	TCEAL4		102637362	1.09E-02	1745	5.34E-11	Down	6.56	719	1.96	1.03
rs6841833	23216	TBC1D1	4	37827532	1.10E-02	1746	4.58E-10	Down	6.23	847	1.96	0.93
rs2282092	119391	GSTO2	10	106058461	1.10E-02	1747	1.21E-09	Down	6.08	906	1.96	0.89
rs3763851	10482	NXF1	11	62329009	1.10E-02	1748	4.77E-01	Down	0.71	10553	1.96	0.03
rs17034354	57535	KIAA1324	1	109455209	1.10E-02	1749	3.13E-02	Up	2.15	5507	1.96	0.15
rs11719053	22908	SACM1L	3	45696019	1.10E-02	1750	8.73E-02	Down	1.71	6758	1.96	0.11
rs11719053	8994	LIMD1	3	45696019	1.10E-02	1751	1.61E-01	Down	1.40	7829	1.96	0.08
rs1628924	2620	GAS2	11	22673696	1.10E-02	1752	7.02E-02	Up	1.81	6429	1.96	0.12
rs12489456	6533	SLC6A6	3	14476178	1.10E-02	1753	9.71E-02	Down	1.66	6943	1.96	0.10
rs1654467	54869	EPS8L1	19	60273323	1.10E-02	1754	2.07E-02	Down	2.31	5104	1.96	0.17
rs1654467	112724	RDH13	19	60273323	1.10E-02	1755	2.51E-01	Up	1.15	8773	1.96	0.06
rs217180	83449	PMFBP1	16	70742130	1.10E-02	1756	1.23E-01	Up	1.54	7341	1.96	0.09
rs11193190	114815	SORCS1	10	108808277	1.11E-02	1757	6.51E-01	Down	0.45	11780	1.96	0.02
rs2067434	3628	INPP1	2	191055852	1.11E-02	1758	1.73E-10	Down	6.38	788	1.96	0.98
rs2306181	51071	DERA	12	16000940	1.11E-02	1759	2.55E-02	Down	2.23	5304	1.96	0.16
rs5973911	340596	LHFPL1		111674026	1.11E-02	1760	3.10E-01	Down	1.01	9272	1.96	0.05
rs12671126	10512	SEMA3C	7	80047836	1.11E-02	1761	1.56E-08	Down	5.66	1094	1.96	0.78
rs17182699	57862	ZNF410	14	73472446	1.11E-02	1762	3.64E-03	Down	2.91	3880	1.96	0.24
rs17182699	51004	COQ6	14	73472446	1.11E-02	1763	2.10E-01	Up	1.25	8346	1.96	0.07
rs7816934	4325	MMP16	8	89381507	1.11E-02	1764	1.61E-01	Up	1.40	7821	1.96	0.08
rs5374	2587	GALR1	18	73091633	1.11E-02	1765	1.50E-02	Up	2.43	4815	1.96	0.18
rs2428684	80221	ACSF2	17	45867133	1.11E-02	1766	3.56E-18	Down	8.69	264	1.95	1.74
rs7098284	4153	MBL2	10	54187678	1.11E-02	1767	3.34E-01	Up	0.97	9478	1.95	0.05
rs220549	2904	GRIN2B	12	13828587	1.11E-02	1768	5.90E-01	Down	0.54	11369	1.95	0.02
rs12154942	6733	SRPK2	7	104453386	1.11E-02	1769	4.99E-01	Down	0.68	10705	1.95	0.03
rs12656965	55781	RIOK2	5	96564341	1.11E-02	1770	2.24E-02	Up	2.28	5177	1.95	0.16
rs1325432	2672	GFI1	1	92652871	1.11E-02	1771	1.60E-01	Up	1.41	7814	1.95	0.08
rs1058511	125965	COX6B2	19	60551251	1.11E-02	1772	7.86E-02	Up	1.76	6606	1.95	0.11
rs1058511	84787	SUV420H2	19	60551251	1.11E-02	1773	5.55E-01	Down	0.59	11115	1.95	0.03
rs13047838	7031	TFF1	21	42665938	1.12E-02	1774	2.24E-03	Down	3.06	3608	1.95	0.27
rs2282869	2115	ETV1	7	13776345	1.12E-02	1775	9.31E-03	Up	2.60	4454	1.95	0.20
rs13047838	64699	TMPRSS3	21	42665938	1.12E-02	1776	7.52E-02	Down	1.78	6531	1.95	0.11
rs13047838	56649	TMPRSS4	21	42665938	1.12E-02	1777	7.36E-01	Down	0.34	12290	1.95	0.01

rs4796604	328	APEX1	17	37144402	1.12E-02	1778	1.93E-10	Up	6.37	790	1.95	0.97
rs4796604	9001	HAP1	17	37144402	1.12E-02	1779	4.99E-01	Up	0.68	10708	1.95	0.03
rs6115181	117532	TMC2	20	2542254	1.12E-02	1780	3.22E-01	Down	0.99	9378	1.95	0.05
rs11862806	26000	TBC1D10B	16	30271572	1.12E-02	1781	9.02E-01	Up	0.12	13261	1.95	0.00
rs11862806	10421	CD2BP2	16	30271572	1.12E-02	1782	9.66E-01	Up	0.04	13664	1.95	0.00
rs11922919	7476	WNT7A	3	13854617	1.12E-02	1783	4.33E-01	Down	0.78	10220	1.95	0.04
rs16830500	785	CACNB4	2	152639637	1.12E-02	1784	8.88E-01	Up	0.14	13178	1.95	0.01
rs12457258	9352	TXNL1	18	52416667	1.12E-02	1785	3.44E-05	Up	4.14	2198	1.95	0.45
rs7897216	80760	ITIH5	10	7762854	1.12E-02	1786	2.70E-04	Down	3.64	2742	1.95	0.36
rs776785	92949	ADAMTSL1	9	18641444	1.12E-02	1787	2.22E-03	Down	3.06	3601	1.95	0.27
rs1427800	1582	CYP8B1	3	42903519	1.12E-02	1788	1.63E-01	Up	1.39	7853	1.95	0.08
rs1890227	11149	BVES	6	105651496	1.12E-02	1789	5.58E-04	Down	3.45	3000	1.95	0.33
rs12726299	55356	SLC22A15	1	116237714	1.13E-02	1790	4.53E-01	Up	0.75	10369	1.95	0.03
rs6477710	158431	ZNF782	9	96664869	1.13E-02	1791	5.84E-01	Up	0.55	11339	1.95	0.02
rs12204438	1310	COL19A1	6	70918208	1.13E-02	1792	9.04E-05	Up	3.92	2424	1.95	0.40
rs13225343	231	AKR1B1	7	133579695	1.13E-02	1793	4.49E-14	Down	7.55	450	1.95	1.33
rs2280401	2217	FCGRT	19	54691821	1.13E-02	1794	7.91E-31	Down	11.54	59	1.95	3.01
rs2280401	6205	RPS11	19	54691821	1.13E-02	1795	3.07E-01	Up	1.02	9244	1.95	0.05
rs11172162	55605	KIF21A	12	38129079	1.13E-02	1796	3.01E-03	Up	2.97	3779	1.95	0.25
rs1769299	6512	SLC1A7	1	53297744	1.13E-02	1797	9.02E-02	Down	1.69	6821	1.95	0.10
rs7526168	115353	LRRC42	1	54125443	1.13E-02	1798	1.12E-01	Down	1.59	7173	1.95	0.10
rs196067	11015	KDELR3	22	37178177	1.13E-02	1799	9.74E-01	Down	0.03	13721	1.95	0.00
rs6441061	7881	KCNAB1	3	157550596	1.14E-02	1800	3.45E-18	Down	8.70	263	1.94	1.75
rs5005161	23322	RPGRIP1L	16	52256177	1.14E-02	1801	1.80E-03	Up	3.12	3474	1.94	0.27
rs988987	11342	RNF13	3	150997486	1.14E-02	1802	1.94E-01	Down	1.30	8194	1.94	0.07
rs165172	29995	LMCD1	3	8591128	1.14E-02	1803	7.98E-10	Down	6.15	878	1.94	0.91
rs165172	29931	LOH3CR2A	3	8591128	1.14E-02	1804	1.55E-01	Down	1.42	7744	1.94	0.08
rs13080849	10686	CLDN16	3	191588153	1.14E-02	1805	2.69E-01	Up	1.11	8942	1.94	0.06
rs1807211	3752	KCND3	1	112202490	1.14E-02	1806	6.50E-01	Down	0.45	11774	1.94	0.02
rs10786637	8945	BTRC	10	103218826	1.14E-02	1807	4.10E-01	Up	0.82	10059	1.94	0.04
rs288324	2487	FRZB	2	183527094	1.14E-02	1808	2.53E-01	Up	1.14	8795	1.94	0.06
rs2899748	26035	GLCE	15	67311772	1.14E-02	1809	3.67E-02	Up	2.09	5671	1.94	0.14
rs597327	26011	ODZ4	11	78115000	1.14E-02	1810	1.22E-03	Down	3.23	3300	1.94	0.29
rs959309	64410	KLHL25	15	84097245	1.14E-02	1811	2.04E-01	Down	1.27	8298	1.94	0.07
rs2686386	5025	P2RX4	12	120102377	1.15E-02	1812	5.54E-17	Up	8.38	311	1.94	1.63
rs17222478	10783	NEK6	9	124145390	1.15E-02	1813	9.43E-03	Up	2.60	4465	1.94	0.20
rs4790666	124935	SLC43A2	17	1440465	1.15E-02	1814	1.07E-01	Down	1.61	7111	1.94	0.10
rs6065338	90187	EMILIN3	20	39421666	1.15E-02	1815	1.59E-08	Down	5.65	1098	1.94	0.78
rs16873582	57670	KIAA1549	7	138053256	1.15E-02	1816	7.30E-13	Up	7.17	546	1.94	1.21
rs7744	4615	MYD88	3	38159025	1.15E-02	1817	9.48E-02	Up	1.67	6906	1.94	0.10
rs7744	30	ACAA1	3	38159025	1.15E-02	1818	2.16E-01	Up	1.24	8428	1.94	0.07
rs941718	60686	C14orf93	14	22564888	1.15E-02	1819	4.32E-01	Down	0.79	10209	1.94	0.04
rs941718	5693	PSMB5	14	22564888	1.15E-02	1820	8.90E-01	Up	0.14	13185	1.94	0.01
rs17500692	116	ADCYAP1	18	890789	1.15E-02	1821	9.78E-01	Up	0.03	13752	1.94	0.00
rs4887041	1381	CRABP1	15	76435017	1.15E-02	1822	8.18E-01	Up	0.23	12778	1.94	0.01
rs1669925	144165	PRICKLE1	12	41172484	1.15E-02	1823	2.48E-01	Down	1.16	8748	1.94	0.06
rs9873604	285349	ZNF660	3	44587054	1.15E-02	1824	3.35E-01	Down	0.96	9487	1.94	0.05
rs9873604	55888	ZNF167	3	44587054	1.15E-02	1825	9.08E-01	Up	0.12	13295	1.94	0.00
rs2247289	84798	C19orf48	19	55999904	1.15E-02	1826	2.50E-09	Up	5.96	949	1.94	0.86
rs2247289	93650	ACPT	19	55999904	1.15E-02	1827	2.45E-01	Up	1.16	8717	1.94	0.06
rs136867	9681	DEPDC5	22	30597212	1.16E-02	1828	7.81E-01	Up	0.28	12551	1.94	0.01
rs1079199	322	APBB1	11	6384682	1.16E-02	1829	9.63E-06	Down	4.43	1944	1.94	0.50
rs1868009	9699	RIMS2	8	105337945	1.16E-02	1830	2.72E-03	Up	3.00	3731	1.94	0.26
rs11693163	248	ALPI	2	233151951	1.16E-02	1831	7.48E-01	Up	0.32	12361	1.94	0.01
rs9890302	8522	GAS7	17	9806152	1.16E-02	1832	2.71E-06	Down	4.69	1717	1.94	0.56
rs6084547	11237	RNF24	20	3941354	1.16E-02	1833	9.86E-01	Down	0.02	13817	1.94	0.00
rs150348	221184	CPNE2	16	55673537	1.16E-02	1834	1.95E-03	Up	3.10	3518	1.93	0.27
rs150348	84166	NLRC5	16	55673537	1.16E-02	1835	9.77E-01	Up	0.03	13743	1.93	0.00
rs3754809	10884	MRPS30	2	79301180	1.17E-02	1836	2.16E-03	Up	3.07	3585	1.93	0.27
rs3754809	10914	PAPOLA	2	79301180	1.17E-02	1837	7.05E-03	Up	2.69	4264	1.93	0.22
rs3754809	11334	TUSC2	2	79301180	1.17E-02	1838	4.57E-01	Down	0.74	10399	1.93	0.03
rs3763015	2878	GPX3	5	150377358	1.17E-02	1839	2.57E-09	Down	5.96	953	1.93	0.86
rs4726499	6916	TBXAS1	7	139141188	1.17E-02	1840	7.39E-01	Up	0.33	12306	1.93	0.01
rs8793	51643	TMBIM4	12	64832367	1.17E-02	1841	1.61E-01	Up	1.40	7831	1.93	0.08
rs6963048	51667	NUB1	7	150506944	1.17E-02	1842	4.49E-01	Up	0.76	10341	1.93	0.03
rs1571332	10439	OLFM1	9	135228659	1.17E-02	1843	1.02E-05	Down	4.41	1952	1.93	0.50
rs6114735	79953	C20orf39	20	24393155	1.17E-02	1844	3.89E-02	Down	2.07	5727	1.93	0.14
rs11235937	51287	CHCHD8	11	73264177	1.17E-02	1845	7.19E-09	Up	5.79	1033	1.93	0.81
rs11235937	80227	PAAF1	11	73264177	1.17E-02	1846	3.36E-02	Up	2.12	5579	1.93	0.15
rs1441555	57626	KLHL1	13	69524947	1.18E-02	1847	5.61E-02	Down	1.91	6139	1.93	0.13
rs1528734	26585	GREM1	15	30802498	1.18E-02	1848	1.18E-06	Up	4.86	1599	1.93	0.59
rs2196521	4311	MME	3	156295340	1.18E-02	1849	8.55E-23	Down	9.83	144	1.93	2.21
rs1288572	83891	SNX25	4	186627287	1.18E-02	1850	2.60E-01	Up	1.13	8864	1.93	0.06
rs6086545	51378	ANGPT4	20	863863	1.18E-02	1851	6.21E-01	Down	0.49	11589	1.93	0.02
rs10787428	57678	GPAM	10	113925369	1.18E-02	1852	1.43E-03	Up	3.19	3357	1.93	0.28
rs2041689	79998	ANKRD53	2	71114997	1.18E-02	1853	2.23E-02	Down	2.29	5175	1.93	0.17
rs2041689	113419	TEX261	2	71114997	1.18E-02	1854	7.57E-01	Down	0.31	12423	1.93	0.01
rs9535762	79758	DHRS12	13	51288604	1.18E-02	1855	7.73E-01	Up	0.29	12520	1.93	0.01
rs4770121	78988	MRP63	13	20630094	1.19E-02	1856	3.15E-09	Up	5.92	975	1.93	0.85
rs4770121	10284	SAP18	13	20630094	1.19E-02	1857	4.37E-05	Down	4.09	2246	1.93	0.44
rs4770121	221150	C13orf3	13	20630094	1.19E-02	1858	3.42E-02	Up	2.12	5597	1.93	0.15

rs4910164	10335	MRVI1	11	10628673	1.19E-02	1859	6.84E-13	Down	7.18	540	1.93	1.22
rs9386049	9749	PHACTR2	6	144171782	1.19E-02	1860	5.11E-11	Down	6.57	714	1.92	1.03
rs11678252	274	BIN1	2	127567927	1.19E-02	1861	1.62E-17	Down	8.52	288	1.92	1.68
rs13393813	51665	ASB1	2	239101438	1.19E-02	1862	2.19E-07	Down	5.18	1385	1.92	0.67
rs1018124	54578	UGT1A6	2	234458118	1.19E-02	1863	2.56E-06	Up	4.70	1705	1.92	0.56
rs1018124	54657	UGT1A4	2	234458118	1.19E-02	1864	4.64E-02	Up	1.99	5904	1.92	0.13
rs1018124	54658	UGT1A1	2	234458118	1.19E-02	1865	3.54E-01	Up	0.93	9628	1.92	0.05
rs1018124	54659	UGT1A3	2	234458118	1.19E-02	1866	6.53E-01	Up	0.45	11786	1.92	0.02
rs1018124	54600	UGT1A9	2	234458118	1.19E-02	1867	7.09E-01	Up	0.37	12125	1.92	0.01
rs12025785	829	CAPZA1	1	112917409	1.19E-02	1868	6.26E-07	Up	4.98	1510	1.92	0.62
rs12025785	4343	MOV10	1	112917409	1.19E-02	1869	2.18E-05	Up	4.25	2103	1.92	0.47
rs854462	6362	CCL18	17	31410203	1.19E-02	1870	8.57E-11	Up	6.49	751	1.92	1.01
rs16946821	58472	SQRDL	15	43753393	1.20E-02	1871	5.77E-08	Down	5.43	1236	1.92	0.72
rs3803	2624	GATA2	3	129682078	1.20E-02	1872	1.38E-07	Up	5.27	1331	1.92	0.69
rs492738	26279	PLA2G2D	1	20175167	1.20E-02	1873	6.75E-01	Up	0.42	11917	1.92	0.02
rs9654570	8871	SYNJ2	6	158358019	1.20E-02	1874	1.33E-08	Up	5.66	1091	1.92	0.79
rs7735198	30820	KCNIP1	5	169931984	1.20E-02	1875	1.57E-01	Down	1.41	7773	1.92	0.08
rs3748303	85004	RERG	12	15196428	1.20E-02	1876	2.09E-01	Down	1.26	8340	1.92	0.07
rs6792496	285313	IGSF10	3	152642019	1.20E-02	1877	7.33E-02	Up	1.79	6491	1.92	0.11
rs6792496	116931	MED12L	3	152642019	1.20E-02	1878	7.35E-01	Up	0.34	12283	1.92	0.01
rs7463863	203062	TSNARE1	8	143300073	1.20E-02	1879	7.17E-01	Down	0.36	12182	1.92	0.01
rs1877250	57194	ATP10A	15	23673136	1.20E-02	1880	7.38E-01	Down	0.33	12303	1.92	0.01
rs8046668	23568	ARL2BP	16	55854965	1.20E-02	1881	9.47E-08	Down	5.34	1292	1.92	0.70
rs7812327	444	ASPH	8	62716966	1.20E-02	1882	5.93E-05	Down	4.02	2329	1.92	0.42
rs5007291	164284	APCDD1L	20	56465163	1.21E-02	1883	2.30E-03	Down	3.05	3629	1.92	0.26
rs6503080	242	ALOX12B	17	7944237	1.21E-02	1884	4.96E-01	Up	0.68	10687	1.92	0.03
rs6503080	59344	ALOXE3	17	7944237	1.21E-02	1885	6.78E-01	Down	0.42	11930	1.92	0.02
rs10089145	9108	MTMR7	8	17281979	1.21E-02	1886	7.92E-02	Up	1.76	6618	1.92	0.11
rs13317985	257068	PLCXD2	3	112938864	1.21E-02	1887	7.79E-01	Down	0.28	12543	1.92	0.01
rs7128926	3784	KCNQ1	11	2653320	1.21E-02	1888	1.30E-03	Up	3.22	3322	1.92	0.29
rs3897137	3134	HLA-F	6	29806029	1.21E-02	1889	4.43E-01	Up	0.77	10285	1.92	0.04
rs2592980	80854	SETD7	4	140799750	1.21E-02	1890	8.48E-01	Down	0.19	12943	1.92	0.01
rs1560834	83879	CDCA7	2	174038179	1.21E-02	1891	3.80E-04	Down	3.55	2862	1.92	0.34
rs928939	2120	ETV6	12	11882934	1.21E-02	1892	2.86E-14	Down	7.60	439	1.92	1.35
rs2257020	23265	EXOC7	17	71588010	1.22E-02	1893	4.03E-04	Up	3.54	2889	1.92	0.34
rs2257020	6730	SRP68	17	71588010	1.22E-02	1894	2.72E-01	Down	1.10	8968	1.92	0.06
rs2257020	8811	GALR2	17	71588010	1.22E-02	1895	5.95E-01	Down	0.53	11402	1.92	0.02
rs10888046	55160	ARHGEF10L	1	17724424	1.22E-02	1896	5.71E-01	Up	0.57	11253	1.92	0.02
rs295302	5922	RASA2	3	142821695	1.22E-02	1897	1.76E-01	Up	1.35	7978	1.91	0.08
rs2833752	54069	C21orf45	21	32565727	1.22E-02	1898	2.94E-03	Up	2.97	3768	1.91	0.25
rs6467883	781	CACNA2D1	7	81346910	1.22E-02	1899	2.78E-02	Down	2.20	5386	1.91	0.16
rs11938463	3954	LETM1	4	1801653	1.22E-02	1900	3.31E-04	Up	3.59	2812	1.91	0.35
rs1843933	84433	CARD11	7	2804433	1.22E-02	1901	1.15E-01	Down	1.58	7210	1.91	0.09
rs10829917	256536	TCERG1L	10	132823689	1.22E-02	1902	3.53E-01	Down	0.93	9616	1.91	0.05
rs17001239	58494	JAM2	21	25924248	1.22E-02	1903	1.67E-01	Down	1.38	7884	1.91	0.08
rs10738395	6619	SNAPC3	9	15419539	1.22E-02	1904	8.70E-01	Down	0.16	13067	1.91	0.01
rs2152555	64388	GREM2	1	236985777	1.22E-02	1905	5.28E-05	Up	4.04	2303	1.91	0.43
rs17494172	8034	SLC25A16	9	72048638	1.22E-02	1906	4.86E-06	Up	4.57	1809	1.91	0.53
rs17494172	5047	PAEP	9	72048638	1.22E-02	1907	2.57E-01	Down	1.13	8837	1.91	0.06
rs17494172	9615	GDA	9	72048638	1.22E-02	1908	4.23E-01	Up	0.80	10143	1.91	0.04
rs781994	60682	SMAP1	6	71414885	1.22E-02	1909	5.31E-01	Up	0.63	10949	1.91	0.03
rs4131935	5139	PDE3A	12	20632200	1.22E-02	1910	2.20E-01	Up	1.23	8455	1.91	0.07
rs2070739	79022	TMEM106C	12	46654243	1.23E-02	1911	8.00E-08	Up	5.37	1274	1.91	0.71
rs2070739	1280	COL2A1	12	46654243	1.23E-02	1912	6.64E-07	Up	4.97	1515	1.91	0.62
rs12288048	22992	FBXL11	11	66768341	1.23E-02	1913	6.79E-03	Down	2.71	4240	1.91	0.22
rs9534013	26747	NUFIP1	13	44441588	1.23E-02	1914	7.95E-01	Up	0.26	12638	1.91	0.01
rs1675955	55341	LSG1	3	195835172	1.23E-02	1915	7.15E-01	Down	0.36	12162	1.91	0.01
rs11800619	80003	PCNXL2	1	229682448	1.23E-02	1916	1.02E-02	Up	2.57	4521	1.91	0.20
rs12451246	23108	GARNL4	17	2788345	1.23E-02	1917	1.40E-12	Up	7.08	565	1.91	1.19
rs3744064	6397	SEC14L1	17	72722803	1.23E-02	1918	2.25E-01	Up	1.21	8503	1.91	0.06
rs31309	814	CAMK4	5	110850399	1.23E-02	1919	3.90E-01	Down	0.86	9919	1.91	0.04
rs9173	9796	PHYHIP	8	22164556	1.23E-02	1920	1.73E-06	Down	4.78	1648	1.91	0.58
rs9173	661	POLR3D	8	22164556	1.23E-02	1921	3.21E-01	Up	0.99	9371	1.91	0.05
rs867901	23022	PALLD	4	170137184	1.23E-02	1922	5.87E-19	Down	8.89	231	1.91	1.82
rs208294	5027	P2RX7	12	120062973	1.23E-02	1923	2.87E-01	Up	1.07	9091	1.91	0.05
rs17752325	2044	EPHA5	4	66074122	1.23E-02	1924	2.20E-01	Down	1.23	8452	1.91	0.07
rs8500	957	ENTPD5	14	73498198	1.23E-02	1925	1.36E-09	Up	6.06	914	1.91	0.89
rs2236527	26164	GTPBP5	20	60201892	1.23E-02	1926	1.08E-01	Down	1.61	7122	1.91	0.10
rs10800913	2331	FMOD	1	200047550	1.24E-02	1927	3.41E-08	Up	5.52	1183	1.91	0.75
rs12694884	130560	SPATA3	2	231700791	1.24E-02	1928	9.48E-01	Up	0.07	13535	1.91	0.00
rs500214	9229	DLGAP1	18	3559834	1.24E-02	1929	6.10E-01	Up	0.51	11501	1.91	0.02
rs662279	871	SERPINH1	11	74939444	1.24E-02	1930	4.90E-02	Down	1.97	5970	1.91	0.13
rs7333340	113622	ADPRHL1	13	113168963	1.24E-02	1931	5.18E-03	Up	2.80	4091	1.91	0.23
rs17272376	117248	GALNTL2	3	16230283	1.24E-02	1932	8.70E-01	Down	0.16	13063	1.91	0.01
rs8014025	5890	RAD51L1	14	67932517	1.24E-02	1933	1.77E-01	Down	1.35	7982	1.91	0.08
rs8107642	4125	MAN2B1	19	12627370	1.24E-02	1934	4.76E-01	Up	0.71	10544	1.91	0.03
rs1156545	10584	COLEC10	8	120149873	1.24E-02	1935	1.97E-01	Up	1.29	8228	1.91	0.07
rs10864777	189	AGXT	1	227174798	1.24E-02	1936	2.67E-01	Down	1.11	8928	1.91	0.06
rs10864777	183	AGT	1	227174798	1.24E-02	1937	4.82E-01	Up	0.70	10589	1.91	0.03
rs10864777	10753	CAPN9	1	227174798	1.24E-02	1938	5.09E-01	Up	0.66	10775	1.91	0.03
rs7164048	2175	FANCA	15	78244221	1.24E-02	1939	2.52E-05	Up	4.21	2133	1.90	0.46

rs7164048	2184	FAH	15	78244221	1.24E-02	1940	1.22E-01	Up	1.55	7327	1.90	0.09
rs2834243	6453	ITSN1	21	33956121	1.25E-02	1941	5.60E-16	Down	8.10	350	1.90	1.53
rs2102471	54453	RIN2	20	19836736	1.25E-02	1942	8.77E-04	Down	3.33	3167	1.90	0.31
rs1808891	51444	RNF138	18	27969216	1.25E-02	1943	1.60E-02	Up	2.41	4886	1.90	0.18
rs4825596	139818	DOCK11		117524529	1.25E-02	1944	3.09E-01	Down	1.02	9263	1.90	0.05
rs408988	404550	C16orf74	16	84327221	1.25E-02	1945	4.35E-03	Down	2.85	3981	1.90	0.24
rs7751456	54511	HMGCLL1	6	55402779	1.25E-02	1946	2.11E-01	Down	1.25	8356	1.90	0.07
rs11624171	283601	C14orf70	14	100181400	1.25E-02	1947	1.45E-01	Up	1.46	7627	1.90	0.08
rs12478941	4148	MATN3	2	20140286	1.26E-02	1948	6.40E-01	Down	0.47	11704	1.90	0.02
rs1078849	131177	FAM3D	3	58598384	1.26E-02	1949	7.12E-04	Up	3.38	3085	1.90	0.31
rs13020302	60482	SLC5A7	2	108058228	1.26E-02	1950	9.56E-01	Up	0.06	13590	1.90	0.00
rs7219624	6909	TBX2	17	56814946	1.26E-02	1951	2.52E-06	Down	4.71	1702	1.90	0.56
rs6424914	23179	RGL1	1	180545515	1.26E-02	1952	3.15E-06	Down	4.66	1749	1.90	0.55
rs7175791	204219	LASS3	15	98866520	1.26E-02	1953	7.85E-01	Down	0.27	12577	1.90	0.01
rs12763964	8879	SGPL1	10	72272897	1.27E-02	1954	3.52E-01	Up	0.93	9609	1.90	0.05
rs11828	64802	NMNAT1	1	9921273	1.27E-02	1955	1.53E-01	Down	1.43	7713	1.90	0.08
rs11828	84328	LZIC	1	9921273	1.27E-02	1956	7.98E-01	Up	0.26	12658	1.90	0.01
rs6089151	140706	C20orf160	20	30080496	1.27E-02	1957	1.42E-01	Up	1.47	7600	1.90	0.08
rs871972	84077	C3orf20	3	14705649	1.27E-02	1958	6.16E-01	Down	0.50	11550	1.90	0.02
rs12794763	54663	WDR74	11	62382088	1.27E-02	1959	1.20E-01	Up	1.56	7294	1.90	0.09
rs12794763	6520	SLC3A2	11	62382088	1.27E-02	1960	2.25E-01	Down	1.21	8505	1.90	0.06
rs4353849	132884	EVC2	4	5756724	1.27E-02	1961	1.39E-02	Down	2.46	4749	1.90	0.19
rs9257802	81696	OR5V1	6	29451334	1.27E-02	1962	2.38E-01	Up	1.18	8642	1.90	0.06
rs9257802	81797	OR12D3	6	29451334	1.27E-02	1963	4.07E-01	Down	0.83	10029	1.90	0.04
rs710709	144453	BEST3	12	68388433	1.27E-02	1964	1.25E-01	Down	1.53	7376	1.90	0.09
rs6553160	339976	TRIML1	4	189437363	1.27E-02	1965	6.25E-01	Up	0.49	11613	1.90	0.02
rs2796077	200150	PLD5	1	238920909	1.27E-02	1966	8.36E-05	Down	3.93	2406	1.90	0.41
rs17528049	8476	CDC42BPA	1	223498391	1.27E-02	1967	2.97E-07	Down	5.13	1415	1.89	0.65
rs17677737	54504	CPVL	7	28958771	1.27E-02	1968	7.64E-05	Down	3.96	2377	1.89	0.41
rs17325547	10178	ODZ1		123513934	1.27E-02	1969	8.00E-01	Down	0.25	12673	1.89	0.01
rs980618	3603	IL16	15	79305264	1.28E-02	1970	8.12E-01	Up	0.24	12731	1.89	0.01
rs223892	6361	CCL17	16	55993654	1.28E-02	1971	2.92E-01	Down	1.05	9131	1.89	0.05
rs31323	23105	FSTL4	5	132859334	1.28E-02	1972	2.98E-01	Up	1.04	9181	1.89	0.05
rs8103218	84467	FBN3	19	8049114	1.28E-02	1973	9.10E-01	Down	0.11	13307	1.89	0.00
rs2824751	5651	PRSS7	21	18635692	1.28E-02	1974	3.28E-02	Up	2.13	5553	1.89	0.15
rs12467466	1058	CENPA	2	26921129	1.28E-02	1975	2.64E-03	Up	3.01	3717	1.89	0.26
rs12467466	54978	C2orf18	2	26921129	1.28E-02	1976	4.89E-01	Down	0.69	10646	1.89	0.03
rs13345270	163051	ZNF709	19	12423702	1.28E-02	1977	1.96E-02	Down	2.33	5050	1.89	0.17
rs1884709	27131	SNX5	20	17873870	1.28E-02	1978	9.99E-03	Up	2.58	4510	1.89	0.20
rs740851	9918	NCAPD2	12	6508611	1.28E-02	1979	2.67E-03	Up	3.00	3724	1.89	0.26
rs268627	51110	LACTB2	8	71723595	1.29E-02	1980	3.16E-03	Up	2.95	3806	1.89	0.25
rs2238023	775	CACNA1C	12	2072798	1.29E-02	1981	3.23E-04	Down	3.60	2801	1.89	0.35
rs6721186	50649	ARHGEF4	2	131497835	1.29E-02	1982	2.79E-03	Down	2.99	3749	1.89	0.26
rs2965042	221938	MMD2	7	4746214	1.29E-02	1983	4.37E-01	Down	0.78	10250	1.89	0.04
rs2286260	221833	SP8	7	20594513	1.30E-02	1984	3.24E-02	Down	2.14	5538	1.89	0.15
rs17106475	81619	TSPAN14	10	82274286	1.30E-02	1985	1.73E-02	Up	2.38	4954	1.89	0.18
rs10917300	2048	EPHB2	1	22863504	1.30E-02	1986	3.74E-01	Up	0.89	9794	1.89	0.04
rs9964724	56853	BRUNOL4	18	33413122	1.31E-02	1987	6.28E-01	Down	0.48	11639	1.88	0.02
rs10515932	10170	DHRS9	2	169779692	1.31E-02	1988	1.66E-01	Up	1.39	7875	1.88	0.08
rs16980298	9947	MAGEC1		140731983	1.31E-02	1989	3.94E-01	Up	0.85	9941	1.88	0.04
rs4943605	341640	FREM2	13	38298169	1.31E-02	1990	5.52E-01	Down	0.60	11089	1.88	0.03
rs2043188	130589	GALM	2	38840632	1.31E-02	1991	5.92E-01	Down	0.54	11384	1.88	0.02
rs11977943	222255	ATXN7L1	7	104988493	1.31E-02	1992	2.48E-04	Down	3.66	2708	1.88	0.36
rs4801298	85569	GALP	19	61398860	1.32E-02	1993	1.61E-01	Down	1.40	7828	1.88	0.08
rs387976	5819	PVRL2	19	50070900	1.32E-02	1994	1.29E-11	Up	6.81	639	1.88	1.09
rs387976	10452	TOMM40	19	50070900	1.32E-02	1995	3.21E-04	Up	3.60	2798	1.88	0.35
rs11852452	4948	OCA2	15	25754287	1.32E-02	1996	6.19E-01	Down	0.50	11575	1.88	0.02
rs5749440	25793	FBXO7	22	31177276	1.32E-02	1997	2.61E-02	Down	2.22	5325	1.88	0.16
rs4135221	7295	TXN	9	110086792	1.32E-02	1998	2.79E-09	Up	5.95	957	1.88	0.86
rs16972308	30844	EHD4	15	40025283	1.32E-02	1999	1.80E-02	Down	2.37	4986	1.88	0.17
rs15783	143662	MUC15	11	26543377	1.32E-02	2000	9.77E-04	Down	3.30	3209	1.88	0.30
rs2110658	130733	TMEM178	2	39841928	1.32E-02	2001	7.65E-03	Up	2.67	4318	1.88	0.21
rs1039898	3623	INHA	2	220255708	1.32E-02	2002	8.45E-02	Up	1.72	6711	1.88	0.11
rs1039898	23363	OBSL1	2	220255708	1.32E-02	2003	2.38E-01	Down	1.18	8636	1.88	0.06
rs6459928	7434	VIPR2	7	158428045	1.32E-02	2004	9.11E-04	Down	3.32	3179	1.88	0.30
rs11203077	3437	IFIT3	10	91087065	1.32E-02	2005	8.34E-03	Down	2.64	4371	1.88	0.21
rs11059910	121260	SLC15A4	12	127796599	1.33E-02	2006	1.03E-02	Down	2.57	4528	1.88	0.20
rs4470262	90011	KIR3DX1	19	59728473	1.33E-02	2007	6.86E-01	Down	0.40	11984	1.88	0.02
rs1957982	6252	RTN1	14	59329346	1.33E-02	2008	3.13E-03	Up	2.95	3798	1.88	0.25
rs10869915	23230	VPS13A	9	77084283	1.33E-02	2009	4.00E-08	Up	5.50	1201	1.88	0.74
rs10132476	256369	C14orf48	14	93538044	1.33E-02	2010	3.77E-01	Up	0.88	9822	1.88	0.04
rs12514417	501	ALDH7A1	5	125915614	1.33E-02	2011	2.09E-02	Up	2.31	5109	1.88	0.17
rs16919597	51606	ATP6V1H	8	54933787	1.33E-02	2012	9.21E-03	Down	2.60	4446	1.88	0.20
rs16919597	8601	RGS20	8	54933787	1.33E-02	2013	5.71E-02	Up	1.90	6160	1.88	0.12
rs1631313	89795	NAV3	12	76818576	1.33E-02	2014	5.51E-02	Down	1.92	6115	1.88	0.13
rs10993711	6850	SYK	9	90691292	1.33E-02	2015	1.18E-06	Down	4.86	1598	1.87	0.59
rs11617401	55647	RAB20	13	109996460	1.33E-02	2016	1.12E-02	Up	2.54	4588	1.87	0.19
rs10736660	347853	TBX10	11	67176431	1.34E-02	2017	2.99E-02	Up	2.17	5460	1.87	0.15
rs10736660	222	ALDH3B2	11	67176431	1.34E-02	2018	6.37E-02	Up	1.85	6318	1.87	0.12
rs10736660	91703	ACY3	11	67176431	1.34E-02	2019	9.26E-02	Down	1.68	6875	1.87	0.10
rs7080052	9937	DCLRE1A	10	115623227	1.34E-02	2020	1.78E-05	Up	4.29	2070	1.87	0.47

rs7080052	374354	NHLRC2	10	115623227	1.34E-02	2021	1.61E-02	Up	2.41	4892	1.87	0.18
rs2195611	5136	PDE1A	2	183082073	1.34E-02	2022	2.22E-04	Up	3.69	2667	1.87	0.37
rs17169196	6119	RPA3	7	7454371	1.34E-02	2023	4.65E-03	Up	2.83	4017	1.87	0.23
rs2274328	765	CA6	1	8943710	1.34E-02	2024	1.01E-01	Down	1.64	7014	1.87	0.10
rs1925685	89874	SLC25A21	14	36366995	1.34E-02	2025	7.03E-02	Up	1.81	6435	1.87	0.12
rs17058153	117531	TMC1	9	72636713	1.34E-02	2026	2.84E-02	Down	2.19	5406	1.87	0.15
rs2292243	4799	NFX1	9	33318812	1.34E-02	2027	7.17E-01	Down	0.36	12179	1.87	0.01
rs11012549	387638	C10orf113	10	21486545	1.34E-02	2028	9.70E-01	Down	0.04	13698	1.87	0.00
rs750582	56477	CCL28	5	43466030	1.34E-02	2029	1.05E-01	Up	1.62	7084	1.87	0.10
rs3805739	4001	LMNB1	5	126194090	1.34E-02	2030	6.80E-05	Up	3.98	2356	1.87	0.42
rs9293145	1008	CDH10	5	24645204	1.35E-02	2031	9.87E-01	Up	0.02	13821	1.87	0.00
rs1687291	152273	FGD5	3	14887406	1.35E-02	2032	2.57E-02	Down	2.23	5311	1.87	0.16
rs7251224	126068	ZNF441	19	11731583	1.35E-02	2033	2.01E-02	Down	2.32	5082	1.87	0.17
rs4937342	2113	ETS1	11	127903519	1.35E-02	2034	9.57E-01	Up	0.05	13593	1.87	0.00
rs2835482	3141	HLCS	21	37135917	1.35E-02	2035	2.95E-05	Up	4.18	2167	1.87	0.45
rs859448	7143	TNR	1	172099905	1.36E-02	2036	5.88E-01	Down	0.54	11357	1.87	0.02
rs2437770	6383	SDC2	8	97574721	1.36E-02	2037	7.46E-19	Down	8.87	235	1.87	1.81
rs5955870	256643	CXorf23		19749207	1.36E-02	2038	5.40E-08	Up	5.44	1228	1.87	0.73
rs12078444	83872	HMCN1	1	182586946	1.36E-02	2039	5.40E-01	Up	0.61	11010	1.87	0.03
rs2149823	79971	GPR177	1	68380313	1.36E-02	2040	1.18E-03	Up	3.24	3282	1.87	0.29
rs999985	143884	CWF19L2	11	106697467	1.36E-02	2041	6.49E-01	Up	0.45	11769	1.87	0.02
rs2159963	1911	PHC1	12	8955888	1.36E-02	2042	5.96E-02	Down	1.88	6231	1.87	0.12
rs4793987	284076	TTLL6	17	44210836	1.36E-02	2043	7.54E-02	Down	1.78	6534	1.87	0.11
rs826423	9467	SH3BP5	3	15316605	1.36E-02	2044	5.33E-02	Down	1.93	6071	1.87	0.13
rs11596235	81603	TRIM8	10	104381024	1.36E-02	2045	2.57E-01	Down	1.13	8828	1.87	0.06
rs11596235	51684	SUFU	10	104381024	1.36E-02	2046	6.64E-01	Down	0.43	11849	1.87	0.02
rs1817023	23414	ZFPM2	8	106698141	1.37E-02	2047	4.94E-02	Down	1.97	5982	1.86	0.13
rs6988340	114	ADCY8	8	132037651	1.37E-02	2048	8.52E-01	Down	0.19	12970	1.86	0.01
rs972984	5529	PPP2R5E	14	63091961	1.37E-02	2049	6.90E-04	Down	3.39	3071	1.86	0.32
rs10521607	55841	WWC3		9842191	1.37E-02	2050	3.32E-09	Down	5.91	983	1.86	0.85
rs1877330	10018	BCL2L11	2	111622993	1.37E-02	2051	1.23E-02	Up	2.50	4650	1.86	0.19
rs1057050	4791	NFKB2	10	104132284	1.37E-02	2052	3.85E-01	Down	0.87	9882	1.86	0.04
rs1057050	8729	GBF1	10	104132284	1.37E-02	2053	5.62E-01	Up	0.58	11174	1.86	0.03
rs2577355	2674	GFRA1	10	118014534	1.37E-02	2054	3.97E-01	Down	0.85	9966	1.86	0.04
rs2957489	25841	ABTB2	11	34148668	1.37E-02	2055	2.52E-03	Down	3.02	3694	1.86	0.26
rs541731	10253	SPRY2	13	79817713	1.37E-02	2056	2.40E-03	Down	3.04	3661	1.86	0.26
rs6492679	10082	GPC6	13	93225054	1.37E-02	2057	1.88E-03	Down	3.11	3500	1.86	0.27
rs1852755	10418	SPON1	11	13953262	1.37E-02	2058	1.89E-18	Down	8.76	254	1.86	1.77
rs9585363	5095	PCCA	13	99592603	1.38E-02	2059	1.09E-02	Up	2.55	4567	1.86	0.20
rs247811	9914	ATP2C2	16	83004558	1.38E-02	2060	7.27E-02	Down	1.79	6479	1.86	0.11
rs17400787	255631	COL24A1	1	85938373	1.38E-02	2061	7.06E-01	Down	0.38	12102	1.86	0.02
rs10513660	2122	EVI1	3	170364032	1.38E-02	2062	3.06E-06	Down	4.67	1744	1.86	0.55
rs1953866	54331	GNG2	14	51387587	1.38E-02	2063	2.32E-01	Down	1.20	8581	1.86	0.06
rs11744822	115548	FCHO2	5	72316160	1.38E-02	2064	3.18E-04	Up	3.60	2796	1.86	0.35
rs4972828	3655	ITGA6	2	173100671	1.38E-02	2065	8.73E-01	Down	0.16	13082	1.86	0.01
rs10033399	51138	COPS4	4	84296350	1.38E-02	2066	2.43E-03	Down	3.03	3667	1.86	0.26
rs10033399	132660	LIN54	4	84296350	1.38E-02	2067	5.38E-01	Up	0.62	11000	1.86	0.03
rs8258	22897	CEP164	11	116788886	1.38E-02	2068	1.86E-01	Down	1.32	8097	1.86	0.07
rs4750100	79746	ECHDC3	10	11835607	1.38E-02	2069	7.20E-03	Down	2.69	4276	1.86	0.21
rs2839401	63977	PRDM15	21	42175148	1.38E-02	2070	5.83E-01	Up	0.55	11330	1.86	0.02
rs7511795	163351	GBP6	1	89554480	1.38E-02	2071	9.84E-01	Up	0.02	13802	1.86	0.00
rs12145292	6518	SLC2A5	1	9052862	1.38E-02	2072	1.53E-16	Down	8.25	331	1.86	1.58
rs891207	60680	BRUNOL5	19	3245279	1.38E-02	2073	9.27E-01	Up	0.09	13410	1.86	0.00
rs1064891	5209	PFKFB3	10	6316580	1.38E-02	2074	3.33E-01	Down	0.97	9463	1.86	0.05
rs10762503	54541	DDIT4	10	73691564	1.39E-02	2075	1.26E-07	Down	5.28	1316	1.86	0.69
rs6916539	57510	XPO5	6	43668810	1.39E-02	2076	3.28E-05	Up	4.15	2185	1.86	0.45
rs6916539	5429	POLH	6	43668810	1.39E-02	2077	7.32E-01	Down	0.34	12266	1.86	0.01
rs6581146	3798	KIF5A	12	56232744	1.39E-02	2078	7.59E-02	Up	1.78	6546	1.86	0.11
rs10936199	57560	IFT80	3	161477647	1.39E-02	2079	2.72E-03	Up	3.00	3732	1.86	0.26
rs7223320	79777	ACBD4	17	40557324	1.39E-02	2080	8.52E-01	Down	0.19	12969	1.86	0.01
rs7223320	113026	PLCD3	17	40557324	1.39E-02	2081	9.31E-01	Up	0.09	13444	1.86	0.00
rs7549105	93474	ZNF670	1	243531097	1.39E-02	2082	4.67E-03	Up	2.83	4018	1.86	0.23
rs1993477	51366	UBR5	8	103337242	1.40E-02	2083	1.68E-03	Up	3.14	3441	1.86	0.28
rs1993477	50484	RRM2B	8	103337242	1.40E-02	2084	8.79E-01	Up	0.15	13116	1.86	0.01
rs888298	55062	WIPI1	17	63955930	1.40E-02	2085	2.78E-01	Up	1.09	9014	1.86	0.06
rs2444889	4147	MATN2	8	98977374	1.40E-02	2086	1.16E-04	Down	3.85	2490	1.85	0.39
rs7250785	58491	ZNF71	19	61818540	1.40E-02	2087	7.98E-01	Down	0.26	12664	1.85	0.01
rs3826555	79058	ASPSCR1	17	77517721	1.40E-02	2088	2.60E-01	Up	1.13	8861	1.85	0.06
rs5924560	55787	CXorf15		16626398	1.40E-02	2089	5.02E-01	Down	0.67	10729	1.85	0.03
rs7164726	145773	FAM81A	15	57502958	1.40E-02	2090	1.83E-01	Up	1.33	8056	1.85	0.07
rs6021460	57167	SALL4	20	49860267	1.40E-02	2091	3.61E-02	Down	2.10	5652	1.85	0.14
rs2166374	26064	RAI14	5	34778433	1.40E-02	2092	8.35E-03	Down	2.64	4372	1.85	0.21
rs6602159	6251	RSU1	10	16872567	1.40E-02	2093	1.11E-01	Down	1.60	7155	1.85	0.10
rs2155053	4317	MMP8	11	102109817	1.40E-02	2094	9.17E-01	Down	0.10	13358	1.85	0.00
rs6495469	54469	ZFAND6	15	78122318	1.41E-02	2095	4.65E-01	Down	0.73	10463	1.85	0.03
rs6832439	56606	SLC2A9	4	9600588	1.41E-02	2096	7.96E-06	Down	4.47	1902	1.85	0.51
rs10846824	65985	AACS	12	124098251	1.41E-02	2097	7.38E-02	Up	1.79	6500	1.85	0.11
rs9792498	1318	SLC31A2	9	112980087	1.41E-02	2098	5.69E-01	Up	0.57	11231	1.85	0.02
rs2286036	23085	ERC1	12	1242089	1.41E-02	2099	2.63E-07	Down	5.15	1401	1.85	0.66
rs2241954	2775	GNAO1	16	54936479	1.41E-02	2100	4.07E-01	Down	0.83	10032	1.85	0.04
rs9374013	28962	OSTM1	6	108488040	1.41E-02	2101	3.40E-08	Down	5.52	1178	1.85	0.75

rs11755463	135458	HUS1B	6	610448	1.41E-02	2102	3.01E-01	Up	1.03	9199	1.85	0.05
rs10521531	5063	PAK3		110206792	1.42E-02	2103	1.43E-02	Down	2.45	4774	1.85	0.18
rs10521531	8874	ARHGEF7		110206792	1.42E-02	2104	3.66E-02	Up	2.09	5667	1.85	0.14
rs4129460	257397	MAP3K7IP3		30711055	1.42E-02	2105	1.68E-01	Up	1.38	7903	1.85	0.08
rs12071303	54873	PALMD	1	99873449	1.42E-02	2106	3.45E-05	Down	4.14	2199	1.85	0.45
rs274074	115584	SLC5A11	16	24795955	1.42E-02	2107	4.97E-01	Down	0.68	10691	1.85	0.03
rs11994326	137868	SGCZ	8	14438596	1.42E-02	2108	6.54E-01	Down	0.45	11787	1.85	0.02
rs10006867	3660	IRF2	4	185702122	1.42E-02	2109	2.20E-06	Down	4.73	1683	1.85	0.57
rs12306837	8798	DYRK4	12	4550729	1.42E-02	2110	2.78E-09	Down	5.94	962	1.85	0.86
rs17124345	148423	C1orf52	1	85438124	1.42E-02	2111	1.31E-04	Up	3.82	2514	1.85	0.39
rs17124345	8915	BCL10	1	85438124	1.42E-02	2112	8.81E-03	Up	2.62	4417	1.85	0.21
rs3861950	7292	TNFSF4	1	169887949	1.42E-02	2113	9.91E-01	Down	0.01	13847	1.85	0.00
rs877818	1947	EFNB1		67836528	1.43E-02	2114	1.67E-05	Down	4.30	2057	1.84	0.48
rs3095314	170679	PSORS1C1	6	31197610	1.43E-02	2115	1.58E-01	Down	1.41	7777	1.84	0.08
rs3095314	1041	CDSN	6	31197610	1.43E-02	2116	2.10E-01	Down	1.25	8349	1.84	0.07
rs3095314	29113	C6orf15	6	31197610	1.43E-02	2117	6.12E-01	Down	0.51	11523	1.84	0.02
rs374988	2158	F9		138356735	1.43E-02	2118	1.94E-01	Up	1.30	8198	1.84	0.07
rs533486	64816	CYP3A43	7	99085345	1.43E-02	2119	3.10E-04	Down	3.61	2788	1.84	0.35
rs761602	23576	DDAH1	1	85563329	1.43E-02	2120	9.65E-01	Down	0.04	13647	1.84	0.00
rs4468807	3635	INPP5D	2	233892954	1.44E-02	2121	8.81E-04	Down	3.33	3169	1.84	0.31
rs4788186	9961	MVP	16	29748726	1.44E-02	2122	7.06E-04	Down	3.39	3079	1.84	0.32
rs4309437	146849	CCDC42	17	8588828	1.44E-02	2123	1.39E-01	Down	1.48	7567	1.84	0.09
rs4788186	79447	C16orf53	16	29748726	1.44E-02	2124	3.59E-01	Down	0.92	9668	1.84	0.04
rs1163448	55211	DPPA4	3	110540087	1.44E-02	2125	3.58E-01	Up	0.92	9664	1.84	0.04
rs7787525	89122	TRIM4	7	99138311	1.44E-02	2126	8.33E-02	Down	1.73	6691	1.84	0.11
rs2412522	26511	CHIC2	4	54779471	1.44E-02	2127	1.51E-02	Up	2.43	4817	1.84	0.18
rs1000858	2995	GYPC	2	127175551	1.44E-02	2128	3.93E-05	Down	4.11	2227	1.84	0.44
rs2838704	754	PTTG1IP	21	45088803	1.44E-02	2129	6.28E-02	Up	1.86	6294	1.84	0.12
rs10494744	343450	KCNT2	1	193274224	1.44E-02	2130	1.65E-01	Down	1.39	7868	1.84	0.08
rs4943417	5994	RFXAP	13	36277373	1.45E-02	2131	6.55E-02	Up	1.84	6352	1.84	0.12
rs3027449	4129	MAOB		43405471	1.45E-02	2132	1.50E-14	Down	7.69	421	1.84	1.38
rs250103	2246	FGF1	5	141966795	1.45E-02	2133	2.21E-01	Up	1.22	8478	1.84	0.07
rs282268	5270	SERPINE2	2	224745681	1.45E-02	2134	1.98E-02	Down	2.33	5059	1.84	0.17
rs39937	2201	FBN2	5	127652527	1.45E-02	2135	1.40E-01	Down	1.48	7571	1.84	0.09
rs910901	92667	C20orf72	20	17881686	1.45E-02	2136	1.35E-02	Up	2.47	4726	1.84	0.19
rs2352745	586	BCAT1	12	24988389	1.45E-02	2137	1.02E-02	Up	2.57	4524	1.84	0.20
rs5940942	53336	CPXCR1		87823394	1.45E-02	2138	3.61E-01	Up	0.91	9689	1.84	0.04
rs16828139	6375	XCL1	1	165290432	1.45E-02	2139	5.50E-03	Up	2.78	4129	1.84	0.23
rs6958427	55536	CDCA7L	7	21726194	1.46E-02	2140	1.84E-03	Down	3.11	3488	1.84	0.27
rs2013566	4915	NTRK2	9	84655852	1.46E-02	2141	1.34E-01	Down	1.50	7494	1.84	0.09
rs11720239	8607	RUVBL1	3	129300653	1.46E-02	2142	1.05E-15	Up	8.02	358	1.84	1.50
rs3805486	5562	PRKAA1	5	40831802	1.46E-02	2143	4.73E-02	Up	1.98	5929	1.84	0.13
rs12189748	84830	C6orf105	6	11845356	1.46E-02	2144	5.74E-02	Down	1.90	6170	1.84	0.12
rs7384927	2692	GHRHR	7	30773340	1.46E-02	2145	1.68E-01	Up	1.38	7892	1.83	0.08
rs3792211	2557	GABRA4	4	46835423	1.46E-02	2146	5.27E-01	Up	0.63	10920	1.83	0.03
rs13275584	10395	DLC1	8	13092155	1.46E-02	2147	2.17E-02	Down	2.30	5150	1.83	0.17
rs13275584	8655	DYNLL1	8	13092155	1.46E-02	2148	2.51E-01	Down	1.15	8771	1.83	0.06
rs10212632	152789	JAKMIP1	4	6234169	1.46E-02	2149	1.92E-01	Down	1.30	8176	1.83	0.07
rs6555873	153745	FAM71B	5	156517097	1.46E-02	2150	7.42E-02	Up	1.79	6510	1.83	0.11
rs7146435	23508	TTC9	14	70221416	1.46E-02	2151	1.70E-06	Up	4.79	1646	1.83	0.58
rs1573736	4833	NME4	16	372752	1.47E-02	2152	1.50E-06	Up	4.81	1626	1.83	0.58
rs1573736	26063	DECR2	16	372752	1.47E-02	2153	3.81E-06	Up	4.62	1776	1.83	0.54
rs1573736	58986	TMEM8	16	372752	1.47E-02	2154	9.08E-04	Up	3.32	3176	1.83	0.30
rs1573736	10573	MRPL28	16	372752	1.47E-02	2155	9.87E-01	Down	0.02	13826	1.83	0.00
rs16914086	55357	TBC1D2	9	98068032	1.47E-02	2156	3.26E-04	Down	3.59	2806	1.83	0.35
rs611151	28960	DCPS	11	125693676	1.47E-02	2157	1.78E-01	Up	1.35	7997	1.83	0.07
rs10032220	11199	ANXA10	4	169383137	1.47E-02	2158	7.51E-01	Up	0.32	12382	1.83	0.01
rs7114018	7275	TUB	11	8046522	1.47E-02	2159	2.27E-01	Down	1.21	8533	1.83	0.06
rs6503190	9482	STX8	17	9096779	1.47E-02	2160	1.28E-01	Down	1.52	7416	1.83	0.09
rs3733026	1894	ECT2	3	173959715	1.47E-02	2161	2.48E-03	Up	3.03	3681	1.83	0.26
rs9470843	3096	HIVEP1	6	12152040	1.47E-02	2162	2.62E-07	Up	5.15	1400	1.83	0.66
rs17013810	9429	ABCG2	4	89365685	1.47E-02	2163	1.14E-05	Down	4.39	1975	1.83	0.49
rs17013810	5311	PKD2	4	89365685	1.47E-02	2164	2.11E-02	Down	2.31	5122	1.83	0.17
rs17013810	25865	PRKD2	4	89365685	1.47E-02	2165	1.05E-01	Up	1.62	7073	1.83	0.10
rs560083	10718	NRG3	10	84604824	1.48E-02	2166	5.84E-01	Up	0.55	11340	1.83	0.02
rs316779	29063	ZCCHC4	4	25010520	1.48E-02	2167	2.41E-03	Down	3.03	3663	1.83	0.26
rs7162639	10391	CORO2B	15	66807985	1.48E-02	2168	5.53E-03	Down	2.77	4133	1.83	0.23
rs622064	374407	DNAJB13	11	73350222	1.48E-02	2169	1.33E-02	Up	2.48	4708	1.83	0.19
rs17266652	23051	ZHX3	20	39352837	1.48E-02	2170	1.95E-02	Up	2.34	5046	1.83	0.17
rs7108356	55031	USP47	11	11859578	1.48E-02	2171	6.35E-02	Up	1.86	6311	1.83	0.12
rs3819721	6890	TAP1	6	32912776	1.48E-02	2172	6.99E-04	Up	3.39	3075	1.83	0.32
rs3819721	5696	PSMB8	6	32912776	1.48E-02	2173	1.06E-03	Down	3.27	3246	1.83	0.30
rs3819721	5698	PSMB9	6	32912776	1.48E-02	2174	2.94E-02	Down	2.18	5445	1.83	0.15
rs3819721	6891	TAP2	6	32912776	1.48E-02	2175	8.16E-02	Down	1.74	6661	1.83	0.11
rs3819721	266629	SEC14L3	6	32912776	1.48E-02	2176	9.91E-01	Up	0.01	13846	1.83	0.00
rs7876141	203562	TMEM31		102783861	1.48E-02	2177	2.63E-02	Down	2.22	5335	1.83	0.16
rs10876228	3164	NR4A1	12	50703829	1.48E-02	2178	2.51E-04	Up	3.66	2711	1.83	0.36
rs10876228	160622	GRASP	12	50703829	1.48E-02	2179	3.48E-03	Down	2.92	3854	1.83	0.25
rs7755068	26054	SENP6	6	76462420	1.49E-02	2180	5.70E-04	Up	3.45	3009	1.83	0.32
rs9657455	169044	COL22A1	8	139750307	1.49E-02	2181	1.12E-01	Down	1.59	7175	1.83	0.10
rs16900410	577	BAI3	6	69769215	1.49E-02	2182	2.55E-03	Down	3.02	3699	1.83	0.26

rs10064140	22987	SV2C	5	75598554	1.49E-02	2183	9.11E-02	Up	1.69	6847	1.83	0.10
rs1549931	84765	ZNF577	19	57095328	1.49E-02	2184	6.61E-11	Up	6.53	734	1.83	1.02
rs1549931	65251	ZNF649	19	57095328	1.49E-02	2185	7.30E-02	Up	1.79	6485	1.83	0.11
rs7724577	202374	STK32A	5	146651214	1.49E-02	2186	6.30E-01	Up	0.48	11644	1.83	0.02
rs1968871	79865	TREML2	6	41293408	1.49E-02	2187	8.86E-02	Up	1.70	6778	1.83	0.11
rs1968871	285852	TREML4	6	41293408	1.49E-02	2188	9.44E-01	Down	0.07	13521	1.83	0.00
rs988098	25842	ASF1A	6	119281925	1.49E-02	2189	3.56E-01	Up	0.92	9644	1.83	0.04
rs16850799	51094	ADIPOR1	1	199651508	1.50E-02	2190	5.23E-10	Up	6.21	861	1.83	0.93
rs1009437	284613	CYB561D1	1	109759375	1.50E-02	2191	4.93E-05	Up	4.06	2279	1.82	0.43
rs1009437	57463	AMIGO1	1	109759375	1.50E-02	2192	5.39E-01	Down	0.61	11007	1.82	0.03
rs328043	91368	CDKN2AIPNL	5	133773297	1.50E-02	2193	4.57E-01	Up	0.74	10400	1.82	0.03
rs7226358	9501	RPH3AL	17	65351	1.50E-02	2194	6.73E-03	Up	2.71	4236	1.82	0.22
rs6485700	4038	LRP4	11	46832509	1.50E-02	2195	5.10E-05	Down	4.05	2294	1.82	0.43
rs6485700	10699	CORIN	11	46832509	1.50E-02	2196	5.26E-02	Down	1.94	6051	1.82	0.13
rs12929558	23247	KIAA0556	16	27609632	1.50E-02	2197	5.58E-05	Up	4.03	2316	1.82	0.43
rs10132619	2954	GSTZ1	14	76875129	1.50E-02	2198	2.46E-05	Up	4.22	2125	1.82	0.46
rs613232	7779	SLC30A1	1	208158288	1.50E-02	2199	1.15E-01	Down	1.57	7222	1.82	0.09
rs10132619	29954	POMT2	14	76875129	1.50E-02	2200	9.34E-01	Down	0.08	13456	1.82	0.00
rs846964	55084	SOBP	6	108075806	1.50E-02	2201	5.80E-04	Down	3.44	3014	1.82	0.32
rs2140364	151790	WDR49	3	168701245	1.50E-02	2202	3.50E-01	Up	0.93	9587	1.82	0.05
rs16838083	2825	GPR1	2	206901530	1.51E-02	2203	4.03E-01	Up	0.84	10003	1.82	0.04
rs6694831	26508	HEYL	1	39764586	1.51E-02	2204	4.21E-13	Up	7.30	507	1.82	1.24
rs12163169	114904	C1QTNF6	22	35914305	1.51E-02	2205	7.07E-01	Down	0.38	12109	1.82	0.02
rs6721685	5147	PDE6D	2	232483251	1.51E-02	2206	1.69E-03	Up	3.14	3443	1.82	0.28
rs6721685	64708	COPS7B	2	232483251	1.51E-02	2207	2.39E-01	Up	1.18	8648	1.82	0.06
rs310574	54212	SNTG1	8	51601449	1.51E-02	2208	1.25E-01	Down	1.53	7375	1.82	0.09
rs1392524	5789	PTPRD	9	8388021	1.51E-02	2209	8.66E-05	Down	3.93	2414	1.82	0.41
rs8082866	3586	IL10	18	3415211	1.51E-02	2210	1.04E-01	Down	1.63	7059	1.82	0.10
rs9554498	6564	SLC15A1	13	98162009	1.51E-02	2211	1.80E-01	Down	1.34	8022	1.82	0.07
rs321776	79722	ANKRD55	5	55443299	1.51E-02	2212	5.44E-02	Down	1.92	6097	1.82	0.13
rs9854442	201595	STT3B	3	31629042	1.51E-02	2213	2.40E-01	Down	1.17	8658	1.82	0.06
rs1264583	56658	TRIM39	6	30401462	1.51E-02	2214	1.28E-01	Down	1.52	7430	1.82	0.09
rs1264583	79897	RPP21	6	30401462	1.51E-02	2215	4.98E-01	Down	0.68	10701	1.82	0.03
rs7684248	1519	CTSO	4	157217996	1.51E-02	2216	1.31E-09	Up	6.06	911	1.82	0.89
rs11902994	10190	TXNDC9	2	99423982	1.52E-02	2217	2.19E-08	Up	5.60	1132	1.82	0.77
rs11902994	9669	EIF5B	2	99423982	1.52E-02	2218	1.05E-02	Down	2.56	4549	1.82	0.20
rs12775064	119467	CLRN3	10	129559801	1.52E-02	2219	8.97E-02	Up	1.70	6802	1.82	0.10
rs892932	9686	VGLL4	3	11599211	1.52E-02	2220	8.92E-01	Down	0.14	13196	1.82	0.00
rs11569429	718	C3	19	6660074	1.52E-02	2221	7.89E-01	Up	0.27	12602	1.82	0.01
rs1652362	147463	ANKRD29	18	19430768	1.53E-02	2222	1.13E-02	Up	2.53	4597	1.82	0.19
rs1652362	4864	NPC1	18	19430768	1.53E-02	2223	7.57E-02	Down	1.78	6543	1.82	0.11
rs10861631	5992	RFX4	12	105461754	1.53E-02	2224	9.23E-01	Down	0.10	13391	1.82	0.00
rs6783990	55287	TMEM40	3	12772747	1.53E-02	2225	6.98E-02	Down	1.81	6421	1.82	0.12
rs3780289	9630	GNA14	9	77300465	1.53E-02	2226	3.18E-01	Down	1.00	9347	1.82	0.05
rs10518980	51496	CTDSPL2	15	42610066	1.53E-02	2227	1.53E-01	Up	1.43	7728	1.81	0.08
rs114266	51106	TFB1M	6	155744495	1.53E-02	2228	1.06E-01	Up	1.62	7091	1.81	0.10
rs7047648	4507	MTAP	9	21847244	1.54E-02	2229	3.60E-01	Up	0.91	9682	1.81	0.04
rs4392253	1285	COL4A3	2	227904605	1.54E-02	2230	7.36E-03	Down	2.68	4289	1.81	0.21
rs11818705	3832	KIF11	10	94382708	1.54E-02	2231	1.29E-08	Up	5.66	1092	1.81	0.79
rs436143	64377	CHST8	19	38937781	1.54E-02	2232	3.60E-02	Down	2.10	5648	1.81	0.14
rs1807426	56895	AGPAT4	6	161531667	1.54E-02	2233	4.62E-01	Up	0.74	10439	1.81	0.03
rs11706953	4336	MOBP	3	39488614	1.54E-02	2234	2.16E-01	Up	1.24	8426	1.81	0.07
rs10973324	9380	GRHPR	9	37403802	1.54E-02	2235	8.72E-05	Up	3.92	2416	1.81	0.41
rs1867723	54463	FAM134B	5	16550102	1.54E-02	2236	4.24E-01	Up	0.80	10149	1.81	0.04
rs17761443	25885	POLR1A	17	1746408	1.54E-02	2237	3.01E-04	Up	3.61	2775	1.81	0.35
rs17761443	6117	RPA1	17	1746408	1.54E-02	2238	1.81E-02	Down	2.36	4992	1.81	0.17
rs3744893	8575	PRKRA	18	55086840	1.54E-02	2239	7.18E-02	Down	1.80	6465	1.81	0.11
rs3744893	30062	RAX	18	55086840	1.54E-02	2240	5.80E-01	Down	0.55	11317	1.81	0.02
rs918545	65982	ZSCAN18	19	63311880	1.55E-02	2241	6.55E-01	Down	0.45	11804	1.81	0.02
rs6747350	9360	PPIG	2	170314277	1.55E-02	2242	5.54E-01	Down	0.59	11112	1.81	0.03
rs11124031	2274	FHL2	2	105479459	1.55E-02	2243	5.67E-22	Down	9.64	160	1.81	2.12
rs2253680	79675	FASTKD1	2	170228537	1.55E-02	2244	4.51E-04	Up	3.51	2921	1.81	0.33
rs10904865	8029	CUBN	10	17120639	1.55E-02	2245	2.34E-01	Down	1.19	8605	1.81	0.06
rs7645934	23007	PLCH1	3	156877597	1.55E-02	2246	2.99E-04	Up	3.62	2771	1.81	0.35
rs2299297	55904	MLL5	7	104339246	1.55E-02	2247	8.50E-01	Up	0.19	12956	1.81	0.01
rs9650418	5520	PPP2R2A	8	26215426	1.55E-02	2248	8.40E-04	Up	3.34	3150	1.81	0.31
rs39823	1794	DOCK2	5	169187984	1.55E-02	2249	9.50E-01	Up	0.06	13550	1.81	0.00
rs6598370	140460	ASB7	15	99004357	1.55E-02	2250	1.96E-01	Up	1.29	8209	1.81	0.07
rs13031275	4703	NEB	2	152172002	1.56E-02	2251	3.29E-02	Down	2.13	5556	1.81	0.15
rs4151448	5925	RB1	13	47815674	1.56E-02	2252	3.41E-01	Up	0.95	9536	1.81	0.05
rs1000427	80020	FOXRED2	22	35214605	1.56E-02	2253	3.52E-04	Up	3.57	2837	1.81	0.35
rs1000427	25828	TXN2	22	35214605	1.56E-02	2254	5.62E-02	Up	1.91	6143	1.81	0.13
rs1866863	51256	TBC1D7	6	13444794	1.56E-02	2255	6.23E-01	Up	0.49	11604	1.81	0.02
rs9616802	410	ARSA	22	49362305	1.56E-02	2256	6.90E-02	Up	1.82	6412	1.81	0.12
rs1875411	58499	ZNF462	9	106824524	1.56E-02	2257	1.44E-01	Up	1.46	7614	1.81	0.08
rs3751624	55930	MYO5C	15	50362462	1.56E-02	2258	3.73E-02	Up	2.08	5686	1.81	0.14
rs183532	4653	MYOC	1	168341138	1.56E-02	2259	5.32E-01	Down	0.63	10951	1.81	0.03
rs3848535	6543	SLC8A2	19	52640040	1.56E-02	2260	1.62E-02	Down	2.40	4899	1.81	0.18
rs4979402	25861	DFNB31	9	114302229	1.56E-02	2261	1.89E-01	Up	1.31	8131	1.81	0.07
rs7809363	57180	ACTR3B	7	151895451	1.56E-02	2262	2.74E-04	Up	3.64	2748	1.81	0.36
rs2275864	7139	TNNT2	1	198059752	1.57E-02	2263	1.76E-03	Up	3.13	3468	1.81	0.28

rs7026254	286343	C9orf150	9	12815978	1.57E-02	2264	6.72E-01	Up	0.42	11896	1.80	0.02
rs11622263	55384	MEG3	14	100350414	1.57E-02	2265	3.82E-10	Down	6.26	827	1.80	0.94
rs2355351	140469	MYO3B	2	170927016	1.57E-02	2266	7.73E-01	Up	0.29	12522	1.80	0.01
rs341237	91133	L3MBTL4	18	6391283	1.57E-02	2267	6.49E-02	Down	1.85	6343	1.80	0.12
rs13103731	2993	GYPA	4	145414092	1.58E-02	2268	1.03E-02	Up	2.56	4535	1.80	0.20
rs13103731	2994	GYPB	4	145414092	1.58E-02	2269	1.64E-02	Up	2.40	4909	1.80	0.18
rs12489992	51555	PEX5L	3	181147627	1.58E-02	2270	2.57E-01	Down	1.13	8833	1.80	0.06
rs2064599	27242	TNFRSF21	6	47367642	1.58E-02	2271	1.32E-02	Up	2.48	4698	1.80	0.19
rs3801778	9732	DOCK4	7	111004593	1.58E-02	2272	1.41E-10	Up	6.42	775	1.80	0.99
rs1397613	2169	FABP2	4	120600532	1.58E-02	2273	9.58E-01	Up	0.05	13604	1.80	0.00
rs1245371	80352	RNF39	6	30146331	1.58E-02	2274	3.10E-02	Down	2.16	5494	1.80	0.15
rs1245371	30834	ZNRD1	6	30146331	1.58E-02	2275	8.71E-02	Up	1.71	6756	1.80	0.11
rs1245371	6992	PPP1R11	6	30146331	1.58E-02	2276	4.46E-01	Down	0.76	10317	1.80	0.04
rs4623769	5129	PCTK3	1	202224336	1.58E-02	2277	7.38E-01	Down	0.34	12300	1.80	0.01
rs936431	2922	GRP	18	55039736	1.58E-02	2278	7.28E-01	Up	0.35	12236	1.80	0.01
rs10454095	2627	GATA6	18	18031438	1.58E-02	2279	3.50E-01	Down	0.93	9591	1.80	0.05
rs12590471	8748	ADAM20	14	70070853	1.58E-02	2280	9.97E-01	Down	0.00	13891	1.80	0.00
rs17373453	56833	SLAMF8	1	156611480	1.58E-02	2281	8.87E-02	Up	1.70	6779	1.80	0.11
rs757158	5444	PON1	7	94600179	1.58E-02	2282	3.59E-01	Down	0.92	9667	1.80	0.04
rs359642	55131	RBM28	7	127594042	1.58E-02	2283	7.39E-03	Up	2.68	4291	1.80	0.21
rs3770557	55686	MREG	2	216690193	1.58E-02	2284	7.39E-01	Down	0.33	12308	1.80	0.01
rs16838062	3760	KCNJ3	2	155416410	1.59E-02	2285	4.55E-01	Down	0.75	10383	1.80	0.03
rs597320	83700	JAM3	11	133524571	1.59E-02	2286	3.59E-17	Down	8.43	300	1.80	1.64
rs597320	23310	NCAPD3	11	133524571	1.59E-02	2287	4.38E-05	Up	4.09	2250	1.80	0.44
rs6018311	2139	EYA2	20	45226041	1.59E-02	2288	2.43E-02	Down	2.25	5261	1.80	0.16
rs6087123	63926	ANKRD5	20	9986994	1.59E-02	2289	3.82E-01	Down	0.87	9860	1.80	0.04
rs7825010	56892	C8orf4	8	40112323	1.59E-02	2290	1.08E-01	Up	1.61	7119	1.80	0.10
rs3768853	8942	KYNU	2	143508558	1.59E-02	2291	8.42E-01	Up	0.20	12913	1.80	0.01
rs7051930	256714	MAP7D2		19818416	1.59E-02	2292	9.64E-01	Down	0.05	13640	1.80	0.00
rs693293	5291	PIK3CB	3	139891874	1.59E-02	2293	3.44E-03	Up	2.93	3845	1.80	0.25
rs2216858	83857	TMTC1	12	29552643	1.59E-02	2294	2.63E-06	Down	4.70	1710	1.80	0.56
rs556731	5740	PTGIS	20	47545027	1.59E-02	2295	2.90E-08	Down	5.55	1162	1.80	0.75
rs4131289	10960	LMAN2	5	176713151	1.59E-02	2296	3.64E-03	Up	2.91	3879	1.80	0.24
rs4131289	10636	RGS14	5	176713151	1.59E-02	2297	9.08E-02	Down	1.69	6842	1.80	0.10
rs1709544	11076	TPPP	5	727921	1.59E-02	2298	1.06E-01	Down	1.62	7094	1.80	0.10
rs997766	135228	CD109	6	74563697	1.59E-02	2299	1.64E-03	Down	3.15	3429	1.80	0.28
rs10247493	9747	FAM115A	7	143043882	1.60E-02	2300	4.92E-06	Up	4.57	1811	1.80	0.53
rs17820032	83445	GSG1	12	13139424	1.60E-02	2301	3.20E-01	Up	0.99	9360	1.80	0.05
rs17644304	134553	C5orf24	5	134224948	1.60E-02	2302	1.49E-01	Up	1.44	7660	1.80	0.08
rs4283545	54756	IL17RD	3	57175798	1.60E-02	2303	6.00E-01	Down	0.52	11429	1.80	0.02
rs4312055	8495	PPFIBP2	11	7574609	1.60E-02	2304	7.24E-02	Down	1.80	6473	1.80	0.11
rs501383	83698	CALN1	7	71189547	1.60E-02	2305	6.97E-02	Up	1.81	6417	1.80	0.12
rs6682627	5784	PTPN14	1	210990105	1.60E-02	2306	1.57E-04	Down	3.78	2560	1.80	0.38
rs4659356	26119	LDLRAP1	1	25584361	1.60E-02	2307	1.61E-01	Down	1.40	7833	1.80	0.08
rs3736535	55689	YEATS2	3	184915685	1.60E-02	2308	4.71E-04	Up	3.50	2939	1.80	0.33
rs17243850	55628	ZNF407	18	70636280	1.60E-02	2309	3.68E-01	Up	0.90	9747	1.79	0.04
rs702045	84561	SLC12A8	3	126332460	1.61E-02	2310	3.86E-05	Up	4.12	2221	1.79	0.44
rs2290376	11069	RAPGEF4	2	173724226	1.61E-02	2311	3.45E-03	Up	2.92	3849	1.79	0.25
rs2292506	112802	KRT71	12	51224587	1.61E-02	2312	1.71E-01	Down	1.37	7929	1.79	0.08
rs7667661	2868	GRK4	4	2991722	1.61E-02	2313	6.00E-04	Up	3.43	3028	1.79	0.32
rs16822826	127700	C1orf102	1	36544499	1.61E-02	2314	8.52E-02	Up	1.72	6725	1.79	0.11
rs16822826	8013	NR4A3	1	36544499	1.61E-02	2315	9.93E-02	Up	1.65	6986	1.79	0.10
rs16822826	84967	LSM10	1	36544499	1.61E-02	2316	2.02E-01	Down	1.28	8273	1.79	0.07
rs7864960	3439	IFNA1	9	21438448	1.61E-02	2317	1.08E-01	Up	1.61	7123	1.79	0.10
rs5753350	23762	OSBP2	22	29580620	1.62E-02	2318	3.96E-01	Down	0.85	9960	1.79	0.04
rs672824	55311	ZNF444	19	61328571	1.62E-02	2319	4.55E-03	Up	2.84	4003	1.79	0.23
rs670345	80168	MOGAT2	11	75123917	1.62E-02	2320	7.39E-01	Up	0.33	12307	1.79	0.01
rs12933806	114780	PKD1L2	16	79806217	1.62E-02	2321	4.24E-01	Down	0.80	10150	1.79	0.04
rs13057428	1399	CRKL	22	19611495	1.62E-02	2322	9.89E-03	Down	2.58	4500	1.79	0.20
rs10503170	9172	MYOM2	8	2079891	1.63E-02	2323	4.58E-04	Down	3.50	2924	1.79	0.33
rs302484	4208	MEF2C	5	88179505	1.63E-02	2324	2.11E-06	Down	4.74	1677	1.79	0.57
rs12978543	23149	FCHO1	19	17743548	1.63E-02	2325	2.47E-01	Up	1.16	8735	1.79	0.06
rs2329845	54084	C21orf29	21	44911592	1.63E-02	2326	9.35E-02	Up	1.68	6893	1.79	0.10
rs164420	117157	SH2D1B	1	159091404	1.63E-02	2327	9.20E-01	Up	0.10	13379	1.79	0.00
rs678776	486	FXYD2	11	117201954	1.63E-02	2328	8.59E-03	Down	2.63	4393	1.79	0.21
rs11120089	26750	RPS6KC1	1	209666720	1.63E-02	2329	1.20E-06	Up	4.85	1602	1.79	0.59
rs11982486	5445	PON2	7	94700249	1.63E-02	2330	6.45E-06	Up	4.51	1862	1.79	0.52
rs1892654	59271	C21orf63	21	32785117	1.64E-02	2331	1.82E-04	Down	3.74	2595	1.79	0.37
rs3745925	8174	MADCAM1	19	452900	1.64E-02	2332	9.21E-02	Up	1.68	6863	1.79	0.10
rs3745925	91978	C19orf20	19	452900	1.64E-02	2333	7.77E-01	Up	0.28	12538	1.79	0.01
rs2215164	23242	COBL	7	51093537	1.64E-02	2334	7.55E-03	Up	2.67	4308	1.78	0.21
rs756875	8216	LZTR1	22	19642987	1.64E-02	2335	3.30E-04	Up	3.59	2811	1.78	0.35
rs756875	150209	AIFM3	22	19642987	1.64E-02	2336	1.87E-01	Down	1.32	8112	1.78	0.07
rs4689545	9778	KIAA0232	4	6885408	1.65E-02	2337	4.06E-02	Down	2.05	5759	1.78	0.14
rs1474520	43849	KLK12	19	56222505	1.65E-02	2338	3.41E-04	Up	3.58	2822	1.78	0.35
rs1474520	5655	KLK10	19	56222505	1.65E-02	2339	1.98E-02	Down	2.33	5061	1.78	0.17
rs1474520	11012	KLK11	19	56222505	1.65E-02	2340	5.82E-02	Down	1.89	6193	1.78	0.12
rs6031752	8839	WISP2	20	42767888	1.65E-02	2341	2.22E-03	Down	3.06	3602	1.78	0.27
rs7905809	27063	ANKRD1	10	92676401	1.65E-02	2342	1.50E-01	Up	1.44	7679	1.78	0.08
rs2194430	1385	CREB1	2	208269921	1.65E-02	2343	1.77E-02	Up	2.37	4969	1.78	0.18
rs11741523	57491	AHRR	5	456069	1.65E-02	2344	6.17E-01	Up	0.50	11555	1.78	0.02

rs12130483	57540	PTCHD2	1	11492236	1.65E-02	2345	2.29E-01	Up	1.20	8548	1.78	0.06
rs9937570	64131	XYLT1	16	17169566	1.65E-02	2346	8.48E-01	Down	0.19	12942	1.78	0.01
rs649859	127795	C1orf87	1	60176189	1.65E-02	2347	5.80E-01	Up	0.55	11315	1.78	0.02
rs17033270	491	ATP2B2	3	10646678	1.65E-02	2348	2.95E-01	Up	1.05	9152	1.78	0.05
rs279025	55707	NECAP2	1	16533821	1.66E-02	2349	1.25E-01	Up	1.53	7370	1.78	0.09
rs2114591	5376	PMP22	2	230876074	1.66E-02	2350	1.27E-25	Down	10.46	105	1.78	2.49
rs2114591	3431	SP110	2	230876074	1.66E-02	2351	5.62E-01	Up	0.58	11181	1.78	0.02
rs12940988	1770	DNAH9	17	11786681	1.66E-02	2352	6.20E-02	Up	1.87	6279	1.78	0.12
rs336288	57057	TBX20	7	35076602	1.66E-02	2353	3.03E-01	Up	1.03	9217	1.78	0.05
rs3858282	8513	LIPF	10	90424391	1.66E-02	2354	9.06E-01	Up	0.12	13283	1.78	0.00
rs6465412	1278	COL1A2	7	93703046	1.66E-02	2355	1.02E-03	Down	3.28	3230	1.78	0.30
rs17112022	206358	SLC36A1	5	150841894	1.66E-02	2356	4.87E-02	Up	1.97	5964	1.78	0.13
rs3785957	5048	PAFAH1B1	17	2507919	1.66E-02	2357	4.93E-10	Down	6.22	856	1.78	0.93
rs2611603	1139	CHRNA7	15	30228824	1.66E-02	2358	2.98E-07	Down	5.12	1416	1.78	0.65
rs4750121	254427	C10orf47	10	11901764	1.67E-02	2359	2.26E-04	Up	3.69	2672	1.78	0.36
rs1556487	91283	C9orf30	9	100293502	1.67E-02	2360	9.26E-01	Up	0.09	13403	1.78	0.00
rs6508441	80000	KIAA1772	18	17312540	1.67E-02	2361	7.07E-01	Up	0.38	12115	1.78	0.02
rs209353	2566	GABRG2	5	161449637	1.67E-02	2362	7.84E-04	Down	3.36	3128	1.78	0.31
rs1421368	7941	PLA2G7	6	46804552	1.67E-02	2363	7.85E-18	Up	8.60	276	1.78	1.71
rs9637198	80781	COL18A1	21	45644510	1.67E-02	2364	4.61E-01	Down	0.74	10437	1.78	0.03
rs4611819	4045	LSAMP	3	117196294	1.67E-02	2365	1.04E-08	Down	5.72	1060	1.78	0.80
rs11918693	1360	CPB1	3	150014056	1.67E-02	2366	6.58E-01	Down	0.44	11819	1.78	0.02
rs1138253	79187	FSD1	19	4276433	1.68E-02	2367	6.78E-01	Down	0.42	11935	1.78	0.02
rs6795240	79718	TBL1XR1	3	178243704	1.68E-02	2368	4.81E-05	Up	4.06	2273	1.78	0.43
rs9526782	115825	WDFY2	13	51059970	1.68E-02	2369	6.45E-08	Down	5.41	1249	1.78	0.72
rs11869519	91607	SLFN11	17	30715522	1.68E-02	2370	1.55E-04	Down	3.78	2559	1.78	0.38
rs1046822	25759	SHC2	19	367963	1.68E-02	2371	1.87E-02	Up	2.35	5010	1.78	0.17
rs4968008	23062	GGA2	16	23397854	1.68E-02	2372	4.78E-09	Down	5.85	1006	1.78	0.83
rs12953411	4158	MC2R	18	13866390	1.68E-02	2373	6.42E-01	Up	0.47	11721	1.77	0.02
rs7037208	57589	KIAA1432	9	5601912	1.68E-02	2374	8.68E-02	Down	1.71	6753	1.77	0.11
rs17035210	6769	STAC	3	36528606	1.69E-02	2375	8.00E-06	Down	4.47	1903	1.77	0.51
rs1094328	26953	RANBP6	9	6019644	1.69E-02	2376	1.89E-01	Down	1.31	8130	1.77	0.07
rs1892022	10207	INADL	1	61950434	1.69E-02	2377	6.78E-04	Up	3.40	3068	1.77	0.32
rs6583351	56975	FAM20C	7	281202	1.69E-02	2378	7.00E-01	Down	0.38	12073	1.77	0.02
rs16972486	1445	CSK	15	72853061	1.69E-02	2379	1.59E-05	Up	4.32	2047	1.77	0.48
rs6076053	29107	NXT1	20	23277095	1.69E-02	2380	2.02E-01	Down	1.28	8278	1.77	0.07
rs640827	219770	GJD4	10	35941721	1.69E-02	2381	4.02E-01	Down	0.84	9991	1.77	0.04
rs1513315	23380	SRGAP2	3	9047286	1.70E-02	2382	1.03E-01	Up	1.63	7046	1.77	0.10
rs10138313	55334	SLC39A9	14	68952145	1.70E-02	2383	7.56E-03	Up	2.67	4311	1.77	0.21
rs10138313	2079	ERH	14	68952145	1.70E-02	2384	3.45E-02	Up	2.11	5609	1.77	0.15
rs16908021	4920	ROR2	9	91778495	1.70E-02	2385	1.80E-08	Down	5.63	1113	1.77	0.77
rs16908021	6095	RORA	9	91778495	1.70E-02	2386	5.29E-03	Up	2.79	4104	1.77	0.23
rs7257986	148170	CDC42EP5	19	59677248	1.70E-02	2387	5.24E-03	Down	2.79	4099	1.77	0.23
rs7257986	94059	LENG9	19	59677248	1.70E-02	2388	4.73E-01	Down	0.72	10516	1.77	0.03
rs4819225	54058	C21orf58	21	46536613	1.70E-02	2389	7.12E-01	Down	0.37	12145	1.77	0.01
rs2817675	6585	SLIT1	10	98806776	1.70E-02	2390	2.72E-07	Up	5.14	1405	1.77	0.66
rs2817675	6586	SLIT3	10	98806776	1.70E-02	2391	7.66E-05	Down	3.95	2378	1.77	0.41
rs6641609	5613	PRKX		3600750	1.70E-02	2392	8.80E-01	Up	0.15	13129	1.77	0.01
rs10025791	817	CAMK2D	4	114881859	1.70E-02	2393	1.31E-16	Down	8.27	328	1.77	1.59
rs3925075	3684	ITGAM	16	31255249	1.70E-02	2394	4.89E-01	Up	0.69	10647	1.77	0.03
rs8106720	10775	POP4	19	34769168	1.71E-02	2395	1.60E-01	Down	1.41	7812	1.77	0.08
rs11701698	10215	OLIG2	21	33315815	1.71E-02	2396	8.68E-01	Down	0.17	13052	1.77	0.01
rs4798890	9218	VAPA	18	9915700	1.71E-02	2397	8.58E-07	Down	4.92	1555	1.77	0.61
rs1061837	26112	CCDC69	5	150541530	1.71E-02	2398	1.09E-09	Down	6.10	899	1.77	0.90
rs11618203	9071	CLDN10	13	94885089	1.71E-02	2399	2.74E-03	Up	3.00	3739	1.77	0.26
rs7108147	9793	CKAP5	11	46787423	1.71E-02	2400	1.35E-02	Up	2.47	4722	1.77	0.19
rs7148939	5891	RAGE	14	101785409	1.71E-02	2401	2.73E-03	Up	3.00	3737	1.77	0.26
rs7148939	177	AGER	14	101785409	1.71E-02	2402	4.67E-01	Down	0.73	10476	1.77	0.03
rs1365888	168667	BMPER	7	33786069	1.72E-02	2403	5.20E-03	Up	2.79	4094	1.77	0.23
rs156031	8974	P4HA2	5	131554089	1.72E-02	2404	2.09E-05	Down	4.25	2099	1.76	0.47
rs7650366	259173	ALS2CL	3	46719970	1.72E-02	2405	3.63E-01	Down	0.91	9708	1.76	0.04
rs7650366	259236	TMIE	3	46719970	1.72E-02	2406	7.84E-01	Up	0.27	12573	1.76	0.01
rs11134551	84868	HAVCR2	5	156437811	1.72E-02	2407	3.16E-03	Up	2.95	3804	1.76	0.25
rs11134551	26762	HAVCR1	5	156437811	1.72E-02	2408	6.85E-01	Up	0.41	11977	1.76	0.02
rs2604874	6626	SNRPA	19	45949656	1.72E-02	2409	8.92E-07	Up	4.91	1564	1.76	0.60
rs2604874	284325	C19orf54	19	45949656	1.72E-02	2410	1.66E-03	Up	3.14	3437	1.76	0.28
rs6659231	148345	C1orf127	1	10958619	1.72E-02	2411	9.93E-02	Down	1.65	6988	1.76	0.10
rs2278238	8178	ELL	19	18437484	1.73E-02	2412	2.31E-01	Down	1.20	8570	1.76	0.06
rs10960751	7306	TYRP1	9	12665264	1.73E-02	2413	6.67E-01	Up	0.43	11861	1.76	0.02
rs6986291	2131	EXT1	8	119088138	1.73E-02	2414	6.58E-05	Down	3.99	2348	1.76	0.42
rs11084329	23547	LILRA4	19	59527583	1.73E-02	2415	8.53E-01	Up	0.18	12977	1.76	0.01
rs9905940	3212	HOXB2	17	43969101	1.73E-02	2416	2.85E-02	Down	2.19	5411	1.76	0.15
rs9905940	3211	HOXB1	17	43969101	1.73E-02	2417	3.70E-01	Down	0.90	9767	1.76	0.04
rs7521072	159	ADSS	1	240919321	1.73E-02	2418	7.64E-02	Down	1.77	6560	1.76	0.11
rs7545290	4879	NPPB	1	11869194	1.73E-02	2419	6.61E-01	Down	0.44	11836	1.76	0.02
rs320996	10800	CYSLTR1		77333617	1.73E-02	2420	4.17E-02	Down	2.04	5792	1.76	0.14
rs7904348	56288	PARD3	10	35108820	1.73E-02	2421	6.17E-03	Down	2.74	4182	1.76	0.22
rs17131658	58511	DNASE2B	1	84569759	1.74E-02	2422	9.57E-04	Up	3.30	3203	1.76	0.30
rs677344	64601	VPS16	20	2794289	1.74E-02	2423	8.37E-01	Down	0.21	12886	1.76	0.01
rs3817121	23066	CAND2	3	12836608	1.74E-02	2424	7.34E-11	Down	6.51	738	1.76	1.01
rs10506727	144455	E2F7	12	75975395	1.74E-02	2425	7.88E-02	Up	1.76	6609	1.76	0.11

rs2064829	6227	RPS21	20	60402998	1.74E-02	2426	1.48E-12	Up	7.08	568	1.76	1.18
rs2064829	81928	CABLES2	20	60402998	1.74E-02	2427	3.09E-01	Up	1.02	9262	1.76	0.05
rs7917456	3698	ITIH2	10	7772329	1.74E-02	2428	6.83E-01	Up	0.41	11965	1.76	0.02
rs140524	9997	SCO2	22	49250826	1.75E-02	2429	4.36E-01	Up	0.78	10245	1.76	0.04
rs140524	29781	NCAPH2	22	49250826	1.75E-02	2430	8.94E-01	Down	0.13	13212	1.76	0.00
rs193908	23090	ZNF423	16	48078809	1.75E-02	2431	6.52E-07	Down	4.98	1511	1.76	0.62
rs4398867	7486	WRN	8	31139701	1.75E-02	2432	2.16E-03	Up	3.07	3581	1.76	0.27
rs2288039	84916	CIRH1A	16	67745613	1.75E-02	2433	3.25E-01	Up	0.98	9406	1.76	0.05
rs7634881	84223	IQCG	3	199119478	1.75E-02	2434	1.66E-01	Down	1.39	7877	1.76	0.08
rs7928477	10003	NAALAD2	11	89535588	1.75E-02	2435	4.66E-01	Down	0.73	10470	1.76	0.03
rs7252142	10469	TIMM44	19	7918015	1.75E-02	2436	9.38E-01	Up	0.08	13480	1.76	0.00
rs3792006	3032	HADHB	2	26409873	1.76E-02	2437	5.00E-01	Up	0.67	10721	1.75	0.03
rs6721460	6670	SP3	2	174670374	1.76E-02	2438	1.76E-02	Down	2.37	4961	1.75	0.18
rs2031709	29101	SSU72	1	1571251	1.76E-02	2439	7.94E-04	Up	3.35	3133	1.75	0.31
rs2098684	587	BCAT2	19	54012819	1.76E-02	2440	1.54E-12	Up	7.07	572	1.75	1.18
rs11756089	7005	TEAD3	6	35560324	1.76E-02	2441	1.04E-14	Down	7.73	410	1.75	1.40
rs524047	1000	CDH2	18	23907945	1.77E-02	2442	2.84E-01	Down	1.07	9068	1.75	0.05
rs17216066	91584	PLXNA4	7	131358506	1.77E-02	2443	1.81E-01	Down	1.34	8039	1.75	0.07
rs995043	84725	PLEKHA8	7	29884802	1.77E-02	2444	9.18E-02	Down	1.69	6854	1.75	0.10
rs4854182	51112	TTC15	2	4905173	1.77E-02	2445	6.97E-01	Down	0.39	12051	1.75	0.02
rs1913077	9729	KIAA0408	6	127895137	1.77E-02	2446	9.13E-01	Down	0.11	13331	1.75	0.00
rs10180853	3790	KCNS3	2	17965405	1.77E-02	2447	6.18E-07	Up	4.99	1506	1.75	0.62
rs8062460	8399	PLA2G10	16	14667269	1.77E-02	2448	1.61E-01	Down	1.40	7826	1.75	0.08
rs8062460	51283	BFAR	16	14667269	1.77E-02	2449	6.44E-01	Up	0.46	11737	1.75	0.02
rs16852884	57501	KIAA1257	3	130187709	1.77E-02	2450	5.45E-01	Up	0.61	11048	1.75	0.03
rs4678681	10491	CRTAP	3	33125833	1.77E-02	2451	5.60E-16	Down	8.10	349	1.75	1.53
rs4696953	9353	SLIT2	4	19999182	1.77E-02	2452	4.98E-03	Down	2.81	4068	1.75	0.23
rs7182141	146057	TTBK2	15	40849840	1.78E-02	2453	1.20E-01	Down	1.56	7291	1.75	0.09
rs4036	6923	TCEB2	16	2761526	1.78E-02	2454	2.60E-03	Up	3.01	3709	1.75	0.26
rs4036	23524	SRRM2	16	2761526	1.78E-02	2455	2.83E-03	Up	2.99	3754	1.75	0.25
rs2278385	3702	ITK	5	156521977	1.78E-02	2456	1.92E-02	Down	2.34	5033	1.75	0.17
rs10235235	9551	ATP5J2	7	98720482	1.78E-02	2457	3.45E-01	Up	0.94	9560	1.75	0.05
rs2318896	126433	FBXO27	19	44202618	1.78E-02	2458	2.87E-01	Down	1.07	9090	1.75	0.05
rs1872761	161835	FSIP1	15	37791108	1.78E-02	2459	6.06E-01	Down	0.52	11477	1.75	0.02
rs2024269	23072	HECW1	7	43247057	1.78E-02	2460	2.40E-02	Down	2.26	5248	1.75	0.16
rs4625870	29789	OLA1	2	174926094	1.78E-02	2461	9.34E-04	Up	3.31	3186	1.75	0.30
rs1659495	23295	MGRN1	16	4659982	1.78E-02	2462	2.01E-01	Down	1.28	8271	1.75	0.07
rs6096751	55734	ZFP64	20	50145753	1.78E-02	2463	4.97E-02	Up	1.96	5990	1.75	0.13
rs27003	79772	MCTP1	5	94227923	1.78E-02	2464	6.70E-04	Up	3.40	3064	1.75	0.32
rs928069	57186	C20orf74	20	20355782	1.78E-02	2465	5.52E-22	Up	9.64	159	1.75	2.13
rs9480384	389434	IYD	6	150821992	1.78E-02	2466	6.92E-01	Up	0.40	12020	1.75	0.02
rs17245731	2770	GNAI1	7	79477284	1.79E-02	2467	6.05E-06	Up	4.52	1846	1.75	0.52
rs10738886	48	ACO1	9	32399442	1.79E-02	2468	6.39E-08	Down	5.41	1245	1.75	0.72
rs7988598	23026	MYO16	13	108055256	1.79E-02	2469	1.04E-03	Down	3.28	3236	1.75	0.30
rs941125	148979	GLIS1	1	53835894	1.79E-02	2470	2.98E-02	Down	2.17	5454	1.75	0.15
rs7486864	57634	EP400	12	131141071	1.79E-02	2471	6.69E-01	Up	0.43	11880	1.75	0.02
rs10987695	6812	STXBP1	9	127441300	1.79E-02	2472	9.10E-01	Up	0.11	13309	1.75	0.00
rs3810053	23255	KIAA0802	18	8810886	1.79E-02	2473	3.54E-01	Up	0.93	9632	1.75	0.05
rs2820304	25802	LMOD1	1	198650184	1.79E-02	2474	1.27E-22	Down	9.79	148	1.75	2.19
rs2820304	10440	TIMM17A	1	198650184	1.79E-02	2475	9.33E-02	Up	1.68	6889	1.75	0.10
rs3016369	923	CD6	11	60482185	1.79E-02	2476	7.10E-01	Down	0.37	12126	1.75	0.01
rs3016369	51296	SLC15A3	11	60482185	1.79E-02	2477	7.97E-01	Up	0.26	12647	1.75	0.01
rs372992	150000	ABCC13	21	14556175	1.79E-02	2478	8.88E-01	Down	0.14	13176	1.75	0.01
rs6839672	118429	ANTXR2	4	81350312	1.80E-02	2479	5.86E-18	Down	8.64	270	1.75	1.72
rs10912870	94241	TP53INP1	1	171719673	1.80E-02	2480	4.48E-11	Up	6.58	711	1.75	1.03
rs10912870	27101	CACYBP	1	171719673	1.80E-02	2481	1.19E-01	Down	1.56	7280	1.75	0.09
rs10912870	10432	RBM14	1	171719673	1.80E-02	2482	1.37E-01	Up	1.49	7535	1.75	0.09
rs10912870	63931	MRPS14	1	171719673	1.80E-02	2483	2.87E-01	Up	1.06	9094	1.75	0.05
rs6682769	192670	EIF2C4	1	35995289	1.80E-02	2484	2.41E-02	Down	2.26	5251	1.75	0.16
rs6900397	154313	C6orf165	6	88186033	1.80E-02	2485	5.76E-01	Down	0.56	11286	1.74	0.02
rs1057691	11165	NUDT3	6	34407210	1.80E-02	2486	5.73E-03	Up	2.76	4155	1.74	0.22
rs3736781	696	BTN1A1	6	26613341	1.81E-02	2487	7.45E-01	Up	0.32	12348	1.74	0.01
rs9486077	5550	PREP	6	105955716	1.81E-02	2488	6.21E-05	Up	4.00	2336	1.74	0.42
rs10801913	81839	VANGL1	1	115926321	1.81E-02	2489	2.14E-01	Up	1.24	8396	1.74	0.07
rs9438989	54933	RHBDL2	1	39041936	1.81E-02	2490	2.72E-02	Down	2.21	5366	1.74	0.16
rs12484402	6942	TCF20	22	40910571	1.81E-02	2491	9.93E-01	Up	0.01	13867	1.74	0.00
rs2278370	2196	FAT2	5	150910379	1.81E-02	2492	8.53E-05	Down	3.93	2410	1.74	0.41
rs1937905	8644	AKR1C3	10	5120610	1.81E-02	2493	2.70E-01	Up	1.10	8950	1.74	0.06
rs9471165	23500	DAAM2	6	39861285	1.81E-02	2494	8.44E-14	Down	7.46	465	1.74	1.31
rs11814806	22986	SORCS3	10	106893008	1.81E-02	2495	2.14E-02	Up	2.30	5138	1.74	0.17
rs4808410	57834	CYP4F11	19	15894485	1.81E-02	2496	6.43E-03	Up	2.72	4204	1.74	0.22
rs6916146	135138	PACRG	6	163680576	1.81E-02	2497	4.28E-01	Up	0.79	10175	1.74	0.04
rs2180768	9372	ZFYVE9	1	52429842	1.81E-02	2498	7.15E-03	Up	2.69	4270	1.74	0.21
rs2276029	726	CAPN5	11	76473807	1.81E-02	2499	3.68E-01	Up	0.90	9743	1.74	0.04
rs2276029	4975	OMP	11	76473807	1.81E-02	2500	4.98E-01	Down	0.68	10702	1.74	0.03
rs9889827	57690	TNRC6C	17	73538266	1.82E-02	2501	1.41E-03	Down	3.19	3352	1.74	0.29
rs9641977	57016	AKR1B10	7	133688982	1.82E-02	2502	3.22E-01	Up	0.99	9381	1.74	0.05
rs763238	55966	AJAP1	1	4654077	1.82E-02	2503	1.23E-05	Down	4.37	1999	1.74	0.49
rs588997	3115	HLA-DPB1	6	33136695	1.82E-02	2504	2.26E-01	Down	1.21	8521	1.74	0.06
rs588997	3113	HLA-DPA1	6	33136695	1.82E-02	2505	3.78E-01	Up	0.88	9825	1.74	0.04
rs4838368	196740	C10orf72	10	49999752	1.82E-02	2506	1.28E-06	Down	4.84	1608	1.74	0.59

rs11201026	54462	KIAA1128	10	86154179	1.82E-02	2507	9.01E-17	Down	8.32	318	1.74	1.60
rs13331198	6645	SNTB2	16	67820240	1.82E-02	2508	2.18E-14	Down	7.64	431	1.74	1.37
rs7209700	3690	ITGB3	17	42706117	1.82E-02	2509	8.06E-02	Down	1.75	6640	1.74	0.11
rs17074629	3146	HMGB1	13	29949738	1.82E-02	2510	3.22E-01	Down	0.99	9379	1.74	0.05
rs736227	1671	DEFA6	8	6780950	1.83E-02	2511	1.59E-01	Down	1.41	7799	1.74	0.08
rs736227	1669	DEFA4	8	6780950	1.83E-02	2512	8.58E-01	Up	0.18	13005	1.74	0.01
rs9284299	440279	UNC13C	15	52685923	1.83E-02	2513	4.10E-02	Down	2.04	5772	1.74	0.14
rs17785113	23348	DOCK9	13	98434671	1.83E-02	2514	2.89E-01	Up	1.06	9103	1.74	0.05
rs2597177	80279	CDK5RAP3	17	43415528	1.83E-02	2515	6.57E-06	Up	4.51	1864	1.74	0.52
rs3897937	9210	BMP15		50488052	1.83E-02	2516	5.89E-01	Down	0.54	11360	1.74	0.02
rs9311896	56999	ADAMTS9	3	64514815	1.83E-02	2517	9.53E-01	Down	0.06	13582	1.74	0.00
rs2885470	9711	KIAA0226	3	198963750	1.83E-02	2518	3.28E-04	Up	3.59	2808	1.74	0.35
rs12679873	401474	SAMD12	8	119505892	1.83E-02	2519	8.05E-02	Down	1.75	6636	1.74	0.11
rs2885470	84248	FYTTD1	3	198963750	1.83E-02	2520	5.80E-01	Down	0.55	11313	1.74	0.02
rs9880989	6165	RPL35A	3	199153909	1.83E-02	2521	4.55E-05	Up	4.08	2260	1.74	0.43
rs6005031	23544	SEZ6L	22	25089721	1.83E-02	2522	6.04E-03	Down	2.75	4174	1.74	0.22
rs17628268	10123	ARL4C	4	113688376	1.84E-02	2523	7.45E-07	Down	4.95	1529	1.74	0.61
rs17628268	80216	ALPK1	4	113688376	1.84E-02	2524	5.10E-01	Down	0.66	10780	1.74	0.03
rs1283652	284	ANGPT1	8	108436220	1.84E-02	2525	6.74E-20	Down	9.13	202	1.74	1.92
rs11102883	10100	TSPAN2	1	115324744	1.84E-02	2526	3.10E-09	Down	5.93	973	1.74	0.85
rs11230097	6947	TCN1	11	59402915	1.84E-02	2527	2.70E-01	Up	1.10	8956	1.74	0.06
rs2087176	26228	STAP1	4	68279068	1.84E-02	2528	3.38E-01	Up	0.96	9510	1.74	0.05
rs7248246	163071	ZNF114	19	53452154	1.84E-02	2529	4.47E-03	Down	2.84	3989	1.74	0.23
rs7248246	22900	CARD8	19	53452154	1.84E-02	2530	1.91E-02	Up	2.34	5026	1.74	0.17
rs6751246	1421	CRYGD	2	208827837	1.84E-02	2531	8.02E-03	Down	2.65	4344	1.73	0.21
rs6751246	1420	CRYGC	2	208827837	1.84E-02	2532	1.12E-01	Up	1.59	7176	1.73	0.10
rs3775464	22915	MMRN1	4	91183938	1.84E-02	2533	6.36E-01	Up	0.47	11677	1.73	0.02
rs10507	5292	PIM1	6	37250400	1.85E-02	2534	2.60E-04	Up	3.65	2726	1.73	0.36
rs10507	9361	LONP1	6	37250400	1.85E-02	2535	6.43E-01	Up	0.46	11729	1.73	0.02
rs11071981	8554	PIAS1	15	66203629	1.85E-02	2536	9.50E-04	Down	3.30	3199	1.73	0.30
rs9895634	8165	AKAP1	17	52563257	1.85E-02	2537	7.95E-03	Up	2.65	4337	1.73	0.21
rs9418387	221074	SLC39A12	10	18342198	1.85E-02	2538	4.55E-01	Up	0.75	10381	1.73	0.03
rs4984898	57799	RAB40C	16	581165	1.85E-02	2539	3.16E-03	Up	2.95	3805	1.73	0.25
rs4984898	9091	PIGQ	16	581165	1.85E-02	2540	3.68E-01	Up	0.90	9746	1.73	0.04
rs1888016	5079	PAX5	9	36948317	1.85E-02	2541	2.83E-01	Up	1.07	9053	1.73	0.05
rs4508853	54951	COMMD8	4	47326039	1.85E-02	2542	8.62E-01	Up	0.17	13020	1.73	0.01
rs11906462	253868	C20orf200	20	60569397	1.85E-02	2543	7.39E-01	Up	0.33	12309	1.73	0.01
rs7897621	51361	HOOK1	10	70726156	1.85E-02	2544	7.67E-06	Up	4.47	1893	1.73	0.51
rs7897621	3098	HK1	10	70726156	1.85E-02	2545	1.31E-01	Down	1.51	7454	1.73	0.09
rs7897621	3739	KCNA4	10	70726156	1.85E-02	2546	5.59E-01	Up	0.58	11153	1.73	0.03
rs17030583	7342	UBP1	3	33415322	1.85E-02	2547	2.73E-01	Up	1.10	8980	1.73	0.06
rs3136187	2072	ERCC4	16	13942000	1.85E-02	2548	5.13E-01	Up	0.65	10800	1.73	0.03
rs4549748	312	ANXA13	8	124812513	1.85E-02	2549	2.33E-02	Down	2.27	5218	1.73	0.16
rs7604809	9330	GTF3C3	2	197457878	1.86E-02	2550	9.00E-02	Up	1.70	6811	1.73	0.10
rs764457	57685	CACHD1	1	64693509	1.86E-02	2551	2.86E-07	Down	5.13	1412	1.73	0.65
rs8130475	56245	C21orf62	21	33114415	1.86E-02	2552	1.01E-01	Down	1.64	7009	1.73	0.10
rs6664413	23271	CAMSAP1L1	1	197423825	1.86E-02	2553	4.95E-01	Down	0.68	10682	1.73	0.03
rs2748243	6208	RPS14	5	149805311	1.87E-02	2554	3.48E-04	Up	3.58	2831	1.73	0.35
rs6436748	22938	SNW1	2	228720413	1.87E-02	2555	6.90E-02	Down	1.82	6411	1.73	0.12
rs30884	5145	PDE6A	5	149218109	1.87E-02	2556	3.14E-01	Up	1.01	9302	1.73	0.05
rs4805560	9745	ZNF536	19	35589705	1.87E-02	2557	2.95E-01	Up	1.05	9154	1.73	0.05
rs1266874	5314	PKHD1	6	51887597	1.87E-02	2558	8.40E-01	Down	0.20	12899	1.73	0.01
rs17176065	23140	ZZEF1	17	3908603	1.87E-02	2559	6.58E-01	Down	0.44	11820	1.73	0.02
rs1703467	26503	SLC17A5	6	74417416	1.87E-02	2560	6.73E-10	Up	6.17	869	1.73	0.92
rs3789607	54665	RSBN1	1	114078476	1.87E-02	2561	1.14E-05	Down	4.39	1974	1.73	0.49
rs3789607	26191	PTPN22	1	114078476	1.87E-02	2562	1.09E-03	Up	3.26	3254	1.73	0.30
rs7514676	58985	IL22RA1	1	24216562	1.87E-02	2563	1.36E-02	Up	2.47	4728	1.73	0.19
rs12607553	1825	DSC3	18	26841557	1.87E-02	2564	2.11E-04	Down	3.71	2649	1.73	0.37
rs2837253	5121	PCP4	21	40143126	1.87E-02	2565	1.38E-20	Down	9.30	185	1.73	1.99
rs9362387	1081	CGA	6	87850120	1.87E-02	2566	3.69E-01	Up	0.90	9758	1.73	0.04
rs17588812	257194	NEGR1	1	72300690	1.88E-02	2567	8.14E-06	Down	4.46	1906	1.73	0.51
rs1421602	10060	ABCC9	12	21952998	1.88E-02	2568	1.85E-01	Down	1.33	8087	1.73	0.07
rs2114039	5156	PDGFRA	4	54933554	1.88E-02	2569	6.38E-08	Down	5.41	1244	1.73	0.72
rs664725	2259	FGF14	13	101580902	1.88E-02	2570	7.21E-02	Down	1.80	6468	1.73	0.11
rs11714786	55214	LEPREL1	3	191276489	1.88E-02	2571	5.35E-07	Down	5.01	1488	1.73	0.63
rs12878879	2581	GALC	14	87548979	1.88E-02	2572	3.71E-03	Up	2.90	3888	1.73	0.24
rs12878879	8477	GPR65	14	87548979	1.88E-02	2573	1.84E-01	Up	1.33	8074	1.73	0.07
rs4679028	9390	SLC22A13	3	38302296	1.88E-02	2574	2.21E-01	Up	1.22	8472	1.72	0.07
rs13086588	7048	TGFBR2	3	30663761	1.88E-02	2575	1.41E-06	Down	4.82	1616	1.72	0.59
rs10883710	9079	LDB2	10	103875547	1.88E-02	2576	7.71E-04	Down	3.36	3122	1.72	0.31
rs10883710	23082	PPRC1	10	103875547	1.88E-02	2577	6.49E-01	Down	0.46	11768	1.72	0.02
rs10883710	8861	LDB1	10	103875547	1.88E-02	2578	8.94E-01	Up	0.13	13203	1.72	0.00
rs2075853	727897	MUC5B	11	1204034	1.89E-02	2579	7.95E-01	Down	0.26	12643	1.72	0.01
rs4843792	54971	BANP	16	86673631	1.89E-02	2580	5.09E-02	Up	1.95	6013	1.72	0.13
rs9830186	7104	TM4SF4	3	150667012	1.89E-02	2581	5.60E-04	Up	3.45	3001	1.72	0.33
rs11115	51073	MRPL4	19	10231542	1.89E-02	2582	4.59E-04	Up	3.50	2927	1.72	0.33
rs11115	3383	ICAM1	19	10231542	1.89E-02	2583	3.05E-02	Down	2.16	5481	1.72	0.15
rs7212403	162514	TRPV3	17	3376053	1.89E-02	2584	1.94E-01	Up	1.30	8192	1.72	0.07
rs1476853	26648	OR7E24	19	9206675	1.89E-02	2585	2.62E-01	Up	1.12	8881	1.72	0.06
rs7860616	84278	HIATL2	9	94345502	1.89E-02	2586	9.95E-01	Down	0.01	13880	1.72	0.00
rs2993066	115572	FAM46B	1	27042934	1.89E-02	2587	6.29E-06	Down	4.52	1854	1.72	0.52

rs7110845	25833	POU2F3	11	119680959	1.89E-02	2588	2.64E-03	Up	3.01	3716	1.72	0.26
rs11769362	7988	ZNF212	7	148384927	1.90E-02	2589	1.93E-01	Up	1.30	8186	1.72	0.07
rs4904723	145567	TTC7B	14	90256983	1.90E-02	2590	6.67E-02	Down	1.83	6372	1.72	0.12
rs2412143	114879	OSBPL5	11	3156422	1.90E-02	2591	6.32E-01	Down	0.48	11660	1.72	0.02
rs1196652	130574	LYPD6	2	150144424	1.90E-02	2592	9.61E-01	Up	0.05	13625	1.72	0.00
rs4926222	9524	GPSN2	19	14488050	1.90E-02	2593	4.94E-03	Down	2.81	4060	1.72	0.23
rs4926222	3337	DNAJB1	19	14488050	1.90E-02	2594	6.58E-01	Down	0.44	11818	1.72	0.02
rs2121021	55852	TEX2	17	59609061	1.90E-02	2595	1.86E-05	Up	4.28	2080	1.72	0.47
rs9493050	5169	ENPP3	6	132047018	1.91E-02	2596	2.89E-01	Up	1.06	9104	1.72	0.05
rs348365	79085	SLC25A23	19	6406414	1.91E-02	2597	3.52E-01	Up	0.93	9605	1.72	0.05
rs348365	92359	CRB3	19	6406414	1.91E-02	2598	3.60E-01	Up	0.91	9678	1.72	0.04
rs3790528	55225	RAVER2	1	64965298	1.91E-02	2599	1.82E-01	Down	1.33	8046	1.72	0.07
rs403321	8202	NCOA3	20	45729011	1.91E-02	2600	3.14E-01	Up	1.01	9309	1.72	0.05
rs3755120	51130	ASB3	2	53841506	1.91E-02	2601	2.60E-05	Down	4.21	2141	1.72	0.46
rs1474574	199870	FAM76A	1	27762996	1.91E-02	2602	7.30E-04	Down	3.38	3092	1.72	0.31
rs3936139	2788	GNG7	19	2538576	1.91E-02	2603	7.98E-01	Down	0.26	12666	1.72	0.01
rs17104645	10915	TCERG1	5	145795966	1.91E-02	2604	6.47E-01	Down	0.46	11759	1.72	0.02
rs10955984	6641	SNTB1	8	121736022	1.91E-02	2605	1.04E-02	Up	2.56	4538	1.72	0.20
rs7488783	65244	SPATS2	12	48046766	1.92E-02	2606	1.69E-13	Up	7.43	477	1.72	1.28
rs11044268	93661	CAPZA3	12	18770866	1.92E-02	2607	6.96E-01	Down	0.39	12041	1.72	0.02
rs6507162	25800	SLC39A6	18	31940201	1.92E-02	2608	1.60E-05	Up	4.31	2051	1.72	0.48
rs12900519	1143	CHRNB4	15	76736182	1.93E-02	2609	3.23E-02	Up	2.14	5536	1.71	0.15
rs3811831	57575	PCDH10	4	134424311	1.93E-02	2610	2.23E-01	Down	1.22	8489	1.71	0.07
rs2922666	5425	POLD2	7	43944445	1.93E-02	2611	-1.20E-13	Up	7.40	481	1.71	#NUM!
rs4308750	63898	SH2D4A	8	19304879	1.93E-02	2612	2.80E-02	Up	2.20	5392	1.71	0.16
rs17638888	8295	TRRAP	7	98142276	1.93E-02	2613	1.83E-04	Up	3.74	2597	1.71	0.37
rs7717650	10923	SUB1	5	32606701	1.93E-02	2614	3.41E-03	Up	2.93	3842	1.71	0.25
rs7717650	3475	IFRD1	5	32606701	1.93E-02	2615	5.08E-02	Up	1.95	6010	1.71	0.13
rs7717650	54760	PCSK4	5	32606701	1.93E-02	2616	5.26E-01	Down	0.63	10911	1.71	0.03
rs1786648	361	AQP4	18	22716609	1.93E-02	2617	8.21E-02	Down	1.74	6670	1.71	0.11
rs2306813	79605	PGBD5	1	226767814	1.94E-02	2618	6.42E-01	Up	0.47	11722	1.71	0.02
rs10025091	81608	FIP1L1	4	54105471	1.94E-02	2619	9.69E-01	Down	0.04	13693	1.71	0.00
rs424827	7447	VSNL1	2	17635428	1.95E-02	2620	5.22E-02	Down	1.94	6041	1.71	0.13
rs4683161	885	CCK	3	42295083	1.95E-02	2621	2.64E-10	Down	6.32	803	1.71	0.96
rs2306604	7019	TFAM	10	59818698	1.95E-02	2622	2.96E-07	Up	5.13	1414	1.71	0.65
rs2587507	57332	CBX8	17	75404730	1.95E-02	2623	8.78E-01	Up	0.15	13113	1.71	0.01
rs2289942	11227	GALNT5	2	157966225	1.95E-02	2624	5.41E-04	Down	3.46	2985	1.71	0.33
rs6564323	85445	CNTNAP4	16	74919514	1.95E-02	2625	4.83E-01	Up	0.70	10593	1.71	0.03
rs6696913	1733	DIO1	1	54098292	1.95E-02	2626	1.10E-01	Up	1.60	7142	1.71	0.10
rs6696913	11083	DIDO1	1	54098292	1.95E-02	2627	1.32E-01	Down	1.51	7465	1.71	0.09
rs2626345	245972	ATP6V0D2	8	87230811	1.95E-02	2628	4.24E-01	Down	0.80	10146	1.71	0.04
rs2797492	54906	C10orf18	10	5830622	1.95E-02	2629	3.10E-08	Up	5.53	1171	1.71	0.75
rs2502634	7402	UTRN	6	144679025	1.95E-02	2630	1.06E-24	Up	10.25	118	1.71	2.40
rs2701285	8825	LIN7A	12	79823819	1.95E-02	2631	1.32E-03	Up	3.21	3329	1.71	0.29
rs17405978	340419	RSPO2	8	109161717	1.95E-02	2632	4.34E-01	Down	0.78	10221	1.71	0.04
rs13250248	79648	MCPH1	8	6389775	1.95E-02	2633	2.35E-01	Up	1.19	8614	1.71	0.06
rs13250248	285	ANGPT2	8	6389775	1.95E-02	2634	3.66E-01	Down	0.90	9731	1.71	0.04
rs8181217	9350	CER1	9	14730682	1.96E-02	2635	2.70E-01	Down	1.10	8955	1.71	0.06
rs1377843	9666	DZIP3	3	109865450	1.96E-02	2636	4.02E-02	Down	2.05	5751	1.71	0.14
rs10893302	64221	ROBO3	11	124258026	1.96E-02	2637	5.57E-01	Down	0.59	11130	1.71	0.03
rs3764903	154881	KCTD7	7	65542632	1.96E-02	2638	2.75E-02	Down	2.20	5379	1.71	0.16
rs17068598	23143	LRCH1	13	46148801	1.96E-02	2639	9.55E-01	Down	0.06	13585	1.71	0.00
rs4836901	158131	OR1Q1	9	122454982	1.96E-02	2640	1.98E-01	Up	1.29	8241	1.71	0.07
rs194073	2561	GABRB2	5	160667218	1.96E-02	2641	6.39E-01	Down	0.47	11700	1.71	0.02
rs6496942	145858	C15orf32	15	90800097	1.97E-02	2642	5.43E-01	Up	0.61	11040	1.71	0.03
rs7296380	10985	GCN1L1	12	119072045	1.97E-02	2643	1.02E-06	Up	4.89	1575	1.71	0.60
rs9313634	8728	ADAM19	5	156897172	1.97E-02	2644	6.18E-06	Down	4.52	1850	1.71	0.52
rs12646523	9848	MFAP3L	4	171319428	1.97E-02	2645	5.75E-01	Down	0.56	11275	1.71	0.02
rs11213921	54766	BTG4	11	110851675	1.97E-02	2646	1.85E-01	Up	1.33	8086	1.71	0.07
rs3784191	9472	AKAP6	14	31993041	1.97E-02	2647	5.52E-01	Down	0.59	11096	1.71	0.03
rs10126378	7543	ZFX		23989410	1.97E-02	2648	2.30E-03	Down	3.05	3630	1.71	0.26
rs7140473	6655	SOS2	14	49746697	1.97E-02	2649	3.92E-01	Down	0.86	9929	1.71	0.04
rs10306202	5742	PTGS1	9	122239075	1.97E-02	2650	9.68E-07	Down	4.90	1572	1.70	0.60
rs12881778	55237	C14orf115	14	73874924	1.97E-02	2651	7.29E-01	Up	0.35	12248	1.70	0.01
rs1426199	399694	SHC4	15	46914426	1.98E-02	2652	6.00E-01	Up	0.52	11434	1.70	0.02
rs11196865	3983	ABLIM1	10	116427164	1.98E-02	2653	2.10E-07	Down	5.19	1378	1.70	0.67
rs4744437	84909	C9orf3	9	94912385	1.98E-02	2654	1.90E-21	Down	9.51	172	1.70	2.07
rs3829578	2034	EPAS1	17	50736509	1.98E-02	2655	5.42E-20	Down	9.16	199	1.70	1.93
rs3829578	3131	HLF	17	50736509	1.98E-02	2656	4.59E-14	Down	7.54	451	1.70	1.33
rs7787029	55112	WDR60	7	158246077	1.98E-02	2657	1.21E-01	Down	1.55	7317	1.70	0.09
rs10514525	3294	HSD17B2	16	80693012	1.98E-02	2658	2.45E-01	Down	1.16	8711	1.70	0.06
rs3218651	51426	POLK	3	122690866	1.98E-02	2659	5.98E-01	Down	0.53	11418	1.70	0.02
rs6903815	667	DST	6	56627418	1.98E-02	2660	2.06E-41	Down	13.48	15	1.70	4.07
rs678957	6327	SCN2B	11	117556065	1.99E-02	2661	4.83E-03	Down	2.82	4036	1.70	0.23
rs787182	79712	GTDC1	2	144566186	1.99E-02	2662	4.16E-03	Down	2.87	3958	1.70	0.24
rs12323563	11161	C14orf1	14	75178176	1.99E-02	2663	9.18E-02	Up	1.69	6857	1.70	0.10
rs1800562	3077	HFE	6	26201120	1.99E-02	2664	1.30E-09	Down	6.07	910	1.70	0.89
rs1800562	8364	HIST1H4C	6	26201120	1.99E-02	2665	1.48E-02	Down	2.44	4800	1.70	0.18
rs842639	5966	REL	2	61006896	1.99E-02	2666	2.26E-02	Down	2.28	5184	1.70	0.16
rs4512347	115908	CTHRC1	8	104473568	1.99E-02	2667	2.56E-07	Up	5.15	1398	1.70	0.66
rs4512347	81034	SLC25A32	8	104473568	1.99E-02	2668	2.93E-01	Up	1.05	9135	1.70	0.05

rs17125608	118427	OLFM3	1	102039164	1.99E-02	2669	7.58E-01	Up	0.31	12431	1.70	0.01
rs16942778	11057	ABHD2	15	87510142	1.99E-02	2670	5.87E-25	Up	10.33	115	1.70	2.42
rs12601255	376412	RNF126P1	17	52471040	1.99E-02	2671	3.54E-01	Up	0.93	9626	1.70	0.05
rs17243407	79072	FASTKD3	5	7911121	2.00E-02	2672	2.67E-02	Up	2.22	5351	1.70	0.16
rs17243407	4552	MTRR	5	7911121	2.00E-02	2673	6.40E-01	Down	0.47	11707	1.70	0.02
rs8089939	4645	MYO5B	18	45748391	2.00E-02	2674	6.91E-01	Down	0.40	12017	1.70	0.02
rs2822787	64092	SAMSN1	21	14876531	2.00E-02	2675	8.41E-01	Up	0.20	12906	1.70	0.01
rs12706065	22797	TFEC	7	115201446	2.00E-02	2676	5.63E-02	Up	1.91	6144	1.70	0.12
rs12215166	2978	GUCA1A	6	42212127	2.00E-02	2677	5.89E-03	Up	2.75	4163	1.70	0.22
rs1961995	55312	RFK	9	76222631	2.00E-02	2678	1.59E-10	Up	6.40	785	1.70	0.98
rs2177482	26610	ELP4	11	31758933	2.00E-02	2679	5.60E-02	Down	1.91	6134	1.70	0.13
rs2177482	5080	PAX6	11	31758933	2.00E-02	2680	4.74E-01	Down	0.72	10524	1.70	0.03
rs11078629	286753	TUSC5	17	1151162	2.00E-02	2681	7.98E-02	Down	1.75	6630	1.70	0.11
rs17318596	593	BCKDHA	19	46628935	2.00E-02	2682	9.60E-02	Up	1.66	6924	1.70	0.10
rs2665993	2125	EVPL	17	71520806	2.00E-02	2683	7.56E-03	Up	2.67	4310	1.70	0.21
rs2665993	1018	CDK3	17	71520806	2.00E-02	2684	1.82E-02	Up	2.36	4995	1.70	0.17
rs4661063	4914	NTRK1	1	153637874	2.01E-02	2685	5.00E-02	Down	1.96	5999	1.70	0.13
rs4661063	3645	INSRR	1	153637874	2.01E-02	2686	5.73E-01	Down	0.56	11265	1.70	0.02
rs860317	5504	PPP1R2	3	196745138	2.01E-02	2687	2.04E-08	Down	5.61	1127	1.70	0.77
rs642926	9663	LPIN2	18	2906054	2.01E-02	2688	1.80E-02	Up	2.36	4988	1.70	0.17
rs6492997	161829	EXDL1	15	39328463	2.01E-02	2689	7.05E-02	Down	1.81	6441	1.70	0.12
rs2836292	3772	KCNJ15	21	38584859	2.01E-02	2690	3.85E-05	Down	4.12	2220	1.70	0.44
rs10917619	339512	C1orf110	1	159567323	2.01E-02	2691	2.10E-02	Down	2.31	5118	1.70	0.17
rs3788530	11078	TRIOBP	22	36483241	2.01E-02	2692	2.57E-04	Down	3.66	2718	1.70	0.36
rs247615	9709	HERPUD1	16	55542264	2.02E-02	2693	3.63E-01	Down	0.91	9699	1.70	0.04
rs1490079	321	APBA2	15	27091095	2.02E-02	2694	9.46E-04	Down	3.31	3194	1.70	0.30
rs1578957	23505	TMEM131	2	97996951	2.02E-02	2695	1.56E-02	Up	2.42	4841	1.69	0.18
rs268594	23471	TRAM1	8	71675684	2.02E-02	2696	7.49E-01	Up	0.32	12372	1.69	0.01
rs17051960	8756	ADAM7	8	24347014	2.02E-02	2697	6.23E-06	Up	4.52	1852	1.69	0.52
rs4328485	3759	KCNJ2	17	65689360	2.02E-02	2698	7.48E-01	Down	0.32	12365	1.69	0.01
rs6787784	9045	RPL14	3	40461474	2.03E-02	2699	1.33E-18	Up	8.80	245	1.69	1.79
rs1800802	4256	MGP	12	14930186	2.03E-02	2700	3.83E-01	Up	0.87	9865	1.69	0.04
rs7550236	127435	PODN	1	53232147	2.03E-02	2701	6.56E-07	Down	4.97	1512	1.69	0.62
rs7550236	10106	CTDSP2	1	53232147	2.03E-02	2702	1.01E-04	Down	3.89	2457	1.69	0.40
rs7550236	10388	SYCP2	1	53232147	2.03E-02	2703	3.61E-04	Up	3.57	2848	1.69	0.34
rs7550236	6342	SCP2	1	53232147	2.03E-02	2704	5.64E-04	Up	3.45	3004	1.69	0.32
rs889090	57106	NAT14	19	60696713	2.04E-02	2705	2.72E-02	Down	2.21	5371	1.69	0.16
rs11994203	9788	MTSS1	8	125672479	2.04E-02	2706	2.48E-04	Down	3.66	2709	1.69	0.36
rs5757512	7441	VPREB1	22	20904868	2.04E-02	2707	3.88E-01	Up	0.86	9900	1.69	0.04
rs9965261	57536	KIAA1328	18	32899637	2.04E-02	2708	3.67E-01	Down	0.90	9739	1.69	0.04
rs17154444	1811	SLC26A3	7	107029819	2.04E-02	2709	7.43E-01	Up	0.33	12330	1.69	0.01
rs6813255	79071	ELOVL6	4	111430589	2.05E-02	2710	5.74E-01	Up	0.56	11268	1.69	0.02
rs3816605	23279	NUP160	11	47813829	2.05E-02	2711	5.63E-01	Up	0.58	11189	1.69	0.02
rs12033204	5525	PPP2R5A	1	208836521	2.05E-02	2712	9.66E-06	Down	4.42	1946	1.69	0.50
rs7521528	5087	PBX1	1	161469246	2.05E-02	2713	9.30E-52	Down	15.14	6	1.69	5.10
rs4789145	30833	NT5C	17	70646939	2.05E-02	2714	6.63E-06	Down	4.51	1866	1.69	0.52
rs4789145	51155	HN1	17	70646939	2.05E-02	2715	2.28E-02	Up	2.28	5188	1.69	0.16
rs4789145	79637	ARMC7	17	70646939	2.05E-02	2716	5.17E-01	Down	0.65	10839	1.69	0.03
rs2290851	23360	FNBP4	11	47709946	2.06E-02	2717	5.22E-10	Up	6.21	859	1.69	0.93
rs13424717	9655	SOCS5	2	46907544	2.06E-02	2718	1.85E-04	Down	3.74	2600	1.69	0.37
rs2290851	79841	AGBL2	11	47709946	2.06E-02	2719	8.43E-02	Down	1.73	6705	1.69	0.11
rs11953270	5098	PCDHGC3	5	140852461	2.06E-02	2720	9.00E-08	Up	5.35	1283	1.69	0.70
rs11953270	9708	PCDHGA8	5	140852461	2.06E-02	2721	5.15E-03	Up	2.80	4083	1.69	0.23
rs11953270	56107	PCDHGA9	5	140852461	2.06E-02	2722	8.87E-02	Down	1.70	6780	1.69	0.11
rs7255065	23476	BRD4	19	15264778	2.06E-02	2723	9.04E-01	Down	0.12	13276	1.69	0.00
rs6564079	83716	CRISPLD2	16	83398384	2.06E-02	2724	1.82E-15	Down	7.95	366	1.69	1.47
rs701232	3775	KCNK1	1	230098204	2.06E-02	2725	5.82E-02	Up	1.89	6194	1.69	0.12
rs2289263	4088	SMAD3	15	65226261	2.06E-02	2726	2.70E-23	Down	9.94	135	1.69	2.26
rs10853973	10501	SEMA6B	19	4506310	2.06E-02	2727	2.50E-01	Down	1.15	8760	1.69	0.06
rs10853973	116844	LRG1	19	4506310	2.06E-02	2728	6.37E-01	Down	0.47	11683	1.69	0.02
rs11867581	642	BLMH	17	25646354	2.06E-02	2729	2.41E-02	Down	2.26	5253	1.69	0.16
rs1867153	192683	SCAMP5	15	73058282	2.06E-02	2730	9.51E-01	Up	0.06	13557	1.69	0.00
rs8108236	3385	ICAM3	19	10326832	2.06E-02	2731	1.61E-14	Up	7.67	427	1.69	1.38
rs8108236	7297	TYK2	19	10326832	2.06E-02	2732	1.33E-05	Up	4.35	2016	1.69	0.49
rs10142131	53349	ZFYVE1	14	72537427	2.07E-02	2733	2.59E-01	Down	1.13	8855	1.68	0.06
rs6994461	8439	NSMAF	8	59729722	2.07E-02	2734	4.69E-14	Up	7.54	452	1.68	1.33
rs6504246	64750	SMURF2	17	60092969	2.07E-02	2735	1.73E-03	Up	3.13	3454	1.68	0.28
rs12650763	53343	NUDT9	4	88730642	2.07E-02	2736	1.45E-11	Up	6.75	650	1.68	1.08
rs2038531	123016	TTC8	14	88407089	2.07E-02	2737	4.81E-02	Down	1.98	5954	1.68	0.13
rs2800880	5906	RAP1A	1	111842666	2.07E-02	2738	3.20E-17	Down	8.44	297	1.68	1.65
rs3801210	2737	GLI3	7	41971800	2.07E-02	2739	2.14E-07	Down	5.19	1382	1.68	0.67
rs4559457	89866	SEC16B	1	174620265	2.07E-02	2740	1.04E-01	Up	1.62	7066	1.68	0.10
rs10882309	118924	C10orf4	10	95448862	2.08E-02	2741	4.76E-03	Down	2.82	4027	1.68	0.23
rs4542114	55558	PLXNA3		153272893	2.08E-02	2742	1.11E-04	Up	3.86	2481	1.68	0.40
rs4542114	8270	LAGE3		153272893	2.08E-02	2743	5.43E-04	Up	3.46	2987	1.68	0.33
rs4542114	8273	SLC10A3		153272893	2.08E-02	2744	8.96E-03	Down	2.61	4433	1.68	0.20
rs2471005	1812	DRD1	5	174814813	2.08E-02	2745	6.31E-01	Down	0.48	11648	1.68	0.02
rs9534021	55425	KIAA1704	13	44487902	2.08E-02	2746	7.98E-05	Down	3.95	2388	1.68	0.41
rs7644322	55975	KLHL7	3	184691439	2.08E-02	2747	9.00E-08	Up	5.34	1287	1.68	0.70
rs7644322	89857	KLHL6	3	184691439	2.08E-02	2748	3.10E-01	Up	1.01	9271	1.68	0.05
rs4821902	4248	MGAT3	22	38207844	2.08E-02	2749	8.53E-02	Up	1.72	6726	1.68	0.11

rs4821902	54471	SMCR7L	22	38207844	2.08E-02	2750	6.73E-01	Up	0.42	11900	1.68	0.02
rs10432174	84307	ZNF397	18	31100214	2.09E-02	2751	1.75E-01	Down	1.36	7966	1.68	0.08
rs13425206	4436	MSH2	2	47566495	2.09E-02	2752	4.32E-06	Up	4.60	1793	1.68	0.54
rs10042992	10605	PAIP1	5	43597516	2.09E-02	2753	3.34E-05	Up	4.15	2189	1.68	0.45
rs4259724	5778	PTPN7	1	198861663	2.09E-02	2754	4.16E-01	Down	0.81	10098	1.68	0.04
rs2505515	5979	RET	10	42925592	2.09E-02	2755	6.98E-02	Up	1.81	6419	1.68	0.12
rs3761543	7454	WAS		48310887	2.09E-02	2756	1.53E-01	Down	1.43	7726	1.68	0.08
rs3761543	6839	SUV39H1		48310887	2.09E-02	2757	4.17E-01	Down	0.81	10099	1.68	0.04
rs12470997	79582	SPAG16	2	215087268	2.09E-02	2758	2.84E-02	Up	2.19	5407	1.68	0.15
rs2554430	6047	RNF4	15	22745575	2.10E-02	2759	3.29E-02	Up	2.13	5560	1.68	0.15
rs2554430	8926	SNURF	15	22745575	2.10E-02	2760	4.45E-01	Down	0.76	10305	1.68	0.04
rs2016465	55824	PAG1	8	82067952	2.10E-02	2761	9.25E-02	Down	1.68	6871	1.68	0.10
rs2269368	5511	PPP1R8		152710666	2.10E-02	2762	1.02E-01	Up	1.63	7037	1.68	0.10
rs2269368	393	ARHGAP4		152710666	2.10E-02	2763	9.67E-01	Down	0.04	13675	1.68	0.00
rs11746118	9509	ADAMTS2	5	178561857	2.10E-02	2764	2.49E-01	Up	1.15	8756	1.68	0.06
rs1876680	3003	GZMK	5	54370181	2.10E-02	2765	5.38E-03	Up	2.78	4110	1.68	0.23
rs1177298	84542	KIAA1841	2	61218405	2.10E-02	2766	1.97E-02	Down	2.33	5058	1.68	0.17
rs7552536	2848	GPR25	1	197568523	2.10E-02	2767	3.34E-01	Down	0.97	9473	1.68	0.05
rs7794389	257415	FAM133B	7	91850009	2.10E-02	2768	2.11E-02	Up	2.31	5121	1.68	0.17
rs7688715	6002	RGS12	4	3379106	2.10E-02	2769	2.59E-04	Down	3.65	2720	1.68	0.36
rs997250	80055	PGAP1	4	15415767	2.11E-02	2770	8.11E-06	Down	4.46	1905	1.68	0.51
rs997250	683	BST1	4	15415767	2.11E-02	2771	9.26E-03	Down	2.60	4450	1.68	0.20
rs12422778	3742	KCNA6	12	4772785	2.11E-02	2772	3.42E-01	Up	0.95	9544	1.68	0.05
rs8065977	10216	PRG4	17	72954962	2.11E-02	2773	3.91E-05	Up	4.11	2225	1.68	0.44
rs7960623	29953	TRHDE	12	70942192	2.11E-02	2774	4.66E-01	Down	0.73	10473	1.68	0.03
rs6512005	126402	CCDC105	19	14998243	2.11E-02	2775	1.21E-01	Up	1.55	7312	1.68	0.09
rs1866739	9312	KCNB2	8	73865386	2.11E-02	2776	8.63E-01	Up	0.17	13023	1.68	0.01
rs2889010	80095	ZNF606	19	63173692	2.11E-02	2777	6.31E-04	Up	3.42	3048	1.68	0.32
rs2889010	147685	C19orf18	19	63173692	2.11E-02	2778	3.37E-03	Up	2.93	3837	1.68	0.25
rs210432	26280	IL1RAPL2		104753441	2.11E-02	2779	5.14E-01	Down	0.65	10813	1.67	0.03
rs3756772	2444	FRK	6	116431835	2.11E-02	2780	1.97E-03	Up	3.09	3524	1.67	0.27
rs1990525	114905	C1QTNF7	4	15097292	2.11E-02	2781	1.65E-01	Down	1.39	7867	1.67	0.08
rs5933828	1183	CLCN4		10022129	2.12E-02	2782	2.37E-02	Up	2.26	5234	1.67	0.16
rs9954287	125704	C18orf51	18	70248446	2.12E-02	2783	3.37E-01	Down	0.96	9502	1.67	0.05
rs7280622	10281	DSCR4	21	38338876	2.12E-02	2784	1.28E-01	Up	1.52	7422	1.67	0.09
rs2276932	79658	ARHGAP10	4	149341926	2.12E-02	2785	6.19E-03	Down	2.74	4185	1.67	0.22
rs9440	55249	YY1AP1	11	1447672	2.12E-02	2786	4.56E-03	Down	2.84	4005	1.67	0.23
rs510834	25850	ZNF345	19	42067340	2.12E-02	2787	4.55E-01	Up	0.75	10384	1.67	0.03
rs9573577	170622	COMMD6	13	75001790	2.12E-02	2788	2.45E-06	Down	4.71	1700	1.67	0.56
rs7007715	5179	PENK	8	57511610	2.13E-02	2789	6.55E-02	Down	1.84	6348	1.67	0.12
rs4346160	51324	SPG21	15	63051804	2.13E-02	2790	3.98E-09	Up	5.91	984	1.67	0.84
rs1373292	4751	NEK2	1	208222321	2.13E-02	2791	2.64E-01	Down	1.12	8904	1.67	0.06
rs578268	2625	GATA3	10	8123894	2.13E-02	2792	3.68E-25	Down	10.36	112	1.67	2.44
rs6534216	11107	PRDM5	4	122123132	2.13E-02	2793	3.36E-01	Down	0.96	9489	1.67	0.05
rs1844358	79752	ZFAND1	8	82801044	2.13E-02	2794	3.39E-04	Up	3.58	2821	1.67	0.35
rs1844358	92421	CHMP4C	8	82801044	2.13E-02	2795	3.42E-03	Up	2.93	3844	1.67	0.25
rs7844465	22882	ZHX2	8	124049878	2.13E-02	2796	5.38E-02	Down	1.93	6084	1.67	0.13
rs8136143	25814	ATXN10	22	44530897	2.13E-02	2797	3.30E-01	Up	0.97	9444	1.67	0.05
rs862991	80199	FUZ	1	155977437	2.13E-02	2798	4.16E-01	Up	0.81	10092	1.67	0.04
rs11743792	9456	HOMER1	5	78702269	2.13E-02	2799	4.38E-04	Up	3.52	2910	1.67	0.34
rs8107849	10172	ZNF256	19	63160968	2.14E-02	2800	4.52E-01	Down	0.75	10362	1.67	0.03
rs6014711	1477	CSTF1	20	54394480	2.14E-02	2801	2.42E-01	Up	1.17	8674	1.67	0.06
rs10423006	30846	EHD2	19	52891156	2.14E-02	2802	3.72E-13	Down	7.27	519	1.67	1.24
rs10423006	29998	GLTSCR1	19	52891156	2.14E-02	2803	2.11E-01	Down	1.25	8357	1.67	0.07
rs12619026	129880	BBS5	2	170180297	2.14E-02	2804	6.73E-01	Down	0.42	11907	1.67	0.02
rs12619026	10324	KBTBD10	2	170180297	2.14E-02	2805	9.10E-01	Down	0.11	13313	1.67	0.00
rs12028511	23252	OTUD3	1	19992980	2.14E-02	2806	1.01E-04	Down	3.89	2454	1.67	0.40
rs12028511	30814	PLA2G2E	1	19992980	2.14E-02	2807	7.55E-01	Down	0.31	12408	1.67	0.01
rs214246	8312	AXIN1	16	289294	2.15E-02	2808	1.43E-03	Up	3.19	3356	1.67	0.28
rs2028241	23429	RYBP	3	72595592	2.15E-02	2809	9.04E-01	Down	0.12	13274	1.67	0.00
rs12539223	28969	BZW2	7	16511246	2.15E-02	2810	2.04E-04	Up	3.71	2638	1.67	0.37
rs10066266	154	ADRB2	5	148196512	2.15E-02	2811	1.47E-15	Up	7.94	370	1.67	1.48
rs3217751	10044	SH2D3C	9	127632469	2.15E-02	2812	7.81E-02	Down	1.76	6596	1.67	0.11
rs3217751	1025	CDK9	9	127632469	2.15E-02	2813	8.95E-02	Down	1.70	6794	1.67	0.10
rs3217751	2356	FPGS	9	127632469	2.15E-02	2814	7.65E-01	Up	0.30	12459	1.67	0.01
rs2772548	3590	IL11RA	9	34660988	2.15E-02	2815	1.30E-02	Down	2.48	4687	1.67	0.19
rs2772548	10850	CCL27	9	34660988	2.15E-02	2816	8.86E-01	Up	0.14	13166	1.67	0.01
rs6062014	6874	TAF4	20	60083924	2.15E-02	2817	5.43E-03	Up	2.78	4121	1.67	0.23
rs16949649	4830	NME1	17	46585307	2.15E-02	2818	4.46E-29	Up	11.23	70	1.67	2.84
rs16949649	4831	NME2	17	46585307	2.15E-02	2819	4.25E-19	Up	8.93	226	1.67	1.84
rs2299047	3720	JARID2	6	15551440	2.15E-02	2820	5.56E-02	Up	1.91	6130	1.67	0.13
rs683969	2650	GCNT1	9	76349306	2.15E-02	2821	4.20E-26	Up	10.57	95	1.67	2.54
rs7078013	3778	KCNMA1	10	78308963	2.15E-02	2822	2.89E-06	Down	4.68	1729	1.67	0.55
rs7903424	6934	TCF7L2	10	114894026	2.16E-02	2823	1.68E-01	Down	1.38	7889	1.67	0.08
rs6932636	55809	TRERF1	6	42324416	2.16E-02	2824	4.08E-08	Down	5.49	1205	1.67	0.74
rs940800	4698	NDUFA5	7	122804575	2.16E-02	2825	3.90E-01	Up	0.86	9920	1.67	0.04
rs10772662	359787	DPPA3	12	7760305	2.16E-02	2826	5.75E-02	Up	1.90	6172	1.67	0.12
rs3811986	51301	GCNT4	5	74360193	2.16E-02	2827	6.81E-01	Up	0.41	11954	1.67	0.02
rs2633418	58158	NEUROD4	12	53715085	2.16E-02	2828	8.41E-01	Up	0.20	12909	1.66	0.01
rs4749955	84991	RBM17	10	6158972	2.16E-02	2829	7.84E-01	Down	0.27	12572	1.66	0.01
rs875495	252839	TMEM9	1	197869157	2.16E-02	2830	9.86E-05	Up	3.89	2449	1.66	0.40

rs7331784	8881	CDC16	13	114013597	2.17E-02	2831	3.48E-03	Up	2.92	3855	1.66	0.25
rs3824369	123	ADFP	9	19116565	2.17E-02	2832	1.40E-03	Down	3.20	3347	1.66	0.29
rs2148634	5738	PTGFRN	1	117185041	2.17E-02	2833	1.26E-01	Down	1.53	7379	1.66	0.09
rs17182151	6035	RNASE1	14	20348310	2.17E-02	2834	9.75E-02	Down	1.66	6951	1.66	0.10
rs890432	3213	HOXB3	17	43984231	2.17E-02	2835	5.23E-01	Down	0.64	10886	1.66	0.03
rs6490971	479	ATP12A	13	24133807	2.18E-02	2836	4.24E-01	Down	0.80	10153	1.66	0.04
rs2236963	9254	CACNA2D2	3	50423636	2.18E-02	2837	3.49E-04	Up	3.58	2832	1.66	0.35
rs9876768	23228	PLCL2	3	17024969	2.18E-02	2838	4.97E-06	Down	4.57	1817	1.66	0.53
rs11026691	7748	ZNF195	11	3331321	2.18E-02	2839	1.28E-05	Up	4.36	2009	1.66	0.49
rs544143	22941	SHANK2	11	70164258	2.18E-02	2840	4.80E-08	Up	5.46	1218	1.66	0.73
rs6943220	9808	KIAA0087	7	26339506	2.18E-02	2841	6.43E-05	Up	4.00	2344	1.66	0.42
rs9561641	160897	GPR180	13	94048529	2.18E-02	2842	1.79E-01	Up	1.34	8013	1.66	0.07
rs9561641	23483	TGDS	13	94048529	2.18E-02	2843	6.14E-01	Up	0.50	11531	1.66	0.02
rs12019764	55901	THSD1	13	51866405	2.18E-02	2844	4.20E-05	Up	4.10	2241	1.66	0.44
rs3740713	3948	LDHC	11	18407672	2.18E-02	2845	1.82E-01	Down	1.34	8044	1.66	0.07
rs6491343	10150	MBNL2	13	96701740	2.18E-02	2846	1.18E-28	Down	11.11	74	1.66	2.79
rs2966312	23406	COTL1	16	83164493	2.18E-02	2847	6.90E-02	Down	1.82	6409	1.66	0.12
rs7902688	219621	C10orf107	10	63177853	2.19E-02	2848	2.16E-01	Down	1.24	8425	1.66	0.07
rs10749624	57604	C8orf79	8	12836723	2.19E-02	2849	9.69E-01	Up	0.04	13692	1.66	0.00
rs4715167	221692	PHACTR1	6	13328301	2.19E-02	2850	6.44E-01	Down	0.46	11738	1.66	0.02
rs10972048	318	NUDT2	9	34300927	2.19E-02	2851	2.70E-01	Up	1.10	8957	1.66	0.06
rs1790733	5499	PPP1CA	11	66942576	2.19E-02	2852	6.40E-08	Up	5.41	1250	1.66	0.72
rs1790733	6199	RPS6KB2	11	66942576	2.19E-02	2853	3.26E-03	Up	2.94	3824	1.66	0.25
rs1790733	374403	TBC1D10C	11	66942576	2.19E-02	2854	2.67E-01	Down	1.11	8927	1.66	0.06
rs9614616	10762	NUP50	22	43904449	2.19E-02	2855	7.99E-04	Up	3.35	3137	1.66	0.31
rs9614616	23313	C22orf9	22	43904449	2.19E-02	2856	1.82E-02	Down	2.36	4997	1.66	0.17
rs1042427	2766	GMPR	6	16403534	2.19E-02	2857	1.56E-02	Down	2.42	4843	1.66	0.18
rs639500	255231	MCOLN2	1	85121057	2.20E-02	2858	1.57E-02	Up	2.42	4862	1.66	0.18
rs4790611	7326	UBE2G1	17	4172052	2.20E-02	2859	9.74E-07	Up	4.90	1573	1.66	0.60
rs16963927	6352	CCL5	17	31228920	2.20E-02	2860	2.87E-01	Down	1.06	9093	1.66	0.05
rs2236944	2771	GNAI2	3	50267197	2.20E-02	2861	8.69E-10	Down	6.13	883	1.66	0.91
rs2236944	7869	SEMA3B	3	50267197	2.20E-02	2862	7.38E-02	Down	1.79	6502	1.66	0.11
rs1046648	55769	ZNF83	19	57807872	2.20E-02	2863	6.73E-01	Down	0.42	11906	1.66	0.02
rs3113252	9255	SCYE1	4	107610006	2.20E-02	2864	3.61E-01	Up	0.91	9684	1.66	0.04
rs1145101	10274	STAG1	3	137551984	2.20E-02	2865	2.20E-01	Down	1.23	8464	1.66	0.07
rs2728770	56852	RAD18	3	8989942	2.20E-02	2866	1.19E-01	Up	1.56	7278	1.66	0.09
rs9563521	27253	PCDH17	13	57152774	2.20E-02	2867	1.20E-01	Up	1.56	7290	1.66	0.09
rs10227466	26031	OSBPL3	7	24616861	2.20E-02	2868	6.66E-01	Up	0.43	11859	1.66	0.02
rs17175810	161882	ZFPM1	16	87072758	2.20E-02	2869	5.63E-01	Down	0.58	11183	1.66	0.02
rs13396065	50940	PDE11A	2	178465313	2.21E-02	2870	7.01E-07	Down	4.96	1522	1.66	0.62
rs6554653	1501	CTNND2	5	11923138	2.21E-02	2871	3.82E-10	Up	6.26	830	1.66	0.94
rs1517914	55843	ARHGAP15	2	144234280	2.21E-02	2872	6.67E-04	Down	3.40	3062	1.66	0.32
rs4668121	4036	LRP2	2	169801612	2.21E-02	2873	3.06E-01	Up	1.02	9237	1.66	0.05
rs6933329	11231	SEC63	6	108296104	2.21E-02	2874	2.97E-01	Up	1.04	9177	1.66	0.05
rs964997	114795	TMEM132B	12	124528770	2.21E-02	2875	9.43E-01	Up	0.07	13508	1.66	0.00
rs7031902	7357	UGCG	9	111784692	2.21E-02	2876	4.40E-11	Up	6.59	705	1.66	1.04
rs12331490	153020	RASGEF1B	4	82704014	2.21E-02	2877	3.82E-01	Up	0.87	9854	1.66	0.04
rs12897976	4140	MARK3	14	102962763	2.21E-02	2878	2.56E-02	Up	2.23	5307	1.66	0.16
rs11189211	5223	PGAM1	10	99163860	2.21E-02	2879	9.11E-06	Down	4.44	1928	1.65	0.50
rs10998985	1305	COL13A1	10	71248589	2.21E-02	2880	2.58E-03	Down	3.01	3703	1.65	0.26
rs6451722	23530	NNT	5	43747135	2.21E-02	2881	1.07E-17	Down	8.57	280	1.65	1.70
rs521040	160364	CLEC12A	12	10039117	2.22E-02	2882	2.28E-01	Up	1.20	8543	1.65	0.06
rs521040	51266	CLEC1B	12	10039117	2.22E-02	2883	9.90E-01	Up	0.01	13840	1.65	0.00
rs7680420	4306	NR3C2	4	149614719	2.22E-02	2884	1.22E-04	Down	3.84	2497	1.65	0.39
rs9875109	6498	SKIL	3	171547478	2.22E-02	2885	1.41E-02	Up	2.45	4760	1.65	0.19
rs11573051	3490	IGFBP7	4	57783767	2.22E-02	2886	2.31E-03	Down	3.05	3638	1.65	0.26
rs1990761	2641	GCG	2	162823210	2.22E-02	2887	2.26E-01	Up	1.21	8523	1.65	0.06
rs4111129	23077	MYCBP2	13	76817834	2.22E-02	2888	3.14E-09	Down	5.92	974	1.65	0.85
rs894939	146664	MGAT5B	17	72382392	2.22E-02	2889	1.96E-01	Down	1.29	8210	1.65	0.07
rs749457	151	ADRA2B	2	96217818	2.22E-02	2890	6.01E-01	Down	0.52	11438	1.65	0.02
rs7036106	8328	GFI1B	9	132880128	2.22E-02	2891	1.23E-01	Down	1.54	7343	1.65	0.09
rs2288904	57153	SLC44A2	19	10603170	2.22E-02	2892	1.55E-03	Down	3.17	3399	1.65	0.28
rs3760269	9120	SLC16A6	17	63800636	2.22E-02	2893	2.77E-03	Up	2.99	3746	1.65	0.26
rs3760269	22901	ARSG	17	63800636	2.22E-02	2894	2.83E-01	Up	1.07	9060	1.65	0.05
rs7968920	8738	CRADD	12	92736221	2.22E-02	2895	9.01E-02	Down	1.69	6817	1.65	0.10
rs7298686	9573	GDF3	12	7749369	2.22E-02	2896	6.28E-01	Down	0.48	11640	1.65	0.02
rs11844189	5729	PTGDR	14	51795595	2.23E-02	2897	1.86E-01	Up	1.32	8102	1.65	0.07
rs8179271	55870	ASH1L	1	152349811	2.23E-02	2898	5.00E-01	Down	0.67	10714	1.65	0.03
rs7164883	10021	HCN4	15	71439227	2.24E-02	2899	7.19E-01	Down	0.36	12193	1.65	0.01
rs7216307	2896	GRN	17	39769007	2.24E-02	2900	6.37E-06	Up	4.51	1859	1.65	0.52
rs7216307	51629	SLC25A39	17	39769007	2.24E-02	2901	4.18E-05	Up	4.10	2239	1.65	0.44
rs134559	83999	KREMEN1	22	27782995	2.24E-02	2902	1.77E-02	Down	2.37	4971	1.65	0.18
rs134559	25770	C22orf31	22	27782995	2.24E-02	2903	2.03E-01	Down	1.27	8283	1.65	0.07
rs2787328	80709	AKNA	9	114228772	2.24E-02	2904	2.65E-01	Down	1.11	8907	1.65	0.06
rs681827	9515	STXBP5L	3	122171440	2.24E-02	2905	8.35E-02	Down	1.73	6692	1.65	0.11
rs17499109	11021	RAB35	12	119004290	2.24E-02	2906	8.03E-01	Down	0.25	12695	1.65	0.01
rs231485	4355	MPP2	17	39337538	2.24E-02	2907	5.21E-08	Down	5.44	1227	1.65	0.73
rs231485	2305	FOXM1	17	39337538	2.24E-02	2908	2.76E-07	Up	5.14	1407	1.65	0.66
rs12294367	797	CALCB	11	15038592	2.24E-02	2909	4.02E-01	Up	0.84	9993	1.65	0.04
rs17691077	11127	KIF3A	5	132071250	2.24E-02	2910	5.50E-01	Up	0.60	11078	1.65	0.03
rs13263714	115111	SLC26A7	8	92382558	2.25E-02	2911	4.94E-01	Down	0.68	10671	1.65	0.03

rs1449562	3242	HPD	12	120750605	2.25E-02	2912	5.59E-01	Up	0.58	11160	1.65	0.03
rs3782860	6540	SLC6A13	12	232257	2.25E-02	2913	7.52E-01	Down	0.32	12391	1.65	0.01
rs531805	91949	COG7	16	23373536	2.25E-02	2914	3.21E-01	Down	0.99	9364	1.65	0.05
rs7182648	84952	CGNL1	15	55518865	2.25E-02	2915	6.69E-01	Down	0.43	11882	1.65	0.02
rs727143	132989	C4orf36	4	88150725	2.25E-02	2916	6.76E-01	Down	0.42	11923	1.65	0.02
rs4851758	79074	C2orf49	2	105410935	2.25E-02	2917	2.75E-01	Down	1.09	8994	1.65	0.06
rs34514	5347	PLK1	16	23578098	2.25E-02	2918	6.44E-02	Up	1.85	6332	1.65	0.12
rs646384	28992	MACROD1	11	63632032	2.25E-02	2919	1.14E-03	Up	3.25	3272	1.65	0.29
rs646384	23769	FLRT1	11	63632032	2.25E-02	2920	2.48E-03	Down	3.03	3680	1.65	0.26
rs12193812	8464	SUPT3H	6	45248739	2.26E-02	2921	3.33E-04	Down	3.59	2813	1.65	0.35
rs6672200	374986	FAM73A	1	77937901	2.26E-02	2922	6.50E-03	Up	2.72	4211	1.65	0.22
rs10229706	219578	ZNF804B	7	88268171	2.26E-02	2923	1.79E-01	Down	1.34	8014	1.65	0.07
rs1369821	59277	NTN4	12	94701962	2.26E-02	2924	1.43E-10	Down	6.41	776	1.65	0.98
rs194545	51108	METTL9	16	21512681	2.26E-02	2925	9.59E-03	Up	2.59	4475	1.65	0.20
rs310712	6419	SETMAR	3	4317353	2.27E-02	2926	3.09E-01	Up	1.02	9260	1.64	0.05
rs17752593	327657	SERPINA9	14	94007781	2.27E-02	2927	1.23E-01	Up	1.54	7349	1.64	0.09
rs706716	5295	PIK3R1	5	67584198	2.27E-02	2928	2.13E-18	Down	8.75	256	1.64	1.77
rs8080321	23326	USP22	17	20895327	2.27E-02	2929	1.34E-05	Up	4.35	2018	1.64	0.49
rs135170	51512	GTSE1	22	44995122	2.27E-02	2930	4.31E-01	Up	0.79	10204	1.64	0.04
rs135170	10343	PKDREJ	22	44995122	2.27E-02	2931	9.38E-01	Down	0.08	13481	1.64	0.00
rs12602297	201305	SPNS3	17	4341639	2.27E-02	2932	8.96E-02	Down	1.70	6798	1.64	0.10
rs1495956	2702	GJA5	1	144463302	2.27E-02	2933	4.11E-01	Up	0.82	10060	1.64	0.04
rs3828886	10866	HCP5	6	31548531	2.27E-02	2934	2.16E-03	Up	3.07	3584	1.64	0.27
rs1546424	196951	C15orf33	15	47610519	2.27E-02	2935	1.72E-02	Up	2.38	4949	1.64	0.18
rs2278842	282991	BLOC1S2	10	102046735	2.27E-02	2936	4.16E-01	Up	0.81	10093	1.64	0.04
rs2278842	9033	PKD2L1	10	102046735	2.27E-02	2937	6.11E-01	Up	0.51	11510	1.64	0.02
rs3799696	2729	GCLC	6	53504855	2.27E-02	2938	3.00E-07	Down	5.12	1418	1.64	0.65
rs6691985	9696	CROCC	1	17035217	2.28E-02	2939	4.79E-01	Down	0.71	10568	1.64	0.03
rs9368603	4739	NEDD9	6	11284020	2.28E-02	2940	4.94E-01	Up	0.68	10670	1.64	0.03
rs3849994	4004	LMO1	11	8218977	2.28E-02	2941	2.97E-01	Down	1.04	9168	1.64	0.05
rs2078385	10873	ME3	11	85845992	2.28E-02	2942	2.52E-02	Down	2.24	5295	1.64	0.16
rs9921255	79068	FTO	16	52566829	2.29E-02	2943	1.05E-06	Down	4.88	1579	1.64	0.60
rs12805353	4647	MYO7A	11	76576445	2.29E-02	2944	3.15E-01	Down	1.01	9314	1.64	0.05
rs6509881	3904	LAIR2	19	59709277	2.29E-02	2945	9.17E-01	Down	0.10	13356	1.64	0.00
rs6052778	23627	PRND	20	4637236	2.29E-02	2946	3.92E-04	Up	3.55	2875	1.64	0.34
rs6052778	5621	PRNP	20	4637236	2.29E-02	2947	1.10E-03	Down	3.26	3259	1.64	0.30
rs2241581	85007	AGXT2L2	5	177601488	2.29E-02	2948	1.18E-07	Up	5.30	1312	1.64	0.69
rs12026324	6913	TBX15	1	119158752	2.30E-02	2949	2.43E-01	Down	1.17	8688	1.64	0.06
rs12493550	170572	HTR3C	3	185235475	2.30E-02	2950	6.97E-01	Up	0.39	12049	1.64	0.02
rs4927072	51253	MRPL37	1	54392079	2.30E-02	2951	6.65E-09	Up	5.80	1030	1.64	0.82
rs9932459	863	CBFA2T3	16	87554829	2.30E-02	2952	2.03E-01	Up	1.27	8290	1.64	0.07
rs2734700	1179	CLCA1	1	86662341	2.31E-02	2953	4.83E-01	Up	0.70	10597	1.64	0.03
rs1511695	25782	RAB3GAP2	1	216836725	2.31E-02	2954	8.67E-01	Up	0.17	13044	1.64	0.01
rs1004764	80115	BAIAP2L2	22	36799352	2.31E-02	2955	4.89E-01	Up	0.69	10644	1.64	0.03
rs1004764	23539	SLC16A8	22	36799352	2.31E-02	2956	6.06E-01	Down	0.52	11474	1.64	0.02
rs7246405	83483	PLVAP	19	17320425	2.31E-02	2957	1.59E-02	Down	2.41	4880	1.64	0.18
rs7246405	84705	GTPBP3	19	17320425	2.31E-02	2958	8.43E-01	Down	0.20	12917	1.64	0.01
rs4961719	54796	BNC2	9	16472083	2.31E-02	2959	1.12E-13	Down	7.43	478	1.64	1.30
rs178228	4857	NOVA1	14	25992010	2.31E-02	2960	4.37E-01	Down	0.78	10253	1.64	0.04
rs2511837	4162	MCAM	11	118684217	2.31E-02	2961	1.54E-11	Down	6.74	657	1.64	1.08
rs2511837	867	CBL	11	118684217	2.31E-02	2962	3.05E-02	Up	2.16	5482	1.64	0.15
rs698475	5887	RAD23B	9	107179093	2.31E-02	2963	1.29E-11	Up	6.78	643	1.64	1.09
rs10164423	55854	ZC3H15	2	187194239	2.31E-02	2964	7.46E-01	Down	0.32	12351	1.64	0.01
rs11726949	81579	PLA2G12A	4	111022234	2.31E-02	2965	6.53E-04	Up	3.41	3058	1.64	0.32
rs11726949	2694	GIF	4	111022234	2.31E-02	2966	2.86E-01	Down	1.07	9084	1.64	0.05
rs1265761	10665	C6orf10	6	32429575	2.31E-02	2967	4.35E-01	Up	0.78	10231	1.64	0.04
rs7211024	58488	PCTP	17	51214837	2.31E-02	2968	1.33E-05	Up	4.35	2015	1.64	0.49
rs10852636	84464	BTBD12	16	3616311	2.31E-02	2969	2.86E-01	Down	1.07	9082	1.64	0.05
rs959354	334	APLP2	11	129514791	2.31E-02	2970	1.32E-07	Up	5.27	1323	1.64	0.69
rs10498245	11262	SP140	2	230936927	2.31E-02	2971	2.69E-02	Down	2.21	5356	1.64	0.16
rs222972	116159	CYYR1	21	26800448	2.31E-02	2972	5.15E-01	Down	0.65	10820	1.64	0.03
rs13148572	23244	PDS5A	4	39781188	2.32E-02	2973	2.40E-04	Up	3.67	2694	1.64	0.36
rs1108864	340273	ABCB5	7	20466782	2.32E-02	2974	2.01E-02	Up	2.33	5076	1.64	0.17
rs1111761	90141	C14orf143	14	89346475	2.32E-02	2975	7.40E-08	Up	5.38	1264	1.64	0.71
rs3754162	57134	MAN1C1	1	25634819	2.32E-02	2976	4.44E-02	Down	2.01	5861	1.64	0.14
rs1033962	56952	PRTFDC1	10	25256812	2.32E-02	2977	7.09E-02	Down	1.81	6450	1.63	0.11
rs1508335	8987	STBD1	4	77575419	2.32E-02	2978	3.52E-01	Up	0.93	9606	1.63	0.05
rs12363800	2977	GUCY1A2	11	106238967	2.32E-02	2979	1.59E-01	Up	1.41	7797	1.63	0.08
rs4814547	6629	SNRPB2	20	16646800	2.32E-02	2980	1.08E-02	Up	2.55	4564	1.63	0.20
rs2026092	8028	MLLT10	10	21951663	2.33E-02	2981	9.49E-01	Up	0.06	13544	1.63	0.00
rs2373530	8491	MAP4K3	2	39517568	2.33E-02	2982	1.15E-01	Up	1.57	7221	1.63	0.09
rs1363448	56106	PCDHGA10	5	140763780	2.33E-02	2983	1.53E-02	Up	2.43	4825	1.63	0.18
rs680545	3796	KIF2A	2	74986910	2.33E-02	2984	1.56E-07	Down	5.25	1346	1.63	0.68
rs680545	29911	HOOK2	2	74986910	2.33E-02	2985	1.20E-03	Up	3.24	3289	1.63	0.29
rs680545	3741	KCNA5	2	74986910	2.33E-02	2986	1.52E-01	Down	1.43	7704	1.63	0.08
rs680545	3099	HK2	2	74986910	2.33E-02	2987	3.77E-01	Up	0.88	9817	1.63	0.04
rs7201958	2013	EMP2	16	10557853	2.33E-02	2988	4.55E-13	Down	7.24	525	1.63	1.23
rs945508	149499	C1orf92	1	153720154	2.33E-02	2989	2.42E-01	Down	1.17	8677	1.63	0.06
rs945508	9826	ARHGEF11	1	153720154	2.33E-02	2990	8.21E-01	Up	0.23	12793	1.63	0.01
rs1745	7110	TMF1	3	69153147	2.33E-02	2991	5.66E-03	Up	2.77	4148	1.63	0.22
rs6702842	81609	SNX27	1	148421169	2.33E-02	2992	3.95E-02	Down	2.06	5736	1.63	0.14

rs3754171	947	CD34	1	204475334	2.33E-02	2993	1.75E-02	Down	2.38	4959	1.63	0.18
rs1860368	83707	TRPT1	11	63751268	2.33E-02	2994	1.59E-08	Up	5.64	1107	1.63	0.78
rs1860368	2286	FKBP2	11	63751268	2.33E-02	2995	4.33E-02	Up	2.02	5833	1.63	0.14
rs1860368	7423	VEGFB	11	63751268	2.33E-02	2996	4.50E-02	Down	2.00	5874	1.63	0.13
rs1860368	3338	DNAJC4	11	63751268	2.33E-02	2997	3.57E-01	Up	0.92	9658	1.63	0.04
rs1860368	84304	NUDT22	11	63751268	2.33E-02	2998	4.08E-01	Up	0.83	10044	1.63	0.04
rs7207286	124535	HSF5	17	53869457	2.34E-02	2999	4.40E-01	Down	0.77	10269	1.63	0.04
rs5923653	117154	DACH2		85892754	2.34E-02	3000	2.70E-01	Up	1.10	8952	1.63	0.06
rs10516490	55024	BANK1	4	103331239	2.34E-02	3001	5.17E-01	Up	0.65	10840	1.63	0.03
rs979976	80731	THSD7B	2	137602434	2.34E-02	3002	3.38E-02	Up	2.12	5584	1.63	0.15
rs3014866	6280	S100A9	1	150142144	2.34E-02	3003	3.03E-03	Down	2.97	3780	1.63	0.25
rs3014866	57115	PGLYRP4	1	150142144	2.34E-02	3004	2.47E-01	Down	1.16	8737	1.63	0.06
rs8047080	55282	LRRC36	16	65960089	2.34E-02	3005	7.17E-01	Up	0.36	12184	1.63	0.01
rs3849120	64420	SUSD1	9	111938331	2.34E-02	3006	1.71E-04	Up	3.76	2577	1.63	0.38
rs9833162	5096	PCCB	3	137518435	2.34E-02	3007	2.05E-08	Up	5.61	1123	1.63	0.77
rs13050927	3150	HMGN1	21	39644194	2.34E-02	3008	7.44E-01	Up	0.33	12339	1.63	0.01
rs1060463	8529	CYP4F2	19	15886176	2.35E-02	3009	1.15E-01	Down	1.58	7218	1.63	0.09
rs7250025	93145	OLFM2	19	9878101	2.35E-02	3010	1.47E-02	Up	2.44	4796	1.63	0.18
rs7881054	9500	MAGED1		51482860	2.35E-02	3011	7.30E-07	Up	4.95	1526	1.63	0.61
rs1379	79091	C16orf68	16	8650728	2.35E-02	3012	6.12E-04	Up	3.43	3036	1.63	0.32
rs16943244	51458	RHCG	15	87860042	2.35E-02	3013	4.29E-01	Up	0.79	10183	1.63	0.04
rs13298270	114991	ZNF618	9	113853019	2.35E-02	3014	4.37E-04	Up	3.52	2908	1.63	0.34
rs16947844	53944	CSNK1G1	15	62328834	2.36E-02	3015	1.70E-02	Up	2.39	4936	1.63	0.18
rs11098219	79642	ARSJ	4	115249361	2.36E-02	3016	2.25E-07	Down	5.18	1388	1.63	0.66
rs4803866	8189	SYMPK	19	51026795	2.36E-02	3017	2.09E-01	Up	1.26	8344	1.63	0.07
rs4803866	81492	RSHL1	19	51026795	2.36E-02	3018	7.74E-01	Up	0.29	12525	1.63	0.01
rs11629006	8110	DPF3	14	72441482	2.36E-02	3019	5.96E-01	Down	0.53	11409	1.63	0.02
rs239932	11116	FGFR1OP	6	167385092	2.36E-02	3020	2.11E-06	Up	4.74	1676	1.63	0.57
rs2946672	10845	CLPX	15	63242364	2.36E-02	3021	4.08E-04	Up	3.53	2891	1.63	0.34
rs4683189	1232	CCR3	3	46249769	2.36E-02	3022	6.07E-02	Up	1.88	6249	1.63	0.12
rs2844498	4277	MICB	6	31584833	2.36E-02	3023	5.66E-01	Down	0.57	11214	1.63	0.02
rs9952325	10928	RALBP1	18	9522097	2.36E-02	3024	7.87E-01	Down	0.27	12591	1.63	0.01
rs2566530	116372	LYPD1	2	133270904	2.36E-02	3025	9.50E-02	Up	1.67	6908	1.63	0.10
rs913199	3953	LEPR	1	65583083	2.37E-02	3026	2.38E-06	Down	4.72	1695	1.63	0.56
rs11247574	29	ABR	17	870666	2.37E-02	3027	1.42E-02	Down	2.45	4768	1.63	0.18
rs913199	54741	LEPROT	1	65583083	2.37E-02	3028	2.40E-02	Down	2.26	5246	1.63	0.16
rs913199	9829	DNAJC6	1	65583083	2.37E-02	3029	5.54E-01	Down	0.59	11113	1.63	0.03
rs2254524	4047	LSS	21	46438897	2.37E-02	3030	7.86E-08	Down	5.37	1271	1.63	0.71
rs2275290	55023	PHIP	6	79713289	2.37E-02	3031	4.75E-01	Down	0.71	10532	1.63	0.03
rs647155	26040	SETBP1	18	40703303	2.37E-02	3032	1.16E-03	Down	3.25	3278	1.63	0.29
rs6482601	84930	MASTL	10	27504606	2.37E-02	3033	3.96E-01	Up	0.85	9959	1.63	0.04
rs10994058	8030	CCDC6	10	61288795	2.37E-02	3034	1.27E-02	Up	2.49	4667	1.62	0.19
rs6453124	9607	CARTPT	5	71036822	2.37E-02	3035	8.18E-01	Down	0.23	12777	1.62	0.01
rs2229205	10287	RGS19	20	62199875	2.38E-02	3036	1.75E-01	Down	1.36	7962	1.62	0.08
rs2229205	4987	OPRL1	20	62199875	2.38E-02	3037	8.25E-01	Down	0.22	12818	1.62	0.01
rs13413029	23671	TMEFF2	2	192800710	2.38E-02	3038	7.60E-08	Up	5.38	1267	1.62	0.71
rs7647305	2119	ETV5	3	187316992	2.38E-02	3039	2.58E-18	Down	8.73	259	1.62	1.76
rs3828680	56120	PCDHGB8P	5	140795532	2.38E-02	3040	2.72E-01	Down	1.10	8964	1.62	0.06
rs6017787	51006	SLC35C2	20	44408103	2.38E-02	3041	3.96E-01	Down	0.85	9955	1.62	0.04
rs11861085	4363	ABCC1	16	16107157	2.38E-02	3042	1.42E-01	Up	1.47	7602	1.62	0.08
rs12531704	63917	GALNT11	7	151255883	2.38E-02	3043	2.01E-01	Up	1.28	8272	1.62	0.07
rs9610624	339669	C22orf33	22	35711757	2.38E-02	3044	5.60E-01	Up	0.58	11164	1.62	0.03
rs247772	7561	ZNF14	19	19707458	2.39E-02	3045	4.54E-01	Down	0.75	10377	1.62	0.03
rs531162	28513	CDH19	18	62334746	2.39E-02	3046	1.57E-05	Down	4.32	2045	1.62	0.48
rs2164360	7155	TOP2B	3	25607898	2.39E-02	3047	3.54E-01	Up	0.93	9631	1.62	0.05
rs6820989	80008	TMEM156	4	38789038	2.39E-02	3048	7.15E-03	Up	2.69	4272	1.62	0.21
rs12457026	56907	SPIRE1	18	12541818	2.39E-02	3049	4.47E-01	Down	0.76	10322	1.62	0.04
rs13173226	140947	C5orf20	5	134814158	2.40E-02	3050	6.03E-01	Down	0.52	11451	1.62	0.02
rs10793048	9873	FCHSD2	11	72258046	2.40E-02	3051	3.53E-08	Down	5.51	1188	1.62	0.75
rs741433	55507	GPRC5D	12	12978328	2.40E-02	3052	3.54E-01	Up	0.93	9622	1.62	0.05
rs263063	23030	JMJD2B	19	4973604	2.40E-02	3053	6.01E-09	Up	5.55	1163	1.62	0.82
rs1478938	158358	KIAA2026	9	5921216	2.41E-02	3054	1.07E-01	Down	1.61	7104	1.62	0.10
rs2273770	26578	OSTF1	9	74985023	2.41E-02	3055	2.92E-01	Down	1.05	9127	1.62	0.05
rs11002928	10105	PPIF	10	80759168	2.41E-02	3056	1.66E-04	Up	3.77	2573	1.62	0.38
rs2641787	54925	ZNF434	16	3407330	2.41E-02	3057	5.04E-03	Up	2.80	4073	1.62	0.23
rs2641787	7727	ZNF174	16	3407330	2.41E-02	3058	1.16E-01	Down	1.57	7229	1.62	0.09
rs1808991	2996	GYPE	4	145150564	2.41E-02	3059	2.04E-01	Up	1.27	8297	1.62	0.07
rs11089853	23616	SH3BP1	22	36434785	2.41E-02	3060	1.89E-01	Up	1.31	8140	1.62	0.07
rs218469	8675	STX16	20	56655940	2.41E-02	3061	2.73E-03	Down	3.00	3735	1.62	0.26
rs6020157	6615	SNAI1	20	48025165	2.41E-02	3062	8.02E-01	Up	0.25	12689	1.62	0.01
rs17705719	7038	TG	8	134209728	2.42E-02	3063	8.26E-01	Up	0.22	12831	1.62	0.01
rs4807532	85300	ATCAY	19	3879369	2.42E-02	3064	3.61E-03	Down	2.91	3873	1.62	0.24
rs4807532	27231	ITGB1BP3	19	3879369	2.42E-02	3065	6.88E-02	Down	1.82	6405	1.62	0.12
rs13098257	55573	CDV3	3	134798701	2.42E-02	3066	5.41E-01	Down	0.61	11023	1.62	0.03
rs276504	53832	IL20RA	6	137394212	2.42E-02	3067	1.78E-02	Up	2.37	4978	1.62	0.17
rs2235108	10221	TRIB1	8	126517971	2.42E-02	3068	5.15E-13	Up	7.23	530	1.62	1.23
rs1467658	84898	PLXDC2	10	20219229	2.43E-02	3069	6.23E-01	Down	0.49	11600	1.61	0.02
rs465566	2897	GRIK1	21	30216183	2.43E-02	3070	8.75E-06	Up	4.45	1922	1.61	0.51
rs11244202	84929	FIBCD1	9	130839329	2.43E-02	3071	2.88E-01	Down	1.06	9098	1.61	0.05
rs4264688	6091	ROBO1	3	79629038	2.43E-02	3072	8.44E-06	Down	4.45	1917	1.61	0.51
rs10490012	79054	TRPM8	2	234625494	2.43E-02	3073	3.22E-01	Up	0.99	9376	1.61	0.05

rs3790085	11060	WWP2	16	68445221	2.43E-02	3074	6.41E-10	Up	6.18	867	1.61	0.92
rs10143181	91748	C14orf43	14	73273421	2.43E-02	3075	7.16E-01	Down	0.36	12170	1.61	0.01
rs1951580	122402	TDRD9	14	103571678	2.43E-02	3076	7.01E-01	Up	0.38	12079	1.61	0.02
rs11622651	26153	KIF26A	14	103689830	2.44E-02	3077	8.75E-01	Down	0.16	13093	1.61	0.01
rs2910188	9232	PTTG1	5	159771241	2.44E-02	3078	4.72E-05	Up	4.07	2267	1.61	0.43
rs2910188	10569	SLU7	5	159771241	2.44E-02	3079	7.84E-01	Up	0.27	12569	1.61	0.01
rs17168992	4223	MEOX2	7	15508799	2.44E-02	3080	3.49E-01	Up	0.94	9580	1.61	0.05
rs9792648	5535	PPP3R2	9	101456156	2.44E-02	3081	3.22E-02	Up	2.14	5532	1.61	0.15
rs292007	713	C1QB	1	22736818	2.44E-02	3082	5.42E-01	Up	0.61	11032	1.61	0.03
rs11153258	262	AMD1	6	111329245	2.44E-02	3083	9.92E-01	Down	0.01	13852	1.61	0.00
rs1510611	6711	SPTBN1	2	54777178	2.44E-02	3084	1.57E-06	Down	4.80	1636	1.61	0.58
rs3803433	220	ALDH1A3	15	99239196	2.44E-02	3085	3.83E-10	Up	6.25	832	1.61	0.94
rs886853	11316	COPE	19	18870470	2.44E-02	3086	6.28E-05	Up	4.00	2339	1.61	0.42
rs2288100	79623	GALNT14	2	31090469	2.45E-02	3087	2.22E-02	Down	2.29	5167	1.61	0.17
rs9333347	23170	TTLL12	22	41884210	2.45E-02	3088	2.71E-12	Up	7.00	595	1.61	1.16
rs1320546	56033	BARX1	9	93799454	2.45E-02	3089	9.80E-01	Up	0.02	13775	1.61	0.00
rs7141353	27133	KCNH5	14	62248388	2.45E-02	3090	1.99E-01	Down	1.28	8244	1.61	0.07
rs7159692	8111	GPR68	14	90799159	2.45E-02	3091	2.12E-02	Down	2.30	5129	1.61	0.17
rs453932	7711	ZNF155	19	49161548	2.45E-02	3092	4.03E-01	Down	0.84	10006	1.61	0.04
rs11161912	127247	ASB17	1	76117183	2.45E-02	3093	4.43E-01	Up	0.77	10288	1.61	0.04
rs2293554	841	CASP8	2	201957093	2.46E-02	3094	4.62E-01	Up	0.73	10445	1.61	0.03
rs16958048	9423	NTN1	17	9051741	2.46E-02	3095	7.69E-01	Down	0.29	12482	1.61	0.01
rs1109859	10400	PEMT	17	17365058	2.46E-02	3096	9.42E-04	Up	3.31	3193	1.61	0.30
rs167771	1814	DRD3	3	115358965	2.46E-02	3097	8.35E-01	Up	0.21	12879	1.61	0.01
rs10513191	56172	ANKH	5	14922666	2.46E-02	3098	1.69E-16	Down	8.24	333	1.61	1.58
rs7035562	286204	CRB2	9	123185696	2.47E-02	3099	3.95E-01	Up	0.85	9943	1.61	0.04
rs6685014	6726	SRP9	1	222265592	2.47E-02	3100	1.11E-01	Up	1.60	7156	1.61	0.10
rs10739976	8789	FBP2	9	94425992	2.47E-02	3101	5.67E-01	Down	0.57	11218	1.61	0.02
rs10739976	8570	KHSRP	9	94425992	2.47E-02	3102	6.27E-01	Up	0.49	11627	1.61	0.02
rs17625547	1060	CENPC1	4	68238775	2.47E-02	3103	2.62E-03	Down	3.01	3712	1.61	0.26
rs11996170	5157	PDGFRL	8	17483455	2.47E-02	3104	2.58E-01	Down	1.13	8844	1.61	0.06
rs6727917	10213	PSMD14	2	162060115	2.47E-02	3105	8.24E-02	Up	1.74	6674	1.61	0.11
rs6019328	57580	PREX1	20	46686159	2.47E-02	3106	2.07E-01	Up	1.26	8320	1.61	0.07
rs10904471	114131	UCN3	10	5381526	2.47E-02	3107	4.92E-01	Up	0.69	10666	1.61	0.03
rs10115619	80036	TRPM3	9	70536091	2.48E-02	3108	8.94E-01	Up	0.13	13208	1.61	0.00
rs10276324	273	AMPH	7	38324037	2.48E-02	3109	6.08E-03	Down	2.74	4176	1.61	0.22
rs11760739	136647	C7orf11	7	39966775	2.48E-02	3110	4.79E-05	Up	4.07	2272	1.60	0.43
rs6661612	280	AMY2B	1	103840639	2.48E-02	3111	9.83E-04	Down	3.30	3216	1.60	0.30
rs264336	9542	NRG2	5	139308922	2.48E-02	3112	6.15E-02	Down	1.87	6265	1.60	0.12
rs226380	2	A2M	12	9159740	2.49E-02	3113	3.31E-01	Down	0.97	9454	1.60	0.05
rs2748901	9962	SLC23A2	20	4896248	2.49E-02	3114	9.49E-01	Up	0.06	13541	1.60	0.00
rs11145835	6621	SNAPC4	9	136560289	2.49E-02	3115	2.49E-03	Up	3.02	3683	1.60	0.26
rs1902921	5932	RBBP8	18	18798341	2.49E-02	3116	8.75E-07	Down	4.92	1560	1.60	0.61
rs855843	4919	ROR1	1	64066846	2.49E-02	3117	1.10E-02	Down	2.54	4576	1.60	0.20
rs314111	3779	KCNMB1	5	169746422	2.49E-02	3118	2.05E-23	Down	9.97	132	1.60	2.27
rs12082399	54953	C1orf27	1	183106069	2.49E-02	3119	1.44E-02	Up	2.45	4781	1.60	0.18
rs12082399	10896	OCLM	1	183106069	2.49E-02	3120	2.22E-01	Up	1.22	8480	1.60	0.07
rs7330299	10808	HSPH1	13	30637448	2.50E-02	3121	1.09E-01	Down	1.60	7138	1.60	0.10
rs4732901	23303	KIF13B	8	28987298	2.50E-02	3122	2.59E-03	Up	3.01	3706	1.60	0.26
rs6080765	6238	RRBP1	20	17589928	2.50E-02	3123	1.30E-06	Up	4.84	1609	1.60	0.59
rs12770361	2869	GRK5	10	121105608	2.50E-02	3124	1.00E-10	Down	6.47	755	1.60	1.00
rs2471017	94081	SFXN1	5	174890088	2.50E-02	3125	3.61E-01	Down	0.91	9683	1.60	0.04
rs5435	6517	SLC2A4	17	7127847	2.50E-02	3126	4.82E-06	Down	4.57	1806	1.60	0.53
rs5435	51087	YBX2	17	7127847	2.50E-02	3127	8.38E-01	Down	0.20	12895	1.60	0.01
rs1990059	58517	RBM25	14	72639228	2.50E-02	3128	5.51E-03	Up	2.78	4131	1.60	0.23
rs11109966	196472	FAM71C	12	98542565	2.50E-02	3129	1.85E-01	Down	1.33	8083	1.60	0.07
rs11882442	126353	C19orf21	19	703878	2.51E-02	3130	1.22E-03	Down	3.23	3298	1.60	0.29
rs11882442	5064	PALM	19	703878	2.51E-02	3131	8.78E-01	Up	0.15	13111	1.60	0.01
rs4883894	1602	DACH1	13	71101093	2.51E-02	3132	2.84E-06	Down	4.68	1725	1.60	0.55
rs174478	3995	FADS3	11	61435152	2.51E-02	3133	4.69E-01	Up	0.72	10494	1.60	0.03
rs174478	5866	RAB3IL1	11	61435152	2.51E-02	3134	5.65E-01	Down	0.58	11204	1.60	0.02
rs2031011	4669	NAGLU	2	15498988	2.51E-02	3135	4.25E-02	Up	2.03	5813	1.60	0.14
rs12586226	7187	TRAF3	14	102320756	2.51E-02	3136	6.41E-01	Up	0.47	11715	1.60	0.02
rs2655986	283576	ZDHHC22	14	76695528	2.51E-02	3137	8.01E-01	Down	0.25	12683	1.60	0.01
rs12662559	8936	WASF1	6	110615389	2.51E-02	3138	7.64E-07	Up	4.94	1533	1.60	0.61
rs12662559	51362	CDC40	6	110615389	2.51E-02	3139	7.29E-01	Down	0.35	12242	1.60	0.01
rs1262460	10630	PDPN	1	13670169	2.51E-02	3140	4.03E-14	Down	7.56	446	1.60	1.34
rs17256042	28959	TMEM176B	7	149927886	2.51E-02	3141	2.03E-02	Down	2.32	5088	1.60	0.17
rs17256042	55365	TMEM176A	7	149927886	2.51E-02	3142	5.08E-01	Down	0.66	10767	1.60	0.03
rs1200814	91074	ANKRD30A	10	37575416	2.52E-02	3143	3.95E-02	Up	2.06	5737	1.60	0.14
rs11160108	84520	C14orf142	14	92762928	2.52E-02	3144	2.38E-05	Up	4.23	2120	1.60	0.46
rs10105517	11236	RNF139	8	125546978	2.52E-02	3145	5.59E-05	Up	4.03	2317	1.60	0.43
rs2925768	57453	DSCAML1	11	116804225	2.52E-02	3146	6.02E-01	Up	0.52	11444	1.60	0.02
rs10735937	85329	LGALS12	12	65340149	2.52E-02	3147	1.78E-01	Down	1.35	7988	1.60	0.08
rs10735937	23426	GRIP1	12	65340149	2.52E-02	3148	5.68E-01	Down	0.57	11223	1.60	0.02
rs12321487	5927	JARID1A	12	258468	2.52E-02	3149	5.69E-02	Up	1.90	6155	1.60	0.12
rs4803403	1550	CYP2A7P1	19	46104782	2.52E-02	3150	9.95E-01	Down	0.01	13875	1.60	0.00
rs2292153	23398	PPWD1	5	64925773	2.52E-02	3151	5.39E-02	Down	1.93	6085	1.60	0.13
rs2380580	81796	SLCO5A1	8	70790837	2.52E-02	3152	6.50E-01	Up	0.45	11776	1.60	0.02
rs2071572	6861	SYT5	19	60378042	2.52E-02	3153	5.78E-02	Up	1.90	6185	1.60	0.12
rs2071572	7137	TNNI3	19	60378042	2.52E-02	3154	4.88E-01	Up	0.69	10637	1.60	0.03

rs2414407	5873	RAB27A	15	53343520	2.53E-02	3155	2.11E-01	Down	1.25	8370	1.60	0.07
rs12609287	1153	CIRBP	19	1222274	2.53E-02	3156	4.13E-01	Down	0.82	10071	1.60	0.04
rs12609287	55009	C19orf24	19	1222274	2.53E-02	3157	9.30E-01	Down	0.09	13433	1.60	0.00
rs17358030	26298	EHF	11	34603458	2.53E-02	3158	7.91E-01	Down	0.27	12615	1.60	0.01
rs17165909	2791	GNG11	7	93196257	2.53E-02	3159	8.79E-19	Down	8.85	238	1.60	1.81
rs2245803	9313	MMP20	11	102001208	2.53E-02	3160	8.41E-01	Up	0.20	12908	1.60	0.01
rs11230906	3619	INCENP	11	61636265	2.53E-02	3161	4.79E-01	Down	0.71	10571	1.60	0.03
rs4484620	375612	LHFPL3	7	103552833	2.53E-02	3162	4.96E-01	Up	0.68	10685	1.60	0.03
rs2275496	8518	IKBKAP	9	108721182	2.54E-02	3163	1.16E-05	Up	4.39	1978	1.60	0.49
rs10813923	3301	DNAJA1	9	33013517	2.54E-02	3164	1.08E-01	Down	1.61	7129	1.60	0.10
rs2072581	859	CAV3	3	8750153	2.54E-02	3165	1.47E-03	Down	3.18	3373	1.60	0.28
rs4448244	92140	MTDH	8	98733704	2.54E-02	3166	2.47E-02	Up	2.25	5274	1.59	0.16
rs11119981	83953	FCAMR	1	203544950	2.54E-02	3167	2.81E-01	Down	1.08	9041	1.59	0.06
rs7574097	54221	SNTG2	2	1004757	2.54E-02	3168	5.02E-01	Up	0.67	10733	1.59	0.03
rs12677785	57210	SLC45A4	8	142322438	2.54E-02	3169	6.89E-01	Up	0.40	12002	1.59	0.02
rs749906	84951	TNS4	17	35888455	2.55E-02	3170	1.18E-01	Down	1.56	7257	1.59	0.09
rs6488519	118426	LOH12CR1	12	12424251	2.55E-02	3171	3.41E-01	Down	0.95	9534	1.59	0.05
rs1149175	6272	SORT1	1	109634419	2.55E-02	3172	9.91E-03	Down	2.58	4502	1.59	0.20
rs4803254	10683	DLL3	19	44696480	2.55E-02	3173	2.68E-01	Down	1.11	8931	1.59	0.06
rs7556606	23673	STX12	1	27791201	2.55E-02	3174	2.98E-07	Down	5.12	1417	1.59	0.65
rs9605957	8218	CLTCL1	22	17561929	2.55E-02	3175	2.00E-06	Down	4.75	1670	1.59	0.57
rs2104880	3445	IFNA8	9	21409723	2.55E-02	3176	4.36E-01	Down	0.78	10237	1.59	0.04
rs7626725	9819	TSC22D2	3	151599933	2.55E-02	3177	7.53E-01	Down	0.32	12395	1.59	0.01
rs2532501	6843	VAMP1	12	6433014	2.55E-02	3178	5.62E-03	Down	2.77	4144	1.59	0.23
rs2532501	55080	TAPBPL	12	6433014	2.55E-02	3179	1.26E-01	Down	1.53	7385	1.59	0.09
rs7755748	2651	GCNT2	6	10708866	2.55E-02	3180	1.57E-03	Down	3.16	3410	1.59	0.28
rs11751668	10455	PECI	6	4070473	2.56E-02	3181	4.78E-14	Up	7.53	453	1.59	1.33
rs11751668	404220	C6orf201	6	4070473	2.56E-02	3182	9.26E-01	Down	0.09	13405	1.59	0.00
rs10971333	54840	APTX	9	33008972	2.56E-02	3183	5.26E-01	Down	0.63	10909	1.59	0.03
rs12904003	4240	MFGE8	15	87274930	2.56E-02	3184	9.22E-05	Down	3.91	2431	1.59	0.40
rs12140633	54810	GIPC2	1	78225645	2.56E-02	3185	1.94E-01	Down	1.30	8197	1.59	0.07
rs17534319	1946	EFNA5	5	106882151	2.56E-02	3186	2.93E-01	Down	1.05	9141	1.59	0.05
rs3789940	1791	DNTT	10	98075328	2.56E-02	3187	2.55E-01	Up	1.14	8810	1.59	0.06
rs3805703	9945	GFPT2	5	179674140	2.57E-02	3188	3.20E-03	Down	2.95	3819	1.59	0.25
rs10483882	10598	AHSA1	14	76984427	2.57E-02	3189	1.14E-02	Up	2.53	4604	1.59	0.19
rs10483882	63894	C14orf133	14	76984427	2.57E-02	3190	8.22E-02	Down	1.74	6672	1.59	0.11
rs793283	79733	E2F8	11	19221073	2.57E-02	3191	8.94E-02	Up	1.70	6791	1.59	0.10
rs6687966	1063	CENPF	1	211236199	2.57E-02	3192	2.51E-09	Up	5.96	950	1.59	0.86
rs847682	84687	PPP1R9B	17	45563273	2.57E-02	3193	9.33E-01	Up	0.08	13454	1.59	0.00
rs10931468	4664	NAB1	2	191364068	2.57E-02	3194	2.68E-03	Down	3.00	3725	1.59	0.26
rs1474758	5105	PCK1	20	55559297	2.57E-02	3195	9.66E-02	Up	1.66	6936	1.59	0.10
rs12149310	6530	SLC6A2	16	54295841	2.58E-02	3196	3.84E-01	Up	0.87	9871	1.59	0.04
rs7729211	133418	EMB	5	49743944	2.58E-02	3197	2.99E-10	Up	6.29	815	1.59	0.95
rs17581597	51027	BOLA1	1	146673445	2.58E-02	3198	1.40E-01	Up	1.48	7570	1.59	0.09
rs17581597	8349	HIST2H2BE	1	146673445	2.58E-02	3199	1.48E-01	Up	1.45	7653	1.59	0.08
rs4452723	259285	TAS2R39	7	142389218	2.58E-02	3200	9.31E-01	Up	0.09	13440	1.59	0.00
rs7919883	79861	TUBAL3	10	5415422	2.59E-02	3201	6.05E-01	Up	0.52	11466	1.59	0.02
rs9823555	152405	C3orf30	3	120360557	2.59E-02	3202	8.01E-01	Up	0.25	12680	1.59	0.01
rs17157040	10647	SCGB1D2	11	61771851	2.59E-02	3203	5.76E-01	Down	0.56	11288	1.59	0.02
rs2275009	5728	PTEN	14	19942580	2.59E-02	3204	3.69E-10	Down	6.27	822	1.59	0.94
rs2275009	7011	TEP1	14	19942580	2.59E-02	3205	3.38E-02	Up	2.12	5586	1.59	0.15
rs13175903	730	C7	5	40943697	2.59E-02	3206	4.06E-12	Down	6.93	618	1.59	1.14
rs13175903	3627	CXCL10	5	40943697	2.59E-02	3207	2.16E-01	Up	1.24	8422	1.59	0.07
rs982447	10466	COG5	7	106567485	2.60E-02	3208	1.10E-08	Up	5.70	1069	1.59	0.80
rs1285953	26495	OR9A1P	7	141046958	2.60E-02	3209	2.08E-02	Down	2.31	5107	1.58	0.17
rs2842950	7172	TPMT	6	18243157	2.60E-02	3210	7.41E-01	Down	0.33	12320	1.58	0.01
rs2832194	56911	C21orf7	21	29416121	2.60E-02	3211	9.92E-02	Up	1.65	6985	1.58	0.10
rs13238458	55972	SLC25A40	7	87155070	2.60E-02	3212	5.58E-06	Up	4.54	1837	1.58	0.53
rs13238458	10926	DBF4	7	87155070	2.60E-02	3213	5.06E-03	Up	2.80	4077	1.58	0.23
rs5935460	7114	TMSB4X		12757911	2.61E-02	3214	1.35E-01	Down	1.49	7518	1.58	0.09
rs487377	64096	GFRA4	20	3606931	2.61E-02	3215	8.54E-03	Down	2.63	4385	1.58	0.21
rs487377	80332	ADAM33	20	3606931	2.61E-02	3216	5.58E-02	Down	1.91	6132	1.58	0.13
rs2052398	57709	SLC7A14	3	171776276	2.61E-02	3217	4.49E-01	Up	0.76	10342	1.58	0.03
rs12749153	22911	WDR47	1	109216705	2.61E-02	3218	2.33E-01	Down	1.19	8596	1.58	0.06
rs12749153	23155	CLCC1	1	109216705	2.61E-02	3219	7.07E-01	Down	0.38	12113	1.58	0.02
rs10518440	10026	PIGK	1	77408843	2.61E-02	3220	1.20E-05	Up	4.38	1990	1.58	0.49
rs3777667	51696	HECA	6	139542435	2.61E-02	3221	6.41E-04	Down	3.41	3052	1.58	0.32
rs2167285	9746	CLSTN3	12	7175568	2.61E-02	3222	9.40E-02	Down	1.67	6899	1.58	0.10
rs2167285	83758	RBP5	12	7175568	2.61E-02	3223	6.99E-01	Up	0.39	12066	1.58	0.02
rs4920550	80834	TAS1R2	1	18905542	2.62E-02	3224	3.66E-01	Up	0.90	9727	1.58	0.04
rs9900615	64446	DNAI2	17	69784702	2.62E-02	3225	8.28E-01	Down	0.22	12840	1.58	0.01
rs950549	26281	FGF20	8	16885663	2.62E-02	3226	8.26E-01	Up	0.22	12826	1.58	0.01
rs1381914	160298	C11orf42	11	6176091	2.62E-02	3227	9.91E-01	Up	0.01	13849	1.58	0.00
rs7624358	26060	APPL1	3	57286580	2.62E-02	3228	2.81E-04	Up	3.63	2757	1.58	0.36
rs7624358	142686	ASB14	3	57286580	2.62E-02	3229	2.08E-01	Down	1.26	8327	1.58	0.07
rs12534005	10643	IGF2BP3	7	23275758	2.63E-02	3230	8.97E-01	Down	0.13	13224	1.58	0.00
rs3027575	2717	GLA		100472981	2.63E-02	3231	1.66E-01	Up	1.39	7878	1.58	0.08
rs3027575	9027	NAT8		100472981	2.63E-02	3232	2.42E-01	Up	1.17	8676	1.58	0.06
rs731945	51477	ISYNA1	19	18428039	2.63E-02	3233	4.18E-05	Down	4.10	2238	1.58	0.44
rs7040903	25769	SLC24A2	9	19656510	2.63E-02	3234	8.67E-01	Down	0.17	13045	1.58	0.01
rs546003	8796	SCEL	13	77003705	2.63E-02	3235	6.78E-01	Up	0.42	11934	1.58	0.02

rs4959388	9450	LY86	6	6527122	2.63E-02	3236	6.06E-01	Up	0.52	11476	1.58	0.02
rs592637	54860	MS4A12	11	60324731	2.63E-02	3237	6.45E-01	Down	0.46	11750	1.58	0.02
rs8129810	9073	CLDN8	21	30521327	2.63E-02	3238	2.15E-17	Up	8.49	290	1.58	1.67
rs10490831	868	CBLB	3	106856526	2.63E-02	3239	4.93E-06	Up	4.57	1813	1.58	0.53
rs11772925	170575	GIMAP1	7	149865757	2.63E-02	3240	3.81E-01	Up	0.88	9844	1.58	0.04
rs10490831	326625	MMAB	3	106856526	2.63E-02	3241	6.63E-01	Down	0.44	11841	1.58	0.02
rs2338544	347689	SOX2OT	3	182773194	2.63E-02	3242	5.32E-01	Up	0.62	10953	1.58	0.03
rs8109640	29785	CYP2S1	19	46387644	2.63E-02	3243	6.32E-01	Down	0.48	11654	1.58	0.02
rs8192207	6715	SRD5A1	5	6709161	2.64E-02	3244	8.80E-08	Up	5.35	1281	1.58	0.71
rs4832254	10989	IMMT	2	86340722	2.64E-02	3245	1.11E-01	Down	1.59	7165	1.58	0.10
rs4832254	51318	MRPL35	2	86340722	2.64E-02	3246	2.03E-01	Down	1.27	8286	1.58	0.07
rs7298053	51258	MRPL51	12	6489315	2.64E-02	3247	2.12E-01	Up	1.25	8384	1.58	0.07
rs1720819	22808	MRAS	3	139583237	2.64E-02	3248	1.55E-03	Down	3.16	3402	1.58	0.28
rs10500136	835	CASP2	7	142531081	2.64E-02	3249	6.26E-03	Up	2.73	4188	1.58	0.22
rs10500136	1180	CLCN1	7	142531081	2.64E-02	3250	1.92E-01	Up	1.30	8169	1.58	0.07
rs2834079	116448	OLIG1	21	33352040	2.64E-02	3251	6.74E-02	Up	1.83	6385	1.58	0.12
rs2838562	7226	TRPM2	21	44692667	2.64E-02	3252	5.24E-01	Up	0.64	10899	1.58	0.03
rs2838562	81543	LRRC3	21	44692667	2.64E-02	3253	6.19E-01	Down	0.50	11567	1.58	0.02
rs12172202	283987	C17orf28	22	37256037	2.64E-02	3254	1.48E-13	Up	7.39	484	1.58	1.28
rs12172202	11144	DMC1	22	37256037	2.64E-02	3255	2.73E-01	Up	1.10	8978	1.58	0.06
rs12218194	53354	PANK1	10	91409976	2.64E-02	3256	1.07E-01	Down	1.61	7110	1.58	0.10
rs6602207	8027	STAM	10	17738884	2.64E-02	3257	8.03E-01	Down	0.25	12694	1.58	0.01
rs4863687	55534	MAML3	4	141035886	2.65E-02	3258	9.77E-03	Up	2.58	4492	1.58	0.20
rs7544816	1301	COL11A1	1	103268080	2.65E-02	3259	4.99E-01	Up	0.68	10707	1.58	0.03
rs133295	164684	WBP2NL	22	40708625	2.65E-02	3260	8.65E-01	Up	0.17	13033	1.58	0.01
rs11188233	9124	PDLIM1	10	96978698	2.65E-02	3261	1.02E-13	Down	7.44	472	1.58	1.30
rs9349688	55227	LRRC1	6	53870051	2.65E-02	3262	3.94E-03	Up	2.88	3918	1.58	0.24
rs11778235	157739	TDH	8	11252946	2.66E-02	3263	1.09E-01	Down	1.60	7139	1.58	0.10
rs1995483	56944	OLFML3	1	114245540	2.66E-02	3264	9.62E-03	Down	2.59	4480	1.58	0.20
rs794078	6813	STXBP2	19	7591843	2.66E-02	3265	1.69E-09	Up	6.02	930	1.58	0.88
rs450514	79587	CARS2	13	110099997	2.66E-02	3266	1.62E-03	Up	3.15	3424	1.57	0.28
rs11263535	9965	FGF19	11	69242310	2.67E-02	3267	8.34E-01	Up	0.21	12873	1.57	0.01
rs10221891	6744	SSFA2	2	182577700	2.67E-02	3268	7.52E-01	Down	0.32	12388	1.57	0.01
rs6702823	163882	C1orf71	1	243081165	2.67E-02	3269	3.23E-03	Up	2.95	3820	1.57	0.25
rs6828598	57728	WDR19	4	39084036	2.67E-02	3270	1.24E-01	Down	1.54	7350	1.57	0.09
rs9547011	84935	C13orf33	13	30377640	2.67E-02	3271	9.07E-03	Down	2.61	4441	1.57	0.20
rs17034027	374470	C12orf42	12	102399062	2.67E-02	3272	6.99E-01	Down	0.39	12067	1.57	0.02
rs2061066	51067	YARS2	12	32807475	2.67E-02	3273	4.57E-01	Down	0.74	10397	1.57	0.03
rs4731678	93979	CPA5	7	129570772	2.67E-02	3274	9.64E-01	Down	0.05	13642	1.57	0.00
rs4900026	56659	KCNK13	14	89698387	2.67E-02	3275	2.40E-01	Down	1.17	8657	1.57	0.06
rs4315495	23175	LPIN1	2	11842788	2.67E-02	3276	9.23E-21	Down	9.34	179	1.57	2.00
rs2071598	10940	POP1	8	99198683	2.67E-02	3277	1.93E-03	Up	3.10	3514	1.57	0.27
rs2071598	10247	HRSP12	8	99198683	2.67E-02	3278	3.20E-02	Up	2.14	5526	1.57	0.15
rs2071598	260434	PYDC1	8	99198683	2.67E-02	3279	1.67E-01	Down	1.38	7887	1.57	0.08
rs2328293	55184	C20orf12	20	18310297	2.68E-02	3280	9.76E-01	Up	0.03	13733	1.57	0.00
rs916930	83992	CTTNBP2	7	117011016	2.68E-02	3281	5.92E-01	Down	0.54	11386	1.57	0.02
rs10900866	285704	RGMB	5	98115219	2.68E-02	3282	2.04E-01	Down	1.27	8296	1.57	0.07
rs9327919	1105	CHD1	5	98298625	2.68E-02	3283	1.50E-01	Up	1.44	7678	1.57	0.08
rs6017335	3172	HNF4A	20	42444239	2.68E-02	3284	4.49E-01	Down	0.76	10340	1.57	0.03
rs6602	5711	PSMD5	9	120658518	2.68E-02	3285	3.91E-01	Up	0.86	9921	1.57	0.04
rs11815437	84458	LCOR	10	98657519	2.69E-02	3286	1.95E-02	Up	2.34	5049	1.57	0.17
rs10997948	84665	MYPN	10	69579808	2.69E-02	3287	7.20E-03	Down	2.69	4275	1.57	0.21
rs10794024	7390	UROS	10	127486598	2.69E-02	3288	4.64E-01	Up	0.73	10460	1.57	0.03
rs2074973	5864	RAB3A	19	18194273	2.69E-02	3289	1.77E-02	Up	2.37	4967	1.57	0.18
rs2074973	5143	PDE4C	19	18194273	2.69E-02	3290	5.27E-01	Down	0.63	10919	1.57	0.03
rs13277177	9	NAT1	8	18130376	2.69E-02	3291	3.92E-09	Down	5.89	991	1.57	0.84
rs13277177	1982	EIF4G2	8	18130376	2.69E-02	3292	8.58E-01	Up	0.18	13004	1.57	0.01
rs29942	79047	KCTD15	19	39001117	2.69E-02	3293	1.40E-03	Up	3.19	3350	1.57	0.29
rs3828054	57530	CGN	1	148325968	2.69E-02	3294	8.85E-10	Up	6.13	885	1.57	0.91
rs3828054	7286	TUFT1	1	148325968	2.69E-02	3295	1.27E-04	Up	3.83	2506	1.57	0.39
rs10819788	8577	TMEFF1	9	100341783	2.69E-02	3296	6.72E-01	Up	0.42	11899	1.57	0.02
rs10438428	9640	ZNF592	15	83131749	2.69E-02	3297	1.46E-01	Up	1.45	7640	1.57	0.08
rs8011890	9623	TCL1B	14	95216646	2.69E-02	3298	1.74E-01	Down	1.36	7952	1.57	0.08
rs8011890	27004	TCL6	14	95216646	2.69E-02	3299	3.82E-01	Up	0.87	9848	1.57	0.04
rs3217906	894	CCND2	12	4276065	2.69E-02	3300	3.25E-13	Down	7.28	514	1.57	1.25
rs12115750	26190	FBXW2	9	120641410	2.69E-02	3301	9.66E-02	Up	1.66	6935	1.57	0.10
rs6981918	1585	CYP11B2	8	144004941	2.69E-02	3302	3.55E-01	Up	0.92	9639	1.57	0.04
rs17796841	115416	C7orf30	7	23126754	2.70E-02	3303	6.90E-01	Down	0.40	12004	1.57	0.02
rs10512263	7046	TGFBR1	9	98965626	2.70E-02	3304	4.93E-02	Down	1.97	5979	1.57	0.13
rs10147939	9556	C14orf2	14	103447985	2.70E-02	3305	-1.90E-10	Up	6.43	772	1.57	#NUM!
rs10162645	4734	NEDD4	15	54089855	2.70E-02	3306	1.72E-02	Down	2.38	4953	1.57	0.18
rs2624842	6405	SEMA3F	3	50171422	2.70E-02	3307	5.00E-01	Up	0.67	10720	1.57	0.03
rs138354	6767	ST13	22	39596643	2.70E-02	3308	1.57E-03	Down	3.16	3408	1.57	0.28
rs138354	150353	DNAJB7	22	39596643	2.70E-02	3309	7.46E-01	Down	0.32	12350	1.57	0.01
rs2635140	85453	TSPYL5	8	98371361	2.70E-02	3310	5.63E-09	Down	5.83	1021	1.57	0.82
rs7387252	427	ASAH1	8	17987316	2.71E-02	3311	4.02E-01	Up	0.84	9989	1.57	0.04
rs13043176	51526	C20orf111	20	42281635	2.71E-02	3312	1.93E-01	Down	1.30	8182	1.57	0.07
rs10144261	1983	EIF5	14	102851569	2.71E-02	3313	2.43E-08	Down	5.58	1144	1.57	0.76
rs3744093	54894	RNF43	17	53847799	2.71E-02	3314	4.23E-02	Up	2.03	5805	1.57	0.14
rs346155	28974	C19orf53	19	13746127	2.71E-02	3315	6.20E-01	Up	0.50	11578	1.57	0.02
rs12148410	3669	ISG20	15	86972726	2.71E-02	3316	9.60E-01	Up	0.05	13624	1.57	0.00

rs11640759	8139	GAN	16	79973370	2.71E-02	3317	3.45E-01	Down	0.94	9561	1.57	0.05
rs1929494	84302	C9orf125	9	101316329	2.71E-02	3318	1.46E-13	Down	7.39	483	1.57	1.28
rs4785648	29123	ANKRD11	16	87855978	2.72E-02	3319	5.99E-01	Up	0.53	11425	1.57	0.02
rs11067101	6910	TBX5	12	113306923	2.72E-02	3320	5.49E-01	Down	0.60	11075	1.57	0.03
rs13409229	200734	SPRED2	2	65460158	2.72E-02	3321	8.94E-08	Down	5.35	1284	1.57	0.70
rs10110267	51633	OTUD6B	8	92142990	2.72E-02	3322	1.03E-02	Up	2.57	4525	1.57	0.20
rs4850381	56171	DNAH7	2	196737573	2.72E-02	3323	6.32E-02	Up	1.86	6303	1.57	0.12
rs6768170	84859	LRCH3	3	199049321	2.72E-02	3324	4.71E-01	Down	0.72	10507	1.57	0.03
rs4900345	7443	VRK1	14	96318766	2.72E-02	3325	2.91E-03	Up	2.98	3761	1.56	0.25
rs7566763	83439	TCF7L1	2	85383117	2.72E-02	3326	8.36E-15	Down	7.76	402	1.56	1.41
rs12895695	3705	ITPK1	14	92664804	2.73E-02	3327	4.71E-04	Down	3.50	2941	1.56	0.33
rs16985044	55422	ZNF331	19	58767245	2.73E-02	3328	1.37E-02	Down	2.46	4737	1.56	0.19
rs1278286	84722	PSRC1	1	109557142	2.73E-02	3329	9.32E-01	Up	0.08	13450	1.56	0.00
rs3561	5106	PCK2	14	23620064	2.73E-02	3330	2.81E-03	Up	2.99	3751	1.56	0.26
rs3561	9362	CPNE6	14	23620064	2.73E-02	3331	5.92E-01	Down	0.54	11385	1.56	0.02
rs3561	4901	NRL	14	23620064	2.73E-02	3332	7.44E-01	Down	0.33	12336	1.56	0.01
rs406601	83987	CCDC8	19	51622541	2.73E-02	3333	5.19E-05	Down	4.05	2298	1.56	0.43
rs237417	149840	C20orf196	20	5698788	2.73E-02	3334	7.23E-01	Down	0.35	12217	1.56	0.01
rs7988601	54602	NDFIP2	13	78947784	2.73E-02	3335	8.68E-04	Down	3.33	3163	1.56	0.31
rs3740819	26973	CHORDC1	11	89568285	2.73E-02	3336	2.46E-01	Up	1.16	8724	1.56	0.06
rs1525629	154865	IQUB	7	122706807	2.73E-02	3337	7.70E-02	Up	1.77	6577	1.56	0.11
rs3795015	5990	RFX2	19	5971761	2.74E-02	3338	4.65E-01	Down	0.73	10462	1.56	0.03
rs4668168	6741	SSB	2	170478494	2.74E-02	3339	1.35E-01	Down	1.50	7507	1.56	0.09
rs3789643	22796	COG2	1	227116169	2.74E-02	3340	6.27E-01	Down	0.49	11628	1.56	0.02
rs1788101	10666	CD226	18	65691348	2.74E-02	3341	7.33E-01	Down	0.34	12272	1.56	0.01
rs1051795	11044	POLS	5	74932554	2.74E-02	3342	2.75E-01	Down	1.09	8996	1.56	0.06
rs268914	25818	KLK5	19	56134511	2.74E-02	3343	7.17E-01	Up	0.36	12174	1.56	0.01
rs2605618	56935	C11orf75	11	92883170	2.75E-02	3344	3.87E-04	Up	3.55	2869	1.56	0.34
rs178058	9342	SNAP29	22	19518158	2.75E-02	3345	3.77E-01	Up	0.88	9820	1.56	0.04
rs942704	779	CACNA1S	1	197781890	2.75E-02	3346	1.36E-01	Down	1.49	7527	1.56	0.09
rs567384	11343	MGLL	3	128976562	2.75E-02	3347	8.64E-04	Down	3.33	3161	1.56	0.31
rs10897315	9376	SLC22A8	11	62545707	2.75E-02	3348	1.34E-02	Down	2.47	4721	1.56	0.19
rs4749090	53904	MYO3A	10	26344078	2.75E-02	3349	1.10E-01	Up	1.60	7149	1.56	0.10
rs1054940	54776	PPP1R12C	19	60290536	2.75E-02	3350	3.71E-01	Down	0.89	9772	1.56	0.04
rs6803280	85443	DCLK3	3	36730707	2.76E-02	3351	6.32E-01	Down	0.48	11656	1.56	0.02
rs435397	9879	DDX46	5	134157963	2.76E-02	3352	5.27E-01	Up	0.63	10927	1.56	0.03
rs7859884	140803	TRPM6	9	74646960	2.76E-02	3353	2.60E-01	Down	1.13	8863	1.56	0.06
rs321930	162963	ZNF610	19	57527022	2.76E-02	3354	8.79E-05	Up	3.92	2420	1.56	0.41
rs6031491	78997	GDAP1L1	20	42329838	2.76E-02	3355	6.37E-01	Down	0.47	11684	1.56	0.02
rs12116291	395	ARHGAP6		11454265	2.76E-02	3356	8.85E-02	Down	1.70	6775	1.56	0.11
rs3815991	56675	NRIP3	11	8964245	2.76E-02	3357	8.71E-01	Up	0.16	13071	1.56	0.01
rs9513770	84899	TMTC4	13	100057953	2.76E-02	3358	4.60E-05	Up	4.08	2261	1.56	0.43
rs4965533	4205	MEF2A	15	98052198	2.76E-02	3359	2.10E-02	Down	2.31	5115	1.56	0.17
rs11597635	253738	EBF3	10	131601355	2.76E-02	3360	3.57E-01	Up	0.92	9651	1.56	0.04
rs6973105	27153	ZNF777	7	148582787	2.77E-02	3361	5.48E-01	Down	0.60	11067	1.56	0.03
rs12635549	50650	ARHGEF3	3	56804701	2.77E-02	3362	4.81E-02	Down	1.98	5952	1.56	0.13
rs4448992	51096	UTP18	17	46702833	2.77E-02	3363	1.14E-02	Up	2.53	4607	1.56	0.19
rs1078264	22983	MAST1	19	12824143	2.77E-02	3364	2.09E-03	Up	3.08	3559	1.56	0.27
rs1078264	83546	RTBDN	19	12824143	2.77E-02	3365	5.04E-02	Down	1.96	6007	1.56	0.13
rs1078264	23332	CLASP1	19	12824143	2.77E-02	3366	2.06E-01	Down	1.26	8317	1.56	0.07
rs3760582	6563	SLC14A1	18	41556762	2.77E-02	3367	3.00E-32	Down	11.82	52	1.56	3.15
rs2101469	6609	SMPD1	11	6353912	2.77E-02	3368	2.62E-02	Up	2.22	5329	1.56	0.16
rs6872314	10087	COL4A3BP	5	74771821	2.77E-02	3369	9.02E-02	Up	1.69	6822	1.56	0.10
rs1488	4216	MAP3K4	6	161508661	2.77E-02	3370	1.99E-04	Down	3.72	2628	1.56	0.37
rs7784465	5879	RAC1	7	6191515	2.77E-02	3371	8.80E-01	Down	0.15	13122	1.56	0.01
rs2164169	140881	DEFB129	20	154402	2.77E-02	3372	2.17E-02	Down	2.29	5151	1.56	0.17
rs6813436	2560	GABRB1	4	47231748	2.77E-02	3373	2.19E-02	Up	2.29	5152	1.56	0.17
rs11812546	58504	ARHGAP22	10	49371390	2.77E-02	3374	2.12E-01	Down	1.25	8378	1.56	0.07
rs12792158	283298	OLFML1	11	7458048	2.78E-02	3375	1.65E-02	Down	2.40	4912	1.56	0.18
rs7692779	92597	MOBKL1A	4	72138247	2.78E-02	3376	1.35E-01	Up	1.50	7506	1.56	0.09
rs10901291	25	ABL1	9	130747435	2.78E-02	3377	2.78E-01	Up	1.08	9017	1.56	0.06
rs17079451	10090	UST	6	149249537	2.78E-02	3378	4.59E-04	Down	3.50	2928	1.56	0.33
rs3745333	27151	CPAMD8	19	16896898	2.79E-02	3379	4.49E-05	Down	4.08	2257	1.55	0.43
rs7130686	10929	SFRS2B	11	94421162	2.79E-02	3380	1.17E-03	Up	3.25	3280	1.55	0.29
rs3903683	56063	C1orf91	1	32342001	2.79E-02	3381	7.67E-04	Up	3.36	3119	1.55	0.31
rs3903683	79140	CCDC28B	1	32342001	2.79E-02	3382	3.22E-01	Up	0.99	9380	1.55	0.05
rs3903683	55721	IQCC	1	32342001	2.79E-02	3383	5.10E-01	Down	0.66	10779	1.55	0.03
rs3903683	200081	TXLNA	1	32342001	2.79E-02	3384	7.19E-01	Down	0.36	12191	1.55	0.01
rs2849494	1837	DTNA	18	30410055	2.79E-02	3385	8.71E-07	Down	4.92	1559	1.55	0.61
rs3826798	257062	TMEM146	19	5736113	2.79E-02	3386	1.62E-01	Down	1.40	7844	1.55	0.08
rs9548848	122046	C13orf26	13	30411369	2.79E-02	3387	1.38E-01	Up	1.48	7551	1.55	0.09
rs2131431	7531	YWHAE	17	1241645	2.79E-02	3388	3.18E-03	Up	2.95	3811	1.55	0.25
rs12118471	54896	PQLC2	1	19404655	2.79E-02	3389	1.53E-01	Down	1.43	7723	1.55	0.08
rs11022342	55742	PARVA	11	12386480	2.80E-02	3390	1.69E-18	Down	8.78	249	1.55	1.78
rs251295	55914	ERBB2IP	5	65299846	2.80E-02	3391	7.13E-01	Up	0.37	12154	1.55	0.01
rs2287595	54476	RNF216	7	5528248	2.80E-02	3392	1.70E-01	Down	1.37	7917	1.55	0.08
rs6020255	7335	UBE2V1	20	48144793	2.80E-02	3393	2.06E-03	Up	3.08	3547	1.55	0.27
rs3829300	6996	TDG	12	102873317	2.80E-02	3394	2.02E-09	Up	6.00	938	1.55	0.87
rs1080307	260425	MAGI3	1	113928491	2.80E-02	3395	2.06E-04	Up	3.71	2642	1.55	0.37
rs16852868	7371	UCK2	1	162613627	2.80E-02	3396	1.03E-31	Up	11.63	56	1.55	3.10
rs4766959	6175	RPLP0	12	119091707	2.80E-02	3397	1.44E-18	Up	8.79	247	1.55	1.78

rs10873831	8543	LMO4	1	87524423	2.80E-02	3398	1.54E-04	Down	3.78	2557	1.55	0.38
rs11188059	1562	CYP2C18	10	96458889	2.80E-02	3399	1.95E-01	Up	1.30	8200	1.55	0.07
rs1790694	1824	DSC2	18	26896127	2.80E-02	3400	4.58E-06	Up	4.58	1800	1.55	0.53
rs7135018	259296	TAS2R50	12	11041507	2.80E-02	3401	6.06E-01	Down	0.52	11475	1.55	0.02
rs3741472	23014	FBXO21	12	116043433	2.80E-02	3402	5.96E-07	Down	4.99	1502	1.55	0.62
rs1374468	7884	SLBP	4	1691810	2.81E-02	3403	7.87E-05	Up	3.95	2386	1.55	0.41
rs1374468	10460	TACC3	4	1691810	2.81E-02	3404	2.01E-04	Up	3.72	2634	1.55	0.37
rs11168228	10411	RAPGEF3	12	46437218	2.81E-02	3405	1.43E-02	Down	2.45	4771	1.55	0.18
rs589811	9658	ZNF516	18	72198776	2.81E-02	3406	1.98E-19	Down	9.01	216	1.55	1.87
rs2057566	6049	RNF6	13	25660100	2.81E-02	3407	4.02E-01	Down	0.84	9997	1.55	0.04
rs13251987	55140	ELP3	8	27991454	2.81E-02	3408	2.67E-05	Down	4.20	2146	1.55	0.46
rs2275163	8564	KMO	1	238056095	2.81E-02	3409	3.80E-01	Down	0.88	9833	1.55	0.04
rs1011313	84062	DTNBP1	6	15741411	2.81E-02	3410	5.48E-01	Down	0.60	11065	1.55	0.03
rs3812874	64881	PCDH20	13	60887176	2.81E-02	3411	4.70E-03	Up	2.83	4020	1.55	0.23
rs7931173	4928	NUP98	11	3701741	2.82E-02	3412	9.14E-01	Down	0.11	13344	1.55	0.00
rs12525311	202	AIM1	6	107133123	2.82E-02	3413	1.54E-06	Up	4.81	1632	1.55	0.58
rs12525311	84816	RTN4IP1	6	107133123	2.82E-02	3414	2.41E-01	Down	1.17	8663	1.55	0.06
rs7657518	323	APBB2	4	40769306	2.82E-02	3415	2.52E-01	Down	1.15	8782	1.55	0.06
rs3851896	84224	NBPF3	1	21502640	2.82E-02	3416	6.19E-01	Up	0.50	11569	1.55	0.02
rs11762273	10095	ARPC1B	7	98621838	2.82E-02	3417	5.41E-01	Down	0.61	11019	1.55	0.03
rs12703600	27010	TPK1	7	143618802	2.82E-02	3418	4.66E-01	Down	0.73	10471	1.55	0.03
rs2429391	9902	MRC2	17	58094696	2.82E-02	3419	3.83E-02	Down	2.07	5712	1.55	0.14
rs9620825	150275	CCDC117	22	27490320	2.82E-02	3420	7.86E-03	Up	2.66	4328	1.55	0.21
rs464901	51807	TUBA8	22	16972056	2.82E-02	3421	3.80E-01	Up	0.88	9835	1.55	0.04
rs3087833	50628	GEMIN4	17	604480	2.82E-02	3422	6.00E-08	Up	5.42	1240	1.55	0.72
rs1390346	2823	GPM6A	4	177092801	2.82E-02	3423	1.44E-02	Down	2.45	4778	1.55	0.18
rs3732652	170506	DHX36	3	155501392	2.82E-02	3424	5.43E-02	Up	1.92	6096	1.55	0.13
rs10813928	55234	SMU1	9	33041685	2.83E-02	3425	4.79E-01	Down	0.71	10564	1.55	0.03
rs10009439	1400	CRMP1	4	5935615	2.83E-02	3426	1.27E-01	Up	1.52	7413	1.55	0.09
rs2924685	1374	CPT1A	11	68306644	2.83E-02	3427	4.75E-01	Down	0.71	10536	1.55	0.03
rs17751502	114794	ELFN2	22	36107851	2.83E-02	3428	5.47E-02	Up	1.92	6106	1.55	0.13
rs12458584	57565	KLHL14	18	28545956	2.83E-02	3429	9.03E-02	Down	1.69	6830	1.55	0.10
rs7604484	81606	LBH	2	30403207	2.83E-02	3430	4.44E-03	Up	2.85	3987	1.55	0.24
rs7604484	221491	C6orf1	2	30403207	2.83E-02	3431	9.29E-01	Up	0.09	13427	1.55	0.00
rs137988	9402	GRAP2	22	38661321	2.83E-02	3432	6.94E-01	Up	0.39	12035	1.55	0.02
rs7667590	952	CD38	4	15484997	2.83E-02	3433	5.00E-07	Down	5.03	1479	1.55	0.63
rs8029246	7782	SLC30A4	15	43608450	2.83E-02	3434	2.38E-02	Up	2.26	5238	1.55	0.16
rs8029246	283651	C15orf21	15	43608450	2.83E-02	3435	2.46E-01	Up	1.16	8727	1.55	0.06
rs742724	81030	ZBP1	20	55618538	2.83E-02	3436	3.54E-01	Up	0.93	9624	1.55	0.05
rs3817079	54946	SLC41A3	3	127224290	2.84E-02	3437	1.21E-01	Up	1.55	7320	1.55	0.09
rs10189072	28951	TRIB2	2	12822261	2.84E-02	3438	1.76E-13	Down	7.37	489	1.55	1.28
rs1475055	84071	ARMC2	6	109350925	2.85E-02	3439	9.79E-02	Up	1.66	6960	1.55	0.10
rs12127347	204962	SLC44A5	1	75491425	2.85E-02	3440	4.18E-04	Up	3.53	2896	1.55	0.34
rs629426	219927	MRPL21	11	68427680	2.85E-02	3441	1.16E-04	Up	3.85	2491	1.55	0.39
rs629426	3508	IGHMBP2	11	68427680	2.85E-02	3442	3.75E-02	Up	2.08	5692	1.55	0.14
rs6879012	134288	TMEM174	5	72498637	2.85E-02	3443	3.48E-01	Up	0.94	9573	1.55	0.05
rs2013562	7363	UGT2B4	4	70535335	2.85E-02	3444	1.95E-11	Up	6.73	662	1.55	1.07
rs3785073	7014	TERF2	16	67959438	2.85E-02	3445	4.66E-02	Down	1.99	5916	1.55	0.13
rs3785073	146456	TMED6	16	67959438	2.85E-02	3446	3.99E-01	Up	0.84	9976	1.55	0.04
rs2243154	3592	IL12A	3	161198944	2.85E-02	3447	1.20E-01	Up	1.56	7293	1.55	0.09
rs11066808	9904	RBM19	12	112810396	2.85E-02	3448	2.16E-01	Down	1.24	8427	1.54	0.07
rs1229363	92241	RCSD1	1	164369909	2.86E-02	3449	4.02E-01	Down	0.84	9990	1.54	0.04
rs12630303	6304	SATB1	3	18367555	2.86E-02	3450	7.71E-01	Down	0.29	12502	1.54	0.01
rs12731891	9015	TAF1A	1	219168343	2.86E-02	3451	2.61E-01	Up	1.12	8867	1.54	0.06
rs8111157	147841	SPC24	19	11119638	2.86E-02	3452	1.78E-01	Up	1.35	7995	1.54	0.07
rs1277459	2492	FSHR	2	49178844	2.87E-02	3453	6.10E-02	Up	1.87	6257	1.54	0.12
rs5063	4878	NPPA	1	11841914	2.87E-02	3454	2.38E-01	Down	1.18	8641	1.54	0.06
rs2357804	30001	ERO1L	14	52228076	2.87E-02	3455	2.22E-02	Down	2.29	5169	1.54	0.17
rs2357804	5706	PSMC6	14	52228076	2.87E-02	3456	1.38E-01	Up	1.48	7544	1.54	0.09
rs12701020	1395	CRHR2	7	30468201	2.87E-02	3457	7.03E-02	Up	1.81	6431	1.54	0.12
rs9999037	201931	TMEM192	4	166357800	2.87E-02	3458	1.03E-02	Down	2.57	4529	1.54	0.20
rs17041115	55530	SVOP	12	107834088	2.87E-02	3459	5.41E-01	Up	0.61	11020	1.54	0.03
rs3738806	79805	VASH2	1	209561852	2.87E-02	3460	2.10E-01	Up	1.25	8350	1.54	0.07
rs4964316	160428	ALDH1L2	12	103935544	2.87E-02	3461	2.22E-08	Down	5.59	1136	1.54	0.77
rs12837663	8239	USP9X		40820463	2.87E-02	3462	2.99E-04	Down	3.62	2770	1.54	0.35
rs7554895	5287	PIK3C2B	1	201183785	2.87E-02	3463	2.69E-02	Down	2.21	5355	1.54	0.16
rs13264830	54904	WHSC1L1	8	38260330	2.88E-02	3464	1.01E-03	Down	3.29	3225	1.54	0.30
rs13264830	84513	PPAPDC1B	8	38260330	2.88E-02	3465	1.37E-03	Up	3.20	3342	1.54	0.29
rs6076072	63908	NAPB	20	23354519	2.88E-02	3466	4.64E-01	Down	0.73	10455	1.54	0.03
rs6076072	256236	NAPSB	20	23354519	2.88E-02	3467	8.14E-01	Down	0.24	12747	1.54	0.01
rs7210758	11011	TLK2	17	58005113	2.88E-02	3468	1.72E-02	Down	2.38	4952	1.54	0.18
rs12564480	128025	WDR64	1	238155627	2.88E-02	3469	4.92E-01	Up	0.69	10663	1.54	0.03
rs17284653	8609	KLF7	2	207849951	2.88E-02	3470	1.04E-05	Down	4.41	1958	1.54	0.50
rs769434	4995	OR3A2	17	3128134	2.88E-02	3471	5.76E-01	Down	0.56	11287	1.54	0.02
rs17033585	2983	GUCY1B3	4	157093044	2.89E-02	3472	3.42E-01	Down	0.95	9538	1.54	0.05
rs11654690	5694	PSMB6	17	4651636	2.89E-02	3473	2.52E-02	Down	2.24	5297	1.54	0.16
rs11654690	5338	PLD2	17	4651636	2.89E-02	3474	5.52E-01	Down	0.59	11095	1.54	0.03
rs10064618	55568	GALNT10	5	153752482	2.89E-02	3475	1.96E-06	Up	4.76	1666	1.54	0.57
rs2409044	3906	LALBA	12	47247657	2.89E-02	3476	6.34E-01	Down	0.48	11668	1.54	0.02
rs1557501	5973	RENBP		152717977	2.89E-02	3477	7.17E-02	Down	1.80	6461	1.54	0.11
rs7191958	113	ADCY7	16	48889674	2.89E-02	3478	2.73E-01	Up	1.10	8976	1.54	0.06

rs17161687	10552	ARPC1A	7	98586560	2.89E-02	3479	1.93E-02	Up	2.34	5040	1.54	0.17
rs10886193	22841	RAB11FIP2	10	119768381	2.89E-02	3480	5.16E-12	Down	6.90	624	1.54	1.13
rs17030752	63892	THADA	2	43542144	2.89E-02	3481	1.78E-01	Up	1.35	7991	1.54	0.08
rs2427416	4923	NTSR1	20	60826690	2.90E-02	3482	1.81E-01	Down	1.34	8038	1.54	0.07
rs2473611	9590	AKAP12	6	151724203	2.90E-02	3483	2.23E-22	Down	9.73	155	1.54	2.17
rs1778013	9653	HS2ST1	1	87266633	2.90E-02	3484	1.48E-10	Up	6.44	762	1.54	0.98
rs864757	26146	TRAF3IP1	2	239098307	2.90E-02	3485	3.59E-01	Up	0.92	9669	1.54	0.04
rs9621049	6948	TCN2	22	29337973	2.90E-02	3486	2.10E-07	Down	5.19	1379	1.54	0.67
rs11048977	56938	ARNTL2	12	27388716	2.90E-02	3487	8.97E-02	Down	1.70	6804	1.54	0.10
rs9621049	339665	SLC35E4	22	29337973	2.90E-02	3488	6.27E-01	Down	0.49	11637	1.54	0.02
rs6969691	54809	SAMD9	7	92379634	2.90E-02	3489	5.36E-01	Down	0.62	10979	1.54	0.03
rs1839795	6092	ROBO2	3	77775815	2.90E-02	3490	9.26E-02	Down	1.68	6876	1.54	0.10
rs6553905	116966	WDR17	4	177349081	2.90E-02	3491	4.95E-01	Up	0.68	10681	1.54	0.03
rs3024718	8858	PROZ	13	112861854	2.91E-02	3492	3.37E-01	Up	0.96	9497	1.54	0.05
rs10743152	3630	INS	11	2152557	2.91E-02	3493	5.69E-01	Down	0.57	11236	1.54	0.02
rs10743152	7054	TH	11	2152557	2.91E-02	3494	8.25E-01	Down	0.22	12824	1.54	0.01
rs1808518	1618	DAZL	3	16602810	2.91E-02	3495	1.09E-01	Up	1.60	7131	1.54	0.10
rs1856193	304	ANXA2P2	9	33620008	2.91E-02	3496	4.43E-04	Down	3.51	2914	1.54	0.34
rs4781677	54820	NDE1	16	15637746	2.91E-02	3497	2.24E-08	Down	5.59	1137	1.54	0.76
rs4781677	9665	KIAA0430	16	15637746	2.91E-02	3498	2.11E-04	Down	3.71	2648	1.54	0.37
rs12670282	56829	ZC3HAV1	7	138259951	2.91E-02	3499	5.95E-02	Down	1.88	6228	1.54	0.12
rs12670282	79989	TTC26	7	138259951	2.91E-02	3500	3.14E-01	Up	1.01	9308	1.54	0.05
rs835207	735	C9	5	39385582	2.91E-02	3501	9.71E-01	Up	0.04	13706	1.54	0.00
rs3828581	1007	CDH9	5	26916282	2.91E-02	3502	3.23E-01	Up	0.99	9389	1.54	0.05
rs2028297	8800	PEX11A	15	88053402	2.91E-02	3503	9.27E-01	Up	0.09	13408	1.54	0.00
rs1688017	5348	FXYD1	19	40305463	2.91E-02	3504	4.51E-08	Down	5.47	1212	1.54	0.73
rs1688017	163175	LGI4	19	40305463	2.91E-02	3505	2.39E-05	Down	4.23	2121	1.54	0.46
rs1688017	5349	FXYD3	19	40305463	2.91E-02	3506	3.59E-01	Up	0.92	9675	1.54	0.04
rs4502159	11001	SLC27A2	15	48256251	2.91E-02	3507	1.45E-10	Up	6.41	777	1.54	0.98
rs4796105	246176	GAS2L2	17	31110080	2.92E-02	3508	8.47E-01	Down	0.19	12935	1.54	0.01
rs9922635	81831	NETO2	16	45713771	2.92E-02	3509	3.83E-10	Up	6.25	831	1.54	0.94
rs12684584	56254	RNF20	9	101387188	2.92E-02	3510	2.91E-01	Down	1.06	9124	1.53	0.05
rs3918003	1441	CSF3R	1	36600466	2.92E-02	3511	1.03E-03	Up	3.28	3234	1.53	0.30
rs3918003	64960	MRPS15	1	36600466	2.92E-02	3512	2.86E-03	Up	2.98	3756	1.53	0.25
rs1470453	5175	PECAM1	17	59758122	2.92E-02	3513	5.99E-01	Down	0.53	11423	1.53	0.02
rs2436514	25873	RPL36	19	5645630	2.92E-02	3514	5.23E-09	Up	5.84	1017	1.53	0.83
rs2436514	374875	HSD11B1L	19	5645630	2.92E-02	3515	2.16E-01	Down	1.24	8429	1.53	0.07
rs12130751	114883	OSBPL9	1	51877246	2.93E-02	3516	9.60E-01	Down	0.05	13621	1.53	0.00
rs7358275	9200	PTPLA	10	17701784	2.93E-02	3517	2.49E-10	Down	6.33	799	1.53	0.96
rs10109589	54108	CHRAC1	8	141599239	2.93E-02	3518	3.06E-03	Up	2.96	3787	1.53	0.25
rs8192440	1407	CRY1	12	105897573	2.93E-02	3519	1.10E-01	Up	1.60	7152	1.53	0.10
rs2839110	1292	COL6A2	21	46363388	2.93E-02	3520	2.73E-08	Down	5.56	1154	1.53	0.76
rs6657743	51097	SCCPDH	1	243219989	2.93E-02	3521	9.26E-10	Up	6.12	890	1.53	0.90
rs10913618	55103	RALGPS2	1	175417956	2.93E-02	3522	8.94E-01	Down	0.13	13207	1.53	0.00
rs12869996	1024	CDK8	13	25885527	2.93E-02	3523	9.39E-04	Down	3.31	3190	1.53	0.30
rs5964290	94122	SYTL5		37664844	2.94E-02	3524	2.00E-03	Down	3.09	3531	1.53	0.27
rs7620601	151887	CCDC80	3	113854268	2.94E-02	3525	1.60E-11	Down	6.74	660	1.53	1.08
rs4722665	3205	HOXA9	7	26970559	2.94E-02	3526	4.90E-09	Up	5.85	1010	1.53	0.83
rs4722665	3204	HOXA7	7	26970559	2.94E-02	3527	4.10E-02	Down	2.04	5773	1.53	0.14
rs4722665	3202	HOXA5	7	26970559	2.94E-02	3528	4.62E-01	Down	0.74	10441	1.53	0.03
rs4722665	3203	HOXA6	7	26970559	2.94E-02	3529	5.91E-01	Down	0.54	11378	1.53	0.02
rs6064731	51497	TH1L	20	56981340	2.94E-02	3530	3.15E-12	Up	6.98	604	1.53	1.15
rs9373046	2070	EYA4	6	133663689	2.94E-02	3531	3.43E-01	Down	0.95	9548	1.53	0.05
rs8142586	1415	CRYBB2	22	23943821	2.94E-02	3532	8.66E-02	Up	1.71	6750	1.53	0.11
rs7601	91433	RCCD1	15	89310596	2.95E-02	3533	7.26E-01	Up	0.35	12233	1.53	0.01
rs1077446	8612	PPAP2C	19	244913	2.95E-02	3534	9.61E-03	Up	2.59	4476	1.53	0.20
rs12199650	4172	MCM3	6	52232503	2.95E-02	3535	5.33E-02	Down	1.93	6073	1.53	0.13
rs10429454	29968	PSAT1	9	78136500	2.95E-02	3536	2.24E-05	Up	4.24	2110	1.53	0.46
rs7918164	503542	SPRN	10	135112204	2.95E-02	3537	6.95E-01	Up	0.39	12037	1.53	0.02
rs182767	55776	C6orf64	6	39199781	2.95E-02	3538	8.96E-03	Up	2.61	4435	1.53	0.20
rs6689634	6051	RNPEP	1	198686644	2.95E-02	3539	2.24E-01	Up	1.22	8497	1.53	0.06
rs2155339	60496	AASDHPPT	11	105467756	2.96E-02	3540	1.33E-01	Down	1.50	7474	1.53	0.09
rs2155339	143879	KBTBD3	11	105467756	2.96E-02	3541	3.54E-01	Down	0.93	9629	1.53	0.05
rs150583	146183	OTOA	16	21620244	2.96E-02	3542	4.79E-01	Up	0.71	10565	1.53	0.03
rs16849691	9283	GPR37L1	1	198830715	2.96E-02	3543	4.35E-01	Up	0.78	10227	1.53	0.04
rs11766495	23534	TNPO3	7	128294957	2.96E-02	3544	5.33E-02	Down	1.93	6070	1.53	0.13
rs6731113	55821	ALLC	2	3252699	2.96E-02	3545	1.87E-01	Down	1.32	8117	1.53	0.07
rs2660489	7409	VAV1	19	6747330	2.96E-02	3546	4.07E-01	Down	0.83	10030	1.53	0.04
rs7891628	3598	IL13RA2		114086016	2.96E-02	3547	8.27E-01	Down	0.22	12838	1.53	0.01
rs11801060	348487	FAM131C	1	16140165	2.97E-02	3548	2.48E-04	Down	3.66	2707	1.53	0.36
rs5759093	11252	PACSIN2	22	41734487	2.97E-02	3549	8.05E-01	Up	0.25	12703	1.53	0.01
rs1422673	10318	TNIP1	5	150419181	2.97E-02	3550	4.65E-02	Down	1.99	5912	1.53	0.13
rs4915503	5317	PKP1	1	197972420	2.97E-02	3551	2.78E-04	Down	3.63	2752	1.53	0.36
rs1483895	23150	FRMD4B	3	69445396	2.97E-02	3552	5.98E-01	Up	0.53	11420	1.53	0.02
rs3744405	1984	EIF5A	17	7133979	2.97E-02	3553	9.77E-01	Down	0.03	13747	1.53	0.00
rs10852892	146754	DNAH2	17	7673682	2.97E-02	3554	7.40E-02	Down	1.79	6507	1.53	0.11
rs10852892	23135	JMJD3	17	7673682	2.97E-02	3555	1.37E-01	Up	1.49	7536	1.53	0.09
rs6451696	64417	C5orf28	5	43489118	2.97E-02	3556	6.83E-02	Up	1.82	6400	1.53	0.12
rs3827730	11124	FAF1	1	50649869	2.97E-02	3557	5.00E-01	Up	0.68	10712	1.53	0.03
rs9841174	5276	SERPINI2	3	168667580	2.97E-02	3558	2.47E-01	Down	1.16	8738	1.53	0.06
rs4971226	7832	BTG2	1	199988457	2.97E-02	3559	7.85E-01	Up	0.27	12582	1.53	0.01

rs3095029	286006	C7orf53	7	111719735	2.98E-02	3560	1.65E-02	Up	2.40	4913	1.53	0.18
rs2670153	28978	TMEM14A	6	52648456	2.98E-02	3561	5.35E-01	Down	0.62	10975	1.53	0.03
rs11604322	51585	PCF11	11	82527700	2.98E-02	3562	6.66E-02	Up	1.83	6370	1.53	0.12
rs7844646	81551	STMN4	8	27159538	2.98E-02	3563	1.34E-01	Down	1.50	7491	1.53	0.09
rs11756337	79624	C6orf211	6	151878380	2.98E-02	3564	2.85E-01	Up	1.07	9075	1.53	0.05
rs4077413	1939	LGTN	1	203167037	2.98E-02	3565	1.13E-03	Up	3.25	3270	1.53	0.29
rs9844218	64332	NFKBIZ	3	103067673	2.99E-02	3566	2.00E-01	Up	1.28	8255	1.52	0.07
rs3127086	4892	NRAP	10	115374457	2.99E-02	3567	8.68E-07	Up	4.92	1558	1.52	0.61
rs3127086	65083	NOL6	10	115374457	2.99E-02	3568	1.57E-05	Up	4.32	2046	1.52	0.48
rs2427340	140893	C20orf151	20	60442015	2.99E-02	3569	8.78E-01	Down	0.15	13107	1.52	0.01
rs718604	154386	C6orf195	6	2589094	3.00E-02	3570	3.19E-01	Down	1.00	9352	1.52	0.05
rs1546929	594	BCKDHB	6	81104278	3.00E-02	3571	1.59E-01	Up	1.41	7800	1.52	0.08
rs1792774	834	CASP1	11	104417763	3.00E-02	3572	2.89E-17	Down	8.45	295	1.52	1.65
rs2074104	10900	RUNDC3A	17	39749020	3.00E-02	3573	9.82E-01	Down	0.02	13784	1.52	0.00
rs3767857	7678	ZNF124	1	243645530	3.00E-02	3574	3.89E-03	Up	2.89	3912	1.52	0.24
rs868150	7067	THRA	17	35466885	3.00E-02	3575	3.96E-02	Down	2.06	5740	1.52	0.14
rs2073724	5460	POU5F1	6	31237686	3.01E-02	3576	1.05E-01	Up	1.62	7077	1.52	0.10
rs2073724	6941	TCF19	6	31237686	3.01E-02	3577	2.30E-01	Down	1.20	8561	1.52	0.06
rs6587299	11255	HRH3	20	60231707	3.01E-02	3578	7.29E-01	Down	0.35	12246	1.52	0.01
rs2302837	23302	WSCD1	17	5954900	3.01E-02	3579	8.12E-02	Down	1.74	6654	1.52	0.11
rs4799869	80206	FHOD3	18	32416808	3.01E-02	3580	3.49E-07	Down	5.09	1434	1.52	0.65
rs2814707	56832	IFNK	9	27526397	3.01E-02	3581	1.68E-01	Up	1.38	7890	1.52	0.08
rs3796517	5981	RFC1	4	39159519	3.01E-02	3582	6.20E-01	Down	0.50	11577	1.52	0.02
rs12552499	11135	CDC42EP1	9	120977049	3.01E-02	3583	6.98E-01	Down	0.39	12055	1.52	0.02
rs4910663	81285	OR51E2	11	4649555	3.01E-02	3584	3.62E-12	Up	6.95	613	1.52	1.14
rs776025	79269	WDR32	9	37859282	3.01E-02	3585	2.38E-02	Up	2.26	5235	1.52	0.16
rs11080209	40	ACCN1	17	28616992	3.01E-02	3586	1.63E-01	Down	1.40	7847	1.52	0.08
rs7293	4151	MB	22	34331575	3.01E-02	3587	1.39E-01	Up	1.48	7558	1.52	0.09
rs7506929	84735	CNDP1	18	70392209	3.02E-02	3588	8.52E-01	Down	0.19	12967	1.52	0.01
rs896388	439	ASNA1	19	12710966	3.02E-02	3589	2.91E-01	Up	1.06	9122	1.52	0.05
rs896388	79002	C19orf43	19	12710966	3.02E-02	3590	3.10E-01	Down	1.02	9266	1.52	0.05
rs896388	30000	TNPO2	19	12710966	3.02E-02	3591	5.07E-01	Down	0.66	10762	1.52	0.03
rs3802357	158248	TTC16	9	127558207	3.02E-02	3592	7.21E-02	Down	1.80	6469	1.52	0.11
rs3802357	286207	C9orf117	9	127558207	3.02E-02	3593	4.33E-01	Down	0.78	10217	1.52	0.04
rs1862479	94030	LRRC4B	19	55721414	3.02E-02	3594	8.40E-02	Down	1.73	6699	1.52	0.11
rs1862479	126119	JOSD2	19	55721414	3.02E-02	3595	3.03E-01	Up	1.03	9209	1.52	0.05
rs1765886	8455	ATRN	20	3586043	3.02E-02	3596	1.44E-06	Up	4.82	1622	1.52	0.58
rs2373001	25797	QPCT	2	37510072	3.02E-02	3597	9.71E-01	Up	0.04	13703	1.52	0.00
rs362784	5649	RELN	7	102784791	3.02E-02	3598	1.28E-02	Up	2.49	4677	1.52	0.19
rs867522	3360	HTR4	5	147946439	3.02E-02	3599	9.08E-01	Down	0.12	13296	1.52	0.00
rs7925666	8322	FZD4	11	86347183	3.02E-02	3600	4.16E-06	Up	4.60	1786	1.52	0.54
rs17284624	25834	MGAT4C	12	85010214	3.02E-02	3601	4.17E-01	Up	0.81	10101	1.52	0.04
rs7508262	51298	THEG	19	314127	3.02E-02	3602	2.08E-01	Down	1.26	8331	1.52	0.07
rs3118766	169841	ZNF169	9	94104853	3.03E-02	3603	8.49E-01	Down	0.19	12946	1.52	0.01
rs17365723	2787	GNG5	1	84681040	3.04E-02	3604	4.27E-03	Up	2.86	3977	1.52	0.24
rs17365723	80135	BXDC5	1	84681040	3.04E-02	3605	1.55E-01	Up	1.42	7743	1.52	0.08
rs17365723	64173	SPATA1	1	84681040	3.04E-02	3606	9.53E-01	Down	0.06	13571	1.52	0.00
rs2607426	53916	RAB4B	19	45966553	3.04E-02	3607	2.33E-02	Down	2.27	5215	1.52	0.16
rs2607426	8190	MIA	19	45966553	3.04E-02	3608	5.14E-02	Down	1.95	6019	1.52	0.13
rs11023371	120227	CYP2R1	11	14852847	3.04E-02	3609	1.56E-01	Up	1.42	7759	1.52	0.08
rs12636440	79885	HDAC11	3	13490826	3.04E-02	3610	2.29E-01	Down	1.20	8557	1.52	0.06
rs17824797	5265	SERPINA1	14	93929206	3.04E-02	3611	8.84E-01	Down	0.15	13149	1.52	0.01
rs11610238	51729	WBP11	12	14866988	3.04E-02	3612	8.14E-03	Up	2.65	4353	1.52	0.21
rs11610238	144608	C12orf60	12	14866988	3.04E-02	3613	6.93E-01	Down	0.39	12025	1.52	0.02
rs10125663	3371	TNC	9	114965970	3.04E-02	3614	4.83E-10	Down	6.22	853	1.52	0.93
rs2251715	1117	CHI3L2	1	111490229	3.04E-02	3615	7.67E-02	Down	1.77	6569	1.52	0.11
rs521116	135152	B3GAT2	6	71666105	3.05E-02	3616	1.80E-01	Down	1.34	8020	1.52	0.07
rs11607454	2132	EXT2	11	44159923	3.05E-02	3617	6.19E-01	Up	0.50	11574	1.52	0.02
rs3903775	147409	DSG4	18	27245503	3.06E-02	3618	1.20E-01	Down	1.55	7301	1.51	0.09
rs2569491	43847	KLK14	19	56276728	3.06E-02	3619	5.05E-01	Up	0.67	10749	1.51	0.03
rs2569491	26085	KLK13	19	56276728	3.06E-02	3620	8.67E-01	Down	0.17	13042	1.51	0.01
rs6492939	56924	PAK6	15	38349182	3.06E-02	3621	1.10E-01	Down	1.60	7146	1.51	0.10
rs174675	10587	TXNRD2	22	18308605	3.06E-02	3622	9.03E-02	Up	1.69	6829	1.51	0.10
rs174675	1312	COMT	22	18308605	3.06E-02	3623	8.60E-01	Up	0.18	13009	1.51	0.01
rs1050331	83637	ZMIZ2	7	44581331	3.06E-02	3624	1.67E-02	Up	2.39	4922	1.51	0.18
rs10818454	54187	NANS	9	97899495	3.06E-02	3625	1.62E-02	Up	2.40	4902	1.51	0.18
rs3776421	23549	DNPEP	5	10763247	3.06E-02	3626	1.14E-02	Up	2.53	4606	1.51	0.19
rs3776421	1611	DAP	5	10763247	3.06E-02	3627	1.91E-02	Up	2.34	5030	1.51	0.17
rs3776421	3375	IAPP	5	10763247	3.06E-02	3628	8.93E-02	Up	1.70	6790	1.51	0.10
rs7909932	26098	C10orf137	10	127390423	3.06E-02	3629	2.66E-10	Up	6.32	802	1.51	0.96
rs1409379	145173	B3GALTL	13	30805741	3.06E-02	3630	2.54E-05	Down	4.21	2136	1.51	0.46
rs2553449	79807	GSTCD	4	107056496	3.06E-02	3631	6.39E-01	Up	0.47	11697	1.51	0.02
rs1005793	596	BCL2	18	59041140	3.07E-02	3632	1.30E-09	Down	6.07	909	1.51	0.89
rs623400	79961	DENND2D	1	111460130	3.07E-02	3633	5.58E-01	Up	0.59	11144	1.51	0.03
rs11170305	196374	KRT78	12	51534088	3.07E-02	3634	4.38E-04	Up	3.52	2909	1.51	0.34
rs11170305	338785	KRT79	12	51534088	3.07E-02	3635	4.46E-01	Up	0.76	10314	1.51	0.04
rs995743	55109	AGGF1	5	76378585	3.07E-02	3636	1.89E-02	Up	2.35	5018	1.51	0.17
rs757232	23526	HMHA1	19	1026979	3.07E-02	3637	3.57E-02	Up	2.10	5637	1.51	0.14
rs757232	3881	KRT31	19	1026979	3.07E-02	3638	8.97E-01	Up	0.13	13229	1.51	0.00
rs6089164	3055	HCK	20	30121280	3.07E-02	3639	4.90E-03	Down	2.81	4053	1.51	0.23
rs10466556	83871	RAB34	11	107308918	3.08E-02	3640	1.57E-21	Down	9.53	170	1.51	2.08

rs10466556	54734	RAB39	11	107308918	3.08E-02	3641	9.83E-01	Down	0.02	13798	1.51	0.00
rs2647462	2058	EPRS	1	216544485	3.09E-02	3642	2.12E-04	Up	3.70	2650	1.51	0.37
rs4666881	10787	NCKAP1	2	183748098	3.09E-02	3643	1.58E-08	Down	5.65	1095	1.51	0.78
rs3856318	7799	PRDM2	1	13765498	3.09E-02	3644	3.99E-01	Down	0.84	9975	1.51	0.04
rs11435	153769	SH3RF2	5	145422326	3.09E-02	3645	6.76E-01	Up	0.42	11921	1.51	0.02
rs2743937	3135	HLA-G	6	29915902	3.10E-02	3646	3.33E-01	Down	0.97	9464	1.51	0.05
rs10134946	256281	NUDT14	14	104725651	3.10E-02	3647	1.28E-04	Up	3.83	2510	1.51	0.39
rs10134946	3714	JAG2	14	104725651	3.10E-02	3648	4.41E-01	Down	0.77	10273	1.51	0.04
rs4713791	29993	PACSIN1	6	34524871	3.10E-02	3649	9.46E-02	Down	1.67	6904	1.51	0.10
rs2055566	9855	FARP2	2	242070927	3.10E-02	3650	3.14E-03	Up	2.95	3800	1.51	0.25
rs3814474	3912	LAMB1	7	107238759	3.10E-02	3651	3.66E-08	Down	5.51	1191	1.51	0.74
rs2946851	24144	TFIP11	19	54626813	3.10E-02	3652	2.70E-02	Down	2.21	5360	1.51	0.16
rs2946851	57030	SLC17A7	19	54626813	3.10E-02	3653	7.18E-01	Down	0.36	12188	1.51	0.01
rs1169	2804	GOLGB1	3	122864894	3.10E-02	3654	2.17E-02	Up	2.30	5148	1.51	0.17
rs1169	3059	HCLS1	3	122864894	3.10E-02	3655	6.98E-02	Down	1.81	6420	1.51	0.12
rs2442825	55209	SETD5	3	9454142	3.10E-02	3656	2.02E-02	Up	2.32	5085	1.51	0.17
rs730483	4689	NCF4	22	35572378	3.10E-02	3657	5.55E-05	Down	4.03	2313	1.51	0.43
rs10219235	25884	CHRDL2	11	74087111	3.11E-02	3658	2.48E-01	Down	1.16	8744	1.51	0.06
rs967935	8714	ABCC3	17	46110387	3.12E-02	3659	4.21E-06	Down	4.60	1787	1.51	0.54
rs1294688	2280	FKBP1A	20	1316127	3.12E-02	3660	7.22E-01	Up	0.36	12212	1.51	0.01
rs236114	51605	TRMT6	20	5883385	3.12E-02	3661	2.61E-02	Up	2.22	5324	1.51	0.16
rs236114	84515	MCM8	20	5883385	3.12E-02	3662	9.49E-01	Up	0.06	13545	1.51	0.00
rs200479	8357	HIST1H3H	6	27879663	3.12E-02	3663	3.55E-05	Up	4.14	2207	1.51	0.44
rs200479	8329	HIST1H2AI	6	27879663	3.12E-02	3664	2.73E-01	Up	1.10	8979	1.51	0.06
rs200479	8342	HIST1H2BM	6	27879663	3.12E-02	3665	2.78E-01	Up	1.09	9013	1.51	0.06
rs200479	8340	HIST1H2BL	6	27879663	3.12E-02	3666	6.07E-01	Up	0.51	11485	1.51	0.02
rs12085929	9672	SDC3	1	31041827	3.12E-02	3667	3.75E-02	Down	2.08	5693	1.51	0.14
rs363016	6616	SNAP25	20	10179174	3.12E-02	3668	1.20E-03	Down	3.24	3295	1.51	0.29
rs11045425	53919	SLCO1C1	12	20785247	3.12E-02	3669	2.18E-01	Up	1.23	8435	1.51	0.07
rs2014827	7565	ZNF17	19	62623115	3.13E-02	3670	1.33E-01	Down	1.50	7477	1.51	0.09
rs2363536	963	CD53	1	111138091	3.13E-02	3671	8.65E-01	Up	0.17	13032	1.51	0.01
rs7314021	51267	CLEC1A	12	10159311	3.13E-02	3672	3.52E-02	Down	2.11	5631	1.50	0.15
rs3816186	57504	MTA3	2	42848198	3.13E-02	3673	1.53E-01	Up	1.43	7717	1.50	0.08
rs1699387	132320	SCLT1	4	130389103	3.13E-02	3674	1.38E-01	Up	1.48	7549	1.50	0.09
rs10488630	3663	IRF5	7	128187899	3.13E-02	3675	9.63E-01	Up	0.05	13638	1.50	0.00
rs3130215	3116	HLA-DPB2	6	33182941	3.13E-02	3676	5.98E-01	Up	0.53	11416	1.50	0.02
rs744454	23633	KPNA6	1	32226766	3.13E-02	3677	5.73E-03	Down	2.76	4154	1.50	0.22
rs744454	55116	TMEM39B	1	32226766	3.13E-02	3678	1.06E-01	Down	1.62	7093	1.50	0.10
rs10889147	5538	PPT1	1	40230564	3.13E-02	3679	1.02E-03	Up	3.28	3232	1.50	0.30
rs888767	9141	PDCD5	19	37768455	3.13E-02	3680	1.29E-02	Up	2.49	4684	1.50	0.19
rs2195525	9099	USP2	11	118740614	3.13E-02	3681	2.47E-03	Down	3.03	3678	1.50	0.26
rs2195525	83552	MFRP	11	118740614	3.13E-02	3682	7.49E-01	Down	0.32	12369	1.50	0.01
rs16854770	27347	STK39	2	168812274	3.13E-02	3683	1.66E-06	Up	4.79	1643	1.50	0.58
rs1662988	3777	KCNK3	2	26851885	3.14E-02	3684	1.75E-04	Down	3.75	2584	1.50	0.38
rs2173091	123624	AGBL1	15	84598403	3.14E-02	3685	1.99E-04	Down	3.72	2629	1.50	0.37
rs4800278	114799	ESCO1	18	17402270	3.14E-02	3686	9.60E-01	Up	0.05	13619	1.50	0.00
rs3795837	467	ATF3	1	209161723	3.14E-02	3687	4.75E-02	Up	1.98	5935	1.50	0.13
rs3823231	2045	EPHA7	6	94016082	3.14E-02	3688	1.52E-01	Up	1.43	7703	1.50	0.08
rs2546098	153643	FAM81B	5	94775044	3.15E-02	3689	5.69E-01	Down	0.57	11233	1.50	0.02
rs17783921	866	SERPINA6	14	93864002	3.15E-02	3690	3.20E-01	Up	0.99	9358	1.50	0.05
rs7342880	7077	TIMP2	17	74386107	3.15E-02	3691	3.34E-08	Down	5.52	1177	1.50	0.75
rs12144133	10277	UBE4B	1	10138452	3.15E-02	3692	9.97E-01	Down	0.00	13887	1.50	0.00
rs2081767	26003	GORASP2	2	171606138	3.15E-02	3693	3.68E-07	Up	5.08	1443	1.50	0.64
rs281413	125950	RAVER1	19	10307734	3.15E-02	3694	2.43E-01	Down	1.17	8690	1.50	0.06
rs16853834	471	ATIC	2	216035742	3.15E-02	3695	3.49E-01	Up	0.94	9579	1.50	0.05
rs9682783	80723	TMEM22	3	138022387	3.15E-02	3696	1.30E-01	Down	1.51	7449	1.50	0.09
rs2307073	27122	DKK3	11	11934149	3.15E-02	3697	3.08E-27	Down	10.81	84	1.50	2.65
rs1196475	5803	PTPRZ1	7	121243174	3.15E-02	3698	1.16E-03	Down	3.25	3279	1.50	0.29
rs2368936	5818	PVRL1	11	119106989	3.16E-02	3699	1.37E-02	Down	2.47	4736	1.50	0.19
rs10109834	2185	PTK2B	8	27268193	3.16E-02	3700	5.52E-02	Down	1.92	6119	1.50	0.13
rs11634652	23251	KIAA1024	15	77536502	3.16E-02	3701	2.78E-03	Up	2.99	3748	1.50	0.26
rs1540053	127	ADH4	4	100439332	3.16E-02	3702	4.85E-01	Down	0.70	10609	1.50	0.03
rs492840	120400	FAM55A	11	113898524	3.17E-02	3703	5.36E-02	Down	1.93	6077	1.50	0.13
rs6475469	54914	KIAA1797	9	20661512	3.17E-02	3704	3.09E-01	Up	1.02	9258	1.50	0.05
rs12117219	84144	SYDE2	1	85393288	3.17E-02	3705	2.64E-01	Down	1.12	8902	1.50	0.06
rs10896172	5883	RAD9A	11	66932268	3.17E-02	3706	3.11E-02	Up	2.16	5498	1.50	0.15
rs9836536	2049	EPHB3	3	185790641	3.18E-02	3707	4.49E-03	Up	2.84	3994	1.50	0.23
rs3732680	6161	RPL32	3	12848265	3.18E-02	3708	9.86E-06	Up	4.42	1948	1.50	0.50
rs3736591	79690	GAL3ST4	7	99395668	3.18E-02	3709	3.48E-01	Down	0.94	9576	1.50	0.05
rs3736591	55262	C7orf43	7	99395668	3.18E-02	3710	6.39E-01	Down	0.47	11701	1.50	0.02
rs9994557	9508	ADAMTS3	4	73511309	3.18E-02	3711	1.14E-07	Up	5.30	1308	1.50	0.69
rs1322179	1557	CYP2C19	10	96565232	3.19E-02	3712	5.70E-01	Up	0.57	11245	1.50	0.02
rs3003217	257044	C1orf101	1	241041480	3.19E-02	3713	5.66E-01	Down	0.57	11215	1.50	0.02
rs13092046	5067	CNTN3	3	74617642	3.19E-02	3714	1.07E-02	Up	2.55	4556	1.50	0.20
rs17001508	150350	ENTHD1	22	38610770	3.19E-02	3715	9.60E-02	Up	1.66	6925	1.50	0.10
rs7879713	56062	KLHL4		86564586	3.19E-02	3716	9.00E-02	Down	1.70	6809	1.50	0.10
rs8112256	79709	GLT25D1	19	17510791	3.19E-02	3717	5.41E-01	Up	0.61	11026	1.50	0.03
rs8112256	199786	FAM129C	19	17510791	3.19E-02	3718	9.81E-01	Down	0.02	13783	1.50	0.00
rs1033717	55346	TCP11L1	11	33023147	3.19E-02	3719	4.64E-02	Up	1.99	5907	1.50	0.13
rs12140760	25824	PRDX5	1	170161652	3.19E-02	3720	4.55E-04	Down	3.51	2922	1.50	0.33
rs12140760	9588	PRDX6	1	170161652	3.19E-02	3721	6.27E-02	Down	1.86	6292	1.50	0.12

rs17095224	259217	HSPA12A	10	118529233	3.19E-02	3722	2.25E-03	Down	3.06	3613	1.50	0.26
rs8065080	7442	TRPV1	17	3427196	3.20E-02	3723	1.10E-02	Up	2.54	4575	1.50	0.20
rs17118767	51350	KRT76	12	51439068	3.20E-02	3724	5.03E-01	Up	0.67	10741	1.50	0.03
rs9901726	8388	OR1E2	17	3290284	3.20E-02	3725	3.86E-01	Down	0.87	9887	1.50	0.04
rs9901726	84690	SPATA22	17	3290284	3.20E-02	3726	5.79E-01	Up	0.55	11309	1.50	0.02
rs9971764	6433	SFRS8	12	130914362	3.20E-02	3727	5.54E-02	Down	1.92	6124	1.49	0.13
rs7549511	4259	MGST3	1	162353261	3.20E-02	3728	6.05E-13	Down	7.20	535	1.49	1.22
rs7549511	223	ALDH9A1	1	162353261	3.20E-02	3729	6.72E-02	Down	1.83	6380	1.49	0.12
rs17035286	23433	RHOQ	2	46710660	3.20E-02	3730	6.32E-04	Down	3.42	3049	1.49	0.32
rs17035286	5281	PIGF	2	46710660	3.20E-02	3731	4.91E-01	Up	0.69	10657	1.49	0.03
rs10911098	6041	RNASEL	1	179268155	3.20E-02	3732	2.38E-03	Down	3.04	3657	1.49	0.26
rs11844642	6400	SEL1L	14	81011029	3.21E-02	3733	4.79E-44	Up	13.96	11	1.49	4.33
rs2453176	57472	CNOT6	5	179908398	3.21E-02	3734	1.82E-11	Up	6.72	669	1.49	1.07
rs3759406	25875	LETMD1	12	49728018	3.21E-02	3735	5.99E-04	Up	3.43	3027	1.49	0.32
rs3922678	22898	DENND3	8	142266863	3.21E-02	3736	1.91E-02	Down	2.34	5028	1.49	0.17
rs9321367	116843	C6orf192	6	133126052	3.21E-02	3737	1.56E-02	Down	2.42	4847	1.49	0.18
rs9321367	8875	VNN2	6	133126052	3.21E-02	3738	8.99E-02	Up	1.70	6806	1.49	0.10
rs1341573	51086	TNNI3K	1	74608837	3.21E-02	3739	7.56E-01	Up	0.31	12414	1.49	0.01
rs10459096	283316	CD163L1	12	7477862	3.21E-02	3740	7.48E-03	Up	2.67	4302	1.49	0.21
rs10503601	57509	MTUS1	8	17569748	3.21E-02	3741	4.30E-07	Down	5.06	1461	1.49	0.64
rs11667458	2639	GCDH	19	12880554	3.21E-02	3742	6.75E-01	Up	0.42	11918	1.49	0.02
rs1468460	54555	DDX49	19	18877108	3.22E-02	3743	3.12E-02	Up	2.15	5500	1.49	0.15
rs7522428	1429	CRYZ	1	74890381	3.22E-02	3744	3.45E-05	Down	4.14	2200	1.49	0.45
rs7558703	2043	EPHA4	2	222199928	3.22E-02	3745	8.74E-03	Down	2.62	4408	1.49	0.21
rs1426878	83539	CHST9	18	23013564	3.22E-02	3746	2.07E-04	Down	3.71	2645	1.49	0.37
rs7046713	11168	PSIP1	9	15448921	3.22E-02	3747	1.80E-12	Down	7.05	574	1.49	1.17
rs7147979	5527	PPP2R5C	14	101417545	3.22E-02	3748	1.49E-02	Down	2.43	4808	1.49	0.18
rs12569261	83593	RASSF5	1	203115655	3.22E-02	3749	8.41E-06	Down	4.45	1915	1.49	0.51
rs9600792	115207	KCTD12	13	76344013	3.22E-02	3750	9.78E-01	Down	0.03	13755	1.49	0.00
rs11608153	53826	FXYD6	11	117221873	3.22E-02	3751	2.01E-02	Down	2.32	5081	1.49	0.17
rs17647588	4780	NFE2L2	2	177961584	3.23E-02	3752	1.58E-08	Down	5.65	1096	1.49	0.78
rs7964528	22818	COPZ1	12	53009051	3.23E-02	3753	7.39E-02	Up	1.79	6505	1.49	0.11
rs12637714	353274	ZNF445	3	44456157	3.23E-02	3754	7.95E-01	Down	0.26	12640	1.49	0.01
rs713253	7984	ARHGEF5	7	143512661	3.23E-02	3755	3.84E-01	Up	0.87	9867	1.49	0.04
rs10272075	26958	COPG2	7	129771354	3.23E-02	3756	1.85E-01	Up	1.32	8089	1.49	0.07
rs11578	79716	NPEPL1	20	56686681	3.23E-02	3757	3.41E-01	Down	0.95	9533	1.49	0.05
rs342774	10910	SUGT1	13	52116249	3.23E-02	3758	9.96E-01	Down	0.01	13883	1.49	0.00
rs4641304	57127	RHBG	1	153155990	3.23E-02	3759	5.29E-01	Up	0.63	10935	1.49	0.03
rs7636389	84343	HPS3	3	150363824	3.24E-02	3760	1.48E-01	Up	1.45	7657	1.49	0.08
rs7965049	9332	CD163	12	7537044	3.24E-02	3761	5.68E-01	Up	0.57	11226	1.49	0.02
rs7030493	23670	TMEM2	9	71577978	3.24E-02	3762	1.39E-09	Up	6.05	916	1.49	0.89
rs17107219	9832	JAKMIP2	5	147064219	3.25E-02	3763	7.31E-01	Up	0.34	12257	1.49	0.01
rs3794186	1119	CHKA	11	67577612	3.25E-02	3764	1.84E-12	Up	7.05	579	1.49	1.17
rs3794186	10312	TCIRG1	11	67577612	3.25E-02	3765	3.47E-03	Down	2.92	3853	1.49	0.25
rs1497050	283461	C12orf40	12	38593865	3.25E-02	3766	8.58E-01	Down	0.18	12999	1.49	0.01
rs4240896	5293	PIK3CD	1	9647766	3.25E-02	3767	3.83E-01	Down	0.87	9863	1.49	0.04
rs7692195	55351	STK32B	4	5178497	3.25E-02	3768	1.10E-03	Down	3.26	3257	1.49	0.30
rs9910163	26073	POLDIP2	17	23715448	3.25E-02	3769	3.38E-02	Up	2.12	5583	1.49	0.15
rs9910163	23098	SARM1	17	23715448	3.25E-02	3770	8.36E-01	Up	0.21	12881	1.49	0.01
rs9910163	7448	VTN	17	23715448	3.25E-02	3771	9.32E-01	Up	0.09	13445	1.49	0.00
rs2215021	7039	TGFA	2	70707912	3.25E-02	3772	6.31E-01	Down	0.48	11649	1.49	0.02
rs12849208	1121	CHM		85049850	3.25E-02	3773	9.75E-02	Up	1.66	6950	1.49	0.10
rs2037012	9908	G3BP2	4	76924154	3.25E-02	3774	1.91E-06	Up	4.76	1661	1.49	0.57
rs2037012	8999	CDKL2	4	76924154	3.25E-02	3775	1.49E-02	Down	2.44	4807	1.49	0.18
rs3734261	6882	TAF11	6	34948376	3.25E-02	3776	5.70E-01	Up	0.57	11237	1.49	0.02
rs363322	6571	SLC18A2	10	118976134	3.25E-02	3777	2.14E-02	Down	2.30	5139	1.49	0.17
rs17510173	55761	TTC17	11	43465745	3.25E-02	3778	1.20E-05	Up	4.38	1989	1.49	0.49
rs11248331	118670	FAM24A	10	124658619	3.26E-02	3779	8.54E-01	Down	0.18	12979	1.49	0.01
rs2627775	87178	PNPT1	2	55788764	3.26E-02	3780	2.41E-17	Up	8.48	291	1.49	1.66
rs784548	50485	SMARCAL1	2	217090863	3.26E-02	3781	9.37E-04	Up	3.31	3188	1.49	0.30
rs10914625	8565	YARS	1	32967915	3.27E-02	3782	2.15E-03	Up	3.07	3579	1.49	0.27
rs10914625	64766	S100PBP	1	32967915	3.27E-02	3783	8.39E-01	Down	0.20	12897	1.49	0.01
rs2284340	10516	FBLN5	14	91458800	3.27E-02	3784	9.56E-08	Down	5.33	1293	1.49	0.70
rs2307418	9970	NR1I3	1	158013659	3.27E-02	3785	2.16E-01	Down	1.24	8419	1.49	0.07
rs2072049	23532	PRAME	22	21215635	3.27E-02	3786	3.31E-01	Up	0.97	9453	1.49	0.05
rs743562	3567	IL5	5	131900282	3.27E-02	3787	5.45E-01	Down	0.61	11049	1.49	0.03
rs637462	114609	TIRAP	11	125681788	3.27E-02	3788	8.86E-01	Up	0.14	13161	1.49	0.01
rs2270003	25973	PARS2	1	54960723	3.28E-02	3789	2.00E-02	Up	2.33	5075	1.48	0.17
rs2270003	55001	TTC22	1	54960723	3.28E-02	3790	1.05E-01	Down	1.62	7074	1.48	0.10
rs4770474	55504	TNFRSF19	13	23153384	3.28E-02	3791	5.68E-01	Down	0.57	11228	1.48	0.02
rs214097	4925	NUCB2	11	17248075	3.28E-02	3792	2.08E-08	Up	5.61	1125	1.48	0.77
rs352165	211	ALAS1	3	52217942	3.28E-02	3793	2.13E-02	Down	2.30	5133	1.48	0.17
rs1448903	10863	ADAM28	2	207134467	3.28E-02	3794	4.50E-02	Down	2.00	5876	1.48	0.13
rs1448903	8745	ADAM23	2	207134467	3.28E-02	3795	8.28E-01	Up	0.22	12843	1.48	0.01
rs2843434	7692	ZNF133	20	18212468	3.28E-02	3796	8.01E-01	Up	0.25	12682	1.48	0.01
rs10899440	79053	ALG8	11	77502923	3.28E-02	3797	1.17E-15	Up	8.00	359	1.48	1.49
rs4301800	64137	ABCG4	11	118524730	3.29E-02	3798	1.78E-01	Up	1.35	8000	1.48	0.07
rs2445837	4606	MYBPC2	19	55613084	3.29E-02	3799	5.95E-02	Down	1.88	6226	1.48	0.12
rs2445837	5424	POLD1	19	55613084	3.29E-02	3800	2.83E-01	Up	1.07	9059	1.48	0.05
rs2445837	6689	SPIB	19	55613084	3.29E-02	3801	8.85E-01	Up	0.14	13158	1.48	0.01
rs2159415	7289	TULP3	12	2924718	3.29E-02	3802	5.79E-01	Down	0.55	11311	1.48	0.02

rs1148095	57476	GRAMD1B	11	122976029	3.29E-02	3803	6.85E-02	Down	1.82	6401	1.48	0.12
rs3743642	9013	TAF1C	16	82770429	3.29E-02	3804	6.16E-01	Down	0.50	11545	1.48	0.02
rs12559968	10735	STAG2		122850318	3.29E-02	3805	1.89E-03	Up	3.11	3508	1.48	0.27
rs3829037	83940	TATDN1	8	125634509	3.29E-02	3806	1.46E-03	Up	3.18	3364	1.48	0.28
rs3829037	4715	NDUFB9	8	125634509	3.29E-02	3807	2.47E-03	Up	3.03	3677	1.48	0.26
rs9637599	152926	PPM1K	4	89563409	3.30E-02	3808	2.72E-01	Down	1.10	8970	1.48	0.06
rs12014280	80231	CXorf21		30352582	3.30E-02	3809	2.42E-02	Down	2.25	5255	1.48	0.16
rs10962128	203238	C9orf93	9	15716057	3.30E-02	3810	2.78E-01	Down	1.09	9015	1.48	0.06
rs6426257	81472	OR2C3	1	244042039	3.30E-02	3811	3.85E-07	Down	5.08	1451	1.48	0.64
rs4684147	2199	FBLN2	3	13596533	3.30E-02	3812	1.95E-06	Down	4.76	1664	1.48	0.57
rs2389907	5911	RAP2A	13	96874912	3.30E-02	3813	2.15E-02	Up	2.30	5143	1.48	0.17
rs941425	54363	HAO1	20	7853283	3.30E-02	3814	7.52E-01	Down	0.32	12383	1.48	0.01
rs4129319	8404	SPARCL1	4	88752564	3.30E-02	3815	2.30E-17	Down	8.48	292	1.48	1.66
rs2073778	54487	DGCR8	22	18449129	3.30E-02	3816	4.28E-02	Up	2.03	5820	1.48	0.14
rs2850992	5530	PPP3CA	4	102479933	3.30E-02	3817	8.37E-15	Up	7.78	399	1.48	1.41
rs6596945	8737	RIPK1	6	3016912	3.30E-02	3818	4.77E-04	Down	3.49	2947	1.48	0.33
rs1620668	7482	WNT2B	1	112736022	3.30E-02	3819	5.09E-02	Down	1.95	6012	1.48	0.13
rs13379920	53346	TM6SF1	15	81547397	3.30E-02	3820	9.16E-01	Up	0.11	13355	1.48	0.00
rs9622555	3560	IL2RB	22	35880085	3.30E-02	3821	8.74E-01	Up	0.16	13087	1.48	0.01
rs10500653	53840	TRIM34	11	5583983	3.31E-02	3822	1.16E-02	Down	2.52	4615	1.48	0.19
rs10500653	117854	TRIM6	11	5583983	3.31E-02	3823	4.54E-01	Down	0.75	10374	1.48	0.03
rs772175	55654	TMEM127	2	96366427	3.31E-02	3824	8.08E-07	Down	4.93	1549	1.48	0.61
rs772175	9391	CIAO1	2	96366427	3.31E-02	3825	9.74E-03	Up	2.58	4491	1.48	0.20
rs1984564	11331	PHB2	12	6960454	3.31E-02	3826	6.72E-07	Up	4.97	1517	1.48	0.62
rs1984564	10436	EMG1	12	6960454	3.31E-02	3827	4.15E-02	Up	2.04	5787	1.48	0.14
rs7350950	10951	CBX1	17	43518866	3.31E-02	3828	3.24E-02	Down	2.14	5537	1.48	0.15
rs888786	134359	C5orf37	5	75006606	3.31E-02	3829	1.49E-01	Up	1.44	7676	1.48	0.08
rs2777888	9208	LRRFIP1	3	49873004	3.31E-02	3830	7.74E-07	Up	4.94	1536	1.48	0.61
rs2777888	79012	CAMKV	3	49873004	3.31E-02	3831	4.73E-03	Down	2.82	4022	1.48	0.23
rs2777888	10293	TRAIP	3	49873004	3.31E-02	3832	7.47E-02	Up	1.78	6523	1.48	0.11
rs3793790	1103	CHAT	10	50510742	3.32E-02	3833	3.08E-01	Down	1.02	9250	1.48	0.05
rs4255644	1284	COL4A2	13	109887574	3.32E-02	3834	1.39E-08	Down	5.67	1083	1.48	0.79
rs2395626	4736	RPL10A	6	35530732	3.32E-02	3835	2.70E-06	Up	4.69	1716	1.48	0.56
rs2395626	2178	FANCE	6	35530732	3.32E-02	3836	2.41E-02	Down	2.26	5250	1.48	0.16
rs157134	114819	CROCCL2	1	16543062	3.32E-02	3837	2.52E-01	Down	1.15	8776	1.48	0.06
rs9494573	5191	PEX7	6	137176697	3.32E-02	3838	1.42E-04	Up	3.80	2533	1.48	0.38
rs7291533	6899	TBX1	22	18112164	3.32E-02	3839	1.81E-03	Up	3.12	3482	1.48	0.27
rs4410081	151354	FAM84A	16	27162850	3.32E-02	3840	2.81E-06	Up	4.68	1723	1.48	0.56
rs4410081	197370	NSMCE1	16	27162850	3.32E-02	3841	4.53E-06	Up	4.59	1797	1.48	0.53
rs12631730	29072	SETD2	3	47104907	3.33E-02	3842	3.23E-01	Up	0.99	9388	1.48	0.05
rs1152005	5468	PPARG	3	12458451	3.33E-02	3843	7.53E-06	Down	4.48	1885	1.48	0.51
rs535614	5991	RFX3	9	3236491	3.33E-02	3844	1.87E-03	Up	3.11	3496	1.48	0.27
rs7779313	100128553	CTAGE4	7	143299398	3.33E-02	3845	8.13E-01	Up	0.24	12742	1.48	0.01
rs10099140	2515	ADAM2	8	39799000	3.33E-02	3846	3.99E-04	Up	3.54	2881	1.48	0.34
rs10739691	84253	GARNL3	9	127187992	3.33E-02	3847	3.90E-01	Up	0.86	9917	1.48	0.04
rs11096544	93034	NT5C1B	2	18689012	3.34E-02	3848	4.36E-01	Down	0.78	10238	1.48	0.04
rs527790	59340	HRH4	18	20314913	3.34E-02	3849	2.69E-01	Up	1.11	8939	1.48	0.06
rs4598427	140886	PABPC5		90509686	3.34E-02	3850	3.60E-01	Up	0.91	9680	1.48	0.04
rs6511689	1786	DNMT1	19	10182089	3.34E-02	3851	1.57E-01	Down	1.41	7770	1.48	0.08
rs6798348	11170	FAM107A	3	58533809	3.34E-02	3852	3.87E-18	Down	8.68	266	1.48	1.74
rs500464	29901	SAC3D1	11	64550536	3.34E-02	3853	1.38E-07	Up	5.27	1332	1.48	0.69
rs500464	402	ARL2	11	64550536	3.34E-02	3854	4.59E-02	Down	2.00	5891	1.48	0.13
rs500464	29907	SNX15	11	64550536	3.34E-02	3855	1.41E-01	Down	1.47	7586	1.48	0.09
rs6952809	55501	CHST12	7	2221734	3.34E-02	3856	5.06E-01	Down	0.67	10758	1.48	0.03
rs682408	5289	PIK3C3	18	37827591	3.34E-02	3857	3.77E-01	Up	0.88	9823	1.48	0.04
rs791608	3952	LEP	7	127466943	3.34E-02	3858	5.79E-01	Up	0.55	11304	1.48	0.02
rs7166031	55180	LINS1	15	98925912	3.35E-02	3859	5.69E-01	Down	0.57	11230	1.48	0.02
rs9297145	57154	SMURF1	7	98403768	3.35E-02	3860	9.82E-01	Up	0.02	13792	1.47	0.00
rs13277059	286128	ZFP41	8	144395067	3.35E-02	3861	3.87E-01	Up	0.87	9892	1.47	0.04
rs2107525	144699	FBXL14	12	1547380	3.35E-02	3862	9.08E-06	Up	4.44	1927	1.47	0.50
rs1046200	64779	MTHFSD	16	85122172	3.35E-02	3863	7.28E-01	Down	0.35	12238	1.47	0.01
rs7661189	55300	PI4K2B	4	24926434	3.35E-02	3864	1.71E-01	Down	1.37	7924	1.47	0.08
rs6947538	2051	EPHB6	7	142079573	3.36E-02	3865	1.48E-04	Down	3.79	2549	1.47	0.38
rs12594938	83440	ADPGK	15	70858335	3.36E-02	3866	1.06E-03	Up	3.28	3245	1.47	0.30
rs6947538	55503	TRPV6	7	142079573	3.36E-02	3867	8.59E-03	Up	2.63	4392	1.47	0.21
rs509360	746	C11orf10	11	61305135	3.36E-02	3868	2.63E-02	Up	2.22	5333	1.47	0.16
rs509360	2237	FEN1	11	61305135	3.36E-02	3869	3.36E-02	Up	2.12	5578	1.47	0.15
rs509360	745	C11orf9	11	61305135	3.36E-02	3870	1.59E-01	Up	1.41	7798	1.47	0.08
rs12446463	6338	SCNN1B	16	23294894	3.36E-02	3871	7.18E-02	Down	1.80	6464	1.47	0.11
rs10502878	9063	PIAS2	18	42645819	3.36E-02	3872	5.36E-03	Up	2.78	4108	1.47	0.23
rs13346080	1954	MEGF8	19	47579332	3.36E-02	3873	4.75E-01	Down	0.71	10533	1.47	0.03
rs11195353	282996	RBM20	10	112594018	3.36E-02	3874	9.27E-01	Up	0.09	13407	1.47	0.00
rs2276307	9177	HTR3B	11	113309097	3.37E-02	3875	5.61E-01	Down	0.58	11171	1.47	0.03
rs1801783	56997	CABC1	1	223487593	3.37E-02	3876	4.76E-03	Up	2.82	4026	1.47	0.23
rs4346637	27235	COQ2	4	84532414	3.37E-02	3877	8.82E-01	Down	0.15	13141	1.47	0.01
rs10510434	7182	NR2C2	3	14995353	3.37E-02	3878	1.03E-02	Up	2.57	4530	1.47	0.20
rs4903606	9517	SPTLC2	14	77106688	3.37E-02	3879	9.78E-05	Up	3.90	2445	1.47	0.40
rs7020893	549	AUH	9	91200885	3.37E-02	3880	1.09E-02	Up	2.55	4569	1.47	0.20
rs13031025	284996	RNF149	2	101376029	3.37E-02	3881	2.87E-04	Up	3.63	2763	1.47	0.35
rs2111459	79828	METTL8	2	171999770	3.38E-02	3882	8.96E-01	Up	0.13	13218	1.47	0.00
rs2837982	25825	BACE2	21	41538601	3.38E-02	3883	5.17E-01	Down	0.65	10838	1.47	0.03

rs2732255	84750	FUT10	8	33416970	3.38E-02	3884	8.73E-02	Down	1.71	6759	1.47	0.11
rs134821	2192	FBLN1	22	44290566	3.38E-02	3885	5.74E-07	Down	5.00	1500	1.47	0.62
rs732686	79669	C3orf52	3	113286470	3.38E-02	3886	5.11E-01	Up	0.66	10787	1.47	0.03
rs1832741	9314	KLF4	9	107321084	3.38E-02	3887	3.71E-01	Up	0.89	9776	1.47	0.04
rs12577147	114902	C1QTNF5	11	118723858	3.38E-02	3888	2.04E-05	Up	4.26	2093	1.47	0.47
rs3743607	10428	CFDP1	16	73896356	3.38E-02	3889	4.75E-01	Down	0.71	10535	1.47	0.03
rs12351219	7091	TLE4	9	79456255	3.38E-02	3890	6.13E-03	Down	2.74	4179	1.47	0.22
rs2073241	5083	PAX9	14	36198687	3.38E-02	3891	3.16E-01	Up	1.00	9331	1.47	0.05
rs3806776	2982	GUCY1A3	4	156944409	3.39E-02	3892	1.64E-28	Up	11.00	78	1.47	2.78
rs4815961	113278	C20orf54	20	700633	3.39E-02	3893	9.75E-04	Down	3.30	3208	1.47	0.30
rs2587472	140766	ADAMTS14	10	72185839	3.39E-02	3894	1.59E-01	Down	1.41	7803	1.47	0.08
rs12067005	22823	MTF2	1	93255140	3.39E-02	3895	2.81E-02	Up	2.20	5393	1.47	0.16
rs17054546	64641	EBF2	8	25810608	3.39E-02	3896	2.33E-05	Up	4.23	2114	1.47	0.46
rs1943012	54808	DYM	18	45114915	3.40E-02	3897	2.39E-01	Down	1.18	8647	1.47	0.06
rs881375	26147	PHF19	9	120732452	3.40E-02	3898	7.23E-03	Down	2.69	4279	1.47	0.21
rs417309	5902	RANBP1	22	18473098	3.40E-02	3899	5.46E-02	Up	1.92	6103	1.47	0.13
rs2638500	3851	KRT4	12	51510668	3.40E-02	3900	1.25E-01	Down	1.53	7373	1.47	0.09
rs2076295	1832	DSP	6	7508231	3.40E-02	3901	7.16E-01	Up	0.36	12171	1.47	0.01
rs2076193	9053	MAP7	6	136784828	3.40E-02	3902	6.06E-23	Up	9.82	147	1.47	2.22
rs7754833	135112	NCOA7	6	126232587	3.40E-02	3903	4.69E-01	Up	0.72	10490	1.47	0.03
rs138270	54456	MOV10L1	22	48890436	3.40E-02	3904	2.72E-02	Up	2.21	5368	1.47	0.16
rs1445410	2138	EYA1	8	72395542	3.40E-02	3905	4.57E-03	Down	2.84	4007	1.47	0.23
rs1230165	128	ADH5	4	100343551	3.41E-02	3906	5.15E-17	Down	8.38	309	1.47	1.63
rs1230165	23173	METAP1	4	100343551	3.41E-02	3907	2.43E-03	Down	3.03	3664	1.47	0.26
rs3789246	5243	ABCB1	7	86812678	3.41E-02	3908	7.99E-01	Up	0.25	12667	1.47	0.01
rs6741884	27436	EML4	2	42352485	3.41E-02	3909	2.61E-12	Up	7.00	594	1.47	1.16
rs3210714	6678	SPARC	5	151022125	3.41E-02	3910	2.63E-08	Down	5.56	1150	1.47	0.76
rs13093428	51560	RAB6B	3	135055017	3.41E-02	3911	4.00E-03	Up	2.88	3934	1.47	0.24
rs2687076	1551	CYP3A7	7	98951919	3.41E-02	3912	8.44E-01	Up	0.20	12925	1.47	0.01
rs3858090	10558	SPTLC1	9	91932563	3.42E-02	3913	4.97E-03	Down	2.81	4067	1.47	0.23
rs10232205	11097	NUPL2	7	23003794	3.42E-02	3914	3.24E-06	Up	4.65	1754	1.47	0.55
rs12410298	8515	ITGA10	1	143037007	3.42E-02	3915	5.37E-01	Down	0.62	10990	1.47	0.03
rs10414066	2872	MKNK2	19	2012715	3.42E-02	3916	1.50E-04	Up	3.79	2550	1.47	0.38
rs10414066	126308	MOBKL2A	19	2012715	3.42E-02	3917	3.08E-01	Down	1.02	9247	1.47	0.05
rs2734827	7352	UCP3	11	73393925	3.42E-02	3918	2.12E-01	Down	1.25	8376	1.47	0.07
rs2733537	7508	XPC	3	14186105	3.42E-02	3919	3.58E-06	Down	4.63	1768	1.47	0.54
rs2733537	27258	LSM3	3	14186105	3.42E-02	3920	1.78E-01	Up	1.35	7990	1.47	0.08
rs13052393	1409	CRYAA	21	43442435	3.42E-02	3921	8.92E-03	Down	2.62	4430	1.47	0.20
rs17624707	9358	ITGBL1	13	101168511	3.42E-02	3922	6.85E-12	Up	6.88	628	1.47	1.12
rs757420	8913	CACNA1G	17	46062181	3.42E-02	3923	3.53E-01	Up	0.93	9617	1.47	0.05
rs3771327	6775	STAT4	2	191746277	3.42E-02	3924	6.71E-01	Up	0.43	11892	1.47	0.02
rs13039601	5203	PFDN4	20	52274441	3.43E-02	3925	1.33E-01	Up	1.50	7481	1.47	0.09
rs740436	6929	TCF3	19	1585595	3.43E-02	3926	2.02E-03	Up	3.09	3537	1.46	0.27
rs7535730	4774	NFIA	1	61583377	3.43E-02	3927	1.29E-07	Down	5.28	1319	1.46	0.69
rs9891711	162461	TMEM92	17	45709230	3.43E-02	3928	2.55E-01	Down	1.14	8809	1.46	0.06
rs11896539	2202	EFEMP1	2	56075555	3.43E-02	3929	3.68E-11	Down	6.62	697	1.46	1.04
rs2646254	1293	COL6A3	2	238049717	3.44E-02	3930	3.31E-06	Down	4.65	1756	1.46	0.55
rs673151	2946	GSTM2	1	109925500	3.44E-02	3931	4.34E-42	Down	13.59	14	1.46	4.14
rs673151	2948	GSTM4	1	109925500	3.44E-02	3932	7.20E-26	Down	10.52	103	1.46	2.51
rs673151	2944	GSTM1	1	109925500	3.44E-02	3933	2.74E-14	Down	7.61	437	1.46	1.36
rs2328537	5325	PLAGL1	6	144365265	3.44E-02	3934	1.42E-15	Down	7.98	363	1.46	1.48
rs476663	6478	SIAH2	3	151932581	3.45E-02	3935	3.83E-02	Up	2.07	5711	1.46	0.14
rs12421228	283208	P4HA3	11	73685163	3.45E-02	3936	5.94E-02	Up	1.89	6223	1.46	0.12
rs11784491	7534	YWHAZ	8	102047339	3.45E-02	3937	3.57E-01	Up	0.92	9649	1.46	0.04
rs9597055	90627	STARD13	13	32722988	3.46E-02	3938	5.92E-04	Down	3.44	3024	1.46	0.32
rs3825271	57613	KIAA1467	12	13107338	3.46E-02	3939	1.24E-09	Up	6.07	907	1.46	0.89
rs315796	3937	LCP2	5	169676139	3.46E-02	3940	7.40E-01	Up	0.33	12315	1.46	0.01
rs650058	5950	RBP4	10	95368537	3.46E-02	3941	5.56E-04	Down	3.45	2999	1.46	0.33
rs650058	5433	POLR2D	10	95368537	3.46E-02	3942	1.52E-02	Up	2.43	4819	1.46	0.18
rs650058	5146	PDE6C	10	95368537	3.46E-02	3943	4.73E-01	Up	0.72	10521	1.46	0.03
rs10791333	219938	SPATA19	11	133238071	3.46E-02	3944	1.79E-01	Up	1.34	8007	1.46	0.07
rs12535503	816	CAMK2B	7	44150073	3.47E-02	3945	9.66E-01	Up	0.04	13658	1.46	0.00
rs746332	6258	RXRG	1	162111230	3.47E-02	3946	8.11E-01	Up	0.24	12724	1.46	0.01
rs17312015	5638	PRRG1		37026026	3.47E-02	3947	7.37E-01	Down	0.34	12296	1.46	0.01
rs3933772	9150	CTDP1	18	75609169	3.47E-02	3948	6.77E-02	Down	1.83	6390	1.46	0.12
rs743466	1476	CSTB	21	44015433	3.47E-02	3949	1.32E-07	Up	5.28	1321	1.46	0.69
rs743466	8568	RRP1	21	44015433	3.47E-02	3950	6.52E-01	Up	0.45	11783	1.46	0.02
rs12695641	5523	PPP2R3A	3	137290307	3.47E-02	3951	7.31E-09	Down	5.78	1034	1.46	0.81
rs9508556	5412	UBL3	13	29278407	3.48E-02	3952	1.92E-02	Down	2.34	5035	1.46	0.17
rs11792480	2022	ENG	9	127677679	3.48E-02	3953	1.58E-01	Down	1.41	7784	1.46	0.08
rs17078914	956	ENTPD3	3	40420122	3.48E-02	3954	8.53E-03	Down	2.63	4384	1.46	0.21
rs4239838	7327	UBE2G2	21	45026737	3.48E-02	3955	1.89E-01	Down	1.31	8134	1.46	0.07
rs2255515	57608	KIAA1462	10	30395199	3.48E-02	3956	1.39E-04	Down	3.81	2525	1.46	0.39
rs16963476	399687	MYO18A	17	24545602	3.48E-02	3957	2.61E-03	Down	3.01	3711	1.46	0.26
rs2422691	7053	TGM3	20	2266925	3.48E-02	3958	1.04E-06	Up	4.88	1578	1.46	0.60
rs6553010	4543	MTNR1A	4	187831492	3.48E-02	3959	2.28E-01	Down	1.21	8538	1.46	0.06
rs3740853	55693	JMJD2D	11	94371407	3.48E-02	3960	4.00E-01	Down	0.84	9983	1.46	0.04
rs6428503	2634	GBP2	1	89296090	3.49E-02	3961	2.05E-20	Down	9.26	190	1.46	1.97
rs726789	84658	EMR3	19	14604003	3.49E-02	3962	8.11E-01	Up	0.24	12727	1.46	0.01
rs26845	1632	DCI	16	2234891	3.49E-02	3963	1.33E-08	Up	5.68	1079	1.46	0.79
rs26845	10921	RNPS1	16	2234891	3.49E-02	3964	3.66E-02	Up	2.09	5668	1.46	0.14

rs26845	1877	E4F1	16	2234891	3.49E-02	3965	8.54E-01	Up	0.18	12981	1.46	0.01
rs26845	1775	DNASE1L2	16	2234891	3.49E-02	3966	8.68E-01	Up	0.17	13054	1.46	0.01
rs6731864	285025	CCDC141	2	179561512	3.49E-02	3967	1.26E-01	Up	1.53	7392	1.46	0.09
rs11829726	1975	EIF4B	12	51724073	3.49E-02	3968	3.74E-05	Up	4.12	2214	1.46	0.44
rs11829726	23371	TENC1	12	51724073	3.49E-02	3969	2.02E-02	Down	2.32	5084	1.46	0.17
rs17004598	11077	HSF2BP	21	43902984	3.50E-02	3970	7.21E-01	Down	0.36	12202	1.46	0.01
rs12517292	54819	ZCCHC10	5	132383046	3.50E-02	3971	1.30E-01	Down	1.51	7452	1.46	0.09
rs6580083	9421	HAND1	5	153845922	3.50E-02	3972	7.34E-01	Down	0.34	12276	1.46	0.01
rs6592090	220042	C11orf82	11	82326005	3.50E-02	3973	6.62E-01	Down	0.44	11838	1.46	0.02
rs9425618	5279	PIGC	1	169146504	3.50E-02	3974	8.37E-01	Up	0.21	12885	1.46	0.01
rs11934603	8310	ACOX3	4	8512447	3.50E-02	3975	6.56E-01	Up	0.45	11805	1.46	0.02
rs2300688	529	ATP6V1E1	22	16468846	3.50E-02	3976	9.28E-08	Down	5.34	1289	1.46	0.70
rs1923939	2746	GLUD1	10	88818148	3.51E-02	3977	1.53E-01	Up	1.43	7710	1.46	0.08
rs8102236	84941	HSH2D	19	16111595	3.51E-02	3978	3.14E-01	Down	1.01	9303	1.45	0.05
rs8102236	4218	RAB8A	19	16111595	3.51E-02	3979	7.39E-01	Down	0.33	12311	1.45	0.01
rs2705343	9990	SLC12A6	15	32333213	3.51E-02	3980	1.14E-01	Up	1.58	7207	1.45	0.09
rs4724425	107	ADCY1	7	45495159	3.51E-02	3981	4.24E-01	Up	0.80	10154	1.45	0.04
rs11195887	6975	TECTB	10	114062662	3.51E-02	3982	9.80E-01	Up	0.03	13770	1.45	0.00
rs2244135	706	TSPO	2	119842166	3.51E-02	3983	1.62E-03	Up	3.15	3426	1.45	0.28
rs2244135	1622	DBI	2	119842166	3.51E-02	3984	4.88E-01	Up	0.69	10639	1.45	0.03
rs9391920	94234	FOXQ1	6	1240625	3.51E-02	3985	1.07E-05	Down	4.40	1963	1.45	0.50
rs1861309	7035	TFPI	2	188195347	3.51E-02	3986	6.55E-01	Down	0.45	11797	1.45	0.02
rs7068223	84159	ARID5B	10	63351428	3.52E-02	3987	4.59E-01	Up	0.74	10421	1.45	0.03
rs6510326	85415	RHPN2	19	38205182	3.52E-02	3988	2.57E-09	Up	5.96	954	1.45	0.86
rs9986517	57699	CPNE5	6	36827057	3.52E-02	3989	9.78E-01	Down	0.03	13753	1.45	0.00
rs12118914	93183	PIGM	1	156805251	3.52E-02	3990	4.21E-02	Up	2.03	5800	1.45	0.14
rs2305779	79883	PODNL1	19	13906033	3.52E-02	3991	3.18E-03	Down	2.95	3813	1.45	0.25
rs2305779	54862	CC2D1A	19	13906033	3.52E-02	3992	6.40E-01	Down	0.47	11708	1.45	0.02
rs7119084	4319	MMP10	11	102154445	3.53E-02	3993	1.63E-04	Up	3.77	2572	1.45	0.38
rs1985119	8685	MARCO	2	119426630	3.53E-02	3994	8.80E-04	Down	3.33	3168	1.45	0.31
rs754382	55048	VPS37C	11	60656343	3.53E-02	3995	4.49E-05	Up	4.08	2256	1.45	0.43
rs4387216	10352	WARS2	1	119392338	3.53E-02	3996	5.82E-04	Up	3.44	3016	1.45	0.32
rs17590228	3927	LASP1	17	34290556	3.53E-02	3997	1.10E-01	Down	1.60	7145	1.45	0.10
rs738993	6523	SLC5A1	22	30761894	3.53E-02	3998	2.42E-01	Down	1.17	8675	1.45	0.06
rs494893	7468	WHSC1	4	1925402	3.54E-02	3999	1.92E-13	Up	7.36	491	1.45	1.27
rs13615	9510	ADAMTS1	21	27131538	3.54E-02	4000	1.76E-11	Down	6.73	667	1.45	1.08
rs1609666	128239	IQGAP3	1	153316064	3.54E-02	4001	8.22E-01	Up	0.22	12802	1.45	0.01
rs1801105	3176	HNMT	2	138593381	3.54E-02	4002	2.55E-02	Down	2.23	5302	1.45	0.16
rs9870250	7348	UPK1B	3	120374687	3.54E-02	4003	8.01E-01	Up	0.25	12681	1.45	0.01
rs1006049	525	ATP6V1B1	2	71088315	3.54E-02	4004	4.94E-01	Down	0.68	10674	1.45	0.03
rs12557174	6756	SSX1		47874017	3.54E-02	4005	2.00E-01	Up	1.28	8260	1.45	0.07
rs4657178	9722	NOS1AP	1	158942268	3.54E-02	4006	4.63E-01	Up	0.73	10450	1.45	0.03
rs7907300	92565	FANK1	10	127646057	3.54E-02	4007	5.70E-01	Down	0.57	11249	1.45	0.02
rs8107315	90317	ZNF616	19	57339705	3.55E-02	4008	1.48E-04	Up	3.79	2546	1.45	0.38
rs166575	50506	DUOX2	15	43169290	3.55E-02	4009	1.28E-02	Down	2.49	4671	1.45	0.19
rs1514399	260436	C4orf7	4	71271883	3.55E-02	4010	5.54E-01	Down	0.59	11108	1.45	0.03
rs1514399	1448	CSN3	4	71271883	3.55E-02	4011	9.80E-01	Up	0.03	13771	1.45	0.00
rs6865121	22948	CCT5	5	10288046	3.55E-02	4012	3.20E-03	Up	2.95	3818	1.45	0.25
rs7917290	55506	H2AFY2	10	71536167	3.56E-02	4013	7.95E-02	Down	1.75	6623	1.45	0.11
rs388914	4624	MYH6	14	22942932	3.56E-02	4014	2.85E-01	Down	1.07	9071	1.45	0.05
rs388914	4625	MYH7	14	22942932	3.56E-02	4015	7.07E-01	Up	0.38	12116	1.45	0.02
rs401302	7060	THBS4	5	79391227	3.56E-02	4016	1.15E-24	Up	10.20	121	1.45	2.39
rs1054623	84330	ZNF414	19	8492086	3.56E-02	4017	8.24E-01	Down	0.22	12816	1.45	0.01
rs1054623	4542	MYO1F	19	8492086	3.56E-02	4018	9.93E-01	Down	0.01	13866	1.45	0.00
rs1871538	4796	NFKBIL2	8	145648828	3.56E-02	4019	1.39E-02	Down	2.46	4748	1.45	0.19
rs1871538	90990	KIFC2	8	145648828	3.56E-02	4020	3.85E-02	Up	2.07	5714	1.45	0.14
rs7815513	81501	TM7SF4	8	105414963	3.56E-02	4021	6.79E-01	Down	0.41	11938	1.45	0.02
rs13402702	23160	WDR43	2	29092053	3.57E-02	4022	4.19E-05	Up	4.10	2240	1.45	0.44
rs4793624	1747	DLX3	17	45414776	3.57E-02	4023	6.52E-02	Down	1.84	6344	1.45	0.12
rs4793624	1748	DLX4	17	45414776	3.57E-02	4024	1.53E-01	Up	1.43	7722	1.45	0.08
rs685017	6445	SGCG	13	22689027	3.57E-02	4025	1.17E-01	Down	1.57	7247	1.45	0.09
rs2185089	28231	SLCO4A1	20	60779985	3.57E-02	4026	8.14E-01	Up	0.24	12745	1.45	0.01
rs11670736	163227	ZNF100	19	21744576	3.57E-02	4027	4.92E-01	Up	0.69	10664	1.45	0.03
rs10202544	9392	TGFBRAP1	2	105346067	3.57E-02	4028	2.42E-02	Up	2.25	5258	1.45	0.16
rs12050150	394	ARHGAP5	14	31637758	3.57E-02	4029	8.86E-01	Down	0.14	13163	1.45	0.01
rs4520196	5168	ENPP2	8	120669575	3.57E-02	4030	6.93E-11	Down	6.52	735	1.45	1.02
rs9783669	122622	ADSSL1	14	104286396	3.58E-02	4031	6.60E-05	Down	3.99	2349	1.45	0.42
rs9783669	10572	SIVA1	14	104286396	3.58E-02	4032	7.09E-02	Down	1.81	6448	1.45	0.11
rs6790564	23171	GPD1L	3	32116895	3.58E-02	4033	2.21E-19	Down	9.00	218	1.45	1.87
rs17799872	109	ADCY3	2	24956608	3.58E-02	4034	2.98E-09	Down	5.93	967	1.45	0.85
rs17799872	79172	CENPO	2	24956608	3.58E-02	4035	3.51E-02	Up	2.11	5628	1.45	0.15
rs916055	246	ALOX15	17	4481583	3.58E-02	4036	1.79E-02	Up	2.37	4982	1.45	0.17
rs332271	23604	DAPK2	15	62126801	3.58E-02	4037	6.89E-01	Down	0.40	12001	1.45	0.02
rs7259103	22847	ZNF507	19	37508411	3.58E-02	4038	4.18E-04	Up	3.53	2897	1.45	0.34
rs6542074	6574	SLC20A1	2	113145983	3.58E-02	4039	6.41E-04	Up	3.41	3053	1.45	0.32
rs7543444	55740	ENAH	1	221997068	3.58E-02	4040	3.80E-30	Down	11.41	61	1.45	2.94
rs937837	7047	TGM4	3	44907274	3.58E-02	4041	5.28E-06	Down	4.55	1828	1.45	0.53
rs12208647	114781	BTBD9	6	38516201	3.58E-02	4042	7.11E-01	Up	0.37	12136	1.45	0.01
rs2292283	5515	PPP2CA	5	133591217	3.59E-02	4043	6.10E-04	Up	3.43	3033	1.45	0.32
rs2270982	2874	GPS2	17	7167436	3.59E-02	4044	1.66E-08	Down	5.64	1100	1.45	0.78
rs750783	55571	C2orf29	2	101347824	3.59E-02	4045	6.70E-03	Up	2.71	4232	1.45	0.22

rs7479861	10522	DEAF1	11	692250	3.59E-02	4046	8.15E-06	Up	4.46	1908	1.45	0.51
rs7479861	64787	EPS8L2	11	692250	3.59E-02	4047	9.84E-01	Up	0.02	13808	1.45	0.00
rs1563357	89766	UMODL1	21	42429404	3.59E-02	4048	7.99E-01	Up	0.25	12670	1.45	0.01
rs8076809	2081	ERN1	17	59535040	3.59E-02	4049	2.29E-03	Up	3.05	3623	1.45	0.26
rs204732	57466	SFRS15	21	32014714	3.59E-02	4050	6.09E-01	Up	0.51	11499	1.45	0.02
rs7540478	58155	PTBP2	1	96945186	3.59E-02	4051	3.14E-03	Down	2.95	3802	1.45	0.25
rs12579612	10154	PLXNC1	12	93168261	3.59E-02	4052	7.75E-02	Down	1.77	6586	1.44	0.11
rs11039528	5795	PTPRJ	11	48100921	3.59E-02	4053	1.81E-03	Up	3.12	3480	1.44	0.27
rs1158066	199221	DZIP1L	3	139336956	3.59E-02	4054	2.05E-01	Down	1.27	8300	1.44	0.07
rs1158066	51146	A4GNT	3	139336956	3.59E-02	4055	8.67E-01	Up	0.17	13043	1.44	0.01
rs7239282	374864	C18orf34	18	28855794	3.59E-02	4056	2.72E-01	Down	1.10	8974	1.44	0.06
rs4982700	26020	LRP10	14	22424907	3.60E-02	4057	2.51E-05	Down	4.21	2131	1.44	0.46
rs4982700	161253	REM2	14	22424907	3.60E-02	4058	2.32E-02	Down	2.27	5209	1.44	0.16
rs10895226	57562	KIAA1377	11	101311849	3.60E-02	4059	6.44E-02	Down	1.85	6334	1.44	0.12
rs1533014	5019	OXCT1	5	41885356	3.60E-02	4060	2.30E-01	Down	1.20	8568	1.44	0.06
rs11166456	8556	CDC14A	1	100637654	3.60E-02	4061	-3.18E-13	Up	7.29	510	1.44	#NUM!
rs1077918	54567	DLL4	15	38991028	3.61E-02	4062	1.92E-01	Down	1.30	8175	1.44	0.07
rs2766113	26278	SACS	13	22800581	3.61E-02	4063	1.40E-02	Down	2.46	4756	1.44	0.19
rs670843	319101	KRT73	12	51311658	3.62E-02	4064	1.68E-01	Down	1.38	7895	1.44	0.08
rs10950961	56164	STK31	7	23596384	3.62E-02	4065	1.99E-01	Up	1.28	8245	1.44	0.07
rs12283125	80071	CCDC15	11	124319971	3.62E-02	4066	5.09E-05	Up	4.05	2293	1.44	0.43
rs12283125	220296	HEPACAM	11	124319971	3.62E-02	4067	4.95E-01	Up	0.68	10679	1.44	0.03
rs3889228	8140	SLC7A5	16	86426028	3.62E-02	4068	8.54E-01	Up	0.18	12980	1.44	0.01
rs3734533	8345	HIST1H2BH	6	26348603	3.62E-02	4069	1.80E-04	Up	3.75	2592	1.44	0.37
rs3734533	8361	HIST1H4F	6	26348603	3.62E-02	4070	1.54E-01	Up	1.43	7735	1.44	0.08
rs3734533	8968	HIST1H3F	6	26348603	3.62E-02	4071	3.64E-01	Up	0.91	9711	1.44	0.04
rs3734533	3007	HIST1H1D	6	26348603	3.62E-02	4072	4.28E-01	Up	0.79	10178	1.44	0.04
rs3734533	8369	HIST1H4G	6	26348603	3.62E-02	4073	7.24E-01	Down	0.35	12224	1.44	0.01
rs815802	805	CALM2	2	47303700	3.63E-02	4074	3.35E-05	Down	4.15	2191	1.44	0.45
rs815802	285051	C2orf61	2	47303700	3.63E-02	4075	9.47E-01	Down	0.07	13534	1.44	0.00
rs17098991	552	AVPR1A	12	61845506	3.64E-02	4076	8.62E-03	Up	2.63	4397	1.44	0.21
rs2272572	2783	GNB2	7	99930539	3.64E-02	4077	3.99E-03	Up	2.88	3932	1.44	0.24
rs1860985	5578	PRKCA	17	62076618	3.64E-02	4078	6.09E-17	Down	8.36	314	1.44	1.62
rs2302190	9110	MTMR4	17	53939507	3.65E-02	4079	2.25E-01	Down	1.21	8513	1.44	0.06
rs6040155	9491	PSMF1	20	1079119	3.65E-02	4080	5.79E-01	Down	0.56	11301	1.44	0.02
rs1242502	55090	MED9	17	17322489	3.65E-02	4081	8.56E-03	Down	2.63	4386	1.44	0.21
rs1242502	81857	MED25	17	17322489	3.65E-02	4082	7.15E-02	Up	1.80	6459	1.44	0.11
rs1209334	51332	SPTBN5	15	39977361	3.65E-02	4083	8.27E-02	Down	1.74	6679	1.44	0.11
rs10217363	10555	AGPAT2	9	136846454	3.65E-02	4084	2.97E-01	Up	1.04	9178	1.44	0.05
rs10217363	51162	EGFL7	9	136846454	3.65E-02	4085	3.87E-01	Down	0.86	9896	1.44	0.04
rs6570057	27115	PDE7B	6	136272078	3.65E-02	4086	1.74E-01	Down	1.36	7956	1.44	0.08
rs4693608	10855	HPSE	4	84598536	3.65E-02	4087	5.90E-01	Up	0.54	11373	1.44	0.02
rs3911998	2962	GTF2F1	19	6360117	3.66E-02	4088	3.30E-02	Up	2.13	5563	1.44	0.15
rs3826706	5050	PAFAH1B3	19	47485430	3.66E-02	4089	3.40E-07	Up	5.10	1433	1.44	0.65
rs3826706	23152	CIC	19	47485430	3.66E-02	4090	5.64E-06	Down	4.54	1838	1.44	0.52
rs17407218	2241	FER	5	108347850	3.66E-02	4091	3.51E-01	Down	0.93	9601	1.44	0.05
rs10514112	201456	FBXO15	18	69957348	3.66E-02	4092	1.54E-02	Up	2.42	4833	1.44	0.18
rs10514112	29090	C18orf55	18	69957348	3.66E-02	4093	4.37E-02	Down	2.02	5843	1.44	0.14
rs2071368	972	CD74	5	149772984	3.66E-02	4094	1.49E-01	Up	1.44	7666	1.44	0.08
rs1862610	3672	ITGA1	5	52163637	3.66E-02	4095	6.00E-11	Down	6.54	727	1.44	1.02
rs2240466	8326	FZD9	7	72300920	3.67E-02	4096	3.30E-01	Down	0.97	9449	1.44	0.05
rs2240466	9031	BAZ1B	7	72300920	3.67E-02	4097	4.35E-01	Down	0.78	10233	1.44	0.04
rs4425183	85359	DGCR6L	22	18669974	3.67E-02	4098	1.70E-01	Down	1.37	7915	1.44	0.08
rs2270256	51172	NAGPA	16	5009356	3.67E-02	4099	1.44E-05	Up	4.34	2029	1.44	0.48
rs11670086	4849	CNOT3	19	59319437	3.67E-02	4100	7.60E-04	Up	3.37	3114	1.44	0.31
rs11670086	29844	TFPT	19	59319437	3.67E-02	4101	7.39E-02	Up	1.79	6504	1.44	0.11
rs11670086	26121	PRPF31	19	59319437	3.67E-02	4102	4.40E-01	Down	0.77	10267	1.44	0.04
rs901687	23158	TBC1D9	4	141996677	3.67E-02	4103	2.39E-02	Up	2.26	5243	1.43	0.16
rs6546670	84693	MCEE	2	71238801	3.68E-02	4104	5.45E-01	Up	0.61	11047	1.43	0.03
rs5745331	4438	MSH4	1	75985334	3.68E-02	4105	6.06E-01	Up	0.52	11471	1.43	0.02
rs2738906	5359	PLSCR1	3	147743733	3.68E-02	4106	4.24E-07	Down	5.06	1459	1.43	0.64
rs6964434	6424	SFRP4	7	37736058	3.68E-02	4107	2.06E-08	Up	5.61	1124	1.43	0.77
rs6964434	54749	EPDR1	7	37736058	3.68E-02	4108	3.41E-04	Down	3.58	2824	1.43	0.35
rs6688148	59349	KLHL12	1	199638301	3.68E-02	4109	6.49E-01	Up	0.46	11767	1.43	0.02
rs12586368	123041	SLC24A4	14	91884210	3.68E-02	4110	2.26E-01	Down	1.21	8522	1.43	0.06
rs679350	731	C8A	1	57076833	3.68E-02	4111	2.21E-03	Up	3.06	3598	1.43	0.27
rs856133	149628	PYHIN1	1	155762004	3.69E-02	4112	5.53E-04	Down	3.45	2998	1.43	0.33
rs6937	5565	PRKAB2	1	143851738	3.69E-02	4113	2.82E-03	Down	2.99	3753	1.43	0.25
rs11878803	59348	ZNF350	19	57193133	3.69E-02	4114	4.69E-16	Up	8.12	347	1.43	1.53
rs11878803	284370	ZNF615	19	57193133	3.69E-02	4115	3.19E-06	Up	4.66	1751	1.43	0.55
rs12466829	81615	TMEM163	2	135161844	3.69E-02	4116	9.67E-01	Down	0.04	13677	1.43	0.00
rs133203	91355	LRP5L	22	24082340	3.69E-02	4117	4.14E-01	Up	0.82	10079	1.43	0.04
rs11079737	7473	WNT3	17	42243374	3.69E-02	4118	3.94E-02	Down	2.06	5734	1.43	0.14
rs2453626	26166	RGS22	8	101207073	3.70E-02	4119	1.55E-02	Down	2.42	4839	1.43	0.18
rs2453626	286151	FBXO43	8	101207073	3.70E-02	4120	6.59E-01	Up	0.44	11826	1.43	0.02
rs11873080	22878	KIAA1012	18	27664066	3.70E-02	4121	4.78E-01	Up	0.71	10560	1.43	0.03
rs17126072	80821	DDHD1	14	52577434	3.70E-02	4122	6.09E-02	Up	1.87	6253	1.43	0.12
rs17361679	27245	AHDC1	1	27543178	3.70E-02	4123	1.41E-07	Down	5.26	1338	1.43	0.69
rs7280100	7033	TFF3	21	42615436	3.70E-02	4124	1.50E-14	Up	7.69	423	1.43	1.38
rs10076102	55100	WDR70	5	37603608	3.71E-02	4125	5.15E-05	Up	4.05	2296	1.43	0.43
rs11858243	9899	SV2B	15	89506341	3.71E-02	4126	1.76E-04	Down	3.75	2587	1.43	0.38

rs2179932	29966	STRN3	14	30503224	3.71E-02	4127	8.30E-01	Up	0.21	12854	1.43	0.01
rs4448423	139170	WDR40B		125425903	3.71E-02	4128	5.58E-01	Down	0.59	11148	1.43	0.03
rs6509916	51206	GP6	19	60254214	3.71E-02	4129	6.46E-01	Down	0.46	11753	1.43	0.02
rs2816341	8521	GCM1	6	53110468	3.71E-02	4130	8.74E-01	Up	0.16	13085	1.43	0.01
rs247389	23417	MLYCD	16	82484220	3.71E-02	4131	1.57E-01	Down	1.41	7774	1.43	0.08
rs2285996	6677	SPAM1	7	123188157	3.71E-02	4132	1.39E-01	Down	1.48	7566	1.43	0.09
rs2360610	6138	RPL15	3	23932933	3.71E-02	4133	3.62E-07	Up	5.09	1442	1.43	0.64
rs2360610	28512	NKIRAS1	3	23932933	3.71E-02	4134	1.83E-01	Down	1.33	8059	1.43	0.07
rs1564808	4482	MSRA	8	10000055	3.71E-02	4135	3.97E-01	Down	0.85	9964	1.43	0.04
rs7754586	5167	ENPP1	6	132254435	3.71E-02	4136	6.59E-03	Up	2.72	4221	1.43	0.22
rs9512673	2971	GTF3A	13	26879532	3.72E-02	4137	2.31E-03	Up	3.05	3633	1.43	0.26
rs11859163	4313	MMP2	16	54059877	3.72E-02	4138	3.99E-07	Down	5.07	1455	1.43	0.64
rs5742858	1603	DAD1	14	22105427	3.72E-02	4139	5.59E-01	Up	0.58	11155	1.43	0.03
rs933724	79726	WDR59	16	73464892	3.72E-02	4140	9.88E-03	Down	2.58	4498	1.43	0.20
rs7428779	6331	SCN5A	3	38621427	3.72E-02	4141	5.27E-01	Down	0.63	10917	1.43	0.03
rs10867647	320	APBA1	9	69336183	3.72E-02	4142	1.37E-02	Down	2.47	4735	1.43	0.19
rs10205923	3667	IRS1	2	227440062	3.72E-02	4143	3.00E-18	Down	8.71	260	1.43	1.75
rs6780507	201562	PTPLB	3	124762791	3.73E-02	4144	7.06E-10	Up	6.17	871	1.43	0.92
rs1540528	5510	PPP1R7	2	241838676	3.73E-02	4145	1.80E-05	Down	4.29	2072	1.43	0.47
rs4832045	7844	RNF103	2	86753832	3.73E-02	4146	8.11E-03	Up	2.65	4351	1.43	0.21
rs2279900	7251	TSG101	11	18459735	3.73E-02	4147	2.30E-05	Up	4.23	2113	1.43	0.46
rs2279900	160287	LDHAL6A	11	18459735	3.73E-02	4148	6.64E-01	Down	0.44	11844	1.43	0.02
rs10849582	5893	RAD52	12	883437	3.73E-02	4149	1.28E-01	Down	1.52	7427	1.43	0.09
rs713467	57188	ADAMTSL3	15	82437477	3.73E-02	4150	2.17E-04	Down	3.70	2658	1.43	0.37
rs16851254	92370	ACPL2	3	142462109	3.73E-02	4151	1.83E-01	Up	1.33	8055	1.43	0.07
rs2854439	6652	SORD	15	43146138	3.74E-02	4152	4.64E-01	Down	0.73	10456	1.43	0.03
rs4729602	51412	ACTL6B	7	99905242	3.74E-02	4153	3.70E-01	Up	0.90	9761	1.43	0.04
rs16968623	9051	PSTPIP1	15	75099096	3.74E-02	4154	2.02E-01	Down	1.27	8280	1.43	0.07
rs7017753	55353	LAPTM4B	8	98928613	3.74E-02	4155	1.46E-14	Down	7.69	419	1.43	1.38
rs2307281	56344	CABP5	19	53250098	3.74E-02	4156	9.05E-02	Down	1.69	6837	1.43	0.10
rs1863641	3248	HPGD	4	175808450	3.74E-02	4157	1.36E-07	Up	5.27	1330	1.43	0.69
rs4347009	6713	SQLE	8	126082484	3.74E-02	4158	5.83E-02	Down	1.89	6197	1.43	0.12
rs10981835	5998	RGS3	9	113428852	3.75E-02	4159	3.95E-01	Up	0.85	9948	1.43	0.04
rs7555931	6195	RPS6KA1	1	26542894	3.75E-02	4160	1.13E-13	Up	7.43	473	1.43	1.29
rs492315	6582	SLC22A2	6	160663105	3.75E-02	4161	7.56E-01	Up	0.31	12415	1.43	0.01
rs2286916	25926	NOL11	17	63166025	3.75E-02	4162	1.83E-01	Up	1.33	8060	1.43	0.07
rs17170220	27241	BBS9	7	33226384	3.75E-02	4163	3.39E-02	Down	2.12	5589	1.43	0.15
rs2722901	5368	PNOC	8	28240467	3.75E-02	4164	4.63E-01	Down	0.73	10451	1.43	0.03
rs740804	27092	CACNG4	17	62373156	3.75E-02	4165	2.97E-02	Down	2.17	5449	1.43	0.15
rs10514260	1404	HAPLN1	5	83007677	3.75E-02	4166	6.35E-01	Down	0.48	11672	1.43	0.02
rs11864516	84264	HAGHL	16	725280	3.76E-02	4167	2.71E-03	Up	3.00	3730	1.43	0.26
rs11864516	124093	CCDC78	16	725280	3.76E-02	4168	4.40E-01	Up	0.77	10266	1.43	0.04
rs11864516	64428	NARFL	16	725280	3.76E-02	4169	8.29E-01	Up	0.22	12851	1.43	0.01
rs17157266	7356	SCGB1A1	11	61956393	3.76E-02	4170	9.58E-09	Down	5.74	1052	1.42	0.80
rs17157266	79026	AHNAK	11	61956393	3.76E-02	4171	2.30E-02	Down	2.27	5196	1.42	0.16
rs2063979	6358	CCL14	17	31327679	3.76E-02	4172	9.24E-02	Down	1.68	6870	1.42	0.10
rs2063979	6360	CCL16	17	31327679	3.76E-02	4173	4.70E-01	Up	0.72	10499	1.42	0.03
rs450320	948	CD36	4	187940305	3.76E-02	4174	8.89E-01	Up	0.14	13183	1.42	0.01
rs2839357	6285	S100B	21	46847315	3.76E-02	4175	1.79E-01	Down	1.35	8005	1.42	0.07
rs1420215	1066	CES1	16	54434310	3.76E-02	4176	5.79E-08	Down	5.43	1237	1.42	0.72
rs2072382	6597	SMARCA4	19	11013650	3.76E-02	4177	6.61E-11	Up	6.61	699	1.42	1.02
rs8079518	79003	MIS12	17	5340901	3.76E-02	4178	3.72E-02	Down	2.08	5683	1.42	0.14
rs8079518	51009	DERL2	17	5340901	3.76E-02	4179	5.06E-01	Up	0.67	10754	1.42	0.03
rs10982990	58483	C9orf27	9	115738270	3.76E-02	4180	4.21E-01	Up	0.81	10128	1.42	0.04
rs11064881	11113	CIT	12	118609645	3.77E-02	4181	3.59E-01	Up	0.92	9673	1.42	0.04
rs1044471	79602	ADIPOR2	12	1767217	3.77E-02	4182	5.24E-01	Up	0.64	10897	1.42	0.03
rs4984391	55784	MCTP2	15	92743820	3.77E-02	4183	5.77E-02	Up	1.90	6179	1.42	0.12
rs4927101	23648	SSBP3	1	54595024	3.77E-02	4184	8.17E-01	Up	0.23	12773	1.42	0.01
rs903903	11079	RER1	1	2351590	3.77E-02	4185	1.29E-01	Up	1.52	7443	1.42	0.09
rs903903	79906	MORN1	1	2351590	3.77E-02	4186	5.99E-01	Down	0.53	11421	1.42	0.02
rs7857325	256691	MAMDC2	9	69966645	3.77E-02	4187	9.48E-04	Up	3.31	3197	1.42	0.30
rs11862609	57338	JPH3	16	86212691	3.77E-02	4188	4.14E-02	Down	2.04	5786	1.42	0.14
rs185289	147	ADRA1B	5	159268430	3.77E-02	4189	1.38E-01	Up	1.48	7546	1.42	0.09
rs6764427	28999	KLF15	3	127552824	3.77E-02	4190	2.18E-03	Down	3.07	3592	1.42	0.27
rs7883434	53344	CHIC1		72562950	3.78E-02	4191	9.57E-01	Down	0.05	13595	1.42	0.00
rs11705143	79680	C22orf29	22	18211259	3.78E-02	4192	8.60E-01	Up	0.18	13010	1.42	0.01
rs11705143	54584	GNB1L	22	18211259	3.78E-02	4193	9.74E-01	Down	0.03	13725	1.42	0.00
rs17415295	23114	NFASC	1	201681483	3.78E-02	4194	7.55E-06	Down	4.48	1888	1.42	0.51
rs2038636	27185	DISC1	1	228361984	3.78E-02	4195	4.28E-04	Up	3.52	2903	1.42	0.34
rs2977283	29943	PADI1	1	17287255	3.78E-02	4196	4.30E-01	Down	0.79	10191	1.42	0.04
rs6993679	25960	GPR124	8	37780229	3.78E-02	4197	1.59E-05	Down	4.32	2049	1.42	0.48
rs11023175	22800	RRAS2	11	14259109	3.78E-02	4198	1.72E-01	Down	1.37	7939	1.42	0.08
rs6025446	655	BMP7	20	55220218	3.78E-02	4199	3.26E-01	Down	0.98	9412	1.42	0.05
rs7031791	4702	NDUFA8	9	121996121	3.78E-02	4200	3.88E-02	Up	2.07	5724	1.42	0.14
rs17165035	84466	MEGF10	5	126729089	3.79E-02	4201	6.38E-01	Down	0.47	11693	1.42	0.02
rs12882423	23503	ZFYVE26	14	67276231	3.79E-02	4202	9.78E-03	Down	2.58	4493	1.42	0.20
rs12882423	145226	RDH12	14	67276231	3.79E-02	4203	8.13E-01	Up	0.24	12743	1.42	0.01
rs3846911	10048	RANBP9	6	13752046	3.79E-02	4204	1.81E-01	Up	1.34	8034	1.42	0.07
rs13205856	4199	ME1	6	84144378	3.79E-02	4205	6.62E-12	Down	6.87	630	1.42	1.12
rs13205856	57717	PCDHB16	6	84144378	3.79E-02	4206	3.41E-03	Up	2.93	3843	1.42	0.25
rs1637995	1820	ARID3A	19	911445	3.79E-02	4207	1.43E-02	Down	2.45	4770	1.42	0.18

rs7237461	8170	SLC14A2	18	41474873	3.79E-02	4208	8.33E-01	Up	0.21	12869	1.42	0.01
rs11807526	10500	SEMA6C	1	147929070	3.79E-02	4209	1.68E-02	Down	2.39	4927	1.42	0.18
rs11807526	79626	TNFAIP8L2	1	147929070	3.79E-02	4210	2.73E-01	Down	1.10	8977	1.42	0.06
rs2581830	91869	RFT1	3	53109138	3.80E-02	4211	6.43E-01	Up	0.46	11726	1.42	0.02
rs4377140	9780	FAM38A	16	87303649	3.80E-02	4212	5.74E-07	Up	5.00	1499	1.42	0.62
rs4377140	115992	RNF166	16	87303649	3.80E-02	4213	9.73E-02	Up	1.66	6947	1.42	0.10
rs660339	7351	UCP2	11	73366752	3.80E-02	4214	4.53E-03	Down	2.84	3998	1.42	0.23
rs782728	115286	SLC25A26	3	66408992	3.80E-02	4215	1.70E-04	Down	3.76	2575	1.42	0.38
rs1557770	5358	PLS3		114618155	3.80E-02	4216	2.95E-10	Down	6.30	809	1.42	0.95
rs2464974	1635	DCTD	4	184188330	3.80E-02	4217	4.03E-03	Up	2.88	3936	1.42	0.24
rs17661298	220134	C18orf24	18	46165996	3.81E-02	4218	5.82E-02	Down	1.89	6195	1.42	0.12
rs414000	4706	NDUFAB1	16	23524715	3.81E-02	4219	6.71E-02	Up	1.83	6377	1.42	0.12
rs6510064	57343	ZNF304	19	62547742	3.81E-02	4220	1.13E-01	Up	1.58	7195	1.42	0.09
rs6510064	284306	ZNF547	19	62547742	3.81E-02	4221	1.32E-01	Up	1.51	7470	1.42	0.09
rs7191281	10664	CTCF	16	66212634	3.81E-02	4222	7.47E-01	Up	0.32	12352	1.42	0.01
rs247445	4166	CHST6	16	74078531	3.81E-02	4223	8.78E-02	Down	1.71	6769	1.42	0.11
rs7841111	93594	WDR67	8	124189470	3.81E-02	4224	6.03E-03	Up	2.75	4173	1.42	0.22
rs2285905	1265	CNN2	19	959504	3.82E-02	4225	7.40E-03	Down	2.68	4293	1.42	0.21
rs2285905	91304	C19orf6	19	959504	3.82E-02	4226	8.76E-01	Up	0.16	13095	1.42	0.01
rs10197283	246243	RNASEH1	2	3079898	3.82E-02	4227	9.86E-03	Down	2.58	4496	1.42	0.20
rs1034109	353135	LCE1E	1	149572751	3.82E-02	4228	9.62E-04	Up	3.30	3205	1.42	0.30
rs3759084	4617	MYF5	12	79612624	3.82E-02	4229	5.26E-01	Up	0.63	10912	1.42	0.03
rs3759084	4618	MYF6	12	79612624	3.82E-02	4230	8.90E-01	Up	0.14	13188	1.42	0.01
rs416162	65249	ZSWIM4	19	13755296	3.83E-02	4231	7.59E-01	Down	0.31	12433	1.42	0.01
rs11965835	116369	SLC26A8	6	36066849	3.83E-02	4232	2.34E-01	Down	1.19	8598	1.42	0.06
rs10900551	55224	ETNK2	1	200829516	3.83E-02	4233	1.62E-04	Down	3.77	2569	1.42	0.38
rs1984749	55720	TSR1	17	2196654	3.83E-02	4234	9.35E-01	Up	0.08	13461	1.42	0.00
rs727852	29760	BLNK	10	97980573	3.83E-02	4235	1.40E-05	Up	4.34	2026	1.42	0.49
rs2697144	6529	SLC6A1	3	11026099	3.84E-02	4236	5.35E-01	Up	0.62	10974	1.42	0.03
rs11119309	57172	CAMK1G	1	206131177	3.84E-02	4237	2.99E-04	Down	3.62	2769	1.42	0.35
rs4839385	10286	BCAS2	1	114833503	3.84E-02	4238	2.93E-01	Down	1.05	9138	1.42	0.05
rs9844476	3273	HRG	3	187882458	3.84E-02	4239	7.23E-01	Up	0.35	12220	1.42	0.01
rs17257176	64793	CCDC21	1	26262375	3.84E-02	4240	5.00E-04	Up	3.48	2961	1.42	0.33
rs12579591	57658	CALCOCO1	12	52382855	3.84E-02	4241	1.34E-02	Down	2.47	4717	1.42	0.19
rs3775110	10417	SPON2	4	1151852	3.84E-02	4242	3.14E-03	Up	2.95	3801	1.42	0.25
rs13369586	7776	ZNF236	18	72792520	3.85E-02	4243	8.10E-03	Up	2.65	4350	1.42	0.21
rs7151036	5926	ARID4A	14	57892786	3.85E-02	4244	6.27E-01	Down	0.49	11626	1.41	0.02
rs11578799	1031	CDKN2C	1	51136577	3.86E-02	4245	5.46E-01	Down	0.60	11057	1.41	0.03
rs7543044	6421	SFPQ	1	35343404	3.86E-02	4246	1.75E-13	Up	7.37	487	1.41	1.28
rs6495309	1136	CHRNA3	15	76702300	3.86E-02	4247	6.70E-02	Down	1.83	6374	1.41	0.12
rs12972385	126328	NDUFA11	19	5842052	3.86E-02	4248	6.10E-04	Up	3.43	3034	1.41	0.32
rs8190996	2936	GSR	8	30673548	3.86E-02	4249	1.53E-02	Down	2.42	4828	1.41	0.18
rs4468946	57408	LRTM1	3	54923909	3.86E-02	4250	5.33E-01	Down	0.62	10963	1.41	0.03
rs10435425	5764	PTN	7	136398055	3.86E-02	4251	3.22E-20	Down	9.21	191	1.41	1.95
rs7850466	8022	LHX3	9	136315662	3.87E-02	4252	9.42E-01	Up	0.07	13503	1.41	0.00
rs5925006	79057	PRRG3		150530916	3.87E-02	4253	8.62E-01	Down	0.17	13021	1.41	0.01
rs11227805	54998	AURKAIP1	11	67003333	3.87E-02	4254	3.98E-02	Up	2.06	5746	1.41	0.14
rs11227805	9049	AIP	11	67003333	3.87E-02	4255	4.07E-02	Down	2.05	5763	1.41	0.14
rs11227805	80194	TMEM134	11	67003333	3.87E-02	4256	1.07E-01	Up	1.61	7099	1.41	0.10
rs6664817	3782	KCNN3	1	151497179	3.87E-02	4257	1.98E-04	Down	3.72	2625	1.41	0.37
rs3818511	3632	INPP5A	10	134309378	3.87E-02	4258	3.27E-07	Down	5.11	1428	1.41	0.65
rs7260544	388555	IGFL3	19	51313304	3.87E-02	4259	2.48E-01	Up	1.16	8741	1.41	0.06
rs497704	134957	STXBP5	6	147622390	3.88E-02	4260	1.91E-01	Up	1.31	8162	1.41	0.07
rs17166268	134549	SHROOM1	5	132208889	3.88E-02	4261	1.41E-02	Down	2.45	4759	1.41	0.19
rs7165146	80208	SPG11	15	42738466	3.88E-02	4262	2.30E-03	Up	3.05	3628	1.41	0.26
rs10111737	55529	TMEM55A	8	92107480	3.88E-02	4263	1.05E-02	Down	2.56	4552	1.41	0.20
rs921336	5553	PRG2	18	72861460	3.88E-02	4264	1.27E-05	Up	4.36	2007	1.41	0.49
rs921336	79948	PRG2	18	72861460	3.88E-02	4265	1.27E-05	Up	4.36	2008	1.41	0.49
rs921336	4155	MBP	18	72861460	3.88E-02	4266	2.97E-02	Up	2.17	5450	1.41	0.15
rs9421580	54537	FAM35A	10	88825234	3.89E-02	4267	1.42E-01	Up	1.47	7596	1.41	0.08
rs4681161	1359	CPA3	3	150091743	3.89E-02	4268	1.51E-03	Down	3.17	3385	1.41	0.28
rs4681161	51200	CPA4	3	150091743	3.89E-02	4269	3.64E-01	Down	0.91	9713	1.41	0.04
rs1746469	9465	AKAP7	6	131544464	3.89E-02	4270	8.46E-03	Down	2.63	4381	1.41	0.21
rs1835815	64240	ABCG5	2	43942300	3.89E-02	4271	8.74E-02	Up	1.71	6763	1.41	0.11
rs6698390	55083	KIF26B	1	242015828	3.89E-02	4272	2.01E-01	Up	1.28	8269	1.41	0.07
rs13239658	95681	TSGA14	7	129641617	3.89E-02	4273	9.12E-02	Up	1.69	6850	1.41	0.10
rs2287692	112398	EGLN2	19	45981596	3.90E-02	4274	2.65E-01	Down	1.11	8908	1.41	0.06
rs1701476	26019	UPF2	10	12103825	3.90E-02	4275	7.05E-05	Down	3.97	2362	1.41	0.42
rs4816144	24141	C20orf103	20	9441480	3.90E-02	4276	2.49E-03	Up	3.02	3684	1.41	0.26
rs787858	5567	PRKACB	1	84417502	3.90E-02	4277	4.64E-07	Up	5.04	1471	1.41	0.63
rs3771362	5334	PLCL1	2	198788337	3.90E-02	4278	1.49E-10	Down	6.41	779	1.41	0.98
rs7200888	267	AMFR	16	55014083	3.90E-02	4279	3.08E-04	Down	3.61	2785	1.41	0.35
rs10923228	79679	VTCN1	1	117462728	3.90E-02	4280	2.60E-04	Down	3.65	2727	1.41	0.36
rs32333	9315	C5orf13	5	111122362	3.90E-02	4281	1.06E-01	Up	1.62	7092	1.41	0.10
rs279904	58524	DMRT3	9	950479	3.90E-02	4282	7.56E-01	Down	0.31	12413	1.41	0.01
rs533887	9723	SEMA3E	7	82942854	3.91E-02	4283	2.04E-01	Down	1.27	8294	1.41	0.07
rs11948188	84340	GFM2	5	74093839	3.91E-02	4284	2.05E-02	Up	2.32	5093	1.41	0.17
rs2425266	57446	NDRG3	20	34794053	3.91E-02	4285	2.15E-01	Down	1.24	8409	1.41	0.07
rs12203580	84624	FNDC1	6	159597439	3.91E-02	4286	6.64E-01	Down	0.43	11846	1.41	0.02
rs1392912	8997	KALRN	3	125849352	3.91E-02	4287	9.44E-03	Up	2.60	4466	1.41	0.20
rs1392912	5892	RAD51L3	3	125849352	3.91E-02	4288	8.08E-01	Up	0.24	12710	1.41	0.01

rs1062708	10856	RUVBL2	19	54205085	3.92E-02	4289	1.13E-04	Up	3.86	2484	1.41	0.39
rs1062708	3972	LHB	19	54205085	3.92E-02	4290	3.55E-01	Up	0.93	9634	1.41	0.05
rs1062708	2997	GYS1	19	54205085	3.92E-02	4291	3.92E-01	Up	0.86	9927	1.41	0.04
rs4130393	8754	ADAM9	8	39060091	3.92E-02	4292	3.80E-03	Up	2.89	3903	1.41	0.24
rs7126870	6786	STIM1	11	3846635	3.92E-02	4293	1.91E-01	Down	1.31	8163	1.41	0.07
rs1051421	182	JAG1	20	10568275	3.92E-02	4294	5.78E-04	Up	3.44	3013	1.41	0.32
rs2279375	80125	CCDC33	15	72378749	3.92E-02	4295	3.34E-01	Up	0.97	9476	1.41	0.05
rs1015896	26297	SERGEF	11	17839962	3.92E-02	4296	2.75E-02	Up	2.20	5378	1.41	0.16
rs4753840	143888	KDELC2	11	107843550	3.92E-02	4297	9.15E-05	Down	3.91	2429	1.41	0.40
rs4753840	160140	C11orf65	11	107843550	3.92E-02	4298	7.45E-01	Down	0.33	12345	1.41	0.01
rs10864943	57628	DPP10	2	116152604	3.93E-02	4299	2.00E-03	Down	3.09	3532	1.41	0.27
rs1156856	54852	PAQR5	15	67405233	3.93E-02	4300	2.05E-01	Down	1.27	8310	1.41	0.07
rs1014095	8454	CUL1	7	147901508	3.93E-02	4301	6.50E-11	Down	6.53	731	1.41	1.02
rs6510123	79673	ZNF329	19	63323006	3.93E-02	4302	4.42E-01	Down	0.77	10284	1.41	0.04
rs10734468	56547	MMP26	11	4962794	3.94E-02	4303	4.30E-02	Up	2.02	5829	1.40	0.14
rs7204900	9289	GPR56	16	56203008	3.94E-02	4304	9.46E-01	Up	0.07	13529	1.40	0.00
rs2247810	3753	KCNE1	21	34761269	3.94E-02	4305	7.34E-01	Up	0.34	12277	1.40	0.01
rs7232558	171586	ABHD3	18	17487429	3.94E-02	4306	5.70E-01	Up	0.57	11246	1.40	0.02
rs7904463	8031	NCOA4	10	51229475	3.94E-02	4307	4.83E-03	Down	2.82	4035	1.40	0.23
rs312456	255877	BCL6B	17	6879059	3.94E-02	4308	8.20E-03	Up	2.64	4358	1.40	0.21
rs4607021	3689	ITGB2	21	45146915	3.94E-02	4309	4.96E-01	Down	0.68	10684	1.40	0.03
rs17004936	51733	UPB1	22	23204728	3.94E-02	4310	9.40E-01	Down	0.07	13495	1.40	0.00
rs1579036	1456	CSNK1G3	5	122949002	3.95E-02	4311	1.05E-08	Up	5.72	1062	1.40	0.80
rs10515638	133522	PPARGC1B	5	149132724	3.95E-02	4312	6.30E-02	Down	1.86	6299	1.40	0.12
rs1925179	81578	COL21A1	6	56129171	3.95E-02	4313	2.06E-03	Down	3.08	3548	1.40	0.27
rs7896470	220963	SLC16A9	10	61168953	3.95E-02	4314	4.53E-02	Down	2.00	5883	1.40	0.13
rs9293744	9522	SCAMP1	5	77672779	3.95E-02	4315	2.62E-02	Up	2.22	5331	1.40	0.16
rs1402752	10168	ZNF197	3	44662304	3.95E-02	4316	4.83E-01	Down	0.70	10592	1.40	0.03
rs1402752	7584	ZNF35	3	44662304	3.95E-02	4317	7.73E-01	Down	0.29	12515	1.40	0.01
rs2042630	57484	RNF150	4	142332954	3.95E-02	4318	1.30E-02	Down	2.48	4692	1.40	0.19
rs10867783	7088	TLE1	9	81468366	3.95E-02	4319	1.89E-09	Up	6.01	936	1.40	0.87
rs3759985	7783	ZP2	16	21131281	3.95E-02	4320	2.46E-01	Up	1.16	8720	1.40	0.06
rs12478256	11250	GPR45	2	105309726	3.95E-02	4321	6.07E-01	Down	0.51	11479	1.40	0.02
rs5751592	9609	RAB36	22	21822228	3.95E-02	4322	1.89E-01	Down	1.31	8142	1.40	0.07
rs5751592	27156	RTDR1	22	21822228	3.95E-02	4323	4.44E-01	Down	0.77	10302	1.40	0.04
rs964785	9821	RB1CC1	8	53724445	3.96E-02	4324	1.94E-02	Up	2.34	5043	1.40	0.17
rs5944042	5165	PDK3		24228487	3.96E-02	4325	4.94E-01	Down	0.68	10675	1.40	0.03
rs1275689	57452	GALNTL1	14	68882032	3.96E-02	4326	1.30E-01	Down	1.51	7451	1.40	0.09
rs2543953	11274	USP18	22	17016699	3.96E-02	4327	1.12E-06	Up	4.87	1589	1.40	0.60
rs13261706	55246	CCDC25	8	27691536	3.96E-02	4328	5.23E-05	Down	4.05	2300	1.40	0.43
rs348389	8744	TNFSF9	19	6486637	3.96E-02	4329	3.86E-01	Up	0.87	9888	1.40	0.04
rs16970280	348158	ACSM2B	16	20459576	3.96E-02	4330	1.24E-01	Up	1.54	7354	1.40	0.09
rs4744342	84641	HIATL1	9	94284159	3.96E-02	4331	2.65E-01	Up	1.11	8909	1.40	0.06
rs3845862	51725	FBXO40	3	122814935	3.97E-02	4332	3.13E-01	Up	1.01	9295	1.40	0.05
rs17221319	22891	ZNF365	10	63876718	3.97E-02	4333	9.75E-01	Up	0.03	13729	1.40	0.00
rs2282402	83998	REG4	1	120056922	3.97E-02	4334	7.31E-03	Down	2.68	4285	1.40	0.21
rs7632209	25937	WWTR1	3	150733850	3.97E-02	4335	4.13E-17	Down	8.41	303	1.40	1.64
rs7632209	6901	TAZ	3	150733850	3.97E-02	4336	1.90E-04	Up	3.73	2608	1.40	0.37
rs12691	1050	CEBPA	19	38482967	3.97E-02	4337	5.48E-04	Up	3.46	2993	1.40	0.33
rs6685856	10163	WASF2	1	27484624	3.97E-02	4338	8.81E-01	Up	0.15	13132	1.40	0.01
rs6820078	140458	ASB5	4	177570294	3.98E-02	4339	9.29E-01	Down	0.09	13426	1.40	0.00
rs4980200	2849	GPR26	10	125447612	3.98E-02	4340	6.02E-01	Down	0.52	11440	1.40	0.02
rs6012846	387521	TMEM189	20	48170921	3.98E-02	4341	1.84E-01	Down	1.33	8070	1.40	0.07
rs3755876	658	BMPR1B	4	96419962	3.98E-02	4342	4.65E-06	Up	4.58	1802	1.40	0.53
rs2412664	132954	PDCL2	4	56298315	3.98E-02	4343	3.11E-03	Up	2.96	3796	1.40	0.25
rs2412664	10874	NMU	4	56298315	3.98E-02	4344	1.12E-01	Up	1.59	7166	1.40	0.10
rs10503869	11030	RBPMS	8	30494350	3.98E-02	4345	1.53E-43	Down	13.84	13	1.40	4.28
rs4075131	241	ALOX5AP	13	30208919	3.98E-02	4346	2.95E-06	Down	4.67	1733	1.40	0.55
rs4362936	11005	SPINK5	5	147498582	3.99E-02	4347	2.11E-02	Down	2.31	5127	1.40	0.17
rs613575	7541	ZFP161	18	5293009	3.99E-02	4348	4.71E-01	Down	0.72	10506	1.40	0.03
rs7068856	84896	ATAD1	10	89532330	3.99E-02	4349	1.03E-07	Down	5.32	1298	1.40	0.70
rs10975615	115426	UHRF2	9	6500453	3.99E-02	4350	2.17E-03	Down	3.07	3587	1.40	0.27
rs3731459	1111	CHEK1	11	125021274	4.00E-02	4351	1.39E-03	Up	3.20	3345	1.40	0.29
rs2097390	7533	YWHAH	22	30650792	4.00E-02	4352	1.01E-01	Down	1.64	7022	1.40	0.10
rs7216389	94103	ORMDL3	17	35323475	4.00E-02	4353	6.57E-03	Down	2.72	4217	1.40	0.22
rs2395911	5172	SLC26A4	7	106918064	4.01E-02	4354	3.29E-03	Up	2.94	3825	1.40	0.25
rs1417478	4660	PPP1R12B	1	199134439	4.01E-02	4355	1.18E-21	Down	9.56	168	1.40	2.09
rs6960004	27044	SND1	7	127327963	4.01E-02	4356	1.65E-20	Up	9.27	189	1.40	1.98
rs7501201	84937	ZNRF1	16	73684404	4.01E-02	4357	2.19E-02	Down	2.29	5156	1.40	0.17
rs6722763	8527	DGKD	2	234064210	4.01E-02	4358	1.62E-05	Up	4.31	2053	1.40	0.48
rs7811926	50833	TAS2R16	7	122221569	4.01E-02	4359	3.68E-01	Up	0.90	9744	1.40	0.04
rs962060	63974	NEUROD6	7	31168377	4.01E-02	4360	9.73E-02	Up	1.66	6948	1.40	0.10
rs9321496	4602	MYB	6	135587153	4.01E-02	4361	2.95E-03	Up	2.97	3770	1.40	0.25
rs11060740	11211	FZD10	12	129157453	4.01E-02	4362	1.20E-03	Down	3.24	3293	1.40	0.29
rs6518754	253143	C22orf30	22	30422329	4.01E-02	4363	6.91E-03	Up	2.70	4251	1.40	0.22
rs950802	58475	MS4A7	11	59909160	4.02E-02	4364	3.98E-05	Up	4.11	2229	1.40	0.44
rs950802	51338	MS4A4A	11	59909160	4.02E-02	4365	1.39E-01	Up	1.48	7559	1.40	0.09
rs4823124	64800	EFCAB6	22	42340192	4.02E-02	4366	1.58E-01	Down	1.41	7778	1.40	0.08
rs4964759	54434	SSH1	12	107706277	4.02E-02	4367	3.05E-04	Down	3.61	2781	1.40	0.35
rs7349107	5792	PTPRF	1	43712004	4.02E-02	4368	4.52E-03	Up	2.84	3997	1.40	0.23
rs742460	8938	BAIAP3	16	1345538	4.02E-02	4369	4.00E-08	Up	5.49	1203	1.40	0.74

rs742460	115939	C16orf42	16	1345538	4.02E-02	4370	8.00E-01	Down	0.25	12675	1.40	0.01
rs16852052	23013	SPEN	1	15946808	4.02E-02	4371	1.87E-01	Down	1.32	8116	1.40	0.07
rs3862476	23475	QPRT	16	29594861	4.03E-02	4372	3.61E-01	Down	0.91	9688	1.40	0.04
rs5006218	6183	MRPS12	6	133167913	4.03E-02	4373	4.43E-11	Up	6.58	707	1.40	1.04
rs5006218	6206	RPS12	6	133167913	4.03E-02	4374	3.00E-01	Up	1.04	9188	1.40	0.05
rs2275387	9247	GCM2	6	10990161	4.03E-02	4375	7.82E-02	Up	1.76	6598	1.39	0.11
rs183965	6555	SLC10A2	13	102502105	4.03E-02	4376	7.97E-01	Down	0.26	12655	1.39	0.01
rs2501857	55509	BATF3	1	209245727	4.04E-02	4377	5.59E-01	Up	0.58	11159	1.39	0.03
rs3184504	10019	SH2B3	12	110347328	4.04E-02	4378	4.75E-01	Down	0.71	10539	1.39	0.03
rs11064024	7450	VWF	12	6072310	4.04E-02	4379	2.24E-02	Down	2.28	5179	1.39	0.16
rs6084217	57761	TRIB3	20	295908	4.04E-02	4380	2.24E-03	Up	3.06	3610	1.39	0.26
rs3798257	2569	GABRR1	6	89958060	4.04E-02	4381	3.06E-03	Up	2.96	3786	1.39	0.25
rs228042	54020	SLC37A1	21	42823373	4.05E-02	4382	2.10E-02	Up	2.31	5117	1.39	0.17
rs10492814	54957	TXNL4B	16	70684015	4.05E-02	4383	1.20E-01	Up	1.55	7296	1.39	0.09
rs10492814	9785	DHX38	16	70684015	4.05E-02	4384	4.06E-01	Down	0.83	10025	1.39	0.04
rs1018575	9751	SNPH	20	1246397	4.05E-02	4385	8.56E-03	Down	2.63	4387	1.39	0.21
rs41789	830	CAPZA2	7	116085942	4.05E-02	4386	2.76E-05	Down	4.19	2153	1.39	0.46
rs2384687	84446	BRSK1	19	60523000	4.06E-02	4387	9.83E-01	Down	0.02	13794	1.39	0.00
rs17184300	64129	TINAGL1	6	131947410	4.06E-02	4388	4.11E-02	Down	2.04	5777	1.39	0.14
rs17184300	383	ARG1	6	131947410	4.06E-02	4389	6.25E-01	Up	0.49	11616	1.39	0.02
rs10515610	81545	FBXO38	5	147733282	4.06E-02	4390	1.51E-03	Down	3.17	3386	1.39	0.28
rs6532013	1758	DMP1	4	88910593	4.06E-02	4391	5.03E-01	Up	0.67	10735	1.39	0.03
rs6532013	9988	DMTF1	4	88910593	4.06E-02	4392	7.76E-01	Down	0.28	12536	1.39	0.01
rs934839	55193	PBRM1	3	52658938	4.06E-02	4393	5.32E-01	Up	0.62	10955	1.39	0.03
rs11258248	55388	MCM10	10	13282385	4.06E-02	4394	1.87E-02	Up	2.35	5011	1.39	0.17
rs2269318	9578	CDC42BPB	14	102478372	4.06E-02	4395	7.65E-02	Down	1.77	6562	1.39	0.11
rs2364482	4055	LTBR	12	6372392	4.06E-02	4396	2.57E-01	Up	1.13	8827	1.39	0.06
rs3744626	28964	GIT1	17	24935683	4.07E-02	4397	6.83E-01	Down	0.41	11962	1.39	0.02
rs3744626	124930	ANKRD13B	17	24935683	4.07E-02	4398	6.91E-01	Up	0.40	12013	1.39	0.02
rs4889826	2548	GAA	17	75714784	4.07E-02	4399	1.58E-13	Up	7.43	476	1.39	1.28
rs1955716	254170	FBXO33	14	38969200	4.07E-02	4400	2.72E-02	Down	2.21	5370	1.39	0.16
rs2376397	54981	C9orf95	9	74920303	4.07E-02	4401	4.76E-02	Down	1.98	5936	1.39	0.13
rs6910541	23036	ZNF292	6	88026199	4.07E-02	4402	2.50E-05	Down	4.22	2130	1.39	0.46
rs15819	197257	LDHD	16	73701766	4.07E-02	4403	2.56E-01	Down	1.14	8817	1.39	0.06
rs584878	359	AQP2	12	48610834	4.08E-02	4404	8.49E-02	Down	1.72	6718	1.39	0.11
rs223875	55715	DOK4	16	56064416	4.08E-02	4405	2.36E-04	Down	3.68	2688	1.39	0.36
rs223875	5432	POLR2C	16	56064416	4.08E-02	4406	5.13E-01	Down	0.65	10801	1.39	0.03
rs7438718	4750	NEK1	4	170741965	4.08E-02	4407	7.33E-03	Up	2.68	4288	1.39	0.21
rs8081984	162517	FBXO39	17	6627290	4.08E-02	4408	2.37E-02	Up	2.26	5229	1.39	0.16
rs8081984	54739	XAF1	17	6627290	4.08E-02	4409	3.09E-01	Up	1.02	9256	1.39	0.05
rs7657186	7098	TLR3	4	187369188	4.08E-02	4410	5.54E-06	Down	4.54	1835	1.39	0.53
rs170365	57017	COQ9	16	56060861	4.08E-02	4411	3.03E-03	Down	2.96	3781	1.39	0.25
rs7693779	7006	TEC	4	48027080	4.08E-02	4412	1.17E-01	Up	1.57	7240	1.39	0.09
rs3825003	5870	RAB6A	11	73066760	4.08E-02	4413	3.59E-01	Up	0.92	9670	1.39	0.04
rs6439501	339855	KY	3	135834422	4.09E-02	4414	1.71E-01	Down	1.37	7932	1.39	0.08
rs1879240	64866	CDCP1	3	45107641	4.09E-02	4415	2.75E-01	Down	1.09	8999	1.39	0.06
rs11776266	26260	FBXO25	8	415541	4.09E-02	4416	1.18E-07	Up	5.30	1311	1.39	0.69
rs4426962	10802	SEC24A	5	134006828	4.09E-02	4417	6.18E-06	Up	4.52	1849	1.39	0.52
rs550942	80829	ZFP91	11	58150730	4.10E-02	4418	2.24E-01	Up	1.21	8499	1.39	0.06
rs550942	1270	CNTF	11	58150730	4.10E-02	4419	3.56E-01	Up	0.92	9645	1.39	0.04
rs2835342	8208	CHAF1B	21	36708547	4.10E-02	4420	6.81E-05	Up	3.98	2357	1.39	0.42
rs3010928	9249	DHRS3	1	12613007	4.10E-02	4421	3.87E-02	Down	2.07	5718	1.39	0.14
rs12420039	11098	PRSS23	11	86206085	4.10E-02	4422	4.99E-28	Down	10.98	79	1.39	2.73
rs12885226	3091	HIF1A	14	61268676	4.10E-02	4423	8.01E-01	Down	0.25	12684	1.39	0.01
rs3769769	29081	METTL5	2	170501272	4.10E-02	4424	1.78E-01	Up	1.35	8001	1.39	0.07
rs16903957	133584	EGFLAM	5	38406013	4.11E-02	4425	1.58E-02	Down	2.41	4867	1.39	0.18
rs11933240	79960	PHF17	4	130151164	4.11E-02	4426	1.57E-02	Up	2.42	4861	1.39	0.18
rs3149	55255	WDR41	5	76764192	4.11E-02	4427	2.02E-01	Down	1.28	8276	1.39	0.07
rs3742370	5687	PSMA6	14	34815393	4.11E-02	4428	4.95E-03	Up	2.81	4062	1.39	0.23
rs3742370	9692	KIAA0391	14	34815393	4.11E-02	4429	2.15E-01	Up	1.24	8411	1.39	0.07
rs12312186	3458	IFNG	12	66857437	4.11E-02	4430	4.86E-01	Up	0.70	10622	1.39	0.03
rs493733	23180	RFTN1	3	16392559	4.11E-02	4431	7.19E-04	Down	3.38	3088	1.39	0.31
rs130072	170680	PSORS1C2	6	31220463	4.12E-02	4432	1.39E-01	Down	1.48	7568	1.39	0.09
rs10791006	8538	BARX2	11	128768364	4.12E-02	4433	2.94E-01	Up	1.05	9148	1.39	0.05
rs7836120	4986	OPRK1	8	54320092	4.12E-02	4434	1.42E-02	Up	2.45	4765	1.39	0.18
rs2305556	3880	KRT19	17	36925250	4.12E-02	4435	1.80E-11	Down	6.72	668	1.39	1.07
rs2305556	3866	KRT15	17	36925250	4.12E-02	4436	1.98E-10	Down	6.36	792	1.39	0.97
rs2305556	3860	KRT13	17	36925250	4.12E-02	4437	2.92E-05	Down	4.18	2165	1.39	0.45
rs11602954	147199	SCGB1C1	11	192856	4.12E-02	4438	3.50E-02	Down	2.11	5623	1.38	0.15
rs11602954	51272	BET1L	11	192856	4.12E-02	4439	2.40E-01	Up	1.18	8656	1.38	0.06
rs11602954	113746	ODF3	11	192856	4.12E-02	4440	5.32E-01	Up	0.62	10959	1.38	0.03
rs1551310	219931	TPCN2	11	68613614	4.12E-02	4441	9.24E-01	Down	0.10	13395	1.38	0.00
rs6828925	51056	LAP3	4	17237408	4.13E-02	4442	1.58E-02	Up	2.41	4864	1.38	0.18
rs806509	51302	CYP39A1	6	46625122	4.13E-02	4443	7.84E-07	Up	4.94	1540	1.38	0.61
rs4533428	79595	SAP130	2	128517454	4.13E-02	4444	5.61E-02	Up	1.91	6138	1.38	0.13
rs4533428	23450	SF3B3	2	128517454	4.13E-02	4445	2.49E-01	Up	1.15	8758	1.38	0.06
rs2910344	148156	ZNF558	19	8781984	4.13E-02	4446	5.13E-04	Up	3.47	2968	1.38	0.33
rs11807825	64123	ELTD1	1	79205957	4.14E-02	4447	9.05E-01	Down	0.12	13280	1.38	0.00
rs1378149	8470	SORBS2	4	187229178	4.14E-02	4448	1.02E-05	Down	4.41	1953	1.38	0.50
rs4842163	1289	COL5A1	9	134888686	4.14E-02	4449	8.40E-03	Down	2.64	4375	1.38	0.21
rs487561	1057	CELP	9	132986671	4.14E-02	4450	9.26E-01	Down	0.09	13402	1.38	0.00

rs4352437	5099	PCDH7	4	30742939	4.14E-02	4451	4.56E-12	Down	6.92	621	1.38	1.13
rs2396835	221391	OPN5	6	47847744	4.14E-02	4452	4.61E-01	Down	0.74	10435	1.38	0.03
rs12973410	10365	KLF2	19	16286013	4.14E-02	4453	6.41E-01	Down	0.47	11719	1.38	0.02
rs13011946	79083	MLPH	2	238225025	4.14E-02	4454	1.82E-02	Up	2.36	4994	1.38	0.17
rs1130866	6439	SFTPB	2	85805399	4.15E-02	4455	8.97E-01	Down	0.13	13221	1.38	0.00
rs551356	92291	CAPN13	2	30896501	4.15E-02	4456	1.87E-01	Down	1.32	8106	1.38	0.07
rs1530599	6875	TAF4B	18	22085647	4.15E-02	4457	7.39E-04	Up	3.37	3099	1.38	0.31
rs6533371	6164	RPL34	4	109904621	4.15E-02	4458	3.93E-04	Up	3.54	2879	1.38	0.34
rs7933285	847	CAT	11	34433701	4.15E-02	4459	1.42E-01	Down	1.47	7597	1.38	0.08
rs4742921	25934	NIPSNAP3A	9	104595670	4.15E-02	4460	6.37E-02	Up	1.85	6315	1.38	0.12
rs4742921	55335	NIPSNAP3B	9	104595670	4.15E-02	4461	5.81E-01	Down	0.55	11326	1.38	0.02
rs11776844	80005	DOCK5	8	25287410	4.15E-02	4462	3.66E-01	Down	0.90	9730	1.38	0.04
rs3815081	89870	TRIM15	6	30222053	4.16E-02	4463	1.05E-01	Down	1.62	7081	1.38	0.10
rs3815081	135644	TRIM40	6	30222053	4.16E-02	4464	5.62E-01	Down	0.58	11180	1.38	0.03
rs3815081	10107	TRIM10	6	30222053	4.16E-02	4465	7.26E-01	Down	0.35	12232	1.38	0.01
rs3747076	7625	ZNF74	22	19084226	4.16E-02	4466	2.19E-02	Up	2.29	5154	1.38	0.17
rs9654959	3696	ITGB8	7	20168426	4.16E-02	4467	3.97E-13	Down	7.26	521	1.38	1.24
rs4659411	10256	CNKSR1	1	26200987	4.16E-02	4468	6.19E-06	Up	4.52	1851	1.38	0.52
rs1317470	3762	KCNJ5	11	128287424	4.16E-02	4469	2.33E-01	Down	1.19	8595	1.38	0.06
rs1317470	219833	C11orf45	11	128287424	4.16E-02	4470	5.98E-01	Down	0.53	11419	1.38	0.02
rs757052	7516	XRCC2	7	151771630	4.16E-02	4471	2.06E-02	Up	2.31	5098	1.38	0.17
rs2856585	368	ABCC6	16	16171164	4.16E-02	4472	1.79E-09	Down	6.02	933	1.38	0.87
rs11756587	84947	SERAC1	6	158543384	4.16E-02	4473	6.59E-01	Up	0.44	11822	1.38	0.02
rs5745325	5876	RABGGTB	1	75981481	4.16E-02	4474	1.60E-14	Up	7.67	426	1.38	1.38
rs5749779	29799	YPEL1	22	20424842	4.17E-02	4475	2.81E-10	Up	6.31	805	1.38	0.96
rs7523029	1503	CTPS	1	41118952	4.17E-02	4476	8.59E-01	Up	0.18	13007	1.38	0.01
rs469987	23466	CBX6	22	37611855	4.17E-02	4477	3.51E-01	Down	0.93	9600	1.38	0.05
rs2078478	11105	PRDM7	16	88657637	4.17E-02	4478	8.50E-01	Down	0.19	12958	1.38	0.01
rs10897222	10648	SCGB1D1	11	61701964	4.17E-02	4479	1.90E-01	Up	1.31	8149	1.38	0.07
rs11785444	5747	PTK2	8	141774178	4.17E-02	4480	4.09E-03	Up	2.87	3945	1.38	0.24
rs914561	50861	STMN3	20	61741349	4.18E-02	4481	5.17E-01	Up	0.65	10842	1.38	0.03
rs914561	26205	GMEB2	20	61741349	4.18E-02	4482	7.67E-01	Up	0.30	12472	1.38	0.01
rs1008680	9095	TBX19	1	165016703	4.18E-02	4483	7.29E-01	Up	0.35	12244	1.38	0.01
rs1892250	10050	SLC17A4	6	25877003	4.18E-02	4484	5.64E-01	Down	0.58	11191	1.38	0.02
rs407179	3293	HSD17B3	9	96091832	4.18E-02	4485	1.92E-01	Down	1.30	8172	1.38	0.07
rs12985354	933	CD22	19	40529268	4.18E-02	4486	1.65E-01	Down	1.39	7870	1.38	0.08
rs9841477	111	ADCY5	3	124640167	4.18E-02	4487	3.24E-03	Down	2.94	3821	1.38	0.25
rs17624672	51761	ATP8A2	13	25385257	4.18E-02	4488	1.56E-03	Up	3.16	3407	1.38	0.28
rs17760172	162282	ANKFN1	17	51918595	4.19E-02	4489	2.53E-01	Down	1.14	8796	1.38	0.06
rs801719	64781	CERK	22	45402864	4.19E-02	4490	9.30E-09	Down	5.74	1050	1.38	0.80
rs12417233	8539	API5	11	43330314	4.19E-02	4491	1.60E-05	Down	4.32	2050	1.38	0.48
rs17565905	11164	NUDT5	10	12279950	4.20E-02	4492	2.56E-01	Up	1.14	8823	1.38	0.06
rs9998530	25854	FAM149A	4	187422961	4.20E-02	4493	9.87E-01	Down	0.02	13827	1.38	0.00
rs2236891	3914	LAMB3	1	206192894	4.20E-02	4494	2.74E-09	Down	5.95	961	1.38	0.86
rs7324781	22873	DZIP1	13	95032754	4.20E-02	4495	3.50E-05	Down	4.14	2203	1.38	0.45
rs1322780	462	SERPINC1	1	170597243	4.20E-02	4496	6.65E-01	Up	0.43	11853	1.38	0.02
rs666229	2915	GRM5	11	88251764	4.20E-02	4497	6.53E-02	Up	1.84	6346	1.38	0.12
rs2701623	1840	DTX1	12	111985494	4.20E-02	4498	3.04E-01	Up	1.03	9223	1.38	0.05
rs1520484	4057	LTF	3	46488685	4.21E-02	4499	1.96E-04	Down	3.72	2623	1.38	0.37
rs7253017	2854	GPR32	19	55972568	4.21E-02	4500	5.02E-01	Up	0.67	10730	1.38	0.03
rs644396	8500	PPFIA1	11	69798149	4.22E-02	4501	4.93E-06	Up	4.57	1812	1.38	0.53
rs205908	1457	CSNK2A1	20	459897	4.22E-02	4502	2.65E-03	Up	3.01	3718	1.38	0.26
rs5916764	55086	CXorf57		105675779	4.22E-02	4503	9.12E-01	Down	0.11	13321	1.37	0.00
rs2303138	4012	LNPEP	5	96376466	4.22E-02	4504	4.62E-01	Down	0.74	10440	1.37	0.03
rs2099792	9647	PPM1F	22	20609067	4.22E-02	4505	6.29E-03	Down	2.73	4191	1.37	0.22
rs17028238	1977	EIF4E	4	100178642	4.22E-02	4506	3.34E-01	Down	0.97	9479	1.37	0.05
rs6115178	9837	GINS1	20	25348154	4.22E-02	4507	1.01E-02	Up	2.57	4517	1.37	0.20
rs1362153	374969	CCDC23	1	44138124	4.22E-02	4508	3.09E-01	Down	1.02	9264	1.37	0.05
rs1362153	6536	SLC6A9	1	44138124	4.22E-02	4509	5.57E-01	Down	0.59	11135	1.37	0.03
rs3765638	89849	ATG16L2	11	72214720	4.22E-02	4510	3.33E-01	Down	0.97	9465	1.37	0.05
rs9887921	6920	TCEA3	1	23446117	4.23E-02	4511	4.54E-05	Up	4.08	2258	1.37	0.43
rs9887921	80818	ZNF436	1	23446117	4.23E-02	4512	9.76E-01	Down	0.03	13732	1.37	0.00
rs7763896	1490	CTGF	6	132321497	4.23E-02	4513	1.64E-01	Up	1.39	7860	1.37	0.08
rs2642995	57116	ZNF695	1	243498606	4.23E-02	4514	2.16E-02	Up	2.30	5145	1.37	0.17
rs11951063	1004	CDH6	5	31277384	4.23E-02	4515	2.79E-02	Down	2.20	5389	1.37	0.16
rs7106795	56	ACRV1	11	125065390	4.23E-02	4516	9.96E-01	Down	0.00	13885	1.37	0.00
rs4648592	2782	GNB1	1	1833196	4.24E-02	4517	9.68E-03	Up	2.59	4486	1.37	0.20
rs12724116	9411	ARHGAP29	1	94401755	4.24E-02	4518	5.39E-02	Up	1.93	6086	1.37	0.13
rs6438542	64091	POPDC2	3	120881010	4.24E-02	4519	6.01E-12	Down	6.88	626	1.37	1.12
rs6438542	10063	COX17	3	120881010	4.24E-02	4520	1.18E-03	Up	3.24	3281	1.37	0.29
rs4736424	137835	TMEM71	8	133782292	4.24E-02	4521	7.84E-01	Up	0.27	12574	1.37	0.01
rs10022462	55008	HERC6	4	89600997	4.25E-02	4522	1.54E-03	Up	3.17	3393	1.37	0.28
rs1058261	1674	DES	2	220110814	4.25E-02	4523	3.14E-02	Down	2.15	5512	1.37	0.15
rs853362	9308	CD83	6	14246406	4.26E-02	4524	8.16E-01	Down	0.23	12762	1.37	0.01
rs1920296	55840	EAF2	3	123026267	4.26E-02	4525	8.92E-02	Down	1.70	6788	1.37	0.10
rs1920296	9657	IQCB1	3	123026267	4.26E-02	4526	9.81E-01	Down	0.02	13781	1.37	0.00
rs4824716	64840	PORCN		48104343	4.26E-02	4527	1.66E-01	Up	1.39	7871	1.37	0.08
rs4824716	92745	SLC38A5		48104343	4.26E-02	4528	2.11E-01	Up	1.25	8359	1.37	0.07
rs4824716	24140	FTSJ1		48104343	4.26E-02	4529	2.52E-01	Up	1.15	8775	1.37	0.06
rs3857957	29028	ATAD2	8	124495277	4.26E-02	4530	3.02E-01	Up	1.03	9204	1.37	0.05
rs11869026	9520	NPEPPS	17	43039650	4.26E-02	4531	3.60E-07	Up	5.09	1441	1.37	0.64

rs11666779	114770	PGLYRP2	19	15436801	4.26E-02	4532	7.24E-01	Down	0.35	12225	1.37	0.01
rs11666779	58525	WIZ	19	15436801	4.26E-02	4533	8.09E-01	Up	0.24	12721	1.37	0.01
rs6591348	51083	GAL	11	68197773	4.26E-02	4534	1.92E-03	Up	3.10	3513	1.37	0.27
rs1275509	10113	PREB	2	27285660	4.27E-02	4535	7.77E-02	Up	1.76	6589	1.37	0.11
rs1275509	339779	C2orf53	2	27285660	4.27E-02	4536	1.14E-01	Down	1.58	7201	1.37	0.09
rs2838616	386678	KRTAP10-11	21	44901550	4.27E-02	4537	1.98E-01	Up	1.29	8236	1.37	0.07
rs4721526	57037	ANKMY2	7	16473350	4.27E-02	4538	3.06E-09	Down	5.93	970	1.37	0.85
rs4889572	10308	ZNF267	16	31844443	4.27E-02	4539	9.70E-02	Down	1.66	6942	1.37	0.10
rs9606146	2812	GP1BB	22	18072683	4.28E-02	4540	6.11E-02	Down	1.87	6259	1.37	0.12
rs2301304	5251	PHEX		21821400	4.28E-02	4541	4.19E-01	Down	0.81	10117	1.37	0.04
rs11133360	3791	KDR	4	55823680	4.28E-02	4542	8.31E-11	Down	6.49	748	1.37	1.01
rs764324	2580	GAK	4	857696	4.29E-02	4543	6.92E-07	Up	4.96	1520	1.37	0.62
rs4083115	90632	C6orf176	6	166344308	4.29E-02	4544	2.13E-01	Up	1.25	8392	1.37	0.07
rs1155002	1573	CYP2J2	1	60085796	4.29E-02	4545	9.82E-10	Up	6.10	897	1.37	0.90
rs3812577	10807	SDCCAG3	9	136575474	4.29E-02	4546	4.05E-06	Up	4.61	1784	1.37	0.54
rs3812577	23203	PMPCA	9	136575474	4.29E-02	4547	3.02E-02	Up	2.17	5471	1.37	0.15
rs793715	2822	GPLD1	6	24553110	4.29E-02	4548	8.50E-01	Down	0.19	12957	1.37	0.01
rs339077	813	CALU	7	127955080	4.29E-02	4549	8.64E-02	Up	1.71	6744	1.37	0.11
rs17695156	6667	SP1	12	52092922	4.30E-02	4550	1.54E-01	Down	1.42	7736	1.37	0.08
rs17695156	269	AMHR2	12	52092922	4.30E-02	4551	6.94E-01	Up	0.39	12028	1.37	0.02
rs11130161	820	CAMP	3	48234635	4.30E-02	4552	7.22E-03	Down	2.69	4278	1.37	0.21
rs9749171	50509	COL5A3	19	9996711	4.30E-02	4553	1.40E-04	Down	3.81	2530	1.37	0.39
rs9749171	50700	RDH8	19	9996711	4.30E-02	4554	7.58E-01	Up	0.31	12430	1.37	0.01
rs4668367	9874	TLK1	2	171727207	4.30E-02	4555	3.07E-08	Up	5.54	1169	1.37	0.75
rs17135888	21	ABCA3	16	2323269	4.31E-02	4556	4.47E-11	Up	6.58	710	1.37	1.03
rs9513116	2321	FLT1	13	27914715	4.31E-02	4557	4.68E-01	Down	0.73	10485	1.37	0.03
rs2075827	23589	CARHSP1	16	8849319	4.31E-02	4558	6.36E-03	Up	2.73	4197	1.37	0.22
rs4958692	4238	MFAP3	5	153411458	4.31E-02	4559	3.74E-01	Up	0.89	9793	1.37	0.04
rs10516047	5917	RARS	5	167868597	4.31E-02	4560	1.30E-02	Up	2.49	4685	1.37	0.19
rs2309944	56977	STOX2	4	185233227	4.31E-02	4561	2.88E-01	Down	1.06	9100	1.37	0.05
rs9379858	10385	BTN2A2	6	26475668	4.32E-02	4562	7.17E-07	Down	4.96	1524	1.36	0.61
rs9379858	11118	BTN3A2	6	26475668	4.32E-02	4563	7.60E-02	Down	1.77	6548	1.36	0.11
rs12023499	8751	ADAM15	1	151844449	4.32E-02	4564	5.40E-08	Up	5.44	1230	1.36	0.73
rs12023499	1945	EFNA4	1	151844449	4.32E-02	4565	9.57E-04	Up	3.30	3204	1.36	0.30
rs7604035	84272	YIPF4	2	32451985	4.32E-02	4566	1.48E-02	Up	2.44	4804	1.36	0.18
rs12023499	149095	DCST1	1	151844449	4.32E-02	4567	3.05E-01	Up	1.03	9233	1.36	0.05
rs12023499	1944	EFNA3	1	151844449	4.32E-02	4568	5.64E-01	Up	0.58	11197	1.36	0.02
rs6540117	763	CA5A	16	86532466	4.32E-02	4569	9.82E-01	Down	0.02	13787	1.36	0.00
rs9344845	8732	RNGTT	6	89486461	4.33E-02	4570	5.16E-02	Down	1.95	6028	1.36	0.13
rs2694618	9736	USP34	2	61448635	4.33E-02	4571	2.28E-01	Up	1.21	8535	1.36	0.06
rs9299	3215	HOXB5	17	44024429	4.33E-02	4572	2.12E-01	Up	1.25	8372	1.36	0.07
rs9299	3214	HOXB4	17	44024429	4.33E-02	4573	3.07E-01	Down	1.02	9239	1.36	0.05
rs9299	3216	HOXB6	17	44024429	4.33E-02	4574	7.16E-01	Down	0.36	12169	1.36	0.01
rs4833392	132720	C4orf32	4	113416005	4.33E-02	4575	4.98E-01	Up	0.68	10696	1.36	0.03
rs1392100	771	CA12	15	61445689	4.33E-02	4576	4.33E-11	Down	6.59	702	1.36	1.04
rs1957889	57161	PELI2	14	55644910	4.33E-02	4577	2.68E-01	Down	1.11	8929	1.36	0.06
rs501397	56160	NDNL2	15	27366703	4.34E-02	4578	9.64E-01	Down	0.04	13643	1.36	0.00
rs12640135	84525	HOPX	4	57348669	4.34E-02	4579	8.00E-05	Down	3.94	2389	1.36	0.41
rs12640135	10963	STIP1	4	57348669	4.34E-02	4580	1.02E-02	Up	2.57	4523	1.36	0.20
rs7433237	200845	KCTD6	3	58441904	4.34E-02	4581	4.80E-02	Down	1.98	5951	1.36	0.13
rs4128287	83394	PITPNM3	17	6364130	4.34E-02	4582	1.63E-01	Down	1.39	7855	1.36	0.08
rs7909236	1558	CYP2C8	10	96819420	4.35E-02	4583	9.25E-02	Up	1.68	6874	1.36	0.10
rs1037170	146722	CD300LF	17	70214509	4.35E-02	4584	5.31E-01	Up	0.63	10944	1.36	0.03
rs1037170	326624	RAB37	17	70214509	4.35E-02	4585	6.45E-01	Down	0.46	11749	1.36	0.02
rs1862808	3801	KIFC3	16	56376519	4.35E-02	4586	1.37E-02	Down	2.46	4738	1.36	0.19
rs17042750	27177	IL1F8	2	113539382	4.35E-02	4587	2.77E-01	Up	1.09	9007	1.36	0.06
rs17042750	84639	IL1F10	2	113539382	4.35E-02	4588	6.08E-01	Up	0.51	11491	1.36	0.02
rs12366033	58473	PLEKHB1	11	73056622	4.35E-02	4589	2.04E-05	Up	4.26	2091	1.36	0.47
rs714948	56971	CEACAM19	19	49857752	4.36E-02	4590	7.56E-03	Up	2.67	4312	1.36	0.21
rs714948	5817	PVR	19	49857752	4.36E-02	4591	6.33E-01	Down	0.48	11663	1.36	0.02
rs2838923	378832	C21orf123	21	45671372	4.36E-02	4592	5.18E-01	Up	0.65	10847	1.36	0.03
rs2174780	25936	NSL1	1	209307802	4.36E-02	4593	9.92E-01	Up	0.01	13860	1.36	0.00
rs3017887	50507	NOX4	11	88865736	4.36E-02	4594	1.55E-06	Up	4.80	1634	1.36	0.58
rs663123	55280	CWF19L1	10	102004496	4.36E-02	4595	1.26E-01	Down	1.53	7383	1.36	0.09
rs6582065	64786	TBC1D15	12	70579433	4.36E-02	4596	5.18E-03	Up	2.80	4088	1.36	0.23
rs10483056		B3GALT5	21	39938899	4.36E-02	4597	7.00E-01	Down	0.39	12070	1.36	0.02
rs3763795	79949	C10orf81	10	115522098	4.37E-02	4598	1.44E-02	Up	2.45	4782	1.36	0.18
rs4713998	1026	CDKN1A	6	36740588	4.37E-02	4599	5.38E-02	Down	1.93	6083	1.36	0.13
rs1038497	29915	HCFC2	12	102946890	4.37E-02	4600	2.82E-01	Down	1.08	9049	1.36	0.05
rs12533064	669	BPGM	7	133773111	4.37E-02	4601	9.05E-01	Up	0.12	13279	1.36	0.00
rs829209	2254	FGF9	13	21133584	4.37E-02	4602	1.35E-05	Down	4.35	2019	1.36	0.49
rs645574	1665	DHX15	5	175040069	4.38E-02	4603	7.77E-05	Up	3.95	2383	1.36	0.41
rs645574	3274	HRH2	5	175040069	4.38E-02	4604	2.13E-01	Up	1.24	8394	1.36	0.07
rs12760299	81788	NUAK2	1	202001982	4.38E-02	4605	1.01E-02	Up	2.57	4514	1.36	0.20
rs4263048	317703	VN1R4	19	58469142	4.38E-02	4606	6.96E-01	Up	0.39	12047	1.36	0.02
rs4263048	342926	ZNF677	19	58469142	4.38E-02	4607	8.38E-01	Down	0.20	12892	1.36	0.01
rs2302867	22899	ARHGEF15	17	8157462	4.38E-02	4608	3.39E-03	Down	2.93	3841	1.36	0.25
rs2302867	399512	SLC25A35	17	8157462	4.38E-02	4609	5.11E-01	Down	0.66	10788	1.36	0.03
rs2670899	7200	TRH	3	131173753	4.38E-02	4610	7.91E-01	Down	0.27	12616	1.36	0.01
rs165924	3053	SERPIND1	22	19474894	4.38E-02	4611	1.89E-01	Up	1.31	8132	1.36	0.07
rs10488076	84668	FAM126A	7	22840978	4.40E-02	4612	3.63E-02	Down	2.09	5658	1.36	0.14

rs11071200	283659	PRTG	15	53737374	4.40E-02	4613	1.50E-01	Up	1.44	7683	1.36	0.08
rs2290239	51279	C1RL	12	7167525	4.40E-02	4614	6.87E-01	Up	0.40	11991	1.36	0.02
rs4708055	25821	MTO1	6	74224702	4.40E-02	4615	6.29E-02	Down	1.86	6295	1.36	0.12
rs4708055	115004	C6orf150	6	74224702	4.40E-02	4616	4.72E-01	Up	0.72	10513	1.36	0.03
rs11680268	79901	CYBRD1	2	172200964	4.40E-02	4617	8.40E-07	Down	4.93	1554	1.36	0.61
rs2562182	4350	MPG	16	73946	4.40E-02	4618	4.25E-02	Up	2.03	5817	1.36	0.14
rs2562182	64285	RHBDF1	16	73946	4.40E-02	4619	4.45E-01	Up	0.76	10307	1.36	0.04
rs7954395	2998	GYS2	12	21619527	4.40E-02	4620	3.30E-01	Up	0.97	9445	1.36	0.05
rs11758087	54435	HCG4	6	29872422	4.41E-02	4621	1.56E-01	Up	1.42	7756	1.36	0.08
rs10079250	1436	CSF1R	5	149430325	4.41E-02	4622	4.95E-01	Down	0.68	10680	1.36	0.03
rs7748974	84154	BXDC1	6	111462583	4.41E-02	4623	5.02E-09	Up	5.85	1013	1.36	0.83
rs4449310	152404	IGSF11	3	120282372	4.41E-02	4624	3.74E-01	Down	0.89	9795	1.36	0.04
rs4747009	55222	LRRC20	10	71784088	4.41E-02	4625	8.91E-01	Up	0.14	13193	1.36	0.01
rs11122478	2590	GALNT2	1	226730216	4.41E-02	4626	1.37E-01	Up	1.49	7534	1.36	0.09
rs2027320	3776	KCNK2	1	211768338	4.41E-02	4627	1.63E-01	Up	1.40	7849	1.36	0.08
rs7639314	26577	PCOLCE2	3	144085808	4.42E-02	4628	3.00E-02	Down	2.17	5462	1.36	0.15
rs1914261	65062	ALS2CR4	2	202319010	4.42E-02	4629	2.31E-04	Down	3.68	2681	1.35	0.36
rs1914261	151254	ALS2CR11	2	202319010	4.42E-02	4630	3.93E-01	Down	0.85	9937	1.35	0.04
rs9911412	140775	SMCR8	17	18146959	4.42E-02	4631	9.68E-05	Up	3.90	2443	1.35	0.40
rs9911412	7156	TOP3A	17	18146959	4.42E-02	4632	7.34E-02	Up	1.79	6492	1.35	0.11
rs8071332	26115	TANC2	17	58495685	4.42E-02	4633	2.04E-07	Up	5.19	1374	1.35	0.67
rs17527308	64850	AGXT2L1	4	110021218	4.42E-02	4634	1.16E-02	Down	2.52	4618	1.35	0.19
rs9388462	135114	HINT3	6	126318495	4.42E-02	4635	3.14E-02	Down	2.15	5513	1.35	0.15
rs7999941	122060	SLAIN1	13	77256389	4.42E-02	4636	2.92E-04	Up	3.62	2765	1.35	0.35
rs2726943	286133	SCARA5	8	27873403	4.43E-02	4637	1.14E-01	Down	1.58	7204	1.35	0.09
rs7722729	27430	MAT2B	5	162875014	4.43E-02	4638	1.36E-19	Down	9.06	211	1.35	1.89
rs1420306	80205	CHD9	16	51854896	4.43E-02	4639	7.87E-12	Down	6.84	632	1.35	1.11
rs4338396	55746	NUP133	1	225886956	4.43E-02	4640	2.51E-02	Down	2.24	5291	1.35	0.16
rs4338396	58	ACTA1	1	225886956	4.43E-02	4641	9.74E-01	Down	0.03	13724	1.35	0.00
rs10110845	286053	NSMCE2	8	126329316	4.43E-02	4642	6.34E-04	Up	3.42	3050	1.35	0.32
rs16986464	827	CAPN6		110310427	4.43E-02	4643	7.55E-07	Down	4.95	1530	1.35	0.61
rs11590351	127002	ATXN7L2	1	109747201	4.43E-02	4644	4.20E-01	Down	0.81	10126	1.35	0.04
rs1477745	23390	ZDHHC17	12	75715383	4.43E-02	4645	3.10E-01	Down	1.01	9269	1.35	0.05
rs6690069	978	CDA	1	20659757	4.43E-02	4646	3.33E-01	Down	0.97	9467	1.35	0.05
rs1887193	57578	KIAA1409	14	93162237	4.43E-02	4647	5.05E-01	Up	0.67	10750	1.35	0.03
rs7856889	4593	MUSK	9	110547101	4.44E-02	4648	6.07E-01	Up	0.51	11480	1.35	0.02
rs4244950	5995	RGR	10	86010680	4.44E-02	4649	1.43E-02	Down	2.45	4775	1.35	0.18
rs902695	23550	PSD4	2	113671305	4.44E-02	4650	4.70E-03	Up	2.83	4019	1.35	0.23
rs6067867	10079	ATP9A	20	49698286	4.44E-02	4651	3.58E-01	Down	0.92	9666	1.35	0.04
rs1373599	2559	GABRA6	5	161030280	4.44E-02	4652	5.26E-01	Up	0.63	10916	1.35	0.03
rs4735851	157695	C8orf42	8	443317	4.45E-02	4653	7.89E-02	Up	1.76	6613	1.35	0.11
rs2072408	2146	EZH2	7	147945845	4.45E-02	4654	2.28E-13	Up	7.32	499	1.35	1.26
rs17383360	473	RERE	1	8738709	4.45E-02	4655	2.33E-08	Up	5.58	1142	1.35	0.76
rs4666321	64342	HS1BP3	2	20762796	4.45E-02	4656	7.89E-01	Down	0.27	12601	1.35	0.01
rs2071232	4312	MMP1	11	102170879	4.45E-02	4657	6.69E-01	Up	0.43	11881	1.35	0.02
rs895459	580	BARD1	2	215425763	4.46E-02	4658	6.97E-01	Up	0.39	12053	1.35	0.02
rs7303892	114882	OSBPL8	12	75455510	4.46E-02	4659	1.24E-03	Up	3.23	3309	1.35	0.29
rs2369452	10316	NMUR1	2	232233523	4.46E-02	4660	9.32E-01	Down	0.08	13448	1.35	0.00
rs4887567	2567	GABRG3	15	25048678	4.47E-02	4661	3.56E-02	Up	2.10	5633	1.35	0.14
rs297802	84970	C1orf94	1	34343907	4.47E-02	4662	2.29E-02	Down	2.28	5191	1.35	0.16
rs2343868	8082	SSPN	12	26222207	4.47E-02	4663	3.13E-04	Down	3.60	2791	1.35	0.35
rs12551214	971	CD72	9	35623795	4.47E-02	4664	5.26E-01	Down	0.63	10910	1.35	0.03
rs6665593	199990	C1orf86	1	2172423	4.47E-02	4665	7.24E-03	Up	2.69	4280	1.35	0.21
rs727948	10576	CCT2	12	68286785	4.47E-02	4666	5.60E-11	Up	6.56	721	1.35	1.03
rs1740107	200010	SLC5A9	1	48431039	4.48E-02	4667	1.61E-01	Down	1.40	7827	1.35	0.08
rs17650369	85459	KIAA1731	11	93045711	4.48E-02	4668	1.21E-02	Up	2.51	4642	1.35	0.19
rs3753067	4152	MBD1	18	46067833	4.49E-02	4669	2.06E-01	Down	1.27	8314	1.35	0.07
rs3753067	30827	CXXC1	18	46067833	4.49E-02	4670	2.20E-01	Down	1.23	8456	1.35	0.07
rs6568622	2830	GPR6	6	110405000	4.49E-02	4671	9.77E-01	Up	0.03	13748	1.35	0.00
rs7729111	26999	CYFIP2	5	156676631	4.49E-02	4672	5.31E-15	Up	7.82	390	1.35	1.43
rs735911	84823	LMNB2	19	2373177	4.49E-02	4673	2.02E-03	Up	3.09	3535	1.35	0.27
rs735911	26517	TIMM13	19	2373177	4.49E-02	4674	8.25E-03	Up	2.64	4364	1.35	0.21
rs282123	2570	GABRR2	6	90016941	4.49E-02	4675	2.77E-01	Down	1.09	9009	1.35	0.06
rs4296949	80028	FBXL18	7	5281885	4.49E-02	4676	1.94E-02	Up	2.34	5042	1.35	0.17
rs11444	79706	PRKRIP1	7	101660593	4.49E-02	4677	6.72E-03	Up	2.71	4234	1.35	0.22
rs2074625	55643	BTBD2	19	1948729	4.49E-02	4678	4.23E-02	Up	2.03	5807	1.35	0.14
rs6135141	245938	DEFB125	20	22347	4.49E-02	4679	6.43E-01	Down	0.46	11731	1.35	0.02
rs37248	2149	F2R	5	76052702	4.49E-02	4680	6.75E-36	Up	12.45	36	1.35	3.52
rs9310783	1663	DDX11	3	292145	4.49E-02	4681	2.43E-01	Up	1.17	8694	1.35	0.06
rs9310783	10752	CHL1	3	292145	4.49E-02	4682	7.14E-01	Down	0.37	12158	1.35	0.01
rs2240865	3736	KCNA1	12	4897914	4.49E-02	4683	4.56E-03	Up	2.84	4006	1.35	0.23
rs16986050	2204	FCAR	19	60092982	4.50E-02	4684	1.48E-02	Up	2.44	4802	1.35	0.18
rs16986050	9437	NCR1	19	60092982	4.50E-02	4685	1.74E-01	Down	1.36	7958	1.35	0.08
rs3803452	6938	TCF12	15	55367038	4.50E-02	4686	6.76E-01	Up	0.42	11922	1.35	0.02
rs5925612	23597	ACOT9		23540345	4.50E-02	4687	2.63E-01	Down	1.12	8892	1.35	0.06
rs6831087	6870	TACR3	4	104976694	4.50E-02	4688	8.11E-01	Up	0.24	12725	1.35	0.01
rs7539544	11147	HHLA3	1	70543862	4.50E-02	4689	1.15E-06	Up	4.86	1594	1.35	0.59
rs7539544	81573	ANKRD13C	1	70543862	4.50E-02	4690	8.48E-01	Down	0.19	12937	1.35	0.01
rs6754934	85461	TANC1	2	159816171	4.51E-02	4691	1.13E-03	Up	3.26	3269	1.35	0.29
rs1054185	84612	PARD6B	20	48802413	4.51E-02	4692	9.27E-01	Down	0.09	13414	1.35	0.00
rs241816	6492	SIM1	6	100986372	4.51E-02	4693	7.24E-02	Up	1.80	6476	1.35	0.11

rs4981369	57096	RPGRIP1	14	20846360	4.52E-02	4694	4.01E-01	Up	0.84	9988	1.35	0.04
rs4838215	169611	OLFML2A	9	124653304	4.52E-02	4695	2.92E-10	Down	6.30	808	1.34	0.95
rs3893128	50944	SHANK1	19	55867867	4.52E-02	4696	4.30E-02	Down	2.02	5828	1.34	0.14
rs1887596	10810	WASF3	13	26169370	4.52E-02	4697	5.00E-08	Up	5.45	1225	1.34	0.73
rs225121	11315	PARK7	1	7980813	4.52E-02	4698	9.37E-04	Up	3.31	3187	1.34	0.30
rs11247860	51042	ZNF593	1	26175497	4.53E-02	4699	5.49E-04	Up	3.46	2995	1.34	0.33
rs11247860	79927	GRRP1	1	26175497	4.53E-02	4700	9.52E-01	Up	0.06	13570	1.34	0.00
rs12112329	9678	PHF14	7	10781705	4.53E-02	4701	3.39E-02	Up	2.12	5588	1.34	0.15
rs1240390	10974	C10orf116	10	88715964	4.53E-02	4702	3.45E-21	Down	9.45	176	1.34	2.05
rs4714505	7942	TFEB	6	41756125	4.54E-02	4703	8.23E-01	Down	0.22	12806	1.34	0.01
rs1345198	84620	ST6GAL2	2	106876554	4.54E-02	4704	8.99E-01	Up	0.13	13235	1.34	0.00
rs2070535	8566	PDXK	21	44000732	4.55E-02	4705	8.41E-03	Up	2.64	4376	1.34	0.21
rs4244808	3481	IGF2	11	2119686	4.55E-02	4706	1.49E-15	Down	7.98	365	1.34	1.48
rs4244808	51214	IGF2AS	11	2119686	4.55E-02	4707	5.41E-01	Down	0.61	11018	1.34	0.03
rs11666402	83854	ANGPTL6	19	10080076	4.55E-02	4708	7.72E-02	Down	1.77	6580	1.34	0.11
rs11666402	56342	PPAN	19	10080076	4.55E-02	4709	3.33E-01	Down	0.97	9469	1.34	0.05
rs11666402	5032	P2RY11	19	10080076	4.55E-02	4710	5.17E-01	Down	0.65	10835	1.34	0.03
rs11713193	4486	MST1R	3	49899428	4.55E-02	4711	4.18E-01	Up	0.81	10107	1.34	0.04
rs7972963	11213	IRAK3	12	64932466	4.55E-02	4712	9.15E-01	Down	0.11	13347	1.34	0.00
rs10778456	9891	NUAK1	12	104950769	4.55E-02	4713	7.45E-06	Down	4.48	1883	1.34	0.51
rs7122944	51092	SIDT2	11	116539829	4.55E-02	4714	3.14E-04	Down	3.60	2793	1.34	0.35
rs7122944	5049	PAFAH1B2	11	116539829	4.55E-02	4715	8.81E-03	Down	2.62	4414	1.34	0.21
rs2297602	1306	COL15A1	9	98868077	4.55E-02	4716	9.43E-01	Down	0.07	13509	1.34	0.00
rs10819596	57720	GPR107	9	129908908	4.56E-02	4717	8.36E-02	Up	1.73	6695	1.34	0.11
rs3790715	10390	CEPT1	1	111425714	4.56E-02	4718	2.69E-01	Up	1.11	8943	1.34	0.06
rs1204897	55920	RCC2	1	17483473	4.56E-02	4719	7.02E-20	Up	9.11	205	1.34	1.92
rs1551343	8125	ANP32A	15	66872811	4.56E-02	4720	6.76E-01	Up	0.42	11925	1.34	0.02
rs931647	147906	DACT3	19	51835208	4.56E-02	4721	2.87E-01	Down	1.07	9089	1.34	0.05
rs931647	5739	PTGIR	19	51835208	4.56E-02	4722	6.15E-01	Down	0.50	11541	1.34	0.02
rs772601	22802	CLCA4	1	86744345	4.57E-02	4723	8.12E-02	Down	1.74	6652	1.34	0.11
rs3764482	4092	SMAD7	18	44722944	4.57E-02	4724	5.15E-03	Down	2.80	4081	1.34	0.23
rs5914241	7216	TRO		54829657	4.57E-02	4725	2.74E-07	Down	5.14	1406	1.34	0.66
rs9860191	84303	CHCHD6	3	128067498	4.57E-02	4726	3.64E-01	Up	0.91	9712	1.34	0.04
rs12475686	1286	COL4A4	2	227799128	4.57E-02	4727	4.65E-01	Down	0.73	10464	1.34	0.03
rs907938	23746	AIPL1	17	6268275	4.58E-02	4728	1.71E-02	Down	2.38	4942	1.34	0.18
rs10496175	270	AMPD1	2	70065499	4.58E-02	4729	5.39E-01	Up	0.61	11003	1.34	0.03
rs1711973	2295	FOXF2	6	1347536	4.58E-02	4730	2.30E-01	Up	1.20	8564	1.34	0.06
rs12487673	25907	TMEM158	3	45256932	4.58E-02	4731	3.24E-10	Down	6.29	816	1.34	0.95
rs12487673	7386	UQCRFS1	3	45256932	4.58E-02	4732	8.56E-03	Up	2.63	4389	1.34	0.21
rs915171	2037	EPB41L2	6	131226607	4.58E-02	4733	7.29E-06	Down	4.49	1877	1.34	0.51
rs2035255	25979	DHRS7B	17	20989505	4.58E-02	4734	6.09E-01	Up	0.51	11495	1.34	0.02
rs10409750	66002	CYP4F12	19	15652606	4.58E-02	4735	2.36E-10	Down	6.34	798	1.34	0.96
rs7576218	64895	PAPOLG	2	60879612	4.59E-02	4736	2.06E-01	Down	1.26	8319	1.34	0.07
rs1883042	386685	KRTAP10-12	21	44925450	4.59E-02	4737	1.18E-01	Up	1.56	7267	1.34	0.09
rs4662787	9394	HS6ST1	2	128752207	4.59E-02	4738	4.89E-01	Down	0.69	10649	1.34	0.03
rs2305115	284459	HKR1	19	42527498	4.59E-02	4739	1.48E-04	Up	3.79	2547	1.34	0.38
rs11654048	11276	AP1GBP1	17	33010707	4.59E-02	4740	5.59E-01	Up	0.58	11154	1.34	0.03
rs2151513	3911	LAMA5	20	60340841	4.59E-02	4741	2.60E-02	Down	2.23	5320	1.34	0.16
rs7245068	9989	PPP4R1	18	9548878	4.59E-02	4742	8.62E-04	Up	3.33	3158	1.34	0.31
rs3890714	22879	MON1B	16	75763458	4.60E-02	4743	1.06E-20	Up	9.34	180	1.34	2.00
rs4305747	9038	TAAR5	6	132959305	4.60E-02	4744	4.54E-01	Down	0.75	10370	1.34	0.03
rs4305747	10002	NR2E3	6	132959305	4.60E-02	4745	5.93E-01	Down	0.53	11392	1.34	0.02
rs6067731	56259	CTNNBL1	20	35878290	4.60E-02	4746	1.71E-05	Down	4.30	2064	1.34	0.48
rs4758888	55743	CHFR	12	132073859	4.60E-02	4747	7.29E-01	Down	0.35	12243	1.34	0.01
rs17704641	54949	C11orf79	11	60939964	4.61E-02	4748	9.82E-01	Up	0.02	13790	1.34	0.00
rs3757552	51384	WNT16	7	120557916	4.61E-02	4749	7.59E-01	Up	0.31	12436	1.34	0.01
rs3917254	3554	IL1R1	2	102235036	4.61E-02	4750	1.21E-02	Down	2.51	4643	1.34	0.19
rs11171681	29095	ORMDL2	12	54479500	4.61E-02	4751	3.16E-03	Up	2.95	3807	1.34	0.25
rs17367052	93035	PKHD1L1	8	110477703	4.61E-02	4752	3.14E-03	Down	2.95	3803	1.34	0.25
rs11167761	5097	PCDH1	5	141218527	4.61E-02	4753	2.88E-05	Up	4.18	2162	1.34	0.45
rs1992868	8743	TNFSF10	3	173703678	4.62E-02	4754	8.64E-07	Up	4.92	1556	1.34	0.61
rs456998	8841	HDAC3	5	141005346	4.62E-02	4755	1.29E-01	Up	1.52	7439	1.34	0.09
rs456998	89848	FCHSD1	5	141005346	4.62E-02	4756	2.25E-01	Down	1.21	8506	1.34	0.06
rs12153009	2690	GHR	5	42589636	4.62E-02	4757	1.56E-07	Up	5.25	1345	1.34	0.68
rs11644557	81533	ITFG1	16	45751552	4.62E-02	4758	5.01E-01	Up	0.67	10726	1.34	0.03
rs16996446	80833	APOL3	22	34884794	4.63E-02	4759	1.16E-02	Up	2.52	4612	1.33	0.19
rs2704219	8854	ALDH1A2	15	56115740	4.63E-02	4760	3.78E-18	Down	8.69	265	1.33	1.74
rs1886302	23569	PADI4	1	17380702	4.63E-02	4761	4.71E-01	Up	0.72	10509	1.33	0.03
rs11927897	132141	IQCF1	3	51904223	4.63E-02	4762	1.14E-01	Up	1.58	7199	1.33	0.09
rs1898462	36	ACADSB	10	124801456	4.64E-02	4763	2.03E-08	Up	5.61	1122	1.33	0.77
rs12229754	283417	DPY19L2	12	62349033	4.64E-02	4764	9.24E-03	Up	2.60	4449	1.33	0.20
rs11557467	124626	ZPBP2	17	35282160	4.64E-02	4765	3.08E-01	Up	1.02	9252	1.33	0.05
rs7111203	1479	CSTF3	11	33053971	4.64E-02	4766	1.40E-09	Up	6.05	917	1.33	0.89
rs6664254	34	ACADM	1	75947105	4.64E-02	4767	5.20E-02	Up	1.94	6040	1.33	0.13
rs866624	26248	OR2K2	9	111167251	4.66E-02	4768	9.85E-02	Up	1.65	6973	1.33	0.10
rs1995874	6675	UAP1	1	159303268	4.66E-02	4769	-7.05E-58	Up	16.77	3	1.33	#NUM!
rs659463	4135	MAP6	11	74994821	4.66E-02	4770	1.46E-01	Down	1.45	7636	1.33	0.08
rs3777218	22836	RHOBTB3	5	95138622	4.66E-02	4771	8.72E-03	Down	2.62	4407	1.33	0.21
rs6928425	221481	C6orf81	6	35821009	4.66E-02	4772	4.25E-02	Up	2.03	5815	1.33	0.14
rs13074670	131831	C3orf44	3	151911829	4.66E-02	4773	9.92E-02	Down	1.65	6984	1.33	0.10
rs9811107	285315	C3orf33	3	156957581	4.66E-02	4774	8.09E-01	Down	0.24	12717	1.33	0.01

rs4753538	24145	PANX1	11	93490696	4.67E-02	4775	7.13E-01	Up	0.37	12155	1.33	0.01
rs1800458	7276	TTR	18	27426863	4.67E-02	4776	2.46E-01	Up	1.16	8718	1.33	0.06
rs870957	7570	ZNF22	10	44798098	4.67E-02	4777	2.30E-02	Up	2.27	5204	1.33	0.16
rs870957	83937	RASSF4	10	44798098	4.67E-02	4778	2.63E-01	Down	1.12	8894	1.33	0.06
rs870957	11067	C10orf10	10	44798098	4.67E-02	4779	7.89E-01	Down	0.27	12603	1.33	0.01
rs2885987	9183	ZW10	11	113107765	4.67E-02	4780	9.73E-01	Up	0.03	13715	1.33	0.00
rs7998964	9445	ITM2B	13	47686991	4.67E-02	4781	2.11E-11	Down	6.70	674	1.33	1.07
rs4300381	196074	METT5D1	11	28269673	4.67E-02	4782	1.05E-03	Up	3.28	3243	1.33	0.30
rs12465555	10128	LRPPRC	2	44029840	4.68E-02	4783	1.36E-04	Up	3.82	2520	1.33	0.39
rs3739719	54534	MRPL50	9	101248644	4.68E-02	4784	2.10E-03	Up	3.08	3566	1.33	0.27
rs3739719	7743	ZNF189	9	101248644	4.68E-02	4785	9.37E-01	Up	0.08	13469	1.33	0.00
rs10139666	10484	SEC23A	14	38562908	4.68E-02	4786	1.30E-27	Down	10.89	80	1.33	2.69
rs211587	349565	NMNAT3	3	140763851	4.68E-02	4787	8.71E-01	Up	0.16	13070	1.33	0.01
rs2486961	1118	CHIT1	1	199923561	4.68E-02	4788	3.53E-02	Up	2.10	5632	1.33	0.15
rs11991630	9639	ARHGEF10	8	1844329	4.69E-02	4789	4.07E-01	Up	0.83	10034	1.33	0.04
rs12515434	3673	ITGA2	5	52433343	4.69E-02	4790	5.05E-25	Down	10.33	114	1.33	2.43
rs12515434	4338	MOCS2	5	52433343	4.69E-02	4791	8.17E-01	Down	0.23	12770	1.33	0.01
rs3781196	55328	C10orf59	10	90032134	4.69E-02	4792	7.50E-02	Up	1.78	6528	1.33	0.11
rs11737692	57732	ZFYVE28	4	2254652	4.69E-02	4793	5.36E-01	Up	0.62	10982	1.33	0.03
rs1050565	6532	SLC6A4	17	25600202	4.69E-02	4794	6.33E-01	Down	0.48	11662	1.33	0.02
rs808964	975	CD81	11	2343176	4.71E-02	4795	5.36E-09	Down	5.84	1018	1.33	0.83
rs724577	254251	LCORL	4	17669679	4.71E-02	4796	7.15E-06	Up	4.49	1874	1.33	0.51
rs9626074	25813	SAMM50	22	42688637	4.71E-02	4797	3.75E-02	Up	2.08	5694	1.33	0.14
rs3807979	26136	TES	7	115487313	4.71E-02	4798	5.74E-10	Down	6.20	863	1.33	0.92
rs1885318	6039	RNASE6	14	20314536	4.71E-02	4799	1.74E-01	Up	1.36	7957	1.33	0.08
rs1885318	64184	FAM12B	14	20314536	4.71E-02	4800	4.21E-01	Down	0.80	10129	1.33	0.04
rs7136515	84125	LRRIQ1	12	84029783	4.71E-02	4801	2.37E-03	Up	3.04	3654	1.33	0.26
rs4541346	11280	SCN11A	3	38868494	4.71E-02	4802	6.84E-01	Up	0.41	11972	1.33	0.02
rs1684618	124401	ANKS3	16	4696680	4.72E-02	4803	3.32E-01	Down	0.97	9458	1.33	0.05
rs1042636	846	CASR	3	123486459	4.72E-02	4804	2.25E-01	Up	1.21	8508	1.33	0.06
rs561285	11251	GPR44	11	60365509	4.72E-02	4805	4.04E-01	Down	0.83	10014	1.33	0.04
rs561285	79080	CCDC86	11	60365509	4.72E-02	4806	6.14E-01	Up	0.50	11540	1.33	0.02
rs12613347	843	CASP10	2	201880818	4.72E-02	4807	8.43E-01	Down	0.20	12922	1.33	0.01
rs16945930	85320	ABCC11	16	46791828	4.73E-02	4808	9.66E-01	Up	0.04	13667	1.33	0.00
rs17449015	5602	MAPK10	4	87579006	4.73E-02	4809	5.18E-06	Down	4.56	1824	1.33	0.53
rs2072341	115	ADCY9	16	3979142	4.73E-02	4810	2.18E-06	Down	4.74	1681	1.33	0.57
rs527051	84881	RPUSD4	11	125572158	4.73E-02	4811	3.06E-08	Up	5.54	1168	1.33	0.75
rs3851706	123811	C16orf63	16	15858148	4.73E-02	4812	1.38E-01	Down	1.48	7547	1.33	0.09
rs11216322	9159	PCSK7	11	116599701	4.73E-02	4813	1.78E-03	Down	3.13	3470	1.32	0.28
rs11216322	257160	RNF214	11	116599701	4.73E-02	4814	3.15E-01	Up	1.01	9313	1.32	0.05
rs12980646	23474	ETHE1	19	48700368	4.73E-02	4815	4.61E-03	Up	2.83	4011	1.32	0.23
rs12980646	653583	PHLDB3	19	48700368	4.73E-02	4816	1.03E-01	Down	1.63	7043	1.32	0.10
rs2965101	602	BCL3	19	49929652	4.74E-02	4817	6.46E-01	Down	0.46	11755	1.32	0.02
rs1992950	23314	SATB2	2	200115865	4.74E-02	4818	6.92E-05	Up	3.98	2360	1.32	0.42
rs916620	1757	SARDH	9	133626304	4.74E-02	4819	6.16E-01	Down	0.50	11551	1.32	0.02
rs2429013	7982	ST7	7	116338468	4.74E-02	4820	7.00E-09	Up	5.81	1027	1.32	0.82
rs2429013	29967	LRP12	7	116338468	4.74E-02	4821	3.19E-02	Up	2.15	5522	1.32	0.15
rs7118248	120114	FAT3	11	92262997	4.74E-02	4822	2.53E-05	Down	4.21	2134	1.32	0.46
rs6851218	152816	C4orf26	4	76850156	4.74E-02	4823	2.19E-01	Down	1.23	8444	1.32	0.07
rs3804348	3083	HGFAC	4	3468311	4.75E-02	4824	3.30E-01	Up	0.97	9439	1.32	0.05
rs889635	29948	OSGIN1	16	82523747	4.75E-02	4825	7.43E-01	Down	0.33	12335	1.32	0.01
rs2272427	166824	RASSF6	4	74812527	4.75E-02	4826	2.77E-02	Up	2.20	5384	1.32	0.16
rs11170980	5502	PPP1R1A	12	53262379	4.75E-02	4827	4.57E-04	Down	3.50	2923	1.32	0.33
rs11170980	5153	PDE1B	12	53262379	4.75E-02	4828	2.23E-01	Down	1.22	8487	1.32	0.07
rs4371861	81847	RNF146	6	127610140	4.75E-02	4829	1.35E-02	Down	2.47	4724	1.32	0.19
rs2141662	26628	OR7E47P	12	50791652	4.76E-02	4830	3.18E-07	Up	5.11	1422	1.32	0.65
rs11767557	2041	EPHA1	7	142625976	4.76E-02	4831	2.73E-06	Up	4.69	1719	1.32	0.56
rs2511224	10072	DPP3	11	66019182	4.76E-02	4832	6.88E-03	Up	2.70	4248	1.32	0.22
rs2511224	582	BBS1	11	66019182	4.76E-02	4833	2.11E-01	Down	1.25	8364	1.32	0.07
rs11613607	55785	FGD6	12	94031895	4.77E-02	4834	8.81E-02	Down	1.71	6772	1.32	0.11
rs17425839	5577	PRKAR2B	7	106357229	4.77E-02	4835	3.46E-05	Down	4.14	2202	1.32	0.45
rs1535453	55848	C9orf46	9	5400624	4.77E-02	4836	9.81E-01	Up	0.02	13778	1.32	0.00
rs3845787	10602	CDC42EP3	2	37827711	4.77E-02	4837	8.22E-27	Down	10.72	88	1.32	2.61
rs4803238	6217	RPS16	19	44631831	4.78E-02	4838	1.78E-16	Up	8.23	334	1.32	1.57
rs4803238	6829	SUPT5H	19	44631831	4.78E-02	4839	2.86E-01	Up	1.07	9077	1.32	0.05
rs2410806	152002	C3orf21	3	196401252	4.78E-02	4840	4.43E-01	Down	0.77	10292	1.32	0.04
rs1054628	3381	IBSP	4	89090090	4.78E-02	4841	5.68E-02	Up	1.90	6154	1.32	0.12
rs12453963	10209	EIF1	17	37091630	4.78E-02	4842	2.29E-06	Down	4.73	1688	1.32	0.56
rs3804665	5352	PLOD2	3	147291028	4.79E-02	4843	1.98E-01	Down	1.29	8233	1.32	0.07
rs7653452	961	CD47	3	109263096	4.79E-02	4844	1.14E-06	Down	4.87	1591	1.32	0.59
rs9439731	5081	PAX7	1	18804777	4.79E-02	4845	4.90E-01	Up	0.69	10656	1.32	0.03
rs4518636	1345	COX6C	8	100973453	4.79E-02	4846	1.30E-01	Down	1.51	7450	1.32	0.09
rs3775494	374	AREG	4	75682171	4.80E-02	4847	9.38E-01	Down	0.08	13478	1.32	0.00
rs7592114	114880	OSBPL6	2	179012036	4.80E-02	4848	1.13E-02	Down	2.53	4594	1.32	0.19
rs626457	732	C8B	1	57119505	4.80E-02	4849	5.48E-01	Up	0.60	11063	1.32	0.03
rs999338	8735	MYH13	17	10139554	4.80E-02	4850	4.02E-01	Down	0.84	9994	1.32	0.04
rs3780280	10319	LAMC3	9	130935804	4.80E-02	4851	6.70E-01	Down	0.43	11885	1.32	0.02
rs7119403	63876	PKNOX2	11	124772376	4.80E-02	4852	9.06E-03	Down	2.61	4438	1.32	0.20
rs2450135	9846	GAB2	11	77605643	4.80E-02	4853	3.38E-03	Down	2.93	3839	1.32	0.25
rs6030932	26013	L3MBTL	20	41579734	4.81E-02	4854	6.31E-01	Down	0.48	11647	1.32	0.02
rs10138768	23368	PPP1R13B	14	103260280	4.81E-02	4855	8.17E-02	Up	1.74	6663	1.32	0.11

rs10138768	79038	ZFYVE21	14	103260280	4.81E-02	4856	1.70E-01	Down	1.37	7918	1.32	0.08
rs10138768	7517	XRCC3	14	103260280	4.81E-02	4857	5.51E-01	Down	0.60	11086	1.32	0.03
rs3782287	949	SCARB1	12	123814145	4.81E-02	4858	1.47E-08	Up	5.64	1109	1.32	0.78
rs2772189	286410	ATP11C		138525068	4.81E-02	4859	2.48E-01	Up	1.16	8746	1.32	0.06
rs10859990	144193	AMDHD1	12	94838507	4.81E-02	4860	4.82E-01	Up	0.70	10588	1.32	0.03
rs10859990	120935	CCDC38	12	94838507	4.81E-02	4861	9.13E-01	Down	0.11	13334	1.32	0.00
rs3803025	904	CCNT1	12	47370701	4.81E-02	4862	2.48E-02	Up	2.24	5278	1.32	0.16
rs3803025	54934	C12orf41	12	47370701	4.81E-02	4863	4.78E-02	Up	1.98	5942	1.32	0.13
rs2280400	51195	RAPGEFL1	17	35602653	4.82E-02	4864	4.09E-04	Down	3.53	2892	1.32	0.34
rs9661462	127254	C1orf173	1	74763443	4.82E-02	4865	9.65E-01	Up	0.04	13646	1.32	0.00
rs4459374	84216	TMEM117	12	42837334	4.82E-02	4866	2.16E-03	Down	3.07	3583	1.32	0.27
rs344783	5329	PLAUR	19	48864028	4.82E-02	4867	2.45E-01	Down	1.16	8712	1.32	0.06
rs17166875	5446	PON3	7	94685922	4.82E-02	4868	7.49E-05	Down	3.96	2376	1.32	0.41
rs503327	93	ACVR2B	3	38499367	4.82E-02	4869	1.84E-11	Up	6.71	670	1.32	1.07
rs17199254	10202	DHRS2	14	23185313	4.82E-02	4870	3.57E-01	Up	0.92	9648	1.32	0.04
rs912879	51429	SNX9	6	158332308	4.83E-02	4871	2.96E-01	Down	1.05	9159	1.32	0.05
rs2883970	27244	SESN1	6	109470427	4.83E-02	4872	3.29E-01	Down	0.98	9429	1.32	0.05
rs1417582	127943	FCRLB	1	158427746	4.83E-02	4873	7.11E-02	Down	1.81	6453	1.32	0.11
rs7104794	51642	MRPL48	11	73216346	4.83E-02	4874	1.30E-02	Up	2.48	4691	1.32	0.19
rs17111079	815	CAMK2A	5	149595967	4.83E-02	4875	2.63E-01	Up	1.12	8890	1.32	0.06
rs10897526	7536	SF1	11	64316474	4.83E-02	4876	2.46E-05	Up	4.22	2126	1.32	0.46
rs11704564	84861	KLHL22	22	19189845	4.83E-02	4877	1.19E-01	Up	1.56	7277	1.32	0.09
rs17296443	8829	NRP1	10	33601580	4.84E-02	4878	7.96E-01	Up	0.26	12646	1.32	0.01
rs556677	3358	HTR2C		113645882	4.84E-02	4879	2.14E-01	Up	1.24	8398	1.31	0.07
rs8102349	2014	EMP3	19	53524366	4.85E-02	4880	3.92E-15	Down	7.86	385	1.31	1.44
rs8102349	55260	TMEM143	19	53524366	4.85E-02	4881	6.05E-01	Up	0.52	11468	1.31	0.02
rs836132	2001	ELF5	11	34511767	4.85E-02	4882	4.13E-02	Up	2.04	5780	1.31	0.14
rs10925503	9453	GGPS1	1	231834504	4.85E-02	4883	9.42E-03	Up	2.60	4463	1.31	0.20
rs10925503	51742	ARID4B	1	231834504	4.85E-02	4884	2.36E-02	Up	2.26	5223	1.31	0.16
rs6009339	9889	ZBED4	22	48594752	4.85E-02	4885	1.23E-03	Up	3.23	3306	1.31	0.29
rs643788	1798	DPAGT1	11	118472968	4.85E-02	4886	2.02E-13	Up	7.31	504	1.31	1.27
rs643788	3014	H2AFX	11	118472968	4.85E-02	4887	9.26E-10	Up	6.12	889	1.31	0.90
rs643788	3145	HMBS	11	118472968	4.85E-02	4888	4.98E-02	Up	1.96	5993	1.31	0.13
rs3923455	64097	EPB41L4A	5	111627022	4.86E-02	4889	9.28E-06	Down	4.43	1930	1.31	0.50
rs7002633	79618	HMBOX1	8	28792835	4.86E-02	4890	5.55E-01	Up	0.59	11118	1.31	0.03
rs12781609	255352	C10orf93	10	134598321	4.86E-02	4891	5.67E-02	Up	1.91	6153	1.31	0.12
rs2277631	55316	RSAD1	17	45894762	4.86E-02	4892	2.28E-03	Up	3.05	3622	1.31	0.26
rs2277631	1101	CHAD	17	45894762	4.86E-02	4893	4.10E-01	Up	0.82	10053	1.31	0.04
rs136478	10739	RFPL2	22	30913577	4.86E-02	4894	4.18E-03	Down	2.86	3963	1.31	0.24
rs9384817	8838	WISP3	6	112471054	4.86E-02	4895	1.94E-01	Down	1.30	8187	1.31	0.07
rs1153942	7159	TP53BP2	1	220277480	4.87E-02	4896	2.00E-02	Up	2.33	5072	1.31	0.17
rs1153942	824	CAPN2	1	220277480	4.87E-02	4897	8.02E-01	Up	0.25	12690	1.31	0.01
rs1040758	8760	CDS2	20	5102912	4.87E-02	4898	9.93E-03	Down	2.58	4505	1.31	0.20
rs433852	6141	RPL18	19	53808916	4.87E-02	4899	8.82E-05	Up	3.92	2421	1.31	0.41
rs433852	56848	SPHK2	19	53808916	4.87E-02	4900	2.36E-03	Up	3.04	3648	1.31	0.26
rs433852	54854	FAM83E	19	53808916	4.87E-02	4901	3.96E-01	Up	0.85	9951	1.31	0.04
rs433852	171169	SPACA4	19	53808916	4.87E-02	4902	9.91E-01	Down	0.01	13851	1.31	0.00
rs7552510	84288	EFCAB2	1	241494079	4.87E-02	4903	6.19E-01	Down	0.50	11571	1.31	0.02
rs6074272	55321	C20orf46	20	1130173	4.87E-02	4904	5.04E-01	Up	0.67	10744	1.31	0.03
rs9493750	6943	TCF21	6	134232660	4.88E-02	4905	1.04E-05	Down	4.41	1957	1.31	0.50
rs1002989	10692	RRH	4	111091446	4.88E-02	4906	4.51E-01	Up	0.75	10350	1.31	0.03
rs2282449	148808	MFSD4	1	202299870	4.88E-02	4907	8.64E-01	Up	0.17	13029	1.31	0.01
rs753381	5335	PLCG1	20	39230879	4.88E-02	4908	4.79E-04	Up	3.49	2949	1.31	0.33
rs2838502	8209	C21orf33	21	44395416	4.88E-02	4909	1.36E-02	Up	2.47	4732	1.31	0.19
rs710106	23641	LDOC1		139995938	4.88E-02	4910	1.89E-02	Down	2.35	5020	1.31	0.17
rs545253	3476	IGBP1		69133302	4.88E-02	4911	2.90E-05	Up	4.18	2163	1.31	0.45
rs10839891	26343	OR5E1P	11	7837104	4.89E-02	4912	2.05E-01	Up	1.27	8309	1.31	0.07
rs10833308	10553	HTATIP2	11	20328897	4.89E-02	4913	6.67E-01	Up	0.43	11868	1.31	0.02
rs9864977	348807	CCDC37	3	127583745	4.89E-02	4914	9.53E-01	Up	0.06	13577	1.31	0.00
rs6963707	221823	PRPS1L1	7	17831767	4.89E-02	4915	7.66E-01	Up	0.30	12468	1.31	0.01
rs391463	9180	OSMR	5	38923537	4.89E-02	4916	3.65E-04	Down	3.56	2850	1.31	0.34
rs11636687	9488	PIGB	15	53392444	4.89E-02	4917	4.88E-01	Up	0.69	10633	1.31	0.03
rs309345	166379	BBS12	4	124002685	4.89E-02	4918	3.62E-01	Down	0.91	9694	1.31	0.04
rs4877950	23287	AGTPBP1	9	85570008	4.90E-02	4919	2.52E-02	Up	2.24	5298	1.31	0.16
rs2303149	7171	TPM4	19	16058421	4.90E-02	4920	4.11E-06	Down	4.61	1785	1.31	0.54
rs10850707	79794	C12orf49	12	115644428	4.90E-02	4921	1.79E-02	Up	2.37	4980	1.31	0.17
rs17041884	2028	ENPEP	4	111821231	4.90E-02	4922	1.50E-03	Up	3.18	3383	1.31	0.28
rs10863438	127018	LYPLAL1	1	215777137	4.90E-02	4923	5.64E-09	Up	5.82	1023	1.31	0.82
rs967785	2710	GK		30433917	4.90E-02	4924	3.64E-01	Down	0.91	9715	1.31	0.04
rs2799359	59084	ENPP5	6	46252211	4.90E-02	4925	6.48E-05	Up	3.99	2346	1.31	0.42
rs11081004	8736	MYOM1	18	3086165	4.90E-02	4926	8.74E-02	Down	1.71	6761	1.31	0.11
rs2136233	23101	MCF2L2	3	184585962	4.91E-02	4927	8.96E-02	Up	1.70	6800	1.31	0.10
rs2586777	83876	MRO	18	46584623	4.91E-02	4928	8.77E-01	Down	0.15	13101	1.31	0.01
rs9311671	1776	DNASE1L3	3	58166473	4.91E-02	4929	9.18E-04	Down	3.31	3182	1.31	0.30
rs6670349	56950	SMYD2	1	210906139	4.91E-02	4930	2.11E-03	Up	3.07	3567	1.31	0.27
rs905589	11266	DUSP12	1	158434856	4.91E-02	4931	6.36E-03	Up	2.73	4198	1.31	0.22
rs7387389	6570	SLC18A1	8	20060734	4.91E-02	4932	6.05E-01	Up	0.52	11467	1.31	0.02
rs16960555	79818	ZNF552	19	63030025	4.91E-02	4933	5.09E-04	Up	3.48	2966	1.31	0.33
rs3828041	26292	MYCBP	1	38999492	4.91E-02	4934	4.05E-02	Up	2.05	5758	1.31	0.14
rs3828041	64121	RRAGC	1	38999492	4.91E-02	4935	7.16E-01	Down	0.36	12167	1.31	0.01
rs3828041	84694	GJA10	1	38999492	4.91E-02	4936	9.89E-01	Up	0.01	13836	1.31	0.00

rs17358518	123720	WHDC1	15	81304431	4.91E-02	4937	6.26E-01	Down	0.49	11621	1.31	0.02
rs11755082	5980	REV3L	6	111930475	4.91E-02	4938	2.64E-09	Down	5.95	955	1.31	0.86
rs4872526	55909	BIN3	8	22551911	4.92E-02	4939	2.89E-03	Down	2.98	3759	1.31	0.25
rs4149965	9156	EXO1	1	238361479	4.92E-02	4940	6.43E-01	Down	0.46	11725	1.31	0.02
rs2952830	51460	SFMBT1	3	52957336	4.92E-02	4941	3.79E-01	Down	0.88	9831	1.31	0.04
rs3777711	51439	FAM8A1	6	17724235	4.92E-02	4942	1.68E-01	Down	1.38	7904	1.31	0.08
rs3777711	9972	NUP153	6	17724235	4.92E-02	4943	6.34E-01	Up	0.48	11666	1.31	0.02
rs12003641	158399	ZNF483	9	111359052	4.93E-02	4944	4.91E-03	Down	2.81	4056	1.31	0.23
rs12003641	23392	KIAA0368	9	111359052	4.93E-02	4945	2.71E-02	Down	2.21	5364	1.31	0.16
rs16963660	126626	GABPB2	15	48442703	4.93E-02	4946	6.60E-08	Up	5.40	1252	1.31	0.72
rs4679909	80117	ARL14	3	161859553	4.94E-02	4947	1.01E-01	Up	1.64	7027	1.31	0.10
rs314756	4041	LRP5	11	67868248	4.94E-02	4948	1.18E-01	Up	1.56	7269	1.31	0.09
rs528472	57582	KCNT1	9	135806792	4.95E-02	4949	8.67E-01	Down	0.17	13049	1.31	0.01
rs2001966	157769	FAM91A1	8	124898576	4.95E-02	4950	4.00E-04	Up	3.54	2883	1.31	0.34
rs9371127	55780	C6orf70	6	170002095	4.95E-02	4951	2.44E-02	Up	2.25	5267	1.31	0.16
rs9371127	80069	C6orf208	6	170002095	4.95E-02	4952	7.60E-01	Up	0.30	12442	1.31	0.01
rs7793131	6100	RP9	7	32900779	4.95E-02	4953	2.80E-01	Up	1.08	9032	1.31	0.06
rs17226479	5787	PTPRB	12	69302773	4.95E-02	4954	4.98E-01	Down	0.68	10698	1.31	0.03
rs1054473	11222	MRPL3	3	132722364	4.96E-02	4955	8.76E-07	Up	4.92	1561	1.30	0.61
rs5909978	2892	GRIA3		122116348	4.96E-02	4956	5.14E-01	Down	0.65	10814	1.30	0.03
rs7375794	419	ART3	4	77370370	4.96E-02	4957	8.56E-01	Up	0.18	12987	1.30	0.01
rs2331886	9213	XPR1	1	177464780	4.96E-02	4958	2.53E-01	Up	1.14	8788	1.30	0.06
rs12125204	2035	EPB41	1	29215311	4.96E-02	4959	1.92E-10	Up	6.36	793	1.30	0.97
rs275137	23432	GPR161	1	164782924	4.97E-02	4960	2.94E-12	Down	6.98	603	1.30	1.15
rs275137	55827	IQWD1	1	164782924	4.97E-02	4961	3.81E-06	Up	4.62	1775	1.30	0.54
rs90192	6876	TAGLN	11	116564557	4.97E-02	4962	5.32E-26	Down	10.55	100	1.30	2.53
rs921970	25953	PNKD	2	219035301	4.97E-02	4963	4.13E-02	Up	2.04	5779	1.30	0.14
rs2336880	51523	CXXC5	5	139043912	4.97E-02	4964	1.78E-07	Up	5.22	1361	1.30	0.67
rs11020998	51503	CWC15	11	94361182	4.98E-02	4965	2.93E-02	Up	2.18	5438	1.30	0.15
rs10500214	7639	ZNF85	19	20914290	4.98E-02	4966	6.96E-06	Up	4.50	1872	1.30	0.52
rs228779	84336	TMEM101	17	39446739	4.98E-02	4967	1.98E-01	Down	1.29	8234	1.30	0.07
rs228779	162417	NAGS	17	39446739	4.98E-02	4968	9.15E-01	Down	0.11	13350	1.30	0.00
rs3817614	4798	NFRKB	11	129240887	4.98E-02	4969	1.88E-01	Down	1.32	8122	1.30	0.07
rs2072496	3718	JAK3	19	17807054	4.98E-02	4970	3.13E-02	Down	2.15	5508	1.30	0.15
rs2072496	3640	INSL3	19	17807054	4.98E-02	4971	3.67E-01	Down	0.90	9740	1.30	0.04
rs11825598	220047	CCDC83	11	85312382	4.98E-02	4972	3.69E-02	Up	2.09	5676	1.30	0.14
rs4806163	93099	DMKN	19	40695946	4.98E-02	4973	7.89E-21	Down	9.36	178	1.30	2.01
rs17649232	151242	PPP1R1C	2	182830244	4.99E-02	4974	7.52E-01	Up	0.32	12389	1.30	0.01
rs7632449	27107	ZBTB11	3	102871883	4.99E-02	4975	1.81E-01	Up	1.34	8036	1.30	0.07
rs13178564	56929	FEM1C	5	114922007	4.99E-02	4976	7.97E-01	Up	0.26	12652	1.30	0.01
rs11126836	60509	AGBL5	2	27184779	5.00E-02	4977	8.42E-01	Up	0.20	12912	1.30	0.01
rs741886	2495	FTH1	11	61481363	5.00E-02	4978	2.68E-01	Down	1.11	8934	1.30	0.06
rs8075200	124925	SEZ6	17	24370680	5.00E-02	4979	7.48E-01	Up	0.32	12363	1.30	0.01
rs12418815	4316	MMP7	11	101923370	5.01E-02	4980	7.43E-02	Down	1.78	6515	1.30	0.11
rs10973537	51010	EXOSC3	9	37764893	5.01E-02	4981	1.15E-01	Up	1.58	7215	1.30	0.09
rs10973537	158234	RG9MTD3	9	37764893	5.01E-02	4982	1.66E-01	Down	1.38	7880	1.30	0.08
rs2286670	50488	MINK1	17	4664993	5.01E-02	4983	8.32E-02	Up	1.73	6688	1.30	0.11
rs6574349	64207	C14orf4	14	76567419	5.02E-02	4984	7.59E-11	Up	6.51	740	1.30	1.01
rs10483611	283554	GPR137C	14	52103281	5.02E-02	4985	1.85E-01	Down	1.33	8082	1.30	0.07
rs2960769	5646	PRSS3	7	141441870	5.02E-02	4986	6.21E-02	Up	1.87	6281	1.30	0.12
rs2960769	5645	PRSS2	7	141441870	5.02E-02	4987	4.27E-01	Up	0.79	10168	1.30	0.04
rs10853751	56915	EXOSC5	19	46595060	5.02E-02	4988	6.96E-04	Up	3.39	3073	1.30	0.32
rs16893963	64105	CENPK	5	64894071	5.02E-02	4989	5.17E-02	Up	1.95	6033	1.30	0.13
rs10993269	2203	FBP1	9	94468508	5.02E-02	4990	1.83E-12	Up	7.05	578	1.30	1.17
rs9864104	59343	SENP2	3	186840233	5.03E-02	4991	3.96E-02	Down	2.06	5738	1.30	0.14
rs10502172	54970	TTC12	11	112704356	5.03E-02	4992	4.77E-01	Up	0.71	10551	1.30	0.03
rs335136	55339	WDR33	2	128283985	5.03E-02	4993	4.84E-01	Up	0.70	10603	1.30	0.03
rs2819358	1999	ELF3	1	198708059	5.03E-02	4994	4.65E-02	Up	1.99	5913	1.30	0.13
rs2295689	64403	CDH24	14	22588631	5.03E-02	4995	2.85E-01	Down	1.07	9076	1.30	0.05
rs2295689	22985	ACIN1	14	22588631	5.03E-02	4996	9.12E-01	Down	0.11	13320	1.30	0.00
rs1489578	8821	INPP4B	4	143688658	5.03E-02	4997	7.07E-01	Up	0.38	12117	1.30	0.02
rs703343	80217	C10orf79	10	105979654	5.03E-02	4998	8.67E-01	Up	0.17	13041	1.30	0.01
rs7220988	4137	MAPT	17	41465321	5.04E-02	4999	1.34E-01	Up	1.50	7490	1.30	0.09
rs11640796	8912	CACNA1H	16	1165629	5.04E-02	5000	1.86E-02	Down	2.35	5006	1.30	0.17
rs17116203	2534	FYN	10	105721400	5.04E-02	5001	2.87E-06	Down	4.68	1727	1.30	0.55
rs17116203	9748	SLK	10	105721400	5.04E-02	5002	7.56E-03	Down	2.67	4309	1.30	0.21
rs3829571	84923	FAM104A	17	68713270	5.05E-02	5003	5.64E-01	Up	0.58	11194	1.30	0.02
rs3829571	9382	COG1	17	68713270	5.05E-02	5004	8.21E-01	Down	0.23	12797	1.30	0.01
rs3790567	3595	IL12RB2	1	67534398	5.05E-02	5005	4.86E-01	Up	0.70	10624	1.30	0.03
rs10897261	80150	ASRGL1	11	61874285	5.05E-02	5006	8.27E-01	Up	0.22	12833	1.30	0.01
rs2228946	7472	WNT2	7	116512036	5.05E-02	5007	6.06E-07	Down	4.99	1504	1.30	0.62
rs934937	388115	C15orf52	15	38401838	5.05E-02	5008	2.37E-01	Down	1.18	8634	1.30	0.06
rs934937	5330	PLCB2	15	38401838	5.05E-02	5009	6.85E-01	Up	0.41	11978	1.30	0.02
rs12061601	481	ATP1B1	1	165802108	5.05E-02	5010	1.57E-05	Down	4.32	2044	1.30	0.48
rs3850749	5108	PCM1	8	17895105	5.06E-02	5011	1.55E-03	Down	3.17	3400	1.30	0.28
rs11629789	57099	AVEN	15	32059505	5.06E-02	5012	1.90E-06	Up	4.76	1659	1.30	0.57
rs17701982	51091	SEPSECS	8	134153349	5.06E-02	5013	1.71E-03	Up	3.14	3448	1.30	0.28
rs17701982	6503	SLA	8	134153349	5.06E-02	5014	7.43E-01	Down	0.33	12334	1.30	0.01
rs7727648	81555	YIPF5	5	143548459	5.06E-02	5015	7.96E-02	Up	1.75	6624	1.30	0.11
rs2808667	4686	NCBP1	9	97522361	5.07E-02	5016	8.11E-05	Up	3.94	2396	1.30	0.41
rs2808667	7507	XPA	9	97522361	5.07E-02	5017	5.92E-02	Down	1.89	6219	1.30	0.12

rs9512551	219333	USP12	13	26603697	5.07E-02	5018	6.33E-02	Down	1.86	6305	1.30	0.12
rs1022920	266553	OFCC1	6	10052943	5.07E-02	5019	2.15E-01	Up	1.24	8407	1.29	0.07
rs4790441	8390	OR1G1	17	2996430	5.07E-02	5020	2.05E-02	Down	2.32	5094	1.29	0.17
rs833836	8085	MLL2	12	47737726	5.07E-02	5021	1.60E-02	Up	2.41	4889	1.29	0.18
rs833836	121268	RHEBL1	12	47737726	5.07E-02	5022	9.50E-01	Down	0.06	13556	1.29	0.00
rs874060	4705	NDUFA10	2	240691440	5.08E-02	5023	7.30E-04	Up	3.38	3093	1.29	0.31
rs2296376	9917	FAM20B	1	175786677	5.08E-02	5024	2.92E-01	Down	1.05	9133	1.29	0.05
rs2296376	64222	TOR3A	1	175786677	5.08E-02	5025	8.33E-01	Up	0.21	12866	1.29	0.01
rs12459350	84444	DOT1L	19	2127586	5.08E-02	5026	1.21E-01	Down	1.55	7305	1.29	0.09
rs13254844	4747	NEFL	8	24874430	5.09E-02	5027	6.52E-04	Down	3.41	3055	1.29	0.32
rs11595408	50624	CUZD1	10	124597815	5.09E-02	5028	6.02E-01	Down	0.52	11442	1.29	0.02
rs11595408	196792	FAM24B	10	124597815	5.09E-02	5029	8.84E-01	Up	0.15	13151	1.29	0.01
rs11056432	10076	PTPRU	12	15389172	5.09E-02	5030	1.67E-02	Down	2.39	4921	1.29	0.18
rs11056432	5800	PTPRO	12	15389172	5.09E-02	5031	1.84E-01	Up	1.33	8061	1.29	0.07
rs1562722	4772	NFATC1	18	75288153	5.10E-02	5032	2.89E-01	Down	1.06	9106	1.29	0.05
rs679602	55223	TRIM62	1	33313737	5.10E-02	5033	1.81E-01	Up	1.34	8040	1.29	0.07
rs6439361	84100	ARL6	3	98955246	5.10E-02	5034	4.34E-01	Up	0.78	10222	1.29	0.04
rs3829126	84647	PLA2G12B	10	74384183	5.10E-02	5035	9.39E-01	Down	0.08	13484	1.29	0.00
rs1522780	161742	SPRED1	15	36323341	5.10E-02	5036	1.25E-05	Down	4.37	2003	1.29	0.49
rs7502869	9146	HGS	17	77241821	5.10E-02	5037	8.09E-02	Up	1.75	6646	1.29	0.11
rs7502869	339230	CCDC137	17	77241821	5.10E-02	5038	2.12E-01	Down	1.25	8373	1.29	0.07
rs7502869	5148	PDE6G	17	77241821	5.10E-02	5039	6.78E-01	Up	0.42	11929	1.29	0.02
rs9257936	4340	MOG	6	29747755	5.10E-02	5040	3.29E-01	Up	0.98	9432	1.29	0.05
rs6660890	127703	C1orf216	1	35869914	5.10E-02	5041	2.53E-01	Up	1.14	8787	1.29	0.06
rs6660890	63967	CLSPN	1	35869914	5.10E-02	5042	8.99E-01	Down	0.13	13237	1.29	0.00
rs1646025	5619	PRM1	16	11287439	5.10E-02	5043	3.45E-01	Down	0.94	9557	1.29	0.05
rs1646025	7142	TNP2	16	11287439	5.10E-02	5044	3.75E-01	Up	0.89	9802	1.29	0.04
rs1646025	5620	PRM2	16	11287439	5.10E-02	5045	4.80E-01	Up	0.71	10574	1.29	0.03
rs235343	6612	SUMO3	21	45074180	5.11E-02	5046	1.81E-01	Up	1.34	8032	1.29	0.07
rs7863476	9373	PLAA	9	26905925	5.11E-02	5047	5.26E-02	Up	1.94	6054	1.29	0.13
rs9033	55336	FBXL8	16	65739500	5.11E-02	5048	5.71E-03	Up	2.76	4152	1.29	0.22
rs9033	3299	HSF4	16	65739500	5.11E-02	5049	1.55E-02	Up	2.42	4840	1.29	0.18
rs9033	8717	TRADD	16	65739500	5.11E-02	5050	4.27E-01	Down	0.79	10167	1.29	0.04
rs2965245	317701	VN1R2	19	58449758	5.11E-02	5051	7.93E-01	Down	0.26	12631	1.29	0.01
rs7722456	2568	GABRP	5	170135562	5.11E-02	5052	5.62E-02	Down	1.91	6142	1.29	0.13
rs1545696	6643	SNX2	5	122130341	5.11E-02	5053	1.17E-05	Down	4.38	1981	1.29	0.49
rs8090480	8932	MBD2	18	49941278	5.11E-02	5054	6.23E-01	Up	0.49	11607	1.29	0.02
rs6600126	23430	TPSD1	16	1253383	5.11E-02	5055	5.33E-01	Up	0.62	10964	1.29	0.03
rs10878400	92797	HELB	12	64968116	5.12E-02	5056	4.83E-01	Up	0.70	10600	1.29	0.03
rs283851	5967	REG1A	2	79243738	5.12E-02	5057	8.19E-01	Up	0.23	12780	1.29	0.01
rs6927483	221395	GPR116	6	46997536	5.13E-02	5058	1.12E-07	Up	5.31	1305	1.29	0.70
rs1108723	57143	ADCK1	14	77400981	5.13E-02	5059	5.89E-03	Up	2.75	4164	1.29	0.22
rs1348	221718	C6orf218	6	10557244	5.13E-02	5060	9.63E-01	Down	0.05	13636	1.29	0.00
rs731450	285489	DOK7	4	3527297	5.13E-02	5061	9.17E-01	Down	0.10	13359	1.29	0.00
rs3745405	5582	PRKCG	19	59086910	5.13E-02	5062	6.45E-02	Up	1.85	6335	1.29	0.12
rs11026529	57084	SLC17A6	11	22334633	5.13E-02	5063	1.21E-01	Up	1.55	7309	1.29	0.09
rs12594823	23102	TBC1D2B	15	76102552	5.14E-02	5064	9.68E-02	Down	1.66	6939	1.29	0.10
rs307805	2324	FLT4	5	180010093	5.14E-02	5065	8.33E-01	Down	0.21	12863	1.29	0.01
rs9410486	10507	SEMA4D	9	89299225	5.15E-02	5066	7.63E-02	Up	1.77	6557	1.29	0.11
rs2236324	80017	C14orf159	14	90769171	5.15E-02	5067	1.51E-09	Down	6.04	922	1.29	0.88
rs27795	123970	C16orf78	16	47987730	5.15E-02	5068	1.49E-01	Up	1.44	7675	1.29	0.08
rs7790357	140545	RNF32	7	155963690	5.15E-02	5069	2.96E-09	Up	5.93	966	1.29	0.85
rs10832020	406	ARNTL	11	13277919	5.16E-02	5070	7.55E-03	Down	2.67	4306	1.29	0.21
rs3751836	197358	NLRC3	16	3522970	5.17E-02	5071	1.99E-01	Down	1.28	8247	1.29	0.07
rs3751836	23059	CLUAP1	16	3522970	5.17E-02	5072	3.41E-01	Down	0.95	9531	1.29	0.05
rs6065998	55713	ZNF334	20	44560642	5.17E-02	5073	3.24E-01	Down	0.99	9396	1.29	0.05
rs975886	53918	PELO	5	52136984	5.18E-02	5074	1.69E-05	Down	4.30	2058	1.29	0.48
rs1613152	84912	SLC35B4	7	133477092	5.18E-02	5075	2.03E-04	Up	3.72	2636	1.29	0.37
rs2305084	146059	CDAN1	15	40805983	5.18E-02	5076	1.68E-01	Down	1.38	7900	1.29	0.08
rs6500596	9093	DNAJA3	16	4410028	5.18E-02	5077	6.94E-03	Up	2.70	4256	1.29	0.22
rs6500596	79585	CORO7	16	4410028	5.18E-02	5078	9.51E-03	Up	2.59	4472	1.29	0.20
rs11692435	10120	ACTR1B	2	97733872	5.18E-02	5079	7.84E-01	Up	0.27	12570	1.29	0.01
rs4772260	7546	ZIC2	13	99443724	5.18E-02	5080	2.90E-11	Up	6.66	684	1.29	1.05
rs10838559	51317	PHF21A	11	46059097	5.19E-02	5081	1.30E-01	Down	1.51	7447	1.28	0.09
rs11771421	5001	ORC5L	7	103430083	5.19E-02	5082	1.94E-11	Up	6.71	673	1.28	1.07
rs7307400	84678	FBXL10	12	120388188	5.19E-02	5083	4.26E-01	Up	0.80	10165	1.28	0.04
rs11084543	201514	ZNF584	19	63624513	5.20E-02	5084	9.26E-01	Up	0.09	13404	1.28	0.00
rs3103074	27201	GPR78	4	8680739	5.20E-02	5085	7.63E-01	Up	0.30	12454	1.28	0.01
rs1744760	140699	C20orf132	20	35174208	5.20E-02	5086	4.72E-02	Down	1.98	5927	1.28	0.13
rs2166898	2736	GLI2	2	121328889	5.20E-02	5087	1.03E-02	Down	2.57	4532	1.28	0.20
rs1125036	9946	CRYZL1	21	33953096	5.20E-02	5088	2.64E-06	Down	4.70	1713	1.28	0.56
rs10845677	339	APOBEC1	12	7717572	5.20E-02	5089	5.66E-01	Up	0.57	11217	1.28	0.02
rs6438865	57493	HEG1	3	126187801	5.21E-02	5090	1.15E-02	Down	2.53	4610	1.28	0.19
rs2300494	3612	IMPA1	8	82741285	5.21E-02	5091	1.46E-06	Up	4.82	1624	1.28	0.58
rs7101470	79096	C11orf49	11	47011024	5.22E-02	5092	4.66E-01	Down	0.73	10468	1.28	0.03
rs3826795	64344	HIF3A	19	51492273	5.22E-02	5093	1.04E-01	Down	1.63	7054	1.28	0.10
rs1091811	3984	LIMK1	7	72935863	5.22E-02	5094	7.00E-01	Up	0.39	12071	1.28	0.02
rs1091811	2006	ELN	7	72935863	5.22E-02	5095	8.16E-01	Down	0.23	12763	1.28	0.01
rs9984245	64968	MRPS6	21	34399933	5.22E-02	5096	4.45E-02	Down	2.01	5865	1.28	0.14
rs9984245	6526	SLC5A3	21	34399933	5.22E-02	5097	9.67E-01	Down	0.04	13674	1.28	0.00
rs13120122	132851	SPATA4	4	177509432	5.22E-02	5098	1.13E-03	Up	3.26	3265	1.28	0.29

rs550276	9538	EI24	11	124951280	5.22E-02	5099	2.72E-09	Up	5.95	956	1.28	0.86
rs893629	7097	TLR2	4	154962573	5.22E-02	5100	9.69E-01	Up	0.04	13694	1.28	0.00
rs4923886	6692	SPINT1	15	38946898	5.23E-02	5101	2.79E-06	Up	4.69	1722	1.28	0.56
rs7554827	5301	PIN1L	1	70098484	5.23E-02	5102	1.48E-01	Up	1.45	7650	1.28	0.08
rs7554827	57554	LRRC7	1	70098484	5.23E-02	5103	3.31E-01	Up	0.97	9457	1.28	0.05
rs12615509	56896	DPYSL5	2	27010417	5.23E-02	5104	7.70E-01	Up	0.29	12491	1.28	0.01
rs2706460	25820	ARIH1	15	70535782	5.23E-02	5105	9.78E-01	Down	0.03	13751	1.28	0.00
rs628629	5238	PGM3	6	83951101	5.23E-02	5106	1.62E-03	Up	3.15	3425	1.28	0.28
rs17013609	6696	SPP1	4	89239274	5.24E-02	5107	8.09E-05	Up	3.94	2394	1.28	0.41
rs17229459	1859	DYRK1A	21	37813457	5.24E-02	5108	4.62E-01	Down	0.74	10442	1.28	0.03
rs586224	9348	NDST3	4	119521387	5.24E-02	5109	7.33E-01	Down	0.34	12275	1.28	0.01
rs2957154	4221	MEN1	11	64341563	5.25E-02	5110	4.88E-09	Up	5.85	1009	1.28	0.83
rs2957154	55561	CDC42BPG	11	64341563	5.25E-02	5111	8.82E-01	Down	0.15	13142	1.28	0.01
rs9927298	29108	PYCARD	16	31139938	5.25E-02	5112	1.42E-02	Down	2.45	4764	1.28	0.18
rs3811876	9627	SNCAIP	5	121810410	5.25E-02	5113	2.13E-08	Down	5.60	1131	1.28	0.77
rs13706	990	CDC6	17	35710677	5.25E-02	5114	6.45E-06	Up	4.51	1861	1.28	0.52
rs8027997	197021	LCTL	15	64654601	5.25E-02	5115	3.57E-01	Down	0.92	9652	1.28	0.04
rs16970648	27033	ZBTB32	19	40909173	5.25E-02	5116	3.85E-01	Up	0.87	9877	1.28	0.04
rs16970648	55851	PSENEN	19	40909173	5.25E-02	5117	9.73E-01	Up	0.03	13719	1.28	0.00
rs11617480	54737	MPHOSPH8	13	19109434	5.26E-02	5118	2.01E-01	Down	1.28	8265	1.28	0.07
rs17256082	79634	SCRN3	2	175117871	5.26E-02	5119	1.27E-01	Up	1.53	7406	1.28	0.09
rs17256082	151556	GPR155	2	175117871	5.26E-02	5120	2.48E-01	Up	1.15	8750	1.28	0.06
rs615020	164045	HFM1	1	91429000	5.26E-02	5121	2.28E-01	Up	1.21	8536	1.28	0.06
rs2306536	2802	GOLGA3	12	132034012	5.26E-02	5122	3.46E-09	Up	5.92	979	1.28	0.85
rs7143254	145581	LRFN5	14	41241324	5.27E-02	5123	8.46E-02	Down	1.72	6713	1.28	0.11
rs6588219	200132	TCTEX1D1	1	66956624	5.27E-02	5124	9.68E-01	Down	0.04	13684	1.28	0.00
rs8101605	10859	LILRB1	19	59840299	5.27E-02	5125	9.01E-02	Up	1.70	6815	1.28	0.10
rs1362965	25840	METTL7A	12	49608261	5.28E-02	5126	1.92E-25	Down	10.42	107	1.28	2.47
rs17340112	81567	TXNDC5	6	7904722	5.28E-02	5127	2.10E-05	Up	4.25	2100	1.28	0.47
rs17432234	4528	MTIF2	2	55385677	5.28E-02	5128	1.25E-06	Up	4.85	1604	1.28	0.59
rs10494757	163486	DENND1B	1	194239068	5.28E-02	5129	2.05E-01	Up	1.27	8302	1.28	0.07
rs12755035	5590	PRKCZ	1	2058523	5.28E-02	5130	7.33E-03	Up	2.68	4287	1.28	0.21
rs2297185	57710	KIAA1614	1	177650912	5.28E-02	5131	9.38E-01	Up	0.08	13477	1.28	0.00
rs4665145	22925	PLA2R1	2	160733376	5.28E-02	5132	1.27E-05	Up	4.37	2005	1.28	0.49
rs10485058	4988	OPRM1	6	154537328	5.28E-02	5133	9.68E-01	Up	0.04	13687	1.28	0.00
rs1548915	29117	BRD7	16	48961028	5.29E-02	5134	9.92E-01	Down	0.01	13853	1.28	0.00
rs4374421	3973	LHCGR	2	48847043	5.29E-02	5135	1.33E-01	Up	1.50	7478	1.28	0.09
rs4374421	11036	GTF2A1L	2	48847043	5.29E-02	5136	8.82E-01	Up	0.15	13143	1.28	0.01
rs1882449	56660	KCNK12	2	47696643	5.29E-02	5137	9.53E-01	Down	0.06	13581	1.28	0.00
rs6972704	6654	SOS1	7	81057988	5.29E-02	5138	1.29E-02	Down	2.49	4678	1.28	0.19
rs6972704	3569	IL6	7	81057988	5.29E-02	5139	6.40E-01	Up	0.47	11709	1.28	0.02
rs6972704	3082	HGF	7	81057988	5.29E-02	5140	8.44E-01	Up	0.20	12927	1.28	0.01
rs7877664	1654	DDX3X		40967196	5.30E-02	5141	4.12E-03	Down	2.87	3953	1.28	0.24
rs7208074	10241	CALCOCO2	17	44249072	5.30E-02	5142	2.53E-01	Down	1.14	8790	1.28	0.06
rs7154721	9321	TRIP11	14	91497101	5.30E-02	5143	1.19E-03	Up	3.24	3286	1.28	0.29
rs12441054	22856	CHSY1	15	99584690	5.30E-02	5144	3.12E-01	Up	1.01	9290	1.28	0.05
rs8102394	10055	SAE1	19	52367930	5.30E-02	5145	1.39E-01	Down	1.48	7560	1.28	0.09
rs309226	54495	TXNDC10	18	64510601	5.30E-02	5146	1.15E-01	Up	1.58	7211	1.28	0.09
rs10861666	55188	RIC8B	12	105697047	5.30E-02	5147	7.94E-01	Up	0.26	12633	1.28	0.01
rs315280	5304	PIP	7	142358796	5.30E-02	5148	7.48E-01	Up	0.32	12364	1.28	0.01
rs6989560	9897	KIAA0196	8	126111223	5.30E-02	5149	1.54E-02	Up	2.42	4838	1.28	0.18
rs1391174	2565	GABRG1	4	45913524	5.31E-02	5150	2.21E-01	Up	1.22	8467	1.28	0.07
rs2900	7681	MKRN3	15	21368814	5.31E-02	5151	4.46E-01	Down	0.76	10318	1.28	0.04
rs11774673	66036	MTMR9	8	11228947	5.31E-02	5152	1.35E-08	Down	5.68	1081	1.27	0.79
rs10802546	3964	LGALS8	1	233008155	5.31E-02	5153	2.72E-06	Up	4.69	1718	1.27	0.56
rs7888694	1964	EIF1AX		19895163	5.31E-02	5154	2.24E-03	Up	3.06	3607	1.27	0.27
rs2304803	57559	STAMBPL1	10	90651678	5.32E-02	5155	5.44E-05	Up	4.04	2308	1.27	0.43
rs893263	79190	IRX6	16	53907338	5.32E-02	5156	2.38E-01	Down	1.18	8639	1.27	0.06
rs2283797	8940	TOP3B	22	20639866	5.32E-02	5157	4.70E-02	Up	1.99	5922	1.27	0.13
rs2073601	6493	SIM2	21	37039178	5.32E-02	5158	1.49E-28	Up	11.02	77	1.27	2.78
rs16823374	140625	ACTRT2	1	2960469	5.32E-02	5159	1.28E-02	Down	2.49	4673	1.27	0.19
rs6501741	124590	USH1G	17	70438718	5.32E-02	5160	3.29E-01	Up	0.98	9434	1.27	0.05
rs6501741	92736	OTOP2	17	70438718	5.32E-02	5161	5.79E-01	Down	0.55	11306	1.27	0.02
rs12971679	81576	CCDC130	19	13742056	5.32E-02	5162	5.94E-02	Down	1.89	6224	1.27	0.12
rs6010061	85358	SHANK3	22	49441868	5.32E-02	5163	7.82E-01	Up	0.28	12556	1.27	0.01
rs6512050	4051	CYP4F3	19	15634915	5.32E-02	5164	9.91E-01	Up	0.01	13845	1.27	0.00
rs7173030	4091	SMAD6	15	64843625	5.32E-02	5165	5.76E-02	Down	1.90	6176	1.27	0.12
rs6547631	10578	GNLY	2	85820684	5.33E-02	5166	9.88E-01	Up	0.02	13831	1.27	0.00
rs10280585	84132	USP42	7	5945895	5.33E-02	5167	1.34E-07	Up	5.27	1325	1.27	0.69
rs7145649	10538	BATF	14	75088537	5.33E-02	5168	5.85E-04	Down	3.44	3018	1.27	0.32
rs11006995	143098	MPP7	10	28625502	5.33E-02	5169	9.33E-01	Up	0.08	13451	1.27	0.00
rs4985855	3768	KCNJ12	17	21243600	5.34E-02	5170	7.76E-01	Down	0.28	12537	1.27	0.01
rs602735	79607	FAM118B	11	125585562	5.34E-02	5171	2.59E-02	Up	2.23	5318	1.27	0.16
rs12724106	360023	ZBTB41	1	193859235	5.34E-02	5172	5.08E-03	Up	2.80	4078	1.27	0.23
rs12724106	259266	ASPM	1	193859235	5.34E-02	5173	1.90E-01	Up	1.31	8148	1.27	0.07
rs4282275	8988	HSPB3	5	53783064	5.34E-02	5174	6.77E-05	Down	3.98	2355	1.27	0.42
rs2720162	51719	CAB39	2	231454865	5.34E-02	5175	3.21E-01	Up	0.99	9366	1.27	0.05
rs4788114	83985	SPNS1	16	28902646	5.34E-02	5176	1.84E-01	Up	1.33	8068	1.27	0.07
rs4788114	27040	LAT	16	28902646	5.34E-02	5177	3.21E-01	Down	0.99	9370	1.27	0.05
rs6465375	55610	CCDC132	7	92534399	5.34E-02	5178	5.16E-01	Up	0.65	10831	1.27	0.03
rs2520237	254158	CXorf58		23679171	5.35E-02	5179	5.53E-01	Down	0.59	11100	1.27	0.03

rs2881579	57542	KLHDC5	12	27813235	5.35E-02	5180	1.12E-05	Down	4.39	1973	1.27	0.50
rs953741	80108	ZFP2	5	178262452	5.35E-02	5181	6.09E-01	Down	0.51	11493	1.27	0.02
rs932185	51100	SH3GLB1	1	86886548	5.35E-02	5182	8.68E-10	Down	6.13	882	1.27	0.91
rs12154725	155435	RBM33	7	155015927	5.36E-02	5183	8.02E-02	Up	1.75	6633	1.27	0.11
rs1332669	2165	F13B	1	193737540	5.36E-02	5184	2.93E-01	Up	1.05	9134	1.27	0.05
rs352163	54106	TLR9	3	52222150	5.36E-02	5185	3.56E-02	Up	2.10	5635	1.27	0.14
rs1274958	199223	TTC21A	3	39159963	5.36E-02	5186	9.31E-03	Up	2.60	4453	1.27	0.20
rs1274958	64651	AXUD1	3	39159963	5.36E-02	5187	3.66E-01	Down	0.90	9725	1.27	0.04
rs2496278	10771	ZMYND11	10	269248	5.36E-02	5188	5.04E-03	Down	2.80	4075	1.27	0.23
rs12138111	83442	SH3BGRL3	1	26274574	5.37E-02	5189	1.37E-03	Up	3.20	3340	1.27	0.29
rs7908760	3988	LIPA	10	90967976	5.37E-02	5190	2.67E-03	Up	3.00	3723	1.27	0.26
rs7908760	9023	CH25H	10	90967976	5.37E-02	5191	6.23E-01	Down	0.49	11608	1.27	0.02
rs11166101	51375	SNX7	1	98926639	5.37E-02	5192	2.74E-08	Down	5.56	1156	1.27	0.76
rs535960	219970	GLYATL2	11	58383264	5.37E-02	5193	9.41E-01	Up	0.07	13499	1.27	0.00
rs11807878	607	BCL9	1	144320155	5.37E-02	5194	1.10E-08	Up	5.70	1071	1.27	0.80
rs4632148	100132341	C16orf67	16	31614758	5.38E-02	5195	6.11E-02	Down	1.87	6258	1.27	0.12
rs4938099	57569	ARHGAP20	11	110004898	5.38E-02	5196	4.09E-02	Down	2.04	5770	1.27	0.14
rs2256682	346689	KLRG2	7	138631679	5.38E-02	5197	8.11E-02	Up	1.74	6650	1.27	0.11
rs1278607	90462	ZNF605	12	132100813	5.38E-02	5198	2.24E-06	Up	4.73	1684	1.27	0.57
rs4758684	22877	MLXIP	12	121152251	5.39E-02	5199	3.34E-01	Down	0.97	9480	1.27	0.05
rs745338	83464	APH1B	15	61385483	5.39E-02	5200	2.11E-01	Up	1.25	8367	1.27	0.07
rs3768286	6894	TARBP1	1	230888626	5.39E-02	5201	9.23E-13	Up	7.15	552	1.27	1.20
rs7622497	57406	ABHD6	3	58257713	5.39E-02	5202	5.01E-08	Down	5.45	1223	1.27	0.73
rs7622497	11102	RPP14	3	58257713	5.39E-02	5203	2.76E-01	Down	1.09	9003	1.27	0.06
rs2295686	54930	C14orf94	14	22486491	5.39E-02	5204	3.81E-01	Up	0.88	9841	1.27	0.04
rs17146253	8976	WASL	7	122937898	5.39E-02	5205	1.29E-02	Up	2.49	4681	1.27	0.19
rs9835081	256076	COL29A1	3	131642694	5.39E-02	5206	7.87E-01	Up	0.27	12590	1.27	0.01
rs2237659	26959	HBP1	7	106441443	5.39E-02	5207	3.11E-01	Up	1.01	9282	1.27	0.05
rs297924	23017	FAIM2	12	48571328	5.39E-02	5208	2.50E-02	Down	2.24	5284	1.27	0.16
rs2161829	51141	INSIG2	2	118573144	5.40E-02	5209	2.33E-02	Up	2.27	5217	1.27	0.16
rs10470697	2317	FLNB	3	58140273	5.41E-02	5210	3.15E-01	Down	1.00	9326	1.27	0.05
rs1047652	84269	CHCHD5	2	113062743	5.41E-02	5211	4.57E-01	Up	0.74	10401	1.27	0.03
rs3776150	64979	MRPL36	5	1856901	5.41E-02	5212	9.49E-02	Up	1.67	6907	1.27	0.10
rs3776150	4726	NDUFS6	5	1856901	5.41E-02	5213	2.88E-01	Up	1.06	9097	1.27	0.05
rs6436417	7857	SCG2	2	224312106	5.41E-02	5214	2.21E-01	Down	1.22	8474	1.27	0.07
rs2711040	8502	PKP4	2	159360396	5.41E-02	5215	3.05E-01	Up	1.03	9230	1.27	0.05
rs5746154	5894	RAF1	3	12680048	5.41E-02	5216	9.69E-03	Down	2.59	4488	1.27	0.20
rs12427363	64211	LHX5	12	112390929	5.41E-02	5217	1.06E-01	Up	1.62	7089	1.27	0.10
rs12430699	1638	DCT	13	93949763	5.42E-02	5218	2.39E-01	Down	1.18	8646	1.27	0.06
rs3790623	55143	CDCA8	1	37838671	5.42E-02	5219	2.27E-04	Up	3.69	2673	1.27	0.36
rs3790623	54955	C1orf109	1	37838671	5.42E-02	5220	6.15E-02	Up	1.87	6267	1.27	0.12
rs10836063	7916	BAT2	11	33432407	5.42E-02	5221	7.46E-03	Up	2.68	4300	1.27	0.21
rs10836063	25758	C11orf41	11	33432407	5.42E-02	5222	2.28E-01	Up	1.21	8537	1.27	0.06
rs12622681	343990	C2orf55	2	98920632	5.42E-02	5223	2.82E-01	Up	1.08	9052	1.27	0.05
rs13042073	54498	SMOX	20	4120258	5.42E-02	5224	7.66E-12	Up	6.84	633	1.27	1.11
rs9595373	220082	SPERT	13	45195872	5.42E-02	5225	6.95E-01	Up	0.39	12039	1.27	0.02
rs6715941	25992	SNED1	2	241713017	5.43E-02	5226	2.95E-03	Down	2.97	3769	1.27	0.25
rs9996597	255403	ZNF718	4	111652	5.43E-02	5227	1.03E-05	Up	4.41	1955	1.27	0.50
rs1874022	23536	ADAT1	16	74212550	5.43E-02	5228	3.39E-01	Up	0.96	9512	1.26	0.05
rs1874022	3735	KARS	16	74212550	5.43E-02	5229	7.08E-01	Down	0.37	12120	1.26	0.01
rs12947270	113235	SLC46A1	17	23763254	5.44E-02	5230	5.97E-04	Up	3.43	3025	1.26	0.32
rs7957595	5250	SLC25A3	12	97478762	5.44E-02	5231	9.01E-01	Up	0.12	13249	1.26	0.00
rs3741701	9498	SLC4A8	12	50102053	5.44E-02	5232	1.59E-01	Up	1.41	7806	1.26	0.08
rs1052886	5272	SERPINB9	6	2835189	5.44E-02	5233	2.24E-03	Down	3.06	3612	1.26	0.26
rs8024461	113201	CASC4	15	42370178	5.45E-02	5234	8.53E-01	Down	0.19	12971	1.26	0.01
rs9934434	92922	CCDC102A	16	56100674	5.45E-02	5235	1.66E-03	Down	3.14	3438	1.26	0.28
rs9840089	131540	ZDHHC19	3	197437851	5.45E-02	5236	4.43E-01	Down	0.77	10291	1.26	0.04
rs17310472	26005	C2CD3	11	73437267	5.45E-02	5237	7.60E-08	Up	5.37	1268	1.26	0.71
rs83615	5337	PLD1	3	172794431	5.46E-02	5238	1.59E-01	Down	1.41	7808	1.26	0.08
rs6541351	1810	DR1	1	93519143	5.46E-02	5239	3.35E-01	Up	0.96	9482	1.26	0.05
rs513061	6926	TBX3	12	113567397	5.46E-02	5240	3.68E-12	Down	6.95	614	1.26	1.14
rs2792022	9044	BTAF1	10	93730409	5.46E-02	5241	8.17E-01	Up	0.23	12776	1.26	0.01
rs713593	4257	MGST1	12	16413194	5.46E-02	5242	4.64E-01	Up	0.73	10453	1.26	0.03
rs10488773	5547	PRCP	11	82217152	5.46E-02	5243	3.34E-01	Down	0.97	9470	1.26	0.05
rs12437294	5267	SERPINA4	14	94114118	5.46E-02	5244	8.88E-01	Up	0.14	13181	1.26	0.01
rs10256972	84310	C7orf50	7	812244	5.47E-02	5245	2.04E-03	Up	3.08	3541	1.26	0.27
rs10256972	54905	CYP2W1	7	812244	5.47E-02	5246	4.36E-02	Down	2.02	5841	1.26	0.14
rs11767519	25913	POT1	7	124149111	5.47E-02	5247	1.37E-02	Up	2.46	4739	1.26	0.19
rs11599921	3363	HTR7	10	92523921	5.47E-02	5248	9.34E-03	Up	2.60	4458	1.26	0.20
rs11822237	7225	TRPC6	11	100835300	5.47E-02	5249	1.62E-02	Down	2.40	4901	1.26	0.18
rs1072755	10663	CXCR6	3	45950987	5.47E-02	5250	5.28E-02	Up	1.94	6061	1.26	0.13
rs10254616	26	ABP1	7	150002868	5.47E-02	5251	5.20E-02	Down	1.94	6039	1.26	0.13
rs3822262	6286	S100P	4	6815838	5.47E-02	5252	2.75E-03	Down	2.99	3742	1.26	0.26
rs3789553	8514	KCNAB2	1	6051437	5.47E-02	5253	3.63E-01	Up	0.91	9702	1.26	0.04
rs9791197	85313	PPIL4	6	149873595	5.48E-02	5254	7.45E-01	Up	0.33	12341	1.26	0.01
rs1721355	164781	WDR69	2	228608481	5.48E-02	5255	5.08E-01	Down	0.66	10772	1.26	0.03
rs7249518	5442	POLRMT	19	597900	5.48E-02	5256	2.63E-02	Down	2.22	5334	1.26	0.16
rs7249518	27006	FGF22	19	597900	5.48E-02	5257	1.44E-01	Up	1.46	7619	1.26	0.08
rs7249518	55658	RNF126	19	597900	5.48E-02	5258	9.63E-01	Up	0.05	13639	1.26	0.00
rs533878	10549	PRDX4		23433141	5.48E-02	5259	5.49E-23	Up	9.86	140	1.26	2.23
rs2664299	64919	BCL11B	14	98811940	5.48E-02	5260	1.98E-01	Down	1.29	8238	1.26	0.07

rs1887546	2947	GSTM3	1	110007685	5.48E-02	5261	1.32E-22	Down	9.78	149	1.26	2.19
rs1887546	79574	EPS8L3	1	110007685	5.48E-02	5262	1.56E-02	Down	2.42	4844	1.26	0.18
rs1105203	29127	RACGAP1	12	48719501	5.49E-02	5263	3.79E-04	Up	3.55	2860	1.26	0.34
rs1105203	41	ACCN2	12	48719501	5.49E-02	5264	4.09E-03	Up	2.87	3944	1.26	0.24
rs17661843	154664	ABCA13	7	48181012	5.49E-02	5265	4.58E-01	Up	0.74	10408	1.26	0.03
rs12445830	197258	FUK	16	69059994	5.49E-02	5266	3.61E-01	Down	0.91	9685	1.26	0.04
rs6425813	149076	ZNF362	1	33401285	5.49E-02	5267	5.41E-01	Down	0.61	11025	1.26	0.03
rs1468734	5493	PPL	16	4941305	5.49E-02	5268	1.40E-01	Down	1.48	7572	1.26	0.09
rs17253619	11169	WDHD1	14	54481494	5.50E-02	5269	8.86E-07	Up	4.92	1563	1.26	0.61
rs151618	9942	XYLB	3	38399189	5.50E-02	5270	2.16E-02	Up	2.30	5146	1.26	0.17
rs7034410	3440	IFNA2	9	21367478	5.50E-02	5271	8.96E-02	Up	1.70	6799	1.26	0.10
rs7034410	3447	IFNA13	9	21367478	5.50E-02	5272	8.57E-01	Up	0.18	12996	1.26	0.01
rs4698599	5860	QDPR	4	17154537	5.51E-02	5273	4.77E-05	Up	4.07	2269	1.26	0.43
rs4676902	151987	PPP4R2	3	73207490	5.51E-02	5274	3.11E-01	Up	1.01	9275	1.26	0.05
rs6578398	7221	TRPC2	11	3594637	5.51E-02	5275	1.38E-01	Up	1.48	7548	1.26	0.09
rs2237061	9547	CXCL14	5	134940229	5.51E-02	5276	1.88E-12	Up	7.04	581	1.26	1.17
rs12699476	8379	MAD1L1	7	1741486	5.51E-02	5277	4.96E-02	Up	1.96	5986	1.26	0.13
rs12228989	55801	IL26	12	66879474	5.52E-02	5278	3.07E-01	Up	1.02	9243	1.26	0.05
rs6493623	51621	KLF13	15	29444540	5.52E-02	5279	3.94E-01	Down	0.85	9938	1.26	0.04
rs10484854	2739	GLO1	6	38772572	5.52E-02	5280	2.56E-07	Up	5.15	1399	1.26	0.66
rs4744881	9413	C9orf61	9	69191477	5.53E-02	5281	2.31E-06	Up	4.72	1690	1.26	0.56
rs4805840	91442	C19orf40	19	38160322	5.53E-02	5282	1.60E-01	Down	1.40	7817	1.26	0.08
rs4805840	84902	CCDC123	19	38160322	5.53E-02	5283	6.51E-01	Down	0.45	11779	1.26	0.02
rs2064501	116379	IL22RA2	6	137519516	5.53E-02	5284	7.88E-01	Down	0.27	12597	1.26	0.01
rs4412433	774	CACNA1B	9	138142026	5.54E-02	5285	5.56E-01	Up	0.59	11124	1.26	0.03
rs7575751	8802	SUCLG1	2	84607661	5.54E-02	5286	2.13E-01	Down	1.25	8390	1.26	0.07
rs11585926	51592	TRIM33	1	114685732	5.54E-02	5287	-1.35E-11	Up	6.60	701	1.26	#NUM!
rs2304851	55036	CCDC40	17	75688355	5.54E-02	5288	1.01E-01	Down	1.64	7029	1.26	0.10
rs10752292	5264	PHYH	10	13387827	5.54E-02	5289	4.43E-02	Up	2.01	5860	1.26	0.14
rs12480575	79175	ZNF343	20	2426707	5.55E-02	5290	6.40E-01	Down	0.47	11705	1.26	0.02
rs258282	25837	RAB26	16	2146592	5.55E-02	5291	1.66E-02	Up	2.40	4918	1.26	0.18
rs258282	84231	TRAF7	16	2146592	5.55E-02	5292	6.31E-01	Up	0.48	11646	1.26	0.02
rs2687087	1577	CYP3A5	7	98939123	5.55E-02	5293	6.85E-26	Down	10.52	102	1.26	2.52
rs1530820	51314	TXNDC3	7	37714492	5.55E-02	5294	3.67E-01	Up	0.90	9734	1.26	0.04
rs2305058	284427	SLC25A41	19	6367123	5.55E-02	5295	6.11E-01	Down	0.51	11515	1.26	0.02
rs7623587	84319	C3orf26	3	101389441	5.55E-02	5296	5.33E-02	Up	1.93	6069	1.26	0.13
rs610949	60467	BPESC1	3	140297218	5.56E-02	5297	5.74E-01	Up	0.56	11273	1.26	0.02
rs1559949	58478	ENOPH1	4	83697784	5.56E-02	5298	1.97E-03	Up	3.09	3525	1.26	0.27
rs1559949	9987	HNRPDL	4	83697784	5.56E-02	5299	1.68E-02	Down	2.39	4928	1.26	0.18
rs6713551	7444	VRK2	2	58181522	5.56E-02	5300	9.87E-02	Up	1.65	6976	1.26	0.10
rs3127124	840	CASP7	10	115413685	5.56E-02	5301	5.32E-01	Down	0.62	10954	1.25	0.03
rs16976549	7138	TNNT1	16	26993895	5.56E-02	5302	5.82E-02	Down	1.89	6192	1.25	0.12
rs2858870	3123	HLA-DRB1	6	32680229	5.57E-02	5303	2.81E-02	Up	2.20	5395	1.25	0.16
rs569919	6581	SLC22A3	6	160737594	5.57E-02	5304	2.38E-04	Down	3.68	2691	1.25	0.36
rs896173	64224	HERPUD2	7	35508777	5.58E-02	5305	3.81E-01	Down	0.88	9845	1.25	0.04
rs2704668	25928	SOSTDC1	7	16293433	5.58E-02	5306	1.89E-01	Up	1.31	8145	1.25	0.07
rs741540	2842	GPR19	12	12699342	5.58E-02	5307	3.91E-01	Up	0.86	9922	1.25	0.04
rs792829	1295	COL8A1	3	100945337	5.58E-02	5308	9.95E-02	Up	1.65	6992	1.25	0.10
rs476916	374868	ATP9B	18	74911018	5.58E-02	5309	4.00E-04	Down	3.54	2884	1.25	0.34
rs11716	4682	NUBP1	16	10770465	5.58E-02	5310	6.68E-01	Up	0.43	11871	1.25	0.02
rs9531843	84189	SLITRK6	13	85281121	5.58E-02	5311	2.17E-06	Down	4.74	1679	1.25	0.57
rs438999	1797	DOM3Z	6	32036285	5.59E-02	5312	2.31E-03	Up	3.05	3635	1.25	0.26
rs438999	8859	STK19	6	32036285	5.59E-02	5313	5.28E-01	Down	0.63	10930	1.25	0.03
rs438999	6499	SKIV2L	6	32036285	5.59E-02	5314	5.38E-01	Up	0.62	10998	1.25	0.03
rs438999	7936	RDBP	6	32036285	5.59E-02	5315	9.15E-01	Down	0.11	13348	1.25	0.00
rs11893547	84279	C2orf7	2	73363059	5.59E-02	5316	3.85E-01	Down	0.87	9880	1.25	0.04
rs11893547	10574	CCT7	2	73363059	5.59E-02	5317	5.47E-01	Up	0.60	11061	1.25	0.03
rs11893547	10322	SMYD5	2	73363059	5.59E-02	5318	8.85E-01	Up	0.14	13157	1.25	0.01
rs9932707	164	AP1G1	16	70419634	5.59E-02	5319	8.69E-03	Down	2.62	4405	1.25	0.21
rs13207315	3107	HLA-C	6	31349106	5.59E-02	5320	5.64E-01	Up	0.58	11192	1.25	0.02
rs2277063	23338	PHF15	5	133887508	5.59E-02	5321	5.92E-13	Down	7.20	534	1.25	1.22
rs6835291	85013	TMEM128	4	4385201	5.59E-02	5322	1.05E-03	Up	3.28	3242	1.25	0.30
rs6835291	55646	LYAR	4	4385201	5.59E-02	5323	2.60E-03	Up	3.01	3708	1.25	0.26
rs4669476	9014	TAF1B	2	9967225	5.59E-02	5324	9.51E-01	Down	0.06	13563	1.25	0.00
rs218655	51003	MED31	17	6514688	5.59E-02	5325	2.44E-01	Down	1.17	8700	1.25	0.06
rs1568003	27180	SIGLEC9	19	56325472	5.59E-02	5326	1.17E-01	Down	1.57	7250	1.25	0.09
rs1568003	27036	SIGLEC7	19	56325472	5.59E-02	5327	9.04E-01	Down	0.12	13275	1.25	0.00
rs1860565	5178	PEG3	19	62026834	5.59E-02	5328	2.63E-13	Down	7.31	502	1.25	1.26
rs1860565	23619	ZIM2	19	62026834	5.59E-02	5329	4.32E-01	Down	0.79	10208	1.25	0.04
rs1492482	257144	GCET2	3	113336779	5.60E-02	5330	3.90E-01	Down	0.86	9916	1.25	0.04
rs4789649	124602	KIF19	17	69838077	5.60E-02	5331	5.90E-02	Down	1.89	6217	1.25	0.12
rs11634375	2252	FGF7	15	47536840	5.60E-02	5332	6.07E-11	Down	6.54	729	1.25	1.02
rs2291473	7087	ICAM5	19	10276781	5.60E-02	5333	9.76E-02	Down	1.66	6952	1.25	0.10
rs5992507	128989	C22orf25	22	18432012	5.61E-02	5334	8.02E-02	Down	1.75	6634	1.25	0.11
rs1531133	9419	CRIPT	2	46755282	5.61E-02	5335	2.74E-01	Up	1.09	8989	1.25	0.06
rs7156186	283579	C14orf178	14	77301286	5.61E-02	5336	4.74E-01	Up	0.72	10523	1.25	0.03
rs825078	4604	MYBPC1	12	100508714	5.62E-02	5337	1.97E-03	Up	3.10	3522	1.25	0.27
rs8035205	22995	CEP152	15	46881686	5.62E-02	5338	9.52E-04	Up	3.30	3200	1.25	0.30
rs4754458	2230	FDX1	11	109844329	5.62E-02	5339	2.05E-01	Up	1.27	8307	1.25	0.07
rs7917581	10367	CBARA1	10	73851763	5.62E-02	5340	1.62E-01	Up	1.40	7845	1.25	0.08
rs986363	84677	DSCR8	21	38451807	5.62E-02	5341	8.28E-01	Down	0.22	12842	1.25	0.01

rs12310569	1108	CHD4	12	6567614	5.62E-02	5342	3.58E-01	Down	0.92	9665	1.25	0.04
rs17472998	143279	HECTD2	10	93268941	5.63E-02	5343	5.70E-01	Down	0.57	11244	1.25	0.02
rs17154020	220992	ZNF485	10	43430347	5.63E-02	5344	1.49E-01	Up	1.44	7672	1.25	0.08
rs737088	7078	TIMP3	22	31512496	5.63E-02	5345	5.74E-36	Down	12.52	34	1.25	3.52
rs1267658	64506	CPEB1	15	81011209	5.64E-02	5346	1.02E-01	Down	1.63	7036	1.25	0.10
rs7543174	126668	TDRD10	1	151340745	5.64E-02	5347	1.37E-02	Down	2.46	4743	1.25	0.19
rs7543174	1141	CHRNB2	1	151340745	5.64E-02	5348	7.79E-01	Down	0.28	12544	1.25	0.01
rs7142209	57596	BEGAIN	14	100063798	5.64E-02	5349	1.64E-02	Down	2.40	4908	1.25	0.18
rs7142209	79446	WDR25	14	100063798	5.64E-02	5350	6.55E-01	Down	0.45	11802	1.25	0.02
rs13232606	3486	IGFBP3	7	45744350	5.65E-02	5351	1.59E-02	Down	2.41	4870	1.25	0.18
rs1569462	6196	RPS6KA2	6	166875338	5.65E-02	5352	6.48E-01	Up	0.46	11764	1.25	0.02
rs2066910		MCAT	22	41854015	5.65E-02	5353	2.04E-03	Up	3.09	3540	1.25	0.27
rs4419859	2731	GLDC	9	6534766	5.66E-02	5354	1.54E-05	Up	4.32	2041	1.25	0.48
rs10100368	11059	WWP1	8	87551360	5.66E-02	5355	1.23E-05	Up	4.37	1996	1.25	0.49
rs13417603	81553	FAM49A	2	16737634	5.66E-02	5356	4.63E-03	Down	2.83	4015	1.25	0.23
rs10100368	51115	FAM82B	8	87551360	5.66E-02	5357	6.55E-02	Up	1.84	6347	1.25	0.12
rs8108252	114132	SIGLEC11	19	55135873	5.66E-02	5358	9.15E-01	Down	0.11	13351	1.25	0.00
rs7315980	84926	SPRYD3	12	51735588	5.66E-02	5359	6.01E-02	Down	1.88	6239	1.25	0.12
rs10418705	54811	ZNF562	19	9629925	5.66E-02	5360	4.08E-02	Up	2.05	5766	1.25	0.14
rs11634329	56963	RGMA	15	91401162	5.66E-02	5361	6.68E-01	Down	0.43	11875	1.25	0.02
rs6427504	480	ATP1A4	1	156937948	5.66E-02	5362	4.72E-02	Down	1.98	5926	1.25	0.13
rs7816300	157753	TMEM74	8	109857032	5.66E-02	5363	1.45E-01	Up	1.46	7632	1.25	0.08
rs2713616	23129	PLXND1	3	130756013	5.66E-02	5364	5.37E-03	Down	2.78	4109	1.25	0.23
rs2713616	132243	H1FOO	3	130756013	5.66E-02	5365	3.57E-01	Up	0.92	9657	1.25	0.04
rs1614377	126	ADH1C	4	100636510	5.66E-02	5366	9.43E-02	Down	1.67	6902	1.25	0.10
rs7179270	7082	TJP1	15	27805919	5.67E-02	5367	3.54E-12	Up	6.95	612	1.25	1.15
rs6592284	51501	C11orf73	11	85733549	5.67E-02	5368	1.60E-01	Up	1.40	7818	1.25	0.08
rs3769689	11320	MGAT4A	2	98752215	5.67E-02	5369	1.05E-12	Up	7.11	560	1.25	1.20
rs7200210	6560	SLC12A4	16	66539397	5.67E-02	5370	9.72E-10	Down	6.11	892	1.25	0.90
rs7200210	3931	LCAT	16	66539397	5.67E-02	5371	1.19E-03	Down	3.24	3285	1.25	0.29
rs7200210	5699	PSMB10	16	66539397	5.67E-02	5372	1.06E-01	Down	1.62	7096	1.25	0.10
rs7200210	1506	CTRL	16	66539397	5.67E-02	5373	3.12E-01	Up	1.01	9286	1.25	0.05
rs4719884	3201	HOXA4	7	26945811	5.67E-02	5374	9.80E-04	Down	3.30	3213	1.25	0.30
rs4719884	3200	HOXA3	7	26945811	5.67E-02	5375	6.47E-01	Down	0.46	11760	1.25	0.02
rs3803869	2535	FZD2	17	39992580	5.68E-02	5376	8.00E-03	Down	2.65	4342	1.25	0.21
rs4718428	55069	C7orf42	7	65865596	5.68E-02	5377	8.14E-01	Up	0.24	12749	1.25	0.01
rs1462977	203111	C8orf47	8	99184535	5.68E-02	5378	2.04E-04	Down	3.71	2637	1.25	0.37
rs10501333	4250	SCGB2A2	11	61779877	5.68E-02	5379	1.10E-04	Up	3.87	2477	1.25	0.40
rs3819331	5805	PTS	11	111604620	5.68E-02	5380	4.41E-05	Up	4.08	2251	1.25	0.44
rs2097628	1621	DBH	9	133549256	5.69E-02	5381	2.08E-01	Down	1.26	8332	1.24	0.07
rs556759	55231	CCDC87	11	66128966	5.69E-02	5382	4.53E-01	Down	0.75	10367	1.24	0.03
rs556759	9973	CCS	11	66128966	5.69E-02	5383	4.82E-01	Up	0.70	10590	1.24	0.03
rs378974	79660	PPP1R3B	8	9064049	5.69E-02	5384	1.24E-03	Down	3.23	3307	1.24	0.29
rs4444435	63946	DMRTC2	19	47035366	5.69E-02	5385	1.19E-02	Up	2.52	4633	1.24	0.19
rs4444435	147719	LYPD4	19	47035366	5.69E-02	5386	5.99E-01	Up	0.53	11426	1.24	0.02
rs12983784	1994	ELAVL1	19	7931491	5.70E-02	5387	2.90E-02	Up	2.18	5425	1.24	0.15
rs1544089	51313	C4orf18	4	159452682	5.70E-02	5388	4.34E-16	Up	8.13	345	1.24	1.54
rs752313	2145	EZH1	17	38155350	5.70E-02	5389	3.55E-07	Down	5.09	1436	1.24	0.64
rs752313	10266	RAMP2	17	38155350	5.70E-02	5390	1.57E-03	Down	3.16	3409	1.24	0.28
rs374361	85409	NKD2	5	1052338	5.71E-02	5391	9.16E-02	Down	1.69	6852	1.24	0.10
rs2096376	8220	DGCR14	22	17521995	5.71E-02	5392	2.72E-02	Down	2.21	5369	1.24	0.16
rs11599388	2665	GDI2	10	5914964	5.71E-02	5393	1.41E-06	Up	4.82	1618	1.24	0.59
rs3928917	1087	CEACAM7	19	46899486	5.71E-02	5394	7.92E-03	Down	2.66	4335	1.24	0.21
rs3928917	1048	CEACAM5	19	46899486	5.71E-02	5395	1.24E-01	Up	1.54	7362	1.24	0.09
rs3735785	7564	ZNF16	8	146142241	5.71E-02	5396	3.25E-01	Up	0.98	9402	1.24	0.05
rs513546	152110	NEK10	3	27323924	5.71E-02	5397	2.00E-02	Down	2.33	5073	1.24	0.17
rs353465	51720	UIMC1	5	176329494	5.71E-02	5398	2.39E-01	Down	1.18	8649	1.24	0.06
rs17632109	80255	SLC35F5	2	114201356	5.72E-02	5399	8.74E-20	Up	9.07	209	1.24	1.91
rs921651	5709	PSMD3	17	35387448	5.73E-02	5400	6.55E-03	Up	2.72	4214	1.24	0.22
rs6864158	6550	SLC9A3	5	559051	5.73E-02	5401	3.96E-02	Down	2.06	5741	1.24	0.14
rs4073716	8817	FGF18	5	170796844	5.73E-02	5402	5.41E-01	Up	0.61	11022	1.24	0.03
rs10772006	29121	CLEC2D	12	9705134	5.73E-02	5403	2.72E-02	Down	2.21	5367	1.24	0.16
rs2872690	56203	LMOD3	3	69254493	5.74E-02	5404	5.65E-01	Down	0.57	11209	1.24	0.02
rs4775090	102	ADAM10	15	56799614	5.74E-02	5405	3.15E-01	Up	1.01	9319	1.24	0.05
rs2076513	2296	FOXC1	6	1536010	5.74E-02	5406	6.99E-01	Up	0.39	12064	1.24	0.02
rs617182	162466	PHOSPHO1	17	44662273	5.74E-02	5407	2.61E-01	Down	1.12	8871	1.24	0.06
rs617182	51225	ABI3	17	44662273	5.74E-02	5408	9.06E-01	Up	0.12	13285	1.24	0.00
rs734108	1084	CEACAM3	19	47005668	5.74E-02	5409	7.64E-03	Down	2.67	4317	1.24	0.21
rs2182647	22849	CPEB3	10	93925324	5.74E-02	5410	2.26E-03	Up	3.05	3616	1.24	0.26
rs6466226	4897	NRCAM	7	107635316	5.74E-02	5411	5.11E-05	Up	4.05	2295	1.24	0.43
rs11058000	140707	BRI3BP	12	124036423	5.75E-02	5412	1.60E-07	Up	5.24	1348	1.24	0.68
rs2351000	55215	FANCI	15	87670737	5.75E-02	5413	1.32E-01	Up	1.51	7469	1.24	0.09
rs2351000	5428	POLG	15	87670737	5.75E-02	5414	5.53E-01	Up	0.59	11102	1.24	0.03
rs3776205	9555	H2AFY	5	134728549	5.75E-02	5415	3.63E-10	Up	6.27	821	1.24	0.94
rs12529592	23506	KIAA0240	6	42910716	5.75E-02	5416	8.48E-01	Down	0.19	12941	1.24	0.01
rs12685502	9830	TRIM14	9	97936002	5.75E-02	5417	7.31E-13	Up	7.16	547	1.24	1.21
rs2307252	3364	HUS1	7	47789682	5.75E-02	5418	1.75E-04	Up	3.75	2585	1.24	0.38
rs12507167	6443	SGCB	4	52759631	5.75E-02	5419	1.70E-37	Down	12.80	25	1.24	3.68
rs11178190	4848	CNOT2	12	69016083	5.76E-02	5420	2.55E-01	Up	1.14	8813	1.24	0.06
rs4409904	2288	FKBP4	12	2756390	5.76E-02	5421	7.91E-12	Up	6.83	637	1.24	1.11
rs4854838	131034	CPNE4	3	132961313	5.76E-02	5422	1.14E-03	Up	3.25	3271	1.24	0.29

rs12097383	261726	TIPRL	1	164869742	5.76E-02	5423	9.75E-01	Up	0.03	13730	1.24	0.00
rs165821	4744	NEFH	22	28202179	5.77E-02	5424	2.33E-04	Down	3.68	2686	1.24	0.36
rs1971762	517	ATP5G2	12	52344505	5.77E-02	5425	5.23E-06	Up	4.56	1825	1.24	0.53
rs7734458	84651	SPINK7	5	147666145	5.77E-02	5426	3.35E-01	Down	0.96	9486	1.24	0.05
rs17610424	1106	CHD2	15	91329058	5.78E-02	5427	2.54E-06	Up	4.70	1704	1.24	0.56
rs7252013	59284	CACNG7	19	59123073	5.78E-02	5428	1.73E-01	Down	1.36	7941	1.24	0.08
rs1041474	49861	CLDN20	6	155663810	5.78E-02	5429	4.48E-01	Down	0.76	10329	1.24	0.03
rs3786521	9476	NAPSA	19	52704810	5.78E-02	5430	2.67E-01	Up	1.11	8922	1.24	0.06
rs3786521	8775	NAPA	19	52704810	5.78E-02	5431	9.66E-01	Up	0.04	13662	1.24	0.00
rs1345229	7726	TRIM26	6	30290374	5.78E-02	5432	7.60E-08	Up	5.38	1265	1.24	0.71
rs3844057	5062	PAK2	3	197950811	5.78E-02	5433	1.19E-02	Up	2.52	4630	1.24	0.19
rs3844057	54965	PIGX	3	197950811	5.78E-02	5434	1.08E-01	Down	1.61	7127	1.24	0.10
rs11204212	1869	E2F1	10	47994338	5.79E-02	5435	5.72E-02	Up	1.90	6162	1.24	0.12
rs11204212	118738	ZNF488	10	47994338	5.79E-02	5436	1.26E-01	Down	1.53	7389	1.24	0.09
rs11204212	5949	RBP3	10	47994338	5.79E-02	5437	8.71E-01	Up	0.16	13074	1.24	0.01
rs170296	4058	LTK	15	39584193	5.79E-02	5438	3.37E-01	Down	0.96	9501	1.24	0.05
rs170296	3706	ITPKA	15	39584193	5.79E-02	5439	6.57E-01	Down	0.44	11815	1.24	0.02
rs4644955	255043	TMEM86B	19	60430446	5.80E-02	5440	3.87E-01	Down	0.86	9897	1.24	0.04
rs1573402	23556	PIGN	18	57902170	5.80E-02	5441	1.48E-02	Up	2.44	4803	1.24	0.18
rs13233948	155185	AMZ1	7	2488552	5.80E-02	5442	8.88E-01	Down	0.14	13180	1.24	0.01
rs4150579	2965	GTF2H1	11	18313756	5.81E-02	5443	4.76E-01	Up	0.71	10547	1.24	0.03
rs4150579	11234	HPS5	11	18313756	5.81E-02	5444	9.44E-01	Down	0.07	13516	1.24	0.00
rs12143020	115019	SLC26A9	1	202629065	5.81E-02	5445	6.35E-02	Down	1.86	6310	1.24	0.12
rs7458161	57786	RBAK	7	4856982	5.82E-02	5446	1.42E-02	Up	2.45	4762	1.24	0.18
rs4676487	1524	CX3CR1	3	39278151	5.82E-02	5447	5.86E-02	Down	1.89	6209	1.24	0.12
rs1426369	9671	WSCD2	12	107126493	5.82E-02	5448	1.58E-01	Down	1.41	7783	1.24	0.08
rs872561	1240	CMKLR1	12	107210482	5.82E-02	5449	7.75E-01	Down	0.29	12531	1.23	0.01
rs2300658	22955	SCMH1	1	41174115	5.82E-02	5450	2.70E-03	Up	3.00	3729	1.23	0.26
rs2300658	200172	SLFNL1	1	41174115	5.82E-02	5451	8.38E-01	Up	0.20	12889	1.23	0.01
rs3777567	54102	CLIC6	6	46008544	5.82E-02	5452	1.88E-06	Down	4.77	1656	1.23	0.57
rs3777567	53405	CLIC5	6	46008544	5.82E-02	5453	1.86E-01	Down	1.32	8100	1.23	0.07
rs12961855	2774	GNAL	18	11660845	5.83E-02	5454	1.19E-02	Down	2.51	4634	1.23	0.19
rs4964629	51559	NT5DC3	12	102724317	5.83E-02	5455	7.70E-02	Down	1.77	6574	1.23	0.11
rs10505783	397	ARHGDIB	12	15018205	5.83E-02	5456	1.47E-04	Up	3.80	2544	1.23	0.38
rs10505783	5149	PDE6H	12	15018205	5.83E-02	5457	7.62E-02	Up	1.77	6554	1.23	0.11
rs13353497	79442	LRRC2	3	46573381	5.83E-02	5458	8.46E-02	Up	1.72	6714	1.23	0.11
rs10407971	388533	KRTDAP	19	40671304	5.83E-02	5459	3.21E-01	Down	0.99	9369	1.23	0.05
rs1126618	722	C4BPA	1	203686075	5.83E-02	5460	6.27E-04	Down	3.42	3045	1.23	0.32
rs13030217	170850	KCNG3	2	42624583	5.83E-02	5461	4.85E-03	Up	2.82	4045	1.23	0.23
rs16854229	126859	C1orf125	1	176228337	5.84E-02	5462	9.36E-01	Down	0.08	13468	1.23	0.00
rs955967	11063	SOX30	5	156980293	5.84E-02	5463	7.03E-01	Up	0.38	12087	1.23	0.02
rs2237344	8887	TAX1BP1	7	27614766	5.84E-02	5464	9.14E-01	Up	0.11	13342	1.23	0.00
rs12082710	7049	TGFBR3	1	91867358	5.84E-02	5465	2.89E-34	Down	12.21	43	1.23	3.35
rs12411706	80351	TNKS2	10	93590282	5.84E-02	5466	4.54E-01	Up	0.75	10376	1.23	0.03
rs10497520	7273	TTN	2	179470361	5.84E-02	5467	1.86E-01	Down	1.32	8099	1.23	0.07
rs13327422	57110	HRASLS	3	194458851	5.84E-02	5468	9.68E-01	Up	0.04	13682	1.23	0.00
rs17088531	1960	EGR3	8	22592029	5.84E-02	5469	2.03E-05	Up	4.26	2090	1.23	0.47
rs6599300	10296	MAEA	4	1295099	5.85E-02	5470	8.86E-04	Up	3.32	3172	1.23	0.31
rs3027641	695	BTK		100423134	5.85E-02	5471	1.98E-02	Down	2.33	5064	1.23	0.17
rs3027641	1678	TIMM8A		100423134	5.85E-02	5472	4.50E-02	Up	2.00	5873	1.23	0.13
rs859582	7512	XPNPEP2		128619344	5.85E-02	5473	7.82E-01	Down	0.28	12555	1.23	0.01
rs11924964	56945	MRPS22	3	140544542	5.85E-02	5474	2.82E-02	Up	2.19	5398	1.23	0.16
rs16984830	51507	C20orf43	20	54468909	5.85E-02	5475	1.98E-08	Down	5.61	1120	1.23	0.77
rs4462057	90161	HS6ST2		131578026	5.85E-02	5476	6.85E-01	Up	0.41	11976	1.23	0.02
rs2782977	55088	C10orf118	10	115865016	5.85E-02	5477	2.48E-03	Up	3.03	3682	1.23	0.26
rs1008898	56913	C1GALT1	7	7046799	5.85E-02	5478	2.75E-02	Up	2.20	5380	1.23	0.16
rs7178111	8925	HERC1	15	61862564	5.85E-02	5479	9.48E-02	Down	1.67	6905	1.23	0.10
rs3785496	54903	MKS1	17	53629132	5.86E-02	5480	1.12E-01	Up	1.59	7184	1.23	0.09
rs6532731	5910	RAP1GDS1	4	99608184	5.86E-02	5481	1.18E-01	Up	1.56	7264	1.23	0.09
rs9390855	10690	FUT9	6	96659997	5.86E-02	5482	5.13E-01	Up	0.65	10803	1.23	0.03
rs1049620	5245	PHB	17	44836513	5.86E-02	5483	1.18E-01	Down	1.56	7262	1.23	0.09
rs12139944	10765	JARID1B	1	199526444	5.86E-02	5484	8.60E-15	Up	7.75	404	1.23	1.41
rs10482863	8204	NRIP1	21	15257340	5.86E-02	5485	1.11E-09	Up	6.09	901	1.23	0.90
rs4478844	26247	OR2L1P	1	244455281	5.86E-02	5486	2.72E-01	Up	1.10	8966	1.23	0.06
rs831501	221472	FGD2	6	37097565	5.87E-02	5487	2.21E-01	Down	1.22	8473	1.23	0.07
rs12500151	2160	F11	4	187592787	5.87E-02	5488	6.74E-02	Up	1.83	6386	1.23	0.12
rs10494343	57216	VANGL2	1	157215612	5.87E-02	5489	2.59E-01	Down	1.13	8854	1.23	0.06
rs2735117	6434	SFRS10	3	187118558	5.87E-02	5490	5.90E-01	Up	0.54	11367	1.23	0.02
rs10987630	29988	SLC2A8	9	127232076	5.87E-02	5491	5.44E-01	Down	0.61	11045	1.23	0.03
rs10883841	22978	NT5C2	10	104924699	5.87E-02	5492	6.79E-02	Up	1.83	6394	1.23	0.12
rs3733697	26167	PCDHB5	5	140482528	5.87E-02	5493	1.02E-03	Up	3.28	3229	1.23	0.30
rs3733697	56131	PCDHB4	5	140482528	5.87E-02	5494	1.34E-01	Up	1.50	7493	1.23	0.09
rs6957319	10049	DNAJB6	7	156714258	5.87E-02	5495	4.54E-03	Down	2.84	4000	1.23	0.23
rs221530	84553	C6orf168	6	99860220	5.87E-02	5496	7.70E-02	Down	1.77	6576	1.23	0.11
rs1003921	3746	KCNC1	11	17756912	5.88E-02	5497	2.79E-02	Down	2.20	5390	1.23	0.16
rs1923662	23048	FNBP1	9	129882760	5.88E-02	5498	3.20E-07	Down	5.11	1424	1.23	0.65
rs8035516	5151	PDE8A	15	83486363	5.88E-02	5499	1.30E-11	Down	6.77	647	1.23	1.09
rs11634818	54832	VPS13C	15	60143678	5.88E-02	5500	1.23E-01	Up	1.54	7346	1.23	0.09
rs102993	8021	NUP214	9	131044626	5.89E-02	5501	4.87E-01	Up	0.69	10629	1.23	0.03
rs11990338	137735	ABRA	8	107859883	5.89E-02	5502	1.19E-01	Up	1.56	7279	1.23	0.09
rs13134529	257236	CCDC96	4	7155774	5.89E-02	5503	3.66E-01	Down	0.90	9729	1.23	0.04

rs13134529	57533	TBC1D14	4	7155774	5.89E-02	5504	4.52E-01	Down	0.75	10355	1.23	0.03
rs9287903	115677	NOSTRIN	2	169505629	5.89E-02	5505	4.96E-02	Down	1.96	5989	1.23	0.13
rs6070703	514	ATP5E	20	57055252	5.90E-02	5506	8.50E-02	Up	1.72	6722	1.23	0.11
rs876941	1512	CTSH	15	77030626	5.90E-02	5507	3.23E-06	Down	4.66	1752	1.23	0.55
rs9394309	2289	FKBP5	6	35729759	5.90E-02	5508	3.36E-02	Up	2.12	5580	1.23	0.15
rs10083915	6093	ROCK1	18	16954953	5.91E-02	5509	9.21E-02	Down	1.68	6866	1.23	0.10
rs2258851	54517	PUS7	7	104704941	5.91E-02	5510	4.50E-15	Up	7.83	387	1.23	1.43
rs9760	1522	CTSZ	20	57005158	5.91E-02	5511	6.04E-02	Down	1.88	6246	1.23	0.12
rs2002042	1244	ABCC2	10	101577921	5.91E-02	5512	2.17E-01	Down	1.24	8430	1.23	0.07
rs4834426	7368	UGT8	4	115941327	5.91E-02	5513	2.63E-01	Up	1.12	8885	1.23	0.06
rs6803929	54861	SNRK	3	43347644	5.92E-02	5514	8.04E-01	Down	0.25	12696	1.23	0.01
rs13059110	25852	ARMC8	3	139480440	5.92E-02	5515	1.01E-02	Down	2.57	4512	1.23	0.20
rs13059110	347736	TXNDC6	3	139480440	5.92E-02	5516	9.02E-01	Down	0.12	13259	1.23	0.00
rs17289226	23607	CD2AP	6	47612472	5.92E-02	5517	3.08E-08	Up	5.53	1170	1.23	0.75
rs6749057	80745	THUMPD2	2	39881333	5.93E-02	5518	1.79E-01	Up	1.34	8015	1.23	0.07
rs927451	6950	TCP1	6	160183847	5.93E-02	5519	7.80E-08	Up	5.37	1269	1.23	0.71
rs927451	29074	MRPL18	6	160183847	5.93E-02	5520	2.58E-01	Down	1.13	8841	1.23	0.06
rs12610559	57693	ZNF317	19	9124536	5.93E-02	5521	9.77E-02	Up	1.66	6958	1.23	0.10
rs9933126	2813	GP2	16	20219693	5.93E-02	5522	3.04E-01	Up	1.03	9224	1.23	0.05
rs17652766	11130	ZWINT	10	57778586	5.93E-02	5523	1.91E-12	Up	7.03	585	1.23	1.17
rs528854	80755	AARSD1	17	38351634	5.94E-02	5524	2.82E-02	Up	2.19	5403	1.23	0.15
rs12282721	23621	BACE1	11	116656622	5.94E-02	5525	1.61E-07	Down	5.24	1349	1.23	0.68
rs1395718	80149	ZC3H12A	1	37611046	5.94E-02	5526	2.62E-01	Down	1.12	8879	1.23	0.06
rs3744017	91107	TRIM47	17	71383062	5.94E-02	5527	2.44E-02	Down	2.25	5264	1.23	0.16
rs6573766	57523	KIAA1305	14	23921886	5.94E-02	5528	2.73E-04	Down	3.64	2746	1.23	0.36
rs6573766	4776	NFATC4	14	23921886	5.94E-02	5529	3.79E-03	Down	2.89	3901	1.23	0.24
rs11137515	53358	SHC3	9	88952560	5.94E-02	5530	4.10E-01	Down	0.82	10051	1.23	0.04
rs7248167	27076	LYPD3	19	48651954	5.95E-02	5531	6.92E-01	Up	0.40	12021	1.23	0.02
rs10860862	3479	IGF1	12	101288539	5.95E-02	5532	2.91E-24	Down	10.16	123	1.23	2.35
rs10112596	2626	GATA4	8	11617211	5.95E-02	5533	3.67E-01	Down	0.90	9736	1.23	0.04
rs10877201	225	ABCD2	12	38298564	5.95E-02	5534	9.12E-01	Up	0.11	13327	1.23	0.00
rs1468673	727	C5	9	120889444	5.96E-02	5535	1.60E-02	Up	2.41	4884	1.23	0.18
rs6552804	79682	MLF1IP	4	186012848	5.96E-02	5536	5.59E-12	Up	6.89	625	1.22	1.13
rs12327639	55762	ZNF701	19	57758669	5.96E-02	5537	2.87E-01	Up	1.06	9095	1.22	0.05
rs1410871	115361	GBP4	1	89368015	5.96E-02	5538	1.27E-01	Down	1.53	7393	1.22	0.09
rs893790	22924	MAPRE3	2	27113508	5.97E-02	5539	5.83E-01	Down	0.55	11335	1.22	0.02
rs1944096	54961	SSH3	11	66842874	5.97E-02	5540	4.58E-02	Down	2.00	5889	1.22	0.13
rs17684048	5965	RECQL	12	21553718	5.97E-02	5541	1.48E-02	Down	2.44	4799	1.22	0.18
rs17684048	51026	GOLT1B	12	21553718	5.97E-02	5542	3.46E-02	Up	2.11	5611	1.22	0.15
rs17684048	80763	C12orf39	12	21553718	5.97E-02	5543	3.52E-01	Up	0.93	9611	1.22	0.05
rs17684048	127845	GOLT1A	12	21553718	5.97E-02	5544	6.56E-01	Down	0.45	11808	1.22	0.02
rs11756526	654	BMP6	6	7726783	5.97E-02	5545	1.50E-03	Up	3.18	3382	1.22	0.28
rs2298455	4926	NUMA1	11	71388126	5.97E-02	5546	2.35E-05	Up	4.23	2116	1.22	0.46
rs2298455	10068	IL18BP	11	71388126	5.97E-02	5547	3.71E-01	Up	0.89	9771	1.22	0.04
rs2298455	55298	RNF121	11	71388126	5.97E-02	5548	6.14E-01	Down	0.50	11539	1.22	0.02
rs2607456	90594	ZNF439	19	11820057	5.97E-02	5549	2.43E-01	Down	1.17	8689	1.22	0.06
rs17107115	115669	TTC6	14	37334707	5.98E-02	5550	1.90E-04	Up	3.73	2610	1.22	0.37
rs12098741	159371	TMEM20	10	95640951	5.98E-02	5551	5.14E-02	Down	1.95	6022	1.22	0.13
rs578026	27098	CLUL1	18	587751	5.98E-02	5552	8.77E-01	Down	0.16	13097	1.22	0.01
rs4398051	79811	SLTM	15	56978288	5.98E-02	5553	2.86E-01	Up	1.07	9081	1.22	0.05
rs4652869	58512	DLGAP3	1	35048027	5.98E-02	5554	1.36E-01	Down	1.49	7523	1.22	0.09
rs7714584	91975	ZNF300	5	150250613	5.99E-02	5555	7.65E-02	Up	1.77	6561	1.22	0.11
rs9897184	55890	GPRC5C	17	69926356	5.99E-02	5556	9.43E-05	Down	3.90	2437	1.22	0.40
rs2281097	3957	LGALS2	22	36290560	5.99E-02	5557	4.39E-01	Up	0.77	10261	1.22	0.04
rs2298229	10562	OLFM4	13	52500971	5.99E-02	5558	1.01E-02	Down	2.57	4513	1.22	0.20
rs4877785	257019	FRMD3	9	83348599	5.99E-02	5559	7.92E-01	Down	0.26	12625	1.22	0.01
rs17149842	51678	MPP6	7	24487369	6.00E-02	5560	3.64E-15	Up	7.86	384	1.22	1.44
rs17149842	10200	MPHOSPH6	7	24487369	6.00E-02	5561	5.21E-05	Down	4.05	2299	1.22	0.43
rs2287170	2984	GUCY2C	12	14717238	6.00E-02	5562	6.43E-02	Up	1.85	6327	1.22	0.12
rs1011024	221785	ZNF498	7	98842052	6.00E-02	5563	8.79E-02	Down	1.71	6770	1.22	0.11
rs7437482	10891	PPARGC1A	4	23532243	6.00E-02	5564	8.25E-07	Down	4.93	1551	1.22	0.61
rs10489638	962	CD48	1	157467139	6.00E-02	5565	6.80E-01	Down	0.41	11947	1.22	0.02
rs1792016	55800	SCN3B	11	123024253	6.00E-02	5566	6.22E-02	Down	1.86	6283	1.22	0.12
rs8100253	9040	UBE2M	19	63779291	6.01E-02	5567	4.29E-02	Up	2.02	5824	1.22	0.14
rs2250870	5152	PDE9A	21	43030256	6.01E-02	5568	6.62E-06	Up	4.51	1865	1.22	0.52
rs6499166	84138	SLC7A6OS	16	66884418	6.01E-02	5569	1.91E-01	Up	1.31	8161	1.22	0.07
rs6499166	54496	PRMT7	16	66884418	6.01E-02	5570	9.44E-01	Down	0.07	13520	1.22	0.00
rs6775695	4711	NDUFB5	3	180834876	6.01E-02	5571	5.51E-04	Up	3.45	2996	1.22	0.33
rs1552458	752	FMNL1	17	40654171	6.01E-02	5572	4.25E-01	Down	0.80	10156	1.22	0.04
rs4652707	2752	GLUL	1	179093916	6.01E-02	5573	3.71E-04	Down	3.56	2856	1.22	0.34
rs4652707	127670	TEDDM1	1	179093916	6.01E-02	5574	4.98E-01	Up	0.68	10700	1.22	0.03
rs2025853	6840	SVIL	10	29829654	6.01E-02	5575	6.89E-15	Down	7.79	398	1.22	1.42
rs12831686	32	ACACB	12	108115120	6.01E-02	5576	2.05E-03	Down	3.08	3545	1.22	0.27
rs10437827	8074	FGF23	12	4343590	6.02E-02	5577	5.63E-01	Down	0.58	11188	1.22	0.02
rs11670126	4037	LRP3	19	38373310	6.02E-02	5578	7.20E-01	Down	0.36	12199	1.22	0.01
rs12461372	166	AES	19	3023859	6.02E-02	5579	9.24E-10	Down	6.12	887	1.22	0.90
rs7512209	2987	GUK1	1	224618451	6.03E-02	5580	2.20E-04	Up	3.69	2664	1.22	0.37
rs7512209	128308	MRPL55	1	224618451	6.03E-02	5581	8.34E-01	Up	0.21	12875	1.22	0.01
rs3740789	9537	TP53I11	11	44909506	6.03E-02	5582	3.13E-01	Up	1.01	9299	1.22	0.05
rs11571805	675	BRCA2	13	31861947	6.03E-02	5583	2.75E-03	Up	2.99	3743	1.22	0.26
rs10120023	2219	FCN1	9	135036204	6.03E-02	5584	4.87E-01	Down	0.69	10631	1.22	0.03

rs179007	51284	TLR7		12669979	6.04E-02	5585	9.60E-01	Up	0.05	13623	1.22	0.00
rs12843603	6758	SSX5		47820030	6.04E-02	5586	5.06E-01	Up	0.67	10755	1.22	0.03
rs9893662	6662	SOX9	17	67623466	6.04E-02	5587	5.81E-02	Up	1.89	6190	1.22	0.12
rs12936361	3773	KCNJ16	17	65620012	6.05E-02	5588	1.98E-03	Down	3.09	3527	1.22	0.27
rs7113533	10714	POLD3	11	73961919	6.05E-02	5589	3.22E-04	Down	3.60	2799	1.22	0.35
rs11900835	84083	ZRANB3	2	135841081	6.05E-02	5590	3.96E-01	Up	0.85	9958	1.22	0.04
rs7593535	3625	INHBB	2	120802909	6.05E-02	5591	2.37E-04	Up	3.68	2690	1.22	0.36
rs1093040	339479	FAM5C	1	186994871	6.06E-02	5592	5.37E-01	Up	0.62	10992	1.22	0.03
rs4921248	7265	TTC1	5	159376384	6.06E-02	5593	1.84E-01	Down	1.33	8065	1.22	0.07
rs1013588	5470	PPEF2	4	77138122	6.06E-02	5594	1.07E-02	Up	2.55	4557	1.22	0.20
rs17281377	2157	F8		153630174	6.06E-02	5595	2.30E-04	Down	3.68	2679	1.22	0.36
rs6692041	93273	LEMD1	1	202166400	6.06E-02	5596	5.06E-01	Down	0.67	10756	1.22	0.03
rs12593575	10518	CIB2	15	76177964	6.07E-02	5597	1.64E-03	Down	3.15	3433	1.22	0.28
rs9956889	57132	CHMP1B	18	1263751	6.07E-02	5598	1.42E-01	Down	1.47	7604	1.22	0.08
rs9956889	56651	C18orf2	18	1263751	6.07E-02	5599	8.05E-01	Down	0.25	12701	1.22	0.01
rs1986011	27328	PCDH11X		91396910	6.08E-02	5600	2.50E-01	Up	1.15	8763	1.22	0.06
rs505404	23410	SIRT3	11	233268	6.08E-02	5601	3.21E-02	Down	2.14	5530	1.22	0.15
rs505404	5719	PSMD13	11	233268	6.08E-02	5602	4.48E-01	Up	0.76	10332	1.22	0.03
rs12132508	576	BAI2	1	31878562	6.08E-02	5603	8.27E-01	Up	0.22	12835	1.22	0.01
rs10900588	493	ATP2B4	1	200388471	6.08E-02	5604	9.68E-37	Down	12.66	31	1.22	3.60
rs928391	5546	PRCC	1	153589790	6.09E-02	5605	2.88E-06	Up	4.68	1728	1.22	0.55
rs928391	9047	SH2D2A	1	153589790	6.09E-02	5606	4.17E-01	Up	0.81	10100	1.22	0.04
rs4131092	283742	FAM98B	15	36525564	6.09E-02	5607	4.20E-02	Down	2.03	5797	1.22	0.14
rs1317577	399	RHOH	4	40019115	6.09E-02	5608	5.83E-01	Up	0.55	11331	1.22	0.02
rs13194280	55862	ECHDC1	6	127698792	6.09E-02	5609	5.15E-17	Down	8.38	308	1.22	1.63
rs1555322	10893	MMP24	20	33312595	6.10E-02	5610	8.46E-01	Up	0.19	12933	1.21	0.01
rs12149656	5257	PHKB	16	46095511	6.10E-02	5611	9.00E-08	Up	5.35	1282	1.21	0.70
rs6427229	57147	SCYL3	1	166611205	6.10E-02	5612	5.21E-01	Up	0.64	10869	1.21	0.03
rs1381640	140801	RPL10L	14	46197769	6.10E-02	5613	3.84E-03	Up	2.89	3905	1.21	0.24
rs9936464	10725	NFAT5	16	68201550	6.10E-02	5614	2.27E-01	Down	1.21	8530	1.21	0.06
rs591283	28971	C11orf67	11	77260391	6.10E-02	5615	4.22E-01	Up	0.80	10135	1.21	0.04
rs237889	5021	OXTR	3	8777483	6.11E-02	5616	7.40E-08	Up	5.38	1263	1.21	0.71
rs7158783	58533	SNX6	14	34137231	6.11E-02	5617	2.10E-01	Up	1.25	8348	1.21	0.07
rs2518805	26220	DGCR5	22	17350141	6.11E-02	5618	8.77E-01	Down	0.16	13096	1.21	0.01
rs2268086	22913	RALY	20	32112399	6.12E-02	5619	8.11E-01	Up	0.24	12729	1.21	0.01
rs17719440	3624	INHBA	7	41528183	6.12E-02	5620	3.36E-08	Up	5.52	1175	1.21	0.75
rs7611106	4292	MLH1	3	36992028	6.12E-02	5621	2.29E-04	Down	3.69	2678	1.21	0.36
rs1806516	9828	ARHGEF17	11	72681701	6.12E-02	5622	3.18E-03	Down	2.95	3810	1.21	0.25
rs9988929	10748	KLRA1	12	10649795	6.12E-02	5623	5.66E-02	Up	1.91	6151	1.21	0.12
rs10854711	9582	APOBEC3B	22	37699172	6.13E-02	5624	1.74E-01	Up	1.36	7954	1.21	0.08
rs1317648	3855	KRT7	12	50927395	6.13E-02	5625	1.29E-02	Down	2.49	4680	1.21	0.19
rs216606	51728	POLR3K	16	57194	6.13E-02	5626	5.91E-01	Down	0.54	11374	1.21	0.02
rs12504148	3600	IL15	4	142947708	6.13E-02	5627	3.50E-01	Down	0.93	9585	1.21	0.05
rs2705163	121273	C12orf54	12	47183381	6.13E-02	5628	1.81E-01	Down	1.34	8033	1.21	0.07
rs617369	84203	TXNDC2	18	9859011	6.14E-02	5629	5.45E-01	Down	0.60	11053	1.21	0.03
rs5761561	89781	HPS4	22	25206696	6.14E-02	5630	6.50E-01	Up	0.45	11771	1.21	0.02
rs7984378	2700	GJA3	13	19632420	6.14E-02	5631	9.08E-01	Down	0.12	13297	1.21	0.00
rs11855134	80031	SEMA6D	15	45834968	6.14E-02	5632	4.94E-05	Down	4.06	2281	1.21	0.43
rs1911793	285555	C4orf37	4	98863972	6.15E-02	5633	7.16E-01	Down	0.36	12166	1.21	0.01
rs12238437	9550	ATP6V1G1	9	114428020	6.15E-02	5634	1.12E-21	Up	9.59	166	1.21	2.10
rs5995668	60489	APOBEC3G	22	37809712	6.15E-02	5635	1.94E-05	Down	4.27	2084	1.21	0.47
rs11588930	9651	PLCH2	1	2440996	6.15E-02	5636	1.14E-01	Down	1.58	7202	1.21	0.09
rs1396829	4632	MYL1	2	211015381	6.15E-02	5637	1.55E-01	Up	1.42	7739	1.21	0.08
rs201933	631	BFSP1	20	17468898	6.16E-02	5638	8.80E-01	Down	0.15	13128	1.21	0.01
rs10498030	26154	ABCA12	2	215691081	6.16E-02	5639	1.91E-03	Up	3.10	3510	1.21	0.27
rs487467	8724	SNX3	6	108656745	6.18E-02	5640	1.65E-03	Down	3.15	3434	1.21	0.28
rs12407003	115209	OMA1	1	58651655	6.18E-02	5641	2.00E-01	Up	1.28	8252	1.21	0.07
rs11648723	255762	C16orf65	16	21892987	6.18E-02	5642	6.97E-02	Down	1.81	6418	1.21	0.12
rs11648723	7385	UQCRC2	16	21892987	6.18E-02	5643	7.06E-01	Up	0.38	12107	1.21	0.02
rs1010651	5641	LGMN	14	92261224	6.19E-02	5644	4.93E-03	Up	2.81	4057	1.21	0.23
rs1168968	1844	DUSP2	2	96247237	6.19E-02	5645	9.79E-01	Down	0.03	13765	1.21	0.00
rs16962786	4225	MEP1B	18	28027169	6.19E-02	5646	3.09E-02	Up	2.16	5492	1.21	0.15
rs7315108	196385	DNAH10	12	122862947	6.19E-02	5647	3.33E-02	Up	2.13	5573	1.21	0.15
rs2075713	90952	ESAM	11	124123149	6.20E-02	5648	1.31E-01	Down	1.51	7456	1.21	0.09
rs2075713	4900	NRGN	11	124123149	6.20E-02	5649	7.33E-01	Down	0.34	12274	1.21	0.01
rs876435	23221	RHOBTB2	8	22929478	6.20E-02	5650	1.49E-02	Down	2.44	4805	1.21	0.18
rs876435	8795	TNFRSF10B	8	22929478	6.20E-02	5651	2.21E-01	Down	1.22	8471	1.21	0.07
rs299216	1657	DMXL1	5	118424127	6.20E-02	5652	4.58E-01	Down	0.74	10406	1.21	0.03
rs10410589	1406	CRX	19	53016534	6.20E-02	5653	3.92E-01	Up	0.86	9923	1.21	0.04
rs1136553	23023	TMCC1	3	130849661	6.20E-02	5654	1.99E-01	Up	1.28	8248	1.21	0.07
rs4955720	5584	PRKCI	3	171511302	6.20E-02	5655	1.16E-04	Up	3.85	2492	1.21	0.39
rs7045407	195827	C9orf21	9	96514648	6.21E-02	5656	1.41E-01	Up	1.47	7582	1.21	0.09
rs3825393	83892	KCTD10	12	108346094	6.21E-02	5657	2.89E-10	Down	6.30	806	1.21	0.95
rs12565115	51440	HPCAL4	1	39837680	6.21E-02	5658	1.03E-01	Down	1.63	7049	1.21	0.10
rs3825393	283446	MYO1H	12	108346094	6.21E-02	5659	7.78E-01	Up	0.28	12541	1.21	0.01
rs7728378	1393	CRHBP	5	76295106	6.21E-02	5660	5.72E-02	Down	1.90	6164	1.21	0.12
rs2294642	26270	FBXO6	1	11655959	6.21E-02	5661	9.65E-04	Up	3.30	3207	1.21	0.30
rs2294642	26232	FBXO2	1	11655959	6.21E-02	5662	3.36E-03	Down	2.93	3835	1.21	0.25
rs2294642	93611	FBXO44	1	11655959	6.21E-02	5663	8.70E-01	Up	0.16	13060	1.21	0.01
rs4753598	10888	GPR83	11	93740661	6.21E-02	5664	7.74E-02	Down	1.77	6584	1.21	0.11
rs17172491	10268	RAMP3	7	44999882	6.22E-02	5665	4.96E-01	Down	0.68	10688	1.21	0.03

rs10405167	4298	MLLT1	19	6218757	6.22E-02	5666	9.40E-01	Down	0.08	13494	1.21	0.00
rs627497	10938	EHD1	11	64395739	6.22E-02	5667	2.30E-02	Down	2.27	5201	1.21	0.16
rs2811728	414332	LCN10	9	136918798	6.22E-02	5668	6.19E-03	Down	2.74	4184	1.21	0.22
rs2811728	158062	LCN6	9	136918798	6.22E-02	5669	1.69E-01	Down	1.38	7909	1.21	0.08
rs2811728	138307	LCN8	9	136918798	6.22E-02	5670	3.60E-01	Down	0.91	9679	1.21	0.04
rs2250350	79913	ACTR5	20	36803410	6.22E-02	5671	9.77E-01	Up	0.03	13744	1.21	0.00
rs2788135	25896	INTS7	1	208513655	6.22E-02	5672	2.30E-01	Up	1.20	8560	1.21	0.06
rs4696828	152992	C4orf23	4	8573675	6.23E-02	5673	3.13E-01	Down	1.01	9300	1.21	0.05
rs4517412	64081	PBLD	10	69733542	6.23E-02	5674	4.07E-05	Up	4.10	2232	1.21	0.44
rs4462945	9797	TATDN2	3	10246265	6.23E-02	5675	8.45E-02	Up	1.73	6710	1.21	0.11
rs4462945	3656	IRAK2	3	10246265	6.23E-02	5676	9.55E-01	Up	0.06	13589	1.21	0.00
rs893363	55349	CHDH	3	53822102	6.23E-02	5677	1.04E-04	Up	3.88	2463	1.21	0.40
rs12251688	1559	CYP2C9	10	96683717	6.23E-02	5678	4.58E-01	Up	0.74	10407	1.21	0.03
rs3815826	2734	GLG1	16	73066203	6.23E-02	5679	3.97E-06	Down	4.61	1780	1.21	0.54
rs11202429	9562	MINPP1	10	89266679	6.23E-02	5680	2.32E-02	Up	2.27	5212	1.21	0.16
rs4816375	337879	KRTAP8-1	21	31115424	6.24E-02	5681	4.58E-02	Up	2.00	5890	1.21	0.13
rs2274913	9295	SFRS11	1	70401236	6.24E-02	5682	6.59E-04	Up	3.41	3059	1.20	0.32
rs2274913	55631	LRRC40	1	70401236	6.24E-02	5683	2.09E-01	Up	1.26	8336	1.20	0.07
rs3801936	1738	DLD	7	107130368	6.24E-02	5684	1.58E-02	Down	2.41	4865	1.20	0.18
rs2651206	10591	C6orf108	6	43321455	6.24E-02	5685	1.71E-25	Up	10.43	106	1.20	2.48
rs2651206	84630	TTBK1	6	43321455	6.24E-02	5686	1.60E-01	Down	1.40	7819	1.20	0.08
rs9611602	50	ACO2	22	40232905	6.25E-02	5687	1.24E-01	Up	1.54	7352	1.20	0.09
rs3771188	8808	IL1RL2	2	102298866	6.25E-02	5688	7.81E-01	Down	0.28	12553	1.20	0.01
rs2045938	6579	SLCO1A2	12	21411619	6.25E-02	5689	1.88E-12	Up	7.04	582	1.20	1.17
rs6683705	2635	GBP3	1	89202728	6.25E-02	5690	4.76E-02	Down	1.98	5937	1.20	0.13
rs1542479	54769	DIRAS2	9	90452603	6.25E-02	5691	3.46E-02	Up	2.11	5612	1.20	0.15
rs1545040	24139	EML2	19	50821839	6.25E-02	5692	9.22E-03	Down	2.60	4447	1.20	0.20
rs13069684	23024	PDZRN3	3	73705307	6.25E-02	5693	9.77E-01	Up	0.03	13746	1.20	0.00
rs9997120	11157	LSM6	4	147450694	6.26E-02	5694	3.14E-09	Down	5.92	976	1.20	0.85
rs3792971	8418	CMAH	6	25207319	6.26E-02	5695	2.61E-02	Down	2.22	5328	1.20	0.16
rs776840	1447	CSN2	4	71016915	6.27E-02	5696	2.25E-01	Down	1.21	8512	1.20	0.06
rs776840	9318	COPS2	4	71016915	6.27E-02	5697	4.87E-01	Down	0.70	10626	1.20	0.03
rs9888197	3636	INPPL1	11	71604308	6.27E-02	5698	3.63E-01	Down	0.91	9705	1.20	0.04
rs9888197	2350	FOLR2	11	71604308	6.27E-02	5699	5.17E-01	Up	0.65	10841	1.20	0.03
rs1002082	1446	CSN1S1	4	70994731	6.27E-02	5700	6.56E-03	Up	2.72	4215	1.20	0.22
rs934093	80254	CEP63	3	135678583	6.27E-02	5701	2.86E-01	Down	1.07	9086	1.20	0.05
rs934093	25847	ANAPC13	3	135678583	6.27E-02	5702	4.04E-01	Up	0.83	10011	1.20	0.04
rs2582558	283638	KIAA0284	14	104418533	6.27E-02	5703	5.84E-01	Up	0.55	11338	1.20	0.02
rs4950887	5877	RABIF	1	199601987	6.28E-02	5704	1.94E-02	Up	2.34	5044	1.20	0.17
rs393766	23635	SSBP2	5	81084943	6.28E-02	5705	5.21E-11	Down	6.56	717	1.20	1.03
rs11696248	8785	MATN4	20	43384230	6.28E-02	5706	1.31E-02	Down	2.48	4697	1.20	0.19
rs11696248	6385	SDC4	20	43384230	6.28E-02	5707	5.52E-02	Down	1.92	6118	1.20	0.13
rs2056998	51567	TTRAP	6	24769578	6.28E-02	5708	1.69E-09	Down	6.03	929	1.20	0.88
rs2056998	9856	KIAA0319	6	24769578	6.28E-02	5709	3.94E-01	Down	0.85	9940	1.20	0.04
rs7214006	85291	KRTAP4-2	17	36600822	6.28E-02	5710	5.94E-01	Down	0.53	11398	1.20	0.02
rs2056998	55856	THEM2	6	24769578	6.28E-02	5711	7.32E-01	Down	0.34	12262	1.20	0.01
rs1489949	23272	C3orf63	3	56649230	6.29E-02	5712	6.78E-01	Up	0.42	11933	1.20	0.02
rs1202397	8427	ZNF282	7	148354275	6.29E-02	5713	1.38E-07	Up	5.27	1333	1.20	0.69
rs17124176	362	AQP5	12	48621895	6.29E-02	5714	7.31E-06	Down	4.48	1878	1.20	0.51
rs2853838	2662	GDF10	10	48051116	6.29E-02	5715	1.64E-01	Down	1.39	7861	1.20	0.08
rs2853838	2658	GDF2	10	48051116	6.29E-02	5716	3.38E-01	Down	0.96	9503	1.20	0.05
rs11961590	11131	CAPN11	6	44238848	6.30E-02	5717	5.61E-01	Up	0.58	11169	1.20	0.03
rs2384319	3797	KIF3C	2	26117906	6.30E-02	5718	2.51E-03	Down	3.02	3691	1.20	0.26
rs745986	240	ALOX5	10	45198914	6.30E-02	5719	9.21E-01	Up	0.10	13383	1.20	0.00
rs3217753	890	CCNA2	4	123103930	6.30E-02	5720	6.64E-07	Up	4.97	1514	1.20	0.62
rs3217753	5393	EXOSC9	4	123103930	6.30E-02	5721	1.01E-01	Up	1.64	7016	1.20	0.10
rs3217753	55212	BBS7	4	123103930	6.30E-02	5722	1.80E-01	Down	1.34	8016	1.20	0.07
rs10037670	79685	SAP30L	5	153791683	6.30E-02	5723	3.94E-03	Up	2.88	3917	1.20	0.24
rs9291949	6880	TAF9	5	68684526	6.31E-02	5724	3.57E-03	Up	2.91	3868	1.20	0.24
rs9291949	5884	RAD17	5	68684526	6.31E-02	5725	4.36E-01	Up	0.78	10242	1.20	0.04
rs1332948	9462	RASAL2	1	174894747	6.31E-02	5726	6.91E-01	Up	0.40	12014	1.20	0.02
rs244127	11142	PKIG	20	42605538	6.31E-02	5727	2.96E-08	Down	5.54	1164	1.20	0.75
rs551444	168090	C6orf118	6	165712776	6.31E-02	5728	6.36E-01	Down	0.47	11675	1.20	0.02
rs10797650	10401	PIAS3	1	143049520	6.32E-02	5729	1.62E-02	Down	2.41	4895	1.20	0.18
rs16853949	4194	MDM4	1	201254920	6.32E-02	5730	1.17E-02	Up	2.52	4619	1.20	0.19
rs2488320	3688	ITGB1	10	33238917	6.32E-02	5731	7.07E-05	Down	3.97	2363	1.20	0.42
rs1940935	4321	MMP12	11	102260225	6.32E-02	5732	1.68E-01	Up	1.38	7891	1.20	0.08
rs13290613	3933	LCN1	9	135632510	6.32E-02	5733	5.03E-01	Down	0.67	10738	1.20	0.03
rs7657630	166815	TIGD2	4	90384598	6.33E-02	5734	7.98E-07	Up	4.94	1546	1.20	0.61
rs10900555	5972	REN	1	200863967	6.33E-02	5735	7.43E-02	Down	1.78	6514	1.20	0.11
rs214590	221656	AOF1	6	18317561	6.33E-02	5736	4.90E-01	Down	0.69	10653	1.20	0.03
rs586355	5588	PRKCQ	10	6598194	6.33E-02	5737	1.56E-01	Down	1.42	7751	1.20	0.08
rs5945958	90843	TCEAL8		102306137	6.34E-02	5738	4.47E-01	Up	0.76	10326	1.20	0.03
rs10091690	137902	PXDNL	8	52421089	6.34E-02	5739	2.50E-01	Down	1.15	8767	1.20	0.06
rs11060762	23457	ABCB9	12	121928590	6.34E-02	5740	7.92E-01	Down	0.26	12623	1.20	0.01
rs3004318	29929	ALG6	1	63612760	6.34E-02	5741	8.50E-09	Up	5.76	1040	1.20	0.81
rs3004318	23421	ITGB3BP	1	63612760	6.34E-02	5742	5.72E-02	Up	1.90	6165	1.20	0.12
rs1994274	57633	LRRN1	3	3867716	6.34E-02	5743	3.48E-13	Up	7.30	506	1.20	1.25
rs537473	114571	SLC22A9	11	62929347	6.34E-02	5744	7.79E-01	Down	0.28	12545	1.20	0.01
rs757694	5814	PURB	7	44705778	6.34E-02	5745	1.39E-03	Up	3.20	3344	1.20	0.29
rs10743052	79608	RIC3	11	8131542	6.35E-02	5746	1.68E-01	Down	1.38	7899	1.20	0.08

rs3803463	1583	CYP11A1	15	72454562	6.35E-02	5747	7.96E-02	Down	1.75	6627	1.20	0.11
rs12046563	10465	PPIH	1	42806373	6.35E-02	5748	3.55E-04	Up	3.57	2841	1.20	0.35
rs4791762	23533	PIK3R5	17	8716222	6.35E-02	5749	1.38E-01	Down	1.48	7552	1.20	0.09
rs288027	1495	CTNNA1	5	138250229	6.36E-02	5750	5.17E-05	Down	4.05	2297	1.20	0.43
rs2304061	5159	PDGFRB	5	149483863	6.36E-02	5751	9.61E-01	Down	0.05	13630	1.20	0.00
rs1951017	145264	SERPINA12	14	94049036	6.36E-02	5752	8.23E-01	Down	0.22	12812	1.20	0.01
rs715180	3572	IL6ST	5	55333056	6.37E-02	5753	1.42E-23	Down	10.01	129	1.20	2.28
rs7975662	140807	KRT72	12	51292672	6.37E-02	5754	7.92E-01	Up	0.26	12624	1.20	0.01
rs2774941	6125	RPL5	1	93027914	6.37E-02	5755	2.07E-05	Up	4.26	2098	1.20	0.47
rs2207609	54516	MTRF1L	6	153393728	6.38E-02	5756	9.81E-01	Down	0.02	13776	1.20	0.00
rs1043550	611	OPN1SW	7	128003176	6.38E-02	5757	1.84E-01	Down	1.33	8062	1.20	0.07
rs741810	2521	FUS	16	31101443	6.38E-02	5758	1.22E-03	Up	3.23	3299	1.20	0.29
rs10133111	215	ABCD1	14	102447074	6.38E-02	5759	5.91E-01	Down	0.54	11381	1.20	0.02
rs10133111	81693	AMN	14	102447074	6.38E-02	5760	9.23E-01	Down	0.10	13388	1.20	0.00
rs5761603	8459	TPST2	22	25282885	6.38E-02	5761	9.53E-01	Up	0.06	13573	1.20	0.00
rs12278238	81930	KIF18A	11	28018835	6.38E-02	5762	7.10E-01	Up	0.37	12131	1.20	0.01
rs2241094	26189	OR1A2	17	3047552	6.38E-02	5763	2.23E-01	Down	1.22	8492	1.20	0.07
rs2241094	8383	OR1A1	17	3047552	6.38E-02	5764	8.77E-01	Down	0.15	13102	1.20	0.01
rs2073927	26301	GBGT1	9	133060900	6.38E-02	5765	2.29E-01	Down	1.20	8551	1.20	0.06
rs4236479	56302	TRPV5	7	142138175	6.38E-02	5766	3.20E-01	Down	1.00	9356	1.19	0.05
rs4236479	135927	C7orf34	7	142138175	6.38E-02	5767	4.56E-01	Down	0.75	10389	1.19	0.03
rs1048603	55230	USP40	2	234176487	6.38E-02	5768	3.30E-01	Down	0.97	9440	1.19	0.05
rs1701706	6821	SUOX	12	54688471	6.38E-02	5769	2.17E-04	Up	3.70	2656	1.19	0.37
rs881191	142940	TRUB1	10	116696951	6.39E-02	5770	9.28E-01	Up	0.09	13418	1.19	0.00
rs13007020	6335	SCN9A	2	166962768	6.39E-02	5771	4.49E-01	Down	0.76	10343	1.19	0.03
rs5755821	80830	APOL6	22	34366150	6.39E-02	5772	3.17E-01	Down	1.00	9341	1.19	0.05
rs3733890	635	BHMT	5	78457715	6.39E-02	5773	6.19E-01	Down	0.50	11572	1.19	0.02
rs4793211	10230	NBR2	17	38552381	6.39E-02	5774	1.99E-01	Down	1.28	8246	1.19	0.07
rs282170	6939	TCF15	20	541641	6.39E-02	5775	3.82E-01	Down	0.87	9856	1.19	0.04
rs4988515	3484	IGFBP1	7	45705840	6.39E-02	5776	9.20E-01	Down	0.10	13374	1.19	0.00
rs7735412	9685	CLINT1	5	157231272	6.40E-02	5777	4.00E-01	Up	0.84	9985	1.19	0.04
rs1921363	57502	NLGN4X		5727572	6.40E-02	5778	1.26E-03	Down	3.23	3317	1.19	0.29
rs4780754	5073	PARN	16	14617455	6.40E-02	5779	8.66E-01	Down	0.17	13037	1.19	0.01
rs7502772	6346	CCL1	17	29723177	6.40E-02	5780	5.10E-01	Up	0.66	10785	1.19	0.03
rs1356118	6779	STATH	4	71027696	6.40E-02	5781	1.40E-02	Up	2.46	4754	1.19	0.19
rs13119057	170712	COX7B2	4	46648429	6.40E-02	5782	2.73E-01	Up	1.10	8981	1.19	0.06
rs17400329	5218	PFTK1	7	90240346	6.41E-02	5783	7.11E-01	Down	0.37	12140	1.19	0.01
rs12128546	2046	EPHA8	1	22682400	6.41E-02	5784	5.95E-01	Down	0.53	11400	1.19	0.02
rs9619748	10521	DDX17	22	37224485	6.42E-02	5785	9.11E-01	Down	0.11	13315	1.19	0.00
rs6724157	81618	ITM2C	2	231575861	6.42E-02	5786	2.73E-25	Down	10.39	109	1.19	2.46
rs2799685	3898	LAD1	1	198100936	6.42E-02	5787	7.20E-08	Up	5.39	1260	1.19	0.71
rs2799685	7135	TNNI1	1	198100936	6.42E-02	5788	2.56E-01	Down	1.13	8825	1.19	0.06
rs4433884	25925	ZNF521	18	20970911	6.43E-02	5789	9.57E-01	Up	0.05	13598	1.19	0.00
rs7002654	5327	PLAT	8	42196227	6.44E-02	5790	8.38E-01	Up	0.20	12893	1.19	0.01
rs10502444	55364	IMPACT	18	20283377	6.44E-02	5791	5.32E-02	Up	1.93	6067	1.19	0.13
rs9461446	7741	ZNF187	6	28335612	6.44E-02	5792	7.37E-05	Down	3.96	2370	1.19	0.41
rs1391767	81565	NDEL1	17	8261713	6.44E-02	5793	6.04E-01	Down	0.52	11457	1.19	0.02
rs12424233	4440	MSI1	12	119263263	6.44E-02	5794	7.83E-03	Up	2.66	4326	1.19	0.21
rs6469845	6873	TAF2	8	120861376	6.44E-02	5795	3.29E-04	Up	3.59	2809	1.19	0.35
rs6590146	9638	FEZ1	11	124855039	6.44E-02	5796	3.11E-07	Down	5.12	1421	1.19	0.65
rs8106725	10298	PAK4	19	44303543	6.44E-02	5797	2.10E-03	Up	3.08	3562	1.19	0.27
rs710835	5593	PRKG2	4	82486109	6.44E-02	5798	1.90E-02	Down	2.35	5023	1.19	0.17
rs4766000	83714	NRIP2	12	2820765	6.45E-02	5799	2.43E-01	Down	1.17	8684	1.19	0.06
rs2208	55164	SHQ1	3	72954463	6.45E-02	5800	7.05E-01	Down	0.38	12101	1.19	0.02
rs11167753	1729	DIAPH1	5	140969594	6.46E-02	5801	5.77E-05	Up	4.02	2321	1.19	0.42
rs952319	79027	ZNF655	7	98816100	6.46E-02	5802	5.46E-22	Down	9.64	158	1.19	2.13
rs3119680	157922	CAMSAP1	9	136007461	6.46E-02	5803	3.42E-01	Up	0.95	9543	1.19	0.05
rs7562897	9290	GPR55	2	231593428	6.46E-02	5804	2.24E-01	Down	1.21	8501	1.19	0.06
rs7554192	6905	TBCE	1	231849108	6.46E-02	5805	1.26E-03	Up	3.23	3316	1.19	0.29
rs1933695	5997	RGS2	1	189496477	6.46E-02	5806	3.44E-05	Down	4.14	2197	1.19	0.45
rs8110742	4782	NFIC	19	3392385	6.47E-02	5807	2.10E-03	Down	3.08	3561	1.19	0.27
rs307238	84280	BTBD10	11	13451340	6.47E-02	5808	9.23E-04	Up	3.31	3184	1.19	0.30
rs11595566	118788	PIK3AP1	10	98426814	6.48E-02	5809	1.12E-03	Up	3.26	3261	1.19	0.30
rs4673048	23704	KCNE4	2	223737008	6.48E-02	5810	8.87E-01	Down	0.14	13168	1.19	0.01
rs17601573	85481	PSKH2	8	87135695	6.48E-02	5811	6.21E-01	Up	0.49	11586	1.19	0.02
rs2018904	10006	ABI1	10	27121908	6.49E-02	5812	1.17E-01	Up	1.57	7244	1.19	0.09
rs2658479	6231	RPS26	12	54703670	6.49E-02	5813	8.89E-01	Up	0.14	13184	1.19	0.01
rs4823458	7380	UPK3A	22	44014365	6.49E-02	5814	5.75E-01	Down	0.56	11280	1.19	0.02
rs3219281	7376	NR1H2	19	55578899	6.49E-02	5815	1.19E-06	Down	4.86	1600	1.19	0.59
rs3895665	2248	FGF3	11	69346074	6.49E-02	5816	3.17E-01	Up	1.00	9339	1.19	0.05
rs329134	51208	CLDN18	3	139237251	6.50E-02	5817	1.57E-01	Up	1.42	7762	1.19	0.08
rs17069904	8792	TNFRSF11A	18	58183929	6.50E-02	5818	4.36E-01	Down	0.78	10243	1.19	0.04
rs415382	1114	CHGB	20	5858339	6.50E-02	5819	5.59E-03	Down	2.77	4142	1.19	0.23
rs4129871	6197	RPS6KA3		19984850	6.50E-02	5820	5.02E-01	Down	0.67	10728	1.19	0.03
rs7064629	5160	PDHA1		19113104	6.51E-02	5821	1.49E-01	Up	1.44	7670	1.19	0.08
rs11870961	85302	FBF1	17	71429859	6.51E-02	5822	2.63E-01	Down	1.12	8889	1.19	0.06
rs11870961	64978	MRPL38	17	71429859	6.51E-02	5823	9.82E-01	Down	0.02	13785	1.19	0.00
rs1497290	11096	ADAMTS5	21	27269954	6.51E-02	5824	7.81E-06	Down	4.47	1895	1.19	0.51
rs651084	26285	CLDN17	21	30480187	6.51E-02	5825	5.23E-01	Down	0.64	10876	1.19	0.03
rs1654671	2906	GRIN2D	19	53616775	6.51E-02	5826	7.86E-01	Up	0.27	12583	1.19	0.01
rs924233	8467	SMARCA5	4	144837618	6.52E-02	5827	7.03E-01	Down	0.38	12089	1.19	0.02

rs2276401	6708	SPTA1	1	155437852	6.52E-02	5828	5.70E-01	Down	0.57	11240	1.19	0.02
rs13336445	10232	MSLN	16	744761	6.52E-02	5829	5.62E-01	Down	0.58	11176	1.19	0.03
rs7999006	10426	TUBGCP3	13	112308712	6.52E-02	5830	2.05E-03	Down	3.08	3544	1.19	0.27
rs10812326	7436	VLDLR	9	2593361	6.53E-02	5831	3.26E-02	Up	2.14	5544	1.19	0.15
rs7669431	51778	MYOZ2	4	120434823	6.53E-02	5832	1.71E-01	Up	1.37	7934	1.19	0.08
rs848596	9637	FEZ2	2	36715514	6.53E-02	5833	5.49E-07	Down	5.01	1494	1.19	0.63
rs4714028	23787	MTCH1	6	37063866	6.53E-02	5834	2.20E-02	Down	2.29	5157	1.19	0.17
rs4788805	7567	ZNF19	16	70072139	6.53E-02	5835	1.38E-01	Down	1.48	7554	1.18	0.09
rs4788805	7571	ZNF23	16	70072139	6.53E-02	5836	7.11E-01	Down	0.37	12135	1.18	0.01
rs1884082	12	SERPINA3	14	94148430	6.54E-02	5837	1.96E-01	Up	1.29	8217	1.18	0.07
rs13152353	4887	NPY2R	4	156472893	6.54E-02	5838	4.69E-01	Up	0.72	10495	1.18	0.03
rs809359	26528	DAZAP1	19	1342809	6.54E-02	5839	1.11E-02	Up	2.54	4580	1.18	0.20
rs809359	2593	GAMT	19	1342809	6.54E-02	5840	1.69E-02	Up	2.39	4930	1.18	0.18
rs809359	374291	NDUFS7	19	1342809	6.54E-02	5841	9.03E-02	Down	1.69	6831	1.18	0.10
rs12253113	9060	PAPSS2	10	89502819	6.54E-02	5842	2.51E-02	Down	2.24	5293	1.18	0.16
rs2232228	3038	HAS3	16	67701078	6.55E-02	5843	6.05E-01	Down	0.52	11469	1.18	0.02
rs660541	5241	PGR	11	100439577	6.55E-02	5844	1.62E-01	Up	1.40	7840	1.18	0.08
rs2910333	56122	PCDHB14	5	140576888	6.55E-02	5845	1.05E-03	Up	3.28	3244	1.18	0.30
rs2910333	56124	PCDHB12	5	140576888	6.55E-02	5846	1.05E-01	Up	1.62	7079	1.18	0.10
rs2910333	56123	PCDHB13	5	140576888	6.55E-02	5847	1.31E-01	Up	1.51	7457	1.18	0.09
rs2910333	54660	PCDHB18	5	140576888	6.55E-02	5848	2.56E-01	Up	1.13	8826	1.18	0.06
rs6940522	57531	HACE1	6	105305511	6.55E-02	5849	4.37E-01	Up	0.78	10249	1.18	0.04
rs369081	57553	MICAL3	22	16661226	6.55E-02	5850	6.32E-03	Down	2.73	4194	1.18	0.22
rs2110410	23369	PUM2	2	20367267	6.56E-02	5851	9.67E-01	Up	0.04	13676	1.18	0.00
rs7168671	3480	IGF1R	15	97272485	6.56E-02	5852	7.87E-01	Up	0.27	12593	1.18	0.01
rs885883	28378	IGHV7-81	14	106345097	6.56E-02	5853	9.28E-01	Up	0.09	13417	1.18	0.00
rs2056993	6755	SSTR5	16	1049654	6.57E-02	5854	6.94E-01	Down	0.39	12033	1.18	0.02
rs13023583	4211	MEIS1	2	66637591	6.57E-02	5855	1.35E-27	Down	10.89	81	1.18	2.69
rs12369972	26290	GALNT8	12	4753436	6.58E-02	5856	8.20E-01	Down	0.23	12790	1.18	0.01
rs9534381	80183	C13orf18	13	45832200	6.58E-02	5857	2.93E-01	Up	1.05	9142	1.18	0.05
rs9844797	131669	UROC1	3	127703237	6.58E-02	5858	4.99E-02	Down	1.96	5994	1.18	0.13
rs3007711	117145	THEM4	1	148710450	6.58E-02	5859	1.02E-02	Up	2.57	4519	1.18	0.20
rs2466583	26497	OR10D3P	11	123564401	6.58E-02	5860	4.30E-01	Up	0.79	10193	1.18	0.04
rs6495864	84529	C15orf41	15	34738925	6.58E-02	5861	4.66E-09	Down	5.86	1002	1.18	0.83
rs7359902	1264	CNN1	19	11522890	6.58E-02	5862	1.70E-23	Down	9.99	130	1.18	2.28
rs665677	219844	HYLS1	11	125257371	6.58E-02	5863	2.05E-04	Up	3.71	2639	1.18	0.37
rs252109	51294	PCDH12	5	141319072	6.58E-02	5864	1.41E-01	Down	1.47	7591	1.18	0.08
rs252109	9604	RNF14	5	141319072	6.58E-02	5865	1.95E-01	Down	1.30	8203	1.18	0.07
rs9965554	10892	MALT1	18	54473838	6.59E-02	5866	4.31E-01	Up	0.79	10202	1.18	0.04
rs10917696	8490	RGS5	1	159880983	6.59E-02	5867	9.36E-05	Up	3.91	2433	1.18	0.40
rs10156646	7270	TTF1	9	132321909	6.59E-02	5868	9.39E-01	Down	0.08	13491	1.18	0.00
rs673526	64776	C11orf1	11	111239353	6.59E-02	5869	4.24E-06	Up	4.60	1788	1.18	0.54
rs4076358	23513	SCRIB	8	144982227	6.59E-02	5870	5.90E-04	Up	3.44	3022	1.18	0.32
rs4076358	22827	PUF60	8	144982227	6.59E-02	5871	2.37E-02	Up	2.26	5227	1.18	0.16
rs6584984	10285	SMNDC1	10	112072982	6.59E-02	5872	6.13E-01	Down	0.51	11525	1.18	0.02
rs7200879	54620	FBXL19	16	30855073	6.59E-02	5873	6.47E-01	Down	0.46	11758	1.18	0.02
rs11037921	60529	ALX4	11	44243624	6.59E-02	5874	2.01E-01	Down	1.28	8263	1.18	0.07
rs7963744	55729	ATF7IP	12	14541339	6.60E-02	5875	8.49E-02	Down	1.72	6720	1.18	0.11
rs10593	51692	CPSF3	2	9496628	6.60E-02	5876	2.92E-05	Up	4.18	2164	1.18	0.45
rs10593	9270	ITGB1BP1	2	9496628	6.60E-02	5877	2.37E-01	Down	1.18	8631	1.18	0.06
rs2112820	1054	CEBPG	19	38553928	6.60E-02	5878	1.28E-01	Down	1.52	7417	1.18	0.09
rs10937615	54360	CYTL1	4	5141184	6.60E-02	5879	2.10E-03	Down	3.08	3563	1.18	0.27
rs7580273	55577	NAGK	2	71188353	6.61E-02	5880	4.02E-01	Down	0.84	9992	1.18	0.04
rs6667701	343099	CCDC18	1	93451249	6.61E-02	5881	1.27E-01	Up	1.53	7394	1.18	0.09
rs7868158	79886	C9orf82	9	26850358	6.61E-02	5882	2.32E-02	Up	2.27	5211	1.18	0.16
rs629508	143686	SESN3	11	94562047	6.61E-02	5883	1.17E-06	Up	4.86	1596	1.18	0.59
rs1865373	165100	C2orf57	2	232281518	6.62E-02	5884	1.34E-02	Down	2.47	4719	1.18	0.19
rs12080886	9859	CEP170	1	239712077	6.62E-02	5885	1.31E-01	Up	1.51	7458	1.18	0.09
rs2229594	570	BAAT	9	101204219	6.62E-02	5886	4.51E-01	Up	0.75	10352	1.18	0.03
rs942152	51552	RAB14	9	121031239	6.63E-02	5887	4.85E-01	Up	0.70	10610	1.18	0.03
rs6115792	22888	UBOX5	20	3042908	6.63E-02	5888	3.20E-02	Down	2.14	5527	1.18	0.15
rs7645033	54625	PARP14	3	123920011	6.63E-02	5889	8.29E-01	Up	0.22	12845	1.18	0.01
rs1901531	567	B2M	15	42792673	6.64E-02	5890	7.93E-09	Down	5.77	1038	1.18	0.81
rs2863098	81621	KAZALD1	10	102800705	6.64E-02	5891	3.62E-01	Down	0.91	9697	1.18	0.04
rs17422	3054	HCFC1		152748273	6.64E-02	5892	3.78E-02	Up	2.08	5701	1.18	0.14
rs12073497	5686	PSMA5	1	109686492	6.64E-02	5893	9.79E-04	Up	3.30	3212	1.18	0.30
rs2352958	79230	ZNF557	19	7035747	6.65E-02	5894	5.38E-02	Up	1.93	6082	1.18	0.13
rs1122634	64847	SPATA20	17	45976364	6.65E-02	5895	1.76E-01	Up	1.35	7971	1.18	0.08
rs1122634	55040	EPN3	17	45976364	6.65E-02	5896	9.44E-01	Down	0.07	13517	1.18	0.00
rs4782247	79838	TMC5	16	19363116	6.65E-02	5897	3.46E-05	Up	4.14	2201	1.18	0.45
rs16950058	23409	SIRT4	12	119213237	6.65E-02	5898	2.07E-02	Down	2.31	5100	1.18	0.17
rs16950058	5319	PLA2G1B	12	119213237	6.65E-02	5899	1.23E-01	Down	1.54	7347	1.18	0.09
rs11647700	84127	RUNDC2A	16	12016244	6.65E-02	5900	5.60E-01	Up	0.58	11166	1.18	0.03
rs1925032	55243	KIRREL	1	154844233	6.66E-02	5901	2.44E-02	Down	2.25	5266	1.18	0.16
rs2523123	6863	TAC1	7	96993500	6.66E-02	5902	1.66E-07	Up	5.23	1353	1.18	0.68
rs1472836	63875	MRPL17	11	6674184	6.66E-02	5903	9.65E-06	Up	4.42	1945	1.18	0.50
rs1064721	23463	ICMT	1	6215643	6.66E-02	5904	1.16E-01	Down	1.57	7233	1.18	0.09
rs11265544	7391	USF1	1	157819092	6.66E-02	5905	3.77E-02	Down	2.08	5700	1.18	0.14
rs11265544	50848	F11R	1	157819092	6.66E-02	5906	1.25E-01	Down	1.54	7368	1.18	0.09
rs4130339	5606	MAP2K3	17	21108730	6.67E-02	5907	6.94E-03	Up	2.70	4254	1.18	0.22
rs16856148	85414	SLC45A3	1	202387382	6.67E-02	5908	6.18E-03	Up	2.74	4183	1.18	0.22

rs2883739	79609	C14orf138	14	49649885	6.67E-02	5909	5.20E-01	Down	0.64	10864	1.18	0.03
rs2516835	51263	MRPL30	2	99237649	6.67E-02	5910	2.53E-03	Up	3.02	3696	1.18	0.26
rs2516835	129531	MITD1	2	99237649	6.67E-02	5911	2.83E-02	Up	2.19	5405	1.18	0.15
rs2516835	51601	LIPT1	2	99237649	6.67E-02	5912	3.74E-01	Up	0.89	9796	1.18	0.04
rs6914422	578	BAK1	6	33642858	6.68E-02	5913	9.09E-02	Up	1.69	6845	1.18	0.10
rs2398180	7026	NR2F2	15	94664173	6.68E-02	5914	2.06E-04	Down	3.71	2641	1.18	0.37
rs6052462	146	ADRA1D	20	4183594	6.68E-02	5915	2.26E-01	Down	1.21	8520	1.18	0.06
rs1798192	27198	GPR81	12	121725648	6.69E-02	5916	9.21E-01	Down	0.10	13381	1.17	0.00
rs7334585	23111	SPG20	13	35773496	6.69E-02	5917	1.30E-26	Down	10.68	90	1.17	2.59
rs8121782	22919	MAPRE1	20	30893499	6.69E-02	5918	2.28E-15	Down	7.92	376	1.17	1.46
rs4460629	200185	KRTCAP2	1	151948408	6.70E-02	5919	4.10E-03	Up	2.87	3948	1.17	0.24
rs4460629	80128	TRIM46	1	151948408	6.70E-02	5920	8.44E-01	Up	0.20	12924	1.17	0.01
rs12519658	57688	ZSWIM6	5	60867496	6.70E-02	5921	1.39E-05	Down	4.35	2022	1.17	0.49
rs6873326	29958	DMGDH	5	78387268	6.70E-02	5922	5.36E-01	Up	0.62	10984	1.17	0.03
rs6873326	23743	BHMT2	5	78387268	6.70E-02	5923	9.80E-01	Down	0.02	13774	1.17	0.00
rs2120182	64232	MS4A5	11	59968873	6.70E-02	5924	1.89E-01	Down	1.31	8143	1.17	0.07
rs1672997	53827	FXYD5	19	40322202	6.70E-02	5925	1.73E-03	Down	3.13	3453	1.17	0.28
rs1672997	53822	FXYD7	19	40322202	6.70E-02	5926	7.83E-01	Up	0.28	12564	1.17	0.01
rs7933280	6288	SAA1	11	18228505	6.70E-02	5927	7.79E-03	Down	2.66	4324	1.17	0.21
rs2405900	161753	ODF3L1	15	73813862	6.71E-02	5928	4.42E-01	Down	0.77	10280	1.17	0.04
rs1991442	79858	NEK11	3	132450879	6.71E-02	5929	2.24E-02	Down	2.28	5180	1.17	0.16
rs13197574	7718	ZNF165	6	28168218	6.71E-02	5930	1.72E-02	Up	2.38	4945	1.17	0.18
rs2796269	822	CAPG	1	204325281	6.72E-02	5931	3.85E-05	Down	4.12	2219	1.17	0.44
rs10973482	22844	FRMPD1	9	37665113	6.72E-02	5932	6.26E-01	Down	0.49	11622	1.17	0.02
rs12201030	7432	VIP	6	153168903	6.72E-02	5933	3.45E-01	Up	0.95	9555	1.17	0.05
rs10997817	56521	DNAJC12	10	69233817	6.72E-02	5934	7.89E-04	Up	3.36	3130	1.17	0.31
rs1028488	28514	DLL1	6	170508107	6.72E-02	5935	2.43E-01	Down	1.17	8687	1.17	0.06
rs10778656	23603	CORO1C	12	107636054	6.72E-02	5936	3.79E-07	Down	5.08	1448	1.17	0.64
rs11609862	160777	CCDC60	12	118352254	6.73E-02	5937	2.20E-01	Down	1.23	8460	1.17	0.07
rs3766160	842	CASP9	1	15554178	6.74E-02	5938	4.92E-04	Down	3.49	2955	1.17	0.33
rs6912923	25803	SPDEF	6	34646522	6.74E-02	5939	4.45E-01	Up	0.76	10308	1.17	0.04
rs277414	26272	FBXO4	5	41963180	6.74E-02	5940	1.07E-03	Up	3.27	3250	1.17	0.30
rs17035154	5195	PEX14	1	10519581	6.74E-02	5941	3.03E-06	Down	4.67	1738	1.17	0.55
rs2430212	90293	KLHL13		116869938	6.75E-02	5942	6.41E-08	Down	5.41	1247	1.17	0.72
rs4886725	161514	TBC1D21	15	71958059	6.75E-02	5943	5.69E-01	Up	0.57	11232	1.17	0.02
rs10860738	121599	SPIC	12	100370465	6.75E-02	5944	2.41E-01	Down	1.17	8660	1.17	0.06
rs7867942	9355	LHX2	9	123850875	6.75E-02	5945	5.42E-01	Down	0.61	11027	1.17	0.03
rs12462101	374918	IGFL1	19	51426846	6.76E-02	5946	1.85E-01	Down	1.33	8080	1.17	0.07
rs11595278	9886	RHOBTB1	10	62346060	6.76E-02	5947	7.90E-02	Down	1.76	6614	1.17	0.11
rs2884731	7325	UBE2E2	3	23333150	6.76E-02	5948	1.62E-04	Up	3.77	2568	1.17	0.38
rs11680810	50618	ITSN2	2	24408547	6.76E-02	5949	2.18E-04	Down	3.70	2662	1.17	0.37
rs2117286	166793	ZNF509	4	4405868	6.76E-02	5950	2.63E-01	Up	1.12	8886	1.17	0.06
rs7649848	10289	EIF1B	3	40316715	6.77E-02	5951	2.25E-06	Down	4.73	1686	1.17	0.56
rs560978	23052	ENDOD1	11	94483564	6.77E-02	5952	7.95E-01	Down	0.26	12639	1.17	0.01
rs12408586	914	CD2	1	116994769	6.77E-02	5953	1.16E-01	Up	1.57	7234	1.17	0.09
rs755535	2180	ACSL1	4	186043524	6.77E-02	5954	7.86E-03	Up	2.66	4330	1.17	0.21
rs7334063	283518	KCNRG	13	49467917	6.77E-02	5955	7.43E-01	Down	0.33	12332	1.17	0.01
rs287234	23358	USP24	1	55378541	6.78E-02	5956	6.90E-01	Down	0.40	12007	1.17	0.02
rs7006363	27085	MTBP	8	121565330	6.78E-02	5957	4.64E-01	Up	0.73	10458	1.17	0.03
rs594942	5331	PLCB3	11	63762868	6.79E-02	5958	1.12E-02	Down	2.54	4585	1.17	0.20
rs443751	65260	C1orf163	1	52865453	6.79E-02	5959	5.86E-02	Up	1.89	6208	1.17	0.12
rs7221979	10238	WDR68	17	58968249	6.79E-02	5960	1.51E-14	Up	7.71	417	1.17	1.38
rs4150355	2073	ERCC5	13	102321313	6.80E-02	5961	1.22E-01	Down	1.55	7333	1.17	0.09
rs10219424	53340	SPA17	11	124070598	6.80E-02	5962	4.35E-03	Up	2.85	3980	1.17	0.24
rs2124910	946	SIGLEC6	19	56717059	6.80E-02	5963	2.83E-01	Up	1.07	9058	1.17	0.05
rs3741920	10536	LEPREL2	12	6809133	6.80E-02	5964	1.74E-06	Down	4.78	1649	1.17	0.58
rs3741920	920	CD4	12	6809133	6.80E-02	5965	3.18E-01	Up	1.00	9346	1.17	0.05
rs3741920	2784	GNB3	12	6809133	6.80E-02	5966	4.29E-01	Down	0.79	10185	1.17	0.04
rs3741920	27239	GPR162	12	6809133	6.80E-02	5967	4.48E-01	Down	0.76	10330	1.17	0.03
rs4320728	127294	MYOM3	1	24154471	6.81E-02	5968	8.87E-01	Up	0.14	13174	1.17	0.01
rs11741807	1958	EGR1	5	137833551	6.81E-02	5969	8.50E-04	Up	3.34	3153	1.17	0.31
rs6657799	5996	RGS1	1	189284257	6.81E-02	5970	6.46E-03	Up	2.72	4208	1.17	0.22
rs4951247	2005	ELK4	1	202328978	6.81E-02	5971	2.47E-03	Up	3.03	3679	1.17	0.26
rs4948564	53828	FXYD4	10	43192004	6.81E-02	5972	3.82E-01	Up	0.87	9851	1.17	0.04
rs11080149	4763	NF1	17	26647414	6.81E-02	5973	4.51E-01	Down	0.75	10348	1.17	0.03
rs11080149	2124	EVI2B	17	26647414	6.81E-02	5974	4.76E-01	Down	0.71	10548	1.17	0.03
rs11080149	4974	OMG	17	26647414	6.81E-02	5975	6.09E-01	Down	0.51	11496	1.17	0.02
rs5761618	1414	CRYBB1	22	25318260	6.82E-02	5976	3.45E-01	Down	0.94	9562	1.17	0.05
rs2238599	8943	AP3D1	19	2091009	6.82E-02	5977	1.55E-01	Up	1.42	7742	1.17	0.08
rs4361456	7466	WFS1	4	6374701	6.82E-02	5978	6.78E-13	Down	7.18	539	1.17	1.22
rs549	114827	FHAD1	1	15292131	6.83E-02	5979	7.36E-01	Down	0.34	12289	1.17	0.01
rs7905091	84171	LOXL4	10	100036068	6.83E-02	5980	8.64E-02	Down	1.71	6746	1.17	0.11
rs3742257	8600	TNFSF11	13	42071198	6.83E-02	5981	5.74E-01	Down	0.56	11272	1.17	0.02
rs1476868	673	BRAF	7	139958373	6.83E-02	5982	2.70E-07	Up	5.14	1402	1.17	0.66
rs7316996	5611	DNAJC3	13	95126839	6.84E-02	5983	2.09E-01	Up	1.26	8339	1.17	0.07
rs4073422	3993	LLGL2	17	71053605	6.84E-02	5984	4.48E-03	Up	2.84	3990	1.16	0.23
rs2068888	1592	CYP26A1	10	94829632	6.84E-02	5985	8.72E-01	Up	0.16	13079	1.16	0.01
rs7358562	57696	DDX55	12	122621125	6.84E-02	5986	9.90E-06	Up	4.42	1949	1.16	0.50
rs7358562	1967	EIF2B1	12	122621125	6.84E-02	5987	5.93E-02	Up	1.89	6221	1.16	0.12
rs2588233	6542	SLC7A2	8	17436984	6.84E-02	5988	1.78E-04	Down	3.75	2590	1.16	0.37
rs3116534	29851	ICOS	2	204659275	6.84E-02	5989	7.41E-01	Down	0.33	12323	1.16	0.01

rs13156156	285605	DTWD2	5	118313295	6.84E-02	5990	1.10E-02	Up	2.54	4571	1.16	0.20
rs232712	8603	C4orf8	4	2781205	6.85E-02	5991	4.22E-02	Up	2.03	5802	1.16	0.14
rs232712	79155	TNIP2	4	2781205	6.85E-02	5992	7.03E-01	Down	0.38	12093	1.16	0.02
rs1620996	6659	SOX4	6	21710531	6.85E-02	5993	3.20E-44	Up	13.98	10	1.16	4.35
rs7263489	3704	ITPA	20	3124072	6.85E-02	5994	7.70E-04	Up	3.36	3121	1.16	0.31
rs7283354	2618	GART	21	33798940	6.85E-02	5995	1.60E-10	Up	6.40	786	1.16	0.98
rs3750729	23560	GTPBP4	10	1058802	6.85E-02	5996	1.70E-09	Up	6.02	932	1.16	0.88
rs3750729	91734	IDI2	10	1058802	6.85E-02	5997	4.20E-03	Up	2.86	3968	1.16	0.24
rs3750729	55853	C10orf110	10	1058802	6.85E-02	5998	5.77E-01	Down	0.56	11290	1.16	0.02
rs12997022	1134	CHRNA1	2	175445481	6.86E-02	5999	5.57E-01	Up	0.59	11129	1.16	0.03
rs2479418	255738	PCSK9	1	55206898	6.86E-02	6000	2.85E-01	Down	1.07	9073	1.16	0.05
rs9660710	254173	TTLL10	1	1139265	6.86E-02	6001	8.36E-01	Down	0.21	12880	1.16	0.01
rs11650416	91608	RASL10B	17	31101541	6.86E-02	6002	6.89E-02	Down	1.82	6408	1.16	0.12
rs10818708	26219	OR1J4	9	122369075	6.87E-02	6003	7.71E-01	Up	0.29	12501	1.16	0.01
rs999885	9179	AP4M1	7	99345827	6.88E-02	6004	1.21E-05	Up	4.38	1994	1.16	0.49
rs999885	245812	CNPY4	7	99345827	6.88E-02	6005	8.41E-03	Down	2.64	4377	1.16	0.21
rs999885	4176	MCM7	7	99345827	6.88E-02	6006	1.42E-02	Up	2.45	4767	1.16	0.18
rs999885	6878	TAF6	7	99345827	6.88E-02	6007	5.41E-02	Down	1.93	6091	1.16	0.13
rs1004243	6525	SMTN	22	29822433	6.88E-02	6008	1.34E-28	Down	11.09	76	1.16	2.79
rs9803868	51702	PADI3	1	17323859	6.88E-02	6009	2.29E-01	Down	1.20	8549	1.16	0.06
rs758053	399671	HEATR4	14	73056667	6.88E-02	6010	6.38E-02	Down	1.85	6320	1.16	0.12
rs2840044	5193	PEX12	17	30916181	6.88E-02	6011	2.94E-05	Down	4.18	2166	1.16	0.45
rs11234962	65084	TMEM135	11	86484231	6.88E-02	6012	1.10E-02	Up	2.54	4578	1.16	0.20
rs10911704	10625	IVNS1ABP	1	182023083	6.88E-02	6013	6.99E-01	Down	0.39	12068	1.16	0.02
rs10462020	8863	PER3	1	7814949	6.89E-02	6014	2.56E-01	Down	1.14	8816	1.16	0.06
rs2181617	1361	CPB2	13	45571318	6.90E-02	6015	3.82E-01	Up	0.87	9847	1.16	0.04
rs606458	5837	PYGM	11	64302967	6.90E-02	6016	5.21E-11	Down	6.56	716	1.16	1.03
rs10502666	25941	C18orf10	18	32677391	6.90E-02	6017	5.00E-01	Down	0.67	10717	1.16	0.03
rs11845875	122481	AK7	14	96002882	6.91E-02	6018	6.39E-01	Up	0.47	11696	1.16	0.02
rs7971575	84102	SLC41A2	12	103727973	6.92E-02	6019	4.14E-01	Up	0.82	10082	1.16	0.04
rs714073	64663	SPANXC		140076432	6.92E-02	6020	9.22E-01	Down	0.10	13387	1.16	0.00
rs7426091	10461	MERTK	2	112361481	6.92E-02	6021	7.82E-04	Up	3.36	3126	1.16	0.31
rs7426091	64682	ANAPC1	2	112361481	6.92E-02	6022	6.38E-01	Up	0.47	11687	1.16	0.02
rs17192622	8417	STX7	6	132867757	6.92E-02	6023	4.63E-02	Down	1.99	5902	1.16	0.13
rs2092324	79180	EFHD2	1	15478700	6.92E-02	6024	9.79E-05	Down	3.90	2446	1.16	0.40
rs16908439	51202	DDX47	12	12864687	6.92E-02	6025	8.16E-03	Down	2.65	4354	1.16	0.21
rs7337571	55213	RCBTB1	13	49052554	6.93E-02	6026	1.49E-02	Up	2.43	4809	1.16	0.18
rs7613767	64343	AZI2	3	28338245	6.93E-02	6027	9.51E-01	Up	0.06	13566	1.16	0.00
rs4785239	6299	SALL1	16	49758032	6.93E-02	6028	7.72E-02	Down	1.77	6578	1.16	0.11
rs534654	55858	TMEM165	4	56131148	6.94E-02	6029	5.26E-01	Down	0.63	10915	1.16	0.03
rs534654	9575	CLOCK	4	56131148	6.94E-02	6030	6.26E-01	Up	0.49	11624	1.16	0.02
rs9894638	7484	WNT9B	17	42267900	6.94E-02	6031	3.85E-01	Up	0.87	9874	1.16	0.04
rs1852598	348980	HCN1	5	45638733	6.94E-02	6032	8.14E-02	Up	1.74	6658	1.16	0.11
rs1273522	126410	CYP4F22	19	15501972	6.94E-02	6033	1.34E-01	Down	1.50	7483	1.16	0.09
rs4420029	23139	MAST2	1	46137520	6.94E-02	6034	1.30E-02	Up	2.48	4686	1.16	0.19
rs651164	6580	SLC22A1	6	160551785	6.95E-02	6035	9.28E-01	Up	0.09	13416	1.16	0.00
rs730283	9925	ZBTB5	9	37473926	6.95E-02	6036	4.13E-01	Down	0.82	10077	1.16	0.04
rs730283	64425	POLR1E	9	37473926	6.95E-02	6037	4.98E-01	Up	0.68	10697	1.16	0.03
rs17441930	4121	MAN1A1	6	119540949	6.95E-02	6038	4.11E-01	Down	0.82	10063	1.16	0.04
rs7593348	1803	DPP4	2	162762494	6.95E-02	6039	5.69E-02	Down	1.90	6156	1.16	0.12
rs6927712	10370	CITED2	6	139747915	6.95E-02	6040	5.49E-02	Up	1.92	6111	1.16	0.13
rs2230344	2838	GPR15	3	99733676	6.96E-02	6041	9.18E-01	Down	0.10	13367	1.16	0.00
rs7861101	286234	C9orf79	9	87707361	6.96E-02	6042	8.98E-01	Up	0.13	13233	1.16	0.00
rs12450478	3691	ITGB4	17	71249042	6.96E-02	6043	3.66E-13	Down	7.27	517	1.16	1.24
rs6124761	63925	ZNF335	20	44048376	6.96E-02	6044	5.25E-01	Down	0.64	10903	1.16	0.03
rs694424	22997	IGSF9B	11	133329749	6.96E-02	6045	1.49E-01	Down	1.44	7664	1.16	0.08
rs1477320	2299	FOXI1	5	169472782	6.96E-02	6046	1.55E-01	Down	1.42	7740	1.16	0.08
rs3003137	9088	PKMYT1	20	62301246	6.97E-02	6047	3.63E-01	Up	0.91	9698	1.16	0.04
rs3003137	4661	MYT1	20	62301246	6.97E-02	6048	8.47E-01	Down	0.19	12936	1.16	0.01
rs2149399	7827	NPHS2	1	176243150	6.97E-02	6049	9.27E-01	Up	0.09	13413	1.16	0.00
rs2498804	207	AKT1	14	104304140	6.97E-02	6050	2.53E-01	Up	1.14	8798	1.16	0.06
rs6000847	26088	GGA1	22	36347122	6.98E-02	6051	9.73E-01	Up	0.03	13718	1.16	0.00
rs2961671	64396	GMCL1L	5	177552795	6.98E-02	6052	9.69E-05	Up	3.90	2444	1.16	0.40
rs5934323	56474	CTPS2		16495136	6.99E-02	6053	2.21E-03	Up	3.06	3599	1.16	0.27
rs780090	64838	FNDC4	2	27630125	6.99E-02	6054	9.18E-03	Down	2.61	4445	1.16	0.20
rs780090	26160	IFT172	2	27630125	6.99E-02	6055	4.03E-01	Up	0.84	10004	1.16	0.04
rs780090	2646	GCKR	2	27630125	6.99E-02	6056	5.31E-01	Up	0.63	10950	1.16	0.03
rs9975850	54039	PCBP3	21	46183343	6.99E-02	6057	3.15E-01	Down	1.00	9320	1.16	0.05
rs4547833	201965	RWDD4A	4	184947185	6.99E-02	6058	5.17E-02	Up	1.95	6034	1.16	0.13
rs4655314	5629	PROX1	1	210555903	6.99E-02	6059	1.49E-01	Down	1.44	7673	1.16	0.08
rs6493442	9101	USP8	15	48489350	6.99E-02	6060	9.98E-01	Up	0.00	13897	1.16	0.00
rs352360	134265	AFAP1L1	5	148643981	7.00E-02	6061	3.10E-01	Down	1.02	9267	1.16	0.05
rs6872619	81848	SPRY4	5	141666251	7.00E-02	6062	5.54E-03	Down	2.77	4135	1.16	0.23
rs1109840	2556	GABRA3		151268211	7.01E-02	6063	1.21E-01	Down	1.55	7318	1.15	0.09
rs10745734	6636	SNRPF	12	94765182	7.01E-02	6064	2.36E-03	Up	3.04	3651	1.15	0.26
rs7793239	1357	CPA1	7	129628646	7.01E-02	6065	5.06E-01	Down	0.66	10760	1.15	0.03
rs10043750	8546	AP3B1	5	77560400	7.01E-02	6066	1.34E-01	Down	1.50	7485	1.15	0.09
rs2235852	5905	RANGAP1	22	39985654	7.01E-02	6067	1.82E-05	Up	4.29	2073	1.15	0.47
rs7011042	85479	DNAJC5B	8	67122878	7.01E-02	6068	4.08E-01	Up	0.83	10040	1.15	0.04
rs9810848	54995	OXSM	3	25801541	7.02E-02	6069	7.53E-06	Up	4.48	1886	1.15	0.51
rs9810848	55768	NGLY1	3	25801541	7.02E-02	6070	1.99E-04	Up	3.72	2630	1.15	0.37

rs20541	3596	IL13	5	132023863	7.02E-02	6071	2.48E-01	Down	1.16	8745	1.15	0.06
rs20541	3565	IL4	5	132023863	7.02E-02	6072	2.89E-01	Down	1.06	9105	1.15	0.05
rs17103608	84312	BRMS1L	14	35422074	7.02E-02	6073	3.22E-01	Down	0.99	9373	1.15	0.05
rs6751956	80067	C2orf37	2	172117123	7.02E-02	6074	3.31E-03	Up	2.94	3829	1.15	0.25
rs9897822	1497	CTNS	17	3476315	7.03E-02	6075	1.98E-01	Down	1.29	8229	1.15	0.07
rs2029524	92181	UBTD2	5	171596274	7.03E-02	6076	2.39E-02	Down	2.26	5242	1.15	0.16
rs11611788	4842	NOS1	12	116201096	7.03E-02	6077	2.12E-02	Down	2.30	5130	1.15	0.17
rs11611788	340719	NANOS1	12	116201096	7.03E-02	6078	1.97E-01	Up	1.29	8224	1.15	0.07
rs4428484	5134	PDCD2	6	170819741	7.05E-02	6079	2.77E-01	Up	1.09	9004	1.15	0.06
rs11613495	2251	FGF6	12	4424891	7.05E-02	6080	3.73E-01	Down	0.89	9787	1.15	0.04
rs12406805	10450	PPIE	1	39859195	7.05E-02	6081	1.04E-01	Up	1.63	7055	1.15	0.10
rs10785669	91523	FAM113B	12	45899747	7.06E-02	6082	7.80E-02	Down	1.76	6595	1.15	0.11
rs752081	2705	GJB1		70232823	7.07E-02	6083	8.25E-36	Up	12.42	38	1.15	3.51
rs132369	129080	EMID1	22	27928165	7.07E-02	6084	3.64E-02	Up	2.09	5662	1.15	0.14
rs4905431	56967	C14orf132	14	95634303	7.07E-02	6085	7.05E-03	Down	2.69	4263	1.15	0.22
rs9866941	79929	MAP6D1	3	185037776	7.07E-02	6086	6.13E-02	Down	1.87	6264	1.15	0.12
rs1112247	25816	TNFAIP8	5	118711401	7.08E-02	6087	7.88E-01	Down	0.27	12595	1.15	0.01
rs10089	6558	SLC12A2	5	127550442	7.08E-02	6088	1.37E-04	Up	3.81	2521	1.15	0.39
rs862792	72	ACTG2	2	74024069	7.08E-02	6089	4.42E-23	Down	9.89	139	1.15	2.24
rs12749729	11221	DUSP10	1	218271891	7.08E-02	6090	1.58E-01	Down	1.41	7779	1.15	0.08
rs4926408	51177	PLEKHO1	1	146919299	7.09E-02	6091	1.75E-05	Down	4.30	2066	1.15	0.48
rs8120307	6185	RPN2	20	35236602	7.09E-02	6092	2.05E-03	Up	3.08	3542	1.15	0.27
rs8120307	5707	PSMD1	20	35236602	7.09E-02	6093	2.80E-01	Down	1.08	9031	1.15	0.06
rs1995106	153572	IRX2	5	2810920	7.09E-02	6094	1.29E-02	Up	2.49	4683	1.15	0.19
rs9506370	55269	PSPC1	13	19226143	7.10E-02	6095	1.18E-02	Up	2.52	4624	1.15	0.19
rs17194742	7761	ZNF214	11	7017638	7.10E-02	6096	3.57E-01	Down	0.92	9656	1.15	0.04
rs17629216	56129	PCDHB7	5	140512593	7.10E-02	6097	1.65E-01	Up	1.39	7869	1.15	0.08
rs17629216	54661	PCDHB17	5	140512593	7.10E-02	6098	3.46E-01	Up	0.94	9563	1.15	0.05
rs17629216	56130	PCDHB6	5	140512593	7.10E-02	6099	6.02E-01	Up	0.52	11441	1.15	0.02
rs7248225	5676	PSG7	19	48114839	7.10E-02	6100	6.26E-01	Up	0.49	11619	1.15	0.02
rs7248225	5675	PSG6	19	48114839	7.10E-02	6101	9.81E-01	Down	0.02	13779	1.15	0.00
rs7243056	3998	LMAN1	18	55172529	7.10E-02	6102	1.83E-12	Up	7.05	577	1.15	1.17
rs4973410	4691	NCL	2	232157239	7.11E-02	6103	1.82E-04	Up	3.74	2596	1.15	0.37
rs13240375	26062	HYALP1	7	123040339	7.11E-02	6104	6.11E-01	Down	0.51	11517	1.15	0.02
rs8064529	57513	CASKIN2	17	71031008	7.11E-02	6105	1.46E-01	Down	1.45	7638	1.15	0.08
rs8064529	283989	TSEN54	17	71031008	7.11E-02	6106	2.27E-01	Up	1.21	8526	1.15	0.06
rs8023466	9960	USP3	15	61628728	7.11E-02	6107	4.83E-06	Up	4.57	1807	1.15	0.53
rs4512021	6373	CXCL11	4	77311586	7.11E-02	6108	4.80E-08	Up	5.46	1215	1.15	0.73
rs2275729	3087	HHEX	10	94442410	7.11E-02	6109	2.84E-01	Up	1.07	9067	1.15	0.05
rs7641394	5274	SERPINI1	3	169014309	7.11E-02	6110	6.77E-01	Down	0.42	11927	1.15	0.02
rs3787950	7113	TMPRSS2	21	41788166	7.11E-02	6111	1.52E-04	Up	3.79	2554	1.15	0.38
rs12536983	5701	PSMC2	7	102601550	7.12E-02	6112	1.03E-02	Up	2.57	4531	1.15	0.20
rs11066320	5781	PTPN11	12	111369155	7.12E-02	6113	9.41E-08	Down	5.34	1291	1.15	0.70
rs645507	128178	EDARADD	1	232899301	7.12E-02	6114	4.13E-01	Down	0.82	10073	1.15	0.04
rs7605342	9792	SERTAD2	2	64767918	7.12E-02	6115	6.87E-09	Down	5.79	1031	1.15	0.82
rs4652903	55700	MAP7D1	1	36311951	7.12E-02	6116	1.43E-02	Down	2.45	4772	1.15	0.18
rs7783685	5782	PTPN12	7	76895741	7.12E-02	6117	5.96E-01	Down	0.53	11406	1.15	0.02
rs10742552	23538	OR52A1	11	5145403	7.12E-02	6118	8.09E-02	Up	1.75	6645	1.15	0.11
rs8110090	7040	TGFB1	19	46537712	7.13E-02	6119	4.53E-02	Down	2.00	5881	1.15	0.13
rs12113404	11335	CBX3	7	25996534	7.13E-02	6120	6.91E-20	Up	9.19	194	1.15	1.92
rs12113404	9603	NFE2L3	7	25996534	7.13E-02	6121	5.38E-13	Up	7.22	531	1.15	1.23
rs3733397	4487	MSX1	4	4967474	7.13E-02	6122	9.22E-01	Down	0.10	13385	1.15	0.00
rs11707321	51304	ZDHHC3	3	44997100	7.13E-02	6123	1.21E-06	Down	4.85	1603	1.15	0.59
rs11707321	23016	EXOSC7	3	44997100	7.13E-02	6124	3.25E-04	Down	3.59	2805	1.15	0.35
rs7126405	4583	MUC2	11	1093296	7.14E-02	6125	1.24E-02	Down	2.50	4652	1.15	0.19
rs6065094	26051	PPP1R16B	20	36886608	7.14E-02	6126	9.00E-01	Down	0.13	13239	1.15	0.00
rs10513526	51319	RSRC1	3	159513084	7.14E-02	6127	8.42E-01	Down	0.20	12911	1.15	0.01
rs9887134	57477	SHROOM4		50323954	7.15E-02	6128	1.87E-01	Down	1.32	8114	1.15	0.07
rs3760802	6820	SULT2B1	19	53768499	7.15E-02	6129	1.47E-08	Down	5.67	1087	1.15	0.78
rs17006436	50489	CD207	2	70971766	7.15E-02	6130	2.73E-02	Down	2.21	5375	1.15	0.16
rs13160267	117608	ZNF354B	5	178207819	7.16E-02	6131	2.76E-01	Down	1.09	9000	1.15	0.06
rs1063902	10681	GNB5	15	50272242	7.16E-02	6132	3.63E-10	Down	6.27	820	1.15	0.94
rs4826594	90121	TSR2		54337427	7.16E-02	6133	1.85E-04	Down	3.74	2599	1.15	0.37
rs4970804	163479	FNDC7	1	108972399	7.16E-02	6134	1.15E-01	Up	1.58	7219	1.15	0.09
rs3825877	54581	SCAND2	15	82975635	7.16E-02	6135	4.79E-01	Up	0.71	10563	1.15	0.03
rs9467062	51473	DCDC2	6	24281361	7.16E-02	6136	1.60E-01	Up	1.41	7809	1.14	0.08
rs4886765	123591	C15orf27	15	74182725	7.17E-02	6137	2.24E-01	Down	1.22	8496	1.14	0.06
rs754532	9092	SART1	11	65503633	7.17E-02	6138	4.58E-01	Down	0.74	10411	1.14	0.03
rs7106849	1850	DUSP8	11	1551839	7.18E-02	6139	2.30E-02	Down	2.27	5198	1.14	0.16
rs2062276	55757	UGCGL2	13	95454145	7.18E-02	6140	1.91E-06	Up	4.76	1660	1.14	0.57
rs3821812	64108	RTP4	3	188571514	7.18E-02	6141	8.33E-04	Down	3.34	3148	1.14	0.31
rs2037595	84124	ZNF394	7	98730530	7.18E-02	6142	4.04E-02	Down	2.05	5756	1.14	0.14
rs1952065	79739	TTLL7	1	84176226	7.18E-02	6143	1.63E-01	Down	1.40	7850	1.14	0.08
rs17458234	2108	ETFA	15	74362076	7.19E-02	6144	4.50E-07	Up	5.05	1466	1.14	0.63
rs757485	6827	SUPT4H1	17	53779709	7.19E-02	6145	4.38E-05	Down	4.09	2249	1.14	0.44
rs13086084	285343	C3orf23	3	44355492	7.20E-02	6146	7.10E-04	Down	3.39	3082	1.14	0.31
rs8139063	150372	NFAM1	22	41138251	7.20E-02	6147	6.35E-06	Down	4.51	1857	1.14	0.52
rs7638400	80012	PHC3	3	171320489	7.20E-02	6148	7.54E-01	Down	0.31	12403	1.14	0.01
rs7619872	84892	C3orf39	3	43091174	7.20E-02	6149	3.47E-02	Down	2.11	5615	1.14	0.15
rs12147950	145282	MIPOL1	14	37059021	7.20E-02	6150	6.59E-03	Up	2.72	4220	1.14	0.22
rs4963516	83461	CDCA3	12	6818289	7.20E-02	6151	9.68E-05	Up	3.90	2442	1.14	0.40

rs4963516	8078	USP5	12	6818289	7.20E-02	6152	1.36E-04	Down	3.82	2519	1.14	0.39
rs4131864	9975	NR1D2	3	23960813	7.20E-02	6153	6.57E-01	Down	0.44	11814	1.14	0.02
rs523200	10235	RASGRP2	11	64289155	7.21E-02	6154	9.87E-09	Down	5.73	1056	1.14	0.80
rs17645731	27097	TAF5L	1	226028767	7.21E-02	6155	5.60E-01	Up	0.58	11163	1.14	0.03
rs4806674	59283	CACNG8	19	59153350	7.21E-02	6156	2.82E-01	Down	1.08	9051	1.14	0.05
rs17245425	51276	ZNF571	19	42766434	7.21E-02	6157	1.31E-02	Up	2.48	4694	1.14	0.19
rs17245425	163255	ZNF540	19	42766434	7.21E-02	6158	1.41E-01	Down	1.47	7588	1.14	0.09
rs1402325	79177	ZNF576	19	48797576	7.21E-02	6159	1.93E-01	Down	1.30	8180	1.14	0.07
rs1402325	126299	ZNF428	19	48797576	7.21E-02	6160	9.58E-01	Down	0.05	13607	1.14	0.00
rs2147878	55118	CRTAC1	10	99733764	7.21E-02	6161	1.66E-01	Down	1.38	7881	1.14	0.08
rs6845322	56034	PDGFC	4	158241710	7.22E-02	6162	5.26E-04	Down	3.47	2976	1.14	0.33
rs7945554	391	RHOG	11	3835554	7.22E-02	6163	6.81E-04	Down	3.40	3070	1.14	0.32
rs1540599	10409	BASP1	5	17272093	7.22E-02	6164	2.02E-03	Down	3.09	3536	1.14	0.27
rs7247433	94039	ZNF101	19	19656560	7.22E-02	6165	6.45E-02	Up	1.85	6336	1.14	0.12
rs4386955	55034	MOCOS	8	57197581	7.22E-02	6166	6.21E-01	Up	0.49	11594	1.14	0.02
rs4386955	4342	MOS	8	57197581	7.22E-02	6167	8.61E-01	Down	0.17	13016	1.14	0.01
rs429608	720	C4A	6	32038441	7.23E-02	6168	1.07E-03	Up	3.27	3248	1.14	0.30
rs2051579	23543	RBM9	22	34555857	7.23E-02	6169	1.14E-18	Down	8.82	241	1.14	1.79
rs6739216	116255	MOGAT1	2	223376652	7.23E-02	6170	8.48E-01	Up	0.19	12944	1.14	0.01
rs3808553	8323	FZD6	8	104406543	7.23E-02	6171	1.57E-02	Up	2.42	4857	1.14	0.18
rs698365	353376	TICAM2	5	114978979	7.24E-02	6172	8.30E-02	Down	1.73	6685	1.14	0.11
rs698365	51014	TMED7	5	114978979	7.24E-02	6173	1.97E-01	Up	1.29	8225	1.14	0.07
rs12459507	8175	SF3A2	19	2175387	7.24E-02	6174	2.61E-02	Up	2.22	5327	1.14	0.16
rs12459507	55111	PLEKHJ1	19	2175387	7.24E-02	6175	9.29E-01	Up	0.09	13425	1.14	0.00
rs9919542	8898	MTMR2	11	95209178	7.25E-02	6176	2.95E-01	Up	1.05	9151	1.14	0.05
rs9919542	9702	CEP57	11	95209178	7.25E-02	6177	8.94E-01	Up	0.13	13204	1.14	0.00
rs7046039	5774	PTPN3	9	109316434	7.25E-02	6178	2.84E-06	Up	4.68	1724	1.14	0.55
rs676114	10435	CDC42EP2	11	64833494	7.25E-02	6179	3.96E-01	Up	0.85	9954	1.14	0.04
rs2290452	307	ANXA4	2	69959295	7.25E-02	6180	1.84E-13	Down	7.36	490	1.14	1.27
rs2290452	25801	GCA	2	69959295	7.25E-02	6181	3.82E-04	Up	3.55	2865	1.14	0.34
rs542605	4046	LSP1	11	1869302	7.25E-02	6182	3.79E-01	Up	0.88	9832	1.14	0.04
rs217463	5236	PGM1	1	63753808	7.25E-02	6183	1.09E-05	Down	4.40	1964	1.14	0.50
rs2106101	23256	SCFD1	14	30275385	7.26E-02	6184	9.49E-06	Up	4.43	1937	1.14	0.50
rs7124995	8048	CSRP3	11	19157426	7.26E-02	6185	8.67E-01	Down	0.17	13040	1.14	0.01
rs2602413	55839	CENPN	16	79594172	7.26E-02	6186	2.52E-08	Up	5.56	1149	1.14	0.76
rs11130077	54585	LZTFL1	3	45852716	7.26E-02	6187	1.35E-01	Down	1.49	7517	1.14	0.09
rs903213	84953	MICALCL	11	12302035	7.27E-02	6188	2.30E-02	Up	2.27	5207	1.14	0.16
rs2910006	56125	PCDHB11	5	140570950	7.27E-02	6189	1.34E-01	Up	1.50	7503	1.14	0.09
rs882820	6345	SRL	16	4196006	7.27E-02	6190	1.20E-01	Down	1.55	7299	1.14	0.09
rs9860290	10393	ANAPC10	3	101321796	7.27E-02	6191	5.40E-01	Down	0.61	11015	1.14	0.03
rs9860290	8099	CDK2AP1	3	101321796	7.27E-02	6192	8.71E-01	Down	0.16	13075	1.14	0.01
rs2128416	54897	CASZ1	1	10634714	7.28E-02	6193	7.31E-01	Down	0.34	12255	1.14	0.01
rs6690242	57459	GATAD2B	1	150614171	7.28E-02	6194	5.40E-03	Down	2.78	4114	1.14	0.23
rs10133979	9240	PNMA1	14	73265072	7.28E-02	6195	7.85E-03	Down	2.66	4327	1.14	0.21
rs1958394	84659	RNASE7	14	20587265	7.28E-02	6196	3.35E-01	Down	0.96	9488	1.14	0.05
rs2007556	165082	GPR113	2	26444142	7.28E-02	6197	1.61E-01	Up	1.40	7820	1.14	0.08
rs337609	51274	KLF3	4	38484625	7.28E-02	6198	3.31E-02	Down	2.13	5566	1.14	0.15
rs584184	5683	PSMA2	7	42764890	7.29E-02	6199	4.59E-02	Down	2.00	5893	1.14	0.13
rs6939396	9436	NCR2	6	41413053	7.29E-02	6200	7.90E-01	Up	0.27	12609	1.14	0.01
rs11768465	5118	PCOLCE	7	99843037	7.30E-02	6201	2.54E-03	Down	3.02	3698	1.14	0.26
rs11768465	64598	MOSPD3	7	99843037	7.30E-02	6202	2.81E-02	Up	2.20	5396	1.14	0.16
rs11768465	4034	LRCH4	7	99843037	7.30E-02	6203	8.87E-01	Down	0.14	13173	1.14	0.01
rs11768465	26261	FBXO24	7	99843037	7.30E-02	6204	9.58E-01	Down	0.05	13606	1.14	0.00
rs3752408	5408	PNLIPRP2	10	118391361	7.30E-02	6205	1.16E-01	Up	1.57	7238	1.14	0.09
rs11692675	6323	SCN1A	2	166751935	7.30E-02	6206	3.57E-01	Up	0.92	9655	1.14	0.04
rs1677234	26108	PYGO1	15	53623996	7.30E-02	6207	8.06E-01	Up	0.25	12706	1.14	0.01
rs738802	66035	SLC2A11	22	22516635	7.30E-02	6208	6.03E-01	Down	0.52	11453	1.14	0.02
rs3762199	10483	SEC23B	20	18435792	7.30E-02	6209	4.61E-10	Up	6.23	848	1.14	0.93
rs3762199	10741	RBBP9	20	18435792	7.30E-02	6210	9.42E-01	Up	0.07	13506	1.14	0.00
rs6025518	8480	RAE1	20	55386960	7.31E-02	6211	1.23E-02	Up	2.50	4651	1.14	0.19
rs3822196	2798	GNRHR	4	68443565	7.31E-02	6212	6.73E-01	Down	0.42	11902	1.14	0.02
rs1529039	6783	SULT1E1	4	70882766	7.31E-02	6213	6.27E-01	Up	0.49	11634	1.14	0.02
rs10415278	51291	GMIP	19	19630157	7.31E-02	6214	4.40E-01	Up	0.77	10270	1.14	0.04
rs12209262	55350	VNN3	6	133114866	7.31E-02	6215	3.69E-01	Down	0.90	9754	1.14	0.04
rs9508	22826	DNAJC8	1	28248421	7.31E-02	6216	1.41E-06	Down	4.82	1619	1.14	0.58
rs9508	93974	ATPIF1	1	28248421	7.31E-02	6217	1.09E-03	Up	3.27	3253	1.14	0.30
rs17576289	23395	LARS2	3	45433737	7.31E-02	6218	1.56E-10	Up	6.40	782	1.14	0.98
rs7096608	9397	NMT2	10	15239759	7.32E-02	6219	1.77E-01	Down	1.35	7984	1.14	0.08
rs2985689	55172	C14orf104	14	49167781	7.32E-02	6220	2.57E-14	Up	7.62	435	1.14	1.36
rs2985689	4247	MGAT2	14	49167781	7.32E-02	6221	7.42E-07	Up	4.95	1528	1.14	0.61
rs2985689	6166	RPL36AL	14	49167781	7.32E-02	6222	8.88E-02	Up	1.70	6783	1.14	0.11
rs9461068	51053	GMNN	6	24887741	7.32E-02	6223	1.57E-03	Down	3.16	3411	1.14	0.28
rs889327	1891	ECH1	19	44002060	7.32E-02	6224	2.11E-01	Up	1.25	8369	1.14	0.07
rs889327	3960	LGALS4	19	44002060	7.32E-02	6225	3.48E-01	Down	0.94	9578	1.14	0.05
rs631905	9094	UNC119	17	23907440	7.33E-02	6226	1.97E-01	Up	1.29	8226	1.14	0.07
rs631905	94005	PIGS	17	23907440	7.33E-02	6227	8.35E-01	Up	0.21	12876	1.14	0.01
rs11881682	30835	CD209	19	7721376	7.33E-02	6228	9.89E-02	Down	1.65	6979	1.13	0.10
rs11881682	339390	CLEC4G	19	7721376	7.33E-02	6229	2.90E-01	Up	1.06	9114	1.13	0.05
rs3746259	54929	TMEM161A	19	19106771	7.33E-02	6230	4.57E-01	Up	0.74	10404	1.13	0.03
rs1562234	30832	ZNF354C	5	178449089	7.34E-02	6231	4.72E-01	Down	0.72	10511	1.13	0.03
rs11191487	54805	CNNM2	10	104724613	7.34E-02	6232	1.87E-01	Up	1.32	8107	1.13	0.07

rs6703000	4009	LMX1A	1	161911532	7.34E-02	6233	8.45E-01	Down	0.20	12929	1.13	0.01
rs7386788	10434	LYPLA1	8	55192855	7.34E-02	6234	5.63E-16	Up	8.08	353	1.13	1.52
rs13261107	137964	AGPAT6	8	41546586	7.34E-02	6235	2.35E-01	Down	1.19	8613	1.13	0.06
rs10420138	5518	PPP2R1A	19	57396773	7.35E-02	6236	9.01E-01	Up	0.12	13251	1.13	0.00
rs2369476	477	ATP1A2	1	156934561	7.35E-02	6237	1.55E-10	Down	6.40	781	1.13	0.98
rs4645768	4174	MCM5	22	34128274	7.35E-02	6238	1.52E-02	Down	2.43	4820	1.13	0.18
rs7214718	9931	HELZ	17	62669917	7.36E-02	6239	8.54E-01	Up	0.18	12982	1.13	0.01
rs11074471	55623	THUMPD1	16	20655832	7.36E-02	6240	1.70E-01	Down	1.37	7911	1.13	0.08
rs9997310	308	ANXA5	4	122986285	7.36E-02	6241	1.26E-03	Up	3.23	3318	1.13	0.29
rs4762075	23592	LEMD3	12	63906816	7.37E-02	6242	1.35E-01	Up	1.49	7512	1.13	0.09
rs12343582	7539	ZFP37	9	112874214	7.37E-02	6243	9.30E-01	Down	0.09	13431	1.13	0.00
rs11874358	162655	ZNF519	18	14087884	7.37E-02	6244	3.81E-01	Down	0.88	9842	1.13	0.04
rs2864436	79763	ISOC2	19	60678449	7.37E-02	6245	2.82E-01	Up	1.08	9047	1.13	0.06
rs3809437	26257	NKX2-8	14	36122614	7.37E-02	6246	5.38E-01	Down	0.62	10993	1.13	0.03
rs2295014	83699	SH3BGRL2	6	80462555	7.37E-02	6247	8.82E-20	Down	9.10	206	1.13	1.91
rs4661037	23381	SMG5	1	153051552	7.38E-02	6248	3.53E-01	Up	0.93	9615	1.13	0.05
rs4661037	84283	TMEM79	1	153051552	7.38E-02	6249	8.43E-01	Down	0.20	12918	1.13	0.01
rs11153198	81491	GPR63	6	97346150	7.38E-02	6250	9.98E-01	Down	0.00	13896	1.13	0.00
rs11064391	3902	LAG3	12	6767788	7.38E-02	6251	2.15E-03	Down	3.07	3577	1.13	0.27
rs1735169	28991	COMMD5	8	146037858	7.38E-02	6252	1.84E-05	Up	4.28	2075	1.13	0.47
rs1735169	7553	ZNF7	8	146037858	7.38E-02	6253	5.75E-02	Up	1.90	6174	1.13	0.12
rs3782415	8835	SOCS2	12	92470223	7.38E-02	6254	4.64E-03	Down	2.83	4016	1.13	0.23
rs10499076	4082	MARCKS	6	114299470	7.38E-02	6255	6.16E-02	Down	1.87	6268	1.13	0.12
rs12529779	3459	IFNGR1	6	137587799	7.38E-02	6256	4.10E-01	Down	0.82	10057	1.13	0.04
rs11219898	219855	SLC37A2	11	124470614	7.39E-02	6257	9.95E-01	Down	0.01	13879	1.13	0.00
rs11794127	10880	ACTL7B	9	108702530	7.39E-02	6258	4.46E-01	Up	0.76	10313	1.13	0.04
rs11794127	10881	ACTL7A	9	108702530	7.39E-02	6259	9.52E-01	Up	0.06	13569	1.13	0.00
rs6901756	25862	USP49	6	41933568	7.39E-02	6260	7.06E-02	Down	1.81	6443	1.13	0.12
rs4984677	117166	WFIKKN1	16	611683	7.39E-02	6261	8.46E-01	Up	0.19	12932	1.13	0.01
rs13144383	23657	SLC7A11	4	139448409	7.40E-02	6262	2.13E-03	Up	3.07	3571	1.13	0.27
rs4539477	7347	UCHL3	13	75051416	7.40E-02	6263	7.59E-01	Up	0.31	12434	1.13	0.01
rs9991367	1950	EGF	4	111251905	7.40E-02	6264	1.89E-01	Up	1.31	8137	1.13	0.07
rs16918878	27130	INVS	9	99926185	7.40E-02	6265	4.46E-04	Up	3.51	2919	1.13	0.34
rs16918878	23071	TXNDC4	9	99926185	7.40E-02	6266	3.49E-02	Up	2.11	5622	1.13	0.15
rs8011839	4522	MTHFD1	14	63940963	7.40E-02	6267	7.03E-09	Up	5.79	1032	1.13	0.82
rs4133249	375061	FAM89A	1	227468807	7.40E-02	6268	2.57E-06	Down	4.70	1707	1.13	0.56
rs4077125	147179	WIPF2	17	35677005	7.41E-02	6269	2.46E-01	Up	1.16	8723	1.13	0.06
rs1801516	472	ATM	11	107680672	7.41E-02	6270	7.65E-01	Up	0.30	12461	1.13	0.01
rs4548256	1317	SLC31A1	9	113048977	7.41E-02	6271	1.05E-03	Down	3.28	3240	1.13	0.30
rs176781	122945	C14orf148	14	76957434	7.41E-02	6272	2.54E-01	Up	1.14	8799	1.13	0.06
rs13003996	119	ADD2	2	70803170	7.41E-02	6273	9.81E-02	Up	1.65	6964	1.13	0.10
rs1133415	64943	NT5DC2	3	52550871	7.41E-02	6274	3.59E-02	Down	2.10	5643	1.13	0.14
rs3760048	9727	RAB11FIP3	16	414181	7.42E-02	6275	4.78E-01	Down	0.71	10559	1.13	0.03
rs1002424	23548	TTC33	5	40803154	7.42E-02	6276	2.28E-02	Down	2.28	5190	1.13	0.16
rs6432221	6664	SOX11	2	5779300	7.42E-02	6277	1.61E-01	Up	1.40	7824	1.13	0.08
rs7919509	22929	SEPHS1	10	13423017	7.42E-02	6278	5.52E-02	Up	1.92	6120	1.13	0.13
rs2743554	84547	PGBD1	6	28373619	7.43E-02	6279	7.08E-03	Down	2.69	4268	1.13	0.21
rs4710191	2853	GPR31	6	167542953	7.43E-02	6280	7.17E-01	Up	0.36	12181	1.13	0.01
rs11563197	6694	SPP2	2	234742021	7.43E-02	6281	5.27E-01	Up	0.63	10924	1.13	0.03
rs11563197	130367	SGPP2	2	234742021	7.43E-02	6282	6.31E-01	Down	0.48	11653	1.13	0.02
rs1245582	9469	CHST3	10	73448273	7.43E-02	6283	1.28E-09	Down	6.07	908	1.13	0.89
rs798149	8905	AP1S2		15645088	7.43E-02	6284	7.76E-01	Up	0.28	12534	1.13	0.01
rs3744473	2670	GFAP	17	40337814	7.43E-02	6285	2.22E-02	Up	2.29	5170	1.13	0.17
rs3027325	2742	GLRA2		14322890	7.43E-02	6286	3.73E-01	Up	0.89	9790	1.13	0.04
rs2038024	10560	SLC19A2	1	166187640	7.44E-02	6287	7.04E-04	Up	3.39	3078	1.13	0.32
rs6499508	794	CALB2	16	69953791	7.44E-02	6288	8.97E-01	Down	0.13	13222	1.13	0.00
rs5744738	5827	PXMP2	12	131867254	7.44E-02	6289	2.45E-03	Down	3.03	3671	1.13	0.26
rs5744738	5426	POLE	12	131867254	7.44E-02	6290	9.18E-01	Up	0.10	13365	1.13	0.00
rs2494606	55229	PANK4	1	2498795	7.44E-02	6291	2.17E-01	Down	1.23	8432	1.13	0.07
rs4817579	6651	SON	21	33832090	7.45E-02	6292	3.00E-01	Down	1.04	9193	1.13	0.05
rs17170153	51251	NT5C3	7	32833995	7.45E-02	6293	9.80E-05	Up	3.90	2447	1.13	0.40
rs11070296	84936	ZFYVE19	15	38885646	7.45E-02	6294	1.26E-04	Down	3.83	2505	1.13	0.39
rs11070296	54866	PPP1R14D	15	38885646	7.45E-02	6295	2.44E-03	Down	3.03	3669	1.13	0.26
rs11070296	55192	DNAJC17	15	38885646	7.45E-02	6296	5.33E-01	Down	0.62	10965	1.13	0.03
rs260422	27300	ZNF544	19	63421645	7.45E-02	6297	2.45E-06	Up	4.71	1699	1.13	0.56
rs260422	10782	ZNF274	19	63421645	7.45E-02	6298	6.68E-01	Up	0.43	11874	1.13	0.02
rs1915440	219623	TMEM26	10	62866140	7.46E-02	6299	8.94E-01	Down	0.13	13205	1.13	0.00
rs6775993	9779	TBC1D5	3	17180351	7.46E-02	6300	8.17E-01	Up	0.23	12766	1.13	0.01
rs397516	57150	C6orf162	6	88107771	7.46E-02	6301	9.44E-01	Up	0.07	13519	1.13	0.00
rs2523966	10255	HCG9	6	30047219	7.46E-02	6302	2.41E-01	Down	1.17	8672	1.13	0.06
rs2275293	54963	UCKL1	20	62046863	7.46E-02	6303	8.43E-01	Up	0.20	12919	1.13	0.01
rs3912631	121391	KRT74	12	51249120	7.47E-02	6304	2.73E-01	Up	1.10	8975	1.13	0.06
rs12587208	8487	SIP1	14	38646046	7.47E-02	6305	5.41E-04	Up	3.46	2986	1.13	0.33
rs12587208	9169	SFRS2IP	14	38646046	7.47E-02	6306	2.58E-02	Up	2.23	5316	1.13	0.16
rs329379	51163	DBR1	3	139376442	7.48E-02	6307	4.03E-01	Up	0.84	10001	1.13	0.04
rs9635366	51495	PTPLAD1	15	63592039	7.48E-02	6308	6.74E-04	Up	3.40	3066	1.13	0.32
rs6446982	1633	DCK	4	72220178	7.48E-02	6309	7.34E-06	Up	4.48	1880	1.13	0.51
rs932134	80704	SLC19A3	2	228393412	7.48E-02	6310	2.17E-04	Down	3.70	2659	1.13	0.37
rs4337792	4589	MUC7	4	71506651	7.49E-02	6311	1.34E-03	Up	3.21	3336	1.13	0.29
rs2508467	2893	GRIA4	11	105263314	7.49E-02	6312	2.88E-02	Down	2.19	5418	1.13	0.15
rs4498196	152	ADRA2C	4	3784811	7.49E-02	6313	1.67E-03	Down	3.14	3439	1.13	0.28

rs1790740	57804	POLD4	11	66886698	7.50E-02	6314	2.39E-02	Up	2.26	5245	1.13	0.16
rs1790740	23529	CLCF1	11	66886698	7.50E-02	6315	3.58E-01	Up	0.92	9661	1.13	0.04
rs1790740	1178	CLC	11	66886698	7.50E-02	6316	9.95E-01	Down	0.01	13881	1.13	0.00
rs6580842	6334	SCN8A	12	50381637	7.50E-02	6317	9.24E-01	Down	0.10	13396	1.13	0.00
rs10133689	91754	NEK9	14	74674808	7.50E-02	6318	5.71E-05	Down	4.02	2320	1.12	0.42
rs10133689	10972	TMED10	14	74674808	7.50E-02	6319	4.05E-03	Up	2.87	3939	1.12	0.24
rs572634	1760	DMPK	19	50974343	7.50E-02	6320	1.03E-01	Down	1.63	7045	1.12	0.10
rs572634	1762	DMWD	19	50974343	7.50E-02	6321	1.28E-01	Down	1.52	7419	1.12	0.09
rs572634	147912	SIX5	19	50974343	7.50E-02	6322	8.90E-01	Up	0.14	13190	1.12	0.01
rs1148476	943	TNFRSF8	1	12121063	7.51E-02	6323	2.81E-01	Down	1.08	9045	1.12	0.06
rs4665947	10669	CGREF1	2	27259680	7.51E-02	6324	2.28E-03	Up	3.05	3621	1.12	0.26
rs4665947	84696	ABHD1	2	27259680	7.51E-02	6325	2.43E-01	Up	1.17	8693	1.12	0.06
rs2143511	5770	PTPN1	20	48578076	7.51E-02	6326	2.08E-01	Up	1.26	8330	1.12	0.07
rs953691	7222	TRPC3	4	123170700	7.51E-02	6327	1.27E-01	Down	1.53	7405	1.12	0.09
rs9920813	26276	VPS33B	15	89376423	7.51E-02	6328	8.65E-01	Down	0.17	13036	1.12	0.01
rs17119328	56142	PCDHA6	5	140331257	7.52E-02	6329	6.80E-03	Up	2.71	4241	1.12	0.22
rs17119328	9752	PCDHA9	5	140331257	7.52E-02	6330	1.59E-02	Up	2.41	4871	1.12	0.18
rs17119328	56146	PCDHA2	5	140331257	7.52E-02	6331	1.21E-01	Up	1.55	7316	1.12	0.09
rs17119328	56139	PCDHA10	5	140331257	7.52E-02	6332	2.58E-01	Up	1.13	8845	1.12	0.06
rs17119328	56143	PCDHA5	5	140331257	7.52E-02	6333	4.99E-01	Up	0.68	10710	1.12	0.03
rs17119328	56145	PCDHA3	5	140331257	7.52E-02	6334	6.39E-01	Up	0.47	11702	1.12	0.02
rs2240079	6281	S100A10	12	46415611	7.52E-02	6335	1.09E-04	Down	3.87	2472	1.12	0.40
rs2240079	118460	EXOSC6	12	46415611	7.52E-02	6336	5.20E-01	Down	0.64	10860	1.12	0.03
rs7213426	84440	RAB11FIP4	17	26808089	7.52E-02	6337	4.42E-02	Up	2.01	5856	1.12	0.14
rs4868257	8614	STC2	5	172682128	7.53E-02	6338	5.70E-01	Up	0.57	11250	1.12	0.02
rs10136680	10876	FAM12A	14	20285532	7.53E-02	6339	4.87E-01	Up	0.70	10627	1.12	0.03
rs6498229	9516	LITAF	16	11607016	7.53E-02	6340	4.86E-02	Down	1.97	5961	1.12	0.13
rs372332	1175	AP2S1	19	52038353	7.53E-02	6341	4.98E-04	Up	3.48	2958	1.12	0.33
rs2377339	8440	NCK2	2	105915809	7.53E-02	6342	4.52E-01	Up	0.75	10353	1.12	0.03
rs1048834	84327	ZBED3	5	76405093	7.53E-02	6343	5.86E-01	Up	0.55	11346	1.12	0.02
rs13342054	9851	KIAA0753	17	6471628	7.54E-02	6344	5.49E-01	Down	0.60	11076	1.12	0.03
rs5994496	6527	SLC5A4	22	30929543	7.54E-02	6345	2.34E-01	Up	1.19	8606	1.12	0.06
rs11586974	55758	RCOR3	1	207852510	7.54E-02	6346	2.93E-01	Down	1.05	9143	1.12	0.05
rs4885948	11061	LECT1	13	52207434	7.54E-02	6347	4.12E-01	Up	0.82	10070	1.12	0.04
rs6080397	56914	OTOR	20	16687852	7.55E-02	6348	3.76E-01	Down	0.89	9805	1.12	0.04
rs875898	3206	HOXA10	7	26974145	7.55E-02	6349	5.67E-04	Up	3.45	3007	1.12	0.32
rs9925239	56052	ALG1	16	5050627	7.55E-02	6350	4.41E-03	Up	2.85	3984	1.12	0.24
rs7896013	170393	C10orf91	10	134092187	7.56E-02	6351	6.04E-01	Down	0.52	11461	1.12	0.02
rs1361135	128346	C1orf162	1	111738996	7.56E-02	6352	5.50E-01	Up	0.60	11081	1.12	0.03
rs1361135	140	ADORA3	1	111738996	7.56E-02	6353	7.80E-01	Up	0.28	12550	1.12	0.01
rs710183	79734	KCTD17	22	35775260	7.56E-02	6354	3.86E-01	Down	0.87	9886	1.12	0.04
rs1482261	79645	EFCAB1	8	49804337	7.57E-02	6355	7.01E-01	Down	0.38	12083	1.12	0.02
rs16999281	9929	JOSD1	22	37433133	7.57E-02	6356	4.88E-03	Down	2.81	4050	1.12	0.23
rs16999281	9567	GTPBP1	22	37433133	7.57E-02	6357	1.58E-01	Down	1.41	7791	1.12	0.08
rs2120853	23178	PASK	2	241800408	7.58E-02	6358	6.32E-01	Up	0.48	11659	1.12	0.02
rs2853418	9427	ECEL1	2	233165053	7.58E-02	6359	1.16E-01	Down	1.57	7226	1.12	0.09
rs13392378	2355	FOSL2	2	28518995	7.59E-02	6360	5.31E-04	Down	3.46	2980	1.12	0.33
rs1151880	10645	CAMKK2	12	120130371	7.59E-02	6361	6.12E-48	Up	14.57	8	1.12	4.72
rs1236716	7768	ZNF225	19	49332769	7.59E-02	6362	6.43E-01	Up	0.46	11734	1.12	0.02
rs1877719	54558	SPATA6	1	48540035	7.59E-02	6363	3.38E-12	Down	6.96	611	1.12	1.15
rs11885626	25791	NGEF	2	233679902	7.60E-02	6364	5.23E-02	Up	1.94	6043	1.12	0.13
rs6017999	81031	SLC2A10	20	44786416	7.60E-02	6365	3.00E-01	Up	1.04	9192	1.12	0.05
rs4242390	8793	TNFRSF10D	8	23041344	7.61E-02	6366	2.58E-02	Down	2.23	5315	1.12	0.16
rs2832281	571	BACH1	21	29607434	7.61E-02	6367	5.41E-02	Up	1.93	6093	1.12	0.13
rs2832281	83990	BRIP1	21	29607434	7.61E-02	6368	4.63E-01	Up	0.73	10448	1.12	0.03
rs7456250	4850	CNOT4	7	134624254	7.61E-02	6369	3.74E-01	Up	0.89	9798	1.12	0.04
rs176943	81892	C14orf156	14	77245550	7.62E-02	6370	2.30E-02	Up	2.27	5205	1.12	0.16
rs3783526	150468	CKAP2L	2	113258038	7.62E-02	6371	1.46E-02	Up	2.44	4791	1.12	0.18
rs3783526	3552	IL1A	2	113258038	7.62E-02	6372	5.37E-02	Down	1.93	6079	1.12	0.13
rs2527335	4771	NF2	22	28390521	7.62E-02	6373	3.17E-01	Up	1.00	9333	1.12	0.05
rs10840439	50862	RNF141	11	10524178	7.62E-02	6374	6.95E-03	Down	2.70	4257	1.12	0.22
rs12199296	83443	SF3B5	6	144459423	7.63E-02	6375	4.07E-01	Up	0.83	10035	1.12	0.04
rs381091	7773	ZNF230	19	49187155	7.64E-02	6376	9.13E-01	Up	0.11	13329	1.12	0.00
rs4758562	7262	PHLDA2	11	2920246	7.64E-02	6377	7.46E-02	Up	1.78	6522	1.12	0.11
rs17800848	144195	SLC2A14	12	7932188	7.65E-02	6378	5.58E-01	Down	0.59	11146	1.12	0.03
rs2625563	26207	PITPNC1	17	63107271	7.65E-02	6379	3.39E-05	Down	4.15	2194	1.12	0.45
rs5955641	5475	PPEF1		18543044	7.65E-02	6380	1.03E-01	Up	1.63	7047	1.12	0.10
rs3733506	64854	USP46	4	53300548	7.65E-02	6381	2.51E-01	Up	1.15	8769	1.12	0.06
rs7623757	80111	C3orf36	3	135127591	7.66E-02	6382	5.56E-01	Down	0.59	11125	1.12	0.03
rs7623757	6578	SLCO2A1	3	135127591	7.66E-02	6383	6.03E-01	Down	0.52	11446	1.12	0.02
rs13110248	11275	KLHL2	4	166469517	7.66E-02	6384	5.34E-01	Down	0.62	10970	1.12	0.03
rs7096890	3434	IFIT1	10	91131477	7.66E-02	6385	8.23E-01	Down	0.22	12804	1.12	0.01
rs7258230	26659	OR7A5	19	14793957	7.66E-02	6386	9.32E-01	Down	0.09	13446	1.12	0.00
rs2555151	144132	DNHD1	11	6488278	7.66E-02	6387	2.41E-02	Down	2.25	5254	1.12	0.16
rs12361540	11309	SLCO2B1	11	74539193	7.66E-02	6388	8.04E-01	Up	0.25	12697	1.12	0.01
rs7760960	22881	ANKRD6	6	90367932	7.66E-02	6389	2.46E-01	Up	1.16	8725	1.12	0.06
rs1050700	7248	TSC1	9	132797497	7.66E-02	6390	3.98E-01	Up	0.85	9972	1.12	0.04
rs1050700	11092	C9orf9	9	132797497	7.66E-02	6391	6.27E-01	Down	0.49	11636	1.12	0.02
rs11080055	27346	TMEM97	17	23673851	7.67E-02	6392	6.16E-06	Up	4.52	1848	1.12	0.52
rs11080055	7126	TNFAIP1	17	23673851	7.67E-02	6393	9.69E-01	Up	0.04	13696	1.12	0.00
rs456374	205	AK3L1	9	4708907	7.67E-02	6394	5.86E-01	Down	0.54	11348	1.12	0.02

rs5919393	367	AR		66608378	7.67E-02	6395	1.01E-05	Up	4.42	1951	1.11	0.50
rs935334	55668	C14orf118	14	75683431	7.67E-02	6396	8.93E-01	Down	0.13	13200	1.11	0.00
rs10176319	151195	CCNYL1	2	208395096	7.68E-02	6397	1.58E-01	Up	1.41	7788	1.11	0.08
rs7245501	114026	ZIM3	19	62355606	7.68E-02	6398	6.24E-01	Down	0.49	11611	1.11	0.02
rs4968214	1973	EIF4A1	17	7418201	7.68E-02	6399	1.90E-19	Up	9.05	212	1.11	1.87
rs4968214	26168	SENP3	17	7418201	7.68E-02	6400	3.79E-02	Down	2.08	5704	1.11	0.14
rs4968214	968	CD68	17	7418201	7.68E-02	6401	5.37E-02	Up	1.93	6080	1.11	0.13
rs4968214	9526	MPDU1	17	7418201	7.68E-02	6402	7.68E-01	Down	0.30	12474	1.11	0.01
rs2472160	7581	ZNF33A	10	38383266	7.68E-02	6403	1.59E-02	Up	2.41	4876	1.11	0.18
rs7041637	1029	CDKN2A	9	21951866	7.69E-02	6404	8.72E-01	Up	0.16	13078	1.11	0.01
rs1058177	3915	LAMC1	1	179840828	7.69E-02	6405	5.04E-01	Up	0.67	10743	1.11	0.03
rs9302112	8773	SNAP23	15	40607743	7.69E-02	6406	8.71E-02	Up	1.71	6755	1.11	0.11
rs1025104	55037	PTCD3	2	86262322	7.69E-02	6407	2.35E-01	Up	1.19	8612	1.11	0.06
rs4616886	3308	HSPA4	5	132451663	7.69E-02	6408	6.93E-03	Down	2.70	4252	1.11	0.22
rs4705873	27125	AFF4	5	132295066	7.69E-02	6409	7.80E-01	Up	0.28	12549	1.11	0.01
rs12014086	80258	EFHC2		43936905	7.70E-02	6410	1.76E-03	Down	3.13	3469	1.11	0.28
rs4778909	10933	MORF4L1	15	76942143	7.70E-02	6411	9.71E-01	Down	0.04	13705	1.11	0.00
rs10777701	84101	USP44	12	94438042	7.70E-02	6412	4.78E-02	Down	1.98	5943	1.11	0.13
rs4542691	2885	GRB2	17	70838560	7.71E-02	6413	8.63E-01	Up	0.17	13025	1.11	0.01
rs769700	1848	DUSP6	12	88246082	7.71E-02	6414	1.92E-02	Down	2.34	5034	1.11	0.17
rs3853683	6317	SERPINB3	18	59482212	7.71E-02	6415	1.16E-02	Down	2.52	4613	1.11	0.19
rs3853683	6318	SERPINB4	18	59482212	7.71E-02	6416	7.06E-01	Up	0.38	12103	1.11	0.02
rs693698	79081	C11orf48	11	62195395	7.71E-02	6417	5.05E-06	Up	4.56	1822	1.11	0.53
rs11575286	1644	DDC	7	50386294	7.71E-02	6418	1.85E-05	Up	4.28	2077	1.11	0.47
rs4857405	1371	CPOX	3	99793656	7.71E-02	6419	4.98E-05	Up	4.06	2285	1.11	0.43
rs7943852	887	CCKBR	11	6241704	7.72E-02	6420	2.57E-01	Down	1.13	8840	1.11	0.06
rs10735783	7813	EVI5	1	92907359	7.72E-02	6421	7.32E-01	Up	0.34	12264	1.11	0.01
rs3025118	4852	NPY	7	24100917	7.72E-02	6422	2.50E-01	Up	1.15	8761	1.11	0.06
rs5921756	1821	DRP2		100298918	7.72E-02	6423	4.09E-01	Up	0.82	10050	1.11	0.04
rs12317704	347918	EP400NL	12	131228521	7.72E-02	6424	8.51E-01	Up	0.19	12961	1.11	0.01
rs2650613	23049	SMG1	16	18768836	7.73E-02	6425	2.82E-02	Up	2.19	5400	1.11	0.15
rs2297454	55755	CDK5RAP2	9	120251309	7.73E-02	6426	1.37E-02	Up	2.46	4740	1.11	0.19
rs2078975	150290	DUSP18	22	29373357	7.73E-02	6427	5.36E-01	Down	0.62	10981	1.11	0.03
rs3829364	10631	POSTN	13	37072533	7.74E-02	6428	7.35E-13	Up	7.16	550	1.11	1.21
rs3752712	26173	INTS1	7	1315364	7.74E-02	6429	3.02E-04	Up	3.61	2776	1.11	0.35
rs3796622	1609	DGKQ	4	972890	7.74E-02	6430	7.68E-03	Up	2.67	4320	1.11	0.21
rs3796622	10861	SLC26A1	4	972890	7.74E-02	6431	3.66E-01	Up	0.90	9728	1.11	0.04
rs3796622	3425	IDUA	4	972890	7.74E-02	6432	6.45E-01	Up	0.46	11745	1.11	0.02
rs3784308	1588	CYP19A1	15	49314690	7.74E-02	6433	3.78E-01	Down	0.88	9824	1.11	0.04
rs2277814	1291	COL6A1	21	46233931	7.74E-02	6434	1.89E-17	Down	8.50	289	1.11	1.67
rs284844	54838	C10orf26	10	104544519	7.75E-02	6435	4.20E-05	Down	4.10	2242	1.11	0.44
rs8058961	23361	ZNF629	16	30716564	7.75E-02	6436	1.83E-02	Down	2.36	5001	1.11	0.17
rs2242081	10658	CUGBP1	11	47456843	7.76E-02	6437	5.87E-04	Up	3.44	3020	1.11	0.32
rs3219487	114034	TOE1	1	45467648	7.76E-02	6438	2.44E-04	Down	3.67	2701	1.11	0.36
rs3219487	4595	MUTYH	1	45467648	7.76E-02	6439	1.06E-01	Down	1.61	7097	1.11	0.10
rs3219487	84842	HPDL	1	45467648	7.76E-02	6440	8.92E-01	Down	0.14	13194	1.11	0.00
rs941410	7064	THOP1	19	2738329	7.76E-02	6441	4.96E-03	Up	2.81	4064	1.11	0.23
rs941410	6449	SGTA	19	2738329	7.76E-02	6442	3.76E-01	Down	0.89	9808	1.11	0.04
rs454904	7673	ZNF222	19	49203429	7.76E-02	6443	3.05E-01	Down	1.03	9231	1.11	0.05
rs3756323	56126	PCDHB10	5	140559542	7.77E-02	6444	5.81E-05	Up	4.02	2322	1.11	0.42
rs3756323	56127	PCDHB9	5	140559542	7.77E-02	6445	5.77E-02	Down	1.90	6177	1.11	0.12
rs7114583	56253	CRTAM	11	122254424	7.78E-02	6446	4.20E-01	Up	0.81	10125	1.11	0.04
rs7114583	79864	C11orf63	11	122254424	7.78E-02	6447	7.43E-01	Down	0.33	12333	1.11	0.01
rs10034874	51176	LEF1	4	109336054	7.78E-02	6448	1.10E-08	Up	5.70	1070	1.11	0.80
rs1030349	55276	PGM2	4	37650365	7.78E-02	6449	4.41E-01	Down	0.77	10278	1.11	0.04
rs696620	126969	SLC44A3	1	95029485	7.78E-02	6450	4.12E-01	Up	0.82	10068	1.11	0.04
rs12086058	3491	CYR61	1	85752075	7.78E-02	6451	5.99E-02	Up	1.88	6233	1.11	0.12
rs4304537	84066	C1orf49	1	175208211	7.79E-02	6452	4.25E-01	Up	0.80	10155	1.11	0.04
rs2112494	55567	DNAH3	16	20952870	7.79E-02	6453	2.29E-01	Down	1.20	8550	1.11	0.06
rs2070871	5034	P4HB	17	77398423	7.79E-02	6454	4.03E-19	Up	8.93	225	1.11	1.84
rs631411	157983	C9orf66	9	199325	7.79E-02	6455	2.32E-01	Down	1.19	8585	1.11	0.06
rs13188087	170591	S100Z	5	76209478	7.80E-02	6456	3.90E-01	Down	0.86	9912	1.11	0.04
rs4252828	10983	CCNI	4	78343907	7.80E-02	6457	1.58E-01	Down	1.41	7776	1.11	0.08
rs5021087	23581	CASP14	19	15008113	7.80E-02	6458	3.81E-01	Down	0.88	9837	1.11	0.04
rs77938	23305	ACSL6	5	131350393	7.81E-02	6459	9.21E-02	Up	1.68	6864	1.11	0.10
rs7672014	27236	ARFIP1	4	154176106	7.81E-02	6460	1.76E-01	Down	1.35	7977	1.11	0.08
rs11085096	55527	FEM1A	19	4734024	7.81E-02	6461	7.04E-01	Down	0.38	12097	1.11	0.02
rs1864589	23555	TSPAN15	10	70925753	7.81E-02	6462	2.11E-01	Down	1.25	8363	1.11	0.07
rs10518683	4644	MYO5A	15	50441415	7.81E-02	6463	9.16E-01	Up	0.11	13354	1.11	0.00
rs17480477	23473	CAPN7	3	15236389	7.81E-02	6464	9.58E-01	Down	0.05	13608	1.11	0.00
rs7800178	84262	PSMG3	7	1400873	7.82E-02	6465	2.99E-01	Up	1.04	9185	1.11	0.05
rs1148413	10970	CKAP4	12	105126742	7.82E-02	6466	8.53E-02	Up	1.72	6727	1.11	0.11
rs3780670	203	AK1	9	127737387	7.82E-02	6467	1.34E-01	Down	1.50	7499	1.11	0.09
rs7201530	4993	OR2C1	16	3339013	7.83E-02	6468	5.65E-01	Down	0.58	11206	1.11	0.02
rs8093865	7525	YES1	18	755920	7.83E-02	6469	5.00E-06	Up	4.56	1819	1.11	0.53
rs2246709	79424	CYP3A5P2	7	99010370	7.83E-02	6470	4.93E-05	Down	4.06	2278	1.11	0.43
rs2246709	1576	CYP3A4	7	99010370	7.83E-02	6471	2.51E-02	Down	2.24	5290	1.11	0.16
rs7560138	5534	PPP3R1	2	68386417	7.83E-02	6472	1.64E-03	Up	3.15	3430	1.11	0.28
rs11829536	10220	GDF11	12	54426294	7.83E-02	6473	3.97E-01	Up	0.85	9965	1.11	0.04
rs11829536	967	CD63	12	54426294	7.83E-02	6474	4.16E-01	Down	0.81	10096	1.11	0.04
rs2581831	3361	HTR5A	7	154314372	7.84E-02	6475	1.97E-01	Down	1.29	8223	1.11	0.07

rs1592159	64421	DCLRE1C	10	15036657	7.84E-02	6476	3.51E-01	Up	0.93	9593	1.11	0.05
rs710998	79192	IRX1	5	3644916	7.84E-02	6477	3.30E-01	Down	0.97	9446	1.11	0.05
rs2540482	4048	LTA4H	12	94937348	7.85E-02	6478	4.15E-03	Up	2.87	3957	1.11	0.24
rs753202	8531	CSDA	12	10777130	7.85E-02	6479	4.28E-02	Down	2.03	5821	1.11	0.14
rs17041442	23243	ANKRD28	3	15694283	7.85E-02	6480	1.63E-11	Up	6.43	766	1.11	1.08
rs10762485	5660	PSAP	10	73289434	7.86E-02	6481	4.71E-04	Up	3.50	2940	1.10	0.33
rs2941062	51074	APIP	11	34858910	7.86E-02	6482	5.94E-01	Up	0.53	11396	1.10	0.02
rs12810635	80196	RNF34	12	120284587	7.86E-02	6483	1.75E-01	Down	1.36	7963	1.10	0.08
rs10847803		B3GNT4	12	121201411	7.86E-02	6484	7.58E-02	Down	1.78	6544	1.10	0.11
rs10847803	254050	LRRC43	12	121201411	7.86E-02	6485	3.42E-01	Up	0.95	9537	1.10	0.05
rs3728	51660	BRP44L	6	166749090	7.87E-02	6486	2.51E-08	Down	5.57	1146	1.10	0.76
rs11076060	10265	IRX5	16	53506358	7.87E-02	6487	6.18E-01	Up	0.50	11560	1.10	0.02
rs9420907	79991	OBFC1	10	105666455	7.88E-02	6488	1.01E-01	Up	1.64	7019	1.10	0.10
rs8059973	79007	DBNDD1	16	88607035	7.88E-02	6489	3.40E-11	Up	6.65	688	1.10	1.05
rs8059973	2622	GAS8	16	88607035	7.88E-02	6490	9.00E-01	Down	0.13	13243	1.10	0.00
rs498194	148	ADRA1A	8	26759138	7.88E-02	6491	2.69E-05	Down	4.20	2148	1.10	0.46
rs6828669	171024	SYNPO2	4	120304738	7.89E-02	6492	3.06E-48	Down	14.59	7	1.10	4.75
rs13021690	55619	DOCK10	2	225640277	7.89E-02	6493	4.85E-01	Down	0.70	10613	1.10	0.03
rs16962177	2585	GALK2	15	47321994	7.89E-02	6494	1.17E-02	Up	2.52	4622	1.10	0.19
rs2486674	2140	EYA3	1	28104526	7.89E-02	6495	1.20E-07	Up	5.29	1314	1.10	0.69
rs1825031	85438	C4orf35	4	71364116	7.90E-02	6496	1.27E-01	Down	1.53	7407	1.10	0.09
rs9607601	27350	APOBEC3C	22	37723938	7.90E-02	6497	4.46E-10	Down	6.24	844	1.10	0.94
rs6642423	84187	TMEM164		109230174	7.90E-02	6498	5.62E-01	Down	0.58	11179	1.10	0.03
rs2416258	85480	TSLP	5	110447787	7.90E-02	6499	3.97E-08	Down	5.49	1202	1.10	0.74
rs2416258	134430	WDR36	5	110447787	7.90E-02	6500	2.55E-02	Down	2.23	5305	1.10	0.16
rs605902	4200	ME2	18	46699496	7.90E-02	6501	2.84E-02	Down	2.19	5408	1.10	0.15
rs9983586	27005	USP21	21	29309312	7.90E-02	6502	1.07E-01	Up	1.61	7103	1.10	0.10
rs9983586	10600	USP16	21	29309312	7.90E-02	6503	5.28E-01	Down	0.63	10929	1.10	0.03
rs13008860	7840	ALMS1	2	73685674	7.91E-02	6504	4.72E-01	Down	0.72	10510	1.10	0.03
rs6814420	23520	ANP32C	4	165488955	7.91E-02	6505	3.39E-01	Up	0.96	9515	1.10	0.05
rs1954173	84824	FCRLA	1	158409442	7.91E-02	6506	5.75E-01	Up	0.56	11281	1.10	0.02
rs12099131	9246	UBE2L6	11	57104563	7.91E-02	6507	2.35E-04	Down	3.68	2687	1.10	0.36
rs3828246	1146	CHRNG	2	233223720	7.91E-02	6508	4.67E-01	Down	0.73	10477	1.10	0.03
rs3828246	1144	CHRND	2	233223720	7.91E-02	6509	6.99E-01	Up	0.39	12065	1.10	0.02
rs6130691	140730	RIMS4	20	42842532	7.91E-02	6510	7.97E-01	Down	0.26	12654	1.10	0.01
rs7183877	8924	HERC2	15	26039328	7.92E-02	6511	5.43E-01	Up	0.61	11039	1.10	0.03
rs196325	9531	BAG3	10	121418818	7.92E-02	6512	8.28E-06	Down	4.46	1909	1.10	0.51
rs1871500	2958	GTF2A2	15	57737182	7.92E-02	6513	2.13E-04	Up	3.70	2652	1.10	0.37
rs7577050	9801	MRPL19	2	75771574	7.92E-02	6514	8.68E-02	Up	1.71	6751	1.10	0.11
rs2073489	5664	PSEN2	1	223370406	7.93E-02	6515	2.77E-02	Up	2.20	5383	1.10	0.16
rs12472343	64859	OBFC2A	2	192351042	7.93E-02	6516	4.24E-01	Up	0.80	10148	1.10	0.04
rs12513357	26057	ANKRD17	4	74404498	7.93E-02	6517	1.91E-04	Up	3.73	2611	1.10	0.37
rs2496466	51569	UFM1	13	37808766	7.94E-02	6518	4.70E-04	Down	3.50	2938	1.10	0.33
rs2269811	7978	MTERF	7	91154202	7.94E-02	6519	8.89E-03	Up	2.62	4428	1.10	0.21
rs9427662	81494	CFHR5	1	193678432	7.94E-02	6520	1.89E-01	Up	1.31	8141	1.10	0.07
rs4422746	203102	ADAM32	8	39242977	7.94E-02	6521	5.38E-01	Up	0.62	10996	1.10	0.03
rs4760707	23519	ANP32D	12	47134907	7.94E-02	6522	8.73E-01	Down	0.16	13084	1.10	0.01
rs168700	2760	GM2A	5	150599195	7.95E-02	6523	9.01E-01	Up	0.12	13254	1.10	0.00
rs7156293	7043	TGFB3	14	75493336	7.95E-02	6524	1.18E-16	Down	8.29	324	1.10	1.59
rs12414186	6865	TACR2	10	70849620	7.95E-02	6525	9.09E-01	Up	0.11	13303	1.10	0.00
rs1392172	57113	TRPC7	5	135659487	7.95E-02	6526	3.22E-02	Up	2.14	5533	1.10	0.15
rs10667	1466	CSRP2	12	75749618	7.96E-02	6527	4.00E-16	Down	8.14	343	1.10	1.54
rs11217128	6230	RPS25	11	118405573	7.96E-02	6528	5.52E-06	Up	4.54	1834	1.10	0.53
rs11217128	2542	SLC37A4	11	118405573	7.96E-02	6529	3.36E-01	Down	0.96	9491	1.10	0.05
rs11217128	51399	TRAPPC4	11	118405573	7.96E-02	6530	6.94E-01	Up	0.39	12029	1.10	0.02
rs4795893	6347	CCL2	17	29598561	7.96E-02	6531	1.69E-02	Down	2.39	4931	1.10	0.18
rs1317444	374655	ZNF710	15	88399175	7.97E-02	6532	1.65E-02	Up	2.40	4915	1.10	0.18
rs10760702	55014	STX17	9	99767258	7.97E-02	6533	3.62E-01	Down	0.91	9696	1.10	0.04
rs2242378	57182	ANKRD50	4	125990186	7.97E-02	6534	9.38E-02	Up	1.68	6897	1.10	0.10
rs11111153	79023	NUP37	12	100978554	7.97E-02	6535	1.04E-03	Up	3.28	3237	1.10	0.30
rs10082776	5916	RARG	12	51907978	7.98E-02	6536	7.37E-03	Down	2.68	4290	1.10	0.21
rs999867	118980	SFXN2	10	104494554	7.98E-02	6537	6.66E-01	Up	0.43	11860	1.10	0.02
rs223502	4126	MANBA	4	103997961	7.98E-02	6538	7.48E-02	Up	1.78	6525	1.10	0.11
rs4830171	10813	UTP14A		128799065	7.98E-02	6539	5.84E-02	Up	1.89	6204	1.10	0.12
rs1263783	56994	CHPT1	12	100574187	7.98E-02	6540	2.63E-03	Down	3.01	3715	1.10	0.26
rs2072102	10347	ABCA7	19	1024073	7.98E-02	6541	3.04E-01	Down	1.03	9225	1.10	0.05
rs3754026	11218	DDX20	1	111995880	7.98E-02	6542	3.38E-01	Up	0.96	9504	1.10	0.05
rs1366272	202333	CMYA5	5	79084318	7.98E-02	6543	3.95E-01	Down	0.85	9947	1.10	0.04
rs1371135	10239	AP3S2	15	88192274	7.99E-02	6544	4.48E-01	Down	0.76	10337	1.10	0.03
rs751239	54839	LRRC49	15	69071031	7.99E-02	6545	1.72E-02	Down	2.38	4946	1.10	0.18
rs7542294	1116	CHI3L1	1	199882833	7.99E-02	6546	1.70E-03	Up	3.14	3446	1.10	0.28
rs7542294	4608	MYBPH	1	199882833	7.99E-02	6547	1.40E-01	Down	1.48	7574	1.10	0.09
rs10814518	84186	ZCCHC7	9	37292155	7.99E-02	6548	1.10E-08	Up	5.70	1072	1.10	0.80
rs3093993	7919	BAT1	6	31598704	7.99E-02	6549	5.26E-02	Up	1.94	6056	1.10	0.13
rs217079	1075	CTSC	11	87708971	7.99E-02	6550	2.51E-01	Up	1.15	8770	1.10	0.06
rs10497113	151188	ARL6IP6	2	153403294	8.00E-02	6551	5.63E-03	Down	2.77	4145	1.10	0.22
rs7966820	51228	GLTP	12	108764990	8.00E-02	6552	2.14E-01	Down	1.24	8401	1.10	0.07
rs5936558	24137	KIF4A		69418051	8.01E-02	6553	1.28E-01	Up	1.52	7423	1.10	0.09
rs5936558	54857	GDPD2		69418051	8.01E-02	6554	1.35E-01	Up	1.50	7509	1.10	0.09
rs12974444	6415	SEPW1	19	52956801	8.01E-02	6555	1.18E-07	Down	5.30	1313	1.10	0.69
rs12974444	29997	GLTSCR2	19	52956801	8.01E-02	6556	9.09E-01	Down	0.11	13302	1.10	0.00

rs12587228	51637	C14orf166	14	51508087	8.01E-02	6557	3.13E-02	Up	2.15	5509	1.10	0.15
rs3793371	1936	EEF1D	8	144735442	8.01E-02	6558	6.42E-05	Up	4.00	2342	1.10	0.42
rs3793371	84948	TIGD5	8	144735442	8.01E-02	6559	1.34E-01	Down	1.50	7496	1.10	0.09
rs3793371	93100	NAPRT1	8	144735442	8.01E-02	6560	8.86E-01	Down	0.14	13165	1.10	0.01
rs11878440	79898	ZNF613	19	57106447	8.01E-02	6561	3.53E-04	Up	3.57	2838	1.10	0.35
rs17741410	759	CA1	8	86419317	8.01E-02	6562	3.29E-02	Up	2.13	5559	1.10	0.15
rs2267113	4330	MN1	22	26479958	8.01E-02	6563	2.75E-01	Down	1.09	8993	1.10	0.06
rs4934027	4143	MAT1A	10	82025540	8.02E-02	6564	9.59E-01	Down	0.05	13614	1.10	0.00
rs2191312	6983	TRGV9	7	38140499	8.02E-02	6565	2.40E-03	Up	3.04	3660	1.10	0.26
rs17371084	9857	CEP350	1	176677047	8.02E-02	6566	4.60E-02	Down	2.00	5896	1.10	0.13
rs6114326	1470	CST2	20	23763299	8.04E-02	6567	1.04E-02	Up	2.56	4539	1.09	0.20
rs3786320	10982	MAPRE2	18	30951841	8.04E-02	6568	2.16E-07	Down	5.19	1383	1.09	0.67
rs157580	348	APOE	19	50087106	8.04E-02	6569	5.97E-02	Up	1.88	6232	1.09	0.12
rs5960	2159	F10	13	112849738	8.05E-02	6570	9.32E-02	Down	1.68	6888	1.09	0.10
rs2512219	283159	OR8D1	11	123694939	8.05E-02	6571	1.16E-02	Up	2.52	4617	1.09	0.19
rs2512219	283160	OR8D2	11	123694939	8.05E-02	6572	4.39E-01	Down	0.77	10262	1.09	0.04
rs1112438	64689	GORASP1	3	39127349	8.06E-02	6573	6.25E-01	Down	0.49	11614	1.09	0.02
rs11102043	10768	AHCYL1	1	110229438	8.06E-02	6574	4.08E-01	Down	0.83	10042	1.09	0.04
rs13193513	51465	UBE2J1	6	90087772	8.06E-02	6575	1.77E-11	Up	6.74	661	1.09	1.08
rs548973	8456	FOXN1	17	23880388	8.06E-02	6576	7.57E-02	Down	1.78	6542	1.09	0.11
rs1508890	11082	ESM1	5	54315764	8.06E-02	6577	2.36E-03	Up	3.04	3650	1.09	0.26
rs1265930	65258	MPPE1	18	11891135	8.07E-02	6578	1.86E-09	Down	6.01	935	1.09	0.87
rs168208	131375	LYZL4	3	42442923	8.07E-02	6579	2.64E-01	Down	1.12	8900	1.09	0.06
rs11691107	10913	EDAR	2	109044955	8.07E-02	6580	6.99E-04	Down	3.39	3076	1.09	0.32
rs6027217	60437	CDH26	20	57981747	8.07E-02	6581	9.56E-03	Down	2.59	4474	1.09	0.20
rs444170	57707	KIAA1609	16	83071842	8.08E-02	6582	1.16E-02	Down	2.52	4611	1.09	0.19
rs973126	9061	PAPSS1	4	108998904	8.08E-02	6583	7.47E-05	Up	3.96	2374	1.09	0.41
rs6010789	54994	C20orf11	20	61031598	8.08E-02	6584	5.58E-01	Up	0.59	11147	1.09	0.03
rs2240615	57418	WDR18	19	915434	8.08E-02	6585	1.26E-01	Up	1.53	7384	1.09	0.09
rs6709507	1876	E2F6	2	11551802	8.08E-02	6586	2.66E-02	Down	2.22	5342	1.09	0.16
rs2492303	154091	SLC2A12	6	134388701	8.09E-02	6587	7.13E-02	Up	1.80	6457	1.09	0.11
rs6616440	55859	BEX1		102118461	8.09E-02	6588	2.71E-09	Down	5.95	960	1.09	0.86
rs10500661	1262	CNGA4	11	6230320	8.09E-02	6589	6.99E-01	Down	0.39	12062	1.09	0.02
rs10904442	1109	AKR1C4	10	5252361	8.09E-02	6590	1.28E-02	Up	2.49	4670	1.09	0.19
rs13175179	408263	C5orf40	5	156714533	8.09E-02	6591	7.68E-02	Down	1.77	6573	1.09	0.11
rs6441295	55076	TMEM45A	3	101707131	8.09E-02	6592	5.06E-05	Down	4.05	2292	1.09	0.43
rs13333521	51704	GPRC5B	16	19811583	8.09E-02	6593	7.94E-32	Down	11.74	53	1.09	3.11
rs238358	11215	AKAP11	13	41736674	8.10E-02	6594	1.45E-04	Down	3.80	2541	1.09	0.38
rs294185	712	C1QA	1	22717198	8.10E-02	6595	4.65E-02	Up	1.99	5910	1.09	0.13
rs13375942	5320	PLA2G2A	1	20037684	8.10E-02	6596	1.48E-11	Up	6.74	658	1.09	1.08
rs7404082	55239	OGFOD1	16	55056012	8.10E-02	6597	1.97E-01	Down	1.29	8222	1.09	0.07
rs749062	83643	CCDC3	10	13043448	8.11E-02	6598	1.05E-05	Down	4.41	1960	1.09	0.50
rs4704514	10184	LHFPL2	5	77855837	8.11E-02	6599	2.96E-15	Down	7.89	379	1.09	1.45
rs7296106	3034	HAL	12	94906362	8.11E-02	6600	2.30E-02	Up	2.27	5203	1.09	0.16
rs7197475	78994	PRR14	16	30550368	8.11E-02	6601	5.43E-01	Down	0.61	11041	1.09	0.03
rs4732850	55893	ZNF395	8	28254635	8.11E-02	6602	4.95E-11	Down	6.57	713	1.09	1.03
rs4883627	22953	P2RX2	12	131817762	8.11E-02	6603	2.22E-03	Down	3.06	3600	1.09	0.27
rs10490798	64147	KIF9	3	47303748	8.12E-02	6604	7.28E-13	Up	7.17	544	1.09	1.21
rs10490798	23276	KLHL18	3	47303748	8.12E-02	6605	1.57E-02	Down	2.41	4863	1.09	0.18
rs2918285	22885	ABLIM3	5	148567746	8.12E-02	6606	4.85E-03	Down	2.82	4042	1.09	0.23
rs7526662	9698	PUM1	1	31208144	8.12E-02	6607	3.17E-02	Up	2.15	5517	1.09	0.15
rs10902222	283229	EFCAB4A	11	800882	8.12E-02	6608	3.05E-08	Up	5.54	1167	1.09	0.75
rs4715723	221687	RNF182	6	14066633	8.12E-02	6609	2.02E-01	Down	1.27	8281	1.09	0.07
rs10902222	6181	RPLP2	11	800882	8.12E-02	6610	2.16E-01	Up	1.24	8421	1.09	0.07
rs10902222	55367	LRDD	11	800882	8.12E-02	6611	5.58E-01	Up	0.59	11142	1.09	0.03
rs10902222	57104	PNPLA2	11	800882	8.12E-02	6612	7.32E-01	Down	0.34	12267	1.09	0.01
rs6577565	387509	GPR153	1	6241521	8.12E-02	6613	8.86E-02	Down	1.70	6776	1.09	0.11
rs1889337	9635	CLCA2	1	86616346	8.12E-02	6614	6.40E-01	Down	0.47	11713	1.09	0.02
rs998794	56128	PCDHB8	5	140546893	8.13E-02	6615	2.31E-03	Up	3.05	3632	1.09	0.26
rs11602501	85456	TNKS1BP1	11	56829809	8.13E-02	6616	4.86E-03	Up	2.82	4046	1.09	0.23
rs2961618	83592	AKR1CL2	10	4840639	8.13E-02	6617	5.45E-01	Down	0.61	11050	1.09	0.03
rs10206984	10109	ARPC2	2	218892486	8.13E-02	6618	4.20E-03	Down	2.86	3967	1.09	0.24
rs11930623	60312	AFAP1	4	8049753	8.14E-02	6619	4.78E-01	Up	0.71	10556	1.09	0.03
rs2007540	3419	IDH3A	15	76216395	8.14E-02	6620	1.37E-12	Down	7.09	564	1.09	1.19
rs11974030	55303	GIMAP4	7	149717180	8.14E-02	6621	8.45E-01	Up	0.20	12928	1.09	0.01
rs4666572	4760	NEUROD1	2	182381070	8.14E-02	6622	2.47E-02	Up	2.25	5276	1.09	0.16
rs5983119	25878	MXRA5		3234617	8.14E-02	6623	8.87E-01	Up	0.14	13170	1.09	0.01
rs38025	10131	TRAP1	16	3703735	8.14E-02	6624	4.33E-17	Up	8.39	306	1.09	1.64
rs7615134	64419	MTMR14	3	9710085	8.14E-02	6625	3.07E-01	Up	1.02	9245	1.09	0.05
rs1400772	8842	PROM1	4	15714611	8.15E-02	6626	1.88E-02	Down	2.35	5015	1.09	0.17
rs241437	3112	HLA-DOB	6	32905662	8.15E-02	6627	4.02E-01	Up	0.84	9995	1.09	0.04
rs2714714	79581	GPR172A	15	22929705	8.15E-02	6628	1.15E-04	Up	3.86	2487	1.09	0.39
rs4773341	121793	C13orf16	13	110752635	8.15E-02	6629	1.37E-01	Down	1.49	7537	1.09	0.09
rs3915052	6750	SST	3	188890618	8.15E-02	6630	5.86E-01	Down	0.54	11349	1.09	0.02
rs17659192	3568	IL5RA	3	3083711	8.15E-02	6631	4.52E-01	Up	0.75	10356	1.09	0.03
rs1981663	26033	ATRNL1	10	117423001	8.15E-02	6632	7.38E-05	Down	3.96	2371	1.09	0.41
rs553316	55795	PCID2	13	112886016	8.16E-02	6633	9.29E-01	Up	0.09	13421	1.09	0.00
rs8061066	8720	MBTPS1	16	82637410	8.16E-02	6634	2.25E-05	Down	4.24	2111	1.09	0.46
rs16892054	57545	CC2D2A	4	15172539	8.16E-02	6635	5.40E-02	Down	1.93	6088	1.09	0.13
rs7038940	2649	NR6A1	9	124589794	8.16E-02	6636	5.41E-03	Down	2.78	4117	1.09	0.23
rs7648620	55779	WDR52	3	114555446	8.16E-02	6637	2.42E-01	Up	1.17	8680	1.09	0.06

rs12187268	202243	CCDC125	5	68661542	8.17E-02	6638	3.45E-03	Up	2.92	3850	1.09	0.25
rs2278426	57572	DOCK6	19	11211488	8.17E-02	6639	2.35E-01	Up	1.19	8618	1.09	0.06
rs12983706	126074	C19orf39	19	11334415	8.18E-02	6640	4.20E-03	Up	2.86	3966	1.09	0.24
rs13424275	56287	GKN1	2	69117593	8.18E-02	6641	9.90E-01	Down	0.01	13844	1.09	0.00
rs4726625	259287	TAS2R41	7	142688239	8.18E-02	6642	6.86E-02	Down	1.82	6403	1.09	0.12
rs11210729	114625	ERMAP	1	42975221	8.18E-02	6643	3.35E-04	Up	3.59	2817	1.09	0.35
rs2259444	7637	ZNF84	12	132252849	8.19E-02	6644	1.63E-05	Up	4.31	2056	1.09	0.48
rs2259444	7699	ZNF140	12	132252849	8.19E-02	6645	1.92E-04	Up	3.73	2620	1.09	0.37
rs2805888	23731	C9orf5	9	108960291	8.19E-02	6646	2.49E-02	Up	2.24	5281	1.09	0.16
rs1548320	8710	SERPINB7	18	59594119	8.20E-02	6647	1.43E-01	Up	1.46	7608	1.09	0.08
rs8088313	81929	SEH1L	18	12967206	8.20E-02	6648	7.44E-14	Up	7.47	464	1.09	1.31
rs4246521	10623	POLR3C	1	143115023	8.20E-02	6649	1.25E-04	Down	3.84	2499	1.09	0.39
rs6016004	60436	TGIF2	20	34654572	8.22E-02	6650	1.80E-01	Down	1.34	8029	1.09	0.07
rs6016004	55969	C20orf24	20	34654572	8.22E-02	6651	8.51E-01	Up	0.19	12965	1.09	0.01
rs10518114	174	AFP	4	74679894	8.22E-02	6652	2.86E-01	Down	1.07	9087	1.09	0.05
rs10049840	2712	GK2	4	80689218	8.22E-02	6653	4.08E-02	Up	2.05	5769	1.09	0.14
rs2270150	9496	TBX4	17	56915467	8.22E-02	6654	7.00E-01	Down	0.38	12075	1.09	0.02
rs937815	55205	ZNF532	18	54718886	8.22E-02	6655	8.53E-32	Down	11.73	54	1.08	3.11
rs6606733	84749	USP30	12	107950951	8.23E-02	6656	9.49E-01	Up	0.06	13546	1.08	0.00
rs6924948	255626	HIST1H2BA	6	25843945	8.23E-02	6657	2.29E-01	Up	1.20	8545	1.08	0.06
rs3895063	6888	TALDO1	11	745659	8.23E-02	6658	3.77E-01	Up	0.88	9821	1.08	0.04
rs12343347	23064	SETX	9	132221617	8.23E-02	6659	3.24E-06	Down	4.66	1753	1.08	0.55
rs3759217	1027	CDKN1B	12	12759719	8.23E-02	6660	5.91E-05	Down	4.02	2328	1.08	0.42
rs376699	54431	DNAJC10	2	183388154	8.24E-02	6661	3.22E-37	Up	12.74	28	1.08	3.65
rs6062894	57642	COL20A1	20	61425120	8.24E-02	6662	3.22E-01	Down	0.99	9375	1.08	0.05
rs10846246	153443	SRFBP1	12	15942757	8.24E-02	6663	5.02E-02	Up	1.96	6005	1.08	0.13
rs10846246	11171	STRAP	12	15942757	8.24E-02	6664	1.37E-01	Down	1.49	7538	1.08	0.09
rs10892592	2900	GRIK4	11	120027213	8.24E-02	6665	1.09E-01	Down	1.60	7135	1.08	0.10
rs1491850	627	BDNF	11	27706301	8.25E-02	6666	2.32E-01	Down	1.20	8583	1.08	0.06
rs3795720	8643	PTCH2	1	44976599	8.25E-02	6667	5.77E-01	Up	0.56	11294	1.08	0.02
rs3795720	8891	EIF2B3	1	44976599	8.25E-02	6668	6.44E-01	Up	0.46	11740	1.08	0.02
rs3788094	56894	AGPAT3	21	44217274	8.25E-02	6669	4.42E-11	Up	6.59	706	1.08	1.04
rs7609320	57448	BIRC6	2	32545341	8.26E-02	6670	1.07E-04	Up	3.87	2470	1.08	0.40
rs11089784	23780	APOL2	22	34950777	8.26E-02	6671	7.69E-01	Down	0.29	12481	1.08	0.01
rs17021996	83894	TTC29	4	148112424	8.26E-02	6672	7.40E-01	Up	0.33	12317	1.08	0.01
rs2043315	113835	ZNF257	19	22044663	8.26E-02	6673	5.00E-02	Up	1.96	5998	1.08	0.13
rs8052106	4500	MT1L	16	55192602	8.27E-02	6674	5.50E-08	Down	5.43	1234	1.08	0.73
rs8052106	4502	MT2A	16	55192602	8.27E-02	6675	1.31E-02	Down	2.48	4695	1.08	0.19
rs2252528	23467	NPTXR	22	37544432	8.27E-02	6676	2.78E-01	Down	1.08	9022	1.08	0.06
rs3762296	7805	LAPTM5	1	30900479	8.27E-02	6677	6.07E-02	Up	1.88	6247	1.08	0.12
rs3849904	360	AQP3	9	33424026	8.27E-02	6678	2.44E-19	Down	8.99	219	1.08	1.86
rs6427053	10223	GPA33	1	163748511	8.28E-02	6679	1.41E-01	Down	1.47	7595	1.08	0.08
rs8063316	6376	CX3CL1	16	55981359	8.29E-02	6680	8.05E-07	Down	4.93	1548	1.08	0.61
rs7259810	808	CALM3	19	51795268	8.29E-02	6681	9.74E-02	Down	1.66	6949	1.08	0.10
rs1479927	140462	ASB9		15042479	8.29E-02	6682	1.24E-05	Up	4.37	2000	1.08	0.49
rs2385799	57677	ZFP14	19	41568771	8.30E-02	6683	4.45E-02	Down	2.01	5866	1.08	0.14
rs1870566	8091	HMGA2	12	64498284	8.30E-02	6684	4.40E-02	Up	2.01	5851	1.08	0.14
rs2580873	57379	AICDA	12	8652941	8.31E-02	6685	1.88E-01	Down	1.32	8119	1.08	0.07
rs2071390	226	ALDOA	16	29988868	8.31E-02	6686	2.45E-07	Down	5.16	1394	1.08	0.66
rs2071390	5531	PPP4C	16	29988868	8.31E-02	6687	1.10E-03	Up	3.26	3255	1.08	0.30
rs4938863	5024	P2RX3	11	56883284	8.32E-02	6688	1.51E-02	Up	2.43	4816	1.08	0.18
rs10947262	56244	BTNL2	6	32481290	8.32E-02	6689	3.64E-01	Down	0.91	9717	1.08	0.04
rs3771395	25806	VAX2	2	71044669	8.33E-02	6690	2.81E-01	Up	1.08	9039	1.08	0.06
rs4653051	1912	PHC2	1	33468021	8.33E-02	6691	1.84E-02	Down	2.36	5004	1.08	0.17
rs7729306	64848	YTHDC2	5	112954080	8.34E-02	6692	8.43E-03	Up	2.63	4378	1.08	0.21
rs579992	10911	UTS2	1	7862247	8.35E-02	6693	7.87E-02	Up	1.76	6607	1.08	0.11
rs11211037	1263	PLK3	1	44944644	8.35E-02	6694	3.82E-01	Up	0.87	9855	1.08	0.04
rs12737517	2882	GPX7	1	52764014	8.35E-02	6695	7.86E-06	Down	4.47	1897	1.08	0.51
rs17836409	11006	LILRB4	19	59875681	8.35E-02	6696	5.72E-02	Up	1.90	6163	1.08	0.12
rs7462301	4609	MYC	8	128826532	8.36E-02	6697	5.31E-41	Up	13.38	17	1.08	4.03
rs9927281	8303	SNN	16	11660924	8.36E-02	6698	6.97E-01	Down	0.39	12048	1.08	0.02
rs877292	1375	CPT1B	22	49297632	8.36E-02	6699	2.28E-08	Up	5.59	1138	1.08	0.76
rs877292	1120	CHKB	22	49297632	8.36E-02	6700	4.43E-01	Down	0.77	10287	1.08	0.04
rs7835528	4062	LY6H	8	144314767	8.37E-02	6701	9.03E-01	Down	0.12	13266	1.08	0.00
rs748472	4649	MYO9A	15	70054218	8.37E-02	6702	1.58E-02	Down	2.41	4868	1.08	0.18
rs17755917	10950	BTG3	21	17878576	8.37E-02	6703	1.27E-18	Down	8.81	244	1.08	1.79
rs657079	51365	PLA1A	3	120828640	8.37E-02	6704	3.34E-23	Up	9.92	138	1.08	2.25
rs10439225	10920	COPS8	2	237772561	8.37E-02	6705	7.90E-06	Down	4.47	1900	1.08	0.51
rs4808521	79041	TMEM38A	19	16646721	8.37E-02	6706	2.97E-01	Up	1.04	9169	1.08	0.05
rs10804774	8833	GMPS	3	157136719	8.38E-02	6707	1.10E-02	Up	2.54	4572	1.08	0.20
rs2275007	4860	NP	14	19990090	8.38E-02	6708	1.08E-02	Up	2.55	4563	1.08	0.20
rs2275007	55644	OSGEP	14	19990090	8.38E-02	6709	3.30E-01	Down	0.97	9438	1.08	0.05
rs2275007	171017	ZNF384	14	19990090	8.38E-02	6710	5.61E-01	Down	0.58	11170	1.08	0.03
rs908511	144402	CPNE8	12	37587008	8.38E-02	6711	1.85E-01	Down	1.33	8084	1.08	0.07
rs2805915	8870	IER3	10	70531982	8.39E-02	6712	5.67E-03	Down	2.77	4150	1.08	0.22
rs2469950	65059	RAPH1	2	204161063	8.39E-02	6713	3.98E-03	Up	2.88	3930	1.08	0.24
rs10992568	89846	FGD3	9	92832874	8.39E-02	6714	6.09E-02	Down	1.87	6251	1.08	0.12
rs5905283	11254	SLC6A14		115377763	8.39E-02	6715	4.24E-01	Up	0.80	10147	1.08	0.04
rs567430	2986	GUCY2F		108533038	8.39E-02	6716	2.57E-01	Down	1.13	8839	1.08	0.06
rs11072518	9377	COX5A	15	73021663	8.40E-02	6717	5.00E-08	Up	5.45	1220	1.08	0.73
rs11864646	6367	CCL22	16	55932768	8.40E-02	6718	8.79E-01	Down	0.15	13117	1.08	0.01

rs12495216	9175	MAP3K13	3	186609544	8.40E-02	6719	6.46E-05	Up	4.00	2345	1.08	0.42
rs11991758	5150	PDE7A	8	66832824	8.40E-02	6720	6.80E-01	Up	0.41	11944	1.08	0.02
rs745327	605	BCL7A	12	120919244	8.41E-02	6721	8.29E-02	Up	1.73	6683	1.08	0.11
rs12325819	4905	NSF	17	42171296	8.41E-02	6722	3.37E-10	Up	6.24	840	1.08	0.95
rs4123535	10267	RAMP1	2	238598201	8.42E-02	6723	3.87E-01	Up	0.87	9890	1.07	0.04
rs2082392	6690	SPINK1	5	147205381	8.43E-02	6724	7.11E-02	Up	1.81	6454	1.07	0.11
rs3851117	349667	RTN4RL2	11	56993689	8.43E-02	6725	3.43E-01	Up	0.95	9550	1.07	0.05
rs5909064	6658	SOX3		139315301	8.43E-02	6726	4.34E-01	Down	0.78	10226	1.07	0.04
rs10518414	10252	SPRY1	4	124663414	8.44E-02	6727	4.62E-02	Down	1.99	5899	1.07	0.13
rs2413583	5155	PDGFB	22	37984273	8.45E-02	6728	5.08E-01	Up	0.66	10768	1.07	0.03
rs4652554	3140	MR1	1	177763812	8.45E-02	6729	4.38E-11	Down	6.59	703	1.07	1.04
rs2526748	10180	RBM6	3	50055443	8.45E-02	6730	2.07E-14	Up	7.64	432	1.07	1.37
rs6851444	4889	NPY5R	4	164640690	8.45E-02	6731	3.21E-01	Up	0.99	9367	1.07	0.05
rs1256430	9455	HOMER2	15	81316526	8.46E-02	6732	9.41E-05	Up	3.91	2436	1.07	0.40
rs1719480	10871	CD300C	17	70062993	8.46E-02	6733	9.95E-01	Up	0.01	13876	1.07	0.00
rs7672337	54798	DCHS2	4	155609215	8.46E-02	6734	9.04E-02	Down	1.69	6833	1.07	0.10
rs2297753	2330	FMO5	1	143908947	8.46E-02	6735	3.58E-15	Up	7.87	383	1.07	1.44
rs4459653	342909	ZNF284	19	49291455	8.46E-02	6736	1.13E-02	Up	2.53	4592	1.07	0.19
rs4459653	7767	ZNF224	19	49291455	8.46E-02	6737	5.80E-01	Down	0.55	11318	1.07	0.02
rs814528	57731	SPTBN4	19	45706762	8.46E-02	6738	1.27E-01	Down	1.53	7395	1.07	0.09
rs7805240	4289	MKLN1	7	130578226	8.47E-02	6739	2.46E-01	Up	1.16	8721	1.07	0.06
rs10846489	91574	C12orf65	12	122268807	8.47E-02	6740	4.49E-05	Down	4.08	2255	1.07	0.43
rs2972607	9704	DHX34	19	52542117	8.48E-02	6741	1.79E-03	Up	3.12	3472	1.07	0.27
rs2972607	27202	GPR77	19	52542117	8.48E-02	6742	2.11E-02	Down	2.31	5119	1.07	0.17
rs6609281	25763	CXorf27		37594311	8.48E-02	6743	1.52E-01	Down	1.43	7705	1.07	0.08
rs1254901	10791	VAMP5	2	85722887	8.49E-02	6744	1.33E-08	Down	5.68	1078	1.07	0.79
rs1254901	8673	VAMP8	2	85722887	8.49E-02	6745	1.24E-01	Up	1.54	7358	1.07	0.09
rs1920145	2328	FMO3	1	167777458	8.49E-02	6746	5.17E-02	Up	1.95	6032	1.07	0.13
rs6761375	316	AOX1	2	201311336	8.49E-02	6747	3.25E-18	Down	8.70	262	1.07	1.75
rs3760049	6650	SOLH	16	515313	8.49E-02	6748	4.61E-01	Up	0.74	10433	1.07	0.03
rs7127877	10902	BRD8	11	16740400	8.50E-02	6749	9.30E-01	Down	0.09	13430	1.07	0.00
rs7127877	10944	C11orf58	11	16740400	8.50E-02	6750	9.48E-01	Down	0.07	13539	1.07	0.00
rs12769713	9211	LGI1	10	95511220	8.50E-02	6751	9.04E-01	Up	0.12	13277	1.07	0.00
rs13102012	132949	AASDH	4	57065447	8.50E-02	6752	5.24E-01	Up	0.64	10893	1.07	0.03
rs4758441	6881	TAF10	11	6583251	8.50E-02	6753	5.25E-05	Up	4.04	2301	1.07	0.43
rs4758441	3611	ILK	11	6583251	8.50E-02	6754	1.04E-04	Down	3.88	2466	1.07	0.40
rs4758441	23378	KIAA0409	11	6583251	8.50E-02	6755	2.44E-01	Up	1.17	8699	1.07	0.06
rs7163031	79768	C15orf29	15	32261745	8.50E-02	6756	3.65E-01	Up	0.91	9719	1.07	0.04
rs1573041	101	ADAM8	10	134980683	8.51E-02	6757	3.00E-04	Up	3.62	2772	1.07	0.35
rs1573041	10844	TUBGCP2	10	134980683	8.51E-02	6758	3.83E-01	Up	0.87	9864	1.07	0.04
rs16930770	796	CALCA	11	14954089	8.51E-02	6759	4.94E-01	Up	0.68	10673	1.07	0.03
rs3795904	80895	ILKAP	2	238884590	8.52E-02	6760	1.27E-01	Down	1.52	7410	1.07	0.09
rs10485768	79133	C20orf7	20	13746549	8.52E-02	6761	3.60E-02	Up	2.10	5649	1.07	0.14
rs4148211	64241	ABCG8	2	43983394	8.52E-02	6762	4.46E-01	Up	0.76	10316	1.07	0.04
rs1805018	221400	TDRD6	6	46787262	8.52E-02	6763	3.98E-03	Up	2.88	3929	1.07	0.24
rs2304673	8864	PER2	2	238967922	8.53E-02	6764	1.70E-04	Down	3.76	2576	1.07	0.38
rs2705886	201973	CCDC111	4	185983740	8.53E-02	6765	6.43E-02	Up	1.85	6326	1.07	0.12
rs2240903	163	AP2B1	17	31076270	8.53E-02	6766	1.19E-01	Up	1.56	7273	1.07	0.09
rs17633078	84056	KATNAL1	13	29728634	8.53E-02	6767	5.09E-07	Down	5.02	1481	1.07	0.63
rs4844484	50486	G0S2	1	206226078	8.53E-02	6768	4.57E-02	Down	2.00	5887	1.07	0.13
rs11641695	29105	C16orf80	16	56739065	8.54E-02	6769	3.23E-01	Up	0.99	9386	1.07	0.05
rs8099878	6253	RTN2	19	50711441	8.54E-02	6770	3.34E-03	Down	2.93	3832	1.07	0.25
rs8099878	7408	VASP	19	50711441	8.54E-02	6771	4.35E-01	Up	0.78	10228	1.07	0.04
rs1051740	2052	EPHX1	1	222326368	8.54E-02	6772	6.66E-02	Down	1.83	6367	1.07	0.12
rs2869580	8787	RGS9	17	60639256	8.54E-02	6773	7.11E-04	Down	3.39	3083	1.07	0.31
rs2869580	388531	RGS9BP	17	60639256	8.54E-02	6774	8.06E-01	Up	0.25	12708	1.07	0.01
rs3884571	27295	PDLIM3	4	186795038	8.54E-02	6775	9.13E-03	Down	2.61	4443	1.07	0.20
rs6776592	6259	RYK	3	135352909	8.55E-02	6776	7.81E-04	Down	3.36	3125	1.07	0.31
rs4140553	8876	VNN1	6	133037470	8.55E-02	6777	6.37E-03	Up	2.73	4201	1.07	0.22
rs4675217	3609	ILF3	2	202369016	8.55E-02	6778	2.33E-03	Up	3.04	3642	1.07	0.26
rs4675217	58538	MPP4	2	202369016	8.55E-02	6779	2.79E-01	Down	1.08	9026	1.07	0.06
rs11132383	3818	KLKB1	4	187557809	8.56E-02	6780	1.75E-01	Up	1.36	7959	1.07	0.08
rs10800784	1465	CSRP1	1	198197714	8.56E-02	6781	6.76E-19	Down	8.88	234	1.07	1.82
rs6656287	90853	SPOCD1	1	31921708	8.56E-02	6782	6.03E-01	Down	0.52	11449	1.07	0.02
rs3804775	57092	PCNP	3	102789197	8.57E-02	6783	8.52E-06	Down	4.45	1919	1.07	0.51
rs998520	57062	DDX24	14	93624032	8.57E-02	6784	7.51E-01	Down	0.32	12379	1.07	0.01
rs4233366		B4GALT3	1	157972220	8.58E-02	6785	4.46E-19	Up	8.92	227	1.07	1.84
rs8032946	7090	TLE3	15	68133223	8.58E-02	6786	7.29E-13	Up	7.17	545	1.07	1.21
rs2250818	2050	EPHB4	7	100066807	8.59E-02	6787	9.76E-02	Down	1.66	6954	1.07	0.10
rs6752371	51652	VPS24	2	86704779	8.59E-02	6788	2.01E-04	Down	3.72	2633	1.07	0.37
rs10188066	3938	LCT	2	136373245	8.59E-02	6789	4.31E-01	Up	0.79	10206	1.07	0.04
rs1248696	9231	DLG5	10	79286611	8.59E-02	6790	4.74E-07	Down	5.04	1475	1.07	0.63
rs11740632	79993	ELOVL7	5	60177002	8.59E-02	6791	2.86E-01	Up	1.07	9080	1.07	0.05
rs215897	10261	IGSF6	16	21585570	8.59E-02	6792	7.91E-01	Up	0.27	12614	1.07	0.01
rs7251590	1455	CSNK1G2	19	1940867	8.60E-02	6793	8.25E-01	Up	0.22	12819	1.07	0.01
rs1062225	5599	MAPK8	10	49313232	8.61E-02	6794	1.97E-03	Up	3.10	3523	1.07	0.27
rs12918570	29070	CCDC113	16	56825937	8.61E-02	6795	1.68E-03	Up	3.14	3442	1.07	0.28
rs3762864	1998	ELF2	4	140397800	8.61E-02	6796	6.01E-02	Down	1.88	6238	1.07	0.12
rs1499966	3693	ITGB5	3	126067904	8.61E-02	6797	5.58E-01	Up	0.59	11150	1.07	0.03
rs17370763	26092	TOR1AIP1	1	176598531	8.61E-02	6798	7.67E-18	Down	8.60	274	1.06	1.71
rs1372325	59	ACTA2	10	90683549	8.62E-02	6799	4.41E-20	Down	9.18	195	1.06	1.94

rs808141	3730	KAL1		8298260	8.62E-02	6800	2.26E-01	Down	1.21	8518	1.06	0.06
rs9685958	55286	C4orf19	4	37392532	8.62E-02	6801	2.36E-03	Down	3.04	3649	1.06	0.26
rs6594950	51397	COMMD10	5	115510693	8.62E-02	6802	4.93E-02	Up	1.97	5976	1.06	0.13
rs5996577	3543	IGLL1	22	22245981	8.62E-02	6803	8.55E-01	Down	0.18	12986	1.06	0.01
rs2369215	5255	PHKA1		71600273	8.62E-02	6804	3.44E-03	Up	2.92	3846	1.06	0.25
rs4679394	56667	MUC13	3	126129527	8.63E-02	6805	3.88E-02	Down	2.07	5722	1.06	0.14
rs9967977	94104	C21orf66	21	33058897	8.63E-02	6806	4.10E-01	Down	0.82	10055	1.06	0.04
rs12800438	1717	DHCR7	11	70848651	8.63E-02	6807	4.83E-02	Up	1.97	5957	1.06	0.13
rs12800438	55191	NADSYN1	11	70848651	8.63E-02	6808	7.01E-01	Down	0.38	12082	1.06	0.02
rs9655959	79689	STEAP4	7	87553039	8.63E-02	6809	2.86E-08	Up	5.56	1152	1.06	0.75
rs707204	88	ACTN2	1	233223417	8.64E-02	6810	7.70E-01	Up	0.29	12496	1.06	0.01
rs7098277	132	ADK	10	76100549	8.64E-02	6811	5.91E-04	Up	3.44	3023	1.06	0.32
rs11576262	9398	IGSF2	1	117238097	8.64E-02	6812	8.84E-01	Down	0.15	13152	1.06	0.01
rs12566176	9019	MPZL1	1	164428717	8.65E-02	6813	1.18E-04	Up	3.85	2493	1.06	0.39
rs4825100	51438	MAGEC2		141029774	8.65E-02	6814	8.70E-01	Up	0.16	13066	1.06	0.01
rs2586238	29928	TIMM22	17	849072	8.65E-02	6815	9.78E-01	Down	0.03	13754	1.06	0.00
rs12709959	63934	ZNF667	19	61660126	8.65E-02	6816	1.49E-01	Up	1.44	7662	1.06	0.08
rs7486178	89910	UBE3B	12	108442986	8.66E-02	6817	2.43E-01	Down	1.17	8697	1.06	0.06
rs7752412	63971	KIF13A	6	18022783	8.66E-02	6818	4.85E-01	Down	0.70	10612	1.06	0.03
rs1553468	8506	CNTNAP1	17	38103368	8.66E-02	6819	9.16E-04	Down	3.32	3181	1.06	0.30
rs928110	5015	OTX2	14	56349192	8.66E-02	6820	4.29E-01	Down	0.79	10187	1.06	0.04
rs744873	251	ALPPL2	2	233106070	8.67E-02	6821	2.09E-01	Down	1.26	8338	1.06	0.07
rs3737445	5519	PPP2R1B	11	111130776	8.67E-02	6822	3.56E-12	Up	6.97	606	1.06	1.14
rs2853585	55127	HEATR1	1	233085866	8.67E-02	6823	4.29E-01	Up	0.79	10188	1.06	0.04
rs12984133	80131	LRRC8E	19	7841716	8.67E-02	6824	8.68E-03	Up	2.62	4404	1.06	0.21
rs11100096	2743	GLRB	4	158393715	8.67E-02	6825	2.09E-01	Up	1.26	8337	1.06	0.07
rs3760595	26256	CABYR	18	19970771	8.68E-02	6826	5.45E-02	Down	1.92	6101	1.06	0.13
rs9639820	8621	CDC2L5	7	39867881	8.68E-02	6827	1.70E-02	Up	2.39	4937	1.06	0.18
rs304303	9682	JMJD2A	1	43847163	8.68E-02	6828	8.00E-05	Up	3.94	2390	1.06	0.41
rs412334	3992	FADS1	11	61316837	8.68E-02	6829	1.19E-28	Down	11.10	75	1.06	2.79
rs8110516	10148	EBI3	19	4165467	8.68E-02	6830	8.50E-02	Up	1.72	6721	1.06	0.11
rs634229	54765	TRIM44	11	35781367	8.68E-02	6831	3.46E-01	Up	0.94	9566	1.06	0.05
rs7141881	3429	IFI27	14	93653617	8.68E-02	6832	2.70E-02	Down	2.21	5362	1.06	0.16
rs10497039	26122	EPC2	2	149257379	8.68E-02	6833	4.48E-01	Down	0.76	10334	1.06	0.03
rs7160965	11154	AP4S1	14	30576256	8.68E-02	6834	3.33E-04	Down	3.59	2814	1.06	0.35
rs2294630	4681	NBL1	1	19727097	8.69E-02	6835	4.20E-30	Down	11.40	62	1.06	2.94
rs2294630	3362	HTR6	1	19727097	8.69E-02	6836	8.56E-01	Up	0.18	12988	1.06	0.01
rs2709501	29969	MDFIC	7	114242204	8.69E-02	6837	3.81E-08	Down	5.50	1195	1.06	0.74
rs5743899	54472	TOLLIP	11	1280140	8.69E-02	6838	5.67E-01	Down	0.57	11220	1.06	0.02
rs12325560	123879	DCUN1D3	16	20816905	8.70E-02	6839	3.32E-02	Down	2.13	5570	1.06	0.15
rs3768991	6160	RPL31	2	101058285	8.70E-02	6840	1.01E-15	Up	7.93	372	1.06	1.50
rs10783418	7024	TFCP2	12	49866486	8.71E-02	6841	5.44E-07	Up	5.01	1490	1.06	0.63
rs10783418	5463	POU6F1	12	49866486	8.71E-02	6842	9.30E-01	Up	0.09	13435	1.06	0.00
rs2241235	57659	ZBTB4	17	7322090	8.71E-02	6843	1.06E-30	Down	11.52	60	1.06	3.00
rs2241235	5430	POLR2A	17	7322090	8.71E-02	6844	1.29E-01	Up	1.52	7436	1.06	0.09
rs10924910	79862	ZNF669	1	243587543	8.72E-02	6845	6.74E-01	Down	0.42	11913	1.06	0.02
rs12419699	6749	SSRP1	11	56867627	8.72E-02	6846	7.16E-01	Up	0.36	12168	1.06	0.01
rs12098747	51182	HSPA14	10	14932416	8.72E-02	6847	1.46E-02	Up	2.44	4792	1.06	0.18
rs12825376	35	ACADS	12	119642659	8.73E-02	6848	1.37E-01	Down	1.49	7533	1.06	0.09
rs17227996	54893	MTMR10	15	29082443	8.73E-02	6849	2.28E-06	Down	4.73	1687	1.06	0.56
rs26020	90441	ZNF622	5	16508266	8.74E-02	6850	2.18E-01	Up	1.23	8439	1.06	0.07
rs4803342	645	BLVRB	19	45674215	8.74E-02	6851	4.85E-01	Down	0.70	10607	1.06	0.03
rs192655	23195	MDN1	6	90574999	8.74E-02	6852	7.54E-02	Up	1.78	6535	1.06	0.11
rs10064968	79616	CCNJL	5	159626420	8.74E-02	6853	1.70E-02	Down	2.39	4935	1.06	0.18
rs2579643	6344	SCTR	2	119933686	8.75E-02	6854	5.90E-01	Down	0.54	11371	1.06	0.02
rs2079626	7566	ZNF18	17	11824053	8.75E-02	6855	9.01E-03	Up	2.61	4436	1.06	0.20
rs8190612	2572	GAD2	10	26552381	8.75E-02	6856	1.92E-02	Up	2.34	5037	1.06	0.17
rs16859774	1660	DHX9	1	179564454	8.75E-02	6857	6.09E-01	Down	0.51	11494	1.06	0.02
rs11984293	54205	CYCS	7	24955645	8.77E-02	6858	1.29E-04	Up	3.83	2511	1.06	0.39
rs11984293	136895	C7orf31	7	24955645	8.77E-02	6859	3.82E-02	Down	2.07	5710	1.06	0.14
rs11877146	29919	C18orf8	18	19322116	8.77E-02	6860	2.98E-02	Down	2.17	5456	1.06	0.15
rs11877146	8780	RIOK3	18	19322116	8.77E-02	6861	1.82E-01	Down	1.33	8048	1.06	0.07
rs1414272	965	CD58	1	116827910	8.78E-02	6862	7.98E-07	Up	4.94	1545	1.06	0.61
rs1414272	3321	IGSF3	1	116827910	8.78E-02	6863	5.57E-01	Up	0.59	11131	1.06	0.03
rs2227928	545	ATR	3	143764310	8.78E-02	6864	4.47E-10	Up	6.43	770	1.06	0.93
rs198775	58526	MID1IP1		38401754	8.78E-02	6865	6.65E-01	Up	0.43	11854	1.06	0.02
rs8141597	4357	MPST	22	35749348	8.78E-02	6866	1.39E-05	Up	4.34	2025	1.06	0.49
rs8141597	7263	TST	22	35749348	8.78E-02	6867	9.71E-01	Down	0.04	13708	1.06	0.00
rs10785439	80070	ADAMTS20	12	42202884	8.78E-02	6868	7.02E-01	Up	0.38	12085	1.06	0.02
rs2035376	9242	MSC	8	72923744	8.79E-02	6869	9.75E-01	Down	0.03	13728	1.06	0.00
rs7209428	125058	TBC1D16	17	75585536	8.80E-02	6870	2.65E-04	Up	3.65	2735	1.06	0.36
rs5748952	51816	CECR1	22	16072335	8.80E-02	6871	2.39E-03	Down	3.04	3659	1.06	0.26
rs3760780	199692	ZNF627	19	11550158	8.81E-02	6872	2.39E-03	Up	3.04	3658	1.06	0.26
rs3760780	54	ACP5	19	11550158	8.81E-02	6873	1.53E-01	Down	1.43	7712	1.06	0.08
rs5951273	54470	ARMCX6		100667824	8.81E-02	6874	2.30E-01	Up	1.20	8567	1.06	0.06
rs5951273	51566	ARMCX3		100667824	8.81E-02	6875	2.90E-01	Down	1.06	9117	1.06	0.05
rs2210914	115352	FCRL3	1	154486701	8.81E-02	6876	2.80E-01	Down	1.08	9035	1.05	0.06
rs7417	9545	RAB3D	19	11293950	8.81E-02	6877	5.04E-04	Up	3.48	2964	1.05	0.33
rs7417	26526	TSPAN16	19	11293950	8.81E-02	6878	4.17E-03	Down	2.87	3960	1.05	0.24
rs3764895	5002	SLC22A18	11	2902521	8.81E-02	6879	5.83E-03	Down	2.76	4161	1.05	0.22
rs3808522	4017	LOXL2	8	23218121	8.81E-02	6880	2.59E-01	Down	1.13	8852	1.05	0.06

rs8058835	57610	RANBP10	16	66372366	8.82E-02	6881	4.54E-07	Up	5.04	1467	1.05	0.63
rs254262	4696	NDUFA3	19	59304440	8.82E-02	6882	2.44E-03	Up	3.03	3668	1.05	0.26
rs254262	126014	OSCAR	19	59304440	8.82E-02	6883	5.04E-01	Down	0.67	10746	1.05	0.03
rs17109031	23583	SMUG1	12	52884634	8.82E-02	6884	7.38E-04	Up	3.37	3096	1.05	0.31
rs3746721	10326	SIRPB1	20	1558894	8.83E-02	6885	3.96E-04	Up	3.54	2880	1.05	0.34
rs3746721	284759	SIRPB2	20	1558894	8.83E-02	6886	7.91E-01	Down	0.27	12617	1.05	0.01
rs2267738	117	ADCYAP1R1	7	30910064	8.83E-02	6887	8.43E-02	Down	1.73	6706	1.05	0.11
rs17264366	7707	ZNF148	3	126462475	8.83E-02	6888	9.01E-01	Up	0.12	13248	1.05	0.00
rs7181518	9325	TRIP4	15	62498227	8.84E-02	6889	9.12E-01	Down	0.11	13326	1.05	0.00
rs6132784	57136	C20orf3	20	24935787	8.84E-02	6890	1.08E-08	Up	5.72	1064	1.05	0.80
rs4845779	54544	CRCT1	1	149292249	8.84E-02	6891	1.71E-01	Down	1.37	7923	1.05	0.08
rs11732095	132612	ADAD1	4	123705950	8.84E-02	6892	9.86E-01	Down	0.02	13818	1.05	0.00
rs17644018	3346	HTN1	4	71113065	8.84E-02	6893	7.83E-01	Up	0.28	12561	1.05	0.01
rs218979	4697	NDUFA4	7	10748251	8.84E-02	6894	4.65E-02	Down	1.99	5911	1.05	0.13
rs913407	375748	C9orf102	9	95850483	8.84E-02	6895	8.46E-01	Down	0.19	12930	1.05	0.01
rs1364043	3350	HTR1A	5	63286607	8.85E-02	6896	7.29E-01	Up	0.35	12239	1.05	0.01
rs11160812	388021	TMEM179	14	104135502	8.85E-02	6897	9.00E-02	Down	1.70	6810	1.05	0.10
rs1427463	11232	POLG2	17	59923044	8.85E-02	6898	1.96E-03	Up	3.10	3519	1.05	0.27
rs1427463	1655	DDX5	17	59923044	8.85E-02	6899	3.82E-01	Down	0.87	9858	1.05	0.04
rs4928119	55773	TBC1D23	3	101466037	8.85E-02	6900	9.02E-01	Down	0.12	13257	1.05	0.00
rs1401908	10606	PAICS	4	57126091	8.85E-02	6901	6.94E-41	Up	13.23	18	1.05	4.02
rs1401908	5471	PPAT	4	57126091	8.85E-02	6902	3.61E-08	Up	5.51	1190	1.05	0.74
rs1401908	80347	COASY	4	57126091	8.85E-02	6903	3.05E-02	Up	2.16	5477	1.05	0.15
rs12810608	9618	TRAF4	12	84177175	8.86E-02	6904	6.57E-11	Up	6.53	733	1.05	1.02
rs10512016	301	ANXA1	9	73022800	8.86E-02	6905	1.29E-16	Down	8.27	326	1.05	1.59
rs9635539	1013	CDH15	16	87778383	8.86E-02	6906	2.99E-02	Down	2.17	5457	1.05	0.15
rs8070091	10594	PRPF8	17	1524782	8.86E-02	6907	2.56E-03	Down	3.02	3700	1.05	0.26
rs765746	90075	ZNF30	19	40126909	8.87E-02	6908	1.25E-03	Up	3.23	3312	1.05	0.29
rs1800566	1728	NQO1	16	68302646	8.87E-02	6909	4.59E-01	Down	0.74	10423	1.05	0.03
rs6005843	11200	CHEK2	22	27432998	8.87E-02	6910	8.55E-01	Up	0.18	12985	1.05	0.01
rs17753508	2530	FUT8	14	65127205	8.88E-02	6911	3.18E-01	Up	1.00	9348	1.05	0.05
rs2983716	11004	KIF2C	1	44855338	8.88E-02	6912	6.10E-04	Up	3.43	3032	1.05	0.32
rs79008	10766	TOB2	22	40175000	8.88E-02	6913	1.04E-02	Up	2.56	4541	1.05	0.20
rs79008	84844	PHF5A	22	40175000	8.88E-02	6914	1.61E-02	Down	2.41	4893	1.05	0.18
rs1144961	1368	CPM	12	67595644	8.89E-02	6915	1.22E-12	Down	7.10	561	1.05	1.19
rs17775212	148103	ZNF599	19	39967308	8.89E-02	6916	8.51E-04	Up	3.34	3154	1.05	0.31
rs2136908	26496	OR10A3	11	7915059	8.89E-02	6917	8.63E-01	Up	0.17	13028	1.05	0.01
rs6510922	10045	SH2D3A	19	6736141	8.89E-02	6918	2.50E-01	Down	1.15	8766	1.05	0.06
rs961358	23321	TRIM2	4	154598081	8.90E-02	6919	2.76E-11	Down	6.66	685	1.05	1.06
rs961358	303	ANXA2P1	4	154598081	8.90E-02	6920	1.65E-07	Down	5.24	1352	1.05	0.68
rs672822	2941	GSTA4	6	52952144	8.90E-02	6921	7.33E-05	Down	3.97	2368	1.05	0.41
rs2111815	54927	CHCHD3	7	132156501	8.90E-02	6922	5.76E-03	Up	2.76	4157	1.05	0.22
rs4810479	5360	PLTP	20	43978455	8.91E-02	6923	8.78E-05	Down	3.92	2419	1.05	0.41
rs10752567	2249	FGF4	11	69313708	8.91E-02	6924	9.90E-02	Down	1.65	6980	1.05	0.10
rs3950680	5286	PIK3C2A	11	17095242	8.91E-02	6925	2.74E-01	Up	1.09	8988	1.05	0.06
rs33660	2935	GSPT1	16	11869359	8.92E-02	6926	4.70E-07	Up	5.04	1473	1.05	0.63
rs33660	26156	RSL1D1	16	11869359	8.92E-02	6927	2.63E-06	Up	4.70	1711	1.05	0.56
rs1688114	7089	TLE2	19	2988787	8.92E-02	6928	3.93E-18	Down	8.68	267	1.05	1.74
rs7102217	219902	TMEM136	11	119692643	8.93E-02	6929	7.00E-02	Down	1.81	6425	1.05	0.12
rs17047144	55252	ASXL2	2	25929757	8.93E-02	6930	8.23E-01	Down	0.22	12805	1.05	0.01
rs1506977	8590	OR6A2	11	6771871	8.93E-02	6931	1.34E-01	Up	1.50	7502	1.05	0.09
rs1654774	7745	ZNF192	6	28204259	8.93E-02	6932	8.26E-01	Up	0.22	12829	1.05	0.01
rs217520	7101	NR2E1	6	108600042	8.93E-02	6933	9.17E-01	Down	0.10	13360	1.05	0.00
rs9656982	157724	SLC7A13	8	87295816	8.93E-02	6934	6.23E-01	Up	0.49	11605	1.05	0.02
rs17198191	11014	KDELR2	7	6289677	8.93E-02	6935	5.59E-11	Up	6.55	723	1.05	1.03
rs7256146	58506	SCAF1	19	54820355	8.93E-02	6936	1.57E-01	Down	1.41	7771	1.05	0.08
rs2591933	57486	NLN	5	65126301	8.93E-02	6937	4.73E-05	Up	4.07	2268	1.05	0.43
rs558660	4282	MIF	11	59360470	8.94E-02	6938	1.13E-04	Up	3.86	2482	1.05	0.39
rs558660	4504	MT3	11	59360470	8.94E-02	6939	7.62E-02	Down	1.77	6556	1.05	0.11
rs571294	374900	ZNF568	19	42132951	8.94E-02	6940	3.14E-01	Down	1.01	9310	1.05	0.05
rs3750817	2263	FGFR2	10	123322567	8.94E-02	6941	4.55E-44	Down	13.92	12	1.05	4.33
rs773107	5869	RAB5B	12	54655773	8.94E-02	6942	8.17E-02	Up	1.74	6665	1.05	0.11
rs773107	1017	CDK2	12	54655773	8.94E-02	6943	3.76E-01	Down	0.88	9813	1.05	0.04
rs7030401	2889	RAPGEF1	9	131481397	8.94E-02	6944	2.36E-01	Down	1.18	8626	1.05	0.06
rs10488671	1028	CDKN1C	11	2853359	8.94E-02	6945	2.04E-18	Down	8.76	255	1.05	1.77
rs10488671	55539	KCNQ1DN	11	2853359	8.94E-02	6946	7.93E-02	Down	1.75	6620	1.05	0.11
rs9865782	254887	ZDHHC23	3	115135464	8.96E-02	6947	1.50E-09	Up	6.04	920	1.05	0.88
rs9865782	54762	GRAMD1C	3	115135464	8.96E-02	6948	1.88E-01	Up	1.32	8127	1.05	0.07
rs1557492	28778	IGLV6-57	22	20885043	8.96E-02	6949	2.67E-01	Up	1.11	8921	1.05	0.06
rs9783503	11197	WIF1	12	63756699	8.96E-02	6950	8.89E-02	Down	1.70	6784	1.05	0.11
rs2750414	85021	REPS1	6	139269078	8.96E-02	6951	3.76E-06	Up	4.62	1774	1.05	0.54
rs16858720	5437	POLR2H	3	185549317	8.97E-02	6952	7.22E-30	Up	11.34	66	1.05	2.91
rs16858720	131408	FAM131A	3	185549317	8.97E-02	6953	5.18E-03	Down	2.80	4090	1.05	0.23
rs16858720	1181	CLCN2	3	185549317	8.97E-02	6954	7.15E-01	Down	0.36	12163	1.05	0.01
rs897165	169522	KCNV2	9	2700537	8.97E-02	6955	7.49E-01	Up	0.32	12368	1.05	0.01
rs300982	5449	POU1F1	3	87408443	8.99E-02	6956	3.21E-01	Up	0.99	9363	1.05	0.05
rs1867448	255426	RASGEF1C	5	179460247	8.99E-02	6957	3.69E-01	Down	0.90	9760	1.05	0.04
rs3741190	29984	RHOD	11	66573083	8.99E-02	6958	6.75E-01	Down	0.42	11919	1.05	0.02
rs3741190	91683	SYT12	11	66573083	8.99E-02	6959	8.26E-01	Up	0.22	12825	1.05	0.01
rs9814648	51095	TRNT1	3	3130229	9.00E-02	6960	2.05E-08	Up	5.60	1135	1.05	0.77
rs10958006	2171	FABP5	8	82335346	9.00E-02	6961	8.17E-19	Up	8.86	236	1.05	1.81

rs3851050	219348	PLAC9	10	81872350	9.00E-02	6962	6.69E-04	Down	3.40	3063	1.05	0.32
rs6049212	6628	SNRPB	20	2390022	9.00E-02	6963	1.82E-01	Up	1.33	8047	1.05	0.07
rs12836478	50814	NSDHL		151690283	9.00E-02	6964	1.45E-05	Down	4.34	2030	1.05	0.48
rs6521178	9104	RGN		46699792	9.00E-02	6965	1.68E-09	Down	6.03	928	1.05	0.88
rs2058604	81575	APOLD1	12	12841126	9.00E-02	6966	6.96E-01	Down	0.39	12045	1.05	0.02
rs2561183	153562	MARVELD2	5	68769454	9.01E-02	6967	1.70E-18	Up	8.77	252	1.05	1.78
rs2702180	4688	NCF2	1	180249628	9.01E-02	6968	9.18E-03	Up	2.61	4444	1.05	0.20
rs6895327	375444	C5orf34	5	43533662	9.01E-02	6969	1.63E-01	Down	1.40	7846	1.05	0.08
rs7126080	57646	USP28	11	113229883	9.01E-02	6970	2.54E-01	Up	1.14	8800	1.05	0.06
rs3761248	5020	OXT	20	2998393	9.02E-02	6971	1.03E-01	Up	1.63	7041	1.05	0.10
rs454886	324	APC	5	112174016	9.03E-02	6972	3.10E-04	Down	3.61	2787	1.04	0.35
rs4713438	253018	HCG27	6	31254825	9.03E-02	6973	1.90E-01	Up	1.31	8154	1.04	0.07
rs2289486	90381	C15orf42	15	87962359	9.04E-02	6974	7.95E-01	Up	0.26	12641	1.04	0.01
rs5993935	65078	RTN4R	22	18595318	9.04E-02	6975	9.90E-01	Up	0.01	13843	1.04	0.00
rs11223753	112937	GLB1L3	11	133686084	9.04E-02	6976	5.26E-04	Up	3.47	2977	1.04	0.33
rs17183482	2353	FOS	14	74799485	9.04E-02	6977	7.45E-01	Up	0.33	12343	1.04	0.01
rs6018027	6714	SRC	20	35424206	9.04E-02	6978	1.49E-03	Up	3.18	3379	1.04	0.28
rs10253046	313	AOAH	7	36395533	9.04E-02	6979	2.92E-01	Up	1.05	9129	1.04	0.05
rs940311	162681	C18orf54	18	50169447	9.04E-02	6980	1.35E-01	Up	1.49	7513	1.04	0.09
rs1013841	2114	ETS2	21	39095215	9.05E-02	6981	1.39E-17	Down	8.54	285	1.04	1.69
rs2834156	3455	IFNAR2	21	33538027	9.05E-02	6982	2.02E-01	Up	1.28	8279	1.04	0.07
rs665908	63951	DMRTA1	9	22450095	9.05E-02	6983	4.74E-01	Down	0.72	10529	1.04	0.03
rs6588640	5563	PRKAA2	1	56820444	9.05E-02	6984	6.85E-01	Down	0.41	11973	1.04	0.02
rs12453250	124637	CYB5D1	17	7702237	9.06E-02	6985	2.38E-03	Down	3.04	3656	1.04	0.26
rs12453250	84316	LSMD1	17	7702237	9.06E-02	6986	5.54E-02	Up	1.92	6123	1.04	0.13
rs7947391	266743	NPAS4	11	65943458	9.06E-02	6987	3.73E-01	Down	0.89	9792	1.04	0.04
rs17046586	57142	RTN4	2	55143579	9.08E-02	6988	9.58E-18	Down	8.58	278	1.04	1.70
rs735740	6461	SHB	9	37934427	9.08E-02	6989	3.60E-04	Down	3.57	2846	1.04	0.34
rs615098	4314	MMP3	11	102225888	9.08E-02	6990	4.31E-01	Down	0.79	10200	1.04	0.04
rs7096226	84504	NKX6-2	10	134457928	9.08E-02	6991	7.42E-01	Up	0.33	12327	1.04	0.01
rs6518223	23275	POFUT2	21	45538977	9.08E-02	6992	2.76E-02	Up	2.20	5382	1.04	0.16
rs12873870	8803	SUCLA2	13	47443974	9.09E-02	6993	5.00E-05	Down	4.06	2287	1.04	0.43
rs2255323	54329	GPR85	7	112312137	9.09E-02	6994	9.28E-02	Up	1.68	6882	1.04	0.10
rs9376080	10767	HBS1L	6	135357091	9.09E-02	6995	1.43E-04	Down	3.80	2536	1.04	0.38
rs3181360	944	TNFSF8	9	114771112	9.09E-02	6996	2.35E-01	Down	1.19	8620	1.04	0.06
rs3794331	83548	COG3	13	44951545	9.09E-02	6997	9.16E-01	Down	0.11	13353	1.04	0.00
rs4963194	81490	PTDSS2	11	474204	9.10E-02	6998	5.87E-01	Down	0.54	11353	1.04	0.02
rs1130183	3766	KCNJ10	1	156824585	9.10E-02	6999	5.76E-01	Up	0.56	11285	1.04	0.02
rs2863171	56981	PRDM11	11	45207308	9.10E-02	7000	9.20E-02	Down	1.68	6862	1.04	0.10
rs7515284	387338	NSUN4	1	46521439	9.11E-02	7001	6.39E-01	Down	0.47	11694	1.04	0.02
rs11062	114826	SMYD4	17	1629762	9.11E-02	7002	7.97E-02	Down	1.75	6629	1.04	0.11
rs11062	5176	SERPINF1	17	1629762	9.11E-02	7003	1.98E-01	Down	1.29	8237	1.04	0.07
rs3742609	9495	AKAP5	14	64006405	9.11E-02	7004	6.42E-02	Up	1.85	6325	1.04	0.12
rs7257863	29985	SLC39A3	19	2697519	9.12E-02	7005	7.10E-01	Down	0.37	12127	1.04	0.01
rs2172257	84864	MINA	3	99147415	9.12E-02	7006	1.57E-06	Up	4.80	1638	1.04	0.58
rs6754024	5077	PAX3	2	222987529	9.12E-02	7007	1.52E-02	Down	2.43	4818	1.04	0.18
rs6754024	151278	CCDC140	2	222987529	9.12E-02	7008	1.58E-01	Up	1.41	7790	1.04	0.08
rs3786248	9388	LIPG	18	45372217	9.13E-02	7009	2.09E-03	Down	3.08	3558	1.04	0.27
rs7837242	91694	LONRF1	8	12644993	9.13E-02	7010	2.31E-04	Down	3.68	2682	1.04	0.36
rs880295	4185	ADAM11	17	40203620	9.13E-02	7011	3.09E-01	Down	1.02	9261	1.04	0.05
rs5759636	613	BCR	22	21863252	9.13E-02	7012	3.39E-02	Up	2.12	5587	1.04	0.15
rs739461	80714	PBX4	19	19576794	9.13E-02	7013	1.28E-03	Down	3.22	3320	1.04	0.29
rs8111379	23370	ARHGEF18	19	7355882	9.13E-02	7014	3.47E-01	Down	0.94	9571	1.04	0.05
rs1584460	151477	C2orf52	2	232195038	9.14E-02	7015	3.26E-01	Down	0.98	9411	1.04	0.05
rs11667591	7691	ZNF132	19	63629449	9.14E-02	7016	6.73E-02	Up	1.83	6383	1.04	0.12
rs2159272	54716	SLC6A20	3	45804999	9.14E-02	7017	9.59E-01	Down	0.05	13613	1.04	0.00
rs6799350	54800	KLHL24	3	184821798	9.15E-02	7018	6.70E-05	Up	3.99	2352	1.04	0.42
rs10516289	26234	FBXL5	4	15348469	9.15E-02	7019	1.14E-02	Down	2.53	4608	1.04	0.19
rs11079520	3384	ICAM2	17	59433066	9.15E-02	7020	1.35E-03	Down	3.20	3338	1.04	0.29
rs7606498	30845	EHD3	2	31405636	9.16E-02	7021	9.74E-01	Down	0.03	13726	1.04	0.00
rs6599079	130497	OSR1	3	38246885	9.16E-02	7022	1.24E-02	Down	2.50	4654	1.04	0.19
rs541862	717	C2	6	32024930	9.17E-02	7023	9.38E-05	Up	3.91	2435	1.04	0.40
rs522894	79109	MAPKAP1	9	125379159	9.17E-02	7024	1.21E-01	Up	1.55	7323	1.04	0.09
rs575605	2899	GRIK3	1	37106869	9.17E-02	7025	5.16E-02	Up	1.95	6026	1.04	0.13
rs385306	58530	LY6G6D	6	31789139	9.18E-02	7026	7.18E-03	Down	2.69	4273	1.04	0.21
rs385306	80740	LY6G6C	6	31789139	9.18E-02	7027	1.01E-02	Down	2.57	4516	1.04	0.20
rs385306	7920	BAT5	6	31789139	9.18E-02	7028	2.63E-01	Down	1.12	8895	1.04	0.06
rs385306	79136	LY6G6E	6	31789139	9.18E-02	7029	4.37E-01	Down	0.78	10254	1.04	0.04
rs385306	80739	C6orf25	6	31789139	9.18E-02	7030	7.01E-01	Down	0.38	12080	1.04	0.02
rs12076073	57326	PBXIP1	1	151757229	9.19E-02	7031	1.88E-04	Down	3.73	2604	1.04	0.37
rs12076073	6464	SHC1	1	151757229	9.19E-02	7032	1.40E-03	Down	3.19	3348	1.04	0.29
rs12076073	90780	PYGO2	1	151757229	9.19E-02	7033	3.35E-01	Down	0.96	9485	1.04	0.05
rs12076073	80308	FLAD1	1	151757229	9.19E-02	7034	7.54E-01	Down	0.31	12400	1.04	0.01
rs12076073	1163	CKS1B	1	151757229	9.19E-02	7035	8.13E-01	Down	0.24	12738	1.04	0.01
rs941406	115196	ZNF554	19	2754623	9.20E-02	7036	7.73E-02	Down	1.77	6582	1.04	0.11
rs2277955	2172	FABP6	5	159598077	9.20E-02	7037	9.10E-01	Down	0.11	13314	1.04	0.00
rs11623700	384	ARG2	14	67195269	9.20E-02	7038	4.06E-03	Down	2.87	3942	1.04	0.24
rs12050393	10490	VTI1B	14	67200289	9.20E-02	7039	6.40E-02	Down	1.85	6324	1.04	0.12
rs12601370	124538	OR4D2	17	53608599	9.20E-02	7040	7.25E-01	Up	0.35	12231	1.04	0.01
rs2968557	90956	ADCK2	7	139827233	9.21E-02	7041	3.32E-01	Up	0.97	9462	1.04	0.05
rs2039081	145389	SLC38A6	14	60575807	9.21E-02	7042	9.94E-03	Up	2.58	4506	1.04	0.20

rs11093451	6567	SLC16A2		73439436	9.21E-02	7043	1.85E-03	Down	3.11	3495	1.04	0.27
rs3817269	23207	PLEKHM2	1	15801670	9.21E-02	7044	4.78E-02	Down	1.98	5946	1.04	0.13
rs562509	259286	TAS2R40	7	142422842	9.21E-02	7045	7.85E-01	Up	0.27	12581	1.04	0.01
rs7357894	4068	SH2D1A		123205534	9.22E-02	7046	7.30E-01	Up	0.34	12252	1.04	0.01
rs919260	2741	GLRA1	5	151298117	9.22E-02	7047	1.03E-01	Down	1.63	7038	1.04	0.10
rs7537265	11189	TNRC4	1	148499313	9.23E-02	7048	1.08E-01	Up	1.61	7125	1.03	0.10
rs2054717	4643	MYO1E	15	57394540	9.23E-02	7049	3.17E-01	Up	1.00	9336	1.03	0.05
rs10956390	5820	PVT1	8	128878021	9.24E-02	7050	8.27E-12	Up	6.83	636	1.03	1.11
rs11667516	339122	RAB43	19	8382316	9.24E-02	7051	6.57E-02	Up	1.84	6357	1.03	0.12
rs11667516	9230	RAB11B	19	8382316	9.24E-02	7052	8.32E-02	Down	1.73	6689	1.03	0.11
rs10064104	56951	C5orf15	5	133315363	9.24E-02	7053	9.44E-05	Up	3.90	2438	1.03	0.40
rs7924071	23268	DNMBP	10	101737154	9.25E-02	7054	3.39E-04	Up	3.58	2820	1.03	0.35
rs8061286	28987	NOB1	16	68323792	9.25E-02	7055	3.64E-04	Up	3.56	2849	1.03	0.34
rs618746	23637	RABGAP1	9	122817077	9.26E-02	7056	7.96E-08	Down	5.37	1273	1.03	0.71
rs16826649	54802	TRIT1	1	40016089	9.26E-02	7057	3.19E-01	Up	1.00	9353	1.03	0.05
rs337484	2188	FANCF	11	22613907	9.26E-02	7058	6.11E-05	Up	4.01	2333	1.03	0.42
rs4526506	6103	RPGR		37954764	9.27E-02	7059	3.83E-01	Up	0.87	9862	1.03	0.04
rs4526506	5009	OTC		37954764	9.27E-02	7060	5.95E-01	Down	0.53	11403	1.03	0.02
rs12060264	4548	MTR	1	233314404	9.28E-02	7061	7.61E-01	Up	0.30	12443	1.03	0.01
rs3026867	1889	ECE1	1	21343466	9.28E-02	7062	1.05E-04	Up	3.88	2469	1.03	0.40
rs897986	9739	SETD1A	16	30888403	9.29E-02	7063	7.01E-04	Up	3.39	3077	1.03	0.32
rs897986	80270	HSD3B7	16	30888403	9.29E-02	7064	1.46E-03	Up	3.18	3368	1.03	0.28
rs4669694	130813	C2orf50	2	11214546	9.29E-02	7065	5.95E-01	Down	0.53	11401	1.03	0.02
rs2221903	59067	IL21	4	123896517	9.29E-02	7066	6.51E-01	Up	0.45	11781	1.03	0.02
rs6464980	155061	ZNF746	7	148639973	9.29E-02	7067	4.84E-04	Up	3.49	2951	1.03	0.33
rs12090453	29899	GPSM2	1	109128267	9.30E-02	7068	5.21E-01	Down	0.64	10867	1.03	0.03
rs12090453	254268	C1orf62	1	109128267	9.30E-02	7069	7.60E-01	Up	0.31	12441	1.03	0.01
rs16989559	56683	C21orf59	21	32896193	9.30E-02	7070	2.37E-02	Up	2.26	5231	1.03	0.16
rs16989559	140290	TCP10L	21	32896193	9.30E-02	7071	8.80E-01	Down	0.15	13127	1.03	0.01
rs7806429	65999	LRRC61	7	149451041	9.31E-02	7072	1.58E-03	Down	3.16	3414	1.03	0.28
rs7806429	113763	C7orf29	7	149451041	9.31E-02	7073	5.80E-01	Down	0.55	11319	1.03	0.02
rs4511593	8742	TNFSF12	17	7396260	9.31E-02	7074	2.82E-02	Down	2.20	5397	1.03	0.16
rs4511593	8741	TNFSF13	17	7396260	9.31E-02	7075	7.38E-02	Down	1.79	6503	1.03	0.11
rs3136614	3601	IL15RA	10	6045680	9.31E-02	7076	3.00E-02	Down	2.17	5465	1.03	0.15
rs17756228	57552	AADACL1	3	173854631	9.32E-02	7077	5.45E-02	Up	1.92	6100	1.03	0.13
rs4667836	2591	GALNT3	2	166452113	9.32E-02	7078	8.18E-24	Up	10.07	126	1.03	2.31
rs2968553	4708	NDUFB2	7	139835340	9.33E-02	7079	1.32E-01	Up	1.51	7461	1.03	0.09
rs3742567	55030	FBXO34	14	54799508	9.33E-02	7080	2.41E-02	Down	2.26	5252	1.03	0.16
rs2231216	1857	DVL3	3	185379343	9.33E-02	7081	7.89E-04	Down	3.36	3129	1.03	0.31
rs2231216	1173	AP2M1	3	185379343	9.33E-02	7082	1.56E-01	Down	1.42	7758	1.03	0.08
rs2231216	55324	ABCF3	3	185379343	9.33E-02	7083	7.52E-01	Down	0.32	12386	1.03	0.01
rs10918607	84944	MAEL	1	163689461	9.33E-02	7084	1.96E-01	Down	1.29	8208	1.03	0.07
rs10425596	6158	RPL28	19	60592911	9.33E-02	7085	5.60E-11	Up	6.55	725	1.03	1.03
rs10425596	3589	IL11	19	60592911	9.33E-02	7086	5.98E-01	Up	0.53	11415	1.03	0.02
rs12403795	54460	MRPS21	1	147091462	9.34E-02	7087	3.02E-04	Up	3.61	2777	1.03	0.35
rs12403795	9129	PRPF3	1	147091462	9.34E-02	7088	1.12E-01	Down	1.59	7181	1.03	0.09
rs2332655	51809	GALNT7	4	174467732	9.34E-02	7089	1.52E-04	Up	3.79	2556	1.03	0.38
rs17483835	23057	NMNAT2	1	180029211	9.34E-02	7090	1.76E-01	Up	1.35	7979	1.03	0.08
rs2027527	64710	NUCKS1	1	202463481	9.35E-02	7091	2.66E-02	Down	2.22	5346	1.03	0.16
rs2027527	8934	RAB7L1	1	202463481	9.35E-02	7092	1.09E-01	Down	1.60	7141	1.03	0.10
rs11070857	23312	DMXL2	15	49644512	9.35E-02	7093	1.88E-03	Up	3.11	3499	1.03	0.27
rs4713340	5514	PPP1R10	6	30707681	9.35E-02	7094	1.89E-03	Up	3.11	3506	1.03	0.27
rs4713340	79969	C6orf134	6	30707681	9.35E-02	7095	1.08E-02	Up	2.55	4565	1.03	0.20
rs4713340	28973	MRPS18B	6	30707681	9.35E-02	7096	3.14E-02	Up	2.15	5511	1.03	0.15
rs4713340	221545	C6orf136	6	30707681	9.35E-02	7097	1.48E-01	Up	1.45	7654	1.03	0.08
rs3739055	51540	SCLY	2	238782182	9.35E-02	7098	3.40E-01	Up	0.95	9519	1.03	0.05
rs3124602	4851	NOTCH1	9	136682328	9.35E-02	7099	1.83E-02	Down	2.36	4998	1.03	0.17
rs13237949	84255	SLC37A3	7	139559688	9.35E-02	7100	8.10E-09	Up	5.76	1042	1.03	0.81
rs443303	1739	DLG1	3	198506435	9.36E-02	7101	1.04E-04	Up	3.88	2462	1.03	0.40
rs1509620	10611	PDLIM5	4	95921150	9.36E-02	7102	1.16E-30	Up	11.56	58	1.03	2.99
rs3746243	126375	ZNF792	19	40140922	9.36E-02	7103	5.72E-06	Up	4.54	1839	1.03	0.52
rs10503716	23039	XPO7	8	21922608	9.36E-02	7104	9.05E-05	Down	3.91	2425	1.03	0.40
rs10503716	10361	NPM2	8	21922608	9.36E-02	7105	3.24E-01	Down	0.99	9397	1.03	0.05
rs1033287	55818	JMJD1A	2	86561405	9.37E-02	7106	7.67E-01	Up	0.30	12471	1.03	0.01
rs10416653	5141	PDE4A	19	10450036	9.37E-02	7107	1.70E-03	Down	3.14	3445	1.03	0.28
rs10416653	9817	KEAP1	19	10450036	9.37E-02	7108	7.17E-01	Up	0.36	12177	1.03	0.01
rs6444147	197	AHSG	3	187799902	9.37E-02	7109	8.15E-02	Down	1.74	6659	1.03	0.11
rs3862907	253152	ABHD7	1	92208676	9.39E-02	7110	2.73E-04	Down	3.64	2747	1.03	0.36
rs130023	1387	CREBBP	16	3773307	9.39E-02	7111	6.25E-03	Down	2.73	4187	1.03	0.22
rs5993516	9993	DGCR2	22	17448166	9.39E-02	7112	1.32E-02	Up	2.48	4699	1.03	0.19
rs216457	6459	SH3GLP2	17	25916969	9.39E-02	7113	5.24E-01	Down	0.64	10894	1.03	0.03
rs1495927	117159	DCD	12	53337030	9.39E-02	7114	1.76E-02	Up	2.37	4962	1.03	0.18
rs1876506	7433	VIPR1	3	42522131	9.40E-02	7115	5.08E-01	Down	0.66	10766	1.03	0.03
rs13279576	27299	ADAMDEC1	8	24277970	9.40E-02	7116	7.70E-01	Up	0.29	12490	1.03	0.01
rs5748239	7353	UFD1L	22	17854249	9.40E-02	7117	4.66E-01	Up	0.73	10474	1.03	0.03
rs5748239	8318	CDC45L	22	17854249	9.40E-02	7118	7.12E-01	Up	0.37	12144	1.03	0.01
rs1383436	54959	ODAM	4	71242602	9.41E-02	7119	2.34E-01	Down	1.19	8599	1.03	0.06
rs9557933	54841	BIVM	13	102263291	9.41E-02	7120	9.48E-06	Up	4.43	1935	1.03	0.50
rs9557933	79070	KDELC1	13	102263291	9.41E-02	7121	1.01E-02	Up	2.57	4515	1.03	0.20
rs1517354	2744	GLS	2	191641372	9.42E-02	7122	1.05E-02	Down	2.56	4550	1.03	0.20
rs7912	25987	TSKU	11	76186491	9.42E-02	7123	2.78E-01	Up	1.08	9018	1.03	0.06

rs11834651	121227	LRIG3	12	57602017	9.42E-02	7124	3.89E-03	Up	2.89	3911	1.03	0.24
rs6429548	6202	RPS8	1	44922721	9.42E-02	7125	2.29E-14	Up	7.66	430	1.03	1.36
rs9944249	7301	TYRO3	15	39634468	9.43E-02	7126	2.59E-04	Down	3.65	2724	1.03	0.36
rs9944249	26015	RPAP1	15	39634468	9.43E-02	7127	9.03E-01	Up	0.12	13262	1.03	0.00
rs2235638	9742	IFT140	16	1513891	9.44E-02	7128	4.02E-02	Up	2.05	5752	1.03	0.14
rs2516808	3834	KIF25	6	168223532	9.44E-02	7129	2.72E-01	Down	1.10	8967	1.03	0.06
rs7794280	29887	SNX10	7	26140233	9.45E-02	7130	3.93E-01	Up	0.85	9936	1.02	0.04
rs3771254	6382	SDC1	2	20327873	9.45E-02	7131	1.11E-01	Down	1.59	7157	1.02	0.10
rs10063817	4869	NPM1	5	170730698	9.46E-02	7132	1.17E-08	Up	5.74	1049	1.02	0.79
rs2746183	4188	MDFI	6	41733407	9.46E-02	7133	4.30E-01	Up	0.79	10196	1.02	0.04
rs10483770	57381	RHOJ	14	62837564	9.46E-02	7134	7.59E-03	Down	2.67	4315	1.02	0.21
rs6702754	89872	AQP10	1	151117049	9.46E-02	7135	1.93E-01	Down	1.30	8179	1.02	0.07
rs6702754	57198	ATP8B2	1	151117049	9.46E-02	7136	1.98E-01	Down	1.29	8232	1.02	0.07
rs7804513	6717	SRI	7	87518231	9.46E-02	7137	2.46E-07	Down	5.16	1395	1.02	0.66
rs31850	56133	PCDHB2	5	140459806	9.47E-02	7138	2.05E-06	Up	4.75	1672	1.02	0.57
rs31850	56132	PCDHB3	5	140459806	9.47E-02	7139	4.64E-01	Up	0.73	10457	1.02	0.03
rs7630033	51122	COMMD2	3	150971007	9.47E-02	7140	7.62E-02	Up	1.77	6552	1.02	0.11
rs11994118	79666	PLEKHF2	8	96233125	9.48E-02	7141	5.76E-01	Up	0.56	11282	1.02	0.02
rs2024566	23264	ZC3H7B	22	40021838	9.48E-02	7142	7.56E-02	Up	1.78	6539	1.02	0.11
rs12461550	339345	NANOS2	19	51106074	9.48E-02	7143	8.26E-01	Up	0.22	12828	1.02	0.01
rs6795737	25819	CCRN4L	3	32980234	9.49E-02	7144	1.05E-01	Down	1.62	7085	1.02	0.10
rs6795737	1233	CCR4	3	32980234	9.49E-02	7145	1.44E-01	Down	1.46	7618	1.02	0.08
rs3799216	670	BPHL	6	3095514	9.49E-02	7146	4.56E-20	Up	9.17	198	1.02	1.93
rs3799216	203068	TUBB	6	3095514	9.49E-02	7147	5.52E-02	Up	1.92	6117	1.02	0.13
rs8089	7058	THBS2	6	169435358	9.50E-02	7148	3.51E-03	Up	2.92	3863	1.02	0.25
rs17742502	79364	ZXDC	3	127698971	9.50E-02	7149	1.14E-02	Up	2.53	4603	1.02	0.19
rs12570214	55526	DHTKD1	10	12205253	9.51E-02	7150	2.35E-06	Up	4.72	1692	1.02	0.56
rs12570214	55176	SEC61A2	10	12205253	9.51E-02	7151	2.66E-01	Up	1.11	8912	1.02	0.06
rs13035955	8324	FZD7	2	202707616	9.51E-02	7152	7.20E-18	Down	8.61	273	1.02	1.71
rs834834	4929	NR4A2	2	157009143	9.51E-02	7153	1.47E-02	Up	2.44	4798	1.02	0.18
rs2267369	8398	PLA2G6	22	36889847	9.52E-02	7154	6.48E-01	Up	0.46	11763	1.02	0.02
rs11076775	197320	ZNF778	16	87815358	9.52E-02	7155	1.81E-01	Down	1.34	8037	1.02	0.07
rs1043627	23005	MAPKBP1	15	39907192	9.52E-02	7156	5.47E-07	Down	5.01	1492	1.02	0.63
rs1043627	100137049	PLA2G4B	15	39907192	9.52E-02	7157	5.48E-04	Down	3.46	2992	1.02	0.33
rs7390098	55064	C9orf68	9	4601901	9.52E-02	7158	5.13E-02	Down	1.95	6018	1.02	0.13
rs3788111	29947	DNMT3L	21	44492599	9.53E-02	7159	1.67E-01	Down	1.38	7885	1.02	0.08
rs6026069	128602	C20orf85	20	56144888	9.54E-02	7160	4.68E-01	Down	0.73	10487	1.02	0.03
rs2159140	6416	MAP2K4	17	11917225	9.54E-02	7161	3.29E-03	Up	2.94	3826	1.02	0.25
rs11673382	90649	ZNF486	19	20153859	9.54E-02	7162	1.83E-02	Down	2.36	4999	1.02	0.17
rs6975771	858	CAV2	7	115716796	9.55E-02	7163	4.83E-53	Down	15.33	5	1.02	5.23
rs7150530	55860	ACTR10	14	57738622	9.56E-02	7164	8.41E-01	Up	0.20	12907	1.02	0.01
rs11758804	6098	ROS1	6	117869923	9.56E-02	7165	2.74E-01	Down	1.09	8987	1.02	0.06
rs12424162	4908	NTF3	12	5475341	9.56E-02	7166	7.78E-04	Down	3.36	3124	1.02	0.31
rs6625359	9754	STARD8		67676387	9.56E-02	7167	1.10E-02	Down	2.54	4574	1.02	0.20
rs4352548	685	BTC	4	76040773	9.56E-02	7168	6.49E-02	Up	1.85	6339	1.02	0.12
rs7950184	6768	ST14	11	129543697	9.57E-02	7169	8.08E-23	Up	9.85	142	1.02	2.21
rs17279897	84925	DIRC2	3	124059778	9.58E-02	7170	2.59E-03	Up	3.01	3707	1.02	0.26
rs7276984	54148	MRPL39	21	25910525	9.58E-02	7171	8.05E-02	Down	1.75	6638	1.02	0.11
rs4691944	51802	ACCN5	4	157110779	9.59E-02	7172	7.66E-01	Down	0.30	12465	1.02	0.01
rs2480	29116	MYLIP	6	16221712	9.59E-02	7173	7.16E-20	Up	9.13	203	1.02	1.91
rs7157525	9252	RPS6KA5	14	90560866	9.59E-02	7174	5.54E-03	Down	2.77	4138	1.02	0.23
rs1549899	153768	PRELID2	5	145133196	9.59E-02	7175	4.95E-01	Down	0.68	10677	1.02	0.03
rs7530308	84919	PPP1R15B	1	201097406	9.59E-02	7176	1.93E-04	Up	3.73	2621	1.02	0.37
rs7453812	26575	RGS17	6	153497313	9.59E-02	7177	1.74E-04	Up	3.75	2582	1.02	0.38
rs6578410	116969	ART5	11	3621003	9.60E-02	7178	3.29E-02	Down	2.13	5557	1.02	0.15
rs6578410	417	ART1	11	3621003	9.60E-02	7179	1.19E-01	Down	1.56	7282	1.02	0.09
rs1890978	8814	CDKL1	14	49914606	9.60E-02	7180	4.92E-02	Down	1.97	5974	1.02	0.13
rs7048	9836	LCMT2	15	41407365	9.60E-02	7181	9.33E-01	Up	0.08	13453	1.02	0.00
rs11577907	83931	STK40	1	36511137	9.60E-02	7182	9.88E-05	Down	3.89	2450	1.02	0.40
rs1018448	26286	ARFGAP3	22	41531448	9.60E-02	7183	9.83E-01	Down	0.02	13795	1.02	0.00
rs17379771	2668	GDNF	5	37849348	9.61E-02	7184	2.44E-01	Up	1.17	8701	1.02	0.06
rs9548568	161003	STOML3	13	38433359	9.61E-02	7185	2.73E-02	Up	2.21	5374	1.02	0.16
rs9895649	23461	ABCA5	17	64769828	9.61E-02	7186	3.69E-02	Up	2.09	5675	1.02	0.14
rs6111852	57325	CSRP2BP	20	18063443	9.62E-02	7187	4.92E-01	Up	0.69	10661	1.02	0.03
rs735295	84318	CCDC77	12	417944	9.62E-02	7188	5.78E-02	Up	1.90	6180	1.02	0.12
rs3743115	55897	MESP1	15	88093615	9.63E-02	7189	8.85E-01	Up	0.14	13160	1.02	0.01
rs13234660	4232	MEST	7	129707021	9.63E-02	7190	1.91E-04	Up	3.73	2615	1.02	0.37
rs140684	2558	GABRA5	15	24771099	9.63E-02	7191	3.38E-01	Down	0.96	9509	1.02	0.05
rs4489077	64083	GOLPH3	5	32225097	9.63E-02	7192	1.43E-11	Up	6.76	649	1.02	1.08
rs10411082	93134	ZNF561	19	9602216	9.63E-02	7193	9.94E-01	Up	0.01	13873	1.02	0.00
rs716733	55656	INTS8	8	95958157	9.63E-02	7194	5.64E-09	Up	5.82	1024	1.02	0.82
rs716733	9134	CCNE2	8	95958157	9.63E-02	7195	5.98E-03	Up	2.75	4171	1.02	0.22
rs481845	8772	FADD	11	69711231	9.64E-02	7196	1.22E-01	Up	1.55	7334	1.02	0.09
rs7306862	57103	C12orf5	12	4316338	9.64E-02	7197	7.85E-01	Down	0.27	12576	1.02	0.01
rs5750678	25777	UNC84B	22	37484617	9.64E-02	7198	2.80E-01	Up	1.08	9033	1.02	0.06
rs3758893	7007	TECTA	11	120480600	9.65E-02	7199	1.90E-01	Down	1.31	8151	1.02	0.07
rs13253966	138050	HGSNAT	8	43177542	9.65E-02	7200	7.17E-01	Down	0.36	12180	1.02	0.01
rs6662930	6900	CNTN2	1	201773122	9.65E-02	7201	6.55E-01	Down	0.45	11801	1.02	0.02
rs2381117	84688	C9orf24	9	34372175	9.65E-02	7202	1.55E-03	Down	3.17	3398	1.02	0.28
rs2381117	57462	KIAA1161	9	34372175	9.65E-02	7203	9.68E-01	Down	0.04	13686	1.02	0.00
rs6556157	4488	MSX2	5	174068278	9.65E-02	7204	7.91E-03	Down	2.66	4333	1.02	0.21

rs2031993	540	ATP7B	13	51404049	9.65E-02	7205	2.45E-23	Up	9.95	134	1.02	2.26
rs662474	4245	MGAT1	5	180175415	9.66E-02	7206	7.82E-02	Down	1.76	6599	1.02	0.11
rs2522391	10111	RAD50	5	131968240	9.66E-02	7207	4.23E-01	Down	0.80	10142	1.02	0.04
rs2292645	9489	PGS1	17	73905790	9.66E-02	7208	4.41E-02	Down	2.01	5854	1.02	0.14
rs10491431	133688	UGT3A1	5	36003757	9.66E-02	7209	2.82E-01	Up	1.08	9046	1.01	0.06
rs13132047	94031	HTRA3	4	8409989	9.66E-02	7210	7.29E-01	Up	0.35	12247	1.01	0.01
rs1729089	80210	ARMC9	2	232031530	9.66E-02	7211	4.32E-02	Up	2.02	5831	1.01	0.14
rs2352262	30061	SLC40A1	2	190249291	9.66E-02	7212	3.15E-06	Down	4.66	1748	1.01	0.55
rs2228561	1294	COL7A1	3	48603018	9.67E-02	7213	1.16E-06	Down	4.86	1595	1.01	0.59
rs2228561	7384	UQCRC1	3	48603018	9.67E-02	7214	8.50E-01	Up	0.19	12953	1.01	0.01
rs4499035	1656	DDX6	11	118115673	9.67E-02	7215	8.05E-03	Up	2.65	4346	1.01	0.21
rs8054172	6693	SPN	16	29563365	9.67E-02	7216	8.23E-01	Down	0.22	12809	1.01	0.01
rs6951867	26157	GIMAP2	7	149834765	9.68E-02	7217	4.59E-01	Up	0.74	10417	1.01	0.03
rs6667629	1676	DFFA	1	10458733	9.68E-02	7218	2.72E-01	Up	1.10	8972	1.01	0.06
rs3105782	5648	MASP1	3	188453998	9.68E-02	7219	1.81E-08	Down	5.63	1114	1.01	0.77
rs17143021	113263	GLCCI1	7	7906669	9.70E-02	7220	7.18E-01	Up	0.36	12189	1.01	0.01
rs7247125	64130	LIN7B	19	54315747	9.70E-02	7221	3.76E-02	Down	2.08	5697	1.01	0.14
rs10800783	23612	PHLDA3	1	198183509	9.70E-02	7222	4.74E-03	Down	2.82	4024	1.01	0.23
rs11236275	2017	CTTN	11	69965089	9.70E-02	7223	1.53E-02	Up	2.43	4826	1.01	0.18
rs10403030	26145	IRF2BP1	19	51087818	9.70E-02	7224	3.30E-01	Down	0.98	9437	1.01	0.05
rs10808507	7373	COL14A1	8	121235610	9.71E-02	7225	3.47E-11	Down	6.63	696	1.01	1.05
rs2766671	7764	ZNF217	20	51617909	9.71E-02	7226	2.14E-27	Up	10.82	83	1.01	2.67
rs1196883	23325	KIAA1033	12	103990608	9.71E-02	7227	8.05E-01	Down	0.25	12702	1.01	0.01
rs1003991	23492	CBX7	22	37890396	9.71E-02	7228	4.37E-05	Down	4.09	2247	1.01	0.44
rs6706925	6509	SLC1A4	2	65111711	9.72E-02	7229	2.29E-04	Up	3.69	2677	1.01	0.36
rs9883607	57129	MRPL47	3	180813394	9.73E-02	7230	8.96E-03	Up	2.61	4434	1.01	0.20
rs2900180	7185	TRAF1	9	120785936	9.73E-02	7231	5.78E-02	Up	1.90	6184	1.01	0.12
rs11517377	79723	SUV39H2	10	14995184	9.73E-02	7232	1.44E-01	Up	1.46	7620	1.01	0.08
rs1182414	9690	UBE3C	7	156557839	9.74E-02	7233	1.57E-01	Up	1.42	7768	1.01	0.08
rs3804778	54931	RG9MTD1	3	102777262	9.74E-02	7234	1.40E-01	Up	1.48	7569	1.01	0.09
rs3198697	123803	NTAN1	16	15037441	9.74E-02	7235	1.51E-06	Down	4.81	1627	1.01	0.58
rs3198697	23042	PDXDC1	16	15037441	9.74E-02	7236	6.39E-04	Up	3.41	3051	1.01	0.32
rs13396021	80303	EFHD1	2	233345884	9.75E-02	7237	1.32E-03	Down	3.21	3331	1.01	0.29
rs4506733	160419	C12orf50	12	86877956	9.75E-02	7238	7.77E-02	Up	1.76	6590	1.01	0.11
rs903756	150677	OTOS	2	240812798	9.75E-02	7239	5.19E-01	Down	0.65	10849	1.01	0.03
rs17108375	118813	ZFYVE27	10	99488113	9.75E-02	7240	9.19E-01	Down	0.10	13372	1.01	0.00
rs13397865	6936	C2orf3	2	75795533	9.75E-02	7241	5.13E-01	Up	0.65	10804	1.01	0.03
rs17451666	11075	STMN2	8	80673099	9.76E-02	7242	6.21E-01	Down	0.49	11590	1.01	0.02
rs896884	80705	TSGA10	2	99110481	9.76E-02	7243	2.09E-03	Up	3.08	3557	1.01	0.27
rs1078363	2039	EPB49	8	21960158	9.76E-02	7244	3.54E-01	Down	0.93	9619	1.01	0.05
rs1078363	8822	FGF17	8	21960158	9.76E-02	7245	3.98E-01	Up	0.85	9971	1.01	0.04
rs2075070	165	AEBP1	7	43928492	9.77E-02	7246	1.46E-05	Down	4.33	2032	1.01	0.48
rs1304403	2167	FABP4	8	82575548	9.77E-02	7247	1.85E-03	Down	3.11	3491	1.01	0.27
rs10103677	51571	FAM49B	8	130967660	9.77E-02	7248	2.12E-01	Up	1.25	8375	1.01	0.07
rs10492943	1677	DFFB	1	3788581	9.77E-02	7249	2.91E-02	Up	2.18	5428	1.01	0.15
rs10492943	9731	KIAA0562	1	3788581	9.77E-02	7250	3.63E-01	Up	0.91	9701	1.01	0.04
rs6777976	92106	OXNAD1	3	16302913	9.77E-02	7251	5.86E-02	Up	1.89	6210	1.01	0.12
rs17340328	142684	RAB40A		102576007	9.77E-02	7252	7.03E-01	Up	0.38	12088	1.01	0.02
rs2412777	116179	TGM7	15	41361016	9.77E-02	7253	3.95E-02	Down	2.06	5735	1.01	0.14
rs17571	1509	CTSD	11	1739170	9.78E-02	7254	9.28E-01	Down	0.09	13420	1.01	0.00
rs869002	222611	GPR111	6	47748056	9.78E-02	7255	5.85E-01	Up	0.55	11342	1.01	0.02
rs869002	221393	GPR115	6	47748056	9.78E-02	7256	7.35E-01	Up	0.34	12286	1.01	0.01
rs16869037	55168	MRPS18A	6	43767478	9.78E-02	7257	1.26E-04	Down	3.83	2504	1.01	0.39
rs10509306	79009	DDX50	10	70315703	9.79E-02	7258	6.25E-02	Up	1.86	6290	1.01	0.12
rs10111621	9583	ENTPD4	8	23366078	9.79E-02	7259	2.07E-01	Up	1.26	8325	1.01	0.07
rs752150	90233	ZNF551	19	62888019	9.79E-02	7260	3.65E-03	Up	2.91	3882	1.01	0.24
rs5978060	56180	MOSPD1		133767852	9.79E-02	7261	7.01E-01	Up	0.38	12081	1.01	0.02
rs2413598	8911	CACNA1I	22	38274672	9.80E-02	7262	1.59E-01	Up	1.41	7801	1.01	0.08
rs7823144	27257	LSM1	8	38147352	9.80E-02	7263	1.42E-03	Down	3.19	3355	1.01	0.28
rs7823144	9530	BAG4	8	38147352	9.80E-02	7264	8.40E-02	Down	1.73	6698	1.01	0.11
rs7823144	6770	STAR	8	38147352	9.80E-02	7265	5.30E-01	Down	0.63	10938	1.01	0.03
rs13236754	57541	ZNF398	7	148283421	9.80E-02	7266	2.54E-02	Down	2.24	5300	1.01	0.16
rs12404187	54823	C1orf26	1	181878931	9.81E-02	7267	1.27E-01	Up	1.53	7409	1.01	0.09
rs1052669	2909	GRLF1	19	52198609	9.81E-02	7268	2.73E-03	Down	3.00	3734	1.01	0.26
rs1052669	4861	NPAS1	19	52198609	9.81E-02	7269	1.98E-02	Down	2.33	5065	1.01	0.17
rs17096208	4329	ALDH6A1	14	73625022	9.82E-02	7270	2.19E-03	Up	3.06	3594	1.01	0.27
rs3094	283	ANG	14	20237895	9.83E-02	7271	1.12E-26	Down	10.69	89	1.01	2.59
rs3094	6038	RNASE4	14	20237895	9.83E-02	7272	5.01E-06	Down	4.56	1820	1.01	0.53
rs603945	192669	EIF2C3	1	36159279	9.83E-02	7273	4.66E-01	Up	0.73	10475	1.01	0.03
rs886936	5802	PTPRS	19	5204432	9.83E-02	7274	4.23E-03	Down	2.86	3969	1.01	0.24
rs4374611	10568	SLC34A2	4	25354928	9.84E-02	7275	5.28E-02	Up	1.94	6062	1.01	0.13
rs1965186	151742	PPM1L	3	162062805	9.84E-02	7276	1.27E-01	Down	1.53	7402	1.01	0.09
rs6994742	137075	CLDN23	8	8577480	9.84E-02	7277	2.22E-02	Up	2.29	5171	1.01	0.17
rs2145078	245936	DEFB123	20	29482203	9.84E-02	7278	2.28E-01	Up	1.20	8541	1.01	0.06
rs7816613	83988	NCALD	8	102798651	9.84E-02	7279	2.57E-06	Up	4.70	1709	1.01	0.56
rs13061203	58477	SRPRB	3	135001833	9.85E-02	7280	1.30E-08	Up	5.69	1077	1.01	0.79
rs1768586	9911	TMCC2	1	201969932	9.85E-02	7281	6.43E-02	Down	1.85	6329	1.01	0.12
rs3786391	2589	GALNT1	18	31531954	9.85E-02	7282	6.27E-20	Up	9.14	200	1.01	1.92
rs10115467	6019	RLN2	9	5301171	9.85E-02	7283	5.32E-02	Up	1.93	6066	1.01	0.13
rs1739828	149563	C1orf64	1	16072728	9.86E-02	7284	1.10E-08	Up	5.71	1068	1.01	0.80
rs5920842	94121	SYTL4		99749248	9.87E-02	7285	1.49E-09	Down	6.05	919	1.01	0.88

rs508398	201625	DNAH12	3	57321642	9.88E-02	7286	2.38E-01	Down	1.18	8637	1.01	0.06
rs708161	55726	C12orf11	12	26984238	9.88E-02	7287	1.84E-01	Up	1.33	8072	1.01	0.07
rs708161	26127	FGFR1OP2	12	26984238	9.88E-02	7288	1.88E-01	Down	1.32	8125	1.01	0.07
rs2302352	4820	NKTR	3	42659137	9.89E-02	7289	1.02E-06	Up	4.89	1576	1.00	0.60
rs2302352	92999	ZBTB47	3	42659137	9.89E-02	7290	8.21E-03	Down	2.64	4360	1.00	0.21
rs9308846	200407	CREG2	2	101456256	9.89E-02	7291	8.09E-02	Up	1.75	6644	1.00	0.11
rs624032	25805	BAMBI	10	28998382	9.89E-02	7292	8.95E-11	Up	6.48	753	1.00	1.00
rs10784938	1272	CNTN1	12	39579125	9.89E-02	7293	1.18E-18	Down	8.82	242	1.00	1.79
rs817355	140700	SAMD10	20	62084782	9.89E-02	7294	2.45E-03	Up	3.03	3672	1.00	0.26
rs847652	10234	LRRC17	7	102153719	9.90E-02	7295	8.13E-01	Down	0.24	12744	1.00	0.01
rs737950	10291	SF3A1	22	29081849	9.90E-02	7296	2.43E-01	Up	1.17	8682	1.00	0.06
rs10934955	30849	PIK3R4	3	131933052	9.90E-02	7297	8.23E-02	Up	1.74	6673	1.00	0.11
rs10797092	81607	PVRL4	1	157850115	9.90E-02	7298	2.03E-01	Down	1.27	8288	1.00	0.07
rs3765945	3283	HSD3B1	1	119763488	9.91E-02	7299	4.73E-01	Down	0.72	10517	1.00	0.03
rs6471254	169200	TMEM64	8	91878147	9.91E-02	7300	1.45E-01	Down	1.46	7623	1.00	0.08
rs17301982	54629	FAM63B	15	56852257	9.91E-02	7301	5.78E-01	Up	0.56	11300	1.00	0.02
rs3027415	4128	MAOA		43370695	9.92E-02	7302	4.40E-07	Up	5.05	1464	1.00	0.64
rs352943	23567	ZNF346	5	176397344	9.92E-02	7303	2.66E-01	Down	1.11	8913	1.00	0.06
rs4149579	55200	PLEKHG6	12	6317618	9.92E-02	7304	3.28E-01	Down	0.98	9423	1.00	0.05
rs790575	694	BTG1	12	91034076	9.93E-02	7305	7.93E-03	Down	2.66	4336	1.00	0.21
rs2410616	4023	LPL	8	19872959	9.93E-02	7306	1.84E-03	Up	3.11	3487	1.00	0.27
rs4766965	84274	COQ5	12	119404785	9.93E-02	7307	2.69E-04	Up	3.64	2741	1.00	0.36
rs2009118	4117	MAK	6	10925491	9.94E-02	7308	1.79E-01	Up	1.34	8010	1.00	0.07
rs3766642	9068	ANGPTL1	1	175545188	9.94E-02	7309	9.57E-10	Down	6.12	891	1.00	0.90
rs12500509	51088	KLHL5	4	38886180	9.94E-02	7310	2.42E-04	Down	3.67	2697	1.00	0.36
rs3744453	124989	C17orf57	17	42737869	9.94E-02	7311	4.80E-01	Up	0.71	10576	1.00	0.03
rs4862848	132625	ZFP42	4	189296589	9.94E-02	7312	8.67E-01	Up	0.17	13050	1.00	0.01
rs4714580	55173	MRPS10	6	42282536	9.94E-02	7313	7.46E-03	Down	2.68	4299	1.00	0.21
rs1078523	84313	VPS25	17	38166286	9.94E-02	7314	4.46E-01	Down	0.76	10321	1.00	0.04
rs4714580	2979	GUCA1B	6	42282536	9.94E-02	7315	7.34E-01	Down	0.34	12282	1.00	0.01
rs296533	55765	C1orf106	1	197597425	9.95E-02	7316	5.24E-09	Down	5.84	1016	1.00	0.83
rs251638	55299	BXDC2	5	34934061	9.95E-02	7317	1.18E-05	Up	4.38	1982	1.00	0.49
rs251638	5810	RAD1	5	34934061	9.95E-02	7318	9.77E-02	Down	1.66	6956	1.00	0.10
rs2474460	163688	CALML6	1	1876208	9.95E-02	7319	1.51E-01	Down	1.44	7689	1.00	0.08
rs1944900	837	CASP4	11	104343681	9.95E-02	7320	5.96E-03	Down	2.75	4166	1.00	0.22
rs13188993	10814	CPLX2	5	175172046	9.95E-02	7321	9.86E-01	Up	0.02	13816	1.00	0.00
rs913930	7099	TLR4	9	117563563	9.95E-02	7322	3.62E-02	Up	2.10	5654	1.00	0.14
rs6050200	8530	CST7	20	24883748	9.96E-02	7323	1.13E-01	Down	1.58	7192	1.00	0.09
rs2852720	9447	AIM2	1	155873633	9.96E-02	7324	9.36E-01	Up	0.08	13465	1.00	0.00
rs12044778	2773	GNAI3	1	109796450	9.97E-02	7325	4.13E-01	Down	0.82	10078	1.00	0.04
rs12044778	83873	GPR61	1	109796450	9.97E-02	7326	7.95E-01	Down	0.26	12642	1.00	0.01
rs1011814	2255	FGF10	5	44371577	9.97E-02	7327	3.30E-02	Down	2.13	5562	1.00	0.15
rs7621788	8801	SUCLG2	3	67710891	9.97E-02	7328	5.55E-01	Up	0.59	11120	1.00	0.03
rs1057888	10868	USP20	9	129729411	9.97E-02	7329	5.16E-01	Up	0.65	10830	1.00	0.03
rs1475853	7152	TOP1P2	22	23470307	9.97E-02	7330	2.98E-03	Up	2.97	3776	1.00	0.25
rs4916567	205564	SENP5	3	198087632	9.97E-02	7331	1.71E-03	Down	3.14	3449	1.00	0.28
rs17104186	8445	DYRK2	12	66320139	9.97E-02	7332	3.25E-02	Up	2.14	5542	1.00	0.15
rs9868848	64432	MRPS25	3	15090876	9.98E-02	7333	1.35E-02	Up	2.47	4725	1.00	0.19
rs9868848	64145	ZFYVE20	3	15090876	9.98E-02	7334	2.20E-02	Down	2.29	5161	1.00	0.17
rs498221	57222	ERGIC1	5	172247401	9.98E-02	7335	4.30E-13	Up	7.25	522	1.00	1.24
rs4696289	5188	PET112L	4	152978842	9.98E-02	7336	5.79E-01	Down	0.56	11303	1.00	0.02
rs11190074	26507	CNNM1	10	101111708	9.98E-02	7337	3.98E-03	Up	2.88	3927	1.00	0.24
rs1802710	8788	DLK1	14	100270398	9.99E-02	7338	4.89E-01	Down	0.69	10642	1.00	0.03
rs3917368	3553	IL1B	2	113299013	9.99E-02	7339	3.27E-02	Down	2.14	5551	1.00	0.15
rs1140809	8449	DHX16	6	30719655	1.00E-01	7340	1.01E-01	Down	1.64	7007	1.00	0.10
rs9288318	130540	ALS2CR12	2	202021569	1.00E-01	7341	9.30E-01	Down	0.09	13432	1.00	0.00
rs624899	51295	ECSIT	19	11473498	1.00E-01	7342	3.72E-02	Up	2.08	5681	1.00	0.14
rs3600	10059	DNM1L	12	32788211	1.00E-01	7343	2.29E-03	Down	3.05	3624	1.00	0.26
rs4713842	222663	SCUBE3	6	35302610	1.00E-01	7344	7.42E-09	Down	5.78	1037	1.00	0.81
rs2248907	10158	PDZK1IP1	1	47367836	1.00E-01	7345	4.43E-01	Up	0.77	10289	1.00	0.04
rs6826085	55153	SDAD1	4	77227408	1.00E-01	7346	1.74E-03	Up	3.13	3459	1.00	0.28
rs4331742	57563	KLHL8	4	88517431	1.00E-01	7347	1.16E-20	Up	9.33	183	1.00	1.99
rs4129682	7201	TRHR	8	110191315	1.00E-01	7348	6.95E-01	Down	0.39	12036	1.00	0.02
rs10950641	29886	SNX8	7	2107627	1.00E-01	7349	8.25E-01	Up	0.22	12820	1.00	0.01
rs10466783	54477	PLEKHA5	12	19329351	1.00E-01	7350	2.83E-02	Down	2.19	5404	1.00	0.15
rs10506399	9194	SLC16A7	12	58460760	1.00E-01	7351	1.01E-03	Down	3.29	3226	1.00	0.30
rs1600138	55297	CCDC91	12	28397773	1.00E-01	7352	2.67E-02	Up	2.22	5349	1.00	0.16
rs938952	8483	CILP	15	63276181	1.00E-01	7353	1.41E-01	Down	1.47	7587	1.00	0.09
rs7153587	80127	C14orf45	14	73579413	1.01E-01	7354	3.07E-01	Down	1.02	9240	1.00	0.05
rs7542797	54583	EGLN1	1	227860522	1.01E-01	7355	3.48E-01	Up	0.94	9575	1.00	0.05
rs17663872	689	BTF3	5	72827065	1.01E-01	7356	3.36E-03	Up	2.93	3836	1.00	0.25
rs17663872	10384	BTN3A3	5	72827065	1.01E-01	7357	1.39E-01	Down	1.48	7555	1.00	0.09
rs5943373	55916	NXT2		108569121	1.01E-01	7358	9.07E-02	Up	1.69	6841	1.00	0.10
rs7975829	6515	SLC2A3	12	7960148	1.01E-01	7359	6.37E-03	Up	2.73	4199	1.00	0.22
rs4802363	56917	MEIS3	19	52632176	1.01E-01	7360	8.23E-01	Down	0.22	12811	1.00	0.01
rs209994	2000	ELF4		128952988	1.01E-01	7361	5.31E-11	Down	6.56	718	1.00	1.03
rs6599418	353497	POLN	4	2179226	1.01E-01	7362	8.79E-01	Down	0.15	13118	1.00	0.01
rs4923889	79094	CHAC1	15	39022224	1.01E-01	7363	1.20E-01	Down	1.56	7288	1.00	0.09
rs734999	8764	TNFRSF14	1	2545378	1.01E-01	7364	2.09E-04	Up	3.71	2647	1.00	0.37
rs734999	127281	C1orf93	1	2545378	1.01E-01	7365	5.27E-03	Up	2.79	4103	1.00	0.23
rs17358651	23349	KIAA1045	9	34958031	1.01E-01	7366	6.60E-01	Up	0.44	11828	1.00	0.02

rs2213150	28823	IGLV1-44	22	21041998	1.01E-01	7367	3.55E-01	Up	0.93	9637	1.00	0.04
rs2303076	3161	HMMR	5	162820228	1.01E-01	7368	7.20E-08	Up	5.39	1259	1.00	0.71
rs3900770	83416	FCRL5	1	154297771	1.01E-01	7369	1.59E-02	Up	2.41	4875	1.00	0.18
rs2145247	23438	HARS2	20	18628688	1.01E-01	7370	1.07E-01	Down	1.61	7112	1.00	0.10
rs8730	113178	SCAMP4	19	1876942	1.01E-01	7371	8.48E-03	Up	2.63	4382	1.00	0.21
rs2007332	8993	PGLYRP1	19	51230737	1.01E-01	7372	2.94E-02	Down	2.18	5441	1.00	0.15
rs12372102	55508	SLC35E3	12	67443967	1.01E-01	7373	2.86E-01	Down	1.07	9078	1.00	0.05
rs10888527	6699	SPRR1B	1	149814436	1.01E-01	7374	2.28E-01	Down	1.21	8540	1.00	0.06
rs11763677	83605	CCM2	7	44866679	1.01E-01	7375	7.84E-01	Up	0.27	12568	1.00	0.01
rs12565770	23065	KIAA0090	1	19300366	1.01E-01	7376	4.98E-03	Up	2.81	4070	1.00	0.23
rs12565770	23352	UBR4	1	19300366	1.01E-01	7377	5.03E-01	Up	0.67	10736	1.00	0.03
rs6528055	51360	MBTPS2		21617248	1.01E-01	7378	3.10E-01	Up	1.02	9265	1.00	0.05
rs2070600	10554	AGPAT1	6	32259421	1.01E-01	7379	1.89E-03	Down	3.11	3504	1.00	0.27
rs2070600	6048	RNF5	6	32259421	1.01E-01	7380	1.85E-01	Up	1.32	8092	1.00	0.07
rs2070600	63940	GPSM3	6	32259421	1.01E-01	7381	6.70E-01	Down	0.43	11884	1.00	0.02
rs2070600	5089	PBX2	6	32259421	1.01E-01	7382	7.94E-01	Down	0.26	12637	1.00	0.01
rs9394248	6631	SNRPC	6	34847853	1.01E-01	7383	6.48E-01	Up	0.46	11762	1.00	0.02
rs1006389	130749	CPO	2	207669218	1.01E-01	7384	1.42E-01	Up	1.47	7599	1.00	0.08
rs7260329	1555	CYP2B6	19	46213478	1.01E-01	7385	2.98E-01	Up	1.04	9180	0.99	0.05
rs11219743	54414	SIAE	11	124027930	1.01E-01	7386	1.21E-01	Up	1.55	7315	0.99	0.09
rs741695	79641	ROGDI	16	4777363	1.01E-01	7387	8.35E-01	Up	0.21	12877	0.99	0.01
rs1609566	3929	LBP	20	36444682	1.01E-01	7388	4.00E-01	Up	0.84	9981	0.99	0.04
rs3767765	8458	TTF2	1	117339522	1.01E-01	7389	4.32E-02	Up	2.02	5830	0.99	0.14
rs12236788	23299	BICD2	9	92622209	1.01E-01	7390	4.07E-07	Down	5.07	1458	0.99	0.64
rs17673031	10973	ASCC3	6	101418991	1.01E-01	7391	3.86E-01	Down	0.87	9889	0.99	0.04
rs12074013	29922	NME7	1	165865279	1.01E-01	7392	8.94E-01	Up	0.13	13210	0.99	0.00
rs887469	1362	CPD	17	25716700	1.01E-01	7393	1.95E-14	Up	7.66	429	0.99	1.37
rs4825239	5256	PHKA2		18752087	1.01E-01	7394	1.01E-08	Up	5.73	1055	0.99	0.80
rs10401807	6511	SLC1A6	19	14932009	1.01E-01	7395	6.86E-01	Down	0.40	11982	0.99	0.02
rs7208561	10743	RAI1	17	17556253	1.01E-01	7396	6.34E-01	Up	0.48	11664	0.99	0.02
rs8	1021	CDK6	7	92052980	1.02E-01	7397	2.84E-07	Up	5.13	1411	0.99	0.65
rs27072	6531	SLC6A3	5	1447522	1.02E-01	7398	5.70E-01	Down	0.57	11247	0.99	0.02
rs9836419	57088	PLSCR4	3	147430947	1.02E-01	7399	1.57E-01	Down	1.42	7760	0.99	0.08
rs4899565	122953	JDP2	14	74981665	1.02E-01	7400	8.65E-02	Down	1.71	6748	0.99	0.11
rs10866851	10687	PNMA2	8	26430215	1.02E-01	7401	2.16E-01	Down	1.24	8420	0.99	0.07
rs7583724	78989	COLEC11	2	3166234	1.02E-01	7402	7.29E-01	Up	0.35	12240	0.99	0.01
rs3821021	54884	RETSAT	2	85508397	1.02E-01	7403	3.99E-03	Down	2.88	3933	0.99	0.24
rs1786224	83608	C18orf21	18	31790143	1.02E-01	7404	1.88E-01	Up	1.32	8123	0.99	0.07
rs3741260	9066	SYT7	11	61039080	1.02E-01	7405	8.72E-02	Up	1.71	6757	0.99	0.11
rs12151222	284443	ZNF493	19	21410878	1.02E-01	7406	1.40E-01	Up	1.47	7578	0.99	0.09
rs10507276	26259	FBXW8	12	115848550	1.02E-01	7407	2.87E-02	Up	2.19	5413	0.99	0.15
rs17784218	2074	ERCC6	10	50435360	1.02E-01	7408	9.23E-01	Down	0.10	13390	0.99	0.00
rs11072220	55323	LARP6	15	68910309	1.02E-01	7409	9.35E-01	Up	0.08	13459	0.99	0.00
rs6769676	9616	RNF7	3	142944545	1.02E-01	7410	5.97E-01	Up	0.53	11411	0.99	0.02
rs7950602	55293	UEVLD	11	18559908	1.02E-01	7411	2.00E-01	Up	1.28	8256	0.99	0.07
rs11227844	2950	GSTP1	11	67116840	1.02E-01	7412	6.24E-36	Down	12.51	35	0.99	3.52
rs11227844	4723	NDUFV1	11	67116840	1.02E-01	7413	4.60E-01	Up	0.74	10425	0.99	0.03
rs4939146	8501	SLC43A1	11	57029188	1.02E-01	7414	7.99E-27	Up	10.76	87	0.99	2.61
rs6539447	6404	SELPLG	12	107511435	1.02E-01	7415	1.49E-01	Down	1.44	7665	0.99	0.08
rs11676727	79954	NOL10	2	10789052	1.02E-01	7416	1.10E-05	Up	4.40	1970	0.99	0.50
rs3760454	55813	UTP6	17	27246115	1.02E-01	7417	6.54E-01	Up	0.45	11793	0.99	0.02
rs11204666	4170	MCL1	1	147356201	1.02E-01	7418	1.38E-09	Up	6.05	915	0.99	0.89
rs7552264	50809	HP1BP3	1	20833233	1.02E-01	7419	5.69E-02	Down	1.90	6158	0.99	0.12
rs11084071	125875	CLDND2	19	56558919	1.02E-01	7420	3.12E-02	Down	2.15	5502	0.99	0.15
rs11084071	2109	ETFB	19	56558919	1.02E-01	7421	1.73E-01	Up	1.36	7940	0.99	0.08
rs11084071	4818	NKG7	19	56558919	1.02E-01	7422	4.20E-01	Down	0.81	10120	0.99	0.04
rs3741007	60494	CCDC81	11	85801005	1.02E-01	7423	4.19E-01	Up	0.81	10112	0.99	0.04
rs12431249	115761	ARL11	13	49112960	1.02E-01	7424	8.84E-01	Up	0.15	13150	0.99	0.01
rs8100491	125919	ZNF543	19	62530962	1.02E-01	7425	8.08E-02	Up	1.75	6642	0.99	0.11
rs1862499	54958	TMEM160	19	52241631	1.02E-01	7426	3.37E-01	Up	0.96	9499	0.99	0.05
rs2329047	1910	EDNRB	13	77399651	1.02E-01	7427	3.18E-13	Down	7.29	512	0.99	1.25
rs965242	51105	PHF20L1	8	133918416	1.02E-01	7428	4.94E-01	Down	0.68	10676	0.99	0.03
rs7344065	140628	GATA5	20	60472738	1.02E-01	7429	2.24E-02	Down	2.28	5176	0.99	0.17
rs157695	6885	MAP3K7	6	91275194	1.02E-01	7430	6.35E-14	Down	7.50	458	0.99	1.32
rs913426	22887	FOXJ3	1	42475284	1.02E-01	7431	6.15E-01	Up	0.50	11543	0.99	0.02
rs3756307	1836	SLC26A2	5	149336679	1.02E-01	7432	3.56E-08	Up	5.51	1186	0.99	0.74
rs2229375	7694	ZNF135	19	63270390	1.02E-01	7433	2.19E-02	Up	2.29	5153	0.99	0.17
rs13107132	80817	KIAA1712	4	175622309	1.02E-01	7434	4.33E-04	Up	3.52	2904	0.99	0.34
rs2075624	3712	IVD	15	38498015	1.02E-01	7435	2.23E-01	Up	1.22	8490	0.99	0.07
rs4808051	79086	C19orf42	19	16617182	1.02E-01	7436	5.30E-01	Up	0.63	10942	0.99	0.03
rs1946518	3606	IL18	11	111540668	1.02E-01	7437	6.83E-01	Up	0.41	11966	0.99	0.02
rs1946518	56158	TEX12	11	111540668	1.02E-01	7438	7.18E-01	Up	0.36	12186	0.99	0.01
rs17740117	54881	TEX10	9	100178672	1.02E-01	7439	9.50E-01	Up	0.06	13551	0.99	0.00
rs9297722	8767	RIPK2	8	90864652	1.02E-01	7440	1.37E-04	Up	3.81	2522	0.99	0.39
rs36664	79165	LENG1	19	59342752	1.02E-01	7441	1.52E-01	Down	1.43	7702	0.99	0.08
rs11756091	89822	KCNK17	6	39390784	1.02E-01	7442	8.04E-01	Down	0.25	12698	0.99	0.01
rs1535923	56897	WRNIP1	6	2701682	1.02E-01	7443	3.52E-01	Up	0.93	9612	0.99	0.05
rs13152048	64116	SLC39A8	4	103633018	1.02E-01	7444	5.24E-04	Up	3.47	2973	0.99	0.33
rs10815149	3717	JAK2	9	5053701	1.02E-01	7445	4.14E-02	Down	2.04	5785	0.99	0.14
rs8055929	80777	CYB5B	16	68058483	1.03E-01	7446	2.77E-12	Down	6.99	601	0.99	1.16
rs12612153	79823	C2orf34	2	44814011	1.03E-01	7447	1.24E-01	Up	1.54	7360	0.99	0.09

rs10279744	53616	ADAM22	7	87315392	1.03E-01	7448	4.50E-01	Up	0.76	10345	0.99	0.03
rs11979330	3792	KEL	7	142153436	1.03E-01	7449	7.37E-02	Up	1.79	6499	0.99	0.11
rs15997	23609	MKRN2	3	12599070	1.03E-01	7450	8.13E-01	Down	0.24	12741	0.99	0.01
rs619864	1326	MAP3K8	10	30790956	1.03E-01	7451	2.15E-03	Down	3.07	3580	0.99	0.27
rs4838910	5016	OVGP1	1	111672025	1.03E-01	7452	4.95E-03	Up	2.81	4061	0.99	0.23
rs130397	53947	A4GALT	22	41417641	1.03E-01	7453	1.64E-01	Down	1.39	7857	0.99	0.08
rs4808652	376497	SLC27A1	19	17456700	1.03E-01	7454	1.54E-01	Down	1.43	7733	0.99	0.08
rs10760260	57684	ZBTB26	9	122793080	1.03E-01	7455	5.84E-01	Down	0.55	11337	0.99	0.02
rs3825389	160762	CCDC63	12	109813491	1.03E-01	7456	3.41E-01	Down	0.95	9528	0.99	0.05
rs3825389	4633	MYL2	12	109813491	1.03E-01	7457	7.33E-01	Up	0.34	12273	0.99	0.01
rs4657207	912	CD1D	1	154947925	1.03E-01	7458	4.24E-01	Down	0.80	10152	0.99	0.04
rs10973593	92014	MCART1	9	37869040	1.03E-01	7459	1.70E-01	Up	1.37	7920	0.99	0.08
rs17080230	5983	RFC3	13	33403786	1.03E-01	7460	2.33E-08	Up	5.59	1140	0.99	0.76
rs1030828	2776	GNAQ	9	77634986	1.03E-01	7461	8.14E-02	Down	1.74	6656	0.99	0.11
rs2571022	84064	HDHD2	18	42923171	1.03E-01	7462	6.79E-01	Down	0.41	11943	0.99	0.02
rs7977617	7296	TXNRD1	12	103250304	1.03E-01	7463	3.12E-02	Down	2.15	5504	0.99	0.15
rs17743530	166785	MMAA	4	146915199	1.03E-01	7464	3.52E-01	Up	0.93	9610	0.99	0.05
rs426434	147172	LRRC37B2	17	25949479	1.03E-01	7465	1.64E-01	Up	1.39	7856	0.99	0.08
rs1889784	2706	GJB2	13	19680232	1.03E-01	7466	1.98E-04	Up	3.72	2626	0.99	0.37
rs4725336	29803	REPIN1	7	149505093	1.03E-01	7467	1.45E-06	Up	4.82	1623	0.99	0.58
rs4725336	285971	ZNF775	7	149505093	1.03E-01	7468	8.12E-03	Down	2.65	4352	0.99	0.21
rs3110788	26266	SLC13A4	7	134821626	1.03E-01	7469	2.36E-01	Down	1.18	8624	0.99	0.06
rs997483	171483	FAM9B		8826585	1.03E-01	7470	5.99E-01	Down	0.53	11424	0.99	0.02
rs9650614	9258	MFHAS1	8	8730796	1.03E-01	7471	4.35E-02	Up	2.02	5840	0.99	0.14
rs3757156	9774	BCLAF1	6	136653804	1.03E-01	7472	2.15E-01	Up	1.24	8404	0.99	0.07
rs12809469	120863	DEPDC4	12	99121558	1.03E-01	7473	2.87E-01	Up	1.06	9092	0.99	0.05
rs12809469	64431	ACTR6	12	99121558	1.03E-01	7474	5.39E-01	Down	0.61	11004	0.99	0.03
rs4982753	51310	SLC22A17	14	22884409	1.03E-01	7475	5.96E-03	Down	2.75	4167	0.99	0.22
rs13337397	9564	BCAR1	16	73853140	1.03E-01	7476	3.43E-02	Down	2.12	5601	0.99	0.15
rs11714	11313	LYPLA2	1	23873773	1.03E-01	7477	1.50E-03	Up	3.17	3384	0.99	0.28
rs11714	3155	HMGCL	1	23873773	1.03E-01	7478	2.64E-02	Down	2.22	5337	0.99	0.16
rs11714	2582	GALE	1	23873773	1.03E-01	7479	3.96E-01	Down	0.85	9962	0.99	0.04
rs11578770	55600	ITLN1	1	157658709	1.03E-01	7480	2.08E-01	Down	1.26	8333	0.99	0.07
rs11578770	51744	CD244	1	157658709	1.03E-01	7481	8.93E-01	Up	0.13	13201	0.99	0.00
rs12372157	10599	SLCO1B1	12	21284285	1.03E-01	7482	8.36E-02	Up	1.73	6694	0.99	0.11
rs3806667	26140	TTLL3	3	9835880	1.03E-01	7483	6.55E-01	Up	0.45	11803	0.99	0.02
rs10497621	129401	NUP35	2	183822949	1.03E-01	7484	1.07E-02	Up	2.55	4555	0.99	0.20
rs689730	2905	GRIN2C	17	70362728	1.04E-01	7485	6.77E-02	Down	1.83	6388	0.98	0.12
rs689730	2232	FDXR	17	70362728	1.04E-01	7486	8.88E-01	Down	0.14	13177	0.98	0.01
rs6940698	6568	SLC17A1	6	25929559	1.04E-01	7487	8.32E-01	Up	0.21	12862	0.98	0.01
rs1889974	60495	HPSE2	10	100258510	1.04E-01	7488	3.22E-01	Down	0.99	9377	0.98	0.05
rs7259256	163081	ZNF567	19	41870187	1.04E-01	7489	9.64E-03	Up	2.59	4481	0.98	0.20
rs8099939	2901	GRIK5	19	47212948	1.04E-01	7490	9.58E-01	Up	0.05	13610	0.98	0.00
rs2249320	127124	ATP6V1G3	1	195216084	1.04E-01	7491	8.47E-02	Down	1.72	6717	0.98	0.11
rs7719726	55789	DEPDC1B	5	59985294	1.04E-01	7492	4.16E-02	Up	2.04	5790	0.98	0.14
rs12461246	11027	LILRA2	19	59773057	1.04E-01	7493	2.48E-01	Down	1.16	8742	0.98	0.06
rs3763045	2528	FUT6	19	5773490	1.04E-01	7494	2.00E-01	Up	1.28	8257	0.98	0.07
rs3763045	4902	NRTN	19	5773490	1.04E-01	7495	2.59E-01	Down	1.13	8851	0.98	0.06
rs2904800	1645	AKR1C1	10	4993148	1.04E-01	7496	3.87E-01	Down	0.86	9895	0.98	0.04
rs9577241	79774	GRTP1	13	113042247	1.04E-01	7497	8.97E-02	Up	1.70	6803	0.98	0.10
rs2292112	93233	CCDC114	19	53491528	1.04E-01	7498	1.32E-01	Down	1.51	7464	0.98	0.09
rs1250090	1487	CTBP1	4	1203527	1.04E-01	7499	8.98E-06	Up	4.44	1925	0.98	0.50
rs2253833	11062	DUS4L	7	106799620	1.04E-01	7500	1.30E-02	Up	2.48	4690	0.98	0.19
rs2253833	55973	BCAP29	7	106799620	1.04E-01	7501	7.66E-01	Down	0.30	12467	0.98	0.01
rs1833288	5874	RAB27B	18	50668904	1.04E-01	7502	3.60E-02	Down	2.10	5647	0.98	0.14
rs16891604	8973	CHRNA6	8	42737870	1.04E-01	7503	7.20E-01	Down	0.36	12198	0.98	0.01
rs246104	9867	PJA2	5	108728881	1.04E-01	7504	6.02E-11	Down	6.54	728	0.98	1.02
rs2235668	50508	NOX3	6	155850723	1.04E-01	7505	7.75E-01	Down	0.29	12528	0.98	0.01
rs6444530	25972	UNC50	3	192465127	1.04E-01	7506	1.27E-01	Up	1.53	7404	0.98	0.09
rs6444530	8233	ZRSR2	3	192465127	1.04E-01	7507	2.27E-01	Down	1.21	8528	0.98	0.06
rs6444530	257313	UTS2D	3	192465127	1.04E-01	7508	4.97E-01	Up	0.68	10694	0.98	0.03
rs6444530	90226	UCN2	3	192465127	1.04E-01	7509	6.19E-01	Down	0.50	11570	0.98	0.02
rs498793	9415	FADS2	11	61381281	1.04E-01	7510	2.03E-13	Down	7.35	494	0.98	1.27
rs7253969	83889	WDR87	19	43069404	1.04E-01	7511	2.67E-01	Down	1.11	8925	0.98	0.06
rs359980	8941	CDK5R2	2	219654711	1.04E-01	7512	2.47E-03	Down	3.03	3676	0.98	0.26
rs11887698	6772	STAT1	2	191680380	1.04E-01	7513	4.83E-01	Up	0.70	10599	0.98	0.03
rs11722977	10815	CPLX1	4	780587	1.04E-01	7514	4.22E-01	Down	0.80	10137	0.98	0.04
rs1057024	3428	IFI16	1	155803320	1.04E-01	7515	4.39E-07	Down	5.05	1465	0.98	0.64
rs7186634	29035	C16orf72	16	9109471	1.04E-01	7516	1.53E-01	Up	1.43	7708	0.98	0.08
rs11767818	7784	ZP3	7	75659232	1.04E-01	7517	6.79E-01	Up	0.41	11937	0.98	0.02
rs11767818	136853	SRCRB4D	7	75659232	1.04E-01	7518	9.77E-01	Up	0.03	13741	0.98	0.00
rs7756421	9532	BAG2	6	57141968	1.04E-01	7519	2.28E-04	Down	3.69	2676	0.98	0.36
rs7756421	26036	ZNF451	6	57141968	1.04E-01	7520	1.01E-02	Up	2.57	4511	0.98	0.20
rs2171605	112464	PRKCDBP	11	6311592	1.04E-01	7521	4.55E-02	Down	2.00	5885	0.98	0.13
rs12106016	140690	CTCFL	20	55538936	1.04E-01	7522	5.23E-01	Down	0.64	10879	0.98	0.03
rs7039375	10655	DMRT2	9	1054985	1.04E-01	7523	3.89E-01	Down	0.86	9908	0.98	0.04
rs164632	5605	MAP2K2	19	4090849	1.04E-01	7524	2.99E-03	Up	2.97	3777	0.98	0.25
rs11870150	3090	HIC1	17	1895351	1.05E-01	7525	1.03E-02	Down	2.57	4533	0.98	0.20
rs12404243	10877	CFHR4	1	193594226	1.05E-01	7526	2.91E-01	Up	1.06	9123	0.98	0.05
rs13002812	678	ZFP36L2	2	43358267	1.05E-01	7527	8.85E-08	Down	5.35	1280	0.98	0.71
rs7260296	10908	PNPLA6	19	7541689	1.05E-01	7528	9.01E-01	Down	0.12	13247	0.98	0.00

rs11730732	9871	SEC24D	4	120119281	1.05E-01	7529	2.09E-08	Up	5.60	1134	0.98	0.77
rs4830340	23096	IQSEC2		53196347	1.05E-01	7530	1.49E-01	Up	1.44	7663	0.98	0.08
rs2843025	11085	ADAM30	1	120140838	1.05E-01	7531	9.61E-01	Up	0.05	13627	0.98	0.00
rs4689064	80273	GRPEL1	4	7173675	1.05E-01	7532	6.30E-03	Up	2.73	4192	0.98	0.22
rs1151503	4296	MAP3K11	11	65114273	1.05E-01	7533	1.31E-04	Up	3.82	2515	0.98	0.39
rs1151503	10089	KCNK7	11	65114273	1.05E-01	7534	1.99E-02	Down	2.33	5068	0.98	0.17
rs1151503	254102	EHBP1L1	11	65114273	1.05E-01	7535	4.02E-02	Down	2.05	5753	0.98	0.14
rs2424994	84557	MAP1LC3A	20	32596578	1.05E-01	7536	5.48E-01	Up	0.60	11068	0.98	0.03
rs10491218	8387	OR1E1	17	3240088	1.05E-01	7537	6.97E-01	Up	0.39	12050	0.98	0.02
rs1042337	3109	HLA-DMB	6	33012959	1.05E-01	7538	7.82E-07	Up	4.94	1538	0.98	0.61
rs9620326	6598	SMARCB1	22	22471183	1.05E-01	7539	5.82E-01	Up	0.55	11327	0.98	0.02
rs3776082	1044	CDX1	5	149524238	1.05E-01	7540	5.89E-01	Up	0.54	11359	0.98	0.02
rs310465	147807	ZNF524	19	60815558	1.05E-01	7541	4.99E-01	Up	0.68	10709	0.98	0.03
rs310465	84922	FIZ1	19	60815558	1.05E-01	7542	9.79E-01	Up	0.03	13769	0.98	0.00
rs3796529	5978	REST	4	57638342	1.05E-01	7543	3.95E-01	Up	0.85	9949	0.98	0.04
rs4726484	5726	TAS2R38	7	141139878	1.05E-01	7544	3.38E-03	Down	2.93	3840	0.98	0.25
rs13298768	27433	TOR2A	9	127569297	1.05E-01	7545	9.18E-01	Down	0.10	13364	0.98	0.00
rs10857437	170371	C10orf128	10	50051927	1.05E-01	7546	5.88E-02	Up	1.89	6214	0.98	0.12
rs4710185	1235	CCR6	6	167492797	1.05E-01	7547	8.15E-01	Up	0.23	12756	0.98	0.01
rs7581935	8886	DDX18	2	118273263	1.05E-01	7548	2.43E-01	Down	1.17	8692	0.98	0.06
rs436321	5122	PCSK1	5	95780239	1.05E-01	7549	4.30E-01	Down	0.79	10195	0.98	0.04
rs2306263	221037	JMJD1C	10	64607421	1.05E-01	7550	2.37E-01	Up	1.18	8635	0.98	0.06
rs2284803	1908	EDN3	20	57325695	1.05E-01	7551	3.80E-07	Down	5.08	1450	0.98	0.64
rs11231671	55611	OTUB1	11	63517745	1.05E-01	7552	1.98E-02	Up	2.33	5060	0.98	0.17
rs11974409	26608	TBL2	7	72434041	1.05E-01	7553	1.47E-13	Up	7.43	475	0.98	1.28
rs11974409	9275	BCL7B	7	72434041	1.05E-01	7554	3.44E-02	Down	2.12	5604	0.98	0.15
rs17614198	55120	FANCL	2	58296478	1.05E-01	7555	2.57E-01	Up	1.13	8835	0.98	0.06
rs7197204	89970	RSPRY1	16	55779366	1.05E-01	7556	3.43E-01	Down	0.95	9546	0.98	0.05
rs7950477	8301	PICALM	11	85473246	1.05E-01	7557	5.59E-13	Up	7.20	533	0.98	1.23
rs10814376	152006	RNF38	9	36333164	1.05E-01	7558	4.15E-03	Down	2.87	3956	0.98	0.24
rs3817859	55732	C1orf112	1	166550723	1.05E-01	7559	1.57E-02	Up	2.42	4859	0.98	0.18
rs4804715	163050	ZNF564	19	12502232	1.05E-01	7560	3.86E-02	Down	2.07	5717	0.98	0.14
rs9284357	433	ASGR2	17	6957913	1.05E-01	7561	5.01E-02	Down	1.96	6002	0.98	0.13
rs1795672	144423	GLT1D1	12	127967659	1.06E-01	7562	9.34E-02	Down	1.68	6892	0.98	0.10
rs1411189	51322	WAC	10	28883484	1.06E-01	7563	5.42E-01	Up	0.61	11038	0.98	0.03
rs16998096	191585	PLAC4	21	41489778	1.06E-01	7564	1.05E-02	Up	2.56	4551	0.98	0.20
rs12603338	3872	KRT17	17	37050079	1.06E-01	7565	7.79E-12	Down	6.84	631	0.98	1.11
rs2247119	51131	PHF11	13	48985143	1.06E-01	7566	1.64E-03	Down	3.15	3428	0.98	0.28
rs284541	6168	RPL37A	2	217194345	1.06E-01	7567	7.93E-05	Up	3.95	2387	0.98	0.41
rs10947788	8645	KCNK5	6	39282590	1.06E-01	7568	4.02E-01	Down	0.84	9999	0.97	0.04
rs6699889	5586	PKN2	1	88881942	1.06E-01	7569	4.22E-01	Up	0.80	10133	0.97	0.04
rs3212310	94120	SYTL3	6	159158958	1.06E-01	7570	4.30E-01	Down	0.79	10197	0.97	0.04
rs8182360	71	ACTG1	17	77086490	1.06E-01	7571	1.20E-03	Up	3.24	3290	0.97	0.29
rs17522524	3707	ITPKB	1	223136725	1.06E-01	7572	2.33E-04	Down	3.68	2685	0.97	0.36
rs7952122	81622	UNC93B1	11	67530473	1.06E-01	7573	3.53E-01	Down	0.93	9614	0.97	0.05
rs7952122	221	ALDH3B1	11	67530473	1.06E-01	7574	8.20E-01	Down	0.23	12788	0.97	0.01
rs2225215	63874	ABHD4	14	22139540	1.06E-01	7575	4.48E-02	Down	2.01	5869	0.97	0.13
rs7305099	378465	HSN2	12	845537	1.06E-01	7576	1.53E-01	Down	1.43	7720	0.97	0.08
rs3790775	8574	AKR7A2	1	19395526	1.06E-01	7577	4.89E-01	Up	0.69	10643	0.97	0.03
rs17677316	10053	AP1M2	19	10541241	1.06E-01	7578	1.17E-05	Up	4.38	1980	0.97	0.49
rs17677316	1032	CDKN2D	19	10541241	1.06E-01	7579	6.12E-02	Down	1.87	6262	0.97	0.12
rs17677316	65095	KRI1	19	10541241	1.06E-01	7580	9.18E-01	Down	0.10	13366	0.97	0.00
rs9400081	51250	C6orf203	6	107477153	1.06E-01	7581	2.89E-01	Up	1.06	9110	0.97	0.05
rs4907646	7027	TFDP1	13	113272497	1.06E-01	7582	2.82E-04	Up	3.63	2758	0.97	0.36
rs4805463	79156	PLEKHF1	19	34828875	1.06E-01	7583	2.41E-01	Up	1.17	8665	0.97	0.06
rs1402012	56670	SUCNR1	3	153072481	1.07E-01	7584	1.91E-01	Up	1.31	8160	0.97	0.07
rs1587265	4258	MGST2	4	140959396	1.07E-01	7585	2.91E-14	Up	7.57	443	0.97	1.35
rs12058214	844	CASQ1	1	156954341	1.07E-01	7586	2.52E-01	Up	1.14	8786	0.97	0.06
rs5925234	1069	CETN2		151687822	1.07E-01	7587	4.79E-05	Down	4.07	2271	0.97	0.43
rs12680842	25962	KIAA1429	8	95651782	1.07E-01	7588	6.38E-01	Up	0.47	11686	0.97	0.02
rs4802576	54795	TRPM4	19	54343602	1.07E-01	7589	1.62E-02	Up	2.41	4896	0.97	0.18
rs4802576	3270	HRC	19	54343602	1.07E-01	7590	8.87E-01	Up	0.14	13172	0.97	0.01
rs1297242	203447	NRK		104898424	1.07E-01	7591	8.33E-02	Down	1.73	6690	0.97	0.11
rs11929195	286676	ILDR1	3	123212589	1.07E-01	7592	3.04E-01	Up	1.03	9221	0.97	0.05
rs3174040	51312	SLC25A37	8	23487352	1.07E-01	7593	7.78E-06	Up	4.47	1894	0.97	0.51
rs2671623	4804	NGFR	17	44911733	1.07E-01	7594	1.31E-03	Down	3.21	3328	0.97	0.29
rs1000940	4927	NUP88	17	5223976	1.07E-01	7595	2.36E-01	Up	1.18	8628	0.97	0.06
rs512071	27074	LAMP3	3	184377038	1.07E-01	7596	9.91E-02	Up	1.65	6982	0.97	0.10
rs2253641	4139	MARK1	1	217160287	1.07E-01	7597	4.06E-01	Up	0.83	10026	0.97	0.04
rs8022285	10668	CGRRF1	14	54061915	1.07E-01	7598	1.82E-08	Down	5.63	1116	0.97	0.77
rs535801	4361	MRE11A	11	93851696	1.07E-01	7599	2.16E-03	Up	3.07	3582	0.97	0.27
rs535801	54851	ANKRD49	11	93851696	1.07E-01	7600	4.87E-01	Down	0.69	10628	0.97	0.03
rs3737483	115265	DDIT4L	4	101472177	1.07E-01	7601	5.65E-01	Up	0.58	11205	0.97	0.02
rs3087687	84698	CAPS2	12	74014152	1.07E-01	7602	1.16E-01	Down	1.57	7232	0.97	0.09
rs3087687	256710	GLIPR1L1	12	74014152	1.07E-01	7603	7.09E-01	Down	0.37	12123	0.97	0.01
rs2071369	1440	CSF3	17	35425831	1.07E-01	7604	6.32E-02	Down	1.86	6304	0.97	0.12
rs4236914	6422	SFRP1	8	41237178	1.07E-01	7605	6.61E-01	Up	0.44	11835	0.97	0.02
rs758692	10157	AASS	7	121301173	1.07E-01	7606	8.22E-01	Down	0.22	12801	0.97	0.01
rs1217566	9480	ONECUT2	18	53240604	1.07E-01	7607	4.42E-05	Up	4.08	2253	0.97	0.44
rs734846	55240	STEAP3	2	119724786	1.07E-01	7608	1.71E-01	Up	1.37	7927	0.97	0.08
rs5747999	150160	CCT8L2	22	15449907	1.07E-01	7609	2.14E-01	Down	1.24	8402	0.97	0.07

rs4703774	4131	MAP1B	5	71473018	1.07E-01	7610	4.46E-20	Down	9.18	196	0.97	1.94
rs1006154	347730	LRRTM1	2	80463122	1.07E-01	7611	3.23E-01	Down	0.99	9387	0.97	0.05
rs5750948	57591	MKL1	22	39211244	1.07E-01	7612	2.79E-01	Down	1.08	9025	0.97	0.06
rs2696247	1277	COL1A1	17	45624902	1.07E-01	7613	8.78E-02	Down	1.71	6768	0.97	0.11
rs17246034	64902	AGXT2	5	35050480	1.07E-01	7614	5.23E-01	Up	0.64	10880	0.97	0.03
rs6964344	54556	ING3	7	120185887	1.07E-01	7615	1.98E-06	Up	4.76	1667	0.97	0.57
rs2943584	3174	HNF4G	8	76603473	1.07E-01	7616	9.11E-04	Up	3.32	3178	0.97	0.30
rs7636635	10188	TNK2	3	197091097	1.07E-01	7617	1.18E-01	Up	1.56	7270	0.97	0.09
rs11084912	83855	KLF16	19	1810390	1.07E-01	7618	3.05E-02	Down	2.16	5478	0.97	0.15
rs6767457	116135	LRRC3B	3	26662121	1.07E-01	7619	2.07E-02	Down	2.31	5101	0.97	0.17
rs716175	8826	IQGAP1	15	88721170	1.07E-01	7620	5.49E-07	Down	5.01	1495	0.97	0.63
rs426132	3594	IL12RB1	19	18067681	1.07E-01	7621	1.07E-01	Down	1.61	7109	0.97	0.10
rs426132	23031	MAST3	19	18067681	1.07E-01	7622	3.40E-01	Down	0.96	9517	0.97	0.05
rs1933107	6846	XCL2	1	165256553	1.07E-01	7623	1.37E-01	Down	1.49	7532	0.97	0.09
rs2057463	11166	SOX21	13	94175128	1.07E-01	7624	5.60E-01	Down	0.58	11162	0.97	0.03
rs6052147	80025	PANK2	20	3816355	1.07E-01	7625	6.09E-02	Up	1.87	6254	0.97	0.12
rs36656	147798	TMC4	19	59356564	1.07E-01	7626	8.16E-07	Up	4.93	1550	0.97	0.61
rs742745	9935	MAFB	20	38741077	1.07E-01	7627	3.86E-01	Up	0.87	9883	0.97	0.04
rs425192	28966	SNX24	5	122287426	1.07E-01	7628	3.00E-09	Down	5.93	968	0.97	0.85
rs10993945	11091	WDR5	9	134020588	1.07E-01	7629	9.92E-01	Up	0.01	13854	0.97	0.00
rs2967465	57711	ZNF529	19	41719057	1.07E-01	7630	5.70E-02	Up	1.90	6159	0.97	0.12
rs2278844	5091	PC	11	66477604	1.07E-01	7631	2.41E-01	Down	1.17	8670	0.97	0.06
rs6843518	64399	HHIP	4	146001091	1.08E-01	7632	1.36E-01	Down	1.49	7521	0.97	0.09
rs1536807	10166	SLC25A15	13	40267790	1.08E-01	7633	1.45E-01	Up	1.46	7629	0.97	0.08
rs13542	85416	ZIC5	13	99436393	1.08E-01	7634	8.57E-01	Up	0.18	12995	0.97	0.01
rs12456583	85019	C18orf45	18	19253694	1.08E-01	7635	4.83E-01	Up	0.70	10595	0.97	0.03
rs6724428	51454	GULP1	2	189203015	1.08E-01	7636	2.62E-01	Up	1.12	8876	0.97	0.06
rs6503653	2588	GALNS	17	37117427	1.08E-01	7637	9.79E-01	Up	0.03	13767	0.97	0.00
rs3794176	4728	NDUFS8	11	67551219	1.08E-01	7638	9.37E-01	Up	0.08	13474	0.97	0.00
rs7587551	84236	RHBDD1	2	227576554	1.08E-01	7639	9.06E-14	Down	7.45	469	0.97	1.30
rs10864625	80835	TAS1R1	1	6548496	1.08E-01	7640	9.12E-01	Down	0.11	13322	0.97	0.00
rs10864625	79707	NOL9	1	6548496	1.08E-01	7641	9.70E-01	Up	0.04	13700	0.97	0.00
rs221954	9619	ABCG1	21	42481032	1.08E-01	7642	1.80E-09	Up	6.01	934	0.97	0.87
rs3751956	84733	CBX2	17	75373079	1.08E-01	7643	5.06E-01	Down	0.67	10759	0.97	0.03
rs4954564	7852	CXCR4	2	136729240	1.08E-01	7644	2.47E-01	Up	1.16	8729	0.97	0.06
rs2187576	3067	HDC	15	48349855	1.08E-01	7645	3.72E-01	Down	0.89	9782	0.97	0.04
rs12420608	10413	YAP1	11	101497559	1.08E-01	7646	6.32E-20	Down	9.14	201	0.97	1.92
rs7793623	340252	ZNF680	7	63415880	1.08E-01	7647	6.91E-09	Up	5.74	1051	0.97	0.82
rs2835559	53820	DSCR6	21	37310681	1.08E-01	7648	5.14E-01	Up	0.65	10812	0.97	0.03
rs819980	83858	ATAD3B	1	1510967	1.08E-01	7649	5.74E-02	Down	1.90	6171	0.97	0.12
rs12345598	51754	C9orf127	9	35845218	1.08E-01	7650	3.14E-01	Down	1.01	9304	0.97	0.05
rs12032282	9783	RIMS3	1	40785354	1.08E-01	7651	5.47E-11	Down	6.56	720	0.97	1.03
rs7614305	5290	PIK3CA	3	180395363	1.08E-01	7652	2.50E-02	Down	2.24	5287	0.97	0.16
rs10513168	58493	C9orf80	9	112556195	1.08E-01	7653	5.83E-05	Up	4.02	2326	0.97	0.42
rs6587341	23456	ABCB10	1	226019075	1.08E-01	7654	6.16E-01	Down	0.50	11548	0.97	0.02
rs1994990	651	BMP3	4	82314103	1.08E-01	7655	2.25E-01	Down	1.21	8515	0.97	0.06
rs4374424	200403	VWA3B	2	98314015	1.08E-01	7656	3.72E-03	Up	2.90	3889	0.97	0.24
rs1055088	330	BIRC3	11	101713061	1.08E-01	7657	1.77E-02	Down	2.37	4968	0.97	0.18
rs1055088	329	BIRC2	11	101713061	1.08E-01	7658	6.07E-01	Down	0.51	11478	0.97	0.02
rs1859493	27072	VPS41	7	38526815	1.08E-01	7659	7.91E-01	Up	0.26	12620	0.97	0.01
rs7710807	729522	AACSL	5	178120567	1.08E-01	7660	3.87E-02	Up	2.07	5720	0.97	0.14
rs12232826	1785	DNM2	19	10717139	1.08E-01	7661	8.72E-01	Up	0.16	13076	0.97	0.01
rs11585	966	CD59	11	33684281	1.08E-01	7662	5.28E-19	Down	8.91	229	0.97	1.83
rs4711741	6722	SRF	6	43252596	1.08E-01	7663	2.92E-06	Down	4.68	1730	0.96	0.55
rs12953405	374860	ANKRD30B	18	14813578	1.08E-01	7664	2.95E-01	Down	1.05	9158	0.96	0.05
rs3735170	5919	RARRES2	7	149471832	1.09E-01	7665	2.42E-13	Down	7.32	500	0.96	1.26
rs4556628	8638	OASL	12	119940632	1.09E-01	7666	7.84E-02	Down	1.76	6603	0.96	0.11
rs17659543	56300	IL1F9	2	113432537	1.09E-01	7667	2.81E-01	Down	1.08	9042	0.96	0.06
rs17620185	27284	SULT1B1	4	70768098	1.09E-01	7668	6.39E-01	Up	0.47	11698	0.96	0.02
rs685487	10588	MTHFS	15	77923184	1.09E-01	7669	3.49E-02	Up	2.11	5621	0.96	0.15
rs2174270	2071	ERCC3	2	127722308	1.09E-01	7670	4.36E-01	Up	0.78	10244	0.96	0.04
rs4649111	6135	RPL11	1	23768438	1.09E-01	7671	-5.08E-10	Up	6.43	773	0.96	#NUM!
rs284227	23266	LPHN2	1	82091467	1.09E-01	7672	4.70E-13	Down	7.23	528	0.96	1.23
rs7147346	6729	SRP54	14	34570453	1.09E-01	7673	2.52E-03	Up	3.02	3693	0.96	0.26
rs6588543	7809	BSND	1	55187987	1.09E-01	7674	3.28E-01	Up	0.98	9424	0.96	0.05
rs12040246	7257	TSNAX	1	228011464	1.09E-01	7675	2.50E-01	Up	1.15	8762	0.96	0.06
rs1758695	4783	NFIL3	9	91245444	1.09E-01	7676	2.67E-05	Down	4.20	2147	0.96	0.46
rs11054767	4040	LRP6	12	12330783	1.09E-01	7677	9.51E-01	Down	0.06	13559	0.96	0.00
rs9535207	81617	CAB39L	13	48830148	1.09E-01	7678	2.20E-01	Up	1.23	8459	0.96	0.07
rs4648870	10250	SRRM1	1	24700798	1.09E-01	7679	9.55E-01	Up	0.06	13588	0.96	0.00
rs12546391	5533	PPP3CC	8	22351173	1.09E-01	7680	3.78E-10	Down	6.26	824	0.96	0.94
rs9861236	51554	CCRL1	3	42855494	1.09E-01	7681	7.97E-01	Down	0.26	12650	0.96	0.01
rs1018199	7294	TXK	4	47927632	1.09E-01	7682	3.38E-02	Up	2.12	5585	0.96	0.15
rs11904625	5757	PTMA	2	232410830	1.09E-01	7683	1.65E-02	Up	2.40	4916	0.96	0.18
rs6691499	90231	KIAA2013	1	11913497	1.09E-01	7684	1.51E-01	Up	1.44	7693	0.96	0.08
rs3821349	23498	HAAO	2	42922390	1.09E-01	7685	1.41E-03	Down	3.19	3354	0.96	0.28
rs3821349	165140	OXER1	2	42922390	1.09E-01	7686	2.82E-02	Up	2.19	5399	0.96	0.15
rs12070002	339500	ZNF678	1	224126225	1.09E-01	7687	1.34E-01	Up	1.50	7504	0.96	0.09
rs566358	7164	TPD52L1	6	125527756	1.09E-01	7688	5.87E-01	Up	0.54	11351	0.96	0.02
rs1266492	4122	MAN2A2	15	89257106	1.09E-01	7689	3.21E-04	Up	3.60	2797	0.96	0.35
rs548927	4992	OR1F1	16	3197869	1.09E-01	7690	1.29E-01	Down	1.52	7432	0.96	0.09

rs10850758	54997	TESC	12	115997427	1.09E-01	7691	2.95E-01	Down	1.05	9157	0.96	0.05
rs2565067	2053	EPHX2	8	27387036	1.09E-01	7692	1.90E-01	Down	1.31	8147	0.96	0.07
rs2565067	1135	CHRNA2	8	27387036	1.09E-01	7693	7.13E-01	Up	0.37	12150	0.96	0.01
rs4696855	8532	CPZ	4	8715211	1.09E-01	7694	7.07E-04	Up	3.39	3080	0.96	0.32
rs10458310	27067	STAU2	8	74629027	1.09E-01	7695	2.75E-05	Up	4.19	2151	0.96	0.46
rs11265618	3570	IL6R	1	151243165	1.09E-01	7696	5.87E-01	Down	0.54	11355	0.96	0.02
rs5750309	5816	PVALB	22	35523522	1.09E-01	7697	4.26E-01	Up	0.80	10166	0.96	0.04
rs3793931	2805	GOT1	10	101140246	1.09E-01	7698	5.65E-01	Up	0.58	11200	0.96	0.02
rs538638	27285	TEKT2	1	36203737	1.09E-01	7699	9.55E-02	Up	1.67	6915	0.96	0.10
rs538638	54936	ADPRHL2	1	36203737	1.09E-01	7700	8.38E-01	Down	0.20	12891	0.96	0.01
rs10876966	4035	LRP1	12	55829839	1.09E-01	7701	6.21E-01	Down	0.49	11593	0.96	0.02
rs10815019	6505	SLC1A1	9	4537288	1.10E-01	7702	6.20E-02	Up	1.87	6280	0.96	0.12
rs16865262	10992	SF3B2	2	198088652	1.10E-01	7703	2.40E-01	Down	1.18	8655	0.96	0.06
rs16865262	23451	SF3B1	2	198088652	1.10E-01	7704	6.70E-01	Up	0.43	11889	0.96	0.02
rs7180455	57369	GJD2	15	32841811	1.10E-01	7705	6.13E-01	Down	0.51	11526	0.96	0.02
rs4759082	121340	SP7	12	52020773	1.10E-01	7706	8.25E-01	Up	0.22	12822	0.96	0.01
rs4759082	8086	AAAS	12	52020773	1.10E-01	7707	9.24E-01	Down	0.09	13397	0.96	0.00
rs1506659	131616	TMEM42	3	44887447	1.10E-01	7708	5.12E-02	Up	1.95	6017	0.96	0.13
rs1007823	54497	HEATR5B	2	37142379	1.10E-01	7709	1.41E-02	Up	2.46	4757	0.96	0.19
rs17669660	7634	ZNF80	3	115450665	1.10E-01	7710	6.97E-03	Up	2.70	4258	0.96	0.22
rs1207739	1749	DLX5	7	96304843	1.10E-01	7711	1.08E-01	Up	1.61	7128	0.96	0.10
rs3176874	7412	VCAM1	1	100911907	1.10E-01	7712	3.73E-02	Down	2.08	5684	0.96	0.14
rs7956133	51699	VPS29	12	109385649	1.10E-01	7713	2.94E-01	Down	1.05	9147	0.96	0.05
rs7956133	29902	C12orf24	12	109385649	1.10E-01	7714	4.22E-01	Up	0.80	10139	0.96	0.04
rs7956133	144715	RAD9B	12	109385649	1.10E-01	7715	6.54E-01	Up	0.45	11788	0.96	0.02
rs10850109	4939	OAS2	12	111876888	1.10E-01	7716	7.85E-16	Up	7.93	373	0.96	1.51
rs10850109	4940	OAS3	12	111876888	1.10E-01	7717	2.25E-01	Up	1.21	8514	0.96	0.06
rs4714664	5754	PTK7	6	43171494	1.10E-01	7718	1.34E-02	Up	2.47	4715	0.96	0.19
rs2086175	9168	TMSB10	2	85051823	1.10E-01	7719	2.61E-01	Up	1.12	8872	0.96	0.06
rs16950217	51224	TCEB3B	18	42830564	1.10E-01	7720	4.68E-01	Down	0.73	10484	0.96	0.03
rs2985828	57708	MIER1	1	67128122	1.10E-01	7721	2.13E-01	Down	1.24	8393	0.96	0.07
rs2305776	79173	C19orf57	19	13890269	1.10E-01	7722	5.47E-01	Down	0.60	11058	0.96	0.03
rs7502835	9772	KIAA0195	17	71010218	1.10E-01	7723	4.66E-02	Up	1.99	5917	0.96	0.13
rs3213894	23107	MRPS27	5	71563866	1.10E-01	7724	7.75E-02	Down	1.77	6587	0.96	0.11
rs6896208	54514	DDX4	5	55123246	1.10E-01	7725	4.09E-01	Down	0.83	10048	0.96	0.04
rs6909995	9521	EEF1E1	6	8058429	1.10E-01	7726	1.42E-12	Up	7.08	566	0.96	1.18
rs524998	2780	GNAT2	1	109881999	1.10E-01	7727	3.11E-02	Down	2.16	5497	0.96	0.15
rs524998	271	AMPD2	1	109881999	1.10E-01	7728	6.12E-01	Down	0.51	11518	0.96	0.02
rs6488285	22914	KLRK1	12	10409973	1.10E-01	7729	7.04E-02	Down	1.81	6436	0.96	0.12
rs223230	2519	FUCA2	6	143859410	1.10E-01	7730	1.04E-03	Up	3.28	3238	0.96	0.30
rs223230	8504	PEX3	6	143859410	1.10E-01	7731	6.50E-03	Down	2.72	4212	0.96	0.22
rs8050463	65988	ZNF747	16	30461660	1.10E-01	7732	1.38E-01	Down	1.48	7553	0.96	0.09
rs10865760	151648	SGOL1	3	20175236	1.11E-01	7733	2.38E-01	Up	1.18	8640	0.96	0.06
rs4648024	4790	NFKB1	4	103854760	1.11E-01	7734	2.08E-12	Down	7.03	586	0.96	1.17
rs2295343	116835	HSPA12B	20	3683601	1.11E-01	7735	2.08E-01	Down	1.26	8334	0.96	0.07
rs4121642	838	CASP5	11	104362276	1.11E-01	7736	2.55E-01	Down	1.14	8812	0.96	0.06
rs12338773	9853	RUSC2	9	35549905	1.11E-01	7737	4.90E-03	Down	2.81	4052	0.96	0.23
rs4521758	5591	PRKDC	8	48904123	1.11E-01	7738	1.25E-03	Up	3.23	3311	0.96	0.29
rs7202826	51451	LCMT1	16	25013707	1.11E-01	7739	4.60E-01	Down	0.74	10430	0.96	0.03
rs2144834	51156	SERPINA10	14	93843387	1.11E-01	7740	2.11E-01	Down	1.25	8362	0.96	0.07
rs7892782	23133	PHF8		53846645	1.11E-01	7741	8.58E-05	Up	3.93	2412	0.96	0.41
rs11465730	8807	IL18RAP	2	102525376	1.11E-01	7742	7.24E-01	Down	0.35	12223	0.96	0.01
rs7632356	2814	GP5	3	195592181	1.11E-01	7743	1.21E-02	Up	2.51	4641	0.96	0.19
rs6539460	1610	DAO	12	107786944	1.11E-01	7744	2.45E-01	Down	1.16	8709	0.96	0.06
rs6956179	8460	TPST1	7	65250809	1.11E-01	7745	1.02E-04	Down	3.89	2459	0.96	0.40
rs4807347	148254	ZNF555	19	2808287	1.11E-01	7746	6.33E-03	Up	2.73	4195	0.96	0.22
rs4807347	80032	ZNF556	19	2808287	1.11E-01	7747	3.42E-01	Up	0.95	9545	0.96	0.05
rs1144116	84873	GPR128	3	101851649	1.11E-01	7748	4.75E-01	Up	0.71	10534	0.95	0.03
rs1789956	7307	U2AF1	21	43392724	1.11E-01	7749	3.95E-01	Down	0.85	9944	0.95	0.04
rs3771891	7130	TNFAIP6	2	152043693	1.11E-01	7750	6.24E-02	Up	1.86	6288	0.95	0.12
rs4397403	55124	PIWIL2	8	22228647	1.11E-01	7751	9.01E-01	Down	0.12	13252	0.95	0.00
rs707969	50619	DEF6	6	35397424	1.11E-01	7752	7.02E-02	Down	1.81	6430	0.95	0.12
rs17173373	58508	MLL3	7	151331323	1.11E-01	7753	4.32E-05	Up	4.09	2244	0.95	0.44
rs1938593	219972	MPEG1	11	58741293	1.11E-01	7754	3.29E-01	Up	0.98	9435	0.95	0.05
rs7240429	5596	MAPK4	18	46497316	1.11E-01	7755	2.06E-03	Down	3.08	3549	0.95	0.27
rs13382983	53335	BCL11A	2	60700704	1.11E-01	7756	3.89E-11	Down	6.61	698	0.95	1.04
rs1009948	7503	XIST		72836694	1.11E-01	7757	5.47E-02	Up	1.92	6105	0.95	0.13
rs17763104	1394	CRHR1	17	41261576	1.11E-01	7758	3.08E-01	Up	1.02	9253	0.95	0.05
rs7300945	55967	NDUFA12	12	93886505	1.11E-01	7759	1.01E-01	Up	1.64	7024	0.95	0.10
rs10128573	116337	PANX3	11	123975636	1.11E-01	7760	5.26E-01	Up	0.63	10913	0.95	0.03
rs7933086	23365	ARHGEF12	11	119774518	1.11E-01	7761	4.16E-01	Up	0.81	10090	0.95	0.04
rs10961677	340481	ZDHHC21	9	14699151	1.11E-01	7762	9.90E-04	Up	3.29	3219	0.95	0.30
rs12590918	89932	PAPLN	14	72794803	1.11E-01	7763	5.25E-02	Up	1.94	6049	0.95	0.13
rs1151102	932	MS4A3	11	59572216	1.11E-01	7764	6.34E-02	Up	1.86	6309	0.95	0.12
rs1151102	219990	PLAC1L	11	59572216	1.11E-01	7765	3.15E-01	Down	1.00	9321	0.95	0.05
rs10012310	84068	SLC10A7	4	147746774	1.11E-01	7766	3.88E-06	Up	4.62	1777	0.95	0.54
rs480253	5690	PSMB2	1	35785484	1.11E-01	7767	6.16E-01	Down	0.50	11547	0.95	0.02
rs6756107	2181	ACSL3	2	223624006	1.11E-01	7768	1.39E-08	Up	5.67	1085	0.95	0.79
rs4308217	942	CD86	3	123275877	1.11E-01	7769	1.54E-02	Up	2.42	4836	0.95	0.18
rs7960003	5542	PRB1	12	11389266	1.11E-01	7770	6.38E-01	Down	0.47	11692	0.95	0.02
rs279205	90592	ZNF700	19	11875274	1.11E-01	7771	3.29E-01	Up	0.98	9427	0.95	0.05

rs886451	4600	MX2	21	41695581	1.11E-01	7772	7.80E-02	Up	1.76	6594	0.95	0.11
rs4534977	255193	C19orf34	19	1903090	1.11E-01	7773	7.68E-01	Up	0.30	12475	0.95	0.01
rs12050562	2200	FBN1	15	46488520	1.11E-01	7774	9.25E-10	Down	6.12	888	0.95	0.90
rs10990609	11046	SLC35D2	9	96180190	1.12E-01	7775	2.31E-02	Up	2.27	5208	0.95	0.16
rs6880864	64924	SLC30A5	5	68458441	1.12E-01	7776	2.09E-03	Up	3.08	3555	0.95	0.27
rs12610507	147837	ZNF563	19	12297410	1.12E-01	7777	4.93E-03	Down	2.81	4058	0.95	0.23
rs1834	85369	FAM40A	1	110293822	1.12E-01	7778	8.48E-01	Up	0.19	12939	0.95	0.01
rs9494601	340146	SLC35D3	6	137283043	1.12E-01	7779	6.35E-02	Down	1.86	6312	0.95	0.12
rs1555133	140688	C20orf112	20	30512043	1.12E-01	7780	1.11E-02	Down	2.54	4579	0.95	0.20
rs2450559	2669	GEM	8	95354756	1.12E-01	7781	3.87E-04	Down	3.55	2871	0.95	0.34
rs2048213	375449	MAST4	5	66312660	1.12E-01	7782	1.32E-22	Down	9.78	150	0.95	2.19
rs6588528	374977	C1orf175	1	54868902	1.12E-01	7783	2.34E-02	Down	2.27	5219	0.95	0.16
rs3751756	9764	KIAA0513	16	83681263	1.12E-01	7784	1.22E-05	Down	4.37	1995	0.95	0.49
rs4801332	140612	ZFP28	19	61749068	1.12E-01	7785	3.79E-01	Up	0.88	9830	0.95	0.04
rs4665809	10890	RAB10	2	26163012	1.12E-01	7786	4.44E-01	Up	0.77	10295	0.95	0.04
rs11053043	969	CD69	12	9823337	1.12E-01	7787	3.11E-01	Up	1.01	9283	0.95	0.05
rs2517302	2267	FGL1	8	17779210	1.12E-01	7788	3.26E-02	Up	2.14	5548	0.95	0.15
rs3219154	57679	ALS2	2	202451943	1.12E-01	7789	6.69E-03	Up	2.71	4228	0.95	0.22
rs2303487	3217	HOXB7	17	44059535	1.12E-01	7790	6.17E-01	Up	0.50	11559	0.95	0.02
rs2303487	3218	HOXB8	17	44059535	1.12E-01	7791	6.19E-01	Down	0.50	11564	0.95	0.02
rs2303487	3219	HOXB9	17	44059535	1.12E-01	7792	7.42E-01	Down	0.33	12326	0.95	0.01
rs5977301	55855	FAM45B		129344002	1.12E-01	7793	2.23E-01	Up	1.22	8485	0.95	0.07
rs8075628	5635	PRPSAP1	17	71850066	1.12E-01	7794	1.28E-07	Up	5.28	1318	0.95	0.69
rs6118035	56255	TXNDC13	20	7899997	1.12E-01	7795	5.35E-04	Down	3.46	2982	0.95	0.33
rs6504698	54799	MBTD1	17	46662763	1.12E-01	7796	1.74E-03	Up	3.13	3460	0.95	0.28
rs7694627	2247	FGF2	4	124146100	1.12E-01	7797	1.03E-07	Down	5.32	1299	0.95	0.70
rs7694627	2258	FGF13	4	124146100	1.12E-01	7798	2.50E-02	Up	2.24	5288	0.95	0.16
rs2964589	475	ATOX1	5	151098382	1.12E-01	7799	6.03E-01	Down	0.52	11452	0.95	0.02
rs11799614	79098	C1orf116	1	203597759	1.12E-01	7800	3.68E-13	Up	7.26	520	0.95	1.24
rs11799614	55432	YOD1	1	203597759	1.12E-01	7801	2.24E-09	Up	5.98	941	0.95	0.87
rs11799614	5208	PFKFB2	1	203597759	1.12E-01	7802	4.14E-01	Up	0.82	10084	0.95	0.04
rs313829	435	ASL	7	64996647	1.12E-01	7803	1.60E-04	Up	3.77	2566	0.95	0.38
rs4452638	10279	PRSS16	6	27337244	1.12E-01	7804	1.93E-02	Down	2.34	5041	0.95	0.17
rs1467561	1152	CKB	14	103064714	1.12E-01	7805	2.68E-12	Down	6.99	598	0.95	1.16
rs3760948	10226	M6PRBP1	19	4819802	1.12E-01	7806	3.94E-03	Down	2.88	3920	0.95	0.24
rs7749020	3400	ID4	6	19956652	1.12E-01	7807	2.34E-26	Down	10.62	93	0.95	2.56
rs2615055	64747	MFSD1	3	160019812	1.12E-01	7808	2.48E-01	Down	1.16	8747	0.95	0.06
rs1535722	51562	MBIP	14	35872483	1.13E-01	7809	1.96E-02	Up	2.33	5051	0.95	0.17
rs2289977	29099	COMMD9	11	36268146	1.13E-01	7810	1.41E-01	Up	1.47	7593	0.95	0.08
rs7978273	28234	SLCO1B3	12	20843432	1.13E-01	7811	1.03E-02	Up	2.57	4526	0.95	0.20
rs17736503	8648	NCOA1	2	24866963	1.13E-01	7812	5.06E-01	Down	0.66	10761	0.95	0.03
rs4763291	1389	CREBL2	12	12644945	1.13E-01	7813	6.88E-08	Down	5.39	1255	0.95	0.72
rs2302898	10381	TUBB3	16	88526295	1.13E-01	7814	1.61E-04	Up	3.77	2567	0.95	0.38
rs2168753	5217	PFN2	3	151176772	1.13E-01	7815	1.74E-02	Up	2.38	4957	0.95	0.18
rs2302898	54849	DEF8	16	88526295	1.13E-01	7816	1.32E-01	Down	1.51	7467	0.95	0.09
rs2302898	10382	TUBB4	16	88526295	1.13E-01	7817	7.47E-01	Down	0.32	12353	0.95	0.01
rs12029680	148932	MOBKL2C	1	46792761	1.13E-01	7818	1.27E-05	Down	4.37	2006	0.95	0.49
rs2304900	60490	PPCDC	15	73127693	1.13E-01	7819	2.04E-01	Up	1.27	8295	0.95	0.07
rs804282	252969	NEIL2	8	11649152	1.13E-01	7820	7.10E-01	Up	0.37	12129	0.95	0.01
rs774299	84669	USP32	16	83366401	1.13E-01	7821	3.86E-01	Up	0.87	9884	0.95	0.04
rs774299	9100	USP10	16	83366401	1.13E-01	7822	4.78E-01	Down	0.71	10561	0.95	0.03
rs1235382	7733	ZNF180	19	49711347	1.13E-01	7823	5.53E-01	Up	0.59	11103	0.95	0.03
rs12077177	911	CD1C	1	155069089	1.13E-01	7824	3.33E-01	Up	0.97	9466	0.95	0.05
rs189737	84239	ATP13A4	3	194771087	1.13E-01	7825	2.95E-01	Down	1.05	9155	0.95	0.05
rs3802881	338657	CCDC84	11	118395232	1.13E-01	7826	5.26E-02	Up	1.94	6055	0.95	0.13
rs9861870	3699	ITIH3	3	52818862	1.13E-01	7827	2.69E-02	Down	2.21	5354	0.95	0.16
rs9861870	3700	ITIH4	3	52818862	1.13E-01	7828	8.25E-01	Up	0.22	12823	0.95	0.01
rs9304882	6794	STK11	19	1140482	1.13E-01	7829	6.77E-03	Down	2.71	4239	0.95	0.22
rs649150	115290	FBXO17	19	44173324	1.13E-01	7830	2.64E-01	Down	1.12	8898	0.95	0.06
rs521805	25998	IBTK	6	83030520	1.13E-01	7831	1.38E-02	Up	2.46	4746	0.95	0.19
rs10485505	83737	ITCH	20	32489228	1.13E-01	7832	3.02E-02	Up	2.17	5473	0.95	0.15
rs6505744	1149	CIDEA	18	12270922	1.13E-01	7833	6.48E-04	Down	3.41	3054	0.95	0.32
rs6747421	56886	UGCGL1	2	128584071	1.13E-01	7834	2.56E-04	Up	3.66	2717	0.95	0.36
rs12097583	65123	INTS3	1	150536110	1.13E-01	7835	7.71E-01	Up	0.29	12503	0.95	0.01
rs3745383	6618	SNAPC2	19	7871339	1.13E-01	7836	3.75E-02	Up	2.08	5695	0.95	0.14
rs3745383	5609	MAP2K7	19	7871339	1.13E-01	7837	5.55E-01	Down	0.59	11116	0.95	0.03
rs7700649	821	CANX	5	179053869	1.13E-01	7838	4.51E-16	Up	8.13	346	0.95	1.53
rs3751501	55017	C14orf119	14	22619125	1.13E-01	7839	6.12E-04	Down	3.43	3035	0.95	0.32
rs2297824	7804	LRP8	1	53475872	1.13E-01	7840	7.43E-02	Up	1.78	6516	0.95	0.11
rs7846567	575	BAI1	8	143609151	1.13E-01	7841	7.88E-01	Down	0.27	12598	0.95	0.01
rs11119388	255928	SYT14	1	206562812	1.14E-01	7842	1.39E-01	Down	1.48	7561	0.94	0.09
rs9532427	57511	COG6	13	39219352	1.14E-01	7843	1.89E-01	Down	1.31	8128	0.94	0.07
rs2121468	120103	SLC36A4	11	92571877	1.14E-01	7844	3.89E-03	Down	2.89	3913	0.94	0.24
rs12482697	539	ATP5O	21	34215069	1.14E-01	7845	3.28E-02	Down	2.13	5554	0.94	0.15
rs17038228	113612	CYP2U1	4	109195233	1.14E-01	7846	3.24E-04	Down	3.60	2803	0.94	0.35
rs17038228	166929	SGMS2	4	109195233	1.14E-01	7847	3.16E-01	Up	1.00	9328	0.94	0.05
rs11046265	55907	CMAS	12	22080897	1.14E-01	7848	1.73E-01	Down	1.36	7944	0.94	0.08
rs13032503	112942	CCDC104	2	55650744	1.14E-01	7849	2.56E-01	Down	1.14	8821	0.94	0.06
rs2001323	54777	C10orf92	10	134522842	1.14E-01	7850	4.25E-01	Down	0.80	10157	0.94	0.04
rs1863703	27148	STK36	2	219369893	1.14E-01	7851	8.54E-19	Up	8.83	240	0.94	1.81
rs1863703	7701	ZNF142	2	219369893	1.14E-01	7852	8.02E-04	Up	3.35	3140	0.94	0.31

rs1863703	64320	RNF25	2	219369893	1.14E-01	7853	3.12E-01	Down	1.01	9287	0.94	0.05
rs1533939	7220	TRPC1	3	143912478	1.14E-01	7854	8.26E-03	Down	2.64	4365	0.94	0.21
rs17726596	3340	NDST1	5	149864637	1.14E-01	7855	2.06E-01	Up	1.27	8311	0.94	0.07
rs6706490	80122	YSK4	2	135592827	1.14E-01	7856	8.41E-02	Down	1.73	6701	0.94	0.11
rs2284827	83733	SLC25A18	22	16421785	1.14E-01	7857	9.87E-02	Down	1.65	6977	0.94	0.10
rs7230661	9306	SOCS6	18	66099091	1.14E-01	7858	1.47E-03	Down	3.18	3371	0.94	0.28
rs2455826	686	BTD	3	15638064	1.14E-01	7859	1.12E-05	Up	4.39	1972	0.94	0.50
rs4646124	59272	ACE2		15376453	1.14E-01	7860	5.66E-01	Down	0.57	11216	0.94	0.02
rs1675414	7020	TFAP2A	6	10520174	1.14E-01	7861	5.27E-01	Down	0.63	10925	0.94	0.03
rs754814	84225	ZMYND15	17	4603783	1.14E-01	7862	6.39E-02	Down	1.85	6321	0.94	0.12
rs754814	9032	TM4SF5	17	4603783	1.14E-01	7863	8.76E-02	Up	1.71	6765	0.94	0.11
rs754814	58191	CXCL16	17	4603783	1.14E-01	7864	9.64E-01	Down	0.05	13641	0.94	0.00
rs730775	347734	SLC35B2	6	44340052	1.14E-01	7865	2.28E-12	Up	7.01	590	0.94	1.16
rs730775	4794	NFKBIE	6	44340052	1.14E-01	7866	8.64E-02	Down	1.71	6745	0.94	0.11
rs2282033	55051	C14orf102	14	89839388	1.14E-01	7867	6.24E-01	Up	0.49	11612	0.94	0.02
rs1011411	139322	APOOL		84111138	1.14E-01	7868	5.49E-04	Up	3.46	2994	0.94	0.33
rs10417922	25796	PGLS	19	17476609	1.14E-01	7869	2.17E-03	Up	3.07	3590	0.94	0.27
rs4354491	55706	TMEM48	1	54026064	1.14E-01	7870	2.42E-05	Up	4.22	2122	0.94	0.46
rs4354491	54432	YIPF1	1	54026064	1.14E-01	7871	2.10E-01	Up	1.25	8347	0.94	0.07
rs875511	3675	ITGA3	17	45514200	1.14E-01	7872	3.63E-16	Down	8.15	341	0.94	1.54
rs1706816	53905	DUOX1	15	43247714	1.14E-01	7873	1.73E-10	Down	6.38	787	0.94	0.98
rs12521153	10468	FST	5	52823958	1.14E-01	7874	5.65E-01	Down	0.58	11207	0.94	0.02
rs13383985	130399	ACVR1C	2	158281488	1.14E-01	7875	9.99E-01	Down	0.00	13903	0.94	0.00
rs16839633	166348	KLHDC6	3	129176115	1.14E-01	7876	1.76E-01	Up	1.35	7969	0.94	0.08
rs32636	54491	FAM105A	5	14627119	1.14E-01	7877	1.27E-01	Down	1.52	7412	0.94	0.09
rs177088	2820	GPD2	2	157100730	1.14E-01	7878	2.24E-05	Up	4.24	2109	0.94	0.47
rs4787938	79831	JMJD5	16	27113841	1.14E-01	7879	7.11E-01	Down	0.37	12134	0.94	0.01
rs5770874	113730	KLHDC7B	22	49270962	1.14E-01	7880	9.08E-01	Up	0.11	13299	0.94	0.00
rs8078781	4991	OR1D2	17	2955632	1.14E-01	7881	6.96E-01	Down	0.39	12044	0.94	0.02
rs5981119	6872	TAF1		70466240	1.14E-01	7882	2.62E-03	Up	3.01	3713	0.94	0.26
rs11116657	55117	SLC6A15	12	83825894	1.14E-01	7883	6.71E-02	Up	1.83	6378	0.94	0.12
rs1475436	80314	EPC1	10	32650709	1.14E-01	7884	5.32E-06	Up	4.55	1829	0.94	0.53
rs4858968	3355	HTR1F	3	88108776	1.14E-01	7885	2.81E-01	Down	1.08	9044	0.94	0.06
rs3213403	284346	ZNF575	19	48738568	1.15E-01	7886	6.98E-01	Up	0.39	12059	0.94	0.02
rs17621311	60481	ELOVL5	6	53236336	1.15E-01	7887	2.37E-03	Up	3.04	3652	0.94	0.26
rs13394090	6508	SLC4A3	2	220341839	1.15E-01	7888	2.31E-01	Up	1.20	8574	0.94	0.06
rs9889879	84081	CCDC55	17	25532524	1.15E-01	7889	7.95E-02	Down	1.75	6622	0.94	0.11
rs11667587	4858	NOVA2	19	51156538	1.15E-01	7890	9.85E-01	Down	0.02	13811	0.94	0.00
rs4984803	7329	UBE2I	16	1289930	1.15E-01	7891	8.20E-04	Up	3.35	3146	0.94	0.31
rs17085709	22828	RBM16	6	155252549	1.15E-01	7892	3.71E-01	Up	0.89	9770	0.94	0.04
rs6503133	4628	MYH10	17	8456361	1.15E-01	7893	3.96E-01	Up	0.85	9953	0.94	0.04
rs1278769	23250	ATP11A	13	112584628	1.15E-01	7894	1.77E-20	Up	9.28	187	0.94	1.98
rs6014755	200232	C20orf106	20	54518573	1.15E-01	7895	9.53E-01	Up	0.06	13580	0.94	0.00
rs716124	29951	PDZRN4	12	40240245	1.15E-01	7896	1.59E-10	Down	6.40	784	0.94	0.98
rs2377419	719	C3AR1	12	8120914	1.15E-01	7897	9.86E-04	Up	3.29	3217	0.94	0.30
rs2377419	25977	NECAP1	12	8120914	1.15E-01	7898	3.32E-01	Up	0.97	9460	0.94	0.05
rs4487686	54784	ALKBH4	7	101694989	1.15E-01	7899	2.46E-01	Down	1.16	8728	0.94	0.06
rs4487686	222229	LRWD1	7	101694989	1.15E-01	7900	6.14E-01	Down	0.50	11537	0.94	0.02
rs4918409	3070	HELLS	10	96333218	1.15E-01	7901	3.05E-06	Up	4.67	1741	0.94	0.55
rs2838037	4599	MX1	21	41733740	1.15E-01	7902	1.14E-02	Down	2.53	4602	0.94	0.19
rs12530477	83732	RIOK1	6	7356830	1.15E-01	7903	1.82E-03	Up	3.12	3485	0.94	0.27
rs11241150	114915	C5orf26	5	111504303	1.15E-01	7904	2.66E-02	Up	2.22	5345	0.94	0.16
rs2276847	93973	ACTR8	3	53900815	1.15E-01	7905	9.17E-01	Down	0.10	13362	0.94	0.00
rs12436047	54916	C14orf101	14	56114801	1.15E-01	7906	6.74E-01	Up	0.42	11910	0.94	0.02
rs2584608	6603	SMARCD2	17	59290442	1.15E-01	7907	1.12E-03	Up	3.26	3260	0.94	0.30
rs5019656	6999	TDO2	12	70608710	1.15E-01	7908	5.01E-02	Up	1.96	6001	0.94	0.13
rs5019656	121278	TPH2	12	70608710	1.15E-01	7909	8.14E-01	Down	0.23	12752	0.94	0.01
rs2783061	222826	C6orf146	6	4012127	1.16E-01	7910	5.51E-02	Down	1.92	6114	0.94	0.13
rs2783061	8899	PRPF4B	6	4012127	1.16E-01	7911	1.68E-01	Up	1.38	7898	0.94	0.08
rs2235307	8862	APLN		128510367	1.16E-01	7912	3.74E-02	Up	2.08	5689	0.94	0.14
rs464582	51339	DACT1	14	58171201	1.16E-01	7913	6.70E-01	Down	0.43	11890	0.94	0.02
rs850610	476	ATP1A1	1	116660754	1.16E-01	7914	3.77E-01	Down	0.88	9819	0.94	0.04
rs782791	8497	PPFIA4	1	199730476	1.16E-01	7915	7.32E-02	Up	1.79	6490	0.94	0.11
rs9401596	2173	FABP7	6	123146402	1.16E-01	7916	2.67E-03	Down	3.00	3722	0.94	0.26
rs9401596	10924	SMPDL3A	6	123146402	1.16E-01	7917	6.27E-03	Up	2.73	4190	0.94	0.22
rs6942044	9096	TBX18	6	85528183	1.16E-01	7918	8.81E-01	Up	0.15	13138	0.94	0.01
rs16989628	6789	STK4	20	43111021	1.16E-01	7919	1.01E-01	Up	1.64	7025	0.94	0.10
rs9851576	1849	DUSP7	3	52066294	1.16E-01	7920	6.74E-01	Down	0.42	11909	0.94	0.02
rs1894410	4288	MKI67	10	129794907	1.16E-01	7921	4.36E-05	Up	4.09	2245	0.94	0.44
rs13422838	3685	ITGAV	2	187328352	1.16E-01	7922	4.05E-01	Up	0.83	10018	0.94	0.04
rs2239713	26240	FAM50B	6	3794304	1.16E-01	7923	1.84E-01	Up	1.33	8069	0.94	0.07
rs10903129	55219	TMEM57	1	25514253	1.16E-01	7924	4.31E-06	Up	4.60	1792	0.94	0.54
rs6700323	63827	BCAN	1	153439431	1.16E-01	7925	2.40E-02	Down	2.26	5249	0.94	0.16
rs12405855	1647	GADD45A	1	67848487	1.16E-01	7926	4.96E-03	Down	2.81	4063	0.94	0.23
rs11135869	54793	KCTD9	8	25391271	1.16E-01	7927	9.45E-03	Down	2.60	4468	0.94	0.20
rs11135869	157313	CDCA2	8	25391271	1.16E-01	7928	1.34E-01	Up	1.50	7500	0.94	0.09
rs2232248	51409	HEMK1	3	50584628	1.16E-01	7929	3.19E-01	Down	1.00	9351	0.94	0.05
rs3828334	2817	GPC1	2	241109992	1.16E-01	7930	1.09E-08	Down	5.72	1066	0.94	0.80
rs12424924	79912	PYROXD1	12	21507490	1.16E-01	7931	7.22E-02	Down	1.80	6471	0.94	0.11
rs6558061	157574	FBXO16	8	28354312	1.16E-01	7932	1.93E-01	Up	1.30	8185	0.94	0.07
rs5957347	55026	FAM70A		119193342	1.16E-01	7933	1.21E-01	Up	1.55	7304	0.94	0.09

rs12667746	221830	TWISTNB	7	19503778	1.16E-01	7934	4.83E-10	Up	6.22	855	0.94	0.93
rs6020792	55653	BCAS4	20	48898809	1.16E-01	7935	9.58E-02	Down	1.67	6921	0.94	0.10
rs583058	29937	NENF	1	208999150	1.16E-01	7936	2.71E-02	Down	2.21	5363	0.94	0.16
rs4766961	1337	COX6A1	12	119325252	1.16E-01	7937	1.56E-07	Up	5.24	1347	0.93	0.68
rs11880333	770	CA11	19	53836602	1.16E-01	7938	7.48E-02	Down	1.78	6526	0.93	0.11
rs11880333	1628	DBP	19	53836602	1.16E-01	7939	8.97E-01	Down	0.13	13223	0.93	0.00
rs916904	10272	FSTL3	19	614929	1.16E-01	7940	2.37E-05	Down	4.23	2119	0.93	0.46
rs5945680	56849	TCEAL7		102397978	1.16E-01	7941	1.07E-04	Down	3.87	2471	0.93	0.40
rs5945680	51186	WBP5		102397978	1.16E-01	7942	5.57E-01	Down	0.59	11136	0.93	0.03
rs13279569	2260	FGFR1	8	38460773	1.16E-01	7943	6.79E-23	Down	9.85	141	0.93	2.22
rs4903542	85457	KIAA1737	14	76640231	1.16E-01	7944	3.29E-01	Up	0.98	9433	0.93	0.05
rs3818711	92399	MRRF	9	122155336	1.16E-01	7945	3.72E-07	Up	5.08	1445	0.93	0.64
rs17795070	23351	KIAA0323	14	23953470	1.16E-01	7946	7.88E-01	Down	0.27	12600	0.93	0.01
rs1284300	5174	PDZK1	1	143236507	1.17E-01	7947	4.08E-01	Up	0.83	10041	0.93	0.04
rs2224	254428	SLC41A1	1	202501208	1.17E-01	7948	1.82E-03	Down	3.12	3484	0.93	0.27
rs6503096	5198	PFAS	17	8122992	1.17E-01	7949	9.80E-08	Up	5.33	1296	0.93	0.70
rs6503096	29098	RANGRF	17	8122992	1.17E-01	7950	2.61E-02	Down	2.22	5323	0.93	0.16
rs2301739	84268	RPAIN	17	5249101	1.17E-01	7951	5.72E-01	Up	0.57	11255	0.93	0.02
rs4926928	79699	ZYG11B	1	52922184	1.17E-01	7952	2.00E-02	Down	2.33	5071	0.93	0.17
rs3800018	51715	RAB23	6	57177565	1.17E-01	7953	1.00E-01	Down	1.64	6999	0.93	0.10
rs2041306	64149	C17orf75	17	27689788	1.17E-01	7954	2.25E-01	Up	1.21	8507	0.93	0.06
rs6093838	6431	SFRS6	20	41510850	1.17E-01	7955	5.60E-03	Up	2.77	4143	0.93	0.23
rs7294681	55198	APPL2	12	104139093	1.17E-01	7956	1.90E-06	Up	4.76	1658	0.93	0.57
rs12848684	1730	DIAPH2		96320747	1.17E-01	7957	3.04E-01	Down	1.03	9220	0.93	0.05
rs3181165	135932	TMEM139	7	142503521	1.17E-01	7958	1.07E-01	Down	1.61	7113	0.93	0.10
rs9860004	8893	EIF2B5	3	185327014	1.17E-01	7959	2.26E-01	Up	1.21	8519	0.93	0.06
rs3776105	29930	PCDHB1	5	140410127	1.17E-01	7960	2.75E-01	Up	1.09	8992	0.93	0.06
rs7230063	9525	VPS4B	18	59220773	1.17E-01	7961	9.29E-01	Down	0.09	13423	0.93	0.00
rs4772795	1948	EFNB2	13	105967395	1.17E-01	7962	1.09E-01	Up	1.60	7136	0.93	0.10
rs16949778	115752	DIS3L	15	64367339	1.17E-01	7963	8.96E-01	Down	0.13	13217	0.93	0.00
rs6674802	8289	ARID1A	1	26688732	1.17E-01	7964	6.54E-15	Up	7.80	394	0.93	1.42
rs13065059	84541	KBTBD8	3	67113940	1.17E-01	7965	9.87E-01	Down	0.02	13822	0.93	0.00
rs1052620	4690	NCK1	3	138057219	1.17E-01	7966	7.61E-01	Up	0.30	12445	0.93	0.01
rs685441	5833	PCYT2	17	72286420	1.17E-01	7967	9.86E-01	Down	0.02	13815	0.93	0.00
rs17750998	57794	SF4	19	19249446	1.17E-01	7968	2.10E-01	Down	1.25	8354	0.93	0.07
rs17750998	53345	TM6SF2	19	19249446	1.17E-01	7969	3.38E-01	Down	0.96	9506	0.93	0.05
rs17750998	404037	HAPLN4	19	19249446	1.17E-01	7970	7.97E-01	Up	0.26	12653	0.93	0.01
rs7609407	150726	FBXO41	2	73411783	1.17E-01	7971	8.57E-01	Down	0.18	12994	0.93	0.01
rs10875995	6602	SMARCD1	12	48748405	1.17E-01	7972	2.31E-01	Up	1.20	8576	0.93	0.06
rs10972023	51271	UBAP1	9	34243720	1.17E-01	7973	9.94E-01	Up	0.01	13874	0.93	0.00
rs1567894	660	BMX		15270512	1.17E-01	7974	5.39E-01	Up	0.62	11001	0.93	0.03
rs1567894	8544	PIR		15270512	1.17E-01	7975	5.49E-01	Down	0.60	11072	0.93	0.03
rs17075440	1005	CDH7	18	61704561	1.17E-01	7976	6.96E-01	Up	0.39	12042	0.93	0.02
rs2753260	6504	SLAMF1	1	157401668	1.18E-01	7977	8.24E-01	Up	0.22	12814	0.93	0.01
rs4824629	23415	KCNH4		47271327	1.18E-01	7978	1.76E-01	Down	1.35	7974	0.93	0.08
rs4824629	2002	ELK1		47271327	1.18E-01	7979	7.92E-01	Up	0.26	12628	0.93	0.01
rs4824629	8409	UXT		47271327	1.18E-01	7980	8.12E-01	Down	0.24	12734	0.93	0.01
rs3182634	27013	C2orf24	2	219863171	1.18E-01	7981	9.24E-02	Up	1.68	6869	0.93	0.10
rs3182634	151295	SLC23A3	2	219863171	1.18E-01	7982	4.89E-01	Down	0.69	10650	0.93	0.03
rs3182634	79840	NHEJ1	2	219863171	1.18E-01	7983	5.17E-01	Up	0.65	10834	0.93	0.03
rs10789626	55531	ELMOD1	11	107008657	1.18E-01	7984	7.50E-01	Down	0.32	12376	0.93	0.01
rs4714384	1906	EDN1	6	12405839	1.18E-01	7985	2.51E-01	Up	1.15	8774	0.93	0.06
rs16847945	80232	WDR26	1	220884132	1.18E-01	7986	7.68E-02	Up	1.77	6572	0.93	0.11
rs7568196	1593	CYP27A1	2	219477878	1.18E-01	7987	3.56E-13	Down	7.27	516	0.93	1.24
rs1475931	2155	F7	13	112811927	1.18E-01	7988	6.44E-01	Up	0.46	11739	0.93	0.02
rs707899	8334	HIST1H2AC	6	26237927	1.18E-01	7989	4.00E-08	Up	5.49	1206	0.93	0.74
rs707899	8347	HIST1H2BC	6	26237927	1.18E-01	7990	8.04E-05	Up	3.94	2391	0.93	0.41
rs7148147	122769	PPIL5	14	49144270	1.18E-01	7991	1.38E-01	Up	1.48	7543	0.93	0.09
rs17795657	29124	LGALS13	19	44789530	1.18E-01	7992	3.80E-01	Down	0.88	9836	0.93	0.04
rs9914220	9021	SOCS3	17	73874485	1.18E-01	7993	8.53E-01	Up	0.19	12973	0.93	0.01
rs6062879	55738	ARFGAP1	20	61394869	1.18E-01	7994	1.85E-03	Up	3.11	3494	0.93	0.27
rs11711336	50853	VILL	3	38001588	1.18E-01	7995	1.60E-04	Down	3.77	2565	0.93	0.38
rs11711336	10217	CTDSPL	3	38001588	1.18E-01	7996	4.81E-02	Up	1.98	5953	0.93	0.13
rs10131232	2643	GCH1	14	54385658	1.18E-01	7997	2.92E-01	Up	1.05	9132	0.93	0.05
rs1329149	1571	CYP2E1	10	135238682	1.18E-01	7998	2.35E-01	Down	1.19	8610	0.93	0.06
rs4789922	114897	C1QTNF1	17	74551723	1.18E-01	7999	5.41E-02	Down	1.93	6090	0.93	0.13
rs803073	10603	SH2B2	7	101521714	1.18E-01	8000	3.63E-01	Up	0.91	9707	0.93	0.04
rs12907018	6557	SLC12A1	15	46307224	1.18E-01	8001	2.83E-01	Up	1.07	9056	0.93	0.05
rs6575121	5700	PSMC1	14	89810027	1.18E-01	8002	8.69E-05	Down	3.92	2415	0.93	0.41
rs2070006	2243	FGA	4	155871471	1.18E-01	8003	8.17E-01	Up	0.23	12768	0.93	0.01
rs2327085	51000	SLC35B3	6	8355535	1.18E-01	8004	8.83E-01	Up	0.15	13146	0.93	0.01
rs1807210	51716	CES4	16	54341207	1.18E-01	8005	8.29E-01	Up	0.22	12848	0.93	0.01
rs6734	22916	NCBP2	3	198150831	1.19E-01	8006	1.15E-01	Up	1.57	7225	0.93	0.09
rs11191293	79946	C10orf95	10	104211529	1.19E-01	8007	9.22E-01	Up	0.10	13386	0.93	0.00
rs3775442	6622	SNCA	4	91072409	1.19E-01	8008	1.15E-01	Down	1.57	7223	0.93	0.09
rs876602	2974	GUCY1B2	13	50522108	1.19E-01	8009	1.98E-01	Up	1.29	8230	0.93	0.07
rs1449079	1829	DSG2	18	27386173	1.19E-01	8010	5.64E-04	Up	3.45	3005	0.93	0.32
rs2444860	6156	RPL30	8	99122071	1.19E-01	8011	9.79E-09	Up	5.75	1046	0.93	0.80
rs11107870	7181	NR2C1	12	93936920	1.19E-01	8012	1.37E-02	Up	2.46	4741	0.93	0.19
rs26819	23262	HISPPD1	5	102557300	1.19E-01	8013	4.10E-03	Up	2.87	3947	0.93	0.24
rs487624	10786	SLC17A3	6	25987518	1.19E-01	8014	3.15E-01	Down	1.01	9312	0.93	0.05

rs12598600	4501	MT1X	16	55279371	1.19E-01	8015	2.89E-17	Down	8.45	294	0.92	1.65
rs12598600	4495	MT1G	16	55279371	1.19E-01	8016	2.14E-03	Down	3.07	3576	0.92	0.27
rs4329955	84311	MRPL45	17	33692790	1.19E-01	8017	2.73E-03	Up	3.00	3736	0.92	0.26
rs10972175	138716	C9orf23	9	34615409	1.19E-01	8018	7.32E-06	Up	4.48	1879	0.92	0.51
rs10972175	11258	DCTN3	9	34615409	1.19E-01	8019	3.50E-01	Down	0.93	9586	0.92	0.05
rs1866347	4824	NKX3-1	8	23603455	1.19E-01	8020	1.80E-01	Up	1.34	8026	0.92	0.07
rs7609080	90423	ATP6V1E2	2	46645434	1.19E-01	8021	3.72E-01	Down	0.89	9781	0.92	0.04
rs496674	9125	RQCD1	2	219258074	1.19E-01	8022	2.79E-05	Up	4.19	2155	0.92	0.46
rs496674	57695	USP37	2	219258074	1.19E-01	8023	2.19E-01	Up	1.23	8447	0.92	0.07
rs7912419	22944	KIN	10	7828501	1.19E-01	8024	9.31E-02	Up	1.68	6887	0.92	0.10
rs2744465	2130	EWSR1	22	27978165	1.19E-01	8025	2.14E-01	Up	1.24	8400	0.92	0.07
rs6962610	79778	MICALL2	7	1280252	1.19E-01	8026	5.12E-01	Up	0.66	10790	0.92	0.03
rs2305762	55900	ZNF302	19	39861380	1.19E-01	8027	8.31E-02	Up	1.73	6687	0.92	0.11
rs2272473	1981	EIF4G1	3	185503607	1.19E-01	8028	2.90E-04	Up	3.62	2764	0.92	0.35
rs12215331	64771	C6orf106	6	34752727	1.19E-01	8029	1.80E-01	Down	1.34	8019	0.92	0.07
rs820386	2584	GALK1	17	71270899	1.19E-01	8030	8.96E-01	Down	0.13	13220	0.92	0.00
rs11185293	29957	SLC25A24	1	108415876	1.19E-01	8031	4.40E-01	Down	0.77	10272	0.92	0.04
rs2180660	10621	POLR3F	20	18387884	1.19E-01	8032	5.37E-01	Up	0.62	10986	0.92	0.03
rs9906553	8493	PPM1D	17	56015065	1.19E-01	8033	9.34E-02	Down	1.68	6891	0.92	0.10
rs3776969	4437	MSH3	5	80042738	1.19E-01	8034	7.02E-07	Up	4.96	1523	0.92	0.62
rs742097	51493	C22orf28	22	31116996	1.19E-01	8035	5.82E-05	Down	4.02	2323	0.92	0.42
rs11189358	6425	SFRP5	10	99506823	1.19E-01	8036	6.60E-01	Down	0.44	11833	0.92	0.02
rs1298190	252995	FNDC5	1	33009131	1.20E-01	8037	4.17E-03	Down	2.87	3962	0.92	0.24
rs1298190	3208	HPCA	1	33009131	1.20E-01	8038	6.16E-02	Up	1.87	6270	0.92	0.12
rs10401741	23770	FKBP8	19	18499405	1.20E-01	8039	2.12E-01	Down	1.25	8374	0.92	0.07
rs7817747	377677	CA13	8	86339131	1.20E-01	8040	1.91E-04	Down	3.73	2616	0.92	0.37
rs470085	25830	SULT4A1	22	42544617	1.20E-01	8041	2.94E-01	Up	1.05	9144	0.92	0.05
rs3821107	7520	XRCC5	2	216856834	1.20E-01	8042	1.13E-01	Down	1.59	7190	0.92	0.09
rs3810481	79025	C20orf195	20	61663889	1.20E-01	8043	1.05E-01	Down	1.62	7086	0.92	0.10
rs3108207	92283	ZNF461	19	41863042	1.20E-01	8044	4.42E-01	Up	0.77	10279	0.92	0.04
rs929250	3209	HOXA13	7	27018341	1.20E-01	8045	6.29E-06	Down	4.52	1853	0.92	0.52
rs7978149	283383	GPR133	12	130031900	1.20E-01	8046	8.73E-01	Down	0.16	13083	0.92	0.01
rs17124827	161436	EML5	14	88237180	1.20E-01	8047	8.63E-03	Down	2.63	4399	0.92	0.21
rs11540270	7706	TRIM25	17	52321804	1.20E-01	8048	3.58E-04	Up	3.57	2844	0.92	0.34
rs785159	57415	C3orf14	3	62271910	1.20E-01	8049	2.24E-01	Down	1.22	8493	0.92	0.07
rs10044000	347732	CATSPER3	5	134371560	1.20E-01	8050	2.10E-01	Up	1.25	8351	0.92	0.07
rs4822062	6721	SREBF2	22	40598787	1.20E-01	8051	2.03E-01	Down	1.27	8291	0.92	0.07
rs7905887	5407	PNLIPRP1	10	118357158	1.20E-01	8052	1.03E-02	Down	2.57	4527	0.92	0.20
rs1254900	129303	TMEM150	2	85727992	1.20E-01	8053	1.16E-01	Up	1.57	7236	0.92	0.09
rs17199026	91010	FMNL3	12	48341609	1.20E-01	8054	7.23E-01	Down	0.35	12221	0.92	0.01
rs12189801	57211	GPR126	6	142683299	1.20E-01	8055	5.35E-02	Down	1.93	6076	0.92	0.13
rs7585732	2019	EN1	2	119337646	1.20E-01	8056	2.47E-02	Up	2.25	5275	0.92	0.16
rs9910373	22794	CASC3	17	35561768	1.20E-01	8057	1.95E-05	Down	4.27	2085	0.92	0.47
rs629631	1991	ELA2	19	797041	1.20E-01	8058	4.59E-01	Down	0.74	10413	0.92	0.03
rs629631	5657	PRTN3	19	797041	1.20E-01	8059	6.73E-01	Down	0.42	11901	0.92	0.02
rs3129871	3122	HLA-DRA	6	32514320	1.20E-01	8060	3.74E-02	Up	2.08	5688	0.92	0.14
rs6537355	2617	GARS	4	146760197	1.20E-01	8061	1.39E-08	Up	5.67	1084	0.92	0.79
rs6537355	4086	SMAD1	4	146760197	1.20E-01	8062	5.96E-05	Down	4.01	2331	0.92	0.42
rs6446768	22902	RUFY3	4	71987456	1.20E-01	8063	5.83E-01	Down	0.55	11332	0.92	0.02
rs12897986	145258	GSC	14	94315023	1.20E-01	8064	1.22E-01	Down	1.55	7338	0.92	0.09
rs2328832	81688	C6orf62	6	24837313	1.20E-01	8065	6.58E-03	Up	2.72	4218	0.92	0.22
rs888995	81	ACTN4	19	43847978	1.20E-01	8066	9.47E-05	Down	3.90	2439	0.92	0.40
rs17477867	3697	ITIH1	3	52771013	1.20E-01	8067	5.25E-04	Up	3.47	2975	0.92	0.33
rs17477867	6787	NEK4	3	52771013	1.20E-01	8068	5.20E-01	Up	0.64	10863	0.92	0.03
rs12192603	353219	KAAG1	6	24450350	1.20E-01	8069	4.30E-01	Down	0.79	10192	0.92	0.04
rs6945133	94239	H2AFV	7	44674275	1.20E-01	8070	5.63E-01	Up	0.58	11185	0.92	0.02
rs16924082	3945	LDHB	12	21672408	1.20E-01	8071	4.70E-09	Down	5.86	1004	0.92	0.83
rs10512602	9368	SLC9A3R1	17	70285770	1.20E-01	8072	6.70E-01	Up	0.43	11883	0.92	0.02
rs7502208	4591	TRIM37	17	54519879	1.21E-01	8073	1.55E-05	Up	4.32	2043	0.92	0.48
rs10758344	8434	RECK	9	36100806	1.21E-01	8074	6.02E-01	Down	0.52	11439	0.92	0.02
rs6506650	23253	ANKRD12	18	9275824	1.21E-01	8075	8.53E-01	Down	0.18	12976	0.92	0.01
rs2677424	131890	GRK7	3	142968702	1.21E-01	8076	9.29E-01	Up	0.09	13428	0.92	0.00
rs12630739	51738	GHRL	3	10316082	1.21E-01	8077	8.58E-01	Down	0.18	13001	0.92	0.01
rs1153799	282966	C10orf53	10	50573041	1.21E-01	8078	5.42E-01	Up	0.61	11028	0.92	0.03
rs7014054	84296	GINS4	8	41487657	1.21E-01	8079	2.71E-01	Up	1.10	8960	0.92	0.06
rs7014054	51125	GOLGA7	8	41487657	1.21E-01	8080	5.50E-01	Down	0.60	11082	0.92	0.03
rs3750551	9414	TJP2	9	69091683	1.21E-01	8081	3.08E-02	Down	2.16	5491	0.92	0.15
rs2926362	55718	POLR3E	16	22237539	1.21E-01	8082	3.89E-02	Up	2.07	5728	0.92	0.14
rs12484697	158	ADSL	22	39060972	1.21E-01	8083	2.47E-11	Up	6.69	676	0.92	1.06
rs8045611	4094	MAF	16	78200350	1.21E-01	8084	3.82E-19	Down	8.94	224	0.92	1.84
rs10507372	2835	GPR12	13	26221607	1.21E-01	8085	7.72E-01	Down	0.29	12509	0.92	0.01
rs2518827	421	ARVCF	22	18385284	1.21E-01	8086	4.04E-07	Up	5.07	1457	0.92	0.64
rs10979019	22869	ZNF510	9	96631192	1.21E-01	8087	4.83E-01	Up	0.70	10596	0.92	0.03
rs1861463	389058	SP5	2	171409611	1.21E-01	8088	2.08E-03	Up	3.08	3554	0.92	0.27
rs9405048	11270	NRM	6	30778271	1.21E-01	8089	1.05E-05	Down	4.41	1959	0.92	0.50
rs9405048	9656	MDC1	6	30778271	1.21E-01	8090	3.81E-04	Down	3.55	2863	0.92	0.34
rs2161373	1843	DUSP1	5	172145974	1.21E-01	8091	5.86E-01	Up	0.54	11347	0.92	0.02
rs10184254	344018	FIGLA	2	70925977	1.21E-01	8092	5.15E-01	Down	0.65	10822	0.92	0.03
rs1111434	27189	IL17C	16	87215936	1.22E-01	8093	4.39E-02	Down	2.02	5848	0.92	0.14
rs774211	5939	RBMS2	12	55207206	1.22E-01	8094	9.71E-03	Down	2.59	4489	0.92	0.20
rs7072552	4838	NODAL	10	71874967	1.22E-01	8095	9.79E-02	Down	1.66	6961	0.91	0.10

rs3786949	9535	GMFG	19	44518319	1.22E-01	8096	2.94E-02	Down	2.18	5444	0.91	0.15
rs3786949	55095	SAMD4B	19	44518319	1.22E-01	8097	1.35E-01	Down	1.49	7519	0.91	0.09
rs3786949	57622	LRFN1	19	44518319	1.22E-01	8098	5.73E-01	Up	0.56	11264	0.91	0.02
rs324727	8615	USO1	4	77079220	1.22E-01	8099	2.49E-05	Up	4.22	2128	0.91	0.46
rs2293429	51380	CSAD	12	51860170	1.22E-01	8100	3.88E-02	Down	2.07	5721	0.91	0.14
rs1668589	283748	PLA2G4D	15	40151061	1.22E-01	8101	3.60E-01	Down	0.92	9676	0.91	0.04
rs2237713	4233	MET	7	115937091	1.22E-01	8102	9.79E-15	Down	7.74	409	0.91	1.40
rs4939148	26519	TIMM10	11	57047537	1.22E-01	8103	8.70E-04	Up	3.33	3164	0.91	0.31
rs11195943	51703	ACSL5	10	114144805	1.22E-01	8104	5.00E-08	Up	5.45	1226	0.91	0.73
rs8063461	7249	TSC2	16	2055820	1.22E-01	8105	2.61E-04	Up	3.65	2728	0.91	0.36
rs8063461	4913	NTHL1	16	2055820	1.22E-01	8106	1.74E-01	Up	1.36	7955	0.91	0.08
rs1160318	25950	RWDD3	1	95415465	1.22E-01	8107	1.41E-06	Up	4.82	1617	0.91	0.59
rs943992	51222	ZNF219	14	20622897	1.22E-01	8108	8.07E-03	Down	2.65	4348	0.91	0.21
rs4687609	25886	WDR51A	3	52115631	1.22E-01	8109	5.68E-01	Down	0.57	11227	0.91	0.02
rs2288080	114960	TSGA13	7	129814773	1.23E-01	8110	4.15E-01	Up	0.82	10087	0.91	0.04
rs2907393	54757	FAM20A	17	64116040	1.23E-01	8111	3.70E-02	Up	2.09	5678	0.91	0.14
rs11778693	64236	PDLIM2	8	22518797	1.23E-01	8112	7.45E-02	Down	1.78	6519	0.91	0.11
rs10870149	26086	GPSM1	9	136530734	1.23E-01	8113	3.88E-03	Up	2.89	3909	0.91	0.24
rs843748	98	ACYP2	2	54414563	1.23E-01	8114	4.56E-01	Down	0.74	10395	0.91	0.03
rs556595	256472	TMEM151A	11	65814658	1.23E-01	8115	2.85E-01	Down	1.07	9072	0.91	0.05
rs556595	254263	CNIH2	11	65814658	1.23E-01	8116	8.01E-01	Down	0.25	12678	0.91	0.01
rs9931084	29855	UBN1	16	4853155	1.23E-01	8117	3.05E-04	Up	3.61	2780	0.91	0.35
rs175057	27030	MLH3	14	74559385	1.23E-01	8118	5.51E-03	Down	2.78	4132	0.91	0.23
rs231461	5697	PYY	17	39388569	1.23E-01	8119	8.97E-01	Down	0.13	13226	0.91	0.00
rs231461	5539	PPY	17	39388569	1.23E-01	8120	9.65E-01	Up	0.04	13655	0.91	0.00
rs4798937	22850	ADNP2	18	75955525	1.23E-01	8121	6.87E-01	Up	0.40	11989	0.91	0.02
rs12818627	55766	H2AFJ	12	14814789	1.23E-01	8122	4.09E-01	Up	0.83	10046	0.91	0.04
rs11190870	10660	LBX1	10	102969197	1.23E-01	8123	3.90E-01	Down	0.86	9915	0.91	0.04
rs12709102	1535	CYBA	16	87239820	1.23E-01	8124	1.06E-02	Down	2.56	4553	0.91	0.20
rs12709102	4597	MVD	16	87239820	1.23E-01	8125	2.74E-01	Down	1.09	8986	0.91	0.06
rs1993120	8749	ADAM18	8	39641770	1.23E-01	8126	2.05E-02	Up	2.32	5096	0.91	0.17
rs888931	56961	SHD	19	4239555	1.23E-01	8127	7.73E-01	Down	0.29	12513	0.91	0.01
rs8111058	9710	KIAA0355	19	39480848	1.23E-01	8128	2.34E-01	Down	1.19	8604	0.91	0.06
rs2296169	253959	GARNL1	14	35260592	1.23E-01	8129	2.11E-02	Up	2.31	5123	0.91	0.17
rs12345155	26468	LHX6	9	122064804	1.23E-01	8130	3.05E-02	Up	2.16	5479	0.91	0.15
rs780689	55315	SLC29A3	10	72796833	1.23E-01	8131	4.04E-03	Down	2.87	3938	0.91	0.24
rs1838039	23001	WDFY3	4	85982880	1.23E-01	8132	9.66E-01	Up	0.04	13670	0.91	0.00
rs6259	482	ATP1B2	17	7477252	1.23E-01	8133	8.60E-06	Down	4.45	1920	0.91	0.51
rs6259	54407	SLC38A2	17	7477252	1.23E-01	8134	7.22E-02	Down	1.80	6470	0.91	0.11
rs6259	6462	SHBG	17	7477252	1.23E-01	8135	1.00E-01	Up	1.64	7003	0.91	0.10
rs6259	9513	FXR2	17	7477252	1.23E-01	8136	1.01E-01	Up	1.64	7021	0.91	0.10
rs6259	112483	SAT2	17	7477252	1.23E-01	8137	8.40E-01	Up	0.20	12904	0.91	0.01
rs178295	80764	THAP7	22	19675434	1.23E-01	8138	1.49E-01	Down	1.44	7667	0.91	0.08
rs1769621	112399	EGLN3	14	33493429	1.23E-01	8139	8.53E-01	Down	0.18	12975	0.91	0.01
rs472631	7474	WNT5A	3	55486715	1.23E-01	8140	2.05E-03	Up	3.08	3543	0.91	0.27
rs12162135	5717	PSMD11	17	27829908	1.23E-01	8141	1.17E-01	Up	1.57	7239	0.91	0.09
rs12162135	8851	CDK5R1	17	27829908	1.23E-01	8142	1.89E-01	Up	1.31	8133	0.91	0.07
rs392066	56954	NIT2	3	101540895	1.23E-01	8143	4.00E-08	Up	5.49	1204	0.91	0.74
rs12407294	10614	HEXIM1	1	101413453	1.23E-01	8144	1.02E-17	Down	8.57	279	0.91	1.70
rs10492146	121506	ERP27	12	14997289	1.23E-01	8145	7.41E-03	Up	2.68	4296	0.91	0.21
rs2279861	3177	SLC29A2	11	65889912	1.23E-01	8146	1.35E-01	Up	1.49	7515	0.91	0.09
rs2279861		B3GNT6	11	65889912	1.23E-01	8147	6.93E-01	Up	0.39	12023	0.91	0.02
rs12943866	146857	SLFN13	17	30796802	1.23E-01	8148	4.49E-03	Down	2.84	3993	0.91	0.23
rs12943866	55106	SLFN12	17	30796802	1.23E-01	8149	6.96E-01	Up	0.39	12040	0.91	0.02
rs16893170	10283	SDCCAG10	5	64155371	1.23E-01	8150	1.04E-01	Down	1.63	7060	0.91	0.10
rs12975066	51599	LSR	19	40415341	1.24E-01	8151	1.09E-05	Up	4.40	1965	0.91	0.50
rs3762404	79573	TTC13	1	227343240	1.24E-01	8152	5.28E-04	Up	3.47	2978	0.91	0.33
rs11260822	1969	EPHA2	1	16231117	1.24E-01	8153	5.33E-08	Down	5.44	1231	0.91	0.73
rs17713368	64089	SNX16	8	82931925	1.24E-01	8154	9.49E-04	Up	3.31	3198	0.91	0.30
rs2369797	23317	DNAJC13	3	133679459	1.24E-01	8155	4.57E-07	Down	5.04	1469	0.91	0.63
rs7255568	610	HCN2	19	556985	1.24E-01	8156	3.03E-01	Up	1.03	9218	0.91	0.05
rs12978642	26532	OR10H3	19	15696522	1.24E-01	8157	3.15E-01	Down	1.01	9317	0.91	0.05
rs12978642	26538	OR10H2	19	15696522	1.24E-01	8158	5.88E-01	Down	0.54	11358	0.91	0.02
rs10842036	9847	KIAA0528	12	22595698	1.24E-01	8159	7.44E-02	Up	1.78	6518	0.91	0.11
rs8067500	1366	CLDN7	17	7113355	1.24E-01	8160	1.54E-11	Up	6.74	655	0.91	1.08
rs8067500	23587	C17orf81	17	7113355	1.24E-01	8161	3.35E-04	Down	3.59	2816	0.91	0.35
rs8067500	23399	DULLARD	17	7113355	1.24E-01	8162	8.23E-01	Down	0.22	12807	0.91	0.01
rs11606738	219928	MRGPRF	11	68542061	1.24E-01	8163	7.65E-01	Down	0.30	12460	0.91	0.01
rs4309612	9938	ARHGAP25	2	68894868	1.24E-01	8164	1.47E-01	Down	1.45	7643	0.91	0.08
rs2605242	132299	OCIAD2	4	48751898	1.24E-01	8165	9.16E-09	Up	5.75	1045	0.91	0.80
rs6851362	84162	KIAA1109	4	123621051	1.24E-01	8166	2.30E-01	Down	1.20	8558	0.91	0.06
rs2414133	10017	BCL2L10	15	50199855	1.24E-01	8167	7.40E-02	Up	1.79	6508	0.91	0.11
rs16831846	23385	NCSTN	1	157132057	1.24E-01	8168	6.52E-06	Up	4.51	1863	0.91	0.52
rs16831846	1314	COPA	1	157132057	1.24E-01	8169	2.21E-05	Up	4.24	2105	0.91	0.47
rs16831846	4807	NHLH1	1	157132057	1.24E-01	8170	6.41E-01	Up	0.47	11717	0.91	0.02
rs6121926	26039	SS18L1	20	60168493	1.24E-01	8171	6.01E-06	Up	4.53	1845	0.91	0.52
rs7187428	2294	FOXF1	16	85097560	1.24E-01	8172	7.39E-14	Down	7.48	462	0.91	1.31
rs2285935	283120	H19	11	1971222	1.24E-01	8173	6.55E-02	Up	1.84	6351	0.91	0.12
rs852251	133690	CAPSL	5	35958219	1.24E-01	8174	5.49E-02	Down	1.92	6112	0.91	0.13
rs4704710	1160	CKMT2	5	80577047	1.24E-01	8175	3.03E-05	Down	4.17	2172	0.91	0.45
rs11867465	2123	EVI2A	17	26676582	1.24E-01	8176	8.86E-01	Down	0.14	13164	0.91	0.01

rs10492234	57102	C12orf4	12	4457860	1.24E-01	8177	8.98E-02	Up	1.70	6805	0.90	0.10
rs17076938	91392	ZNF502	3	44736733	1.24E-01	8178	5.36E-01	Down	0.62	10980	0.90	0.03
rs17076938	115560	ZNF501	3	44736733	1.24E-01	8179	7.42E-01	Up	0.33	12328	0.90	0.01
rs4809019	10994	ILVBL	19	15115571	1.24E-01	8180	1.32E-03	Down	3.21	3332	0.90	0.29
rs11222109	170689	ADAMTS15	11	129841446	1.25E-01	8181	5.56E-01	Down	0.59	11123	0.90	0.03
rs17702096	51377	UCHL5	1	189711904	1.25E-01	8182	9.12E-01	Up	0.11	13325	0.90	0.00
rs6680421	9670	IPO13	1	44078062	1.25E-01	8183	5.37E-01	Up	0.62	10988	0.90	0.03
rs920666	10563	CXCL13	4	78868382	1.25E-01	8184	8.67E-03	Down	2.62	4403	0.90	0.21
rs736066	122616	C14orf79	14	104506694	1.25E-01	8185	1.55E-03	Up	3.17	3397	0.90	0.28
rs10783580	5094	PCBP2	12	52133740	1.25E-01	8186	7.92E-02	Up	1.76	6619	0.90	0.11
rs10783580	54458	PRR13	12	52133740	1.25E-01	8187	8.74E-01	Up	0.16	13088	0.90	0.01
rs7587968	55825	PECR	2	216768538	1.25E-01	8188	5.54E-01	Down	0.59	11106	0.90	0.03
rs3811787	7350	UCP1	4	141848024	1.25E-01	8189	6.88E-01	Up	0.40	11994	0.90	0.02
rs4891559	55748	CNDP2	18	70321377	1.25E-01	8190	2.45E-01	Up	1.16	8714	0.90	0.06
rs12695689	55179	FAIM	3	139814989	1.25E-01	8191	2.11E-02	Up	2.31	5128	0.90	0.17
rs12695689	80321	CEP70	3	139814989	1.25E-01	8192	4.01E-01	Up	0.84	9987	0.90	0.04
rs2641889	6210	RPS15A	16	18714812	1.25E-01	8193	2.02E-06	Up	4.75	1671	0.90	0.57
rs854802	51168	MYO15A	17	18023829	1.25E-01	8194	3.59E-02	Down	2.10	5646	0.90	0.14
rs854802	54890	ALKBH5	17	18023829	1.25E-01	8195	1.10E-01	Down	1.60	7151	0.90	0.10
rs457420	8019	BRD3	9	133938815	1.25E-01	8196	7.23E-10	Up	6.16	873	0.90	0.91
rs2074206	7693	ZNF134	19	62807955	1.25E-01	8197	5.52E-02	Up	1.92	6116	0.90	0.13
rs2074206	348327	ZNF530	19	62807955	1.25E-01	8198	1.56E-01	Up	1.42	7757	0.90	0.08
rs2713203	29842	TFCP2L1	2	121712618	1.25E-01	8199	1.72E-11	Down	6.73	665	0.90	1.08
rs10129316	161145	C14orf83	14	67028853	1.26E-01	8200	7.91E-01	Down	0.26	12622	0.90	0.01
rs3824406	54586	C9orf11	9	27286705	1.26E-01	8201	8.34E-01	Down	0.21	12874	0.90	0.01
rs17717907	51666	ASB4	7	94776618	1.26E-01	8202	8.95E-01	Down	0.13	13216	0.90	0.00
rs8039242	7168	TPM1	15	61107561	1.26E-01	8203	6.67E-33	Down	11.95	50	0.90	3.22
rs11960458	309	ANXA6	5	150460713	1.26E-01	8204	1.25E-04	Down	3.84	2500	0.90	0.39
rs767896	4295	MLN	6	33899493	1.26E-01	8205	2.37E-02	Down	2.26	5228	0.90	0.16
rs1771612	51114	ZDHHC9		128700116	1.26E-01	8206	1.07E-19	Up	9.04	214	0.90	1.90
rs13011826	55041	PLEKHB2	2	131747626	1.26E-01	8207	7.36E-05	Up	3.96	2369	0.90	0.41
rs1347047	10941	UGT2A1	4	70702515	1.26E-01	8208	4.02E-01	Up	0.84	9996	0.90	0.04
rs738033	27439	CECR6	22	15974398	1.26E-01	8209	3.00E-02	Down	2.17	5466	0.90	0.15
rs2553318	10117	ENAM	4	71869455	1.26E-01	8210	1.51E-01	Down	1.43	7698	0.90	0.08
rs922521	54436	SH3TC1	4	8325881	1.26E-01	8211	2.72E-01	Down	1.10	8969	0.90	0.06
rs2000875	23220	DTX4	11	58738162	1.26E-01	8212	1.71E-05	Down	4.30	2062	0.90	0.48
rs707896	3010	HIST1H1T	6	26224403	1.26E-01	8213	2.29E-01	Up	1.20	8547	0.90	0.06
rs1302	11138	TBC1D8	2	101087516	1.26E-01	8214	3.77E-01	Down	0.88	9816	0.90	0.04
rs17483886	4931	NVL	1	220722244	1.27E-01	8215	1.13E-06	Up	4.87	1590	0.90	0.59
rs2910368	85509	MBD3L1	19	8802717	1.27E-01	8216	9.27E-01	Down	0.09	13412	0.90	0.00
rs12545272	137209	ZNF572	8	126042870	1.27E-01	8217	9.95E-02	Down	1.65	6990	0.90	0.10
rs301486	55664	CDC37L1	9	4670653	1.27E-01	8218	5.03E-01	Down	0.67	10740	0.90	0.03
rs6660106	57085	AGTRAP	1	11741117	1.27E-01	8219	3.87E-02	Down	2.07	5719	0.90	0.14
rs10138506	91612	CHURC1	14	64457996	1.27E-01	8220	1.54E-01	Down	1.43	7732	0.90	0.08
rs7529794	84879	MFSD2	1	40072463	1.27E-01	8221	7.57E-03	Down	2.67	4314	0.90	0.21
rs3813805	27293	SMPDL3B	1	27966066	1.27E-01	8222	1.17E-09	Up	6.08	904	0.90	0.89
rs3813805	55113	XKR8	1	27966066	1.27E-01	8223	1.09E-04	Up	3.87	2473	0.90	0.40
rs3762379	3738	KCNA3	1	110931386	1.27E-01	8224	9.51E-01	Up	0.06	13558	0.90	0.00
rs4946977	9841	ZBTB24	6	109882059	1.27E-01	8225	1.36E-02	Up	2.47	4734	0.90	0.19
rs4946977	285755	PPIL6	6	109882059	1.27E-01	8226	5.10E-02	Down	1.95	6015	0.90	0.13
rs2366366	129831	RBM45	2	178818720	1.27E-01	8227	7.22E-01	Up	0.36	12213	0.90	0.01
rs10766473	3939	LDHA	11	18358107	1.27E-01	8228	1.19E-01	Up	1.56	7281	0.90	0.09
rs17115203	57412	AS3MT	10	104629959	1.27E-01	8229	3.60E-01	Down	0.91	9681	0.90	0.04
rs4502283	2194	FASN	17	77638015	1.27E-01	8230	2.40E-09	Up	5.98	944	0.90	0.86
rs5935925	140456	ASB11		15061190	1.27E-01	8231	8.70E-01	Down	0.16	13065	0.90	0.01
rs17353138	4159	MC3R	20	54242712	1.27E-01	8232	3.33E-01	Down	0.97	9468	0.90	0.05
rs2857998	3050	HBZ	16	125123	1.27E-01	8233	5.25E-01	Down	0.64	10906	0.90	0.03
rs11128111	10550	ARL6IP5	3	69228322	1.27E-01	8234	2.54E-02	Down	2.24	5299	0.90	0.16
rs400345	58189	WFDC1	16	82885995	1.27E-01	8235	9.11E-14	Down	7.45	471	0.90	1.30
rs7721068	3094	HINT1	5	130542971	1.27E-01	8236	3.58E-07	Up	5.09	1439	0.90	0.64
rs7464066	2738	GLI4	8	144401827	1.27E-01	8237	3.82E-01	Down	0.87	9853	0.90	0.04
rs10188643	3398	ID2	2	8761023	1.27E-01	8238	3.12E-04	Down	3.60	2790	0.89	0.35
rs11170881	53831	GPR84	12	53035101	1.27E-01	8239	8.20E-01	Down	0.23	12792	0.89	0.01
rs4963136	115399	LRRC56	11	539119	1.27E-01	8240	3.23E-02	Up	2.14	5535	0.89	0.15
rs4963136	256329	C11orf35	11	539119	1.27E-01	8241	5.42E-01	Down	0.61	11034	0.89	0.03
rs4963136	3265	HRAS	11	539119	1.27E-01	8242	7.85E-01	Up	0.27	12580	0.89	0.01
rs2723154	27178	IL1F7	2	113374306	1.27E-01	8243	6.90E-01	Down	0.40	12009	0.89	0.02
rs1045553	84519	ACRBP	12	6617556	1.27E-01	8244	2.47E-01	Down	1.16	8739	0.89	0.06
rs1264379	2719	GPC3		132791286	1.27E-01	8245	5.65E-01	Up	0.58	11199	0.89	0.02
rs13778	23593	HEBP2	6	138776005	1.28E-01	8246	6.51E-14	Up	7.49	460	0.89	1.32
rs6706062	57669	EPB41L5	2	120650328	1.28E-01	8247	5.60E-02	Up	1.91	6135	0.89	0.13
rs2056918	9452	ITM2A		78435323	1.28E-01	8248	4.93E-02	Down	1.97	5977	0.89	0.13
rs949538	7299	TYR	11	88542052	1.28E-01	8249	2.27E-01	Up	1.21	8527	0.89	0.06
rs6032205	10406	WFDC2	20	43516213	1.28E-01	8250	3.02E-17	Down	8.45	296	0.89	1.65
rs9568203	83852	SETDB2	13	48906936	1.28E-01	8251	3.78E-01	Up	0.88	9827	0.89	0.04
rs1674154	2527	FUT5	19	5832194	1.28E-01	8252	3.97E-01	Down	0.85	9963	0.89	0.04
rs4359651	55258	THNSL2	2	88304102	1.28E-01	8253	9.70E-01	Up	0.04	13699	0.89	0.00
rs2298375	4320	MMP11	22	22431002	1.28E-01	8254	8.27E-02	Up	1.73	6681	0.89	0.11
rs2298375	29802	VPREB3	22	22431002	1.28E-01	8255	2.97E-01	Down	1.04	9176	0.89	0.05
rs2298375	7621	ZNF70	22	22431002	1.28E-01	8256	4.31E-01	Up	0.79	10203	0.89	0.04
rs2298375	150248	C22orf15	22	22431002	1.28E-01	8257	6.08E-01	Down	0.51	11486	0.89	0.02

rs281380	54922	RASIP1	19	53906282	1.28E-01	8258	3.51E-02	Down	2.11	5625	0.89	0.15
rs281380	2524	FUT2	19	53906282	1.28E-01	8259	8.71E-01	Down	0.16	13072	0.89	0.01
rs12170895	6634	SNRPD3	22	23267848	1.28E-01	8260	1.43E-06	Up	4.82	1621	0.89	0.58
rs12170895	83606	C22orf13	22	23267848	1.28E-01	8261	4.26E-01	Down	0.80	10161	0.89	0.04
rs391300	63826	SRR	17	2163008	1.28E-01	8262	4.72E-01	Down	0.72	10512	0.89	0.03
rs506028	6712	SPTBN2	11	66225045	1.28E-01	8263	7.74E-07	Up	4.94	1535	0.89	0.61
rs2268089	8894	EIF2S2	20	32130959	1.28E-01	8264	3.67E-03	Up	2.91	3883	0.89	0.24
rs11647067	57478	USP31	16	22998479	1.28E-01	8265	6.31E-01	Up	0.48	11645	0.89	0.02
rs8187889	216	ALDH1A1	9	72789555	1.28E-01	8266	4.59E-01	Up	0.74	10419	0.89	0.03
rs730554	5300	PIN1	19	9822546	1.28E-01	8267	1.05E-01	Up	1.62	7078	0.89	0.10
rs6981400	91782	CHMP7	8	23160557	1.28E-01	8268	6.92E-01	Down	0.40	12022	0.89	0.02
rs9927989	25823	TPSG1	16	1237146	1.28E-01	8269	7.04E-01	Up	0.38	12098	0.89	0.02
rs9927989	64499	TPSB2	16	1237146	1.28E-01	8270	7.73E-01	Down	0.29	12516	0.89	0.01
rs9376137	51805	COQ3	6	99948777	1.28E-01	8271	4.33E-01	Up	0.78	10213	0.89	0.04
rs2188015	137492	VPS37A	8	17182938	1.28E-01	8272	1.09E-20	Down	9.33	181	0.89	2.00
rs6603134	6370	CCL25	19	8012521	1.28E-01	8273	3.03E-01	Down	1.03	9208	0.89	0.05
rs2575680	83478	ARHGAP24	4	87272389	1.28E-01	8274	6.22E-15	Down	7.80	395	0.89	1.42
rs7741552	171558	PTCRA	6	42981801	1.28E-01	8275	3.40E-01	Down	0.95	9525	0.89	0.05
rs12194317	154075	SAMD3	6	130585380	1.29E-01	8276	5.52E-01	Up	0.60	11090	0.89	0.03
rs7725097	10769	PLK2	5	57805292	1.29E-01	8277	4.36E-03	Down	2.85	3982	0.89	0.24
rs10416031	2879	GPX4	19	1046151	1.29E-01	8278	2.01E-01	Up	1.28	8267	0.89	0.07
rs10416031	5434	POLR2E	19	1046151	1.29E-01	8279	5.57E-01	Down	0.59	11127	0.89	0.03
rs312462	124944	C17orf49	17	6854376	1.29E-01	8280	4.25E-01	Up	0.80	10159	0.89	0.04
rs1863704	9654	TTLL4	2	219388180	1.29E-01	8281	5.48E-02	Up	1.92	6108	0.89	0.13
rs11012	9842	PLEKHM1	17	40869224	1.29E-01	8282	7.08E-03	Down	2.69	4267	0.89	0.21
rs1796520	11119	BTN3A1	6	26518779	1.29E-01	8283	2.42E-02	Down	2.25	5257	0.89	0.16
rs1796520	54718	BTN2A3	6	26518779	1.29E-01	8284	2.90E-01	Down	1.06	9116	0.89	0.05
rs7604334	11037	STON1	2	48744084	1.29E-01	8285	2.97E-05	Down	4.18	2168	0.89	0.45
rs2729722	4065	LY75	2	160491443	1.29E-01	8286	1.82E-01	Down	1.33	8051	0.89	0.07
rs2287799	343	AQP8	16	25143526	1.29E-01	8287	3.01E-02	Up	2.17	5469	0.89	0.15
rs11955226	51289	RXFP3	5	33959363	1.29E-01	8288	5.94E-01	Down	0.53	11395	0.89	0.02
rs2304681	11117	EMILIN1	2	27226903	1.29E-01	8289	8.98E-07	Down	4.91	1565	0.89	0.60
rs2304681	3795	KHK	2	27226903	1.29E-01	8290	8.79E-01	Up	0.15	13119	0.89	0.01
rs15394	1515	CTSL2	9	96874680	1.29E-01	8291	8.60E-01	Up	0.18	13012	0.89	0.01
rs691141	84376	HOOK3	5	176255904	1.29E-01	8292	7.24E-02	Up	1.80	6474	0.89	0.11
rs691141	3101	HK3	5	176255904	1.29E-01	8293	6.55E-01	Up	0.45	11796	0.89	0.02
rs7946	51655	RASD1	17	17350285	1.29E-01	8294	3.26E-02	Up	2.14	5545	0.89	0.15
rs3761562	9248	GPR50		150013596	1.29E-01	8295	1.34E-01	Up	1.50	7498	0.89	0.09
rs1406076	9957	HS3ST1	4	11079973	1.29E-01	8296	6.59E-01	Down	0.44	11824	0.89	0.02
rs10494829	23046	KIF21B	1	197669686	1.29E-01	8297	2.21E-01	Up	1.22	8475	0.89	0.07
rs17134769	50640	PNPLA8	7	107705307	1.29E-01	8298	3.20E-02	Down	2.14	5529	0.89	0.15
rs6962380	23161	SNX13	7	17747143	1.29E-01	8299	4.79E-02	Up	1.98	5949	0.89	0.13
rs2383177	3456	IFNB1	9	21058841	1.29E-01	8300	3.77E-01	Down	0.88	9818	0.89	0.04
rs823673	4802	NFYC	1	40909612	1.29E-01	8301	7.97E-03	Down	2.65	4340	0.89	0.21
rs823673	9132	KCNQ4	1	40909612	1.29E-01	8302	2.01E-01	Down	1.28	8266	0.89	0.07
rs13011502	2697	GJA1	2	19471609	1.29E-01	8303	5.16E-30	Down	11.38	64	0.89	2.93
rs446641	23446	SLC44A1	9	105067752	1.30E-01	8304	6.57E-10	Up	6.17	868	0.89	0.92
rs6060930	22974	TPX2	20	29811493	1.30E-01	8305	1.87E-08	Up	5.62	1119	0.89	0.77
rs6974500	340277	C7orf46	7	23517789	1.30E-01	8306	7.32E-13	Up	7.16	548	0.89	1.21
rs1264013	158787	RIBC1		53340643	1.30E-01	8307	7.31E-02	Up	1.79	6487	0.89	0.11
rs2250402	6727	SRP14	15	38109844	1.30E-01	8308	4.89E-02	Down	1.97	5968	0.89	0.13
rs9892996	60386	SLC25A19	17	70814812	1.30E-01	8309	2.86E-01	Up	1.07	9079	0.89	0.05
rs606970	23650	TRIM29	11	119495102	1.30E-01	8310	8.68E-24	Down	10.06	127	0.89	2.31
rs6441867	57456	KIAA1143	3	44785111	1.30E-01	8311	9.10E-01	Up	0.11	13311	0.89	0.00
rs279559	63924	CIDEC	3	9907112	1.30E-01	8312	6.30E-02	Down	1.86	6300	0.89	0.12
rs279559	132014	IL17RE	3	9907112	1.30E-01	8313	1.01E-01	Up	1.64	7008	0.89	0.10
rs279559	84522	JAGN1	3	9907112	1.30E-01	8314	2.29E-01	Up	1.20	8546	0.89	0.06
rs17695477	1662	DDX10	11	108035127	1.30E-01	8315	9.51E-01	Up	0.06	13564	0.89	0.00
rs7064056	57526	PCDH19		99373580	1.30E-01	8316	3.51E-01	Up	0.93	9596	0.89	0.05
rs1787663	6357	CCL13	11	65051406	1.30E-01	8317	3.34E-01	Down	0.97	9477	0.89	0.05
rs1787663	57410	SCYL1	11	65051406	1.30E-01	8318	5.23E-01	Up	0.64	10881	0.89	0.03
rs805512	5744	PTHLH	12	28010114	1.30E-01	8319	1.12E-02	Up	2.54	4582	0.89	0.20
rs16894387	54210	TREM1	6	41348149	1.30E-01	8320	4.19E-01	Down	0.81	10110	0.89	0.04
rs1062108	58528	RRAGD	6	90132078	1.30E-01	8321	7.52E-01	Up	0.32	12384	0.89	0.01
rs2176473	8548	BLZF1	1	166063460	1.30E-01	8322	4.29E-01	Down	0.79	10189	0.89	0.04
rs17680667	22876	INPP5F	10	121540796	1.30E-01	8323	7.26E-11	Down	6.52	736	0.89	1.01
rs4345834	11000	SLC27A3	1	150556390	1.30E-01	8324	4.80E-06	Down	4.57	1805	0.89	0.53
rs1053817	163033	ZNF579	19	60781031	1.30E-01	8325	6.66E-02	Down	1.83	6371	0.89	0.12
rs1390376	5732	PTGER2	14	51848225	1.30E-01	8326	1.67E-03	Down	3.14	3440	0.89	0.28
rs3863641	2166	FAAH	1	46555236	1.30E-01	8327	2.33E-12	Up	7.02	588	0.88	1.16
rs3863641	79152	FA2H	1	46555236	1.30E-01	8328	3.56E-02	Up	2.10	5634	0.88	0.14
rs12453262	54478	FAM64A	17	6272560	1.30E-01	8329	2.54E-03	Up	3.02	3697	0.88	0.26
rs8057184	55815	TSNAXIP1	16	66384748	1.31E-01	8330	5.44E-01	Down	0.61	11043	0.88	0.03
rs6576599	54551	MAGEL2	15	21457592	1.31E-01	8331	4.43E-01	Down	0.77	10290	0.88	0.04
rs963333	79705	LRRK1	15	99375164	1.31E-01	8332	1.57E-01	Down	1.41	7769	0.88	0.08
rs7657036	1040	CDS1	4	85844120	1.31E-01	8333	5.48E-04	Up	3.46	2991	0.88	0.33
rs3738154	5929	RBBP5	1	201795606	1.31E-01	8334	1.86E-01	Down	1.32	8103	0.88	0.07
rs1020593	266812	NAP1L5	4	89969622	1.31E-01	8335	4.38E-08	Down	5.47	1210	0.88	0.74
rs3745746	64100	ELSPBP1	19	53229397	1.31E-01	8336	5.07E-01	Up	0.66	10764	0.88	0.03
rs502747	6450	SH3BGR	21	39794258	1.31E-01	8337	5.38E-05	Down	4.04	2304	0.88	0.43
rs12205310	29777	ABT1	6	26713843	1.31E-01	8338	1.44E-02	Up	2.45	4783	0.88	0.18

rs11204726	1520	CTSS	1	147556565	1.31E-01	8339	3.66E-04	Up	3.56	2852	0.88	0.34
rs2007683	5603	MAPK13	6	36209540	1.31E-01	8340	1.87E-01	Up	1.32	8113	0.88	0.07
rs4951471	28982	FLVCR1	1	209463799	1.31E-01	8341	7.90E-02	Up	1.76	6615	0.88	0.11
rs303212	24138	IFIT5	10	91151335	1.31E-01	8342	5.26E-06	Down	4.55	1827	0.88	0.53
rs4751107	4255	MGMT	10	131317651	1.31E-01	8343	3.41E-02	Up	2.12	5596	0.88	0.15
rs2769270	5692	PSMB4	1	148168931	1.31E-01	8344	9.79E-01	Down	0.03	13763	0.88	0.00
rs1878326	145864	HAPLN3	15	87251591	1.31E-01	8345	6.09E-02	Up	1.87	6252	0.88	0.12
rs17052201	23166	STAB1	3	52505827	1.31E-01	8346	3.59E-03	Down	2.91	3872	0.88	0.24
rs17052201	11188	NISCH	3	52505827	1.31E-01	8347	4.46E-01	Down	0.76	10320	0.88	0.04
rs6751655	54454	ATAD2B	2	24067129	1.31E-01	8348	9.92E-01	Up	0.01	13862	0.88	0.00
rs2981270	56704	JPH1	8	75408680	1.31E-01	8349	5.14E-02	Up	1.95	6021	0.88	0.13
rs2981270	54332	GDAP1	8	75408680	1.31E-01	8350	2.79E-01	Up	1.08	9027	0.88	0.06
rs7573270	3694	ITGB6	2	160775040	1.31E-01	8351	4.86E-05	Down	4.06	2275	0.88	0.43
rs362510	10537	UBD	6	29648724	1.31E-01	8352	4.84E-03	Up	2.82	4039	0.88	0.23
rs1608	79174	CRELD2	22	48641804	1.31E-01	8353	1.34E-01	Up	1.50	7492	0.88	0.09
rs1608	79087	ALG12	22	48641804	1.31E-01	8354	1.95E-01	Down	1.30	8201	0.88	0.07
rs1608	415116	PIM3	22	48641804	1.31E-01	8355	9.03E-01	Down	0.12	13268	0.88	0.00
rs6969383	155465	AGR3	7	16699513	1.31E-01	8356	5.26E-02	Up	1.94	6053	0.88	0.13
rs4761243	10818	FRS2	12	68235667	1.31E-01	8357	6.45E-01	Up	0.46	11748	0.88	0.02
rs714510	3614	IMPDH1	7	127625249	1.31E-01	8358	3.90E-05	Up	4.11	2223	0.88	0.44
rs7844675	63978	PRDM14	8	71124068	1.31E-01	8359	2.18E-01	Down	1.23	8440	0.88	0.07
rs3776921	6902	TBCA	5	77023468	1.31E-01	8360	2.45E-01	Up	1.16	8710	0.88	0.06
rs816073	11099	PTPN21	14	88027035	1.31E-01	8361	2.07E-07	Down	5.19	1376	0.88	0.67
rs845761	55812	SPATA7	14	87928452	1.31E-01	8362	7.66E-04	Up	3.36	3118	0.88	0.31
rs4694636	3576	IL8	4	74963844	1.32E-01	8363	8.90E-01	Down	0.14	13186	0.88	0.01
rs7560764	9448	MAP4K4	2	101970399	1.32E-01	8364	2.16E-05	Up	4.25	2102	0.88	0.47
rs3178292	6187	RPS2	16	1955122	1.32E-01	8365	1.76E-08	Up	5.64	1104	0.88	0.78
rs3178292	4716	NDUFB10	16	1955122	1.32E-01	8366	5.98E-05	Up	4.01	2332	0.88	0.42
rs3178292	6123	RPL3L	16	1955122	1.32E-01	8367	1.71E-01	Down	1.37	7926	0.88	0.08
rs3178292	146310	RNF151	16	1955122	1.32E-01	8368	2.06E-01	Down	1.27	8315	0.88	0.07
rs3178292	2671	GFER	16	1955122	1.32E-01	8369	3.78E-01	Up	0.88	9826	0.88	0.04
rs3178292	10607	TBL3	16	1955122	1.32E-01	8370	7.57E-01	Down	0.31	12424	0.88	0.01
rs6838700	256309	CCDC110	4	186747975	1.32E-01	8371	4.65E-05	Up	4.07	2264	0.88	0.43
rs2271097	55500	ETNK1	12	22728549	1.32E-01	8372	1.01E-04	Up	3.89	2455	0.88	0.40
rs7772160	7738	ZNF184	6	27520365	1.32E-01	8373	1.03E-05	Up	4.41	1954	0.88	0.50
rs2271992	23552	CCRK	9	87814032	1.32E-01	8374	3.24E-01	Down	0.99	9395	0.88	0.05
rs988166	116151	C20orf108	20	54354265	1.32E-01	8375	1.60E-01	Up	1.41	7815	0.88	0.08
rs6664	9435	CHST2	3	144324438	1.32E-01	8376	1.01E-12	Down	7.13	557	0.88	1.20
rs3213767	10463	SLC30A9	4	41866135	1.32E-01	8377	3.79E-08	Up	5.55	1160	0.88	0.74
rs17347617	91775	FAM55C	3	103029537	1.32E-01	8378	2.49E-02	Down	2.24	5282	0.88	0.16
rs3743926	84229	CCDC135	16	56293023	1.32E-01	8379	6.01E-01	Down	0.52	11437	0.88	0.02
rs2891602	9424	KCNK6	19	43484485	1.32E-01	8380	6.07E-01	Up	0.51	11483	0.88	0.02
rs2891602	64073	C19orf33	19	43484485	1.32E-01	8381	8.17E-01	Up	0.23	12775	0.88	0.01
rs4278789	10140	TOB1	17	46306334	1.32E-01	8382	8.64E-03	Up	2.63	4400	0.88	0.21
rs220722	4142	MAS1	6	160297101	1.32E-01	8383	3.26E-02	Down	2.14	5547	0.88	0.15
rs611908	408	ARRB1	11	74694735	1.32E-01	8384	4.63E-04	Down	3.50	2929	0.88	0.33
rs11820303	26338	OR5L2	11	55352889	1.32E-01	8385	7.61E-01	Down	0.30	12448	0.88	0.01
rs2281870	922	CD5L	1	154624658	1.32E-01	8386	2.69E-02	Up	2.21	5359	0.88	0.16
rs12619064	55133	SRBD1	2	45569955	1.32E-01	8387	3.48E-03	Up	2.92	3857	0.88	0.25
rs12491981	1475	CSTA	3	123540563	1.32E-01	8388	1.79E-12	Down	7.05	573	0.88	1.17
rs2614226	25914	RTTN	18	66031485	1.33E-01	8389	2.21E-01	Down	1.22	8470	0.88	0.07
rs3766550	134	ADORA1	1	199873346	1.33E-01	8390	4.16E-01	Down	0.81	10091	0.88	0.04
rs17302632	51278	IER5	1	177771185	1.33E-01	8391	5.67E-04	Down	3.45	3006	0.88	0.32
rs11098602	79931	TNIP3	4	122410246	1.33E-01	8392	5.98E-01	Up	0.53	11417	0.88	0.02
rs1163073	9118	INA	10	105012924	1.33E-01	8393	1.92E-01	Down	1.30	8171	0.88	0.07
rs482388	23118	MAP3K7IP2	6	149674215	1.33E-01	8394	1.07E-01	Down	1.61	7102	0.88	0.10
rs2293067	8578	SCARF1	17	1496856	1.33E-01	8395	6.08E-04	Up	3.43	3031	0.88	0.32
rs2293067	83547	RILP	17	1496856	1.33E-01	8396	9.57E-01	Down	0.05	13602	0.88	0.00
rs2273885	81550	TDRD3	13	60037723	1.33E-01	8397	3.88E-01	Down	0.86	9898	0.88	0.04
rs1291211	8771	TNFRSF6B	20	61808066	1.33E-01	8398	1.24E-01	Down	1.54	7363	0.88	0.09
rs1291211	10139	ARFRP1	20	61808066	1.33E-01	8399	8.61E-01	Up	0.18	13014	0.88	0.01
rs3827494	6997	TDGF1	3	46577235	1.33E-01	8400	1.91E-01	Up	1.31	8164	0.88	0.07
rs11246386	4588	MUC6	11	1034938	1.33E-01	8401	4.22E-01	Up	0.80	10136	0.88	0.04
rs4516970	9589	WTAP	6	160108098	1.33E-01	8402	9.25E-01	Down	0.09	13398	0.88	0.00
rs12532878	7036	TFR2	7	99856905	1.33E-01	8403	4.13E-01	Up	0.82	10072	0.88	0.04
rs2745523	656	BMP8B	1	39937461	1.33E-01	8404	5.84E-01	Up	0.55	11336	0.88	0.02
rs2270941	27294	DHDH	19	54130175	1.33E-01	8405	8.05E-01	Up	0.25	12699	0.88	0.01
rs4786416	146434	ZNF597	16	3438952	1.33E-01	8406	6.39E-01	Down	0.47	11695	0.88	0.02
rs2181139	219	ALDH1B1	9	38364977	1.33E-01	8407	2.48E-06	Down	4.71	1701	0.88	0.56
rs885975	64976	MRPL40	22	17805026	1.33E-01	8408	1.93E-06	Up	4.76	1663	0.88	0.57
rs885975	7290	HIRA	22	17805026	1.33E-01	8409	1.56E-02	Up	2.42	4853	0.88	0.18
rs11187969	23232	TBC1D12	10	96221159	1.33E-01	8410	9.84E-02	Up	1.65	6972	0.88	0.10
rs669379	3852	KRT5	12	51213665	1.33E-01	8411	5.48E-05	Down	4.03	2310	0.88	0.43
rs1044997	64901	RANBP17	5	170658594	1.33E-01	8412	5.19E-02	Up	1.94	6037	0.88	0.13
rs1044997	30012	TLX3	5	170658594	1.33E-01	8413	4.57E-01	Down	0.74	10398	0.88	0.03
rs4760600	51303	FKBP11	12	47601261	1.33E-01	8414	7.38E-07	Up	4.95	1527	0.87	0.61
rs4760600	85478	CCDC65	12	47601261	1.33E-01	8415	7.50E-01	Down	0.32	12377	0.87	0.01
rs723654	222166	C7orf41	7	29984462	1.34E-01	8416	4.71E-10	Down	6.23	849	0.87	0.93
rs10506870	84190	C12orf26	12	81253568	1.34E-01	8417	2.14E-03	Up	3.07	3575	0.87	0.27
rs10506870	29080	CCDC59	12	81253568	1.34E-01	8418	7.56E-02	Down	1.78	6541	0.87	0.11
rs9937539	79905	TMC7	16	18941493	1.34E-01	8419	3.29E-02	Down	2.13	5558	0.87	0.15

rs12457980	252884	ZNF396	18	31205020	1.34E-01	8420	3.00E-01	Up	1.04	9191	0.87	0.05
rs7755143	221476	PI16	6	37041148	1.34E-01	8421	2.47E-04	Down	3.67	2706	0.87	0.36
rs12484030	6122	RPL3	22	38037436	1.34E-01	8422	1.34E-01	Up	1.50	7488	0.87	0.09
rs7396	11054	OGFR	20	60901948	1.34E-01	8423	2.57E-01	Down	1.13	8838	0.87	0.06
rs7396	55257	C20orf20	20	60901948	1.34E-01	8424	3.39E-01	Up	0.96	9513	0.87	0.05
rs7396	1299	COL9A3	20	60901948	1.34E-01	8425	5.47E-01	Down	0.60	11059	0.87	0.03
rs12840724	10075	HUWE1		53503195	1.34E-01	8426	8.57E-01	Up	0.18	12997	0.87	0.01
rs1493521	1191	CLU	8	27539186	1.34E-01	8427	6.20E-24	Down	10.09	125	0.87	2.32
rs1493521	51435	SCARA3	8	27539186	1.34E-01	8428	1.21E-05	Down	4.38	1993	0.87	0.49
rs1105451	3981	LIG4	13	107651324	1.34E-01	8429	1.04E-02	Down	2.56	4540	0.87	0.20
rs12932521	51693	TRAPPC2L	16	87441736	1.34E-01	8430	9.38E-01	Down	0.08	13479	0.87	0.00
rs12383252	4674	NAP1L2		72230581	1.34E-01	8431	3.76E-01	Up	0.89	9810	0.87	0.04
rs7563350	150696	PROM2	2	95353092	1.34E-01	8432	4.10E-04	Down	3.53	2893	0.87	0.34
rs4783554	55512	SMPD3	16	66941511	1.34E-01	8433	6.10E-01	Down	0.51	11507	0.87	0.02
rs4945787	256380	SCML4	6	108156915	1.34E-01	8434	8.43E-01	Down	0.20	12921	0.87	0.01
rs11639620	865	CBFB	16	65631053	1.34E-01	8435	6.62E-03	Up	2.72	4222	0.87	0.22
rs11869485	443	ASPA	17	3347986	1.35E-01	8436	8.63E-03	Down	2.63	4398	0.87	0.21
rs10096810	4956	ODF1	8	103614612	1.35E-01	8437	5.82E-01	Down	0.55	11329	0.87	0.02
rs2611757	51701	NLK	17	23542848	1.35E-01	8438	5.58E-15	Up	7.81	391	0.87	1.43
rs2611757	2821	GPI	17	23542848	1.35E-01	8439	1.18E-09	Up	6.08	905	0.87	0.89
rs17171525	26249	KLHL3	5	136989465	1.35E-01	8440	5.35E-01	Down	0.62	10976	0.87	0.03
rs6485451	51144	HSD17B12	11	43699878	1.35E-01	8441	6.98E-01	Up	0.39	12057	0.87	0.02
rs6122779	57169	ZNFX1	20	47326421	1.35E-01	8442	4.82E-01	Down	0.70	10584	0.87	0.03
rs17311679	25888	ZNF473	19	55213143	1.35E-01	8443	4.39E-02	Up	2.01	5850	0.87	0.14
rs17311679	51231	VRK3	19	55213143	1.35E-01	8444	4.80E-01	Up	0.71	10575	0.87	0.03
rs6759180	6241	RRM2	2	10217161	1.35E-01	8445	2.14E-04	Up	3.70	2653	0.87	0.37
rs2471320	80853	JHDM1D	7	139368304	1.35E-01	8446	2.84E-04	Up	3.63	2759	0.87	0.35
rs10405246	4099	MAG	19	40460789	1.35E-01	8447	1.28E-01	Down	1.52	7414	0.87	0.09
rs10405246	7392	USF2	19	40460789	1.35E-01	8448	1.80E-01	Up	1.34	8030	0.87	0.07
rs10405246	57817	HAMP	19	40460789	1.35E-01	8449	3.71E-01	Down	0.89	9779	0.87	0.04
rs10405246	2068	ERCC2	19	40460789	1.35E-01	8450	9.14E-01	Up	0.11	13341	0.87	0.00
rs6536991	255520	ELMOD2	4	141839186	1.35E-01	8451	2.09E-02	Up	2.31	5113	0.87	0.17
rs826682	3987	LIMS1	2	108690906	1.35E-01	8452	1.50E-08	Up	5.69	1074	0.87	0.78
rs11658299	57602	USP36	17	74337317	1.35E-01	8453	8.94E-02	Down	1.70	6793	0.87	0.10
rs8018220	145270	PRIMA1	14	93293352	1.35E-01	8454	9.06E-06	Down	4.44	1926	0.87	0.50
rs7857137	375743	PTAR1	9	69558281	1.35E-01	8455	5.36E-04	Up	3.46	2983	0.87	0.33
rs5962323	139221	MUM1L1		105264898	1.35E-01	8456	6.36E-02	Up	1.85	6313	0.87	0.12
rs537455	90427	BMF	15	38195116	1.35E-01	8457	4.79E-01	Down	0.71	10567	0.87	0.03
rs10867025	1670	DEFA5	8	6896851	1.35E-01	8458	7.83E-01	Down	0.28	12566	0.87	0.01
rs9442235	1188	CLCNKB	1	16138663	1.35E-01	8459	5.84E-02	Down	1.89	6203	0.87	0.12
rs8006357	10278	EFS	14	22923469	1.35E-01	8460	5.74E-37	Down	12.70	29	0.87	3.62
rs4878811	347252	IGFBPL1	9	38398525	1.35E-01	8461	9.92E-01	Down	0.01	13858	0.87	0.00
rs10194053	84128	WDR75	2	190154646	1.35E-01	8462	4.19E-09	Up	5.87	999	0.87	0.84
rs10987952	79695	GALNT12	9	98687580	1.35E-01	8463	4.97E-03	Up	2.81	4065	0.87	0.23
rs7047760	7633	ZNF79	9	127254650	1.35E-01	8464	1.04E-01	Up	1.62	7067	0.87	0.10
rs209914	1298	COL9A2	1	40444322	1.36E-01	8465	1.88E-21	Up	9.52	171	0.87	2.07
rs3817666	30851	TAX1BP3	17	3515150	1.36E-01	8466	1.69E-08	Down	5.64	1101	0.87	0.78
rs3817666	5026	P2RX5	17	3515150	1.36E-01	8467	4.79E-03	Up	2.82	4031	0.87	0.23
rs3817666	83460	TMEM93	17	3515150	1.36E-01	8468	3.14E-01	Up	1.01	9305	0.87	0.05
rs4147719	4719	NDUFS1	2	206832182	1.36E-01	8469	1.34E-03	Up	3.21	3337	0.87	0.29
rs4147719	1933	EEF1B2	2	206832182	1.36E-01	8470	6.20E-01	Up	0.50	11580	0.87	0.02
rs7155841	5494	PPM1A	14	59797784	1.36E-01	8471	2.40E-09	Up	5.89	987	0.87	0.86
rs10793656	9557	CHD1L	1	143976088	1.36E-01	8472	7.66E-05	Up	3.95	2379	0.87	0.41
rs594144	10825	NEU3	11	74404476	1.36E-01	8473	3.74E-01	Down	0.89	9801	0.87	0.04
rs1350666	2069	EREG	4	75589625	1.36E-01	8474	2.21E-01	Up	1.22	8477	0.87	0.07
rs1565356	2914	GRM4	6	34154043	1.36E-01	8475	1.60E-02	Down	2.41	4887	0.87	0.18
rs7590991	26275	HIBCH	2	190938759	1.36E-01	8476	8.45E-03	Up	2.63	4380	0.87	0.21
rs7193955	94160	ABCC12	16	46680083	1.36E-01	8477	8.39E-01	Up	0.20	12898	0.87	0.01
rs10894199	11095	ADAMTS8	11	129814089	1.36E-01	8478	1.43E-04	Down	3.80	2538	0.87	0.38
rs1046088	2033	EP300	22	39898883	1.36E-01	8479	7.31E-01	Up	0.34	12260	0.87	0.01
rs10799340	64746	ACBD3	1	222682002	1.36E-01	8480	1.49E-05	Up	4.33	2035	0.87	0.48
rs6424377	10628	TXNIP	1	142917444	1.36E-01	8481	4.62E-06	Down	4.58	1801	0.87	0.53
rs4587804	55089	SLC38A4	12	45503781	1.37E-01	8482	6.04E-01	Up	0.52	11459	0.86	0.02
rs2273828	39	ACAT2	6	160177460	1.37E-01	8483	1.97E-01	Up	1.29	8220	0.86	0.07
rs2273828	8435	SOAT2	6	160177460	1.37E-01	8484	6.64E-01	Up	0.43	11845	0.86	0.02
rs11749558	6534	SLC6A7	5	149563368	1.37E-01	8485	8.50E-01	Down	0.19	12959	0.86	0.01
rs11658900	55731	C17orf63	17	24119611	1.37E-01	8486	5.38E-14	Up	7.41	479	0.86	1.33
rs7322754	10240	MRPS31	13	40220977	1.37E-01	8487	4.67E-01	Down	0.73	10482	0.86	0.03
rs11564100	57171	DOLPP1	9	128914365	1.37E-01	8488	3.77E-01	Up	0.88	9814	0.86	0.04
rs3931036	25839	COG4	16	69105798	1.37E-01	8489	6.38E-01	Down	0.47	11691	0.86	0.02
rs2101248	6514	SLC2A2	3	172245828	1.37E-01	8490	5.07E-01	Up	0.66	10763	0.86	0.03
rs908590	79884	MAP9	4	156669001	1.37E-01	8491	4.14E-20	Up	9.20	192	0.86	1.94
rs17664478	349075	ZNF713	7	55766281	1.37E-01	8492	9.07E-01	Down	0.12	13291	0.86	0.00
rs691	3653	IPW	15	22915888	1.37E-01	8493	3.19E-02	Up	2.15	5521	0.86	0.15
rs197480	3097	HIVEP2	6	143112198	1.37E-01	8494	8.65E-02	Up	1.71	6747	0.86	0.11
rs357728	10199	MPHOSPH10	2	71293639	1.37E-01	8495	1.32E-04	Up	3.82	2516	0.86	0.39
rs9348689	10590	SCGN	6	25792632	1.37E-01	8496	9.61E-02	Up	1.66	6927	0.86	0.10
rs10046843	2592	GALT	9	34621159	1.37E-01	8497	1.08E-02	Down	2.55	4562	0.86	0.20
rs490998	89	ACTN3	11	66075548	1.37E-01	8498	2.19E-02	Down	2.29	5155	0.86	0.17
rs10419232	3727	JUND	19	18265148	1.37E-01	8499	9.60E-07	Up	4.90	1571	0.86	0.60
rs10419232	80726	KIAA1683	19	18265148	1.37E-01	8500	3.39E-04	Down	3.58	2819	0.86	0.35

rs3766924	103	ADAR	1	151384899	1.37E-01	8501	2.38E-02	Up	2.26	5239	0.86	0.16
rs10748672	7093	TLL2	10	98107051	1.37E-01	8502	1.23E-01	Up	1.54	7345	0.86	0.09
rs749003	200162	SPAG17	1	118244022	1.37E-01	8503	2.81E-01	Down	1.08	9043	0.86	0.06
rs12125221	6548	SLC9A1	1	27102059	1.37E-01	8504	7.38E-02	Down	1.79	6501	0.86	0.11
rs4632970	55750	AGK	7	140725900	1.37E-01	8505	6.00E-08	Up	5.42	1239	0.86	0.72
rs1052607	8503	PIK3R3	1	46211546	1.37E-01	8506	2.22E-03	Up	3.06	3604	0.86	0.27
rs12040764	79084	WDR77	1	111713621	1.37E-01	8507	1.99E-04	Up	3.72	2627	0.86	0.37
rs12040764	515	ATP5F1	1	111713621	1.37E-01	8508	1.01E-01	Up	1.64	7017	0.86	0.10
rs12696105	9868	TOMM70A	3	101595506	1.37E-01	8509	9.84E-01	Down	0.02	13803	0.86	0.00
rs11605162	26579	MYEOV	11	68807443	1.38E-01	8510	3.85E-01	Up	0.87	9879	0.86	0.04
rs12479885	245932	DEFB119	20	29449488	1.38E-01	8511	4.05E-01	Down	0.83	10021	0.86	0.04
rs11673407	2525	FUT3	19	5802336	1.38E-01	8512	7.57E-01	Down	0.31	12422	0.86	0.01
rs762523	6576	SLC25A1	22	17555143	1.38E-01	8513	9.13E-01	Up	0.11	13332	0.86	0.00
rs616315	163747	C1orf177	1	54990862	1.38E-01	8514	8.40E-01	Down	0.20	12900	0.86	0.01
rs6867972	3074	HEXB	5	74051106	1.38E-01	8515	1.24E-02	Up	2.50	4657	0.86	0.19
rs6674407	9903	KLHL21	1	6597179	1.38E-01	8516	3.10E-06	Down	4.66	1746	0.86	0.55
rs6674407	148479	PHF13	1	6597179	1.38E-01	8517	5.23E-02	Down	1.94	6045	0.86	0.13
rs10018215	353322	ANKRD37	4	186677555	1.38E-01	8518	6.04E-02	Up	1.88	6240	0.86	0.12
rs10018215	55805	LRP2BP	4	186677555	1.38E-01	8519	7.92E-01	Down	0.26	12629	0.86	0.01
rs3138488	10912	GADD45G	9	89448336	1.38E-01	8520	6.24E-02	Up	1.86	6286	0.86	0.12
rs8107449	29128	UHRF1	19	4884777	1.38E-01	8521	1.02E-02	Up	2.57	4520	0.86	0.20
rs7085830	80201	HKDC1	10	70669498	1.38E-01	8522	9.98E-01	Up	0.00	13899	0.86	0.00
rs17819140	55273	TMEM100	17	51166359	1.38E-01	8523	3.20E-01	Down	1.00	9355	0.86	0.05
rs1921952	255394	TCP11L2	12	105227161	1.38E-01	8524	3.40E-02	Up	2.12	5594	0.86	0.15
rs7199026	10164	CHST4	16	70098727	1.38E-01	8525	8.85E-01	Up	0.14	13154	0.86	0.01
rs1021504	56987	BBX	3	108884295	1.38E-01	8526	1.75E-08	Down	5.63	1111	0.86	0.78
rs1513831	9741	LAPTM4A	2	20178484	1.38E-01	8527	1.02E-12	Down	7.13	558	0.86	1.20
rs2686555	9478	CABP1	12	119557892	1.38E-01	8528	1.49E-02	Down	2.43	4810	0.86	0.18
rs2686555	795	S100G	12	119557892	1.38E-01	8529	8.09E-02	Up	1.75	6643	0.86	0.11
rs893703	5947	RBP1	3	140733347	1.38E-01	8530	5.86E-06	Down	4.53	1841	0.86	0.52
rs7765791	26716	OR2H1	6	29518072	1.38E-01	8531	9.36E-02	Up	1.68	6894	0.86	0.10
rs7765791	26531	OR11A1	6	29518072	1.38E-01	8532	8.52E-01	Down	0.19	12968	0.86	0.01
rs1980243	55329	MNS1	15	54562095	1.38E-01	8533	3.76E-01	Up	0.89	9807	0.86	0.04
rs3938421	9675	KIAA0406	20	36061992	1.38E-01	8534	1.65E-02	Up	2.40	4911	0.86	0.18
rs8102902	116541	MRPL54	19	3720049	1.38E-01	8535	3.46E-03	Up	2.92	3851	0.86	0.25
rs8102902	84839	RAX2	19	3720049	1.38E-01	8536	2.38E-01	Up	1.18	8645	0.86	0.06
rs8102902	9546	APBA3	19	3720049	1.38E-01	8537	2.98E-01	Down	1.04	9179	0.86	0.05
rs8102902	4145	MATK	19	3720049	1.38E-01	8538	6.13E-01	Down	0.51	11528	0.86	0.02
rs10797788	85397	RGS8	1	179355166	1.38E-01	8539	9.66E-01	Up	0.04	13665	0.86	0.00
rs4688938	10141	C4orf6	4	5634102	1.38E-01	8540	6.54E-01	Down	0.45	11794	0.86	0.02
rs10858121	29991	OBP2A	9	135671600	1.38E-01	8541	1.75E-03	Down	3.13	3464	0.86	0.28
rs2603229	145748	LYSMD4	15	98081022	1.38E-01	8542	2.71E-01	Down	1.10	8961	0.86	0.06
rs7259646	170958	ZNF525	19	58565418	1.38E-01	8543	5.01E-09	Up	5.86	1001	0.86	0.83
rs2636885	5004	ORM1	9	114158841	1.38E-01	8544	4.64E-01	Down	0.73	10459	0.86	0.03
rs2636885	5005	ORM2	9	114158841	1.38E-01	8545	5.83E-01	Down	0.55	11334	0.86	0.02
rs4958728	11346	SYNPO	5	149990292	1.38E-01	8546	1.23E-03	Down	3.23	3305	0.86	0.29
rs9816344	57577	KIAA1407	3	115162780	1.39E-01	8547	2.56E-02	Down	2.23	5308	0.86	0.16
rs2074748	22798	LAMB4	7	107338980	1.39E-01	8548	3.63E-01	Up	0.91	9710	0.86	0.04
rs4616261	1464	CSPG4	15	73752224	1.39E-01	8549	4.35E-07	Down	5.05	1463	0.86	0.64
rs2239942	10430	TMEM147	19	40722288	1.39E-01	8550	1.44E-07	Up	5.26	1341	0.86	0.68
rs2239942	374897	SBSN	19	40722288	1.39E-01	8551	4.14E-01	Down	0.82	10085	0.86	0.04
rs2057099	9338	TCEAL1		102687599	1.39E-01	8552	1.23E-08	Down	5.70	1073	0.86	0.79
rs6889194	117156	SCGB3A2	5	147223834	1.39E-01	8553	9.37E-01	Down	0.08	13472	0.86	0.00
rs1805355	1719	DHFR	5	80001785	1.39E-01	8554	2.73E-12	Up	6.99	602	0.86	1.16
rs1509122	83659	TEKT1	17	6634190	1.39E-01	8555	6.31E-01	Up	0.48	11651	0.86	0.02
rs7422405	245973	ATP6V1C2	2	10801527	1.39E-01	8556	1.68E-07	Up	5.23	1355	0.86	0.68
rs1282731	29114	TAGLN3	3	113206460	1.39E-01	8557	9.92E-06	Down	4.42	1950	0.86	0.50
rs6509860	10288	LILRB2	19	59484170	1.39E-01	8558	1.91E-01	Down	1.31	8157	0.86	0.07
rs3754283	2981	GUCA2B	1	42286125	1.39E-01	8559	6.64E-01	Down	0.43	11850	0.86	0.02
rs7897675	7587	ZNF37A	10	38448572	1.39E-01	8560	3.70E-01	Up	0.90	9762	0.86	0.04
rs3210839	27	ABL2	1	175801086	1.39E-01	8561	1.84E-01	Down	1.33	8067	0.86	0.07
rs2125290	10149	GPR64		18835017	1.39E-01	8562	1.39E-01	Down	1.48	7557	0.86	0.09
rs11676348	3579	IL8RB	2	218835652	1.39E-01	8563	8.29E-02	Down	1.73	6684	0.86	0.11
rs8080016	404093	CUEDC1	17	53309402	1.39E-01	8564	2.64E-02	Down	2.22	5339	0.86	0.16
rs3765144	6897	TARS	5	33491967	1.39E-01	8565	8.06E-03	Up	2.65	4347	0.86	0.21
rs6641352	4110	MAGEA11		148479449	1.39E-01	8566	3.29E-01	Up	0.98	9430	0.86	0.05
rs6641352	51402	HSFX1		148479449	1.39E-01	8567	7.53E-01	Down	0.31	12397	0.86	0.01
rs6543282	64965	MRPS9	2	105167541	1.39E-01	8568	6.10E-02	Up	1.87	6255	0.86	0.12
rs739431	10991	SLC38A3	3	50230309	1.39E-01	8569	1.61E-01	Up	1.40	7825	0.86	0.08
rs8060015	84901	NFATC2IP	16	28889728	1.39E-01	8570	1.23E-03	Up	3.23	3304	0.86	0.29
rs2002384	388662	SLC6A17	1	110410693	1.40E-01	8571	3.17E-02	Down	2.15	5515	0.86	0.15
rs7542831	51611	DPH5	1	101184763	1.40E-01	8572	2.32E-08	Up	5.59	1139	0.86	0.76
rs10124667	26267	FBXO10	9	37506500	1.40E-01	8573	2.50E-02	Up	2.24	5285	0.86	0.16
rs3813792	84958	SYTL1	1	27346377	1.40E-01	8574	9.55E-01	Down	0.06	13587	0.86	0.00
rs239500	7272	TTK	6	80761863	1.40E-01	8575	3.89E-04	Up	3.55	2872	0.86	0.34
rs232840	4070	TACSTD2	1	58752740	1.40E-01	8576	3.24E-01	Down	0.99	9394	0.85	0.05
rs2327282	8195	MKKS	20	10375960	1.40E-01	8577	1.88E-03	Down	3.11	3502	0.85	0.27
rs3785354	6279	S100A8	16	28458168	1.40E-01	8578	1.38E-02	Down	2.46	4744	0.85	0.19
rs2183124		B4GALT1	9	33105556	1.40E-01	8579	8.88E-01	Up	0.14	13179	0.85	0.01
rs1730775	4043	LRPAP1	4	3562026	1.40E-01	8580	7.04E-02	Up	1.81	6437	0.85	0.12
rs6836717	92610	TIFA	4	113552279	1.40E-01	8581	7.42E-06	Up	4.48	1882	0.85	0.51

rs6836717	55435	C4orf16	4	113552279	1.40E-01	8582	2.23E-02	Up	2.29	5172	0.85	0.17
rs4687770	2912	GRM2	3	51730105	1.40E-01	8583	7.32E-01	Down	0.34	12263	0.85	0.01
rs4896702	8676	STX11	6	144537126	1.40E-01	8584	5.71E-01	Down	0.57	11254	0.85	0.02
rs7872037	59335	PRDM12	9	130557529	1.40E-01	8585	5.29E-01	Down	0.63	10934	0.85	0.03
rs1041740	6647	SOD1	21	31962033	1.40E-01	8586	3.36E-01	Down	0.96	9496	0.85	0.05
rs1912579	7594	ZNF43	19	21774192	1.40E-01	8587	4.92E-07	Up	5.03	1477	0.85	0.63
rs3810925	54107	POLE3	9	113252760	1.40E-01	8588	3.58E-03	Up	2.91	3870	0.85	0.24
rs11106710	8411	EEA1	12	91717205	1.40E-01	8589	6.21E-01	Up	0.49	11591	0.85	0.02
rs8064638	6777	STAT5B	17	37677781	1.40E-01	8590	3.27E-13	Down	7.28	515	0.85	1.25
rs8064638	6776	STAT5A	17	37677781	1.40E-01	8591	9.27E-03	Down	2.60	4452	0.85	0.20
rs6589422	4837	NNMT	11	113657119	1.40E-01	8592	7.08E-01	Up	0.38	12118	0.85	0.02
rs12337896	64922	LRRC19	9	27008020	1.40E-01	8593	1.11E-01	Up	1.59	7161	0.85	0.10
rs557135	2940	GSTA3	6	52873719	1.40E-01	8594	2.97E-01	Down	1.04	9174	0.85	0.05
rs3824088	5375	PMP2	8	82523684	1.40E-01	8595	4.48E-02	Down	2.01	5871	0.85	0.13
rs4820268	164656	TMPRSS6	22	35794091	1.40E-01	8596	2.63E-01	Down	1.12	8896	0.85	0.06
rs17605251	83787	ARMC10	7	102339902	1.40E-01	8597	9.80E-04	Up	3.30	3214	0.85	0.30
rs2755242	23169	SLC35D1	1	67191619	1.40E-01	8598	3.19E-06	Down	4.66	1750	0.85	0.55
rs447833	100	ADA	20	42696770	1.41E-01	8599	1.78E-02	Down	2.37	4972	0.85	0.18
rs709046	1789	DNMT3B	20	30854890	1.41E-01	8600	1.88E-01	Up	1.32	8120	0.85	0.07
rs10947649	221477	C6orf89	6	37005741	1.41E-01	8601	5.19E-01	Up	0.64	10857	0.85	0.03
rs10846581	144348	ZNF664	12	123008413	1.41E-01	8602	1.88E-07	Up	5.21	1366	0.85	0.67
rs2034598	10294	DNAJA2	16	45559127	1.41E-01	8603	2.89E-01	Down	1.06	9102	0.85	0.05
rs10044860	6949	TCOF1	5	149711264	1.41E-01	8604	4.73E-01	Up	0.72	10520	0.85	0.03
rs3751691	28998	MRPL13	16	88155882	1.41E-01	8605	1.03E-03	Up	3.28	3233	0.85	0.30
rs3751691	6137	RPL13	16	88155882	1.41E-01	8606	2.99E-02	Up	2.17	5459	0.85	0.15
rs3751691	27132	CPNE7	16	88155882	1.41E-01	8607	1.33E-01	Up	1.50	7472	0.85	0.09
rs3751691	6687	SPG7	16	88155882	1.41E-01	8608	2.41E-01	Up	1.17	8662	0.85	0.06
rs4744150	83744	ZNF484	9	92709918	1.41E-01	8609	9.05E-01	Down	0.12	13281	0.85	0.00
rs2062595	54751	FBLIM1	1	15820434	1.41E-01	8610	1.81E-03	Down	3.12	3479	0.85	0.27
rs17125588	51411	BIN2	12	49952353	1.41E-01	8611	5.17E-02	Down	1.95	6031	0.85	0.13
rs9299108	6887	TAL2	9	105513562	1.41E-01	8612	6.80E-01	Down	0.41	11950	0.85	0.02
rs4530322	151056	PLB1	2	28764577	1.41E-01	8613	4.35E-01	Down	0.78	10234	0.85	0.04
rs2516633	6094	ROM1	11	62128349	1.41E-01	8614	6.73E-02	Down	1.83	6382	0.85	0.12
rs2516633	9219	MTA2	11	62128349	1.41E-01	8615	3.13E-01	Down	1.01	9294	0.85	0.05
rs2516633	256364	EML3	11	62128349	1.41E-01	8616	6.17E-01	Up	0.50	11558	0.85	0.02
rs248217	23061	TBC1D9B	5	179242936	1.41E-01	8617	5.34E-07	Up	5.01	1487	0.85	0.63
rs1039153	26986	PABPC1	8	101791542	1.41E-01	8618	3.76E-08	Up	5.51	1192	0.85	0.74
rs2589970	23766	GABARAPL3	15	88696962	1.41E-01	8619	3.97E-03	Down	2.88	3925	0.85	0.24
rs4693626	51023	MRPS18C	4	84730177	1.41E-01	8620	9.65E-01	Down	0.04	13650	0.85	0.00
rs12568353	3814	KISS1	1	200896348	1.41E-01	8621	4.89E-01	Down	0.69	10641	0.85	0.03
rs10517073	29945	ANAPC4	4	25093640	1.41E-01	8622	1.71E-01	Down	1.37	7933	0.85	0.08
rs4908898	57449	PLEKHG5	1	6494629	1.41E-01	8623	3.21E-01	Down	0.99	9368	0.85	0.05
rs7813708	84985	FAM83A	8	124275505	1.41E-01	8624	8.99E-02	Down	1.70	6807	0.85	0.10
rs1894644	23464	GCAT	22	36527052	1.41E-01	8625	8.89E-03	Up	2.62	4427	0.85	0.21
rs1894644	3005	H1F0	22	36527052	1.41E-01	8626	5.81E-01	Down	0.55	11325	0.85	0.02
rs2466293	169026	SLC30A8	8	118255119	1.41E-01	8627	6.08E-02	Up	1.87	6250	0.85	0.12
rs3788891	190	NR0B1		30083573	1.41E-01	8628	4.12E-01	Up	0.82	10069	0.85	0.04
rs181219	26689	OR4D1	17	53540080	1.41E-01	8629	9.00E-01	Down	0.13	13242	0.85	0.00
rs12252986	50674	NEUROG3	10	71022062	1.42E-01	8630	4.59E-01	Down	0.74	10414	0.85	0.03
rs2305668	25989	ULK3	15	72929814	1.42E-01	8631	1.19E-06	Up	4.86	1601	0.85	0.59
rs2305668	10066	SCAMP2	15	72929814	1.42E-01	8632	1.95E-01	Down	1.30	8205	0.85	0.07
rs3747965	30836	DNTTIP2	1	94054585	1.42E-01	8633	7.21E-03	Down	2.69	4277	0.85	0.21
rs291360	148789	B3GALNT2	1	232011711	1.42E-01	8634	6.24E-02	Up	1.86	6287	0.85	0.12
rs4614101	89891	WDR34	9	128505318	1.42E-01	8635	2.86E-01	Up	1.07	9085	0.85	0.05
rs1870839	79621	RNASEH2B	13	50371869	1.42E-01	8636	1.43E-04	Down	3.80	2535	0.85	0.38
rs672534	6188	RPS3	11	74774679	1.42E-01	8637	2.55E-05	Up	4.21	2138	0.85	0.46
rs488007	9320	TRIP12	2	230510475	1.42E-01	8638	1.38E-02	Down	2.46	4745	0.85	0.19
rs4567871	1373	CPS1	2	211364808	1.42E-01	8639	2.00E-01	Up	1.28	8251	0.85	0.07
rs4567871	148811	PM20D1	2	211364808	1.42E-01	8640	4.37E-01	Up	0.78	10251	0.85	0.04
rs4567871	1589	CYP21A2	2	211364808	1.42E-01	8641	5.51E-01	Up	0.60	11088	0.85	0.03
rs11646911	7041	TGFB1I1	16	31391123	1.42E-01	8642	1.21E-17	Down	8.55	284	0.85	1.69
rs11646911	79798	ARMC5	16	31391123	1.42E-01	8643	5.17E-01	Down	0.65	10833	0.85	0.03
rs11646911	6524	SLC5A2	16	31391123	1.42E-01	8644	9.81E-01	Down	0.02	13780	0.85	0.00
rs10499669	256979	SUNC1	7	47795514	1.42E-01	8645	2.62E-01	Down	1.12	8874	0.85	0.06
rs7550874	353133	LCE1C	1	149606496	1.42E-01	8646	1.01E-01	Down	1.64	7028	0.85	0.10
rs606119	566	AZU1	19	778655	1.42E-01	8647	7.89E-02	Down	1.76	6612	0.85	0.11
rs1043395	9145	SYNGR1	22	38105461	1.42E-01	8648	1.02E-16	Down	8.30	321	0.85	1.60
rs380885	117289	TAGAP	6	159437760	1.42E-01	8649	7.87E-01	Up	0.27	12586	0.85	0.01
rs11085749	11018	TMED1	19	10822273	1.42E-01	8650	7.69E-01	Up	0.29	12489	0.85	0.01
rs1874113	64757	MOSC1	1	217368389	1.42E-01	8651	2.64E-11	Up	6.69	677	0.85	1.06
rs242243	56953	NT5M	17	17190331	1.42E-01	8652	4.88E-01	Down	0.69	10635	0.85	0.03
rs574769	8087	FXR1	3	182126289	1.42E-01	8653	1.92E-01	Up	1.30	8177	0.85	0.07
rs10239188	8910	SGCE	7	93944080	1.42E-01	8654	5.35E-17	Down	8.38	310	0.85	1.63
rs3108171	84911	ZNF382	19	41809142	1.42E-01	8655	1.10E-01	Up	1.60	7144	0.85	0.10
rs6488518	54682	MANSC1	12	12406451	1.42E-01	8656	1.28E-01	Up	1.52	7420	0.85	0.09
rs1674139	160	AP2A1	19	54963314	1.43E-01	8657	5.16E-03	Down	2.80	4085	0.85	0.23
rs6048993	1472	CST4	20	23615657	1.43E-01	8658	8.80E-01	Up	0.15	13126	0.85	0.01
rs11639440	79631	EFTUD1	15	80358101	1.43E-01	8659	1.86E-02	Up	2.35	5008	0.85	0.17
rs17834111	64582	GPR135	14	59017107	1.43E-01	8660	1.58E-03	Down	3.16	3413	0.85	0.28
rs17834111	51528	C14orf100	14	59017107	1.43E-01	8661	4.75E-02	Up	1.98	5933	0.85	0.13
rs17834111	112849	C14orf149	14	59017107	1.43E-01	8662	8.24E-01	Up	0.22	12813	0.85	0.01

rs513438	60492	CCDC90B	11	82645611	1.43E-01	8663	7.46E-04	Up	3.37	3106	0.85	0.31
rs513438	338699	ANKRD42	11	82645611	1.43E-01	8664	9.76E-01	Down	0.03	13739	0.85	0.00
rs11102213	113802	C1orf59	1	108933654	1.43E-01	8665	-3.93E-16	Up	7.90	378	0.85	#NUM!
rs11102213	55119	PRPF38B	1	108933654	1.43E-01	8666	8.82E-04	Up	3.33	3170	0.85	0.31
rs7951206	143501	C11orf40	11	4568230	1.43E-01	8667	9.45E-01	Up	0.07	13527	0.85	0.00
rs3804456	6732	SRPK1	6	35926373	1.43E-01	8668	1.22E-01	Up	1.55	7329	0.84	0.09
rs6088713	26133	TRPC4AP	20	33157518	1.43E-01	8669	9.83E-02	Up	1.65	6971	0.84	0.10
rs9845098	9626	GUCA1C	3	110117789	1.43E-01	8670	7.39E-01	Down	0.33	12312	0.84	0.01
rs10499261	80329	ULBP1	6	150375277	1.43E-01	8671	1.46E-01	Up	1.45	7635	0.84	0.08
rs11558492	8443	GNPAT	1	227714826	1.43E-01	8672	7.91E-01	Down	0.27	12613	0.84	0.01
rs13386477	4759	NEU2	2	233720001	1.43E-01	8673	7.48E-01	Down	0.32	12358	0.84	0.01
rs11729968	3558	IL2	4	123728654	1.43E-01	8674	2.77E-04	Up	3.64	2751	0.84	0.36
rs3735008	92092	ZC3HAV1L	7	138189712	1.43E-01	8675	3.49E-02	Up	2.11	5620	0.84	0.15
rs3818003	83746	L3MBTL2	22	39934190	1.43E-01	8676	3.69E-01	Down	0.90	9757	0.84	0.04
rs13256221	81790	RNF170	8	42861656	1.43E-01	8677	1.04E-02	Up	2.56	4548	0.84	0.20
rs5939319	7499	XG		2693518	1.43E-01	8678	5.39E-01	Up	0.61	11005	0.84	0.03
rs1625605	285362	SUMF1	3	4431185	1.43E-01	8679	9.92E-09	Up	5.73	1054	0.84	0.80
rs1967432	3823	KLRC3	12	10496212	1.43E-01	8680	4.39E-02	Up	2.02	5849	0.84	0.14
rs1967432	3822	KLRC2	12	10496212	1.43E-01	8681	4.47E-01	Up	0.76	10325	0.84	0.03
rs6452473	167465	ZNF366	5	71776850	1.44E-01	8682	9.65E-01	Up	0.04	13649	0.84	0.00
rs9354985	79627	OGFRL1	6	72044374	1.44E-01	8683	2.72E-01	Up	1.10	8971	0.84	0.06
rs3733488	57050	UTP3	4	71919310	1.44E-01	8684	2.87E-02	Up	2.19	5416	0.84	0.15
rs16835131	5928	RBBP4	1	32818028	1.44E-01	8685	7.91E-09	Up	5.77	1039	0.84	0.81
rs7615619	10242	KCNMB2	3	179957506	1.44E-01	8686	8.22E-03	Up	2.64	4361	0.84	0.21
rs17168271	55281	TMEM140	7	134310486	1.44E-01	8687	1.41E-01	Down	1.47	7581	0.84	0.09
rs17168271	78996	C7orf49	7	134310486	1.44E-01	8688	9.92E-01	Up	0.01	13857	0.84	0.00
rs1950252	90673	PPP1R3E	14	22848538	1.44E-01	8689	1.42E-11	Up	6.74	659	0.84	1.08
rs1950252	599	BCL2L2	14	22848538	1.44E-01	8690	1.35E-06	Down	4.83	1611	0.84	0.59
rs1950252	8106	PABPN1	14	22848538	1.44E-01	8691	5.17E-03	Up	2.80	4087	0.84	0.23
rs3817442	26273	FBXO3	11	33753984	1.44E-01	8692	4.56E-01	Down	0.74	10393	0.84	0.03
rs10419880	3982	LIM2	19	56589204	1.44E-01	8693	4.23E-01	Down	0.80	10144	0.84	0.04
rs879982	85366	MYLK2	20	29862171	1.44E-01	8694	1.50E-01	Down	1.44	7685	0.84	0.08
rs1440101	4016	LOXL1	15	72011684	1.44E-01	8695	9.26E-03	Down	2.60	4451	0.84	0.20
rs6792607	56648	EIF5A2	3	172093018	1.44E-01	8696	1.31E-02	Down	2.48	4696	0.84	0.19
rs488512	54733	SLC35F2	11	107234316	1.44E-01	8697	1.89E-06	Up	4.76	1657	0.84	0.57
rs734567	9002	F2RL3	19	16876607	1.44E-01	8698	1.03E-02	Up	2.56	4534	0.84	0.20
rs9481408	3066	HDAC2	6	114371980	1.44E-01	8699	2.74E-02	Up	2.21	5376	0.84	0.16
rs4944158	5058	PAK1	11	76703887	1.44E-01	8700	1.25E-11	Down	6.77	645	0.84	1.09
rs6596271	3578	IL9	5	135248728	1.44E-01	8701	3.17E-01	Up	1.00	9334	0.84	0.05
rs9972635	50855	PARD6A	16	66240081	1.44E-01	8702	5.63E-01	Up	0.58	11186	0.84	0.02
rs689931	9677	HISPPD2A	15	41610120	1.44E-01	8703	1.92E-05	Up	4.27	2083	0.84	0.47
rs689931	4130	MAP1A	15	41610120	1.44E-01	8704	3.80E-04	Down	3.55	2861	0.84	0.34
rs3773820	51421	AMOTL2	3	135566340	1.44E-01	8705	1.75E-03	Down	3.13	3462	0.84	0.28
rs10956911	25788	RAD54B	8	95453051	1.44E-01	8706	3.18E-01	Up	1.00	9344	0.84	0.05
rs474225	2693	GHSR	3	173659642	1.44E-01	8707	1.80E-02	Down	2.37	4985	0.84	0.17
rs7596758	129787	TMEM18	2	648595	1.44E-01	8708	2.43E-13	Up	7.31	503	0.84	1.26
rs2419773	79056	PRRG4	11	32834885	1.44E-01	8709	5.01E-12	Down	6.91	623	0.84	1.13
rs6006148	5988	RFPL1	22	28151810	1.45E-01	8710	2.26E-01	Up	1.21	8517	0.84	0.06
rs2331902	10228	STX6	1	177726247	1.45E-01	8711	9.66E-03	Up	2.59	4484	0.84	0.20
rs359965	54738	FEV	2	219666396	1.45E-01	8712	3.66E-06	Up	4.63	1772	0.84	0.54
rs6842123	166863	RBM46	4	156116177	1.45E-01	8713	6.82E-01	Up	0.41	11957	0.84	0.02
rs1790098	10198	MPHOSPH9	12	122180361	1.45E-01	8714	8.02E-07	Up	4.93	1547	0.84	0.61
rs3785425	55697	VAC14	16	69285978	1.45E-01	8715	9.53E-01	Up	0.06	13574	0.84	0.00
rs11764195	64983	MRPL32	7	42754872	1.45E-01	8716	2.41E-01	Down	1.17	8668	0.84	0.06
rs9295784	282890	ZNF311	6	29077127	1.45E-01	8717	1.47E-03	Up	3.18	3370	0.84	0.28
rs7299720	28977	MRPL42	12	92393673	1.45E-01	8718	7.72E-23	Up	9.83	145	0.84	2.21
rs680465	23187	PHLDB1	11	118006173	1.45E-01	8719	1.39E-05	Down	4.35	2024	0.84	0.49
rs7214863	8711	TNK1	17	7214801	1.45E-01	8720	8.74E-01	Down	0.16	13090	0.84	0.01
rs16964483	256586	LYSMD2	15	49796033	1.45E-01	8721	5.01E-02	Up	1.96	6003	0.84	0.13
rs16964483	29106	SCG3	15	49796033	1.45E-01	8722	1.08E-01	Up	1.61	7130	0.84	0.10
rs1284108	28970	C11orf54	11	93138481	1.45E-01	8723	8.72E-01	Up	0.16	13077	0.84	0.01
rs924283	27068	PPA2	4	106639769	1.45E-01	8724	2.96E-01	Up	1.04	9167	0.84	0.05
rs13344253	81856	ZNF611	19	57932919	1.45E-01	8725	3.27E-01	Down	0.98	9420	0.84	0.05
rs4727443	563	AZGP1	7	99237997	1.45E-01	8726	6.27E-03	Down	2.73	4189	0.84	0.22
rs1192113	123283	TARSL2	15	100029954	1.45E-01	8727	1.21E-01	Up	1.55	7314	0.84	0.09
rs1192113	80213	TM2D3	15	100029954	1.45E-01	8728	2.18E-01	Down	1.23	8441	0.84	0.07
rs12972098	79891	ZNF671	19	62941116	1.45E-01	8729	1.76E-02	Down	2.37	4966	0.84	0.18
rs13406085	3557	IL1RN	2	113577792	1.45E-01	8730	3.96E-01	Up	0.85	9961	0.84	0.04
rs12972098	284309	ZNF776	19	62941116	1.45E-01	8731	6.63E-01	Up	0.44	11840	0.84	0.02
rs533017	229	ALDOB	9	101274087	1.45E-01	8732	9.59E-01	Down	0.05	13612	0.84	0.00
rs432157	29888	STRN4	19	51906251	1.45E-01	8733	1.60E-03	Up	3.16	3416	0.84	0.28
rs3771706	29994	BAZ2B	2	160067931	1.45E-01	8734	6.99E-01	Down	0.39	12063	0.84	0.02
rs7015100	3551	IKBKB	8	42240133	1.45E-01	8735	9.73E-01	Down	0.03	13720	0.84	0.00
rs2305806	9322	TRIP10	19	6706007	1.46E-01	8736	2.38E-11	Down	6.68	680	0.84	1.06
rs2305806	56927	GPR108	19	6706007	1.46E-01	8737	1.04E-02	Down	2.56	4545	0.84	0.20
rs4479364	126526	C19orf47	19	45516128	1.46E-01	8738	2.67E-01	Down	1.11	8923	0.84	0.06
rs3851465	28965	SLC27A6	5	128363923	1.46E-01	8739	9.94E-02	Down	1.65	6989	0.84	0.10
rs4343071	94	ACVRL1	12	50573043	1.46E-01	8740	4.93E-02	Down	1.97	5978	0.84	0.13
rs4343071	341405	ANKRD33	12	50573043	1.46E-01	8741	7.15E-01	Down	0.37	12161	0.84	0.01
rs683856	3748	KCNC3	19	55514220	1.46E-01	8742	6.78E-01	Up	0.41	11936	0.84	0.02
rs916661	4836	NMT1	17	40514428	1.46E-01	8743	1.10E-05	Down	4.40	1966	0.84	0.50

rs3763381	55698	RADIL	7	4650253	1.46E-01	8744	6.79E-02	Down	1.83	6393	0.84	0.12
rs4523231	64321	SOX17	8	55527743	1.46E-01	8745	4.44E-01	Down	0.77	10296	0.84	0.04
rs904800	84627	ZNF469	16	87044441	1.46E-01	8746	1.95E-02	Down	2.34	5047	0.84	0.17
rs11072213	55075	UACA	15	68754608	1.46E-01	8747	3.98E-03	Up	2.88	3928	0.84	0.24
rs9978525	29980	DONSON	21	33869726	1.46E-01	8748	2.59E-02	Down	2.23	5317	0.84	0.16
rs494620	4758	NEU1	6	31946692	1.46E-01	8749	3.66E-02	Down	2.09	5670	0.84	0.14
rs2298265	57592	ZNF687	1	148072116	1.46E-01	8750	9.08E-01	Down	0.12	13293	0.84	0.00
rs2302365	5763	PTMS	12	6754505	1.46E-01	8751	8.27E-02	Up	1.74	6678	0.84	0.11
rs12956554	125228	C18orf19	18	13650590	1.46E-01	8752	3.64E-02	Up	2.09	5660	0.84	0.14
rs17165876	7980	TFPI2	7	93182726	1.46E-01	8753	4.80E-02	Up	1.98	5950	0.84	0.13
rs17165876	2792	GNGT1	7	93182726	1.46E-01	8754	1.57E-01	Up	1.42	7764	0.84	0.08
rs4455975	4010	LMX1B	9	126462753	1.46E-01	8755	1.49E-01	Up	1.44	7674	0.84	0.08
rs5743291	1540	CYLD	16	49314777	1.46E-01	8756	2.75E-13	Down	7.31	505	0.84	1.26
rs4671800	54465	ETAA1	2	67533999	1.46E-01	8757	1.18E-02	Up	2.52	4625	0.84	0.19
rs4981345	29986	SLC39A2	14	20525813	1.46E-01	8758	1.39E-02	Down	2.46	4750	0.84	0.19
rs4981345	64745	METT11D1	14	20525813	1.46E-01	8759	1.17E-01	Down	1.57	7248	0.84	0.09
rs3796861	83888	FGFBP2	4	15654000	1.46E-01	8760	5.75E-02	Down	1.90	6173	0.84	0.12
rs268882	10097	ACTR2	2	65414453	1.46E-01	8761	6.03E-01	Down	0.52	11445	0.84	0.02
rs1784254	10632	ATP5L	11	117760894	1.46E-01	8762	1.58E-01	Up	1.41	7782	0.84	0.08
rs1784254	9354	UBE4A	11	117760894	1.46E-01	8763	5.27E-01	Up	0.63	10923	0.84	0.03
rs12708003	767	CA8	8	61313704	1.46E-01	8764	9.96E-01	Down	0.01	13882	0.84	0.00
rs6434197	151112	ZSWIM2	2	187524213	1.46E-01	8765	5.95E-01	Down	0.53	11399	0.84	0.02
rs3017319	219525	OR5AK4P	11	56555819	1.46E-01	8766	5.12E-01	Up	0.66	10792	0.83	0.03
rs4756961	283284	IGSF22	11	18713931	1.46E-01	8767	7.91E-01	Up	0.27	12611	0.83	0.01
rs553251	80235	PIGZ	3	198173431	1.46E-01	8768	9.76E-01	Up	0.03	13734	0.83	0.00
rs4810841	10904	BLCAP	20	35608980	1.46E-01	8769	1.47E-03	Down	3.18	3375	0.83	0.28
rs11655670	84073	MYCBPAP	17	45929201	1.46E-01	8770	8.33E-01	Up	0.21	12867	0.83	0.01
rs3757351	8528	DDO	6	110842323	1.46E-01	8771	8.66E-01	Up	0.17	13038	0.83	0.01
rs33543	167227	DCP2	5	112349387	1.46E-01	8772	4.56E-01	Down	0.75	10392	0.83	0.03
rs2290176	222487	GPR97	16	56253011	1.47E-01	8773	2.07E-01	Up	1.26	8322	0.83	0.07
rs624786	9789	SPCS2	11	74371677	1.47E-01	8774	3.24E-02	Up	2.14	5540	0.83	0.15
rs2910337	84054	PCDHB19P	5	140612109	1.47E-01	8775	4.33E-01	Down	0.78	10212	0.83	0.04
rs2910337	56121	PCDHB15	5	140612109	1.47E-01	8776	6.59E-01	Up	0.44	11825	0.83	0.02
rs1281174	3744	KCNA10	1	110773104	1.47E-01	8777	6.65E-02	Down	1.83	6366	0.83	0.12
rs2846364	64066	MMP27	11	102078038	1.47E-01	8778	7.12E-01	Up	0.37	12146	0.83	0.01
rs960893	4332	MNDA	1	155633226	1.47E-01	8779	2.29E-01	Up	1.20	8552	0.83	0.06
rs3784563	9399	STOML1	15	72078046	1.47E-01	8780	4.86E-02	Down	1.97	5962	0.83	0.13
rs3784563	5371	PML	15	72078046	1.47E-01	8781	1.17E-01	Up	1.57	7255	0.83	0.09
rs12980619	8907	AP1M1	19	16207176	1.47E-01	8782	4.71E-01	Down	0.72	10508	0.83	0.03
rs5964488	11326	VSIG4		65036790	1.47E-01	8783	5.95E-01	Up	0.53	11405	0.83	0.02
rs17496172	79819	WDR78	1	67014431	1.47E-01	8784	6.38E-03	Up	2.73	4202	0.83	0.22
rs17824786	122876	GPHB5	14	62855408	1.47E-01	8785	2.48E-01	Down	1.15	8752	0.83	0.06
rs6428492	56267	CCBL2	1	89179808	1.47E-01	8786	9.06E-01	Up	0.12	13287	0.83	0.00
rs287127	284415	VSTM1	19	59233264	1.47E-01	8787	5.06E-01	Up	0.67	10757	0.83	0.03
rs8041622	290	ANPEP	15	88153073	1.47E-01	8788	5.53E-01	Up	0.59	11101	0.83	0.03
rs9326783	4124	MAN2A1	5	109203066	1.47E-01	8789	8.83E-01	Down	0.15	13144	0.83	0.01
rs8041357	10620	ARID3B	15	72656491	1.47E-01	8790	1.29E-01	Down	1.52	7442	0.83	0.09
rs874540	1687	DFNA5	7	24531401	1.47E-01	8791	7.82E-05	Down	3.95	2385	0.83	0.41
rs3813720	153	ADRB1	10	115797006	1.47E-01	8792	1.85E-02	Up	2.36	5005	0.83	0.17
rs1805772	4074	M6PR	12	8978348	1.47E-01	8793	4.17E-02	Down	2.04	5794	0.83	0.14
rs12403443	23095	KIF1B	1	10358254	1.47E-01	8794	1.04E-01	Down	1.63	7061	0.83	0.10
rs3754898	1261	CNGA3	2	98456326	1.47E-01	8795	1.92E-01	Up	1.31	8166	0.83	0.07
rs3755652	9497	SLC4A7	3	27447940	1.47E-01	8796	2.14E-03	Up	3.07	3574	0.83	0.27
rs13333329	57585	CRAMP1L	16	1635777	1.47E-01	8797	2.93E-01	Up	1.05	9137	0.83	0.05
rs7583755	84910	TMEM87B	2	112574716	1.47E-01	8798	1.25E-01	Up	1.53	7371	0.83	0.09
rs2052329	50865	HEBP1	12	13027996	1.47E-01	8799	8.09E-01	Up	0.24	12720	0.83	0.01
rs6425931	79830	ZMYM1	1	35237105	1.48E-01	8800	4.70E-02	Up	1.99	5924	0.83	0.13
rs12290860	4544	MTNR1B	11	92351826	1.48E-01	8801	1.01E-01	Down	1.64	7011	0.83	0.10
rs6060752	140894	C20orf152	20	34061376	1.48E-01	8802	2.13E-01	Down	1.25	8389	0.83	0.07
rs10494363	10903	MTMR11	1	146722568	1.48E-01	8803	6.08E-10	Down	6.19	865	0.83	0.92
rs10494363	10262	SF3B4	1	146722568	1.48E-01	8804	1.35E-01	Up	1.49	7511	0.83	0.09
rs178740	7032	TFF2	21	42650741	1.48E-01	8805	3.20E-02	Down	2.14	5524	0.83	0.15
rs9894841	6329	SCN4A	17	59391417	1.48E-01	8806	9.28E-02	Down	1.68	6880	0.83	0.10
rs1204396	27286	SRPX2		99688344	1.48E-01	8807	2.81E-01	Up	1.08	9036	0.83	0.06
rs6006807	84247	LDOC1L	22	43206127	1.48E-01	8808	2.05E-01	Up	1.27	8308	0.83	0.07
rs8051691	9688	NUP93	16	55393213	1.48E-01	8809	4.03E-03	Up	2.88	3935	0.83	0.24
rs4414157	7414	VCL	10	75478319	1.48E-01	8810	2.90E-38	Down	12.93	21	0.83	3.75
rs896126	137695	TMEM68	8	56864537	1.48E-01	8811	1.26E-06	Up	4.85	1605	0.83	0.59
rs10868362	60560	MAK10	9	85864216	1.48E-01	8812	2.48E-12	Up	6.97	608	0.83	1.16
rs2290708	6556	SLC11A1	2	219077882	1.48E-01	8813	4.55E-01	Down	0.75	10382	0.83	0.03
rs2290708	58190	CTDSP1	2	219077882	1.48E-01	8814	6.17E-01	Down	0.50	11557	0.83	0.02
rs10897430	117245	HRASLS5	11	63032139	1.48E-01	8815	8.20E-03	Down	2.64	4359	0.83	0.21
rs8074524	6774	STAT3	17	37723124	1.48E-01	8816	3.47E-15	Down	7.87	381	0.83	1.45
rs2708081	64897	C12orf43	12	119926008	1.48E-01	8817	5.00E-01	Down	0.67	10716	0.83	0.03
rs16891725	11120	BTN2A1	6	26587129	1.48E-01	8818	3.60E-07	Down	5.09	1440	0.83	0.64
rs10998624	6832	SUPV3L1	10	70609807	1.48E-01	8819	6.59E-01	Up	0.44	11823	0.83	0.02
rs6425977	9967	THRAP3	1	36421526	1.48E-01	8820	8.15E-01	Down	0.23	12755	0.83	0.01
rs3765266	63922	CHTF18	16	780770	1.48E-01	8821	3.70E-01	Up	0.90	9765	0.83	0.04
rs3765266	51764	GNG13	16	780770	1.48E-01	8822	3.95E-01	Up	0.85	9946	0.83	0.04
rs7248719	51129	ANGPTL4	19	8324902	1.48E-01	8823	6.42E-08	Down	5.41	1248	0.83	0.72
rs9361459	134728	IRAK1BP1	6	79629641	1.49E-01	8824	3.17E-01	Down	1.00	9337	0.83	0.05

rs1881998	6565	SLC15A2	3	123096865	1.49E-01	8825	6.21E-01	Up	0.50	11584	0.83	0.02
rs5942907	9949	AMMECR1		109276999	1.49E-01	8826	1.59E-09	Up	6.03	926	0.83	0.88
rs2287363	56904	SH3GLB2	9	128847222	1.49E-01	8827	2.30E-02	Up	2.27	5197	0.83	0.16
rs2287363	23511	NUP188	9	128847222	1.49E-01	8828	5.16E-01	Up	0.65	10825	0.83	0.03
rs2273405	29920	PYCR2	1	222430876	1.49E-01	8829	9.09E-01	Up	0.11	13301	0.83	0.00
rs16974228	55114	ARHGAP17	16	24861045	1.49E-01	8830	5.57E-03	Down	2.77	4140	0.83	0.23
rs4791881	9340	GLP2R	17	9675351	1.49E-01	8831	1.98E-02	Up	2.33	5062	0.83	0.17
rs13284038	138649	ANKRD19	9	92635043	1.49E-01	8832	6.39E-01	Up	0.47	11699	0.83	0.02
rs17829472	9907	KIAA0415	7	4587483	1.49E-01	8833	8.41E-02	Up	1.73	6702	0.83	0.11
rs17829472	221937	FOXK1	7	4587483	1.49E-01	8834	1.27E-01	Up	1.53	7399	0.83	0.09
rs1568277	5624	PROC	2	127906285	1.49E-01	8835	8.63E-01	Up	0.17	13022	0.83	0.01
rs7899442	79741	C10orf68	10	32995111	1.49E-01	8836	4.85E-01	Up	0.70	10611	0.83	0.03
rs4694075	258	AMBN	4	71647674	1.49E-01	8837	5.70E-01	Down	0.57	11251	0.83	0.02
rs3026457	488	ATP2A2	12	109215918	1.49E-01	8838	1.48E-10	Down	6.41	778	0.83	0.98
rs9630209	156	ADRBK1	11	66778094	1.49E-01	8839	4.69E-06	Up	4.58	1803	0.83	0.53
rs6676644	10269	ZMPSTE24	1	40410992	1.49E-01	8840	1.04E-14	Up	7.72	414	0.83	1.40
rs4563088	4884	NPTX1	17	76050172	1.49E-01	8841	4.48E-01	Down	0.76	10331	0.83	0.03
rs2057209	84631	SLITRK2		144619923	1.49E-01	8842	1.21E-01	Down	1.55	7302	0.83	0.09
rs2057209	9142	CXorf1		144619923	1.49E-01	8843	3.41E-01	Up	0.95	9529	0.83	0.05
rs3730064	112	ADCY6	12	47462849	1.49E-01	8844	3.92E-04	Up	3.55	2876	0.83	0.34
rs11842558	2621	GAS6	13	113587835	1.49E-01	8845	1.80E-23	Down	9.98	131	0.83	2.27
rs219761	23562	CLDN14	21	36761280	1.50E-01	8846	5.60E-01	Down	0.58	11161	0.83	0.03
rs7619362	339883	C3orf35	3	37449036	1.50E-01	8847	2.87E-01	Down	1.07	9088	0.82	0.05
rs4676410	2859	GPR35	2	241283729	1.50E-01	8848	1.80E-01	Down	1.34	8017	0.82	0.07
rs4676410	11132	CAPN10	2	241283729	1.50E-01	8849	3.47E-01	Down	0.94	9567	0.82	0.05
rs10251163	2020	EN2	7	154763853	1.50E-01	8850	8.99E-01	Down	0.13	13234	0.82	0.00
rs9277932	1302	COL11A2	6	33249231	1.50E-01	8851	6.15E-01	Up	0.50	11542	0.82	0.02
rs6576743	55283	MCOLN3	1	85213073	1.50E-01	8852	2.34E-07	Up	5.17	1390	0.82	0.66
rs7152879	4149	MAX	14	64604942	1.50E-01	8853	4.24E-08	Down	5.48	1209	0.82	0.74
rs7152879	2342	FNTB	14	64604942	1.50E-01	8854	4.41E-02	Down	2.01	5853	0.82	0.14
rs9856967	23122	CLASP2	3	33623719	1.50E-01	8855	3.37E-03	Up	2.93	3838	0.82	0.25
rs6017486	3787	KCNS1	20	43158293	1.50E-01	8856	7.77E-01	Down	0.28	12539	0.82	0.01
rs9471941	285855	RPL7L1	6	42965805	1.50E-01	8857	8.16E-11	Up	6.50	744	0.82	1.01
rs805693	1308	COL17A1	10	105805314	1.50E-01	8858	6.01E-19	Down	8.89	233	0.82	1.82
rs3204635	6472	SHMT2	12	55923860	1.50E-01	8859	1.61E-11	Up	6.74	656	0.82	1.08
rs3204635	22864	R3HDM2	12	55923860	1.50E-01	8860	1.10E-02	Up	2.54	4573	0.82	0.20
rs3204635	246329	STAC3	12	55923860	1.50E-01	8861	3.52E-01	Down	0.93	9607	0.82	0.05
rs12401436	10485	C1orf61	1	153195543	1.50E-01	8862	2.37E-01	Up	1.18	8632	0.82	0.06
rs2903754	84079	ANKRD27	19	37783522	1.50E-01	8863	7.88E-02	Down	1.76	6610	0.82	0.11
rs6511789	79973	ZNF442	19	12347228	1.50E-01	8864	1.09E-01	Down	1.60	7134	0.82	0.10
rs17283264	1230	CCR1	3	46228570	1.50E-01	8865	5.31E-01	Up	0.63	10948	0.82	0.03
rs6033250	22903	BTBD3	20	11813731	1.50E-01	8866	7.80E-07	Down	4.94	1537	0.82	0.61
rs6784925	4154	MBNL1	3	153505588	1.50E-01	8867	5.46E-06	Down	4.55	1832	0.82	0.53
rs159143	10093	ARPC4	3	9810140	1.50E-01	8868	1.18E-05	Down	4.38	1984	0.82	0.49
rs159143	10474	TADA3L	3	9810140	1.50E-01	8869	9.35E-01	Up	0.08	13458	0.82	0.00
rs10793339	79731	NARS2	11	77966722	1.50E-01	8870	4.94E-13	Up	7.30	508	0.82	1.23
rs2732811	130827	TMEM182	2	102865217	1.50E-01	8871	2.56E-04	Up	3.66	2716	0.82	0.36
rs2822447	54033	RBM11	21	14521834	1.51E-01	8872	4.30E-01	Up	0.79	10199	0.82	0.04
rs1052690	414328	C9orf103	9	83488239	1.51E-01	8873	5.88E-02	Down	1.89	6215	0.82	0.12
rs1873318	266977	GPR110	6	47074233	1.51E-01	8874	1.98E-06	Up	4.76	1668	0.82	0.57
rs17199551	79711	IPO4	14	23716309	1.51E-01	8875	2.92E-06	Up	4.68	1731	0.82	0.55
rs12139552	2060	EPS15	1	51704357	1.51E-01	8876	4.00E-04	Up	3.54	2885	0.82	0.34
rs4363498	509	ATP5C1	10	7865536	1.51E-01	8877	6.37E-03	Up	2.73	4200	0.82	0.22
rs4979239	54836	BSPRY	9	113201125	1.51E-01	8878	1.82E-12	Up	7.05	576	0.82	1.17
rs4979239	114987	WDR31	9	113201125	1.51E-01	8879	4.70E-01	Up	0.72	10500	0.82	0.03
rs11588568	148867	SLC30A7	1	101138981	1.51E-01	8880	2.14E-05	Up	4.25	2101	0.82	0.47
rs4723884	5898	RALA	7	39422515	1.51E-01	8881	2.36E-02	Up	2.26	5224	0.82	0.16
rs2709386	151194	FAM119A	2	208322540	1.51E-01	8882	9.88E-03	Up	2.58	4499	0.82	0.20
rs2472394	2539	G6PD		153335055	1.51E-01	8883	1.61E-03	Down	3.15	3421	0.82	0.28
rs2472394	8517	IKBKG		153335055	1.51E-01	8884	8.32E-01	Up	0.21	12860	0.82	0.01
rs1879497	199704	ZNF585A	19	42324717	1.51E-01	8885	6.88E-01	Down	0.40	11996	0.82	0.02
rs2868730	51316	PLAC8	4	84365441	1.51E-01	8886	1.19E-01	Down	1.56	7276	0.82	0.09
rs2916238	114771	PGLYRP3	1	150073784	1.51E-01	8887	8.22E-01	Down	0.22	12803	0.82	0.01
rs6989495	157506	RDH10	8	74392777	1.51E-01	8888	1.96E-03	Down	3.10	3521	0.82	0.27
rs7252027	126272	EID2B	19	44722544	1.52E-01	8889	7.49E-01	Up	0.32	12371	0.82	0.01
rs13317337	604	BCL6	3	188965138	1.52E-01	8890	3.45E-04	Down	3.58	2829	0.82	0.35
rs2515847	4111	MAGEA12		151578744	1.52E-01	8891	6.37E-02	Up	1.85	6316	0.82	0.12
rs11912889	3162	HMOX1	22	34108171	1.52E-01	8892	3.50E-04	Up	3.57	2834	0.82	0.35
rs9924599	56061	UBFD1	16	23480607	1.52E-01	8893	5.03E-05	Up	4.05	2289	0.82	0.43
rs10947842	4337	MOCS1	6	40023327	1.52E-01	8894	8.97E-01	Down	0.13	13225	0.82	0.00
rs2211129	10905	MAN1A2	1	117726949	1.52E-01	8895	5.87E-01	Up	0.54	11350	0.82	0.02
rs7101294	83860	TAF3	10	8062879	1.52E-01	8896	4.42E-05	Down	4.08	2252	0.82	0.44
rs12761929	56889	TM9SF3	10	98323159	1.52E-01	8897	2.21E-04	Up	3.69	2666	0.82	0.37
rs870215	23516	SLC39A14	8	22314082	1.52E-01	8898	4.57E-01	Up	0.74	10396	0.82	0.03
rs1885523	4005	LMO2	11	33860390	1.52E-01	8899	7.68E-04	Down	3.36	3120	0.82	0.31
rs2489	51659	GINS2	16	84288500	1.52E-01	8900	7.57E-01	Up	0.31	12425	0.82	0.01
rs10515921	8809	IL18R1	2	102439536	1.52E-01	8901	8.79E-01	Up	0.15	13120	0.82	0.01
rs16973407	64857	PLEKHG2	19	44605398	1.52E-01	8902	1.71E-02	Down	2.38	4943	0.82	0.18
rs11809572	2135	EXTL2	1	101062327	1.52E-01	8903	4.06E-01	Down	0.83	10027	0.82	0.04
rs518357	2488	FSHB	11	30197405	1.52E-01	8904	9.87E-01	Up	0.02	13824	0.82	0.00
rs2432560	65009	NDRG4	16	57091210	1.52E-01	8905	3.86E-02	Down	2.07	5715	0.82	0.14

rs2432560	79918	SETD6	16	57091210	1.52E-01	8906	1.40E-01	Up	1.47	7580	0.82	0.09
rs7025502	5090	PBX3	9	125779022	1.52E-01	8907	1.00E+00	Up	0.00	13905	0.82	0.00
rs1045811	84932	RAB2B	14	20998223	1.52E-01	8908	1.39E-02	Up	2.46	4753	0.82	0.19
rs1565010	55345	C4orf21	4	113901746	1.52E-01	8909	3.69E-01	Up	0.90	9756	0.82	0.04
rs7015219	203100	HTRA4	8	38939594	1.52E-01	8910	3.59E-01	Up	0.92	9674	0.82	0.04
rs5963262	7504	XK		37346450	1.52E-01	8911	1.16E-01	Down	1.57	7228	0.82	0.09
rs7523438	51538	ZCCHC17	1	31457406	1.52E-01	8912	4.13E-01	Up	0.82	10074	0.82	0.04
rs12562167	27237	ARHGEF16	1	3404855	1.52E-01	8913	2.85E-04	Up	3.63	2760	0.82	0.35
rs3749665	64374	SIL1	5	138385110	1.52E-01	8914	1.56E-03	Up	3.16	3404	0.82	0.28
rs3041	219287	FAM123A	13	24662061	1.52E-01	8915	2.57E-01	Down	1.13	8834	0.82	0.06
rs10412882	23396	PIP5K1C	19	3577114	1.53E-01	8916	5.91E-05	Down	4.02	2327	0.82	0.42
rs10412882	6915	TBXA2R	19	3577114	1.53E-01	8917	1.32E-02	Down	2.48	4700	0.82	0.19
rs4880087	89958	C9orf140	9	137238928	1.53E-01	8918	9.41E-04	Up	3.31	3192	0.82	0.30
rs4880087	11253	MAN1B1	9	137238928	1.53E-01	8919	3.67E-01	Up	0.90	9741	0.82	0.04
rs4880087	954	ENTPD2	9	137238928	1.53E-01	8920	7.01E-01	Down	0.38	12076	0.82	0.02
rs4880087	91373	UAP1L1	9	137238928	1.53E-01	8921	9.96E-01	Up	0.00	13884	0.82	0.00
rs2377717	79570	NKAIN1	1	31342356	1.53E-01	8922	1.56E-02	Up	2.42	4845	0.82	0.18
rs10002500	1259	CNGA1	4	47799607	1.53E-01	8923	2.13E-03	Up	3.07	3572	0.82	0.27
rs16958421	9755	TBKBP1	17	43117118	1.53E-01	8924	7.03E-02	Down	1.81	6434	0.82	0.12
rs16958421	3837	KPNB1	17	43117118	1.53E-01	8925	3.12E-01	Up	1.01	9285	0.82	0.05
rs17714829	55379	LRRC59	17	45807480	1.53E-01	8926	1.71E-05	Up	4.30	2061	0.82	0.48
rs17714829	146956	EME1	17	45807480	1.53E-01	8927	3.62E-01	Up	0.91	9693	0.82	0.04
rs17714829	51264	MRPL27	17	45807480	1.53E-01	8928	8.99E-01	Up	0.13	13236	0.82	0.00
rs1783811	55867	SLC22A11	11	64089872	1.53E-01	8929	6.08E-01	Down	0.51	11489	0.82	0.02
rs7556035	128061	C1orf131	1	227662817	1.53E-01	8930	5.43E-03	Up	2.78	4120	0.82	0.23
rs1057335	5345	SERPINF2	17	1604403	1.53E-01	8931	2.00E-01	Up	1.28	8254	0.82	0.07
rs2341479	22926	ATF6	1	158617010	1.53E-01	8932	7.87E-01	Down	0.27	12587	0.82	0.01
rs10748280	11010	GLIPR1	12	74166964	1.53E-01	8933	1.64E-03	Down	3.15	3432	0.82	0.28
rs299638	80746	TSEN2	3	12509327	1.53E-01	8934	2.30E-02	Up	2.27	5202	0.81	0.16
rs696625	57648	KIAA1522	1	32896704	1.53E-01	8935	3.13E-02	Up	2.15	5505	0.81	0.15
rs370534	28831	IGLJ3	22	21581605	1.53E-01	8936	5.72E-01	Up	0.57	11256	0.81	0.02
rs7918386	1979	EIF4EBP2	10	71839631	1.53E-01	8937	1.28E-05	Down	4.36	2010	0.81	0.49
rs9651273	54991	C1orf159	1	1071463	1.53E-01	8938	9.82E-01	Up	0.02	13791	0.81	0.00
rs16910559	2040	STOM	9	121185763	1.53E-01	8939	8.77E-10	Down	6.13	884	0.81	0.91
rs11012794	64215	DNAJC1	10	22145041	1.53E-01	8940	1.80E-01	Down	1.34	8024	0.81	0.07
rs11008641	94134	ARHGAP12	10	32125254	1.54E-01	8941	1.38E-05	Up	4.35	2021	0.81	0.49
rs3814526	3934	LCN2	9	127990241	1.54E-01	8942	8.21E-02	Down	1.74	6671	0.81	0.11
rs3814526	79095	C9orf16	9	127990241	1.54E-01	8943	2.77E-01	Up	1.09	9010	0.81	0.06
rs3814526	80142	PTGES2	9	127990241	1.54E-01	8944	7.11E-01	Down	0.37	12139	0.81	0.01
rs4507742	5828	PXMP3	8	78062307	1.54E-01	8945	9.58E-02	Up	1.67	6920	0.81	0.10
rs2275849	84270	C9orf89	9	92949498	1.54E-01	8946	1.93E-01	Down	1.30	8181	0.81	0.07
rs12439771	23269	MGA	15	39764985	1.54E-01	8947	1.68E-02	Up	2.39	4929	0.81	0.18
rs3759443	2841	GPR18	13	98708323	1.54E-01	8948	3.62E-01	Up	0.91	9695	0.81	0.04
rs246085	7374	UNG	12	108020917	1.54E-01	8949	2.85E-05	Up	4.19	2160	0.81	0.45
rs4837264	4957	ODF2	9	128281449	1.54E-01	8950	9.80E-08	Up	5.33	1295	0.81	0.70
rs7921970	3799	KIF5B	10	32392935	1.54E-01	8951	1.22E-01	Down	1.55	7337	0.81	0.09
rs2712156	54940	OCIAD1	4	48684158	1.54E-01	8952	6.71E-01	Up	0.43	11891	0.81	0.02
rs1160142	81570	CLPB	11	71784836	1.54E-01	8953	4.84E-02	Up	1.97	5958	0.81	0.13
rs7503175	359845	FAM101B	17	285508	1.54E-01	8954	2.10E-01	Down	1.25	8353	0.81	0.07
rs7308097	80824	DUSP16	12	12554537	1.54E-01	8955	3.63E-05	Down	4.13	2211	0.81	0.44
rs2031078	257629	ANKS4B	16	21163622	1.54E-01	8956	9.65E-01	Down	0.04	13656	0.81	0.00
rs234712	875	CBS	21	43360522	1.54E-01	8957	4.28E-06	Up	4.60	1791	0.81	0.54
rs2257791	57698	KIAA1598	10	118633660	1.54E-01	8958	2.81E-01	Up	1.08	9038	0.81	0.06
rs700651	66037	BOLL	2	198457220	1.54E-01	8959	1.86E-01	Down	1.32	8094	0.81	0.07
rs2466605	26492	OR8G2	11	123605456	1.54E-01	8960	5.69E-01	Down	0.57	11234	0.81	0.02
rs2286614	50801	KCNK4	11	63824886	1.54E-01	8961	5.26E-02	Down	1.94	6057	0.81	0.13
rs2286614	572	BAD	11	63824886	1.54E-01	8962	1.12E-01	Up	1.59	7183	0.81	0.09
rs2286614	2101	ESRRA	11	63824886	1.54E-01	8963	3.71E-01	Up	0.89	9773	0.81	0.04
rs745749	5601	MAPK9	5	179648409	1.54E-01	8964	4.59E-04	Up	3.50	2926	0.81	0.33
rs3130257	5863	RGL2	6	33364449	1.55E-01	8965	3.60E-19	Up	8.94	223	0.81	1.84
rs3130257	6293	VPS52	6	33364449	1.55E-01	8966	5.61E-01	Up	0.58	11172	0.81	0.03
rs3130257		B3GALT4	6	33364449	1.55E-01	8967	7.30E-01	Down	0.34	12251	0.81	0.01
rs13159461	340024	SLC6A19	5	1256437	1.55E-01	8968	3.74E-03	Down	2.90	3892	0.81	0.24
rs11658922	284111	SLC13A5	17	6566109	1.55E-01	8969	9.20E-01	Down	0.10	13375	0.81	0.00
rs2734838	1813	DRD2	11	112791711	1.55E-01	8970	1.26E-01	Down	1.53	7378	0.81	0.09
rs7576391	54980	C2orf42	2	70300322	1.55E-01	8971	4.70E-01	Down	0.72	10501	0.81	0.03
rs11149470	387496	RASL11A	13	26750569	1.55E-01	8972	8.71E-01	Down	0.16	13069	0.81	0.01
rs9933843	1489	CTF1	16	30811180	1.55E-01	8973	2.75E-12	Down	6.99	599	0.81	1.16
rs9933843	9274	BCL7C	16	30811180	1.55E-01	8974	4.93E-01	Down	0.69	10668	0.81	0.03
rs4729036	54467	ANKIB1	7	91663110	1.55E-01	8975	1.81E-06	Up	4.77	1653	0.81	0.57
rs12144658	6814	STXBP3	1	108997867	1.55E-01	8976	4.58E-01	Down	0.74	10410	0.81	0.03
rs1350386	79725	THAP9	4	84176643	1.55E-01	8977	1.62E-02	Up	2.40	4903	0.81	0.18
rs3803217	56163	RNF17	13	24229027	1.55E-01	8978	5.58E-01	Up	0.59	11140	0.81	0.03
rs12335593	9991	ROD1	9	112181256	1.55E-01	8979	4.57E-06	Up	4.58	1799	0.81	0.53
rs2420416	22859	LPHN1	19	14159442	1.55E-01	8980	1.20E-02	Down	2.51	4637	0.81	0.19
rs10005531	65008	MRPL1	4	79177692	1.55E-01	8981	4.59E-02	Up	2.00	5892	0.81	0.13
rs12441858	91947	ARRDC4	15	96301463	1.56E-01	8982	3.87E-03	Up	2.89	3907	0.81	0.24
rs6991974	10565	ARFGEF1	8	68286724	1.56E-01	8983	1.75E-04	Up	3.75	2586	0.81	0.38
rs2071258	7739	ZNF185		151759607	1.56E-01	8984	1.09E-09	Down	6.10	900	0.81	0.90
rs12936240	125144	C17orf45	17	16271644	1.56E-01	8985	4.90E-04	Up	3.49	2953	0.81	0.33
rs12936240	51393	TRPV2	17	16271644	1.56E-01	8986	2.91E-02	Up	2.18	5430	0.81	0.15

rs950254	89885	FATE1		150544972	1.56E-01	8987	1.15E-01	Up	1.58	7214	0.81	0.09
rs17163816	55061	SUSD4	1	219875761	1.56E-01	8988	2.39E-06	Down	4.72	1696	0.81	0.56
rs17163816	7100	TLR5	1	219875761	1.56E-01	8989	9.03E-02	Down	1.69	6827	0.81	0.10
rs500709	4301	MLLT4	6	168027007	1.56E-01	8990	2.97E-01	Up	1.04	9173	0.81	0.05
rs500709	26238	C6orf123	6	168027007	1.56E-01	8991	7.73E-01	Down	0.29	12512	0.81	0.01
rs4243982	57631	LRCH2		114297750	1.56E-01	8992	1.53E-02	Down	2.43	4827	0.81	0.18
rs2058425	3820	KLRB1	12	9640033	1.56E-01	8993	6.89E-01	Up	0.40	11999	0.81	0.02
rs11856999	5607	MAP2K5	15	65877754	1.56E-01	8994	4.17E-01	Down	0.81	10106	0.81	0.04
rs2226827	874	CBR3	21	36415880	1.56E-01	8995	5.62E-04	Up	3.45	3002	0.81	0.33
rs4473307	23640	HSPBP1	19	60458618	1.56E-01	8996	8.96E-02	Down	1.70	6801	0.81	0.10
rs722306	26353	HSPB8	12	118073205	1.56E-01	8997	2.74E-29	Down	11.24	69	0.81	2.86
rs722306	84530	KIAA1853	12	118073205	1.56E-01	8998	9.20E-01	Up	0.10	13376	0.81	0.00
rs277952	64087	MCCC2	5	70948437	1.56E-01	8999	7.09E-36	Up	12.54	33	0.81	3.51
rs3762614	27302	BMP10	2	69012704	1.56E-01	9000	5.10E-01	Down	0.66	10781	0.81	0.03
rs2076101	200316	APOBEC3F	22	37770054	1.56E-01	9001	1.15E-06	Down	4.86	1593	0.81	0.59
rs3761684	93185	IGSF8	1	156897236	1.56E-01	9002	1.30E-11	Up	6.60	700	0.81	1.09
rs11070293	2644	GCHFR	15	38829167	1.56E-01	9003	5.15E-03	Down	2.80	4082	0.81	0.23
rs11123523	140738	TMEM37	2	119903574	1.56E-01	9004	5.65E-01	Down	0.58	11203	0.81	0.02
rs639318	8089	YEATS4	12	68034718	1.56E-01	9005	2.74E-06	Up	4.69	1721	0.81	0.56
rs639318	4069	LYZ	12	68034718	1.56E-01	9006	3.91E-05	Up	4.11	2226	0.81	0.44
rs203433	202018	TAPT1	4	15910056	1.56E-01	9007	6.16E-01	Down	0.50	11549	0.81	0.02
rs6501880	84074	QRICH2	17	71801300	1.56E-01	9008	3.40E-01	Up	0.95	9524	0.81	0.05
rs494734	57403	RAB22A	20	56341969	1.56E-01	9009	1.70E-02	Down	2.39	4939	0.81	0.18
rs166451	148223	C19orf25	19	1426391	1.56E-01	9010	4.71E-02	Up	1.99	5925	0.81	0.13
rs166451	10297	APC2	19	1426391	1.56E-01	9011	5.19E-01	Up	0.64	10855	0.81	0.03
rs166451	29882	ANAPC2	19	1426391	1.56E-01	9012	5.38E-01	Down	0.62	10994	0.81	0.03
rs8081168	79643	CHMP6	17	76585878	1.56E-01	9013	3.81E-01	Down	0.88	9839	0.81	0.04
rs7868842	203197	C9orf91	9	114440283	1.56E-01	9014	1.12E-24	Up	10.24	120	0.81	2.39
rs9206	57621	ZBTB2	6	151771312	1.56E-01	9015	3.82E-01	Up	0.87	9852	0.81	0.04
rs2055498	260293	CYP4X1	1	47201021	1.56E-01	9016	2.12E-07	Down	5.19	1381	0.81	0.67
rs11985037	10987	COPS5	8	68146350	1.56E-01	9017	3.75E-03	Up	2.90	3893	0.81	0.24
rs7789805	9238	TBRG4	7	44926546	1.56E-01	9018	6.12E-01	Up	0.51	11522	0.81	0.02
rs5944784	84460	ZMAT1		100980864	1.56E-01	9019	3.13E-01	Up	1.01	9296	0.81	0.05
rs2089402	79567	FAM65A	16	66107247	1.56E-01	9020	4.40E-02	Down	2.01	5852	0.81	0.14
rs873308	6006	RHCE	1	25503971	1.57E-01	9021	1.37E-01	Up	1.49	7539	0.81	0.09
rs6697826	8682	PEA15	1	156979308	1.57E-01	9022	2.46E-14	Down	7.62	434	0.80	1.36
rs3759387	4598	MVK	12	108475187	1.57E-01	9023	5.45E-02	Down	1.92	6099	0.80	0.13
rs7222840	2793	GNGT2	17	44635914	1.57E-01	9024	7.11E-01	Down	0.37	12132	0.80	0.01
rs4610966	163589	TDRD5	1	176329836	1.57E-01	9025	8.35E-01	Down	0.21	12878	0.80	0.01
rs1675517	26339	OR5K1	3	99707635	1.57E-01	9026	8.48E-01	Up	0.19	12938	0.80	0.01
rs12713820	56655	POLE4	2	75101679	1.57E-01	9027	9.06E-01	Up	0.12	13286	0.80	0.00
rs11263839	26523	EIF2C1	1	36063818	1.57E-01	9028	5.83E-04	Up	3.44	3017	0.80	0.32
rs6138482	30813	VSX1	20	25007442	1.57E-01	9029	1.20E-01	Up	1.55	7298	0.80	0.09
rs3811454	7170	TPM3	1	150977212	1.57E-01	9030	8.81E-01	Up	0.15	13137	0.80	0.01
rs8106699	4701	NDUFA7	19	8305272	1.57E-01	9031	6.92E-07	Up	4.96	1521	0.80	0.62
rs2710696	9840	KIAA0748	12	53683341	1.57E-01	9032	3.22E-01	Up	0.99	9374	0.80	0.05
rs243404	6455	SH3GL1	19	4335674	1.57E-01	9033	3.64E-03	Down	2.91	3881	0.80	0.24
rs243404	10036	CHAF1A	19	4335674	1.57E-01	9034	8.51E-01	Up	0.19	12960	0.80	0.01
rs2555575	137872	ADHFE1	8	67538073	1.58E-01	9035	2.47E-04	Down	3.67	2705	0.80	0.36
rs11077773	10476	ATP5H	17	70571668	1.58E-01	9036	7.91E-03	Up	2.66	4334	0.80	0.21
rs11077773	23510	KCTD2	17	70571668	1.58E-01	9037	4.85E-02	Down	1.97	5959	0.80	0.13
rs10935282	83850	FAM62C	3	139673930	1.58E-01	9038	1.23E-01	Down	1.54	7348	0.80	0.09
rs7787329	168374	ZNF92	7	64279352	1.58E-01	9039	6.81E-03	Up	2.71	4243	0.80	0.22
rs1832739	2213	FCGR2B	1	158387000	1.58E-01	9040	1.36E-02	Up	2.47	4729	0.80	0.19
rs1832739	9103	FCGR2C	1	158387000	1.58E-01	9041	5.68E-01	Up	0.57	11229	0.80	0.02
rs831271	573	BAG1	9	33260748	1.58E-01	9042	3.56E-06	Down	4.64	1765	0.80	0.54
rs50871	147700	KLC3	19	50554355	1.58E-01	9043	1.12E-01	Down	1.59	7172	0.80	0.10
rs5926203	168400	DDX53		22779003	1.58E-01	9044	8.36E-01	Down	0.21	12883	0.80	0.01
rs7121818	8534	CHST1	11	45641133	1.58E-01	9045	1.28E-02	Up	2.49	4674	0.80	0.19
rs2488264	84569	LYZL1	10	29609361	1.58E-01	9046	9.68E-01	Down	0.04	13685	0.80	0.00
rs4722670	3207	HOXA11	7	27002789	1.58E-01	9047	3.31E-01	Down	0.97	9456	0.80	0.05
rs9959968	147495	APCDD1	18	10427781	1.58E-01	9048	9.06E-14	Down	7.45	470	0.80	1.30
rs7533306	149647	FAM71A	1	209186285	1.58E-01	9049	4.85E-01	Up	0.70	10615	0.80	0.03
rs995901	3229	HOXC13	12	52613281	1.58E-01	9050	9.43E-01	Up	0.07	13514	0.80	0.00
rs10130904	26175	C14orf109	14	92710201	1.58E-01	9051	7.39E-03	Up	2.68	4292	0.80	0.21
rs10130904	64112	MOAP1	14	92710201	1.58E-01	9052	1.29E-02	Up	2.49	4679	0.80	0.19
rs13299607	6709	SPTAN1	9	128451226	1.58E-01	9053	4.23E-09	Down	5.87	998	0.80	0.84
rs1071962	84164	ASCC2	22	28548695	1.58E-01	9054	1.94E-01	Down	1.30	8190	0.80	0.07
rs4072037	4580	MTX1	1	151975140	1.58E-01	9055	9.52E-07	Up	4.90	1568	0.80	0.60
rs4072037	7059	THBS3	1	151975140	1.58E-01	9056	1.03E-01	Up	1.63	7042	0.80	0.10
rs4072037	4582	MUC1	1	151975140	1.58E-01	9057	2.02E-01	Down	1.28	8277	0.80	0.07
rs11010252	8325	FZD8	10	35987557	1.58E-01	9058	1.22E-17	Up	8.56	283	0.80	1.69
rs1024611	6354	CCL7	17	29603901	1.58E-01	9059	8.37E-01	Up	0.21	12888	0.80	0.01
rs11662125	10939	AFG3L2	18	12385904	1.58E-01	9060	4.04E-01	Up	0.84	10010	0.80	0.04
rs2485662	4000	LMNA	1	152896541	1.58E-01	9061	6.73E-03	Down	2.71	4238	0.80	0.22
rs10490692	3676	ITGA4	2	182206211	1.58E-01	9062	2.19E-01	Up	1.23	8448	0.80	0.07
rs2055543	10342	TFG	3	101907418	1.58E-01	9063	2.29E-01	Down	1.20	8553	0.80	0.06
rs2154659	9166	EBAG9	8	110653350	1.58E-01	9064	2.46E-01	Up	1.16	8722	0.80	0.06
rs3794977	3036	HAS1	19	56920571	1.59E-01	9065	3.70E-01	Up	0.90	9768	0.80	0.04
rs2126579	55074	OXR1	8	107785629	1.59E-01	9066	3.80E-02	Down	2.07	5706	0.80	0.14
rs1428175	147686	ZNF418	19	63157760	1.59E-01	9067	3.89E-01	Up	0.86	9905	0.80	0.04

rs6827722	57495	KIAA1239	4	37239603	1.59E-01	9068	6.52E-01	Down	0.45	11782	0.80	0.02
rs12689122	100131816	UBE2DNL		83997948	1.59E-01	9069	3.08E-01	Down	1.02	9248	0.80	0.05
rs266717	5984	RFC4	3	188013186	1.59E-01	9070	1.39E-04	Up	3.81	2526	0.80	0.39
rs2790108	23528	ZNF281	1	197101353	1.59E-01	9071	2.09E-02	Up	2.31	5112	0.80	0.17
rs541098	1133	CHRM5	15	32145617	1.59E-01	9072	9.18E-02	Down	1.69	6858	0.80	0.10
rs838530	51179	HAO2	1	119609541	1.59E-01	9073	8.60E-01	Up	0.18	13013	0.80	0.01
rs702104	23291	FBXW11	5	171211693	1.59E-01	9074	1.27E-01	Up	1.53	7408	0.80	0.09
rs3777632	22875	ENPP4	6	46220846	1.59E-01	9075	2.69E-03	Up	3.00	3726	0.80	0.26
rs1321196	4018	LPA	6	161052253	1.59E-01	9076	2.89E-02	Up	2.18	5422	0.80	0.15
rs760867	9843	HEPH		65163304	1.59E-01	9077	2.18E-04	Down	3.70	2660	0.80	0.37
rs267733	29956	LASS2	1	147771909	1.59E-01	9078	4.43E-12	Up	6.93	620	0.80	1.14
rs267733	8416	ANXA9	1	147771909	1.59E-01	9079	7.25E-01	Up	0.35	12230	0.80	0.01
rs267733	55793	FAM63A	1	147771909	1.59E-01	9080	7.53E-01	Down	0.32	12396	0.80	0.01
rs1140195	3681	ITGAD	16	31301680	1.59E-01	9081	2.49E-01	Up	1.15	8759	0.80	0.06
rs1864466	79800	ALS2CR8	2	203681963	1.59E-01	9082	2.76E-05	Down	4.19	2152	0.80	0.46
rs10506070	10526	IPO8	12	30720941	1.59E-01	9083	1.74E-03	Down	3.13	3461	0.80	0.28
rs7696175	7096	TLR1	4	38643552	1.59E-01	9084	9.54E-01	Up	0.06	13583	0.80	0.00
rs7696175	10333	TLR6	4	38643552	1.59E-01	9085	9.93E-01	Up	0.01	13868	0.80	0.00
rs2274932	55370	PPP4R1L	20	56241198	1.59E-01	9086	9.23E-01	Down	0.10	13392	0.80	0.00
rs6568828	222537	HS3ST5	6	114491394	1.59E-01	9087	8.16E-01	Up	0.23	12764	0.80	0.01
rs33700	153396	TMEM161B	5	87526664	1.59E-01	9088	3.11E-06	Up	4.66	1747	0.80	0.55
rs7624355	3827	KNG1	3	187910042	1.59E-01	9089	2.44E-01	Down	1.16	8706	0.80	0.06
rs2402198	9950	GOLGA5	14	92348050	1.59E-01	9090	1.40E-03	Up	3.19	3351	0.80	0.29
rs6734498		B3GALT1	2	168537548	1.59E-01	9091	1.14E-01	Down	1.58	7200	0.80	0.09
rs13268487	5516	PPP2CB	8	30766081	1.59E-01	9092	3.97E-17	Down	8.41	302	0.80	1.64
rs11587479	9341	VAMP3	1	7757351	1.59E-01	9093	1.26E-08	Down	5.69	1076	0.80	0.79
rs7712322	10622	POLR3G	5	89813651	1.59E-01	9094	8.60E-02	Up	1.72	6737	0.80	0.11
rs3754822	80219	COQ10B	2	198152820	1.59E-01	9095	9.76E-01	Down	0.03	13738	0.80	0.00
rs6542658	6201	RPS7	2	3109191	1.59E-01	9096	3.76E-16	Up	8.17	340	0.80	1.54
rs2240025	7710	ZNF154	19	62903856	1.59E-01	9097	6.34E-01	Up	0.48	11667	0.80	0.02
rs8081176	57714	KIAA1618	17	75898582	1.60E-01	9098	5.21E-01	Down	0.64	10865	0.80	0.03
rs2292702	2967	GTF2H3	12	122655008	1.60E-01	9099	7.97E-01	Down	0.26	12649	0.80	0.01
rs2772179	11193	WBP4	13	40560028	1.60E-01	9100	2.21E-12	Down	7.02	587	0.80	1.17
rs3181096	940	CD28	2	204395598	1.60E-01	9101	1.56E-02	Up	2.42	4851	0.80	0.18
rs7207125	10350	ABCA9	17	64518609	1.60E-01	9102	3.42E-01	Down	0.95	9542	0.80	0.05
rs11057065	55810	FOXJ2	12	8099511	1.60E-01	9103	1.25E-05	Down	4.37	2002	0.80	0.49
rs4679834	5918	RARRES1	3	159935297	1.60E-01	9104	4.18E-10	Down	6.25	836	0.80	0.94
rs16965246	85464	SSH2	17	25173931	1.60E-01	9105	2.34E-01	Up	1.19	8603	0.80	0.06
rs1359893	163732	CITED4	1	41007758	1.60E-01	9106	1.47E-04	Down	3.80	2542	0.80	0.38
rs197367	23366	KIAA0895	7	36219096	1.60E-01	9107	2.51E-03	Up	3.02	3689	0.80	0.26
rs158898	79646	PANK3	5	167913103	1.60E-01	9108	9.61E-01	Up	0.05	13626	0.80	0.00
rs12910984	1138	CHRNA5	15	76678682	1.60E-01	9109	2.13E-03	Up	3.07	3569	0.80	0.27
rs2084756	55835	CENPJ	13	24406649	1.60E-01	9110	3.86E-02	Down	2.07	5716	0.80	0.14
rs6120777	2937	GSS	20	33023833	1.60E-01	9111	1.41E-01	Down	1.47	7594	0.80	0.08
rs3027247	9212	AURKB	17	8071592	1.60E-01	9112	1.85E-03	Up	3.11	3493	0.80	0.27
rs3027247	80169	C17orf68	17	8071592	1.60E-01	9113	2.15E-01	Down	1.24	8405	0.80	0.07
rs556439	3298	HSF2	6	122786708	1.60E-01	9114	7.98E-01	Up	0.26	12659	0.80	0.01
rs8076343	54883	CCDC49	17	34235398	1.60E-01	9115	3.91E-03	Down	2.89	3914	0.80	0.24
rs266858	55554	KLK15	19	56031774	1.60E-01	9116	5.60E-01	Up	0.58	11165	0.80	0.03
rs266858	3816	KLK1	19	56031774	1.60E-01	9117	7.82E-01	Down	0.28	12557	0.80	0.01
rs6700677	79078	C1orf50	1	42892582	1.60E-01	9118	3.91E-03	Up	2.89	3915	0.79	0.24
rs6700677	64175	LEPRE1	1	42892582	1.60E-01	9119	3.92E-03	Up	2.88	3916	0.79	0.24
rs6700677	149461	CLDN19	1	42892582	1.60E-01	9120	1.77E-01	Up	1.35	7986	0.79	0.08
rs11211383	2275	FHL3	1	38151449	1.60E-01	9121	2.36E-04	Down	3.68	2689	0.79	0.36
rs11211383	51118	UTP11L	1	38151449	1.60E-01	9122	8.00E-01	Down	0.25	12674	0.79	0.01
rs2134847	124599	CD300LB	17	70056817	1.60E-01	9123	2.56E-01	Up	1.14	8820	0.79	0.06
rs12205313	5689	PSMB1	6	170769068	1.61E-01	9124	1.57E-02	Up	2.42	4856	0.79	0.18
rs12205313	6908	TBP	6	170769068	1.61E-01	9125	2.44E-01	Up	1.16	8704	0.79	0.06
rs207426	7498	XDH	2	31461610	1.61E-01	9126	9.87E-02	Down	1.65	6975	0.79	0.10
rs518673	54888	NSUN2	5	6682930	1.61E-01	9127	5.96E-07	Up	4.99	1503	0.79	0.62
rs7530038	148581	UBE2U	1	64417909	1.61E-01	9128	6.36E-01	Up	0.47	11676	0.79	0.02
rs167769	6778	STAT6	12	55790042	1.61E-01	9129	3.73E-01	Down	0.89	9788	0.79	0.04
rs2186990	114908	TMEM123	11	101795811	1.61E-01	9130	7.90E-09	Up	5.76	1043	0.79	0.81
rs4702340	84246	MED10	5	6436710	1.61E-01	9131	4.81E-01	Up	0.71	10578	0.79	0.03
rs7483870	161	AP2A2	11	966019	1.61E-01	9132	9.72E-06	Up	4.42	1947	0.79	0.50
rs10133592	78990	OTUB2	14	93553813	1.61E-01	9133	5.09E-01	Up	0.66	10777	0.79	0.03
rs10410404	29924	EPN1	19	60897449	1.61E-01	9134	5.70E-01	Down	0.57	11243	0.79	0.02
rs11586824	23499	MACF1	1	39486255	1.61E-01	9135	6.22E-02	Down	1.86	6282	0.79	0.12
rs12450695	6398	SECTM1	17	77896874	1.61E-01	9136	7.08E-03	Down	2.69	4266	0.79	0.22
rs339716	131377	KBTBD5	3	42686659	1.61E-01	9137	3.30E-01	Up	0.98	9436	0.79	0.05
rs432788	341880	SLC35F4	14	57094033	1.61E-01	9138	3.21E-02	Down	2.14	5531	0.79	0.15
rs3218660	27434	POLM	7	43886113	1.61E-01	9139	1.94E-01	Up	1.30	8193	0.79	0.07
rs3218660	5224	PGAM2	7	43886113	1.61E-01	9140	5.27E-01	Down	0.63	10921	0.79	0.03
rs6065904	128497	C20orf165	20	43968058	1.61E-01	9141	1.97E-01	Down	1.29	8221	0.79	0.07
rs6065904	140825	NEURL2	20	43968058	1.61E-01	9142	7.49E-01	Down	0.32	12370	0.79	0.01
rs8102196	147923	ZNF420	19	42274044	1.61E-01	9143	5.32E-01	Up	0.62	10956	0.79	0.03
rs7734102	9794	MAML1	5	179086403	1.61E-01	9144	5.28E-01	Up	0.63	10928	0.79	0.03
rs10005483	84076	TKTL2	4	164741814	1.61E-01	9145	6.18E-02	Up	1.87	6276	0.79	0.12
rs10459647	585	BBS4	15	70822294	1.61E-01	9146	5.12E-10	Up	6.21	858	0.79	0.93
rs7701443	2908	NR3C1	5	142772843	1.62E-01	9147	8.65E-17	Down	8.32	317	0.79	1.61
rs3115761	7175	TPR	1	183079350	1.62E-01	9148	9.02E-02	Down	1.69	6820	0.79	0.10

rs5746014	7133	TNFRSF1B	1	12185154	1.62E-01	9149	3.42E-04	Down	3.58	2826	0.79	0.35
rs10799	55139	ANKZF1	2	219910443	1.62E-01	9150	9.09E-04	Up	3.32	3177	0.79	0.30
rs10799	79065	ATG9A	2	219910443	1.62E-01	9151	3.75E-02	Down	2.08	5690	0.79	0.14
rs10799	10058	ABCB6	2	219910443	1.62E-01	9152	9.54E-01	Down	0.06	13584	0.79	0.00
rs9577426	23263	MCF2L	13	112723751	1.62E-01	9153	1.25E-05	Up	4.37	2001	0.79	0.49
rs3782979	55270	NUDT15	13	47517108	1.62E-01	9154	1.04E-02	Down	2.56	4544	0.79	0.20
rs2074108	6521	SLC4A1	17	39691675	1.62E-01	9155	5.34E-03	Up	2.79	4107	0.79	0.23
rs1654531	5653	KLK6	19	56167461	1.62E-01	9156	6.72E-04	Up	3.40	3065	0.79	0.32
rs1654531	5650	KLK7	19	56167461	1.62E-01	9157	2.68E-01	Down	1.11	8932	0.79	0.06
rs11485101	204851	HIPK1	1	114214010	1.62E-01	9158	1.81E-03	Down	3.12	3481	0.79	0.27
rs2243682	1062	CENPE	4	104417146	1.62E-01	9159	8.50E-03	Up	2.63	4383	0.79	0.21
rs2833145	337880	KRTAP11-1	21	31179148	1.62E-01	9160	8.79E-03	Down	2.62	4412	0.79	0.21
rs4898457	3897	L1CAM		152679203	1.62E-01	9161	9.29E-01	Down	0.09	13429	0.79	0.00
rs4898457	554	AVPR2		152679203	1.62E-01	9162	9.37E-01	Up	0.08	13471	0.79	0.00
rs2807171	60506	NYX		41099173	1.62E-01	9163	9.18E-02	Down	1.69	6856	0.79	0.10
rs7789085	7205	TRIP6	7	100117477	1.62E-01	9164	3.13E-25	Down	10.38	111	0.79	2.45
rs7789085	56996	SLC12A9	7	100117477	1.62E-01	9165	1.16E-02	Up	2.52	4614	0.79	0.19
rs3898677	80765	STARD5	15	79383617	1.63E-01	9166	4.10E-02	Down	2.04	5775	0.79	0.14
rs941289	7551	ZNF3	7	99323142	1.63E-01	9167	5.99E-06	Up	4.53	1843	0.79	0.52
rs941289	10980	COPS6	7	99323142	1.63E-01	9168	9.88E-01	Up	0.02	13829	0.79	0.00
rs4239761	5111	PCNA	20	5062584	1.63E-01	9169	8.16E-02	Up	1.74	6662	0.79	0.11
rs16943581	23347	SMCHD1	18	2629683	1.63E-01	9170	2.07E-02	Down	2.31	5103	0.79	0.17
rs11666426	79788	ZNF665	19	58386359	1.63E-01	9171	2.79E-01	Down	1.08	9030	0.79	0.06
rs11668932	5680	PSG11	19	48205499	1.63E-01	9172	1.63E-01	Up	1.40	7851	0.79	0.08
rs9382107	114327	EFHC1	6	52393916	1.63E-01	9173	2.25E-03	Up	3.05	3615	0.79	0.26
rs6488271	23710	GABARAPL1	12	10241957	1.63E-01	9174	1.49E-19	Down	9.05	213	0.79	1.88
rs6488271	120939	C12orf59	12	10241957	1.63E-01	9175	3.31E-02	Up	2.13	5568	0.79	0.15
rs273249	10561	IFI44	1	78836044	1.63E-01	9176	2.61E-01	Up	1.12	8870	0.79	0.06
rs2071306	2564	GABRE		150808747	1.63E-01	9177	2.36E-14	Down	7.63	433	0.79	1.36
rs2091817	3754	KCNF1	2	10997236	1.63E-01	9178	1.51E-01	Down	1.43	7696	0.79	0.08
rs411805	4677	NARS	18	53449884	1.63E-01	9179	2.79E-01	Down	1.08	9029	0.79	0.06
rs7404339	1003	CDH5	16	64973293	1.63E-01	9180	3.49E-02	Down	2.11	5619	0.79	0.15
rs17115100	1586	CYP17A1	10	104581383	1.63E-01	9181	4.21E-01	Down	0.80	10130	0.79	0.04
rs2887603	81029	WNT5B	12	1628004	1.63E-01	9182	7.57E-06	Down	4.48	1891	0.79	0.51
rs17305549	57663	USP29	19	62338030	1.63E-01	9183	4.04E-01	Down	0.83	10012	0.79	0.04
rs10508204	3422	IDI1	10	1074360	1.63E-01	9184	6.14E-03	Down	2.74	4180	0.79	0.22
rs10505752	4973	OLR1	12	10224614	1.63E-01	9185	1.38E-06	Up	4.83	1614	0.79	0.59
rs5752196	157	ADRBK2	22	24396112	1.63E-01	9186	4.33E-06	Down	4.60	1794	0.79	0.54
rs5918213	8573	CASK		41250159	1.63E-01	9187	5.50E-02	Up	1.92	6113	0.79	0.13
rs17637266	55759	WDR12	2	203598410	1.63E-01	9188	5.54E-03	Up	2.77	4137	0.79	0.23
rs1976165	57649	PHF12	17	24310977	1.63E-01	9189	8.00E-11	Up	6.50	743	0.79	1.01
rs6538949	79738	BBS10	12	75257894	1.63E-01	9190	9.89E-01	Down	0.01	13835	0.79	0.00
rs4660758	533	ATP6V0B	1	44093972	1.63E-01	9191	3.46E-08	Up	5.52	1180	0.79	0.75
rs2071229	6173	RPL36A		100447719	1.63E-01	9192	4.29E-05	Up	4.09	2243	0.79	0.44
rs10846516	55206	SBNO1	12	122328349	1.64E-01	9193	2.92E-01	Down	1.05	9130	0.79	0.05
rs1057278	285367	RPUSD3	3	9851919	1.64E-01	9194	2.43E-01	Up	1.17	8691	0.79	0.06
rs1732771	8437	RASAL1	12	112006548	1.64E-01	9195	7.19E-02	Up	1.80	6466	0.79	0.11
rs696833	4883	NPR3	5	32762394	1.64E-01	9196	1.68E-07	Up	5.23	1354	0.79	0.68
rs1757171	154467	C6orf129	6	37595022	1.64E-01	9197	2.62E-02	Up	2.22	5332	0.79	0.16
rs4143897	26520	TIMM9	14	57976159	1.64E-01	9198	9.90E-03	Up	2.58	4501	0.79	0.20
rs3123630	80350	LPAL2	6	160881464	1.64E-01	9199	9.72E-01	Up	0.03	13714	0.79	0.00
rs4804000	6510	SLC1A5	19	51968294	1.64E-01	9200	6.16E-01	Down	0.50	11546	0.79	0.02
rs1111102	6746	SSR2	1	152806529	1.64E-01	9201	1.29E-15	Up	8.00	360	0.79	1.49
rs7253540	126321	C19orf28	19	3490488	1.64E-01	9202	5.98E-03	Up	2.75	4170	0.79	0.22
rs7253540	51343	FZR1	19	3490488	1.64E-01	9203	2.22E-02	Down	2.29	5168	0.79	0.17
rs6600436	10946	SF3A3	1	38138593	1.64E-01	9204	1.76E-03	Down	3.13	3467	0.79	0.28
rs1014324	51389	RWDD1	6	116994554	1.64E-01	9205	7.82E-01	Down	0.28	12558	0.79	0.01
rs6789043	9852	EPM2AIP1	3	37000871	1.64E-01	9206	2.69E-01	Down	1.11	8944	0.79	0.06
rs11584887	114971	PTPMT1	1	156574506	1.64E-01	9207	3.51E-02	Up	2.11	5627	0.79	0.15
rs11584887	54935	DUSP23	1	156574506	1.64E-01	9208	4.97E-01	Down	0.68	10693	0.79	0.03
rs2074982	5714	PSMD8	19	43564376	1.64E-01	9209	3.21E-01	Up	0.99	9365	0.78	0.05
rs2074982	199720	GGN	19	43564376	1.64E-01	9210	8.58E-01	Down	0.18	12998	0.78	0.01
rs1566822	339761	CYP27C1	2	127703621	1.64E-01	9211	2.09E-01	Down	1.26	8343	0.78	0.07
rs10115552	2516	NR5A1	9	124354405	1.64E-01	9212	3.24E-01	Up	0.99	9400	0.78	0.05
rs352797	7991	TUSC3	8	15660060	1.64E-01	9213	8.60E-05	Up	3.93	2413	0.78	0.41
rs6914864	7754	ZNF204	6	27444468	1.64E-01	9214	3.90E-07	Down	5.07	1452	0.78	0.64
rs11032431	390035	OR52K3P	11	4458152	1.64E-01	9215	7.99E-02	Down	1.75	6631	0.78	0.11
rs6893145	7416	VDAC1	5	133356724	1.64E-01	9216	1.47E-03	Up	3.18	3376	0.78	0.28
rs2286336	60528	ELAC2	17	12856666	1.65E-01	9217	1.68E-01	Up	1.38	7896	0.78	0.08
rs959195	4190	MDH1	2	63715776	1.65E-01	9218	1.57E-02	Down	2.42	4855	0.78	0.18
rs2923144	10300	KATNB1	16	56316233	1.65E-01	9219	2.02E-01	Up	1.28	8274	0.78	0.07
rs10416445	11129	SFRS16	19	50248502	1.65E-01	9220	5.42E-01	Down	0.61	11031	0.78	0.03
rs8014577	79697	C14orf169	14	73033136	1.65E-01	9221	9.62E-01	Up	0.05	13631	0.78	0.00
rs789239	28976	ACAD9	3	130116506	1.65E-01	9222	5.16E-04	Up	3.47	2969	0.78	0.33
rs300259	8507	ENC1	5	73959815	1.65E-01	9223	1.51E-21	Up	9.57	167	0.78	2.08
rs3770472	3488	IGFBP5	2	217384241	1.65E-01	9224	2.97E-03	Down	2.97	3773	0.78	0.25
rs10876575	90070	LACRT	12	53329500	1.65E-01	9225	1.94E-01	Down	1.30	8191	0.78	0.07
rs747526	1474	CST6	11	65532647	1.65E-01	9226	3.32E-02	Up	2.13	5569	0.78	0.15
rs747526	8815	BANF1	11	65532647	1.65E-01	9227	5.44E-02	Down	1.92	6098	0.78	0.13
rs747526	117144	CATSPER1	11	65532647	1.65E-01	9228	9.00E-01	Up	0.13	13241	0.78	0.00
rs1551625	6904	TBCD	17	78470842	1.65E-01	9229	1.87E-04	Up	3.74	2602	0.78	0.37

rs2304196	5623	PSPN	19	6343006	1.65E-01	9230	7.55E-01	Up	0.31	12409	0.78	0.01
rs3844453	91663	MYADM	19	59049576	1.65E-01	9231	2.36E-06	Down	4.72	1693	0.78	0.56
rs1075728	478	ATP1A3	19	47154628	1.65E-01	9232	6.61E-02	Down	1.84	6363	0.78	0.12
rs1075728	10567	RABAC1	19	47154628	1.65E-01	9233	3.76E-01	Down	0.89	9806	0.78	0.04
rs9306955	10744	PTTG2	4	37793416	1.65E-01	9234	5.14E-01	Up	0.65	10816	0.78	0.03
rs1054899	7915	ALDH5A1	6	24642172	1.65E-01	9235	5.51E-01	Down	0.60	11084	0.78	0.03
rs16940987	54778	RNF111	15	57141155	1.65E-01	9236	5.32E-03	Down	2.79	4105	0.78	0.23
rs12582216	11226	GALNT6	12	50034181	1.65E-01	9237	5.39E-05	Down	4.04	2305	0.78	0.43
rs12582216	1990	ELA1	12	50034181	1.65E-01	9238	4.50E-01	Up	0.76	10346	0.78	0.03
rs10119678	3443	IFNA6	9	21330786	1.65E-01	9239	7.15E-03	Up	2.69	4271	0.78	0.21
rs10119678	55958	KLHL9	9	21330786	1.65E-01	9240	7.69E-01	Down	0.29	12488	0.78	0.01
rs1024782	5536	PPP5C	19	51579407	1.65E-01	9241	2.27E-01	Up	1.21	8531	0.78	0.06
rs11046186	3764	KCNJ8	12	21829811	1.65E-01	9242	5.36E-14	Down	7.52	454	0.78	1.33
rs1129980	2239	GPC4		132164392	1.65E-01	9243	1.34E-02	Down	2.47	4713	0.78	0.19
rs12728900	79727	LIN28	1	26430949	1.65E-01	9244	7.23E-01	Down	0.35	12216	0.78	0.01
rs12728900	79947	DHDDS	1	26430949	1.65E-01	9245	8.27E-01	Down	0.22	12832	0.78	0.01
rs3789482	11332	ACOT7	1	6331638	1.65E-01	9246	1.80E-03	Down	3.12	3476	0.78	0.27
rs6982014	79139	DERL1	8	124092802	1.66E-01	9247	6.38E-01	Down	0.47	11688	0.78	0.02
rs2364354	26251	KCNG2	18	75752524	1.66E-01	9248	9.30E-02	Up	1.68	6885	0.78	0.10
rs740422	27440	CECR5	22	15993846	1.66E-01	9249	1.33E-06	Up	4.84	1610	0.78	0.59
rs6744975	7141	TNP1	2	217562255	1.66E-01	9250	1.02E-01	Down	1.64	7032	0.78	0.10
rs752261	6610	SMPD2	6	109877562	1.66E-01	9251	9.49E-01	Down	0.06	13542	0.78	0.00
rs9536079	26586	CKAP2	13	51928566	1.66E-01	9252	4.85E-03	Up	2.82	4044	0.78	0.23
rs408359	9374	PPT2	6	32249861	1.66E-01	9253	5.87E-04	Down	3.44	3021	0.78	0.32
rs408359	80864	EGFL8	6	32249861	1.66E-01	9254	6.03E-01	Down	0.52	11448	0.78	0.02
rs7243052	84552	PARD6G	18	76069787	1.66E-01	9255	1.78E-06	Down	4.78	1652	0.78	0.58
rs11704395	284904	SEC14L4	22	29218500	1.66E-01	9256	2.19E-01	Down	1.23	8442	0.78	0.07
rs152186	51194	IPO11	5	61755263	1.66E-01	9257	2.17E-02	Down	2.30	5149	0.78	0.17
rs152186	27292	DIMT1L	5	61755263	1.66E-01	9258	8.62E-01	Down	0.17	13018	0.78	0.01
rs2411163	26574	AATF	17	32484430	1.66E-01	9259	2.64E-02	Down	2.22	5336	0.78	0.16
rs761002	7052	TGM2	20	36230414	1.66E-01	9260	9.90E-01	Up	0.01	13841	0.78	0.00
rs10406431	2696	GIPR	19	50848859	1.66E-01	9261	6.22E-01	Up	0.49	11596	0.78	0.02
rs6446909	2926	GRSF1	4	72089741	1.66E-01	9262	3.80E-08	Up	5.50	1196	0.78	0.74
rs738089	91179	SCARF2	22	19097152	1.66E-01	9263	2.45E-01	Down	1.16	8716	0.78	0.06
rs4808722	3780	KCNN1	19	17908283	1.66E-01	9264	4.07E-02	Down	2.05	5764	0.78	0.14
rs319598	84105	PCBD2	5	134268134	1.66E-01	9265	5.78E-01	Down	0.56	11295	0.78	0.02
rs177614	51635	DHRS7	14	59706291	1.66E-01	9266	2.07E-01	Up	1.26	8324	0.78	0.07
rs8058578	10847	SRCAP	16	30633749	1.66E-01	9267	4.02E-01	Down	0.84	9998	0.78	0.04
rs1291602	51735	RAPGEF6	5	130794561	1.66E-01	9268	1.21E-01	Up	1.55	7322	0.78	0.09
rs7071536	54522	ANKRD16	10	5943524	1.67E-01	9269	8.91E-06	Up	4.44	1923	0.78	0.51
rs4383153	7369	UMOD	16	20246123	1.67E-01	9270	5.22E-01	Up	0.64	10873	0.78	0.03
rs959678	11055	ZPBP	7	49837871	1.67E-01	9271	4.30E-01	Down	0.79	10198	0.78	0.04
rs1920045	23468	CBX5	12	52956665	1.67E-01	9272	2.34E-02	Up	2.27	5221	0.78	0.16
rs323565	6167	RPL37	5	40884560	1.67E-01	9273	2.87E-06	Up	4.68	1726	0.78	0.55
rs323565	84674	CARD6	5	40884560	1.67E-01	9274	6.18E-01	Down	0.50	11561	0.78	0.02
rs5967281	54457	TAF7L		100321131	1.67E-01	9275	2.30E-01	Down	1.20	8559	0.78	0.06
rs13447427	60	ACTB	7	5338675	1.67E-01	9276	4.71E-07	Down	5.04	1474	0.78	0.63
rs10489683	115350	FCRL1	1	154622735	1.67E-01	9277	8.83E-01	Down	0.15	13145	0.78	0.01
rs9938539	1723	DHODH	16	70588081	1.67E-01	9278	6.20E-01	Up	0.50	11582	0.78	0.02
rs3195944	54858	PGPEP1	19	18337711	1.67E-01	9279	7.10E-01	Up	0.37	12130	0.78	0.01
rs5771086	56666	PANX2	22	48898299	1.67E-01	9280	2.92E-01	Down	1.05	9128	0.78	0.05
rs10794449	80324	PUS1	12	131088474	1.67E-01	9281	3.82E-01	Up	0.87	9849	0.78	0.04
rs1878583	8604	SLC25A12	2	172550808	1.67E-01	9282	1.18E-05	Down	4.38	1983	0.78	0.49
rs10860361	317	APAF1	12	97631965	1.67E-01	9283	8.21E-01	Up	0.23	12794	0.78	0.01
rs2132397	51463	GPR89B	1	144606374	1.67E-01	9284	1.62E-05	Up	4.31	2054	0.78	0.48
rs11698371	140856	C20orf79	20	18731521	1.67E-01	9285	2.62E-01	Down	1.12	8884	0.78	0.06
rs2132397	2703	GJA8	1	144606374	1.67E-01	9286	7.91E-01	Down	0.26	12618	0.78	0.01
rs12137333	199857	ALG14	1	95190147	1.67E-01	9287	6.88E-02	Up	1.82	6406	0.78	0.12
rs4793621	11143	MYST2	17	45218367	1.67E-01	9288	3.15E-05	Down	4.16	2180	0.78	0.45
rs730592	3903	LAIR1	19	59555976	1.68E-01	9289	6.47E-01	Down	0.46	11757	0.78	0.02
rs9814951	5210	PFKFB4	3	48585929	1.68E-01	9290	5.63E-01	Down	0.58	11182	0.78	0.02
rs2172358	23219	FBXO28	1	220634665	1.68E-01	9291	9.32E-03	Up	2.60	4455	0.78	0.20
rs11210824	128218	TMEM125	1	43404289	1.68E-01	9292	1.58E-04	Up	3.78	2562	0.78	0.38
rs3821869	55540	IL17RB	3	53868880	1.68E-01	9293	2.40E-07	Up	5.17	1392	0.78	0.66
rs6829581	53371	NUP54	4	77416309	1.68E-01	9294	6.00E-01	Down	0.52	11433	0.78	0.02
rs7075288	8187	ZNF239	10	43390593	1.68E-01	9295	9.94E-03	Up	2.58	4507	0.77	0.20
rs9636161	147947	ZNF542	19	61592298	1.68E-01	9296	6.25E-10	Up	6.20	864	0.77	0.92
rs9636161	147948	ZNF582	19	61592298	1.68E-01	9297	5.21E-03	Up	2.79	4097	0.77	0.23
rs9636161	147949	ZNF583	19	61592298	1.68E-01	9298	2.96E-01	Up	1.04	9166	0.77	0.05
rs6510624	148229	ATP8B3	19	1734794	1.68E-01	9299	1.39E-01	Down	1.48	7563	0.77	0.09
rs7303722	2026	ENO2	12	6904084	1.68E-01	9300	1.08E-07	Down	5.31	1301	0.77	0.70
rs7303722	113246	C12orf57	12	6904084	1.68E-01	9301	7.44E-01	Up	0.33	12340	0.77	0.01
rs2577620	9648	GCC2	2	108548470	1.68E-01	9302	7.49E-04	Up	3.37	3110	0.77	0.31
rs16940970	5501	PPP1CC	12	109627835	1.68E-01	9303	1.09E-02	Up	2.55	4568	0.77	0.20
rs6946228	6624	FSCN1	7	5392319	1.68E-01	9304	2.34E-03	Down	3.04	3644	0.77	0.26
rs2194141	84249	PSD2	5	139153625	1.68E-01	9305	5.64E-01	Up	0.58	11190	0.77	0.02
rs6826373	8929	PHOX2B	4	41587388	1.68E-01	9306	9.65E-01	Up	0.04	13651	0.77	0.00
rs10896104	55690	PACS1	11	65758445	1.68E-01	9307	2.23E-01	Up	1.22	8486	0.77	0.07
rs4979935	11128	POLR3A	10	79405479	1.68E-01	9308	7.09E-03	Up	2.69	4269	0.77	0.21
rs8110348	7766	ZNF223	19	49245220	1.68E-01	9309	7.45E-01	Up	0.33	12342	0.77	0.01
rs10084615	140578	CHODL	21	18568140	1.68E-01	9310	9.66E-01	Up	0.04	13666	0.77	0.00

rs8067516	5023	P2RX1	17	3784997	1.69E-01	9311	3.44E-08	Down	5.52	1181	0.77	0.75
rs8067516	489	ATP2A3	17	3784997	1.69E-01	9312	2.99E-01	Down	1.04	9184	0.77	0.05
rs7922546	118856	MMP21	10	127450743	1.69E-01	9313	2.23E-01	Down	1.22	8491	0.77	0.07
rs3827351	11020	RABL4	22	35481740	1.69E-01	9314	4.75E-02	Up	1.98	5934	0.77	0.13
rs3856341	10314	LANCL1	2	211160153	1.69E-01	9315	3.61E-02	Down	2.10	5650	0.77	0.14
rs10487364	136991	ASZ1	7	116652171	1.69E-01	9316	1.24E-01	Down	1.54	7351	0.77	0.09
rs6734024	1653	DDX1	2	15668778	1.69E-01	9317	4.39E-02	Down	2.02	5847	0.77	0.14
rs2273918	9820	CUL7	6	43121640	1.69E-01	9318	3.89E-03	Up	2.89	3910	0.77	0.24
rs2273918	51069	MRPL2	6	43121640	1.69E-01	9319	7.54E-01	Up	0.31	12401	0.77	0.01
rs11573114	5431	POLR2B	4	57747222	1.69E-01	9320	1.38E-01	Down	1.48	7542	0.77	0.09
rs3779354	10898	CPSF4	7	98697079	1.69E-01	9321	6.16E-02	Up	1.87	6271	0.77	0.12
rs17462354	151306	GPBAR1	2	218935388	1.69E-01	9322	1.56E-02	Up	2.42	4852	0.77	0.18
rs11135109	23120	ATP10B	5	160032018	1.70E-01	9323	7.73E-01	Down	0.29	12519	0.77	0.01
rs7965892	5128	PCTK2	12	95197644	1.70E-01	9324	5.71E-01	Down	0.57	11252	0.77	0.02
rs3136247	2956	MSH6	2	47924750	1.70E-01	9325	7.92E-01	Down	0.26	12626	0.77	0.01
rs1718301	5053	PAH	12	101773660	1.70E-01	9326	4.04E-01	Up	0.83	10013	0.77	0.04
rs8102086	27134	TJP3	19	3703874	1.70E-01	9327	8.49E-05	Down	3.93	2409	0.77	0.41
rs5964151	1536	CYBB		37426946	1.70E-01	9328	1.47E-01	Up	1.45	7646	0.77	0.08
rs1299525	1508	CTSB	8	11759534	1.70E-01	9329	2.27E-35	Down	12.41	39	0.77	3.46
rs11878377	10498	CARM1	19	10826270	1.70E-01	9330	6.84E-03	Up	2.70	4245	0.77	0.22
rs281437	3386	ICAM4	19	10258238	1.70E-01	9331	4.83E-03	Down	2.82	4037	0.77	0.23
rs9296189	6428	SFRS3	6	36655373	1.70E-01	9332	2.94E-03	Down	2.97	3766	0.77	0.25
rs8108134	81616	ACSBG2	19	6075921	1.70E-01	9333	4.14E-02	Down	2.04	5782	0.77	0.14
rs10518294	117178	SSX2IP	1	84816904	1.70E-01	9334	1.23E-05	Down	4.37	1998	0.77	0.49
rs3020790	1852	DUSP9		152414503	1.70E-01	9335	6.00E-02	Down	1.88	6237	0.77	0.12
rs3796352	375346	TMEM110	3	52888319	1.70E-01	9336	2.25E-04	Down	3.69	2669	0.77	0.36
rs12977991	27338	UBE2S	19	60617312	1.70E-01	9337	1.77E-04	Up	3.75	2589	0.77	0.38
rs7210	51002	TPRKB	2	73868779	1.70E-01	9338	4.73E-02	Up	1.98	5931	0.77	0.13
rs11971753	57488	FAM62B	7	158071749	1.70E-01	9339	8.12E-01	Down	0.24	12732	0.77	0.01
rs6538254	196477	C12orf12	12	89847456	1.70E-01	9340	8.07E-02	Down	1.75	6641	0.77	0.11
rs9426910	80133	C1orf129	1	167645729	1.70E-01	9341	1.96E-01	Up	1.29	8212	0.77	0.07
rs6531779	404201	C4orf12	4	86230630	1.70E-01	9342	1.30E-01	Up	1.52	7445	0.77	0.09
rs16967028	55352	C17orf79	17	27214461	1.70E-01	9343	3.81E-01	Down	0.88	9843	0.77	0.04
rs7319446	65110	UPF3A	13	114047264	1.70E-01	9344	8.34E-04	Up	3.34	3149	0.77	0.31
rs1054174	6389	SDHA	5	257952	1.71E-01	9345	1.96E-01	Down	1.29	8219	0.77	0.07
rs10434724	56923	NMUR2	5	151758407	1.71E-01	9346	9.09E-02	Up	1.69	6846	0.77	0.10
rs3826700	79016	DDA1	19	17287501	1.71E-01	9347	5.16E-02	Up	1.95	6029	0.77	0.13
rs3826700	79575	ABHD8	19	17287501	1.71E-01	9348	6.32E-01	Down	0.48	11658	0.77	0.02
rs3826700	64981	MRPL34	19	17287501	1.71E-01	9349	9.31E-01	Down	0.09	13437	0.77	0.00
rs10934250	79691	QTRTD1	3	115295608	1.71E-01	9350	5.99E-01	Up	0.53	11427	0.77	0.02
rs12287250	29118	DDX25	11	125263496	1.71E-01	9351	7.97E-02	Down	1.75	6628	0.77	0.11
rs12287250	83480	PUS3	11	125263496	1.71E-01	9352	1.24E-01	Up	1.54	7357	0.77	0.09
rs618838	8722	CTSF	11	66085295	1.71E-01	9353	7.37E-01	Down	0.34	12294	0.77	0.01
rs2844704	10211	FLOT1	6	30813992	1.71E-01	9354	1.12E-01	Down	1.59	7170	0.77	0.10
rs2351390	57336	ZNF287	17	16419896	1.71E-01	9355	6.02E-01	Up	0.52	11443	0.77	0.02
rs11169067	23416	KCNH3	12	48227767	1.71E-01	9356	6.24E-01	Down	0.49	11609	0.77	0.02
rs890836	22838	RNF44	5	175897089	1.71E-01	9357	4.00E-06	Up	4.61	1783	0.77	0.54
rs11828884	91057	CCDC34	11	27319306	1.71E-01	9358	2.80E-04	Up	3.63	2755	0.77	0.36
rs2516393	534	ATP6V1G2	6	31614723	1.71E-01	9359	2.39E-01	Down	1.18	8653	0.77	0.06
rs2516393	4795	NFKBIL1	6	31614723	1.71E-01	9360	7.40E-01	Up	0.33	12319	0.77	0.01
rs5935623	25975	EGFL6		13358934	1.71E-01	9361	9.42E-02	Down	1.67	6901	0.77	0.10
rs1080750	1271	CNTFR	9	34571525	1.71E-01	9362	1.75E-01	Down	1.36	7965	0.77	0.08
rs1877780	160728	SLC5A8	12	100120265	1.71E-01	9363	5.23E-01	Up	0.64	10878	0.77	0.03
rs3785181	750	C16orf3	16	88632834	1.72E-01	9364	3.54E-01	Up	0.93	9630	0.77	0.05
rs7949651	56980	PRDM10	11	129337480	1.72E-01	9365	9.01E-02	Up	1.69	6819	0.77	0.10
rs10739078	2315	MLANA	9	5865294	1.72E-01	9366	3.24E-03	Up	2.94	3822	0.77	0.25
rs7303895	79657	RPAP3	12	46363340	1.72E-01	9367	1.41E-01	Up	1.47	7585	0.77	0.09
rs4722966	55033	FKBP14	7	29833844	1.72E-01	9368	1.05E-01	Up	1.62	7083	0.77	0.10
rs11706857	131870	NUDT16	3	132590986	1.72E-01	9369	5.55E-03	Down	2.77	4139	0.76	0.23
rs8073784	6355	CCL8	17	29652449	1.72E-01	9370	9.84E-01	Up	0.02	13801	0.76	0.00
rs1800359	9605	C16orf7	16	88332762	1.72E-01	9371	2.62E-03	Down	3.01	3714	0.76	0.26
rs4729686	64792	RABL5	7	100553985	1.72E-01	9372	2.90E-02	Up	2.18	5426	0.76	0.15
rs11716884	2850	GPR27	3	71892087	1.72E-01	9373	1.86E-01	Up	1.32	8101	0.76	0.07
rs3766509	51205	ACP6	1	144355091	1.72E-01	9374	9.65E-02	Up	1.66	6934	0.76	0.10
rs10886296	143384	C10orf46	10	120512222	1.72E-01	9375	2.64E-01	Up	1.12	8905	0.76	0.06
rs17342414	64860	ARMCX5		101643878	1.72E-01	9376	3.92E-01	Up	0.86	9930	0.76	0.04
rs2892059	219402	MTIF3	13	26919642	1.72E-01	9377	6.71E-01	Up	0.43	11894	0.76	0.02
rs1938725	10249	GLYAT	11	58262269	1.72E-01	9378	3.26E-01	Down	0.98	9414	0.76	0.05
rs5968480	79983	POF1B		84431661	1.72E-01	9379	2.21E-02	Down	2.29	5162	0.76	0.17
rs17063744	7424	VEGFC	4	178074556	1.72E-01	9380	1.43E-04	Down	3.80	2537	0.76	0.38
rs3176835	924	CD7	17	77862035	1.72E-01	9381	3.88E-01	Up	0.86	9901	0.76	0.04
rs10488675	3046	HBE1	11	5254606	1.72E-01	9382	4.20E-01	Up	0.81	10123	0.76	0.04
rs12505475	2110	ETFDH	4	159992849	1.72E-01	9383	2.23E-01	Down	1.22	8488	0.76	0.07
rs12505475	5481	PPID	4	159992849	1.72E-01	9384	7.05E-01	Up	0.38	12100	0.76	0.02
rs9526070	253512	SLC25A30	13	44887456	1.73E-01	9385	4.87E-01	Down	0.69	10630	0.76	0.03
rs6774354	84315	MON1A	3	49936784	1.73E-01	9386	7.23E-01	Down	0.35	12222	0.76	0.01
rs2504786	8547	FCN3	1	27404320	1.73E-01	9387	2.90E-01	Down	1.06	9113	0.76	0.05
rs2504786	2827	GPR3	1	27404320	1.73E-01	9388	9.16E-01	Up	0.11	13352	0.76	0.00
rs222378	8776	MTMR1		149531683	1.73E-01	9389	4.22E-01	Down	0.80	10132	0.76	0.04
rs7981756	10804	GJB6	13	19688742	1.73E-01	9390	5.66E-01	Down	0.57	11210	0.76	0.02
rs8137951	49	ACR	22	49455808	1.73E-01	9391	9.69E-01	Down	0.04	13695	0.76	0.00

rs880182	56751	BARHL1	9	132492187	1.73E-01	9392	1.34E-01	Down	1.50	7487	0.76	0.09
rs880182	64794	DDX31	9	132492187	1.73E-01	9393	2.47E-01	Down	1.16	8733	0.76	0.06
rs482670	5900	RALGDS	9	133036912	1.73E-01	9394	2.31E-01	Up	1.20	8579	0.76	0.06
rs463426	23119	HIC2	22	20133739	1.73E-01	9395	3.66E-04	Up	3.56	2851	0.76	0.34
rs2450246	891	CCNB1	5	68486504	1.73E-01	9396	2.09E-08	Up	5.61	1126	0.76	0.77
rs11204764	56882	CDC42SE1	1	147829469	1.73E-01	9397	1.00E-09	Up	6.10	896	0.76	0.90
rs11204764	54964	C1orf56	1	147829469	1.73E-01	9398	2.57E-06	Up	4.70	1708	0.76	0.56
rs11204764	149428	BNIPL	1	147829469	1.73E-01	9399	4.34E-04	Down	3.52	2905	0.76	0.34
rs11204764	58497	PRUNE	1	147829469	1.73E-01	9400	6.44E-02	Up	1.85	6331	0.76	0.12
rs11204764	10962	MLLT11	1	147829469	1.73E-01	9401	7.71E-01	Down	0.29	12504	0.76	0.01
rs11672955	83475	DOHH	19	3462709	1.73E-01	9402	3.56E-01	Up	0.92	9643	0.76	0.04
rs8178990	6572	SLC18A3	10	50500177	1.73E-01	9403	4.35E-01	Up	0.78	10229	0.76	0.04
rs1561606	55137	FIGN	2	164282968	1.73E-01	9404	8.63E-02	Up	1.72	6742	0.76	0.11
rs6673408	5788	PTPRC	1	195340962	1.73E-01	9405	3.58E-02	Up	2.10	5640	0.76	0.14
rs11165947	676	BRDT	1	92184744	1.73E-01	9406	1.43E-02	Up	2.45	4776	0.76	0.18
rs2277289	916	CD3E	11	117687870	1.74E-01	9407	1.00E-01	Down	1.64	7000	0.76	0.10
rs477895	56834	GPR137	11	63805488	1.74E-01	9408	8.52E-02	Up	1.72	6724	0.76	0.11
rs2990087	79937	CNTNAP3	9	39174065	1.74E-01	9409	2.91E-01	Up	1.06	9119	0.76	0.05
rs2353097	10472	ZNF238	1	240554740	1.74E-01	9410	9.14E-01	Up	0.11	13338	0.76	0.00
rs1886333	3354	HTR1E	6	87721328	1.74E-01	9411	2.29E-01	Down	1.20	8556	0.76	0.06
rs7357341	1129	CHRM2	7	136148708	1.74E-01	9412	8.86E-03	Down	2.62	4425	0.76	0.21
rs2228001	79188	TMEM43	3	14162450	1.74E-01	9413	4.74E-04	Down	3.50	2944	0.76	0.33
rs636987	9825	SPATA2	20	47957695	1.74E-01	9414	8.40E-02	Up	1.73	6700	0.76	0.11
rs7918867	59338	PLEKHA1	10	124159656	1.74E-01	9415	4.86E-10	Down	6.22	854	0.76	0.93
rs11778246	3574	IL7	8	79868027	1.74E-01	9416	7.83E-01	Up	0.28	12565	0.76	0.01
rs4760176	91419	XRCC6BP1	12	56645814	1.74E-01	9417	6.54E-01	Up	0.45	11791	0.76	0.02
rs4898502	5630	PRPH	12	47968243	1.74E-01	9418	1.65E-01	Down	1.39	7865	0.76	0.08
rs17280682	27288	RBMXL2	11	7048145	1.74E-01	9419	7.68E-01	Up	0.29	12477	0.76	0.01
rs9375689	5454	POU3F2	6	99377254	1.74E-01	9420	2.04E-02	Up	2.32	5091	0.76	0.17
rs432448	164237	WFDC13	20	43765712	1.74E-01	9421	1.49E-01	Down	1.44	7669	0.76	0.08
rs432448	280664	WFDC10B	20	43765712	1.74E-01	9422	1.70E-01	Up	1.37	7922	0.76	0.08
rs6918631	64288	ZNF323	6	28420435	1.75E-01	9423	2.49E-01	Up	1.15	8755	0.76	0.06
rs7973350	246213	SLC17A8	12	99265634	1.75E-01	9424	1.22E-01	Down	1.55	7336	0.76	0.09
rs7540	8192	CLPP	19	6325890	1.75E-01	9425	1.53E-03	Up	3.17	3390	0.76	0.28
rs7075080	3257	HPS1	10	100202364	1.75E-01	9426	1.07E-06	Down	4.88	1585	0.76	0.60
rs7909151	1646	AKR1C2	10	5016096	1.75E-01	9427	3.90E-01	Down	0.86	9914	0.76	0.04
rs8100241	29086	C19orf62	19	17253894	1.75E-01	9428	4.56E-01	Up	0.75	10387	0.76	0.03
rs8100241	83878	USHBP1	19	17253894	1.75E-01	9429	8.70E-01	Up	0.16	13064	0.76	0.01
rs12422111	7465	WEE1	11	9570023	1.75E-01	9430	1.27E-01	Up	1.53	7401	0.76	0.09
rs481168	7753	ZNF202	11	123099450	1.75E-01	9431	2.76E-01	Up	1.09	9002	0.76	0.06
rs9415064	54788	DNAJB12	10	73759370	1.75E-01	9432	8.98E-01	Down	0.13	13230	0.76	0.00
rs6049002	6666	SOX12	20	244172	1.75E-01	9433	1.57E-04	Up	3.78	2561	0.76	0.38
rs1955611	10243	GPHN	14	66539340	1.75E-01	9434	3.74E-07	Up	5.08	1446	0.76	0.64
rs10123856	84720	PIGO	9	35104614	1.75E-01	9435	4.50E-03	Up	2.84	3995	0.76	0.23
rs10123856	80256	KIAA1539	9	35104614	1.75E-01	9436	9.19E-01	Up	0.10	13368	0.76	0.00
rs2361797	79168	LILRA6	19	59445355	1.75E-01	9437	2.44E-01	Down	1.16	8705	0.76	0.06
rs2361797	10990	LILRB5	19	59445355	1.75E-01	9438	6.96E-01	Up	0.39	12043	0.76	0.02
rs2361797	11025	LILRB3	19	59445355	1.75E-01	9439	9.19E-01	Down	0.10	13370	0.76	0.00
rs1330320	3448	IFNA14	9	21245150	1.75E-01	9440	1.58E-01	Up	1.41	7792	0.76	0.08
rs7552312	10103	TSPAN1	1	46369240	1.75E-01	9441	1.40E-15	Up	7.99	361	0.76	1.49
rs7552312	55624	POMGNT1	1	46369240	1.75E-01	9442	9.08E-01	Up	0.12	13292	0.76	0.00
rs10755694	51513	ETV7	6	36432203	1.75E-01	9443	2.20E-02	Down	2.29	5159	0.76	0.17
rs4908830	9563	H6PD	1	9232137	1.75E-01	9444	9.58E-06	Down	4.43	1940	0.76	0.50
rs12089373	84320	ACBD6	1	177186964	1.75E-01	9445	6.15E-03	Up	2.74	4181	0.76	0.22
rs10861159	4801	NFYB	12	103008785	1.75E-01	9446	5.78E-04	Down	3.44	3012	0.76	0.32
rs2043805	133022	TRAM1L1	4	118372416	1.75E-01	9447	3.70E-04	Up	3.56	2855	0.76	0.34
rs861020	148304	C1orf74	1	206365506	1.75E-01	9448	3.72E-01	Up	0.89	9785	0.76	0.04
rs861020	3664	IRF6	1	206365506	1.75E-01	9449	9.98E-01	Down	0.00	13898	0.76	0.00
rs17580223	51691	LSM8	7	117429235	1.75E-01	9450	3.13E-01	Down	1.01	9298	0.76	0.05
rs11766656	51119	SBDS	7	65916348	1.75E-01	9451	8.34E-03	Down	2.64	4369	0.76	0.21
rs11766656	55253	TYW1	7	65916348	1.75E-01	9452	4.58E-01	Down	0.74	10409	0.76	0.03
rs11763147	154807	VKORC1L1	7	64770971	1.75E-01	9453	2.43E-05	Up	4.22	2123	0.76	0.46
rs2294011	54742	LY6K	8	143781973	1.75E-01	9454	4.34E-02	Up	2.02	5837	0.76	0.14
rs7962629	716	C1S	12	7037031	1.75E-01	9455	6.75E-05	Down	3.99	2354	0.76	0.42
rs2288542	2063	NR2F6	19	17198223	1.76E-01	9456	6.89E-02	Up	1.82	6407	0.76	0.12
rs2866908	27123	DKK2	4	108263112	1.76E-01	9457	7.54E-02	Up	1.78	6533	0.76	0.11
rs1873389	152065	C3orf22	3	127772060	1.76E-01	9458	4.99E-01	Down	0.68	10704	0.76	0.03
rs3213627	50834	TAS2R1	5	9694628	1.76E-01	9459	4.74E-01	Down	0.72	10531	0.76	0.03
rs1868827	114804	RNF157	17	71686612	1.76E-01	9460	1.59E-06	Up	4.80	1640	0.76	0.58
rs11654159	10966	RAB40B	17	78246238	1.76E-01	9461	1.13E-01	Up	1.58	7193	0.75	0.09
rs2232965	25804	LSM4	19	18287950	1.76E-01	9462	4.03E-01	Up	0.84	10005	0.75	0.04
rs13138020	5458	POU4F2	4	147903076	1.76E-01	9463	5.22E-01	Up	0.64	10870	0.75	0.03
rs851023	1432	MAPK14	6	36114198	1.76E-01	9464	1.07E-09	Down	6.10	898	0.75	0.90
rs9855669	79663	HSPBAP1	3	123993453	1.76E-01	9465	4.00E-01	Up	0.84	9982	0.75	0.04
rs9618419	8214	DGCR6	22	17281959	1.76E-01	9466	3.57E-02	Up	2.10	5636	0.75	0.14
rs9618419	5625	PRODH	22	17281959	1.76E-01	9467	3.62E-02	Down	2.09	5655	0.75	0.14
rs2576	89792	GAL3ST3	11	65565043	1.76E-01	9468	9.58E-01	Down	0.05	13605	0.75	0.00
rs2288645	9311	ACCN3	7	150185394	1.76E-01	9469	2.76E-03	Up	2.99	3744	0.75	0.26
rs2288645	1020	CDK5	7	150185394	1.76E-01	9470	9.69E-02	Up	1.66	6941	0.75	0.10
rs2288645	11194	ABCB8	7	150185394	1.76E-01	9471	1.07E-01	Up	1.61	7105	0.75	0.10
rs2288645	6522	SLC4A2	7	150185394	1.76E-01	9472	3.99E-01	Up	0.84	9978	0.75	0.04

rs10489202	25874	BRP44	1	164634737	1.76E-01	9473	5.62E-16	Up	8.09	352	0.75	1.53
rs1023435	29944	PNMA3		151878032	1.76E-01	9474	2.68E-01	Up	1.11	8933	0.75	0.06
rs3181187	839	CASP6	4	110980514	1.76E-01	9475	1.62E-04	Up	3.77	2570	0.75	0.38
rs3782353	7480	WNT10B	12	47645147	1.76E-01	9476	3.31E-02	Up	2.13	5564	0.75	0.15
rs3782353	377	ARF3	12	47645147	1.76E-01	9477	3.15E-01	Up	1.00	9327	0.75	0.05
rs3782353	7471	WNT1	12	47645147	1.76E-01	9478	7.62E-01	Up	0.30	12452	0.75	0.01
rs7902637	9806	SPOCK2	10	73495958	1.76E-01	9479	2.36E-01	Down	1.18	8623	0.75	0.06
rs3923716	8408	ULK1	12	131078490	1.76E-01	9480	4.56E-01	Up	0.75	10386	0.75	0.03
rs876479	6330	SCN4B	11	117533304	1.76E-01	9481	5.19E-01	Down	0.64	10853	0.75	0.03
rs7113199	27087	B3GAT1	11	133752397	1.76E-01	9482	5.27E-11	Up	6.57	715	0.75	1.03
rs3788409	7494	XBP1	22	27522705	1.77E-01	9483	4.64E-02	Up	1.99	5906	0.75	0.13
rs12181780	8763	CD164	6	109816229	1.77E-01	9484	1.52E-04	Up	3.79	2555	0.75	0.38
rs10919242	92342	C1orf156	1	166487646	1.77E-01	9485	3.13E-04	Up	3.60	2792	0.75	0.35
rs2303112	2208	FCER2	19	7672866	1.77E-01	9486	2.69E-02	Down	2.21	5357	0.75	0.16
rs7009648	55872	PBK	8	27732734	1.77E-01	9487	9.25E-02	Up	1.68	6873	0.75	0.10
rs2302319	1145	CHRNE	17	4738081	1.77E-01	9488	7.13E-01	Up	0.37	12151	0.75	0.01
rs1533423	4534	MTM1		149463033	1.77E-01	9489	9.59E-01	Up	0.05	13616	0.75	0.00
rs4393070	29934	SNX12		70082132	1.77E-01	9490	1.26E-05	Down	4.37	2004	0.75	0.49
rs10416371	79090	TRAPPC6A	19	50351976	1.77E-01	9491	5.93E-14	Up	7.51	455	0.75	1.32
rs2053984	6235	RPS29	14	49129772	1.77E-01	9492	5.49E-01	Up	0.60	11070	0.75	0.03
rs1469087	284312	ZSCAN1	19	63264791	1.77E-01	9493	4.65E-02	Down	1.99	5909	0.75	0.13
rs11823140	9610	RIN1	11	65854898	1.77E-01	9494	5.80E-02	Down	1.90	6189	0.75	0.12
rs11823140	25855	BRMS1	11	65854898	1.77E-01	9495	9.72E-01	Down	0.03	13712	0.75	0.00
rs710887	4967	OGDH	7	44482612	1.77E-01	9496	4.54E-01	Up	0.75	10372	0.75	0.03
rs7259253	55621	TRMT1	19	13098502	1.77E-01	9497	7.05E-03	Up	2.69	4265	0.75	0.22
rs7029757	27348	TOR1B	9	129646220	1.77E-01	9498	1.86E-12	Up	7.04	580	0.75	1.17
rs156425	10457	GPNMB	7	23082359	1.77E-01	9499	9.40E-11	Up	6.45	760	0.75	1.00
rs8141815	5594	MAPK1	22	20516139	1.77E-01	9500	1.26E-01	Down	1.53	7386	0.75	0.09
rs10282700	55915	LANCL2	7	55217012	1.77E-01	9501	3.55E-01	Up	0.93	9636	0.75	0.04
rs3816873	93587	RG9MTD2	4	100861842	1.77E-01	9502	4.47E-02	Up	2.01	5868	0.75	0.13
rs2992644	2271	FH	1	238004709	1.77E-01	9503	4.98E-11	Up	6.64	692	0.75	1.03
rs2862745	54464	XRN1	3	143554630	1.77E-01	9504	8.20E-02	Up	1.74	6667	0.75	0.11
rs12819494	8302	KLRC4	12	10442808	1.77E-01	9505	4.39E-01	Up	0.77	10260	0.75	0.04
rs4685611	51185	CRBN	3	3183731	1.77E-01	9506	4.53E-02	Down	2.00	5879	0.75	0.13
rs4546744	55684	C9orf86	9	136970358	1.77E-01	9507	5.94E-01	Down	0.53	11397	0.75	0.02
rs4546744	85014	TMEM141	9	136970358	1.77E-01	9508	8.82E-01	Down	0.15	13140	0.75	0.01
rs5921669	1478	CSTF2		99911563	1.77E-01	9509	6.85E-05	Up	3.98	2358	0.75	0.42
rs5921669	27035	NOX1		99911563	1.77E-01	9510	1.80E-01	Down	1.34	8018	0.75	0.07
rs2758331	6648	SOD2	6	160075481	1.77E-01	9511	3.54E-05	Down	4.14	2206	0.75	0.45
rs1061474	10181	RBM5	3	50119955	1.78E-01	9512	9.65E-01	Down	0.04	13654	0.75	0.00
rs6890223	9366	RAB9P1	5	104464778	1.78E-01	9513	9.35E-01	Up	0.08	13463	0.75	0.00
rs6690864	84838	ZNF496	1	243816748	1.78E-01	9514	6.08E-01	Up	0.51	11487	0.75	0.02
rs6068824	1591	CYP24A1	20	52243550	1.78E-01	9515	5.84E-02	Down	1.89	6202	0.75	0.12
rs3815501	9689	BZW1	2	201511628	1.78E-01	9516	1.16E-02	Up	2.52	4616	0.75	0.19
rs17097403	85016	C11orf70	11	101440077	1.78E-01	9517	4.89E-02	Down	1.97	5967	0.75	0.13
rs11921535	11235	PDCD10	3	168943085	1.78E-01	9518	5.74E-01	Up	0.56	11267	0.75	0.02
rs17253702	3958	LGALS3	14	54653636	1.78E-01	9519	8.29E-13	Down	7.16	551	0.75	1.21
rs10496561	5899	RALB	2	120768554	1.78E-01	9520	7.52E-08	Down	5.38	1266	0.75	0.71
rs1506869	2796	GNRH1	8	25325019	1.78E-01	9521	3.93E-01	Down	0.85	9933	0.75	0.04
rs10421422	55723	ASF1B	19	14107429	1.78E-01	9522	4.62E-02	Up	1.99	5900	0.75	0.13
rs10421422	5566	PRKACA	19	14107429	1.78E-01	9523	3.49E-01	Down	0.94	9581	0.75	0.05
rs12048236	54627	KIAA1383	1	229248986	1.78E-01	9524	8.27E-01	Up	0.22	12834	0.75	0.01
rs10868072	55582	KIF27	9	83764034	1.78E-01	9525	9.96E-03	Up	2.58	4508	0.75	0.20
rs630014	28	ABO	9	133179276	1.78E-01	9526	4.59E-01	Down	0.74	10412	0.75	0.03
rs2140287	92292	GLYATL1	3	186699891	1.78E-01	9527	9.72E-32	Up	11.68	55	0.75	3.10
rs2140287	200879	LIPH	3	186699891	1.78E-01	9528	1.04E-01	Up	1.63	7053	0.75	0.10
rs2140287	90407	TMEM41A	3	186699891	1.78E-01	9529	1.90E-01	Up	1.31	8153	0.75	0.07
rs8053839	83752	LONP2	16	46948013	1.78E-01	9530	7.94E-01	Up	0.26	12636	0.75	0.01
rs8053839	6477	SIAH1	16	46948013	1.78E-01	9531	8.50E-01	Up	0.19	12954	0.75	0.01
rs2145222	60493	FASTKD5	20	3078175	1.78E-01	9532	6.93E-01	Up	0.39	12024	0.75	0.02
rs4793171	79877	DCAKD	17	40487816	1.79E-01	9533	4.76E-01	Down	0.71	10546	0.75	0.03
rs3751289	5583	PRKCH	14	61053696	1.79E-01	9534	8.75E-02	Up	1.71	6764	0.75	0.11
rs6849345	2891	GRIA2	4	158497734	1.79E-01	9535	3.39E-01	Down	0.96	9514	0.75	0.05
rs3130267	1616	DAXX	6	33414772	1.79E-01	9536	1.36E-01	Up	1.49	7522	0.75	0.09
rs3784381	5479	PPIB	15	62236892	1.79E-01	9537	5.81E-22	Up	9.62	161	0.75	2.12
rs3784381	79856	SNX22	15	62236892	1.79E-01	9538	1.63E-06	Up	4.79	1641	0.75	0.58
rs12741305	8672	EIF4G3	1	21065493	1.79E-01	9539	1.40E-04	Up	3.81	2528	0.75	0.39
rs9357371	705	BYSL	6	41993297	1.79E-01	9540	5.52E-07	Up	5.01	1496	0.75	0.63
rs10889687	26135	SERBP1	1	67621803	1.79E-01	9541	1.12E-07	Up	5.31	1307	0.75	0.70
rs16953566	55055	ZWILCH	15	64637258	1.79E-01	9542	9.32E-06	Up	4.43	1931	0.75	0.50
rs8096764	8731	RNMT	18	13722454	1.79E-01	9543	1.81E-04	Down	3.74	2593	0.75	0.37
rs7030941	26130	GAPVD1	9	125214136	1.79E-01	9544	5.38E-01	Down	0.62	10997	0.75	0.03
rs10100154	225689	MAPK15	8	144863114	1.79E-01	9545	6.74E-02	Down	1.83	6387	0.75	0.12
rs9749567	81794	ADAMTS10	19	8563166	1.79E-01	9546	9.87E-01	Up	0.02	13823	0.75	0.00
rs2573182	4291	MLF1	3	159811528	1.79E-01	9547	1.18E-06	Up	4.86	1597	0.75	0.59
rs673495	10657	KHDRBS1	1	32145443	1.80E-01	9548	3.50E-01	Down	0.93	9589	0.75	0.05
rs495124	27102	EIF2AK1	7	5875275	1.80E-01	9549	2.52E-08	Up	5.57	1148	0.75	0.76
rs4762495	7112	TMPO	12	97436573	1.80E-01	9550	8.02E-01	Up	0.25	12688	0.75	0.01
rs971214	9892	SNAP91	6	84333660	1.80E-01	9551	1.96E-02	Up	2.33	5054	0.75	0.17
rs931127	6494	SIPA1	11	65161876	1.80E-01	9552	5.85E-01	Down	0.55	11343	0.75	0.02
rs931127	399909	PCNXL3	11	65161876	1.80E-01	9553	6.62E-01	Down	0.44	11839	0.75	0.02

rs697222	4640	MYO1A	12	55721389	1.80E-01	9554	9.83E-01	Up	0.02	13800	0.75	0.00
rs507757	1068	CETN1	18	572838	1.80E-01	9555	8.95E-01	Up	0.13	13214	0.75	0.00
rs1867312	5341	PLEK	2	68531632	1.80E-01	9556	4.42E-03	Up	2.85	3985	0.75	0.24
rs13398187	253635	CCDC75	2	37216855	1.80E-01	9557	3.45E-02	Down	2.11	5608	0.75	0.15
rs8113868	6590	SLPI	20	43331004	1.80E-01	9558	7.87E-06	Down	4.47	1899	0.74	0.51
rs3867146	80212	CCDC92	12	122962885	1.80E-01	9559	5.31E-01	Up	0.63	10947	0.74	0.03
rs4667257	1281	COL3A1	2	189706316	1.80E-01	9560	2.06E-02	Up	2.32	5097	0.74	0.17
rs875213	79922	MRM1	17	32029147	1.80E-01	9561	1.76E-01	Down	1.35	7970	0.74	0.08
rs13417920	55679	LIMS2	2	128104167	1.80E-01	9562	1.74E-04	Down	3.75	2580	0.74	0.38
rs13417920	2840	GPR17	2	128104167	1.80E-01	9563	6.03E-01	Up	0.52	11450	0.74	0.02
rs13417920	4648	MYO7B	2	128104167	1.80E-01	9564	6.80E-01	Down	0.41	11945	0.74	0.02
rs3816021	51247	PAIP2	5	138685843	1.80E-01	9565	5.25E-02	Down	1.94	6048	0.74	0.13
rs3816021	9782	MATR3	5	138685843	1.80E-01	9566	7.14E-01	Up	0.37	12156	0.74	0.01
rs2460701	221016	CCDC7	10	32792191	1.80E-01	9567	7.21E-01	Up	0.36	12206	0.74	0.01
rs4536493	64755	C16orf58	16	31411252	1.80E-01	9568	1.97E-03	Up	3.09	3526	0.74	0.27
rs1076362	90273	CEACAM21	19	46783003	1.80E-01	9569	1.84E-01	Down	1.33	8073	0.74	0.07
rs7904252	403	ARL3	10	104446715	1.81E-01	9570	5.55E-02	Up	1.92	6127	0.74	0.13
rs6960062	23353	UNC84A	7	657906	1.81E-01	9571	1.08E-01	Up	1.61	7118	0.74	0.10
rs11651881	6348	CCL3	17	31431019	1.81E-01	9572	1.53E-03	Up	3.17	3391	0.74	0.28
rs8047814	1504	CTRB1	16	73793655	1.81E-01	9573	2.60E-01	Down	1.13	8859	0.74	0.06
rs200480	8363	HIST1H4J	6	27881643	1.81E-01	9574	1.90E-05	Up	4.28	2082	0.74	0.47
rs200480	8331	HIST1H2AJ	6	27881643	1.81E-01	9575	3.55E-01	Up	0.93	9635	0.74	0.05
rs6804135	165918	RNF168	3	197679744	1.81E-01	9576	3.76E-01	Down	0.88	9812	0.74	0.04
rs9532824	28984	C13orf15	13	40935920	1.81E-01	9577	2.01E-02	Down	2.32	5078	0.74	0.17
rs131190	10634	GAS2L1	22	28017051	1.81E-01	9578	3.51E-01	Down	0.93	9592	0.74	0.05
rs10935023	84129	ACAD11	3	133833160	1.81E-01	9579	5.14E-02	Up	1.95	6020	0.74	0.13
rs2070720	974	CD79B	17	59372505	1.81E-01	9580	3.17E-01	Up	1.00	9338	0.74	0.05
rs7745815	64208	POPDC3	6	105742097	1.81E-01	9581	3.61E-06	Up	4.63	1769	0.74	0.54
rs4910812	143630	UBQLNL	11	5491583	1.81E-01	9582	8.61E-02	Down	1.72	6740	0.74	0.11
rs4910812	50613	UBQLN3	11	5491583	1.81E-01	9583	6.94E-01	Down	0.39	12032	0.74	0.02
rs3213465	10838	ZNF275		152095237	1.81E-01	9584	7.66E-02	Up	1.77	6566	0.74	0.11
rs932652	3026	HABP2	10	115334779	1.81E-01	9585	1.78E-02	Down	2.37	4975	0.74	0.17
rs17127091	3716	JAK1	1	65029710	1.81E-01	9586	2.88E-16	Down	8.18	338	0.74	1.55
rs749670	9726	ZNF646	16	30996126	1.81E-01	9587	8.46E-02	Down	1.72	6712	0.74	0.11
rs749670	79759	ZNF668	16	30996126	1.81E-01	9588	7.91E-01	Down	0.27	12612	0.74	0.01
rs2273454	55178	RNMTL1	17	632390	1.81E-01	9589	2.31E-01	Up	1.20	8569	0.74	0.06
rs2236074	6118	RPA2	1	27895526	1.81E-01	9590	1.91E-03	Down	3.10	3511	0.74	0.27
rs2236074	9473	C1orf38	1	27895526	1.81E-01	9591	5.62E-01	Up	0.58	11178	0.74	0.03
rs2089886	51433	ANAPC5	12	120224496	1.82E-01	9592	1.06E-19	Up	9.10	207	0.74	1.90
rs10791889	79703	C11orf80	11	66250401	1.82E-01	9593	2.56E-01	Up	1.14	8822	0.74	0.06
rs7380834	3593	IL12B	5	158668511	1.82E-01	9594	5.24E-01	Down	0.64	10890	0.74	0.03
rs7812287	6718	AKR1D1	7	137210930	1.82E-01	9595	1.93E-02	Up	2.34	5039	0.74	0.17
rs6914547	6862	T	6	166537756	1.82E-01	9596	7.52E-01	Down	0.32	12392	0.74	0.01
rs7967152	7421	VDR	12	46530451	1.82E-01	9597	9.36E-01	Down	0.08	13467	0.74	0.00
rs7638388	4134	MAP4	3	47889978	1.82E-01	9598	7.84E-07	Down	4.94	1539	0.74	0.61
rs10927875	7709	ZBTB17	1	16044618	1.82E-01	9599	2.00E-03	Up	3.09	3530	0.74	0.27
rs550	79591	C10orf76	10	103595408	1.82E-01	9600	3.34E-03	Down	2.93	3831	0.74	0.25
rs550	30819	KCNIP2	10	103595408	1.82E-01	9601	3.93E-01	Down	0.85	9934	0.74	0.04
rs1539872	4087	SMAD2	18	43679476	1.82E-01	9602	8.12E-01	Down	0.24	12737	0.74	0.01
rs7954843	83448	PUS7L	12	42399277	1.82E-01	9603	7.92E-04	Up	3.36	3131	0.74	0.31
rs4889545	51327	ERAF	16	31428777	1.82E-01	9604	9.38E-01	Up	0.08	13482	0.74	0.00
rs3743913	197335	WDR90	16	660987	1.83E-01	9605	2.36E-05	Up	4.23	2118	0.74	0.46
rs2306660	57787	MARK4	19	50494703	1.83E-01	9606	3.20E-01	Down	0.99	9361	0.74	0.05
rs2306660	1158	CKM	19	50494703	1.83E-01	9607	4.22E-01	Up	0.80	10138	0.74	0.04
rs200440	55735	DNAJC11	1	6647548	1.83E-01	9608	3.29E-03	Up	2.94	3827	0.74	0.25
rs1059264	123096	SLC25A29	14	99838916	1.83E-01	9609	2.72E-01	Down	1.10	8973	0.74	0.06
rs10930689	1123	CHN1	2	175560002	1.83E-01	9610	2.49E-04	Down	3.66	2710	0.74	0.36
rs8065479	388403	YPEL2	17	54768829	1.83E-01	9611	2.94E-02	Up	2.18	5442	0.74	0.15
rs2356455	60485	SAV1	14	50162892	1.83E-01	9612	6.74E-01	Down	0.42	11908	0.74	0.02
rs16956149	79875	THSD4	15	69828693	1.83E-01	9613	1.27E-12	Down	7.10	562	0.74	1.19
rs1465245	2769	GNA15	19	3087845	1.83E-01	9614	2.86E-04	Down	3.63	2761	0.74	0.35
rs13154131	23367	LARP1	5	154067247	1.83E-01	9615	5.52E-10	Up	6.24	842	0.74	0.93
rs342056	388	RHOB	2	20574135	1.83E-01	9616	4.26E-03	Down	2.86	3971	0.74	0.24
rs4936414	3587	IL10RA	11	117358823	1.83E-01	9617	2.61E-04	Up	3.65	2729	0.74	0.36
rs1190452	200765	TIGD1	2	233243057	1.83E-01	9618	5.61E-01	Up	0.58	11168	0.74	0.03
rs1028590	81889	FAHD1	16	1798070	1.83E-01	9619	1.10E-01	Up	1.60	7143	0.74	0.10
rs1028590	3483	IGFALS	16	1798070	1.83E-01	9620	3.97E-01	Up	0.85	9967	0.74	0.04
rs1028590	3029	HAGH	16	1798070	1.83E-01	9621	4.17E-01	Up	0.81	10104	0.74	0.04
rs3813331	58527	C6orf115	6	139396851	1.83E-01	9622	2.77E-11	Up	6.66	683	0.74	1.06
rs3110570	124857	WFIKKN2	17	46252077	1.83E-01	9623	4.83E-01	Down	0.70	10598	0.74	0.03
rs3898431	79672	FN3KRP	17	78251628	1.83E-01	9624	3.82E-01	Down	0.87	9857	0.74	0.04
rs6554660	348932	SLC6A18	5	1260527	1.83E-01	9625	5.33E-01	Down	0.62	10962	0.74	0.03
rs2235950	27022	FOXD3	1	63497732	1.83E-01	9626	7.86E-01	Down	0.27	12584	0.74	0.01
rs2301993		B4GALT2	1	44095118	1.83E-01	9627	9.15E-01	Up	0.11	13345	0.74	0.00
rs12878503	2957	GTF2A1	14	80712806	1.84E-01	9628	1.78E-01	Up	1.35	8004	0.74	0.07
rs11667601	10523	CHERP	19	16531660	1.84E-01	9629	5.99E-04	Up	3.43	3026	0.74	0.32
rs11667601	79939	SLC35E1	19	16531660	1.84E-01	9630	1.45E-01	Up	1.46	7630	0.74	0.08
rs9530	2990	GUSB	7	64870044	1.84E-01	9631	3.41E-18	Up	8.70	261	0.74	1.75
rs2071518	6134	RPL10	8	120504993	1.84E-01	9632	2.29E-03	Up	3.05	3626	0.74	0.26
rs2071518	4856	NOV	8	120504993	1.84E-01	9633	1.28E-01	Up	1.52	7415	0.74	0.09
rs416661	23759	PPIL2	22	20354825	1.84E-01	9634	4.53E-03	Up	2.84	3999	0.74	0.23

rs7699456	201798	TIGD4	4	154078297	1.84E-01	9635	6.83E-01	Down	0.41	11963	0.74	0.02
rs10483285	196883	ADCY4	14	23869198	1.84E-01	9636	3.93E-04	Down	3.54	2877	0.73	0.34
rs10483285	27141	CIDEB	14	23869198	1.84E-01	9637	2.55E-01	Down	1.14	8811	0.73	0.06
rs10483285	11035	RIPK3	14	23869198	1.84E-01	9638	2.55E-01	Down	1.14	8815	0.73	0.06
rs10138997	2287	FKBP3	14	44676037	1.84E-01	9639	6.46E-03	Up	2.72	4207	0.73	0.22
rs6026567	2778	GNAS	20	56878310	1.84E-01	9640	2.52E-01	Up	1.15	8783	0.73	0.06
rs254460	81786	TRIM7	5	180555221	1.84E-01	9641	9.03E-01	Up	0.12	13270	0.73	0.00
rs8664	2059	EPS8	12	15664688	1.84E-01	9642	4.50E-06	Down	4.59	1795	0.73	0.53
rs17209837	5244	ABCB4	7	86769473	1.84E-01	9643	5.05E-02	Up	1.96	6008	0.73	0.13
rs3750010	56903	PAPOLB	7	4674432	1.84E-01	9644	3.90E-01	Up	0.86	9913	0.73	0.04
rs873833	23523	CABIN1	22	22752432	1.84E-01	9645	4.05E-01	Up	0.83	10023	0.73	0.04
rs7931291	5682	PSMA1	11	14601002	1.84E-01	9646	4.55E-03	Up	2.84	4004	0.73	0.23
rs1546839	10682	EBP		48126727	1.84E-01	9647	5.50E-01	Down	0.60	11079	0.73	0.03
rs1546839	152503	SH3D19		48126727	1.84E-01	9648	7.83E-01	Down	0.28	12567	0.73	0.01
rs2469862	5636	PRPSAP2	17	18762550	1.84E-01	9649	6.75E-01	Down	0.42	11915	0.73	0.02
rs784568	11016	ATF7	12	52213821	1.85E-01	9650	2.95E-04	Up	3.62	2767	0.73	0.35
rs2249665	6886	TAL1	1	47399104	1.85E-01	9651	1.92E-01	Down	1.30	8174	0.73	0.07
rs16932803	56656	OR2S2	9	35951087	1.85E-01	9652	6.53E-01	Down	0.45	11785	0.73	0.02
rs11230281	245802	MS4A6E	11	59864236	1.85E-01	9653	1.25E-01	Up	1.54	7366	0.73	0.09
rs7350420	10431	TIMM23	10	51264468	1.85E-01	9654	3.20E-02	Up	2.14	5523	0.73	0.15
rs10233736	7791	ZYX	7	142582857	1.85E-01	9655	2.39E-08	Down	5.58	1143	0.73	0.76
rs10233736	9715	FAM131B	7	142582857	1.85E-01	9656	3.30E-01	Up	0.97	9447	0.73	0.05
rs10416742	7705	ZNF146	19	41414672	1.85E-01	9657	1.53E-12	Up	7.07	571	0.73	1.18
rs162096	133396	IL31RA	5	55165185	1.85E-01	9658	6.16E-02	Up	1.87	6269	0.73	0.12
rs7556644	8674	VAMP4	1	168444525	1.85E-01	9659	2.14E-02	Down	2.30	5136	0.73	0.17
rs11681053	84826	SFT2D3	2	128181777	1.85E-01	9660	1.53E-01	Up	1.43	7709	0.73	0.08
rs14792	144110	TMEM86A	11	18681134	1.85E-01	9661	1.91E-04	Up	3.73	2613	0.73	0.37
rs537786	84153	RNASEH2C	11	65251563	1.85E-01	9662	2.13E-02	Down	2.30	5132	0.73	0.17
rs12652973	5066	PAM	5	102334221	1.85E-01	9663	8.54E-05	Down	3.93	2411	0.73	0.41
rs12425042	10993	SDS	12	112288399	1.85E-01	9664	2.18E-06	Up	4.74	1680	0.73	0.57
rs9357194	6954	TCP11	6	35224199	1.85E-01	9665	7.65E-01	Up	0.30	12457	0.73	0.01
rs711746	6559	SLC12A3	16	55504305	1.85E-01	9666	9.68E-01	Up	0.04	13681	0.73	0.00
rs4343924	80328	ULBP2	6	150355300	1.85E-01	9667	5.58E-01	Down	0.59	11145	0.73	0.03
rs2248003	1525	CXADR	21	17817942	1.85E-01	9668	9.21E-11	Up	6.47	754	0.73	1.00
rs1054072	84366	PRAC	17	44154811	1.85E-01	9669	1.95E-06	Up	4.76	1665	0.73	0.57
rs1054072	10481	HOXB13	17	44154811	1.85E-01	9670	6.15E-04	Up	3.43	3038	0.73	0.32
rs2163294	7029	TFDP2	3	143151151	1.85E-01	9671	2.39E-04	Down	3.67	2693	0.73	0.36
rs2040048	1266	CNN3	1	95124435	1.86E-01	9672	3.10E-07	Down	5.12	1420	0.73	0.65
rs1932067	5051	PAFAH2	1	26006223	1.86E-01	9673	5.93E-01	Up	0.53	11391	0.73	0.02
rs1500218	202151	RANBP3L	5	36348269	1.86E-01	9674	6.37E-02	Down	1.85	6317	0.73	0.12
rs2001217	55898	UNC45A	15	89299250	1.86E-01	9675	5.08E-01	Down	0.66	10774	0.73	0.03
rs590105	143570	XRRA1	11	74301659	1.86E-01	9676	6.47E-01	Up	0.46	11756	0.73	0.02
rs10790680	26493	OR8B8	11	123820969	1.86E-01	9677	1.65E-01	Up	1.39	7864	0.73	0.08
rs1970456	167	CRISP1	6	49931773	1.86E-01	9678	7.33E-01	Up	0.34	12270	0.73	0.01
rs316144	22858	ICK	6	52987654	1.86E-01	9679	3.01E-08	Up	5.56	1157	0.73	0.75
rs2854702	9144	SYNGR2	17	73682925	1.86E-01	9680	4.52E-13	Up	7.24	526	0.73	1.23
rs2854702	7083	TK1	17	73682925	1.86E-01	9681	2.47E-01	Up	1.16	8734	0.73	0.06
rs2854702	125061	AFMID	17	73682925	1.86E-01	9682	4.66E-01	Up	0.73	10469	0.73	0.03
rs3773364	7079	TIMP4	3	12164968	1.86E-01	9683	2.35E-03	Down	3.04	3645	0.73	0.26
rs5953066	6853	SYN1		47217803	1.86E-01	9684	9.11E-01	Down	0.11	13317	0.73	0.00
rs917292	7224	TRPC5		110977631	1.86E-01	9685	3.66E-01	Up	0.90	9724	0.73	0.04
rs13197142	10799	RPP40	6	4931249	1.86E-01	9686	2.88E-05	Up	4.18	2161	0.73	0.45
rs2275591	27109	ATP5S	14	49848566	1.87E-01	9687	8.93E-10	Up	6.13	886	0.73	0.90
rs210567	4104	MAGEA5		150948667	1.87E-01	9688	8.14E-02	Up	1.74	6657	0.73	0.11
rs4779046	8120	AP3B2	15	81139827	1.87E-01	9689	1.01E-03	Down	3.29	3224	0.73	0.30
rs7015644	253943	YTHDF3	8	64257961	1.87E-01	9690	3.58E-07	Up	5.09	1438	0.73	0.64
rs2273102	10564	ARFGEF2	20	47022529	1.87E-01	9691	5.19E-01	Down	0.65	10851	0.73	0.03
rs13082666	50512	PODXL2	3	128834190	1.87E-01	9692	1.06E-06	Up	4.88	1580	0.73	0.60
rs6995969	83877	TM2D2	8	38980156	1.87E-01	9693	2.81E-07	Down	5.14	1409	0.73	0.66
rs4822748	1413	CRYBA4	22	25329518	1.87E-01	9694	6.22E-04	Up	3.42	3041	0.73	0.32
rs652673	7481	WNT11	11	75608939	1.87E-01	9695	5.26E-01	Down	0.63	10914	0.73	0.03
rs12934166	4493	MT1E	16	55207975	1.87E-01	9696	1.59E-05	Down	4.32	2048	0.73	0.48
rs1805421	2332	FMR1		146700462	1.87E-01	9697	4.96E-06	Down	4.57	1816	0.73	0.53
rs4808206	83983	TSSK6	19	19506645	1.87E-01	9698	1.96E-01	Up	1.29	8211	0.73	0.07
rs4808206	148113	CILP2	19	19506645	1.87E-01	9699	9.43E-01	Down	0.07	13512	0.73	0.00
rs4808206	51079	NDUFA13	19	19506645	1.87E-01	9700	9.88E-01	Down	0.01	13833	0.73	0.00
rs10414451	10520	ZNF211	19	62823388	1.87E-01	9701	3.01E-01	Down	1.03	9198	0.73	0.05
rs16865226	8540	AGPS	2	178108259	1.87E-01	9702	4.73E-03	Down	2.82	4023	0.73	0.23
rs4801891	2357	FPR1	19	56946163	1.87E-01	9703	1.92E-02	Up	2.34	5031	0.73	0.17
rs3863064	7372	UMPS	3	125916202	1.87E-01	9704	1.00E-07	Up	5.33	1297	0.73	0.70
rs762651	6748	SSR4		152566285	1.88E-01	9705	1.10E-07	Up	5.31	1303	0.73	0.70
rs762651	5365	PLXNB3		152566285	1.88E-01	9706	1.16E-04	Up	3.85	2489	0.73	0.39
rs762651	3421	IDH3G		152566285	1.88E-01	9707	8.59E-03	Up	2.63	4391	0.73	0.21
rs715156	136319	MTPN	7	135070371	1.88E-01	9708	1.84E-02	Down	2.36	5002	0.73	0.17
rs1256854	348110	C15orf38	15	88272921	1.88E-01	9709	7.88E-01	Down	0.27	12596	0.73	0.01
rs310586	136371	ASB10	7	150331277	1.88E-01	9710	9.94E-01	Up	0.01	13872	0.73	0.00
rs6780013	22937	SCAP	3	47427122	1.88E-01	9711	1.82E-02	Up	2.36	4996	0.73	0.17
rs6780013	25930	PTPN23	3	47427122	1.88E-01	9712	6.74E-01	Down	0.42	11914	0.73	0.02
rs9910083	146723	C17orf77	17	70081239	1.88E-01	9713	9.64E-01	Up	0.04	13644	0.73	0.00
rs1800440	1545	CYP1B1	2	38209790	1.88E-01	9714	9.11E-02	Down	1.69	6849	0.73	0.10
rs13170849	84135	UTP15	5	72917884	1.88E-01	9715	3.07E-02	Down	2.16	5489	0.73	0.15

rs7296418	79676	OGFOD2	12	121982499	1.88E-01	9716	3.74E-03	Up	2.90	3891	0.73	0.24
rs7296418	51329	ARL6IP4	12	121982499	1.88E-01	9717	9.66E-03	Down	2.59	4485	0.73	0.20
rs7144433	801	CALM1	14	89919982	1.88E-01	9718	1.61E-44	Down	14.00	9	0.73	4.38
rs838818	54942	C9orf6	9	108772687	1.88E-01	9719	9.12E-05	Up	3.91	2427	0.73	0.40
rs7153523	117153	MIA2	14	38780677	1.88E-01	9720	4.72E-01	Down	0.72	10515	0.73	0.03
rs716817	9601	PDIA4	7	148132587	1.88E-01	9721	6.46E-13	Up	7.19	537	0.73	1.22
rs12950368	10351	ABCA8	17	64472921	1.88E-01	9722	4.82E-03	Down	2.82	4034	0.72	0.23
rs12194183	57120	GOPC	6	117992652	1.88E-01	9723	3.59E-02	Down	2.10	5645	0.72	0.14
rs1861952	146852	ODF4	17	8176029	1.89E-01	9724	9.97E-01	Up	0.00	13893	0.72	0.00
rs1107179	84942	WDR73	15	82999366	1.89E-01	9725	4.70E-05	Up	4.07	2265	0.72	0.43
rs1107179	4828	NMB	15	82999366	1.89E-01	9726	3.04E-01	Down	1.03	9228	0.72	0.05
rs6896	310	ANXA7	10	74805452	1.89E-01	9727	5.58E-05	Down	4.03	2315	0.72	0.43
rs6896	118491	TTC18	10	74805452	1.89E-01	9728	3.10E-02	Up	2.16	5496	0.72	0.15
rs201615	4621	MYH3	17	10501485	1.89E-01	9729	6.55E-02	Down	1.84	6350	0.72	0.12
rs3003600	51117	COQ4	9	128168018	1.89E-01	9730	2.28E-07	Up	5.17	1389	0.72	0.66
rs3003600	26995	TRUB2	9	128168018	1.89E-01	9731	8.65E-03	Up	2.63	4401	0.72	0.21
rs3003600	10999	SLC27A4	9	128168018	1.89E-01	9732	3.58E-02	Up	2.10	5641	0.72	0.14
rs6632488	286464	CXorf59		35883517	1.89E-01	9733	2.44E-01	Up	1.17	8703	0.72	0.06
rs10520994	51663	ZFR	5	32444559	1.89E-01	9734	1.62E-01	Up	1.40	7843	0.72	0.08
rs11590105	8799	PEX11B	1	143022219	1.89E-01	9735	1.18E-01	Down	1.56	7256	0.72	0.09
rs4832311	64795	RMND5A	2	86890553	1.89E-01	9736	2.34E-09	Up	5.98	943	0.72	0.86
rs4844486	3290	HSD11B1	1	206232912	1.89E-01	9737	1.74E-04	Down	3.75	2579	0.72	0.38
rs6565549	284185	C17orf55	17	76882829	1.89E-01	9738	8.02E-01	Up	0.25	12686	0.72	0.01
rs2276470	2354	FOSB	19	50666508	1.89E-01	9739	1.71E-06	Up	4.79	1647	0.72	0.58
rs7521837	10456	HAX1	1	151065933	1.89E-01	9740	1.02E-02	Down	2.57	4522	0.72	0.20
rs7521837	9898	UBAP2L	1	151065933	1.89E-01	9741	2.66E-02	Up	2.22	5343	0.72	0.16
rs12600601	27043	PELP1	17	4512466	1.90E-01	9742	8.77E-01	Up	0.15	13103	0.72	0.01
rs11721430	23324	MAN2B2	4	6728740	1.90E-01	9743	5.12E-01	Down	0.66	10795	0.72	0.03
rs11861084	84501	SPIRE2	16	88403211	1.90E-01	9744	1.73E-01	Down	1.36	7945	0.72	0.08
rs7553840	7780	SLC30A2	1	26064947	1.90E-01	9745	7.05E-02	Down	1.81	6442	0.72	0.12
rs12520035	134510	UBLCP1	5	158637948	1.90E-01	9746	7.69E-01	Down	0.29	12479	0.72	0.01
rs7527472	267002	PGBD2	1	245412318	1.90E-01	9747	4.26E-03	Up	2.86	3972	0.72	0.24
rs3770214	617	BCS1L	2	219334493	1.90E-01	9748	7.55E-06	Up	4.48	1887	0.72	0.51
rs3770214	84812	PLCD4	2	219334493	1.90E-01	9749	7.70E-01	Down	0.29	12497	0.72	0.01
rs16970911	57082	CASC5	15	38704093	1.90E-01	9750	1.54E-02	Up	2.42	4834	0.72	0.18
rs2213430	5880	RAC2	22	35963460	1.90E-01	9751	1.27E-01	Down	1.53	7397	0.72	0.09
rs2860580	3105	HLA-A	6	30014670	1.90E-01	9752	2.92E-02	Down	2.18	5433	0.72	0.15
rs2860580	80868	HCG4P6	6	30014670	1.90E-01	9753	7.03E-02	Up	1.81	6432	0.72	0.12
rs1572263	64783	RBM15	1	110597778	1.90E-01	9754	5.78E-11	Up	6.64	694	0.72	1.02
rs13139935	22824	HSPA4L	4	129082906	1.90E-01	9755	1.74E-11	Down	6.73	666	0.72	1.08
rs12078573	9900	SV2A	1	146716682	1.90E-01	9756	1.12E-01	Down	1.59	7178	0.72	0.09
rs10515513	80762	NDFIP1	5	141514029	1.91E-01	9757	3.42E-05	Down	4.14	2195	0.72	0.45
rs1268524	114836	SLAMF6	1	157283331	1.91E-01	9758	9.37E-02	Up	1.68	6896	0.72	0.10
rs7035313	92400	RBM18	9	122106673	1.91E-01	9759	7.38E-04	Up	3.38	3095	0.72	0.31
rs10759636	9128	PRPF4	9	113098331	1.91E-01	9760	7.11E-02	Up	1.80	6455	0.72	0.11
rs11823366	55343	SLC35C1	11	45784908	1.91E-01	9761	1.53E-01	Down	1.43	7715	0.72	0.08
rs356349	84146	ZNF644	1	91183062	1.91E-01	9762	3.75E-03	Up	2.90	3894	0.72	0.24
rs1135945	54872	PIGG	4	506586	1.91E-01	9763	1.67E-01	Up	1.38	7882	0.72	0.08
rs2970358	3418	IDH2	15	88423508	1.91E-01	9764	3.06E-01	Up	1.02	9235	0.72	0.05
rs3131115	3133	HLA-E	6	30576770	1.91E-01	9765	9.43E-03	Down	2.60	4464	0.72	0.20
rs12153359	25836	NIPBL	5	37065642	1.91E-01	9766	8.17E-01	Down	0.23	12772	0.72	0.01
rs17511835	7629	ZNF76	6	35351648	1.91E-01	9767	3.34E-02	Up	2.13	5574	0.72	0.15
rs7078219	159296	NKX2-3	10	101264355	1.91E-01	9768	3.06E-02	Up	2.16	5485	0.72	0.15
rs4611492	51755	CRKRS	17	34869863	1.91E-01	9769	7.20E-08	Up	5.38	1261	0.72	0.71
rs4965373	6627	SNRPA1	15	99629918	1.91E-01	9770	3.08E-03	Up	2.96	3790	0.72	0.25
rs11768678	22853	LMTK2	7	97391038	1.91E-01	9771	7.74E-01	Down	0.29	12524	0.72	0.01
rs168109	558	AXL	19	46408111	1.92E-01	9772	1.01E-01	Down	1.64	7015	0.72	0.10
rs9841006	55171	TBCCD1	3	187775837	1.92E-01	9773	8.57E-01	Up	0.18	12992	0.72	0.01
rs607877	11201	POLI	18	50068773	1.92E-01	9774	1.15E-01	Up	1.58	7212	0.72	0.09
rs1144943	4193	MDM2	12	67486012	1.92E-01	9775	3.10E-03	Up	2.96	3791	0.72	0.25
rs4294650	64328	XPO4	13	20254540	1.92E-01	9776	8.69E-01	Up	0.16	13058	0.72	0.01
rs12473402	6868	ADAM17	2	9583316	1.92E-01	9777	9.04E-01	Up	0.12	13272	0.72	0.00
rs7789	10008	KCNE3	11	73843968	1.92E-01	9778	4.05E-01	Down	0.83	10017	0.72	0.04
rs11588779	9927	MFN2	1	12017147	1.92E-01	9779	9.75E-09	Down	5.74	1053	0.72	0.80
rs555835	79073	TMEM109	11	60446021	1.92E-01	9780	1.72E-07	Down	5.23	1358	0.72	0.68
rs17266816	54554	WDR5B	3	123611564	1.92E-01	9781	3.73E-01	Up	0.89	9791	0.72	0.04
rs2808022	55699	IARS2	1	216690512	1.92E-01	9782	1.10E-05	Up	4.40	1969	0.72	0.50
rs7095575	810	CALML3	10	5562893	1.92E-01	9783	8.73E-01	Down	0.16	13080	0.72	0.01
rs17330794	55511	SAGE1		134700110	1.92E-01	9784	2.91E-01	Up	1.06	9120	0.72	0.05
rs2789686	311	ANXA11	10	81905116	1.92E-01	9785	4.42E-02	Down	2.01	5857	0.72	0.14
rs3092921	9459	ARHGEF6		135468520	1.92E-01	9786	8.18E-03	Down	2.64	4355	0.72	0.21
rs990432	57393	TMEM27		15417768	1.92E-01	9787	2.17E-01	Up	1.23	8431	0.72	0.07
rs6998793	55290	BRF2	8	37816032	1.92E-01	9788	7.00E-01	Up	0.38	12074	0.72	0.02
rs770221	64863	METTL4	18	2524877	1.92E-01	9789	1.00E-03	Down	3.29	3223	0.72	0.30
rs267286	153733	CCDC112	5	114673406	1.92E-01	9790	1.10E-07	Up	5.31	1302	0.72	0.70
rs987237	7021	TFAP2B	6	50911009	1.92E-01	9791	9.02E-01	Up	0.12	13255	0.72	0.00
rs6011419	10732	TCFL5	20	60948657	1.92E-01	9792	7.65E-01	Up	0.30	12464	0.72	0.01
rs9917993	2483	FRG1	4	191266852	1.92E-01	9793	2.42E-01	Down	1.17	8678	0.72	0.06
rs12190755	24149	ZNF318	6	43430209	1.93E-01	9794	2.03E-01	Down	1.27	8287	0.72	0.07
rs2064317	7287	TULP1	6	35585010	1.93E-01	9795	2.97E-01	Down	1.04	9170	0.72	0.05
rs10489579	81627	C1orf25	1	181875378	1.93E-01	9796	1.47E-01	Down	1.45	7644	0.72	0.08

rs7678298	213	ALB	4	74649463	1.93E-01	9797	8.86E-03	Down	2.62	4422	0.72	0.21
rs6716817	9475	ROCK2	2	11336432	1.93E-01	9798	1.53E-13	Down	7.38	485	0.72	1.28
rs8092980	80323	CCDC68	18	50714939	1.93E-01	9799	1.06E-02	Down	2.55	4554	0.72	0.20
rs12513554	79628	SH3TC2	5	148421887	1.93E-01	9800	7.91E-01	Down	0.26	12621	0.71	0.01
rs7185040	5310	PKD1	16	2085788	1.93E-01	9801	6.64E-02	Down	1.84	6365	0.71	0.12
rs9344525	4907	NT5E	6	86208157	1.93E-01	9802	2.25E-23	Down	9.96	133	0.71	2.26
rs12022453	10136	ELA3A	1	22088604	1.93E-01	9803	7.89E-01	Up	0.27	12605	0.71	0.01
rs4919850	682	BSG	19	507632	1.93E-01	9804	2.50E-03	Up	3.02	3685	0.71	0.26
rs10172421	10152	ABI2	2	204063908	1.93E-01	9805	8.59E-02	Up	1.72	6734	0.71	0.11
rs3008192	83881	MIXL1	1	222711192	1.93E-01	9806	4.77E-01	Down	0.71	10549	0.71	0.03
rs943842	10497	UNC13B	9	35385276	1.93E-01	9807	3.07E-05	Up	4.17	2174	0.71	0.45
rs480092	57176	VARS2	6	31872878	1.93E-01	9808	3.84E-02	Up	2.07	5713	0.71	0.14
rs480092	3303	HSPA1A	6	31872878	1.93E-01	9809	9.58E-02	Up	1.67	6919	0.71	0.10
rs480092	57819	LSM2	6	31872878	1.93E-01	9810	4.54E-01	Up	0.75	10373	0.71	0.03
rs11764572	1365	CLDN3	7	72623086	1.93E-01	9811	1.38E-07	Up	5.27	1335	0.71	0.69
rs2271750	84833	USMG5	10	105164859	1.93E-01	9812	8.82E-02	Up	1.71	6773	0.71	0.11
rs6948804	51631	LUC7L2	7	138487895	1.93E-01	9813	2.75E-08	Up	5.56	1151	0.71	0.76
rs10423215	84671	ZNF347	19	58340885	1.93E-01	9814	3.30E-04	Up	3.59	2810	0.71	0.35
rs855676	168544	ZNF467	7	148909960	1.93E-01	9815	1.84E-01	Down	1.33	8063	0.71	0.07
rs9901120	91369	ANKRD40	17	46151551	1.93E-01	9816	1.45E-01	Down	1.46	7626	0.71	0.08
rs1054132	4835	NQO2	6	2935893	1.93E-01	9817	2.41E-01	Up	1.17	8667	0.71	0.06
rs7778766	136051	ZNF786	7	148244377	1.94E-01	9818	1.58E-02	Up	2.41	4869	0.71	0.18
rs2032809	27113	BBC3	19	52428056	1.94E-01	9819	4.56E-01	Up	0.74	10394	0.71	0.03
rs1882013	9934	P2RY14	3	152449967	1.94E-01	9820	6.64E-01	Up	0.43	11848	0.71	0.02
rs2267134	162	AP1B1	22	28091520	1.94E-01	9821	2.29E-03	Up	3.05	3625	0.71	0.26
rs999466	760	CA2	8	86582896	1.94E-01	9822	1.13E-04	Up	3.86	2485	0.71	0.39
rs3744714	708	C1QBP	17	5294801	1.94E-01	9823	4.16E-04	Up	3.53	2895	0.71	0.34
rs3744714	56919	DHX33	17	5294801	1.94E-01	9824	1.26E-03	Up	3.22	3319	0.71	0.29
rs938213	79413	ZBED2	3	112796544	1.94E-01	9825	5.09E-01	Down	0.66	10776	0.71	0.03
rs6688807	6121	RPE65	1	68633309	1.94E-01	9826	8.50E-02	Up	1.72	6723	0.71	0.11
rs3017036	57534	MIB1	18	17566764	1.94E-01	9827	3.65E-01	Down	0.91	9720	0.71	0.04
rs16956600	7084	TK2	16	65103483	1.94E-01	9828	3.62E-03	Down	2.91	3874	0.71	0.24
rs12165392	10738	RFPL3	22	31089435	1.94E-01	9829	5.20E-01	Down	0.64	10859	0.71	0.03
rs17106714	8816	WDR22	14	68587751	1.94E-01	9830	1.59E-01	Down	1.41	7802	0.71	0.08
rs7948839	78999	LRFN4	11	66390973	1.94E-01	9831	4.38E-02	Down	2.02	5845	0.71	0.14
rs6073993	57468	SLC12A5	20	44095820	1.94E-01	9832	6.06E-01	Up	0.52	11473	0.71	0.02
rs11632888	114791	TUBGCP5	15	20418062	1.94E-01	9833	8.53E-01	Up	0.18	12978	0.71	0.01
rs10415156	53615	MBD3	19	1523369	1.94E-01	9834	6.75E-01	Up	0.42	11920	0.71	0.02
rs7110260	401	PHOX2A	11	71634231	1.94E-01	9835	7.17E-01	Down	0.36	12176	0.71	0.01
rs2535707	23786	BCL2L13	22	16556423	1.94E-01	9836	9.00E-02	Up	1.70	6812	0.71	0.10
rs649392	595	CCND1	11	69173974	1.94E-01	9837	1.13E-01	Down	1.58	7194	0.71	0.09
rs7498385	54988	ACSM5	16	20354340	1.94E-01	9838	3.69E-01	Down	0.90	9753	0.71	0.04
rs3748971	250	ALPP	2	233076188	1.94E-01	9839	3.63E-01	Up	0.91	9703	0.71	0.04
rs2296351	4752	NEK3	13	51607939	1.95E-01	9840	4.64E-09	Up	5.91	985	0.71	0.83
rs3761673	9576	SPAG6	10	22673484	1.95E-01	9841	9.94E-01	Down	0.01	13870	0.71	0.00
rs11119344	80342	TRAF3IP3	1	206331884	1.95E-01	9842	6.07E-01	Up	0.51	11482	0.71	0.02
rs1397527	128869	PIGU	4	144683990	1.95E-01	9843	4.98E-02	Up	1.96	5991	0.71	0.13
rs1397527	2549	GAB1	4	144683990	1.95E-01	9844	6.73E-01	Down	0.42	11904	0.71	0.02
rs2464698	7072	TIA1	2	70394102	1.95E-01	9845	2.97E-10	Up	6.30	810	0.71	0.95
rs2464698	51449	PCYOX1	2	70394102	1.95E-01	9846	2.01E-03	Down	3.09	3533	0.71	0.27
rs10638	55884	WSB2	12	116934480	1.95E-01	9847	3.35E-12	Up	6.98	605	0.71	1.15
rs10638	5985	RFC5	12	116934480	1.95E-01	9848	8.27E-01	Down	0.22	12836	0.71	0.01
rs8081659	2319	FLOT2	17	24238116	1.95E-01	9849	1.19E-05	Down	4.38	1985	0.71	0.49
rs10764372	219681	ARMC3	10	23347052	1.95E-01	9850	5.91E-01	Up	0.54	11377	0.71	0.02
rs8048423	6236	RRAD	16	65504864	1.95E-01	9851	2.37E-03	Down	3.04	3653	0.71	0.26
rs8048423	1014	CDH16	16	65504864	1.95E-01	9852	4.28E-01	Down	0.79	10171	0.71	0.04
rs16981486	53635	PTOV1	19	55048976	1.95E-01	9853	6.20E-07	Up	4.98	1508	0.71	0.62
rs16981486	11284	PNKP	19	55048976	1.95E-01	9854	7.82E-02	Up	1.76	6600	0.71	0.11
rs4930243	9633	MTL5	11	68256450	1.95E-01	9855	5.10E-02	Up	1.95	6014	0.71	0.13
rs1773225	131583	FAM43A	3	195884875	1.95E-01	9856	1.13E-03	Down	3.26	3267	0.71	0.29
rs3815291	130916	MTERFD2	2	241752530	1.95E-01	9857	6.27E-02	Down	1.86	6291	0.71	0.12
rs884427	10971	YWHAQ	2	9734233	1.95E-01	9858	1.62E-07	Up	5.24	1351	0.71	0.68
rs10506328	4778	NFE2	12	52973499	1.96E-01	9859	4.46E-01	Up	0.76	10311	0.71	0.04
rs5936548	158833	DGAT2L3		69247429	1.96E-01	9860	4.24E-01	Down	0.80	10145	0.71	0.04
rs7917657	64429	ZDHHC6	10	114207244	1.96E-01	9861	1.64E-02	Up	2.40	4907	0.71	0.18
rs9494944	64065	PERP	6	138489887	1.96E-01	9862	2.57E-12	Down	7.00	596	0.71	1.16
rs309152	1615	DARS	2	136490984	1.96E-01	9863	9.48E-04	Down	3.31	3195	0.71	0.30
rs6920432	5467	PPARD	6	35406640	1.96E-01	9864	3.58E-01	Up	0.92	9659	0.71	0.04
rs7725428	353189	SLCO4C1	5	101594006	1.96E-01	9865	3.46E-01	Up	0.94	9565	0.71	0.05
rs3812475	55039	TRMT12	8	125532431	1.96E-01	9866	8.71E-01	Down	0.16	13073	0.71	0.01
rs746439	90933	TRIM41	5	180571016	1.96E-01	9867	1.94E-01	Up	1.30	8189	0.71	0.07
rs1239017	305	ANXA2P3	10	66262827	1.96E-01	9868	1.30E-11	Down	6.77	646	0.71	1.09
rs3105807	3001	GZMA	5	54428130	1.96E-01	9869	3.75E-02	Up	2.08	5691	0.71	0.14
rs6897488	65983	GRAMD3	5	125797578	1.96E-01	9870	5.54E-01	Down	0.59	11109	0.71	0.03
rs2675117	142679	DUSP19	2	183762421	1.96E-01	9871	1.19E-01	Down	1.56	7283	0.71	0.09
rs7229278	6139	RPL17	18	45276924	1.96E-01	9872	7.78E-04	Up	3.36	3123	0.71	0.31
rs7229278	9349	RPL23	18	45276924	1.96E-01	9873	2.19E-03	Up	3.06	3595	0.71	0.27
rs2297866	23452	ANGPTL2	9	126933753	1.96E-01	9874	9.49E-13	Down	7.14	555	0.71	1.20
rs1202543	84886	C1orf198	1	227273534	1.96E-01	9875	4.99E-02	Down	1.96	5996	0.71	0.13
rs2240300	22834	ZNF652	17	44732520	1.96E-01	9876	1.64E-08	Up	5.64	1106	0.71	0.78
rs12333	81556	C15orf44	15	63656325	1.96E-01	9877	5.41E-02	Up	1.93	6092	0.71	0.13

rs11703808	23761	PISD	22	30349534	1.96E-01	9878	3.06E-09	Down	5.93	971	0.71	0.85
rs6418944	3547	IGSF1		130133966	1.96E-01	9879	7.35E-09	Down	5.78	1035	0.71	0.81
rs11085825	10661	KLF1	19	12868458	1.96E-01	9880	4.55E-02	Down	2.00	5886	0.71	0.13
rs11085825	1777	DNASE2	19	12868458	1.96E-01	9881	3.14E-01	Down	1.01	9307	0.71	0.05
rs11640526	317702	VN1R3	16	31728308	1.96E-01	9882	9.86E-01	Up	0.02	13820	0.71	0.00
rs11544042	10713	USP39	2	85755006	1.96E-01	9883	6.67E-01	Up	0.43	11869	0.71	0.02
rs1259022	112936	VPS26B	11	133610240	1.96E-01	9884	2.63E-06	Up	4.70	1712	0.71	0.56
rs1259022	27034	ACAD8	11	133610240	1.96E-01	9885	2.33E-03	Up	3.04	3641	0.71	0.26
rs1105547	255061	TAC4	17	45267796	1.97E-01	9886	3.95E-03	Down	2.88	3921	0.71	0.24
rs4582459	28988	DBNL	7	43872105	1.97E-01	9887	2.61E-01	Down	1.13	8865	0.71	0.06
rs598855	64326	RFWD2	1	172873523	1.97E-01	9888	2.16E-01	Up	1.24	8423	0.71	0.07
rs1984224	639	PRDM1	6	106644047	1.97E-01	9889	6.55E-01	Up	0.45	11798	0.71	0.02
rs2412547	5888	RAD51	15	38799987	1.97E-01	9890	2.52E-01	Up	1.15	8779	0.71	0.06
rs9762579	152756	C4orf39	4	166235595	1.97E-01	9891	1.20E-02	Down	2.51	4638	0.71	0.19
rs9796234	496	ATP4B	13	113371998	1.97E-01	9892	2.77E-01	Up	1.09	9005	0.71	0.06
rs9796234	6011	GRK1	13	113371998	1.97E-01	9893	9.01E-01	Down	0.12	13253	0.71	0.00
rs570844	6584	SLC22A5	10	102122633	1.97E-01	9894	2.30E-01	Up	1.20	8562	0.71	0.06
rs570844	6319	SCD	10	102122633	1.97E-01	9895	5.33E-01	Down	0.62	10967	0.71	0.03
rs4839391	84432	PROK1	1	110706032	1.97E-01	9896	7.25E-01	Down	0.35	12228	0.71	0.01
rs3788426	55000	TUG1	22	29678224	1.97E-01	9897	7.97E-01	Up	0.26	12651	0.71	0.01
rs3760386	79990	PLEKHH3	17	38095610	1.97E-01	9898	1.05E-03	Down	3.28	3241	0.71	0.30
rs3791221	26751	SH3YL1	2	216933	1.97E-01	9899	3.83E-12	Up	6.95	617	0.71	1.14
rs353547	132160	PPM1M	3	52243906	1.97E-01	9900	7.45E-03	Down	2.68	4298	0.71	0.21
rs4662721	10746	MAP3K2	2	127818648	1.97E-01	9901	1.88E-02	Down	2.35	5013	0.71	0.17
rs2230265	2245	FGD1		54355527	1.97E-01	9902	7.12E-01	Up	0.37	12148	0.71	0.01
rs12137480	127396	ZNF684	1	40677535	1.97E-01	9903	3.24E-02	Up	2.14	5541	0.71	0.15
rs10864390	80045	GPR157	1	9117180	1.97E-01	9904	6.85E-01	Up	0.41	11979	0.71	0.02
rs11690921	90411	MCFD2	2	47065413	1.97E-01	9905	7.65E-01	Down	0.30	12463	0.70	0.01
rs7558942	666	BOK	2	242229002	1.97E-01	9906	3.58E-01	Down	0.92	9662	0.70	0.04
rs4979142	158405	KIAA1958	9	112500979	1.97E-01	9907	4.71E-05	Up	4.07	2266	0.70	0.43
rs10489095	9364	RAB28	4	13178681	1.98E-01	9908	1.42E-02	Down	2.45	4763	0.70	0.18
rs1029420	2242	FES	15	89242090	1.98E-01	9909	4.38E-03	Down	2.85	3983	0.70	0.24
rs10405511	162998	OR7D2	19	9156591	1.98E-01	9910	3.16E-01	Up	1.00	9329	0.70	0.05
rs2093107	1137	CHRNA4	20	61464386	1.98E-01	9911	9.45E-01	Up	0.07	13525	0.70	0.00
rs1665105	57464	FAM40B	7	128662929	1.98E-01	9912	2.22E-01	Up	1.22	8482	0.70	0.07
rs719787	10981	RAB32	6	146891370	1.98E-01	9913	6.39E-02	Down	1.85	6323	0.70	0.12
rs8096750	54877	ZCCHC2	18	58388368	1.98E-01	9914	5.41E-03	Down	2.78	4115	0.70	0.23
rs287624	80320	SP6	7	129895260	1.98E-01	9915	7.95E-03	Down	2.65	4338	0.70	0.21
rs287624	136259	KLF14	7	129895260	1.98E-01	9916	2.20E-01	Down	1.23	8454	0.70	0.07
rs12749354	5321	PLA2G4A	1	183587905	1.98E-01	9917	5.50E-03	Down	2.78	4127	0.70	0.23
rs10280960	115330	GPR146	7	876938	1.98E-01	9918	5.30E-01	Down	0.63	10937	0.70	0.03
rs3759495	3936	LCP1	13	45657269	1.98E-01	9919	1.55E-01	Up	1.42	7747	0.70	0.08
rs3759495	9878	TOX4	13	45657269	1.98E-01	9920	1.73E-01	Up	1.36	7942	0.70	0.08
rs132905	7008	TEF	22	40123606	1.98E-01	9921	2.12E-01	Down	1.25	8380	0.70	0.07
rs3091242	23585	TMEM50A	1	25420101	1.98E-01	9922	2.67E-03	Down	3.00	3721	0.70	0.26
rs2959265	10736	SIX2	2	45161077	1.98E-01	9923	7.31E-01	Down	0.34	12253	0.70	0.01
rs4788821	91862	MARVELD3	16	70217811	1.98E-01	9924	2.56E-01	Up	1.14	8819	0.70	0.06
rs9442907	55510	DDX43	6	74180084	1.98E-01	9925	2.40E-01	Up	1.18	8654	0.70	0.06
rs4720539	26100	WIPI2	7	5040765	1.98E-01	9926	2.51E-03	Up	3.02	3690	0.70	0.26
rs3851113	79097	TRIM48	11	54784999	1.98E-01	9927	8.90E-02	Up	1.70	6785	0.70	0.11
rs10804661	668	FOXL2	3	140136186	1.98E-01	9928	2.20E-01	Down	1.23	8462	0.70	0.07
rs9535836	9724	UTP14C	13	51507916	1.99E-01	9929	3.71E-01	Down	0.89	9778	0.70	0.04
rs3130617	57827	C6orf47	6	31735502	1.99E-01	9930	1.12E-03	Down	3.26	3262	0.70	0.30
rs3130617	7917	BAT3	6	31735502	1.99E-01	9931	7.41E-03	Up	2.68	4295	0.70	0.21
rs3130617	55937	APOM	6	31735502	1.99E-01	9932	2.57E-01	Down	1.13	8830	0.70	0.06
rs3130617	1460	CSNK2B	6	31735502	1.99E-01	9933	4.95E-01	Up	0.68	10678	0.70	0.03
rs3130617	58496	LY6G5B	6	31735502	1.99E-01	9934	5.04E-01	Down	0.67	10747	0.70	0.03
rs3130617	7918	BAT4	6	31735502	1.99E-01	9935	8.34E-01	Up	0.21	12871	0.70	0.01
rs17834108	118	ADD1	4	2947710	1.99E-01	9936	2.59E-04	Down	3.65	2722	0.70	0.36
rs1548412	6545	SLC7A4	22	19701888	1.99E-01	9937	6.55E-01	Down	0.45	11799	0.70	0.02
rs4899460	8650	NUMB	14	72866989	1.99E-01	9938	2.58E-01	Up	1.13	8846	0.70	0.06
rs9399083	9519	TBPL1	6	134333950	1.99E-01	9939	4.95E-05	Up	4.06	2284	0.70	0.43
rs2278291	54807	ZNF586	19	62989408	1.99E-01	9940	1.43E-01	Down	1.46	7607	0.70	0.08
rs841227	84309	NUDT16L1	16	4674514	1.99E-01	9941	1.39E-01	Up	1.48	7562	0.70	0.09
rs7433481	8820	HESX1	3	57201631	1.99E-01	9942	4.77E-01	Up	0.71	10552	0.70	0.03
rs3120693	64216	TFB2M	1	243024694	1.99E-01	9943	1.51E-01	Up	1.44	7690	0.70	0.08
rs1801311	4700	NDUFA6	22	40811223	1.99E-01	9944	3.70E-04	Down	3.56	2854	0.70	0.34
rs1801311	4668	NAGA	22	40811223	1.99E-01	9945	1.04E-02	Down	2.56	4543	0.70	0.20
rs1426810	1974	EIF4A2	3	187986137	1.99E-01	9946	4.52E-01	Up	0.75	10360	0.70	0.03
rs12364461	5029	P2RY2	11	72612267	1.99E-01	9947	3.52E-03	Down	2.92	3866	0.70	0.25
rs5959255	84636	GPR174		78231331	1.99E-01	9948	9.10E-03	Down	2.61	4442	0.70	0.20
rs11913210	23774	BRD1	22	48516053	1.99E-01	9949	1.01E-01	Down	1.64	7006	0.70	0.10
rs3756007	2555	GABRA2	4	46231992	1.99E-01	9950	2.13E-01	Up	1.25	8388	0.70	0.07
rs6790048	2596	GAP43	3	116872244	2.00E-01	9951	1.03E-01	Up	1.63	7039	0.70	0.10
rs6789870	131578	LRRC15	3	195561214	2.00E-01	9952	1.48E-01	Down	1.45	7655	0.70	0.08
rs1541042	23443	SLC35A3	1	100142741	2.00E-01	9953	1.70E-15	Up	7.94	369	0.70	1.48
rs4801810	79735	TBC1D17	19	55092860	2.00E-01	9954	9.65E-01	Up	0.04	13648	0.70	0.00
rs10946808	8353	HIST1H3E	6	26341366	2.00E-01	9955	5.54E-03	Up	2.77	4136	0.70	0.23
rs11191727	9148	NEURL	10	105312363	2.00E-01	9956	3.94E-02	Down	2.06	5733	0.70	0.14
rs6673301	845	CASQ2	1	115953253	2.00E-01	9957	4.15E-03	Down	2.87	3955	0.70	0.24
rs734158	26206	SPAG8	9	35813009	2.00E-01	9958	6.69E-02	Down	1.83	6373	0.70	0.12

rs734158	84681	HINT2	9	35813009	2.00E-01	9959	4.42E-01	Down	0.77	10281	0.70	0.04
rs4932742	9534	ZNF254	19	24047473	2.00E-01	9960	1.24E-03	Up	3.23	3308	0.70	0.29
rs899432	27031	NPHP3	3	133940093	2.00E-01	9961	1.07E-01	Down	1.61	7108	0.70	0.10
rs10274145	202915	TMEM184A	7	1387502	2.00E-01	9962	7.17E-01	Down	0.36	12175	0.70	0.01
rs371825	255101	CCDC108	2	219689161	2.00E-01	9963	8.13E-01	Up	0.24	12739	0.70	0.01
rs371825	1412	CRYBA2	2	219689161	2.00E-01	9964	8.75E-01	Up	0.16	13092	0.70	0.01
rs17434286	55589	BMP2K	4	80111833	2.00E-01	9965	1.02E-09	Up	6.11	893	0.70	0.90
rs8060347	221188	GPR114	16	56145135	2.00E-01	9966	2.89E-01	Down	1.06	9101	0.70	0.05
rs1321075	860	RUNX2	6	45499921	2.00E-01	9967	3.78E-08	Down	5.50	1193	0.70	0.74
rs2189659	5201	PFDN1	5	139677404	2.00E-01	9968	2.83E-01	Up	1.07	9054	0.70	0.05
rs6105777	11034	DSTN	20	17494222	2.01E-01	9969	5.59E-13	Down	7.21	532	0.70	1.23
rs4646537	1019	CDK4	12	56443548	2.01E-01	9970	1.48E-07	Up	5.26	1342	0.70	0.68
rs4646537	10102	TSFM	12	56443548	2.01E-01	9971	1.88E-06	Up	4.77	1655	0.70	0.57
rs4646537	4234	METTL1	12	56443548	2.01E-01	9972	2.83E-05	Up	4.19	2158	0.70	0.45
rs4646537	25895	FAM119B	12	56443548	2.01E-01	9973	1.52E-03	Up	3.17	3389	0.70	0.28
rs4646537	1594	CYP27B1	12	56443548	2.01E-01	9974	9.37E-01	Down	0.08	13473	0.70	0.00
rs1464245	130340	AP1S3	2	224534251	2.01E-01	9975	4.83E-01	Up	0.70	10594	0.70	0.03
rs9456047	168002	DACT2	6	168522434	2.01E-01	9976	2.41E-01	Down	1.17	8666	0.70	0.06
rs968529	217	ALDH2	12	110697088	2.01E-01	9977	2.48E-09	Down	5.96	947	0.70	0.86
rs4652781	3918	LAMC2	1	179867853	2.01E-01	9978	2.33E-01	Up	1.19	8597	0.70	0.06
rs3885951	5685	PSMA4	15	76612972	2.01E-01	9979	5.28E-04	Up	3.47	2979	0.70	0.33
rs878291	29893	PSMC3IP	17	37981755	2.01E-01	9980	2.63E-01	Down	1.12	8888	0.70	0.06
rs3820623	7483	WNT9A	1	224413997	2.01E-01	9981	2.44E-01	Up	1.16	8708	0.70	0.06
rs12047887	3737	KCNA2	1	110856049	2.01E-01	9982	6.40E-01	Down	0.47	11714	0.70	0.02
rs4491793	1469	CST1	20	23681242	2.01E-01	9983	2.56E-02	Up	2.23	5309	0.70	0.16
rs9369454	64928	MRPL14	6	44195643	2.01E-01	9984	1.66E-01	Up	1.39	7873	0.70	0.08
rs781542	6691	SPINK2	4	57528232	2.01E-01	9985	2.62E-01	Up	1.12	8880	0.70	0.06
rs160544	79147	FKRP	19	51926725	2.01E-01	9986	6.21E-01	Down	0.49	11585	0.70	0.02
rs2025670	26499	PLEK2	14	66960075	2.01E-01	9987	8.80E-01	Up	0.15	13124	0.70	0.01
rs6668505	5724	PTAFR	1	28193941	2.01E-01	9988	7.32E-01	Down	0.34	12265	0.70	0.01
rs9913935	9527	GOSR1	17	25876414	2.01E-01	9989	2.24E-05	Up	4.24	2108	0.70	0.47
rs12128686	2215	FCGR3B	1	158331812	2.01E-01	9990	6.49E-02	Up	1.85	6341	0.70	0.12
rs12128686	2214	FCGR3A	1	158331812	2.01E-01	9991	8.05E-01	Down	0.25	12700	0.70	0.01
rs2147167	4093	SMAD9	13	36365404	2.01E-01	9992	6.67E-01	Down	0.43	11866	0.70	0.02
rs4271068	2801	GOLGA2	9	128111912	2.01E-01	9993	3.51E-08	Up	5.52	1185	0.70	0.75
rs12338625	375757	C9orf119	9	128102695	2.01E-01	9994	6.09E-01	Up	0.51	11497	0.70	0.02
rs17353380	27019	DNAI1	9	34502893	2.01E-01	9995	2.33E-02	Up	2.27	5214	0.70	0.16
rs35837	9861	PSMD6	3	63988289	2.01E-01	9996	8.19E-01	Up	0.23	12787	0.70	0.01
rs6989203	157848	NKX6-3	8	41642902	2.01E-01	9997	3.79E-01	Up	0.88	9828	0.70	0.04
rs954145	284695	ZNF326	1	90210561	2.02E-01	9998	4.03E-02	Up	2.05	5754	0.70	0.14
rs3823646	221914	GPC2	7	99402263	2.02E-01	9999	3.67E-01	Up	0.90	9732	0.70	0.04
rs3823646	10734	STAG3	7	99402263	2.02E-01	10000	6.73E-01	Down	0.42	11905	0.70	0.02
rs1045253	2629	GBA	1	152014308	2.02E-01	10001	8.33E-01	Down	0.21	12868	0.70	0.01
rs1938302	57643	ZSWIM5	1	45346911	2.02E-01	10002	5.18E-01	Down	0.65	10848	0.70	0.03
rs7055734	54413	NLGN3		70175575	2.02E-01	10003	2.70E-02	Down	2.21	5361	0.70	0.16
rs9898774	84254	CAMKK1	17	3725228	2.02E-01	10004	5.65E-01	Up	0.58	11202	0.70	0.02
rs1797052	11126	CD160	1	143216727	2.02E-01	10005	1.01E-01	Down	1.64	7026	0.70	0.10
rs9608247	56241	SUSD2	22	22911857	2.02E-01	10006	2.78E-01	Down	1.08	9016	0.70	0.06
rs921863	26048	ZNF500	16	4741724	2.02E-01	10007	7.37E-01	Down	0.34	12295	0.70	0.01
rs4944804	10809	STARD10	11	72198178	2.02E-01	10008	5.70E-01	Up	0.57	11242	0.69	0.02
rs1572912	54662	TBC1D13	9	128645108	2.02E-01	10009	4.03E-01	Down	0.84	10008	0.69	0.04
rs1572912	2021	ENDOG	9	128645108	2.02E-01	10010	4.75E-01	Up	0.71	10541	0.69	0.03
rs339083	64753	CCDC136	7	128019719	2.02E-01	10011	7.98E-04	Down	3.35	3136	0.69	0.31
rs4471334	51806	CALML5	10	5527569	2.02E-01	10012	5.37E-01	Up	0.62	10989	0.69	0.03
rs1560725	5971	RELB	19	50235627	2.02E-01	10013	7.21E-04	Down	3.38	3089	0.69	0.31
rs610308	7288	TULP2	19	54069131	2.02E-01	10014	9.04E-03	Down	2.61	4437	0.69	0.20
rs610308	23645	PPP1R15A	19	54069131	2.02E-01	10015	5.24E-01	Down	0.64	10891	0.69	0.03
rs1860181	6356	CCL11	17	29641700	2.02E-01	10016	1.69E-01	Up	1.38	7906	0.69	0.08
rs8007293	10175	CNIH	14	53987417	2.02E-01	10017	6.00E-01	Up	0.52	11432	0.69	0.02
rs2945152	1486	CTBS	1	84760662	2.02E-01	10018	7.01E-02	Up	1.81	6426	0.69	0.12
rs13160147	133686	C5orf33	5	36227164	2.02E-01	10019	7.76E-05	Up	3.95	2382	0.69	0.41
rs2291897	25827	FBXL2	3	33394426	2.02E-01	10020	4.94E-01	Down	0.68	10672	0.69	0.03
rs16831647	50717	WDR42A	1	157010025	2.02E-01	10021	9.19E-01	Up	0.10	13369	0.69	0.00
rs7587636	1788	DNMT3A	2	25430513	2.02E-01	10022	7.52E-10	Up	6.15	877	0.69	0.91
rs8007427	283600	C14orf68	14	99841325	2.02E-01	10023	1.05E-01	Up	1.62	7069	0.69	0.10
rs400037	8786	RGS11	16	276397	2.03E-01	10024	4.98E-06	Up	4.57	1818	0.69	0.53
rs400037	398	ARHGDIG	16	276397	2.03E-01	10025	6.25E-01	Up	0.49	11617	0.69	0.02
rs884113	54461	FBXW5	9	137117349	2.03E-01	10026	2.44E-01	Up	1.16	8707	0.69	0.06
rs884113	733	C8G	9	137117349	2.03E-01	10027	3.89E-01	Up	0.86	9910	0.69	0.04
rs2665833	117246	FTSJ3	17	59260578	2.03E-01	10028	5.41E-03	Up	2.78	4116	0.69	0.23
rs2665833	5705	PSMC5	17	59260578	2.03E-01	10029	7.53E-01	Up	0.32	12394	0.69	0.01
rs6976053	43	ACHE	7	100156770	2.03E-01	10030	5.46E-01	Up	0.60	11056	0.69	0.03
rs2304748	80185	C8orf41	8	33489486	2.03E-01	10031	2.60E-01	Down	1.13	8858	0.69	0.06
rs1772283	164127	C1orf65	1	219924968	2.03E-01	10032	9.12E-01	Down	0.11	13324	0.69	0.00
rs429083	8635	RNASET2	6	167354383	2.03E-01	10033	1.76E-03	Up	3.13	3466	0.69	0.28
rs12813376	4326	MMP17	12	130968554	2.03E-01	10034	9.04E-01	Up	0.12	13278	0.69	0.00
rs7827287	9420	CYP7B1	8	65768273	2.03E-01	10035	1.27E-01	Up	1.53	7403	0.69	0.09
rs6122777	55661	DDX27	20	47302598	2.03E-01	10036	1.22E-01	Down	1.55	7335	0.69	0.09
rs6910087	4276	MICA	6	31485026	2.03E-01	10037	5.65E-02	Down	1.91	6147	0.69	0.12
rs7094415	84445	LZTS2	10	102766860	2.03E-01	10038	7.12E-01	Down	0.37	12141	0.69	0.01
rs10799722	23436	ELA3B	1	22053822	2.03E-01	10039	1.02E-01	Up	1.64	7035	0.69	0.10

rs9912556	146779	EFCAB3	17	57806382	2.03E-01	10040	3.74E-01	Down	0.89	9800	0.69	0.04
rs274137	1296	COL8A2	1	36247038	2.03E-01	10041	7.09E-04	Down	3.39	3081	0.69	0.31
rs4700810	80230	RUFY1	5	178924400	2.03E-01	10042	1.86E-01	Down	1.32	8098	0.69	0.07
rs3792091	6295	SAG	2	234033322	2.03E-01	10043	8.93E-01	Down	0.14	13199	0.69	0.00
rs3749237	29925	GMPPB	3	49745036	2.03E-01	10044	5.88E-02	Up	1.89	6213	0.69	0.12
rs3749237	386724	AMIGO3	3	49745036	2.03E-01	10045	3.27E-01	Down	0.98	9421	0.69	0.05
rs12293750	51352	WIT1	11	32403561	2.03E-01	10046	6.17E-01	Up	0.50	11552	0.69	0.02
rs12293750	7490	WT1	11	32403561	2.03E-01	10047	9.06E-01	Up	0.12	13284	0.69	0.00
rs2547305	27181	SIGLEC8	19	56639096	2.03E-01	10048	5.20E-01	Down	0.64	10858	0.69	0.03
rs7752945	9444	QKI	6	163968721	2.03E-01	10049	4.19E-02	Down	2.03	5795	0.69	0.14
rs446227	6101	RP1	8	55704003	2.04E-01	10050	4.07E-01	Down	0.83	10033	0.69	0.04
rs1476892	10652	YKT6	7	44017193	2.04E-01	10051	1.54E-01	Up	1.42	7737	0.69	0.08
rs12462622		B3GNT3	19	17788285	2.04E-01	10052	3.72E-02	Down	2.08	5680	0.69	0.14
rs7212099	90507	SCRN2	17	43280889	2.04E-01	10053	4.74E-03	Down	2.82	4025	0.69	0.23
rs7212099	124995	MRPL10	17	43280889	2.04E-01	10054	2.84E-01	Up	1.07	9069	0.69	0.05
rs10818789	55342	STRBP	9	123036015	2.04E-01	10055	1.14E-01	Up	1.58	7206	0.69	0.09
rs8114696	2691	GHRH	20	35304638	2.04E-01	10056	6.72E-01	Down	0.42	11895	0.69	0.02
rs12675850	23212	RRS1	8	67511094	2.04E-01	10057	2.32E-04	Up	3.68	2683	0.69	0.36
rs9767555	11104	KATNA1	6	150009851	2.04E-01	10058	1.61E-01	Down	1.40	7822	0.69	0.08
rs7830160	65986	ZBTB10	8	81544426	2.04E-01	10059	2.60E-10	Up	6.32	801	0.69	0.96
rs10057083	1452	CSNK1A1	5	148854047	2.04E-01	10060	4.80E-08	Up	5.46	1219	0.69	0.73
rs8034551	374659	HDDC3	15	89287309	2.04E-01	10061	3.77E-04	Up	3.56	2859	0.69	0.34
rs7708414	79668	PARP8	5	50069900	2.04E-01	10062	2.28E-03	Up	3.05	3619	0.69	0.26
rs11068958	9815	GIT2	12	108836380	2.04E-01	10063	7.39E-01	Down	0.33	12310	0.69	0.01
rs847146	3235	HOXD9	2	176798589	2.04E-01	10064	5.47E-06	Down	4.55	1833	0.69	0.53
rs847146	3236	HOXD10	2	176798589	2.04E-01	10065	5.39E-04	Down	3.46	2984	0.69	0.33
rs847146	3237	HOXD11	2	176798589	2.04E-01	10066	2.14E-03	Down	3.07	3573	0.69	0.27
rs847146	3238	HOXD12	2	176798589	2.04E-01	10067	6.23E-01	Down	0.49	11603	0.69	0.02
rs782586	57223	SMEK2	2	55747816	2.04E-01	10068	3.47E-03	Up	2.92	3852	0.69	0.25
rs3213544	10389	SCML2		18101691	2.04E-01	10069	3.72E-01	Down	0.89	9780	0.69	0.04
rs1560730	92609	TIMM50	19	44647631	2.04E-01	10070	2.93E-02	Up	2.18	5439	0.69	0.15
rs1575279	6013	RLN1	9	5335673	2.04E-01	10071	9.48E-01	Down	0.07	13537	0.69	0.00
rs10489605	2730	GCLM	1	94064402	2.04E-01	10072	6.23E-01	Up	0.49	11602	0.69	0.02
rs7194895	4496	MT1H	16	55254592	2.04E-01	10073	6.32E-09	Down	5.81	1026	0.69	0.82
rs7194895	4490	MT1B	16	55254592	2.04E-01	10074	4.25E-07	Down	5.06	1460	0.69	0.64
rs11085023	10362	HMG20B	19	3522446	2.04E-01	10075	6.61E-18	Up	8.63	271	0.69	1.72
rs11085023	126326	GIPC3	19	3522446	2.04E-01	10076	8.75E-01	Up	0.16	13091	0.69	0.01
rs12114565	6591	SNAI2	8	49985036	2.04E-01	10077	2.95E-39	Down	13.11	19	0.69	3.85
rs10490302	80204	FBXO11	2	47951395	2.04E-01	10078	3.59E-02	Up	2.10	5644	0.69	0.14
rs6609437	390916	NUDT19		46442329	2.05E-01	10079	8.32E-05	Up	3.93	2403	0.69	0.41
rs6609437	6102	RP2		46442329	2.05E-01	10080	1.59E-02	Up	2.41	4873	0.69	0.18
rs2336029	170063	CXorf22		35728954	2.05E-01	10081	9.34E-01	Down	0.08	13457	0.69	0.00
rs755163	89777	SERPINB12	18	59384115	2.05E-01	10082	2.77E-01	Up	1.09	9006	0.69	0.06
rs8042411	51187	C15orf15	15	53272786	2.05E-01	10083	1.05E-01	Up	1.62	7087	0.69	0.10
rs3743266	79664	NARG2	15	58568805	2.05E-01	10084	8.83E-04	Up	3.33	3171	0.69	0.31
rs1252951	55320	C14orf106	14	44798484	2.05E-01	10085	6.35E-06	Up	4.51	1856	0.69	0.52
rs7081796	84287	ZDHHC16	10	99194516	2.05E-01	10086	1.75E-02	Up	2.38	4958	0.69	0.18
rs7081796	51013	EXOSC1	10	99194516	2.05E-01	10087	3.07E-02	Down	2.16	5486	0.69	0.15
rs11880140	92960	PEX11G	19	7444117	2.05E-01	10088	3.18E-01	Up	1.00	9345	0.69	0.05
rs11090076	1565	CYP2D6	22	40838690	2.05E-01	10089	6.66E-04	Down	3.40	3061	0.69	0.32
rs1037257	6752	SSTR2	17	68662948	2.05E-01	10090	4.00E-02	Down	2.05	5749	0.69	0.14
rs6876997	6932	TCF7	5	133505610	2.05E-01	10091	5.69E-01	Down	0.57	11235	0.69	0.02
rs12477095	8436	SDPR	2	192525205	2.05E-01	10092	2.41E-06	Down	4.72	1697	0.69	0.56
rs11166413	1629	DBT	1	100368515	2.05E-01	10093	8.62E-03	Up	2.63	4396	0.69	0.21
rs11166413	127495	LRRC39	1	100368515	2.05E-01	10094	1.62E-02	Up	2.40	4897	0.69	0.18
rs301694	339416	ANKRD45	1	170303089	2.05E-01	10095	7.81E-01	Down	0.28	12552	0.69	0.01
rs301694	284525	SLC9A11	1	170303089	2.05E-01	10096	8.48E-01	Up	0.19	12940	0.69	0.01
rs1051442	7057	THBS1	15	37674941	2.05E-01	10097	1.04E-08	Down	5.72	1061	0.69	0.80
rs11636245	56905	C15orf39	15	73270805	2.05E-01	10098	2.11E-01	Up	1.25	8358	0.69	0.07
rs411089	909	CD1A	1	155037898	2.05E-01	10099	6.26E-01	Up	0.49	11620	0.69	0.02
rs1805152	27129	HSPB7	1	16101807	2.06E-01	10100	1.28E-01	Down	1.52	7428	0.69	0.09
rs1805152	1187	CLCNKA	1	16101807	2.06E-01	10101	3.03E-01	Down	1.03	9212	0.69	0.05
rs39681	9971	NR1H4	12	99469317	2.06E-01	10102	4.87E-01	Down	0.70	10625	0.69	0.03
rs955915	84804	MFSD9	2	102810254	2.06E-01	10103	4.27E-01	Up	0.79	10170	0.69	0.04
rs9916485	83902	KRTAP17-1	17	36728319	2.06E-01	10104	8.42E-01	Down	0.20	12914	0.69	0.01
rs7297320	121536	AEBP2	12	19491448	2.06E-01	10105	8.14E-05	Down	3.94	2397	0.69	0.41
rs2029623	2318	FLNC	7	128101248	2.06E-01	10106	3.29E-19	Down	8.96	222	0.69	1.85
rs2029623	9296	ATP6V1F	7	128101248	2.06E-01	10107	1.08E-06	Up	4.88	1586	0.69	0.60
rs518721	57823	SLAMF7	1	157536590	2.06E-01	10108	9.95E-01	Up	0.01	13877	0.69	0.00
rs3771744	57835	SLC4A5	2	74490191	2.06E-01	10109	2.57E-06	Up	4.70	1706	0.69	0.56
rs3771744	1639	DCTN1	2	74490191	2.06E-01	10110	1.66E-03	Down	3.15	3435	0.69	0.28
rs12564699	27252	KLHL20	1	170409696	2.06E-01	10111	1.89E-03	Down	3.11	3507	0.69	0.27
rs7185307	608	TNFRSF17	16	11954400	2.06E-01	10112	8.36E-02	Up	1.73	6696	0.69	0.11
rs2772372	221504	ZBTB9	6	33535328	2.06E-01	10113	1.92E-01	Up	1.31	8167	0.69	0.07
rs2238973	4693	NDP		43570337	2.06E-01	10114	3.51E-05	Down	4.14	2204	0.69	0.45
rs504103	23546	SYNGR4	19	53545465	2.06E-01	10115	2.96E-01	Up	1.04	9164	0.69	0.05
rs3812259	64005	MYO1G	7	44773103	2.06E-01	10116	5.63E-01	Up	0.58	11184	0.69	0.02
rs12938061	23495	TNFRSF13B	17	16786880	2.06E-01	10117	3.41E-04	Down	3.58	2823	0.69	0.35
rs17759085	83893	SPATA16	3	174152208	2.06E-01	10118	5.77E-01	Up	0.56	11293	0.69	0.02
rs2501278	998	CDC42	1	22117017	2.06E-01	10119	6.03E-01	Up	0.52	11455	0.69	0.02
rs4674267	3577	IL8RA	2	218871943	2.07E-01	10120	5.42E-01	Down	0.61	11036	0.68	0.03

rs4782286	124152	IQCK	16	19741923	2.07E-01	10121	1.99E-06	Down	4.75	1669	0.68	0.57
rs6431590	55502	HES6	2	238947128	2.07E-01	10122	4.71E-01	Up	0.72	10503	0.68	0.03
rs11547035	203259	C9orf25	9	34449025	2.07E-01	10123	1.24E-04	Down	3.84	2498	0.68	0.39
rs10088108	526	ATP6V1B2	8	20107092	2.07E-01	10124	6.11E-01	Up	0.51	11516	0.68	0.02
rs17700714	55226	NAT10	11	34101249	2.07E-01	10125	8.58E-01	Down	0.18	13000	0.68	0.01
rs2325216	7178	TPT1	13	44807981	2.07E-01	10126	4.97E-01	Up	0.68	10695	0.68	0.03
rs2275171	80896	NPL	1	179515679	2.07E-01	10127	2.11E-02	Down	2.31	5126	0.68	0.17
rs4819000	85395	C21orf70	21	45208369	2.07E-01	10128	4.60E-01	Down	0.74	10429	0.68	0.03
rs1057985	2030	SLC29A1	6	44293879	2.07E-01	10129	7.16E-01	Down	0.36	12172	0.68	0.01
rs1181888	148870	CCDC27	1	3694948	2.07E-01	10130	5.22E-01	Down	0.64	10872	0.68	0.03
rs11517805	116534	MRGPRE	11	3219587	2.07E-01	10131	3.14E-01	Down	1.01	9306	0.68	0.05
rs2676795	8405	SPOP	17	45076014	2.07E-01	10132	6.75E-08	Down	5.40	1254	0.68	0.72
rs6601518	83595	SOX7	8	10642644	2.07E-01	10133	1.03E-01	Down	1.63	7050	0.68	0.10
rs1783978	219541	MED19	11	57220076	2.07E-01	10134	4.78E-04	Up	3.49	2948	0.68	0.33
rs1783978	51075	TXNDC14	11	57220076	2.07E-01	10135	2.05E-01	Up	1.27	8301	0.68	0.07
rs1564295	204	AK2	1	33181985	2.07E-01	10136	5.17E-02	Up	1.95	6030	0.68	0.13
rs3801382	10447	FAM3C	7	120592228	2.07E-01	10137	1.74E-08	Up	5.64	1103	0.68	0.78
rs9606756	23481	PES1	22	29331414	2.07E-01	10138	2.04E-02	Up	2.32	5092	0.68	0.17
rs849370	5294	PIK3CG	7	106113894	2.08E-01	10139	1.20E-02	Up	2.51	4636	0.68	0.19
rs2769069	138199	C9orf41	9	74837185	2.08E-01	10140	3.04E-08	Up	5.54	1166	0.68	0.75
rs3758780	5612	PRKRIR	11	75771667	2.08E-01	10141	1.30E-04	Up	3.83	2513	0.68	0.39
rs1455915	1846	DUSP4	8	29262045	2.08E-01	10142	6.32E-03	Down	2.73	4193	0.68	0.22
rs1379707	2250	FGF5	4	81537558	2.08E-01	10143	9.12E-01	Down	0.11	13323	0.68	0.00
rs11990855	55156	ARMC1	8	66689638	2.08E-01	10144	4.03E-01	Up	0.84	10000	0.68	0.04
rs10889677	149233	IL23R	1	67437141	2.08E-01	10145	9.57E-01	Down	0.05	13599	0.68	0.00
rs2025258	10548	TM9SF1	14	23724329	2.08E-01	10146	2.25E-02	Up	2.28	5181	0.68	0.16
rs2068825	10114	HIPK3	11	33302464	2.08E-01	10147	3.46E-08	Down	5.52	1184	0.68	0.75
rs7115477	10527	IPO7	11	9386195	2.08E-01	10148	5.86E-02	Up	1.89	6206	0.68	0.12
rs16973285	282616	IL28A	19	44436536	2.08E-01	10149	6.45E-02	Up	1.85	6337	0.68	0.12
rs16973285	282617	IL28B	19	44436536	2.08E-01	10150	4.56E-01	Down	0.75	10391	0.68	0.03
rs10038086	83890	SPATA9	5	95048167	2.09E-01	10151	5.54E-01	Down	0.59	11107	0.68	0.03
rs12255839	23283	CSTF2T	10	53137204	2.09E-01	10152	1.86E-01	Down	1.32	8104	0.68	0.07
rs3752591	115650	TNFRSF13C	22	40664016	2.09E-01	10153	1.41E-01	Up	1.47	7592	0.68	0.08
rs6458238	5225	PGC	6	41825683	2.09E-01	10154	9.13E-01	Up	0.11	13330	0.68	0.00
rs11880374	126282	TNFAIP8L1	19	4587946	2.09E-01	10155	4.72E-02	Up	1.98	5928	0.68	0.13
rs8111	7148	TNXB	6	32191153	2.09E-01	10156	5.00E-02	Down	1.96	5997	0.68	0.13
rs3747941	80305	TRABD	22	48939650	2.09E-01	10157	9.33E-03	Up	2.60	4456	0.68	0.20
rs7651929	285268	ZNF621	3	40541004	2.09E-01	10158	4.66E-02	Up	1.99	5915	0.68	0.13
rs10220437	1033	CDKN3	14	53964046	2.09E-01	10159	2.16E-02	Up	2.30	5144	0.68	0.17
rs11186642	5507	PPP1R3C	10	93393048	2.09E-01	10160	7.54E-17	Down	8.34	315	0.68	1.61
rs12241923	221002	RASGEF1A	10	43033053	2.09E-01	10161	2.12E-01	Down	1.25	8386	0.68	0.07
rs5953452	139341	FUNDC1		44177540	2.09E-01	10162	5.86E-02	Up	1.89	6207	0.68	0.12
rs12041215	64801	ARV1	1	227404834	2.10E-01	10163	1.57E-02	Up	2.42	4860	0.68	0.18
rs7528905	11080	DNAJB4	1	78168376	2.10E-01	10164	3.64E-06	Down	4.63	1770	0.68	0.54
rs7528905	8880	FUBP1	1	78168376	2.10E-01	10165	6.52E-04	Up	3.41	3056	0.68	0.32
rs9902474	26118	WSB1	17	22630151	2.10E-01	10166	5.00E-09	Down	5.85	1011	0.68	0.83
rs8014403	8892	EIF2B2	14	74525761	2.10E-01	10167	5.68E-04	Down	3.45	3008	0.68	0.32
rs1390450	83941	TM2D1	1	61852409	2.10E-01	10168	8.33E-01	Down	0.21	12870	0.68	0.01
rs2842197	81888	HYI	1	43596970	2.10E-01	10169	2.06E-03	Down	3.08	3550	0.68	0.27
rs3771105	5861	RAB1A	2	65235740	2.10E-01	10170	1.16E-01	Down	1.57	7235	0.68	0.09
rs12435764	85439	STON2	14	80906619	2.10E-01	10171	2.44E-02	Down	2.25	5263	0.68	0.16
rs3784934	23563	CHST5	16	74127240	2.10E-01	10172	1.67E-01	Down	1.38	7888	0.68	0.08
rs7218656	51	ACOX1	17	71468385	2.10E-01	10173	4.11E-03	Up	2.87	3950	0.68	0.24
rs548726	261734	NPHP4	1	5847887	2.11E-01	10174	2.51E-02	Up	2.24	5292	0.68	0.16
rs8130607	29761	USP25	21	16040093	2.11E-01	10175	9.45E-02	Down	1.67	6903	0.68	0.10
rs1994487	25978	CHMP2B	3	87345688	2.11E-01	10176	3.02E-07	Down	5.12	1419	0.68	0.65
rs10824274	142891	SAMD8	10	76538473	2.11E-01	10177	4.48E-01	Down	0.76	10328	0.68	0.03
rs10824274	51207	DUSP13	10	76538473	2.11E-01	10178	5.10E-01	Up	0.66	10784	0.68	0.03
rs6083128	140880	CST11	20	23376458	2.11E-01	10179	7.52E-01	Up	0.32	12393	0.68	0.01
rs11590496	79762	C1orf115	1	217250063	2.11E-01	10180	1.07E-03	Down	3.27	3249	0.68	0.30
rs3795809	84033	OBSCN	1	224831743	2.11E-01	10181	2.17E-03	Down	3.07	3586	0.68	0.27
rs7176734	64963	MRPS11	15	86816290	2.11E-01	10182	1.14E-02	Up	2.53	4601	0.68	0.19
rs7176734	26589	MRPL46	15	86816290	2.11E-01	10183	3.70E-01	Up	0.90	9766	0.68	0.04
rs6698335	388753	C1orf31	1	230814117	2.11E-01	10184	1.70E-01	Up	1.37	7914	0.68	0.08
rs1496593	306	ANXA3	4	79836862	2.11E-01	10185	6.05E-04	Up	3.43	3030	0.68	0.32
rs3783501	4616	GADD45B	19	2428316	2.11E-01	10186	9.09E-02	Up	1.69	6844	0.68	0.10
rs6589226	5450	POU2AF1	11	110754436	2.11E-01	10187	5.93E-01	Up	0.53	11390	0.68	0.02
rs4590193	64777	RMND5B	5	177496952	2.11E-01	10188	9.05E-02	Down	1.69	6835	0.68	0.10
rs2051943	4999	ORC2L	2	201602095	2.11E-01	10189	7.86E-07	Up	4.94	1541	0.68	0.61
rs2051943	60491	NIF3L1	2	201602095	2.11E-01	10190	8.17E-01	Down	0.23	12765	0.68	0.01
rs625910	57192	MCOLN1	19	7500159	2.11E-01	10191	8.94E-03	Up	2.61	4432	0.67	0.20
rs625910	140467	ZNF358	19	7500159	2.11E-01	10192	1.32E-01	Up	1.51	7466	0.67	0.09
rs6941129	5190	PEX6	6	43063079	2.11E-01	10193	4.61E-03	Up	2.83	4012	0.67	0.23
rs6941129	5528	PPP2R5D	6	43063079	2.11E-01	10194	6.50E-01	Up	0.45	11775	0.67	0.02
rs2345424	55028	C17orf80	17	68745378	2.11E-01	10195	2.12E-01	Down	1.25	8381	0.67	0.07
rs12636277	165631	PARP15	3	123823316	2.11E-01	10196	9.73E-01	Up	0.03	13717	0.67	0.00
rs2886268	8309	ACOX2	3	58509166	2.12E-01	10197	2.06E-05	Down	4.26	2096	0.67	0.47
rs10149970	84684	INSM2	14	35059194	2.12E-01	10198	1.95E-01	Down	1.30	8199	0.67	0.07
rs8060693	333929	SNAI3	16	87285340	2.12E-01	10199	3.03E-01	Up	1.03	9215	0.67	0.05
rs261676	170261	ZCCHC12		117734537	2.12E-01	10200	3.71E-01	Down	0.89	9777	0.67	0.04
rs422140	6374	CXCL5	4	75247003	2.12E-01	10201	3.24E-01	Up	0.99	9392	0.67	0.05

rs2928596	55326	AGPAT5	8	6610695	2.13E-01	10202	1.04E-02	Up	2.56	4547	0.67	0.20
rs17058647	7871	SLMAP	3	57860103	2.13E-01	10203	5.90E-19	Down	8.89	232	0.67	1.82
rs1622986	8535	CBX4	17	75438157	2.13E-01	10204	5.80E-02	Up	1.90	6188	0.67	0.12
rs988463	140870	WFDC6	20	43612386	2.13E-01	10205	7.01E-02	Down	1.81	6427	0.67	0.12
rs988463	57119	SPINLW1	20	43612386	2.13E-01	10206	3.99E-01	Up	0.84	9980	0.67	0.04
rs988463	90199	WFDC8	20	43612386	2.13E-01	10207	6.77E-01	Up	0.42	11928	0.67	0.02
rs4800455	91768	CABLES1	18	19002731	2.13E-01	10208	8.61E-01	Down	0.17	13017	0.67	0.01
rs6702877	148545	NBPF4	1	108467476	2.13E-01	10209	5.19E-01	Up	0.65	10850	0.67	0.03
rs2297643	112817	C10orf65	10	99349977	2.13E-01	10210	5.10E-01	Down	0.66	10782	0.67	0.03
rs10229583	5078	PAX4	7	126840854	2.13E-01	10211	3.65E-02	Up	2.09	5665	0.67	0.14
rs10229583	29999	FSCN3	7	126840854	2.13E-01	10212	5.13E-01	Down	0.65	10799	0.67	0.03
rs5758093	10478	SLC25A17	22	39540528	2.13E-01	10213	1.35E-02	Up	2.47	4727	0.67	0.19
rs2059927	23478	SEC11A	15	83078490	2.13E-01	10214	4.95E-05	Up	4.06	2282	0.67	0.43
rs4880127	138311	FAM69B	9	136866771	2.13E-01	10215	2.18E-01	Down	1.23	8438	0.67	0.07
rs211170	9457	FHL5	6	97176821	2.13E-01	10216	9.08E-01	Up	0.12	13298	0.67	0.00
rs10941592	6414	SEPP1	5	42867271	2.13E-01	10217	5.77E-01	Down	0.56	11291	0.67	0.02
rs9393175	9113	LATS1	6	150090837	2.13E-01	10218	9.29E-02	Up	1.68	6883	0.67	0.10
rs6441322	3840	KPNA4	3	161726775	2.13E-01	10219	9.41E-01	Down	0.07	13500	0.67	0.00
rs453116	63893	UBE2O	17	71913399	2.13E-01	10220	1.28E-01	Up	1.52	7424	0.67	0.09
rs11124088	80146	UXS1	2	106255350	2.13E-01	10221	5.75E-03	Up	2.76	4156	0.67	0.22
rs2807362	54361	WNT4	1	22234246	2.13E-01	10222	5.09E-01	Down	0.66	10778	0.67	0.03
rs9892460	11056	DDX52	17	33049289	2.13E-01	10223	4.43E-01	Down	0.77	10294	0.67	0.04
rs3753372	29889	GNL2	1	37718466	2.13E-01	10224	6.25E-04	Up	3.42	3043	0.67	0.32
rs7306525	53373	TPCN1	12	112169567	2.13E-01	10225	3.08E-01	Down	1.02	9251	0.67	0.05
rs16992609	140691	TRIM69	19	4788501	2.14E-01	10226	5.93E-02	Down	1.89	6222	0.67	0.12
rs16992609	148022	TICAM1	19	4788501	2.14E-01	10227	5.14E-01	Up	0.65	10809	0.67	0.03
rs12460985	388552	BLOC1S3	19	50382729	2.14E-01	10228	3.76E-01	Down	0.89	9811	0.67	0.04
rs12539172	81628	TSC22D4	7	99736446	2.14E-01	10229	8.76E-04	Up	3.33	3166	0.67	0.31
rs12539172	222950	C7orf51	7	99736446	2.14E-01	10230	1.08E-01	Up	1.61	7116	0.67	0.10
rs2421847	23215	BAT2D1	1	168289257	2.14E-01	10231	1.34E-01	Down	1.50	7501	0.67	0.09
rs2302920	130752	MDH1B	2	207436847	2.14E-01	10232	1.15E-01	Up	1.58	7209	0.67	0.09
rs1415961	6707	SPRR3	1	149786537	2.14E-01	10233	4.09E-01	Down	0.83	10045	0.67	0.04
rs10511302	55347	ABHD10	3	113170446	2.14E-01	10234	1.86E-04	Up	3.74	2601	0.67	0.37
rs10511302	90102	PHLDB2	3	113170446	2.14E-01	10235	9.95E-02	Down	1.65	6991	0.67	0.10
rs3852700	6553	SLC9A5	16	65829359	2.14E-01	10236	9.20E-01	Up	0.10	13378	0.67	0.00
rs3852700	29109	FHOD1	16	65829359	2.14E-01	10237	9.77E-01	Up	0.03	13750	0.67	0.00
rs2442719	3106	HLA-B	6	31428517	2.14E-01	10238	1.87E-01	Up	1.32	8105	0.67	0.07
rs5907633	4168	MCF2		138457604	2.14E-01	10239	2.83E-01	Down	1.07	9063	0.67	0.05
rs7639294	27230	SERP1	3	151751801	2.14E-01	10240	1.07E-23	Up	10.04	128	0.67	2.30
rs4296261	54442	KCTD5	16	2690697	2.14E-01	10241	2.65E-03	Up	3.01	3719	0.67	0.26
rs6677770	8412	BCAR3	1	93778656	2.14E-01	10242	2.38E-02	Down	2.26	5237	0.67	0.16
rs2871029	7122	CLDN5	22	17888484	2.14E-01	10243	1.97E-02	Down	2.33	5056	0.67	0.17
rs2028898	2677	GGCX	2	85688928	2.14E-01	10244	1.79E-03	Up	3.12	3473	0.67	0.27
rs2028898	4144	MAT2A	2	85688928	2.14E-01	10245	2.46E-03	Down	3.03	3674	0.67	0.26
rs4647709	1643	DDB2	11	47193935	2.14E-01	10246	1.11E-11	Down	6.79	640	0.67	1.10
rs4941122	57614	KIAA1468	18	58058043	2.14E-01	10247	5.14E-01	Down	0.65	10815	0.67	0.03
rs405500	8651	SOCS1	16	11276650	2.14E-01	10248	6.40E-01	Up	0.47	11710	0.67	0.02
rs2162236	60677	BRUNOL6	15	70396752	2.15E-01	10249	1.12E-01	Down	1.59	7168	0.67	0.10
rs2059258	2303	FOXC2	16	85138867	2.15E-01	10250	3.68E-01	Down	0.90	9748	0.67	0.04
rs1560413	483	ATP1B3	3	143079546	2.15E-01	10251	1.14E-01	Down	1.58	7205	0.67	0.09
rs9930956	9351	SLC9A3R2	16	2025296	2.15E-01	10252	1.16E-01	Up	1.57	7231	0.67	0.09
rs2325750	79170	ATAD4	17	43377329	2.15E-01	10253	3.75E-10	Up	6.28	818	0.67	0.94
rs2325750	55163	PNPO	17	43377329	2.15E-01	10254	7.80E-08	Up	5.37	1270	0.67	0.71
rs6657823	864	RUNX3	1	24961800	2.15E-01	10255	2.78E-01	Down	1.08	9023	0.67	0.06
rs886589	29843	SENP1	12	46763464	2.15E-01	10256	7.94E-01	Up	0.26	12635	0.67	0.01
rs10864439	56998	CTNNBIP1	1	9837166	2.15E-01	10257	1.03E-02	Down	2.56	4537	0.67	0.20
rs10864439	22883	CLSTN1	1	9837166	2.15E-01	10258	1.84E-01	Up	1.33	8071	0.67	0.07
rs10777131	4254	KITLG	12	87464479	2.15E-01	10259	1.20E-05	Down	4.38	1988	0.67	0.49
rs2003154	3749	KCNC4	1	110462319	2.15E-01	10260	7.13E-01	Down	0.37	12153	0.67	0.01
rs2070203	16	AARS	16	68861081	2.15E-01	10261	3.61E-02	Down	2.10	5651	0.67	0.14
rs7947046	220004	C11orf66	11	61016327	2.15E-01	10262	1.33E-01	Down	1.50	7473	0.67	0.09
rs6675866	2633	GBP1	1	89229831	2.15E-01	10263	3.83E-08	Down	5.50	1197	0.67	0.74
rs13156669	29093	MRPL22	5	154326180	2.15E-01	10264	1.90E-03	Up	3.11	3509	0.67	0.27
rs9322188	116254	C6orf72	6	149951184	2.15E-01	10265	1.37E-01	Down	1.49	7530	0.67	0.09
rs7923528	80013	C10orf97	10	15929378	2.15E-01	10266	5.54E-02	Up	1.92	6122	0.67	0.13
rs6928448	57231	SNX14	6	86346134	2.15E-01	10267	1.20E-03	Down	3.24	3294	0.67	0.29
rs11878620	2077	ERF	19	47439069	2.15E-01	10268	2.74E-03	Down	3.00	3740	0.67	0.26
rs11878620	2931	GSK3A	19	47439069	2.15E-01	10269	6.51E-01	Down	0.45	11778	0.67	0.02
rs2842079	135154	C6orf57	6	71361507	2.15E-01	10270	7.38E-05	Up	3.96	2372	0.67	0.41
rs422732	10007	GNPDA1	19	39540290	2.16E-01	10271	1.53E-01	Down	1.43	7711	0.67	0.08
rs11581093	63948	DMRTB1	1	53654604	2.16E-01	10272	1.80E-02	Up	2.37	4984	0.67	0.17
rs6730110	51281	ANKMY1	2	241173116	2.16E-01	10273	9.59E-01	Down	0.05	13618	0.67	0.00
rs1010616	158866	ZDHHC15		74416716	2.16E-01	10274	9.31E-01	Down	0.09	13436	0.67	0.00
rs237777	5867	RAB4A	1	225738651	2.16E-01	10275	2.52E-04	Down	3.66	2713	0.67	0.36
rs4075202	7015	TERT	5	1296475	2.16E-01	10276	7.23E-01	Up	0.35	12218	0.67	0.01
rs10800309	2212	FCGR2A	1	158285213	2.16E-01	10277	4.44E-01	Up	0.77	10298	0.67	0.04
rs4653296	64769	C1orf149	1	37647866	2.16E-01	10278	2.32E-04	Down	3.68	2684	0.67	0.36
rs4335184	4814	NINJ1	9	92991571	2.16E-01	10279	3.38E-02	Down	2.12	5582	0.67	0.15
rs13140326	84640	USP38	4	144472018	2.16E-01	10280	5.65E-02	Up	1.91	6149	0.67	0.12
rs959692	5885	RAD21	8	117968392	2.16E-01	10281	4.54E-01	Down	0.75	10379	0.66	0.03
rs2569538	3949	LDLR	19	11099548	2.16E-01	10282	6.49E-02	Down	1.85	6342	0.66	0.12

rs7165405	9768	KIAA0101	15	62449953	2.16E-01	10283	1.51E-12	Up	7.08	569	0.66	1.18
rs9988739	5076	PAX2	10	102504499	2.17E-01	10284	8.61E-03	Up	2.63	4395	0.66	0.21
rs4938653	7070	THY1	11	118810821	2.17E-01	10285	6.07E-03	Up	2.74	4175	0.66	0.22
rs6808709	256356	GK5	3	143365157	2.17E-01	10286	8.19E-05	Up	3.94	2398	0.66	0.41
rs7890052	5303	PIN4		71194680	2.17E-01	10287	1.02E-03	Up	3.28	3231	0.66	0.30
rs9975103	114038	C21orf84	21	43699988	2.17E-01	10288	1.44E-01	Down	1.46	7613	0.66	0.08
rs2276366	220136	CCDC11	18	46065303	2.17E-01	10289	5.33E-01	Up	0.62	10960	0.66	0.03
rs10400305	84883	AIFM2	11	56905307	2.17E-01	10290	7.12E-04	Down	3.39	3084	0.66	0.31
rs10400305	10394	PRG3	11	56905307	2.17E-01	10291	1.69E-02	Down	2.39	4933	0.66	0.18
rs10422818	4854	NOTCH3	19	15146272	2.17E-01	10292	3.51E-03	Up	2.92	3862	0.66	0.25
rs2472640	27164	SALL3	18	74852046	2.17E-01	10293	7.87E-03	Down	2.66	4331	0.66	0.21
rs6895902	4056	LTC4S	5	179134453	2.17E-01	10294	8.89E-01	Up	0.14	13182	0.66	0.01
rs1938012	139599	MAGEE2		74782970	2.17E-01	10295	1.11E-01	Down	1.59	7163	0.66	0.10
rs2718107	6608	SMO	7	128432610	2.17E-01	10296	4.20E-01	Up	0.81	10124	0.66	0.04
rs615474	138724	C9orf131	9	35033291	2.17E-01	10297	2.49E-01	Down	1.15	8757	0.66	0.06
rs6641322	3423	IDS		148281911	2.17E-01	10298	1.41E-02	Down	2.45	4761	0.66	0.18
rs2838950	6573	SLC19A1	21	45750725	2.17E-01	10299	5.60E-11	Up	6.54	726	0.66	1.03
rs3687	3021	H3F3B	17	71284185	2.17E-01	10300	2.47E-09	Down	5.96	946	0.66	0.86
rs3213562	7547	ZIC3		136363671	2.18E-01	10301	1.76E-02	Up	2.37	4965	0.66	0.18
rs17453055	223082	ZNRF2	7	30092458	2.18E-01	10302	1.06E-14	Up	7.72	413	0.66	1.40
rs10494126	128338	TMEM77	1	111383749	2.18E-01	10303	5.74E-01	Down	0.56	11270	0.66	0.02
rs6088578	58476	TP53INP2	20	32745265	2.18E-01	10304	2.18E-04	Down	3.70	2663	0.66	0.37
rs17541818	506	ATP5B	12	55344876	2.18E-01	10305	2.38E-01	Up	1.18	8638	0.66	0.06
rs17541818	10728	PTGES3	12	55344876	2.18E-01	10306	8.59E-01	Down	0.18	13006	0.66	0.01
rs2972574	6234	RPS28	19	8300092	2.18E-01	10307	2.95E-01	Up	1.05	9150	0.66	0.05
rs4675971	3069	HDLBP	2	241917163	2.18E-01	10308	1.74E-03	Up	3.13	3457	0.66	0.28
rs4861313	79730	NSUN7	4	40645465	2.18E-01	10309	6.96E-05	Up	3.98	2361	0.66	0.42
rs2584624	11325	DDX42	17	59258278	2.18E-01	10310	4.98E-02	Down	1.96	5992	0.66	0.13
rs13122139	55161	TMEM33	4	41772709	2.18E-01	10311	9.52E-04	Up	3.30	3201	0.66	0.30
rs2071214	332	BIRC5	17	73731186	2.18E-01	10312	1.94E-01	Up	1.30	8196	0.66	0.07
rs2439451	29015	SLC43A3	11	56969156	2.18E-01	10313	6.22E-05	Down	4.00	2337	0.66	0.42
rs555394	84174	SLA2	20	34670739	2.18E-01	10314	5.25E-01	Up	0.64	10905	0.66	0.03
rs11230985	4246	SCGB2A1	11	61738801	2.18E-01	10315	2.93E-03	Down	2.98	3763	0.66	0.25
rs2306270	10514	MYBBP1A	17	4404329	2.18E-01	10316	1.84E-01	Up	1.33	8064	0.66	0.07
rs9568353	57213	C13orf1	13	49379714	2.18E-01	10317	1.30E-05	Down	4.36	2013	0.66	0.49
rs165935	5663	PSEN1	14	72756897	2.18E-01	10318	4.67E-01	Down	0.73	10480	0.66	0.03
rs7207883	5718	PSMD12	17	62792756	2.18E-01	10319	3.02E-02	Down	2.17	5472	0.66	0.15
rs835036	23318	ZCCHC11	1	52709261	2.18E-01	10320	6.23E-01	Up	0.49	11606	0.66	0.02
rs803732	81571	C9orf45	9	122961095	2.18E-01	10321	4.12E-02	Down	2.04	5778	0.66	0.14
rs11466657	81793	TLR10	4	38598360	2.18E-01	10322	3.23E-01	Down	0.99	9385	0.66	0.05
rs6452722	26469	PTPN18	5	70798815	2.18E-01	10323	4.17E-03	Up	2.87	3961	0.66	0.24
rs6452722	55814	BDP1	5	70798815	2.18E-01	10324	3.63E-01	Up	0.91	9709	0.66	0.04
rs2297679	1307	COL16A1	1	31826102	2.18E-01	10325	1.39E-02	Down	2.46	4751	0.66	0.19
rs2483489	91452	ACBD5	10	27566537	2.18E-01	10326	4.26E-02	Up	2.03	5818	0.66	0.14
rs4879913	7016	TESK1	9	35600912	2.18E-01	10327	4.00E-01	Down	0.84	9986	0.66	0.04
rs4674520	1195	CLK1	2	201538960	2.18E-01	10328	1.58E-02	Up	2.41	4866	0.66	0.18
rs7586596	2673	GFPT1	2	69530226	2.19E-01	10329	5.68E-05	Up	4.03	2319	0.66	0.42
rs860579	4214	MAP3K1	5	56222832	2.19E-01	10330	2.33E-03	Down	3.04	3639	0.66	0.26
rs860579	133383	C5orf35	5	56222832	2.19E-01	10331	4.82E-01	Down	0.70	10585	0.66	0.03
rs4328010	92002	FAM58A		152398844	2.19E-01	10332	6.27E-01	Up	0.49	11632	0.66	0.02
rs12606223	79863	C18orf22	18	75914383	2.19E-01	10333	1.46E-02	Up	2.44	4793	0.66	0.18
rs6528754	1038	CDR1		139581495	2.19E-01	10334	8.50E-01	Up	0.19	12952	0.66	0.01
rs12445000	10229	COQ7	16	19002875	2.19E-01	10335	1.26E-02	Up	2.50	4663	0.66	0.19
rs1944053	9404	LPXN	11	58088444	2.19E-01	10336	7.22E-01	Down	0.36	12214	0.66	0.01
rs6656611	128272	ARHGEF19	1	16261111	2.19E-01	10337	6.87E-02	Down	1.82	6404	0.66	0.12
rs10053942	4015	LOX	5	121430496	2.19E-01	10338	1.13E-02	Up	2.53	4596	0.66	0.19
rs2301647	535	ATP6V0A1	17	37864814	2.19E-01	10339	6.10E-01	Up	0.51	11504	0.66	0.02
rs12944999	4619	MYH1	17	10364658	2.19E-01	10340	1.68E-01	Up	1.38	7902	0.66	0.08
rs12944999	4620	MYH2	17	10364658	2.19E-01	10341	5.24E-01	Down	0.64	10896	0.66	0.03
rs11155687	5110	PCMT1	6	150206859	2.19E-01	10342	7.36E-02	Down	1.79	6496	0.66	0.11
rs2595453	283297	OR10A4	11	6855071	2.19E-01	10343	8.87E-01	Down	0.14	13175	0.66	0.01
rs7209228	147184	TMEM99	17	36209517	2.19E-01	10344	9.85E-01	Up	0.02	13814	0.66	0.00
rs1468924	27094	KCNMB3	3	180465671	2.20E-01	10345	3.27E-01	Up	0.98	9417	0.66	0.05
rs907606	7136	TNNI2	11	1813688	2.20E-01	10346	5.49E-02	Down	1.92	6110	0.66	0.13
rs907606	90019	SYT8	11	1813688	2.20E-01	10347	7.75E-01	Up	0.29	12530	0.66	0.01
rs12985247	4946	OAZ1	19	2225359	2.20E-01	10348	1.47E-04	Up	3.80	2543	0.66	0.38
rs12985247	126306	JSRP1	19	2225359	2.20E-01	10349	1.45E-02	Down	2.44	4787	0.66	0.18
rs3803892	342977	NANOS3	19	13861170	2.20E-01	10350	1.19E-01	Down	1.56	7275	0.66	0.09
rs758427	146802	SLC47A2	17	19561069	2.20E-01	10351	2.59E-01	Up	1.13	8849	0.66	0.06
rs1559195	84306	PDCD2L	19	39586660	2.20E-01	10352	1.88E-05	Up	4.28	2081	0.66	0.47
rs10865330	150684	COMMD1	2	62199594	2.20E-01	10353	2.11E-03	Down	3.07	3568	0.66	0.27
rs16835204		B3GALT2	1	189906830	2.20E-01	10354	1.53E-02	Down	2.43	4822	0.66	0.18
rs2192930	57683	ZDBF2	2	206946045	2.20E-01	10355	5.01E-01	Up	0.67	10725	0.66	0.03
rs16912145	7321	UBE2D1	10	59752674	2.20E-01	10356	5.96E-01	Up	0.53	11407	0.66	0.02
rs4074940	83452	RAB33B	4	140753188	2.20E-01	10357	7.28E-02	Up	1.79	6482	0.66	0.11
rs2152373	9766	KIAA0247	14	69216746	2.20E-01	10358	9.56E-05	Up	3.90	2440	0.66	0.40
rs1175549	57470	LRRC47	1	3714884	2.20E-01	10359	7.99E-01	Up	0.25	12672	0.66	0.01
rs2303680	684	BST2	19	17392450	2.20E-01	10360	3.82E-05	Down	4.12	2218	0.66	0.44
rs623585	2352	FOLR3	11	71529215	2.20E-01	10361	3.65E-02	Up	2.09	5666	0.66	0.14
rs2297880	27240	SIT1	9	35640458	2.20E-01	10362	1.77E-01	Down	1.35	7980	0.66	0.08
rs2297880	84904	C9orf100	9	35640458	2.20E-01	10363	3.57E-01	Down	0.92	9650	0.66	0.04

rs17215777	54960	GEMIN8		13805585	2.20E-01	10364	9.63E-04	Up	3.30	3206	0.66	0.30
rs11622925	6554	SLC10A1	14	69331418	2.20E-01	10365	8.25E-02	Up	1.74	6676	0.66	0.11
rs9389574	57221	KIAA1244	6	138611694	2.20E-01	10366	6.73E-03	Up	2.71	4237	0.66	0.22
rs10077713	91977	MYOZ3	5	150028807	2.21E-01	10367	3.79E-01	Down	0.88	9829	0.66	0.04
rs729405	6613	SUMO2	17	70697252	2.21E-01	10368	5.13E-01	Up	0.65	10797	0.66	0.03
rs2334108	23542	MAPK8IP2	22	49346044	2.21E-01	10369	7.45E-04	Up	3.37	3105	0.66	0.31
rs2521655	4935	GPR143		9551779	2.21E-01	10370	5.00E-01	Up	0.68	10713	0.66	0.03
rs6586	51531	C9orf156	9	97746486	2.21E-01	10371	2.31E-01	Up	1.20	8571	0.66	0.06
rs6463900	7975	MAFK	7	1336642	2.21E-01	10372	1.40E-04	Up	3.81	2529	0.66	0.39
rs2306718	91300	C19orf22	19	850978	2.21E-01	10373	2.47E-01	Up	1.16	8731	0.66	0.06
rs17148885	58503	PROL1	4	71448870	2.21E-01	10374	8.14E-01	Up	0.23	12751	0.66	0.01
rs11869662	54475	NLE1	17	30506635	2.21E-01	10375	2.50E-07	Up	5.16	1397	0.65	0.66
rs9402956	59351	PBOV1	6	138573629	2.21E-01	10376	6.90E-01	Down	0.40	12008	0.65	0.02
rs7428403	59345	GNB4	3	180663639	2.21E-01	10377	2.57E-01	Up	1.13	8832	0.65	0.06
rs11745596	2107	ETF1	5	137862038	2.21E-01	10378	8.33E-06	Down	4.46	1912	0.65	0.51
rs5945968	84707	BEX2		102380709	2.21E-01	10379	1.72E-03	Down	3.13	3451	0.65	0.28
rs2044124	57003	CCDC47	17	59199157	2.22E-01	10380	6.25E-04	Up	3.42	3044	0.65	0.32
rs679563	3604	TNFRSF9	1	7905344	2.22E-01	10381	9.65E-01	Up	0.04	13653	0.65	0.00
rs2358538	51474	LIMA1	12	48872894	2.22E-01	10382	1.24E-01	Down	1.54	7355	0.65	0.09
rs3792849	11285	B4GALT7	5	176967392	2.22E-01	10383	1.41E-01	Up	1.47	7584	0.65	0.09
rs1125541	53836	GPR87	3	152510057	2.22E-01	10384	1.49E-03	Down	3.18	3381	0.65	0.28
rs13194781	8362	HIST1H4K	6	27923618	2.22E-01	10385	2.80E-05	Up	4.19	2156	0.65	0.46
rs13194781	8332	HIST1H2AL	6	27923618	2.22E-01	10386	5.18E-03	Up	2.80	4092	0.65	0.23
rs13194781	8330	HIST1H2AK	6	27923618	2.22E-01	10387	3.43E-02	Up	2.12	5602	0.65	0.15
rs13194781	8341	HIST1H2BN	6	27923618	2.22E-01	10388	8.40E-01	Down	0.20	12901	0.65	0.01
rs11809649	93190	C1orf158	1	12742857	2.22E-01	10389	7.88E-03	Down	2.66	4332	0.65	0.21
rs11699009	149954	C20orf186	20	31151902	2.22E-01	10390	4.00E-01	Down	0.84	9984	0.65	0.04
rs6087828	9777	TM9SF4	20	30208874	2.22E-01	10391	3.75E-05	Up	4.12	2215	0.65	0.44
rs12422330	80024	SLC24A6	12	112205599	2.22E-01	10392	1.45E-01	Up	1.46	7631	0.65	0.08
rs1801274	3310	HSPA6	1	158292800	2.22E-01	10393	1.55E-01	Up	1.42	7745	0.65	0.08
rs11865	23199	KIAA0182	16	84266817	2.22E-01	10394	4.73E-14	Up	7.56	445	0.65	1.33
rs2282456	54855	FAM46C	1	117881505	2.22E-01	10395	2.81E-04	Down	3.63	2756	0.65	0.36
rs11071986	91860	CALML4	15	66279082	2.22E-01	10396	1.48E-04	Up	3.79	2548	0.65	0.38
rs11071986	54982	CLN6	15	66279082	2.22E-01	10397	3.82E-01	Up	0.87	9861	0.65	0.04
rs2705338	56851	C15orf24	15	32157779	2.22E-01	10398	1.36E-02	Up	2.47	4731	0.65	0.19
rs11714815	80325	ABTB1	3	128856284	2.22E-01	10399	2.51E-02	Down	2.24	5289	0.65	0.16
rs7953158	50614	GALNT9	12	131351796	2.22E-01	10400	4.14E-01	Down	0.82	10081	0.65	0.04
rs1557033	2681	GGTA1	9	121321570	2.23E-01	10401	3.98E-01	Down	0.84	9973	0.65	0.04
rs7723	222068	TMED4	7	44391033	2.23E-01	10402	5.60E-11	Up	6.55	724	0.65	1.03
rs7723	54606	DDX56	7	44391033	2.23E-01	10403	5.05E-05	Up	4.05	2290	0.65	0.43
rs10509698	54619	CCNJ	10	97782342	2.23E-01	10404	4.63E-05	Up	4.07	2262	0.65	0.43
rs2293966	883	CCBL1	9	128671386	2.23E-01	10405	1.25E-03	Down	3.23	3310	0.65	0.29
rs2293966	51490	C9orf114	9	128671386	2.23E-01	10406	6.40E-01	Up	0.47	11706	0.65	0.02
rs11061209	5901	RAN	12	129889868	2.23E-01	10407	4.37E-01	Up	0.78	10248	0.65	0.04
rs4674916	8452	CUL3	2	225191140	2.23E-01	10408	6.72E-01	Up	0.42	11897	0.65	0.02
rs2915558	83903	GSG2	17	3577867	2.23E-01	10409	1.78E-01	Down	1.35	7998	0.65	0.07
rs12742463	913	CD1E	1	155143315	2.23E-01	10410	8.06E-01	Up	0.25	12707	0.65	0.01
rs1047128	84914	ZNF587	19	63049358	2.23E-01	10411	1.26E-02	Up	2.49	4665	0.65	0.19
rs4246621	80856	KIAA1715	2	176687878	2.23E-01	10412	5.40E-02	Down	1.93	6087	0.65	0.13
rs4236051	11329	STK38	6	36577799	2.24E-01	10413	1.19E-03	Down	3.24	3287	0.65	0.29
rs7550306	85440	DOCK7	1	62709594	2.24E-01	10414	2.15E-02	Up	2.30	5142	0.65	0.17
rs2278935	51480	VCX2		7961924	2.24E-01	10415	5.08E-01	Down	0.66	10771	0.65	0.03
rs7627545	339829	CCDC39	3	181921164	2.24E-01	10416	4.04E-03	Up	2.88	3937	0.65	0.24
rs1156044	4729	NDUFV2	18	9092140	2.24E-01	10417	3.08E-06	Up	4.67	1745	0.65	0.55
rs2076519	7802	DNALI1	1	37702140	2.24E-01	10418	3.20E-01	Down	0.99	9357	0.65	0.05
rs2076519	79753	SNIP1	1	37702140	2.24E-01	10419	6.74E-01	Down	0.42	11911	0.65	0.02
rs1421144	80318	GKAP1	9	83608321	2.24E-01	10420	4.80E-01	Down	0.71	10572	0.65	0.03
rs4614573	1045	CDX2	13	27454252	2.24E-01	10421	3.11E-05	Down	4.16	2176	0.65	0.45
rs6699938	56956	LHX9	1	194624809	2.24E-01	10422	3.55E-01	Up	0.93	9638	0.65	0.04
rs12940450	51479	ANKFY1	17	4010483	2.24E-01	10423	4.42E-02	Down	2.01	5858	0.65	0.14
rs12940450	124936	CYB5D2	17	4010483	2.24E-01	10424	7.73E-01	Up	0.29	12514	0.65	0.01
rs9656754	51669	TMEM66	8	30038184	2.24E-01	10425	1.45E-01	Down	1.46	7625	0.65	0.08
rs707939	4439	MSH5	6	31834667	2.24E-01	10426	1.89E-12	Up	7.04	583	0.65	1.17
rs707939	80737	C6orf27	6	31834667	2.24E-01	10427	9.79E-01	Up	0.03	13766	0.65	0.00
rs2176870	80021	TMEM62	15	41223702	2.24E-01	10428	5.04E-01	Down	0.67	10742	0.65	0.03
rs6494069	9133	CCNB2	15	57173642	2.24E-01	10429	2.59E-04	Up	3.65	2721	0.65	0.36
rs11671779	57828	C19orf15	19	43525190	2.25E-01	10430	1.36E-01	Down	1.49	7525	0.65	0.09
rs11247945	257101	ZNF683	1	26398347	2.25E-01	10431	5.67E-01	Down	0.57	11222	0.65	0.02
rs10445262	55065	GPR172B	17	4883900	2.25E-01	10432	5.34E-01	Up	0.62	10971	0.65	0.03
rs11083959	3743	KCNA7	19	54265018	2.25E-01	10433	1.45E-01	Down	1.46	7622	0.65	0.08
rs2285346	5526	PPP2R5B	11	64440864	2.25E-01	10434	1.36E-01	Down	1.49	7528	0.65	0.09
rs2285346	23130	ATG2A	11	64440864	2.25E-01	10435	9.17E-01	Down	0.10	13361	0.65	0.00
rs6768998	51099	ABHD5	3	43693274	2.25E-01	10436	4.30E-02	Down	2.02	5825	0.65	0.14
rs1594758	51050	PI15	8	75888202	2.25E-01	10437	3.26E-01	Down	0.98	9415	0.65	0.05
rs3007671	6282	S100A11	1	148812420	2.25E-01	10438	7.42E-04	Down	3.37	3102	0.65	0.31
rs7955473	5544	PRB3	12	11322890	2.25E-01	10439	4.52E-01	Down	0.75	10359	0.65	0.03
rs16888892	259282	FAM44A	4	13319706	2.25E-01	10440	7.72E-01	Up	0.29	12510	0.65	0.01
rs16985278	57665	RDH14	2	18655700	2.26E-01	10441	3.40E-01	Up	0.95	9522	0.65	0.05
rs896253	1327	COX4I1	16	84377703	2.26E-01	10442	2.23E-04	Down	3.69	2668	0.65	0.37
rs1981664	9167	COX7A2L	2	42488821	2.26E-01	10443	1.23E-05	Up	4.37	1997	0.65	0.49
rs4403552	412	STS		7103040	2.26E-01	10444	3.25E-01	Down	0.98	9408	0.65	0.05

rs10177996	7475	WNT6	2	219572066	2.26E-01	10445	6.32E-05	Down	4.00	2341	0.65	0.42
rs10177996	80326	WNT10A	2	219572066	2.26E-01	10446	4.34E-02	Down	2.02	5839	0.65	0.14
rs529553	163933	FAM43B	1	20630139	2.26E-01	10447	2.44E-02	Up	2.25	5265	0.65	0.16
rs1879145	8526	DGKE	17	52257054	2.26E-01	10448	3.01E-01	Down	1.04	9196	0.65	0.05
rs7202243	6340	SCNN1G	16	23094518	2.26E-01	10449	2.33E-03	Down	3.04	3640	0.65	0.26
rs11614913	3225	HOXC9	12	52671866	2.26E-01	10450	4.87E-02	Down	1.97	5963	0.65	0.13
rs11614913	3226	HOXC10	12	52671866	2.26E-01	10451	7.74E-02	Up	1.77	6585	0.65	0.11
rs11614913	3224	HOXC8	12	52671866	2.26E-01	10452	7.88E-01	Down	0.27	12599	0.65	0.01
rs581468	83935	TMEM133	11	100357806	2.26E-01	10453	1.93E-04	Down	3.73	2622	0.65	0.37
rs3830068	83882	TSPAN10	17	77233304	2.26E-01	10454	9.00E-01	Down	0.13	13244	0.65	0.00
rs16976620	145645	C15orf43	15	43037184	2.26E-01	10455	1.70E-02	Up	2.39	4938	0.65	0.18
rs4656539	5451	POU2F1	1	164089219	2.26E-01	10456	2.89E-01	Up	1.06	9107	0.65	0.05
rs2067477	1128	CHRM1	11	62434882	2.26E-01	10457	1.59E-01	Down	1.41	7795	0.65	0.08
rs1195962	823	CAPN1	11	64697995	2.26E-01	10458	3.08E-03	Down	2.96	3789	0.65	0.25
rs8045738	1339	COX6A2	16	31357499	2.27E-01	10459	6.43E-01	Down	0.46	11727	0.64	0.02
rs2073362	3588	IL10RB	21	33542671	2.27E-01	10460	2.32E-02	Up	2.27	5210	0.64	0.16
rs7255589	27106	ARRDC2	19	17984965	2.27E-01	10461	9.73E-03	Down	2.59	4490	0.64	0.20
rs476209	143689	PIWIL4	11	93972379	2.27E-01	10462	3.51E-01	Down	0.93	9598	0.64	0.05
rs11722533	55728	N4BP2	4	39976799	2.27E-01	10463	2.37E-02	Up	2.26	5230	0.64	0.16
rs12733930	84791	C1orf97	1	207991421	2.27E-01	10464	1.04E-01	Up	1.63	7058	0.64	0.10
rs7379110	114899	C1QTNF3	5	34089378	2.27E-01	10465	4.48E-01	Up	0.76	10336	0.64	0.03
rs10886515	7073	TIAL1	10	121333579	2.27E-01	10466	9.97E-01	Down	0.00	13888	0.64	0.00
rs12323805	6036	RNASE2	14	20494558	2.27E-01	10467	1.41E-01	Down	1.47	7590	0.64	0.08
rs967582	1996	ELAVL4	1	50294192	2.27E-01	10468	2.63E-04	Down	3.65	2731	0.64	0.36
rs2844665	285834	HCG22	6	31114834	2.27E-01	10469	9.92E-01	Up	0.01	13861	0.64	0.00
rs4674194	151246	SGOL2	2	201270658	2.27E-01	10470	8.18E-01	Up	0.23	12779	0.64	0.01
rs16847433	8403	SOX14	3	138964040	2.27E-01	10471	2.98E-02	Up	2.17	5453	0.64	0.15
rs2373929	4846	NOS3	7	150152460	2.27E-01	10472	2.84E-01	Down	1.07	9070	0.64	0.05
rs9852952	22906	TRAK1	3	42159365	2.27E-01	10473	2.52E-01	Down	1.15	8781	0.64	0.06
rs1115935	6847	SYCP1	1	115172111	2.27E-01	10474	2.93E-02	Up	2.18	5436	0.64	0.15
rs1480618	6005	RHAG	6	49685925	2.27E-01	10475	3.32E-01	Up	0.97	9461	0.64	0.05
rs11264329	1942	EFNA1	1	151908231	2.27E-01	10476	2.70E-01	Up	1.10	8949	0.64	0.06
rs313624	5589	PRKCSH	19	11413910	2.27E-01	10477	7.00E-08	Up	5.39	1257	0.64	0.72
rs313624	1995	ELAVL3	19	11413910	2.27E-01	10478	5.42E-01	Down	0.61	11037	0.64	0.03
rs2230018	7403	UTX		44685331	2.27E-01	10479	1.10E-03	Up	3.26	3258	0.64	0.30
rs9309717	55256	ADI1	2	5033361	2.27E-01	10480	7.47E-01	Up	0.32	12354	0.64	0.01
rs9303601	79089	TMUB2	17	39634367	2.27E-01	10481	1.85E-05	Down	4.28	2078	0.64	0.47
rs9303601	56970	ATXN7L3	17	39634367	2.27E-01	10482	2.19E-01	Down	1.23	8445	0.64	0.07
rs9303601	7343	UBTF	17	39634367	2.27E-01	10483	4.53E-01	Down	0.75	10364	0.64	0.03
rs8072896	59342	SCPEP1	17	52437831	2.28E-01	10484	8.62E-06	Down	4.45	1921	0.64	0.51
rs2239963	65109	UPF3B		118750957	2.28E-01	10485	5.88E-01	Down	0.54	11356	0.64	0.02
rs12900413	145873	MESP2	15	88122043	2.28E-01	10486	5.25E-01	Down	0.64	10908	0.64	0.03
rs13248008	84933	C8orf76	8	124323709	2.28E-01	10487	1.89E-03	Up	3.11	3505	0.64	0.27
rs13248008	11244	ZHX1	8	124323709	2.28E-01	10488	3.19E-01	Down	1.00	9354	0.64	0.05
rs2302210	64151	NCAPG	4	17514805	2.28E-01	10489	3.14E-04	Up	3.60	2794	0.64	0.35
rs6626288	158521	FMR1NB		146765673	2.28E-01	10490	4.69E-02	Down	1.99	5920	0.64	0.13
rs2812377	6366	CCL21	9	34700450	2.28E-01	10491	6.07E-02	Down	1.88	6248	0.64	0.12
rs2812377	6363	CCL19	9	34700450	2.28E-01	10492	4.60E-01	Down	0.74	10424	0.64	0.03
rs11242417	2676	GFRA3	5	137627233	2.28E-01	10493	2.71E-05	Down	4.20	2149	0.64	0.46
rs2832917	337882	KRTAP19-1	21	30794190	2.28E-01	10494	1.13E-02	Down	2.53	4593	0.64	0.19
rs2832917	337970	KRTAP19-3	21	30794190	2.28E-01	10495	8.74E-01	Down	0.16	13086	0.64	0.01
rs6672638	5132	PDC	1	183149141	2.28E-01	10496	4.26E-02	Up	2.03	5819	0.64	0.14
rs17544734	1080	CFTR	7	116706073	2.28E-01	10497	5.23E-01	Up	0.64	10889	0.64	0.03
rs12810816	4637	MYL6	12	54839227	2.28E-01	10498	1.26E-14	Down	7.71	418	0.64	1.39
rs12810816	140465	MYL6B	12	54839227	2.28E-01	10499	8.81E-07	Down	4.92	1562	0.64	0.61
rs12810816	6601	SMARCC2	12	54839227	2.28E-01	10500	7.37E-01	Up	0.34	12297	0.64	0.01
rs1051774	786	CACNG1	17	62459514	2.28E-01	10501	4.79E-01	Down	0.71	10569	0.64	0.03
rs1636874	6469	SHH	7	155085576	2.28E-01	10502	7.37E-02	Down	1.79	6497	0.64	0.11
rs1044773	23309	SIN3B	19	16851687	2.28E-01	10503	6.56E-01	Down	0.45	11809	0.64	0.02
rs953280	133482	SLCO6A1	5	101871658	2.28E-01	10504	9.66E-01	Down	0.04	13659	0.64	0.00
rs2293079	79411	GLB1L	2	219926292	2.28E-01	10505	4.17E-02	Down	2.04	5793	0.64	0.14
rs2293079	8576	STK16	2	219926292	2.28E-01	10506	1.54E-01	Up	1.42	7738	0.64	0.08
rs7644750	8723	SNX4	3	126737800	2.28E-01	10507	7.36E-06	Up	4.48	1881	0.64	0.51
rs7644750	114885	OSBPL11	3	126737800	2.28E-01	10508	9.36E-03	Down	2.60	4460	0.64	0.20
rs2345289	23580	CDC42EP4	17	68790130	2.28E-01	10509	4.72E-34	Down	12.17	44	0.64	3.33
rs6998248	55174	INTS10	8	19756844	2.29E-01	10510	1.61E-01	Up	1.40	7830	0.64	0.08
rs11205415	80851	SH3BP5L	1	245330435	2.29E-01	10511	3.04E-01	Up	1.03	9227	0.64	0.05
rs11205415	79894	ZNF672	1	245330435	2.29E-01	10512	7.50E-01	Down	0.32	12374	0.64	0.01
rs1858706	51201	ZDHHC2	8	17039941	2.29E-01	10513	4.62E-03	Down	2.83	4013	0.64	0.23
rs4299259	11202	KLK8	19	56183411	2.29E-01	10514	5.24E-01	Down	0.64	10898	0.64	0.03
rs7156468	81537	SGPP1	14	63238590	2.29E-01	10515	7.03E-01	Up	0.38	12090	0.64	0.02
rs3751633	3175	ONECUT1	15	50829179	2.29E-01	10516	2.35E-01	Up	1.19	8611	0.64	0.06
rs4758929	7574	ZNF26	12	132188413	2.29E-01	10517	1.57E-06	Up	4.80	1637	0.64	0.58
rs479844	5017	OVOL1	11	65308533	2.29E-01	10518	3.02E-01	Down	1.03	9203	0.64	0.05
rs1015004	2551	GABPA	21	26050964	2.29E-01	10519	2.98E-02	Up	2.17	5452	0.64	0.15
rs2008018	1443	CSH2	17	59308203	2.29E-01	10520	1.82E-01	Down	1.33	8052	0.64	0.07
rs2008018	2689	GH2	17	59308203	2.29E-01	10521	7.44E-01	Down	0.33	12338	0.64	0.01
rs7503353	54785	C17orf59	17	8048704	2.29E-01	10522	1.33E-02	Down	2.48	4707	0.64	0.19
rs2462907	3198	HOXA1	7	26900471	2.29E-01	10523	6.90E-01	Up	0.40	12012	0.64	0.02
rs12127781	10542	HBXIP	1	110648599	2.29E-01	10524	2.26E-03	Up	3.05	3617	0.64	0.26
rs12127781	9122	SLC16A4	1	110648599	2.29E-01	10525	2.84E-01	Down	1.07	9066	0.64	0.05

rs3744477	80174	DBF4B	17	40183199	2.29E-01	10526	6.48E-01	Down	0.46	11766	0.64	0.02
rs1770345	2475	FRAP1	1	11148846	2.29E-01	10527	2.58E-02	Up	2.23	5314	0.64	0.16
rs6710432	150737	TTC30B	2	178253927	2.29E-01	10528	5.42E-01	Up	0.61	11029	0.64	0.03
rs10774442	196500	C12orf53	12	6699733	2.29E-01	10529	9.28E-02	Down	1.68	6881	0.64	0.10
rs6459662	6991	TCTE3	6	169983922	2.29E-01	10530	9.94E-01	Up	0.01	13871	0.64	0.00
rs6947660	57002	C7orf36	7	39383417	2.29E-01	10531	1.08E-03	Up	3.27	3252	0.64	0.30
rs4876948	79813	EHMT1	9	137970168	2.29E-01	10532	2.82E-02	Up	2.19	5401	0.64	0.15
rs3756954	51522	TMEM14C	6	10832546	2.29E-01	10533	9.63E-01	Down	0.05	13637	0.64	0.00
rs4901065	5836	PYGL	14	50440871	2.29E-01	10534	2.20E-15	Down	7.93	374	0.64	1.47
rs647397	643646	HSD17BP1	17	37932924	2.29E-01	10535	1.09E-01	Up	1.60	7133	0.64	0.10
rs2525070	4281	MID1		10352363	2.29E-01	10536	2.71E-07	Down	5.14	1404	0.64	0.66
rs4346095	79612	NARG1L	13	40810340	2.30E-01	10537	8.98E-01	Down	0.13	13232	0.64	0.00
rs1010222	5886	RAD23A	19	12909608	2.30E-01	10538	5.72E-01	Up	0.56	11260	0.64	0.02
rs1848031	89778	SERPINB11	18	59543253	2.30E-01	10539	1.54E-05	Down	4.32	2042	0.64	0.48
rs923118	64220	STRA6	15	72253324	2.30E-01	10540	7.75E-02	Down	1.77	6588	0.64	0.11
rs923118	3671	ISLR	15	72253324	2.30E-01	10541	2.21E-01	Down	1.22	8468	0.64	0.07
rs5916793	11043	MID2		106953693	2.30E-01	10542	3.54E-01	Down	0.93	9620	0.64	0.05
rs982511	160365	CLECL1	12	9773595	2.30E-01	10543	7.41E-01	Down	0.33	12321	0.64	0.01
rs10518140	25898	RCHY1	4	76774963	2.30E-01	10544	1.38E-07	Up	5.27	1334	0.64	0.69
rs1946482	124044	SPATA2L	16	88289911	2.30E-01	10545	8.47E-02	Up	1.72	6715	0.64	0.11
rs4788073	6799	SULT1A2	16	28502050	2.30E-01	10546	7.08E-02	Down	1.81	6446	0.64	0.12
rs17122920	121274	ZNF641	12	47041909	2.30E-01	10547	4.29E-01	Up	0.79	10186	0.64	0.04
rs5758837	267020	ATP5L2	22	41373512	2.30E-01	10548	1.50E-01	Down	1.44	7686	0.64	0.08
rs17597589	10798	OR5I1	11	55460342	2.30E-01	10549	3.01E-01	Down	1.03	9197	0.64	0.05
rs3885994	6698	SPRR1A	1	149750999	2.31E-01	10550	5.72E-02	Up	1.90	6161	0.64	0.12
rs3885994	163778	SPRR4	1	149750999	2.31E-01	10551	3.23E-01	Up	0.99	9383	0.64	0.05
rs353268	78991	PCYOX1L	5	148737396	2.31E-01	10552	7.28E-04	Up	3.38	3091	0.64	0.31
rs353268	27190	IL17B	5	148737396	2.31E-01	10553	7.07E-02	Down	1.81	6445	0.64	0.12
rs6764110	25871	C3orf17	3	114214095	2.31E-01	10554	3.50E-02	Up	2.11	5624	0.64	0.15
rs11656955	9043	SPAG9	17	46403063	2.31E-01	10555	2.45E-02	Down	2.25	5270	0.64	0.16
rs12930313	23162	MAPK8IP3	16	1705233	2.31E-01	10556	4.93E-01	Up	0.68	10669	0.64	0.03
rs10860831	51019	CCDC53	12	100912014	2.31E-01	10557	7.54E-01	Down	0.31	12405	0.64	0.01
rs11185546	348793	WDR53	3	197791848	2.31E-01	10558	3.34E-01	Down	0.97	9475	0.64	0.05
rs4432111	2322	FLT3	13	27488258	2.31E-01	10559	6.83E-01	Up	0.41	11968	0.64	0.02
rs1056995	5452	POU2F2	19	47283324	2.31E-01	10560	2.27E-01	Down	1.21	8529	0.64	0.06
rs1056995	64763	ZNF574	19	47283324	2.31E-01	10561	7.84E-01	Down	0.27	12575	0.64	0.01
rs9911615	3859	KRT12	17	36277563	2.32E-01	10562	3.31E-01	Up	0.97	9452	0.64	0.05
rs9911615	54474	KRT20	17	36277563	2.32E-01	10563	6.38E-01	Up	0.47	11685	0.64	0.02
rs2241748	10124	ARL4A	7	12502217	2.32E-01	10564	9.66E-01	Down	0.04	13669	0.64	0.00
rs10519697	133923	ZNF474	5	121524993	2.32E-01	10565	3.04E-01	Up	1.03	9222	0.64	0.05
rs2712381	6184	RPN1	3	129821298	2.32E-01	10566	3.95E-05	Up	4.11	2228	0.64	0.44
rs10887466	80195	C10orf57	10	81818390	2.32E-01	10567	2.14E-01	Down	1.24	8399	0.64	0.07
rs2782650	23334	KIAA0467	1	43581683	2.32E-01	10568	4.11E-01	Up	0.82	10061	0.64	0.04
rs1476607	5213	PFKM	12	46811071	2.32E-01	10569	1.18E-03	Down	3.24	3283	0.64	0.29
rs10405576	2828	GPR4	19	50790725	2.32E-01	10570	1.51E-01	Down	1.44	7692	0.64	0.08
rs7544301	9804	TOMM20	1	231589203	2.32E-01	10571	1.35E-02	Up	2.47	4723	0.63	0.19
rs17592080	8790	FPGT	1	74386715	2.32E-01	10572	5.49E-03	Up	2.78	4124	0.63	0.23
rs11077405	3959	LGALS3BP	17	74481477	2.32E-01	10573	1.16E-33	Down	12.09	46	0.63	3.29
rs11574452	5473	PPBP	4	75211696	2.32E-01	10574	9.67E-02	Up	1.66	6938	0.63	0.10
rs11574452	5196	PF4	4	75211696	2.32E-01	10575	3.52E-01	Up	0.93	9613	0.63	0.05
rs1442849	84667	HES7	17	7964846	2.32E-01	10576	1.83E-01	Down	1.33	8054	0.63	0.07
rs2638031	55032	SLC35A5	3	113770450	2.33E-01	10577	1.75E-01	Up	1.36	7960	0.63	0.08
rs2305107	1938	EEF2	19	3934302	2.33E-01	10578	9.88E-22	Up	9.59	165	0.63	2.10
rs2305107	1613	DAPK3	19	3934302	2.33E-01	10579	7.57E-04	Down	3.37	3112	0.63	0.31
rs2143943	128488	WFDC12	20	43199481	2.33E-01	10580	9.71E-02	Up	1.66	6944	0.63	0.10
rs349030	284348	LYPD5	19	49023650	2.33E-01	10581	7.97E-01	Down	0.26	12656	0.63	0.01
rs349030	284349	ZNF283	19	49023650	2.33E-01	10582	8.78E-01	Up	0.15	13110	0.63	0.01
rs6913660	8969	HIST1H2AG	6	27199404	2.33E-01	10583	3.58E-01	Up	0.92	9660	0.63	0.04
rs6913660	8294	HIST1H4I	6	27199404	2.33E-01	10584	6.19E-01	Up	0.50	11568	0.63	0.02
rs6913660	8970	HIST1H2BJ	6	27199404	2.33E-01	10585	6.24E-01	Down	0.49	11610	0.63	0.02
rs2612092	7298	TYMS	18	672399	2.33E-01	10586	9.37E-05	Up	3.91	2434	0.63	0.40
rs2612092	55556	ENOSF1	18	672399	2.33E-01	10587	4.63E-04	Up	3.50	2930	0.63	0.33
rs7294	10295	BCKDK	16	31009822	2.33E-01	10588	4.77E-03	Up	2.82	4028	0.63	0.23
rs7294	79001	VKORC1	16	31009822	2.33E-01	10589	6.94E-01	Up	0.39	12031	0.63	0.02
rs7607428	130888	FBXO36	2	230621742	2.33E-01	10590	4.39E-01	Down	0.77	10264	0.63	0.04
rs7066605	2824	GPM6B		13609352	2.33E-01	10591	1.94E-22	Down	9.74	152	0.63	2.17
rs4509550	54546	RNF186	1	19903547	2.33E-01	10592	3.77E-03	Down	2.90	3897	0.63	0.24
rs3803932	7769	ZNF226	19	49362089	2.33E-01	10593	1.59E-02	Up	2.41	4874	0.63	0.18
rs4253755	5465	PPARA	22	44935895	2.33E-01	10594	3.24E-04	Down	3.60	2802	0.63	0.35
rs235711	650	BMP2	20	6688089	2.33E-01	10595	1.58E-03	Down	3.16	3412	0.63	0.28
rs3822030	53834	FGFRL1	4	977173	2.33E-01	10596	4.51E-11	Up	6.57	712	0.63	1.03
rs7925108	246330	PELI3	11	65990599	2.33E-01	10597	4.26E-01	Down	0.80	10163	0.63	0.04
rs11248212	23246	BOP1	8	145465106	2.33E-01	10598	1.66E-06	Up	4.79	1644	0.63	0.58
rs3749164	57140	RNPEPL1	2	241245681	2.34E-01	10599	4.48E-01	Down	0.76	10333	0.63	0.03
rs17024661	2949	GSTM5	1	109970406	2.34E-01	10600	1.00E-24	Down	10.27	117	0.63	2.40
rs251742	7023	TFAP4	16	4256169	2.34E-01	10601	8.92E-01	Down	0.14	13197	0.63	0.00
rs3806698		B4GALT4	3	120438578	2.34E-01	10602	3.64E-02	Down	2.09	5661	0.63	0.14
rs6699228	317705	VN1R5	1	243737095	2.34E-01	10603	9.89E-01	Up	0.01	13839	0.63	0.00
rs7973701	7334	UBE2N	12	92299126	2.34E-01	10604	4.45E-11	Up	6.58	709	0.63	1.04
rs7973701	11163	NUDT4	12	92299126	2.34E-01	10605	5.66E-03	Up	2.77	4149	0.63	0.22
rs3827102	7022	TFAP2C	20	54636391	2.34E-01	10606	4.70E-02	Up	1.99	5923	0.63	0.13

rs6561575	10301	DLEU1	13	49575612	2.34E-01	10607	7.60E-01	Up	0.31	12438	0.63	0.01
rs1266746	1288	COL4A6		107283885	2.34E-01	10608	8.24E-35	Down	12.31	42	0.63	3.41
rs3844608	255027	MPV17L	16	15420076	2.34E-01	10609	4.22E-02	Up	2.03	5803	0.63	0.14
rs2289600	10237	SLC35B1	17	45136676	2.34E-01	10610	4.95E-04	Up	3.48	2957	0.63	0.33
rs7035040	158401	C9orf84	9	111593888	2.34E-01	10611	1.46E-01	Down	1.45	7641	0.63	0.08
rs12561931	91687	CENPL	1	170506609	2.34E-01	10612	2.90E-14	Up	7.57	442	0.63	1.35
rs12561931	55157	DARS2	1	170506609	2.34E-01	10613	1.60E-02	Up	2.41	4883	0.63	0.18
rs2075589	8482	SEMA7A	15	72497028	2.34E-01	10614	2.97E-06	Up	4.67	1736	0.63	0.55
rs11787401	6781	STC1	8	23750369	2.34E-01	10615	9.03E-01	Up	0.12	13265	0.63	0.00
rs11070327	11339	OIP5	15	39390158	2.34E-01	10616	7.31E-02	Up	1.79	6486	0.63	0.11
rs3211663	1164	CKS2	9	89155642	2.34E-01	10617	6.23E-26	Up	10.53	101	0.63	2.52
rs3211663	79048	SECISBP2	9	89155642	2.34E-01	10618	8.14E-06	Up	4.46	1907	0.63	0.51
rs4889294	53630	BCMO1	16	79861260	2.35E-01	10619	5.01E-01	Up	0.67	10724	0.63	0.03
rs11670895	199699	DAND5	19	12942089	2.35E-01	10620	5.74E-01	Up	0.56	11269	0.63	0.02
rs11670895	90480	GADD45GIP1	19	12942089	2.35E-01	10621	8.68E-01	Up	0.17	13055	0.63	0.01
rs11919639	151888	BTLA	3	113691440	2.35E-01	10622	5.19E-01	Up	0.64	10856	0.63	0.03
rs2815170	158158	RASEF	9	82819469	2.35E-01	10623	5.55E-01	Up	0.59	11117	0.63	0.03
rs12779247	22852	ANKRD26	10	27330096	2.35E-01	10624	1.98E-02	Up	2.33	5063	0.63	0.17
rs1129101	6194	RPS6	9	19362990	2.35E-01	10625	4.58E-07	Up	5.04	1470	0.63	0.63
rs10409222	3171	FOXA3	19	51071487	2.35E-01	10626	3.44E-01	Down	0.95	9554	0.63	0.05
rs2425256	22839	DLGAP4	20	34489986	2.35E-01	10627	3.43E-01	Up	0.95	9549	0.63	0.05
rs4793036	7283	TUBG1	17	38011090	2.35E-01	10628	1.38E-01	Up	1.48	7541	0.63	0.09
rs10156978	265	AMELX		11055628	2.35E-01	10629	3.45E-02	Up	2.11	5607	0.63	0.15
rs10221448	85360	SYDE1	19	15070043	2.35E-01	10630	6.25E-01	Down	0.49	11615	0.63	0.02
rs10499761	51142	CHCHD2	7	55967852	2.35E-01	10631	8.91E-02	Up	1.70	6787	0.63	0.10
rs739673	29850	TRPM5	11	2377076	2.35E-01	10632	9.87E-02	Down	1.65	6978	0.63	0.10
rs739673	10078	TSSC4	11	2377076	2.35E-01	10633	7.92E-01	Down	0.26	12627	0.63	0.01
rs704374	80145	THOC7	3	63840987	2.35E-01	10634	9.49E-06	Up	4.43	1938	0.63	0.50
rs10808897	7336	UBE2V2	8	49137169	2.35E-01	10635	1.32E-03	Down	3.21	3333	0.63	0.29
rs1109303	4641	MYO1C	17	1350227	2.35E-01	10636	2.50E-02	Down	2.24	5286	0.63	0.16
rs4468605	4324	MMP15	16	56646525	2.36E-01	10637	9.85E-01	Up	0.02	13813	0.63	0.00
rs10044838	3575	IL7R	5	35900347	2.36E-01	10638	2.68E-01	Up	1.11	8936	0.63	0.06
rs3750111	10142	AKAP9	7	91373574	2.36E-01	10639	1.48E-05	Up	4.33	2034	0.63	0.48
rs10236856	5189	PEX1	7	91780337	2.36E-01	10640	7.21E-06	Up	4.49	1875	0.63	0.51
rs788016	3329	HSPD1	2	198177799	2.36E-01	10641	2.20E-13	Up	7.33	497	0.63	1.27
rs788016	3336	HSPE1	2	198177799	2.36E-01	10642	6.00E-08	Up	5.42	1241	0.63	0.72
rs427056	900	CCNG1	5	162782337	2.36E-01	10643	5.42E-01	Up	0.61	11033	0.63	0.03
rs997547	56910	STARD7	2	96280908	2.36E-01	10644	4.95E-01	Up	0.68	10683	0.63	0.03
rs2191377	85280	KRTAP9-4	17	36662596	2.36E-01	10645	1.59E-01	Up	1.41	7796	0.63	0.08
rs12679786	55806	HR	8	22063238	2.36E-01	10646	6.09E-01	Up	0.51	11498	0.63	0.02
rs234748	5316	PKNOX1	21	43265339	2.36E-01	10647	9.09E-01	Up	0.11	13300	0.63	0.00
rs7160073	376267	RAB15	14	64520383	2.36E-01	10648	1.58E-03	Up	3.16	3415	0.63	0.28
rs44637	9119	KRT75	12	51101732	2.36E-01	10649	4.59E-01	Down	0.74	10422	0.63	0.03
rs7059689	158800	RHOXF1		119037642	2.36E-01	10650	5.05E-01	Down	0.67	10752	0.63	0.03
rs3823995	64101	LRRC4	7	127260040	2.36E-01	10651	8.77E-01	Up	0.15	13100	0.63	0.01
rs454387	3853	KRT6A	12	51166638	2.36E-01	10652	2.71E-09	Down	5.95	959	0.63	0.86
rs454387	286887	KRT6C	12	51166638	2.36E-01	10653	9.09E-01	Down	0.11	13304	0.63	0.00
rs3775801	54726	OTUD4	4	146442032	2.36E-01	10654	2.27E-02	Down	2.28	5185	0.63	0.16
rs6005863	150274	HSCB	22	27481002	2.36E-01	10655	2.51E-03	Up	3.02	3687	0.63	0.26
rs2049909	57645	POGK	1	163563603	2.36E-01	10656	9.29E-02	Down	1.68	6884	0.63	0.10
rs2049909	117143	TADA1L	1	163563603	2.36E-01	10657	1.10E-01	Down	1.60	7147	0.63	0.10
rs591556	7166	TPH1	11	18017976	2.37E-01	10658	1.07E-01	Down	1.61	7115	0.63	0.10
rs12357826	27069	GHITM	10	85877034	2.37E-01	10659	3.09E-04	Down	3.61	2786	0.63	0.35
rs2073838	6583	SLC22A4	5	131677121	2.37E-01	10660	4.88E-02	Down	1.97	5966	0.63	0.13
rs8075459	254863	C17orf61	17	7251546	2.37E-01	10661	1.93E-03	Up	3.10	3515	0.63	0.27
rs8075459	57555	NLGN2	17	7251546	2.37E-01	10662	1.99E-02	Down	2.33	5067	0.63	0.17
rs8075459	57048	PLSCR3	17	7251546	2.37E-01	10663	2.52E-01	Up	1.15	8778	0.63	0.06
rs4796649	284119	PTRF	17	37804137	2.37E-01	10664	7.07E-54	Down	15.45	4	0.63	5.32
rs2453568	55244	SLC47A1	17	19398101	2.37E-01	10665	1.54E-06	Down	4.81	1633	0.63	0.58
rs2241592	51293	CD320	19	8292306	2.37E-01	10666	7.22E-01	Up	0.36	12211	0.63	0.01
rs17356664	90332	EXOC3L2	19	50432611	2.37E-01	10667	5.40E-01	Up	0.61	11011	0.62	0.03
rs610932	64231	MS4A6A	11	59695883	2.37E-01	10668	2.41E-04	Up	3.67	2695	0.62	0.36
rs1567522	869	CBLN1	16	47860327	2.37E-01	10669	1.36E-02	Up	2.47	4730	0.62	0.19
rs4919652	10121	ACTR1A	10	104264147	2.38E-01	10670	3.24E-07	Down	5.11	1425	0.62	0.65
rs184110	51654	CDK5RAP1	20	31445093	2.38E-01	10671	6.35E-01	Up	0.47	11674	0.62	0.02
rs16964783	9220	TIAF1	17	24439733	2.38E-01	10672	2.64E-01	Up	1.12	8901	0.62	0.06
rs4414954	80319	CXXC4	4	105789853	2.38E-01	10673	6.64E-01	Up	0.43	11847	0.62	0.02
rs1410054	8036	SHOC2	10	112764145	2.38E-01	10674	3.08E-04	Down	3.61	2784	0.62	0.35
rs2288481	8463	TEAD2	19	54570008	2.38E-01	10675	6.97E-03	Down	2.70	4259	0.62	0.22
rs2288481	27120	DKKL1	19	54570008	2.38E-01	10676	5.93E-01	Down	0.53	11393	0.62	0.02
rs749767	84148	MYST1	16	31031908	2.38E-01	10677	3.29E-07	Down	5.11	1430	0.62	0.65
rs11795627	56159	TEX11		69740462	2.38E-01	10678	4.36E-01	Down	0.78	10240	0.62	0.04
rs12695294	122830	NAT12	3	114919537	2.38E-01	10679	2.15E-01	Up	1.24	8412	0.62	0.07
rs768451	284756	C20orf197	20	58089229	2.38E-01	10680	4.19E-01	Down	0.81	10113	0.62	0.04
rs10050268	2266	FGG	4	155906959	2.38E-01	10681	1.19E-02	Up	2.52	4629	0.62	0.19
rs12941802	4222	MEOX1	17	39084753	2.38E-01	10682	1.70E-01	Up	1.37	7916	0.62	0.08
rs2303492	23647	ARFIP2	11	6459703	2.38E-01	10683	2.09E-08	Up	5.63	1112	0.62	0.77
rs2303492	26515	FXC1	11	6459703	2.38E-01	10684	1.48E-04	Down	3.80	2545	0.62	0.38
rs2303492	10612	TRIM3	11	6459703	2.38E-01	10685	2.09E-02	Up	2.31	5108	0.62	0.17
rs1093992	9400	RECQL5	17	71125952	2.38E-01	10686	2.61E-01	Down	1.12	8866	0.62	0.06
rs1093992	80022	MYO15B	17	71125952	2.38E-01	10687	6.82E-01	Down	0.41	11960	0.62	0.02

rs2982229	100129482	ZNF37B	10	42368847	2.38E-01	10688	9.86E-02	Up	1.65	6974	0.62	0.10
rs6090314	54915	YTHDF1	20	61327997	2.39E-01	10689	7.61E-02	Up	1.77	6551	0.62	0.11
rs6090314	79444	BIRC7	20	61327997	2.39E-01	10690	2.14E-01	Down	1.24	8397	0.62	0.07
rs743998	10110	SGK2	20	41618950	2.39E-01	10691	8.59E-01	Down	0.18	13008	0.62	0.01
rs6767498	54899	PXK	3	58384166	2.39E-01	10692	7.04E-02	Down	1.81	6440	0.62	0.12
rs6767498	5162	PDHB	3	58384166	2.39E-01	10693	2.15E-01	Up	1.24	8416	0.62	0.07
rs12645336	93621	MRFAP1	4	6751903	2.39E-01	10694	1.62E-01	Down	1.40	7842	0.62	0.08
rs12289836	5970	RELA	11	65193464	2.39E-01	10695	1.88E-01	Up	1.32	8124	0.62	0.07
rs12522503	6940	ZNF354A	5	178064020	2.39E-01	10696	2.58E-03	Up	3.01	3704	0.62	0.26
rs2841555	85411	C6orf114	6	13574809	2.39E-01	10697	6.50E-01	Up	0.45	11772	0.62	0.02
rs1050813	1174	AP1S1	7	100375050	2.39E-01	10698	1.63E-01	Up	1.40	7848	0.62	0.08
rs1050813	5054	SERPINE1	7	100375050	2.39E-01	10699	1.64E-01	Up	1.39	7862	0.62	0.08
rs11809802	51430	C1orf9	1	169298498	2.39E-01	10700	1.58E-01	Down	1.41	7793	0.62	0.08
rs9840852	23200	ATP11B	3	183980962	2.39E-01	10701	1.12E-20	Up	9.33	182	0.62	2.00
rs9988954	7316	UBC	12	123938302	2.39E-01	10702	1.88E-02	Up	2.35	5012	0.62	0.17
rs3922	283149	BCL9L	11	118270810	2.40E-01	10703	8.70E-01	Up	0.16	13062	0.62	0.01
rs6142508	2036	EPB41L1	20	34160719	2.40E-01	10704	6.44E-03	Down	2.72	4205	0.62	0.22
rs10737000	10276	NET1	10	5499387	2.40E-01	10705	1.24E-16	Up	8.27	325	0.62	1.59
rs17500235	84514	GHDC	17	37609886	2.40E-01	10706	7.56E-02	Up	1.78	6537	0.62	0.11
rs17500235	3060	HCRT	17	37609886	2.40E-01	10707	5.97E-01	Down	0.53	11412	0.62	0.02
rs7605442	659	BMPR2	2	203197895	2.40E-01	10708	5.80E-01	Down	0.55	11320	0.62	0.02
rs16994499	23767	FLRT3	20	14275758	2.40E-01	10709	1.32E-16	Down	8.27	329	0.62	1.59
rs877518	3399	ID3	1	23640460	2.40E-01	10710	1.58E-10	Down	6.40	783	0.62	0.98
rs6535915	84057	MND1	4	154636850	2.40E-01	10711	2.31E-01	Up	1.20	8575	0.62	0.06
rs10878177	29110	TBK1	12	63170028	2.40E-01	10712	3.88E-01	Down	0.86	9899	0.62	0.04
rs5945801	9643	MORF4L2		102757797	2.40E-01	10713	1.38E-02	Down	2.46	4747	0.62	0.19
rs34813	54826	GIN1	5	102461308	2.40E-01	10714	5.56E-02	Down	1.91	6129	0.62	0.13
rs11248424	9184	BUB3	10	124919753	2.40E-01	10715	4.79E-10	Up	6.23	852	0.62	0.93
rs7766404	222659	PXT1	6	36471797	2.40E-01	10716	7.66E-01	Down	0.30	12469	0.62	0.01
rs7253352	4207	MEF2B	19	19114197	2.40E-01	10717	1.12E-01	Up	1.59	7167	0.62	0.10
rs17476297	1350	COX7C	5	85945880	2.40E-01	10718	7.94E-01	Down	0.26	12634	0.62	0.01
rs1059702	3654	IRAK1		152805039	2.40E-01	10719	3.17E-10	Up	6.24	841	0.62	0.95
rs1059702	4204	MECP2		152805039	2.40E-01	10720	1.15E-04	Down	3.86	2488	0.62	0.39
rs4922579	5954	RCN1	11	32093012	2.41E-01	10721	5.16E-01	Up	0.65	10828	0.62	0.03
rs3760627	1209	CLPTM1	19	50149020	2.41E-01	10722	1.01E-04	Up	3.89	2456	0.62	0.40
rs3760627	344	APOC2	19	50149020	2.41E-01	10723	1.46E-03	Up	3.18	3365	0.62	0.28
rs3760627	346	APOC4	19	50149020	2.41E-01	10724	2.11E-02	Down	2.31	5124	0.62	0.17
rs7954360	9700	ESPL1	12	51931465	2.41E-01	10725	6.39E-01	Up	0.47	11703	0.62	0.02
rs7954360	84975	MFSD5	12	51931465	2.41E-01	10726	9.37E-01	Down	0.08	13475	0.62	0.00
rs718780		B4GALT6	18	27509259	2.41E-01	10727	2.28E-01	Down	1.21	8539	0.62	0.06
rs4808171	10147	SFRS14	19	18961115	2.41E-01	10728	1.90E-12	Up	7.03	584	0.62	1.17
rs1057463	80124	VCPIP1	8	67754706	2.41E-01	10729	1.24E-02	Up	2.50	4656	0.62	0.19
rs1057463	56260	C8orf44	8	67754706	2.41E-01	10730	1.86E-01	Down	1.32	8093	0.62	0.07
rs3823035	26235	FBXL4	6	99449239	2.41E-01	10731	1.04E-01	Up	1.62	7065	0.62	0.10
rs1321578	85236	HIST1H2BK	6	27212762	2.41E-01	10732	6.13E-11	Up	6.54	730	0.62	1.02
rs4886707	25942	SIN3A	15	73542520	2.41E-01	10733	5.21E-07	Down	5.02	1485	0.62	0.63
rs4886707	5780	PTPN9	15	73542520	2.41E-01	10734	3.10E-03	Down	2.96	3794	0.62	0.25
rs11991741	29883	CNOT7	8	17156004	2.41E-01	10735	3.14E-05	Down	4.16	2178	0.62	0.45
rs12491229	132112	RTP1	3	188383701	2.41E-01	10736	7.52E-01	Up	0.32	12385	0.62	0.01
rs10043779	84250	ANKRD32	5	93969574	2.41E-01	10737	2.41E-01	Up	1.17	8673	0.62	0.06
rs10043779	285600	C5orf36	5	93969574	2.41E-01	10738	8.77E-01	Down	0.15	13105	0.62	0.01
rs7321735	9375	TM9SF2	13	99001574	2.42E-01	10739	2.44E-07	Up	5.16	1393	0.62	0.66
rs487989	23649	POLA2	11	64821266	2.42E-01	10740	3.43E-01	Up	0.95	9551	0.62	0.05
rs4714675	401262	CRIP3	6	43395871	2.42E-01	10741	2.36E-02	Down	2.26	5225	0.62	0.16
rs612456	50836	TAS2R8	12	10866008	2.42E-01	10742	2.33E-01	Up	1.19	8593	0.62	0.06
rs612456	50839	TAS2R10	12	10866008	2.42E-01	10743	4.74E-01	Up	0.72	10530	0.62	0.03
rs612456	50837	TAS2R7	12	10866008	2.42E-01	10744	6.17E-01	Down	0.50	11556	0.62	0.02
rs612456	50835	TAS2R9	12	10866008	2.42E-01	10745	8.27E-01	Up	0.22	12837	0.62	0.01
rs2229611	2538	G6PC	17	38316992	2.42E-01	10746	6.23E-01	Up	0.49	11601	0.62	0.02
rs2234034	6906	SERPINA7		105085644	2.42E-01	10747	5.76E-01	Up	0.56	11284	0.62	0.02
rs201823	9227	LRAT	4	156026490	2.42E-01	10748	5.89E-01	Up	0.54	11363	0.62	0.02
rs7548659	6723	SRM	1	11041705	2.42E-01	10749	1.51E-15	Up	7.98	364	0.62	1.48
rs7548659	10747	MASP2	1	11041705	2.42E-01	10750	3.84E-01	Down	0.87	9870	0.62	0.04
rs7809344	51608	C7orf20	7	674815	2.42E-01	10751	1.34E-01	Up	1.50	7497	0.62	0.09
rs1699221	79400	NOX5	15	67103840	2.42E-01	10752	2.11E-01	Up	1.25	8365	0.62	0.07
rs2223526	117285	DEFB118	20	29434300	2.42E-01	10753	3.50E-01	Down	0.93	9590	0.62	0.05
rs2016394	1746	DLX2	2	172798478	2.42E-01	10754	4.69E-04	Up	3.50	2937	0.62	0.33
rs8179078	5346	PLIN	15	88026747	2.43E-01	10755	8.08E-01	Up	0.24	12712	0.62	0.01
rs2467779	23462	HEY1	8	80845876	2.43E-01	10756	1.15E-01	Down	1.57	7224	0.62	0.09
rs3761292	5075	PAX1	20	21627930	2.43E-01	10757	1.57E-01	Up	1.42	7765	0.62	0.08
rs7603501	10096	ACTR3	2	114397112	2.43E-01	10758	1.41E-01	Down	1.47	7589	0.61	0.09
rs1550721	51762	RAB8B	15	61282338	2.43E-01	10759	8.12E-02	Down	1.74	6653	0.61	0.11
rs7202185	81577	GFOD2	16	66272061	2.43E-01	10760	7.85E-02	Down	1.76	6605	0.61	0.11
rs7828983	2843	GPR20	8	142439838	2.43E-01	10761	9.64E-02	Down	1.66	6931	0.61	0.10
rs7202185	84080	C16orf48	16	66272061	2.43E-01	10762	2.35E-01	Down	1.19	8619	0.61	0.06
rs17108140	54987	C1orf123	1	53394699	2.43E-01	10763	3.00E-08	Down	5.54	1165	0.61	0.75
rs17108140	1376	CPT2	1	53394699	2.43E-01	10764	2.43E-03	Up	3.03	3666	0.61	0.26
rs17108140	4116	MAGOH	1	53394699	2.43E-01	10765	7.91E-02	Down	1.76	6616	0.61	0.11
rs423367	59285	CACNG6	19	59216837	2.43E-01	10766	2.19E-01	Up	1.23	8450	0.61	0.07
rs753663	3728	JUP	17	37199931	2.43E-01	10767	3.70E-03	Up	2.90	3885	0.61	0.24
rs2195933	153830	RNF145	5	158570574	2.43E-01	10768	4.59E-01	Down	0.74	10415	0.61	0.03

rs12842228	6451	SH3BGRL		80324621	2.43E-01	10769	1.68E-02	Down	2.39	4926	0.61	0.18
rs839768	112950	MED8	1	43540716	2.43E-01	10770	3.43E-02	Up	2.12	5603	0.61	0.15
rs10037844	114825	PWWP2A	5	159446445	2.44E-01	10771	1.40E-01	Up	1.47	7577	0.61	0.09
rs2979896	5423	POLB	8	42328661	2.44E-01	10772	7.79E-05	Up	3.95	2384	0.61	0.41
rs632324	8867	SYNJ1	21	33024506	2.44E-01	10773	4.35E-01	Down	0.78	10235	0.61	0.04
rs3027845	27336	HTATSF1		135314266	2.44E-01	10774	7.12E-01	Up	0.37	12143	0.61	0.01
rs12533649	5439	POLR2J	7	101704610	2.44E-01	10775	7.57E-06	Up	4.48	1890	0.61	0.51
rs759996	495	ATP4A	19	40730061	2.44E-01	10776	7.34E-01	Down	0.34	12278	0.61	0.01
rs4851526	7850	IL1R2	2	102076888	2.44E-01	10777	1.31E-01	Down	1.51	7455	0.61	0.09
rs2859593	91	ACVR1B	12	50613223	2.44E-01	10778	6.36E-01	Up	0.47	11678	0.61	0.02
rs12625505	5266	PI3	20	43245460	2.44E-01	10779	1.59E-01	Down	1.41	7805	0.61	0.08
rs4371935	339175	METTL2A	7	127744604	2.44E-01	10780	3.80E-07	Up	5.08	1449	0.61	0.64
rs4829777	203523	ZNF449		134218804	2.44E-01	10781	2.67E-02	Down	2.22	5350	0.61	0.16
rs3059	977	CD151	11	830319	2.44E-01	10782	4.76E-01	Down	0.71	10542	0.61	0.03
rs3059	5441	POLR2L	11	830319	2.44E-01	10783	5.79E-01	Up	0.55	11307	0.61	0.02
rs10119535	3376	IARS	9	92123784	2.44E-01	10784	3.18E-10	Up	6.29	812	0.61	0.95
rs4129537	474	ATOH1	4	95112320	2.44E-01	10785	3.77E-01	Down	0.88	9815	0.61	0.04
rs12177	4938	OAS1	12	111798145	2.44E-01	10786	1.22E-01	Up	1.55	7331	0.61	0.09
rs16944762	65264	UBE2Z	17	44362496	2.44E-01	10787	7.34E-01	Up	0.34	12279	0.61	0.01
rs16944762	11267	SNF8	17	44362496	2.44E-01	10788	9.41E-01	Up	0.07	13497	0.61	0.00
rs11177325	57122	NUP107	12	67351245	2.44E-01	10789	8.77E-02	Down	1.71	6767	0.61	0.11
rs2213842	51175	TUBE1	6	112530503	2.45E-01	10790	3.82E-14	Down	7.57	444	0.61	1.34
rs3782323	2819	GPD1	12	48769188	2.45E-01	10791	8.65E-01	Down	0.17	13034	0.61	0.01
rs249038	9765	ZFYVE16	5	79781225	2.45E-01	10792	2.19E-01	Up	1.23	8443	0.61	0.07
rs3810182	94115	CGB8	19	54252273	2.45E-01	10793	4.11E-01	Up	0.82	10066	0.61	0.04
rs11168427	140461	ASB8	12	46825246	2.45E-01	10794	3.29E-01	Up	0.98	9431	0.61	0.05
rs849749	375	ARF1	1	224603480	2.45E-01	10795	4.36E-04	Up	3.52	2907	0.61	0.34
rs849749	79169	C1orf35	1	224603480	2.45E-01	10796	5.15E-01	Down	0.65	10818	0.61	0.03
rs595379	85463	ZC3H12C	11	109518082	2.45E-01	10797	9.68E-01	Up	0.04	13689	0.61	0.00
rs7430653	64393	ZMAT3	3	180248428	2.45E-01	10798	1.05E-01	Down	1.62	7075	0.61	0.10
rs7183034	3658	IREB2	15	76497821	2.45E-01	10799	1.54E-01	Down	1.43	7734	0.61	0.08
rs836178	84987	C12orf62	12	48784406	2.45E-01	10800	5.15E-02	Down	1.95	6024	0.61	0.13
rs12142240	10489	LRRC41	1	46459321	2.45E-01	10801	2.53E-01	Down	1.14	8797	0.61	0.06
rs12142240	8438	RAD54L	1	46459321	2.45E-01	10802	5.00E-01	Up	0.67	10719	0.61	0.03
rs1891373	4601	MXI1	10	111960864	2.45E-01	10803	4.10E-10	Down	6.25	834	0.61	0.94
rs15413	10618	TGOLN2	2	85481037	2.45E-01	10804	1.33E-01	Up	1.50	7479	0.61	0.09
rs7954087	5830	PEX5	12	7242343	2.46E-01	10805	5.54E-01	Up	0.59	11110	0.61	0.03
rs222837	1856	DVL2	17	7073280	2.46E-01	10806	2.00E-04	Down	3.72	2631	0.61	0.37
rs222837	79142	PHF23	17	7073280	2.46E-01	10807	4.33E-02	Down	2.02	5834	0.61	0.14
rs222837	37	ACADVL	17	7073280	2.46E-01	10808	1.54E-01	Down	1.43	7730	0.61	0.08
rs222837	1742	DLG4	17	7073280	2.46E-01	10809	4.05E-01	Down	0.83	10022	0.61	0.04
rs2642273	22931	RAB18	10	27855671	2.46E-01	10810	6.83E-06	Up	4.50	1870	0.61	0.52
rs874269	208	AKT2	19	45423785	2.46E-01	10811	1.74E-01	Up	1.36	7951	0.61	0.08
rs874269	79935	CNTD2	19	45423785	2.46E-01	10812	3.80E-01	Down	0.88	9834	0.61	0.04
rs11687764	10988	METAP2	2	210315272	2.46E-01	10813	4.27E-06	Up	4.60	1790	0.61	0.54
rs11687764	4133	MAP2	2	210315272	2.46E-01	10814	4.74E-04	Up	3.50	2943	0.61	0.33
rs2078674	2765	GML	8	143925235	2.46E-01	10815	3.47E-01	Up	0.94	9572	0.61	0.05
rs2172494	761	CA3	8	86527569	2.46E-01	10816	6.04E-01	Up	0.52	11465	0.61	0.02
rs3917419	6401	SELE	1	166431477	2.46E-01	10817	4.29E-01	Down	0.79	10180	0.61	0.04
rs3917419	6402	SELL	1	166431477	2.46E-01	10818	9.39E-01	Up	0.08	13488	0.61	0.00
rs12741800	3151	HMGN2	1	26486690	2.46E-01	10819	7.42E-02	Up	1.79	6509	0.61	0.11
rs400774	3854	KRT6B	12	51122489	2.46E-01	10820	4.41E-01	Down	0.77	10274	0.61	0.04
rs1560687	54815	GATAD2A	19	19423902	2.46E-01	10821	1.87E-03	Up	3.11	3497	0.61	0.27
rs10483618	6815	STYX	14	52273340	2.46E-01	10822	7.64E-02	Up	1.77	6559	0.61	0.11
rs4804903	898	CCNE1	19	34984906	2.46E-01	10823	9.13E-01	Up	0.11	13328	0.61	0.00
rs1866046	200576	PIP5K3	2	209020485	2.46E-01	10824	8.18E-03	Up	2.64	4356	0.61	0.21
rs12555920	8727	CTNNAL1	9	108845292	2.46E-01	10825	2.95E-04	Up	3.62	2768	0.61	0.35
rs6458690	4594	MUT	6	49519578	2.47E-01	10826	1.60E-03	Up	3.16	3418	0.61	0.28
rs11579964	29097	CNIH4	1	220845675	2.47E-01	10827	8.74E-01	Down	0.16	13089	0.61	0.01
rs4747069	5551	PRF1	10	72044845	2.47E-01	10828	5.40E-01	Up	0.61	11016	0.61	0.03
rs2879130	284486	THEM5	1	148643171	2.47E-01	10829	5.23E-01	Up	0.64	10874	0.61	0.03
rs3787041	284439	SLC25A42	19	19092332	2.47E-01	10830	2.37E-06	Up	4.72	1694	0.61	0.56
rs10283236	4061	LY6E	8	144179352	2.47E-01	10831	1.64E-01	Down	1.39	7859	0.61	0.08
rs10283236	286122	C8orf31	8	144179352	2.47E-01	10832	8.90E-01	Down	0.14	13187	0.61	0.01
rs6717729	150590	C2orf15	2	99215459	2.47E-01	10833	3.84E-01	Down	0.87	9868	0.61	0.04
rs752340	8677	STX10	19	13137345	2.47E-01	10834	3.63E-03	Up	2.91	3876	0.61	0.24
rs224995	1734	DIO2	14	79754457	2.47E-01	10835	2.07E-08	Up	5.60	1130	0.61	0.77
rs6709104	3234	HOXD8	2	176804903	2.47E-01	10836	1.22E-02	Up	2.51	4645	0.61	0.19
rs9918635	7425	VGF	7	100399139	2.47E-01	10837	2.49E-01	Down	1.15	8754	0.61	0.06
rs9918635	375607	C7orf52	7	100399139	2.47E-01	10838	5.49E-01	Up	0.60	11074	0.61	0.03
rs13003408	9262	STK17B	2	196871194	2.47E-01	10839	9.62E-03	Up	2.59	4477	0.61	0.20
rs917046	1750	DLX6	7	96273558	2.47E-01	10840	8.66E-01	Down	0.17	13039	0.61	0.01
rs2305529	29115	SAP30BP	17	71213848	2.47E-01	10841	1.20E-03	Down	3.24	3292	0.61	0.29
rs10492102	50840	TAS2R14	12	10977650	2.48E-01	10842	6.10E-01	Up	0.51	11502	0.61	0.02
rs1983298	3300	DNAJB2	2	219981339	2.48E-01	10843	5.52E-01	Down	0.60	11091	0.61	0.03
rs1983298	5798	PTPRN	2	219981339	2.48E-01	10844	8.67E-01	Down	0.17	13046	0.61	0.01
rs8063084	57407	NMRAL1	16	4476556	2.48E-01	10845	5.07E-04	Up	3.48	2965	0.61	0.33
rs8063084	3163	HMOX2	16	4476556	2.48E-01	10846	2.50E-03	Up	3.02	3686	0.61	0.26
rs2254883	5440	POLR2K	8	101232134	2.48E-01	10847	4.85E-04	Down	3.49	2952	0.61	0.33
rs9675392	9050	PSTPIP2	18	41839534	2.48E-01	10848	5.14E-01	Up	0.65	10807	0.61	0.03
rs9381084	116113	FOXP4	6	41678328	2.48E-01	10849	3.24E-01	Up	0.99	9398	0.61	0.05

rs11651687	5573	PRKAR1A	17	64029901	2.48E-01	10850	8.63E-12	Down	6.83	635	0.61	1.11
rs4246529	54344	DPM3	1	151928333	2.48E-01	10851	5.81E-01	Down	0.55	11322	0.61	0.02
rs3754876	3631	INPP4A	2	98644385	2.48E-01	10852	7.81E-01	Up	0.28	12554	0.61	0.01
rs11970361	7128	TNFAIP3	6	138220829	2.49E-01	10853	2.61E-02	Down	2.23	5322	0.60	0.16
rs8065874	6470	SHMT1	17	18216572	2.49E-01	10854	9.07E-01	Down	0.12	13290	0.60	0.00
rs4827379	60401	EDA2R		65596981	2.49E-01	10855	1.82E-01	Down	1.33	8050	0.60	0.07
rs1528827	56906	THAP10	15	68974838	2.49E-01	10856	2.13E-01	Down	1.25	8391	0.60	0.07
rs464541	55670	PEX26	22	16937442	2.49E-01	10857	1.48E-01	Up	1.45	7656	0.60	0.08
rs10211808	63916	ELMO2	20	44419171	2.49E-01	10858	1.86E-02	Up	2.35	5009	0.60	0.17
rs16941437	144717	FAM109A	12	110255759	2.49E-01	10859	2.36E-01	Up	1.18	8625	0.60	0.06
rs10305724	405	ARNT	1	147608438	2.49E-01	10860	5.72E-01	Down	0.57	11257	0.60	0.02
rs10305724	1513	CTSK	1	147608438	2.49E-01	10861	7.69E-01	Down	0.29	12484	0.60	0.01
rs1969025	2916	GRM6	5	178357798	2.49E-01	10862	8.16E-01	Down	0.23	12761	0.60	0.01
rs7541966	2329	FMO4	1	168038813	2.50E-01	10863	8.68E-01	Down	0.17	13051	0.60	0.01
rs9907921	283991	FAM100B	17	71768508	2.50E-01	10864	2.06E-04	Up	3.71	2643	0.60	0.37
rs1106841	9533	POLR1C	6	43604640	2.50E-01	10865	6.20E-07	Up	4.99	1507	0.60	0.62
rs1106841	221424	C6orf154	6	43604640	2.50E-01	10866	8.40E-01	Down	0.20	12902	0.60	0.01
rs10845242	50838	TAS2R13	12	10957806	2.50E-01	10867	3.00E-02	Up	2.17	5463	0.60	0.15
rs10859110	4060	LUM	12	90007313	2.50E-01	10868	8.66E-02	Down	1.71	6749	0.60	0.11
rs10749572	84332	DYDC2	10	82126644	2.50E-01	10869	1.96E-01	Down	1.29	8218	0.60	0.07
rs5756774	79159	NOL12	22	36416160	2.50E-01	10870	5.31E-01	Down	0.63	10945	0.60	0.03
rs2544809	1627	DBN1	5	176818105	2.50E-01	10871	3.07E-01	Down	1.02	9242	0.60	0.05
rs2544809	80758	PRR7	5	176818105	2.50E-01	10872	4.91E-01	Down	0.69	10659	0.60	0.03
rs13103963	55013	CCDC109B	4	110934967	2.50E-01	10873	4.10E-01	Up	0.82	10056	0.60	0.04
rs17798043	90	ACVR1	2	158488785	2.50E-01	10874	2.05E-03	Down	3.08	3546	0.60	0.27
rs6750645	84317	CCDC115	2	130826327	2.50E-01	10875	1.57E-02	Down	2.42	4854	0.60	0.18
rs6750645	92856	IMP4	2	130826327	2.50E-01	10876	6.70E-01	Up	0.43	11886	0.60	0.02
rs12661230	9994	CASP8AP2	6	90592188	2.50E-01	10877	6.45E-01	Down	0.46	11746	0.60	0.02
rs11595324	1147	CHUK	10	101969408	2.50E-01	10878	1.01E-01	Up	1.64	7013	0.60	0.10
rs10920260	55705	IPO9	1	198532239	2.50E-01	10879	1.24E-02	Down	2.50	4653	0.60	0.19
rs7223332	125113	KRT222P	17	36078162	2.50E-01	10880	3.20E-03	Down	2.95	3816	0.60	0.25
rs6502005	23460	ABCA6	17	64615106	2.51E-01	10881	4.44E-01	Down	0.76	10303	0.60	0.04
rs10414149	8778	SIGLEC5	19	56819556	2.51E-01	10882	1.01E-01	Down	1.64	7004	0.60	0.10
rs1267070	10010	TANK	2	161813990	2.51E-01	10883	8.28E-02	Down	1.73	6682	0.60	0.11
rs2748531	26277	TINF2	14	23791741	2.51E-01	10884	2.46E-01	Up	1.16	8719	0.60	0.06
rs2748531	7051	TGM1	14	23791741	2.51E-01	10885	4.70E-01	Down	0.72	10497	0.60	0.03
rs3792815	3887	KRT81	5	143172404	2.51E-01	10886	5.01E-02	Up	1.96	6000	0.60	0.13
rs3792815	57824	HMHB1	5	143172404	2.51E-01	10887	8.53E-01	Down	0.19	12972	0.60	0.01
rs819164	434	ASIP	20	32303115	2.51E-01	10888	9.57E-01	Up	0.05	13596	0.60	0.00
rs5757715	91582	RPS19BP1	22	38265654	2.51E-01	10889	5.31E-02	Up	1.93	6064	0.60	0.13
rs2613225	1015	CDH17	8	95301625	2.51E-01	10890	4.36E-01	Down	0.78	10239	0.60	0.04
rs10487060	222223	KIAA1324L	7	86235004	2.51E-01	10891	2.01E-01	Up	1.28	8264	0.60	0.07
rs7673610	152815	THAP6	4	76817715	2.52E-01	10892	2.64E-01	Up	1.12	8899	0.60	0.06
rs6673387	127933	UHMK1	1	159190070	2.52E-01	10893	1.28E-02	Up	2.49	4668	0.60	0.19
rs5911060	2747	GLUD2		119896870	2.52E-01	10894	2.21E-01	Down	1.22	8479	0.60	0.07
rs4398451	5868	RAB5A	3	19985890	2.52E-01	10895	4.13E-01	Up	0.82	10075	0.60	0.04
rs10915239	51102	MECR	1	29344565	2.52E-01	10896	2.01E-03	Up	3.09	3534	0.60	0.27
rs10915239	6429	SFRS4	1	29344565	2.52E-01	10897	2.24E-02	Down	2.28	5178	0.60	0.16
rs17660212	64063	PRSS22	16	2858293	2.52E-01	10898	7.39E-01	Down	0.33	12313	0.60	0.01
rs13434441	3347	HTN3	4	71086157	2.52E-01	10899	9.83E-02	Up	1.65	6970	0.60	0.10
rs6495119	80153	EDC3	15	72721493	2.52E-01	10900	4.64E-05	Up	4.07	2263	0.60	0.43
rs6571499	6037	RNASE3	14	20410989	2.52E-01	10901	1.85E-01	Down	1.33	8075	0.60	0.07
rs4794779	30837	SOCS7	17	33745974	2.52E-01	10902	3.18E-02	Up	2.15	5519	0.60	0.15
rs17646919	8897	MTMR3	22	28725415	2.52E-01	10903	6.14E-05	Up	4.01	2334	0.60	0.42
rs6708488	7855	FZD5	2	208466680	2.52E-01	10904	1.81E-01	Up	1.34	8041	0.60	0.07
rs17126535	55775	TDP1	14	89571430	2.52E-01	10905	1.43E-08	Up	5.66	1088	0.60	0.78
rs481835	1351	COX8A	11	63482358	2.52E-01	10906	1.38E-04	Up	3.81	2523	0.60	0.39
rs481835	79829	NAT11	11	63482358	2.52E-01	10907	8.12E-01	Down	0.24	12735	0.60	0.01
rs8131020	54145	H2BFS	21	43805491	2.52E-01	10908	1.91E-04	Up	3.73	2617	0.60	0.37
rs238224	9552	SPAG7	17	4804133	2.52E-01	10909	3.45E-04	Down	3.58	2828	0.60	0.35
rs238224	23125	CAMTA2	17	4804133	2.52E-01	10910	2.94E-03	Up	2.97	3767	0.60	0.25
rs238224	5216	PFN1	17	4804133	2.52E-01	10911	7.79E-02	Down	1.76	6592	0.60	0.11
rs238224	2027	ENO3	17	4804133	2.52E-01	10912	1.19E-01	Up	1.56	7285	0.60	0.09
rs9844683	344838	PAQR9	3	144155467	2.52E-01	10913	5.63E-01	Down	0.58	11187	0.60	0.02
rs1947187	5308	PITX2	4	111903101	2.52E-01	10914	7.79E-01	Down	0.28	12547	0.60	0.01
rs11665903	1346	COX7A1	19	41344025	2.52E-01	10915	7.26E-22	Down	9.61	163	0.60	2.11
rs4792930	27175	TUBG2	17	38059370	2.52E-01	10916	3.78E-03	Down	2.90	3899	0.60	0.24
rs231622	10309	CCNO	5	54564347	2.52E-01	10917	2.95E-06	Up	4.67	1732	0.60	0.55
rs516906	5737	PTGFR	1	78725127	2.53E-01	10918	6.61E-02	Down	1.84	6362	0.60	0.12
rs997154	84962	JUB	14	22534322	2.53E-01	10919	8.26E-11	Down	6.50	746	0.60	1.01
rs2685498	51649	MRPS23	17	53287987	2.53E-01	10920	4.42E-01	Down	0.77	10283	0.60	0.04
rs2279597	2799	GNS	12	63396519	2.53E-01	10921	2.65E-01	Up	1.11	8910	0.60	0.06
rs11975493	340348	TSPAN33	7	128367721	2.53E-01	10922	5.08E-01	Up	0.66	10773	0.60	0.03
rs12811983	79720	VPS37B	12	121901451	2.53E-01	10923	3.51E-01	Up	0.93	9597	0.60	0.05
rs1044938	1392	CRH	8	67271779	2.53E-01	10924	4.16E-01	Up	0.81	10097	0.60	0.04
rs5906714	10013	HDAC6		48440895	2.53E-01	10925	4.81E-01	Down	0.70	10580	0.60	0.03
rs5906714	27344	PCSK1N		48440895	2.53E-01	10926	9.03E-01	Up	0.12	13267	0.60	0.00
rs11787849	203328	SUSD3	9	92881842	2.53E-01	10927	2.86E-01	Down	1.07	9083	0.60	0.05
rs2288322	51661	FKBP7	2	179148624	2.53E-01	10928	3.68E-01	Down	0.90	9749	0.60	0.04
rs11893208	6332	SCN7A	2	167163242	2.53E-01	10929	6.19E-01	Down	0.50	11565	0.60	0.02
rs1557534	8542	APOL1	22	34957725	2.53E-01	10930	3.49E-04	Down	3.58	2833	0.60	0.35

rs12142905	29914	UBIAD1	1	11253249	2.53E-01	10931	6.75E-04	Up	3.40	3067	0.60	0.32
rs727266	3875	KRT18	12	51642678	2.53E-01	10932	3.32E-16	Up	8.17	339	0.60	1.55
rs6474422	55145	THAP1	8	42824859	2.53E-01	10933	9.05E-01	Down	0.12	13282	0.60	0.00
rs7547496	55057	AIM1L	1	26374623	2.53E-01	10934	6.21E-01	Up	0.49	11592	0.60	0.02
rs2265932	6646	SOAT1	1	175999440	2.53E-01	10935	1.56E-01	Up	1.42	7754	0.60	0.08
rs10426895	5670	PSG2	19	48285261	2.53E-01	10936	2.69E-01	Up	1.11	8938	0.60	0.06
rs2862797	51427	ZNF107	7	63594417	2.53E-01	10937	7.24E-02	Up	1.80	6475	0.60	0.11
rs11975590	79161	C7orf23	7	86511167	2.53E-01	10938	2.68E-04	Down	3.64	2739	0.60	0.36
rs4375633	85455	DISP2	15	38426862	2.53E-01	10939	1.01E-01	Up	1.64	7030	0.60	0.10
rs4282910	10556	RPP30	10	92621224	2.54E-01	10940	1.68E-01	Down	1.38	7893	0.60	0.08
rs12640749	3148	HMGB2	4	174641956	2.54E-01	10941	3.57E-02	Down	2.10	5638	0.60	0.14
rs775700	57396	CLK4	5	177967605	2.54E-01	10942	5.91E-01	Up	0.54	11382	0.60	0.02
rs6929796	4049	LTA	6	31630648	2.54E-01	10943	6.10E-01	Up	0.51	11506	0.60	0.02
rs8057997	79191	IRX3	16	52885828	2.54E-01	10944	9.49E-01	Up	0.06	13547	0.60	0.00
rs762485	2152	F3	1	94716105	2.54E-01	10945	1.49E-02	Up	2.43	4812	0.60	0.18
rs3740487	57715	SEMA4G	10	102740773	2.54E-01	10946	5.09E-03	Down	2.80	4079	0.60	0.23
rs3740487	84545	MRPL43	10	102740773	2.54E-01	10947	5.67E-02	Down	1.91	6152	0.60	0.12
rs166670	219670	C10orf63	10	25305309	2.54E-01	10948	9.65E-01	Down	0.04	13657	0.60	0.00
rs888060	58157	NGB	14	76796486	2.54E-01	10949	7.83E-01	Up	0.28	12562	0.59	0.01
rs3649	26258	PLDN	15	43696596	2.54E-01	10950	6.20E-01	Up	0.50	11576	0.59	0.02
rs1065356	23564	DDAH2	6	31794987	2.54E-01	10951	3.24E-01	Up	0.99	9393	0.59	0.05
rs6858087	173	AFM	4	74735873	2.54E-01	10952	6.81E-02	Up	1.82	6397	0.59	0.12
rs7600843	129804	FBLN7	2	112655273	2.54E-01	10953	3.33E-02	Up	2.13	5571	0.59	0.15
rs758664	27091	CACNG5	17	62301734	2.55E-01	10954	4.82E-01	Up	0.70	10586	0.59	0.03
rs1148399	388536	ZNF790	19	42021145	2.55E-01	10955	2.03E-01	Up	1.27	8282	0.59	0.07
rs16865717	91543	RSAD2	2	6983216	2.55E-01	10956	7.61E-01	Up	0.30	12446	0.59	0.01
rs2008413	2273	FHL1		134979879	2.55E-01	10957	4.03E-36	Down	12.55	32	0.59	3.54
rs2008413	3075	CFH		134979879	2.55E-01	10958	6.40E-08	Down	5.41	1246	0.59	0.72
rs3794746	1070	CETN3	5	89719620	2.55E-01	10959	7.80E-03	Up	2.66	4325	0.59	0.21
rs8192539	3769	KCNJ13	2	233458271	2.55E-01	10960	6.67E-03	Up	2.71	4226	0.59	0.22
rs8039808	56986	DTWD1	15	47727138	2.55E-01	10961	4.92E-01	Up	0.69	10662	0.59	0.03
rs12325861	5878	RAB5C	17	37542938	2.55E-01	10962	9.62E-01	Up	0.05	13632	0.59	0.00
rs666842	257	ALX3	1	110310494	2.55E-01	10963	8.70E-01	Up	0.16	13061	0.59	0.01
rs2269346	7922	SLC39A7	6	33266876	2.55E-01	10964	3.51E-02	Up	2.11	5626	0.59	0.15
rs2269346	7923	HSD17B8	6	33266876	2.55E-01	10965	1.23E-01	Up	1.54	7344	0.59	0.09
rs2269346	6015	RING1	6	33266876	2.55E-01	10966	3.13E-01	Down	1.01	9292	0.59	0.05
rs2269346	6257	RXRB	6	33266876	2.55E-01	10967	5.77E-01	Down	0.56	11292	0.59	0.02
rs6766	51052	PRLH	2	238265164	2.56E-01	10968	6.57E-01	Down	0.44	11813	0.59	0.02
rs209677	9139	CBFA2T2	20	31603948	2.56E-01	10969	2.74E-06	Up	4.69	1720	0.59	0.56
rs10279978	222962	SLC29A4	7	5092628	2.56E-01	10970	7.01E-02	Down	1.81	6428	0.59	0.12
rs1726744	51400	PPME1	11	73543430	2.57E-01	10971	2.30E-01	Down	1.20	8565	0.59	0.06
rs11154839	85015	USP45	6	99978789	2.57E-01	10972	1.10E-02	Up	2.54	4570	0.59	0.20
rs6599389	84286	TMEM175	4	928943	2.57E-01	10973	3.35E-01	Up	0.96	9481	0.59	0.05
rs1468358	8985	PLOD3	7	100446608	2.57E-01	10974	4.75E-10	Up	6.23	851	0.59	0.93
rs1468358	10467	ZNHIT1	7	100446608	2.57E-01	10975	3.50E-03	Up	2.92	3861	0.59	0.25
rs6587625	6097	RORC	1	148633397	2.57E-01	10976	7.39E-04	Up	3.37	3098	0.59	0.31
rs12716783	162239	ZFP1	16	73760499	2.57E-01	10977	1.18E-01	Down	1.56	7258	0.59	0.09
rs1870071	58513	EPS15L1	19	16366106	2.57E-01	10978	3.47E-01	Down	0.94	9570	0.59	0.05
rs28513	6203	RPS9	19	59391198	2.57E-01	10979	3.06E-09	Up	5.93	972	0.59	0.85
rs226043	1428	CRYM	16	21195011	2.57E-01	10980	5.16E-02	Down	1.95	6027	0.59	0.13
rs1830792	171389	NLRP6	13	27771907	2.57E-01	10981	3.37E-01	Down	0.96	9498	0.59	0.05
rs1830792	255967	PAN3	13	27771907	2.57E-01	10982	7.12E-01	Up	0.37	12142	0.59	0.01
rs10914555	55108	BSDC1	1	32548679	2.57E-01	10983	2.29E-02	Up	2.28	5194	0.59	0.16
rs8064518	284129	SLC26A11	17	75837698	2.57E-01	10984	5.04E-01	Down	0.67	10745	0.59	0.03
rs9276991	3111	HLA-DOA	6	33089826	2.57E-01	10985	1.40E-01	Up	1.48	7575	0.59	0.09
rs6697911	2306	FOXD2	1	47619624	2.57E-01	10986	9.91E-02	Up	1.65	6981	0.59	0.10
rs713446	6519	SLC3A1	2	44416508	2.57E-01	10987	5.62E-01	Down	0.58	11173	0.59	0.03
rs12649108	91746	YTHDC1	4	69010708	2.57E-01	10988	8.99E-01	Down	0.13	13238	0.59	0.00
rs4338423	22874	PLEKHA6	1	200962821	2.57E-01	10989	3.36E-04	Down	3.59	2818	0.59	0.35
rs2136613	1959	EGR2	10	64264170	2.57E-01	10990	6.22E-15	Up	7.81	393	0.59	1.42
rs1523670	138804	OR13C4	9	104371870	2.57E-01	10991	9.66E-01	Up	0.04	13668	0.59	0.00
rs12472418	10494	STK25	2	242171705	2.58E-01	10992	1.52E-03	Up	3.17	3387	0.59	0.28
rs10490658	6801	STRN	2	37048049	2.58E-01	10993	3.25E-02	Up	2.14	5543	0.59	0.15
rs3317	6728	SRP19	5	112240050	2.58E-01	10994	4.10E-05	Up	4.10	2234	0.59	0.44
rs7192639	79767	ELMO3	16	65772821	2.58E-01	10995	1.68E-06	Up	4.79	1645	0.59	0.58
rs7192639	1874	E2F4	16	65772821	2.58E-01	10996	3.61E-02	Down	2.10	5653	0.59	0.14
rs7192639	8996	NOL3	16	65772821	2.58E-01	10997	6.21E-01	Down	0.49	11587	0.59	0.02
rs7201173	255919	TMEM188	16	48620112	2.58E-01	10998	8.91E-03	Down	2.62	4429	0.59	0.21
rs3758653	1815	DRD4	11	626399	2.58E-01	10999	2.33E-01	Down	1.19	8592	0.59	0.06
rs3758653	6343	SCT	11	626399	2.58E-01	11000	6.94E-01	Down	0.39	12034	0.59	0.02
rs2043932	6328	SCN3A	2	165881221	2.58E-01	11001	1.13E-02	Up	2.53	4600	0.59	0.19
rs2229358	6668	SP2	17	43349153	2.58E-01	11002	5.34E-01	Up	0.62	10972	0.59	0.03
rs3761011	26539	OR10H1	19	15793760	2.58E-01	11003	1.48E-01	Down	1.45	7652	0.59	0.08
rs2536	5394	EXOSC10	1	11100979	2.58E-01	11004	7.09E-01	Up	0.37	12122	0.59	0.01
rs13008	25798	BRI3	7	97567526	2.58E-01	11005	6.83E-03	Up	2.71	4244	0.59	0.22
rs4927943	22820	COPG	3	130461706	2.58E-01	11006	6.54E-01	Up	0.45	11790	0.59	0.02
rs4927943	56941	C3orf37	3	130461706	2.58E-01	11007	9.03E-01	Up	0.12	13263	0.59	0.00
rs8107142	3249	HPN	19	40228204	2.58E-01	11008	3.69E-41	Up	13.41	16	0.59	4.04
rs8107142	6324	SCN1B	19	40228204	2.58E-01	11009	6.18E-01	Down	0.50	11562	0.59	0.02
rs10872646	348995	NUP43	6	150159789	2.58E-01	11010	8.19E-01	Up	0.23	12784	0.59	0.01
rs10772380	5554	PRH1	12	10936779	2.59E-01	11011	5.70E-01	Up	0.57	11238	0.59	0.02

rs6537600	1892	ECHS1	10	135093680	2.59E-01	11012	1.42E-07	Up	5.26	1339	0.59	0.68
rs6537600	196743	PAOX	10	135093680	2.59E-01	11013	1.63E-04	Up	3.77	2571	0.59	0.38
rs9426298	10691	GMEB1	1	28811538	2.59E-01	11014	2.07E-01	Up	1.26	8326	0.59	0.07
rs3104001	119180	LYZL2	10	30977910	2.59E-01	11015	1.12E-01	Up	1.59	7182	0.59	0.09
rs713863	10126	DNAL4	22	37522439	2.59E-01	11016	7.22E-02	Up	1.80	6472	0.59	0.11
rs17303283	56916	SMARCAD1	4	95509591	2.59E-01	11017	4.77E-03	Up	2.82	4029	0.59	0.23
rs3172417	2177	FANCD2	3	10117949	2.60E-01	11018	3.08E-02	Up	2.16	5490	0.59	0.15
rs3172417	115795	C3orf24	3	10117949	2.60E-01	11019	2.63E-01	Down	1.12	8893	0.59	0.06
rs3172417	55845	C3orf10	3	10117949	2.60E-01	11020	9.53E-01	Up	0.06	13576	0.59	0.00
rs510396	84259	DCUN1D5	11	102462262	2.60E-01	11021	7.60E-04	Up	3.37	3115	0.59	0.31
rs7849845	11191	PTENP1	9	33665629	2.60E-01	11022	7.40E-01	Up	0.33	12318	0.59	0.01
rs2071136	6611	SMS		21762887	2.60E-01	11023	2.44E-03	Up	3.03	3670	0.59	0.26
rs2868767	64405	CDH22	20	44258031	2.60E-01	11024	7.74E-01	Up	0.29	12526	0.58	0.01
rs1547669	221496	LEMD2	6	33883619	2.60E-01	11025	9.60E-01	Up	0.05	13620	0.58	0.00
rs478309	55572	FOXRED1	11	125645678	2.60E-01	11026	1.97E-02	Up	2.33	5057	0.58	0.17
rs478309	6734	SRPR	11	125645678	2.60E-01	11027	8.40E-01	Down	0.20	12903	0.58	0.01
rs7891099	84889	SLC7A3		69948555	2.60E-01	11028	1.19E-01	Down	1.56	7272	0.58	0.09
rs17168179	57001	ACN9	7	96380187	2.60E-01	11029	1.56E-09	Up	6.04	924	0.58	0.88
rs8177191	7018	TF	3	134950837	2.60E-01	11030	2.32E-11	Down	6.68	678	0.58	1.06
rs16925377	57053	CHRNA10	11	3633651	2.60E-01	11031	1.49E-01	Down	1.44	7677	0.58	0.08
rs4793658	114881	OSBPL7	17	43233732	2.60E-01	11032	7.72E-01	Down	0.29	12511	0.58	0.01
rs2073665	7419	VDAC3	8	42351646	2.60E-01	11033	2.37E-09	Down	5.97	945	0.58	0.86
rs2073665	27121	DKK4	8	42351646	2.60E-01	11034	2.69E-02	Down	2.21	5353	0.58	0.16
rs6779410	378	ARF4	3	57537734	2.60E-01	11035	2.40E-03	Up	3.04	3662	0.58	0.26
rs7950649	57830	KRTAP5-8	11	70898979	2.60E-01	11036	5.57E-01	Up	0.59	11134	0.58	0.03
rs10425463	199777	ZNF626	19	20618710	2.60E-01	11037	9.84E-01	Up	0.02	13809	0.58	0.00
rs11212495	8065	CUL5	11	107424109	2.60E-01	11038	2.69E-01	Down	1.10	8948	0.58	0.06
rs1869362	4498	MT1JP	7	157944110	2.61E-01	11039	3.89E-09	Down	5.89	990	0.58	0.84
rs1869362	54892	NCAPG2	7	157944110	2.61E-01	11040	3.01E-05	Up	4.17	2170	0.58	0.45
rs2451741	79692	ZNF322A	6	26737383	2.61E-01	11041	1.76E-02	Up	2.37	4963	0.58	0.18
rs829021	4891	SLC11A2	12	49662086	2.61E-01	11042	6.36E-02	Up	1.85	6314	0.58	0.12
rs7169359	302	ANXA2	15	58420968	2.61E-01	11043	9.01E-37	Down	12.67	30	0.58	3.60
rs6962551	51373	MRPS17	7	55802304	2.61E-01	11044	1.29E-05	Up	4.36	2012	0.58	0.49
rs6962551	2631	GBAS	7	55802304	2.61E-01	11045	1.66E-01	Up	1.39	7872	0.58	0.08
rs7259375	83639	TEX101	19	48576925	2.61E-01	11046	5.83E-01	Down	0.55	11333	0.58	0.02
rs2275380	9813	KIAA0494	1	46859748	2.61E-01	11047	6.70E-03	Down	2.71	4230	0.58	0.22
rs2275380	64756	ATPAF1	1	46859748	2.61E-01	11048	1.22E-01	Down	1.55	7325	0.58	0.09
rs1357365	6351	CCL4	17	31460645	2.61E-01	11049	4.43E-04	Up	3.51	2915	0.58	0.34
rs9919624	6392	SDHD	11	111464772	2.61E-01	11050	8.53E-09	Down	5.76	1044	0.58	0.81
rs9919624	26521	TIMM8B	11	111464772	2.61E-01	11051	1.81E-08	Up	5.63	1115	0.58	0.77
rs9919624	55216	C11orf57	11	111464772	2.61E-01	11052	2.38E-02	Up	2.26	5240	0.58	0.16
rs3789042	5824	PEX19	1	157078588	2.61E-01	11053	2.87E-02	Down	2.19	5412	0.58	0.15
rs12104272	6237	RRAS	19	54839864	2.61E-01	11054	1.56E-03	Down	3.16	3403	0.58	0.28
rs4255049	79970	ZNF767	7	148724343	2.61E-01	11055	7.54E-01	Down	0.31	12402	0.58	0.01
rs17103227	2301	FOXE3	1	47601437	2.61E-01	11056	4.41E-01	Up	0.77	10277	0.58	0.04
rs920658	9536	PTGES	9	129591789	2.61E-01	11057	2.01E-01	Down	1.28	8270	0.58	0.07
rs10912537	2327	FMO2	1	167898190	2.62E-01	11058	4.65E-01	Down	0.73	10467	0.58	0.03
rs7481525	11187	PKP3	11	368188	2.62E-01	11059	5.65E-09	Up	5.82	1025	0.58	0.82
rs2964574	10146	G3BP1	5	151135904	2.62E-01	11060	1.36E-07	Up	5.27	1328	0.58	0.69
rs6994464	255926	ADAM5P	8	39311337	2.62E-01	11061	9.64E-02	Up	1.66	6933	0.58	0.10
rs4865117	132946	ARL9	4	57239229	2.62E-01	11062	1.86E-02	Down	2.35	5007	0.58	0.17
rs7759295	54209	TREM2	6	41243828	2.62E-01	11063	2.97E-01	Up	1.04	9172	0.58	0.05
rs7759295	340205	TREML1	6	41243828	2.62E-01	11064	4.04E-01	Up	0.84	10009	0.58	0.04
rs6966675	221927	C7orf27	7	2370230	2.62E-01	11065	1.66E-03	Up	3.15	3436	0.58	0.28
rs1871892	54752	FNDC8	17	30472931	2.62E-01	11066	6.64E-01	Up	0.43	11852	0.58	0.02
rs151352	81027	TUBB1	20	57035390	2.62E-01	11067	6.68E-01	Up	0.43	11870	0.58	0.02
rs4391967	23588	KLHDC2	14	49304327	2.62E-01	11068	4.60E-01	Up	0.74	10426	0.58	0.03
rs10845289	259294	TAS2R48	12	11058493	2.62E-01	11069	4.34E-01	Up	0.78	10224	0.58	0.04
rs4844121	1741	DLG3		69474979	2.62E-01	11070	1.51E-05	Up	4.33	2037	0.58	0.48
rs2294120	80778	ZNF34	8	145974371	2.62E-01	11071	5.18E-01	Down	0.65	10843	0.58	0.03
rs7811753	3199	HOXA2	7	26934079	2.62E-01	11072	3.03E-02	Down	2.17	5475	0.58	0.15
rs8914	392	ARHGAP1	11	46655700	2.63E-01	11073	1.81E-02	Down	2.36	4990	0.58	0.17
rs8914	9776	KIAA0652	11	46655700	2.63E-01	11074	9.67E-01	Down	0.04	13679	0.58	0.00
rs4345522	157638	FAM84B	8	127656556	2.63E-01	11075	7.08E-20	Up	9.12	204	0.58	1.92
rs4288464	51759	C9orf78	9	129695557	2.63E-01	11076	9.73E-01	Up	0.03	13716	0.58	0.00
rs10519308	161502	C15orf26	15	79161506	2.63E-01	11077	2.69E-01	Down	1.11	8937	0.58	0.06
rs10458655	5033	P4HA1	10	74479602	2.63E-01	11078	2.98E-08	Up	5.56	1153	0.58	0.75
rs11651313	64518	TEKT3	17	15163443	2.64E-01	11079	1.28E-02	Down	2.49	4669	0.58	0.19
rs16846748	344752	AADACL2	3	152920945	2.64E-01	11080	7.71E-01	Down	0.29	12500	0.58	0.01
rs11815205	22943	DKK1	10	53730107	2.64E-01	11081	6.48E-01	Down	0.46	11765	0.58	0.02
rs8176373	7127	TNFAIP2	14	102673975	2.64E-01	11082	9.57E-05	Down	3.90	2441	0.58	0.40
rs7734699	79810	PTCD2	5	71690514	2.64E-01	11083	3.25E-01	Down	0.98	9403	0.58	0.05
rs17807215	29062	WDR91	7	134341246	2.64E-01	11084	1.76E-02	Up	2.37	4964	0.58	0.18
rs508448	4985	OPRD1	1	29002141	2.64E-01	11085	5.58E-01	Up	0.59	11141	0.58	0.03
rs400106	126070	ZNF440	19	11804697	2.64E-01	11086	2.70E-01	Up	1.10	8951	0.58	0.06
rs7307516	55846	ITFG2	12	2811124	2.64E-01	11087	3.49E-03	Down	2.92	3859	0.58	0.25
rs11170567	6895	TARBP2	12	52195106	2.64E-01	11088	1.52E-09	Up	6.04	921	0.58	0.88
rs11170567	8620	NPFF	12	52195106	2.64E-01	11089	6.66E-03	Up	2.71	4225	0.58	0.22
rs11170567	7786	MAP3K12	12	52195106	2.64E-01	11090	1.71E-01	Down	1.37	7930	0.58	0.08
rs747004	80155	NARG1	4	140612123	2.64E-01	11091	1.82E-01	Up	1.34	8043	0.58	0.07
rs3745367	126003	TRAPPC5	19	7640511	2.65E-01	11092	8.56E-02	Up	1.72	6729	0.58	0.11

rs3745367	56729	RETN	19	7640511	2.65E-01	11093	4.60E-01	Down	0.74	10428	0.58	0.03
rs12045477	2980	GUCA2A	1	42307256	2.65E-01	11094	2.52E-01	Up	1.15	8780	0.58	0.06
rs12045807	2029	ENSA	1	147397907	2.65E-01	11095	1.56E-01	Up	1.42	7749	0.58	0.08
rs11853191	80349	WDR61	15	76352896	2.65E-01	11096	3.12E-01	Up	1.01	9288	0.58	0.05
rs10121	2877	GPX2	14	64482526	2.65E-01	11097	9.15E-15	Down	7.75	405	0.58	1.40
rs2329020	8927	BSN	3	49660077	2.65E-01	11098	2.23E-02	Down	2.29	5174	0.58	0.17
rs16876489	23484	LEPROTL1	8	30090832	2.65E-01	11099	1.37E-03	Down	3.20	3341	0.58	0.29
rs649937	283209	PGM2L1	11	73713183	2.65E-01	11100	6.11E-02	Up	1.87	6260	0.58	0.12
rs1065206	1212	CLTB	5	175743839	2.65E-01	11101	2.85E-09	Down	5.94	964	0.58	0.85
rs1065206	192286	HIGD2A	5	175743839	2.65E-01	11102	8.17E-01	Down	0.23	12771	0.58	0.01
rs2302832	9870	KIAA0317	14	74207417	2.65E-01	11103	5.86E-04	Up	3.44	3019	0.58	0.32
rs6048205	3170	FOXA2	20	22507601	2.65E-01	11104	9.31E-01	Up	0.09	13443	0.58	0.00
rs10488500	10875	FGL2	7	76485910	2.65E-01	11105	1.55E-01	Down	1.42	7741	0.58	0.08
rs11668660	162968	ZNF497	19	63565396	2.65E-01	11106	7.48E-01	Down	0.32	12362	0.58	0.01
rs11668660	1	A1BG	19	63565396	2.65E-01	11107	9.04E-01	Up	0.12	13273	0.58	0.00
rs7929406	54979	HRASLS2	11	63101778	2.65E-01	11108	3.08E-01	Down	1.02	9249	0.58	0.05
rs12756745	2326	FMO1	1	167961166	2.65E-01	11109	2.71E-01	Up	1.10	8963	0.58	0.06
rs12840611	7592	ZNF41		47109248	2.66E-01	11110	1.62E-02	Up	2.40	4900	0.58	0.18
rs1154454	131	ADH7	4	100695520	2.66E-01	11111	3.64E-01	Up	0.91	9714	0.58	0.04
rs602609	54843	SYTL2	11	85077332	2.66E-01	11112	2.89E-02	Up	2.18	5421	0.58	0.15
rs602609	220388	CCDC89	11	85077332	2.66E-01	11113	4.18E-01	Up	0.81	10109	0.58	0.04
rs571413	143684	FAM76B	11	95166236	2.66E-01	11114	5.87E-03	Up	2.76	4162	0.58	0.22
rs3740078	57089	ENTPD7	10	101435581	2.66E-01	11115	2.06E-01	Up	1.27	8313	0.58	0.07
rs2855039	3045	HBD	11	5228247	2.66E-01	11116	2.19E-01	Down	1.23	8449	0.58	0.07
rs2855039	3043	HBB	11	5228247	2.66E-01	11117	2.42E-01	Down	1.17	8679	0.58	0.06
rs2855039	3047	HBG1	11	5228247	2.66E-01	11118	3.08E-01	Up	1.02	9254	0.58	0.05
rs2855039	3048	HBG2	11	5228247	2.66E-01	11119	4.69E-01	Up	0.72	10496	0.58	0.03
rs2855039	3044	HBBP1	11	5228247	2.66E-01	11120	9.87E-01	Up	0.02	13825	0.58	0.00
rs4365560	200315	APOBEC3A	22	37679609	2.66E-01	11121	2.10E-01	Up	1.25	8352	0.58	0.07
rs1374912	60675	PROK2	3	71894565	2.66E-01	11122	4.91E-01	Up	0.69	10660	0.58	0.03
rs5931049	83550	GPR101		135855038	2.66E-01	11123	4.81E-01	Up	0.70	10579	0.58	0.03
rs932998	10541	ANP32B	9	97828151	2.66E-01	11124	7.69E-01	Down	0.29	12486	0.58	0.01
rs7114704	8567	MADD	11	47250033	2.66E-01	11125	1.29E-01	Down	1.52	7437	0.57	0.09
rs7114704	10062	NR1H3	11	47250033	2.66E-01	11126	2.33E-01	Down	1.19	8591	0.57	0.06
rs1806440	4585	MUC4	3	197005432	2.67E-01	11127	1.91E-04	Down	3.73	2618	0.57	0.37
rs9848745	64110	MAGEF1	3	185932250	2.67E-01	11128	4.78E-02	Down	1.98	5944	0.57	0.13
rs16983414	9668	ZNF432	19	57262595	2.67E-01	11129	4.83E-03	Up	2.82	4038	0.57	0.23
rs2920022	5908	RAP1B	12	67326322	2.67E-01	11130	2.43E-05	Down	4.22	2124	0.57	0.46
rs11658169	11216	AKAP10	17	19812514	2.67E-01	11131	6.89E-01	Down	0.40	11998	0.57	0.02
rs3738593	57111	RAB25	1	152859046	2.67E-01	11132	1.07E-05	Up	4.40	1962	0.57	0.50
rs13244770	135886	WBSCR28	7	72725342	2.67E-01	11133	1.33E-01	Down	1.50	7476	0.57	0.09
rs756014	6305	SBF1	22	49174105	2.67E-01	11134	5.93E-01	Down	0.53	11388	0.57	0.02
rs8178847	350	APOH	17	61647277	2.67E-01	11135	9.21E-01	Up	0.10	13382	0.57	0.00
rs882390	5775	PTPN4	2	120415972	2.67E-01	11136	6.04E-01	Down	0.52	11464	0.57	0.02
rs479658	85458	DIXDC1	11	111361622	2.67E-01	11137	3.80E-10	Down	6.26	825	0.57	0.94
rs1838173	3228	HOXC12	12	52629089	2.68E-01	11138	5.53E-01	Up	0.59	11098	0.57	0.03
rs17015309	27042	C1orf107	1	206395613	2.68E-01	11139	9.50E-03	Up	2.59	4471	0.57	0.20
rs1345151	51646	YPEL5	2	30286837	2.68E-01	11140	4.95E-05	Down	4.06	2283	0.57	0.43
rs12156640	3442	IFNA5	9	21296241	2.68E-01	11141	2.57E-02	Up	2.23	5312	0.57	0.16
rs6035613	3642	INSM1	20	20304386	2.68E-01	11142	5.95E-22	Up	9.61	162	0.57	2.12
rs12621441	8837	CFLAR	2	201794446	2.68E-01	11143	1.52E-01	Down	1.43	7706	0.57	0.08
rs180326	84811	BUD13	11	116129913	2.68E-01	11144	1.13E-02	Down	2.53	4595	0.57	0.19
rs2216054	85407	NKD1	16	49158378	2.68E-01	11145	7.42E-02	Down	1.79	6511	0.57	0.11
rs3118229	1001	CDH3	16	67267666	2.68E-01	11146	4.03E-04	Down	3.54	2888	0.57	0.34
rs7507442	162966	ZNF600	19	57970765	2.68E-01	11147	4.91E-03	Up	2.81	4054	0.57	0.23
rs17082035	9107	MTMR6	13	24721451	2.68E-01	11148	2.71E-01	Down	1.10	8962	0.57	0.06
rs1077216	4634	MYL3	3	46867165	2.68E-01	11149	6.97E-01	Down	0.39	12052	0.57	0.02
rs2079063	7757	ZNF208	19	21952514	2.69E-01	11150	5.46E-01	Up	0.60	11054	0.57	0.03
rs7631506	57337	SENP7	3	102725304	2.69E-01	11151	2.27E-01	Up	1.21	8525	0.57	0.06
rs10512687	3977	LIFR	5	38617019	2.69E-01	11152	6.50E-01	Up	0.45	11770	0.57	0.02
rs11781564	3297	HSF1	8	145466386	2.69E-01	11153	1.93E-01	Down	1.30	8184	0.57	0.07
rs17065164	144811	C13orf31	13	43342706	2.69E-01	11154	8.93E-04	Down	3.32	3174	0.57	0.30
rs17065164	160857	CCDC122	13	43342706	2.69E-01	11155	6.79E-01	Down	0.41	11941	0.57	0.02
rs6673081	127579	DCST2	1	151802668	2.70E-01	11156	8.95E-01	Down	0.13	13215	0.57	0.00
rs492939	51347	TAOK3	12	117291475	2.70E-01	11157	6.08E-07	Up	4.99	1505	0.57	0.62
rs492939	64426	SUDS3	12	117291475	2.70E-01	11158	8.39E-03	Up	2.64	4374	0.57	0.21
rs7395319	10410	IFITM3	11	321326	2.70E-01	11159	7.16E-16	Down	8.07	355	0.57	1.51
rs7061470	84295	PHF6		133276246	2.70E-01	11160	1.76E-01	Down	1.35	7973	0.57	0.08
rs2032918	23035	PHLPPL	16	70260937	2.70E-01	11161	9.87E-04	Down	3.29	3218	0.57	0.30
rs1043313	10020	GNE	9	36204971	2.70E-01	11162	2.24E-03	Up	3.06	3606	0.57	0.27
rs1043313	1211	CLTA	9	36204971	2.70E-01	11163	8.86E-03	Up	2.62	4423	0.57	0.21
rs7119	10363	HMG20A	15	75564687	2.70E-01	11164	5.26E-02	Down	1.94	6052	0.57	0.13
rs2287214	11137	PWP1	12	106592985	2.70E-01	11165	5.29E-01	Up	0.63	10933	0.57	0.03
rs6962019	223117	SEMA3D	7	84352576	2.70E-01	11166	3.47E-04	Down	3.58	2830	0.57	0.35
rs6798807	131474	CHCHD4	3	14147076	2.70E-01	11167	5.18E-01	Down	0.65	10845	0.57	0.03
rs8066529	29916	SNX11	17	43542564	2.70E-01	11168	6.12E-02	Down	1.87	6263	0.57	0.12
rs7129065	51700	CYB5R2	11	7635444	2.70E-01	11169	8.43E-04	Down	3.34	3152	0.57	0.31
rs3886478	27077	B9D1	17	19211040	2.70E-01	11170	1.60E-01	Up	1.41	7810	0.57	0.08
rs3886478	5598	MAPK7	17	19211040	2.70E-01	11171	5.68E-01	Down	0.57	11224	0.57	0.02
rs3745469	3661	IRF3	19	54868983	2.71E-01	11172	7.70E-01	Down	0.29	12498	0.57	0.01
rs1470625	1329	COX5B	2	97724009	2.71E-01	11173	1.23E-03	Up	3.23	3301	0.57	0.29

rs11170795	3221	HOXC4	12	52743962	2.71E-01	11174	4.50E-02	Up	2.00	5875	0.57	0.13
rs13299805	2800	GOLGA1	9	124789866	2.71E-01	11175	1.14E-02	Up	2.53	4605	0.57	0.19
rs13299805	286205	C9orf126	9	124789866	2.71E-01	11176	8.38E-01	Down	0.20	12894	0.57	0.01
rs3814803	8900	CCNA1	13	35902964	2.71E-01	11177	1.83E-01	Down	1.33	8057	0.57	0.07
rs10781329	5568	PRKACG	9	68847799	2.71E-01	11178	2.69E-01	Down	1.11	8945	0.57	0.06
rs3792252	64080	RBKS	2	27907582	2.71E-01	11179	8.38E-05	Up	3.93	2407	0.57	0.41
rs3792252	9553	MRPL33	2	27907582	2.71E-01	11180	2.74E-01	Down	1.09	8990	0.57	0.06
rs478941	1737	DLAT	11	111409205	2.71E-01	11181	1.96E-02	Up	2.33	5052	0.57	0.17
rs9928269	79412	KREMEN2	16	2938912	2.71E-01	11182	4.78E-01	Down	0.71	10558	0.57	0.03
rs16845288	3512	IGJ	4	71879540	2.71E-01	11183	7.24E-01	Down	0.35	12227	0.57	0.01
rs10754377	4808	NHLH2	1	116092214	2.71E-01	11184	2.28E-02	Up	2.28	5189	0.57	0.16
rs10883438	8904	CPNE1	10	101816676	2.72E-01	11185	1.14E-03	Up	3.25	3273	0.57	0.29
rs10883438	1584	CYP11B1	10	101816676	2.72E-01	11186	1.81E-02	Up	2.36	4991	0.57	0.17
rs10883438	1369	CPN1	10	101816676	2.72E-01	11187	6.79E-01	Down	0.41	11939	0.57	0.02
rs4074838	55063	ZCWPW1	7	99677316	2.72E-01	11188	2.17E-02	Down	2.30	5147	0.57	0.17
rs4074838	221908	C7orf47	7	99677316	2.72E-01	11189	3.23E-02	Up	2.14	5534	0.57	0.15
rs4074838	56257	MEPCE	7	99677316	2.72E-01	11190	5.18E-01	Up	0.65	10844	0.57	0.03
rs1601717	259240	WFDC9	20	43667838	2.72E-01	11191	3.12E-01	Up	1.01	9291	0.57	0.05
rs2131109	327	APEH	3	49679991	2.72E-01	11192	7.64E-06	Up	4.47	1892	0.57	0.51
rs4880094	29952	DPP7	9	137293975	2.72E-01	11193	2.59E-04	Up	3.65	2723	0.57	0.36
rs4880094	114787	GPRIN1	9	137293975	2.72E-01	11194	3.26E-02	Down	2.14	5546	0.57	0.15
rs4880094	2902	GRIN1	9	137293975	2.72E-01	11195	5.35E-01	Up	0.62	10977	0.57	0.03
rs6965510	5166	PDK4	7	94874499	2.72E-01	11196	1.85E-05	Down	4.28	2079	0.57	0.47
rs5909662	4694	NDUFA1		118772265	2.72E-01	11197	6.73E-02	Up	1.83	6381	0.57	0.12
rs10794580	80007	C10orf88	10	124687188	2.72E-01	11198	4.85E-03	Up	2.82	4043	0.57	0.23
rs4660293	8761	PABPC4	1	39697273	2.72E-01	11199	5.23E-10	Up	6.20	862	0.57	0.93
rs8102979	5639	PRRG2	19	54761706	2.72E-01	11200	7.68E-02	Up	1.77	6570	0.57	0.11
rs8102979	51070	NOSIP	19	54761706	2.72E-01	11201	2.74E-01	Down	1.09	8984	0.57	0.06
rs4148974	4731	NDUFV3	21	43196789	2.72E-01	11202	4.30E-03	Up	2.86	3979	0.57	0.24
rs1558333	55695	NSUN5	12	6545316	2.72E-01	11203	2.77E-03	Up	2.99	3745	0.57	0.26
rs3934594	286223	C9orf47	9	88840097	2.73E-01	11204	2.14E-08	Down	5.60	1133	0.56	0.77
rs10520260	9464	HAND2	4	174822079	2.73E-01	11205	6.17E-01	Down	0.50	11553	0.56	0.02
rs17025827	56888	KCMF1	2	85186324	2.73E-01	11206	4.88E-03	Down	2.81	4051	0.56	0.23
rs545226	1030	CDKN2B	9	22002422	2.73E-01	11207	6.80E-01	Up	0.41	11951	0.56	0.02
rs11073332	10125	RASGRP1	15	36604906	2.73E-01	11208	7.30E-01	Up	0.35	12249	0.56	0.01
rs12276758	3263	HPX	11	6407473	2.73E-01	11209	5.95E-02	Up	1.88	6227	0.56	0.12
rs7649147	3280	HES1	3	195325284	2.73E-01	11210	5.21E-01	Down	0.64	10868	0.56	0.03
rs9333016	1579	CYP4A11	1	47111054	2.74E-01	11211	2.13E-03	Up	3.07	3570	0.56	0.27
rs4242396	8797	TNFRSF10A	8	23156852	2.74E-01	11212	2.29E-01	Up	1.20	8555	0.56	0.06
rs11811735	81875	ISG20L2	1	153502554	2.74E-01	11213	1.93E-01	Up	1.30	8183	0.56	0.07
rs11811735	51093	C1orf66	1	153502554	2.74E-01	11214	8.58E-01	Down	0.18	13003	0.56	0.01
rs2339524	84329	HVCN1	12	109579375	2.74E-01	11215	2.57E-01	Down	1.13	8829	0.56	0.06
rs9938547	80063	ATF7IP2	16	10485162	2.74E-01	11216	4.06E-02	Down	2.05	5762	0.56	0.14
rs4865414	23142	DCUN1D4	4	52599222	2.74E-01	11217	4.06E-14	Down	7.56	447	0.56	1.34
rs12945695	3965	LGALS9	17	22980475	2.74E-01	11218	1.64E-01	Down	1.39	7858	0.56	0.08
rs9654749	6804	STX1A	7	72585037	2.74E-01	11219	9.51E-01	Down	0.06	13562	0.56	0.00
rs10863888	7188	TRAF5	1	207891164	2.75E-01	11220	2.08E-07	Down	5.19	1377	0.56	0.67
rs16973746	5930	RBBP6	16	24445287	2.75E-01	11221	7.01E-03	Up	2.70	4262	0.56	0.22
rs2363863	11024	LILRA1	19	59803597	2.75E-01	11222	9.65E-01	Up	0.04	13652	0.56	0.00
rs11763150	23386	NUDCD3	7	44228882	2.75E-01	11223	6.20E-03	Down	2.74	4186	0.56	0.22
rs11957276	166968	MIER3	5	56257877	2.75E-01	11224	9.10E-01	Up	0.11	13308	0.56	0.00
rs4792750	388341	C17orf76	17	16299739	2.75E-01	11225	3.88E-01	Up	0.86	9903	0.56	0.04
rs1325511	80263	TRIM45	1	117370808	2.75E-01	11226	1.87E-01	Down	1.32	8111	0.56	0.07
rs2306595	284098	PIGW	17	31945834	2.76E-01	11227	4.24E-02	Up	2.03	5812	0.56	0.14
rs6091236	23394	ADNP	20	48950402	2.76E-01	11228	9.23E-04	Up	3.31	3183	0.56	0.30
rs2543659	79843	FAM124B	2	225080519	2.76E-01	11229	9.93E-01	Down	0.01	13869	0.56	0.00
rs2846412	23086	EXPH5	11	107889417	2.76E-01	11230	2.69E-01	Up	1.10	8946	0.56	0.06
rs1487328	140832	WFDC10A	20	43681107	2.76E-01	11231	6.98E-01	Up	0.39	12054	0.56	0.02
rs11167280	51282	SCAND1	20	34023940	2.76E-01	11232	2.08E-04	Up	3.71	2646	0.56	0.37
rs645078	8986	RPS6KA4	11	63891874	2.76E-01	11233	5.25E-04	Up	3.47	2974	0.56	0.33
rs1329126	359948	IRF2BP2	1	231047284	2.76E-01	11234	8.01E-03	Down	2.65	4343	0.56	0.21
rs877396	143379	C10orf82	10	118419113	2.76E-01	11235	1.45E-04	Down	3.80	2540	0.56	0.38
rs10814059	55833	UBAP2	9	33998406	2.76E-01	11236	1.30E-03	Up	3.22	3326	0.56	0.29
rs1473601	9650	MTFR1	8	66709080	2.76E-01	11237	1.50E-02	Up	2.43	4814	0.56	0.18
rs3856982	57654	KIAA1530	4	1318693	2.76E-01	11238	1.50E-08	Up	5.67	1086	0.56	0.78
rs8068796	64118	DUS1L	17	77615650	2.76E-01	11239	4.02E-09	Up	5.88	993	0.56	0.84
rs8068796	2873	GPS1	17	77615650	2.76E-01	11240	1.76E-05	Up	4.29	2067	0.56	0.48
rs2014704	6605	SMARCE1	17	36061775	2.76E-01	11241	5.23E-01	Up	0.64	10884	0.56	0.03
rs6586261	10785	WDR4	21	43191838	2.77E-01	11242	6.33E-02	Up	1.86	6306	0.56	0.12
rs3759270	3824	KLRD1	12	10350458	2.77E-01	11243	2.87E-02	Up	2.19	5414	0.56	0.15
rs1854779	3713	IVL	1	149693745	2.77E-01	11244	3.93E-01	Down	0.85	9931	0.56	0.04
rs13168712	5370	PMCHL2	5	70715382	2.77E-01	11245	9.76E-01	Down	0.03	13736	0.56	0.00
rs10092842	27012	KCNV1	8	111059332	2.77E-01	11246	1.75E-01	Up	1.36	7964	0.56	0.08
rs3795820	54879	ST7L	1	112875084	2.77E-01	11247	1.78E-01	Down	1.35	8002	0.56	0.07
rs2376997	598	BCL2L1	20	29782860	2.77E-01	11248	6.11E-01	Up	0.51	11513	0.56	0.02
rs12056725	83482	SCRT1	8	145546295	2.77E-01	11249	8.17E-01	Up	0.23	12769	0.56	0.01
rs12056725	26233	FBXL6	8	145546295	2.77E-01	11250	9.88E-01	Up	0.02	13830	0.56	0.00
rs2254266	818	CAMK2G	10	75271694	2.77E-01	11251	1.71E-20	Down	9.28	188	0.56	1.98
rs11194976	120	ADD3	10	111834306	2.77E-01	11252	1.57E-14	Down	7.68	424	0.56	1.38
rs9378127	6046	BRD2	6	33030437	2.77E-01	11253	1.08E-10	Up	6.46	756	0.56	1.00
rs9378127	3108	HLA-DMA	6	33030437	2.77E-01	11254	5.36E-02	Up	1.93	6078	0.56	0.13

rs12767760	80019	UBTD1	10	99284787	2.77E-01	11255	7.67E-02	Down	1.77	6567	0.56	0.11
rs2302178	9143	SYNGR3	16	1961894	2.77E-01	11256	1.18E-01	Up	1.56	7266	0.56	0.09
rs2302178	124056	NOXO1	16	1961894	2.77E-01	11257	7.90E-01	Down	0.27	12608	0.56	0.01
rs10491118	6359	CCL15	17	31338999	2.77E-01	11258	4.60E-02	Up	2.00	5894	0.56	0.13
rs940904	57605	PITPNM2	12	122016452	2.77E-01	11259	2.36E-01	Down	1.19	8621	0.56	0.06
rs10279462	11185	INMT	7	30557934	2.77E-01	11260	4.66E-07	Down	5.04	1472	0.56	0.63
rs330843	59336	PRDM13	6	100167482	2.78E-01	11261	2.62E-01	Up	1.12	8882	0.56	0.06
rs4783573	999	CDH1	16	67398089	2.78E-01	11262	1.13E-03	Up	3.26	3268	0.56	0.29
rs7836875	11156	PTP4A3	8	142512371	2.78E-01	11263	2.31E-11	Up	6.73	664	0.56	1.06
rs4141075	2861	GPR37	7	124004528	2.78E-01	11264	4.98E-03	Up	2.81	4069	0.56	0.23
rs12945599	6442	SGCA	17	45610182	2.78E-01	11265	2.84E-03	Down	2.98	3755	0.56	0.25
rs12945599	373861	HILS1	17	45610182	2.78E-01	11266	5.82E-01	Down	0.55	11328	0.56	0.02
rs2899826	84993	UBL7	15	72521553	2.78E-01	11267	1.91E-01	Up	1.31	8159	0.56	0.07
rs2841512	23408	SIRT5	6	13695959	2.78E-01	11268	5.16E-01	Down	0.65	10829	0.56	0.03
rs2299956	9569	GTF2IRD1	7	73367961	2.78E-01	11269	1.07E-01	Up	1.61	7114	0.56	0.10
rs902726	3396	ICT1	17	70527851	2.78E-01	11270	3.26E-07	Up	5.11	1427	0.56	0.65
rs6616910	27330	RPS6KA6		83227075	2.78E-01	11271	5.45E-01	Up	0.60	11052	0.56	0.03
rs2613843	29946	SERTAD3	19	45661043	2.78E-01	11272	8.16E-01	Down	0.23	12760	0.56	0.01
rs9673242	57496	MKL2	16	14078070	2.78E-01	11273	2.72E-05	Down	4.20	2150	0.56	0.46
rs2285487	11093	ADAMTS13	9	133307036	2.78E-01	11274	9.13E-01	Up	0.11	13336	0.56	0.00
rs2285487	169436	C9orf96	9	133307036	2.78E-01	11275	9.27E-01	Down	0.09	13409	0.56	0.00
rs157386	9706	ULK2	17	19639713	2.79E-01	11276	5.27E-02	Down	1.94	6058	0.56	0.13
rs11920251	7375	USP4	3	49331675	2.79E-01	11277	9.81E-02	Up	1.65	6965	0.55	0.10
rs6754561	65080	MRPL44	2	224665201	2.79E-01	11278	1.18E-01	Up	1.56	7265	0.55	0.09
rs6952793	23480	SEC61G	7	54594395	2.79E-01	11279	3.09E-10	Up	6.29	814	0.55	0.95
rs2078486	7157	TP53	17	7523808	2.79E-01	11280	8.79E-03	Up	2.62	4411	0.55	0.21
rs11671781	58510	PRODH2	19	40987215	2.79E-01	11281	1.90E-01	Down	1.31	8150	0.55	0.07
rs1709128	1572	CYP2F1	19	46334906	2.79E-01	11282	5.95E-01	Up	0.53	11404	0.55	0.02
rs1888274	26268	FBXO9	6	53040467	2.79E-01	11283	4.75E-01	Up	0.71	10538	0.55	0.03
rs7689475	55296	TBC1D19	4	26442956	2.79E-01	11284	9.44E-01	Up	0.07	13518	0.55	0.00
rs11213821	341032	C11orf53	11	110669861	2.80E-01	11285	1.71E-02	Down	2.39	4941	0.55	0.18
rs3890727	55361	PI4K2A	10	99404192	2.80E-01	11286	2.93E-01	Down	1.05	9140	0.55	0.05
rs178467	516	ATP5G1	17	44305868	2.80E-01	11287	3.48E-08	Up	5.52	1176	0.55	0.75
rs1548476	162972	ZNF550	19	62750348	2.80E-01	11288	1.07E-02	Up	2.55	4559	0.55	0.20
rs1031551	8836	GGH	8	64126401	2.80E-01	11289	6.60E-02	Up	1.84	6361	0.55	0.12
rs1031551	7274	TTPA	8	64126401	2.80E-01	11290	8.81E-01	Up	0.15	13134	0.55	0.01
rs3803120	11108	PRDM4	12	106642499	2.80E-01	11291	7.17E-01	Up	0.36	12178	0.55	0.01
rs1787824	147323	STARD6	18	50137100	2.80E-01	11292	9.64E-01	Down	0.04	13645	0.55	0.00
rs4824747	89801	PPP1R3F		48885394	2.80E-01	11293	4.97E-01	Up	0.68	10689	0.55	0.03
rs4824747	50943	FOXP3		48885394	2.80E-01	11294	5.91E-01	Down	0.54	11380	0.55	0.02
rs2284923	9025	RNF8	6	37456898	2.81E-01	11295	5.57E-05	Down	4.03	2314	0.55	0.43
rs2066557	8847	DLEU2	13	49508271	2.81E-01	11296	8.37E-03	Up	2.64	4373	0.55	0.21
rs2286806	23531	MMD	17	50840185	2.81E-01	11297	9.34E-04	Down	3.31	3185	0.55	0.30
rs948414	79102	RNF26	11	118720008	2.81E-01	11298	4.29E-02	Down	2.03	5822	0.55	0.14
rs6141422	84905	ZNF341	20	31805940	2.81E-01	11299	7.59E-01	Down	0.31	12437	0.55	0.01
rs740059	10635	RAD51AP1	12	4541751	2.81E-01	11300	1.86E-01	Up	1.32	8095	0.55	0.07
rs1296028	2222	FDFT1	8	11736156	2.81E-01	11301	1.99E-13	Down	7.35	493	0.55	1.27
rs1866146	5443	POMC	2	25292224	2.81E-01	11302	2.68E-04	Up	3.64	2738	0.55	0.36
rs2287179	57798	GATAD1	7	91725046	2.81E-01	11303	8.94E-01	Up	0.13	13213	0.55	0.00
rs11636338	597	BCL2A1	15	78032645	2.81E-01	11304	9.33E-02	Up	1.68	6890	0.55	0.10
rs3846281	84992	PIGY	4	89808687	2.82E-01	11305	2.55E-01	Up	1.14	8808	0.55	0.06
rs4227	6665	SOX15	17	7431901	2.82E-01	11306	2.80E-03	Down	2.99	3750	0.55	0.26
rs3788756	23676	SMPX		21522578	2.82E-01	11307	7.79E-01	Down	0.28	12546	0.55	0.01
rs17165184	51015	ISOC1	5	128470847	2.82E-01	11308	4.17E-05	Up	4.10	2237	0.55	0.44
rs4788102	7284	TUFM	16	28780899	2.82E-01	11309	5.19E-03	Up	2.80	4093	0.55	0.23
rs4788102	487	ATP2A1	16	28780899	2.82E-01	11310	3.50E-01	Down	0.94	9584	0.55	0.05
rs4788102	25970	SH2B1	16	28780899	2.82E-01	11311	7.21E-01	Up	0.36	12203	0.55	0.01
rs7513934	200014	CC2D1B	1	52530209	2.82E-01	11312	9.32E-01	Down	0.08	13449	0.55	0.00
rs2159214	4066	LYL1	19	13092995	2.82E-01	11313	3.51E-01	Down	0.93	9603	0.55	0.05
rs343419	9695	EDEM1	3	5207074	2.82E-01	11314	4.76E-09	Up	5.86	1005	0.55	0.83
rs2676790	11248	NXPH3	17	45017682	2.82E-01	11315	1.12E-01	Down	1.59	7171	0.55	0.10
rs3025646	2550	GABBR1	6	29674821	2.82E-01	11316	4.24E-02	Down	2.03	5810	0.55	0.14
rs2235218	51499	TRIAP1	12	119351339	2.83E-01	11317	2.06E-03	Up	3.08	3551	0.55	0.27
rs4247000	115817	DHRS1	14	23840977	2.83E-01	11318	4.26E-01	Down	0.80	10160	0.55	0.04
rs4247000	1241	LTB4R	14	23840977	2.83E-01	11319	4.89E-01	Up	0.69	10652	0.55	0.03
rs4247000	56413	LTB4R2	14	23840977	2.83E-01	11320	7.12E-01	Up	0.37	12147	0.55	0.01
rs4247000	161424	C14orf21	14	23840977	2.83E-01	11321	8.49E-01	Up	0.19	12950	0.55	0.01
rs1208606	219749	ZNF25	10	38281073	2.83E-01	11322	3.06E-04	Down	3.61	2782	0.55	0.35
rs1451240	1142	CHRNB3	8	42665868	2.83E-01	11323	3.96E-03	Up	2.88	3922	0.55	0.24
rs861171	27032	ATP2C1	3	132131604	2.83E-01	11324	6.91E-13	Up	7.18	541	0.55	1.22
rs7176592	90417	C15orf23	15	38464289	2.83E-01	11325	1.06E-06	Up	4.88	1583	0.55	0.60
rs4287627	3000	GUCY2D	17	7873513	2.83E-01	11326	3.74E-01	Up	0.89	9799	0.55	0.04
rs7007	65005	MRPL9	1	148548649	2.83E-01	11327	2.38E-02	Down	2.26	5236	0.55	0.16
rs7007	11022	TDRKH	1	148548649	2.83E-01	11328	3.80E-02	Up	2.07	5705	0.55	0.14
rs7007	51686	OAZ3	1	148548649	2.83E-01	11329	3.67E-01	Down	0.90	9737	0.55	0.04
rs3811958	79614	C5orf23	5	32807800	2.83E-01	11330	2.70E-01	Up	1.10	8953	0.55	0.06
rs2731664	2870	GRK6	5	176792454	2.84E-01	11331	7.54E-01	Up	0.31	12406	0.55	0.01
rs8070792	57597	BAHCC1	17	76979761	2.84E-01	11332	9.48E-01	Up	0.07	13538	0.55	0.00
rs1233454	7770	ZNF227	19	49433667	2.84E-01	11333	2.77E-03	Up	2.99	3747	0.55	0.26
rs1543572	140732	SPAG4L	20	31056677	2.84E-01	11334	3.40E-02	Up	2.12	5592	0.55	0.15
rs1543572	80341	BPIL1	20	31056677	2.84E-01	11335	5.15E-02	Up	1.95	6025	0.55	0.13

rs3782360	27289	RND1	12	47543059	2.84E-01	11336	1.60E-01	Up	1.40	7816	0.55	0.08
rs3782360	9416	DDX23	12	47543059	2.84E-01	11337	2.54E-01	Down	1.14	8801	0.55	0.06
rs9907177	30850	CDR2L	17	70518591	2.84E-01	11338	1.95E-01	Up	1.29	8207	0.55	0.07
rs1905045	54968	TMEM70	8	75062568	2.84E-01	11339	4.49E-02	Up	2.01	5872	0.55	0.13
rs1905045	23643	LY96	8	75062568	2.84E-01	11340	5.89E-01	Up	0.54	11362	0.55	0.02
rs2301249	79748	LMAN1L	15	72879437	2.84E-01	11341	7.80E-02	Down	1.76	6593	0.55	0.11
rs1050483	8625	RFXANK	19	19164802	2.84E-01	11342	6.08E-01	Up	0.51	11488	0.55	0.02
rs708692	4302	MLLT6	17	34144475	2.84E-01	11343	3.11E-01	Up	1.01	9284	0.55	0.05
rs4977177	8733	GPAA1	8	145195417	2.84E-01	11344	2.24E-13	Up	7.33	498	0.55	1.26
rs4977177	54512	EXOSC4	8	145195417	2.84E-01	11345	8.06E-05	Up	3.94	2392	0.55	0.41
rs4977177	26873	OPLAH	8	145195417	2.84E-01	11346	2.03E-02	Up	2.32	5086	0.55	0.17
rs9932251	4775	NFATC3	16	66689233	2.84E-01	11347	1.38E-04	Up	3.81	2524	0.55	0.39
rs6521861	85417	CCNB3		49892875	2.84E-01	11348	3.11E-01	Down	1.01	9280	0.55	0.05
rs1954874	130013	ACMSD	2	135428431	2.84E-01	11349	6.86E-02	Up	1.82	6402	0.55	0.12
rs587263	7379	UPK2	11	118321366	2.85E-01	11350	7.75E-03	Down	2.66	4323	0.55	0.21
rs17752670	85377	MICALL1	22	36607397	2.85E-01	11351	5.82E-05	Down	4.02	2324	0.55	0.42
rs2273866	254295	PHYHD1	9	128782445	2.85E-01	11352	4.51E-01	Down	0.75	10349	0.54	0.03
rs2111705	56252	YLPM1	14	74331394	2.85E-01	11353	9.65E-03	Up	2.59	4482	0.54	0.20
rs923828	65012	SLC26A10	12	56301761	2.85E-01	11354	3.56E-01	Down	0.92	9641	0.54	0.04
rs10197534	4118	MAL	2	95093621	2.85E-01	11355	1.45E-11	Down	6.75	652	0.54	1.08
rs2335534	57333	RCN3	19	54706789	2.86E-01	11356	8.47E-02	Down	1.72	6716	0.54	0.11
rs2682338	3856	KRT8	12	51601000	2.86E-01	11357	5.41E-02	Up	1.93	6094	0.54	0.13
rs6752623	3239	HOXD13	2	176776433	2.86E-01	11358	9.11E-04	Down	3.32	3180	0.54	0.30
rs11933187	26269	FBXO8	4	175584662	2.86E-01	11359	3.13E-02	Down	2.15	5510	0.54	0.15
rs4081877	80148	PQLC1	18	75822113	2.86E-01	11360	3.28E-01	Up	0.98	9425	0.54	0.05
rs650298	54455	FBXO42	1	16410914	2.86E-01	11361	6.31E-01	Down	0.48	11650	0.54	0.02
rs12603190	2837	UTS2R	17	77917676	2.86E-01	11362	4.23E-02	Up	2.03	5806	0.54	0.14
rs10911676	6045	RNF2	1	181803488	2.86E-01	11363	1.51E-05	Up	4.33	2039	0.54	0.48
rs1151625	54923	LIME1	20	61840441	2.86E-01	11364	9.48E-04	Down	3.31	3196	0.54	0.30
rs1151625	56731	SLC2A4RG	20	61840441	2.86E-01	11365	1.03E-01	Up	1.63	7040	0.54	0.10
rs1243439	57447	NDRG2	14	20550283	2.86E-01	11366	2.55E-05	Down	4.21	2139	0.54	0.46
rs11205341	81611	ANP32E	1	146996133	2.86E-01	11367	8.90E-14	Down	7.46	468	0.54	1.31
rs2290771	23558	WBP2	17	71356343	2.87E-01	11368	1.78E-01	Down	1.35	7992	0.54	0.07
rs2290771	201294	UNC13D	17	71356343	2.87E-01	11369	2.34E-01	Down	1.19	8600	0.54	0.06
rs4982180	171546	C14orf147	14	33991651	2.87E-01	11370	2.07E-04	Down	3.71	2644	0.54	0.37
rs342015	375484	C5orf25	5	175683091	2.87E-01	11371	8.23E-03	Down	2.64	4362	0.54	0.21
rs1004783	91833	WDR20	14	101739765	2.87E-01	11372	1.32E-02	Up	2.48	4702	0.54	0.19
rs279545	78987	CRELD1	3	9947493	2.87E-01	11373	1.40E-02	Up	2.46	4755	0.54	0.19
rs279545	84818	IL17RC	3	9947493	2.87E-01	11374	1.29E-01	Down	1.52	7438	0.54	0.09
rs4471211	9939	RBM8A	1	143006605	2.87E-01	11375	8.80E-01	Down	0.15	13125	0.54	0.01
rs10783815	11247	NXPH4	12	55902280	2.87E-01	11376	7.27E-01	Down	0.35	12235	0.54	0.01
rs12041331	375033	PEAR1	1	153682787	2.87E-01	11377	5.64E-01	Down	0.58	11196	0.54	0.02
rs13155119	54439	RBM27	5	145622200	2.87E-01	11378	1.39E-01	Down	1.48	7564	0.54	0.09
rs359974	3549	IHH	2	219755696	2.87E-01	11379	2.60E-01	Up	1.13	8860	0.54	0.06
rs12831884	2065	ERBB3	12	54752395	2.87E-01	11380	8.71E-19	Up	8.84	239	0.54	1.81
rs10413359	4680	CEACAM6	19	46962846	2.88E-01	11381	2.99E-02	Down	2.17	5461	0.54	0.15
rs7038280	20	ABCA2	9	137209081	2.88E-01	11382	3.94E-03	Up	2.88	3919	0.54	0.24
rs7038280	56654	NPDC1	9	137209081	2.88E-01	11383	9.19E-02	Up	1.69	6860	0.54	0.10
rs7038280	2529	FUT7	9	137209081	2.88E-01	11384	9.83E-01	Down	0.02	13796	0.54	0.00
rs5756562	6753	SSTR3	22	35936307	2.88E-01	11385	3.60E-01	Down	0.92	9677	0.54	0.04
rs9595247	2963	GTF2F2	13	44612730	2.88E-01	11386	4.18E-03	Down	2.86	3964	0.54	0.24
rs17840857	3227	HOXC11	12	52644024	2.88E-01	11387	8.06E-01	Up	0.25	12704	0.54	0.01
rs12950390	30009	TBX21	17	43185947	2.88E-01	11388	6.86E-01	Down	0.40	11981	0.54	0.02
rs5029846	50616	IL22	12	66938708	2.88E-01	11389	4.74E-01	Down	0.72	10528	0.54	0.03
rs7240884	5366	PMAIP1	18	55719673	2.88E-01	11390	1.74E-03	Up	3.13	3455	0.54	0.28
rs10165098	84450	ZNF512	2	27708177	2.88E-01	11391	1.70E-04	Down	3.76	2574	0.54	0.38
rs10165098	84226	C2orf16	2	27708177	2.88E-01	11392	7.72E-01	Up	0.29	12508	0.54	0.01
rs1056143	5989	RFX1	19	13926488	2.88E-01	11393	7.01E-01	Up	0.38	12077	0.54	0.02
rs618923	116519	APOA5	11	116159369	2.88E-01	11394	5.70E-01	Down	0.57	11248	0.54	0.02
rs618923	8882	ZNF259	11	116159369	2.88E-01	11395	8.63E-01	Down	0.17	13026	0.54	0.01
rs11264505	128240	APOA1BP	1	153381325	2.89E-01	11396	4.41E-02	Up	2.01	5855	0.54	0.14
rs11264505	54865	GPATCH4	1	153381325	2.89E-01	11397	6.60E-01	Up	0.44	11829	0.54	0.02
rs2270943	4924	NUCB1	19	54093449	2.89E-01	11398	6.67E-01	Up	0.43	11864	0.54	0.02
rs10751722	23038	WDTC1	1	27227665	2.89E-01	11399	8.93E-02	Down	1.70	6789	0.54	0.10
rs12347887	90871	C9orf123	9	7779577	2.89E-01	11400	1.90E-02	Up	2.35	5022	0.54	0.17
rs2075302	2191	FAP	2	162901653	2.89E-01	11401	2.17E-04	Up	3.70	2657	0.54	0.37
rs891750	79882	ZC3H14	14	88086953	2.89E-01	11402	6.66E-02	Up	1.83	6369	0.54	0.12
rs5973962	154796	AMOT		111873526	2.90E-01	11403	3.42E-26	Down	10.59	94	0.54	2.55
rs11191055	27343	POLL	10	103344172	2.90E-01	11404	2.27E-02	Down	2.28	5186	0.54	0.16
rs4234955	4886	NPY1R	4	164617881	2.90E-01	11405	2.15E-01	Down	1.24	8413	0.54	0.07
rs1395371	54876	C4orf30	4	17489980	2.90E-01	11406	1.22E-04	Up	3.84	2496	0.54	0.39
rs6708754	84908	FAM136A	2	70426470	2.90E-01	11407	1.42E-07	Up	5.26	1340	0.54	0.68
rs6708754	6637	SNRPG	2	70426470	2.90E-01	11408	1.56E-01	Up	1.42	7752	0.54	0.08
rs6804995	84666	RETNLB	3	109948147	2.90E-01	11409	3.57E-01	Down	0.92	9653	0.54	0.04
rs806970	3017	HIST1H2BD	6	26249354	2.90E-01	11410	2.28E-01	Up	1.20	8542	0.54	0.06
rs806970	3008	HIST1H1E	6	26249354	2.90E-01	11411	6.91E-01	Up	0.40	12015	0.54	0.02
rs11119925	5284	PIGR	1	203524386	2.90E-01	11412	8.13E-01	Up	0.24	12740	0.54	0.01
rs4825232	6248	RSC1A1		18444451	2.90E-01	11413	1.44E-05	Up	4.34	2028	0.54	0.48
rs4825232	6247	RS1		18444451	2.90E-01	11414	9.51E-01	Up	0.06	13565	0.54	0.00
rs4788213	83723	FAM57B	16	29942025	2.90E-01	11415	1.49E-01	Down	1.44	7659	0.54	0.08
rs4788213	8448	DOC2A	16	29942025	2.90E-01	11416	3.69E-01	Down	0.90	9759	0.54	0.04

rs3741049	38	ACAT1	11	107515137	2.90E-01	11417	2.43E-01	Up	1.17	8696	0.54	0.06
rs11062035	6539	SLC6A12	12	208734	2.90E-01	11418	1.19E-02	Up	2.51	4635	0.54	0.19
rs7041863	89882	TPD52L3	9	6317690	2.90E-01	11419	6.30E-04	Down	3.42	3046	0.54	0.32
rs2072196	9514	GAL3ST1	22	29300067	2.90E-01	11420	4.70E-03	Down	2.83	4021	0.54	0.23
rs2356837	55027	HEATR3	16	48678394	2.90E-01	11421	1.67E-02	Up	2.39	4920	0.54	0.18
rs2491015	26128	KIAA1279	10	70436819	2.90E-01	11422	2.43E-02	Down	2.25	5260	0.54	0.16
rs12481567	671	BPI	20	36357096	2.90E-01	11423	2.78E-01	Up	1.08	9019	0.54	0.06
rs3771719	9936	CD302	2	160456471	2.90E-01	11424	1.71E-02	Up	2.38	4944	0.54	0.18
rs169219	10475	TRIM38	6	26065371	2.90E-01	11425	9.67E-02	Down	1.66	6937	0.54	0.10
rs4790694	409	ARRB2	17	4573103	2.90E-01	11426	3.76E-03	Up	2.90	3896	0.54	0.24
rs4790694	400569	MED11	17	4573103	2.90E-01	11427	5.16E-03	Up	2.80	4084	0.54	0.23
rs7982860	9818	NUPL1	13	24758273	2.90E-01	11428	3.57E-06	Up	4.64	1767	0.54	0.54
rs12871648	3916	LAMP1	13	113018663	2.91E-01	11429	1.14E-04	Up	3.86	2486	0.54	0.39
rs11568311	51647	FAM96B	16	65528758	2.91E-01	11430	4.76E-01	Down	0.71	10543	0.54	0.03
rs11568311	8824	CES2	16	65528758	2.91E-01	11431	7.87E-01	Down	0.27	12589	0.54	0.01
rs11461	4942	OAT	10	126079424	2.91E-01	11432	4.72E-27	Down	10.77	85	0.54	2.63
rs3802901	79684	C11orf61	11	124140354	2.91E-01	11433	5.92E-02	Down	1.89	6220	0.54	0.12
rs5942835	2182	ACSL4		108795583	2.91E-01	11434	4.67E-09	Down	5.86	1003	0.54	0.83
rs10845221	11272	PRR4	12	10880801	2.91E-01	11435	2.58E-02	Up	2.23	5313	0.54	0.16
rs7555395	50999	TMED5	1	93322956	2.91E-01	11436	9.79E-01	Down	0.03	13761	0.54	0.00
rs12467951	5013	OTX1	2	63203673	2.91E-01	11437	9.38E-01	Down	0.08	13483	0.54	0.00
rs9874357	13	AADAC	3	152997403	2.91E-01	11438	4.67E-01	Up	0.73	10481	0.54	0.03
rs896962	7264	TSTA3	8	144758235	2.91E-01	11439	5.99E-20	Up	9.19	193	0.54	1.92
rs896962	65263	PYCRL	8	144758235	2.91E-01	11440	6.48E-02	Up	1.85	6338	0.54	0.12
rs13205436	79694	MANEA	6	96140771	2.91E-01	11441	1.25E-03	Up	3.23	3313	0.54	0.29
rs2509217	7940	LST1	6	31674183	2.91E-01	11442	3.31E-02	Down	2.13	5565	0.54	0.15
rs2509217	199	AIF1	6	31674183	2.91E-01	11443	4.63E-02	Down	1.99	5901	0.54	0.13
rs2509217	259197	NCR3	6	31674183	2.91E-01	11444	7.56E-01	Down	0.31	12417	0.54	0.01
rs2509217	4050	LTB	6	31674183	2.91E-01	11445	9.24E-01	Down	0.10	13393	0.54	0.00
rs853907	63877	C10orf84	10	120061536	2.92E-01	11446	5.79E-01	Down	0.56	11302	0.54	0.02
rs8110888	23646	PLD3	19	45547095	2.92E-01	11447	7.15E-02	Down	1.80	6458	0.54	0.11
rs17319517	54885	TBC1D8B		105880752	2.92E-01	11448	1.61E-02	Up	2.41	4890	0.53	0.18
rs3745157	79760	GEMIN7	19	50286435	2.92E-01	11449	1.74E-01	Up	1.36	7948	0.53	0.08
rs7643434	55186	SLC25A36	3	142159655	2.92E-01	11450	2.03E-03	Up	3.09	3539	0.53	0.27
rs6694932	57185	NPAL3	1	24488909	2.92E-01	11451	3.12E-12	Down	6.97	607	0.53	1.15
rs6823331	54532	USP53	4	120538393	2.92E-01	11452	2.00E-04	Up	3.72	2632	0.53	0.37
rs6057638	149951	COMMD7	20	30802993	2.92E-01	11453	5.30E-01	Up	0.63	10940	0.53	0.03
rs4238383	29767	TMOD2	15	49836002	2.92E-01	11454	4.49E-01	Down	0.76	10339	0.53	0.03
rs8078650	7266	DNAJC7	17	37374297	2.92E-01	11455	5.76E-03	Up	2.76	4158	0.53	0.22
rs8078650	83538	TTC25	17	37374297	2.92E-01	11456	1.20E-01	Up	1.55	7297	0.53	0.09
rs8078650	1267	CNP	17	37374297	2.92E-01	11457	1.64E-01	Down	1.39	7863	0.53	0.08
rs3733236	4283	CXCL9	4	77281177	2.92E-01	11458	6.00E-06	Up	4.53	1844	0.53	0.52
rs7925824	120071	GYLTL1B	11	45886537	2.93E-01	11459	2.67E-02	Up	2.22	5347	0.53	0.16
rs7925824	9409	PEX16	11	45886537	2.93E-01	11460	9.77E-01	Up	0.03	13745	0.53	0.00
rs224215	7752	ZNF200	16	3241361	2.93E-01	11461	1.30E-03	Up	3.22	3323	0.53	0.29
rs224215	4210	MEFV	16	3241361	2.93E-01	11462	5.53E-01	Down	0.59	11104	0.53	0.03
rs3014824	57402	S100A14	1	150214661	2.93E-01	11463	9.11E-02	Down	1.69	6848	0.53	0.10
rs2467584	10480	EIF3M	11	32542951	2.93E-01	11464	1.78E-05	Up	4.29	2069	0.53	0.48
rs7112229	55626	AMBRA1	11	46469825	2.93E-01	11465	6.52E-02	Up	1.84	6345	0.53	0.12
rs10493090	6018	RLF	1	40296011	2.93E-01	11466	3.92E-01	Up	0.86	9924	0.53	0.04
rs1709558	55722	CEP72	5	689613	2.93E-01	11467	7.68E-01	Down	0.29	12476	0.53	0.01
rs7395165	915	CD3D	11	117710912	2.93E-01	11468	1.22E-01	Up	1.55	7330	0.53	0.09
rs7395165	917	CD3G	11	117710912	2.93E-01	11469	6.16E-01	Down	0.50	11544	0.53	0.02
rs12662324	91749	KIAA1919	6	111681430	2.93E-01	11470	9.07E-03	Up	2.61	4440	0.53	0.20
rs8037	25984	KRT23	17	36335239	2.93E-01	11471	4.52E-10	Down	6.23	845	0.53	0.93
rs12394713	159013	CXorf38		40280427	2.93E-01	11472	8.26E-01	Up	0.22	12830	0.53	0.01
rs4911305	128861	C20orf71	20	31276795	2.93E-01	11473	4.46E-01	Up	0.76	10312	0.53	0.04
rs7017582	9046	DOK2	8	21815972	2.93E-01	11474	2.15E-01	Down	1.24	8415	0.53	0.07
rs6966714	142685	ASB15	7	122847445	2.94E-01	11475	5.79E-01	Up	0.55	11312	0.53	0.02
rs805274	80741	LY6G5C	6	31773173	2.94E-01	11476	2.03E-02	Up	2.32	5087	0.53	0.17
rs3806234	3608	ILF2	1	150444144	2.94E-01	11477	3.02E-02	Up	2.17	5470	0.53	0.15
rs17705732	29766	TMOD3	15	49907720	2.94E-01	11478	1.78E-04	Up	3.75	2591	0.53	0.37
rs2832852	140258	KRTAP13-1	21	30694271	2.94E-01	11479	1.04E-01	Up	1.63	7056	0.53	0.10
rs7702532	79844	ZDHHC11	5	905207	2.94E-01	11480	1.47E-03	Up	3.18	3372	0.53	0.28
rs1968752	6817	SULT1A1	16	28539086	2.95E-01	11481	6.64E-03	Up	2.71	4223	0.53	0.22
rs1968618	91404	SESTD1	2	179792310	2.95E-01	11482	1.30E-02	Down	2.48	4689	0.53	0.19
rs2036912	285440	CYP4V2	4	187474192	2.95E-01	11483	5.52E-05	Down	4.03	2312	0.53	0.43
rs4789128	9121	SLC16A5	17	70582383	2.95E-01	11484	4.82E-08	Down	5.46	1217	0.53	0.73
rs1885796	27250	PDCD4	10	112602461	2.95E-01	11485	1.60E-03	Up	3.16	3419	0.53	0.28
rs12539089	28639	TRBC1	7	142013136	2.95E-01	11486	2.15E-01	Up	1.24	8418	0.53	0.07
rs6577166	8634	RTCD1	1	100477717	2.96E-01	11487	1.54E-03	Up	3.17	3396	0.53	0.28
rs3760923	8857	FCGBP	19	45133082	2.96E-01	11488	3.06E-06	Down	4.67	1743	0.53	0.55
rs12848359	8852	AKAP4		49669525	2.96E-01	11489	4.82E-01	Up	0.70	10587	0.53	0.03
rs2233911	6426	SFRS1	17	53438933	2.96E-01	11490	3.62E-03	Up	2.91	3875	0.53	0.24
rs11079764	4635	MYL4	17	42664692	2.96E-01	11491	2.27E-03	Down	3.05	3618	0.53	0.26
rs2569987	121642	ALKBH2	12	108003891	2.96E-01	11492	1.34E-04	Up	3.82	2518	0.53	0.39
rs1359313	144809	C13orf30	13	42269010	2.96E-01	11493	7.45E-01	Down	0.33	12344	0.53	0.01
rs12748814	23623	RUSC1	1	152123516	2.96E-01	11494	8.98E-12	Up	6.83	634	0.53	1.10
rs12748814	284618	C1orf104	1	152123516	2.96E-01	11495	5.64E-01	Down	0.58	11195	0.53	0.02
rs6692382	8832	CD84	1	157364665	2.96E-01	11496	8.84E-01	Up	0.15	13148	0.53	0.01
rs2187152	84897	TBRG1	11	124007131	2.96E-01	11497	9.05E-02	Down	1.69	6834	0.53	0.10

rs4952224	6716	SRD5A2	2	31731907	2.96E-01	11498	1.17E-11	Down	6.78	641	0.53	1.09
rs1018503	23054	NCOA6	20	32789177	2.96E-01	11499	5.23E-01	Up	0.64	10888	0.53	0.03
rs10953708	11179	ZNF277	7	111478939	2.96E-01	11500	5.76E-04	Up	3.44	3011	0.53	0.32
rs521895	5354	PLP1		102842557	2.96E-01	11501	1.13E-01	Down	1.59	7189	0.53	0.09
rs6752442	2637	GBX2	2	236855776	2.96E-01	11502	3.17E-01	Down	1.00	9335	0.53	0.05
rs445310	9812	KIAA0141	5	141281090	2.96E-01	11503	2.66E-09	Up	5.92	982	0.53	0.86
rs4498037	26998	FETUB	3	187823965	2.97E-01	11504	5.95E-02	Down	1.88	6229	0.53	0.12
rs4748864	256815	C10orf67	10	23670201	2.97E-01	11505	6.14E-01	Up	0.50	11530	0.53	0.02
rs11694911	4953	ODC1	2	10523796	2.97E-01	11506	2.06E-04	Up	3.71	2640	0.53	0.37
rs1626670	4521	NUDT1	7	2042211	2.97E-01	11507	1.62E-01	Down	1.40	7837	0.53	0.08
rs1626670	29960	FTSJ2	7	2042211	2.97E-01	11508	1.71E-01	Up	1.37	7925	0.53	0.08
rs8100796	147694	ZNF548	19	62590645	2.97E-01	11509	9.99E-02	Up	1.65	6995	0.53	0.10
rs778294	267012	DAOA	13	104940236	2.97E-01	11510	2.77E-01	Up	1.09	9011	0.53	0.06
rs11638513	55070	DET1	15	86865823	2.97E-01	11511	5.76E-02	Up	1.90	6175	0.53	0.12
rs11629129	2999	GZMH	14	24164696	2.97E-01	11512	1.23E-01	Down	1.54	7340	0.53	0.09
rs11629129	3002	GZMB	14	24164696	2.97E-01	11513	7.70E-01	Down	0.29	12494	0.53	0.01
rs861029	79644	SRD5A3	4	56086565	2.97E-01	11514	1.23E-03	Up	3.23	3303	0.53	0.29
rs541207	283234	CCDC88B	11	63881718	2.97E-01	11515	2.35E-01	Up	1.19	8609	0.53	0.06
rs2712423	23434	C3orf27	3	129773636	2.97E-01	11516	3.84E-01	Up	0.87	9873	0.53	0.04
rs10860845	55010	C12orf48	12	101100169	2.97E-01	11517	2.64E-01	Up	1.12	8903	0.53	0.06
rs10860845	5367	PMCH	12	101100169	2.97E-01	11518	9.41E-01	Down	0.07	13498	0.53	0.00
rs310619	1917	EEF1A2	20	61597965	2.98E-01	11519	4.80E-01	Down	0.71	10577	0.53	0.03
rs3749804	57179	KIAA1191	5	175708761	2.98E-01	11520	3.54E-01	Up	0.93	9623	0.53	0.05
rs256545	5378	PMS1	2	190576945	2.98E-01	11521	1.45E-02	Up	2.45	4784	0.53	0.18
rs7062216	633	BGN		152290349	2.98E-01	11522	1.17E-17	Up	8.56	282	0.53	1.69
rs4886636	4351	MPI	15	72983229	2.98E-01	11523	3.42E-08	Up	5.52	1179	0.53	0.75
rs4886636	57184	C15orf17	15	72983229	2.98E-01	11524	5.75E-01	Up	0.56	11276	0.53	0.02
rs12602520	9703	KIAA0100	17	23979457	2.98E-01	11525	8.93E-05	Up	3.92	2423	0.53	0.40
rs2526275	64386	MMP25	16	3024110	2.98E-01	11526	3.98E-02	Down	2.06	5744	0.53	0.14
rs2526275	54985	HCFC1R1	16	3024110	2.98E-01	11527	1.67E-01	Down	1.38	7886	0.53	0.08
rs2526275	79228	THOC6	16	3024110	2.98E-01	11528	4.85E-01	Down	0.70	10618	0.53	0.03
rs2526275	9074	CLDN6	16	3024110	2.98E-01	11529	6.48E-01	Up	0.46	11761	0.53	0.02
rs4702	5045	FURIN	15	89227564	2.98E-01	11530	2.26E-01	Down	1.21	8524	0.53	0.06
rs7591929	64172	OSGEPL1	2	190468763	2.98E-01	11531	1.36E-07	Up	5.27	1327	0.53	0.69
rs7591929	94101	ORMDL1	2	190468763	2.98E-01	11532	8.81E-03	Up	2.62	4415	0.53	0.21
rs2245928	9982	FGFBP1	4	15615462	2.99E-01	11533	2.28E-03	Down	3.05	3620	0.52	0.26
rs7192417	55625	ZDHHC7	16	83591618	2.99E-01	11534	8.85E-03	Down	2.62	4421	0.52	0.21
rs1342137	81623	DEFB126	20	73121	2.99E-01	11535	7.78E-01	Up	0.28	12540	0.52	0.01
rs2259964	2969	GTF2I	7	73559909	2.99E-01	11536	7.49E-02	Up	1.78	6527	0.52	0.11
rs7019752	7186	TRAF2	9	137099507	2.99E-01	11537	1.36E-01	Down	1.49	7526	0.52	0.09
rs3779536	381	ARF5	7	126827928	3.00E-01	11538	4.63E-02	Up	1.99	5903	0.52	0.13
rs3779536	79571	GCC1	7	126827928	3.00E-01	11539	9.51E-01	Down	0.06	13560	0.52	0.00
rs10493050	8073	PTP4A2	1	32047633	3.00E-01	11540	2.03E-26	Down	10.64	92	0.52	2.57
rs4750390	8559	PRPF18	10	13708722	3.00E-01	11541	1.78E-01	Up	1.35	7989	0.52	0.08
rs4368910	222865	TMEM130	7	98084351	3.00E-01	11542	8.21E-04	Down	3.35	3147	0.52	0.31
rs6746926	3417	IDH1	2	208960845	3.00E-01	11543	9.91E-03	Up	2.58	4503	0.52	0.20
rs11170231	3848	KRT1	12	51354900	3.00E-01	11544	2.11E-01	Down	1.25	8366	0.52	0.07
rs17668126	996	CDC27	17	42636256	3.00E-01	11545	2.62E-01	Up	1.12	8883	0.52	0.06
rs12097239	65094	JMJD4	1	224219209	3.00E-01	11546	2.52E-02	Down	2.24	5296	0.52	0.16
rs13140566	7345	UCHL1	4	41080536	3.00E-01	11547	1.34E-07	Down	5.27	1326	0.52	0.69
rs200932	57209	ZNF248	10	38168774	3.00E-01	11548	1.82E-04	Down	3.74	2594	0.52	0.37
rs3826942	84939	MUM1	19	1311575	3.00E-01	11549	3.97E-06	Up	4.61	1781	0.52	0.54
rs7008137	2675	GFRA2	8	21695078	3.00E-01	11550	2.82E-02	Down	2.19	5402	0.52	0.15
rs885838	8646	CHRD	3	185579280	3.00E-01	11551	1.92E-01	Up	1.30	8170	0.52	0.07
rs729228	23514	KIAA0146	8	48736526	3.00E-01	11552	6.17E-02	Up	1.87	6272	0.52	0.12
rs11640420	23214	XPO6	16	28109841	3.00E-01	11553	4.21E-15	Up	7.84	386	0.52	1.44
rs9967229	147339	C18orf25	18	42064227	3.00E-01	11554	2.31E-03	Up	3.05	3637	0.52	0.26
rs2071675	7169	TPM2	9	35663891	3.00E-01	11555	2.10E-25	Down	10.42	108	0.52	2.47
rs2071675	768	CA9	9	35663891	3.00E-01	11556	7.51E-01	Up	0.32	12380	0.52	0.01
rs12588784	317761	C14orf39	14	59966493	3.01E-01	11557	3.48E-02	Up	2.11	5618	0.52	0.15
rs12390419	416	ARSF		3024238	3.01E-01	11558	6.22E-01	Up	0.49	11598	0.52	0.02
rs2162610	1493	CTLA4	2	204544235	3.01E-01	11559	2.52E-01	Down	1.14	8785	0.52	0.06
rs6746036	57590	WDFY1	2	224617761	3.01E-01	11560	3.66E-01	Up	0.90	9726	0.52	0.04
rs2255627	6617	SNAPC1	14	61309786	3.01E-01	11561	5.52E-01	Down	0.60	11092	0.52	0.03
rs2437150	83932	C1orf124	1	227795259	3.01E-01	11562	6.23E-01	Up	0.49	11599	0.52	0.02
rs10484432	8359	HIST1H4A	6	26116855	3.01E-01	11563	9.57E-02	Up	1.67	6917	0.52	0.10
rs10484432	3024	HIST1H1A	6	26116855	3.01E-01	11564	1.26E-01	Down	1.53	7388	0.52	0.09
rs10484432	8350	HIST1H3A	6	26116855	3.01E-01	11565	9.66E-01	Up	0.04	13672	0.52	0.00
rs2254437	6136	RPL12	9	127304481	3.01E-01	11566	9.97E-11	Up	6.46	758	0.52	1.00
rs3783438	79178	THTPA	14	23078390	3.01E-01	11567	1.07E-01	Up	1.61	7106	0.52	0.10
rs226205	6228	RPS23	5	81629410	3.01E-01	11568	8.02E-04	Up	3.35	3141	0.52	0.31
rs12915032	3073	HEXA	15	70431715	3.01E-01	11569	1.51E-01	Down	1.43	7699	0.52	0.08
rs11024884	259249	MRGPRX1	11	18906668	3.02E-01	11570	2.01E-01	Up	1.28	8262	0.52	0.07
rs9383640	80177	MYCT1	6	153143148	3.02E-01	11571	3.39E-01	Up	0.96	9511	0.52	0.05
rs3829997	2277	FIGF		15174274	3.02E-01	11572	9.70E-01	Down	0.04	13701	0.52	0.00
rs4291799	11340	EXOSC8	13	36456968	3.02E-01	11573	3.82E-01	Down	0.87	9846	0.52	0.04
rs4291799	29880	ALG5	13	36456968	3.02E-01	11574	4.34E-01	Down	0.78	10223	0.52	0.04
rs17067435	55278	QRSL1	6	107165189	3.02E-01	11575	8.59E-02	Down	1.72	6732	0.52	0.11
rs4982704	55147	RBM23	14	22433377	3.02E-01	11576	9.08E-02	Down	1.69	6843	0.52	0.10
rs2301661	9454	HOMER3	19	18898053	3.02E-01	11577	1.70E-05	Up	4.30	2059	0.52	0.48
rs818816	9389	SLC22A14	3	38326298	3.02E-01	11578	3.37E-01	Down	0.96	9500	0.52	0.05

rs12905620	23742	C15orf2	15	22481471	3.02E-01	11579	2.21E-01	Up	1.22	8465	0.52	0.07
rs874069	219899	TBCEL	11	120459124	3.03E-01	11580	7.34E-01	Down	0.34	12280	0.52	0.01
rs1888057	9365	KL	13	32520695	3.03E-01	11581	4.89E-01	Down	0.69	10651	0.52	0.03
rs10752073	7431	VIM	10	17304492	3.03E-01	11582	1.19E-05	Down	4.38	1986	0.52	0.49
rs7099098	387695	C10orf99	10	85940386	3.03E-01	11583	1.75E-02	Down	2.38	4960	0.52	0.18
rs9973663	64090	GAL3ST2	2	242445742	3.03E-01	11584	1.12E-01	Down	1.59	7186	0.52	0.09
rs126092	79879	CCDC134	22	40502941	3.03E-01	11585	1.31E-01	Up	1.51	7459	0.52	0.09
rs4669018	10408	MYCNOS	2	16040213	3.03E-01	11586	7.46E-02	Up	1.78	6520	0.52	0.11
rs4669018	4613	MYCN	2	16040213	3.03E-01	11587	7.67E-01	Up	0.30	12470	0.52	0.01
rs10957057	1581	CYP7A1	8	59562894	3.03E-01	11588	5.33E-02	Up	1.93	6072	0.52	0.13
rs1542313	22890	ZBTB1	14	64069791	3.03E-01	11589	4.48E-05	Up	4.08	2254	0.52	0.43
rs1542313	3306	HSPA2	14	64069791	3.03E-01	11590	3.70E-01	Down	0.90	9764	0.52	0.04
rs854672	6368	CCL23	17	31384951	3.03E-01	11591	1.79E-02	Up	2.37	4979	0.52	0.17
rs3813230	10617	STAMBP	2	74005307	3.03E-01	11592	4.84E-01	Up	0.70	10604	0.52	0.03
rs6576987	51239	ANKRD39	2	96933933	3.03E-01	11593	8.16E-01	Up	0.23	12759	0.52	0.01
rs6576987	200539	ANKRD23	2	96933933	3.03E-01	11594	9.52E-01	Down	0.06	13568	0.52	0.00
rs2640	5395	PMS2	7	5839691	3.03E-01	11595	7.04E-01	Down	0.38	12099	0.52	0.02
rs3786853	26993	AKAP8L	19	15389983	3.03E-01	11596	5.63E-02	Down	1.91	6145	0.52	0.12
rs10760575	29941	PKN3	9	128569005	3.03E-01	11597	2.04E-01	Down	1.27	8293	0.52	0.07
rs10760575	84885	ZDHHC12	9	128569005	3.03E-01	11598	9.03E-01	Down	0.12	13269	0.52	0.00
rs8887	729359	KIAA1881	19	4453201	3.04E-01	11599	3.84E-08	Down	5.50	1198	0.52	0.74
rs4378078	158055	C9orf163	9	136651799	3.04E-01	11600	2.00E-01	Up	1.28	8259	0.52	0.07
rs2040041	10745	PHTF1	1	114024665	3.04E-01	11601	2.99E-01	Up	1.04	9187	0.52	0.05
rs4927868	7037	TFRC	3	197293073	3.04E-01	11602	1.37E-03	Down	3.20	3343	0.52	0.29
rs2070776	2688	GH1	17	59361230	3.04E-01	11603	1.51E-01	Up	1.44	7691	0.52	0.08
rs2070776	1444	CSHL1	17	59361230	3.04E-01	11604	5.05E-01	Up	0.67	10753	0.52	0.03
rs12975781	2523	FUT1	19	53941510	3.04E-01	11605	2.53E-05	Down	4.21	2135	0.52	0.46
rs12975781	26291	FGF21	19	53941510	3.04E-01	11606	1.46E-02	Down	2.44	4790	0.52	0.18
rs12975781	284359	IZUMO1	19	53941510	3.04E-01	11607	9.99E-01	Down	0.00	13904	0.52	0.00
rs16896994	3788	KCNS2	8	99515761	3.04E-01	11608	9.44E-01	Up	0.07	13523	0.52	0.00
rs2893321	10673	TNFSF13B	13	107741035	3.04E-01	11609	7.83E-02	Up	1.76	6601	0.52	0.11
rs2295320	4738	NEDD8	14	23752607	3.04E-01	11610	7.61E-04	Down	3.37	3116	0.52	0.31
rs2295320	51292	GMPR2	14	23752607	3.04E-01	11611	7.47E-01	Up	0.32	12356	0.52	0.01
rs12884265	3895	KTN1	14	55154883	3.04E-01	11612	1.77E-01	Up	1.35	7987	0.52	0.08
rs955434	9356	SLC22A6	11	62513689	3.04E-01	11613	7.83E-01	Down	0.28	12563	0.52	0.01
rs4280362	126123	C19orf41	19	55373590	3.05E-01	11614	2.46E-01	Down	1.16	8726	0.52	0.06
rs12974420	90324	CCDC97	19	46490500	3.05E-01	11615	3.53E-01	Down	0.93	9618	0.52	0.05
rs2292260	8572	PDLIM4	5	131634835	3.05E-01	11616	8.49E-14	Down	7.46	467	0.52	1.31
rs12049605	9877	ZC3H11A	1	200518860	3.05E-01	11617	9.02E-01	Up	0.12	13256	0.52	0.00
rs1998289	51076	CUTC	10	101481010	3.05E-01	11618	4.39E-01	Up	0.77	10259	0.52	0.04
rs1998289	1355	COX15	10	101481010	3.05E-01	11619	4.56E-01	Up	0.75	10385	0.52	0.03
rs10016483	901	CCNG2	4	78420866	3.05E-01	11620	-7.24E-13	Up	7.29	511	0.52	#NUM!
rs3825080	64333	ARHGAP9	12	56157822	3.05E-01	11621	2.15E-01	Down	1.24	8406	0.52	0.07
rs4715210	2509	FTHP1	6	51005210	3.05E-01	11622	4.45E-04	Down	3.51	2917	0.52	0.34
rs11887205	7514	XPO1	2	61686124	3.06E-01	11623	3.93E-02	Up	2.06	5732	0.51	0.14
rs2472304	1544	CYP1A2	15	72831291	3.06E-01	11624	8.85E-01	Down	0.14	13155	0.51	0.01
rs2269228	1962	EHHADH	3	186412940	3.06E-01	11625	5.23E-04	Up	3.47	2972	0.51	0.33
rs152220	134429	STARD4	5	110881025	3.06E-01	11626	7.94E-08	Down	5.37	1272	0.51	0.71
rs2016575	7134	TNNC1	3	52452120	3.06E-01	11627	3.13E-02	Up	2.15	5506	0.51	0.15
rs12408942	4014	LOR	1	150054750	3.06E-01	11628	8.29E-01	Down	0.22	12849	0.51	0.01
rs10838191	221120	ALKBH3	11	43850234	3.06E-01	11629	6.11E-01	Up	0.51	11511	0.51	0.02
rs11603236	57175	CORO1B	11	66987331	3.06E-01	11630	1.78E-02	Up	2.37	4973	0.51	0.17
rs11603236	57010	CABP4	11	66987331	3.06E-01	11631	6.04E-02	Up	1.88	6242	0.51	0.12
rs323344	56154	TEX15	8	30822067	3.07E-01	11632	2.76E-01	Down	1.09	9001	0.51	0.06
rs3732765	64805	P2RY12	3	152573122	3.07E-01	11633	4.40E-01	Down	0.77	10271	0.51	0.04
rs159533	3925	STMN1	1	25923420	3.07E-01	11634	3.78E-12	Up	6.95	616	0.51	1.14
rs7807085	6369	CCL24	7	75081758	3.07E-01	11635	4.55E-01	Down	0.75	10380	0.51	0.03
rs13005184	79031	PDCL3	2	100654629	3.07E-01	11636	1.59E-04	Up	3.78	2563	0.51	0.38
rs2394644	219743	TYSND1	10	71560636	3.07E-01	11637	1.35E-01	Up	1.50	7510	0.51	0.09
rs2074890	10626	TRIM16	17	15495286	3.07E-01	11638	1.63E-02	Down	2.40	4905	0.51	0.18
rs11803889	51603	KIAA0859	1	168475232	3.08E-01	11639	5.92E-01	Up	0.54	11383	0.51	0.02
rs8060934	22980	TCF25	16	88447526	3.08E-01	11640	3.65E-01	Up	0.91	9721	0.51	0.04
rs10783425	9802	DAZAP2	12	49916559	3.08E-01	11641	1.60E-02	Down	2.41	4888	0.51	0.18
rs749512	151903	CCDC12	3	47010904	3.08E-01	11642	2.19E-01	Down	1.23	8446	0.51	0.07
rs749512	23218	NBEAL2	3	47010904	3.08E-01	11643	5.67E-01	Up	0.57	11221	0.51	0.02
rs6667575	1870	E2F2	1	23604827	3.08E-01	11644	4.49E-01	Up	0.76	10338	0.51	0.03
rs12124784	653121	ZBTB8	1	32597187	3.08E-01	11645	1.92E-01	Up	1.31	8168	0.51	0.07
rs7960152	55592	GOLGA2L1	12	99078241	3.08E-01	11646	8.65E-01	Up	0.17	13035	0.51	0.01
rs4808178	93436	ARMC6	19	19000051	3.08E-01	11647	4.38E-01	Up	0.78	10257	0.51	0.04
rs2129848	51111	SUV420H1	11	67728122	3.08E-01	11648	1.42E-04	Up	3.80	2534	0.51	0.38
rs4808272	79852	ABHD9	19	15213372	3.08E-01	11649	5.66E-01	Down	0.57	11212	0.51	0.02
rs11798121	83692	CD99L2		149695747	3.08E-01	11650	7.62E-01	Up	0.30	12450	0.51	0.01
rs889135	84258	SYT3	19	55833298	3.08E-01	11651	8.49E-01	Up	0.19	12949	0.51	0.01
rs2963769	5229	PGGT1B	5	114645183	3.09E-01	11652	6.68E-07	Up	4.97	1516	0.51	0.62
rs802036	54677	CROT	7	86622545	3.09E-01	11653	4.82E-01	Up	0.70	10591	0.51	0.03
rs3788941	23439	ATP1B4		119285333	3.09E-01	11654	6.89E-01	Up	0.40	12003	0.51	0.02
rs12491673	55669	MFN1	3	180533423	3.09E-01	11655	2.76E-02	Up	2.20	5381	0.51	0.16
rs12031625	113451	ADC	1	33259966	3.09E-01	11656	3.15E-01	Up	1.01	9318	0.51	0.05
rs2230365	7124	TNF	6	31633427	3.09E-01	11657	2.92E-03	Up	2.98	3762	0.51	0.25
rs10797649	148738	HFE2	1	142883999	3.09E-01	11658	9.83E-01	Up	0.02	13799	0.51	0.00
rs999157	114803	MYSM1	1	58893618	3.09E-01	11659	4.81E-03	Up	2.82	4032	0.51	0.23

rs4238833	172	AFG3L1	16	88578190	3.09E-01	11660	2.02E-01	Down	1.28	8275	0.51	0.07
rs2286472	90850	ZNF598	16	2010569	3.09E-01	11661	8.89E-03	Up	2.62	4426	0.51	0.21
rs2286472	283869	NPW	16	2010569	3.09E-01	11662	9.58E-01	Down	0.05	13603	0.51	0.00
rs1046139	26056	RAB11FIP5	2	73212316	3.09E-01	11663	1.12E-02	Down	2.54	4589	0.51	0.19
rs7806980	140453	MUC17	7	100287010	3.09E-01	11664	4.32E-01	Up	0.79	10211	0.51	0.04
rs6662946	60484	HAPLN2	1	153413974	3.09E-01	11665	2.00E-01	Down	1.28	8258	0.51	0.07
rs3785784	22905	EPN2	17	19136024	3.09E-01	11666	1.72E-01	Down	1.37	7936	0.51	0.08
rs2295797	7094	TLN1	9	35712282	3.10E-01	11667	2.36E-11	Down	6.68	679	0.51	1.06
rs2295797	148327	CREB3L4	9	35712282	3.10E-01	11668	3.05E-06	Up	4.67	1739	0.51	0.55
rs2295797	10488	CREB3	9	35712282	3.10E-01	11669	1.17E-01	Down	1.57	7241	0.51	0.09
rs9879397	7867	MAPKAPK3	3	50660646	3.10E-01	11670	7.67E-11	Up	6.51	741	0.51	1.01
rs423757	124801	LSM12	17	39463235	3.10E-01	11671	2.08E-02	Up	2.31	5105	0.51	0.17
rs2299851	1192	CLIC1	6	31826581	3.10E-01	11672	5.72E-02	Up	1.90	6167	0.51	0.12
rs793523	84986	ARHGAP19	10	99013536	3.10E-01	11673	3.82E-01	Up	0.87	9850	0.51	0.04
rs6488386	5545	PRB4	12	11353103	3.10E-01	11674	8.98E-01	Down	0.13	13231	0.51	0.00
rs461964	3833	KIFC1	6	33468552	3.11E-01	11675	2.60E-04	Up	3.65	2725	0.51	0.36
rs461964	5252	PHF1	6	33468552	3.11E-01	11676	1.55E-03	Down	3.17	3401	0.51	0.28
rs1157468	284697	BTBD8	1	92310575	3.11E-01	11677	9.63E-01	Up	0.05	13634	0.51	0.00
rs4899301	56936	C14orf162	14	69098300	3.11E-01	11678	9.19E-01	Up	0.10	13371	0.51	0.00
rs1044189	5777	PTPN6	12	6923410	3.11E-01	11679	8.21E-01	Up	0.23	12795	0.51	0.01
rs7640258	84107	ZIC4	3	148624077	3.11E-01	11680	3.69E-01	Down	0.90	9750	0.51	0.04
rs7640258	7545	ZIC1	3	148624077	3.11E-01	11681	6.28E-01	Up	0.48	11638	0.51	0.02
rs25748	54908	CCDC99	5	168933735	3.11E-01	11682	1.84E-01	Up	1.33	8066	0.51	0.07
rs9729157	22977	AKR7A3	1	19363907	3.11E-01	11683	2.20E-01	Up	1.23	8458	0.51	0.07
rs8049439	11273	ATXN2L	16	28745016	3.11E-01	11684	3.42E-08	Up	5.52	1182	0.51	0.75
rs1108842	55830	GLT8D1	3	52695120	3.11E-01	11685	7.34E-13	Up	7.16	549	0.51	1.21
rs1108842	26354	GNL3	3	52695120	3.11E-01	11686	1.25E-12	Up	7.13	556	0.51	1.19
rs12726220	25912	C1orf43	1	150984623	3.11E-01	11687	3.79E-03	Up	2.89	3902	0.51	0.24
rs7198494	123775	C16orf46	16	79652592	3.11E-01	11688	3.51E-04	Up	3.57	2835	0.51	0.35
rs3731866	7429	VIL1	2	219114404	3.12E-01	11689	3.11E-01	Up	1.01	9277	0.51	0.05
rs7223281	63897	HEATR6	17	55527229	3.12E-01	11690	2.82E-01	Up	1.08	9048	0.51	0.06
rs9834639	65010	SLC26A6	3	48634013	3.12E-01	11691	1.33E-04	Up	3.82	2517	0.51	0.39
rs10263979	440	ASNS	7	97140830	3.12E-01	11692	2.00E-05	Up	4.27	2086	0.51	0.47
rs3737267	84419	C15orf48	15	43490091	3.12E-01	11693	1.44E-03	Up	3.19	3359	0.51	0.28
rs3737267	79029	SPATA5L1	15	43490091	3.12E-01	11694	9.37E-02	Up	1.68	6895	0.51	0.10
rs8108738	5296	PIK3R2	19	18116359	3.12E-01	11695	8.29E-03	Up	2.64	4367	0.51	0.21
rs10747626	60673	C12orf44	12	50746691	3.12E-01	11696	4.08E-01	Up	0.83	10043	0.51	0.04
rs7010330	5339	PLEC1	8	145108603	3.12E-01	11697	3.34E-01	Down	0.97	9474	0.51	0.05
rs6125699		B4GALT5	20	47696256	3.13E-01	11698	4.28E-01	Up	0.79	10179	0.50	0.04
rs223340	7323	UBE2D3	4	104142295	3.13E-01	11699	2.66E-01	Down	1.11	8919	0.50	0.06
rs16948889	9187	SLC24A1	15	63717937	3.13E-01	11700	1.26E-02	Up	2.49	4666	0.50	0.19
rs661821	374877	C19orf45	19	7488649	3.13E-01	11701	1.56E-02	Up	2.42	4842	0.50	0.18
rs12443981	22928	SEPHS2	16	30367556	3.13E-01	11702	1.44E-02	Up	2.45	4780	0.50	0.18
rs13021	122553	TRAPPC6B	14	38720675	3.13E-01	11703	4.46E-01	Down	0.76	10310	0.50	0.04
rs13021	5411	PNN	14	38720675	3.13E-01	11704	6.82E-01	Down	0.41	11958	0.50	0.02
rs7897947	5662	PSD	10	104147701	3.13E-01	11705	1.95E-01	Down	1.30	8206	0.50	0.07
rs10484431	10246	SLC17A2	6	26034653	3.13E-01	11706	2.12E-01	Up	1.25	8382	0.50	0.07
rs929502	1089	CEACAM4	19	46822616	3.13E-01	11707	2.43E-01	Down	1.17	8685	0.50	0.06
rs10485358	23469	PHF3	6	64444019	3.13E-01	11708	9.98E-01	Up	0.00	13901	0.50	0.00
rs947863	55863	TMEM126B	11	85005503	3.13E-01	11709	9.12E-01	Up	0.11	13318	0.50	0.00
rs7253473	8193	DPF1	19	43409218	3.13E-01	11710	4.53E-02	Down	2.00	5880	0.50	0.13
rs527365	2189	FANCG	9	35088155	3.14E-01	11711	1.60E-05	Up	4.31	2052	0.50	0.48
rs6731616	7277	TUBA4A	2	132068718	3.14E-01	11712	2.58E-18	Down	8.73	258	0.50	1.76
rs6731616	113457	TUBA3D	2	132068718	3.14E-01	11713	3.97E-09	Down	5.89	992	0.50	0.84
rs10985794	5082	PDCL	9	122686171	3.14E-01	11714	5.21E-01	Up	0.64	10866	0.50	0.03
rs7523070	54797	MED18	1	28324915	3.14E-01	11715	9.10E-01	Up	0.11	13306	0.50	0.00
rs1186675	3765	KCNJ9	1	156852157	3.14E-01	11716	1.48E-01	Down	1.45	7649	0.50	0.08
rs10024839	6307	SC4MOL	4	166622572	3.14E-01	11717	3.83E-09	Down	5.89	988	0.50	0.84
rs17516450	8492	PRSS12	4	119605348	3.14E-01	11718	1.17E-01	Down	1.57	7245	0.50	0.09
rs11881242	115703	SNX26	19	40975687	3.15E-01	11719	1.88E-01	Up	1.32	8126	0.50	0.07
rs655970	23596	OPN3	1	238080877	3.15E-01	11720	3.80E-08	Up	5.50	1199	0.50	0.74
rs3731940	81562	LMAN2L	2	96799757	3.15E-01	11721	1.73E-01	Down	1.36	7946	0.50	0.08
rs1012919	4792	NFKBIA	14	34954390	3.15E-01	11722	9.00E-02	Down	1.70	6814	0.50	0.10
rs5987126	139728	PNCK		152453473	3.15E-01	11723	5.06E-05	Down	4.05	2291	0.50	0.43
rs3180427	26985	AP3M1	10	75551353	3.16E-01	11724	9.60E-01	Down	0.05	13622	0.50	0.00
rs351734	285753	C6orf182	6	109582109	3.16E-01	11725	6.50E-01	Down	0.45	11777	0.50	0.02
rs10866705	6569	SLC34A1	5	176733737	3.16E-01	11726	3.06E-01	Down	1.02	9234	0.50	0.05
rs6676800	58480	RHOU	1	225197838	3.16E-01	11727	3.25E-01	Up	0.98	9409	0.50	0.05
rs1317850	51226	COPZ2	17	43476838	3.17E-01	11728	4.84E-16	Down	8.12	348	0.50	1.53
rs1563416	85403	EAF1	3	15448768	3.17E-01	11729	6.04E-01	Up	0.52	11460	0.50	0.02
rs1563416	131965	METTL6	3	15448768	3.17E-01	11730	7.12E-01	Down	0.37	12149	0.50	0.01
rs539822	90249	UNC5A	5	176242770	3.17E-01	11731	4.11E-03	Down	2.87	3949	0.50	0.24
rs4646485	1580	CYP4B1	1	46990859	3.17E-01	11732	9.32E-16	Down	8.04	357	0.50	1.50
rs4988186	4175	MCM6	2	136455590	3.17E-01	11733	4.44E-02	Down	2.01	5862	0.50	0.14
rs497551	55869	HDAC8		71345539	3.17E-01	11734	8.26E-01	Down	0.22	12827	0.50	0.01
rs3213565	9921	RNF10	12	119455121	3.17E-01	11735	9.39E-01	Down	0.08	13486	0.50	0.00
rs9304712	89790	SIGLEC10	19	56617331	3.17E-01	11736	1.98E-01	Up	1.29	8231	0.50	0.07
rs561136	23028	AOF2	1	23090357	3.17E-01	11737	4.06E-03	Up	2.87	3941	0.50	0.24
rs8065686	10014	HDAC5	17	39549423	3.17E-01	11738	7.43E-04	Down	3.37	3103	0.50	0.31
rs2757545	7528	YY1	14	99765121	3.17E-01	11739	1.29E-01	Up	1.52	7441	0.50	0.09
rs6019615	1434	CSE1L	20	47102103	3.17E-01	11740	3.01E-06	Up	4.67	1737	0.50	0.55

rs11178927	23011	RAB21	12	70429787	3.17E-01	11741	9.20E-02	Down	1.68	6861	0.50	0.10
rs3741690	55266	TMEM19	12	70377292	3.17E-01	11742	1.25E-01	Down	1.53	7369	0.50	0.09
rs2072840	84679	SLC9A7		46223014	3.17E-01	11743	1.54E-03	Up	3.17	3395	0.50	0.28
rs2072840	56548	CHST7		46223014	3.17E-01	11744	9.32E-01	Down	0.09	13447	0.50	0.00
rs2108411	55744	C7orf44	7	43439048	3.18E-01	11745	9.21E-02	Up	1.68	6865	0.50	0.10
rs2108411	9263	STK17A	7	43439048	3.18E-01	11746	7.89E-01	Down	0.27	12606	0.50	0.01
rs5940453	7411	VBP1		154009463	3.18E-01	11747	4.96E-06	Up	4.57	1815	0.50	0.53
rs2314664	7311	UBA52	19	18552942	3.18E-01	11748	2.60E-03	Up	3.01	3710	0.50	0.26
rs2314664	55049	C19orf60	19	18552942	3.18E-01	11749	4.27E-01	Down	0.79	10169	0.50	0.04
rs573836	245	ALOX12P2	17	6739102	3.18E-01	11750	2.46E-04	Down	3.67	2703	0.50	0.36
rs10467685	6144	RPL21	13	26726041	3.18E-01	11751	4.08E-01	Down	0.83	10038	0.50	0.04
rs920980	9319	TRIP13	5	949983	3.18E-01	11752	2.94E-07	Up	5.13	1413	0.50	0.65
rs920980	65980	BRD9	5	949983	3.18E-01	11753	5.12E-01	Up	0.66	10789	0.50	0.03
rs12534545	23608	MKRN1	7	139648647	3.18E-01	11754	7.04E-02	Up	1.81	6439	0.50	0.12
rs7246856	57191	VN1R1	19	62648640	3.18E-01	11755	1.63E-01	Down	1.39	7854	0.50	0.08
rs10736492	372	ARCN1	11	117964479	3.18E-01	11756	7.38E-01	Down	0.33	12304	0.50	0.01
rs11679767	150709	ANKAR	2	190371806	3.18E-01	11757	1.02E-01	Up	1.64	7034	0.50	0.10
rs1476080	196	AHR	7	17131113	3.18E-01	11758	6.24E-07	Down	4.98	1509	0.50	0.62
rs4630763	79919	C2orf54	2	241558494	3.18E-01	11759	6.12E-01	Up	0.51	11521	0.50	0.02
rs5931014	27316	RBMX		135698425	3.18E-01	11760	2.18E-09	Up	5.99	940	0.50	0.87
rs9394952	89845	ABCC10	6	43509083	3.18E-01	11761	3.57E-01	Down	0.92	9647	0.50	0.04
rs611704	84662	GLIS2	16	4335242	3.18E-01	11762	6.00E-01	Up	0.52	11430	0.50	0.02
rs1964986	5644	PRSS1	7	141933929	3.18E-01	11763	4.41E-01	Down	0.77	10276	0.50	0.04
rs6511183	80264	ZNF430	19	21041269	3.18E-01	11764	5.87E-01	Up	0.54	11354	0.50	0.02
rs10477292	5813	PURA	5	139455795	3.19E-01	11765	1.39E-05	Down	4.35	2023	0.50	0.49
rs6627746	84968	PNMA6A		151921824	3.19E-01	11766	7.09E-02	Down	1.81	6451	0.50	0.11
rs17141139	56311	ANKRD7	7	117479749	3.19E-01	11767	5.49E-01	Up	0.60	11071	0.50	0.03
rs10767973	79832	QSER1	11	32915185	3.19E-01	11768	9.40E-01	Up	0.08	13492	0.50	0.00
rs10409376	26333	OR7A17	19	14858907	3.19E-01	11769	7.85E-01	Up	0.27	12579	0.50	0.01
rs10185938	79809	TTC21B	2	166557437	3.19E-01	11770	5.80E-01	Down	0.55	11316	0.50	0.02
rs1190715	1735	DIO3	14	101092832	3.19E-01	11771	7.07E-01	Up	0.38	12112	0.50	0.02
rs1968201	349152	DPY19L2P2	7	102401386	3.19E-01	11772	2.21E-01	Down	1.22	8466	0.50	0.07
rs7970839	84934	C12orf52	12	112100163	3.19E-01	11773	2.51E-09	Up	5.96	951	0.50	0.86
rs7970839	79039	DDX54	12	112100163	3.19E-01	11774	4.24E-01	Up	0.80	10151	0.50	0.04
rs1129700	26470	SEZ6L2	16	29825535	3.19E-01	11775	3.32E-07	Up	5.10	1431	0.50	0.65
rs1129700	253980	KCTD13	16	29825535	3.19E-01	11776	2.23E-02	Up	2.29	5173	0.50	0.17
rs17066865	4160	MC4R	18	56204175	3.20E-01	11777	6.03E-01	Up	0.52	11456	0.50	0.02
rs4836593	8818	DPM2	9	127776264	3.20E-01	11778	6.71E-02	Up	1.83	6376	0.50	0.12
rs4836593	138429	PIP5KL1	9	127776264	3.20E-01	11779	9.14E-01	Down	0.11	13339	0.50	0.00
rs3813766	57547	ZNF624	17	16498331	3.20E-01	11780	2.72E-01	Up	1.10	8965	0.50	0.06
rs336592	165679	C3orf57	3	162559888	3.20E-01	11781	8.81E-01	Down	0.15	13133	0.49	0.01
rs6051098	3420	IDH3B	20	2604328	3.20E-01	11782	6.82E-02	Down	1.82	6399	0.49	0.12
rs13091025	51533	PHF7	3	52442364	3.20E-01	11783	2.25E-01	Up	1.21	8510	0.49	0.06
rs9993942	246175	CNOT6L	4	79006415	3.20E-01	11784	2.96E-01	Up	1.05	9160	0.49	0.05
rs17639574	147929	ZNF565	19	41366905	3.20E-01	11785	2.35E-05	Up	4.23	2117	0.49	0.46
rs841718	4665	NAB2	12	55779263	3.20E-01	11786	6.30E-04	Down	3.42	3047	0.49	0.32
rs11238609	7580	ZNF32	10	43453997	3.20E-01	11787	5.40E-01	Up	0.61	11012	0.49	0.03
rs11688	3725	JUN	1	58960014	3.21E-01	11788	3.77E-02	Down	2.08	5698	0.49	0.14
rs5947379	7789	ZXDA		57811755	3.21E-01	11789	2.11E-01	Down	1.25	8361	0.49	0.07
rs6036478	1471	CST3	20	23559359	3.21E-01	11790	3.26E-01	Down	0.98	9413	0.49	0.05
rs7463174	11212	PROSC	8	37754807	3.21E-01	11791	8.38E-06	Down	4.46	1913	0.49	0.51
rs8133991	522	ATP5J	21	26031357	3.21E-01	11792	7.17E-02	Down	1.80	6462	0.49	0.11
rs6947240	3757	KCNH2	7	150094857	3.21E-01	11793	9.44E-18	Down	8.58	277	0.49	1.70
rs11048401	10219	KLRG1	12	9030549	3.21E-01	11794	8.70E-01	Up	0.16	13068	0.49	0.01
rs1001974	2091	FBL	19	45038875	3.21E-01	11795	6.19E-01	Up	0.50	11566	0.49	0.02
rs1344958	466	ATF1	12	49483984	3.22E-01	11796	1.22E-01	Up	1.55	7332	0.49	0.09
rs727422	6309	SC5DL	11	120673388	3.22E-01	11797	6.43E-02	Down	1.85	6328	0.49	0.12
rs1205961	5617	PRL	6	22393991	3.22E-01	11798	1.13E-02	Up	2.53	4591	0.49	0.19
rs3737557	537	ATP6AP1		153180917	3.22E-01	11799	2.61E-11	Up	6.65	686	0.49	1.06
rs4657015	4359	MPZ	1	158085514	3.22E-01	11800	7.17E-04	Down	3.38	3086	0.49	0.31
rs4657015	6391	SDHC	1	158085514	3.22E-01	11801	7.49E-01	Down	0.32	12366	0.49	0.01
rs797770	928	CD9	12	6173418	3.22E-01	11802	4.59E-03	Down	2.83	4008	0.49	0.23
rs6600191	83986	ITFG3	16	235796	3.22E-01	11803	1.32E-01	Up	1.51	7468	0.49	0.09
rs6600191	55692	LUC7L	16	235796	3.22E-01	11804	6.13E-01	Up	0.51	11527	0.49	0.02
rs10846782	57647	DHX37	12	123957498	3.22E-01	11805	1.54E-01	Down	1.43	7731	0.49	0.08
rs682585	6004	RGS16	1	179291167	3.22E-01	11806	9.61E-02	Up	1.66	6926	0.49	0.10
rs13143848	84103	C4orf17	4	100800898	3.23E-01	11807	2.30E-01	Down	1.20	8563	0.49	0.06
rs4740358	23404	EXOSC2	9	130597313	3.23E-01	11808	1.82E-01	Up	1.33	8045	0.49	0.07
rs329973	7696	ZNF137	19	57797359	3.23E-01	11809	3.53E-05	Up	4.14	2205	0.49	0.45
rs2109069	91039	DPP9	19	4670443	3.23E-01	11810	6.68E-01	Down	0.43	11872	0.49	0.02
rs2250315	9468	PCYT1B		24427924	3.23E-01	11811	6.44E-01	Up	0.46	11741	0.49	0.02
rs858759	10203	CALCRL	2	188061117	3.23E-01	11812	9.82E-01	Up	0.02	13786	0.49	0.00
rs17128136	122809	SOCS4	14	54579863	3.23E-01	11813	7.40E-01	Down	0.33	12314	0.49	0.01
rs1274500	1431	CS	12	54945126	3.23E-01	11814	6.93E-03	Up	2.70	4253	0.49	0.22
rs1274500	283373	ANKRD52	12	54945126	3.23E-01	11815	1.72E-02	Up	2.38	4951	0.49	0.18
rs1274500	93058	COQ10A	12	54945126	3.23E-01	11816	2.37E-02	Down	2.26	5232	0.49	0.16
rs4830891	9367	RAB9A		13485598	3.23E-01	11817	3.68E-01	Up	0.90	9742	0.49	0.04
rs1008236	3169	FOXA1	14	37143990	3.23E-01	11818	4.06E-08	Up	5.55	1161	0.49	0.74
rs272827	27095	TRAPPC3	1	36296284	3.23E-01	11819	4.80E-01	Up	0.71	10573	0.49	0.03
rs13044870	112858	TP53RK	20	44737128	3.23E-01	11820	1.57E-01	Down	1.42	7766	0.49	0.08
rs4435039	4297	MLL	11	117802601	3.23E-01	11821	3.59E-01	Down	0.92	9671	0.49	0.04

rs2546199	22936	ELL2	5	95307847	3.24E-01	11822	7.48E-05	Up	3.96	2375	0.49	0.41
rs6647953	51260	CXorf26		75176768	3.24E-01	11823	6.09E-01	Down	0.51	11500	0.49	0.02
rs7087762	11319	ECD	10	74570127	3.24E-01	11824	1.40E-01	Up	1.48	7576	0.49	0.09
rs7087762	25961	NUDT13	10	74570127	3.24E-01	11825	7.14E-01	Down	0.37	12159	0.49	0.01
rs7143579	2764	GMFB	14	54010435	3.24E-01	11826	9.25E-01	Down	0.09	13399	0.49	0.00
rs1006957	7314	UBB	17	16222963	3.24E-01	11827	6.54E-01	Up	0.45	11792	0.49	0.02
rs12343206	5695	PSMB7	9	124263621	3.24E-01	11828	8.34E-07	Up	4.93	1553	0.49	0.61
rs3803172	83956	RACGAP1P	12	43745176	3.24E-01	11829	7.21E-01	Up	0.36	12205	0.49	0.01
rs8015976	122970	ACOT4	14	73124868	3.24E-01	11830	4.82E-02	Down	1.98	5955	0.49	0.13
rs2130248	886	CCKAR	4	26181205	3.24E-01	11831	2.23E-03	Down	3.06	3605	0.49	0.27
rs736574	7728	ZNF175	19	56746638	3.24E-01	11832	3.19E-01	Up	1.00	9349	0.49	0.05
rs4771260	27199	OXGR1	13	96447701	3.25E-01	11833	1.91E-02	Up	2.34	5025	0.49	0.17
rs6010952	79144	C20orf149	20	61618340	3.25E-01	11834	5.90E-03	Up	2.75	4165	0.49	0.22
rs17071357	5275	SERPINB13	18	59413347	3.25E-01	11835	9.80E-02	Up	1.65	6963	0.49	0.10
rs17728589	79801	SHCBP1	16	45199510	3.25E-01	11836	2.82E-05	Up	4.19	2157	0.49	0.46
rs7247246	79414	LRFN3	19	41133957	3.25E-01	11837	2.50E-01	Up	1.15	8764	0.49	0.06
rs26534	1176	AP3S1	5	115203600	3.25E-01	11838	6.80E-01	Up	0.41	11952	0.49	0.02
rs4319546	8608	RDH16	12	55633095	3.25E-01	11839	9.27E-02	Up	1.68	6879	0.49	0.10
rs805536	57148	KIAA1219	20	36551894	3.25E-01	11840	3.04E-02	Up	2.17	5476	0.49	0.15
rs13087701	116832	RPL39L	3	188322482	3.25E-01	11841	2.00E-02	Down	2.33	5074	0.49	0.17
rs6593937	54900	LAX1	1	200484936	3.25E-01	11842	2.08E-01	Up	1.26	8329	0.49	0.07
rs3737604	1122	CHML	1	238124672	3.25E-01	11843	1.92E-11	Up	6.71	672	0.49	1.07
rs3094550	26529	OR12D2	6	29462788	3.25E-01	11844	1.70E-01	Down	1.37	7919	0.49	0.08
rs7531583	65220	NADK	1	1738322	3.25E-01	11845	5.83E-02	Up	1.89	6200	0.49	0.12
rs3087615	9512	PMPCB	7	102546074	3.25E-01	11846	3.05E-06	Up	4.67	1740	0.49	0.55
rs2305862	27340	UTP20	12	100259998	3.25E-01	11847	2.77E-01	Up	1.09	9008	0.49	0.06
rs11083522	7538	ZFP36	19	44593451	3.25E-01	11848	4.25E-02	Up	2.03	5814	0.49	0.14
rs11083522	54623	PAF1	19	44593451	3.25E-01	11849	4.92E-02	Up	1.97	5975	0.49	0.13
rs1560514	158584	FAAH2		57269669	3.25E-01	11850	9.13E-05	Down	3.91	2428	0.49	0.40
rs3800794	10061	ABCF2	7	150354187	3.25E-01	11851	5.42E-01	Up	0.61	11030	0.49	0.03
rs9932951	3240	HP	16	70632842	3.26E-01	11852	5.65E-01	Up	0.58	11198	0.49	0.02
rs347515	3783	KCNN4	19	48961471	3.26E-01	11853	5.61E-05	Up	4.03	2318	0.49	0.43
rs347515	56006	C19orf61	19	48961471	3.26E-01	11854	7.08E-01	Down	0.37	12119	0.49	0.02
rs7833780	6132	RPL8	8	145981915	3.26E-01	11855	6.34E-06	Up	4.51	1855	0.49	0.52
rs7833780	340385	ZNF517	8	145981915	3.26E-01	11856	6.95E-02	Down	1.82	6415	0.49	0.12
rs6898507	134187	POU5F2	5	93108099	3.26E-01	11857	8.97E-01	Down	0.13	13228	0.49	0.00
rs1466260	3868	KRT16	17	37018570	3.26E-01	11858	1.50E-01	Down	1.44	7684	0.49	0.08
rs1043144	84437	KIAA1826	11	105384054	3.26E-01	11859	2.33E-01	Down	1.19	8589	0.49	0.06
rs4970986	9869	SETDB1	1	147721028	3.26E-01	11860	4.90E-04	Up	3.49	2954	0.49	0.33
rs3934711	80233	C17orf70	17	77143096	3.26E-01	11861	2.74E-03	Up	3.00	3741	0.49	0.26
rs6847164	8654	PDE5A	4	120892337	3.27E-01	11862	1.44E-02	Down	2.45	4779	0.49	0.18
rs11568454	9058	SLC13A2	17	23848283	3.27E-01	11863	4.44E-01	Up	0.77	10300	0.49	0.04
rs5468	3250	HPR	16	70645832	3.27E-01	11864	6.28E-01	Up	0.48	11641	0.49	0.02
rs4800718	143471	PSMA8	18	21970901	3.27E-01	11865	4.67E-01	Up	0.73	10483	0.49	0.03
rs2242295	4327	MMP19	12	54518443	3.27E-01	11866	1.10E-04	Down	3.87	2478	0.49	0.40
rs2242295	85406	DNAJC14	12	54518443	3.27E-01	11867	2.84E-02	Up	2.19	5410	0.49	0.15
rs2571030	162699	TCEB3C	18	42827852	3.27E-01	11868	8.86E-01	Down	0.14	13162	0.49	0.01
rs7042473	8555	CDC14B	9	96426125	3.27E-01	11869	1.08E-16	Down	8.30	323	0.48	1.60
rs4531246	5192	PEX10	1	2394182	3.28E-01	11870	9.42E-14	Up	7.46	466	0.48	1.30
rs1550868	144108	SPTY2D1	11	18628567	3.28E-01	11871	4.88E-01	Up	0.69	10634	0.48	0.03
rs371500	5704	PSMC4	19	45172763	3.28E-01	11872	5.55E-01	Up	0.59	11119	0.48	0.03
rs9350803	9324	HMGN3	6	79999595	3.28E-01	11873	8.40E-04	Down	3.34	3151	0.48	0.31
rs6599400	2261	FGFR3	4	1752256	3.28E-01	11874	3.07E-02	Up	2.16	5488	0.48	0.15
rs4335267	79742	CXorf36		44811643	3.28E-01	11875	5.00E-03	Down	2.81	4071	0.48	0.23
rs13165088	9631	NUP155	5	37385652	3.28E-01	11876	3.92E-02	Up	2.06	5731	0.48	0.14
rs1300253	55749	CCAR1	10	70172294	3.28E-01	11877	1.48E-02	Up	2.44	4801	0.48	0.18
rs2194898	4109	MAGEA10		150970202	3.29E-01	11878	4.85E-01	Down	0.70	10617	0.48	0.03
rs6625811	2833	CXCR3		70638539	3.29E-01	11879	7.55E-01	Up	0.31	12410	0.48	0.01
rs1916961	116093	DIRC1	2	189417390	3.29E-01	11880	2.66E-01	Up	1.11	8918	0.48	0.06
rs2572925	66004	LYNX1	8	143864907	3.29E-01	11881	7.56E-06	Down	4.48	1889	0.48	0.51
rs2572925	8581	LY6D	8	143864907	3.29E-01	11882	2.30E-02	Down	2.27	5199	0.48	0.16
rs268691	29950	SERTAD1	19	45641512	3.29E-01	11883	3.54E-01	Down	0.93	9625	0.48	0.05
rs903770	8739	HRK	12	115754677	3.29E-01	11884	6.25E-01	Down	0.49	11618	0.48	0.02
rs602345	51441	YTHDF2	1	28895908	3.29E-01	11885	4.46E-04	Up	3.51	2918	0.48	0.34
rs464921	6222	RPS18	6	33348484	3.30E-01	11886	4.10E-01	Up	0.82	10052	0.48	0.04
rs1781994	54521	WDR44		117251385	3.30E-01	11887	1.28E-01	Down	1.52	7429	0.48	0.09
rs1001413	22933	SIRT2	19	44058951	3.30E-01	11888	5.72E-03	Down	2.76	4153	0.48	0.22
rs4777499	23060	ZNF609	15	62721035	3.31E-01	11889	9.64E-02	Up	1.66	6932	0.48	0.10
rs11771619	222194	RSBN1L	7	77047929	3.31E-01	11890	4.77E-01	Down	0.71	10550	0.48	0.03
rs16891235	8366	HIST1H4B	6	26125521	3.31E-01	11891	7.54E-01	Up	0.31	12404	0.48	0.01
rs17304583	91646	TDRD12	19	37998062	3.31E-01	11892	6.30E-01	Down	0.48	11643	0.48	0.02
rs587771	222545	GPRC6A	6	117212212	3.31E-01	11893	5.30E-02	Up	1.93	6063	0.48	0.13
rs10493107	79717	PPCS	1	42578435	3.31E-01	11894	2.15E-02	Up	2.30	5141	0.48	0.17
rs10493107	84217	ZMYND12	1	42578435	3.31E-01	11895	6.98E-02	Up	1.81	6423	0.48	0.12
rs4073394	55004	C11orf59	11	71509887	3.31E-01	11896	6.98E-02	Up	1.81	6422	0.48	0.12
rs4073394	25906	C11orf51	11	71509887	3.31E-01	11897	9.46E-01	Down	0.07	13528	0.48	0.00
rs1050671	10945	KDELR1	19	53577730	3.31E-01	11898	1.54E-07	Up	5.25	1344	0.48	0.68
rs3745364	126006	PCP2	19	7605205	3.31E-01	11899	8.76E-02	Up	1.71	6766	0.48	0.11
rs4907251	26505	CNNM3	2	96906688	3.31E-01	11900	6.17E-04	Up	3.42	3039	0.48	0.32
rs4907251	26504	CNNM4	2	96906688	3.31E-01	11901	8.14E-01	Up	0.24	12746	0.48	0.01
rs10231529	81844	TRIM56	7	100314772	3.31E-01	11902	2.74E-01	Down	1.09	8985	0.48	0.06

rs3126254	158880	USP51		55391120	3.31E-01	11903	1.70E-01	Down	1.37	7913	0.48	0.08
rs8100801	6193	RPS5	19	63583872	3.31E-01	11904	2.64E-05	Up	4.20	2145	0.48	0.46
rs2440083	9712	USP6NL	10	11552654	3.32E-01	11905	9.82E-02	Up	1.65	6969	0.48	0.10
rs1045017	57095	C1orf128	1	23833434	3.32E-01	11906	3.36E-01	Down	0.96	9490	0.48	0.05
rs1045017	6924	TCEB3	1	23833434	3.32E-01	11907	8.51E-01	Down	0.19	12966	0.48	0.01
rs2304445	10749	KIF1C	17	4877622	3.32E-01	11908	7.62E-01	Up	0.30	12453	0.48	0.01
rs10277874	51530	ZC3HC1	7	129304087	3.32E-01	11909	3.85E-01	Up	0.87	9878	0.48	0.04
rs4514909	200504	GKN2	2	69089995	3.32E-01	11910	1.68E-01	Down	1.38	7901	0.48	0.08
rs1171554	4209	MEF2D	1	153287192	3.32E-01	11911	5.37E-01	Down	0.62	10991	0.48	0.03
rs1476810	9487	PIGL	17	16173942	3.32E-01	11912	1.88E-02	Up	2.35	5016	0.48	0.17
rs878749	4882	NPR2	9	35802623	3.33E-01	11913	2.00E-03	Down	3.09	3529	0.48	0.27
rs7260171	80110	ZNF614	19	57222307	3.33E-01	11914	7.91E-06	Up	4.47	1901	0.48	0.51
rs597210	149175	MANEAL	1	37915797	3.33E-01	11915	6.89E-23	Up	9.83	146	0.48	2.22
rs597210	284656	EPHA10	1	37915797	3.33E-01	11916	2.33E-02	Up	2.27	5216	0.48	0.16
rs2236876	8995	TNFSF18	1	169749143	3.33E-01	11917	8.28E-01	Down	0.22	12844	0.48	0.01
rs132793	4809	NHP2L1	22	40388181	3.33E-01	11918	1.79E-01	Down	1.35	8006	0.48	0.07
rs2436391	84418	C5orf32	5	139537569	3.33E-01	11919	3.27E-02	Down	2.14	5549	0.48	0.15
rs6729914	114790	STK11IP	2	220269021	3.33E-01	11920	1.29E-01	Up	1.52	7440	0.48	0.09
rs7259470	9149	DYRK1B	19	45021318	3.34E-01	11921	2.03E-01	Up	1.27	8284	0.48	0.07
rs476136	5100	PCDH8	13	52311499	3.34E-01	11922	4.79E-02	Up	1.98	5948	0.48	0.13
rs10898459	8726	EED	11	85650587	3.34E-01	11923	2.98E-02	Down	2.17	5455	0.48	0.15
rs944949	4969	OGN	9	92220168	3.34E-01	11924	8.35E-05	Down	3.93	2404	0.48	0.41
rs17132385	79441	C4orf15	4	2204788	3.34E-01	11925	4.22E-09	Up	5.88	997	0.48	0.84
rs550483	7469	WHSC2	4	1962216	3.34E-01	11926	1.41E-04	Up	3.81	2531	0.48	0.39
rs6643623	492	ATP2B3		152330589	3.34E-01	11927	4.96E-01	Down	0.68	10686	0.48	0.03
rs12520287	8611	PPAP2A	5	54882855	3.34E-01	11928	9.34E-05	Up	3.91	2432	0.48	0.40
rs2274969	11224	RPL35	9	124700928	3.35E-01	11929	2.06E-07	Up	5.19	1375	0.48	0.67
rs2274969	81873	ARPC5L	9	124700928	3.35E-01	11930	6.73E-03	Up	2.71	4235	0.48	0.22
rs10488192	54664	TMEM106B	7	12050321	3.35E-01	11931	2.54E-02	Down	2.23	5301	0.48	0.16
rs2630772	3223	HOXC6	12	52712788	3.35E-01	11932	3.11E-08	Up	5.53	1173	0.48	0.75
rs2630772	3222	HOXC5	12	52712788	3.35E-01	11933	6.63E-01	Down	0.44	11842	0.48	0.02
rs6816703	4717	NDUFC1	4	140574017	3.35E-01	11934	4.14E-02	Up	2.04	5783	0.48	0.14
rs6793102	51188	SS18L2	3	42592658	3.35E-01	11935	2.48E-08	Up	5.56	1155	0.48	0.76
rs3809790	84940	CORO6	17	24979666	3.35E-01	11936	2.75E-01	Up	1.09	8995	0.47	0.06
rs896086	51192	CKLF	16	65164689	3.35E-01	11937	1.45E-02	Up	2.44	4786	0.47	0.18
rs16963185	83636	C19orf12	19	34895712	3.35E-01	11938	1.85E-01	Up	1.33	8081	0.47	0.07
rs12653946	50805	IRX4	5	1948829	3.35E-01	11939	7.52E-02	Up	1.78	6532	0.47	0.11
rs4651133	81626	C1orf14	1	179620454	3.35E-01	11940	1.47E-01	Down	1.45	7647	0.47	0.08
rs448419	58487	CREBZF	11	85071886	3.35E-01	11941	8.70E-03	Up	2.62	4406	0.47	0.21
rs17299119	23553	HYAL4	7	123069623	3.35E-01	11942	1.17E-01	Up	1.57	7246	0.47	0.09
rs13069780	57650	KIAA1524	3	109760767	3.35E-01	11943	7.30E-01	Up	0.34	12250	0.47	0.01
rs9393777	6606	SMN1	6	27050006	3.36E-01	11944	2.48E-01	Up	1.16	8743	0.47	0.06
rs4858660	151651	EFHB	3	19969479	3.36E-01	11945	5.64E-01	Up	0.58	11193	0.47	0.02
rs6951350	10793	ZNF273	7	63799994	3.36E-01	11946	9.78E-01	Up	0.03	13758	0.47	0.00
rs549312	26580	BSCL2	11	62236422	3.36E-01	11947	2.77E-04	Up	3.64	2750	0.47	0.36
rs549312	283237	TTC9C	11	62236422	3.36E-01	11948	3.40E-01	Down	0.95	9526	0.47	0.05
rs549312	2785	GNG3	11	62236422	3.36E-01	11949	7.59E-01	Down	0.31	12432	0.47	0.01
rs599670	116071	BATF2	11	64527755	3.36E-01	11950	3.48E-02	Down	2.11	5617	0.47	0.15
rs4879849	25822	DNAJB5	9	34986014	3.36E-01	11951	9.22E-17	Down	8.31	319	0.47	1.60
rs11264236	4881	NPR1	1	150471553	3.37E-01	11952	9.26E-01	Down	0.09	13400	0.47	0.00
rs11793787	5730	PTGDS	9	137136468	3.37E-01	11953	2.52E-12	Down	7.00	593	0.47	1.16
rs2280964	93953	ACRC		70621075	3.37E-01	11954	3.41E-01	Down	0.95	9535	0.47	0.05
rs7558608	10575	CCT4	2	62032316	3.37E-01	11955	5.93E-05	Up	4.02	2330	0.47	0.42
rs7958174	317781	DDX51	12	131292898	3.37E-01	11956	8.19E-01	Up	0.23	12783	0.47	0.01
rs7958174	79050	NOC4L	12	131292898	3.37E-01	11957	8.91E-01	Down	0.14	13191	0.47	0.01
rs1852015	51351	ZNF117	7	63881130	3.37E-01	11958	2.39E-04	Up	3.67	2692	0.47	0.36
rs11793897	29979	UBQLN1	9	83562397	3.37E-01	11959	1.63E-02	Up	2.40	4904	0.47	0.18
rs6588502	127428	C1orf83	1	54275177	3.37E-01	11960	5.48E-01	Up	0.60	11069	0.47	0.03
rs898085	8877	SPHK1	17	71905276	3.37E-01	11961	5.57E-01	Down	0.59	11133	0.47	0.03
rs4151374	4331	MNAT1	14	60471706	3.37E-01	11962	8.69E-01	Down	0.16	13059	0.47	0.01
rs1880661	141	ADPRH	3	120761538	3.38E-01	11963	2.25E-02	Down	2.28	5182	0.47	0.16
rs1982288	8520	HAT1	2	172647246	3.38E-01	11964	7.46E-04	Up	3.37	3107	0.47	0.31
rs300168	348654	GEN1	2	17884078	3.38E-01	11965	4.50E-01	Up	0.76	10344	0.47	0.03
rs12001	100128782	C9orf130	9	95716985	3.38E-01	11966	5.78E-02	Down	1.90	6182	0.47	0.12
rs6018954	4826	NNAT	20	35566360	3.38E-01	11967	2.51E-03	Up	3.02	3688	0.47	0.26
rs3170660	63906	GPATCH3	1	26894863	3.39E-01	11968	1.01E-01	Down	1.64	7031	0.47	0.10
rs17563587	10380	BPNT1	1	216670703	3.39E-01	11969	1.14E-01	Up	1.58	7198	0.47	0.09
rs4348312	51645	PPIL1	6	36940006	3.39E-01	11970	6.60E-02	Up	1.84	6360	0.47	0.12
rs17760953	254773	LYG2	2	99318381	3.39E-01	11971	6.50E-01	Down	0.45	11773	0.47	0.02
rs3809470	29933	GPR132	14	104602990	3.39E-01	11972	4.47E-01	Down	0.76	10324	0.47	0.04
rs4489379	29989	OBP2B	9	133114495	3.39E-01	11973	9.41E-02	Down	1.67	6900	0.47	0.10
rs11240426	55220	KLHDC8A	1	202033679	3.40E-01	11974	1.21E-01	Up	1.55	7308	0.47	0.09
rs8071435	1398	CRK	17	1312497	3.40E-01	11975	1.39E-03	Down	3.20	3346	0.47	0.29
rs16968987	8161	COIL	17	52365061	3.40E-01	11976	3.57E-01	Up	0.92	9646	0.47	0.04
rs2277324	196403	DTX3	12	56299442	3.40E-01	11977	3.74E-04	Down	3.56	2858	0.47	0.34
rs7206076	54768	HYDIN	16	69626176	3.41E-01	11978	5.30E-01	Down	0.63	10939	0.47	0.03
rs1863898	6229	RPS24	10	79449156	3.41E-01	11979	4.94E-07	Up	5.03	1478	0.47	0.63
rs2861442	6387	CXCL12	10	44204965	3.41E-01	11980	1.40E-04	Down	3.81	2527	0.47	0.39
rs4646018	23341	DNAJC16	1	15585680	3.41E-01	11981	5.32E-02	Up	1.93	6065	0.47	0.13
rs3010110	10726	NUDC	1	26962663	3.41E-01	11982	3.11E-04	Down	3.61	2789	0.47	0.35
rs3010110	126695	C1orf172	1	26962663	3.41E-01	11983	6.62E-02	Up	1.84	6364	0.47	0.12

rs4658973	10885	WDR3	1	118211096	3.41E-01	11984	2.86E-10	Up	6.43	769	0.47	0.95
rs12961029	1830	DSG3	18	27263407	3.41E-01	11985	1.71E-01	Down	1.37	7935	0.47	0.08
rs758335	51734	SEPX1	16	1951127	3.41E-01	11986	9.80E-03	Down	2.58	4494	0.47	0.20
rs6683473	29949	IL19	1	203355547	3.42E-01	11987	9.43E-01	Up	0.07	13513	0.47	0.00
rs40401	1437	CSF2	5	131424377	3.42E-01	11988	2.21E-02	Up	2.29	5165	0.47	0.17
rs40401	3562	IL3	5	131424377	3.42E-01	11989	4.37E-02	Up	2.02	5844	0.47	0.14
rs11707101	57599	WDR48	3	39093108	3.42E-01	11990	9.93E-03	Down	2.58	4504	0.47	0.20
rs3130100	6892	TAPBP	6	33391744	3.42E-01	11991	2.41E-04	Up	3.67	2696	0.47	0.36
rs7055282	6170	RPL39		118705058	3.42E-01	11992	7.27E-04	Up	3.38	3090	0.47	0.31
rs7742164	4829	NMBR	6	142440944	3.42E-01	11993	7.40E-01	Down	0.33	12316	0.47	0.01
rs131654	7332	UBE2L3	22	20241744	3.42E-01	11994	1.18E-02	Down	2.52	4627	0.47	0.19
rs12145743	79590	MRPL24	1	153513724	3.43E-01	11995	6.51E-03	Up	2.72	4213	0.47	0.22
rs623318	7415	VCP	9	35053789	3.43E-01	11996	8.34E-03	Up	2.64	4368	0.46	0.21
rs10986626	5537	PPP6C	9	125027875	3.43E-01	11997	8.60E-04	Up	3.33	3157	0.46	0.31
rs10986626	10244	RABEPK	9	125027875	3.43E-01	11998	3.62E-01	Up	0.91	9691	0.46	0.04
rs16887011	80139	ZNF703	8	37657180	3.43E-01	11999	2.41E-01	Down	1.17	8669	0.46	0.06
rs1617105	9587	MAD2L1BP	6	43721158	3.43E-01	12000	9.03E-02	Down	1.69	6832	0.46	0.10
rs1617105	54676	GTPBP2	6	43721158	3.43E-01	12001	1.44E-01	Down	1.46	7615	0.46	0.08
rs671892	8498	RANBP3	19	5920186	3.44E-01	12002	1.17E-02	Down	2.52	4620	0.46	0.19
rs6587557	79005	SCNM1	1	147950217	3.44E-01	12003	2.73E-01	Down	1.10	8982	0.46	0.06
rs6587557	388695	LYSMD1	1	147950217	3.44E-01	12004	6.14E-01	Up	0.50	11535	0.46	0.02
rs6587557	29765	TMOD4	1	147950217	3.44E-01	12005	6.81E-01	Up	0.41	11956	0.46	0.02
rs2812094	6751	SSTR1	14	37736641	3.44E-01	12006	2.34E-01	Up	1.19	8601	0.46	0.06
rs2303933	10922	FASTK	7	150204447	3.44E-01	12007	6.80E-02	Up	1.83	6395	0.46	0.12
rs2306192	9466	IL27RA	19	14004016	3.45E-01	12008	5.77E-02	Down	1.90	6178	0.46	0.12
rs2306192	117579	RLN3	19	14004016	3.45E-01	12009	7.44E-01	Down	0.33	12337	0.46	0.01
rs7682616	7700	ZNF141	4	342955	3.45E-01	12010	3.01E-03	Up	2.97	3778	0.46	0.25
rs11043310	84876	ORAI1	12	120552079	3.45E-01	12011	6.14E-06	Up	4.52	1847	0.46	0.52
rs988132	28461	IGHV1-69	14	106256042	3.45E-01	12012	3.12E-02	Up	2.16	5499	0.46	0.15
rs1530864	5170	PDPK1	2	173286310	3.45E-01	12013	3.90E-02	Up	2.06	5729	0.46	0.14
rs1530864	5163	PDK1	2	173286310	3.45E-01	12014	4.87E-02	Up	1.97	5965	0.46	0.13
rs17201246	3836	KPNA1	3	123628160	3.45E-01	12015	9.47E-01	Up	0.07	13533	0.46	0.00
rs7568449	54529	ASNSD1	2	190346560	3.45E-01	12016	5.23E-01	Down	0.64	10885	0.46	0.03
rs3789724	84172	POLR1B	2	113030025	3.45E-01	12017	8.44E-02	Up	1.73	6709	0.46	0.11
rs4076557	2147	F2	11	46721225	3.46E-01	12018	8.60E-03	Up	2.63	4394	0.46	0.21
rs4305581	6731	SRP72	4	57220461	3.46E-01	12019	8.12E-20	Up	9.07	210	0.46	1.91
rs6565624	6182	MRPL12	17	77288155	3.46E-01	12020	4.56E-07	Up	5.04	1468	0.46	0.63
rs6565624	1468	SLC25A10	17	77288155	3.46E-01	12021	6.49E-03	Up	2.72	4210	0.46	0.22
rs3909468	1601	DAB2	5	39446888	3.46E-01	12022	4.02E-07	Up	5.07	1456	0.46	0.64
rs529949	1805	DPT	1	165423642	3.46E-01	12023	1.08E-14	Down	7.73	412	0.46	1.40
rs2955245	1442	CSH1	17	59333577	3.46E-01	12024	4.71E-01	Down	0.72	10505	0.46	0.03
rs4815566	114548	NLRP3	20	3029638	3.46E-01	12025	6.28E-02	Up	1.86	6293	0.46	0.12
rs4815566	551	AVP	20	3029638	3.46E-01	12026	1.50E-01	Down	1.44	7680	0.46	0.08
rs556507	63901	FAM111A	11	58687412	3.46E-01	12027	1.41E-03	Up	3.19	3353	0.46	0.29
rs370655	2921	CXCL3	4	75268169	3.46E-01	12028	9.03E-02	Up	1.69	6826	0.46	0.10
rs12129555	9214	FAIM3	1	203489659	3.46E-01	12029	3.40E-01	Up	0.95	9521	0.46	0.05
rs6918969	4800	NFYA	6	41157873	3.47E-01	12030	2.49E-09	Up	5.96	948	0.46	0.86
rs6918969	221443	C6orf130	6	41157873	3.47E-01	12031	5.89E-07	Down	4.99	1501	0.46	0.62
rs10521014	1549	CYP2A7	19	46084725	3.47E-01	12032	1.78E-02	Down	2.37	4974	0.46	0.17
rs5945108	139716	GAB3		153468101	3.47E-01	12033	1.87E-01	Up	1.32	8115	0.46	0.07
rs3830140	84219	WDR24	16	665336	3.47E-01	12034	5.40E-01	Down	0.61	11009	0.46	0.03
rs2715694	50852	TRAT1	3	110023833	3.47E-01	12035	6.90E-03	Up	2.70	4250	0.46	0.22
rs788158	1745	DLX1	2	172768404	3.47E-01	12036	1.73E-10	Up	6.38	789	0.46	0.98
rs854762	1819	DRG2	17	17949827	3.48E-01	12037	1.78E-02	Up	2.37	4976	0.46	0.17
rs6681027	79871	RPAP2	1	92558604	3.48E-01	12038	6.53E-04	Up	3.41	3057	0.46	0.32
rs302923	9329	GTF3C4	9	132602201	3.48E-01	12039	1.83E-01	Up	1.33	8058	0.46	0.07
rs11094949	1968	EIF2S3		23864303	3.48E-01	12040	1.48E-11	Up	6.75	651	0.46	1.08
rs9420	280636	C11orf31	11	57266870	3.48E-01	12041	5.83E-05	Up	4.02	2325	0.46	0.42
rs10483158	150280	HORMAD2	22	28899717	3.48E-01	12042	9.15E-01	Up	0.11	13346	0.46	0.00
rs13117427	60559	SPCS3	4	177630556	3.48E-01	12043	2.65E-06	Up	4.70	1714	0.46	0.56
rs1135205	64577	ALDH8A1	6	135323488	3.48E-01	12044	8.58E-01	Up	0.18	13002	0.46	0.01
rs474600	81559	TRIM11	1	224892572	3.49E-01	12045	1.74E-02	Up	2.38	4956	0.46	0.18
rs474600	51127	TRIM17	1	224892572	3.49E-01	12046	5.25E-01	Down	0.64	10907	0.46	0.03
rs13127257	56955	MEPE	4	89122695	3.49E-01	12047	3.14E-02	Down	2.15	5514	0.46	0.15
rs12461941	148268	ZNF570	19	42676490	3.49E-01	12048	6.52E-01	Down	0.45	11784	0.46	0.02
rs3788337	2781	GNAZ	22	21736571	3.49E-01	12049	1.25E-02	Down	2.50	4660	0.46	0.19
rs7225351	140735	DYNLL2	17	53510010	3.49E-01	12050	7.74E-02	Up	1.77	6583	0.46	0.11
rs1770372	50604	IL20	1	203424042	3.49E-01	12051	8.05E-02	Down	1.75	6637	0.46	0.11
rs3744357	3980	LIG3	17	30358225	3.49E-01	12052	1.25E-13	Up	7.43	474	0.46	1.29
rs13030474	518	ATP5G3	2	175877010	3.49E-01	12053	1.44E-01	Up	1.46	7617	0.46	0.08
rs13030474	1386	ATF2	2	175877010	3.49E-01	12054	6.87E-01	Down	0.40	11988	0.46	0.02
rs2304497	47	ACLY	17	37319300	3.49E-01	12055	2.81E-12	Up	6.99	600	0.46	1.16
rs9876490	287015	TRIM42	3	141889958	3.50E-01	12056	5.20E-01	Up	0.64	10861	0.46	0.03
rs2530685	164633	CABP7	22	28431213	3.50E-01	12057	5.57E-01	Up	0.59	11132	0.46	0.03
rs8104242	11338	U2AF2	19	60882627	3.50E-01	12058	4.84E-03	Down	2.82	4041	0.46	0.23
rs17655652	29881	NPC1L1	7	44354231	3.50E-01	12059	1.28E-02	Up	2.49	4676	0.46	0.19
rs730821	23411	SIRT1	10	69297765	3.50E-01	12060	4.84E-01	Down	0.70	10601	0.46	0.03
rs6850417	55584	CHRNA9	4	40206798	3.51E-01	12061	3.13E-01	Down	1.01	9293	0.45	0.05
rs12573841	51021	MRPS16	10	74670745	3.51E-01	12062	5.46E-03	Up	2.78	4123	0.45	0.23
rs12573841	23234	DNAJC9	10	74670745	3.51E-01	12063	7.32E-03	Up	2.68	4286	0.45	0.21
rs4251425	51135	IRAK4	12	42440176	3.51E-01	12064	1.78E-01	Down	1.35	7999	0.45	0.07

rs6096210	8813	DPM1	20	49016774	3.51E-01	12065	1.51E-01	Down	1.43	7695	0.45	0.08
rs6096210	27304	MOCS3	20	49016774	3.51E-01	12066	3.88E-01	Down	0.86	9904	0.45	0.04
rs11135990	23087	TRIM35	8	27192033	3.51E-01	12067	9.52E-02	Down	1.67	6911	0.45	0.10
rs4478405	896	CCND3	6	42035791	3.52E-01	12068	9.47E-03	Down	2.59	4469	0.45	0.20
rs6074018	63905	MANBAL	20	35358710	3.52E-01	12069	1.49E-06	Down	4.81	1625	0.45	0.58
rs213621	164091	PAQR7	1	25891386	3.52E-01	12070	4.43E-02	Down	2.01	5859	0.45	0.14
rs7192268	55308	DDX19A	16	68939680	3.52E-01	12071	8.19E-03	Down	2.64	4357	0.45	0.21
rs7309966	363	AQP6	12	48665934	3.52E-01	12072	6.35E-01	Down	0.48	11671	0.45	0.02
rs7340453	10797	MTHFD2	2	74339670	3.52E-01	12073	3.37E-17	Up	8.43	298	0.45	1.65
rs4825655	7319	UBE2A		118503301	3.53E-01	12074	2.80E-04	Down	3.63	2754	0.45	0.36
rs4825655	55922	NKRF		118503301	3.53E-01	12075	3.01E-02	Up	2.17	5467	0.45	0.15
rs538399	3487	IGFBP4	17	35864191	3.53E-01	12076	3.96E-06	Down	4.61	1779	0.45	0.54
rs17758259	1768	DNAH6	2	84670740	3.53E-01	12077	4.53E-01	Up	0.75	10368	0.45	0.03
rs17410643	5825	ABCD3	1	94653911	3.53E-01	12078	1.94E-03	Down	3.10	3516	0.45	0.27
rs6573908	6430	SFRS5	14	69308717	3.53E-01	12079	5.28E-02	Up	1.94	6060	0.45	0.13
rs11573534	170302	ARX		24774441	3.53E-01	12080	9.77E-01	Up	0.03	13742	0.45	0.00
rs1043424	1650	DDOST	1	20722306	3.53E-01	12081	1.04E-16	Up	8.30	322	0.45	1.60
rs1043424	65018	PINK1	1	20722306	3.53E-01	12082	4.39E-11	Down	6.59	704	0.45	1.04
rs907482	65082	VPS33A	12	121271903	3.54E-01	12083	4.75E-01	Up	0.71	10540	0.45	0.03
rs7316493	9735	KNTC1	12	121641558	3.54E-01	12084	1.40E-03	Up	3.19	3349	0.45	0.29
rs12983988	7576	ZNF28	19	57989578	3.54E-01	12085	8.19E-01	Up	0.23	12786	0.45	0.01
rs548355	10022	INSL5	1	66987048	3.54E-01	12086	5.78E-01	Down	0.56	11297	0.45	0.02
rs889548	5652	PRSS8	16	31045213	3.54E-01	12087	6.67E-13	Up	7.19	538	0.45	1.22
rs12123002	57829	ZP4	1	234390771	3.54E-01	12088	1.08E-01	Down	1.61	7121	0.45	0.10
rs328902	23333	DPY19L1	7	34794083	3.54E-01	12089	7.65E-01	Down	0.30	12462	0.45	0.01
rs2207701	7812	CSDE1	1	115021554	3.55E-01	12090	5.51E-01	Up	0.60	11083	0.45	0.03
rs13264654	51236	C8orf30A	8	145247517	3.55E-01	12091	4.26E-03	Up	2.86	3975	0.45	0.24
rs13264654	81858	SHARPIN	8	145247517	3.55E-01	12092	2.12E-01	Up	1.25	8379	0.45	0.07
rs13264654	340390	KIAA1875	8	145247517	3.55E-01	12093	9.80E-01	Up	0.03	13772	0.45	0.00
rs255301	662	BNIP1	5	172519158	3.55E-01	12094	8.79E-01	Up	0.15	13121	0.45	0.01
rs11162567	431707	LHX8	1	75305896	3.55E-01	12095	6.45E-01	Up	0.46	11744	0.45	0.02
rs9830791	353324	SPATA12	3	57066615	3.55E-01	12096	9.62E-03	Up	2.59	4478	0.45	0.20
rs2675679	5328	PLAU	10	75328587	3.55E-01	12097	9.14E-02	Down	1.69	6851	0.45	0.10
rs6499157	54920	DUS2L	16	66662975	3.55E-01	12098	2.37E-01	Down	1.18	8633	0.45	0.06
rs2405378	84654	SPZ1	5	79658228	3.56E-01	12099	2.62E-01	Down	1.12	8878	0.45	0.06
rs3794170	8050	PDHX	11	34893714	3.56E-01	12100	8.33E-01	Down	0.21	12864	0.45	0.01
rs10911905	5743	PTGS2	1	183384052	3.56E-01	12101	7.23E-06	Down	4.49	1876	0.45	0.51
rs7608304	6697	SPR	2	73040336	3.56E-01	12102	5.03E-01	Up	0.67	10734	0.45	0.03
rs366542	637	BID	22	16632936	3.56E-01	12103	3.11E-03	Up	2.96	3795	0.45	0.25
rs2445192	117196	MRGPRX4	11	18156606	3.56E-01	12104	9.02E-01	Up	0.12	13260	0.45	0.00
rs2642219	29935	RPA4		95945551	3.56E-01	12105	3.07E-02	Up	2.16	5487	0.45	0.15
rs11672440	126231	ZNF573	19	42942798	3.56E-01	12106	1.46E-01	Up	1.45	7642	0.45	0.08
rs11604602	80975	TMPRSS5	11	113068898	3.56E-01	12107	7.70E-01	Down	0.29	12492	0.45	0.01
rs100774	4976	OPA1	3	194841436	3.56E-01	12108	2.22E-07	Down	5.18	1386	0.45	0.67
rs100774	9968	MED12	3	194841436	3.56E-01	12109	2.11E-01	Down	1.25	8371	0.45	0.07
rs379527	8740	TNFSF14	19	6627442	3.56E-01	12110	2.48E-01	Up	1.15	8749	0.45	0.06
rs11753326	135656	DPCR1	6	31033964	3.56E-01	12111	3.71E-01	Down	0.89	9775	0.45	0.04
rs13294	1893	ECM1	1	147298060	3.56E-01	12112	4.45E-13	Down	7.24	523	0.45	1.24
rs13294	80222	TARS2	1	147298060	3.56E-01	12113	6.81E-01	Up	0.41	11955	0.45	0.02
rs652265	64122	FN3K	17	78311676	3.56E-01	12114	6.08E-01	Down	0.51	11490	0.45	0.02
rs4466388	7994	MYST3	8	42046343	3.57E-01	12115	1.11E-04	Down	3.87	2480	0.45	0.40
rs928948	2011	MARK2	11	63396457	3.57E-01	12116	1.57E-01	Up	1.41	7772	0.45	0.08
rs2475298	11196	SEC23IP	10	121669003	3.57E-01	12117	1.33E-03	Up	3.21	3335	0.45	0.29
rs735830	1773	DNASE1	16	3635355	3.57E-01	12118	1.59E-02	Up	2.41	4872	0.45	0.18
rs3810794	899	CCNF	16	2452524	3.57E-01	12119	3.63E-01	Down	0.91	9704	0.45	0.04
rs3810794	80178	C16orf59	16	2452524	3.57E-01	12120	9.36E-01	Up	0.08	13466	0.45	0.00
rs5936033	11238	CA5B		15508688	3.57E-01	12121	7.58E-01	Up	0.31	12428	0.45	0.01
rs849165	644	BLVRA	7	43600654	3.57E-01	12122	6.12E-02	Down	1.87	6261	0.45	0.12
rs8073976	10882	C1QL1	17	40388751	3.57E-01	12123	7.76E-01	Down	0.28	12532	0.45	0.01
rs7034424	2619	GAS1	9	86804456	3.57E-01	12124	1.81E-33	Down	12.06	47	0.45	3.27
rs7978288	4673	NAP1L1	12	74726001	3.57E-01	12125	6.70E-02	Up	1.83	6375	0.45	0.12
rs7978288	22822	PHLDA1	12	74726001	3.57E-01	12126	3.02E-01	Down	1.03	9201	0.45	0.05
rs2241469	9948	WDR1	4	9756731	3.58E-01	12127	8.37E-10	Down	6.14	880	0.45	0.91
rs2806566	7798	LUZP1	1	23252478	3.58E-01	12128	1.00E-01	Down	1.64	7002	0.45	0.10
rs2820579	89886	SLAMF9	1	156733059	3.58E-01	12129	7.91E-01	Down	0.27	12610	0.45	0.01
rs4919115	26287	ANKRD2	10	99338825	3.58E-01	12130	5.32E-01	Down	0.63	10952	0.45	0.03
rs1996455	56978	PRDM8	4	81463361	3.59E-01	12131	3.15E-01	Down	1.00	9325	0.45	0.05
rs1283	7866	IFRD2	3	50312426	3.59E-01	12132	7.58E-07	Up	4.95	1531	0.44	0.61
rs1283	24142	NAT6	3	50312426	3.59E-01	12133	1.49E-02	Up	2.43	4811	0.44	0.18
rs1283	3373	HYAL1	3	50312426	3.59E-01	12134	3.00E-02	Down	2.17	5464	0.44	0.15
rs1283	8372	HYAL3	3	50312426	3.59E-01	12135	3.44E-02	Up	2.12	5606	0.44	0.15
rs12288706	120066	OR5P3	11	7795017	3.59E-01	12136	4.28E-01	Up	0.79	10177	0.44	0.04
rs12288706	120065	OR5P2	11	7795017	3.59E-01	12137	8.77E-01	Down	0.15	13099	0.44	0.01
rs268822	55195	C14orf105	14	57045399	3.59E-01	12138	7.62E-02	Up	1.77	6553	0.44	0.11
rs6089922	5753	PTK6	20	61650561	3.59E-01	12139	1.80E-03	Up	3.12	3478	0.44	0.27
rs7209138	3857	KRT9	17	36973917	3.59E-01	12140	7.02E-01	Down	0.38	12086	0.44	0.02
rs12375332	6386	SDCBP	8	59622612	3.60E-01	12141	1.26E-02	Up	2.50	4662	0.44	0.19
rs4428010	79853	TM4SF20	2	228052599	3.60E-01	12142	3.96E-01	Down	0.85	9952	0.44	0.04
rs9915181	84282	RNF135	17	26307820	3.60E-01	12143	8.75E-03	Up	2.62	4409	0.44	0.21
rs2469534	64843	ISL2	15	74396778	3.60E-01	12144	1.10E-03	Down	3.26	3256	0.44	0.30
rs7731320	84240	ZCCHC9	5	80618615	3.60E-01	12145	4.86E-01	Down	0.70	10620	0.44	0.03

rs13376108	2959	GTF2B	1	89062762	3.60E-01	12146	5.48E-01	Down	0.60	11064	0.44	0.03
rs10130183	5684	PSMA3	14	57768091	3.61E-01	12147	9.43E-01	Up	0.07	13510	0.44	0.00
rs1205171	10535	RNASEH2A	19	12772340	3.61E-01	12148	8.62E-02	Up	1.72	6741	0.44	0.11
rs1205171	7001	PRDX2	19	12772340	3.61E-01	12149	1.08E-01	Up	1.61	7126	0.44	0.10
rs1205171	3726	JUNB	19	12772340	3.61E-01	12150	7.87E-01	Up	0.27	12592	0.44	0.01
rs10446729	4085	MAD2L1	4	121346155	3.61E-01	12151	2.34E-05	Up	4.23	2115	0.44	0.46
rs9286999	286826	LIN9	1	222801250	3.61E-01	12152	2.94E-01	Up	1.05	9145	0.44	0.05
rs12709744	55520	ELAC1	18	46778159	3.61E-01	12153	4.34E-02	Down	2.02	5835	0.44	0.14
rs12981904	9592	IER2	19	13109856	3.61E-01	12154	1.51E-06	Up	4.81	1628	0.44	0.58
rs769430	4994	OR3A1	17	3161719	3.61E-01	12155	6.04E-01	Up	0.52	11463	0.44	0.02
rs7968222	65117	RSRC2	12	121555668	3.61E-01	12156	2.66E-01	Up	1.11	8914	0.44	0.06
rs3864937	79600	TCTN1	12	109545804	3.62E-01	12157	3.97E-03	Up	2.88	3924	0.44	0.24
rs6657670	54856	GON4L	1	152600940	3.62E-01	12158	5.58E-01	Up	0.59	11139	0.44	0.03
rs16967511	11136	SLC7A9	19	38050367	3.62E-01	12159	9.48E-01	Up	0.07	13536	0.44	0.00
rs4728533	5447	POR	7	75231187	3.62E-01	12160	3.23E-01	Down	0.99	9390	0.44	0.05
rs849815	83417	FCRL4	1	154355235	3.62E-01	12161	6.38E-01	Up	0.47	11689	0.44	0.02
rs11804831	118424	UBE2J2	1	1234727	3.62E-01	12162	5.24E-03	Down	2.79	4101	0.44	0.23
rs2858016	3039	HBA1	16	175490	3.62E-01	12163	1.15E-01	Up	1.58	7217	0.44	0.09
rs2858016	3049	HBQ1	16	175490	3.62E-01	12164	9.13E-01	Up	0.11	13335	0.44	0.00
rs12705918	5506	PPP1R3A	7	113107988	3.63E-01	12165	1.33E-01	Up	1.50	7475	0.44	0.09
rs687059	115362	GBP5	1	89458038	3.63E-01	12166	9.25E-02	Down	1.68	6872	0.44	0.10
rs2816323	163859	C1orf55	1	222490329	3.63E-01	12167	1.22E-02	Up	2.51	4647	0.44	0.19
rs5982868	8908	GYG2		2725457	3.63E-01	12168	1.32E-02	Down	2.48	4704	0.44	0.19
rs1404511	7979	SHFM1	7	95996494	3.63E-01	12169	1.26E-01	Up	1.53	7390	0.44	0.09
rs17117895	3679	ITGA7	12	54384305	3.63E-01	12170	3.27E-09	Down	5.92	981	0.44	0.85
rs17117895	5959	RDH5	12	54384305	3.63E-01	12171	2.55E-02	Down	2.23	5303	0.44	0.16
rs17117895	2647	BLOC1S1	12	54384305	3.63E-01	12172	6.10E-01	Up	0.51	11508	0.44	0.02
rs2274636	10730	YME1L1	10	27483018	3.63E-01	12173	4.19E-01	Down	0.81	10111	0.44	0.04
rs3744391	4239	MFAP4	17	19247759	3.63E-01	12174	8.17E-04	Down	3.35	3144	0.44	0.31
rs12420402	6150	MRPL23	11	1943621	3.63E-01	12175	2.80E-07	Up	5.14	1408	0.44	0.66
rs17463708	152518	NFXL1	4	47726942	3.64E-01	12176	2.89E-14	Up	7.59	441	0.44	1.35
rs9393638	81853	TMEM14B	6	10866876	3.64E-01	12177	3.73E-01	Up	0.89	9789	0.44	0.04
rs3103174	11146	GLMN	1	92429687	3.64E-01	12178	5.70E-01	Down	0.57	11241	0.44	0.02
rs17578859	9022	CLIC3	9	137155007	3.64E-01	12179	1.64E-03	Down	3.15	3431	0.44	0.28
rs7745519	4224	MEP1A	6	46849690	3.64E-01	12180	3.52E-02	Up	2.11	5629	0.44	0.15
rs2847117	6632	SNRPD1	18	17462849	3.64E-01	12181	2.09E-01	Up	1.26	8341	0.44	0.07
rs12163565	8314	BAP1	3	52405566	3.64E-01	12182	1.69E-01	Up	1.38	7908	0.44	0.08
rs10201535	51078	THAP4	2	242237956	3.65E-01	12183	6.83E-01	Up	0.41	11967	0.44	0.02
rs17782975	7158	TP53BP1	15	41585331	3.65E-01	12184	7.35E-01	Up	0.34	12287	0.44	0.01
rs11164839	8317	CDC7	1	91674848	3.65E-01	12185	7.63E-02	Up	1.77	6558	0.44	0.11
rs7246498	57573	ZNF471	19	61699460	3.65E-01	12186	4.20E-01	Up	0.81	10121	0.44	0.04
rs1529717	80776	B9D2	19	46575038	3.65E-01	12187	1.22E-01	Down	1.55	7326	0.44	0.09
rs230260	84063	KIRREL2	19	41050403	3.65E-01	12188	4.24E-02	Down	2.03	5808	0.44	0.14
rs230260	4868	NPHS1	19	41050403	3.65E-01	12189	4.74E-01	Up	0.72	10525	0.44	0.03
rs230260	333	APLP1	19	41050403	3.65E-01	12190	9.68E-01	Down	0.04	13690	0.44	0.00
rs2073015	222642	BZRPL1	6	41116725	3.65E-01	12191	1.79E-01	Up	1.34	8011	0.44	0.07
rs2073015	10930	APOBEC2	6	41116725	3.65E-01	12192	7.16E-01	Down	0.36	12173	0.44	0.01
rs2073015	222643	UNC5CL	6	41116725	3.65E-01	12193	9.93E-01	Down	0.01	13864	0.44	0.00
rs6523526	9823	ARMCX2		100724055	3.65E-01	12194	2.96E-06	Down	4.67	1735	0.44	0.55
rs12726763	2268	FGR	1	27642387	3.66E-01	12195	8.79E-01	Up	0.15	13115	0.44	0.01
rs3748671	725	C4BPB	1	203641426	3.66E-01	12196	3.47E-02	Up	2.11	5616	0.44	0.15
rs9865965	523	ATP6V1A	3	114933185	3.67E-01	12197	6.78E-07	Up	4.97	1518	0.44	0.62
rs501700	64645	HIAT1	1	100232332	3.67E-01	12198	1.98E-01	Up	1.29	8235	0.44	0.07
rs12570134	2018	EMX2	10	119274398	3.67E-01	12199	4.79E-01	Down	0.71	10566	0.44	0.03
rs3749894	7259	TSPYL1	6	116707146	3.67E-01	12200	6.57E-02	Down	1.84	6356	0.44	0.12
rs1443434	2304	FOXE1	9	97697034	3.67E-01	12201	6.32E-02	Up	1.86	6302	0.44	0.12
rs10448143	51160	VPS28	8	145644192	3.67E-01	12202	7.72E-01	Up	0.29	12507	0.44	0.01
rs17204376	53829	P2RY13	3	152518659	3.67E-01	12203	1.09E-01	Down	1.60	7132	0.43	0.10
rs10441723	25853	WDR40A	9	34072144	3.67E-01	12204	4.00E-08	Up	5.49	1207	0.43	0.74
rs10797007	910	CD1B	1	155108702	3.68E-01	12205	4.57E-01	Up	0.74	10405	0.43	0.03
rs867186	10544	PROCR	20	33228215	3.68E-01	12206	8.09E-05	Down	3.94	2395	0.43	0.41
rs4781070	116028	C16orf75	16	11358802	3.68E-01	12207	1.28E-01	Up	1.52	7426	0.43	0.09
rs3810168	147744	TMEM190	19	60588521	3.68E-01	12208	9.89E-01	Up	0.01	13837	0.43	0.00
rs542998	10313	RTN3	11	63243962	3.68E-01	12209	5.44E-04	Up	3.46	2990	0.43	0.33
rs2074205	55659	ZNF416	19	62793122	3.68E-01	12210	3.75E-01	Up	0.89	9803	0.43	0.04
rs5979742	5634	PRPS2		12593163	3.68E-01	12211	4.07E-02	Down	2.05	5765	0.43	0.14
rs738304	29775	CARD10	22	36226677	3.68E-01	12212	7.65E-02	Down	1.77	6563	0.43	0.11
rs738304	4242	MFNG	22	36226677	3.68E-01	12213	9.84E-01	Down	0.02	13807	0.43	0.00
rs11146249	10570	DPYSL4	10	133870687	3.68E-01	12214	2.53E-01	Up	1.14	8793	0.43	0.06
rs685523	11094	C9orf7	9	133340462	3.68E-01	12215	2.48E-01	Up	1.16	8740	0.43	0.06
rs1744163	7803	PTP4A1	6	64346169	3.68E-01	12216	1.18E-01	Down	1.56	7261	0.43	0.09
rs7086282	79896	THNSL1	10	25353824	3.69E-01	12217	7.17E-01	Up	0.36	12183	0.43	0.01
rs12711521	23435	TARDBP	1	11025182	3.69E-01	12218	1.33E-02	Up	2.48	4709	0.43	0.19
rs11679748	57162	PELI1	2	64254277	3.69E-01	12219	1.14E-01	Up	1.58	7203	0.43	0.09
rs11574849	79176	FBXL15	10	104149686	3.69E-01	12220	3.09E-01	Down	1.02	9259	0.43	0.05
rs742086	1564	CYP2D7P1	22	40876751	3.69E-01	12221	1.58E-01	Up	1.41	7780	0.43	0.08
rs10152994	10519	CIB1	15	88585934	3.69E-01	12222	5.43E-04	Up	3.46	2988	0.43	0.33
rs1054743	148930	KNCN	1	46747663	3.69E-01	12223	9.59E-01	Up	0.05	13617	0.43	0.00
rs2938234	10671	DCTN6	8	30167862	3.69E-01	12224	1.24E-10	Down	6.43	768	0.43	0.99
rs3935479	2055	CLN8	8	1715596	3.69E-01	12225	9.53E-02	Up	1.67	6913	0.43	0.10
rs1020075	7644	ZNF91	19	23332057	3.70E-01	12226	4.71E-06	Up	4.58	1804	0.43	0.53

rs3792220	10477	UBE2E3	2	181752701	3.70E-01	12227	3.62E-15	Up	7.93	375	0.43	1.44
rs4927936	57461	ISY1	3	130361427	3.70E-01	12228	4.44E-03	Down	2.84	3988	0.43	0.24
rs9586	200942	KLHDC8B	3	49188641	3.71E-01	12229	1.56E-02	Down	2.42	4848	0.43	0.18
rs9586	64925	CCDC71	3	49188641	3.71E-01	12230	8.22E-01	Up	0.23	12799	0.43	0.01
rs567895	3626	INHBC	12	56109459	3.71E-01	12231	6.17E-01	Up	0.50	11554	0.43	0.02
rs9769506	3638	INSIG1	7	154537503	3.71E-01	12232	5.90E-15	Down	7.81	392	0.43	1.42
rs17500203	400	ARL1	12	100297477	3.71E-01	12233	8.85E-01	Down	0.14	13156	0.43	0.01
rs12431669	97	ACYP1	14	74615983	3.71E-01	12234	7.89E-01	Down	0.27	12604	0.43	0.01
rs12770228	399726	C10orf114	10	21823640	3.72E-01	12235	5.89E-01	Up	0.54	11364	0.43	0.02
rs595333	147381	CBLN2	18	68370818	3.72E-01	12236	5.85E-02	Down	1.89	6205	0.43	0.12
rs1429638	2919	CXCL1	4	75102965	3.72E-01	12237	9.99E-04	Down	3.29	3221	0.43	0.30
rs174994	5228	PGF	14	74503855	3.72E-01	12238	5.13E-01	Down	0.65	10798	0.43	0.03
rs2567039	8545	CGGBP1	3	88179714	3.72E-01	12239	1.80E-01	Up	1.34	8021	0.43	0.07
rs7254601	11045	UPK1A	19	40839155	3.72E-01	12240	4.43E-03	Down	2.85	3986	0.43	0.24
rs1894752	84820	POLR2J4	7	43770315	3.72E-01	12241	9.53E-01	Down	0.06	13572	0.43	0.00
rs7178634	51203	NUSAP1	15	39421879	3.72E-01	12242	7.92E-07	Up	4.94	1543	0.43	0.61
rs1889568	1847	DUSP5	10	112253020	3.72E-01	12243	1.29E-01	Up	1.52	7435	0.43	0.09
rs622463	6830	SUPT6H	17	23999701	3.73E-01	12244	6.34E-03	Down	2.73	4196	0.43	0.22
rs622463	6388	SDF2	17	23999701	3.73E-01	12245	2.45E-01	Down	1.16	8715	0.43	0.06
rs3741028	196294	IMMP1L	11	31411551	3.73E-01	12246	2.73E-03	Up	3.00	3733	0.43	0.26
rs11076720	353	APRT	16	87423638	3.73E-01	12247	4.39E-17	Up	8.39	307	0.43	1.64
rs1813	51382	ATP6V1D	14	66874128	3.73E-01	12248	3.38E-01	Down	0.96	9505	0.43	0.05
rs1813	64398	MPP5	14	66874128	3.73E-01	12249	8.49E-01	Up	0.19	12948	0.43	0.01
rs6462820	6967	TRGC2	7	38046545	3.74E-01	12250	8.64E-04	Up	3.33	3160	0.43	0.31
rs6462820	83930	STARD3NL	7	38046545	3.74E-01	12251	8.10E-02	Up	1.74	6648	0.43	0.11
rs10502009	4322	MMP13	11	102326614	3.74E-01	12252	2.44E-02	Down	2.25	5268	0.43	0.16
rs5914143	54552	GNL3L		54424175	3.74E-01	12253	4.76E-02	Up	1.98	5938	0.43	0.13
rs4449134	9451	EIF2AK3	2	88686223	3.74E-01	12254	5.19E-02	Up	1.94	6036	0.43	0.13
rs4898334	140597	TCEAL2		101194113	3.74E-01	12255	2.02E-05	Down	4.26	2089	0.43	0.47
rs4403789	4654	MYOD1	11	17687639	3.74E-01	12256	7.99E-01	Up	0.25	12671	0.43	0.01
rs894801	5912	RAP2B	3	154361536	3.74E-01	12257	8.64E-01	Down	0.17	13030	0.43	0.01
rs2567575	83853	ROPN1L	5	10513700	3.75E-01	12258	7.53E-03	Down	2.67	4304	0.43	0.21
rs11805172	83667	SESN2	1	28265206	3.75E-01	12259	2.39E-01	Down	1.18	8650	0.43	0.06
rs2446718	84457	PHYHIPL	10	60682093	3.75E-01	12260	6.69E-05	Down	3.99	2351	0.43	0.42
rs2277153	10585	POMT1	9	131425241	3.75E-01	12261	1.79E-02	Down	2.37	4983	0.43	0.17
rs2277153	83549	UCK1	9	131425241	3.75E-01	12262	1.03E-01	Up	1.63	7044	0.43	0.10
rs2236624	135	ADORA2A	22	23160578	3.75E-01	12263	1.39E-01	Up	1.48	7556	0.43	0.09
rs400688	26001	RNF167	17	4780674	3.75E-01	12264	2.34E-01	Up	1.19	8608	0.43	0.06
rs400688	2811	GP1BA	17	4780674	3.75E-01	12265	6.27E-01	Down	0.49	11631	0.43	0.02
rs400688	8402	SLC25A11	17	4780674	3.75E-01	12266	9.95E-01	Down	0.01	13878	0.43	0.00
rs753279	4695	NDUFA2	5	140004002	3.75E-01	12267	1.89E-11	Up	6.71	671	0.43	1.07
rs753279	3550	IK	5	140004002	3.75E-01	12268	2.61E-05	Down	4.20	2142	0.43	0.46
rs753279	55374	TMCO6	5	140004002	3.75E-01	12269	1.85E-01	Up	1.33	8079	0.43	0.07
rs753279	929	CD14	5	140004002	3.75E-01	12270	8.94E-01	Up	0.13	13206	0.43	0.00
rs17594910	90527	DUOXA1	15	43200833	3.75E-01	12271	5.05E-01	Down	0.67	10751	0.43	0.03
rs17594910	10755	GIPC1	15	43200833	3.75E-01	12272	8.60E-01	Up	0.18	13011	0.43	0.01
rs770374	282809	WDR51B	12	88320490	3.75E-01	12273	7.55E-01	Up	0.31	12407	0.43	0.01
rs9316335	253832	ZDHHC20	13	20856918	3.75E-01	12274	5.84E-02	Up	1.89	6201	0.43	0.12
rs4653178	79729	C1orf113	1	36464523	3.75E-01	12275	3.22E-01	Down	0.99	9382	0.43	0.05
rs10863388	51018	RRP15	1	214891720	3.75E-01	12276	4.24E-02	Up	2.03	5809	0.43	0.14
rs11915803	152206	CCDC13	3	42775295	3.76E-01	12277	8.29E-01	Down	0.22	12850	0.43	0.01
rs17110081	83591	THAP2	12	70325202	3.76E-01	12278	6.54E-01	Up	0.45	11789	0.43	0.02
rs6765618	50939	IMPG2	3	102537485	3.76E-01	12279	9.20E-01	Down	0.10	13377	0.43	0.00
rs9530803	5457	POU4F1	13	78066312	3.76E-01	12280	2.18E-03	Up	3.06	3593	0.43	0.27
rs6087456	140683	C20orf70	20	31204465	3.76E-01	12281	5.78E-01	Down	0.56	11298	0.42	0.02
rs3761093	79171	RBM42	19	40828289	3.76E-01	12282	9.50E-01	Down	0.06	13553	0.42	0.00
rs2268420	84058	WDR54	2	74566516	3.76E-01	12283	6.16E-05	Up	4.01	2335	0.42	0.42
rs2268420	6242	RTKN	2	74566516	3.76E-01	12284	2.01E-02	Up	2.33	5077	0.42	0.17
rs1165703	22934	RPIA	2	88892785	3.76E-01	12285	8.60E-08	Up	5.35	1279	0.42	0.71
rs9920347	196968	C15orf51	15	98156854	3.76E-01	12286	4.22E-01	Down	0.80	10134	0.42	0.04
rs2017009	9693	RAPGEF2	4	160601892	3.76E-01	12287	1.54E-03	Up	3.17	3394	0.42	0.28
rs11804512	65979	PHACTR4	1	28649509	3.77E-01	12288	6.20E-08	Up	5.41	1243	0.42	0.72
rs17744318	22803	XRN2	20	21288699	3.77E-01	12289	1.23E-02	Up	2.50	4648	0.42	0.19
rs6647999	57692	MAGEE1		75441886	3.77E-01	12290	2.29E-02	Down	2.28	5192	0.42	0.16
rs7308481	11052	CPSF6	12	67945566	3.77E-01	12291	1.61E-16	Up	8.25	332	0.42	1.58
rs548102	119392	C10orf78	10	105854556	3.78E-01	12292	3.02E-02	Up	2.17	5474	0.42	0.15
rs17426315	4189	DNAJB9	7	107818998	3.78E-01	12293	2.08E-03	Up	3.08	3553	0.42	0.27
rs17426315	168451	THAP5	7	107818998	3.78E-01	12294	6.56E-01	Down	0.45	11806	0.42	0.02
rs2368524	147968	CAPN12	19	43944078	3.78E-01	12295	4.93E-02	Down	1.97	5981	0.42	0.13
rs12684385	138065	RNF183	9	113157248	3.78E-01	12296	2.89E-01	Down	1.06	9108	0.42	0.05
rs7210970	23512	SUZ12	17	27286282	3.78E-01	12297	2.03E-03	Up	3.09	3538	0.42	0.27
rs3138158	8718	TNFRSF25	1	6454578	3.78E-01	12298	5.58E-03	Down	2.77	4141	0.42	0.23
rs3138158	83715	ESPN	1	6454578	3.78E-01	12299	7.98E-01	Up	0.26	12661	0.42	0.01
rs12223473	374393	FAM111B	11	58647068	3.78E-01	12300	3.20E-02	Up	2.14	5525	0.42	0.15
rs12223473	826	CAPNS1	11	58647068	3.78E-01	12301	3.27E-01	Up	0.98	9416	0.42	0.05
rs4658309	84230	LRRC8C	1	89855249	3.79E-01	12302	2.38E-03	Down	3.04	3655	0.42	0.26
rs6759996	92104	TTC30A	2	178296078	3.79E-01	12303	2.75E-04	Up	3.64	2749	0.42	0.36
rs12578934	11081	KERA	12	89968964	3.79E-01	12304	9.97E-01	Up	0.00	13892	0.42	0.00
rs4660378	10487	CAP1	1	40185489	3.79E-01	12305	5.00E-05	Down	4.06	2286	0.42	0.43
rs7677983	80014	WWC2	4	184605057	3.79E-01	12306	5.02E-05	Down	4.05	2288	0.42	0.43
rs339520	4294	MAP3K10	19	45383472	3.80E-01	12307	2.58E-01	Up	1.13	8843	0.42	0.06

rs2290823	84233	TMEM126A	11	85036902	3.80E-01	12308	2.43E-01	Up	1.17	8686	0.42	0.06
rs6800204	285368	PRRT3	3	9970404	3.80E-01	12309	1.13E-01	Up	1.58	7191	0.42	0.09
rs767876	10778	ZNF271	18	31145654	3.81E-01	12310	2.69E-02	Down	2.21	5358	0.42	0.16
rs803218	2661	GDF9	5	132244163	3.81E-01	12311	1.18E-03	Down	3.24	3284	0.42	0.29
rs803218	116842	LEAP2	5	132244163	3.81E-01	12312	1.82E-02	Up	2.36	4993	0.42	0.17
rs1211911	57157	PHTF2	7	77238624	3.81E-01	12313	3.51E-01	Down	0.93	9595	0.42	0.05
rs2425202	25980	C20orf4	20	34317738	3.81E-01	12314	1.73E-03	Down	3.13	3452	0.42	0.28
rs10518793	197131	UBR1	15	41106723	3.81E-01	12315	3.17E-03	Down	2.95	3808	0.42	0.25
rs7125189	2348	FOLR1	11	71579493	3.81E-01	12316	9.80E-01	Up	0.02	13773	0.42	0.00
rs901799	1358	CPA2	7	129525353	3.81E-01	12317	4.57E-01	Up	0.74	10403	0.42	0.03
rs7869742	54829	ASPN	9	92335729	3.81E-01	12318	4.51E-20	Up	9.18	197	0.42	1.93
rs7869742	1842	ECM2	9	92335729	3.81E-01	12319	1.25E-02	Down	2.50	4658	0.42	0.19
rs2304493	60681	FKBP10	17	37219694	3.82E-01	12320	3.00E-01	Up	1.04	9189	0.42	0.05
rs7373302	9276	COPB2	3	140584818	3.82E-01	12321	2.18E-05	Up	4.25	2104	0.42	0.47
rs11636443	5597	MAPK6	15	50106988	3.82E-01	12322	1.69E-18	Up	8.78	251	0.42	1.78
rs5909069	6792	CDKL5		18340385	3.82E-01	12323	1.46E-02	Down	2.44	4794	0.42	0.18
rs11672431	81890	QTRT1	19	10684940	3.82E-01	12324	5.49E-05	Up	4.03	2311	0.42	0.43
rs6478108	9966	TNFSF15	9	114638257	3.82E-01	12325	1.19E-01	Up	1.56	7284	0.42	0.09
rs8139993	5372	PMM1	22	40319835	3.82E-01	12326	7.88E-02	Up	1.76	6611	0.42	0.11
rs200968	8336	HIST1H2AM	6	27967547	3.82E-01	12327	1.71E-03	Up	3.14	3447	0.42	0.28
rs200968	8348	HIST1H2BO	6	27967547	3.82E-01	12328	1.54E-01	Up	1.43	7729	0.42	0.08
rs200968	8354	HIST1H3I	6	27967547	3.82E-01	12329	3.11E-01	Up	1.01	9273	0.42	0.05
rs200968	8356	HIST1H3J	6	27967547	3.82E-01	12330	4.62E-01	Down	0.73	10446	0.42	0.03
rs200968	8368	HIST1H4L	6	27967547	3.82E-01	12331	4.79E-01	Up	0.71	10570	0.42	0.03
rs9595973	57105	CYSLTR2	13	48184439	3.83E-01	12332	4.53E-01	Up	0.75	10366	0.42	0.03
rs11860782	9344	TAOK2	16	29897240	3.83E-01	12333	8.80E-03	Up	2.62	4413	0.42	0.21
rs7050878	7712	ZNF157		46998375	3.83E-01	12334	7.61E-02	Up	1.77	6550	0.42	0.11
rs11231640	283248	RCOR2	11	63447625	3.84E-01	12335	6.57E-01	Down	0.44	11812	0.42	0.02
rs4220	2244	FGB	4	155849364	3.84E-01	12336	3.44E-02	Up	2.12	5605	0.42	0.15
rs8071475	6198	RPS6KB1	17	55328702	3.84E-01	12337	5.40E-08	Up	5.44	1233	0.42	0.73
rs8071475	51174	TUBD1	17	55328702	3.84E-01	12338	1.59E-01	Up	1.41	7804	0.42	0.08
rs9398675	347735	SERINC2	6	122853798	3.85E-01	12339	7.40E-09	Up	5.78	1036	0.41	0.81
rs3761034	79036	C19orf50	19	18541478	3.85E-01	12340	3.90E-01	Up	0.86	9911	0.41	0.04
rs17591036	129450	C2orf60	2	200617610	3.85E-01	12341	2.56E-03	Up	3.02	3701	0.41	0.26
rs371513	23753	SDF2L1	22	20313153	3.85E-01	12342	2.28E-02	Up	2.28	5187	0.41	0.16
rs371513	164592	CCDC116	22	20313153	3.85E-01	12343	3.40E-01	Up	0.95	9520	0.41	0.05
rs12977564	126069	ZNF491	19	11781215	3.85E-01	12344	7.13E-01	Up	0.37	12152	0.41	0.01
rs1832197	3416	IDE	10	94288311	3.85E-01	12345	6.55E-01	Down	0.45	11800	0.41	0.02
rs11092612	1287	COL4A5		107518051	3.85E-01	12346	2.84E-09	Down	5.94	963	0.41	0.85
rs9302815	124222	PAQR4	16	2943835	3.85E-01	12347	1.24E-01	Up	1.54	7356	0.41	0.09
rs4720087	2281	FKBP1B	7	32768571	3.86E-01	12348	1.45E-02	Up	2.44	4785	0.41	0.18
rs4720087	11328	FKBP9	7	32768571	3.86E-01	12349	3.78E-02	Down	2.08	5703	0.41	0.14
rs12629572	132228	C3orf45	3	50299676	3.86E-01	12350	1.03E-01	Down	1.63	7048	0.41	0.10
rs981446	29927	SEC61A1	3	129236428	3.86E-01	12351	2.66E-03	Up	3.00	3720	0.41	0.26
rs3172494	51517	NCKIPSD	3	48706491	3.86E-01	12352	1.12E-02	Up	2.54	4586	0.41	0.20
rs4846217	5226	PGD	1	10386065	3.86E-01	12353	1.16E-05	Up	4.39	1979	0.41	0.49
rs12467104	9486	CHST10	2	100471602	3.86E-01	12354	6.92E-01	Down	0.40	12019	0.41	0.02
rs17763463	80304	C2orf44	2	24167722	3.86E-01	12355	1.23E-01	Up	1.54	7342	0.41	0.09
rs4242202	6620	SNCB	5	175988626	3.86E-01	12356	5.57E-01	Up	0.59	11137	0.41	0.03
rs12451779	10513	APPBP2	17	55875936	3.86E-01	12357	7.29E-03	Down	2.68	4284	0.41	0.21
rs7532845	2170	FABP3	1	31510712	3.86E-01	12358	1.41E-11	Down	6.76	648	0.41	1.09
rs904081	3975	LHX1	17	32350237	3.86E-01	12359	6.21E-01	Down	0.49	11588	0.41	0.02
rs13269094	29942	PURG	8	31015693	3.87E-01	12360	6.65E-01	Down	0.43	11855	0.41	0.02
rs730947	53632	PRKAG3	2	219528803	3.87E-01	12361	4.48E-03	Down	2.84	3991	0.41	0.23
rs1259525	118881	COMTD1	10	76674875	3.87E-01	12362	5.58E-01	Down	0.59	11143	0.41	0.03
rs11696527	10137	RBM12	20	33708793	3.87E-01	12363	8.44E-01	Up	0.20	12923	0.41	0.01
rs6510698	148252	DIRAS1	19	2670367	3.88E-01	12364	3.34E-01	Down	0.97	9472	0.41	0.05
rs8045560	4157	MC1R	16	88506995	3.88E-01	12365	2.29E-01	Down	1.20	8554	0.41	0.06
rs2469764	10174	SORBS3	8	22475136	3.88E-01	12366	4.48E-08	Down	5.47	1211	0.41	0.73
rs9929621	84331	C16orf14	16	619413	3.88E-01	12367	2.03E-10	Up	6.36	791	0.41	0.97
rs9929621	84326	C16orf13	16	619413	3.88E-01	12368	3.28E-05	Up	4.15	2184	0.41	0.45
rs140289	1652	DDT	22	22660881	3.88E-01	12369	1.34E-11	Up	6.78	644	0.41	1.09
rs140289	25774	GSTTP1	22	22660881	3.88E-01	12370	5.86E-01	Down	0.55	11345	0.41	0.02
rs2764085	6745	SSR1	6	7223328	3.89E-01	12371	2.17E-03	Up	3.07	3591	0.41	0.27
rs2300892	55471	C2orf56	2	37394176	3.89E-01	12372	6.74E-01	Up	0.42	11912	0.41	0.02
rs4907254	54910	SEMA4C	2	96971542	3.89E-01	12373	1.52E-03	Down	3.17	3388	0.41	0.28
rs11581974	8290	HIST3H3	1	224923798	3.89E-01	12374	5.83E-02	Up	1.89	6196	0.41	0.12
rs2402218	1113	CHGA	14	92442123	3.89E-01	12375	2.20E-01	Down	1.23	8463	0.41	0.07
rs680343	149465	WDR65	1	43335547	3.89E-01	12376	1.33E-01	Up	1.50	7471	0.41	0.09
rs12574668	1132	CHRM4	11	46379262	3.89E-01	12377	2.68E-01	Down	1.11	8935	0.41	0.06
rs11757000	2880	GPX5	6	28592848	3.89E-01	12378	3.65E-01	Up	0.91	9722	0.41	0.04
rs6439154	2815	GP9	3	130245531	3.89E-01	12379	4.37E-02	Up	2.02	5842	0.41	0.14
rs6439154	79825	CCDC48	3	130245531	3.89E-01	12380	4.59E-01	Up	0.74	10420	0.41	0.03
rs10853057	10672	GNA13	17	60428485	3.89E-01	12381	1.02E-02	Down	2.57	4518	0.41	0.20
rs4984683	146330	FBXL16	16	686885	3.89E-01	12382	2.94E-01	Down	1.05	9149	0.41	0.05
rs2242993	2150	F2RL1	5	76151277	3.89E-01	12383	8.87E-19	Up	8.86	237	0.41	1.81
rs12058607	54834	GDAP2	1	118133151	3.90E-01	12384	5.27E-02	Up	1.94	6059	0.41	0.13
rs320881	163087	ZNF383	19	42429690	3.90E-01	12385	8.78E-01	Down	0.15	13112	0.41	0.01
rs2304911	5187	PER1	17	7991704	3.90E-01	12386	3.64E-05	Down	4.13	2212	0.41	0.44
rs805360	23198	PSME4	2	54076499	3.90E-01	12387	8.03E-03	Up	2.65	4345	0.41	0.21
rs4925114	6720	SREBF1	17	17651995	3.90E-01	12388	9.27E-01	Up	0.09	13406	0.41	0.00

rs1222208	120534	C11orf46	11	30317953	3.90E-01	12389	4.95E-02	Up	1.96	5985	0.41	0.13
rs1008917	348093	RBPMS2	15	62846704	3.90E-01	12390	7.84E-11	Down	6.50	742	0.41	1.01
rs527113	22889	KIAA0907	1	152692329	3.90E-01	12391	1.50E-04	Up	3.79	2551	0.41	0.38
rs527113	6016	RIT1	1	152692329	3.90E-01	12392	1.60E-03	Down	3.16	3420	0.41	0.28
rs6980605	7976	FZD3	8	28472470	3.90E-01	12393	8.83E-03	Down	2.62	4419	0.41	0.21
rs11240609	6635	SNRPE	1	200562918	3.90E-01	12394	2.32E-06	Up	4.72	1691	0.41	0.56
rs9660662	4656	MYOG	1	199800377	3.91E-01	12395	3.93E-01	Up	0.85	9935	0.41	0.04
rs17109675	84795	C10orf33	10	100157941	3.91E-01	12396	3.26E-05	Up	4.15	2183	0.41	0.45
rs750438	11184	MAP4K1	19	43796215	3.91E-01	12397	4.21E-02	Up	2.03	5799	0.41	0.14
rs750438	27335	EIF3K	19	43796215	3.91E-01	12398	9.05E-02	Down	1.69	6838	0.41	0.10
rs3134269	25879	WDSOF1	8	104491078	3.91E-01	12399	6.95E-11	Up	6.44	765	0.41	1.02
rs6468443	80223	RAB11FIP1	8	37846439	3.91E-01	12400	7.08E-05	Up	3.97	2365	0.41	0.41
rs1879658	55314	TMEM144	4	159543238	3.91E-01	12401	3.73E-12	Up	6.95	615	0.41	1.14
rs4660882	60313	GPBP1L1	1	45785385	3.91E-01	12402	2.83E-01	Up	1.07	9065	0.41	0.05
rs11171747	23344	FAM62A	12	54804675	3.92E-01	12403	9.96E-05	Down	3.89	2452	0.41	0.40
rs11171747	84872	ZC3H10	12	54804675	3.92E-01	12404	1.53E-02	Down	2.42	4830	0.41	0.18
rs11171747	6171	RPL41	12	54804675	3.92E-01	12405	2.14E-02	Up	2.30	5140	0.41	0.17
rs4690523	175	AGA	4	178736311	3.92E-01	12406	2.39E-11	Up	6.68	681	0.41	1.06
rs7730157	54505	DHX29	5	54632624	3.93E-01	12407	6.10E-01	Down	0.51	11505	0.41	0.02
rs202718	2346	FOLH1	11	49151666	3.93E-01	12408	4.40E-22	Up	9.67	156	0.41	2.14
rs2932005	137994	LETM2	8	38351031	3.93E-01	12409	8.21E-02	Down	1.74	6668	0.41	0.11
rs5910940	29071	C1GALT1C1		119540692	3.93E-01	12410	9.71E-01	Up	0.04	13709	0.41	0.00
rs5952639	63904	DUSP21		44465377	3.93E-01	12411	9.57E-01	Down	0.05	13600	0.41	0.00
rs17720826	284086	NEK8	17	24085398	3.93E-01	12412	2.93E-02	Down	2.18	5440	0.41	0.15
rs11869840	9570	GOSR2	17	42351099	3.94E-01	12413	3.40E-02	Down	2.12	5595	0.40	0.15
rs4135385	1499	CTNNB1	3	41254444	3.94E-01	12414	4.10E-02	Down	2.04	5774	0.40	0.14
rs10124609	3444	IFNA7	9	21186907	3.94E-01	12415	8.17E-01	Up	0.23	12767	0.40	0.01
rs10124609	3441	IFNA4	9	21186907	3.94E-01	12416	8.30E-01	Up	0.21	12857	0.40	0.01
rs10124609	3446	IFNA10	9	21186907	3.94E-01	12417	9.66E-01	Up	0.04	13673	0.40	0.00
rs7612543	253461	ZBTB38	3	142640910	3.94E-01	12418	7.72E-02	Down	1.77	6581	0.40	0.11
rs335675	26263	FBXO22	15	74007340	3.95E-01	12419	2.21E-03	Up	3.06	3597	0.40	0.27
rs6075668	140901	STK35	20	2051264	3.95E-01	12420	3.85E-01	Up	0.87	9875	0.40	0.04
rs7245564	6142	RPL18A	19	17824878	3.95E-01	12421	4.32E-07	Up	5.05	1462	0.40	0.64
rs7245564	6528	SLC5A5	19	17824878	3.95E-01	12422	3.25E-01	Up	0.99	9401	0.40	0.05
rs9383583	135250	RAET1E	6	150304117	3.95E-01	12423	9.41E-01	Up	0.07	13501	0.40	0.00
rs1950955	23768	FLRT2	14	85154613	3.95E-01	12424	1.82E-01	Down	1.34	8042	0.40	0.07
rs9686746	81789	TIGD6	5	149380161	3.95E-01	12425	3.54E-03	Up	2.92	3867	0.40	0.25
rs2959356	6760	SS18	18	21844201	3.95E-01	12426	6.66E-02	Down	1.83	6368	0.40	0.12
rs7827865	8694	DGAT1	8	145537864	3.96E-01	12427	4.44E-02	Up	2.01	5863	0.40	0.14
rs30461	282618	IL29	19	44480955	3.96E-01	12428	3.61E-01	Up	0.91	9687	0.40	0.04
rs3810650	55879	GABRQ		151489891	3.96E-01	12429	5.84E-01	Down	0.55	11341	0.40	0.02
rs2224410	4990	SIX6	14	60040284	3.96E-01	12430	5.33E-02	Down	1.93	6068	0.40	0.13
rs568298	7511	XPNPEP1	10	111645055	3.96E-01	12431	8.88E-04	Down	3.32	3173	0.40	0.31
rs273266	10437	IFI30	19	18144501	3.96E-01	12432	3.98E-01	Up	0.85	9969	0.40	0.04
rs3895740	57467	HHATL	3	42729916	3.96E-01	12433	2.28E-01	Down	1.21	8534	0.40	0.06
rs7954050	55832	CAND1	12	65964384	3.96E-01	12434	4.20E-02	Up	2.03	5798	0.40	0.14
rs12148207	59274	MESDC1	15	79092356	3.96E-01	12435	9.50E-03	Up	2.59	4470	0.40	0.20
rs7842946	55284	UBE2W	8	74907275	3.96E-01	12436	5.38E-03	Up	2.78	4112	0.40	0.23
rs2289247	28972	SPCS1	3	52702297	3.97E-01	12437	1.29E-01	Up	1.52	7434	0.40	0.09
rs7918775	10557	RPP38	10	15191913	3.97E-01	12438	1.89E-04	Up	3.73	2607	0.40	0.37
rs6556301	2264	FGFR4	5	176460183	3.97E-01	12439	2.49E-07	Down	5.16	1396	0.40	0.66
rs3092169	57727	NCOA5	20	44135493	3.97E-01	12440	1.25E-02	Up	2.50	4659	0.40	0.19
rs11951885	902	CCNH	5	86763322	3.98E-01	12441	1.33E-02	Up	2.48	4711	0.40	0.19
rs10491216	8392	OR3A3	17	3267156	3.98E-01	12442	7.32E-01	Up	0.34	12269	0.40	0.01
rs1258201	55753	OGDHL	10	50658701	3.98E-01	12443	2.85E-01	Up	1.07	9074	0.40	0.05
rs6606651	220064	ORAOV1	11	69207473	3.99E-01	12444	5.16E-01	Down	0.65	10824	0.40	0.03
rs11071852	8766	RAB11A	15	63928895	3.99E-01	12445	4.22E-21	Up	9.46	175	0.40	2.04
rs901865	1659	DHX8	3	11275707	3.99E-01	12446	1.77E-06	Down	4.78	1651	0.40	0.58
rs901865	3269	HRH1	3	11275707	3.99E-01	12447	1.75E-03	Down	3.13	3465	0.40	0.28
rs12138787	23632	CA14	1	147059143	3.99E-01	12448	7.73E-05	Down	3.95	2381	0.40	0.41
rs12138787	79630	C1orf54	1	147059143	3.99E-01	12449	3.32E-03	Down	2.94	3830	0.40	0.25
rs12138787	51107	APH1A	1	147059143	3.99E-01	12450	1.89E-01	Up	1.31	8129	0.40	0.07
rs1458292	84869	CBR4	4	170310611	4.00E-01	12451	7.35E-18	Up	8.62	272	0.40	1.71
rs639434	9481	SLC25A27	6	46714287	4.00E-01	12452	4.91E-06	Up	4.57	1810	0.40	0.53
rs1127152	56623	INPP5E	9	136611436	4.00E-01	12453	2.83E-01	Down	1.07	9057	0.40	0.05
rs4964310	121053	C12orf45	12	103897312	4.01E-01	12454	6.00E-08	Up	5.42	1238	0.40	0.72
rs10097505	23237	ARC	8	143691186	4.01E-01	12455	7.32E-01	Down	0.34	12268	0.40	0.01
rs381852	166979	CDC20B	5	54495718	4.01E-01	12456	7.98E-01	Up	0.26	12665	0.40	0.01
rs7932174	5962	RDX	11	109627636	4.01E-01	12457	1.38E-01	Down	1.48	7540	0.40	0.09
rs13290	2256	FGF11	17	7270356	4.01E-01	12458	1.26E-01	Down	1.53	7387	0.40	0.09
rs13290	1140	CHRNB1	17	7270356	4.01E-01	12459	3.31E-01	Down	0.97	9455	0.40	0.05
rs13290	284114	TMEM102	17	7270356	4.01E-01	12460	6.27E-01	Down	0.49	11633	0.40	0.02
rs1042728	5731	PTGER1	19	14442063	4.02E-01	12461	4.33E-01	Down	0.78	10214	0.40	0.04
rs10776672	118471	PRAP1	10	135046836	4.02E-01	12462	4.00E-02	Down	2.05	5748	0.40	0.14
rs10505008	83955	NACAP1	8	102430238	4.02E-01	12463	3.27E-01	Up	0.98	9418	0.40	0.05
rs10132688	1073	CFL2	14	34254899	4.02E-01	12464	2.69E-09	Down	5.95	958	0.40	0.86
rs3744382	51651	PTRH2	17	55139310	4.02E-01	12465	1.11E-08	Up	5.74	1048	0.40	0.80
rs3744382	81671	TMEM49	17	55139310	4.02E-01	12466	2.11E-01	Up	1.25	8360	0.40	0.07
rs6534323	132332	TMEM155	4	123053493	4.02E-01	12467	3.03E-01	Up	1.03	9213	0.40	0.05
rs9462165	27154	BRPF3	6	36313418	4.02E-01	12468	8.19E-05	Up	3.94	2399	0.40	0.41
rs3138189	1634	DCN	12	90068162	4.02E-01	12469	6.90E-02	Down	1.82	6410	0.40	0.12

rs816411	5260	PHKG1	7	55945698	4.03E-01	12470	1.03E-05	Down	4.41	1956	0.39	0.50
rs11631564	4947	OAZ2	15	62760664	4.03E-01	12471	2.60E-02	Down	2.23	5319	0.39	0.16
rs4073710	84275	SLC25A33	1	9590061	4.03E-01	12472	1.38E-07	Up	5.27	1336	0.39	0.69
rs11797341	4112	MAGEB1		30036872	4.03E-01	12473	5.15E-02	Up	1.95	6023	0.39	0.13
rs788805	29089	UBE2T	1	199025573	4.03E-01	12474	8.64E-04	Up	3.33	3159	0.39	0.31
rs6104012	149708	WFDC5	20	43167985	4.03E-01	12475	4.28E-01	Down	0.79	10176	0.39	0.04
rs1982157	3955	LFNG	7	2329398	4.04E-01	12476	1.49E-03	Up	3.18	3378	0.39	0.28
rs9916811	6427	SFRS2	17	72260868	4.04E-01	12477	1.63E-01	Up	1.40	7852	0.39	0.08
rs5996039	27254	CSDC2	22	40306457	4.04E-01	12478	5.04E-03	Up	2.80	4076	0.39	0.23
rs631134	64600	PLA2G2F	1	20210197	4.04E-01	12479	3.57E-01	Up	0.92	9654	0.39	0.04
rs3744165	79755	ZNF750	17	78383731	4.05E-01	12480	2.60E-01	Down	1.13	8857	0.39	0.06
rs11264177	339488	TFAP2E	1	35716095	4.05E-01	12481	5.14E-01	Down	0.65	10810	0.39	0.03
rs2491	6130	RPL7A	9	133227525	4.05E-01	12482	1.98E-07	Up	5.20	1370	0.39	0.67
rs2491	6838	SURF6	9	133227525	4.05E-01	12483	1.61E-01	Up	1.40	7832	0.39	0.08
rs7635560	56922	MCCC1	3	184211995	4.06E-01	12484	1.12E-01	Up	1.59	7187	0.39	0.09
rs3785138	9683	N4BP1	16	47146115	4.06E-01	12485	2.12E-01	Down	1.25	8383	0.39	0.07
rs2298468	64837	KLC2	11	65799538	4.06E-01	12486	5.75E-01	Up	0.56	11277	0.39	0.02
rs2298468	81876	RAB1B	11	65799538	4.06E-01	12487	5.89E-01	Up	0.54	11365	0.39	0.02
rs447375	85290	KRTAP4-3	17	36592496	4.06E-01	12488	2.37E-01	Up	1.18	8630	0.39	0.06
rs11853141	79661	NEIL1	15	73419920	4.07E-01	12489	3.42E-01	Down	0.95	9539	0.39	0.05
rs11853141	54939	COMMD4	15	73419920	4.07E-01	12490	5.65E-01	Up	0.58	11208	0.39	0.02
rs4766588	121643	FOXN4	12	108190360	4.07E-01	12491	5.66E-01	Up	0.57	11211	0.39	0.02
rs4983411	9112	MTA1	14	104987842	4.07E-01	12492	3.47E-01	Up	0.94	9569	0.39	0.05
rs2213324	55998	NXF5		100938649	4.07E-01	12493	1.53E-01	Down	1.43	7724	0.39	0.08
rs1037680	5315	PKM2	15	70301373	4.07E-01	12494	5.98E-03	Down	2.75	4169	0.39	0.22
rs2302985	26093	CCDC9	19	52453383	4.07E-01	12495	8.23E-01	Down	0.22	12810	0.39	0.01
rs4807928	6209	RPS15	19	1399180	4.07E-01	12496	3.34E-04	Up	3.59	2815	0.39	0.35
rs538549	9363	RAB33A		129053516	4.08E-01	12497	2.89E-01	Down	1.06	9109	0.39	0.05
rs3204848	90488	C12orf23	12	105869906	4.08E-01	12498	2.95E-06	Up	4.67	1734	0.39	0.55
rs11020819	2526	FUT4	11	93909884	4.08E-01	12499	3.83E-04	Up	3.55	2867	0.39	0.34
rs12842916	139420	SMEK3P		27238996	4.09E-01	12500	3.40E-01	Down	0.95	9527	0.39	0.05
rs1043879	25949	SYF2	1	25315387	4.09E-01	12501	1.17E-01	Down	1.57	7252	0.39	0.09
rs3891250	521	ATP5I	4	648304	4.09E-01	12502	1.42E-11	Up	6.75	653	0.39	1.08
rs3891250	4636	MYL5	4	648304	4.09E-01	12503	6.98E-01	Up	0.39	12056	0.39	0.02
rs3891250	5158	PDE6B	4	648304	4.09E-01	12504	8.23E-01	Down	0.22	12808	0.39	0.01
rs10027817	79799	UGT2A3	4	70009982	4.09E-01	12505	6.27E-01	Up	0.49	11635	0.39	0.02
rs7163	10969	EBNA1BP2	1	43301629	4.09E-01	12506	1.06E-03	Up	3.27	3247	0.39	0.30
rs9327744	4090	SMAD5	5	135501661	4.09E-01	12507	6.79E-06	Up	4.50	1867	0.39	0.52
rs7932167	3665	IRF7	11	610599	4.09E-01	12508	5.94E-02	Up	1.89	6225	0.39	0.12
rs9934227	79650	C16orf57	16	56576897	4.09E-01	12509	8.83E-02	Down	1.70	6774	0.39	0.11
rs9934227	57567	ZNF319	16	56576897	4.09E-01	12510	1.21E-01	Down	1.55	7324	0.39	0.09
rs411942	339487	ZBTB8OS	1	32779553	4.10E-01	12511	4.91E-03	Up	2.81	4055	0.39	0.23
rs1058678	1937	EEF1G	11	62117613	4.10E-01	12512	3.14E-05	Up	4.16	2179	0.39	0.45
rs6726634	9111	NMI	2	151985388	4.10E-01	12513	1.49E-07	Down	5.25	1343	0.39	0.68
rs2675347	283652	SLC24A5	15	46205937	4.10E-01	12514	1.36E-09	Up	6.06	913	0.39	0.89
rs6526366	80311	KLHL15		23799434	4.11E-01	12515	4.50E-02	Down	2.00	5877	0.39	0.13
rs2473355	11330	CTRC	1	15495523	4.11E-01	12516	7.01E-01	Up	0.38	12078	0.39	0.02
rs1938405	2134	EXTL1	1	26034109	4.11E-01	12517	8.32E-01	Up	0.21	12859	0.39	0.01
rs16862370	8659	ALDH4A1	1	18987210	4.11E-01	12518	9.59E-01	Up	0.05	13611	0.39	0.00
rs7529205	54626	HES2	1	6407560	4.11E-01	12519	1.74E-02	Up	2.38	4955	0.39	0.18
rs11070256	701	BUB1B	15	38307302	4.12E-01	12520	1.10E-05	Up	4.40	1967	0.39	0.50
rs4828037	64102	TNMD		99651829	4.12E-01	12521	4.62E-01	Down	0.74	10443	0.39	0.03
rs194320	79576	NKAP		118860005	4.12E-01	12522	9.76E-02	Up	1.66	6953	0.39	0.10
rs967426	5569	PKIA	8	79660454	4.12E-01	12523	4.50E-01	Down	0.75	10347	0.39	0.03
rs1076649	83933	HDAC10	22	48999975	4.12E-01	12524	1.30E-02	Down	2.48	4688	0.38	0.19
rs1076649	85378	TUBGCP6	22	48999975	4.12E-01	12525	1.89E-02	Up	2.35	5017	0.38	0.17
rs1076649	6300	MAPK12	22	48999975	4.12E-01	12526	8.77E-01	Up	0.15	13104	0.38	0.01
rs1076649	5600	MAPK11	22	48999975	4.12E-01	12527	9.51E-01	Down	0.06	13561	0.38	0.00
rs799923	672	BRCA1	17	38505457	4.12E-01	12528	2.43E-02	Up	2.25	5259	0.38	0.16
rs11191666	6877	TAF5	10	105117268	4.13E-01	12529	5.15E-01	Up	0.65	10819	0.38	0.03
rs11671851	7730	ZNF177	19	9350454	4.13E-01	12530	2.52E-02	Up	2.24	5294	0.38	0.16
rs2468849	117195	MRGPRX3	11	18094729	4.13E-01	12531	9.31E-01	Up	0.09	13439	0.38	0.00
rs9890432	284004	HEXDC	17	77978909	4.14E-01	12532	6.88E-01	Down	0.40	11995	0.38	0.02
rs7791492	23658	LSM5	7	32293439	4.14E-01	12533	2.29E-02	Up	2.28	5193	0.38	0.16
rs2233817	5948	RBP2	3	140656263	4.14E-01	12534	3.71E-01	Down	0.89	9774	0.38	0.04
rs6599308	285464	CRIPAK	4	1377610	4.14E-01	12535	1.77E-04	Up	3.75	2588	0.38	0.38
rs4963129	66005	CHID1	11	888518	4.14E-01	12536	9.27E-02	Up	1.68	6877	0.38	0.10
rs1975802	80004	RBM35B	16	66843348	4.14E-01	12537	6.86E-03	Up	2.70	4247	0.38	0.22
rs10831519	79780	CCDC82	11	95756092	4.15E-01	12538	1.74E-05	Down	4.30	2065	0.38	0.48
rs10831519	8690	JRKL	11	95756092	4.15E-01	12539	4.77E-02	Down	1.98	5941	0.38	0.13
rs12644	5532	PPP3CB	10	74867057	4.15E-01	12540	1.21E-04	Down	3.84	2495	0.38	0.39
rs12644	118490	ZMYND17	10	74867057	4.15E-01	12541	2.79E-01	Up	1.08	9024	0.38	0.06
rs4581040	10947	AP3M2	8	42151183	4.15E-01	12542	7.39E-01	Down	0.33	12305	0.38	0.01
rs7036404	881	CCIN	9	36163148	4.15E-01	12543	8.78E-01	Down	0.15	13114	0.38	0.01
rs8108064	828	CAPS	19	5854807	4.15E-01	12544	3.36E-01	Down	0.96	9495	0.38	0.05
rs13390931	150465	TTL	2	112936137	4.16E-01	12545	1.15E-03	Up	3.25	3274	0.38	0.29
rs12103666	2118	ETV4	17	38991836	4.16E-01	12546	5.29E-01	Up	0.63	10931	0.38	0.03
rs9998052	6059	ABCE1	4	146380496	4.16E-01	12547	3.52E-03	Up	2.92	3865	0.38	0.25
rs16999232	57615	ZNF492	19	22589615	4.16E-01	12548	4.28E-01	Up	0.79	10173	0.38	0.04
rs7152548	6495	SIX1	14	60191793	4.17E-01	12549	2.84E-02	Up	2.19	5409	0.38	0.15
rs673995	170589	GPHA2	11	64458776	4.17E-01	12550	1.93E-02	Up	2.34	5038	0.38	0.17

rs11879005	342898	SYCN	19	44402743	4.17E-01	12551	1.00E-01	Up	1.64	7001	0.38	0.10
rs1866452	57181	SLC39A10	2	196362011	4.17E-01	12552	6.15E-04	Down	3.43	3037	0.38	0.32
rs564249	84618	NT5C1A	1	39824716	4.17E-01	12553	8.21E-01	Up	0.23	12796	0.38	0.01
rs3135688	5982	RFC2	7	73096394	4.17E-01	12554	3.48E-01	Down	0.94	9577	0.38	0.05
rs1976101	64946	CENPH	5	68532208	4.17E-01	12555	1.06E-01	Up	1.61	7098	0.38	0.10
rs1976101	92259	MRPS36	5	68532208	4.17E-01	12556	9.92E-01	Up	0.01	13856	0.38	0.00
rs2715555	7153	TOP2A	17	35798150	4.17E-01	12557	1.32E-12	Up	7.09	563	0.38	1.19
rs2526162	3950	LECT2	5	135336658	4.17E-01	12558	5.55E-02	Up	1.91	6128	0.38	0.13
rs6740879	5903	RANBP2	2	108866726	4.17E-01	12559	8.83E-11	Up	6.49	752	0.38	1.01
rs6740879	165055	CCDC138	2	108866726	4.17E-01	12560	1.70E-05	Up	4.30	2060	0.38	0.48
rs13325586	54977	SLC25A38	3	39388528	4.18E-01	12561	6.22E-01	Up	0.49	11595	0.38	0.02
rs9922624	9114	ATP6V0D1	16	66036477	4.18E-01	12562	1.19E-01	Down	1.56	7286	0.38	0.09
rs9922624	3291	HSD11B2	16	66036477	4.18E-01	12563	2.06E-01	Down	1.27	8312	0.38	0.07
rs8108626	147687	ZNF417	19	63124308	4.18E-01	12564	5.91E-01	Down	0.54	11375	0.38	0.02
rs10507969	26050	SLITRK5	13	87109501	4.18E-01	12565	9.47E-01	Up	0.07	13532	0.38	0.00
rs2274201	25831	HECTD1	14	30716992	4.19E-01	12566	7.74E-03	Up	2.66	4322	0.38	0.21
rs7725222	153579	BTNL9	5	180394512	4.19E-01	12567	5.40E-01	Down	0.61	11014	0.38	0.03
rs2298146	55268	ECHDC2	1	53083940	4.19E-01	12568	2.22E-02	Down	2.29	5166	0.38	0.17
rs9303029	26502	NARF	17	78002104	4.19E-01	12569	2.95E-02	Up	2.18	5446	0.38	0.15
rs9303029	79415	C17orf62	17	78002104	4.19E-01	12570	1.36E-01	Up	1.49	7529	0.38	0.09
rs1038292	6921	TCEB1	8	75016260	4.19E-01	12571	2.19E-06	Up	4.74	1682	0.38	0.57
rs2856229	374286	CDRT1	17	15435991	4.19E-01	12572	3.49E-01	Down	0.94	9582	0.38	0.05
rs4717112	155368	WBSCR27	7	72710068	4.19E-01	12573	7.61E-01	Down	0.30	12444	0.38	0.01
rs12728955	26994	RNF11	1	51421543	4.19E-01	12574	4.29E-02	Down	2.02	5823	0.38	0.14
rs12827688	1606	DGKA	12	54595771	4.19E-01	12575	6.67E-03	Down	2.71	4227	0.38	0.22
rs12827688	84305	WIBG	12	54595771	4.19E-01	12576	4.85E-01	Up	0.70	10608	0.38	0.03
rs2286265	29992	PILRA	7	99651616	4.19E-01	12577	2.03E-02	Up	2.32	5089	0.38	0.17
rs9906612	84961	FBXL20	17	34801157	4.19E-01	12578	8.94E-02	Up	1.70	6792	0.38	0.10
rs11218954	3312	HSPA8	11	122451270	4.19E-01	12579	2.52E-03	Up	3.02	3692	0.38	0.26
rs4919096	23401	FRAT2	10	99094539	4.20E-01	12580	2.45E-02	Up	2.25	5271	0.38	0.16
rs9899404	2695	GIP	17	44408827	4.20E-01	12581	4.31E-01	Up	0.79	10205	0.38	0.04
rs3866330	1605	DAG1	3	49557998	4.21E-01	12582	3.06E-03	Up	2.96	3784	0.38	0.25
rs8038097	64799	IQCH	15	65369071	4.21E-01	12583	4.28E-01	Up	0.79	10172	0.38	0.04
rs732967	64132	XYLT2	17	45763551	4.21E-01	12584	9.79E-01	Down	0.03	13762	0.38	0.00
rs10484911	6903	TBCC	6	42838267	4.22E-01	12585	6.39E-02	Up	1.85	6322	0.38	0.12
rs12940055	4215	MAP3K3	17	59075874	4.22E-01	12586	5.68E-03	Down	2.77	4151	0.38	0.22
rs10824306	84858	ZNF503	10	76830688	4.22E-01	12587	1.07E-02	Down	2.55	4558	0.37	0.20
rs10824306	100131213	C10orf41	10	76830688	4.22E-01	12588	7.58E-01	Up	0.31	12427	0.37	0.01
rs12152351	201627	FAM116A	3	57605058	4.22E-01	12589	8.69E-01	Up	0.17	13056	0.37	0.01
rs17349	29058	C20orf30	20	5047516	4.22E-01	12590	7.09E-02	Down	1.81	6449	0.37	0.11
rs2364403	9181	ARHGEF2	1	152740825	4.22E-01	12591	8.94E-06	Down	4.44	1924	0.37	0.50
rs9307912	54510	PCDH18	4	138825771	4.22E-01	12592	4.77E-04	Down	3.49	2946	0.37	0.33
rs3734637	23493	HEY2	6	126123012	4.22E-01	12593	5.27E-03	Up	2.79	4102	0.37	0.23
rs2290130		B3GNT7	2	232088632	4.23E-01	12594	4.85E-02	Down	1.97	5960	0.37	0.13
rs12302890	84920	ALG10	12	34075946	4.23E-01	12595	7.42E-01	Down	0.33	12325	0.37	0.01
rs1806552	79034	C7orf26	7	6430137	4.23E-01	12596	1.49E-01	Up	1.44	7671	0.37	0.08
rs3784929	54386	TERF2IP	16	74234528	4.23E-01	12597	4.52E-02	Down	2.00	5878	0.37	0.13
rs6981869	2961	GTF2E2	8	30636038	4.24E-01	12598	4.57E-01	Up	0.74	10402	0.37	0.03
rs2242508	25870	SUMF2	7	55925698	4.24E-01	12599	3.23E-01	Down	0.99	9384	0.37	0.05
rs7956285	5469	MED1	12	117033027	4.24E-01	12600	2.20E-01	Up	1.23	8457	0.37	0.07
rs10817858	158427	C9orf97	9	97467674	4.24E-01	12601	6.24E-02	Down	1.86	6289	0.37	0.12
rs723729	192666	KRT24	17	36129935	4.25E-01	12602	2.56E-01	Up	1.14	8824	0.37	0.06
rs7525139	25932	CLIC4	1	24825828	4.25E-01	12603	1.26E-21	Down	9.55	169	0.37	2.09
rs2301301	3232	HOXD3	2	176857774	4.25E-01	12604	8.30E-01	Up	0.21	12856	0.37	0.01
rs4686428	1427	CRYGS	3	187730075	4.25E-01	12605	1.35E-01	Down	1.50	7508	0.37	0.09
rs3907609	284047	CCDC144B	17	18446401	4.25E-01	12606	9.29E-01	Down	0.09	13422	0.37	0.00
rs2715553	5914	RARA	17	35749846	4.25E-01	12607	1.03E-03	Down	3.28	3235	0.37	0.30
rs7313278	4922	NTS	12	84752731	4.25E-01	12608	4.08E-02	Up	2.05	5768	0.37	0.14
rs2038903	2023	ENO1	1	8850783	4.26E-01	12609	8.14E-01	Up	0.24	12750	0.37	0.01
rs5975721	139378	GPR112		135151005	4.26E-01	12610	6.96E-01	Up	0.39	12046	0.37	0.02
rs2271775	9791	PTDSS1	8	97412352	4.26E-01	12611	8.20E-08	Up	5.36	1275	0.37	0.71
rs4255023	1364	CLDN4	7	72684269	4.26E-01	12612	9.40E-08	Up	5.34	1290	0.37	0.70
rs6714710	7535	ZAP70	2	97803604	4.26E-01	12613	5.01E-01	Up	0.67	10723	0.37	0.03
rs6527494	5277	PIGA		15113967	4.26E-01	12614	3.20E-01	Up	0.99	9362	0.37	0.05
rs10814275	57704	GBA2	9	35748578	4.26E-01	12615	9.96E-02	Up	1.65	6993	0.37	0.10
rs4077460	1022	CDK7	5	68608424	4.26E-01	12616	2.73E-04	Up	3.64	2744	0.37	0.36
rs4400059	10720	UGT2B11	4	70248611	4.26E-01	12617	3.65E-01	Up	0.91	9718	0.37	0.04
rs7277820	257203	DSCR9	21	37502179	4.27E-01	12618	9.61E-01	Up	0.05	13629	0.37	0.00
rs16860002	29926	GMPPA	2	220198138	4.27E-01	12619	4.99E-01	Up	0.68	10703	0.37	0.03
rs16860002	55515	ACCN4	2	220198138	4.27E-01	12620	9.20E-01	Up	0.10	13380	0.37	0.00
rs4954204	905	CCNT2	2	135553242	4.27E-01	12621	7.52E-02	Up	1.78	6530	0.37	0.11
rs3448	387	RHOA	3	49371755	4.27E-01	12622	9.55E-07	Down	4.90	1569	0.37	0.60
rs3448	2876	GPX1	3	49371755	4.27E-01	12623	3.98E-02	Up	2.06	5745	0.37	0.14
rs11547281	925	CD8A	2	86915504	4.27E-01	12624	2.61E-01	Up	1.12	8868	0.37	0.06
rs2472299	1543	CYP1A1	15	72820453	4.27E-01	12625	2.20E-01	Down	1.23	8453	0.37	0.07
rs3858032	9933	KIAA0020	9	2816674	4.27E-01	12626	6.90E-10	Up	6.17	870	0.37	0.92
rs16957511	9333	TGM5	15	41327409	4.28E-01	12627	7.31E-01	Up	0.34	12256	0.37	0.01
rs4892781	1046	CDX4		72458659	4.28E-01	12628	8.28E-01	Down	0.22	12839	0.37	0.01
rs2118204	83607	AMMECR1L	2	128340341	4.28E-01	12629	5.58E-01	Down	0.59	11152	0.37	0.03
rs8067794	218	ALDH3A1	17	19604339	4.28E-01	12630	7.21E-01	Down	0.36	12208	0.37	0.01
rs4820844	23541	SEC14L2	22	29119769	4.28E-01	12631	3.75E-01	Down	0.89	9804	0.37	0.04

rs6475526	3451	IFNA17	9	21232161	4.28E-01	12632	2.78E-01	Down	1.09	9012	0.37	0.06
rs4777489	123228	SENP8	15	70217488	4.28E-01	12633	1.43E-02	Down	2.45	4773	0.37	0.18
rs11861842	83719	YPEL3	16	30001681	4.29E-01	12634	2.32E-01	Down	1.19	8586	0.37	0.06
rs11861842	6911	TBX6	16	30001681	4.29E-01	12635	6.27E-01	Up	0.49	11625	0.37	0.02
rs4873772	4173	MCM4	8	49021486	4.29E-01	12636	1.72E-07	Up	5.23	1359	0.37	0.68
rs2864474	284186	TMEM105	17	76916744	4.29E-01	12637	1.85E-01	Up	1.33	8076	0.37	0.07
rs888709	10399	GNB2L1	5	180605834	4.29E-01	12638	4.40E-26	Up	10.55	99	0.37	2.54
rs2073893	25999	CLIP3	19	41190255	4.29E-01	12639	3.39E-06	Down	4.65	1758	0.37	0.55
rs2073893	163183	C19orf46	19	41190255	4.29E-01	12640	3.87E-03	Up	2.89	3908	0.37	0.24
rs2073893	84964	ALKBH6	19	41190255	4.29E-01	12641	3.10E-02	Down	2.16	5493	0.37	0.15
rs7157322	23405	DICER1	14	94652297	4.29E-01	12642	2.74E-01	Up	1.09	8983	0.37	0.06
rs1554656	5920	RARRES3	11	63076815	4.29E-01	12643	2.64E-01	Up	1.12	8906	0.37	0.06
rs1234612	1845	DUSP3	17	39196328	4.29E-01	12644	1.04E-25	Down	10.48	104	0.37	2.50
rs1234612	50964	SOST	17	39196328	4.29E-01	12645	7.73E-01	Up	0.29	12518	0.37	0.01
rs11624049	7453	WARS	14	99922573	4.29E-01	12646	1.01E-01	Up	1.64	7020	0.37	0.10
rs4903268	83694	RPS6KL1	14	74440813	4.30E-01	12647	5.33E-03	Down	2.79	4106	0.37	0.23
rs2921558	84215	ZNF541	19	52757676	4.30E-01	12648	2.25E-01	Up	1.21	8509	0.37	0.06
rs7002959	50626	CYHR1	8	145656204	4.30E-01	12649	1.79E-02	Up	2.37	4981	0.37	0.17
rs4824157	79924	ADM2	22	49219725	4.30E-01	12650	8.10E-01	Up	0.24	12722	0.37	0.01
rs17532840	9617	MTRF1	13	40735126	4.30E-01	12651	1.61E-01	Down	1.40	7823	0.37	0.08
rs2729812	114294	LACTB	15	61223681	4.30E-01	12652	4.96E-02	Up	1.96	5987	0.37	0.13
rs2729812	51065	RPS27L	15	61223681	4.30E-01	12653	7.70E-01	Up	0.29	12495	0.37	0.01
rs6499969	2806	GOT2	16	57291708	4.31E-01	12654	2.94E-02	Down	2.18	5443	0.37	0.15
rs965912	7140	TNNT3	11	1900778	4.31E-01	12655	8.80E-01	Up	0.15	13130	0.37	0.01
rs2074907	10942	PRSS21	16	2808642	4.32E-01	12656	9.84E-01	Down	0.02	13806	0.36	0.00
rs2228528	267004	PGBD3	10	50402286	4.32E-01	12657	8.06E-01	Up	0.25	12705	0.36	0.01
rs1954611	79366	NSBP1		80184465	4.32E-01	12658	7.65E-01	Down	0.30	12458	0.36	0.01
rs3811803	10608	MXD4	4	2217973	4.33E-01	12659	1.20E-07	Up	5.29	1315	0.36	0.69
rs6948488	2128	EVX1	7	27047874	4.33E-01	12660	1.50E-01	Down	1.44	7681	0.36	0.08
rs746738	84335	AKT1S1	19	55078152	4.33E-01	12661	9.48E-06	Up	4.43	1936	0.36	0.50
rs3733925	51164	DCTN4	5	150070537	4.33E-01	12662	1.18E-02	Up	2.52	4626	0.36	0.19
rs3733925	55696	RBM22	5	150070537	4.33E-01	12663	2.34E-01	Down	1.19	8602	0.36	0.06
rs8021984	51527	C14orf129	14	95913343	4.33E-01	12664	4.37E-01	Down	0.78	10252	0.36	0.04
rs6520015	55267	C22orf26	22	44783950	4.33E-01	12665	8.80E-01	Down	0.15	13123	0.36	0.01
rs7431578	25904	CNOT10	3	32728377	4.34E-01	12666	4.52E-03	Up	2.84	3996	0.36	0.23
rs6471412	54704	PPM2C	8	94987590	4.34E-01	12667	8.48E-15	Up	7.77	401	0.36	1.41
rs9894254	6448	SGSH	17	75799274	4.34E-01	12668	3.06E-02	Up	2.16	5484	0.36	0.15
rs9894254	79092	CARD14	17	75799274	4.34E-01	12669	5.49E-01	Down	0.60	11077	0.36	0.03
rs5943140	1641	DCX		110360980	4.35E-01	12670	1.06E-01	Down	1.62	7090	0.36	0.10
rs3761445	23764	MAFF	22	36919911	4.35E-01	12671	7.68E-02	Down	1.77	6571	0.36	0.11
rs1108472	10935	PRDX3	10	120944340	4.35E-01	12672	1.94E-01	Up	1.30	8195	0.36	0.07
rs9608781	8508	NIPSNAP1	22	28285322	4.35E-01	12673	1.76E-07	Up	5.22	1360	0.36	0.68
rs7523732	7161	TP73	1	3608198	4.35E-01	12674	4.69E-02	Down	1.99	5919	0.36	0.13
rs7523732	49856	WDR8	1	3608198	4.35E-01	12675	1.25E-01	Up	1.53	7372	0.36	0.09
rs4682859	25994	HIGD1A	3	42838743	4.36E-01	12676	8.49E-02	Down	1.72	6719	0.36	0.11
rs2186411	5007	OSBP	11	59116417	4.36E-01	12677	1.12E-01	Down	1.59	7179	0.36	0.09
rs2415251	54913	RPP25	15	73029208	4.36E-01	12678	2.68E-01	Up	1.11	8930	0.36	0.06
rs491973	254187	TSGA10IP	11	65483877	4.36E-01	12679	8.12E-01	Up	0.24	12733	0.36	0.01
rs6623023	139324	HDX		83479764	4.37E-01	12680	5.17E-01	Up	0.65	10837	0.36	0.03
rs10788875	55657	ZNF692	1	245380462	4.37E-01	12681	1.97E-02	Up	2.33	5055	0.36	0.17
rs952160	9798	KIAA0174	16	70523226	4.37E-01	12682	1.59E-02	Down	2.41	4878	0.36	0.18
rs1342789	56940	DUSP22	6	257023	4.37E-01	12683	5.72E-01	Up	0.57	11258	0.36	0.02
rs11183220	196528	ARID2	12	44523379	4.37E-01	12684	6.57E-02	Up	1.84	6355	0.36	0.12
rs3744231	51081	MRPS7	17	70780396	4.37E-01	12685	2.60E-01	Up	1.13	8862	0.36	0.06
rs3744231	57409	MIF4GD	17	70780396	4.37E-01	12686	7.15E-01	Down	0.36	12164	0.36	0.01
rs3744231	23163	GGA3	17	70780396	4.37E-01	12687	7.58E-01	Down	0.31	12426	0.36	0.01
rs205650	10505	SEMA4F	2	74796266	4.38E-01	12688	7.29E-02	Up	1.79	6484	0.36	0.11
rs1052176	1196	CLK2	1	152073456	4.38E-01	12689	6.70E-03	Up	2.71	4231	0.36	0.22
rs1052176	57657	HCN3	1	152073456	4.38E-01	12690	7.28E-02	Down	1.79	6480	0.36	0.11
rs1052176	2224	FDPS	1	152073456	4.38E-01	12691	1.32E-01	Down	1.51	7463	0.36	0.09
rs1052176	5313	PKLR	1	152073456	4.38E-01	12692	8.67E-01	Up	0.17	13047	0.36	0.01
rs861204	283471	TMPRSS12	12	49524083	4.38E-01	12693	9.31E-01	Down	0.09	13441	0.36	0.00
rs9972591	10116	FEM1B	15	66346504	4.39E-01	12694	1.21E-02	Down	2.51	4639	0.36	0.19
rs6568960	3841	KPNA5	6	117097203	4.39E-01	12695	3.99E-01	Down	0.84	9974	0.36	0.04
rs11870415	79132	DHX58	17	37497533	4.39E-01	12696	1.81E-01	Down	1.34	8035	0.36	0.07
rs6603535	63932	CXorf56		118493887	4.39E-01	12697	7.31E-01	Down	0.34	12261	0.36	0.01
rs11795930	158747	MOSPD2		14648472	4.39E-01	12698	3.40E-01	Up	0.95	9518	0.36	0.05
rs11795930	2187	FANCB		14648472	4.39E-01	12699	7.71E-01	Up	0.29	12506	0.36	0.01
rs2585738	2302	FOXJ1	17	71658328	4.39E-01	12700	1.53E-01	Up	1.43	7725	0.36	0.08
rs6695883	6883	TAF12	1	28759754	4.40E-01	12701	9.01E-01	Up	0.12	13245	0.36	0.00
rs3757460	24146	CLDN15	7	100475075	4.40E-01	12702	7.97E-01	Down	0.26	12648	0.36	0.01
rs773120	5036	PA2G4	12	54789193	4.41E-01	12703	2.54E-01	Up	1.14	8802	0.36	0.06
rs1528402	79635	CCDC121	2	27769168	4.41E-01	12704	4.92E-02	Down	1.97	5973	0.36	0.13
rs9544427	4081	MAB21L1	13	34952972	4.41E-01	12705	5.83E-03	Up	2.76	4160	0.36	0.22
rs17197107	57680	CHD8	14	20924551	4.41E-01	12706	4.11E-03	Up	2.87	3951	0.36	0.24
rs17197107	11198	SUPT16H	14	20924551	4.41E-01	12707	4.23E-01	Down	0.80	10140	0.36	0.04
rs8112449	11140	CDC37	19	10381064	4.41E-01	12708	9.62E-03	Up	2.59	4479	0.36	0.20
rs11126616	80059	LRRTM4	2	77670107	4.42E-01	12709	8.51E-01	Up	0.19	12962	0.35	0.01
rs2028827	51588	PIAS4	19	3975020	4.42E-01	12710	2.01E-02	Up	2.32	5080	0.35	0.17
rs1449272	2297	FOXD1	5	72785919	4.43E-01	12711	1.51E-05	Up	4.33	2038	0.35	0.48
rs3796343	132158	GLYCTK	3	52299209	4.43E-01	12712	3.36E-02	Up	2.13	5577	0.35	0.15

rs12646225	84179	MFSD7	4	686848	4.44E-01	12713	3.88E-02	Down	2.07	5723	0.35	0.14
rs4744648	138241	C9orf85	9	71764154	4.44E-01	12714	7.60E-01	Up	0.31	12439	0.35	0.01
rs2384285	8453	CUL2	10	35377222	4.44E-01	12715	2.61E-02	Down	2.22	5326	0.35	0.16
rs3758549	5309	PITX3	10	103994185	4.44E-01	12716	1.92E-01	Down	1.30	8173	0.35	0.07
rs3752904	9718	ECE2	3	185478770	4.44E-01	12717	3.93E-01	Up	0.85	9932	0.35	0.04
rs3752904	94032	CAMK2N2	3	185478770	4.44E-01	12718	7.36E-01	Up	0.34	12293	0.35	0.01
rs4548800	4707	NDUFB1	14	91656320	4.45E-01	12719	5.43E-05	Up	4.04	2307	0.35	0.43
rs4548800	53981	CPSF2	14	91656320	4.45E-01	12720	2.78E-01	Up	1.08	9020	0.35	0.06
rs3188055	79892	C10orf119	10	121576872	4.45E-01	12721	6.86E-01	Up	0.40	11986	0.35	0.02
rs10413885	56005	C19orf10	19	4607355	4.46E-01	12722	1.87E-10	Up	6.36	794	0.35	0.97
rs4974079	788	SLC25A20	3	48881473	4.46E-01	12723	3.57E-03	Down	2.91	3869	0.35	0.24
rs873132	25945	PVRL3	3	112271782	4.47E-01	12724	5.00E-08	Up	5.45	1224	0.35	0.73
rs1220996	29909	GPR171	3	152412563	4.47E-01	12725	1.18E-01	Up	1.56	7259	0.35	0.09
rs11604561	6207	RPS13	11	17075263	4.48E-01	12726	1.09E-08	Up	5.71	1067	0.35	0.80
rs12817211	91012	LASS5	12	48865665	4.48E-01	12727	3.66E-02	Up	2.09	5669	0.35	0.14
rs1554753	3695	ITGB7	12	51890005	4.48E-01	12728	7.20E-01	Down	0.36	12197	0.35	0.01
rs6130713	7529	YWHAB	20	42976996	4.48E-01	12729	6.65E-01	Up	0.43	11857	0.35	0.02
rs4147451	23126	POGZ	1	148231552	4.49E-01	12730	5.64E-02	Up	1.91	6146	0.35	0.12
rs11852420	10509	SEMA4B	15	88519545	4.49E-01	12731	4.33E-01	Down	0.78	10219	0.35	0.04
rs7885457	1184	CLCN5		49534467	4.49E-01	12732	7.24E-03	Up	2.69	4281	0.35	0.21
rs2278069	10445	MCRS1	12	48230775	4.49E-01	12733	3.59E-01	Down	0.92	9672	0.35	0.04
rs235824	64743	WDR13		48212477	4.50E-01	12734	1.95E-02	Up	2.34	5045	0.35	0.17
rs7259026	84167	C19orf44	19	16484119	4.50E-01	12735	2.18E-01	Down	1.23	8437	0.35	0.07
rs7259026	125972	CALR3	19	16484119	4.50E-01	12736	5.20E-01	Up	0.64	10862	0.35	0.03
rs6884881	1482	NKX2-5	5	172605925	4.50E-01	12737	7.53E-01	Down	0.31	12399	0.35	0.01
rs1216813	9860	LRIG2	1	113357910	4.50E-01	12738	7.55E-01	Up	0.31	12412	0.35	0.01
rs6492325	55608	ANKRD10	13	110334744	4.50E-01	12739	1.79E-01	Up	1.34	8009	0.35	0.07
rs2891868	84924	ZNF566	19	41670024	4.51E-01	12740	2.22E-05	Up	4.24	2106	0.35	0.47
rs2157949	79872	CBLL1	7	106963487	4.52E-01	12741	1.64E-02	Up	2.40	4910	0.35	0.18
rs9292871	3157	HMGCS1	5	43340159	4.53E-01	12742	2.42E-12	Down	7.01	592	0.34	1.16
rs9644704	8658	TNKS	8	9597579	4.53E-01	12743	2.30E-02	Down	2.27	5206	0.34	0.16
rs3759895	9153	SLC28A2	15	43331162	4.53E-01	12744	7.15E-01	Down	0.37	12160	0.34	0.01
rs7756521	780	DDR1	6	30956232	4.53E-01	12745	7.42E-01	Down	0.33	12324	0.34	0.01
rs1754183	22932	POMZP3	7	75874930	4.53E-01	12746	4.43E-04	Up	3.51	2916	0.34	0.34
rs10936158	56925	LXN	3	159886969	4.53E-01	12747	2.53E-01	Down	1.14	8792	0.34	0.06
rs9878797	151827	LRRC34	3	171012660	4.53E-01	12748	2.66E-01	Up	1.11	8916	0.34	0.06
rs9671696	1397	CRIP2	14	105003589	4.54E-01	12749	9.71E-01	Up	0.04	13704	0.34	0.00
rs4794848	51268	PIPOX	17	24382934	4.54E-01	12750	1.72E-02	Down	2.38	4948	0.34	0.18
rs12200607	84085	FBXO30	6	146163830	4.54E-01	12751	1.04E-07	Down	5.32	1300	0.34	0.70
rs4899260	677	ZFP36L1	14	68347957	4.54E-01	12752	2.47E-05	Down	4.22	2127	0.34	0.46
rs6593296	908	CCT6A	7	55892502	4.54E-01	12753	1.34E-02	Up	2.47	4720	0.34	0.19
rs6593296	5723	PSPH	7	55892502	4.54E-01	12754	8.08E-01	Up	0.24	12714	0.34	0.01
rs4787483	10423	CDIPT	16	29792948	4.55E-01	12755	4.85E-01	Down	0.70	10605	0.34	0.03
rs6678136	5999	RGS4	1	159768975	4.55E-01	12756	2.12E-04	Down	3.70	2651	0.34	0.37
rs1378602	3996	LLGL1	17	18052593	4.55E-01	12757	3.39E-02	Up	2.12	5590	0.34	0.15
rs12856464	170685	NUDT10		50895472	4.55E-01	12758	5.59E-11	Down	6.55	722	0.34	1.03
rs8066154	57532	NUFIP2	17	24627212	4.55E-01	12759	1.62E-01	Up	1.40	7834	0.34	0.08
rs4401110	7572	ZNF24	18	31189608	4.56E-01	12760	1.50E-02	Up	2.43	4813	0.34	0.18
rs5960739	29978	UBQLN2		56482703	4.56E-01	12761	4.93E-04	Down	3.48	2956	0.34	0.33
rs10493324	7398	USP1	1	62610163	4.56E-01	12762	1.60E-01	Down	1.41	7813	0.34	0.08
rs9964979	84617	TUBB6	18	12321501	4.56E-01	12763	6.32E-01	Down	0.48	11655	0.34	0.02
rs3130041	2794	GNL1	6	30644112	4.56E-01	12764	1.53E-01	Up	1.43	7719	0.34	0.08
rs3130041	80742	PRR3	6	30644112	4.56E-01	12765	7.06E-01	Down	0.38	12108	0.34	0.02
rs3130041	23	ABCF1	6	30644112	4.56E-01	12766	9.72E-01	Down	0.04	13711	0.34	0.00
rs2904254	1051	CEBPB	20	48245534	4.56E-01	12767	6.92E-04	Down	3.39	3072	0.34	0.32
rs1076230	513	ATP5D	19	1195900	4.56E-01	12768	4.38E-10	Up	6.25	835	0.34	0.94
rs1076230	90007	MIDN	19	1195900	4.56E-01	12769	1.85E-01	Down	1.33	8085	0.34	0.07
rs1076230	255057	C19orf26	19	1195900	4.56E-01	12770	8.85E-01	Down	0.14	13159	0.34	0.01
rs2285415	23354	KIAA0841	19	40814281	4.56E-01	12771	1.70E-01	Up	1.37	7910	0.34	0.08
rs858701	29068	ZBTB44	11	129618647	4.56E-01	12772	3.69E-04	Up	3.56	2853	0.34	0.34
rs3750736	170392	OIT3	10	74341617	4.57E-01	12773	4.93E-02	Up	1.97	5980	0.34	0.13
rs659555	28968	SLC6A16	19	54521637	4.57E-01	12774	1.85E-01	Down	1.33	8077	0.34	0.07
rs659555	951	CD37	19	54521637	4.57E-01	12775	6.90E-01	Up	0.40	12005	0.34	0.02
rs7552175	7252	TSHB	1	115294665	4.57E-01	12776	7.61E-01	Down	0.30	12449	0.34	0.01
rs3738976	6496	SIX3	2	45069889	4.57E-01	12777	9.57E-01	Up	0.05	13597	0.34	0.00
rs819173	191	AHCY	20	32330223	4.57E-01	12778	1.39E-06	Up	4.83	1615	0.34	0.59
rs757114	2657	GDF1	19	18831357	4.58E-01	12779	4.30E-02	Up	2.02	5826	0.34	0.14
rs10783308	10376	TUBA1B	12	47817725	4.58E-01	12780	1.12E-07	Down	5.31	1304	0.34	0.70
rs976751	1823	DSC1	18	27002934	4.58E-01	12781	9.42E-01	Up	0.07	13504	0.34	0.00
rs17725970	342908	ZNF404	19	49079660	4.58E-01	12782	6.64E-03	Up	2.71	4224	0.34	0.22
rs10838692	4607	MYBPC3	11	47301676	4.58E-01	12783	3.17E-01	Down	1.00	9343	0.34	0.05
rs3751877	54700	RRN3	16	15062399	4.58E-01	12784	1.11E-01	Up	1.59	7162	0.34	0.10
rs7599548	200523	C2orf51	2	88657283	4.59E-01	12785	6.31E-01	Down	0.48	11652	0.34	0.02
rs1702669	27290	SPINK4	9	33226164	4.59E-01	12786	4.52E-01	Up	0.75	10357	0.34	0.03
rs1159964	5455	POU3F3	2	104939960	4.59E-01	12787	6.20E-01	Down	0.50	11579	0.34	0.02
rs4934471	3433	IFIT2	10	91061650	4.59E-01	12788	2.28E-04	Down	3.69	2675	0.34	0.36
rs1057640	91828	C14orf73	14	102636657	4.59E-01	12789	6.57E-02	Down	1.84	6358	0.34	0.12
rs960821	5197	PF4V1	4	75092867	4.59E-01	12790	6.11E-01	Up	0.51	11509	0.34	0.02
rs5910595	292	SLC25A5		118389870	4.59E-01	12791	4.48E-13	Up	7.24	527	0.34	1.23
rs11822779	9866	TRIM66	11	8589127	4.60E-01	12792	9.82E-01	Down	0.02	13789	0.34	0.00
rs2500347	9659	PDE4DIP	1	142427364	4.60E-01	12793	1.85E-08	Up	5.62	1118	0.34	0.77

rs10838708	91252	SLC39A13	11	47398089	4.60E-01	12794	1.63E-05	Up	4.31	2055	0.34	0.48
rs10838708	5702	PSMC3	11	47398089	4.60E-01	12795	3.71E-01	Down	0.89	9769	0.34	0.04
rs12516561	8382	NME5	5	137472899	4.60E-01	12796	6.99E-03	Up	2.70	4261	0.34	0.22
rs11796743	23708	GSPT2		51319231	4.60E-01	12797	2.77E-02	Up	2.20	5385	0.34	0.16
rs10971789	54926	UBE2R2	9	33905558	4.60E-01	12798	5.46E-07	Up	5.01	1491	0.34	0.63
rs933489	79957	PAQR6	1	153048193	4.61E-01	12799	4.89E-02	Up	1.97	5969	0.34	0.13
rs4826287	10325	RRAGB		55659801	4.62E-01	12800	5.11E-04	Down	3.48	2967	0.34	0.33
rs10481837	9767	PHF16		46543050	4.62E-01	12801	3.34E-01	Up	0.97	9471	0.34	0.05
rs10504398	4603	MYBL1	8	67649469	4.62E-01	12802	1.16E-01	Down	1.57	7237	0.34	0.09
rs4919593	10360	NPM3	10	103530508	4.62E-01	12803	8.94E-03	Up	2.61	4431	0.34	0.20
rs4919593	10724	MGEA5	10	103530508	4.62E-01	12804	6.18E-02	Down	1.87	6275	0.34	0.12
rs4919593	2253	FGF8	10	103530508	4.62E-01	12805	4.93E-01	Up	0.69	10667	0.34	0.03
rs5983595	286499	FAM133A		92760444	4.62E-01	12806	6.43E-01	Up	0.46	11733	0.34	0.02
rs9351064	10492	SYNCRIP	6	86396825	4.62E-01	12807	3.51E-01	Down	0.93	9599	0.34	0.05
rs1150197	4863	NPAT	11	107574164	4.62E-01	12808	8.04E-02	Down	1.75	6635	0.34	0.11
rs4925	9446	GSTO1	10	106012779	4.62E-01	12809	6.19E-02	Down	1.87	6277	0.34	0.12
rs2708975	10865	ARID5A	2	96606034	4.62E-01	12810	3.63E-02	Down	2.09	5659	0.33	0.14
rs189664	7180	CRISP2	6	49783503	4.63E-01	12811	1.13E-03	Up	3.26	3266	0.33	0.29
rs12031	10953	TOMM34	20	42995074	4.64E-01	12812	2.36E-07	Up	5.17	1391	0.33	0.66
rs2282295	79004	CUEDC2	10	104164885	4.64E-01	12813	8.55E-04	Up	3.33	3156	0.33	0.31
rs17463266	4867	NPHP1	2	110315704	4.64E-01	12814	3.10E-01	Up	1.01	9270	0.33	0.05
rs7209772	23277	KIAA0664	17	2555238	4.64E-01	12815	2.70E-01	Up	1.10	8954	0.33	0.06
rs1545650	5204	PFDN5	12	51987371	4.64E-01	12816	3.61E-05	Down	4.13	2210	0.33	0.44
rs1545650	60314	C12orf10	12	51987371	4.64E-01	12817	4.15E-02	Up	2.04	5788	0.33	0.14
rs4786590	196483	FAM86A	16	5065366	4.64E-01	12818	5.03E-01	Up	0.67	10739	0.33	0.03
rs9915228	51181	DCXR	17	77601176	4.64E-01	12819	2.69E-03	Up	3.00	3728	0.33	0.26
rs10444068	60370	AVPI1	10	99433965	4.65E-01	12820	3.24E-09	Down	5.92	978	0.33	0.85
rs16833613	54165	DCUN1D1	3	184149239	4.65E-01	12821	9.81E-02	Up	1.65	6966	0.33	0.10
rs4746727	26091	HERC4	10	69490915	4.65E-01	12822	7.14E-05	Up	3.97	2367	0.33	0.41
rs2063002	51520	LARS	5	145548070	4.66E-01	12823	3.57E-09	Up	5.92	980	0.33	0.84
rs2075800	3304	HSPA1B	6	31885925	4.66E-01	12824	1.45E-01	Up	1.46	7624	0.33	0.08
rs2075800	3305	HSPA1L	6	31885925	4.66E-01	12825	2.96E-01	Down	1.04	9165	0.33	0.05
rs261532	7322	UBE2D2	5	138929546	4.66E-01	12826	4.45E-01	Up	0.76	10309	0.33	0.04
rs722571	1725	DHPS	19	12672379	4.66E-01	12827	4.19E-14	Up	7.56	448	0.33	1.34
rs722571	84261	FBXW9	19	12672379	4.66E-01	12828	6.90E-01	Down	0.40	12006	0.33	0.02
rs7648992	285267	ZNF619	3	40512020	4.66E-01	12829	8.82E-01	Down	0.15	13139	0.33	0.01
rs2305896	51121	RPL26L1	5	172315508	4.66E-01	12830	3.98E-01	Up	0.85	9968	0.33	0.04
rs1259498	7417	VDAC2	10	76656747	4.66E-01	12831	3.70E-02	Down	2.09	5677	0.33	0.14
rs3810398	56891	LGALS14	19	44879183	4.67E-01	12832	9.43E-01	Down	0.07	13511	0.33	0.00
rs17304039	83786	FRMD8	11	64896785	4.67E-01	12833	9.85E-01	Down	0.02	13812	0.33	0.00
rs11675428	5496	PPM1G	2	27554385	4.67E-01	12834	1.12E-03	Up	3.26	3264	0.33	0.29
rs11965886	10864	SLC22A7	6	43389745	4.68E-01	12835	3.03E-01	Down	1.03	9214	0.33	0.05
rs1336899	55204	GOLPH3L	1	147491983	4.68E-01	12836	4.24E-04	Up	3.52	2900	0.33	0.34
rs17608972	80775	TMEM177	2	120146893	4.68E-01	12837	1.12E-02	Up	2.54	4584	0.33	0.20
rs7104753	5897	RAG2	11	36573443	4.68E-01	12838	1.87E-01	Up	1.32	8109	0.33	0.07
rs2675239	23225	NUP210	3	13455595	4.68E-01	12839	2.30E-16	Up	8.20	336	0.33	1.56
rs9911256	26022	TMEM98	17	28282465	4.68E-01	12840	3.32E-01	Up	0.97	9459	0.33	0.05
rs11696113	128859	BPIL3	20	31076305	4.68E-01	12841	3.86E-01	Up	0.87	9885	0.33	0.04
rs3820011	85452	KIAA1751	1	1920355	4.68E-01	12842	1.71E-01	Down	1.37	7928	0.33	0.08
rs4669765	23620	NTSR2	2	11759252	4.69E-01	12843	1.11E-01	Up	1.60	7154	0.33	0.10
rs2548993	3659	IRF1	5	131836768	4.69E-01	12844	1.34E-01	Down	1.50	7484	0.33	0.09
rs568922	10525	HYOU1	11	118424416	4.69E-01	12845	3.36E-07	Up	5.10	1432	0.33	0.65
rs568922	55823	VPS11	11	118424416	4.69E-01	12846	5.40E-02	Down	1.93	6089	0.33	0.13
rs1064576	10716	TBR1	2	162093615	4.69E-01	12847	7.29E-01	Up	0.35	12245	0.33	0.01
rs588584	7069	THRSP	11	77463484	4.70E-01	12848	6.00E-01	Up	0.53	11428	0.33	0.02
rs588584	4718	NDUFC2	11	77463484	4.70E-01	12849	6.65E-01	Up	0.43	11856	0.33	0.02
rs4661745	26099	C1orf144	1	16473645	4.70E-01	12850	8.16E-01	Up	0.23	12758	0.33	0.01
rs6747253	53938	PPIL3	2	201573921	4.70E-01	12851	5.14E-01	Up	0.65	10811	0.33	0.03
rs11811998	4610	MYCL1	1	40027819	4.70E-01	12852	1.26E-07	Up	5.28	1317	0.33	0.69
rs7953302	10795	ZNF268	12	132381325	4.70E-01	12853	9.66E-01	Down	0.04	13660	0.33	0.00
rs255049	64174	DPEP2	16	66570972	4.71E-01	12854	8.67E-01	Up	0.17	13048	0.33	0.01
rs255049	64180	DPEP3	16	66570972	4.71E-01	12855	9.47E-01	Up	0.07	13531	0.33	0.00
rs6739704	3800	KIF5C	2	149681589	4.71E-01	12856	4.67E-04	Up	3.50	2935	0.33	0.33
rs11079016	317719	KLHL10	17	37229550	4.71E-01	12857	2.95E-01	Down	1.05	9153	0.33	0.05
rs11079016	115024	NT5C3L	17	37229550	4.71E-01	12858	9.45E-01	Down	0.07	13524	0.33	0.00
rs775990	10493	VAT1	17	38412059	4.72E-01	12859	1.71E-07	Down	5.23	1357	0.33	0.68
rs775990	6155	RPL27	17	38412059	4.72E-01	12860	5.02E-06	Up	4.56	1821	0.33	0.53
rs775990	3430	IFI35	17	38412059	4.72E-01	12861	7.09E-02	Down	1.81	6452	0.33	0.11
rs10896016	10534	SSSCA1	11	65092281	4.72E-01	12862	5.26E-05	Down	4.04	2302	0.33	0.43
rs10896016	4054	LTBP3	11	65092281	4.72E-01	12863	7.57E-01	Up	0.31	12421	0.33	0.01
rs6087733	9054	NFS1	20	33762042	4.72E-01	12864	4.38E-04	Up	3.52	2911	0.33	0.34
rs6056	5356	PLRG1	4	155846426	4.73E-01	12865	2.64E-02	Up	2.22	5338	0.33	0.16
rs715158	26658	OR7C2	19	14900616	4.73E-01	12866	3.77E-02	Down	2.08	5699	0.32	0.14
rs225132	54206	ERRFI1	1	8029766	4.74E-01	12867	8.60E-02	Down	1.72	6736	0.32	0.11
rs7889897	158809	MAGEB6		25982179	4.74E-01	12868	9.44E-01	Up	0.07	13522	0.32	0.00
rs11869450	54902	TTC19	17	15864434	4.74E-01	12869	7.19E-01	Up	0.36	12192	0.32	0.01
rs9881563	205717	KIAA2018	3	114896721	4.74E-01	12870	9.62E-06	Up	4.43	1942	0.32	0.50
rs10739927	55035	NOL8	9	92131636	4.75E-01	12871	6.98E-03	Up	2.70	4260	0.32	0.22
rs640030	163786	SASS6	1	100287013	4.75E-01	12872	1.56E-01	Up	1.42	7753	0.32	0.08
rs2074175	9244	CRLF1	19	18561047	4.75E-01	12873	9.01E-02	Up	1.69	6816	0.32	0.10
rs12602084	3292	HSD17B1	17	37965295	4.76E-01	12874	6.36E-01	Down	0.47	11680	0.32	0.02

rs393569	8425	LTBP4	19	45786678	4.76E-01	12875	6.80E-03	Down	2.71	4242	0.32	0.22
rs1909560	1237	CCR8	3	39335221	4.76E-01	12876	4.47E-01	Down	0.76	10327	0.32	0.03
rs12221497	53	ACP2	11	47237229	4.77E-01	12877	9.23E-02	Up	1.68	6868	0.32	0.10
rs11665986	3812	KIR3DL2	19	60075753	4.77E-01	12878	4.16E-01	Down	0.81	10095	0.32	0.04
rs8105066	339327	ZNF546	19	45181310	4.77E-01	12879	1.62E-01	Down	1.40	7839	0.32	0.08
rs1979444	152559	PAQR3	4	80203749	4.77E-01	12880	1.48E-01	Down	1.45	7651	0.32	0.08
rs3002216	5042	PABPC3	13	24579106	4.77E-01	12881	4.48E-04	Up	3.51	2920	0.32	0.33
rs9610342	80831	APOL5	22	34455077	4.78E-01	12882	9.11E-01	Down	0.11	13316	0.32	0.00
rs13358429	3156	HMGCR	5	74653942	4.78E-01	12883	8.19E-01	Up	0.23	12785	0.32	0.01
rs754043	84706	GPT2	16	45502429	4.78E-01	12884	9.72E-02	Up	1.66	6946	0.32	0.10
rs2411759	10642	IGF2BP1	17	44426073	4.78E-01	12885	2.75E-01	Down	1.09	8998	0.32	0.06
rs4150196	83697	SLC4A9	5	139706825	4.78E-01	12886	1.60E-01	Down	1.41	7811	0.32	0.08
rs1877032	84152	PPP1R1B	17	35024590	4.79E-01	12887	1.56E-06	Up	4.80	1635	0.32	0.58
rs1877032	4761	NEUROD2	17	35024590	4.79E-01	12888	9.90E-01	Up	0.01	13842	0.32	0.00
rs579589	4063	LY9	1	157600798	4.79E-01	12889	6.29E-02	Up	1.86	6296	0.32	0.12
rs11987297	116039	OSR2	8	100007646	4.80E-01	12890	2.23E-05	Down	4.24	2107	0.32	0.47
rs200953	3009	HIST1H1B	6	27945246	4.80E-01	12891	4.39E-01	Down	0.77	10263	0.32	0.04
rs250430	113829	SLC35A4	5	139921412	4.80E-01	12892	9.33E-03	Up	2.60	4457	0.32	0.20
rs250430	10011	SRA1	5	139921412	4.80E-01	12893	2.78E-02	Down	2.20	5387	0.32	0.16
rs250430	10307	APBB3	5	139921412	4.80E-01	12894	4.85E-01	Up	0.70	10619	0.32	0.03
rs738703	6676	SPAG4	20	33654113	4.81E-01	12895	5.25E-01	Up	0.64	10902	0.32	0.03
rs3811732		B3GNT5	3	184470217	4.81E-01	12896	5.82E-02	Down	1.89	6191	0.32	0.12
rs549280	2920	CXCL2	4	75336231	4.81E-01	12897	4.06E-02	Down	2.05	5761	0.32	0.14
rs9910801	124961	ZFP3	17	4929970	4.81E-01	12898	2.20E-02	Up	2.29	5158	0.32	0.17
rs4714651	116138	KLHDC3	6	43078485	4.81E-01	12899	7.98E-03	Up	2.65	4341	0.32	0.21
rs4714651	88745	C6orf153	6	43078485	4.81E-01	12900	3.29E-01	Up	0.98	9428	0.32	0.05
rs1121693	8385	OR1D4	17	2932566	4.81E-01	12901	3.42E-01	Up	0.95	9541	0.32	0.05
rs529980	51209	RAB9B		102884368	4.81E-01	12902	1.35E-09	Down	6.06	912	0.32	0.89
rs448374	254528	C16orf73	16	1848257	4.81E-01	12903	6.35E-01	Up	0.47	11673	0.32	0.02
rs16943700	1213	CLTC	17	55055363	4.81E-01	12904	1.83E-03	Up	3.12	3486	0.32	0.27
rs12270283	144124	OR10A5	11	6817416	4.81E-01	12905	5.54E-01	Up	0.59	11111	0.32	0.03
rs235827	5935	RBM3		48193272	4.82E-01	12906	1.36E-03	Up	3.20	3339	0.32	0.29
rs3117143	26692	OR2W1	6	29139121	4.82E-01	12907	5.75E-01	Down	0.56	11274	0.32	0.02
rs687513	54482	CCDC76	1	100310888	4.82E-01	12908	9.80E-05	Up	3.90	2448	0.32	0.40
rs12601110	6147	RPL23A	17	24059462	4.83E-01	12909	7.41E-04	Up	3.37	3101	0.32	0.31
rs1875125	10677	AVIL	12	56513508	4.83E-01	12910	5.17E-01	Down	0.65	10836	0.32	0.03
rs13289459	22927	HABP4	9	96319888	4.84E-01	12911	5.17E-07	Down	5.02	1483	0.32	0.63
rs3862193	55630	SLC39A4	8	145625602	4.84E-01	12912	6.58E-07	Up	4.97	1513	0.32	0.62
rs1886889	8242	JARID1C		53103857	4.84E-01	12913	1.28E-04	Up	3.83	2509	0.32	0.39
rs222851	11337	GABARAP	17	7079962	4.85E-01	12914	8.24E-03	Down	2.64	4363	0.31	0.21
rs7999710	81602	CDADC1	13	48766342	4.85E-01	12915	5.11E-07	Down	5.02	1482	0.31	0.63
rs7497973	4692	NDN	15	21499664	4.85E-01	12916	1.27E-04	Down	3.83	2507	0.31	0.39
rs628462	1325	CORT	1	10445810	4.85E-01	12917	4.68E-01	Up	0.73	10486	0.31	0.03
rs12050732	5604	MAP2K1	15	64537669	4.86E-01	12918	7.88E-07	Up	4.94	1542	0.31	0.61
rs10784408	11260	XPOT	12	63103879	4.86E-01	12919	1.19E-05	Up	4.38	1987	0.31	0.49
rs7650849	79782	LRRC31	3	171079610	4.86E-01	12920	6.14E-01	Up	0.50	11533	0.31	0.02
rs178831	9611	NCOR1	17	16021574	4.86E-01	12921	9.76E-01	Down	0.03	13737	0.31	0.00
rs11078716	116840	CNTROB	17	7760908	4.86E-01	12922	4.91E-01	Up	0.69	10658	0.31	0.03
rs11078716	9196	KCNAB3	17	7760908	4.86E-01	12923	8.78E-01	Down	0.15	13109	0.31	0.01
rs7384157	7697	ZNF138	7	63682847	4.86E-01	12924	8.15E-02	Up	1.74	6660	0.31	0.11
rs3802232	8629	JRK	8	143743703	4.86E-01	12925	2.95E-02	Down	2.18	5447	0.31	0.15
rs3802232	8000	PSCA	8	143743703	4.86E-01	12926	8.81E-01	Up	0.15	13135	0.31	0.01
rs964823	222008	VSTM2A	7	54395343	4.86E-01	12927	2.43E-03	Up	3.03	3665	0.31	0.26
rs2899292	3956	LGALS1	22	36402218	4.87E-01	12928	4.38E-05	Down	4.09	2248	0.31	0.44
rs4806834	268	AMH	19	2211361	4.87E-01	12929	1.60E-02	Up	2.41	4881	0.31	0.18
rs2298630	10449	ACAA2	18	45575834	4.87E-01	12930	6.94E-06	Down	4.50	1871	0.31	0.52
rs1174657	10092	ARPC5	1	180348583	4.87E-01	12931	6.10E-03	Down	2.74	4178	0.31	0.22
rs1174657	403314	APOBEC4	1	180348583	4.87E-01	12932	1.90E-01	Up	1.31	8152	0.31	0.07
rs4241767	55602	CDKN2AIP	4	184728287	4.88E-01	12933	1.13E-04	Down	3.86	2483	0.31	0.39
rs9424932	128077	LIX1L	1	142955137	4.88E-01	12934	5.07E-02	Down	1.95	6009	0.31	0.13
rs9424932	84265	POLR3GL	1	142955137	4.88E-01	12935	9.19E-02	Down	1.69	6859	0.31	0.10
rs3180018	10067	SCAMP3	1	152043204	4.90E-01	12936	8.04E-06	Up	4.46	1904	0.31	0.51
rs3180018	10712	C1orf2	1	152043204	4.90E-01	12937	1.44E-04	Up	3.80	2539	0.31	0.38
rs5933290	51270	TFDP3		132097872	4.90E-01	12938	5.76E-01	Up	0.56	11283	0.31	0.02
rs7723693	10016	PDCD6	5	359032	4.91E-01	12939	1.26E-08	Up	5.66	1093	0.31	0.79
rs6525579	340526	RGAG4		71147085	4.91E-01	12940	1.46E-03	Down	3.18	3366	0.31	0.28
rs2498045	57187	THOC2		122451783	4.91E-01	12941	2.44E-16	Up	8.19	337	0.31	1.56
rs11796927	28952	CCDC22		48844942	4.91E-01	12942	3.33E-02	Up	2.13	5572	0.31	0.15
rs11796927	778	CACNA1F		48844942	4.91E-01	12943	4.25E-01	Down	0.80	10158	0.31	0.04
rs9593836	114798	SLITRK1	13	83350752	4.91E-01	12944	2.36E-01	Down	1.18	8627	0.31	0.06
rs10988	153770	PLAC8L1	5	145480189	4.91E-01	12945	9.79E-01	Up	0.03	13760	0.31	0.00
rs1176811	55071	C9orf40	9	74787861	4.91E-01	12946	5.12E-01	Up	0.66	10794	0.31	0.03
rs3213075	983	CDC2	10	62221282	4.91E-01	12947	3.61E-08	Up	5.51	1187	0.31	0.74
rs11646450	2300	FOXL1	16	85151679	4.92E-01	12948	4.20E-01	Up	0.81	10122	0.31	0.04
rs12563627	23208	SYT11	1	152676039	4.92E-01	12949	3.06E-03	Down	2.96	3785	0.31	0.25
rs2272051	8446	DUSP11	2	73918791	4.92E-01	12950	9.81E-01	Up	0.02	13777	0.31	0.00
rs12150592	51030	FAM18B	17	18620558	4.92E-01	12951	1.38E-01	Up	1.48	7550	0.31	0.09
rs1802029	10870	HCST	19	41087286	4.93E-01	12952	1.05E-01	Up	1.62	7071	0.31	0.10
rs1802029	7305	TYROBP	19	41087286	4.93E-01	12953	5.90E-01	Up	0.54	11368	0.31	0.02
rs3830076	63943	FKBPL	6	32204222	4.93E-01	12954	7.75E-01	Up	0.29	12527	0.31	0.01
rs583338	6860	SYT4	18	39126231	4.94E-01	12955	1.99E-02	Down	2.33	5070	0.31	0.17

rs4587017	7364	UGT2B7	4	70128158	4.94E-01	12956	3.68E-02	Up	2.09	5673	0.31	0.14
rs837763	81620	CDT1	16	87381230	4.94E-01	12957	8.29E-01	Up	0.22	12846	0.31	0.01
rs6463522	51622	C7orf28A	7	5701197	4.94E-01	12958	8.09E-02	Down	1.75	6647	0.31	0.11
rs12196014	6785	ELOVL4	6	80697566	4.94E-01	12959	9.31E-01	Down	0.09	13438	0.31	0.00
rs6778080	375341	C3orf62	3	49292342	4.95E-01	12960	5.27E-01	Up	0.63	10922	0.31	0.03
rs10484434	8358	HIST1H3B	6	26139592	4.95E-01	12961	7.07E-02	Up	1.81	6444	0.31	0.12
rs10484434	8352	HIST1H3C	6	26139592	4.95E-01	12962	8.10E-02	Down	1.74	6649	0.31	0.11
rs10484434	8335	HIST1H2AB	6	26139592	4.95E-01	12963	6.42E-01	Up	0.47	11723	0.31	0.02
rs2271933	3061	HCRTR1	1	31761618	4.96E-01	12964	7.04E-01	Down	0.38	12096	0.30	0.02
rs17501521	5790	PTPRCAP	11	66981564	4.98E-01	12965	9.47E-01	Down	0.07	13530	0.30	0.00
rs968726	26284	ERAL1	17	24218760	4.98E-01	12966	3.47E-02	Up	2.11	5614	0.30	0.15
rs11582620	2312	FLG	1	149099199	4.99E-01	12967	4.52E-01	Up	0.75	10361	0.30	0.03
rs917197	50831	TAS2R3	7	140921153	4.99E-01	12968	4.53E-02	Down	2.00	5882	0.30	0.13
rs917197	50832	TAS2R4	7	140921153	4.99E-01	12969	7.27E-01	Down	0.35	12234	0.30	0.01
rs10005846	8819	SAP30	4	174650243	4.99E-01	12970	9.49E-01	Down	0.06	13548	0.30	0.00
rs7848472	4958	OMD	9	92277026	4.99E-01	12971	7.62E-01	Down	0.30	12451	0.30	0.01
rs2043449	57404	CYP20A1	2	203942196	5.00E-01	12972	1.34E-02	Up	2.47	4714	0.30	0.19
rs2274226	7203	CCT3	1	153127700	5.00E-01	12973	1.69E-09	Up	6.02	931	0.30	0.88
rs2274226	128229	C1orf182	1	153127700	5.00E-01	12974	8.01E-01	Down	0.25	12679	0.30	0.01
rs10500585	5713	PSMD7	16	72882363	5.00E-01	12975	4.91E-02	Down	1.97	5971	0.30	0.13
rs8078815	1411	CRYBA1	17	24602366	5.00E-01	12976	2.44E-04	Down	3.67	2700	0.30	0.36
rs764828	164832	LONRF2	2	100367152	5.01E-01	12977	9.99E-04	Up	3.29	3220	0.30	0.30
rs11098945	80167	C4orf29	4	129318174	5.01E-01	12978	1.60E-02	Up	2.41	4882	0.30	0.18
rs7602534	8890	EIF2B4	2	27504074	5.02E-01	12979	4.80E-07	Up	5.03	1476	0.30	0.63
rs7602534	9784	SNX17	2	27504074	5.02E-01	12980	7.88E-02	Down	1.76	6608	0.30	0.11
rs7602534	130557	ZNF513	2	27504074	5.02E-01	12981	1.13E-01	Up	1.59	7188	0.30	0.09
rs3795393	6275	S100A4	1	150322963	5.02E-01	12982	3.89E-08	Down	5.50	1200	0.30	0.74
rs3795393	6277	S100A6	1	150322963	5.02E-01	12983	1.45E-05	Down	4.34	2031	0.30	0.48
rs3795393	6276	S100A5	1	150322963	5.02E-01	12984	7.56E-02	Down	1.78	6538	0.30	0.11
rs3795393	6274	S100A3	1	150322963	5.02E-01	12985	4.97E-01	Down	0.68	10690	0.30	0.03
rs7812312	137682	C8orf38	8	96089576	5.03E-01	12986	7.46E-02	Up	1.78	6521	0.30	0.11
rs2499043	10857	PGRMC1		118155188	5.03E-01	12987	1.59E-17	Down	8.52	286	0.30	1.68
rs4016338	6866	TAC3	12	55685282	5.03E-01	12988	8.58E-02	Down	1.72	6731	0.30	0.11
rs4016338	9880	ZBTB39	12	55685282	5.03E-01	12989	2.52E-01	Up	1.15	8777	0.30	0.06
rs7105672	6588	SLN	11	107107564	5.03E-01	12990	1.20E-01	Down	1.55	7295	0.30	0.09
rs782428	4171	MCM2	3	128817940	5.03E-01	12991	1.22E-01	Up	1.55	7328	0.30	0.09
rs950166	196410	METTL7B	12	54342579	5.04E-01	12992	5.02E-01	Down	0.67	10732	0.30	0.03
rs2269727	1595	CYP51A1	7	91397341	5.04E-01	12993	1.10E-04	Down	3.87	2476	0.30	0.40
rs9462856	27232	GNMT	6	43034002	5.04E-01	12994	5.96E-02	Up	1.88	6230	0.30	0.12
rs1023064	2857	GPR34		41302497	5.04E-01	12995	4.95E-02	Up	1.96	5983	0.30	0.13
rs570113	29087	THYN1	11	133627460	5.05E-01	12996	8.83E-03	Up	2.62	4418	0.30	0.21
rs4821905	468	ATF4	22	38231110	5.05E-01	12997	7.87E-01	Down	0.27	12594	0.30	0.01
rs1030216	126374	WTIP	19	39650771	5.05E-01	12998	9.13E-01	Down	0.11	13333	0.30	0.00
rs11231168	23193	GANAB	11	62158196	5.05E-01	12999	2.16E-13	Up	7.34	496	0.30	1.27
rs11231168	26229	B3GAT3	11	62158196	5.05E-01	13000	1.15E-02	Up	2.53	4609	0.30	0.19
rs2428236	158798	AKAP14		118817026	5.06E-01	13001	3.56E-01	Down	0.92	9642	0.30	0.04
rs10835678	6240	RRM1	11	4123992	5.06E-01	13002	1.88E-01	Down	1.32	8121	0.30	0.07
rs1567348	5921	RASA1	5	86621630	5.06E-01	13003	1.69E-01	Down	1.38	7907	0.30	0.08
rs2714805	55294	FBXW7	4	153652480	5.07E-01	13004	6.68E-01	Down	0.43	11873	0.30	0.02
rs9400895	23270	TSPYL4	6	116694762	5.07E-01	13005	3.45E-03	Down	2.92	3848	0.30	0.25
rs13233308	154661	RUNDC3B	7	86889611	5.07E-01	13006	5.57E-02	Down	1.91	6131	0.30	0.13
rs8070859	136	ADORA2B	17	15828514	5.07E-01	13007	2.32E-01	Down	1.20	8582	0.29	0.06
rs7732626	53917	RAB24	5	176669915	5.08E-01	13008	1.03E-06	Up	4.88	1577	0.29	0.60
rs7732626	27166	PRELID1	5	176669915	5.08E-01	13009	1.32E-03	Up	3.21	3330	0.29	0.29
rs7732626	83463	MXD3	5	176669915	5.08E-01	13010	3.84E-01	Down	0.87	9866	0.29	0.04
rs2070615	784	CACNB3	12	47504438	5.08E-01	13011	1.12E-02	Up	2.54	4583	0.29	0.20
rs11168910	7846	TUBA1A	12	47872757	5.08E-01	13012	1.09E-08	Down	5.72	1065	0.29	0.80
rs989710	8351	HIST1H3D	6	26314053	5.08E-01	13013	3.12E-05	Up	4.16	2177	0.29	0.45
rs989710	8343	HIST1H2BF	6	26314053	5.08E-01	13014	4.55E-05	Up	4.08	2259	0.29	0.43
rs989710	3012	HIST1H2AE	6	26314053	5.08E-01	13015	5.49E-03	Up	2.78	4125	0.29	0.23
rs989710	8367	HIST1H4E	6	26314053	5.08E-01	13016	5.99E-03	Up	2.75	4172	0.29	0.22
rs989710	3013	HIST1H2AD	6	26314053	5.08E-01	13017	2.91E-02	Up	2.18	5432	0.29	0.15
rs989710	8360	HIST1H4D	6	26314053	5.08E-01	13018	9.92E-01	Up	0.01	13863	0.29	0.00
rs12687891	5456	POU3F4		82573602	5.08E-01	13019	1.83E-01	Up	1.33	8053	0.29	0.07
rs4803	200634	KRTCAP3	2	27578948	5.09E-01	13020	2.84E-05	Up	4.19	2159	0.29	0.45
rs942335	6656	SOX1	13	111751180	5.09E-01	13021	9.99E-01	Up	0.00	13902	0.29	0.00
rs2955359	91647	ATPAF2	17	17887455	5.09E-01	13022	8.73E-02	Up	1.71	6760	0.29	0.11
rs2955359	79018	C17orf39	17	17887455	5.09E-01	13023	9.66E-01	Down	0.04	13661	0.29	0.00
rs2722711	9310	ZNF235	19	49506683	5.09E-01	13024	1.01E-01	Up	1.64	7018	0.29	0.10
rs7071752	84293	C10orf58	10	82149519	5.10E-01	13025	1.03E-08	Down	5.73	1058	0.29	0.80
rs1152187	5030	P2RY4		69261963	5.10E-01	13026	6.87E-01	Down	0.40	11990	0.29	0.02
rs1152187	407	ARR3		69261963	5.10E-01	13027	8.36E-01	Down	0.21	12882	0.29	0.01
rs11651306	4356	MPP3	17	39241297	5.10E-01	13028	5.68E-01	Down	0.57	11225	0.29	0.02
rs10082815	23545	ATP6V0A2	12	122774199	5.11E-01	13029	8.24E-02	Up	1.74	6675	0.29	0.11
rs2231856	2117	ETV3	1	153908543	5.11E-01	13030	2.53E-01	Up	1.14	8791	0.29	0.06
rs1990760	64135	IFIH1	2	162949558	5.12E-01	13031	1.19E-02	Up	2.52	4632	0.29	0.19
rs12870	56616	DIABLO	12	121217700	5.12E-01	13032	5.79E-01	Down	0.55	11310	0.29	0.02
rs5904577	257240	KLHL34		21454330	5.12E-01	13033	5.55E-01	Up	0.59	11114	0.29	0.03
rs4796588	3861	KRT14	17	37003449	5.12E-01	13034	1.48E-11	Down	6.75	654	0.29	1.08
rs11878710	51690	LSM7	19	2287008	5.13E-01	13035	4.26E-06	Up	4.60	1789	0.29	0.54
rs347236	57763	ANKRA2	5	72898045	5.13E-01	13036	9.08E-01	Down	0.12	13294	0.29	0.00

rs913859	6284	S100A13	1	150433159	5.14E-01	13037	3.13E-10	Down	6.29	813	0.29	0.95
rs913859	26097	C1orf77	1	150433159	5.14E-01	13038	1.17E-01	Down	1.57	7249	0.29	0.09
rs2275111	119559	SFXN4	10	120907435	5.14E-01	13039	1.51E-06	Up	4.81	1629	0.29	0.58
rs8072274	339221	ENPP7	17	75316196	5.14E-01	13040	8.09E-01	Up	0.24	12718	0.29	0.01
rs222732	84502	JPH4	14	23129120	5.14E-01	13041	1.29E-10	Down	6.43	771	0.29	0.99
rs2279865	65003	MRPL11	11	65959601	5.14E-01	13042	4.61E-01	Down	0.74	10431	0.29	0.03
rs941686	84301	DDI2	1	15728607	5.14E-01	13043	2.50E-01	Up	1.15	8765	0.29	0.06
rs2874316	84874	ZNF514	2	95231478	5.14E-01	13044	9.79E-01	Down	0.03	13759	0.29	0.00
rs9290469	5627	PROS1	3	95067163	5.15E-01	13045	8.84E-03	Up	2.62	4420	0.29	0.21
rs4807567	55702	CCDC94	19	4229502	5.15E-01	13046	5.36E-01	Down	0.62	10978	0.29	0.03
rs2832837	337959	KRTAP13-2	21	30659580	5.15E-01	13047	3.04E-01	Down	1.03	9226	0.29	0.05
rs11599920	1390	CREM	10	35547170	5.16E-01	13048	4.20E-17	Down	8.41	304	0.29	1.64
rs11748	59307	SIGIRR	11	393980	5.16E-01	13049	4.10E-01	Down	0.82	10054	0.29	0.04
rs7836362	79873	NUDT18	8	22026371	5.17E-01	13050	9.23E-03	Down	2.60	4448	0.29	0.20
rs4239020	9123	SLC16A3	17	77769930	5.17E-01	13051	4.77E-04	Down	3.49	2945	0.29	0.33
rs12449298	84622	ZNF594	17	5045771	5.18E-01	13052	7.48E-01	Up	0.32	12357	0.29	0.01
rs735539	53342	IL17D	13	20178034	5.18E-01	13053	5.81E-01	Down	0.55	11323	0.29	0.02
rs10828247	387640	C10orf140	10	21862862	5.18E-01	13054	1.25E-03	Down	3.23	3314	0.29	0.29
rs4653342	3633	INPP5B	1	38104012	5.18E-01	13055	2.26E-04	Down	3.69	2670	0.29	0.36
rs11882642	2067	ERCC1	19	50625333	5.18E-01	13056	1.03E-04	Up	3.88	2461	0.29	0.40
rs4796754	28511	NKIRAS2	17	37407211	5.18E-01	13057	4.09E-02	Up	2.04	5771	0.29	0.14
rs9954175	5055	SERPINB2	18	59691368	5.19E-01	13058	4.04E-01	Up	0.83	10015	0.29	0.04
rs3797570	1036	CDO1	5	115197819	5.19E-01	13059	8.57E-01	Up	0.18	12993	0.29	0.01
rs11642466	9810	RNF40	16	30689443	5.20E-01	13060	8.11E-01	Down	0.24	12728	0.28	0.01
rs2291903	6599	SMARCC1	3	47692630	5.21E-01	13061	1.88E-22	Up	9.74	153	0.28	2.17
rs11070396	2038	EPB42	15	41310214	5.22E-01	13062	8.87E-01	Down	0.14	13171	0.28	0.01
rs3784230	2972	BRF1	14	104750100	5.22E-01	13063	2.46E-04	Down	3.67	2704	0.28	0.36
rs3887446	9098	USP6	17	4971928	5.22E-01	13064	5.58E-01	Down	0.59	11138	0.28	0.03
rs3887446	7775	ZNF232	17	4971928	5.22E-01	13065	6.80E-01	Down	0.41	11949	0.28	0.02
rs13365490	1587	ADAM3A	8	39518214	5.22E-01	13066	5.44E-04	Up	3.46	2989	0.28	0.33
rs10470517	131118	DNAJC19	3	182208758	5.23E-01	13067	1.11E-01	Up	1.59	7159	0.28	0.10
rs17029662	79982	DNAJB14	4	101204283	5.23E-01	13068	9.10E-01	Up	0.11	13312	0.28	0.00
rs8058922	55159	RFWD3	16	73252580	5.23E-01	13069	3.96E-02	Up	2.06	5742	0.28	0.14
rs12595883	11345	GABARAPL2	16	74141000	5.23E-01	13070	1.45E-03	Down	3.18	3361	0.28	0.28
rs12591947	6642	SNX1	15	62223508	5.23E-01	13071	1.14E-06	Down	4.87	1592	0.28	0.59
rs12052202	10155	TRIM28	19	63740123	5.23E-01	13072	4.79E-05	Up	4.07	2270	0.28	0.43
rs9755490	5576	PRKAR2A	3	48846668	5.23E-01	13073	2.37E-02	Down	2.26	5233	0.28	0.16
rs3759763	51109	RDH11	14	67212693	5.24E-01	13074	3.71E-03	Up	2.90	3886	0.28	0.24
rs4834214	83447	SLC25A31	4	129009832	5.24E-01	13075	9.55E-01	Down	0.06	13586	0.28	0.00
rs653079	79020	C7orf25	7	42738878	5.24E-01	13076	7.28E-03	Down	2.68	4282	0.28	0.21
rs2890915	5000	ORC4L	2	148623275	5.24E-01	13077	5.63E-04	Up	3.45	3003	0.28	0.32
rs7827088	6129	RPL7	8	74381873	5.24E-01	13078	7.95E-04	Up	3.35	3134	0.28	0.31
rs10420134	126393	HSPB6	19	40953768	5.25E-01	13079	1.31E-05	Down	4.36	2014	0.28	0.49
rs1800197	5626	PROP1	5	177352573	5.26E-01	13080	1.35E-01	Down	1.49	7516	0.28	0.09
rs6964005	51657	STYXL1	7	75327534	5.26E-01	13081	1.84E-07	Up	5.22	1362	0.28	0.67
rs6964005	4191	MDH2	7	75327534	5.26E-01	13082	1.88E-04	Up	3.73	2603	0.28	0.37
rs4150893	1875	E2F5	8	86292006	5.26E-01	13083	6.88E-29	Up	11.17	72	0.28	2.82
rs10516140	11282	MGAT4B	5	179173516	5.26E-01	13084	8.40E-08	Up	5.36	1277	0.28	0.71
rs10516140	8878	SQSTM1	5	179173516	5.26E-01	13085	8.18E-04	Down	3.35	3145	0.28	0.31
rs2070235	4605	MYBL2	20	41764871	5.26E-01	13086	2.30E-03	Up	3.05	3631	0.28	0.26
rs2005219	5595	MAPK3	16	30048759	5.27E-01	13087	8.25E-05	Down	3.94	2402	0.28	0.41
rs2005219	79153	GDPD3	16	30048759	5.27E-01	13088	7.44E-02	Down	1.78	6517	0.28	0.11
rs42898	3920	LAMP2		119374576	5.27E-01	13089	8.86E-03	Up	2.62	4424	0.28	0.21
rs190081	10127	ZNF263	16	3263570	5.27E-01	13090	4.38E-01	Up	0.78	10255	0.28	0.04
rs2488769	55917	CTTNBP2NL	1	112648999	5.27E-01	13091	5.52E-04	Up	3.45	2997	0.28	0.33
rs6711843	1961	EGR4	2	73449192	5.28E-01	13092	8.09E-01	Up	0.24	12719	0.28	0.01
rs11672077	163115	ZNF781	19	42859811	5.28E-01	13093	8.56E-03	Down	2.63	4388	0.28	0.21
rs10476222	64324	NSD1	5	176641725	5.28E-01	13094	3.00E-01	Up	1.04	9194	0.28	0.05
rs1078604	4666	NACA	12	55393421	5.28E-01	13095	9.49E-15	Up	7.75	407	0.28	1.40
rs6737156	1716	DGUOK	2	74071389	5.29E-01	13096	8.25E-01	Down	0.22	12817	0.28	0.01
rs3365	6157	RPL27A	11	8661287	5.29E-01	13097	4.86E-05	Up	4.06	2276	0.28	0.43
rs11038977	79797	ZNF408	11	46690960	5.29E-01	13098	5.24E-01	Up	0.64	10901	0.28	0.03
rs2495622	3597	IL13RA1		117634874	5.29E-01	13099	1.54E-02	Up	2.42	4837	0.28	0.18
rs6720131	375189	PFN4	2	24276387	5.29E-01	13100	5.08E-01	Down	0.66	10765	0.28	0.03
rs2965121	23624	CBLC	19	49982525	5.30E-01	13101	2.06E-01	Up	1.26	8318	0.28	0.07
rs7445264	202052	DNAJC18	5	138803451	5.30E-01	13102	2.46E-02	Down	2.25	5272	0.28	0.16
rs1559908	65977	PLEKHA3	2	179161854	5.30E-01	13103	4.17E-03	Down	2.87	3959	0.28	0.24
rs6088273	28954	REM1	20	29509054	5.30E-01	13104	7.91E-02	Down	1.76	6617	0.28	0.11
rs8022613	10901	DHRS4	14	23476758	5.30E-01	13105	4.87E-03	Down	2.82	4048	0.28	0.23
rs2293764	129807	NEU4	2	242478169	5.31E-01	13106	8.63E-01	Up	0.17	13027	0.27	0.01
rs12555631	3449	IFNA16	9	21196427	5.31E-01	13107	6.04E-01	Up	0.52	11458	0.27	0.02
rs1555885	6278	S100A7	1	150265904	5.32E-01	13108	4.33E-01	Down	0.78	10218	0.27	0.04
rs6584300	81894	SLC25A28	10	101375856	5.33E-01	13109	2.89E-02	Down	2.18	5420	0.27	0.15
rs11147250	7556	ZNF10	12	132323790	5.33E-01	13110	9.02E-02	Down	1.69	6824	0.27	0.10
rs3757966	9401	RECQL4	8	145715426	5.33E-01	13111	2.21E-02	Down	2.29	5164	0.27	0.17
rs3757966	9684	LRRC14	8	145715426	5.33E-01	13112	1.53E-01	Up	1.43	7727	0.27	0.08
rs7295247	10024	TROAP	12	47984140	5.33E-01	13113	1.88E-02	Up	2.35	5014	0.27	0.17
rs324748	147660	ZNF578	19	57697415	5.33E-01	13114	7.45E-01	Up	0.32	12349	0.27	0.01
rs3810918	246184	CDC26	9	113116120	5.33E-01	13115	5.91E-02	Down	1.89	6218	0.27	0.12
rs3747303	6191	RPS4X		71278890	5.33E-01	13116	1.54E-04	Up	3.78	2558	0.27	0.38
rs17320750	57529	RGAG1		109486258	5.33E-01	13117	1.19E-01	Up	1.56	7287	0.27	0.09

rs318699	2057	EPOR	19	11362240	5.33E-01	13118	8.74E-04	Up	3.33	3165	0.27	0.31
rs318699	57139	RGL3	19	11362240	5.33E-01	13119	2.29E-01	Up	1.20	8544	0.27	0.06
rs1356168	1290	COL5A2	2	189843605	5.33E-01	13120	4.81E-01	Down	0.70	10581	0.27	0.03
rs6057559	9371	KIF3B	20	30355615	5.34E-01	13121	7.96E-04	Up	3.35	3135	0.27	0.31
rs2859418	222662	LHFPL5	6	35905827	5.34E-01	13122	9.17E-02	Down	1.69	6853	0.27	0.10
rs4319307	6003	RGS13	1	189321409	5.35E-01	13123	3.40E-02	Up	2.12	5591	0.27	0.15
rs6519520	2678	GGT1	22	23316449	5.35E-01	13124	4.54E-01	Up	0.75	10378	0.27	0.03
rs171842	5509	PPP1R3D	20	57968238	5.36E-01	13125	1.65E-01	Up	1.39	7866	0.27	0.08
rs2841523	51406	NOL7	6	13721762	5.36E-01	13126	1.57E-01	Up	1.42	7761	0.27	0.08
rs10813843	138474	TAF1L	9	32615622	5.37E-01	13127	1.58E-01	Down	1.41	7785	0.27	0.08
rs12187921	10826	C5orf4	5	154189993	5.39E-01	13128	4.60E-09	Down	5.86	1000	0.27	0.83
rs11216162	335	APOA1	11	116233487	5.39E-01	13129	1.05E-01	Down	1.62	7082	0.27	0.10
rs9817842	28990	ASTE1	3	132224385	5.39E-01	13130	3.61E-04	Up	3.57	2847	0.27	0.34
rs3824867	5913	RAPSN	11	47425145	5.39E-01	13131	9.37E-01	Up	0.08	13470	0.27	0.00
rs17003003	11279	KLF8		56144046	5.39E-01	13132	7.45E-05	Down	3.96	2373	0.27	0.41
rs6634990	3251	HPRT1		133319541	5.40E-01	13133	7.22E-01	Up	0.36	12209	0.27	0.01
rs10167564	6819	SULT1C2	2	108447143	5.40E-01	13134	1.72E-04	Up	3.76	2578	0.27	0.38
rs1233708	7746	ZNF193	6	28281198	5.40E-01	13135	4.66E-04	Up	3.50	2932	0.27	0.33
rs839375	23630	KCNE1L		108676750	5.40E-01	13136	5.19E-01	Down	0.64	10854	0.27	0.03
rs4786370	9235	IL32	16	3053899	5.40E-01	13137	8.46E-01	Down	0.19	12931	0.27	0.01
rs4855882	63891	RNF123	3	49690358	5.41E-01	13138	4.84E-01	Up	0.70	10602	0.27	0.03
rs7781715	54674	LRRN3	7	110307171	5.42E-01	13139	2.71E-04	Down	3.64	2743	0.27	0.36
rs1015111	55132	LARP2	4	129329962	5.42E-01	13140	9.53E-06	Up	4.43	1939	0.27	0.50
rs4898764	64841	GNPNAT1	14	52334821	5.42E-01	13141	4.26E-17	Up	8.40	305	0.27	1.64
rs9934806	146540	ZNF785	16	30500560	5.43E-01	13142	2.00E-01	Down	1.28	8250	0.27	0.07
rs6526959	4113	MAGEB2		29982452	5.44E-01	13143	4.14E-01	Up	0.82	10083	0.26	0.04
rs597544	10321	CRISP3	6	49811816	5.44E-01	13144	7.41E-14	Up	7.48	463	0.26	1.31
rs917361	4622	MYH4	17	10296488	5.45E-01	13145	6.43E-01	Down	0.46	11732	0.26	0.02
rs1880458	27039	PKD2L2	5	137272416	5.45E-01	13146	1.71E-01	Up	1.37	7931	0.26	0.08
rs12844754	23229	ARHGEF9		62725785	5.45E-01	13147	1.02E-03	Down	3.28	3228	0.26	0.30
rs753725	2968	GTF2H4	6	30998850	5.45E-01	13148	1.28E-01	Up	1.52	7431	0.26	0.09
rs10089769	65265	C8orf33	8	146241933	5.46E-01	13149	5.01E-02	Up	1.96	6004	0.26	0.13
rs7532935	8394	PIP5K1A	1	148023862	5.47E-01	13150	1.24E-01	Up	1.54	7361	0.26	0.09
rs7532935	5710	PSMD4	1	148023862	5.47E-01	13151	7.73E-01	Up	0.29	12521	0.26	0.01
rs1138294	9064	MAP3K6	1	27372775	5.47E-01	13152	3.10E-03	Down	2.96	3792	0.26	0.25
rs13015146	7341	SUMO1	2	202887609	5.47E-01	13153	8.30E-02	Up	1.73	6686	0.26	0.11
rs1006771	2953	GSTT2	22	22638560	5.47E-01	13154	6.91E-02	Up	1.82	6413	0.26	0.12
rs2835272	873	CBR1	21	36374789	5.48E-01	13155	8.02E-01	Down	0.25	12691	0.26	0.01
rs4802138	634	CEACAM1	19	47696238	5.48E-01	13156	8.12E-11	Down	6.50	745	0.26	1.01
rs3027859	51442	VGLL1		135343744	5.48E-01	13157	6.18E-01	Up	0.50	11563	0.26	0.02
rs500161	83638	C11orf68	11	65452014	5.49E-01	13158	5.31E-01	Down	0.63	10946	0.26	0.03
rs500161	10589	DRAP1	11	65452014	5.49E-01	13159	5.78E-01	Down	0.56	11299	0.26	0.02
rs10437653	90993	CREB3L1	11	46254207	5.51E-01	13160	3.77E-13	Up	7.27	518	0.26	1.24
rs3798709	54898	ELOVL2	6	11109262	5.51E-01	13161	4.81E-04	Up	3.49	2950	0.26	0.33
rs13336470	23491	CES3	16	65556871	5.52E-01	13162	6.04E-01	Up	0.52	11462	0.26	0.02
rs2192020	378925	RNF148	7	121947808	5.52E-01	13163	5.33E-01	Up	0.62	10966	0.26	0.03
rs2192020	168433	RNF133	7	121947808	5.52E-01	13164	6.70E-01	Up	0.43	11888	0.26	0.02
rs3805547	56979	PRDM9	5	23563764	5.52E-01	13165	6.67E-01	Up	0.43	11863	0.26	0.02
rs405509	341	APOC1	19	50100676	5.52E-01	13166	6.31E-15	Up	7.89	380	0.26	1.42
rs3794964	27243	CHMP2A	19	63762728	5.53E-01	13167	8.93E-01	Up	0.13	13202	0.26	0.00
rs2290829	29904	EEF2K	16	22203987	5.53E-01	13168	2.58E-05	Down	4.21	2140	0.26	0.46
rs2230282	8693	GALNT4	12	88419505	5.53E-01	13169	4.16E-01	Down	0.81	10094	0.26	0.04
rs1799286	83743	GRWD1	19	53624149	5.54E-01	13170	5.49E-01	Up	0.60	11073	0.26	0.03
rs7740535	202500	TCTE1	6	44382775	5.55E-01	13171	5.89E-01	Up	0.54	11361	0.26	0.02
rs7767980	55274	PHF10	6	169960018	5.56E-01	13172	1.37E-02	Up	2.46	4742	0.25	0.19
rs4350617	374786	EFCAB5	17	25447214	5.56E-01	13173	9.30E-01	Down	0.09	13434	0.25	0.00
rs6017519	6407	SEMG2	20	43288091	5.56E-01	13174	6.20E-02	Up	1.87	6278	0.25	0.12
rs2301137	10233	LRRC23	12	6889210	5.57E-01	13175	4.68E-01	Up	0.73	10488	0.25	0.03
rs803224	27089	UQCRQ	5	132234403	5.57E-01	13176	7.51E-03	Up	2.67	4303	0.25	0.21
rs803224	7381	UQCRB	5	132234403	5.57E-01	13177	4.39E-01	Up	0.77	10258	0.25	0.04
rs9439462	55210	ATAD3A	1	1548033	5.57E-01	13178	7.21E-01	Up	0.36	12201	0.25	0.01
rs559362	26242	OR4C1P	11	55032032	5.58E-01	13179	1.07E-01	Down	1.61	7107	0.25	0.10
rs7726729	25929	GEMIN5	5	154287987	5.58E-01	13180	1.22E-01	Up	1.55	7339	0.25	0.09
rs7667551	51191	HERC5	4	89751084	5.58E-01	13181	3.28E-02	Down	2.13	5552	0.25	0.15
rs737524	2810	SFN	1	26864971	5.58E-01	13182	3.01E-11	Down	6.65	687	0.25	1.05
rs737524	84243	ZDHHC18	1	26864971	5.58E-01	13183	1.42E-01	Up	1.47	7603	0.25	0.08
rs3813155	54967	CXorf48		134019845	5.58E-01	13184	8.30E-01	Down	0.21	12855	0.25	0.01
rs2190245	54429	TAS2R5	7	140937278	5.59E-01	13185	4.93E-03	Down	2.81	4059	0.25	0.23
rs962801	224	ALDH3A2	17	19499687	5.59E-01	13186	5.16E-09	Down	5.84	1015	0.25	0.83
rs11077820	15	AANAT	17	71966263	5.60E-01	13187	7.50E-02	Up	1.78	6529	0.25	0.11
rs3732530	10675	CSPG5	3	47593957	5.60E-01	13188	4.42E-04	Up	3.51	2913	0.25	0.34
rs3087660	10302	SNAPC5	15	64584546	5.60E-01	13189	2.33E-08	Down	5.59	1141	0.25	0.76
rs3087660	6124	RPL4	15	64584546	5.60E-01	13190	2.04E-07	Up	5.20	1373	0.25	0.67
rs10876921	8630	HSD17B6	12	55462453	5.60E-01	13191	1.96E-02	Down	2.33	5053	0.25	0.17
rs5970513	3149	HMGB3		149806077	5.60E-01	13192	3.80E-05	Up	4.12	2217	0.25	0.44
rs7222751	9020	MAP3K14	17	40710713	5.60E-01	13193	2.14E-02	Down	2.30	5137	0.25	0.17
rs7222751	124783	C17orf46	17	40710713	5.60E-01	13194	5.92E-01	Up	0.54	11387	0.25	0.02
rs1977269	280661	EIF1AP1	1	16750156	5.61E-01	13195	1.01E-01	Up	1.64	7012	0.25	0.10
rs7246760	54850	FBXL12	19	9779229	5.62E-01	13196	8.95E-02	Down	1.70	6796	0.25	0.10
rs6596432	51780	JMJD1B	5	137741660	5.62E-01	13197	3.81E-01	Down	0.88	9838	0.25	0.04
rs6736786	3233	HOXD4	2	176834421	5.63E-01	13198	2.13E-02	Down	2.30	5134	0.25	0.17

rs12119764	79363	C1orf89	1	16298078	5.63E-01	13199	3.35E-01	Down	0.96	9484	0.25	0.05
rs8133819	10694	CCT8	21	29380625	5.64E-01	13200	1.53E-01	Up	1.43	7721	0.25	0.08
rs1150082	117247	SLC16A10	6	111617415	5.64E-01	13201	4.46E-01	Up	0.76	10315	0.25	0.04
rs7255779	6822	SULT2A1	19	53084271	5.65E-01	13202	8.42E-01	Up	0.20	12916	0.25	0.01
rs3897489	124808	CCDC43	17	40109399	5.65E-01	13203	1.78E-01	Up	1.35	7996	0.25	0.07
rs6465693	4885	NPTX2	7	97911489	5.65E-01	13204	6.82E-01	Up	0.41	11961	0.25	0.02
rs7780209	8321	FZD1	7	90521451	5.65E-01	13205	6.40E-14	Down	7.50	459	0.25	1.32
rs2123157	123207	C15orf40	15	81454945	5.65E-01	13206	5.22E-02	Down	1.94	6042	0.25	0.13
rs2269911	51330	TNFRSF12A	16	3005925	5.66E-01	13207	1.56E-02	Down	2.42	4846	0.25	0.18
rs2269911	9080	CLDN9	16	3005925	5.66E-01	13208	2.80E-01	Down	1.08	9034	0.25	0.06
rs17012750	10733	PLK4	4	129175163	5.66E-01	13209	1.63E-03	Up	3.15	3427	0.25	0.28
rs9689245	3006	HIST1H1C	6	26158824	5.66E-01	13210	3.11E-05	Up	4.17	2175	0.25	0.45
rs9689245	3018	HIST1H2BB	6	26158824	5.66E-01	13211	1.34E-02	Up	2.47	4718	0.25	0.19
rs6534677	10424	PGRMC2	4	129579506	5.66E-01	13212	9.40E-01	Down	0.07	13496	0.25	0.00
rs1065663	4832	NME3	16	1779024	5.66E-01	13213	2.02E-07	Up	5.20	1371	0.25	0.67
rs1065663	65993	MRPS34	16	1779024	5.66E-01	13214	4.90E-05	Up	4.06	2277	0.25	0.43
rs1065663	10101	NUBP2	16	1779024	5.66E-01	13215	7.18E-01	Up	0.36	12187	0.25	0.01
rs747842	53833	IL20RB	3	138185819	5.67E-01	13216	7.42E-01	Down	0.33	12329	0.25	0.01
rs9623414	171568	POLR3H	22	40255022	5.67E-01	13217	4.11E-01	Up	0.82	10065	0.25	0.04
rs6064733	149685	ADIG	20	36641941	5.68E-01	13218	5.52E-01	Down	0.60	11093	0.25	0.03
rs6104350	11065	UBE2C	20	43857250	5.68E-01	13219	7.00E-08	Up	5.39	1256	0.25	0.72
rs1123373	166012	CHST13	3	127748404	5.69E-01	13220	9.50E-01	Up	0.06	13555	0.24	0.00
rs3094471	83886	PRSS27	16	2693181	5.70E-01	13221	5.62E-02	Down	1.91	6141	0.24	0.13
rs7054102	80316	PPP1R2P9		42413683	5.70E-01	13222	4.19E-01	Up	0.81	10114	0.24	0.04
rs5957416	28985	MCTS1		119509883	5.71E-01	13223	5.46E-05	Up	4.04	2309	0.24	0.43
rs6925243	6204	RPS10	6	34489915	5.71E-01	13224	8.78E-14	Up	7.30	509	0.24	1.31
rs4322170	27018	NGFRAP1		102426592	5.71E-01	13225	4.23E-08	Down	5.48	1208	0.24	0.74
rs2858575	27334	P2RY10		78009386	5.72E-01	13226	1.46E-02	Up	2.44	4788	0.24	0.18
rs7221190	25794	FSCN2	17	77108300	5.72E-01	13227	6.27E-01	Up	0.49	11630	0.24	0.02
rs11731252	10934	MORF4	4	174905657	5.73E-01	13228	4.76E-01	Down	0.71	10545	0.24	0.03
rs7217180	79665	DHX40	17	54979905	5.73E-01	13229	9.31E-01	Up	0.09	13442	0.24	0.00
rs17096232	9337	CNOT8	5	154205142	5.73E-01	13230	7.59E-02	Up	1.78	6547	0.24	0.11
rs1008318	6742	SSBP1	7	140886818	5.74E-01	13231	7.86E-06	Up	4.47	1898	0.24	0.51
rs9887125	347344	ZNF81		47502090	5.74E-01	13232	2.08E-02	Down	2.31	5106	0.24	0.17
rs1572018	89890	KBTBD6	13	40613282	5.74E-01	13233	2.39E-01	Down	1.18	8652	0.24	0.06
rs6490267	5564	PRKAB1	12	118569096	5.74E-01	13234	1.17E-01	Down	1.57	7254	0.24	0.09
rs7459020	7378	UPP1	7	47901836	5.74E-01	13235	2.72E-02	Down	2.21	5372	0.24	0.16
rs3747394	414	ARSD		2840494	5.74E-01	13236	1.26E-01	Up	1.53	7380	0.24	0.09
rs3747394	415	ARSE		2840494	5.74E-01	13237	9.70E-01	Up	0.04	13697	0.24	0.00
rs1776897	3159	HMGA1	6	34302989	5.75E-01	13238	5.37E-02	Up	1.93	6081	0.24	0.13
rs6654021	6322	SCML1		17515665	5.75E-01	13239	1.19E-02	Down	2.52	4631	0.24	0.19
rs11120592	51133	KCTD3	1	212109519	5.75E-01	13240	2.42E-08	Up	5.58	1145	0.24	0.76
rs8077052	348235	FAM33A	17	54563703	5.76E-01	13241	7.59E-01	Down	0.31	12435	0.24	0.01
rs138760	10043	TOM1	22	34026622	5.76E-01	13242	7.31E-01	Up	0.34	12259	0.24	0.01
rs11934856	4825	NKX6-1	4	85796181	5.76E-01	13243	5.61E-01	Up	0.58	11167	0.24	0.03
rs1805873	793	CALB1	8	91150756	5.77E-01	13244	1.96E-01	Up	1.29	8214	0.24	0.07
rs1431975	55421	C17orf85	17	3677728	5.77E-01	13245	2.42E-01	Down	1.17	8681	0.24	0.06
rs12205892	81833	SPACA1	6	88800220	5.78E-01	13246	6.03E-01	Up	0.52	11447	0.24	0.02
rs12757772	8431	NR0B2	1	26935145	5.78E-01	13247	8.00E-01	Down	0.25	12676	0.24	0.01
rs7512076	51060	TXNDC12	1	52198125	5.78E-01	13248	9.83E-03	Up	2.58	4495	0.24	0.20
rs9892427	8557	TCAP	17	35058384	5.78E-01	13249	2.24E-01	Up	1.21	8500	0.24	0.06
rs9892427	5409	PNMT	17	35058384	5.78E-01	13250	5.00E-01	Down	0.67	10718	0.24	0.03
rs9892427	10948	STARD3	17	35058384	5.78E-01	13251	8.33E-01	Down	0.21	12865	0.24	0.01
rs6130946	140831	ZSWIM3	20	43913733	5.78E-01	13252	7.43E-01	Down	0.33	12331	0.24	0.01
rs7501461	284001	CCDC57	17	77703095	5.79E-01	13253	6.80E-06	Up	4.50	1868	0.24	0.52
rs10857253	6189	RPS3A	4	152362389	5.80E-01	13254	4.24E-02	Up	2.03	5811	0.24	0.14
rs16851855	79814	AGMAT	1	15676175	5.80E-01	13255	3.50E-03	Up	2.92	3860	0.24	0.25
rs4938603	283150	FOXR1	11	118351692	5.80E-01	13256	6.14E-01	Down	0.50	11538	0.24	0.02
rs2276852	1951	CELSR3	3	48641927	5.81E-01	13257	4.59E-04	Up	3.50	2925	0.24	0.33
rs7680140	84273	C4orf14	4	57664385	5.82E-01	13258	1.12E-17	Up	8.56	281	0.24	1.70
rs7146643	23241	PACS2	14	104828437	5.82E-01	13259	7.52E-01	Up	0.32	12387	0.24	0.01
rs1791403	115106	CCDC5	18	41969504	5.82E-01	13260	3.08E-01	Up	1.02	9255	0.23	0.05
rs6652410	340547	VSIG1		107087249	5.83E-01	13261	1.27E-01	Up	1.53	7400	0.23	0.09
rs10038	1311	COMP	19	18753728	5.83E-01	13262	1.58E-09	Up	6.04	925	0.23	0.88
rs2285634	8481	OFD1		13497376	5.83E-01	13263	3.97E-03	Up	2.88	3923	0.23	0.24
rs422945	766	CA7	16	65424134	5.85E-01	13264	1.91E-02	Down	2.34	5027	0.23	0.17
rs2240584	8228	PNPLA4		7693630	5.85E-01	13265	3.93E-04	Up	3.54	2878	0.23	0.34
rs7833986	5324	PLAG1	8	57262703	5.86E-01	13266	4.16E-02	Down	2.04	5791	0.23	0.14
rs4788811	6898	TAT	16	70182710	5.86E-01	13267	4.89E-01	Down	0.69	10648	0.23	0.03
rs17136316	7627	ZNF75A	16	3306142	5.86E-01	13268	6.71E-03	Up	2.71	4233	0.23	0.22
rs17136316	91151	TIGD7	16	3306142	5.86E-01	13269	2.90E-02	Up	2.18	5423	0.23	0.15
rs12589666	1215	CMA1	14	24062459	5.86E-01	13270	1.14E-01	Down	1.58	7208	0.23	0.09
rs1916659	5273	SERPINB10	18	59716188	5.86E-01	13271	3.87E-01	Up	0.87	9893	0.23	0.04
rs11654648	90506	LRRC46	17	43268652	5.87E-01	13272	7.36E-01	Down	0.34	12288	0.23	0.01
rs442513	130120	REG3G	2	79152489	5.87E-01	13273	5.65E-02	Down	1.91	6150	0.23	0.12
rs11060112	84660	CCDC62	12	121817383	5.87E-01	13274	5.89E-01	Down	0.54	11366	0.23	0.02
rs1944438	84649	DGAT2	11	75161271	5.89E-01	13275	2.45E-01	Up	1.16	8713	0.23	0.06
rs710251	64834	ELOVL1	1	43495405	5.89E-01	13276	3.82E-02	Down	2.07	5709	0.23	0.14
rs710251	4352	MPL	1	43495405	5.89E-01	13277	3.11E-01	Up	1.01	9279	0.23	0.05
rs710251	991	CDC20	1	43495405	5.89E-01	13278	5.13E-01	Up	0.65	10802	0.23	0.03
rs4578216	10654	PMVK	1	151713794	5.90E-01	13279	5.78E-02	Up	1.90	6181	0.23	0.12

rs863701	116225	ZMYND19	9	137745956	5.91E-01	13280	7.07E-05	Up	3.97	2364	0.23	0.42
rs12136213	116362	RBP7	1	9980726	5.92E-01	13281	1.45E-01	Down	1.46	7628	0.23	0.08
rs11718848	2803	GOLGA4	3	37341463	5.94E-01	13282	8.33E-06	Up	4.46	1911	0.23	0.51
rs6542108	27179	IL1F6	2	113471421	5.94E-01	13283	2.41E-01	Down	1.17	8664	0.23	0.06
rs12752036	55635	DEPDC1	1	68690505	5.95E-01	13284	1.62E-01	Down	1.40	7841	0.23	0.08
rs3813853	91147	TMEM67	8	94821930	5.95E-01	13285	7.99E-04	Up	3.35	3138	0.23	0.31
rs1146581	1744	DLSTP	1	75925045	5.95E-01	13286	6.73E-01	Up	0.42	11903	0.23	0.02
rs1122326	94086	HSPB9	17	37528399	5.95E-01	13287	1.43E-01	Down	1.46	7609	0.23	0.08
rs3729989	6688	SPI1	11	47326617	5.96E-01	13288	3.92E-01	Down	0.86	9925	0.23	0.04
rs3014878	6283	S100A12	1	150156251	5.96E-01	13289	9.23E-01	Down	0.10	13389	0.22	0.00
rs9896514	9720	CCDC144A	17	16528787	5.97E-01	13290	2.56E-02	Up	2.23	5306	0.22	0.16
rs811382	54853	WDR55	5	140037400	5.97E-01	13291	1.84E-08	Up	5.63	1117	0.22	0.77
rs811382	373863	DND1	5	140037400	5.97E-01	13292	1.24E-02	Up	2.50	4655	0.22	0.19
rs811382	3035	HARS	5	140037400	5.97E-01	13293	3.46E-01	Down	0.94	9564	0.22	0.05
rs10401135	84527	ZNF559	19	9304332	5.97E-01	13294	2.75E-01	Up	1.09	8997	0.22	0.06
rs7617214	57047	PLSCR2	3	147665966	5.97E-01	13295	8.87E-01	Up	0.14	13167	0.22	0.01
rs11741240	7478	WNT8A	5	137463629	5.97E-01	13296	6.78E-01	Down	0.42	11931	0.22	0.02
rs10854157	147741	ZNF560	19	9467461	5.97E-01	13297	3.36E-01	Up	0.96	9494	0.22	0.05
rs12035585	84284	C1orf57	1	229409527	5.98E-01	13298	3.02E-04	Up	3.61	2778	0.22	0.35
rs1106287	389	RHOC	1	112954164	5.98E-01	13299	2.97E-03	Down	2.97	3775	0.22	0.25
rs721575	80774	LIMD2	17	59136652	5.99E-01	13300	5.40E-01	Down	0.61	11017	0.22	0.03
rs1124351	58529	MYOZ1	10	75081260	5.99E-01	13301	1.42E-02	Down	2.45	4769	0.22	0.18
rs1124351	79933	SYNPO2L	10	75081260	5.99E-01	13302	9.97E-01	Down	0.00	13890	0.22	0.00
rs7653652	55279	ZNF654	3	88272031	5.99E-01	13303	1.07E-01	Up	1.61	7100	0.22	0.10
rs7653652	285237	C3orf38	3	88272031	5.99E-01	13304	1.96E-01	Up	1.29	8215	0.22	0.07
rs5973840	5716	PSMD10		107125174	5.99E-01	13305	4.67E-01	Down	0.73	10478	0.22	0.03
rs1718969	64418	TMEM168	7	111991273	6.00E-01	13306	1.40E-07	Up	5.27	1337	0.22	0.69
rs6087840	23509	POFUT1	20	30283573	6.00E-01	13307	6.43E-01	Up	0.46	11728	0.22	0.02
rs9379829	8344	HIST1H2BE	6	26280198	6.00E-01	13308	1.04E-02	Up	2.56	4546	0.22	0.20
rs7143637	1511	CTSG	14	24119443	6.01E-01	13309	3.54E-01	Down	0.93	9627	0.22	0.05
rs5916887	56157	TEX13A		104274401	6.01E-01	13310	4.99E-01	Down	0.68	10711	0.22	0.03
rs7612685	8971	H1FX	3	130537036	6.01E-01	13311	6.79E-01	Down	0.41	11940	0.22	0.02
rs805373	10936	GPR75	2	53998246	6.01E-01	13312	5.51E-01	Up	0.60	11085	0.22	0.03
rs5970164	4103	MAGEA4		150769674	6.01E-01	13313	3.43E-02	Up	2.12	5600	0.22	0.15
rs17412284	55450	CAMK2N1	1	20575433	6.01E-01	13314	1.26E-01	Up	1.53	7391	0.22	0.09
rs4760259	83729	INHBE	12	56147093	6.03E-01	13315	1.17E-01	Up	1.57	7251	0.22	0.09
rs12706498	6561	SLC13A1	7	122382616	6.03E-01	13316	8.08E-05	Up	3.94	2393	0.22	0.41
rs6502629	146691	TOM1L2	17	17810367	6.03E-01	13317	4.14E-03	Down	2.87	3954	0.22	0.24
rs6502629	83450	LRRC48	17	17810367	6.03E-01	13318	2.24E-01	Down	1.22	8495	0.22	0.07
rs5937091	8473	OGT		70528344	6.03E-01	13319	6.06E-38	Up	12.82	23	0.22	3.72
rs1125807	57724	KIAA1632	18	41735753	6.03E-01	13320	8.53E-01	Down	0.19	12974	0.22	0.01
rs10984516	55363	HEMGN	9	97798330	6.03E-01	13321	3.19E-01	Up	1.00	9350	0.22	0.05
rs2855475	146547	PRSS36	16	31055049	6.04E-01	13322	2.05E-01	Down	1.27	8304	0.22	0.07
rs6955367	29990	PILRB	7	99605845	6.04E-01	13323	3.30E-05	Up	4.15	2186	0.22	0.45
rs4653440	3020	H3F3A	1	222564457	6.05E-01	13324	5.59E-09	Up	5.83	1020	0.22	0.83
rs4653137	23154	NCDN	1	35678758	6.05E-01	13325	6.47E-03	Up	2.72	4209	0.22	0.22
rs10513432	5028	P2RY1	3	154018063	6.05E-01	13326	4.09E-01	Up	0.83	10049	0.22	0.04
rs4930737	79867	TCTN2	12	122664477	6.06E-01	13327	2.61E-01	Up	1.12	8869	0.22	0.06
rs11260237	10009	ZBTB33		119180979	6.06E-01	13328	1.83E-02	Up	2.36	5000	0.22	0.17
rs5953326	2543	GAGE1		49076810	6.06E-01	13329	7.21E-01	Up	0.36	12207	0.22	0.01
rs17280294	8471	IRS4		107794547	6.07E-01	13330	8.19E-01	Up	0.23	12782	0.22	0.01
rs1122242	79943	ZNF696	8	144458860	6.07E-01	13331	5.58E-01	Down	0.59	11151	0.22	0.03
rs488873	84078	KBTBD7	13	40656416	6.08E-01	13332	8.51E-01	Up	0.19	12964	0.22	0.01
rs12245521	5406	PNLIP	10	118287023	6.09E-01	13333	7.02E-01	Up	0.38	12084	0.22	0.02
rs3774208	7428	VHL	3	10139722	6.09E-01	13334	4.02E-05	Up	4.11	2230	0.22	0.44
rs2834179	3454	IFNAR1	21	33601601	6.09E-01	13335	3.81E-02	Up	2.07	5708	0.22	0.14
rs1279912	84851	TRIM52	5	180623543	6.10E-01	13336	2.66E-02	Up	2.22	5341	0.22	0.16
rs6872579	54882	ANKHD1	5	139756023	6.11E-01	13337	3.87E-04	Up	3.55	2870	0.21	0.34
rs4947510	63979	FIGNL1	7	50299629	6.11E-01	13338	5.01E-03	Up	2.81	4072	0.21	0.23
rs16968038	8639	AOC3	17	38250089	6.11E-01	13339	4.08E-21	Down	9.43	177	0.21	2.04
rs16968038	314	AOC2	17	38250089	6.11E-01	13340	7.13E-02	Up	1.80	6456	0.21	0.11
rs16968038	10197	PSME3	17	38250089	6.11E-01	13341	8.20E-01	Up	0.23	12789	0.21	0.01
rs7585022	29798	C2orf27	2	132330946	6.11E-01	13342	4.81E-01	Up	0.70	10583	0.21	0.03
rs3753362	7388	UQCRH	1	46478771	6.11E-01	13343	2.92E-09	Up	5.94	965	0.21	0.85
rs2276643	79586	CHPF	2	220225456	6.11E-01	13344	2.88E-02	Up	2.19	5417	0.21	0.15
rs12207471	23376	KIAA0776	6	97070503	6.11E-01	13345	6.61E-01	Down	0.44	11834	0.21	0.02
rs1997392	55892	MYNN	3	170992354	6.11E-01	13346	2.82E-01	Up	1.08	9050	0.21	0.05
rs11672943	199745	THAP8	19	41241530	6.11E-01	13347	7.06E-01	Down	0.38	12106	0.21	0.02
rs7502947	55771	PRR11	17	54584498	6.12E-01	13348	2.56E-01	Down	1.14	8818	0.21	0.06
rs6104289	259239	WFDC11	20	43748325	6.12E-01	13349	2.22E-01	Down	1.22	8483	0.21	0.07
rs11902184	10263	CDK2AP2	2	24193135	6.12E-01	13350	4.90E-01	Up	0.69	10655	0.21	0.03
rs1278537	63950	DMRTA2	1	50594179	6.13E-01	13351	8.15E-01	Up	0.23	12753	0.21	0.01
rs401514	9197	SLC33A1	3	157074635	6.13E-01	13352	7.49E-06	Up	4.48	1884	0.21	0.51
rs2273301	5875	RABGGTA	14	23802314	6.14E-01	13353	1.61E-03	Up	3.15	3422	0.21	0.28
rs3827040	90204	ZSWIM1	20	43948966	6.14E-01	13354	3.51E-01	Down	0.93	9604	0.21	0.05
rs12821008	50846	DHH	12	47760872	6.14E-01	13355	2.79E-01	Up	1.08	9028	0.21	0.06
rs5944317	286514	MAGEB18		25916877	6.14E-01	13356	7.36E-02	Up	1.79	6495	0.21	0.11
rs804904	2653	GCSH	16	79664445	6.15E-01	13357	1.60E-03	Up	3.16	3417	0.21	0.28
rs4803586	5672	PSG4	19	48387680	6.15E-01	13358	6.96E-02	Down	1.81	6416	0.21	0.12
rs4803586	5673	PSG5	19	48387680	6.15E-01	13359	7.65E-02	Down	1.77	6564	0.21	0.11
rs4764812	50511	SYCP3	12	100647796	6.16E-01	13360	1.12E-02	Down	2.53	4590	0.21	0.19

rs1063355	3119	HLA-DQB1	6	32735692	6.17E-01	13361	7.64E-07	Up	4.94	1532	0.21	0.61
rs7556852	23559	WBP1	2	74581170	6.17E-01	13362	1.42E-02	Up	2.45	4766	0.21	0.18
rs1872111	6010	RHO	3	130745838	6.18E-01	13363	8.15E-01	Down	0.23	12754	0.21	0.01
rs958305	84775	ZNF607	19	42881280	6.18E-01	13364	1.98E-02	Up	2.33	5066	0.21	0.17
rs6644221	51213	LUZP4		114352228	6.19E-01	13365	6.79E-01	Down	0.41	11942	0.21	0.02
rs7113073	83661	MS4A8B	11	60235369	6.19E-01	13366	8.20E-01	Up	0.23	12791	0.21	0.01
rs436266	85289	KRTAP4-5	17	36567324	6.21E-01	13367	1.90E-01	Down	1.31	8155	0.21	0.07
rs12898794	1198	CLK3	15	72671500	6.21E-01	13368	7.28E-02	Up	1.79	6481	0.21	0.11
rs2336219	6050	RNH1	19	50604246	6.22E-01	13369	9.54E-03	Down	2.59	4473	0.21	0.20
rs2336219	10848	PPP1R13L	19	50604246	6.22E-01	13370	6.55E-02	Down	1.84	6349	0.21	0.12
rs604904	9600	PITPNM1	11	67040467	6.23E-01	13371	4.74E-01	Down	0.72	10526	0.21	0.03
rs604904	51475	CABP2	11	67040467	6.23E-01	13372	9.97E-01	Down	0.00	13894	0.21	0.00
rs15580	10473	HMGN4	6	26654027	6.23E-01	13373	6.26E-30	Down	11.36	65	0.21	2.92
rs11038863	8525	DGKZ	11	46304900	6.24E-01	13374	2.67E-01	Up	1.11	8920	0.21	0.06
rs10908496	112770	C1orf85	1	153077073	6.24E-01	13375	2.88E-01	Down	1.06	9096	0.21	0.05
rs6035877	4821	NKX2-2	20	21460532	6.24E-01	13376	1.54E-02	Up	2.42	4835	0.20	0.18
rs1617182	120425	AMICA1	11	117574159	6.25E-01	13377	2.63E-01	Down	1.12	8887	0.20	0.06
rs3738934	6120	RPE	2	210699752	6.25E-01	13378	3.86E-04	Up	3.55	2868	0.20	0.34
rs4399321	133	ADM	11	10280054	6.26E-01	13379	1.67E-01	Up	1.38	7883	0.20	0.08
rs9972533	4123	MAN2C1	15	73457132	6.26E-01	13380	5.59E-01	Up	0.58	11157	0.20	0.03
rs2861357	5093	PCBP1	2	70214869	6.26E-01	13381	3.51E-03	Down	2.92	3864	0.20	0.25
rs1894386	4114	MAGEB3		29996051	6.26E-01	13382	8.17E-01	Up	0.23	12774	0.20	0.01
rs4641033	6224	RPS20	8	57158229	6.27E-01	13383	7.66E-01	Up	0.30	12466	0.20	0.01
rs4815546	128653	C20orf141	20	2749409	6.27E-01	13384	7.71E-01	Down	0.29	12505	0.20	0.01
rs2438490	134266	GRPEL2	5	148706355	6.28E-01	13385	3.96E-02	Up	2.06	5739	0.20	0.14
rs3740955	5896	RAG1	11	36552176	6.28E-01	13386	5.34E-01	Down	0.62	10969	0.20	0.03
rs4845360	10899	JTB	1	150779733	6.28E-01	13387	3.34E-05	Up	4.15	2190	0.20	0.45
rs4845360	5872	RAB13	1	150779733	6.28E-01	13388	8.61E-01	Down	0.17	13015	0.20	0.01
rs4845360	6232	RPS27	1	150779733	6.28E-01	13389	9.92E-01	Up	0.01	13855	0.20	0.00
rs2413891	1854	DUT	15	46392869	6.28E-01	13390	3.04E-01	Up	1.03	9219	0.20	0.05
rs3109522	170062	FAM47B		34706849	6.28E-01	13391	2.03E-01	Up	1.27	8289	0.20	0.07
rs1045477	25948	KBTBD2	7	32681944	6.29E-01	13392	6.90E-01	Down	0.40	12010	0.20	0.02
rs7120548	23788	MTCH2	11	47619508	6.29E-01	13393	7.19E-10	Up	6.16	872	0.20	0.91
rs602618	150	ADRA2A	10	112833075	6.30E-01	13394	4.12E-04	Up	3.53	2894	0.20	0.34
rs10494878	9261	MAPKAPK2	1	203304204	6.30E-01	13395	2.49E-02	Down	2.24	5280	0.20	0.16
rs2280699	3489	IGFBP6	12	51783965	6.31E-01	13396	8.31E-11	Down	6.49	747	0.20	1.01
rs2517817	80867	HCG2P7	6	29967496	6.31E-01	13397	8.03E-01	Down	0.25	12693	0.20	0.01
rs5937937	22	ABCB7		74052178	6.32E-01	13398	7.34E-01	Up	0.34	12281	0.20	0.01
rs2281680	8906	AP1G2	14	23102910	6.32E-01	13399	1.70E-07	Up	5.23	1356	0.20	0.68
rs4279134	3913	LAMB2	3	49131680	6.33E-01	13400	2.56E-13	Down	7.32	501	0.20	1.26
rs4279134	5859	QARS	3	49131680	6.33E-01	13401	1.01E-06	Up	4.89	1574	0.20	0.60
rs4279134	10869	USP19	3	49131680	6.33E-01	13402	3.92E-01	Up	0.86	9928	0.20	0.04
rs6765828	57018	CCNL1	3	158341002	6.33E-01	13403	5.23E-01	Up	0.64	10887	0.20	0.03
rs2291193	55181	C17orf71	17	54626861	6.33E-01	13404	1.68E-02	Up	2.39	4925	0.20	0.18
rs1015169	139596	UPRT		74304004	6.33E-01	13405	6.33E-01	Up	0.48	11661	0.20	0.02
rs10403583	3963	LGALS7	19	43983611	6.33E-01	13406	4.44E-01	Up	0.77	10299	0.20	0.04
rs4705232	404203	SPINK6	5	147556650	6.34E-01	13407	4.57E-02	Up	2.00	5888	0.20	0.13
rs7539455	10459	MAD2L2	1	11688434	6.34E-01	13408	1.56E-03	Up	3.16	3406	0.20	0.28
rs9271568	3117	HLA-DQA1	6	32698441	6.35E-01	13409	7.95E-03	Up	2.65	4339	0.20	0.21
rs484583	8774	NAPG	18	10545120	6.35E-01	13410	1.30E-03	Down	3.22	3327	0.20	0.29
rs954679	333926	PPM1J	1	112960407	6.36E-01	13411	9.74E-01	Up	0.03	13727	0.20	0.00
rs8453	4893	NRAS	1	114971641	6.37E-01	13412	8.62E-01	Up	0.17	13019	0.20	0.01
rs7754710	892	CCNC	6	100092916	6.38E-01	13413	2.11E-02	Down	2.31	5125	0.20	0.17
rs4969481	201254	STRA13	17	77586501	6.39E-01	13414	8.27E-17	Up	8.34	316	0.19	1.61
rs4969481	5881	RAC3	17	77586501	6.39E-01	13415	3.27E-08	Up	5.55	1158	0.19	0.75
rs4969481	201255	LRRC45	17	77586501	6.39E-01	13416	4.61E-01	Up	0.74	10434	0.19	0.03
rs796398	7162	TPBG	6	83113039	6.40E-01	13417	5.04E-07	Down	5.02	1480	0.19	0.63
rs388389	64430	C14orf135	14	59678210	6.40E-01	13418	7.93E-02	Up	1.75	6621	0.19	0.11
rs2274739	6271	S100A1	1	150425206	6.40E-01	13419	5.58E-02	Down	1.91	6133	0.19	0.13
rs2854344	10161	P2RY5	13	47895694	6.41E-01	13420	9.60E-06	Down	4.43	1941	0.19	0.50
rs7168431	51103	NDUFAF1	15	39459676	6.41E-01	13421	2.21E-01	Down	1.22	8476	0.19	0.07
rs2129301	78995	C17orf53	17	39557004	6.41E-01	13422	9.84E-01	Down	0.02	13805	0.19	0.00
rs17834412	51804	SIX4	14	60256016	6.41E-01	13423	2.51E-08	Up	5.57	1147	0.19	0.76
rs16958780	161779	PGBD4	15	32189560	6.42E-01	13424	6.82E-01	Up	0.41	11959	0.19	0.02
rs893185	25799	ZNF324	19	63681307	6.42E-01	13425	9.77E-02	Down	1.66	6955	0.19	0.10
rs893185	55663	ZNF446	19	63681307	6.42E-01	13426	2.59E-01	Down	1.13	8850	0.19	0.06
rs3027267	84314	TMEM107	17	8031620	6.43E-01	13427	2.72E-02	Down	2.21	5373	0.19	0.16
rs4746136	159195	USP54	10	74971000	6.44E-01	13428	1.72E-03	Up	3.14	3450	0.19	0.28
rs3027471	7030	TFE3		48647584	6.44E-01	13429	1.78E-14	Down	7.67	428	0.19	1.37
rs2627907	762	CA4	17	55573663	6.45E-01	13430	9.53E-02	Down	1.67	6912	0.19	0.10
rs2715556	10052	GJC1	17	35790509	6.45E-01	13431	6.26E-13	Down	7.19	536	0.19	1.22
rs3136685	1236	CCR7	17	35973325	6.45E-01	13432	6.44E-01	Down	0.46	11735	0.19	0.02
rs2625051	205147	FAM123C	2	131346589	6.45E-01	13433	9.42E-01	Up	0.07	13505	0.19	0.00
rs706950	245806	VGLL2	6	117694261	6.45E-01	13434	1.89E-01	Up	1.31	8146	0.19	0.07
rs3743040	23184	MESDC2	15	79057196	6.45E-01	13435	7.34E-02	Up	1.79	6493	0.19	0.11
rs2903813	3315	HSPB1	7	75575440	6.45E-01	13436	7.65E-02	Down	1.77	6565	0.19	0.11
rs10267271	114049	WBSCR22	7	72534070	6.46E-01	13437	7.16E-01	Up	0.36	12165	0.19	0.01
rs6512130	10781	ZNF266	19	9407351	6.47E-01	13438	4.33E-02	Up	2.02	5832	0.19	0.14
rs4801083	284257	FAM44C	18	52955230	6.47E-01	13439	7.35E-01	Down	0.34	12285	0.19	0.01
rs2403088	85444	LRRCC1	8	86229096	6.48E-01	13440	3.17E-03	Down	2.95	3809	0.19	0.25
rs716886	53339	BTBD1	15	81506857	6.49E-01	13441	6.94E-01	Down	0.39	12030	0.19	0.02

rs2278637	6844	VAMP2	17	8002827	6.51E-01	13442	3.85E-01	Down	0.87	9876	0.19	0.04
rs1888576	112487	C14orf126	14	30998407	6.52E-01	13443	2.78E-05	Up	4.19	2154	0.19	0.46
rs3210511	57226	LYRM2	6	90396997	6.52E-01	13444	2.69E-01	Down	1.11	8940	0.19	0.06
rs11653735	1453	CSNK1D	17	77802222	6.53E-01	13445	1.48E-03	Up	3.18	3377	0.19	0.28
rs9534063	386618	KCTD4	13	44682603	6.53E-01	13446	3.14E-01	Down	1.01	9301	0.18	0.05
rs10954732	83862	TMEM120A	7	75255800	6.53E-01	13447	2.99E-01	Up	1.04	9182	0.18	0.05
rs2665390	25976	TIPARP	3	157880451	6.54E-01	13448	1.99E-01	Up	1.29	8243	0.18	0.07
rs2296482	84334	C14orf153	14	103099572	6.54E-01	13449	1.46E-01	Up	1.45	7634	0.18	0.08
rs2296482	9529	BAG5	14	103099572	6.54E-01	13450	5.39E-01	Down	0.61	11002	0.18	0.03
rs2030534	84984	C3orf34	3	197920477	6.55E-01	13451	5.80E-01	Down	0.55	11314	0.18	0.02
rs6059958	81502	HM13	20	29606939	6.55E-01	13452	4.25E-02	Up	2.03	5816	0.18	0.14
rs17757020	56256	SERTAD4	1	206784952	6.55E-01	13453	3.25E-01	Down	0.98	9404	0.18	0.05
rs4925368	11047	ADRM1	20	60294339	6.55E-01	13454	1.07E-02	Down	2.55	4561	0.18	0.20
rs6804473	152302	CIDECP	3	10035425	6.56E-01	13455	3.43E-02	Down	2.12	5598	0.18	0.15
rs11879029	4793	NFKBIB	19	44071746	6.56E-01	13456	4.73E-02	Down	1.98	5930	0.18	0.13
rs3861866	3309	HSPA5	9	125100428	6.56E-01	13457	1.22E-02	Up	2.51	4646	0.18	0.19
rs4981494	5721	PSME2	14	23699931	6.56E-01	13458	8.23E-05	Up	3.94	2401	0.18	0.41
rs4981494	55072	RNF31	14	23699931	6.56E-01	13459	5.41E-01	Up	0.61	11021	0.18	0.03
rs1292337	7957	EPM2A	6	146009500	6.56E-01	13460	6.53E-08	Down	5.40	1251	0.18	0.72
rs17586405	3357	HTR2B	2	231804763	6.57E-01	13461	1.10E-04	Up	3.87	2475	0.18	0.40
rs3817681	203054	ADCK5	8	145553647	6.58E-01	13462	3.11E-01	Down	1.01	9281	0.18	0.05
rs9576693	80209	C13orf23	13	38480048	6.58E-01	13463	4.26E-01	Up	0.80	10164	0.18	0.04
rs6420181	375686	SPATC1	8	145153236	6.59E-01	13464	4.43E-01	Up	0.77	10286	0.18	0.04
rs6420181	84875	PARP10	8	145153236	6.59E-01	13465	4.73E-01	Up	0.72	10522	0.18	0.03
rs10509308	9188	DDX21	10	70367117	6.59E-01	13466	1.42E-06	Up	4.82	1620	0.18	0.58
rs10199511	55676	SLC30A6	2	32320658	6.59E-01	13467	7.44E-03	Up	2.68	4297	0.18	0.21
rs10042892	7025	NR2F1	5	92929338	6.60E-01	13468	1.05E-04	Down	3.88	2467	0.18	0.40
rs4455191	52	ACP1	2	246278	6.60E-01	13469	4.14E-05	Up	4.10	2236	0.18	0.44
rs12937692	92591	ASB16	17	39611956	6.60E-01	13470	1.80E-01	Down	1.34	8023	0.18	0.07
rs12937692	339201	C17orf65	17	39611956	6.60E-01	13471	6.78E-01	Up	0.42	11932	0.18	0.02
rs2271271	23053	KIAA0913	10	75228873	6.61E-01	13472	1.27E-01	Down	1.53	7396	0.18	0.09
rs2271271	8509	NDST2	10	75228873	6.61E-01	13473	3.74E-01	Up	0.89	9797	0.18	0.04
rs9426442	115273	RAB42	1	28722071	6.62E-01	13474	1.29E-01	Up	1.52	7433	0.18	0.09
rs11576830	5202	PFDN2	1	157881909	6.62E-01	13475	6.99E-02	Up	1.81	6424	0.18	0.12
rs11576830	4817	NIT1	1	157881909	6.62E-01	13476	3.02E-01	Down	1.03	9202	0.18	0.05
rs7141	1949	EFNB3	17	7555326	6.62E-01	13477	3.63E-01	Down	0.91	9706	0.18	0.04
rs291107	11009	IL24	1	203463566	6.64E-01	13478	5.56E-01	Up	0.59	11126	0.18	0.03
rs7199018	5934	RBL2	16	52020053	6.64E-01	13479	9.22E-01	Up	0.10	13384	0.18	0.00
rs633742	10629	TAF6L	11	62301922	6.65E-01	13480	4.72E-01	Up	0.72	10514	0.18	0.03
rs8040336	10169	SERF2	15	41840909	6.65E-01	13481	3.40E-01	Up	0.95	9523	0.18	0.05
rs2784466	149466	C1orf210	1	43421070	6.66E-01	13482	9.68E-01	Down	0.04	13680	0.18	0.00
rs2805053	51478	HSD17B7	1	159513445	6.66E-01	13483	9.91E-02	Down	1.65	6983	0.18	0.10
rs2805053	158160	HSD17B7P2	1	159513445	6.66E-01	13484	5.30E-01	Up	0.63	10943	0.18	0.03
rs948588	4089	SMAD4	18	46840342	6.67E-01	13485	1.30E-03	Down	3.22	3325	0.18	0.29
rs7265661	60625	DHX35	20	37020256	6.69E-01	13486	2.49E-02	Down	2.24	5283	0.17	0.16
rs9837294	200894	ARL13B	3	95241769	6.69E-01	13487	9.28E-01	Down	0.09	13415	0.17	0.00
rs1602084	256471	MFSD8	4	129201085	6.72E-01	13488	1.01E-01	Up	1.64	7023	0.17	0.10
rs1052080	285521	COX18	4	74285657	6.72E-01	13489	3.45E-01	Up	0.94	9558	0.17	0.05
rs4435493	6233	RPS27A	2	55360969	6.73E-01	13490	2.74E-02	Up	2.21	5377	0.17	0.16
rs4236927	2339	FNTA	8	43056765	6.73E-01	13491	7.56E-04	Down	3.37	3111	0.17	0.31
rs174764	10633	RASL10A	22	28045715	6.73E-01	13492	9.52E-01	Up	0.06	13567	0.17	0.00
rs2071309	4115	MAGEB4		30014033	6.74E-01	13493	5.53E-01	Down	0.59	11099	0.17	0.03
rs6609159	27197	GPR82		41329820	6.75E-01	13494	7.58E-01	Up	0.31	12429	0.17	0.01
rs2305517	55217	TMLHE		154338367	6.75E-01	13495	3.89E-05	Down	4.11	2222	0.17	0.44
rs6716800	7851	MALL	2	110214618	6.76E-01	13496	6.60E-02	Down	1.84	6359	0.17	0.12
rs3936211	2907	GRINA	8	145133612	6.77E-01	13497	9.00E-08	Up	5.35	1285	0.17	0.70
rs13037087	6406	SEMG1	20	43267727	6.77E-01	13498	4.22E-02	Down	2.03	5801	0.17	0.14
rs3744383	284161	GDPD1	17	54645165	6.78E-01	13499	1.02E-04	Up	3.89	2458	0.17	0.40
rs1799281	3770	KCNJ14	19	53640582	6.79E-01	13500	7.21E-01	Up	0.36	12204	0.17	0.01
rs2042200	79088	ZNF426	19	9507898	6.80E-01	13501	9.03E-02	Up	1.69	6828	0.17	0.10
rs1932919	55613	MTMR8		63336872	6.80E-01	13502	3.43E-02	Up	2.12	5599	0.17	0.15
rs2983296	10047	CST8	20	23427088	6.81E-01	13503	4.54E-03	Down	2.84	4002	0.17	0.23
rs3849174	6566	SLC16A1	1	113172959	6.81E-01	13504	3.98E-03	Up	2.88	3926	0.17	0.24
rs4835726	9963	SLC23A1	5	138761710	6.81E-01	13505	1.32E-02	Up	2.48	4703	0.17	0.19
rs1465542	113675	SDSL	12	112325663	6.81E-01	13506	1.12E-01	Up	1.59	7169	0.17	0.10
rs11747579	4888	NPY6R	5	137168963	6.81E-01	13507	4.99E-02	Down	1.96	5995	0.17	0.13
rs4531702	646	BNC1	15	81708383	6.83E-01	13508	2.00E-01	Up	1.28	8253	0.17	0.07
rs5925336	4100	MAGEA1		151987490	6.83E-01	13509	2.93E-02	Up	2.18	5437	0.17	0.15
rs6130940	7125	TNNC2	20	43881699	6.84E-01	13510	1.67E-02	Down	2.39	4923	0.17	0.18
rs2041126	4626	MYH8	17	10257004	6.85E-01	13511	8.96E-02	Down	1.70	6797	0.16	0.10
rs1927282	90737	PAGE5		55113309	6.85E-01	13512	1.59E-01	Down	1.41	7807	0.16	0.08
rs329363	9833	MELK	9	36546091	6.85E-01	13513	2.47E-02	Up	2.25	5277	0.16	0.16
rs10088698	1052	CEBPD	8	48817337	6.86E-01	13514	1.24E-01	Down	1.54	7353	0.16	0.09
rs4642918	26245	OR2M4	1	244737098	6.87E-01	13515	2.69E-01	Up	1.11	8941	0.16	0.06
rs2005390	3284	HSD3B2	1	119652423	6.88E-01	13516	2.57E-01	Down	1.13	8836	0.16	0.06
rs6661480	91408	BTF3L4	1	52249576	6.89E-01	13517	9.28E-07	Up	4.91	1567	0.16	0.60
rs3734166	995	CDC25C	5	137693222	6.89E-01	13518	2.47E-02	Up	2.25	5273	0.16	0.16
rs1565922	93210	PERLD1	17	35084561	6.90E-01	13519	2.58E-03	Down	3.01	3705	0.16	0.26
rs679626	5436	POLR2G	11	62297808	6.91E-01	13520	1.29E-02	Up	2.49	4682	0.16	0.19
rs679626	79842	ZBTB3	11	62297808	6.91E-01	13521	1.95E-02	Up	2.34	5048	0.16	0.17
rs175169	29801	ZDHHC8	22	18491899	6.91E-01	13522	1.05E-01	Up	1.62	7080	0.16	0.10

rs1474056	114900	C1QTNF4	11	47589283	6.93E-01	13523	5.32E-01	Down	0.62	10958	0.16	0.03
rs3126174	382	ARF6	14	49412701	6.93E-01	13524	3.70E-07	Up	5.08	1444	0.16	0.64
rs1233434	353355	ZNF233	19	49480964	6.95E-01	13525	5.00E-04	Up	3.48	2960	0.16	0.33
rs213627	79000	C1orf135	1	25856257	6.97E-01	13526	8.38E-01	Up	0.20	12890	0.16	0.01
rs8038527	57611	ISLR2	15	72221249	6.97E-01	13527	2.90E-01	Down	1.06	9111	0.16	0.05
rs6031782	60598	KCNK15	20	42817246	6.97E-01	13528	1.76E-01	Up	1.35	7972	0.16	0.08
rs3745004	498	ATP5A1	18	41914398	6.97E-01	13529	5.16E-01	Down	0.65	10827	0.16	0.03
rs12744386	2517	FUCA1	1	23913325	6.98E-01	13530	5.87E-02	Up	1.89	6211	0.16	0.12
rs310475	51545	ZNF581	19	60845680	6.98E-01	13531	2.31E-03	Up	3.05	3636	0.16	0.26
rs310475	51157	ZNF580	19	60845680	6.98E-01	13532	7.94E-01	Up	0.26	12632	0.16	0.01
rs310475	29903	CCDC106	19	60845680	6.98E-01	13533	9.39E-01	Down	0.08	13485	0.16	0.00
rs2216058	84290	CAPNS2	16	54152312	6.98E-01	13534	2.95E-01	Down	1.05	9156	0.16	0.05
rs10733788	29982	NRBF2	10	64587871	6.98E-01	13535	7.07E-01	Up	0.38	12111	0.16	0.02
rs1382357	148266	ZNF569	19	42669449	6.99E-01	13536	3.97E-02	Down	2.06	5743	0.16	0.14
rs111524	117286	CIB3	19	16126494	7.00E-01	13537	8.78E-01	Down	0.15	13108	0.16	0.01
rs893546	90589	ZNF625	19	12135521	7.00E-01	13538	3.84E-01	Down	0.87	9872	0.15	0.04
rs893546	7695	ZNF136	19	12135521	7.00E-01	13539	4.52E-01	Up	0.75	10358	0.15	0.03
rs3856557	375287	RBM43	2	151962469	7.01E-01	13540	7.98E-01	Down	0.26	12663	0.15	0.01
rs38725	51650	MRPS33	7	140152604	7.01E-01	13541	4.59E-11	Up	6.64	691	0.15	1.03
rs6037206	26152	ZNF337	20	25636001	7.01E-01	13542	5.34E-01	Down	0.62	10973	0.15	0.03
rs10131341	8115	TCL1A	14	95252369	7.01E-01	13543	2.47E-01	Down	1.16	8732	0.15	0.06
rs11242213	51265	CDKL3	5	133747910	7.01E-01	13544	1.74E-03	Down	3.13	3458	0.15	0.28
rs11242213	7320	UBE2B	5	133747910	7.01E-01	13545	7.48E-02	Down	1.78	6524	0.15	0.11
rs10512488	124817	CNTD1	17	38217430	7.03E-01	13546	2.15E-01	Up	1.24	8408	0.15	0.07
rs10512488	28958	CCDC56	17	38217430	7.03E-01	13547	2.52E-01	Down	1.14	8784	0.15	0.06
rs10512488	8678	BECN1	17	38217430	7.03E-01	13548	7.07E-01	Down	0.38	12114	0.15	0.02
rs11072096	9493	KIF23	15	67504090	7.03E-01	13549	5.80E-04	Up	3.44	3015	0.15	0.32
rs4837238	114789	SLC25A25	9	127942267	7.03E-01	13550	5.61E-02	Up	1.91	6137	0.15	0.13
rs1044581	80381	CD276	15	71793744	7.04E-01	13551	1.78E-01	Down	1.35	7993	0.15	0.07
rs6980337	129790	C7orf13	7	155934022	7.04E-01	13552	7.70E-02	Up	1.77	6575	0.15	0.11
rs7773163	8346	HIST1H2BI	6	26375779	7.04E-01	13553	7.67E-03	Up	2.67	4319	0.15	0.21
rs7773163	8355	HIST1H3G	6	26375779	7.04E-01	13554	7.45E-01	Up	0.33	12347	0.15	0.01
rs10744720	7167	TPI1	12	6834474	7.04E-01	13555	3.61E-01	Up	0.91	9686	0.15	0.04
rs8063431	197342	EME2	16	1744358	7.04E-01	13556	2.91E-01	Up	1.06	9118	0.15	0.05
rs11060180	9026	HIP1R	12	121828466	7.04E-01	13557	4.69E-01	Up	0.72	10492	0.15	0.03
rs7975791	5571	PRKAG1	12	47699753	7.04E-01	13558	5.34E-01	Up	0.62	10968	0.15	0.03
rs2070822	2316	FLNA		153100295	7.05E-01	13559	3.83E-16	Down	8.14	342	0.15	1.54
rs11076522	64282	PAPD5	16	48800875	7.05E-01	13560	1.78E-03	Down	3.12	3471	0.15	0.27
rs4452075	84503	ZNF527	19	42571429	7.06E-01	13561	8.55E-01	Down	0.18	12983	0.15	0.01
rs11207993	27329	ANGPTL3	1	62756485	7.06E-01	13562	7.57E-01	Up	0.31	12420	0.15	0.01
rs5933120	57826	RAP2C		131057127	7.07E-01	13563	3.40E-02	Down	2.12	5593	0.15	0.15
rs11814305	83742	MARVELD1	10	99470543	7.07E-01	13564	6.46E-03	Down	2.72	4206	0.15	0.22
rs12054518	51574	LARP7	4	113907593	7.09E-01	13565	9.37E-03	Down	2.60	4461	0.15	0.20
rs2811750	29085	PHPT1	9	137000957	7.10E-01	13566	4.52E-01	Up	0.75	10354	0.15	0.03
rs2366267	10270	AKAP8	19	15353848	7.11E-01	13567	3.84E-03	Down	2.89	3906	0.15	0.24
rs535056	5453	POU3F1	1	38191185	7.11E-01	13568	3.42E-01	Down	0.95	9540	0.15	0.05
rs10917360	3352	HTR1D	1	23284316	7.11E-01	13569	4.99E-01	Down	0.68	10706	0.15	0.03
rs1662	387893	SETD8	12	122425016	7.11E-01	13570	1.74E-01	Up	1.36	7950	0.15	0.08
rs720583	680	BRS3		135283761	7.12E-01	13571	8.15E-01	Up	0.23	12757	0.15	0.01
rs942024	122416	ANKRD9	14	102049216	7.12E-01	13572	3.95E-01	Down	0.85	9945	0.15	0.04
rs6826194	60558	GUF1	4	44504106	7.12E-01	13573	1.36E-06	Up	4.83	1612	0.15	0.59
rs1988065	5669	PSG1	19	48066441	7.12E-01	13574	6.67E-01	Up	0.43	11867	0.15	0.02
rs3739942	8721	EDF1	9	137052237	7.13E-01	13575	4.64E-02	Up	1.99	5905	0.15	0.13
rs1860433	83695	C12orf32	12	2858205	7.13E-01	13576	3.43E-04	Down	3.58	2827	0.15	0.35
rs6799805	51068	NMD3	3	162419066	7.14E-01	13577	1.57E-02	Up	2.42	4858	0.15	0.18
rs3087484	84950	PRPF38A	1	52562467	7.14E-01	13578	2.10E-02	Down	2.31	5114	0.15	0.17
rs3087474	4998	ORC1L	1	52559492	7.14E-01	13579	4.01E-02	Up	2.05	5750	0.15	0.14
rs736222	2862	MLNR	13	48674341	7.16E-01	13580	4.56E-01	Up	0.75	10390	0.15	0.03
rs9419406	664	BNIP3	10	133661191	7.16E-01	13581	6.69E-05	Up	3.99	2350	0.15	0.42
rs7208838	55288	RHOT1	17	27492679	7.17E-01	13582	3.43E-01	Up	0.95	9547	0.14	0.05
rs9288967	29083	GTPBP8	3	114200009	7.18E-01	13583	4.91E-02	Up	1.97	5972	0.14	0.13
rs16974263	147746	HIPK4	19	45605379	7.18E-01	13584	7.28E-02	Up	1.79	6483	0.14	0.11
rs16974263	57716	PRX	19	45605379	7.18E-01	13585	7.52E-01	Down	0.32	12390	0.14	0.01
rs7718958	819	CAMLG	5	134121831	7.19E-01	13586	5.50E-03	Up	2.78	4128	0.14	0.23
rs653914	8061	FOSL1	11	65433092	7.19E-01	13587	1.04E-01	Down	1.62	7068	0.14	0.10
rs606443	8683	SFRS9	12	119373350	7.20E-01	13588	1.36E-08	Up	5.66	1090	0.14	0.79
rs17336718	8277	TKTL1		153056966	7.20E-01	13589	1.21E-01	Up	1.55	7303	0.14	0.09
rs8047148	1039	CDR2	16	22282331	7.21E-01	13590	5.23E-02	Down	1.94	6044	0.14	0.13
rs1128863	79184	BRCC3		153920566	7.21E-01	13591	2.04E-05	Up	4.26	2092	0.14	0.47
rs11671846	284340	CXCL17	19	47632143	7.22E-01	13592	5.21E-03	Down	2.79	4098	0.14	0.23
rs11671846	3991	LIPE	19	47632143	7.22E-01	13593	5.93E-01	Up	0.53	11389	0.14	0.02
rs1553578	5734	PTGER4	5	40704941	7.22E-01	13594	1.53E-01	Down	1.43	7716	0.14	0.08
rs10857706	282969	C10orf125	10	135067492	7.23E-01	13595	5.99E-01	Up	0.53	11422	0.14	0.02
rs13082820	22865	SLITRK3	3	166409307	7.25E-01	13596	9.64E-02	Down	1.66	6928	0.14	0.10
rs8060686	10204	NUTF2	16	66469018	7.26E-01	13597	3.11E-01	Down	1.01	9278	0.14	0.05
rs8060686	23644	EDC4	16	66469018	7.26E-01	13598	7.48E-01	Up	0.32	12359	0.14	0.01
rs8060686	5681	PSKH1	16	66469018	7.26E-01	13599	9.00E-01	Down	0.13	13240	0.14	0.00
rs3809504	6176	RPLP1	15	67531184	7.26E-01	13600	6.60E-01	Up	0.44	11831	0.14	0.02
rs6726677	84365	MKI67IP	2	122206162	7.26E-01	13601	4.50E-06	Up	4.59	1796	0.14	0.53
rs11626	389125	MUSTN1	3	52849024	7.27E-01	13602	6.29E-05	Down	4.00	2340	0.14	0.42
rs4968647	1534	CYB561	17	58881409	7.28E-01	13603	8.60E-02	Up	1.72	6735	0.14	0.11

rs3781216	118487	CHCHD1	10	75192410	7.28E-01	13604	1.71E-05	Up	4.30	2063	0.14	0.48
rs3781216	170384	FUT11	10	75192410	7.28E-01	13605	2.60E-02	Up	2.23	5321	0.14	0.16
rs3781216	9632	SEC24C	10	75192410	7.28E-01	13606	4.33E-01	Up	0.78	10216	0.14	0.04
rs12640665	132789	GNPDA2	4	44567599	7.29E-01	13607	2.97E-02	Up	2.17	5451	0.14	0.15
rs2277665	147015	DHRS13	17	24262201	7.29E-01	13608	3.73E-03	Up	2.90	3890	0.14	0.24
rs17468652	80761	UPK3B	7	75781040	7.29E-01	13609	5.55E-01	Up	0.59	11121	0.14	0.03
rs17468652	113878	DTX2	7	75781040	7.29E-01	13610	7.79E-01	Up	0.28	12542	0.14	0.01
rs2277016	5459	POU4F3	5	145701438	7.31E-01	13611	1.14E-01	Down	1.58	7197	0.14	0.09
rs9782	429	ASCL1	12	101854293	7.31E-01	13612	2.66E-02	Down	2.22	5340	0.14	0.16
rs7515488	51150	SDF4	1	1203727	7.33E-01	13613	3.53E-06	Up	4.64	1762	0.14	0.55
rs7515488	7293	TNFRSF4	1	1203727	7.33E-01	13614	1.36E-01	Up	1.49	7524	0.14	0.09
rs7515488		B3GALT6	1	1203727	7.33E-01	13615	8.12E-01	Up	0.24	12736	0.14	0.01
rs4911222	171023	ASXL1	20	30424861	7.34E-01	13616	7.37E-01	Down	0.34	12298	0.13	0.01
rs11650232	432	ASGR1	17	7029647	7.34E-01	13617	8.11E-01	Up	0.24	12730	0.13	0.01
rs17154203	54948	MRPL16	11	59325519	7.36E-01	13618	1.10E-04	Up	3.87	2479	0.13	0.40
rs7745708	10957	PNRC1	6	89857997	7.37E-01	13619	3.30E-06	Down	4.65	1755	0.13	0.55
rs585535	4435	CITED1		71327310	7.37E-01	13620	9.19E-01	Down	0.10	13373	0.13	0.00
rs9660185	84614	ZBTB37	1	170563758	7.37E-01	13621	3.02E-01	Down	1.03	9200	0.13	0.05
rs3735840	29894	CPSF1	8	145623814	7.38E-01	13622	1.04E-04	Up	3.88	2465	0.13	0.40
rs3752221	4713	NDUFB7	19	14538312	7.38E-01	13623	5.24E-01	Up	0.64	10892	0.13	0.03
rs2280743	79713	TMEM149	19	40917839	7.39E-01	13624	4.68E-01	Down	0.72	10489	0.13	0.03
rs2280743	55957	LIN37	19	40917839	7.39E-01	13625	5.13E-01	Down	0.65	10805	0.13	0.03
rs1204418	402415	XKRX		99987747	7.39E-01	13626	4.69E-01	Down	0.72	10493	0.13	0.03
rs17271418	11133	KPTN	19	52677032	7.39E-01	13627	2.31E-01	Up	1.20	8573	0.13	0.06
rs440655	56985	C17orf48	17	10558528	7.39E-01	13628	7.56E-02	Down	1.78	6540	0.13	0.11
rs440655	6341	SCO1	17	10558528	7.39E-01	13629	4.20E-01	Up	0.81	10127	0.13	0.04
rs12912744	146050	ZSCAN29	15	41446227	7.41E-01	13630	1.48E-01	Up	1.45	7658	0.13	0.08
rs12912744	27229	TUBGCP4	15	41446227	7.41E-01	13631	5.16E-01	Down	0.65	10823	0.13	0.03
rs7628719	10425	ARIH2	3	48970491	7.42E-01	13632	3.90E-02	Down	2.06	5730	0.13	0.14
rs2072704	27173	SLC39A1	1	150748616	7.43E-01	13633	4.61E-02	Down	1.99	5897	0.13	0.13
rs2072704	200186	CRTC2	1	150748616	7.43E-01	13634	7.22E-01	Up	0.36	12210	0.13	0.01
rs7653960	6423	SFRP2	4	155055035	7.43E-01	13635	2.10E-03	Down	3.08	3565	0.13	0.27
rs10882891	10023	FRAT1	10	99049635	7.43E-01	13636	1.69E-02	Down	2.39	4932	0.13	0.18
rs1146359	126868	C1orf161	1	116369953	7.43E-01	13637	8.02E-01	Up	0.25	12685	0.13	0.01
rs1573040	118472	ZNF511	10	134996251	7.44E-01	13638	2.70E-14	Up	7.61	438	0.13	1.36
rs2889854	51385	ZNF589	3	48243513	7.44E-01	13639	5.80E-01	Down	0.55	11321	0.13	0.02
rs5987128	6535	SLC6A8		152460575	7.45E-01	13640	2.67E-02	Up	2.22	5348	0.13	0.16
rs678084	4236	MFAP1	15	41907851	7.46E-01	13641	3.55E-07	Down	5.09	1435	0.13	0.64
rs678084	79968	WDR76	15	41907851	7.46E-01	13642	9.02E-01	Down	0.12	13258	0.13	0.00
rs9729550	8784	TNFRSF18	1	1175165	7.49E-01	13643	6.69E-01	Down	0.43	11876	0.13	0.02
rs2741932	7755	ZNF205	16	3093407	7.49E-01	13644	9.68E-03	Up	2.59	4487	0.13	0.20
rs13399758	124872	B4GALNT2	2	27367377	7.50E-01	13645	1.18E-01	Up	1.56	7260	0.13	0.09
rs13399758	790	CAD	2	27367377	7.50E-01	13646	3.35E-01	Up	0.96	9483	0.13	0.05
rs7004029	90987	ZNF251	8	145916846	7.50E-01	13647	6.76E-01	Up	0.42	11924	0.12	0.02
rs3810213	404217	CTXN1	19	7900233	7.51E-01	13648	8.49E-01	Down	0.19	12951	0.12	0.01
rs12382571	55285	RBM41		106128770	7.51E-01	13649	6.83E-01	Down	0.41	11964	0.12	0.02
rs762514	2664	GDI1		153238937	7.51E-01	13650	5.36E-01	Down	0.62	10985	0.12	0.03
rs762514	9130	FAM50A		153238937	7.51E-01	13651	8.30E-01	Down	0.21	12853	0.12	0.01
rs4074426	8636	SSNA1	9	137344179	7.53E-01	13652	5.05E-01	Up	0.67	10748	0.12	0.03
rs11853333	22893	BAHD1	15	38529630	7.54E-01	13653	1.19E-01	Down	1.56	7271	0.12	0.09
rs11750814	10112	KIF20A	5	137528564	7.54E-01	13654	6.59E-03	Up	2.72	4219	0.12	0.22
rs12011862	54328	GPR173		52942340	7.54E-01	13655	4.22E-01	Down	0.80	10131	0.12	0.04
rs749326	57026	PDXP	22	36375519	7.56E-01	13656	1.66E-01	Up	1.39	7876	0.12	0.08
rs2286963	33	ACADL	2	210885556	7.57E-01	13657	2.38E-01	Down	1.18	8644	0.12	0.06
rs3815076	92579	G6PC3	17	39507629	7.57E-01	13658	4.07E-01	Up	0.83	10031	0.12	0.04
rs2397105	2938	GSTA1	6	52761283	7.57E-01	13659	2.09E-02	Down	2.31	5110	0.12	0.17
rs5933159	55796	MBNL3		131315348	7.57E-01	13660	6.72E-01	Down	0.42	11898	0.12	0.02
rs2277339	5557	PRIM1	12	55432336	7.59E-01	13661	1.91E-02	Up	2.34	5029	0.12	0.17
rs5919577	286451	YIPF6		67490618	7.60E-01	13662	5.90E-01	Up	0.54	11372	0.12	0.02
rs10072450	10917	BTNL3	5	180366486	7.60E-01	13663	1.44E-01	Down	1.46	7616	0.12	0.08
rs5906742	5355	PLP2		48764854	7.63E-01	13664	5.67E-29	Down	11.17	71	0.12	2.82
rs5906742	79917	MAGIX		48764854	7.63E-01	13665	1.34E-01	Up	1.50	7505	0.12	0.09
rs10058772	92304	SCGB3A1	5	179965898	7.63E-01	13666	1.27E-04	Down	3.83	2508	0.12	0.39
rs1063739	2875	GPT	8	145700535	7.63E-01	13667	3.73E-02	Down	2.08	5685	0.12	0.14
rs1063739	84988	PPP1R16A	8	145700535	7.63E-01	13668	8.09E-01	Down	0.24	12716	0.12	0.01
rs11650083	58485	TRAPPC1	17	7788680	7.64E-01	13669	4.68E-04	Down	3.50	2936	0.12	0.33
rs4824419	8241	RBM10		46744227	7.64E-01	13670	3.18E-03	Up	2.95	3814	0.12	0.25
rs475025	116442	RAB39B		154048641	7.65E-01	13671	1.80E-05	Up	4.29	2071	0.12	0.47
rs11780874	1537	CYC1	8	145221292	7.65E-01	13672	3.53E-08	Up	5.55	1159	0.12	0.75
rs11780874	84232	MAF1	8	145221292	7.65E-01	13673	7.87E-01	Down	0.27	12588	0.12	0.01
rs13817	30008	EFEMP2	11	65384910	7.66E-01	13674	1.57E-20	Down	9.29	186	0.12	1.98
rs13817	1072	CFL1	11	65384910	7.66E-01	13675	1.84E-02	Up	2.36	5003	0.12	0.17
rs13817	1521	CTSW	11	65384910	7.66E-01	13676	4.64E-02	Up	1.99	5908	0.12	0.13
rs13817	80198	MUS81	11	65384910	7.66E-01	13677	3.15E-01	Down	1.00	9324	0.12	0.05
rs2098386	10153	CEBPZ	2	37367683	7.67E-01	13678	6.41E-01	Up	0.47	11716	0.12	0.02
rs5934066	171484	FAM9C		12830927	7.67E-01	13679	7.22E-01	Up	0.36	12215	0.12	0.01
rs2066808	6773	STAT2	12	55024240	7.67E-01	13680	4.97E-01	Up	0.68	10692	0.11	0.03
rs2066808	51561	IL23A	12	55024240	7.67E-01	13681	8.32E-01	Up	0.21	12861	0.11	0.01
rs3813619	5498	PPOX	1	157956874	7.68E-01	13682	8.17E-04	Up	3.35	3143	0.11	0.31
rs5906535	57232	ZNF630		47668125	7.69E-01	13683	2.09E-02	Up	2.31	5111	0.11	0.17
rs10198499	55627	SMPD4	2	130659743	7.70E-01	13684	4.77E-02	Up	1.98	5940	0.11	0.13

rs10198499	80097	FAM128B	2	130659743	7.70E-01	13685	4.34E-01	Up	0.78	10225	0.11	0.04
rs11796677	50945	TBX22		79081124	7.72E-01	13686	8.31E-01	Up	0.21	12858	0.11	0.01
rs3924892	55038	CDCA4	14	104565307	7.75E-01	13687	8.06E-02	Up	1.75	6639	0.11	0.11
rs9843295	63899	NSUN3	3	95273232	7.76E-01	13688	2.09E-03	Up	3.08	3556	0.11	0.27
rs7814319	51001	MTERFD1	8	97332594	7.77E-01	13689	5.88E-02	Up	1.89	6212	0.11	0.12
rs507127	1193	CLIC2		154078623	7.77E-01	13690	1.66E-02	Down	2.40	4917	0.11	0.18
rs12690217	203430	ZCCHC5		77733469	7.77E-01	13691	2.47E-01	Up	1.16	8736	0.11	0.06
rs6071089	10398	MYL9	20	34617113	7.78E-01	13692	1.62E-18	Down	8.78	248	0.11	1.78
rs11058562	55596	ZCCHC8	12	121528197	7.79E-01	13693	5.01E-01	Up	0.67	10722	0.11	0.03
rs1346268	2628	GATM	15	43460321	7.79E-01	13694	5.02E-30	Down	11.38	63	0.11	2.93
rs10184772	64969	MRPS5	2	95200239	7.83E-01	13695	6.60E-01	Up	0.44	11830	0.11	0.02
rs2517396	9070	ASH2L	8	38108462	7.84E-01	13696	1.20E-03	Down	3.24	3291	0.11	0.29
rs84770	6663	SOX10	22	36714628	7.85E-01	13697	2.35E-03	Down	3.04	3646	0.11	0.26
rs11264542	3068	HDGF	1	153551042	7.85E-01	13698	3.63E-02	Up	2.09	5657	0.11	0.14
rs11684134	2976	GTF3C2	2	27469903	7.86E-01	13699	2.69E-03	Up	3.00	3727	0.10	0.26
rs11684134	4358	MPV17	2	27469903	7.86E-01	13700	4.09E-03	Up	2.87	3946	0.10	0.24
rs2072590	3231	HOXD1	2	176868140	7.87E-01	13701	1.77E-01	Up	1.35	7981	0.10	0.08
rs5977248	9016	SLC25A14		129227022	7.87E-01	13702	3.25E-03	Up	2.94	3823	0.10	0.25
rs7155081	341947	COX8C	14	92869929	7.87E-01	13703	2.81E-02	Up	2.20	5394	0.10	0.16
rs11555927	10916	MAGED2		54724758	7.89E-01	13704	1.20E-01	Down	1.56	7292	0.10	0.09
rs7877190	79589	RNF128		105852802	7.89E-01	13705	1.32E-10	Down	6.42	774	0.10	0.99
rs2472707	10750	GRAP	17	18858049	7.90E-01	13706	6.07E-01	Down	0.51	11481	0.10	0.02
rs1938690	219988	PATL1	11	59188413	7.92E-01	13707	1.09E-06	Up	4.87	1587	0.10	0.60
rs12632423	6159	RPL29	3	52016606	7.93E-01	13708	2.89E-19	Up	8.97	220	0.10	1.85
rs3088114	55146	ZDHHC4	7	6401482	7.93E-01	13709	2.17E-03	Down	3.07	3588	0.10	0.27
rs494560	11181	TREH	11	118026759	7.94E-01	13710	4.32E-01	Down	0.79	10207	0.10	0.04
rs12860055	1538	CYLC1		82929821	7.94E-01	13711	3.45E-01	Up	0.94	9559	0.10	0.05
rs10416852	51710	ZNF44	19	12213086	7.94E-01	13712	2.63E-01	Up	1.12	8897	0.10	0.06
rs2858942	3040	HBA2	16	165653	7.94E-01	13713	7.81E-02	Up	1.76	6597	0.10	0.11
rs13408493	7781	SLC30A3	2	27402154	7.96E-01	13714	2.91E-01	Down	1.05	9125	0.10	0.05
rs13408493	285126	DNAJC5G	2	27402154	7.96E-01	13715	9.50E-01	Up	0.06	13554	0.10	0.00
rs4930713	10959	TMED2	12	122605499	7.97E-01	13716	1.71E-08	Up	5.64	1102	0.10	0.78
rs337365	54732	TMED9	5	176936222	7.97E-01	13717	3.15E-01	Up	1.01	9315	0.10	0.05
rs9303318	55175	KLHL11	17	37273611	7.97E-01	13718	7.00E-01	Down	0.39	12072	0.10	0.02
rs11211176	3652	IPP	1	45935106	7.98E-01	13719	5.83E-02	Up	1.89	6198	0.10	0.12
rs17268265	51634	RBMX2		129277539	7.98E-01	13720	8.61E-02	Up	1.72	6738	0.10	0.11
rs3027514	7355	SLC35A2		48531188	8.00E-01	13721	7.73E-03	Up	2.66	4321	0.10	0.21
rs3027514	10245	TIMM17B		48531188	8.00E-01	13722	8.61E-02	Up	1.72	6739	0.10	0.11
rs3027514	11040	PIM2		48531188	8.00E-01	13723	1.11E-01	Up	1.59	7164	0.10	0.10
rs3027514	55593	OTUD5		48531188	8.00E-01	13724	1.85E-01	Up	1.32	8090	0.10	0.07
rs2731672	2161	F12	5	176775080	8.02E-01	13725	1.46E-02	Up	2.44	4789	0.10	0.18
rs2073236	79649	MAP7D3		135103902	8.02E-01	13726	6.11E-01	Down	0.51	11512	0.10	0.02
rs9290727	6657	SOX2	3	182903320	8.04E-01	13727	4.88E-01	Down	0.69	10636	0.09	0.03
rs7617915	2779	GNAT1	3	50214016	8.05E-01	13728	4.17E-01	Down	0.81	10103	0.09	0.04
rs2224198	2939	GSTA2	6	52713868	8.05E-01	13729	9.19E-05	Down	3.91	2430	0.09	0.40
rs9874152	79572	ATP13A3	3	195671723	8.05E-01	13730	1.12E-07	Up	5.31	1306	0.09	0.70
rs928046	92714	ARRDC1	9	137762945	8.06E-01	13731	9.79E-04	Up	3.30	3211	0.09	0.30
rs473279	79693	YRDC	1	37944895	8.06E-01	13732	2.12E-06	Up	4.74	1678	0.09	0.57
rs473279	4520	MTF1	1	37944895	8.06E-01	13733	4.05E-01	Up	0.83	10020	0.09	0.04
rs473279	127687	C1orf122	1	37944895	8.06E-01	13734	5.08E-01	Up	0.66	10770	0.09	0.03
rs16869698	57561	ARRDC3	5	90690335	8.06E-01	13735	9.14E-01	Down	0.11	13340	0.09	0.00
rs2518461	51368	TEX264	3	51713141	8.06E-01	13736	5.38E-01	Down	0.62	10995	0.09	0.03
rs12840573	3561	IL2RG		70121139	8.08E-01	13737	8.36E-02	Up	1.73	6693	0.09	0.11
rs6797587	993	CDC25A	3	48172618	8.09E-01	13738	9.64E-02	Up	1.66	6930	0.09	0.10
rs9824	10195	ALG3	3	185459896	8.11E-01	13739	1.28E-05	Up	4.36	2011	0.09	0.49
rs1643494	114783	LMTK3	19	53712127	8.11E-01	13740	4.85E-01	Up	0.70	10616	0.09	0.03
rs6984782	79145	CHCHD7	8	57298443	8.13E-01	13741	7.49E-01	Down	0.32	12373	0.09	0.01
rs192705	84267	C9orf64	9	83821258	8.13E-01	13742	1.73E-01	Up	1.36	7947	0.09	0.08
rs6939589	8365	HIST1H4H	6	26397182	8.13E-01	13743	1.24E-15	Up	7.94	371	0.09	1.49
rs2279439	29763	PACSIN3	11	47153558	8.15E-01	13744	3.17E-02	Up	2.15	5516	0.09	0.15
rs8110543	10975	UQCR	19	1540064	8.16E-01	13745	3.52E-04	Up	3.57	2836	0.09	0.35
rs3087657	80237	ELL3	15	41851151	8.17E-01	13746	1.09E-08	Up	5.73	1059	0.09	0.80
rs2286845	10282	BET1	7	93268109	8.17E-01	13747	8.44E-02	Up	1.73	6708	0.09	0.11
rs560947	7760	ZNF213	16	3125738	8.18E-01	13748	8.29E-01	Up	0.22	12847	0.09	0.01
rs11657325	124790	HEXIM2	17	40598477	8.18E-01	13749	1.94E-01	Down	1.30	8188	0.09	0.07
rs2769267	5993	RFX5	1	148146240	8.18E-01	13750	3.96E-01	Down	0.85	9957	0.09	0.04
rs10883511	4714	NDUFB8	10	102289397	8.21E-01	13751	8.68E-07	Down	4.92	1557	0.09	0.61
rs10883511	55662	HIF1AN	10	102289397	8.21E-01	13752	3.57E-04	Down	3.57	2843	0.09	0.34
rs10504229	26138	C8orf71	8	58359049	8.26E-01	13753	8.27E-02	Up	1.74	6680	0.08	0.11
rs2293445	23109	DDN	12	47685129	8.28E-01	13754	2.11E-02	Down	2.31	5120	0.08	0.17
rs910397	11264	PXMP4	20	31759202	8.28E-01	13755	3.67E-01	Down	0.90	9738	0.08	0.04
rs6801801	10201	NME6	3	48301437	8.30E-01	13756	6.62E-01	Up	0.44	11837	0.08	0.02
rs4974445	131601	GPR175	3	128775486	8.31E-01	13757	4.37E-01	Up	0.78	10247	0.08	0.04
rs2302009	10344	CCL26	7	75043649	8.31E-01	13758	5.62E-01	Down	0.58	11177	0.08	0.03
rs2927071	161497	STRC	15	41706373	8.31E-01	13759	3.37E-02	Down	2.12	5581	0.08	0.15
rs2927071	117155	CATSPER2	15	41706373	8.31E-01	13760	7.69E-01	Down	0.29	12480	0.08	0.01
rs7063261	10134	BCAP31		152518295	8.32E-01	13761	4.19E-01	Up	0.81	10116	0.08	0.04
rs3813922	128854	TSPYL3	20	30240997	8.33E-01	13762	5.57E-01	Up	0.59	11128	0.08	0.03
rs3813922	5326	PLAGL2	20	30240997	8.33E-01	13763	8.10E-01	Down	0.24	12723	0.08	0.01
rs2280231	4722	NDUFS3	11	47557014	8.34E-01	13764	1.28E-01	Up	1.52	7418	0.08	0.09
rs2280231	55709	KBTBD4	11	47557014	8.34E-01	13765	4.54E-01	Up	0.75	10375	0.08	0.03

rs9781965	8823	FGF16		76523219	8.35E-01	13766	7.20E-02	Down	1.80	6467	0.08	0.11
rs6562326	283491	OR7E156P	13	63208758	8.35E-01	13767	9.72E-02	Down	1.66	6945	0.08	0.10
rs1052053	632	BGLAP	1	153015246	8.36E-01	13768	4.26E-03	Down	2.86	3973	0.08	0.24
rs1052053	11243	PMF1	1	153015246	8.36E-01	13769	4.48E-01	Up	0.76	10335	0.08	0.03
rs11736218	3015	H2AFZ	4	101233826	8.40E-01	13770	6.23E-04	Down	3.42	3042	0.08	0.32
rs2301723	6128	RPL6	12	111306083	8.40E-01	13771	5.96E-14	Up	7.51	456	0.08	1.32
rs3780616	10952	SEC61B	9	99067315	8.40E-01	13772	5.78E-06	Up	4.53	1840	0.08	0.52
rs3780616	85365	ALG2	9	99067315	8.40E-01	13773	7.96E-02	Up	1.75	6626	0.08	0.11
rs725979	53940	FTHL17		30862608	8.43E-01	13774	8.56E-01	Down	0.18	12990	0.07	0.01
rs6791696	6152	RPL24	3	102891786	8.47E-01	13775	1.92E-07	Up	5.21	1367	0.07	0.67
rs2211211	161291	TMEM30B	14	60806890	8.49E-01	13776	1.99E-03	Up	3.09	3528	0.07	0.27
rs1465789	388569	ZNF324B	19	63637868	8.49E-01	13777	3.34E-02	Down	2.13	5576	0.07	0.15
rs8108647	23383	KIAA0892	19	19288623	8.51E-01	13778	2.39E-02	Up	2.26	5241	0.07	0.16
rs3094378	11182	SLC2A6	9	133366722	8.53E-01	13779	7.31E-01	Up	0.34	12254	0.07	0.01
rs285236	26017	FAM32A	19	16160174	8.53E-01	13780	4.38E-02	Up	2.02	5846	0.07	0.14
rs11265558	51506	UFC1	1	157920254	8.53E-01	13781	1.32E-02	Up	2.48	4701	0.07	0.19
rs11265558	9191	DEDD	1	157920254	8.53E-01	13782	3.57E-02	Up	2.10	5639	0.07	0.14
rs11126918	8884	SLC5A6	2	27350361	8.54E-01	13783	1.58E-01	Up	1.41	7781	0.07	0.08
rs11126918	51374	C2orf28	2	27350361	8.54E-01	13784	2.30E-01	Down	1.20	8566	0.07	0.06
rs11706549	22907	DHX30	3	47821570	8.54E-01	13785	1.95E-01	Up	1.30	8204	0.07	0.07
rs8076790	146923	RUNDC1	17	38408126	8.57E-01	13786	5.46E-01	Up	0.60	11055	0.07	0.03
rs7256033	1082	CGB	19	54209025	8.57E-01	13787	2.39E-02	Up	2.26	5244	0.07	0.16
rs1054487	51398	C19orf56	19	12633165	8.58E-01	13788	2.21E-02	Down	2.29	5163	0.07	0.17
rs6604082	23285	KIAA1107	1	92353628	8.59E-01	13789	2.63E-01	Up	1.12	8891	0.07	0.06
rs7890805	2925	GRPR		15912345	8.61E-01	13790	3.12E-02	Up	2.15	5503	0.07	0.15
rs6647583	340533	KIAA2022		73929270	8.63E-01	13791	1.89E-01	Down	1.31	8138	0.06	0.07
rs5983240	4675	NAP1L3		92729569	8.64E-01	13792	1.89E-04	Down	3.73	2606	0.06	0.37
rs1446966	325	APCS	1	156376556	8.65E-01	13793	9.05E-02	Up	1.69	6836	0.06	0.10
rs2987621	85026	C9orf37	9	137802276	8.67E-01	13794	1.52E-01	Down	1.43	7701	0.06	0.08
rs3745231	162989	DEDD2	19	47405675	8.69E-01	13795	7.39E-04	Down	3.37	3097	0.06	0.31
rs3745231	116115	ZNF526	19	47405675	8.69E-01	13796	6.44E-01	Up	0.46	11736	0.06	0.02
rs992157	14	AAMP	2	218980286	8.69E-01	13797	3.57E-06	Up	4.64	1766	0.06	0.54
rs992157	64114	TMBIM1	2	218980286	8.69E-01	13798	1.33E-03	Down	3.21	3334	0.06	0.29
rs17589398	10586	MAB21L2	4	151854364	8.70E-01	13799	5.14E-01	Up	0.65	10808	0.06	0.03
rs12091870	92815	HIST3H2A	1	224963515	8.71E-01	13800	1.53E-05	Up	4.32	2040	0.06	0.48
rs2236947	11068	CYB561D2	3	50346436	8.71E-01	13801	6.04E-02	Up	1.88	6241	0.06	0.12
rs2236947	11186	RASSF1	3	50346436	8.71E-01	13802	4.65E-01	Down	0.73	10466	0.06	0.03
rs2236947	51364	ZMYND10	3	50346436	8.71E-01	13803	7.09E-01	Down	0.37	12124	0.06	0.01
rs2236947	8692	HYAL2	3	50346436	8.71E-01	13804	7.21E-01	Up	0.36	12200	0.06	0.01
rs231531	146894	CD300LG	17	39301706	8.73E-01	13805	6.91E-01	Up	0.40	12018	0.06	0.02
rs11713363	6474	SHOX2	3	159311024	8.73E-01	13806	6.98E-01	Up	0.39	12060	0.06	0.02
rs5950587	186	AGTR2		115119313	8.74E-01	13807	6.44E-02	Up	1.85	6333	0.06	0.12
rs2076139	23654	PLXNB2	22	49007118	8.76E-01	13808	5.24E-03	Up	2.79	4100	0.06	0.23
rs6685064	6339	SCNN1D	1	1251215	8.79E-01	13809	2.21E-01	Down	1.22	8469	0.06	0.07
rs6737556	4709	NDUFB3	2	201749584	8.80E-01	13810	3.58E-01	Up	0.92	9663	0.06	0.04
rs6737556	285172	FAM126B	2	201749584	8.80E-01	13811	6.86E-01	Up	0.40	11985	0.06	0.02
rs4925810	8928	FOXH1	8	145676021	8.80E-01	13812	6.00E-01	Up	0.52	11431	0.06	0.02
rs2978916	1667	DEFA1	8	6815651	8.80E-01	13813	1.73E-01	Up	1.36	7943	0.06	0.08
rs7876304	55634	ZNF673		46080847	8.81E-01	13814	1.59E-02	Up	2.41	4877	0.05	0.18
rs17326689	5631	PRPS1		106659200	8.81E-01	13815	3.59E-03	Up	2.91	3871	0.05	0.24
rs12315146	8550	MAPKAPK5	12	110785736	8.84E-01	13816	1.75E-03	Up	3.13	3463	0.05	0.28
rs4149818	5977	DPF2	11	64840067	8.84E-01	13817	8.68E-02	Down	1.71	6752	0.05	0.11
rs6071036	63939	C20orf177	20	57950070	8.86E-01	13818	1.70E-08	Down	5.64	1105	0.05	0.78
rs17428	2010	EMD		153130810	8.87E-01	13819	3.54E-01	Down	0.93	9621	0.05	0.05
rs16950305	51367	POP5	12	119485201	8.88E-01	13820	9.78E-01	Up	0.03	13757	0.05	0.00
rs695161	3065	HDAC1	1	32412959	8.89E-01	13821	5.64E-16	Up	8.08	354	0.05	1.52
rs695161	3932	LCK	1	32412959	8.89E-01	13822	1.87E-01	Up	1.32	8110	0.05	0.07
rs4776783	54962	TIPIN	15	64449161	8.90E-01	13823	7.59E-02	Up	1.78	6545	0.05	0.11
rs10779958	550	AUP1	2	74667433	8.92E-01	13824	1.61E-02	Up	2.41	4891	0.05	0.18
rs10779958	84695	LOXL3	2	74667433	8.92E-01	13825	3.52E-01	Down	0.93	9608	0.05	0.05
rs10779958	165545	DQX1	2	74667433	8.92E-01	13826	6.12E-01	Down	0.51	11520	0.05	0.02
rs730566	11277	TREX1	3	48462052	8.92E-01	13827	1.86E-07	Up	5.21	1364	0.05	0.67
rs730566	51372	CCDC72	3	48462052	8.92E-01	13828	2.27E-04	Up	3.69	2674	0.05	0.36
rs730566	79714	CCDC51	3	48462052	8.92E-01	13829	5.04E-03	Up	2.80	4074	0.05	0.23
rs10888650	4725	NDUFS5	1	39176254	8.94E-01	13830	1.25E-04	Down	3.84	2503	0.05	0.39
rs576950	84866	TMEM25	11	117896585	8.97E-01	13831	1.46E-03	Up	3.18	3367	0.05	0.28
rs1880948	8468	FKBP6	7	72167632	8.98E-01	13832	3.24E-02	Up	2.14	5539	0.05	0.15
rs12299505	25766	PRPF40B	12	48306161	9.01E-01	13833	5.23E-01	Down	0.64	10877	0.05	0.03
rs17134462	5478	PPIA	7	44618272	9.01E-01	13834	2.37E-01	Up	1.18	8629	0.05	0.06
rs4239975	10214	SSX3		47975323	9.07E-01	13835	2.60E-01	Down	1.13	8856	0.04	0.06
rs7100	3615	IMPDH2	3	49028223	9.08E-01	13836	9.38E-23	Up	9.83	143	0.04	2.20
rs7100	25915	C3orf60	3	49028223	9.08E-01	13837	8.65E-03	Up	2.63	4402	0.04	0.21
rs7100	11180	WDR6	3	49028223	9.08E-01	13838	3.52E-02	Up	2.11	5630	0.04	0.15
rs7100	55152	DALRD3	3	49028223	9.08E-01	13839	2.67E-01	Up	1.11	8924	0.04	0.06
rs1057227	10817	FRS3	6	41846136	9.09E-01	13840	5.83E-02	Down	1.89	6199	0.04	0.12
rs7541207	7389	UROD	1	45143101	9.10E-01	13841	2.65E-06	Down	4.70	1715	0.04	0.56
rs7541207	79654	HECTD3	1	45143101	9.10E-01	13842	3.25E-01	Down	0.98	9407	0.04	0.05
rs10242703	221960	C7orf28B	7	6643875	9.14E-01	13843	6.67E-01	Up	0.43	11862	0.04	0.02
rs6087487	6640	SNTA1	20	31478697	9.15E-01	13844	6.38E-01	Up	0.47	11690	0.04	0.02
rs743548	1774	DNASE1L1		153171267	9.15E-01	13845	4.19E-11	Up	6.64	690	0.04	1.04
rs11689101	7247	TSN	2	122225800	9.15E-01	13846	1.34E-01	Down	1.50	7489	0.04	0.09

rs11190578	7479	WNT8B	10	102228943	9.17E-01	13847	7.74E-01	Up	0.29	12523	0.04	0.01
rs3112998	7549	ZNF2	2	95244321	9.18E-01	13848	1.61E-03	Up	3.15	3423	0.04	0.28
rs1810132	2064	ERBB2	17	35119531	9.19E-01	13849	6.73E-02	Down	1.83	6384	0.04	0.12
rs708382	3674	ITGA2B	17	39797870	9.23E-01	13850	3.95E-01	Up	0.85	9950	0.03	0.04
rs1298577	54830	NUP62CL		106202547	9.26E-01	13851	1.77E-01	Up	1.35	7985	0.03	0.08
rs12008279	9075	CLDN2		105966847	9.26E-01	13852	9.51E-02	Down	1.67	6910	0.03	0.10
rs5987005	30848	CTAG2		153456571	9.27E-01	13853	7.43E-02	Up	1.78	6512	0.03	0.11
rs839354	8562	DENR	12	121790290	9.27E-01	13854	2.51E-05	Up	4.21	2132	0.03	0.46
rs11625677	10965	ACOT2	14	73109406	9.30E-01	13855	7.93E-04	Down	3.36	3132	0.03	0.31
rs7379033	79908	BTNL8	5	180246943	9.31E-01	13856	7.04E-01	Down	0.38	12095	0.03	0.02
rs16974961	1553	CYP2A13	19	46291894	9.32E-01	13857	8.81E-01	Down	0.15	13136	0.03	0.01
rs3760779	7568	ZNF20	19	12114327	9.35E-01	13858	2.94E-01	Up	1.05	9146	0.03	0.05
rs2344484	4762	NEUROG1	5	134904530	9.35E-01	13859	1.12E-02	Down	2.54	4581	0.03	0.20
rs2064034	56850	GRIPAP1		48580224	9.37E-01	13860	1.78E-01	Up	1.35	8003	0.03	0.07
rs2064034	3750	KCND1		48580224	9.37E-01	13861	6.99E-01	Down	0.39	12061	0.03	0.02
rs12154743	155370	SBDSP	7	71728216	9.39E-01	13862	9.62E-06	Down	4.43	1943	0.03	0.50
rs4955416	54870	QRICH1	3	49083432	9.40E-01	13863	2.25E-01	Down	1.21	8504	0.03	0.06
rs6707475	64427	TTC31	2	74622146	9.40E-01	13864	5.48E-07	Up	5.01	1493	0.03	0.63
rs6707475	116540	MRPL53	2	74622146	9.40E-01	13865	9.78E-04	Up	3.30	3210	0.03	0.30
rs6707475	84865	CCDC142	2	74622146	9.40E-01	13866	6.10E-01	Up	0.51	11503	0.03	0.02
rs11632941	81698	C15orf5	15	75320943	9.42E-01	13867	1.31E-01	Down	1.51	7460	0.03	0.09
rs8129	29965	C16orf5	16	4502352	9.43E-01	13868	1.92E-15	Down	7.95	368	0.03	1.47
rs12763	6834	SURF1	9	133256814	9.43E-01	13869	7.18E-04	Up	3.38	3087	0.03	0.31
rs12763	6836	SURF4	9	133256814	9.43E-01	13870	1.05E-03	Up	3.28	3239	0.03	0.30
rs12763	6835	SURF2	9	133256814	9.43E-01	13871	3.08E-03	Up	2.96	3788	0.03	0.25
rs2290822	5364	PLXNB1	3	48448208	9.45E-01	13872	6.56E-02	Up	1.84	6354	0.02	0.12
rs699669	56156	TEX13B		107034069	9.46E-01	13873	1.13E-01	Down	1.58	7196	0.02	0.09
rs5945279	65991	FUNDC2		153836170	9.47E-01	13874	3.69E-01	Down	0.90	9752	0.02	0.04
rs11666790	10998	SLC27A5	19	63725153	9.48E-01	13875	3.94E-01	Up	0.85	9939	0.02	0.04
rs10975003	3641	INSL4	9	5203687	9.50E-01	13876	6.01E-01	Up	0.52	11435	0.02	0.02
rs5987623	9737	GPRASP1		101704269	9.50E-01	13877	2.92E-10	Down	6.30	807	0.02	0.95
rs876896	51275	C12orf47	12	110760977	9.51E-01	13878	4.99E-04	Up	3.48	2959	0.02	0.33
rs2746025	125170	SMCR7	17	18092336	9.51E-01	13879	2.29E-02	Up	2.27	5195	0.02	0.16
rs11736402	152641	C4orf38	4	184385866	9.51E-01	13880	1.20E-01	Up	1.55	7300	0.02	0.09
rs3767027	91624	NEXN	1	78128690	9.52E-01	13881	2.69E-10	Down	6.32	804	0.02	0.96
rs2057831	8697	CDC23	5	137563032	9.53E-01	13882	4.06E-01	Up	0.83	10024	0.02	0.04
rs717193	10084	PQBP1		48519729	9.53E-01	13883	7.26E-02	Up	1.80	6478	0.02	0.11
rs7831467	286077	FAM83H	8	144894025	9.55E-01	13884	1.50E-14	Up	7.69	422	0.02	1.38
rs4074453	80757	TMEM121	14	105069589	9.57E-01	13885	1.43E-01	Down	1.46	7610	0.02	0.08
rs10151805	283643	C14orf80	14	105045826	9.59E-01	13886	2.96E-01	Up	1.05	9161	0.02	0.05
rs622614	11007	CCDC85B	11	65419815	9.60E-01	13887	2.91E-02	Down	2.18	5429	0.02	0.15
rs622614	9158	FIBP	11	65419815	9.60E-01	13888	4.60E-02	Up	2.00	5895	0.02	0.13
rs7412746	3276	PRMT1	1	147673544	9.60E-01	13889	4.11E-01	Up	0.82	10067	0.02	0.04
rs17680262	84260	TCHP	12	108817256	9.61E-01	13890	9.40E-01	Up	0.08	13493	0.02	0.00
rs10280764	81554	WBSCR16	7	73934540	9.62E-01	13891	3.30E-03	Up	2.94	3828	0.02	0.25
rs281767	79568	C2orf47	2	200648787	9.64E-01	13892	2.01E-01	Up	1.28	8268	0.02	0.07
rs4397290	54919	HEATR2	7	601734	9.67E-01	13893	2.68E-04	Up	3.64	2740	0.01	0.36
rs2306899	23259	DDHD2	8	38214819	9.67E-01	13894	1.53E-06	Down	4.81	1630	0.01	0.58
rs139873	5435	POLR2F	22	36684167	9.68E-01	13895	1.85E-03	Up	3.11	3490	0.01	0.27
rs139873	84645	C22orf23	22	36684167	9.68E-01	13896	1.11E-01	Down	1.59	7160	0.01	0.10
rs12977802	5438	POLR2I	19	41313483	9.68E-01	13897	3.56E-01	Up	0.92	9640	0.01	0.04
rs1008628	90135	BTBD6	14	104793771	9.77E-01	13898	9.49E-01	Down	0.06	13540	0.01	0.00
rs7202714	11151	CORO1A	16	30085308	9.77E-01	13899	8.96E-01	Down	0.13	13219	0.01	0.00
rs9728169	199974	CYP4Z1	1	47236284	9.81E-01	13900	4.88E-03	Down	2.82	4049	0.01	0.23
rs3129157	26707	OR2J2	6	29249722	9.82E-01	13901	4.23E-01	Up	0.80	10141	0.01	0.04
rs5945282	4515	MTCP1		153846095	9.83E-01	13902	4.34E-04	Down	3.52	2906	0.01	0.34
rs11628672	3500	IGHG1	14	105270624	9.86E-01	13903	1.79E-01	Up	1.34	8008	0.01	0.07
rs6443700	151613	TTC14	3	181783505	9.86E-01	13904	3.92E-07	Up	5.07	1453	0.01	0.64
rs10193210	22950	SLC4A1AP	2	27787314	9.97E-01	13905	7.10E-01	Down	0.37	12128	0.00	0.01
